# Supplementary material for: Locational memory of macrovessel vascular cells is transcriptionally imprinted
Source: Sci Rep. 2023 Aug 10;13:13028. doi: 10.1038/s41598-023-38880-6 (PMC10415317; doi:10.1038/s41598-023-38880-6)
Supplement: Supplementary file 15 — Supplementary Table 6. [file 41598_2023_38880_MOESM15_ESM.pdf]

Supplemental Table 6. Gene ontology terms for contrasts of all sample groups.

category	over_represented_pvalue	under_represented_pvalue	numDEInCat	numInCat	term	ontology	p.adjust	EnsemblID	vessel
GO:0051276	4.52E-18	1	88	398	chromosome organization	BP	1.46E-14	HMGB1,MCM6,SREBF1,MCM4,POLE3,MCM7,RPA1,NCAPD2,ATAD2,HMGA1,HNRNPD,SMC1A,ZW10,ENSCAFG00000016482,SMC4,CENPA,SLC29A1,ENSCAFG00000017264,NDC1,CENPN,TOP3A,TRIP13,SGO1,GINS4,FBXO5,RAN,BIRC5,PRMT1,CDK1,RAD51,AURKB,DSN1,EZH2,CENPO,NCAPD3,NEK2,FBL,FEN1,SMARCC1,HMGB3,KIF22,UHRF1,CDCA8,CENPH,NCAPH,BRCA1,CENPI,NOC2L,SMC2,ESPL1,CDC6,NDC80,OIP5,IPO4,HIST1H1A,NUP62,SUV39H1,HASPIN,HMGB2,DMAP1,HIST1H2BB,SMC3,SMARCB1,CCNA2,ENSCAFG00000005299,SMC6,BUB1,RBM14,CENPW,CDC20,TTK,BAP1,SGO2,CENPP,GRWD1,SUPT16H,TADA2A,HAT1,HIST2H2AC,TINF2,H2AFX,UBE2C,MIS18A,HMGN1,CCT4,H3F3B,ZWINT,GEN1	down_ACOR_VSMC
GO:1903047	2.3E-17	1	66	257	mitotic cell cycle process	BP	3.71E-14	NCAPD2,ANLN,SMC1A,HINFP,UBE2S,ZW10,SMC4,CENPA,ENSCAFG00000017264,TRIP13,SGO1,POLE,FBXO5,RAN,BIRC5,CDK1,RAD51,CASP2,FANCI,AURKB,CDKN2C,TOPBP1,DSN1,EZH2,STMN1,NCAPD3,MAP3K20,NEK2,CDK4,RNASEH2B,PLK3,RCC1,KIF22,CKS2,CDCA8,NCAPH,POC1A,WDR62,SMC2,ESPL1,SPC25,CDC6,NDC80,NUP62,DTL,PDXP,HASPIN,REEP4,SMC3,CDK2,CCNA2,RAB35,STAG1,ENSCAFG00000005299,RBBP8,CLSPN,BUB1,CDC20,TTK,KIF11,CCNB3,UBE2C,CDKN3,MTBP,ZWINT,GEN1	down_ACOR_VSMC
GO:0022402	4.81E-17	1	87	407	cell cycle process	BP	7.06E-14	NCAPD2,ANLN,SMC1A,HINFP,UBE2S,ZW10,SMC4,CENPA,SLC29A1,ENSCAFG00000017264,NDC1,PPM1G,TRIP13,SGO1,POLE,FBXO5,RAN,PSMD13,BIRC5,CDK1,RAD51,CASP2,FANCI,TIMELESS,AURKB,CDKN2C,TOPBP1,DSN1,EZH2,STMN1,HAUS8,NCAPD3,MAP3K20,NEK2,FEN1,CDK4,MND1,RNASEH2B,PLK3,RCC1,KIF22,CKS2,GMNN,CDCA8,NCAPH,POC1A,BRCA1,WDR62,E2F8,SMC2,ESPL1,SPC25,CDC6,NDC80,NUP62,DTL,PDXP,PRR11,PLK4,HASPIN,REEP4,SMC3,CDK2,CCNA2,RAB35,STAG1,ENSCAFG00000005299,RBBP8,EZR,CLSPN,SASS6,BUB1,RBM14,CDC20,TTK,ASPM,SGO2,KNL1,CDC25A,KIF11,GLI1,CCNB3,UBE2C,CDKN3,MTBP,ZWINT,GEN1	down_ACOR_VSMC
GO:0007059	2.55E-16	1	43	127	chromosome segregation	BP	3.43E-13	NCAPD2,SMC1A,ZW10,SMC4,ENSCAFG00000017264,NDC1,CENPN,TRIP13,SGO1,FBXO5,RAN,BIRC5,AURKB,DSN1,NCAPD3,NEK2,FEN1,RCC1,KIF22,CDCA8,NCAPH,SMC2,ESPL1,SPC25,CDC6,NDC80,OIP5,NUP62,HASPIN,SMC3,ENSCAFG00000005299,SMC6,BUB1,CENPW,CDC20,TTK,SGO2,KNL1,KIF11,UBE2C,MIS18A,ZWINT,GEN1	down_ACOR_VSMC
GO:0007049	3.57E-16	1	105	560	cell cycle	BP	4.44E-13	MCM7,NCAPD2,ANLN,SMC1A,HINFP,UBE2S,WDR76,ZW10,INTS7,SMC4,CENPA,SLC29A1,ENSCAFG00000017264,NDC1,PPM1G,TRIP13,SGO1,POLE,FBXO5,RAN,PSMD13,BIRC5,CDK1,RAD51,CASP2,FANCI,TIMELESS,NF2,AURKB,CDKN2C,TOPBP1,DSN1,EZH2,STMN1,HAUS8,NCAPD3,MAP3K20,NEK2,CLIC1,CDCA3,FEN1,CDK4,TRIM35,MND1,RNASEH2B,PLK3,RCC1,KIF22,CKS2,GMNN,CDCA8,NCAPH,POC1A,BRCA1,WDR62,E2F8,SMC2,ESPL1,SPC25,CDC6,NDC80,NUP62,DTL,PDXP,PRR11,PLK4,IQGAP1,HASPIN,REEP4,SMC3,CDK2,DDX39B,ENSCAFG00000030087,CCNA2,RAB35,STAG1,ENSCAFG00000005299,RBBP8,EZR,CLSPN,SASS6,BUB1,RBM14,RAD23A,CENPW,PHACTR4,CDC20,TTK,BAP1,ASPM,SGO2,ARHGEF2,KNL1,CDC25A,KIF11,TADA2A,GLI1,H2AFX,ATRIP,CCNB3,UBE2C,CDKN3,MTBP,ZWINT,GEN1	down_ACOR_VSMC

GO:0000280	1.19E-14	1	44	145	nuclear division	BP	1.07E-11	NCAPD2,SMC1A,UBE2S,ZW10,SMC4,ENSCAFG00000017264,NDC1,TRIP13,SGO1,FBXO5,RAN,PSMD13,BIRC5,RAD51,AURKB,DSN1,NCAPD3,NEK2,MND1,RCC1,KIF22,CKS2,CDCA8,NCAPH,SMC2,ESPL1,CDC6,NDC80,NUP62,PDXP,HASPIN,SMC3,STAG1,ENSCAFG00000005299,BUB1,CDC20,TTK,ASPM,SGO2,KIF11,UBE2C,MTBP,ZWINT,GEN1	down_ACOR_VSMC
GO:0000819	2.34E-14	1	33	87	sister chromatid segregation	BP	1.99E-11	NCAPD2,SMC1A,ZW10,SMC4,ENSCAFG00000017264,TRIP13,SGO1,FBXO5,RAN,BIRC5,AURKB,DSN1,NCAPD3,NEK2,FEN1,KIF22,CDCA8,NCAPH,SMC2,ESPL1,CDC6,NDC80,NUP62,HASPIN,SMC3,ENSCAFG00000005299,BUB1,CDC20,TTK,SGO2,UBE2C,ZWINT,GEN1	down_ACOR_VSMC
GO:0006259	2.53E-14	1	75	354	DNA metabolic process	BP	2.04E-11	HMGB1,MCM6,MCM4,USP1,MCM7,RPA1,HMGA1,HNRNPD,MCM3,SMC1A,HINFP,RRM1,POLA1,SLC29A1,TOP3A,TRIP13,PARPBP,POLA2,GINS4,RAD54L,POLE,UBE2T,RFC2,CDK1,RFC5,RFC3,RAD51,FANCI,FANCD2,TIMELESS,FAM129B,AURKB,TK1,TOPBP1,EZH2,GREM1,RFC4,NEK2,FEN1,MND1,PCNA,TONSL,MCM10,UHRF1,GMNN,BRCA1,E2F8,TFRC,CDC6,POLH,NEIL3,DTL,HMGB2,DMAP1,SMC3,CDK2,SMARCB1,RBBP8,SMC6,RBM17,RAD23A,CDC45,SMARCAL1,GRWD1,AIFM1,GLI1,TINF2,H2AFX,NUDT1,EXOSC5,MIS18A,HMGN1,APEX1,CCT4,GEN1	down_ACOR_VSMC
GO:0000278	3.82E-14	1	71	331	mitotic cell cycle	BP	2.93E-11	NCAPD2,ANLN,SMC1A,HINFP,UBE2S,ZW10,SMC4,CENPA,ENSCAFG00000017264,TRIP13,SGO1,POLE,FBXO5,RAN,BIRC5,CDK1,RAD51,CASP2,FANCI,AURKB,CDKN2C,TOPBP1,DSN1,EZH2,STMN1,NCAPD3,MAP3K20,NEK2,CDK4,TRIM35,RNASEH2B,PLK3,RCC1,KIF22,CKS2,CDCA8,NCAPH,POC1A,WDR62,E2F8,SMC2,ESPL1,SPC25,CDC6,NDC80,NUP62,DTL,PDXP,IQGAP1,HASPIN,REEP4,SMC3,CDK2,CCNA2,RAB35,STAG1,ENSCAFG00000005299,RBBP8,CLSPN,BUB1,CENPW,CDC20,TTK,KIF11,TADA2A,CCNB3,UBE2C,CDKN3,MTBP,ZWINT,GEN1	down_ACOR_VSMC
GO:0140014	4.26E-14	1	37	110	mitotic nuclear division	BP	3.12E-11	NCAPD2,SMC1A,UBE2S,ZW10,SMC4,ENSCAFG00000017264,TRIP13,SGO1,FBXO5,RAN,BIRC5,AURKB,DSN1,NCAPD3,NEK2,RCC1,KIF22,CDCA8,NCAPH,SMC2,ESPL1,CDC6,NDC80,NUP62,PDXP,HASPIN,SMC3,STAG1,ENSCAFG00000005299,BUB1,CDC20,TTK,KIF11,UBE2C,MTBP,ZWINT,GEN1	down_ACOR_VSMC
GO:0000070	5.22E-14	1	30	75	mitotic sister chromatid segregation	BP	3.66E-11	NCAPD2,SMC1A,ZW10,SMC4,ENSCAFG00000017264,TRIP13,SGO1,FBXO5,RAN,BIRC5,AURKB,DSN1,NCAPD3,NEK2,KIF22,CDCA8,NCAPH,SMC2,ESPL1,CDC6,NDC80,NUP62,HASPIN,ENSCAFG00000005299,BUB1,CDC20,TTK,UBE2C,ZWINT,GEN1	down_ACOR_VSMC
GO:0098813	1.27E-13	1	35	102	nuclear chromosome segregation	BP	8.52E-11	NCAPD2,SMC1A,ZW10,SMC4,ENSCAFG00000017264,NDC1,TRIP13,SGO1,FBXO5,RAN,BIRC5,AURKB,DSN1,NCAPD3,NEK2,FEN1,KIF22,CDCA8,NCAPH,SMC2,ESPL1,CDC6,NDC80,NUP62,HASPIN,SMC3,ENSCAFG00000005299,BUB1,CDC20,TTK,SGO2,KNL1,UBE2C,ZWINT,GEN1	down_ACOR_VSMC
GO:0048285	1.95E-12	1	44	166	organelle fission	BP	1.12E-09	NCAPD2,SMC1A,UBE2S,ZW10,SMC4,ENSCAFG00000017264,NDC1,TRIP13,SGO1,FBXO5,RAN,PSMD13,BIRC5,RAD51,AURKB,DSN1,NCAPD3,NEK2,MND1,RCC1,KIF22,CKS2,CDCA8,NCAPH,SMC2,ESPL1,CDC6,NDC80,NUP62,PDXP,HASPIN,SMC3,STAG1,ENSCAFG00000005299,BUB1,CDC20,TTK,ASPM,SGO2,KIF11,UBE2C,MTBP,ZWINT,GEN1	down_ACOR_VSMC
GO:0006261	2.59E-12	1	27	68	DNA-dependent DNA replication	BP	1.4E-09	MCM6,MCM4,MCM7,MCM3,SMC1A,POLA1,POLA2,GINS4,POLE,RFC2,RFC5,RFC3,RAD51,TOPBP1,RFC4,FEN1,PCNA,TONSL,MCM10,GMNN,E2F8,CDC6,SMC3,CDK2,CDC45,SMARCAL1,GEN1	down_ACOR_VSMC
GO:0006260	6.05E-11	1	31	98	DNA replication	BP	3.15E-08	MCM6,MCM4,MCM7,RPA1,MCM3,SMC1A,RRM1,POLA1,POLA2,GINS4,POLE,RFC2,RFC5,RFC3,RAD51,TOPBP1,RFC4,FEN1,PCNA,TONSL,MCM10,GMNN,E2F8,CDC6,SMC3,CDK2,CDC45,SMARCAL1,GRWD1,GLI1,GEN1	down_ACOR_VSMC

GO:0051321	3.41E-10	1	23	61	meiotic cell cycle	BP	1.57E-07	NCAPD2,SMC1A,SMC4,ENSCAFG00000017264,NDC1,TRIP13,SGO1,FBXO5,PSMD13,CDK1,RAD51,DSN1,MND1,CKS2,NCAPH,SMC2,ESPL1,SMC3,CDC20,TTK,ASPM,SGO2,CCNB3	down_ACOR_VSMC
GO:0071103	6.32E-10	1	26	80	DNA conformation change	BP	2.83E-07	HMGB1,MCM6,MCM4,MCM7,NCAPD2,HMGA1,SMC4,CENPA,TOP3A,GINS4,RAD51,NCAPD3,HMGB3,NCAPH,NOC2L,SMC2,OIP5,IPO4,HIST1H1A,HMGB2,HIST1H2BB,CENPP,GRWD1,HAT1,MIS18A,H3F3B	down_ACOR_VSMC
GO:2000145	1.92E-09	1	54	279	regulation of cell motility	BP	0.0000105	PLK2,TEK,SELP,PRCP,CXCL8,DOCK4,TIE1,ADAMTS9,ATP8A1,AMOT,PDGFB,PODXL,STK10,RELN,MEF2C,DLL4,STC1,GATA3,MMP28,EDN1,RAB11A,ARAP3,NEDD9,PIK3C2A,PPP3CA,MTOR,MAP3K3,BMP2,SLK,CCAR1,ANGPT2,PROX1,ENSCAFG00000010224,MCC,PRKD2,SWAP70,APOD,NCK1,LDB2,FES,JAG1,RAPGEF2,CTNNA1,HMGB1,ITGA6,PDGFD,ARHGDIB,HIF1A,VASH1,PTP4A1,JCAD,ARHGEF2,FBXO5,ZNF609	up_VCAV_EC
GO:0051270	1.96E-09	1	56	296	regulation of cellular component movement	BP	0.0000105	PLK2,TEK,SELP,PRCP,CXCL8,DOCK4,TIE1,ADAMTS9,ATP8A1,AMOT,PDGFB,PODXL,STK10,RELN,PDE4D,MEF2C,DLL4,STC1,GATA3,MMP28,EDN1,RAB11A,ARAP3,NEDD9,PIK3C2A,PPP3CA,MTOR,MAP3K3,BMP2,SLK,CCAR1,ANGPT2,PROX1,ENSCAFG00000010224,STMN1,MCC,PRKD2,SWAP70,APOD,NCK1,LDB2,FES,JAG1,RAPGEF2,CTNNA1,HMGB1,ITGA6,PDGFD,ARHGDIB,HIF1A,VASH1,PTP4A1,JCAD,ARHGEF2,FBXO5,ZNF609	up_VCAV_EC
GO:0071459	2.19E-09	1	9	10	protein localization to chromosome,	BP	8.83E-07	ZW10,CENPA,CDK1,AURKB,NDC80,HASPIN,TTK,KNL1,MTBP	down_ACOR_VSMC
GO:0006974	2.86E-09	1	61	333	cellular response to DNA damage stimulus	BP	0.00000112	HMGB1,USP1,MCM7,RPA1,DDX39A,CBX5,HMGA1,SMC1A,HINFP,NEDD4,WDR76,INTS7,MNDA,POLA1,TRIP13,PARPBP,GINS4,RAD54L,POLE,UBE2T,RFC2,CDK1,RFC5,RFC3,RAD51,CASP2,FANCI,FANCD2,TIMELESS,TOPBP1,MAP3K20,RFC4,FEN1,MND1,RNASEH2B,PLK3,PCNA,TONSL,MCM10,BRCA1,POLH,NEIL3,DTL,SUV39H1,DMAP1,SMARCB1,DDX39B,ENSCAFG00000030087,RBBP8,CLSPN,SMC6,RBM17,RAD23A,SMARCAL1,H2AFX,CDC5L,ATRIP,NUDT1,HMGN1,APEX1,GEN1	down_ACOR_VSMC
GO:0023052	2.92E-09	1	191	1602	signaling	BP	0.0000106	GNGT2,ACKR1,EPHB1,PLK2,EDNRB,TEK,RASGRP3,HCLS1,PCDH12,IL7R,NPR1,ADGRF5,SELP,RGS16,KIT,PRCP,PPP2R5A,CXCL8,PTCHD1,PRKCH,ARHGEF15,SHE,CXCR4,FGD5,ENSCAFG00000007112,DOCK4,CALCRL,TNFAIP3,ADGRL2,MAP3K11,TNIP1,LRRC8D,AMOT,PDGFB,STK10,SH2D3C,BAIAP2L1,TSPAN8,RELN,FGD6,KDR,CXHXorf36,PDE4D,MEF2C,FERMT1,ATF3,CHD7,TIPARP,DLL4,RAPGEF1,TLR4,NLRC5,GPR4,TSPAN15,EIF2B2,GATA3,EDN1,RALB,APLNR,ARAP3,SMAD1,NTN4,SNCAIP,HOMER3,IGSF3,ARHGAP28,PIK3C2A,EGFL7,FIGNL1,PELI1,PPP3CA,RAPGEF3,GUCY1A1,CASP8,NECTIN2,MTOR,ACPP,SNAP23,LRRC8C,PDE7B,ASB9,TSPAN14,PLEKHG1,HHAT,MAP3K3,BMP2,HHEX,SPNS3,CHN2,CAV2,PREX2,SLC12A7,SLK,BMP2K,SH3BP5,RALBP1,PDZD8,GNG11,ARHGAP29,DOCK6,ABCA1,PPP1R2,ERC1,MAPK13,ITPKB,NCKAP1,GDPD5,IFNGR1,RIOK3,ANGPT2,ARHGEF12,IQGAP2,ENSCAFG00000010224,ENSCAFG00000010596,MADD,CTNNAL1,ELMO1,STMN1,TSPAN12,RAP1A,PAK2,MCC,PRKD2,THOC1,SH3GLB1,SMARCA4,TLR3,MFNG,AKAP12,APOD,TSPAN9,NCK1,KCNJ2,OTUD4,WSB1,NET1,FES,JAG1,PITPNM2,GUCY1B1,GRM8,PTGS2,RAPGEF2,PLCG2,CTDSPL2,CREB1,BCL10	up_VCAV_EC
GO:0030334	3.28E-09	1	52	267	regulation of cell migration	BP	0.0000106	PLK2,TEK,SELP,PRCP,CXCL8,DOCK4,TIE1,ADAMTS9,ATP8A1,AMOT,PDGFB,PODXL,STK10,RELN,MEF2C,DLL4,STC1,GATA3,MMP28,EDN1,RAB11A,ARAP3,NEDD9,PIK3C2A,PPP3CA,MTOR,MAP3K3,BMP2,SLK,CCAR1,ANGPT2,PROX1,ENSCAFG00000010224,MCC,PRKD2,SWAP70,APOD,NCK1,LDB2,JAG1,RAPGEF2,HMGB1,ITGA6,PDGFD,ARHGDIB,HIF1A,VASH1,PTP4A1,JCAD,ARHGEF2,FBXO5,ZNF609	up_VCAV_EC

GO:0016477	4.94E-09	1	71	426	cell migration	BP	0.0000133	EPHB1,PLK2,EDNRB,TEK,SELP,KIT,SELE,PRCP,CXCL8,DOCK4,CLEC14A,TIE1,ADAMTS9,ATP8A1,AMOT,PDGFB,PODXL,STK10,RELN,CD34,SELL,PDE4D,MEF2C,FERMT1,DLL4,STC1,GATA3,MMP28,EDN1,RAB11A,ARAP3,NEDD9,SOX18,PIK3C2A,PPP3CA,EPHB4,MTOR,MAP3K3,BMP2,SLK,CCAR1,ANGPT2,PROX1,ENSCAFG00000010224,ELMO1,PAK2,MCC,PRKD2,SWAP70,APOD,NCK1,LDB2,FES,JAG1,RAPGEF2,FMNL3,PRAG1,HMGB1,ARPC5L,ITGA6,PDGFD,ARHGDIB,HIF1A,VASH1,TNFAIP1,PTP4A1,JCAD,ARHGEF2,FSCN1,FBXO5,ZNF609	up_VCAV_EC
GO:0051726	7.2E-09	1	67	393	regulation of cell cycle	BP	0.0000027	SMC1A,HINFP,WDR76,ZW10,INTS7,SLC29A1,PPM1G,TRIP13,FBXO5,BIRC5,CDK1,CASP2,FANCI,NF2,AURKB,CDKN2C,TOPBP1,EZH2,MAP3K20,NEK2,CLIC1,FEN1,CDK4,TRIM35,RNASEH2B,PLK3,RCC1,CKS2,GMNN,POC1A,BRCA1,E2F8,CDC6,NDC80,NUP62,DTL,PDXP,PRR11,PLK4,IQGAP1,SMC3,CDK2,DDX39B,ENSCAFG00000030087,STAG1,ENSCAFG00000005299,CLSPN,BUB1,RBM14,RAD23A,PHACTR4,CDC20,TTK,BAP1,ASPM,CDC25A,KIF11,GLI1,H2AFX,CDC5L,ATRIP,CCNB3,UBE2C,CDKN3,MTBP,ZWINT,GEN1	down_ACOR_VSMC
GO:0045786	8.62E-09	1	42	195	negative regulation of cell cycle	BP	0.00000316	SMC1A,HINFP,WDR76,ZW10,INTS7,SLC29A1,PPM1G,TRIP13,FBXO5,BIRC5,CDK1,CASP2,FANCI,AURKB,CDKN2C,TOPBP1,EZH2,MAP3K20,NEK2,CDK4,TRIM35,RNASEH2B,GMNN,BRCA1,E2F8,NDC80,DTL,PRR11,SMC3,DDX39B,ENSCAFG00000005299,CLSPN,BUB1,RBM14,TTK,H2AFX,CDC5L,ATRIP,CDKN3,MTBP,ZWINT,GEN1	down_ACOR_VSMC
GO:0007165	8.97E-09	1	179	1496	signal transduction	BP	0.0000194	GNGT2,ACKR1,EPHB1,EDNRB,TEK,RASGRP3,HCLS1,IL7R,NPR1,ADGRF5,SELP,RGS16,KIT,PRCP,PPP2R5A,CXCL8,PTCHD1,PRKCH,ARHGEF15,SHE,CXCR4,FGD5,ENSCAFG00000007112,DOCK4,CALCRL,TNFAIP3,ADGRL2,MAP3K11,TNIP1,LRRC8D,AMOT,PDGFB,STK10,SH2D3C,BAIAP2L1,TSPAN8,RELN,FGD6,KDR,CXHXorf36,PDE4D,MEF2C,FERMT1,ATF3,TIPARP,DLL4,RAPGEF1,TLR4,NLRC5,GPR4,TSPAN15,EIF2B2,GATA3,EDN1,RALB,APLNR,ARAP3,SMAD1,HOMER3,IGSF3,ARHGAP28,PIK3C2A,EGFL7,FIGNL1,PELI1,PPP3CA,RAPGEF3,GUCY1A1,CASP8,NECTIN2,MTOR,ACPP,LRRC8C,PDE7B,ASB9,TSPAN14,PLEKHG1,HHAT,MAP3K3,BMP2,HHEX,SPNS3,CHN2,CAV2,PREX2,SLK,BMP2K,SH3BP5,RALBP1,PDZD8,GNG11,ARHGAP29,DOCK6,ABCA1,PPP1R2,ERC1,MAPK13,ITPKB,NCKAP1,GDPD5,IFNGR1,RIOK3,ANGPT2,ARHGEF12,IQGAP2,ENSCAFG00000010224,ENSCAFG00000010596,MADD,CTNNAL1,ELMO1,STMN1,TSPAN12,RAP1A,PAK2,MCC,PRKD2,THOC1,SH3GLB1,SMARCA4,TLR3,MFNG,AKAP12,APOD,TSPAN9,NCK1,OTUD4,WSB1,NET1,FES,JAG1,PITPNM2,GUCY1B1,GRM8,PTGS2,RAPGEF2,PLCG2,CTDSPL2,BCL10,USP28,TAOK1,CTNNA1,CNOT7,RPS6KL1,ADIPOR2,HMGB1,NO	up_VCAV_EC
GO:0007264	9.61E-09	1	39	164	small GTPase mediated signal transduction	BP	0.0000194	RASGRP3,ARHGEF15,FGD5,DOCK4,AMOT,SH2D3C,RELN,FGD6,RAPGEF1,GPR4,RALB,ARAP3,RAPGEF3,PLEKHG1,CHN2,PREX2,RALBP1,DOCK6,ABCA1,ITPKB,NCKAP1,ARHGEF12,MADD,CTNNAL1,ELMO1,STMN1,RAP1A,PAK2,NET1,RAPGEF2,USP28,ENSCAFG00000024183,ARHGDIB,ARHGAP26,ALS2,TNFAIP1,RGL2,ARHGEF2,CDON	up_VCAV_EC
GO:0040012	1.28E-08	1	55	303	regulation of locomotion	BP	0.0000216	PLK2,TEK,SELP,PRCP,CXCL8,CXCR4,DOCK4,TIE1,ADAMTS9,ATP8A1,AMOT,PDGFB,PODXL,STK10,RELN,MEF2C,DLL4,STC1,GATA3,MMP28,EDN1,RAB11A,ARAP3,NEDD9,PIK3C2A,PPP3CA,MTOR,MAP3K3,BMP2,SLK,CCAR1,ANGPT2,PROX1,ENSCAFG00000010224,MCC,PRKD2,SWAP70,APOD,NCK1,LDB2,FES,JAG1,RAPGEF2,CTNNA1,HMGB1,ITGA6,PDGFD,ARHGDIB,HIF1A,VASH1,PTP4A1,JCAD,ARHGEF2,FBXO5,ZNF609	up_VCAV_EC

GO:0007154	1.34E-08	1	190	1622	cell communication	BP	0.0000216	GNGT2,ACKR1,EPHB1,PLK2,EDNRB,TEK,RASGRP3,HCLS1,PCDH12,IL7R,NPR1,ADGRF5,SELP,RGS16,KIT,PRCP,PPP2R5A,CXCL8,PTCHD1,PRKCH,ARHGEF15,SHE,CXCR4,FGD5,ENSCAFG00000007112,DOCK4,CALCRL,TNFAIP3,ADGRL2,MAP3K11,TNIP1,LRRC8D,AMOT,PDGFB,STK10,SH2D3C,BAIAP2L1,TSPAN8,RELN,FGD6,KDR,CXHXorf36,PDE4D,MEF2C,FERMT1,ATF3,CHD7,TIPARP,DLL4,RAPGEF1,TLR4,NLRC5,GPR4,TSPAN15,EIF2B2,GATA3,EDN1,RALB,APLNR,ARAP3,SMAD1,NTN4,SNCAIP,HOMER3,IGSF3,ARHGAP28,PIK3C2A,EGFL7,FIGNL1,PELI1,PPP3CA,RAPGEF3,GUCY1A1,CASP8,NECTIN2,MTOR,ACPP,SNAP23,LRRC8C,PDE7B,ASB9,TSPAN14,PLEKHG1,HHAT,MAP3K3,BMP2,HHEX,SPNS3,CHN2,CAV2,PREX2,SLC12A7,SLK,BMP2K,SH3BP5,RALBP1,PDZD8,GNG11,ARHGAP29,DOCK6,ABCA1,PPP1R2,ERC1,MAPK13,ITPKB,NCKAP1,GDPD5,IFNGR1,RIOK3,ANGPT2,ARHGEF12,IQGAP2,ENSCAFG00000010224,ENSCAFG00000010596,MADD,CTNNAL1,ELMO1,STMN1,TSPAN12,RAP1A,PAK2,MCC,PRKD2,THOC1,SH3GLB1,SMARCA4,TLR3,MFNG,AKAP12,APOD,TSPAN9,NCK1,OTUD4,WSB1,NET1,FES,JAG1,PITPNM2,GUCY1B1,GRM8,PTGS2,RAPGEF2,PLCG2,CTDSPL2,CREB1,BCL10,USP28	up_VCAV_EC
GO:0048870	1.86E-08	1	72	451	cell motility	BP	0.0000245	EPHB1,PLK2,EDNRB,TEK,SELP,KIT,SELE,PRCP,CXCL8,DOCK4,CLEC14A,TIE1,ADAMTS9,ATP8A1,AMOT,PDGFB,PODXL,STK10,RELN,CD34,SELL,PDE4D,MEF2C,FERMT1,DLL4,STC1,GATA3,MMP28,EDN1,RAB11A,ARAP3,NEDD9,SOX18,PIK3C2A,PPP3CA,EPHB4,MTOR,MAP3K3,BMP2,SLK,CCAR1,ANGPT2,PROX1,ENSCAFG00000010224,ELMO1,PAK2,MCC,PRKD2,SWAP70,APOD,NCK1,LDB2,FES,JAG1,RAPGEF2,CTNNA1,FMNL3,PRAG1,HMGB1,ARPC5L,ITGA6,PDGFD,ARHGDIB,HIF1A,VASH1,TNFAIP1,PTP4A1,JCAD,ARHGEF2,FSCN1,FBXO5,ZNF609	up_VCAV_EC
GO:0051674	1.86E-08	1	72	451	localization of cell	BP	0.0000245	EPHB1,PLK2,EDNRB,TEK,SELP,KIT,SELE,PRCP,CXCL8,DOCK4,CLEC14A,TIE1,ADAMTS9,ATP8A1,AMOT,PDGFB,PODXL,STK10,RELN,CD34,SELL,PDE4D,MEF2C,FERMT1,DLL4,STC1,GATA3,MMP28,EDN1,RAB11A,ARAP3,NEDD9,SOX18,PIK3C2A,PPP3CA,EPHB4,MTOR,MAP3K3,BMP2,SLK,CCAR1,ANGPT2,PROX1,ENSCAFG00000010224,ELMO1,PAK2,MCC,PRKD2,SWAP70,APOD,NCK1,LDB2,FES,JAG1,RAPGEF2,CTNNA1,FMNL3,PRAG1,HMGB1,ARPC5L,ITGA6,PDGFD,ARHGDIB,HIF1A,VASH1,TNFAIP1,PTP4A1,JCAD,ARHGEF2,FSCN1,FBXO5,ZNF609	up_VCAV_EC
GO:0060065	0.000000025	1	3	7	uterus development	BP	0.000404	HOXA9,WNT7A,ESR1	up_AORTRT_VSMC
GO:0034501	2.72E-08	1	8	9	protein localization to kinetochore	BP	0.00000913	ZW10,CDK1,AURKB,NDC80,HASPIN,TTK,KNL1,MTBP	down_ACOR_VSMC
GO:0044770	2.96E-08	1	36	161	cell cycle phase transition	BP	0.00000975	HINFP,UBE2S,ZW10,TRIP13,POLE,FBXO5,BIRC5,CDK1,CASP2,FANCI,TIMELESS,CDKN2C,TOPBP1,EZH2,CDK4,RNASEH2B,PLK3,RCC1,CKS2,CDC6,NDC80,DTL,CDK2,CCNA2,ENSCAFG00000005299,RBBP8,CLSPN,BUB1,TTK,CDC25A,GLI1,CCNB3,UBE2C,CDKN3,MTBP,GEN1	down_ACOR_VSMC

GO:0051179	3.96E-08	1	152	1747	localization	BP	0.000213	ARHGEF2,PRICKLE1,VEGFA,SULF1,MYH9,SLC39A7,SHROOM2,NR1H4,FMNL3,PLK3,HSPA5,SERPINE1,ACTN1,HM13,SPARC,ANKRD50,GOLT1B,TRAM1,ERO1A,PTPN14,SLC7A5,ENPP1,ITGA2,SEC24D,JCAD,ENSCAFG00000003477,HYOU1,PTPN11,EXOC5,ATP2B1,FSCN1,SEC23A,MZT1,DPYSL3,LRRC8A,HOOK3,SLC38A1,TRIB3,ILK,PPP1R12A,PRCP,SESN2,SRP54,TMEM87B,REM1,WDR1,MYO1C,ENSCAFG00000031588,CANX,PIK3C2A,MSN,RAB6A,SEL1L,RPGR,KIF5B,LMAN1,HIF1A,NDUFA4L2,CX3CL1,DYNLL1,TRAK1,SSR1,SLC20A1,IWS1,YIPF5,SLC2A1,TNIK,OSBPL9,ZPR1,PTPN2,STAM2,YES1,SMG7,SDC3,XPOT,USO1,SRPRA,SLC25A16,DDX39B,COPG1,ADAM17,AGRN,PALLD,CTNNB1,KPNB1,ANLN,COPB2,SRP72,CRYAB,STARD13,ENSCAFG00000002015,TFRC,APOE,PTP4A1,KPNA4,PLOD3,MMP28,LYN,BNIP3,STRAP,DDIT3,PDS5B,CCNYL1,SLC2A3,TLN1,MMD,CLCN3,OSBPL6,GPR107,SLC7A11,GSK3B,NKAIN1,NEXN,ARMCX3,GBP5,SH3GLB1,TAX1BP3,SLC20A2,MYO1E,SDC4,KPNA1,HTT,REST,ABHD5,CLINT1,ZMYND8,SCAMP1,CALM1,RAB11FIP1,DISC1,KDELR2,AP3S1,PAK2,CLTC,CREB3L2,LRRTM2,INHBA,STX6,ICAM5,ESYT3,SIX4,CDKN1A,NRP1,RHBDF2,ARF4,YWHAH,BLZF1,TNFAIP1,	down_VSAP_EC
GO:1901360	4.42E-08	1	226	2016	organic cyclic compound metabolic process	BP	0.000014	ELK3,PRPSAP1,SQLE,HMGB1,MCM6,RNPS1,LSS,SREBF1,ACLY,MCM4,USP1,IRAK1,MCM7,RPA1,PTBP1,ILF3,DDX39A,CBX5,PPRC1,USP4,SF3B2,ATAD2,HMGA1,EIF4A3,HMGCR,HNRNPUL1,EDC3,NNT,HNRNPD,SREBF2,MCM3,SMC1A,HINFP,VARS,NEDD4,SRSF2,RRM1,MED17,U2AF2,INTS7,POLA1,SLC29A1,MTDH,HEY1,SNRPB2,TOP3A,TRIP13,SF1,PARPBP,POLA2,SRRT,GINS4,NOP56,RAD54L,POLE,DDX23,NAB2,RAN,UBE2T,CDCA7L,FUS,RFC2,BIRC5,MYBBP1A,INSIG1,PRMT1,CAV1,CDK1,RFC5,RFC3,RAD51,FANCI,FANCD2,U2AF1,TIMELESS,THRAP3,TRUB2,ENSCAFG00000018572,FAM129B,TCEA1,PFAS,EDC4,AURKB,NONO,HNRNPL,TK1,TOPBP1,EZH2,GREM1,RFC4,NEK2,FBL,FEN1,SMARCC1,MND1,RNASEH2B,PLK3,SFSWAP,PCNA,DHX33,HOXA7,SMG9,TONSL,MCM10,SF3B4,PHF5A,UHRF1,CKS2,DIS3,GMNN,DHCR24,BRCA1,NOC4L,MVD,WDR62,FASTKD3,NOC2L,E2F8,ELOVL1,FARSA,TFRC,DDX51,ENSCAFG00000015065,PPARD,CDC6,POLH,NEIL3,YBX3,HNRNPF,CYCS,NCL,NUP62,DTL,DDX28,PDXP,FOSL1,SUV39H1,RSL1D1,THUMPD2,RRP1,ZNF367,CACTIN,HMGB2,DMAP1,DHX15,SMC3,CDK2,SMARCB1,DDX39B,CCNA2,ENSCAFG00000005299,RBBP8,EZR,SMC6,SF3A2,ELOVL	down_ACOR_VSMC
GO:0006139	5.29E-08	1	216	1909	nucleobase-containing compound metabolic process	BP	0.0000164	ELK3,PRPSAP1,HMGB1,MCM6,RNPS1,SREBF1,ACLY,MCM4,USP1,IRAK1,MCM7,RPA1,PTBP1,ILF3,DDX39A,CBX5,PPRC1,USP4,SF3B2,ATAD2,HMGA1,EIF4A3,HMGCR,HNRNPUL1,EDC3,NNT,HNRNPD,SREBF2,MCM3,SMC1A,HINFP,VARS,NEDD4,SRSF2,RRM1,MED17,U2AF2,INTS7,POLA1,SLC29A1,MTDH,HEY1,SNRPB2,TOP3A,TRIP13,SF1,PARPBP,POLA2,SRRT,GINS4,NOP56,RAD54L,POLE,DDX23,NAB2,RAN,UBE2T,CDCA7L,FUS,RFC2,BIRC5,MYBBP1A,INSIG1,PRMT1,CAV1,CDK1,RFC5,RFC3,RAD51,FANCI,FANCD2,U2AF1,TIMELESS,THRAP3,TRUB2,FAM129B,TCEA1,PFAS,EDC4,AURKB,NONO,HNRNPL,TK1,TOPBP1,EZH2,GREM1,RFC4,NEK2,FBL,FEN1,SMARCC1,MND1,RNASEH2B,PLK3,SFSWAP,PCNA,DHX33,HOXA7,SMG9,TONSL,MCM10,SF3B4,PHF5A,UHRF1,CKS2,DIS3,GMNN,BRCA1,NOC4L,MVD,WDR62,FASTKD3,NOC2L,E2F8,ELOVL1,FARSA,TFRC,DDX51,ENSCAFG00000015065,PPARD,CDC6,POLH,NEIL3,YBX3,HNRNPF,CYCS,NCL,NUP62,DTL,DDX28,FOSL1,SUV39H1,RSL1D1,THUMPD2,RRP1,ZNF367,CACTIN,HMGB2,DMAP1,DHX15,SMC3,CDK2,SMARCB1,DDX39B,CCNA2,ENSCAFG00000005299,RBBP8,EZR,SMC6,SF3A2,ELOVL6,DHX38,RBM14,RBM17,RAD23A,OSGEP,CYC1,	down_ACOR_VSMC

GO:0010564	5.65E-08	1	43	217	regulation of cell cycle process	BP	0.0000171	SMC1A,ZW10,SLC29A1,TRIP13,FBXO5,BIRC5,CDK1,CASP2,FANCI,AURKB,TOPBP1,EZH2,NEK2,FEN1,CDK4,RNASEH2B,PLK3,RCC1,POC1A,BRCA1,E2F8,CDC6,NDC80,NUP62,DTL,PDXP,PLK4,SMC3,CDK2,STAG1,ENSCAFG00000005299,CLSPN,BUB1,RBM14,CDC20,TTK,CDC25A,KIF11,GLI1,CCNB3,UBE2C,MTBP,GEN1	down_ACOR_VSMC
GO:1903046	5.77E-08	1	19	55	meiotic cell cycle process	BP	0.0000171	NCAPD2,SMC4,ENSCAFG00000017264,NDC1,TRIP13,SGO1,FBXO5,PSMD13,RAD51,DSN1,MND1,CKS2,NCAPH,SMC2,ESPL1,CDC20,TTK,ASPM,SGO2	down_ACOR_VSMC
GO:0044772	5.81E-08	1	33	144	mitotic cell cycle phase transition	BP	0.0000171	HINFP,UBE2S,ZW10,TRIP13,POLE,FBXO5,BIRC5,CDK1,CASP2,FANCI,CDKN2C,TOPBP1,EZH2,CDK4,RNASEH2B,PLK3,RCC1,CKS2,CDC6,NDC80,DTL,CDK2,CCNA2,ENSCAFG00000005299,RBBP8,CLSPN,BUB1,TTK,CCNB3,UBE2C,CDKN3,MTBP,GEN1	down_ACOR_VSMC
GO:0140013	6.19E-08	1	18	50	meiotic nuclear division	BP	0.0000178	SMC4,ENSCAFG00000017264,NDC1,TRIP13,SGO1,FBXO5,PSMD13,RAD51,DSN1,MND1,CKS2,NCAPH,SMC2,ESPL1,CDC20,TTK,ASPM,SGO2	down_ACOR_VSMC
GO:0046483	6.56E-08	1	219	1948	heterocycle metabolic process	BP	0.0000186	ELK3,PRPSAP1,HMGB1,MCM6,RNPS1,SREBF1,ACLY,MCM4,USP1,IRAK1,MCM7,RPA1,PTBP1,ILF3,DDX39A,CBX5,PPRC1,USP4,SF3B2,ATAD2,HMGA1,EIF4A3,HMGCR,HNRNPUL1,EDC3,NNT,HNRNPD,SREBF2,MCM3,SMC1A,HINFP,VARS,NEDD4,SRSF2,RRM1,MED17,U2AF2,INTS7,POLA1,SLC29A1,MTDH,HEY1,SNRPB2,TOP3A,TRIP13,SF1,PARPBP,POLA2,SRRT,GINS4,NOP56,RAD54L,POLE,DDX23,NAB2,RAN,UBE2T,CDCA7L,FUS,RFC2,BIRC5,MYBBP1A,INSIG1,PRMT1,CAV1,CDK1,RFC5,RFC3,RAD51,FANCI,FANCD2,U2AF1,TIMELESS,THRAP3,TRUB2,FAM129B,TCEA1,PFAS,EDC4,AURKB,NONO,HNRNPL,TK1,TOPBP1,EZH2,GREM1,RFC4,NEK2,FBL,FEN1,SMARCC1,MND1,RNASEH2B,PLK3,SFSWAP,PCNA,DHX33,HOXA7,SMG9,TONSL,MCM10,SF3B4,PHF5A,UHRF1,CKS2,DIS3,GMNN,BRCA1,NOC4L,MVD,WDR62,FASTKD3,NOC2L,E2F8,ELOVL1,FARSA,TFRC,DDX51,ENSCAFG00000015065,PPARD,CDC6,POLH,NEIL3,YBX3,HNRNPF,CYCS,NCL,NUP62,DTL,DDX28,PDXP,FOSL1,SUV39H1,RSL1D1,THUMPD2,RRP1,ZNF367,CACTIN,HMGB2,DMAP1,DHX15,SMC3,CDK2,SMARCB1,DDX39B,CCNA2,ENSCAFG00000005299,RBBP8,EZR,SMC6,SF3A2,ELOVL6,DHX38,RBM14,RBM17,RAD23A,OSGEP	down_ACOR_VSMC
GO:0030326	6.73E-08	1	6	51	embryonic limb morphogenesis	BP	0.000517	HOXA11,HOXA9,PITX1,SALL1,HOXC10,SHOX2	up_VSAP_VSMC
GO:0035113	6.73E-08	1	6	51	embryonic appendage morphogenesis	BP	0.000517	HOXA11,HOXA9,PITX1,SALL1,HOXC10,SHOX2	up_VSAP_VSMC
GO:0006270	7.38E-08	1	11	19	DNA replication initiation	BP	0.0000204	MCM6,MCM4,MCM7,MCM3,POLA2,GINS4,TOPBP1,MCM10,CDC6,CDK2,CDC45	down_ACOR_VSMC
GO:0090304	7.57E-08	1	196	1694	nucleic acid metabolic process	BP	0.0000204	ELK3,HMGB1,MCM6,RNPS1,SREBF1,MCM4,USP1,IRAK1,MCM7,RPA1,PTBP1,ILF3,DDX39A,CBX5,PPRC1,USP4,SF3B2,ATAD2,HMGA1,EIF4A3,HNRNPUL1,EDC3,HNRNPD,SREBF2,MCM3,SMC1A,HINFP,VARS,NEDD4,SRSF2,RRM1,MED17,U2AF2,INTS7,POLA1,SLC29A1,MTDH,HEY1,SNRPB2,TOP3A,TRIP13,SF1,PARPBP,POLA2,SRRT,GINS4,NOP56,RAD54L,POLE,DDX23,NAB2,UBE2T,CDCA7L,FUS,RFC2,BIRC5,MYBBP1A,INSIG1,PRMT1,CAV1,CDK1,RFC5,RFC3,RAD51,FANCI,FANCD2,U2AF1,TIMELESS,THRAP3,TRUB2,FAM129B,TCEA1,EDC4,AURKB,NONO,HNRNPL,TK1,TOPBP1,EZH2,GREM1,RFC4,NEK2,FBL,FEN1,SMARCC1,MND1,RNASEH2B,PLK3,SFSWAP,PCNA,DHX33,HOXA7,SMG9,TONSL,MCM10,SF3B4,PHF5A,UHRF1,CKS2,DIS3,GMNN,BRCA1,NOC4L,WDR62,FASTKD3,NOC2L,E2F8,FARSA,TFRC,DDX51,ENSCAFG00000015065,PPARD,CDC6,POLH,NEIL3,YBX3,HNRNPF,NCL,NUP62,DTL,DDX28,FOSL1,SUV39H1,RSL1D1,THUMPD2,RRP1,ZNF367,CACTIN,HMGB2,DMAP1,DHX15,SMC3,CDK2,SMARCB1,DDX39B,CCNA2,ENSCAFG00000005299,RBBP8,EZR,SMC6,SF3A2,DHX38,RBM14,RBM17,RAD23A,OSGEP,POLR3F,HOXA10,ERF,ENSCAFG00000019620,TCERG1,RARS,CDC45,METT	down_ACOR_VSMC

GO:0006281	7.58E-08	1	42	208	DNA repair	BP	0.0000204	HMGB1,USP1,RPA1,HMGA1,SMC1A,HINFP,POLA1,TRIP13,PARPBP,GINS4,RAD54L,POLE,UBE2T,RFC2,CDK1,RFC5,RFC3,RAD51,FANCI,FANCD2,TIMELESS,RFC4,FEN1,MND1,PCNA,TONSL,BRCA1,POLH,NEIL3,DTL,DMAP1,SMARCB1,RBBP8,SMC6,RBM17,RAD23A,SMARCAL1,H2AFX,NUDT1,HMGN1,APEX1,GEN1	down_ACOR_VSMC
GO:0032501	7.96E-08	1	202	1782	multicellular organismal process	BP	0.0000918	ACKR1,EPHB1,PLK2,EDNRB,HOXB7,TEK,HCLS1,PCDH12,IL7R,GBP5,ADGRF5,SELP,KIT,PRCP,CXCL8,F8,PTCHD1,PRKCH,ARHGEF15,IFRD1,EMCN,DOCK4,CLEC14A,IL6,CALCRL,FLI1,TIE1,TNFAIP3,ADAMTS9,ATP8A1,AMOT,PDGFB,MALL,PODXL,TMEM173,TSPAN8,RELN,VWF,CD34,LAMC2,PDE4D,MEF2C,FERMT1,ATF3,CHD7,KLKB1,TIPARP,DLL4,RAPGEF1,TLR4,QKI,GPR4,STC1,CDH5,EIF2B2,GATA3,EDN1,HOXB8,RAB11A,APLNR,RAB27A,TCF4,SMAD1,NTN4,FOXN2,HOMER3,PTGS1,TFPI,IGSF3,PSME4,ENSCAFG00000003530,IRF8,SOX18,PIK3C2A,TMOD1,FIGNL1,PELI1,PPP3CA,PTPRB,SERINC5,MTSS1,GUCY1A1,TMEM150C,ODC1,ARG2,CASP8,EPHB4,NECTIN2,B3GNT5,MTOR,NAV2,ACPP,TENT2,HOOK3,SNAP23,MMRN1,MAP3K3,BMP2,CDYL,HMG20A,HHEX,CREM,CAV2,PREX2,BMP2K,ENC1,ABCA1,MAPK13,ITPKB,GDPD5,RIOK3,ANGPT2,PROX1,ENSCAFG00000010224,MDFI,ENSCAFG00000010596,ARID4B,STMN1,FLNB,TSPAN12,MMP24,PAK2,MCC,PRKD2,THOC1,SWAP70,SMARCA4,TLR3,MFNG,APOD,BTBD7,PROCR,NCK1,KCNJ2,EXOC5,TIMELESS,HOXB5,LDB2,CD200,SLC25A25,FES,JAG1,HIST1H1A,PTGS2,MMD,TOP1,RAPGEF2,PLCG2,CREB1,BCL10,BASP1,ZC4H2,TAOK1,CT	up_VCAV_EC
GO:0000075	8.17E-08	1	25	91	cell cycle checkpoint	BP	0.0000216	HINFP,WDR76,ZW10,INTS7,TRIP13,BIRC5,CDK1,CASP2,FANCI,AURKB,TOPBP1,MAP3K20,RNASEH2B,NDC80,DTL,DDX39B,ENSCAFG00000005299,CLSPN,BUB1,TTK,H2AFX,CDC5L,ATRIP,ZWINT,GEN1	down_ACOR_VSMC
GO:0006725	0.000000092	1	219	1956	cellular aromatic compound metabolic process	BP	0.0000232	ELK3,PRPSAP1,HMGB1,MCM6,RNPS1,SREBF1,ACLY,MCM4,USP1,IRAK1,MCM7,RPA1,PTBP1,ILF3,DDX39A,CBX5,PPRC1,USP4,SF3B2,ATAD2,HMGA1,EIF4A3,HMGCR,HNRNPUL1,EDC3,NNT,HNRNPD,SREBF2,MCM3,SMC1A,HINFP,VARS,NEDD4,SRSF2,RRM1,MED17,U2AF2,INTS7,POLA1,SLC29A1,MTDH,HEY1,SNRPB2,TOP3A,TRIP13,SF1,PARPBP,POLA2,SRRT,GINS4,NOP56,RAD54L,POLE,DDX23,NAB2,RAN,UBE2T,CDCA7L,FUS,RFC2,BIRC5,MYBBP1A,INSIG1,PRMT1,CAV1,CDK1,RFC5,RFC3,RAD51,FANCI,FANCD2,U2AF1,TIMELESS,THRAP3,TRUB2,FAM129B,TCEA1,PFAS,EDC4,AURKB,NONO,HNRNPL,TK1,TOPBP1,EZH2,GREM1,RFC4,NEK2,FBL,FEN1,SMARCC1,MND1,RNASEH2B,PLK3,SFSWAP,PCNA,DHX33,HOXA7,SMG9,TONSL,MCM10,SF3B4,PHF5A,UHRF1,CKS2,DIS3,GMNN,BRCA1,NOC4L,MVD,WDR62,FASTKD3,NOC2L,E2F8,ELOVL1,FARSA,TFRC,DDX51,ENSCAFG00000015065,PPARD,CDC6,POLH,NEIL3,YBX3,HNRNPF,CYCS,NCL,NUP62,DTL,DDX28,PDXP,FOSL1,SUV39H1,RSL1D1,THUMPD2,RRP1,ZNF367,CACTIN,HMGB2,DMAP1,DHX15,SMC3,CDK2,SMARCB1,DDX39B,CCNA2,ENSCAFG00000005299,RBBP8,EZR,SMC6,SF3A2,ELOVL6,DHX38,RBM14,RBM17,RAD23A,OSGEP	down_ACOR_VSMC
GO:0000375	0.000000103	1	29	120	RNA splicing, via transesterification reactions	BP	0.0000255	RNPS1,DDX39A,USP4,SF3B2,SRSF2,U2AF2,SNRPB2,SF1,DDX23,U2AF1,THRAP3,NONO,HNRNPL,SF3B4,PHF5A,ENSCAFG00000015065,DHX15,DDX39B,SF3A2,DHX38,RBM14,RBM17,PRMT5,WTAP,JMJD6,ENSCAFG00000003222,CDC5L,GEMIN7,SF3A1	down_ACOR_VSMC
GO:0007088	0.000000112	1	20	64	regulation of mitotic nuclear division	BP	0.0000273	SMC1A,ZW10,TRIP13,FBXO5,BIRC5,NEK2,RCC1,CDC6,NDC80,NUP62,PDXP,SMC3,STAG1,ENSCAFG00000005299,BUB1,TTK,KIF11,UBE2C,MTBP,GEN1	down_ACOR_VSMC
GO:1902850	0.000000117	1	18	52	microtubule cytoskeleton organization involved in mitosis	BP	0.0000282	SMC1A,ZW10,CENPA,BIRC5,AURKB,STMN1,NEK2,RCC1,POC1A,WDR62,SPC25,NDC80,NUP62,SMC3,STAG1,ENSCAFG00000005299,CDC20,KIF11	down_ACOR_VSMC

GO:0007052	0.000000123	1	16	42	mitotic spindle organization	BP	0.0000293	SMC1A,BIRC5,AURKB,STMN1,NEK2,RCC1,POC1A,WDR62,SPC25,NDC80,NUP62,SMC3,STAG1,ENSCAFG00000005299,CDC20,KIF11	down_ACOR_VSMC
GO:0008380	0.000000131	1	33	149	RNA splicing	BP	0.0000307	RNPS1,DDX39A,USP4,SF3B2,EIF4A3,SRSF2,U2AF2,SNRPB2,SF1,DDX23,U2AF1,THRAP3,NONO,HNRNPL,SF3B4,PHF5A,WDR62,ENSCAFG00000015065,HNRNPF,DHX15,DDX39B,SF3A2,DHX38,RBM14,RBM17,PRMT5,WTAP,JMJD6,ENSCAFG00000003222,CDC5L,RTCB,GEMIN7,SF3A1	down_ACOR_VSMC
GO:0048598	0.000000141	1	9	207	embryonic morphogenesis	BP	0.000517	HOXA11,TBX15,HOXA9,PITX1,SALL1,HOXC10,IRX5,SHOX2,GJB5	up_VSAP_VSMC
GO:0050896	0.000000149	1	249	2346	response to stimulus	BP	0.00016	GNGT2,ACKR1,EPHB1,PLK2,EDNRB,TEK,RASGRP3,HCLS1,IL7R,GBP5,NPR1,ADGRF5,SELP,RGS16,KIT,SELE,LYZ,PRCP,PPP2R5A,CXCL8,F8,PTCHD1,PRKCH,ARHGEF15,IFRD1,LAPTM5,SHE,CXCR4,FGD5,ENSCAFG00000007112,DOCK4,IL6,CALCRL,TIE1,TNFAIP3,ADAMTS9,PPARG,ADGRL2,MAP3K11,TNIP1,LRRC8D,MX2,AMOT,PDGFB,BCL6B,P2RX7,STK10,SH2D3C,BAIAP2L1,TMEM173,TSPAN8,RELN,VWF,CD34,SELL,FGD6,KDR,CXHXorf36,PDE4D,MEF2C,FERMT1,ATF3,CHD7,KLKB1,TIPARP,DLL4,RAPGEF1,TLR4,NLRC5,GPR4,TSPAN15,EIF2B2,GATA3,MMP28,EDN1,RALB,APLNR,ARAP3,RAB27A,SMAD1,HOMER3,PTGS1,TFPI,IGSF3,PSME4,IRF8,ARHGAP28,PIK3C2A,EGFL7,FIGNL1,PELI1,PPP3CA,SERINC5,YES1,RAPGEF3,MTSS1,GUCY1A1,TMEM150C,ODC1,ARG2,CASP8,NECTIN2,MTOR,ACPP,SNAP23,MMRN1,LRRC8C,PDE7B,ELF4,ASB9,TSPAN14,PLEKHG1,HHAT,MAP3K3,BMP2,TJP1,HHEX,SPNS3,CHN2,CAV2,PREX2,SLK,BMP2K,SH3BP5,RALBP1,PDZD8,ST3GAL6,GNG11,ARHGAP29,DOCK6,ABCA1,PPP1R2,ERC1,MAPK13,ITPKB,NCKAP1,GDPD5,IFNGR1,RIOK3,ANGPT2,ARHGEF12,IQGAP2,ENSCAFG00000010224,ENSCAFG00000010596,MADD,CTNNAL1,ELMO1,STMN1,FLNB,TSPAN	up_VCAV_EC
GO:0048706	0.000000158	1	6	59	embryonic skeletal system development	BP	0.00255	TBX15,EYA1,HOXA9,HOXB7,HOXA7,SIX1	up_VFEM_EC
GO:0035107	0.00000016	1	6	60	appendage morphogenesis	BP	0.000517	HOXA11,HOXA9,PITX1,SALL1,HOXC10,SHOX2	up_VSAP_VSMC
GO:0035108	0.00000016	1	6	60	limb morphogenesis	BP	0.000517	HOXA11,HOXA9,PITX1,SALL1,HOXC10,SHOX2	up_VSAP_VSMC
GO:0006996	0.000000168	1	156	1301	organelle organization	BP	0.0000388	FSCN1,HMGB1,MCM6,SREBF1,MCM4,FLNB,POLE3,MCM7,RPA1,NCAPD2,STXBP6,FMNL3,ATAD2,HMGA1,EDC3,HNRNPD,SMC1A,UBE2S,ZW10,ENSCAFG00000016482,CAPG,SMC4,CENPA,SLC29A1,ENSCAFG00000017264,NDC1,CENPN,TOP3A,TRIP13,SLC25A5,TIMM44,SF1,SGO1,GINS4,SDC1,CTDNEP1,FBXO5,RAN,CFAP298,PRELID1,PSMD13,BIRC5,INSIG1,PRMT1,CAV1,CDK1,RAD51,LIMA1,NF2,AURKB,DSN1,EZH2,STMN1,CENPO,HAUS8,NCAPD3,MAP3K20,NEK2,CHCHD3,FBL,FEN1,SMARCC1,MND1,GAS2L3,PLK3,HMGB3,RCC1,KIF22,UHRF1,CKS2,CDCA8,CENPH,NCAPH,POC1A,BRCA1,WDR62,CENPI,FASTKD3,NOC2L,SMC2,PREB,ESPL1,SPC25,CDC6,NDC80,OIP5,IPO4,HIST1H1A,RAC1,NUP62,EMP2,DDX28,PDXP,PLK4,SUV39H1,HASPIN,TOMM34,IQGAP2,SAMM50,REEP4,HMGB2,DMAP1,CDC42EP4,HIST1H2BB,SMC3,CDK2,SMARCB1,CCNA2,STAG1,ENSCAFG00000005299,EZR,SMC6,SASS6,BUB1,RBM14,CORO1A,NOLC1,CENPW,FOXRED1,PHACTR4,CDC20,TTK,BAP1,ASPM,SGO2,MAST2,ARPC1B,CENPP,GRWD1,ENSCAFG00000010224,PRMT5,ARHGEF2,TRAPPC6A,ENSCAFG00000015189,SUPT16H,KIF11,TADA2A,PMPCA,HAT1,HIST2H2AC,TINF2,H2AFX,EMG1,RTTN,UBE	down_ACOR_VSMC
GO:0007051	0.00000018	1	21	70	spindle organization	BP	0.0000409	SMC1A,FBXO5,BIRC5,AURKB,STMN1,HAUS8,NEK2,RCC1,POC1A,WDR62,ESPL1,SPC25,NDC80,NUP62,SMC3,STAG1,ENSCAFG00000005299,EZR,CDC20,ASPM,KIF11	down_ACOR_VSMC

GO:0035556	0.000000189	1	111	845	intracellular signal transduction	BP	0.00019	EPHB1,EDNRB,TEK,RASGRP3,HCLS1,IL7R,NPR1,SELP,KIT,CXCL8,PRKCH,ARHGEF15,CXCR4,FGD5,ENSCAFG00000007112,DOCK4,CALCRL,TNFAIP3,MAP3K11,TNIP1,AMOT,PDGFB,STK10,SH2D3C,RELN,FGD6,PDE4D,MEF2C,ATF3,RAPGEF1,TLR4,GPR4,GATA3,EDN1,RALB,APLNR,ARAP3,HOMER3,PIK3C2A,FIGNL1,PELI1,PPP3CA,RAPGEF3,GUCY1A1,CASP8,MTOR,ASB9,PLEKHG1,MAP3K3,BMP2,CHN2,PREX2,SLK,SH3BP5,RALBP1,PDZD8,ARHGAP29,DOCK6,ABCA1,ERC1,MAPK13,ITPKB,NCKAP1,RIOK3,ARHGEF12,ENSCAFG00000010596,MADD,CTNNAL1,ELMO1,STMN1,RAP1A,PAK2,PRKD2,TLR3,AKAP12,NCK1,WSB1,NET1,PITPNM2,GUCY1B1,PTGS2,RAPGEF2,PLCG2,BCL10,USP28,TAOK1,CNOT7,HMGB1,NOS2,PMAIP1,RIPK1,LSM14A,SKIL,ENSCAFG00000024183,MCU,PDGFD,ARHGDIB,BMX,HIF1A,ARHGAP26,ALS2,ATRX,ENSCAFG00000002015,TNFAIP1,IVNS1ABP,RGL2,JCAD,ATP2A1,ARHGEF2,PEAR1,CDON	up_VCAV_EC
GO:0050789	0.000000213	1	327	3295	regulation of biological process	BP	0.000202	GNGT2,ACKR1,EPHB1,PLK2,EDNRB,HOXB7,TEK,RASGRP3,HCLS1,IL7R,GBP5,NPR1,ADGRF5,SELP,RGS16,KIT,SELE,PRCP,PPP2R5A,CXCL8,PTCHD1,PRKCH,ARHGEF15,IFRD1,SHE,CXCR4,EMCN,FGD5,ENSCAFG00000007112,DOCK4,CALCRL,FLI1,TIE1,CDA,TNFAIP3,ADAMTS9,PPARG,ADGRL2,MAP3K11,ATP8A1,TNIP1,LRRC8D,MX2,AMOT,PDGFB,BCL6B,PODXL,STK10,SH2D3C,BAIAP2L1,TMEM173,TSPAN8,RELN,CD34,FGD6,KDR,CXHXorf36,PDE4D,MOV10,IWS1,MEF2C,FERMT1,ATF3,CHD7,KLKB1,TIPARP,DLL4,RAPGEF1,WWP1,TLR4,NLRC5,QKI,GPR4,TSPAN15,STC1,EIF2B2,GATA3,MMP28,EDN1,HOXB8,RAB11A,RALB,APLNR,ARAP3,RAB27A,TCF4,SMAD1,CTTNBP2NL,NTN4,SNCAIP,FOXN2,MEOX1,HOMER3,PTGS1,TNFAIP8,TFPI,ENSCAFG00000017103,IGSF3,NEDD9,IRF8,ARHGAP28,SOX18,PIK3C2A,TMOD1,EGFL7,FIGNL1,PELI1,PPP3CA,YES1,RAPGEF3,FAM3C,MTSS1,GUCY1A1,ODC1,SPTLC2,ARG2,CASP8,NECTIN2,MTOR,ACPP,TENT2,SAMD8,HOOK3,LRRC8C,PDE7B,ELF4,ASB9,TSPAN14,PLEKHG1,HHAT,MAP3K3,BMP2,CDYL,HMG20A,HHEX,SPNS3,CREM,CHN2,CAV2,PREX2,TUT7,SLK,BMP2K,SH3BP5,RALBP1,CNOT6,DEPP1,RSC1A1,PDZD8,ENC1,GNG11,ARHGAP29,DOCK6,C	up_VCAV_EC
GO:0051783	0.000000216	1	21	72	regulation of nuclear division	BP	0.0000479	SMC1A,ZW10,TRIP13,FBXO5,BIRC5,NEK2,RCC1,CDC6,NDC80,NUP62,PDXP,SMC3,STAG1,ENSCAFG00000005299,BUB1,CDC20,TTK,KIF11,UBE2C,MTBP,GEN1	down_ACOR_VSMC
GO:0051716	0.000000253	1	219	2001	cellular response to stimulus	BP	0.000227	GNGT2,ACKR1,EPHB1,PLK2,EDNRB,TEK,RASGRP3,HCLS1,IL7R,GBP5,NPR1,ADGRF5,SELP,RGS16,KIT,PRCP,PPP2R5A,CXCL8,PTCHD1,PRKCH,ARHGEF15,LAPTM5,SHE,CXCR4,FGD5,ENSCAFG00000007112,DOCK4,CALCRL,TNFAIP3,PPARG,ADGRL2,MAP3K11,TNIP1,LRRC8D,AMOT,PDGFB,STK10,SH2D3C,BAIAP2L1,TMEM173,TSPAN8,RELN,CD34,FGD6,KDR,CXHXorf36,PDE4D,MEF2C,FERMT1,ATF3,TIPARP,DLL4,RAPGEF1,TLR4,NLRC5,GPR4,TSPAN15,EIF2B2,GATA3,MMP28,EDN1,RALB,APLNR,ARAP3,SMAD1,HOMER3,TFPI,IGSF3,PSME4,IRF8,ARHGAP28,PIK3C2A,EGFL7,FIGNL1,PELI1,PPP3CA,YES1,RAPGEF3,MTSS1,GUCY1A1,TMEM150C,ARG2,CASP8,NECTIN2,MTOR,ACPP,LRRC8C,PDE7B,ASB9,TSPAN14,PLEKHG1,HHAT,MAP3K3,BMP2,TJP1,HHEX,SPNS3,CHN2,CAV2,PREX2,SLK,BMP2K,SH3BP5,RALBP1,PDZD8,ST3GAL6,GNG11,ARHGAP29,DOCK6,ABCA1,PPP1R2,ERC1,MAPK13,ITPKB,NCKAP1,GDPD5,IFNGR1,RIOK3,ANGPT2,ARHGEF12,IQGAP2,ENSCAFG00000010224,ENSCAFG00000010596,MADD,CTNNAL1,ELMO1,STMN1,FLNB,TSPAN12,RAP1A,PAK2,MCC,PRKD2,THOC1,SH3GLB1,SWAP70,SMARCA4,TLR3,MFNG,AKAP12,APOD,TSPAN9,NCK1,TIMELESS,OTUD4,PDIA5,WS	up_VCAV_EC
GO:0048736	0.000000279	1	6	66	appendage development	BP	0.000643	HOXA11,HOXA9,PITX1,SALL1,HOXC10,SHOX2	up_VSAP_VSMC

GO:0060173	0.000000279	1	6	66	limb development	BP	0.000643	HOXA11,HOXA9,PITX1,SALL1,HOXC10,SHOX2	up_VSAP_VSMC
GO:0000377	0.000000306	1	28	119	RNA splicing, via transesterification reactions with bulged adenosine as nucleophile	BP	0.0000659	RNPS1,DDX39A,USP4,SF3B2,SRSF2,U2AF2,SNRPB2,SF1,U2AF1,THRAP3,NONO,HNRNPL,SF3B4,PHF5A,ENSCAFG00000015065,DHX15,DDX39B,SF3A2,DHX38,RBM14,RBM17,PRMT5,WTAP,JMJD6,ENSCAFG00000003222,CDC5L,GEMIN7,SF3A1	down_ACOR_VSMC
GO:0000398	0.000000306	1	28	119	mRNA splicing, via spliceosome	BP	0.0000659	RNPS1,DDX39A,USP4,SF3B2,SRSF2,U2AF2,SNRPB2,SF1,U2AF1,THRAP3,NONO,HNRNPL,SF3B4,PHF5A,ENSCAFG00000015065,DHX15,DDX39B,SF3A2,DHX38,RBM14,RBM17,PRMT5,WTAP,JMJD6,ENSCAFG00000003222,CDC5L,GEMIN7,SF3A1	down_ACOR_VSMC
GO:0048706	0.000000344	1	5	59	embryonic skeletal system development	BP	0.00555	EYA1,HOXA9,TBX15,HOXA11,SHOX2	up_VFEM_VSMC
GO:0034508	0.000000347	1	9	15	centromere complex assembly	BP	0.0000736	CENPA,CENPN,CENPO,CENPH,CENPI,OIP5,CENPW,CENPP,MIS18A	down_ACOR_VSMC
GO:0040011	0.00000037	1	74	505	locomotion	BP	0.000314	EPHB1,PLK2,EDNRB,TEK,SELP,KIT,SELE,PRCP,CXCL8,CXCR4,DOCK4,CLEC14A,TIE1,ADAMTS9,ATP8A1,AMOT,PDGFB,PODXL,STK10,RELN,CD34,SELL,PDE4D,MEF2C,FERMT1,DLL4,STC1,GATA3,MMP28,EDN1,RAB11A,ARAP3,NEDD9,SOX18,PIK3C2A,PPP3CA,EPHB4,MTOR,MAP3K3,BMP2,SPNS3,SLK,CCAR1,ANGPT2,PROX1,ENSCAFG00000010224,ELMO1,PAK2,MCC,PRKD2,SWAP70,APOD,NCK1,LDB2,FES,JAG1,RAPGEF2,CTNNA1,FMNL3,PRAG1,HMGB1,ARPC5L,ITGA6,PDGFD,ARHGDIB,HIF1A,VASH1,TNFAIP1,PTP4A1,JCAD,ARHGEF2,FSCN1,FBXO5,ZNF609	up_VCAV_EC
GO:0034502	0.00000037	1	13	30	protein localization to chromosome	BP	0.0000776	RPA1,ZW10,CENPA,CDK1,AURKB,EZH2,NDC80,HASPIN,TTK,KNL1,TINF2,CCT4,MTBP	down_ACOR_VSMC
GO:0001501	0.000000466	1	8	178	skeletal system development	BP	0.00376	HOXC10,TBX15,EYA1,HOXA9,HOXB7,HOXA7,SIX1,CYP26B1	up_VFEM_EC
GO:0051239	0.000000504	1	110	859	regulation of multicellular organismal process	BP	0.000407	ACKR1,EPHB1,PLK2,EDNRB,HOXB7,TEK,HCLS1,IL7R,GBP5,SELP,KIT,PRCP,CXCL8,PRKCH,ARHGEF15,IFRD1,DOCK4,TIE1,TNFAIP3,ADAMTS9,AMOT,PDGFB,TMEM173,RELN,CD34,PDE4D,MEF2C,FERMT1,CHD7,KLKB1,DLL4,RAPGEF1,TLR4,GPR4,STC1,GATA3,EDN1,HOXB8,RAB11A,APLNR,TCF4,SMAD1,NTN4,HOMER3,TFPI,IRF8,PIK3C2A,PELI1,PPP3CA,GUCY1A1,ARG2,CASP8,MTOR,ACPP,HOOK3,MAP3K3,BMP2,HMG20A,HHEX,BMP2K,MAPK13,ITPKB,GDPD5,RIOK3,ANGPT2,PROX1,TSPAN12,MCC,PRKD2,THOC1,TLR3,APOD,BTBD7,PROCR,NCK1,KCNJ2,CD200,FES,JAG1,PTGS2,MMD,RAPGEF2,PLCG2,CREB1,BCL10,ZC4H2,CTNNA1,GCH1,ADIPOR2,HMGB1,NOS2,SPTBN4,RIPK1,LSM14A,ATP11C,ARHGDIB,CUL4A,NR1D2,HIF1A,KDM3A,VASH1,ZC3H13,JCAD,ATP2A1,ZEB1,ARHGEF2,FBXO5,CDON,TJP2,ZNF609	up_VCAV_EC
GO:0006323	0.000000531	1	18	58	DNA packaging	BP	0.000107	NCAPD2,HMGA1,SMC4,CENPA,NCAPD3,NCAPH,NOC2L,SMC2,OIP5,IPO4,HIST1H1A,HMGB2,HIST1H2BB,CENPP,GRWD1,HAT1,MIS18A,H3F3B	down_ACOR_VSMC
GO:0034976	0.000000534	1	22	112	response to endoplasmic reticulum stress	BP	0.00144	P4HB,ENSCAFG00000013214,HSPA5,HM13,ERO1A,HSP90B1,ENSCAFG00000003477,HYOU1,SESN2,SEL1L,DNAJC10,PTPN2,PDIA4,DNAJB9,UBA5,DDIT3,ATF3,GSK3B,EIF2S1,CREB3L2,TMTC3,USP14	down_VSAP_EC
GO:0001501	0.000000644	1	8	178	skeletal system development	BP	0.0013	HOXA11,TBX15,HOXA9,PITX1,HOXC10,IRX5,SHOX2,HAPLN1	up_VSAP_VSMC
GO:0071824	0.000000879	1	23	92	protein-DNA complex subunit organization	BP	0.000175	HMGB1,CENPA,CENPN,RAD51,CENPO,SMARCC1,GMNN,CENPH,CENPI,OIP5,IPO4,HIST1H1A,HMGB2,HIST1H2BB,SMARCB1,CENPW,CENPP,GRWD1,PSMC1,SUPT16H,HAT1,MIS18A,H3F3B	down_ACOR_VSMC

GO:0050794	0.000000886	1	306	3075	regulation of cellular process	BP	0.000658	GNGT2,ACKR1,EPHB1,PLK2,EDNRB,TEK,RASGRP3,HCLS1,IL7R,GBP5,NPR1,ADGRF5,SELP,RGS16,KIT,SELE,PRCP,PPP2R5A,CXCL8,PTCHD1,PRKCH,ARHGEF15,IFRD1,SHE,CXCR4,FGD5,ENSCAFG00000007112,DOCK4,CALCRL,FLI1,TIE1,CDA,TNFAIP3,ADAMTS9,PPARG,ADGRL2,MAP3K11,ATP8A1,TNIP1,LRRC8D,MX2,AMOT,PDGFB,BCL6B,PODXL,STK10,SH2D3C,BAIAP2L1,TMEM173,TSPAN8,RELN,CD34,FGD6,KDR,CXHXorf36,PDE4D,IWS1,MEF2C,FERMT1,ATF3,CHD7,TIPARP,DLL4,RAPGEF1,WWP1,TLR4,NLRC5,QKI,GPR4,TSPAN15,STC1,EIF2B2,GATA3,MMP28,EDN1,HOXB8,RAB11A,RALB,APLNR,ARAP3,RAB27A,TCF4,SMAD1,SNCAIP,FOXN2,MEOX1,HOMER3,PTGS1,TNFAIP8,ENSCAFG00000017103,IGSF3,NEDD9,IRF8,ARHGAP28,SOX18,PIK3C2A,TMOD1,EGFL7,FIGNL1,PELI1,PPP3CA,YES1,RAPGEF3,FAM3C,MTSS1,GUCY1A1,ODC1,SPTLC2,ARG2,CASP8,NECTIN2,MTOR,ACPP,TENT2,SAMD8,HOOK3,LRRC8C,PDE7B,ELF4,ASB9,TSPAN14,PLEKHG1,HHAT,MAP3K3,BMP2,CDYL,HMG20A,HHEX,SPNS3,CREM,CHN2,CAV2,PREX2,SLK,BMP2K,SH3BP5,RALBP1,CNOT6,DEPP1,RSC1A1,PDZD8,ENC1,GNG11,ARHGAP29,DOCK6,CCAR1,ABCA1,PPP1R2,ERC1,MAPK13,ITPKB,NCKAP1,GDPD5,IFNG	up_VCAV_EC
GO:0045132	0.00000089	1	11	23	meiotic chromosome segregation	BP	0.000175	SMC4,ENSCAFG00000017264,NDC1,TRIP13,SGO1,DSN1,NCAPH,SMC2,ESPL1,TTK,SGO2	down_ACOR_VSMC
GO:0043542	0.000000897	1	22	86	endothelial cell migration	BP	0.000658	PLK2,TEK,PRCP,CLEC14A,ADAMTS9,AMOT,PDGFB,MEF2C,DLL4,STC1,GATA3,SOX18,PIK3C2A,EPHB4,MAP3K3,ANGPT2,PROX1,PRKD2,HMGB1,HIF1A,VASH1,JCAD	up_VCAV_EC
GO:0010948	0.000000975	1	24	96	negative regulation of cell cycle process	BP	0.00019	SMC1A,ZW10,SLC29A1,TRIP13,FBXO5,BIRC5,CDK1,CASP2,FANCI,TOPBP1,EZH2,NEK2,CDK4,E2F8,NDC80,DTL,SMC3,ENSCAFG00000005299,CLSPN,BUB1,RBM14,TTK,MTBP,GEN1	down_ACOR_VSMC
GO:0002376	0.000000985	1	84	622	immune system process	BP	0.000661	EDNRB,HOXB7,HCLS1,IL7R,GBP5,ADGRF5,SELP,KIT,SELE,CXCL8,PRKCH,IL6,FLI1,TNFAIP3,TNIP1,MX2,PDGFB,BCL6B,STK10,TMEM173,CD34,SELL,PDE4D,MEF2C,CHD7,TIPARP,DLL4,TLR4,NLRC5,EIF2B2,GATA3,MMP28,HOXB8,RAB27A,ENSCAFG00000003530,IRF8,PELI1,SERINC5,ARG2,CASP8,NECTIN2,MTOR,TENT2,SNAP23,ELF4,HHEX,ITPKB,RIOK3,FLNB,PRKD2,THOC1,SWAP70,TLR3,MFNG,OAS3,APOD,NCK1,OTUD4,CD200,FES,JAG1,PLCG2,CREB1,BCL10,CNOT7,GCH1,HMGB1,PMAIP1,RIPK1,CRIP2,LSM14A,ITGA6,ATP11C,SKIL,CUL4A,BMX,HIF1A,TNFAIP1,PSMB9,TMOD3,ZEB1,TET2,ARHGEF2,TJP2	up_VCAV_EC
GO:0048706	0.00000103	1	4	59	embryonic skeletal system development	BP	0.0166	TBX15,EYA1,HOXA9,IRX5	up_AFEM_VSMC
GO:0051240	0.00000105	1	71	489	positive regulation of multicellular organismal process	BP	0.000661	EPHB1,PLK2,HOXB7,TEK,HCLS1,IL7R,GBP5,SELP,KIT,CXCL8,PRKCH,TNFAIP3,AMOT,PDGFB,TMEM173,RELN,CD34,PDE4D,MEF2C,FERMT1,CHD7,RAPGEF1,TLR4,GATA3,EDN1,RAB11A,APLNR,TCF4,SMAD1,IRF8,PIK3C2A,PELI1,PPP3CA,CASP8,MTOR,MAP3K3,BMP2,MAPK13,ITPKB,GDPD5,RIOK3,PROX1,PRKD2,TLR3,NCK1,CD200,FES,JAG1,PTGS2,MMD,RAPGEF2,PLCG2,CREB1,BCL10,ZC4H2,GCH1,ADIPOR2,HMGB1,SPTBN4,RIPK1,LSM14A,ATP11C,HIF1A,KDM3A,JCAD,ATP2A1,ZEB1,ARHGEF2,FBXO5,CDON,ZNF609	up_VCAV_EC
GO:0010631	0.00000106	1	27	118	epithelial cell migration	BP	0.000661	PLK2,TEK,KIT,PRCP,CLEC14A,ADAMTS9,AMOT,PDGFB,MEF2C,FERMT1,DLL4,STC1,GATA3,RAB11A,SOX18,PIK3C2A,EPHB4,MTOR,MAP3K3,ANGPT2,PROX1,MCC,PRKD2,HMGB1,HIF1A,VASH1,JCAD	up_VCAV_EC
GO:0090132	0.00000106	1	27	118	epithelium migration	BP	0.000661	PLK2,TEK,KIT,PRCP,CLEC14A,ADAMTS9,AMOT,PDGFB,MEF2C,FERMT1,DLL4,STC1,GATA3,RAB11A,SOX18,PIK3C2A,EPHB4,MTOR,MAP3K3,ANGPT2,PROX1,MCC,PRKD2,HMGB1,HIF1A,VASH1,JCAD	up_VCAV_EC
GO:0090068	0.0000011	1	21	78	positive regulation of cell cycle process	BP	0.000206	BIRC5,CDK1,CASP2,AURKB,EZH2,FEN1,CDK4,POC1A,BRCA1,E2F8,CDC6,NDC80,NUP62,DTL,PLK4,ENSCAFG00000005299,CDC25A,GLI1,UBE2C,MTBP,GEN1	down_ACOR_VSMC

GO:0051304	0.00000115	1	14	38	chromosome separation	BP	0.000211	NCAPD2,ZW10,TRIP13,FBXO5,BIRC5,NCAPH,ESPL1,CDC6,NDC80,ENSCAFG00000005299,BUB1,TTK,UBE2C,GEN1	down_ACOR_VSMC
GO:0048665	0.00000132	1	3	6	neuron fate specification	BP	0.00402	HOXC10,EYA1,SIX1	up_VFEM_EC
GO:0048704	0.00000137	1	5	46	embryonic skeletal system morphogenesis	BP	0.00402	TBX15,EYA1,HOXB7,HOXA7,SIX1	up_VFEM_EC
GO:0001944	0.00000138	1	45	263	vasculature development	BP	0.000776	EPHB1,PLK2,TEK,ADGRF5,KIT,PRCP,CXCL8,ARHGEF15,EMCN,CLEC14A,CALCRL,TIE1,ADAMTS9,AMOT,PDGFB,CD34,MEF2C,CHD7,TIPARP,DLL4,RAPGEF1,GPR4,CDH5,EDN1,APLNR,SMAD1,SOX18,PIK3C2A,PTPRB,EPHB4,MAP3K3,HHEX,ANGPT2,PROX1,TSPAN12,PRKD2,TLR3,JAG1,RAPGEF2,ADIPOR2,HIF1A,LUZP1,VASH1,JCAD,ELK3	up_VCAV_EC
GO:0007265	0.0000014	1	28	121	Ras protein signal transduction	BP	0.000776	RASGRP3,ARHGEF15,FGD5,FGD6,RAPGEF1,GPR4,RALB,ARAP3,PLEKHG1,PREX2,ABCA1,ITPKB,NCKAP1,ARHGEF12,MADD,CTNNAL1,ELMO1,STMN1,RAP1A,PAK2,NET1,RAPGEF2,USP28,ARHGDIB,ALS2,TNFAIP1,RGL2,ARHGEF2	up_VCAV_EC
GO:0071897	0.00000141	1	17	54	DNA biosynthetic process	BP	0.000253	HNRNPD,SLC29A1,POLE,RFC2,RFC5,RFC3,FAM129B,AURKB,TK1,GREM1,RFC4,NEK2,PCNA,POLH,DTL,TINF2,CCT4	down_ACOR_VSMC
GO:0007389	0.00000144	1	7	137	pattern specification process	BP	0.00402	HOXC10,EYA1,HOXA9,HOXB7,HOXA7,SIX1,CYP26B1	up_VFEM_EC
GO:0065007	0.00000149	1	340	3499	biological regulation	BP	0.000803	GNGT2,ACKR1,EPHB1,PLK2,EDNRB,HOXB7,TEK,RASGRP3,HCLS1,IL7R,GBP5,NPR1,ADGRF5,SELP,RGS16,KIT,SELE,PRCP,PPP2R5A,CXCL8,F8,PTCHD1,PRKCH,ARHGEF15,IFRD1,SHE,CP,CXCR4,EMCN,FGD5,ENSCAFG00000007112,DOCK4,CALCRL,FLI1,TIE1,CDA,TNFAIP3,ADAMTS9,PPARG,ADGRL2,MAP3K11,ATP8A1,TNIP1,LRRC8D,MX2,AMOT,PDGFB,BCL6B,PODXL,STK10,SH2D3C,BAIAP2L1,TMEM173,ATP2B1,TSPAN8,RELN,VWF,CD34,FGD6,KDR,CXHXorf36,PDE4D,MOV10,IWS1,MEF2C,FERMT1,ATF3,CHD7,KLKB1,TIPARP,DLL4,RAPGEF1,WWP1,TLR4,NOS3,NLRC5,QKI,GPR4,TSPAN15,STC1,EIF2B2,GATA3,MMP28,EDN1,HOXB8,RAB11A,RALB,APLNR,ARAP3,RAB27A,TCF4,SLC39A8,SMAD1,CTTNBP2NL,NTN4,SNCAIP,FOXN2,MEOX1,HOMER3,PTGS1,TNFAIP8,TFPI,ENSCAFG00000017103,IGSF3,NEDD9,IRF8,ARHGAP28,SOX18,PIK3C2A,TMOD1,EGFL7,FIGNL1,PELI1,PPP3CA,YES1,RAPGEF3,FAM3C,MTSS1,GUCY1A1,ODC1,SPTLC2,ARG2,CASP8,NECTIN2,MTOR,NAV2,ACPP,TENT2,SAMD8,HOOK3,SNAP23,MMRN1,LRRC8C,PDE7B,ELF4,ASB9,TSPAN14,PLEKHG1,HHAT,MAP3K3,BMP2,CDYL,HMG20A,HHEX,SPNS3,CREM,CHN2,CAV2,PREX2,TUT7,SLC12A7,SLK,BMP2K,SH3BP5,RALB	up_VCAV_EC
GO:0048598	0.00000149	1	8	207	embryonic morphogenesis	BP	0.00402	HOXC10,TBX15,EYA1,HOXA9,HOXB7,HOXA7,SIX1,CYP26B1	up_VFEM_EC
GO:0034641	0.00000154	1	232	2171	cellular nitrogen compound metabolic process	BP	0.000274	ELK3,PRPSAP1,HMGB1,MCM6,RNPS1,SREBF1,ACLY,MCM4,USP1,IRAK1,MCM7,RPA1,PTBP1,ILF3,DDX39A,CBX5,PPRC1,USP4,SF3B2,ATAD2,HMGA1,EIF4A3,HMGCR,HNRNPUL1,EDC3,NNT,HNRNPD,SREBF2,MCM3,SMC1A,HINFP,VARS,NEDD4,SRSF2,RRM1,THOP1,MED17,U2AF2,INTS7,POLA1,SLC29A1,MTDH,HEY1,RRBP1,SNRPB2,TOP3A,TRIP13,PPP1R15B,SF1,PARPBP,POLA2,SRRT,GINS4,NOP56,RAD54L,POLE,DDX23,NAB2,MRPS18B,RAN,UBE2T,CDCA7L,FUS,RFC2,BIRC5,MYBBP1A,INSIG1,PRMT1,CAV1,CDK1,RFC5,RFC3,RAD51,FANCI,FANCD2,U2AF1,TIMELESS,THRAP3,TRUB2,FAM129B,TCEA1,PFAS,EDC4,AURKB,NONO,HNRNPL,TK1,TOPBP1,EZH2,GREM1,RFC4,NEK2,FBL,FEN1,SMARCC1,MND1,RNASEH2B,PLK3,SFSWAP,PCNA,DHX33,HOXA7,SMG9,TONSL,ARG2,MCM10,SF3B4,PHF5A,UHRF1,CKS2,DIS3,GMNN,BRCA1,NOC4L,MVD,WDR62,FASTKD3,NOC2L,EIF5A,E2F8,ELOVL1,FARSA,TFRC,DDX51,ENSCAFG00000015065,PPARD,CDC6,POLH,NEIL3,YBX3,HNRNPF,CYCS,NCL,NUP62,MRPL37,DTL,DDX28,PDXP,FOSL1,SUV39H1,RSL1D1,THUMPD2,RRP1,ZNF367,CACTIN,HMGB2,GSTO1,DMAP1,DHX15,SMC3,CDK2,SMARCB1,DDX39B,CCNA2,ENSCAFG00000005299,RB	down_ACOR_VSMC

GO:0006810	0.00000155	1	111	1244	transport	BP	0.00313	PRICKLE1,VEGFA,MYH9,SLC39A7,NR1H4,PLK3,HSPA5,SERPINE1,HM13,ANKRD50,GOLT1B,TRAM1,ERO1A,PTPN14,SLC7A5,ENPP1,SEC24D,ENSCAFG00000003477,HYOU1,PTPN11,EXOC5,ATP2B1,SEC23A,LRRC8A,HOOK3,SLC38A1,TRIB3,PPP1R12A,SESN2,SRP54,TMEM87B,REM1,WDR1,MYO1C,ENSCAFG00000031588,CANX,PIK3C2A,MSN,RAB6A,SEL1L,RPGR,KIF5B,LMAN1,HIF1A,NDUFA4L2,DYNLL1,TRAK1,SSR1,SLC20A1,IWS1,YIPF5,SLC2A1,OSBPL9,ZPR1,STAM2,YES1,SMG7,XPOT,USO1,SRPRA,SLC25A16,DDX39B,COPG1,CTNNB1,KPNB1,COPB2,SRP72,CRYAB,TFRC,APOE,KPNA4,LYN,BNIP3,DDIT3,SLC2A3,CLCN3,OSBPL6,GPR107,SLC7A11,GSK3B,NKAIN1,GBP5,SH3GLB1,SLC20A2,MYO1E,SDC4,KPNA1,HTT,REST,CLINT1,SCAMP1,CALM1,RAB11FIP1,KDELR2,AP3S1,CLTC,CREB3L2,LRRTM2,INHBA,STX6,ICAM5,ESYT3,CDKN1A,NRP1,RHBDF2,ARF4,YWHAH,BLZF1,SEC13,RINT1,HOMER1	down_VSAP_EC
GO:0090130	0.00000164	1	27	121	tissue migration	BP	0.00083	PLK2,TEK,KIT,PRCP,CLEC14A,ADAMTS9,AMOT,PDGFB,MEF2C,FERMT1,DLL4,STC1,GATA3,RAB11A,SOX18,PIK3C2A,EPHB4,MTOR,MAP3K3,ANGPT2,PROX1,MCC,PRKD2,HMGB1,HIF1A,VASH1,JCAD	up_VCAV_EC
GO:0051056	0.00000165	1	24	87	regulation of small GTPase mediated signal transduction	BP	0.00083	ARHGEF15,FGD5,AMOT,RELN,FGD6,RAPGEF1,GPR4,ARAP3,PLEKHG1,CHN2,PREX2,ABCA1,ITPKB,ARHGEF12,MADD,STMN1,NET1,ARHGDIB,ARHGAP26,ALS2,TNFAIP1,RGL2,ARHGEF2,CDON	up_VCAV_EC
GO:1901987	0.0000018	1	25	107	regulation of cell cycle phase transition	BP	0.000312	ZW10,TRIP13,FBXO5,BIRC5,CDK1,CASP2,FANCI,TOPBP1,EZH2,CDK4,RNASEH2B,CDC6,NDC80,DTL,CDK2,ENSCAFG00000005299,CLSPN,BUB1,TTK,CDC25A,GLI1,CCNB3,UBE2C,MTBP,GEN1	down_ACOR_VSMC
GO:0048731	0.00000198	1	147	1247	system development	BP	0.000943	EPHB1,PLK2,EDNRB,HOXB7,TEK,HCLS1,PCDH12,IL7R,ADGRF5,KIT,PRCP,CXCL8,PTCHD1,PRKCH,ARHGEF15,IFRD1,EMCN,CLEC14A,IL6,CALCRL,FLI1,TIE1,TNFAIP3,ADAMTS9,AMOT,PDGFB,MALL,PODXL,RELN,CD34,LAMC2,MEF2C,FERMT1,ATF3,CHD7,TIPARP,DLL4,RAPGEF1,TLR4,GPR4,STC1,CDH5,EIF2B2,GATA3,EDN1,HOXB8,RAB11A,APLNR,TCF4,SMAD1,NTN4,FOXN2,IGSF3,ENSCAFG00000003530,IRF8,SOX18,PIK3C2A,TMOD1,PPP3CA,PTPRB,SERINC5,MTSS1,ODC1,ARG2,CASP8,EPHB4,B3GNT5,MTOR,NAV2,TENT2,HOOK3,MAP3K3,BMP2,HMG20A,HHEX,CAV2,PREX2,BMP2K,ENC1,ITPKB,GDPD5,ANGPT2,PROX1,ENSCAFG00000010224,MDFI,ENSCAFG00000010596,ARID4B,STMN1,FLNB,TSPAN12,MMP24,PAK2,PRKD2,THOC1,SWAP70,SMARCA4,TLR3,MFNG,APOD,BTBD7,NCK1,TIMELESS,HOXB5,LDB2,SLC25A25,FES,JAG1,PTGS2,MMD,RAPGEF2,PLCG2,CREB1,BCL10,BASP1,ZC4H2,TAOK1,CTNNA1,ADIPOR2,HMGB1,NUP133,SPTBN4,RIPK1,CRIP2,ITGA6,ATP11C,SKIL,CUL4A,BMX,NR1D2,HIF1A,ALS2,ATRX,ALDH1A3,P2RX2,LUZP1,RBBP6,VASH1,JCAD,TMOD3,ZEB1,TET2,ARHGEF2,FBXO5,CDON,ELK3,TJP2,ZNF609	up_VCAV_EC
GO:1901990	0.00000199	1	23	94	regulation of mitotic cell cycle phase transition	BP	0.000341	ZW10,TRIP13,FBXO5,BIRC5,CDK1,CASP2,FANCI,TOPBP1,EZH2,CDK4,RNASEH2B,CDC6,NDC80,DTL,CDK2,ENSCAFG00000005299,CLSPN,BUB1,TTK,CCNB3,UBE2C,MTBP,GEN1	down_ACOR_VSMC
GO:0043534	0.00000199	1	17	57	blood vessel endothelial cell migration	BP	0.000943	PLK2,PRCP,CLEC14A,AMOT,PDGFB,MEF2C,DLL4,SOX18,PIK3C2A,EPHB4,MAP3K3,ANGPT2,PRKD2,HMGB1,HIF1A,VASH1,JCAD	up_VCAV_EC
GO:0001501	0.00000216	1	5	178	skeletal system development	BP	0.0174	HOXC10,TBX15,EYA1,HOXA9,IRX5	up_AFEM_VSMC

GO:0051234	0.00000222	1	113	1283	establishment of localization	BP	0.00326	PRICKLE1,VEGFA,MYH9,SLC39A7,SHROOM2,NR1H4,PLK3,HSPA5,SERPINE1,HM13,ANKRD50,GOLT1B,TRAM1,ERO1A,PTPN14,SLC7A5,ENPP1,SEC24D,ENSCAFG00000003477,HYOU1,PTPN11,EXOC5,ATP2B1,SEC23A,LRRC8A,HOOK3,SLC38A1,TRIB3,PPP1R12A,SESN2,SRP54,TMEM87B,REM1,WDR1,MYO1C,ENSCAFG00000031588,CANX,PIK3C2A,MSN,RAB6A,SEL1L,RPGR,KIF5B,LMAN1,HIF1A,NDUFA4L2,DYNLL1,TRAK1,SSR1,SLC20A1,IWS1,YIPF5,SLC2A1,OSBPL9,ZPR1,STAM2,YES1,SMG7,XPOT,USO1,SRPRA,SLC25A16,DDX39B,COPG1,CTNNB1,KPNB1,COPB2,SRP72,CRYAB,ENSCAFG00000002015,TFRC,APOE,KPNA4,LYN,BNIP3,DDIT3,SLC2A3,CLCN3,OSBPL6,GPR107,SLC7A11,GSK3B,NKAIN1,GBP5,SH3GLB1,SLC20A2,MYO1E,SDC4,KPNA1,HTT,REST,CLINT1,SCAMP1,CALM1,RAB11FIP1,KDELR2,AP3S1,CLTC,CREB3L2,LRRTM2,INHBA,STX6,ICAM5,ESYT3,CDKN1A,NRP1,RHBDF2,ARF4,YWHAH,BLZF1,SEC13,RINT1,HOMER1	down_VSAP_EC
GO:0048705	0.00000245	1	6	95	skeletal system morphogenesis	BP	0.00566	TBX15,EYA1,HOXB7,HOXA7,SIX1,CYP26B1	up_VFEM_EC
GO:0072358	0.00000253	1	45	268	cardiovascular system development	BP	0.00112	EPHB1,PLK2,TEK,ADGRF5,KIT,PRCP,CXCL8,ARHGEF15,EMCN,CLEC14A,CALCRL,TIE1,ADAMTS9,AMOT,PDGFB,CD34,MEF2C,CHD7,TIPARP,DLL4,RAPGEF1,GPR4,CDH5,EDN1,APLNR,SMAD1,SOX18,PIK3C2A,PTPRB,EPHB4,MAP3K3,HHEX,ANGPT2,PROX1,TSPAN12,PRKD2,TLR3,JAG1,RAPGEF2,ADIPOR2,HIF1A,LUZP1,VASH1,JCAD,ELK3	up_VCAV_EC
GO:0007155	0.00000256	1	54	324	cell adhesion	BP	0.00112	EPHB1,TEK,PCDH12,IL7R,SELP,KIT,SELE,CXCL8,EMCN,PCDH1,ADAMTS9,TNIP1,PODXL,STK10,DSC2,VWF,CD34,SELL,FERMT1,RAPGEF1,LYVE1,CDH5,ESAM,GATA3,COL4A5,NEDD9,PELI1,PPP3CA,ARG2,EPHB4,NECTIN2,BMP2,SLK,DSG2,ITPKB,ANGPT2,CTNNAL1,MMP24,PRKD2,SWAP70,APOD,CDH24,NCK1,CD200,FES,JAG1,BCL10,CTNNA1,PPP1CB,HMGB1,ITGA6,ITGB3,ENSCAFG00000002015,PEAR1	up_VCAV_EC
GO:0001568	0.00000256	1	43	252	blood vessel development	BP	0.00112	EPHB1,PLK2,TEK,ADGRF5,PRCP,CXCL8,EMCN,CLEC14A,CALCRL,TIE1,ADAMTS9,AMOT,PDGFB,CD34,MEF2C,CHD7,TIPARP,DLL4,RAPGEF1,GPR4,CDH5,EDN1,APLNR,SMAD1,SOX18,PIK3C2A,PTPRB,EPHB4,MAP3K3,HHEX,ANGPT2,PROX1,TSPAN12,PRKD2,TLR3,JAG1,RAPGEF2,ADIPOR2,HIF1A,LUZP1,VASH1,JCAD,ELK3	up_VCAV_EC
GO:0031577	0.00000264	1	9	17	spindle checkpoint	BP	0.000448	ZW10,TRIP13,BIRC5,AURKB,NDC80,ENSCAFG00000005299,BUB1,TTK,GEN1	down_ACOR_VSMC
GO:0030071	0.00000276	1	11	26	regulation of mitotic metaphase/anaphase transition	BP	0.000459	ZW10,TRIP13,FBXO5,BIRC5,CDC6,NDC80,ENSCAFG00000005299,BUB1,TTK,UBE2C,GEN1	down_ACOR_VSMC
GO:1902099	0.00000276	1	11	26	regulation of metaphase/anaphase transition of cell cycle	BP	0.000459	ZW10,TRIP13,FBXO5,BIRC5,CDC6,NDC80,ENSCAFG00000005299,BUB1,TTK,UBE2C,GEN1	down_ACOR_VSMC
GO:0051272	0.00000287	1	33	170	positive regulation of cellular component movement	BP	0.00122	PLK2,TEK,SELP,CXCL8,DOCK4,ATP8A1,PDGFB,PODXL,RELN,GATA3,EDN1,RAB11A,NEDD9,PIK3C2A,PPP3CA,MTOR,MAP3K3,BMP2,CCAR1,PROX1,STMN1,PRKD2,SWAP70,RAPGEF2,HMGB1,ITGA6,PDGFD,HIF1A,PTP4A1,JCAD,ARHGEF2,FBXO5,ZNF609	up_VCAV_EC
GO:0022610	0.00000308	1	54	326	biological adhesion	BP	0.00127	EPHB1,TEK,PCDH12,IL7R,SELP,KIT,SELE,CXCL8,EMCN,PCDH1,ADAMTS9,TNIP1,PODXL,STK10,DSC2,VWF,CD34,SELL,FERMT1,RAPGEF1,LYVE1,CDH5,ESAM,GATA3,COL4A5,NEDD9,PELI1,PPP3CA,ARG2,EPHB4,NECTIN2,BMP2,SLK,DSG2,ITPKB,ANGPT2,CTNNAL1,MMP24,PRKD2,SWAP70,APOD,CDH24,NCK1,CD200,FES,JAG1,BCL10,CTNNA1,PPP1CB,HMGB1,ITGA6,ITGB3,ENSCAFG00000002015,PEAR1	up_VCAV_EC
GO:0001501	0.00000321	1	6	178	skeletal system development	BP	0.0139	HOXC10,EYA1,HOXA9,TBX15,HOXA11,SHOX2	up_VFEM_VSMC
GO:0006333	0.00000344	1	17	59	chromatin assembly or disassembly	BP	0.000555	HMGA1,ENSCAFG00000016482,CENPA,SMARCC1,NOC2L,OIP5,IPO4,HIST1H1A,HMGB2,HIST1H2BB,SMARCB1,CENPP,GRWD1,SUPT16H,HAT1,MIS18A,H3F3B	down_ACOR_VSMC

GO:0035115	0.00000363	1	3	12	embryonic forelimb morphogenesis	BP	0.0139	HOXA9,HOXA11,SHOX2	up_VFEM_VSMC
GO:0098609	0.00000369	1	35	181	cell-cell adhesion	BP	0.00149	PCDH12,IL7R,SELP,KIT,SELE,EMCN,PCDH1,TNIP1,PODXL,STK10,DSC2,CD34,SELL,RAPGEF1,CDH5,ESAM,GATA3,PELI1,ARG2,NECTIN2,BMP2,DSG2,ITPKB,MMP24,SWAP70,CDH24,NCK1,CD200,JAG1,BCL10,CTNNA1,HMGB1,ITGA6,ITGB3,PEAR1	up_VCAV_EC
GO:0001944	0.00000371	1	9	263	vasculature development	BP	0.0354	ACVRL1,HOXA3,PDPN,NRP2,HOXA7,ANPEP,SULF1,ELK3,HOXA1	down_ACOR_EC
GO:0035295	0.00000404	1	55	356	tube development	BP	0.00159	EPHB1,PLK2,EDNRB,HOXB7,TEK,ADGRF5,KIT,PRCP,CXCL8,EMCN,CLEC14A,CALCRL,TIE1,ADAMTS9,AMOT,PODXL,CD34,MEF2C,CHD7,TIPARP,DLL4,GPR4,GATA3,EDN1,APLNR,SMAD1,SOX18,PIK3C2A,PTPRB,MTSS1,ARG2,EPHB4,MAP3K3,BMP2,HHEX,ANGPT2,PROX1,TSPAN12,PRKD2,TLR3,TIMELESS,JAG1,RAPGEF2,CREB1,BCL10,ADIPOR2,NUP133,ITGA6,HIF1A,ATRX,LUZP1,VASH1,JCAD,ZEB1,ELK3	up_VCAV_EC
GO:0048514	0.00000435	1	38	214	blood vessel morphogenesis	BP	0.00167	EPHB1,PLK2,TEK,ADGRF5,PRCP,CXCL8,EMCN,CLEC14A,CALCRL,TIE1,ADAMTS9,AMOT,CD34,CHD7,TIPARP,DLL4,GPR4,EDN1,APLNR,SMAD1,SOX18,PIK3C2A,PTPRB,EPHB4,MAP3K3,HHEX,ANGPT2,PROX1,TSPAN12,PRKD2,TLR3,JAG1,RAPGEF2,ADIPOR2,HIF1A,VASH1,JCAD,ELK3	up_VCAV_EC
GO:0072358	0.00000439	1	9	268	cardiovascular system development	BP	0.0354	ACVRL1,HOXA3,PDPN,NRP2,HOXA7,ANPEP,SULF1,ELK3,HOXA1	down_ACOR_EC
GO:0030335	0.00000444	1	32	165	positive regulation of cell migration	BP	0.00167	PLK2,TEK,SELP,CXCL8,DOCK4,ATP8A1,PDGFB,PODXL,RELN,GATA3,EDN1,RAB11A,NEDD9,PIK3C2A,PPP3CA,MTOR,MAP3K3,BMP2,CCAR1,PROX1,PRKD2,SWAP70,RAPGEF2,HMGB1,ITGA6,PDGFD,HIF1A,PTP4A1,JCAD,ARHGEF2,FBXO5,ZNF609	up_VCAV_EC
GO:0007091	0.00000444	1	11	27	metaphase/anaphase transition of mitotic cell cycle	BP	0.000683	ZW10,TRIP13,FBXO5,BIRC5,CDC6,NDC80,ENSCAFG00000005299,BUB1,TTK,UBE2C,GEN1	down_ACOR_VSMC
GO:0010965	0.00000444	1	11	27	regulation of mitotic sister chromatid separation	BP	0.000683	ZW10,TRIP13,FBXO5,BIRC5,CDC6,NDC80,ENSCAFG00000005299,BUB1,TTK,UBE2C,GEN1	down_ACOR_VSMC
GO:0044784	0.00000444	1	11	27	metaphase/anaphase transition of cell cycle	BP	0.000683	ZW10,TRIP13,FBXO5,BIRC5,CDC6,NDC80,ENSCAFG00000005299,BUB1,TTK,UBE2C,GEN1	down_ACOR_VSMC
GO:0065004	0.00000454	1	20	81	protein-DNA complex assembly	BP	0.000691	HMGB1,CENPA,CENPN,RAD51,CENPO,GMNN,CENPH,CENPI,OIP5,IPO4,HIST1H1A,HMGB2,HIST1H2BB,CENPW,CENPP,GRWD1,PSMC1,HAT1,MIS18A,H3F3B	down_ACOR_VSMC
GO:0048598	0.00000457	1	5	207	embryonic morphogenesis	BP	0.0246	HOXC10,TBX15,EYA1,HOXA9,IRX5	up_AFEM_VSMC
GO:0036294	0.00000484	1	11	37	cellular response to decreased oxygen levels	BP	0.00492	VEGFA,P4HB,PLK3,HYOU1,PGK1,PTGS2,HIF1A,BNIP3,FAM162A,BACH1,HIF1AN	down_VSAP_EC
GO:0071456	0.00000484	1	11	37	cellular response to hypoxia	BP	0.00492	VEGFA,P4HB,PLK3,HYOU1,PGK1,PTGS2,HIF1A,BNIP3,FAM162A,BACH1,HIF1AN	down_VSAP_EC
GO:0045841	0.00000499	1	9	18	negative regulation of mitotic	BP	0.000732	ZW10,TRIP13,FBXO5,BIRC5,NDC80,ENSCAFG00000005299,BUB1,TTK,GEN1	down_ACOR_VSMC
GO:1902100	0.00000499	1	9	18	negative regulation of metaphase/anaphase transition of cell cycle	BP	0.000732	ZW10,TRIP13,FBXO5,BIRC5,NDC80,ENSCAFG00000005299,BUB1,TTK,GEN1	down_ACOR_VSMC
GO:1905819	0.00000499	1	9	18	negative regulation of chromosome separation	BP	0.000732	ZW10,TRIP13,FBXO5,BIRC5,NDC80,ENSCAFG00000005299,BUB1,TTK,GEN1	down_ACOR_VSMC
GO:2000816	0.00000499	1	9	18	negative regulation of mitotic sister chromatid	BP	0.000732	ZW10,TRIP13,FBXO5,BIRC5,NDC80,ENSCAFG00000005299,BUB1,TTK,GEN1	down_ACOR_VSMC
GO:0018126	0.00000531	1	7	14	protein hydroxylation	BP	0.00492	P4HB,PLOD2,P4HA1,PLOD3,ASPH,P3H4,HIF1AN	down_VSAP_EC
GO:0001775	0.0000055	1	39	219	cell activation	BP	0.00202	EPHB1,IL7R,ADGRF5,SELP,KIT,CXCL8,F8,IL6,TNFAIP3,PDGFB,VWF,MEF2C,CHD7,DLL4,TLR4,GATA3,RAB27A,PELI1,ARG2,NECTIN2,MTOR,SNAP23,ELF4,HHEX,ITPKB,THOC1,SWAP70,MFNG,NCK1,CD200,FES,PLCG2,BCL10,HMGB1,ATP11C,BMX,ITGB3,ZEB1,PEAR1	up_VCAV_EC
GO:0048704	0.00000563	1	4	46	embryonic skeletal system morphogenesis	BP	0.0139	EYA1,TBX15,HOXA11,SHOX2	up_VFEM_VSMC
GO:0001666	0.0000058	1	15	67	response to hypoxia	BP	0.00492	VEGFA,P4HB,PLOD2,PLK3,HYOU1,PGK1,PTGS2,HIF1A,ADAM17,CRYAB,BNIP3,FAM162A,BACH1,REST,HIF1AN	down_VSAP_EC
GO:0036293	0.0000058	1	15	67	response to decreased oxygen levels	BP	0.00492	VEGFA,P4HB,PLOD2,PLK3,HYOU1,PGK1,PTGS2,HIF1A,ADAM17,CRYAB,BNIP3,FAM162A,BACH1,REST,HIF1AN	down_VSAP_EC

GO:0048706	0.000006	1	5	59	embryonic skeletal system development	BP	0.00968	HOXA11,TBX15,HOXA9,IRX5,SHOX2	up_VSAP_VSMC
GO:0006952	0.00000601	1	49	319	defense response	BP	0.00215	ACKR1,GBP5,SELP,KIT,LYZ,PRCP,CXCL8,F8,IL6,TNFAIP3,TNIP1,MX2,BCL6B,TMEM173,MEF2C,TLR4,NLRC5,GATA3,RAB27A,PTGS1,IRF8,PELI1,SERINC5,ARG2,NECTIN2,SNAP23,ELF4,BMP2,RIOK3,FLNB,TLR3,OAS3,APOD,OTUD4,FES,PTGS2,BCL10,CNOT7,GCH1,HMGB1,NOS2,PMAIP1,LSM14A,BMX,NR1D2,ENSCAFG00000025410,HIF1A,ALS2,ARHGEF2	up_VCAV_EC
GO:0006396	0.00000611	1	56	368	RNA processing	BP	0.000888	RNPS1,PTBP1,DDX39A,USP4,SF3B2,EIF4A3,HNRNPUL1,SRSF2,U2AF2,INTS7,SNRPB2,SF1,SRRT,NOP56,DDX23,U2AF1,THRAP3,TRUB2,NONO,HNRNPL,FBL,SFSWAP,DHX33,SF3B4,PHF5A,DIS3,NOC4L,WDR62,ENSCAFG00000015065,HNRNPF,RSL1D1,THUMPD2,RRP1,DHX15,DDX39B,SF3A2,DHX38,RBM14,RBM17,OSGEP,ENSCAFG00000019620,NOP14,PRMT5,NOP58,ENSCAFG00000015189,WTAP,JMJD6,WDR33,ENSCAFG00000003222,CDC5L,EMG1,RTCB,EXOSC5,GEMIN7,SF3A1,NOB1	down_ACOR_VSMC
GO:0001667	0.00000667	1	29	146	ameboidal-type cell migration	BP	0.00229	PLK2,EDNRB,TEK,KIT,PRCP,CLEC14A,ADAMTS9,AMOT,PDGFB,MEF2C,FERMT1,DLL4,STC1,GATA3,RAB11A,SOX18,PIK3C2A,EPHB4,MTOR,MAP3K3,ANGPT2,PROX1,MCC,PRKD2,HMGB1,ARHGDIB,HIF1A,VASH1,JCAD	up_VCAV_EC
GO:1905818	0.0000067	1	11	28	regulation of chromosome separation	BP	0.000965	ZW10,TRIP13,FBXO5,BIRC5,CDC6,NDC80,ENSCAFG00000005299,BUB1,TTK,UBE2C,GEN1	down_ACOR_VSMC
GO:0035239	0.0000068	1	48	299	tube morphogenesis	BP	0.00229	EPHB1,PLK2,HOXB7,TEK,ADGRF5,PRCP,CXCL8,EMCN,CLEC14A,CALCRL,TIE1,ADAMTS9,AMOT,PODXL,CD34,MEF2C,CHD7,TIPARP,DLL4,GPR4,GATA3,EDN1,APLNR,SMAD1,SOX18,PIK3C2A,PTPRB,MTSS1,EPHB4,MAP3K3,BMP2,HHEX,ANGPT2,PROX1,TSPAN12,PRKD2,TLR3,TIMELESS,JAG1,RAPGEF2,BCL10,ADIPOR2,HIF1A,LUZP1,VASH1,JCAD,ZEB1,ELK3	up_VCAV_EC
GO:0030326	0.00000691	1	4	51	embryonic limb morphogenesis	BP	0.0139	HOXC10,HOXA9,HOXA11,SHOX2	up_VFEM_VSMC
GO:0035113	0.00000691	1	4	51	embryonic appendage morphogenesis	BP	0.0139	HOXC10,HOXA9,HOXA11,SHOX2	up_VFEM_VSMC
GO:2000147	0.00000713	1	32	169	positive regulation of cell motility	BP	0.00235	PLK2,TEK,SELP,CXCL8,DOCK4,ATP8A1,PDGFB,PODXL,RELN,GATA3,EDN1,RAB11A,NEDD9,PIK3C2A,PPP3CA,MTOR,MAP3K3,BMP2,CCAR1,PROX1,PRKD2,SWAP70,RAPGEF2,HMGB1,ITGA6,PDGFD,HIF1A,PTP4A1,JCAD,ARHGEF2,FBXO5,ZNF609	up_VCAV_EC
GO:0009790	0.00000738	1	9	335	embryo development	BP	0.0108	HOXA11,TBX15,HOXA9,PITX1,SALL1,HOXC10,IRX5,SHOX2,GJB5	up_VSAP_VSMC
GO:0009954	0.0000074	1	3	16	proximal/distal pattern formation	BP	0.0139	HOXC10,HOXA9,HOXA11	up_VFEM_VSMC
GO:0048598	0.00000756	1	6	207	embryonic morphogenesis	BP	0.0139	HOXC10,EYA1,HOXA9,TBX15,HOXA11,SHOX2	up_VFEM_VSMC
GO:0035136	0.00000776	1	3	16	forelimb morphogenesis	BP	0.0139	HOXA9,HOXA11,SHOX2	up_VFEM_VSMC
GO:0048514	0.00000777	1	8	214	blood vessel morphogenesis	BP	0.0418	ACVRL1,HOXA3,NRP2,HOXA7,ANPEP,SULF1,ELK3,HOXA1	down_ACOR_EC
GO:0000226	0.00000801	1	36	197	microtubule cytoskeleton organization	BP	0.00112	SMC1A,ZW10,CENPA,FBXO5,BIRC5,CDK1,AURKB,STMN1,HAUS8,NEK2,GAS2L3,PLK3,RCC1,POC1A,WDR62,ESPL1,SPC25,NDC80,RAC1,NUP62,PLK4,SMC3,CDK2,STAG1,ENSCAFG00000005299,EZR,SASS6,RBM14,CDC20,ASPM,ENSCAFG00000010224,ARHGEF2,KIF11,TTLL1,GEN1,KIF24	down_ACOR_VSMC
GO:0001525	0.00000837	1	32	173	angiogenesis	BP	0.00265	EPHB1,PLK2,TEK,PRCP,CXCL8,EMCN,CLEC14A,CALCRL,TIE1,ADAMTS9,AMOT,CD34,DLL4,GPR4,EDN1,APLNR,SMAD1,SOX18,PIK3C2A,PTPRB,EPHB4,MAP3K3,HHEX,ANGPT2,TSPAN12,PRKD2,TLR3,ADIPOR2,HIF1A,VASH1,JCAD,ELK3	up_VCAV_EC

GO:0051094	0.00000909	1	57	388	positive regulation of developmental process	BP	0.00282	EPHB1,PLK2,HOXB7,TEK,HCLS1,IL7R,KIT,CXCL8,PRKCH,TNFAIP3,ADAMTS9,PPARG,AMOT,PDGFB,RELN,CD34,MEF2C,CHD7,RAPGEF1,GATA3,EDN1,RAB11A,APLNR,TCF4,SMAD1,NEDD9,PIK3C2A,ARG2,CASP8,MTOR,MAP3K3,BMP2,ITPKB,GDPD5,PROX1,PRKD2,TLR3,NCK1,FES,JAG1,PTGS2,MMD,RAPGEF2,CREB1,ZC4H2,HMGB1,SPTBN4,RIPK1,ATP11C,HIF1A,ENSCAFG00000002015,JCAD,ZEB1,ARHGEF2,FBXO5,CDON,ZNF609	up_VCAV_EC
GO:0007165	9.27579E-06	1	65	1496	signal transduction	BP	0.0566569	SH3TC2,CDH3,NR1H4,BRSK1,PLCL1,CALY,SOX9,RHOU,ARHGAP6,PTH1R,RGS1,NDRG2,AQP1,PORCN,ENSCAFG00000004331,ADAMTS13,EDEM2,FZD5,CCDC3,AGRN,PDE1A,IFI6,GNAO1,DUSP26,DIXDC1,RASL11A,PTGIR,NOD2,RGMA,PDE4A,INSYN1,SMAD6,PLCE1,ACTN2,ARHGAP28,ESM1,PAK3,GUCY1B1,GRK7,TNFRSF19,PTGER4,ARHGDIB,DCLK2,ADAMTSL2,MAPK8IP2,RAPGEF4,MAVS,FOXC2,FIGNL1,LRRC15,LRP4,CLSTN1,SH3RF3,LRRC4,CLU,C23H3orf58,PRKACA,STK32C,NRP1,ARHGEF26,PRKCD,GNAZ,RIN2,PYGO2,ADGRL1	up_ACOR_EC
GO:0003002	0.00000935	1	6	118	regionalization	BP	0.0189	HOXC10,HOXA9,HOXB7,HOXA7,SIX1,CYP26B1	up_VFEM_EC
GO:0010594	0.00000947	1	18	71	regulation of endothelial cell migration	BP	0.00288	PLK2,TEK,PRCP,ADAMTS9,PDGFB,MEF2C,DLL4,STC1,GATA3,PIK3C2A,MAP3K3,ANGPT2,PROX1,PRKD2,HMGB1,HIF1A,VASH1,JCAD	up_VCAV_EC
GO:1901342	0.0000101	1	24	114	regulation of vasculature development	BP	0.00302	PLK2,TEK,KIT,CXCL8,TIE1,ADAMTS9,AMOT,PDGFB,CD34,DLL4,GPR4,APLNR,SMAD1,PIK3C2A,MAP3K3,HHEX,ANGPT2,TSPAN12,PRKD2,TLR3,RAPGEF2,HIF1A,VASH1,JCAD	up_VCAV_EC
GO:0051306	0.0000101	1	11	29	mitotic sister chromatid separation	BP	0.00138	ZW10,TRIP13,FBXO5,BIRC5,CDC6,NDC80,ENSCAFG00000005299,BUB1,TTK,UBE2C,GEN1	down_ACOR_VSMC
GO:0045787	0.0000108	1	25	117	positive regulation of cell cycle	BP	0.00146	SLC29A1,BIRC5,CDK1,CASP2,AURKB,EZH2,FEN1,CDK4,CKS2,POC1A,BRCA1,E2F8,CDC6,NDC80,NUP62,DTL,PLK4,DDX39B,ENSCAFG00000005299,RAD23A,CDC25A,GLI1,UBE2C,MTBP,GEN1	down_ACOR_VSMC
GO:2001199	0.000011	1	2	3	negative regulation of dendritic cell	BP	0.176	TMEM176A,TMEM176B	up_VPUL_VSMC
GO:0022414	0.0000114	1	47	294	reproductive process	BP	0.00153	NCAPD2,ACSL4,SMC1A,SMC4,SLC29A1,ENSCAFG00000017264,NDC1,TRIP13,SF1,SGO1,CTDNEP1,FBXO5,PSMD13,CDK1,RAD51,CASP2,AAAS,DSN1,MND1,CKS2,DHCR24,NCAPH,POC1A,E2F8,B4GALT1,SMC2,PPARD,ESPL1,HIST1H1A,EMP2,FOSL1,PLK4,HMGB2,SMC3,SGPL1,CDC20,TTK,ASPM,SGO2,HOXA9,GLI1,H2AFX,RTCB,CCNB3,CCT4,SIX4,TTLL1	down_ACOR_VSMC
GO:0023052	1.14358E-05	0.99999	68	1602	signaling	BP	0.0566569	CNTNAP4,SH3TC2,CDH3,NR1H4,BRSK1,PLCL1,CALY,SOX9,RHOU,ARHGAP6,PTH1R,RGS1,NDRG2,AQP1,PORCN,ENSCAFG00000004331,KCNB1,ADAMTS13,EDEM2,FZD5,CCDC3,AGRN,PDE1A,IFI6,GNAO1,DUSP26,DIXDC1,RASL11A,PTGIR,NOD2,RGMA,PDE4A,INSYN1,SMAD6,PLCE1,ACTN2,ARHGAP28,ESM1,PAK3,GUCY1B1,GRK7,TNFRSF19,PTGER4,ARHGDIB,DCLK2,ADAMTSL2,MAPK8IP2,RAPGEF4,MAVS,FOXC2,FIGNL1,LRRC15,TBX5,LRP4,CLSTN1,SH3RF3,LRRC4,CLU,C23H3orf58,PRKACA,STK32C,NRP1,ARHGEF26,PRKCD,GNAZ,RIN2,PYGO2,ADGRL1	up_ACOR_EC
GO:0035107	0.000012	1	4	60	appendage morphogenesis	BP	0.0177	HOXC10,HOXA9,HOXA11,SHOX2	up_VFEM_VSMC
GO:0035108	0.000012	1	4	60	limb morphogenesis	BP	0.0177	HOXC10,HOXA9,HOXA11,SHOX2	up_VFEM_VSMC
GO:0061333	0.0000121	1	4	35	renal tubule morphogenesis	BP	0.0217	EYA1,HOXB7,SIX1,MTSS1	up_VFEM_EC
GO:0000003	0.0000125	1	47	295	reproduction	BP	0.00167	NCAPD2,ACSL4,SMC1A,SMC4,SLC29A1,ENSCAFG00000017264,NDC1,TRIP13,SF1,SGO1,CTDNEP1,FBXO5,PSMD13,CDK1,RAD51,CASP2,AAAS,DSN1,MND1,CKS2,DHCR24,NCAPH,POC1A,E2F8,B4GALT1,SMC2,PPARD,ESPL1,HIST1H1A,EMP2,FOSL1,PLK4,HMGB2,SMC3,SGPL1,CDC20,TTK,ASPM,SGO2,HOXA9,GLI1,H2AFX,RTCB,CCNB3,CCT4,SIX4,TTLL1	down_ACOR_VSMC

GO:0002764	0.0000128	1	19	80	immune response-regulating signaling pathway	BP	0.00368	KIT,PRKCH,TNFAIP3,TNIP1,PDE4D,MEF2C,TLR4,EIF2B2,GATA3,PELI1,NECTIN2,RIOK3,PRKD2,TLR3,OTUD4,PLCG2,BCL10,HMGB1,LSM14A	up_VCAV_EC
GO:0016126	0.0000129	1	9	20	sterol biosynthetic process	BP	0.00171	SQLE,LSS,SREBF1,HMGCR,INSIG1,ENSCAFG00000018572,DHCR24,MVD,FDFT1	down_ACOR_VSMC
GO:0035023	0.0000132	1	15	46	regulation of Rho protein signal transduction	BP	0.00372	ARHGEF15,FGD5,FGD6,GPR4,ARAP3,PLEKHG1,PREX2,ABCA1,ARHGEF12,STMN1,NET1,ARHGDIB,ALS2,TNFAIP1,ARHGEF2	up_VCAV_EC
GO:0001708	0.0000138	1	3	20	cell fate specification	BP	0.0185	HOXC10,EYA1,HOXA11	up_VFEM_VSMC
GO:0006928	0.000014	1	76	557	movement of cell or subcellular component	BP	0.00389	EPHB1,PLK2,EDNRB,TEK,SELP,KIT,SELE,PRCP,CXCL8,DOCK4,CLEC14A,TIE1,ADAMTS9,ATP8A1,AMOT,PDGFB,KIF21B,PODXL,STK10,RELN,CD34,SELL,PDE4D,MEF2C,FERMT1,DLL4,STC1,GATA3,MMP28,EDN1,RAB11A,KIF3C,ARAP3,NEDD9,SOX18,PIK3C2A,PPP3CA,EPHB4,MTOR,MAP3K3,BMP2,SLK,CCAR1,ANGPT2,PROX1,ENSCAFG00000010224,ELMO1,STMN1,PAK2,MCC,PRKD2,SWAP70,APOD,NCK1,KCNJ2,LDB2,FES,JAG1,RAPGEF2,CTNNA1,FMNL3,PRAG1,HMGB1,ARPC5L,ITGA6,PDGFD,ARHGDIB,HIF1A,VASH1,TNFAIP1,PTP4A1,JCAD,ARHGEF2,FSCN1,FBXO5,ZNF609	up_VCAV_EC
GO:0051383	0.0000142	1	7	12	kinetochore organization	BP	0.00186	SMC4,CENPA,CENPN,CENPH,SMC2,NDC80,CENPW	down_ACOR_VSMC
GO:0042391	0.0000143	1	21	81	regulation of membrane potential	BP	0.0384	FGF13,ACTN2,SLC26A7,KCNE3,TPC3,GJA1,SCARB2,PTPN3,TMEM108,USP53,BNIP3,AKAP6,DMPK,APP,PSEN1,JUP,INSYN1,FXYD1,GNAQ,PARK7,BVES	up_ACOR_VSMC
GO:0001525	0.0000167	1	7	173	angiogenesis	BP	0.0636	ACVRL1,HOXA3,NRP2,HOXA7,ANPEP,SULF1,ELK3	down_ACOR_EC
GO:1903522	0.0000168	1	17	58	regulation of blood circulation	BP	0.0387	TNNT2,FGF13,ADRA1B,KCNE3,GJA1,HRH1,PDE5A,F2RL1,HRH2,DMPK,RNLS,JUP,EDN1,GAA,DOCK5,SLC8A1,BVES	up_ACOR_VSMC
GO:0007094	0.000017	1	8	16	mitotic spindle assembly checkpoint	BP	0.00216	ZW10,TRIP13,BIRC5,NDC80,ENSCAFG00000005299,BUB1,TTK,GEN1	down_ACOR_VSMC
GO:0071173	0.000017	1	8	16	spindle assembly checkpoint	BP	0.00216	ZW10,TRIP13,BIRC5,NDC80,ENSCAFG00000005299,BUB1,TTK,GEN1	down_ACOR_VSMC
GO:0071174	0.000017	1	8	16	mitotic spindle checkpoint	BP	0.00216	ZW10,TRIP13,BIRC5,NDC80,ENSCAFG00000005299,BUB1,TTK,GEN1	down_ACOR_VSMC
GO:0048736	0.0000172	1	4	66	appendage development	BP	0.0198	HOXC10,HOXA9,HOXA11,SHOX2	up_VFEM_VSMC
GO:0060173	0.0000172	1	4	66	limb development	BP	0.0198	HOXC10,HOXA9,HOXA11,SHOX2	up_VFEM_VSMC
GO:0007154	1.7555E-05	0.99999	68	1622	cell communication	BP	0.0566569	CNTNAP4,SH3TC2,CDH3,NR1H4,BRSK1,PLCL1,CALY,SOX9,RHOU,ARHGAP6,PTH1R,RGS1,NDRG2,AQP1,PORCN,ENSCAFG00000004331,KCNB1,ADAMTS13,EDEM2,FZD5,CCDC3,AGRN,PDE1A,IFI6,GNAO1,DUSP26,DIXDC1,RASL11A,PTGIR,NOD2,RGMA,PDE4A,INSYN1,SMAD6,PLCE1,ACTN2,ARHGAP28,ESM1,PAK3,GUCY1B1,GRK7,TNFRSF19,PTGER4,ARHGDIB,DCLK2,ADAMTSL2,MAPK8IP2,RAPGEF4,MAVS,FOXC2,FIGNL1,LRRC15,TBX5,LRP4,CLSTN1,SH3RF3,LRRC4,CLU,C23H3orf58,PRKACA,STK32C,NRP1,ARHGEF26,PRKCD,GNAZ,RIN2,PYGO2,ADGRL1	up_ACOR_EC
GO:2000026	0.0000176	1	75	560	regulation of multicellular organismal development	BP	0.00468	EPHB1,PLK2,EDNRB,HOXB7,TEK,HCLS1,IL7R,KIT,CXCL8,PRKCH,ARHGEF15,IFRD1,TIE1,TNFAIP3,ADAMTS9,AMOT,PDGFB,RELN,CD34,MEF2C,FERMT1,CHD7,DLL4,RAPGEF1,GPR4,GATA3,EDN1,HOXB8,RAB11A,APLNR,TCF4,SMAD1,NTN4,PIK3C2A,PPP3CA,CASP8,MTOR,HOOK3,MAP3K3,BMP2,HMG20A,HHEX,BMP2K,ITPKB,GDPD5,ANGPT2,PROX1,TSPAN12,PRKD2,THOC1,TLR3,BTBD7,NCK1,FES,JAG1,MMD,RAPGEF2,CREB1,ZC4H2,CTNNA1,HMGB1,RIPK1,ATP11C,ARHGDIB,CUL4A,NR1D2,HIF1A,VASH1,JCAD,ZEB1,ARHGEF2,FBXO5,CDON,TJP2,ZNF609	up_VCAV_EC
GO:0045839	0.0000183	1	10	25	negative regulation of mitotic nuclear division	BP	0.0023	ZW10,TRIP13,FBXO5,BIRC5,NDC80,ENSCAFG00000005299,BUB1,TTK,MTBP,GEN1	down_ACOR_VSMC
GO:0070192	0.0000191	1	9	20	chromosome organization involved in meiotic cell	BP	0.0024	NCAPD2,SMC4,NDC1,TRIP13,RAD51,DSN1,NCAPH,SMC2,SGO2	down_ACOR_VSMC
GO:0035115	0.0000193	1	3	12	embryonic forelimb morphogenesis	BP	0.0259	HOXA11,HOXA9,SHOX2	up_VSAP_VSMC

GO:0051983	0.0000201	1	14	47	regulation of chromosome segregation	BP	0.0025	ZW10,TRIP13,FBXO5,BIRC5,NEK2,FEN1,CDC6,NDC80,ENSCAFG00000005299,SMC6,BUB1,TTK,UBE2C,GEN1	down_ACOR_VSMC
GO:0007389	0.0000201	1	5	137	pattern specification process	BP	0.0208	HOXC10,EYA1,HOXA9,EMX2,HOXA11	up_VFEM_VSMC
GO:0007017	0.0000203	1	44	273	microtubule-based process	BP	0.0025	SMC1A,KIF2A,ZW10,CENPA,FBXO5,CFAP298,BIRC5,CDK1,CENPE,AURKB,STMN1,HAUS8,NEK2,GAS2L3,PLK3,RCC1,KIF22,POC1A,WDR62,ESPL1,SPC25,NDC80,RAC1,NUP62,PLK4,TRAK1,KIF4A,SMC3,CDK2,KIF15,STAG1,ENSCAFG00000005299,EZR,SASS6,RBM14,CDC20,ASPM,ENSCAFG00000010224,ARHGEF2,KIF11,TTLL1,KIF21B,GEN1,KIF24	down_ACOR_VSMC
GO:0007266	0.0000203	1	17	64	Rho protein signal transduction	BP	0.0052	ARHGEF15,FGD5,FGD6,GPR4,ARAP3,PLEKHG1,PREX2,ABCA1,ARHGEF12,CTNNAL1,STMN1,PAK2,NET1,ARHGDIB,ALS2,TNFAIP1,ARHGEF2	up_VCAV_EC
GO:0009887	0.0000209	1	9	314	animal organ morphogenesis	BP	0.0636	ACVRL1,HOXA3,NRP2,HOXA7,SULF1,HOXA1,CSGALNACT1,HOXC4,ASPN	down_ACOR_EC
GO:0010632	0.0000211	1	21	95	regulation of epithelial cell migration	BP	0.00531	PLK2,TEK,PRCP,ADAMTS9,PDGFB,MEF2C,DLL4,STC1,GATA3,RAB11A,PIK3C2A,MTOR,MAP3K3,ANGPT2,PROX1,MCC,PRKD2,HMGB1,HIF1A,VASH1,JCAD	up_VCAV_EC
GO:0048663	0.0000211	1	3	13	neuron fate commitment	BP	0.0308	HOXC10,EYA1,SIX1	up_VFEM_EC
GO:0048583	0.0000214	1	127	1095	regulation of response to stimulus	BP	0.00531	EPHB1,PLK2,TEK,HCLS1,IL7R,GBP5,SELP,KIT,PRCP,CXCL8,PRKCH,ARHGEF15,CXCR4,FGD5,TNFAIP3,MAP3K11,TNIP1,AMOT,PDGFB,BCL6B,BAIAP2L1,TMEM173,RELN,CD34,FGD6,CXHXorf36,PDE4D,MEF2C,FERMT1,ATF3,KLKB1,DLL4,RAPGEF1,TLR4,NLRC5,GPR4,EIF2B2,GATA3,MMP28,EDN1,APLNR,ARAP3,HOMER3,TFPI,EGFL7,FIGNL1,PELI1,PPP3CA,GUCY1A1,ARG2,CASP8,NECTIN2,MTOR,ACPP,TSPAN14,PLEKHG1,MAP3K3,BMP2,HHEX,CHN2,CAV2,PREX2,BMP2K,ABCA1,PPP1R2,ITPKB,GDPD5,RIOK3,ANGPT2,ARHGEF12,MADD,STMN1,PAK2,MCC,PRKD2,THOC1,SH3GLB1,SWAP70,SMARCA4,TLR3,MFNG,AKAP12,APOD,NCK1,TIMELESS,OTUD4,CD200,NET1,FES,JAG1,PTGS2,ETAA1,RAPGEF2,PLCG2,CTDSPL2,BCL10,TAOK1,CTNNA1,CNOT7,GCH1,HMGB1,NOS2,PMAIP1,RIPK1,LSM14A,ITGA6,RGS2,SKIL,PDGFD,ARHGDIB,CUL4A,NR1D2,UFSP2,ENSCAFG00000025410,HIF1A,ARHGAP26,ALS2,ENSCAFG00000002015,VASH1,TNFAIP1,IVNS1ABP,RGL2,JCAD,ZEB1,ARHGEF2,PEAR1,CDON	up_VCAV_EC
GO:2001198	0.0000219	1	2	4	regulation of dendritic cell differentiation	BP	0.176	TMEM176A,TMEM176B	up_VPUL_VSMC
GO:0061326	0.0000229	1	4	41	renal tubule development	BP	0.0308	EYA1,HOXB7,SIX1,MTSS1	up_VFEM_EC
GO:0072080	0.0000229	1	4	41	nephron tubule development	BP	0.0308	EYA1,HOXB7,SIX1,MTSS1	up_VFEM_EC
GO:0009952	0.0000236	1	4	66	anterior/posterior pattern specification	BP	0.0224	HOXC10,HOXA9,EMX2,HOXA11	up_VFEM_VSMC
GO:0033046	0.0000243	1	9	21	negative regulation of sister chromatid	BP	0.00292	ZW10,TRIP13,FBXO5,BIRC5,NDC80,ENSCAFG00000005299,BUB1,TTK,GEN1	down_ACOR_VSMC
GO:0033048	0.0000243	1	9	21	negative regulation of mitotic sister chromatid	BP	0.00292	ZW10,TRIP13,FBXO5,BIRC5,NDC80,ENSCAFG00000005299,BUB1,TTK,GEN1	down_ACOR_VSMC
GO:0051985	0.0000243	1	9	21	negative regulation of chromosome segregation	BP	0.00292	ZW10,TRIP13,FBXO5,BIRC5,NDC80,ENSCAFG00000005299,BUB1,TTK,GEN1	down_ACOR_VSMC
GO:0001568	0.0000244	1	8	252	blood vessel development	BP	0.0636	ACVRL1,HOXA3,NRP2,HOXA7,ANPEP,SULF1,ELK3,HOXA1	down_ACOR_EC
GO:0030326	0.0000245	1	3	51	embryonic limb morphogenesis	BP	0.101	HOXC10,HOXA9,WNT7A	up_AORTRT_VSMC
GO:0035113	0.0000245	1	3	51	embryonic appendage morphogenesis	BP	0.101	HOXC10,HOXA9,WNT7A	up_AORTRT_VSMC
GO:0070482	0.0000247	1	15	75	response to oxygen levels	BP	0.0143	VEGFA,P4HB,PLOD2,PLK3,HYOU1,PGK1,PTGS2,HIF1A,ADAM17,CRYAB,BNIP3,FAM162A,BACH1,REST,HIF1AN	down_VSAP_EC
GO:0043627	0.000025	1	2	8	response to estrogen	BP	0.101	WNT7A,ESR1	up_AORTRT_VSMC
GO:0009954	0.0000251	1	5	16	proximal/distal pattern formation	BP	0.241	HOXA9,IRX3,HOXC10,DLL1,GREM1	up_AFEM_EC

GO:0009605	0.0000254	1	71	538	response to external stimulus	BP	0.0062	EPHB1,EDNRB,GBP5,SELP,KIT,LYZ,CXCL8,CXCR4,DOCK4,TNFAIP3,ADAMTS9,TNIP1,MX2,AMOT,PDGFB,BCL6B,P2RX7,BAIAP2L1,TMEM173,RELN,CD34,SELL,PDE4D,MEF2C,ATF3,CHD7,KLKB1,TLR4,NLRC5,GATA3,MMP28,RALB,TFPI,IRF8,PELI1,SERINC5,GUCY1A1,TMEM150C,ODC1,ARG2,CASP8,MTOR,ABCA1,RIOK3,ANGPT2,ENSCAFG00000010596,STMN1,MMP24,PRKD2,SWAP70,TLR3,OAS3,APOD,NCK1,SLC25A25,FES,PTGS2,PLCG2,BCL10,CNOT7,GCH1,HMGB1,NOS2,PMAIP1,LSM14A,ITGA6,NR1D2,ENSCAFG00000025410,ALDH1A3,P2RX2,IVNS1ABP	up_VCAV_EC
GO:0048665	0.0000269	1	2	6	neuron fate specification	BP	0.109	HOXC10,EYA1	up_AFEM_VSMC
GO:0021675	0.0000276	1	4	29	nerve development	BP	0.0636	HOXA3,NRP2,SULF1,HOXA1	down_ACOR_EC
GO:0040017	0.0000288	1	32	180	positive regulation of locomotion	BP	0.00685	PLK2,TEK,SELP,CXCL8,DOCK4,ATP8A1,PDGFB,PODXL,RELN,GATA3,EDN1,RAB11A,NEDD9,PIK3C2A,PPP3CA,MTOR,MAP3K3,BMP2,CCAR1,PROX1,PRKD2,SWAP70,RAPGEF2,HMGB1,ITGA6,PDGFD,HIF1A,PTP4A1,JCAD,ARHGEF2,FBXO5,ZNF609	up_VCAV_EC
GO:0007346	0.0000293	1	36	210	regulation of mitotic cell cycle	BP	0.00348	SMC1A,ZW10,TRIP13,FBXO5,BIRC5,CDK1,CASP2,FANCI,TOPBP1,EZH2,MAP3K20,NEK2,CDK4,TRIM35,RNASEH2B,RCC1,CKS2,CDC6,NDC80,NUP62,DTL,PDXP,IQGAP1,SMC3,CDK2,STAG1,ENSCAFG00000005299,CLSPN,BUB1,TTK,KIF11,CCNB3,UBE2C,MTBP,ZWINT,GEN1	down_ACOR_VSMC
GO:0002757	0.0000306	1	18	78	immune response-activating signal transduction	BP	0.00709	PRKCH,TNFAIP3,TNIP1,PDE4D,MEF2C,TLR4,EIF2B2,GATA3,PELI1,NECTIN2,RIOK3,PRKD2,TLR3,OTUD4,PLCG2,BCL10,HMGB1,LSM14A	up_VCAV_EC
GO:0008217	0.0000308	1	12	38	regulation of blood pressure	BP	0.00709	EDNRB,PRCP,PDGFB,CD34,EDN1,PTGS1,GUCY1A1,NAV2,PTGS2,GCH1,NOS2,P2RX2	up_VCAV_EC
GO:0009653	0.0000312	1	12	773	anatomical structure morphogenesis	BP	0.0365	HOXC10,TBX15,EYA1,HOXA9,HOXB7,HOXA7,SIX1,RADIL,CYP26B1,CDC42EP5,MTSS1,CA9	up_VFEM_EC
GO:0048468	0.0000313	1	58	553	cell development	BP	0.0174	ARHGEF2,SIPA1L1,SULF1,CELF1,SERPINH1,MYH9,ACTN1,SGK1,PTPN11,FLNB,DPYSL3,HOOK3,IFRD1,ILK,FHL2,WDR1,CHSY1,MSN,KIF5B,HIF1A,SLC45A3,TNIK,UBA6,ZPR1,EIF2B1,NEO1,ACTA2,UST,CDH11,CTNNB1,ENSCAFG00000002015,APOE,PLOD3,KLF5,LYN,MMD,GSK3B,VLDLR,NEXN,MYO1E,SDC4,REST,MAPK10,PRMT1,EXT1,DISC1,CDYL,PAK2,INHBA,BTK,SIX4,NRP1,SPINK2,ARF4,YWHAH,KDM3A,MYBPC2,HOMER1	down_VSAP_EC
GO:0048513	0.0000316	1	13	906	animal organ development	BP	0.0365	HOXC10,TBX15,EYA1,HOXA9,TOX,MME,HOXB7,HOXA7,SIX1,MAB21L1,TIAM1,CYP26B1,MTSS1	up_VFEM_EC
GO:2000278	0.0000321	1	12	37	regulation of DNA biosynthetic process	BP	0.00375	HNRNPD,SLC29A1,RFC2,RFC5,RFC3,FAM129B,AURKB,GREM1,RFC4,NEK2,TINF2,CCT4	down_ACOR_VSMC
GO:0048704	0.0000339	1	3	46	embryonic skeletal system morphogenesis	BP	0.109	TBX15,EYA1,IRX5	up_AFEM_VSMC
GO:0048645	0.0000343	1	3	33	animal organ formation	BP	0.184	HOXA3,TBX5,GATA5	up_VPUL_VSMC
GO:0009607	0.0000344	1	37	232	response to biotic stimulus	BP	0.00742	GBP5,LYZ,CXCL8,TNFAIP3,ADAMTS9,TNIP1,MX2,BAIAP2L1,TMEM173,PDE4D,MEF2C,CHD7,TLR4,NLRC5,GATA3,IRF8,PELI1,SERINC5,GUCY1A1,ODC1,ARG2,NECTIN2,ABCA1,RIOK3,STMN1,TLR3,OAS3,NCK1,PLCG2,BCL10,CNOT7,GCH1,HMGB1,NOS2,PMAIP1,LSM14A,IVNS1ABP	up_VCAV_EC
GO:0098742	0.0000346	1	14	44	cell-cell adhesion via plasma-membrane adhesion molecules	BP	0.00742	PCDH12,SELP,SELE,PCDH1,DSC2,SELL,CDH5,ESAM,NECTIN2,BMP2,DSG2,MMP24,CDH24,CD200	up_VCAV_EC
GO:0043207	0.000035	1	35	216	response to external biotic stimulus	BP	0.00742	GBP5,LYZ,CXCL8,TNFAIP3,ADAMTS9,TNIP1,MX2,BAIAP2L1,TMEM173,PDE4D,MEF2C,CHD7,TLR4,NLRC5,GATA3,IRF8,PELI1,SERINC5,GUCY1A1,ODC1,ARG2,ABCA1,RIOK3,STMN1,TLR3,OAS3,NCK1,PLCG2,BCL10,CNOT7,GCH1,NOS2,PMAIP1,LSM14A,IVNS1ABP	up_VCAV_EC
GO:0051707	0.000035	1	35	216	response to other organism	BP	0.00742	GBP5,LYZ,CXCL8,TNFAIP3,ADAMTS9,TNIP1,MX2,BAIAP2L1,TMEM173,PDE4D,MEF2C,CHD7,TLR4,NLRC5,GATA3,IRF8,PELI1,SERINC5,GUCY1A1,ODC1,ARG2,ABCA1,RIOK3,STMN1,TLR3,OAS3,NCK1,PLCG2,BCL10,CNOT7,GCH1,NOS2,PMAIP1,LSM14A,IVNS1ABP	up_VCAV_EC

GO:0031331	0.0000362	1	19	110	positive regulation of cellular catabolic process	BP	0.0188	PRICKLE1,PLK3,SOCS5,TRIB3,SESN2,MSN,HIF1A,MTDH,CNOT7,APOE,BNIP3,GSK3B,CDC37,SH3GLB1,HTT,ABHD5,DISC1,RAD23A,TMTC3	down_VSAP_EC
GO:0009954	0.0000368	1	3	16	proximal/distal pattern formation	BP	0.0395	HOXC10,HOXA9,CYP26B1	up_VFEM_EC
GO:0048568	0.000037	1	6	148	embryonic organ development	BP	0.0458	HOXA11,TBX15,SALL1,IRX5,SHOX2,GJB5	up_VSAP_VSMC
GO:0043535	0.0000387	1	13	45	regulation of blood vessel endothelial cell migration	BP	0.00801	PLK2,PRCP,PDGFB,MEF2C,DLL4,PIK3C2A,MAP3K3,ANGPT2,PRKD2,HMGB1,HIF1A,VASH1,JCAD	up_VCAV_EC
GO:0033045	0.0000402	1	12	38	regulation of sister chromatid segregation	BP	0.0046	ZW10,TRIP13,FBXO5,BIRC5,FEN1,CDC6,NDC80,ENSCAFG00000005299,BUB1,TTK,UBE2C,GEN1	down_ACOR_VSMC
GO:0051052	0.0000405	1	28	145	regulation of DNA metabolic process	BP	0.00461	HMGB1,USP1,HNRNPD,SMC1A,SLC29A1,PARPBP,RFC2,RFC5,RFC3,RAD51,TIMELESS,FAM129B,AURKB,GREM1,RFC4,NEK2,PCNA,UHRF1,GMNN,E2F8,TFRC,SMC3,CDK2,AIFM1,GLI1,TINF2,MIS18A,CCT4	down_ACOR_VSMC
GO:0072009	0.0000408	1	4	48	nephron epithelium development	BP	0.0395	EYA1,HOXB7,SIX1,MTSS1	up_VFEM_EC
GO:1901991	0.000041	1	15	56	negative regulation of mitotic cell cycle phase transition	BP	0.00462	ZW10,TRIP13,FBXO5,BIRC5,CDK1,CASP2,FANCI,TOPBP1,EZH2,NDC80,ENSCAFG00000005299,CLSPN,BUB1,TTK,GEN1	down_ACOR_VSMC
GO:0001822	0.0000416	1	5	98	kidney development	BP	0.0395	EYA1,MME,HOXB7,SIX1,MTSS1	up_VFEM_EC
GO:0035107	0.0000419	1	3	60	appendage morphogenesis	BP	0.113	HOXC10,HOXA9,WNT7A	up_AORTRT_VSMC
GO:0035108	0.0000419	1	3	60	limb morphogenesis	BP	0.113	HOXC10,HOXA9,WNT7A	up_AORTRT_VSMC
GO:0009954	0.0000419	1	3	16	proximal/distal pattern formation	BP	0.0458	HOXA11,HOXA9,HOXC10	up_VSAP_VSMC
GO:0035136	0.0000426	1	3	16	forelimb morphogenesis	BP	0.0458	HOXA11,HOXA9,SHOX2	up_VSAP_VSMC
GO:1900122	0.0000427	1	2	3	positive regulation of receptor binding	BP	0.689	HOXA3,PLCL1	up_APUL_EC
GO:0033047	0.0000443	1	11	33	regulation of mitotic sister chromatid segregation	BP	0.0049	ZW10,TRIP13,FBXO5,BIRC5,CDC6,NDC80,ENSCAFG00000005299,BUB1,TTK,UBE2C,GEN1	down_ACOR_VSMC
GO:1900262	0.0000443	1	4	4	regulation of DNA-directed DNA polymerase	BP	0.0049	RFC2,RFC5,RFC3,RFC4	down_ACOR_VSMC
GO:1900264	0.0000443	1	4	4	positive regulation of DNA-directed DNA polymerase	BP	0.0049	RFC2,RFC5,RFC3,RFC4	down_ACOR_VSMC
GO:0071453	0.0000449	1	11	46	cellular response to oxygen levels	BP	0.0226	VEGFA,P4HB,PLK3,HYOU1,PGK1,PTGS2,HIF1A,BNIP3,FAM162A,BACH1,HIF1AN	down_VSAP_EC
GO:0016071	0.0000459	1	40	248	mRNA metabolic process	BP	0.00504	RNPS1,SREBF1,PTBP1,DDX39A,USP4,SF3B2,EIF4A3,EDC3,HNRNPD,SRSF2,U2AF2,SNRPB2,SF1,U2AF1,THRAP3,EDC4,NONO,HNRNPL,SMG9,SF3B4,PHF5A,FASTKD3,ENSCAFG00000015065,DHX15,DDX39B,SF3A2,DHX38,RBM14,RBM17,ENSCAFG00000019620,PRMT5,ENSCAFG00000015189,WTAP,JMJD6,WDR33,ENSCAFG00000003222,CDC5L,EXOSC5,GEMIN7,SF3A1	down_ACOR_VSMC
GO:0009790	0.0000463	1	5	335	embryo development	BP	0.125	HOXC10,TBX15,EYA1,HOXA9,IRX5	up_AFEM_VSMC
GO:1901988	0.0000467	1	16	63	negative regulation of cell cycle phase transition	BP	0.0051	ZW10,TRIP13,FBXO5,BIRC5,CDK1,CASP2,FANCI,TOPBP1,EZH2,NDC80,DTL,ENSCAFG00000005299,CLSPN,BUB1,TTK,GEN1	down_ACOR_VSMC

GO:0048856	0.0000474	1	167	1533	anatomical structure development	BP	0.00953	EPHB1,PLK2,EDNRB,HOXB7,TEK,HCLS1,PCDH12,IL7R,ADGRF5,KIT,PRCP,CXCL8,PTCHD1,PRKCH,ARHGEF15,IFRD1,EMCN,CLEC14A,IL6,CALCRL,FLI1,TIE1,TNFAIP3,ADAMTS9,AMOT,PDGFB,MALL,PODXL,RELN,CD34,LAMC2,PDE4D,MEF2C,FERMT1,ATF3,CHD7,TIPARP,DLL4,RAPGEF1,TLR4,QKI,GPR4,STC1,CDH5,EIF2B2,GATA3,EDN1,HOXB8,RAB11A,APLNR,ARAP3,TCF4,SMAD1,NTN4,FOXN2,IGSF3,PSME4,ENSCAFG00000003530,NEDD9,IRF8,SOX18,PIK3C2A,TMOD1,PPP3CA,PTPRB,SERINC5,MTSS1,ODC1,ARG2,CASP8,EPHB4,NECTIN2,B3GNT5,MTOR,NAV2,TENT2,HOOK3,MAP3K3,BMP2,CDYL,HMG20A,HHEX,CAV2,PREX2,BMP2K,PDZD8,ENC1,ITPKB,GDPD5,ANGPT2,PROX1,ENSCAFG00000010224,MDFI,ENSCAFG00000010596,ARID4B,STMN1,FLNB,SNX2,TSPAN12,MMP24,PAK2,PRKD2,THOC1,SWAP70,SMARCA4,TLR3,MFNG,APOD,BTBD7,NCK1,EXOC5,TIMELESS,HOXB5,LDB2,SLC25A25,FES,JAG1,PTGS2,MMD,TOP1,RAPGEF2,PLCG2,CREB1,BCL10,BASP1,ZC4H2,TAOK1,CTNNA1,FMNL3,ASF1A,ADIPOR2,HMGB1,NUP133,SPTBN4,RIPK1,CRIP2,AFF4,ITGA6,ATP11C,SKIL,ARHGDIB,CUL4A,BMX,NR1D2,HIF1A,KDM3A,ALS2,ATRX,ALDH1A3,ENSCAFG00000002015,P2RX2,LUZP	up_VCAV_EC
GO:0048869	0.0000476	1	129	1119	cellular developmental process	BP	0.00953	EPHB1,PLK2,EDNRB,HOXB7,TEK,HCLS1,IL7R,ADGRF5,KIT,PRKCH,IFRD1,IL6,FLI1,ADAMTS9,PPARG,AMOT,PDGFB,BCL6B,PODXL,RELN,CD34,PDE4D,MEF2C,ATF3,CHD7,TIPARP,DLL4,RAPGEF1,TLR4,QKI,GPR4,STC1,EIF2B2,GATA3,EDN1,HOXB8,RAB11A,APLNR,ARAP3,RAB27A,TCF4,NTN4,FOXN2,PSME4,ENSCAFG00000003530,NEDD9,IRF8,SOX18,TMOD1,FIGNL1,PPP3CA,MTSS1,ARG2,CASP8,NECTIN2,MTOR,NAV2,TENT2,HOOK3,LRRC8C,ELF4,MAP3K3,BMP2,CDYL,HMG20A,HHEX,CAV2,PREX2,PDZD8,ITPKB,GDPD5,ANGPT2,PROX1,UHRF2,ENSCAFG00000010224,MDFI,ENSCAFG00000010596,ARID4B,STMN1,FLNB,SNX2,MMP24,PAK2,MFNG,APOD,NCK1,HOXB5,FES,JAG1,PTGS2,MMD,RAPGEF2,PLCG2,CREB1,BASP1,ZC4H2,CTNNA1,FMNL3,ASF1A,HMGB1,NUP133,SPTBN4,RIPK1,SMC5,AFF4,ITGA6,ATP11C,SKIL,CUL4A,BMX,NR1D2,HIF1A,KDM3A,ALS2,ATRX,NR4A1,ENSCAFG00000002015,P2RX2,VASH1,TMOD3,ZEB1,TET2,ARHGEF2,FBXO5,CDON,ELK3,TJP2,ZMYM2,ZNF609	up_VCAV_EC
GO:0007166	0.0000478	1	86	686	cell surface receptor signaling pathway	BP	0.00953	ACKR1,TEK,NPR1,ADGRF5,KIT,CXCL8,PTCHD1,PRKCH,CXCR4,CALCRL,TNFAIP3,ADGRL2,PDGFB,BAIAP2L1,TSPAN8,RELN,KDR,PDE4D,MEF2C,FERMT1,ATF3,TIPARP,DLL4,RAPGEF1,NLRC5,TSPAN15,EIF2B2,GATA3,EDN1,SMAD1,IGSF3,EGFL7,PELI1,PPP3CA,CASP8,NECTIN2,ACPP,TSPAN14,HHAT,BMP2,HHEX,BMP2K,ITPKB,GDPD5,IFNGR1,ANGPT2,MADD,STMN1,TSPAN12,PAK2,MCC,PRKD2,SMARCA4,TLR3,MFNG,APOD,TSPAN9,NCK1,OTUD4,FES,JAG1,GRM8,RAPGEF2,PLCG2,CTDSPL2,BCL10,CTNNA1,CNOT7,ADIPOR2,PMAIP1,RIPK1,LSM14A,ITGA6,SKIL,PDGFD,BMX,HIF1A,IQUB,ITGB3,ENSCAFG00000002015,JCAD,ZEB1,ARHGEF2,PEAR1,CDON,TJP2	up_VCAV_EC
GO:0051641	0.0000488	1	79	858	cellular localization	BP	0.0238	PRICKLE1,MYH9,SHROOM2,NR1H4,PLK3,HSPA5,HM13,ANKRD50,TRAM1,ERO1A,PTPN14,SEC24D,HYOU1,PTPN11,EXOC5,SEC23A,MZT1,HOOK3,PPP1R12A,SESN2,SRP54,TMEM87B,MYO1C,CANX,MSN,RAB6A,SEL1L,RPGR,KIF5B,LMAN1,HIF1A,DYNLL1,TRAK1,SSR1,IWS1,YIPF5,TNIK,ZPR1,STAM2,SMG7,XPOT,USO1,SRPRA,DDX39B,COPG1,AGRN,CTNNB1,KPNB1,COPB2,SRP72,CRYAB,APOE,KPNA4,LYN,DDIT3,TLN1,GSK3B,ARMCX3,GBP5,SH3GLB1,TAX1BP3,KPNA1,HTT,CALM1,DISC1,KDELR2,AP3S1,CLTC,CREB3L2,STX6,ESYT3,SIX4,CDKN1A,NRP1,ARF4,YWHAH,BLZF1,SEC13,MID1	down_VSAP_EC
GO:0035295	0.0000497	1	9	356	tube development	BP	0.0898	ACVRL1,HOXA3,PDPN,NRP2,HOXA7,ANPEP,SULF1,ELK3,HOXA1	down_ACOR_EC

GO:0048646	0.0000501	1	9	349	anatomical structure formation involved in	BP	0.0898	ACVRL1,HOXA3,PDPN,NRP2,HOXA7,ANPEP,SULF1,ELK3,HOXA1	down_ACOR_EC
GO:0009790	0.0000517	1	8	335	embryo development	BP	0.0451	HOXC10,TBX15,EYA1,HOXA9,HOXB7,HOXA7,SIX1,CYP26B1	up_VFEM_EC
GO:0050817	0.0000521	1	14	50	coagulation	BP	0.0102	SELP,F8,PDGFB,VWF,CD34,KLKB1,TLR4,RAB27A,TFPI,MMRN1,PROCR,PDGFD,ITGB3,PEAR1	up_VCAV_EC
GO:0048704	0.0000526	1	4	46	embryonic skeletal system morphogenesis	BP	0.053	HOXA11,TBX15,IRX5,SHOX2	up_VSAP_VSMC
GO:0001936	0.0000538	1	13	49	regulation of endothelial cell proliferation	BP	0.0105	PDGFB,MEF2C,DLL4,APLNR,EGFL7,BMP2,CAV2,PROX1,PRKD2,HMGB1,NRAS,VASH1,JCAD	up_VCAV_EC
GO:0072513	0.0000546	1	2	3	positive regulation of secondary heart field cardioblast proliferation	BP	0.0451	EYA1,SIX1	up_VFEM_EC
GO:2000573	0.000055	1	10	28	positive regulation of DNA biosynthetic process	BP	0.00588	HNRNPD,SLC29A1,RFC2,RFC5,RFC3,AURKB,GREM1,RFC4,NEK2,CCT4	down_ACOR_VSMC
GO:0008015	0.0000551	1	26	120	blood circulation	BP	0.111	TNNT2,ADRA1B,KCNE3,HTR2B,NPPB,GJA1,HRH1,PDE5A,SLC2A5,ADCY6,F2RL1,HRH2,CLSTN1,DMPK,PTGDR,TBX20,RNLS,CHD7,JUP,EDN1,NCALD,GAA,DOCK5,ERAP1,SLC8A1,BVES	up_ACOR_VSMC
GO:0090257	0.0000551	1	2	52	regulation of muscle system process	BP	0.438	JUP,GATA5	down_VFEM_VSMC
GO:0044057	0.0000554	1	11	110	regulation of system process	BP	0.244	IRX5,LEP,CRY2,LMCD1,CHRM2,THRB,TMEM100,AVPR1A,TBXA2R,BVES,DMPK	up_AFEM_EC
GO:0072001	0.0000559	1	5	104	renal system development	BP	0.0451	EYA1,MME,HOXB7,SIX1,MTSS1	up_VFEM_EC
GO:0007275	0.0000564	1	152	1370	multicellular organism development	BP	0.0107	EPHB1,PLK2,EDNRB,HOXB7,TEK,HCLS1,PCDH12,IL7R,ADGRF5,KIT,PRCP,CXCL8,PTCHD1,PRKCH,ARHGEF15,IFRD1,EMCN,CLEC14A,IL6,CALCRL,FLI1,TIE1,TNFAIP3,ADAMTS9,AMOT,PDGFB,MALL,PODXL,RELN,CD34,LAMC2,MEF2C,FERMT1,ATF3,CHD7,TIPARP,DLL4,RAPGEF1,TLR4,QKI,GPR4,STC1,CDH5,EIF2B2,GATA3,EDN1,HOXB8,RAB11A,APLNR,TCF4,SMAD1,NTN4,FOXN2,IGSF3,ENSCAFG00000003530,IRF8,SOX18,PIK3C2A,TMOD1,PPP3CA,PTPRB,SERINC5,MTSS1,ODC1,ARG2,CASP8,EPHB4,B3GNT5,MTOR,NAV2,TENT2,HOOK3,MAP3K3,BMP2,HMG20A,HHEX,CAV2,PREX2,BMP2K,ENC1,ITPKB,GDPD5,ANGPT2,PROX1,ENSCAFG00000010224,MDFI,ENSCAFG00000010596,ARID4B,STMN1,FLNB,TSPAN12,MMP24,PAK2,PRKD2,THOC1,SWAP70,SMARCA4,TLR3,MFNG,APOD,BTBD7,NCK1,TIMELESS,HOXB5,LDB2,SLC25A25,FES,JAG1,PTGS2,MMD,TOP1,RAPGEF2,PLCG2,CREB1,BCL10,BASP1,ZC4H2,TAOK1,CTNNA1,ADIPOR2,HMGB1,NUP133,SPTBN4,RIPK1,CRIP2,ITGA6,ATP11C,SKIL,ARHGDIB,CUL4A,BMX,NR1D2,HIF1A,ALS2,ATRX,ALDH1A3,P2RX2,LUZP1,RBBP6,VASH1,JCAD,TMOD3,ZEB1,TET2,ARHGEF2,FBXO5,CDON,ELK3,TJP2,ZMYM2,ZNF609,GINS4	up_VCAV_EC
GO:0010720	0.0000566	1	23	110	positive regulation of cell development	BP	0.0107	PRKCH,RELN,MEF2C,RAPGEF1,EDN1,RAB11A,TCF4,NEDD9,MTOR,BMP2,GDPD5,PROX1,NCK1,FES,MMD,RAPGEF2,ZC4H2,HIF1A,ENSCAFG00000002015,ZEB1,ARHGEF2,CDON,ZNF609	up_VCAV_EC
GO:0048736	0.0000569	1	3	66	appendage development	BP	0.115	HOXC10,HOXA9,WNT7A	up_AORTRT_VSMC
GO:0060173	0.0000569	1	3	66	limb development	BP	0.115	HOXC10,HOXA9,WNT7A	up_AORTRT_VSMC
GO:0051784	0.000059	1	10	28	negative regulation of nuclear division	BP	0.00619	ZW10,TRIP13,FBXO5,BIRC5,NDC80,ENSCAFG00000005299,BUB1,TTK,MTBP,GEN1	down_ACOR_VSMC
GO:0006397	0.0000591	1	30	168	mRNA processing	BP	0.00619	RNPS1,PTBP1,DDX39A,USP4,SF3B2,SRSF2,U2AF2,SNRPB2,SF1,U2AF1,THRAP3,NONO,HNRNPL,SF3B4,PHF5A,ENSCAFG00000015065,DHX15,DDX39B,SF3A2,DHX38,RBM14,RBM17,PRMT5,WTAP,JMJD6,WDR33,ENSCAFG00000003222,CDC5L,GEMIN7,SF3A1	down_ACOR_VSMC

GO:0050793	0.0000597	1	90	731	regulation of developmental process	BP	0.0112	EPHB1,PLK2,EDNRB,HOXB7,TEK,HCLS1,IL7R,KIT,CXCL8,PRKCH,ARHGEF15,IFRD1,TIE1,TNFAIP3,ADAMTS9,PPARG,AMOT,PDGFB,BCL6B,RELN,CD34,MEF2C,FERMT1,CHD7,DLL4,RAPGEF1,GPR4,GATA3,EDN1,HOXB8,RAB11A,APLNR,ARAP3,TCF4,SMAD1,NTN4,NEDD9,PIK3C2A,PPP3CA,ARG2,CASP8,MTOR,HOOK3,MAP3K3,BMP2,HMG20A,HHEX,BMP2K,PDZD8,ITPKB,GDPD5,ANGPT2,PROX1,ENSCAFG00000010596,TSPAN12,PRKD2,THOC1,TLR3,BTBD7,NCK1,FES,JAG1,PTGS2,MMD,RAPGEF2,CREB1,ZC4H2,CTNNA1,FMNL3,HMGB1,SPTBN4,RIPK1,ATP11C,SKIL,ARHGDIB,CUL4A,NR1D2,HIF1A,KDM3A,ENSCAFG00000002015,VASH1,ZC3H13,JCAD,ZEB1,ARHGEF2,FBXO5,CDON,TJP2,ZMYM2,ZNF609	up_VCAV_EC
GO:0032501	0.0000604	1	65	1782	multicellular organismal process	BP	0.244	TBX15,HOXA9,CMKLR1,IRX3,IRX5,HOXC10,LEP,LBH,CRY2,XYLT1,F5,ANGPT4,HOXA7,COL8A2,CD40,DLL1,CRYAB,F3,VXN,PLXNA4,BPI,GPAM,NEFH,LMCD1,CHRM2,TWIST2,MAB21L1,THRB,LGI4,TMEM100,PLAGL2,GREM1,HTR1D,NFE2L1,OVOL1,AVPR1A,CAMK2A,GREM2,HOXA6,CYLD,LUZP1,RECK,PTHLH,TBXA2R,EVI2B,CNNM4,TFPI2,REEP2,BVES,PTPRB,AXIN2,ACACB,NAPEPLD,PPARGC1A,SPATA6,FAP,DMPK,ADAM17,CLIC4,LRRN2,AGPAT2,GCNT4,FOSL2,KANK1,TNC	up_AFEM_EC
GO:0097435	0.0000615	1	30	230	supramolecular fiber organization	BP	0.0292	ARHGEF2,SERPINH1,PLK3,ACTN1,VASP,P4HA1,TPM4,FSCN1,MZT1,DPYSL3,HOOK3,WDR1,TMSB10,ACTR3,KPNB1,CRYAB,APOE,PLOD3,TWF1,PLEKHG2,PLS3,P3H4,CAPG,SIX4,NRP1,COL12A1,ENSCAFG00000030508,TNFAIP1,MYBPC2,MID1	down_VSAP_EC
GO:0032879	0.0000618	1	93	767	regulation of localization	BP	0.0115	PLK2,TEK,HCLS1,SELP,SELE,PRCP,PPP2R5A,CXCL8,DOCK4,TIE1,ADAMTS9,ATP8A1,MX2,AMOT,PDGFB,PODXL,STK10,TMEM173,RELN,PDE4D,IWS1,MEF2C,FERMT1,CHD7,DLL4,TLR4,STC1,GATA3,MMP28,EDN1,RAB11A,APLNR,ARAP3,RAB27A,CTTNBP2NL,SNCAIP,NEDD9,PIK3C2A,PPP3CA,YES1,ARG2,MTOR,MAP3K3,BMP2,SLK,BMP2K,RSC1A1,CCAR1,ABCA1,KCNJ3,ANGPT2,PROX1,ENSCAFG00000010224,EPB41,STMN1,MCC,PRKD2,SH3GLB1,SWAP70,APOD,NCK1,KCNJ2,LDB2,CD200,FES,JAG1,HIST1H1A,MMD,RAPGEF2,PLCG2,CTDSPL2,CREB1,GCC2,CTNNA1,HMGB1,TBC1D1,SPTBN4,RIPK1,ITGA6,MCU,DNAJC13,PDGFD,ARHGDIB,HIF1A,VASH1,DCP2,PTP4A1,JCAD,RABGAP1,ARHGEF2,FBXO5,USP6NL,ZNF609	up_VCAV_EC
GO:0007501	0.0000624	1	2	5	mesodermal cell fate specification	BP	0.0559	EYA1,HOXA11	up_VFEM_VSMC
GO:0048562	0.0000628	1	5	102	embryonic organ morphogenesis	BP	0.0483	TBX15,EYA1,HOXB7,HOXA7,SIX1	up_VFEM_EC
GO:0003013	0.0000639	1	26	121	circulatory system process	BP	0.115	TNNT2,ADRA1B,KCNE3,HTR2B,NPPB,GJA1,HRH1,PDE5A,SLC2A5,ADCY6,F2RL1,HRH2,CLSTN1,DMPK,PTGDR,TBX20,RNLS,CHD7,JUP,EDN1,NCALD,GAA,DOCK5,ERAP1,SLC8A1,BVES	up_ACOR_VSMC
GO:0001935	0.0000642	1	13	50	endothelial cell proliferation	BP	0.0116	PDGFB,MEF2C,DLL4,APLNR,EGFL7,BMP2,CAV2,PROX1,PRKD2,HMGB1,NRAS,VASH1,JCAD	up_VCAV_EC
GO:0035115	0.0000645	1	2	12	embryonic forelimb morphogenesis	BP	0.116	HOXA9,WNT7A	up_AORTRT_VSMC
GO:0006457	0.0000712	1	14	77	protein folding	BP	0.0326	P4HB,ENSCAFG00000013214,ERO1A,HSP90B1,ENSCAFG00000003477,FKBP11,CANX,PPIB,FKBP10,DNAJC10,PDIA4,CCT8,CDC37,SH3GLB1	down_VSAP_EC

GO:0033036	0.0000727	1	78	852	macromolecule localization	BP	0.0326	PRICKLE1,MYH9,SHROOM2,NR1H4,PLK3,HSPA5,HM13,TRAM1,PTPN14,ENPP1,SEC24D,PTPN11,EXOC5,SEC23A,MZT1,HOOK3,SESN2,SRP54,MYO1C,ENSCAFG00000031588,MSN,SEL1L,KIF5B,HIF1A,SSR1,IWS1,TNIK,OSBPL9,ZPR1,PTPN2,STAM2,SMG7,XPOT,USO1,SRPRA,DDX39B,COPG1,AGRN,CTNNB1,KPNB1,COPB2,SRP72,ENSCAFG00000002015,TFRC,APOE,KPNA4,PLOD3,LYN,DDIT3,TLN1,MMD,OSBPL6,GSK3B,ARMCX3,GBP5,SH3GLB1,TAX1BP3,KPNA1,REST,ABHD5,SCAMP1,RAB11FIP1,DISC1,KDELR2,AP3S1,CLTC,INHBA,STX6,ESYT3,SIX4,CDKN1A,NRP1,RHBDF2,ARF4,YWHAH,BLZF1,SEC13,MID1	down_VSAP_EC
GO:0001708	0.0000729	1	3	20	cell fate specification	BP	0.0535	HOXC10,EYA1,SIX1	up_VFEM_EC
GO:0072359	0.0000733	1	9	368	circulatory system development	BP	0.102	ACVRL1,HOXA3,PDPN,NRP2,HOXA7,ANPEP,SULF1,ELK3,HOXA1	down_ACOR_EC
GO:0048562	0.0000733	1	5	102	embryonic organ morphogenesis	BP	0.0696	HOXA11,TBX15,SALL1,IRX5,SHOX2	up_VSAP_VSMC
GO:0050789	7.35887E-05	0.99996	113	3295	regulation of biological process	BP	0.19791669	C7,CNTNAP4,SH3TC2,CDH3,NR1H4,TMOD1,GATA4,BRSK1,TRPS1,PLCL1,PKIB,CALY,SOX9,RHOU,PKNOX2,ARHGAP6,PTH1R,RGS1,PCSK1N,FNDC4,NDRG2,AQP1,PORCN,ENSCAFG00000004331,SYNPO2,KCNB1,ADAMTS13,EDEM2,FZD5,CCDC3,AGRN,PDE1A,IFI6,SEMA6D,GNAO1,DUSP26,DIXDC1,RASL11A,PTGIR,NOD2,IL33,RGMA,CASP10,LHX9,PDE4A,INSYN1,SMAD6,KLHL25,PLCE1,PLS1,ACTN2,ZMYND8,ARHGAP28,RNLS,ESM1,PTGDS,PAK3,MMP28,GUCY1B1,GRK7,BCL2,TRIM8,TNFRSF19,PTGER4,ARHGDIB,PREB,CST3,PIAS1,DCLK2,ADAMTSL2,MAPK8IP2,RAPGEF4,MAVS,FOXC2,FIGNL1,LRRC15,IFIT2,TBX5,LRP4,CLSTN1,SH3RF3,LRRC4,RBM38,CLU,SFMBT2,C23H3orf58,ZNF229,CHMP4B,TP53INP2,RARRES2,VPS16,GMEB2,RIOX2,PRKACA,RNF114,STK32C,NRP1,AZIN2,AGPAT2,ARHGEF26,PRKCD,GNAZ,RIN2,PPP1R26,PM20D1,PYGO2,DBNDD2,EFEMP1,CCNYL1,MFAP4,ADGRL1,TUBB4A,TLE2	up_ACOR_EC
GO:0006955	0.0000738	1	47	327	immune response	BP	0.013	IL7R,GBP5,KIT,CXCL8,PRKCH,IL6,TNFAIP3,TNIP1,MX2,BCL6B,TMEM173,PDE4D,MEF2C,TLR4,NLRC5,EIF2B2,GATA3,RAB27A,IRF8,PELI1,SERINC5,ARG2,NECTIN2,MTOR,SNAP23,ELF4,RIOK3,FLNB,PRKD2,THOC1,SWAP70,TLR3,MFNG,OAS3,OTUD4,CD200,FES,JAG1,PLCG2,BCL10,CNOT7,GCH1,HMGB1,LSM14A,BMX,TNFAIP1,ARHGEF2	up_VCAV_EC
GO:0007389	0.0000739	1	3	137	pattern specification process	BP	0.398	HOXA3,TBX5,HOXB5	up_APUL_VSMC
GO:0106049	0.0000742	1	2	4	regulation of cellular response to osmotic	BP	0.339	PTGER2,ARHGEF2	up_VPUL_EC
GO:1902218	0.0000742	1	2	4	regulation of intrinsic apoptotic signaling pathway in response to	BP	0.339	PTGER2,ARHGEF2	up_VPUL_EC
GO:1902219	0.0000742	1	2	4	negative regulation of intrinsic apoptotic signaling pathway in	BP	0.339	PTGER2,ARHGEF2	up_VPUL_EC
GO:0002682	0.0000744	1	49	343	regulation of immune system process	BP	0.013	HCLS1,IL7R,GBP5,ADGRF5,SELP,KIT,CXCL8,PRKCH,TNFAIP3,TNIP1,STK10,TMEM173,PDE4D,MEF2C,TLR4,NLRC5,EIF2B2,GATA3,MMP28,HOXB8,PELI1,ARG2,CASP8,NECTIN2,MTOR,ITPKB,RIOK3,PRKD2,THOC1,SWAP70,TLR3,APOD,NCK1,OTUD4,CD200,FES,JAG1,PLCG2,CREB1,BCL10,CNOT7,HMGB1,RIPK1,LSM14A,ATP11C,CUL4A,HIF1A,ZEB1,TJP2	up_VCAV_EC
GO:0010032	0.0000744	1	4	4	meiotic chromosome condensation	BP	0.00775	NCAPD2,SMC4,NCAPH,SMC2	down_ACOR_VSMC
GO:0045597	0.0000782	1	39	253	positive regulation of cell differentiation	BP	0.0136	HCLS1,IL7R,KIT,PRKCH,ADAMTS9,PPARG,RELN,CD34,MEF2C,RAPGEF1,GATA3,EDN1,RAB11A,TCF4,NEDD9,CASP8,MTOR,BMP2,ITPKB,GDPD5,PROX1,NCK1,FES,JAG1,PTGS2,MMD,RAPGEF2,CREB1,ZC4H2,HMGB1,RIPK1,ATP11C,HIF1A,ENSCAFG00000002015,ZEB1,ARHGEF2,FBXO5,CDON,ZNF609	up_VCAV_EC
GO:0009653	0.0000802	1	13	773	anatomical structure morphogenesis	BP	0.102	ACVRL1,HOXA3,SPG11,PDPN,NRP2,HOXA7,ANPEP,SULF1,ELK3,HOXA1,CSGALNACT1,HOXC4,ASPN	down_ACOR_EC

GO:0007093	0.0000813	1	15	59	mitotic cell cycle checkpoint	BP	0.00835	ZW10,TRIP13,BIRC5,CDK1,CASP2,FANCI,TOPBP1,MAP3K20,NDC80,ENSCAFG00000005299,CLSPN,BUB1,TTK,ZWINT,GEN1	down_ACOR_VSMC
GO:0044057	0.0000832	1	24	110	regulation of system process	BP	0.128	TNNT2,FGF13,ADRA1B,LEP,KCNE3,GATA5,GJA1,HRH1,WASF3,PDE5A,TMEM108,F2RL1,HRH2,DMPK,NPNT,RNLS,APP,JUP,EDN1,GAA,INPP5K,DOCK5,SLC8A1,BVES	up_ACOR_VSMC
GO:0045765	0.0000835	1	21	105	regulation of angiogenesis	BP	0.0143	PLK2,TEK,CXCL8,TIE1,ADAMTS9,AMOT,CD34,DLL4,GPR4,APLNR,SMAD1,PIK3C2A,MAP3K3,HHEX,ANGPT2,TSPAN12,PRKD2,TLR3,HIF1A,VASH1,JCAD	up_VCAV_EC
GO:0021602	0.0000836	1	3	16	cranial nerve morphogenesis	BP	0.102	HOXA3,NRP2,HOXA1	down_ACOR_EC
GO:0030036	0.0000846	1	29	223	actin cytoskeleton organization	BP	0.0369	ARHGEF2,SIPA1L1,MYH9,FMNL3,ACTN1,VASP,TPM4,CORO1C,FSCN1,FLNB,DPYSL3,WDR1,TNIK,TMSB10,ACTR3,PALLD,TWF1,PLEKHG2,TLN1,PLS3,DAAM1,ENSCAFG00000017326,PAK2,CAPG,SIX4,NRP1,ENSCAFG00000030508,TNFAIP1,MYBPC2	down_VSAP_EC
GO:0048665	0.0000856	1	2	6	neuron fate specification	BP	0.0704	HOXC10,EYA1	up_VFEM_VSMC
GO:0048705	0.0000873	1	4	95	skeletal system morphogenesis	BP	0.0704	EYA1,TBX15,HOXA11,SHOX2	up_VFEM_VSMC
GO:0007200	0.0000874	1	8	18	phospholipase C-activating G protein-coupled receptor signaling	BP	0.128	ADRA1B,ACTN2,HTR2B,HRH1,F2RL1,GNA14,EDN1,GNAQ	up_ACOR_VSMC
GO:0048705	0.0000878	1	5	95	skeletal system morphogenesis	BP	0.102	HOXA3,HOXA7,SULF1,HOXA1,CSGALNACT1	down_ACOR_EC
GO:0032502	0.0000883	1	177	1661	developmental process	BP	0.0148	EPHB1,PLK2,EDNRB,HOXB7,TEK,HCLS1,PCDH12,IL7R,ADGRF5,KIT,PRCP,CXCL8,PTCHD1,PRKCH,ARHGEF15,IFRD1,EMCN,CLEC14A,IL6,CALCRL,FLI1,TIE1,TNFAIP3,ADAMTS9,PPARG,AMOT,PDGFB,BCL6B,MALL,PODXL,RELN,CD34,LAMC2,PDE4D,MEF2C,FERMT1,ATF3,CHD7,TIPARP,DLL4,RAPGEF1,TLR4,QKI,GPR4,STC1,CDH5,EIF2B2,GATA3,EDN1,HOXB8,RAB11A,APLNR,ARAP3,RAB27A,TCF4,SMAD1,NTN4,FOXN2,IGSF3,PSME4,ENSCAFG00000003530,NEDD9,IRF8,SOX18,PIK3C2A,TMOD1,FIGNL1,PPP3CA,PTPRB,SERINC5,MTSS1,ODC1,ARG2,CASP8,EPHB4,NECTIN2,B3GNT5,MTOR,NAV2,TENT2,HOOK3,LRRC8C,ELF4,MAP3K3,BMP2,CDYL,HMG20A,HHEX,CAV2,PREX2,BMP2K,PDZD8,ENC1,ITPKB,GDPD5,ANGPT2,PROX1,UHRF2,ENSCAFG00000010224,MDFI,ENSCAFG00000010596,ARID4B,STMN1,FLNB,SNX2,TSPAN12,MMP24,PAK2,PRKD2,THOC1,SWAP70,SMARCA4,TLR3,MFNG,APOD,BTBD7,NCK1,EXOC5,TIMELESS,HOXB5,LDB2,SLC25A25,FES,JAG1,PTGS2,MMD,TOP1,RAPGEF2,PLCG2,CREB1,BCL10,BASP1,ZC4H2,TAOK1,CTNNA1,FMNL3,ASF1A,ADIPOR2,HMGB1,NUP133,SPTBN4,RIPK1,CRIP2,SMC5,AFF4,ITGA6,ATP11C,SKIL,ARHGDIB,CUL4A,BMX,NR1D2,HIF1A,KDM3A,AL	up_VCAV_EC
GO:0046578	0.0000886	1	19	77	regulation of Ras protein signal transduction	BP	0.0148	ARHGEF15,FGD5,FGD6,RAPGEF1,GPR4,ARAP3,PLEKHG1,PREX2,ABCA1,ITPKB,ARHGEF12,MADD,STMN1,NET1,ARHGDIB,ALS2,TNFAIP1,RGL2,ARHGEF2	up_VCAV_EC
GO:0030029	0.000089	1	38	239	actin filament-based process	BP	0.0148	TEK,HCLS1,KIT,SELE,ARHGEF15,ENSCAFG00000007112,AMOT,BAIAP2L1,PDE4D,MEF2C,STC1,EDN1,ARHGAP28,TMOD1,MTSS1,MTOR,NCKAP1,PROX1,IQGAP2,EPB41,ELMO1,STMN1,FLNB,PAK2,SWAP70,NCK1,KCNJ2,FES,CTNNA1,FMNL3,ARPC5L,ARHGDIB,ARHGAP26,GAS2L3,TNFAIP1,TMOD3,ARHGEF2,FSCN1	up_VCAV_EC
GO:0008104	0.0000903	1	71	763	protein localization	BP	0.0373	PRICKLE1,MYH9,SHROOM2,NR1H4,PLK3,HSPA5,HM13,TRAM1,PTPN14,SEC24D,PTPN11,EXOC5,SEC23A,MZT1,HOOK3,SESN2,SRP54,MYO1C,ENSCAFG00000031588,MSN,SEL1L,KIF5B,HIF1A,SSR1,IWS1,TNIK,ZPR1,STAM2,SMG7,XPOT,USO1,SRPRA,DDX39B,COPG1,AGRN,CTNNB1,KPNB1,COPB2,SRP72,ENSCAFG00000002015,TFRC,APOE,KPNA4,PLOD3,LYN,DDIT3,TLN1,MMD,GSK3B,ARMCX3,GBP5,SH3GLB1,TAX1BP3,KPNA1,REST,SCAMP1,RAB11FIP1,DISC1,KDELR2,AP3S1,CLTC,STX6,SIX4,CDKN1A,NRP1,RHBDF2,ARF4,YWHAH,BLZF1,SEC13,MID1	down_VSAP_EC

GO:0072006	0.0000903	1	4	59	nephron development	BP	0.0603	EYA1,HOXB7,SIX1,MTSS1	up_VFEM_EC
GO:0032392	0.0000928	1	8	19	DNA geometric change	BP	0.00936	HMGB1,MCM6,MCM4,MCM7,GINS4,RAD51,HMGB3,HMGB2	down_ACOR_VSMC
GO:0043062	0.0000932	1	15	99	extracellular structure organization	BP	0.251	ADAMTS2,FBLN2,LUM,SDC4,LOX,RECK,LCAT,CRISPLD2,B4GALT1,ADAMTSL4,LOXL3,DDR2,GREM1,SOAT1,ECM2	down_VCAV_EC
GO:0008406	0.0000943	1	4	57	gonad development	BP	0.0801	HOXA11,HOXA9,SALL1,IRX5	up_VSAP_VSMC
GO:0045137	0.0000943	1	4	57	development of primary sexual characteristics	BP	0.0801	HOXA11,HOXA9,SALL1,IRX5	up_VSAP_VSMC
GO:0001655	0.0000959	1	5	116	urogenital system development	BP	0.0603	EYA1,MME,HOXB7,SIX1,MTSS1	up_VFEM_EC
GO:0007076	0.0000964	1	5	7	mitotic chromosome condensation	BP	0.00966	NCAPD2,SMC4,NCAPD3,NCAPH,SMC2	down_ACOR_VSMC
GO:0043587	0.0000988	1	2	4	tongue morphogenesis	BP	0.0603	SIX1,CYP26B1	up_VFEM_EC
GO:0072073	0.0000994	1	4	60	kidney epithelium development	BP	0.0603	EYA1,HOXB7,SIX1,MTSS1	up_VFEM_EC
GO:0060836	0.0000997	1	2	4	lymphatic endothelial cell differentiation	BP	0.102	ACVRL1,PDPN	down_ACOR_EC
GO:0035239	0.000101	1	8	299	tube morphogenesis	BP	0.102	ACVRL1,HOXA3,NRP2,HOXA7,ANPEP,SULF1,ELK3,HOXA1	down_ACOR_EC
GO:0003263	0.000101	1	2	4	cardioblast proliferation	BP	0.0603	EYA1,SIX1	up_VFEM_EC
GO:0045321	0.000106	1	32	191	leukocyte activation	BP	0.0174	IL7R,ADGRF5,KIT,CXCL8,IL6,TNFAIP3,MEF2C,CHD7,DLL4,TLR4,GATA3,RAB27A,PELI1,ARG2,NECTIN2,MTOR,SNAP23,ELF4,HHEX,ITPKB,THOC1,SWAP70,MFNG,NCK1,CD200,FES,PLCG2,BCL10,HMGB1,ATP11C,BMX,ZEB1	up_VCAV_EC
GO:0030029	0.000108	1	30	239	actin filament-based process	BP	0.0438	ARHGEF2,SIPA1L1,MYH9,FMNL3,ACTN1,VASP,TPM4,CORO1C,FSCN1,FLNB,DPYSL3,WDR1,MYO1C,TNIK,TMSB10,ACTR3,PALLD,TWF1,PLEKHG2,TLN1,PLS3,DAAM1,ENSCAFG00000017326,PAK2,CAPG,SIX4,NRP1,ENSCAFG00000030508,TNFAIP1,MYBPC2	down_VSAP_EC
GO:0003180	0.000108	1	5	7	aortic valve morphogenesis	BP	0.145	SMAD6,GATA5,GATA4,BMP4,TBX20	up_ACOR_VSMC
GO:0009966	0.000109	1	102	868	regulation of signal transduction	BP	0.0176	EPHB1,TEK,HCLS1,IL7R,SELP,KIT,PRCP,CXCL8,PRKCH,ARHGEF15,FGD5,TNFAIP3,MAP3K11,TNIP1,AMOT,PDGFB,BAIAP2L1,RELN,FGD6,CXHXorf36,PDE4D,MEF2C,FERMT1,ATF3,DLL4,RAPGEF1,TLR4,NLRC5,GPR4,GATA3,EDN1,APLNR,ARAP3,HOMER3,EGFL7,FIGNL1,PELI1,PPP3CA,GUCY1A1,CASP8,NECTIN2,MTOR,ACPP,TSPAN14,PLEKHG1,MAP3K3,BMP2,HHEX,CHN2,CAV2,PREX2,BMP2K,ABCA1,PPP1R2,ITPKB,GDPD5,RIOK3,ARHGEF12,MADD,STMN1,PAK2,MCC,PRKD2,SH3GLB1,SMARCA4,TLR3,MFNG,AKAP12,APOD,NCK1,OTUD4,NET1,JAG1,PTGS2,RAPGEF2,CTDSPL2,BCL10,TAOK1,CTNNA1,CNOT7,HMGB1,PMAIP1,RIPK1,LSM14A,ITGA6,RGS2,SKIL,PDGFD,ARHGDIB,UFSP2,HIF1A,ARHGAP26,ALS2,ENSCAFG00000002015,TNFAIP1,IVNS1ABP,RGL2,JCAD,ZEB1,ARHGEF2,PEAR1,CDON	up_VCAV_EC
GO:0031497	0.000109	1	13	49	chromatin assembly	BP	0.0109	HMGA1,CENPA,NOC2L,OIP5,IPO4,HIST1H1A,HMGB2,HIST1H2BB,CENPP,GRWD1,HAT1,MIS18A,H3F3B	down_ACOR_VSMC
GO:0009790	0.000109	1	6	335	embryo development	BP	0.0812	HOXC10,EYA1,HOXA9,TBX15,HOXA11,SHOX2	up_VFEM_VSMC
GO:0048562	0.000111	1	4	102	embryonic organ morphogenesis	BP	0.0812	EYA1,TBX15,HOXA11,SHOX2	up_VFEM_VSMC
GO:0042060	0.000117	1	21	103	wound healing	BP	0.0188	SELP,PRCP,F8,IFRD1,PDGFB,VWF,CD34,KLKB1,TLR4,GPR4,RAB27A,TFPI,MTOR,MMRN1,APOD,ADIPOR2,PDGFD,HIF1A,ITGB3,PEAR1,ELK3	up_VCAV_EC
GO:0048024	0.000117	1	10	30	regulation of mRNA splicing, via spliceosome	BP	0.0116	RNPS1,SRSF2,U2AF2,THRAP3,HNRNPL,SF3B4,RBM14,WTAP,JMJD6,SF3A1	down_ACOR_VSMC

GO:0006082	0.000118	1	50	305	organic acid metabolic process	BP	0.494	PEX7,HNF4A,ACSM3,LIPC,INSIG2,ACAA1,ACACB,PEX2,GOT1,DLST,GLUL,ADHFE1,ENSCAFG00000013986,WARS2,C1QTNF2,ALDH6A1,HSD17B8,FAAH,PDK2,GALK1,PTGES,SUOX,HACD4,GCDH,ABAT,BCKDHB,ENSCAFG00000016140,AZIN2,DAGLB,MARC1,EHHADH,FARS2,LONP2,ACSS2,PC,DHRS9,ABHD16A,ENSCAFG00000002977,HPGD,PNP,QARS,ENSCAFG00000023820,IDH1,IDH3A,ENSCAFG00000006639,ASS1,CHST7,LARS2,DDIT4,IDO2	up_VSAP_EC
GO:1901989	0.000119	1	10	31	positive regulation of cell cycle phase transition	BP	0.0116	BIRC5,CDK1,EZH2,CDK4,DTL,ENSCAFG00000005299,CDC25A,GLI1,UBE2C,MTBP	down_ACOR_VSMC
GO:0048513	0.000119	1	8	906	animal organ development	BP	0.393	HOXA3,TBX5,GATA5,TMEM176A,TMEM176B,PHEX,HOXB5,SLIT2	up_VPUL_VSMC
GO:0008627	0.00012	1	2	5	intrinsic apoptotic signaling pathway in response to osmotic	BP	0.339	PTGER2,ARHGEF2	up_VPUL_EC
GO:0050878	0.000121	1	17	75	regulation of body fluid levels	BP	0.0191	SELP,F8,PDGFB,VWF,CD34,KLKB1,TLR4,EDN1,RAB27A,TFPI,MMRN1,STMN1,CREB1,PDGFD,HIF1A,ITGB3,PEAR1	up_VCAV_EC
GO:0007389	0.000122	1	4	137	pattern specification process	BP	0.393	HOXA3,TBX5,GATA5,HOXB5	up_VPUL_VSMC
GO:0051241	0.000123	1	48	336	negative regulation of multicellular organismal process	BP	0.0193	EPHB1,PLK2,EDNRB,TEK,ARHGEF15,IFRD1,DOCK4,TIE1,TNFAIP3,ADAMTS9,AMOT,PDGFB,CD34,PDE4D,MEF2C,FERMT1,KLKB1,DLL4,TLR4,GPR4,STC1,GATA3,HOXB8,HOMER3,TFPI,PPP3CA,GUCY1A1,ARG2,HOOK3,BMP2,HMG20A,HHEX,ITPKB,ANGPT2,MCC,THOC1,APOD,PROCR,JAG1,RAPGEF2,CTNNA1,HMGB1,SPTBN4,ARHGDIB,CUL4A,HIF1A,VASH1,TJP2	up_VCAV_EC
GO:0002253	0.000124	1	19	92	activation of immune response	BP	0.0193	PRKCH,TNFAIP3,TNIP1,TMEM173,PDE4D,MEF2C,TLR4,EIF2B2,GATA3,PELI1,NECTIN2,RIOK3,PRKD2,TLR3,OTUD4,PLCG2,BCL10,HMGB1,LSM14A	up_VCAV_EC
GO:0000077	0.000124	1	15	60	DNA damage checkpoint	BP	0.0121	HINFP,WDR76,INTS7,CDK1,CASP2,FANCI,TOPBP1,MAP3K20,RNASEH2B,DTL,DDX39B,CLSPN,H2AFX,CDC5L,ATRIP	down_ACOR_VSMC
GO:0047484	0.000126	1	2	5	regulation of response to osmotic stress	BP	0.339	PTGER2,ARHGEF2	up_VPUL_EC
GO:0007596	0.000128	1	13	47	blood coagulation	BP	0.0195	SELP,F8,PDGFB,VWF,CD34,KLKB1,TLR4,RAB27A,TFPI,MMRN1,PDGFD,ITGB3,PEAR1	up_VCAV_EC
GO:0045666	0.000129	1	16	68	positive regulation of neuron differentiation	BP	0.0195	RELN,MEF2C,RAPGEF1,RAB11A,TCF4,BMP2,GDPD5,PROX1,NCK1,FES,MMD,RAPGEF2,ZC4H2,ZEB1,ARHGEF2,CDON	up_VCAV_EC
GO:0048856	0.000129	1	16	1533	anatomical structure development	BP	0.0736	HOXC10,TBX15,EYA1,HOXA9,TOX,MME,HOXB7,HOXA7,SIX1,MAB21L1,TIAM1,RADIL,CYP26B1,CDC42EP5,MTSS1,CA9	up_VFEM_EC
GO:0001658	0.000132	1	3	24	branching involved in ureteric bud	BP	0.0736	EYA1,HOXB7,SIX1	up_VFEM_EC
GO:0008283	0.000133	1	68	545	cell proliferation	BP	0.0199	EPHB1,HCLS1,IL7R,KIT,CXCL8,PRKCH,CALCRL,TNFAIP3,MAP3K11,PDGFB,BCL6B,CD34,MEF2C,FERMT1,DLL4,RAPGEF1,TLR4,STC1,GATA3,EDN1,APLNR,PTGS1,SOX18,EGFL7,FIGNL1,PELI1,MTSS1,ODC1,ARG2,HOOK3,ELF4,MAP3K3,BMP2,HHEX,CAV2,CNOT6,CCAR1,ITPKB,PROX1,UHRF2,MCC,PRKD2,APOD,NCK1,FES,JAG1,PTGS2,RAPGEF2,USP28,CTNNA1,CNOT7,HMGB1,CRIP2,NRAS,USP13,PDGFD,CUL4A,BMX,HIF1A,NR4A1,HP1BP3,VASH1,JCAD,ZEB1,ARHGEF2,CDON,ZNF609,GINS4	up_VCAV_EC
GO:0030111	0.000137	1	4	108	regulation of Wnt signaling pathway	BP	0.788	BARX1,NKX2-5,BAMBI,ANKRD6	up_VPOR_VSMC
GO:0001710	0.000137	1	2	7	mesodermal cell fate commitment	BP	0.0961	EYA1,HOXA11	up_VFEM_VSMC
GO:0035480	0.000139	1	1	1	regulation of Notch signaling pathway involved in heart	BP	0.32	GATA5	down_AFEM_VSMC
GO:0035481	0.000139	1	1	1	positive regulation of Notch signaling pathway involved in heart	BP	0.32	GATA5	down_AFEM_VSMC
GO:0003137	0.000139	1	1	1	Notch signaling pathway involved in heart	BP	0.32	GATA5	down_AFEM_VSMC
GO:0061999	0.000139	1	1	1	regulation of cardiac endothelial to	BP	0.32	GATA5	down_AFEM_VSMC
GO:0062000	0.000139	1	1	1	positive regulation of cardiac endothelial to mesenchymal transition	BP	0.32	GATA5	down_AFEM_VSMC

GO:0090381	0.000139	1	1	1	regulation of heart induction	BP	0.32	GATA5	down_AFEM_VSMC
GO:1901321	0.000139	1	1	1	positive regulation of heart induction	BP	0.32	GATA5	down_AFEM_VSMC
GO:0035136	0.00014	1	2	16	forelimb morphogenesis	BP	0.217	HOXA9,WNT7A	up_AORTRT_VSMC
GO:0048706	0.000141	1	4	59	embryonic skeletal system development	BP	0.133	HOXA3,HOXA7,SULF1,HOXA1	down_ACOR_EC
GO:0034728	0.000144	1	14	56	nucleosome organization	BP	0.0137	CENPA,SMARCC1,OIP5,IPO4,HIST1H1A,HMGB2,HIST1H2BB,SMARCB1,CENPP,GRWD1,SUPT16H,HAT1,MIS18A,H3F3B	down_ACOR_VSMC
GO:0006695	0.000147	1	7	16	cholesterol biosynthetic process	BP	0.0138	LSS,SREBF1,HMGCR,INSIG1,DHCR24,MVD,FDFT1	down_ACOR_VSMC
GO:1902653	0.000147	1	7	16	secondary alcohol biosynthetic process	BP	0.0138	LSS,SREBF1,HMGCR,INSIG1,DHCR24,MVD,FDFT1	down_ACOR_VSMC
GO:0048856	0.000148	1	8	1533	anatomical structure development	BP	0.69	HOXC6,SLITRK5,LIMK1,GATA5,CA2,LRRC17,C6,TGFBI	up_VCAV_VSMC
GO:0045165	0.000148	1	4	65	cell fate commitment	BP	0.0794	HOXC10,EYA1,SIX1,CYP26B1	up_VFEM_EC
GO:0009954	0.000148	1	2	16	proximal/distal pattern formation	BP	0.217	HOXC10,HOXA9	up_AORTRT_VSMC
GO:0030154	0.00015	1	91	1040	cell differentiation	BP	0.0539	ARHGEF2,PRICKLE1,VEGFA,SIPA1L1,SULF1,CELF1,SERPINH1,MYH9,TAGLN2,SERPINE1,ACTN1,SOCS5,ETV6,SGK1,ERO1A,ENPP1,ERRFI1,ITGA2,CASP3,PGK1,PTPN11,FLNB,DPYSL3,HOOK3,TRIB3,IFRD1,ILK,FHL2,TGFBR1,PTGS2,WDR1,CHSY1,MSN,HOXB7,KIF5B,HIF1A,FOXP4,SLC45A3,TNIK,UBA6,ZPR1,PTPN2,EIF2B1,EVI2B,NEO1,ACTA2,UST,CDH11,CTNNB1,ANLN,ENSCAFG00000002015,TFRC,APOE,PLOD3,KLF5,LYN,BNIP3,STRAP,DDIT3,MMD,ATF3,GSK3B,VLDLR,NEXN,MYO1E,SDC4,REST,MAPK10,PRMT1,SH2B3,EXT1,GLO1,DISC1,CDYL,PAK2,CREB3L2,A2M,INHBA,CAMK2D,NR4A1,HIF1AN,BTK,SIX4,NRP1,SPINK2,ARF4,YWHAH,COL12A1,KDM3A,MYBPC2,HOMER1	down_VSAP_EC
GO:0030154	0.000151	1	119	1040	cell differentiation	BP	0.0223	EPHB1,EDNRB,HOXB7,TEK,HCLS1,IL7R,ADGRF5,KIT,PRKCH,IFRD1,IL6,FLI1,ADAMTS9,PPARG,AMOT,PDGFB,BCL6B,PODXL,RELN,CD34,PDE4D,MEF2C,ATF3,CHD7,TIPARP,DLL4,RAPGEF1,TLR4,QKI,GPR4,STC1,EIF2B2,GATA3,EDN1,HOXB8,RAB11A,APLNR,RAB27A,TCF4,NTN4,FOXN2,PSME4,ENSCAFG00000003530,NEDD9,IRF8,SOX18,TMOD1,FIGNL1,PPP3CA,MTSS1,CASP8,NECTIN2,MTOR,NAV2,TENT2,HOOK3,LRRC8C,ELF4,BMP2,CDYL,HMG20A,HHEX,CAV2,PREX2,ITPKB,GDPD5,ANGPT2,PROX1,UHRF2,ENSCAFG00000010224,MDFI,ENSCAFG00000010596,ARID4B,STMN1,FLNB,MMP24,PAK2,MFNG,APOD,NCK1,HOXB5,FES,JAG1,PTGS2,MMD,RAPGEF2,PLCG2,CREB1,BASP1,ZC4H2,CTNNA1,ASF1A,HMGB1,NUP133,SPTBN4,RIPK1,AFF4,ITGA6,ATP11C,SKIL,CUL4A,BMX,NR1D2,HIF1A,KDM3A,ALS2,ATRX,NR4A1,ENSCAFG00000002015,P2RX2,TMOD3,ZEB1,TET2,ARHGEF2,FBXO5,CDON,ELK3,TJP2,ZNF609	up_VCAV_EC
GO:0006325	0.000151	1	37	234	chromatin organization	BP	0.014	SREBF1,POLE3,ATAD2,HMGA1,ENSCAFG00000016482,CENPA,PRMT1,CDK1,AURKB,EZH2,FBL,SMARCC1,UHRF1,BRCA1,NOC2L,OIP5,IPO4,HIST1H1A,SUV39H1,HASPIN,HMGB2,DMAP1,HIST1H2BB,SMARCB1,CCNA2,RBM14,BAP1,CENPP,GRWD1,SUPT16H,TADA2A,HAT1,HIST2H2AC,H2AFX,MIS18A,HMGN1,H3F3B	down_ACOR_VSMC
GO:0003002	0.000151	1	4	118	regionalization	BP	0.788	HOXA3,BARX1,NKX2-5,CYP26B1	up_VPOR_VSMC
GO:0031570	0.000152	1	15	61	DNA integrity checkpoint	BP	0.014	HINFP,WDR76,INTS7,CDK1,CASP2,FANCI,TOPBP1,MAP3K20,RNASEH2B,DTL,DDX39B,CLSPN,H2AFX,CDC5L,ATRIP	down_ACOR_VSMC
GO:0048663	0.000152	1	2	13	neuron fate commitment	BP	0.306	HOXC10,EYA1	up_AFEM_VSMC
GO:0060065	0.000152	1	2	7	uterus development	BP	0.102	HOXA9,HOXA11	up_VFEM_VSMC

GO:0065007	0.000155308	0.99991	117	3499	biological regulation	BP	0.35802952	C7,CNTNAP4,SH3TC2,CDH3,NR1H4,TMOD1,GATA4,BRSK1,TRPS1,PLCL1,PKIB,CALY,SOX9,RHOU,PKNOX2,ARHGAP6,PTH1R,RGS1,PCSK1N,FNDC4,NDRG2,AQP1,PORCN,ENSCAFG00000004331,SYNPO2,KCNB1,ADAMTS13,EDEM2,FZD5,CCDC3,AGRN,PDE1A,IFI6,SEMA6D,GNAO1,DUSP26,DIXDC1,RASL11A,PTGIR,NOD2,IL33,RGMA,CASP10,LHX9,PDE4A,INSYN1,SMAD6,DYNC1I1,KLHL25,PLCE1,PLS1,ACTN2,ZMYND8,ARHGAP28,RNLS,ESM1,PTGDS,PAK3,MMP28,GUCY1B1,GRK7,BCL2,TRIM8,TNFRSF19,PTGER4,ARHGDIB,PREB,CST3,PIAS1,DCLK2,ADAMTSL2,USP53,MAPK8IP2,RAPGEF4,MAVS,FOXC2,FIGNL1,LRRC15,IFIT2,TBX5,LRP4,CLSTN1,SH3RF3,LRRC4,RBM38,CLU,SLC9A9,SFMBT2,C23H3orf58,ZNF229,CHMP4B,TP53INP2,TMTC2,RARRES2,VPS16,GMEB2,RIOX2,PRKACA,RNF114,STK32C,NRP1,AZIN2,AGPAT2,ARHGEF26,PRKCD,GNAZ,RIN2,PPP1R26,PM20D1,PYGO2,DBNDD2,EFEMP1,CCNYL1,MFAP4,ADGRL1,TUBB4A,TLE2	up_ACOR_EC
GO:0090329	0.000156	1	9	25	regulation of DNA-dependent DNA	BP	0.0143	SMC1A,RFC2,RFC5,RFC3,RFC4,GMNN,E2F8,SMC3,CDK2	down_ACOR_VSMC
GO:0007599	0.000157	1	13	48	hemostasis	BP	0.0231	SELP,F8,PDGFB,VWF,CD34,KLKB1,TLR4,RAB27A,TFPI,MMRN1,PDGFD,ITGB3,PEAR1	up_VCAV_EC
GO:0043484	0.000157	1	12	43	regulation of RNA splicing	BP	0.0143	RNPS1,SRSF2,U2AF2,THRAP3,HNRNPL,SF3B4,HNRNPF,RBM14,RBM17,WTAP,JMJD6,SF3A1	down_ACOR_VSMC
GO:0060272	0.00016	1	2	7	embryonic skeletal joint morphogenesis	BP	0.104	HOXA11,SHOX2	up_VFEM_VSMC
GO:0007275	0.000161	1	17	1370	multicellular organism development	BP	0.144	ACVRL1,LYN,HOXA3,ACSL4,SPG11,PDPN,NRP2,HIVEP2,HOXA7,ANPEP,SULF1,ELK3,HOXA1,CSGALNACT1,HOXC4,ASPN,NEDD4	down_ACOR_EC
GO:0051225	0.000162	1	13	49	spindle assembly	BP	0.0146	SMC1A,FBXO5,BIRC5,AURKB,HAUS8,NEK2,RCC1,SMC3,STAG1,ENSCAFG00000005299,CDC20,ASPM,KIF11	down_ACOR_VSMC
GO:0051649	0.000163	1	60	630	establishment of localization in cell	BP	0.0548	PRICKLE1,MYH9,SHROOM2,NR1H4,PLK3,HM13,ANKRD50,TRAM1,ERO1A,PTPN14,SEC24D,HYOU1,PTPN11,SEC23A,HOOK3,PPP1R12A,SRP54,TMEM87B,MYO1C,CANX,MSN,RAB6A,SEL1L,RPGR,KIF5B,LMAN1,HIF1A,DYNLL1,TRAK1,SSR1,IWS1,YIPF5,ZPR1,STAM2,SMG7,XPOT,USO1,SRPRA,DDX39B,COPG1,CTNNB1,KPNB1,COPB2,SRP72,CRYAB,KPNA4,LYN,DDIT3,GSK3B,SH3GLB1,KPNA1,HTT,CALM1,AP3S1,CLTC,CREB3L2,STX6,CDKN1A,YWHAH,SEC13	down_VSAP_EC
GO:0050769	0.000164	1	20	95	positive regulation of neurogenesis	BP	0.0238	PRKCH,RELN,MEF2C,RAPGEF1,RAB11A,TCF4,MTOR,BMP2,GDPD5,PROX1,NCK1,FES,MMD,RAPGEF2,ZC4H2,HIF1A,ZEB1,ARHGEF2,CDON,ZNF609	up_VCAV_EC
GO:0090307	0.000168	1	9	26	mitotic spindle assembly	BP	0.0151	SMC1A,BIRC5,AURKB,NEK2,SMC3,STAG1,ENSCAFG00000005299,CDC20,KIF11	down_ACOR_VSMC
GO:0003012	0.000168	1	2	90	muscle system process	BP	0.438	JUP,GATA5	down_VFEM_VSMC
GO:0003264	0.00017	1	2	5	regulation of cardioblast proliferation	BP	0.0857	EYA1,SIX1	up_VFEM_EC
GO:0003266	0.00017	1	2	5	regulation of secondary heart field cardioblast	BP	0.0857	EYA1,SIX1	up_VFEM_EC
GO:0010639	0.000172	1	24	128	negative regulation of organelle organization	BP	0.0154	ATAD2,ZW10,CAPG,TRIP13,SLC25A5,FBXO5,PRELID1,BIRC5,INSIG1,LIMA1,STMN1,NEK2,BRCA1,NOC2L,NDC80,ENSCAFG00000005299,BUB1,RBM14,CORO1A,TTK,ARHGEF2,TINF2,MTBP,GEN1	down_ACOR_VSMC

GO:0023051	0.000175	1	109	954	regulation of signaling	BP	0.0252	EPHB1,PLK2,TEK,HCLS1,IL7R,SELP,KIT,PRCP,CXCL8,PRKCH,ARHGEF15,FGD5,TNFAIP3,MAP3K11,TNIP1,AMOT,PDGFB,BAIAP2L1,RELN,FGD6,CXHXorf36,PDE4D,MEF2C,FERMT1,ATF3,CHD7,DLL4,RAPGEF1,TLR4,NLRC5,GPR4,GATA3,EDN1,APLNR,ARAP3,SNCAIP,HOMER3,EGFL7,FIGNL1,PELI1,PPP3CA,GUCY1A1,CASP8,NECTIN2,MTOR,ACPP,TSPAN14,PLEKHG1,MAP3K3,BMP2,HHEX,CHN2,CAV2,PREX2,BMP2K,ABCA1,PPP1R2,ITPKB,GDPD5,RIOK3,ARHGEF12,MADD,STMN1,PAK2,MCC,PRKD2,SH3GLB1,SMARCA4,TLR3,MFNG,AKAP12,APOD,NCK1,OTUD4,NET1,JAG1,GRM8,PTGS2,RAPGEF2,CTDSPL2,CREB1,BCL10,TAOK1,CTNNA1,CNOT7,HMGB1,PMAIP1,RIPK1,LSM14A,ITGA6,RGS2,SKIL,MCU,PDGFD,ARHGDIB,UFSP2,HIF1A,ARHGAP26,ALS2,LMTK2,ENSCAFG00000002015,TNFAIP1,IVNS1ABP,RGL2,JCAD,ZEB1,ARHGEF2,PEAR1,CDON	up_VCAV_EC
GO:0048731	0.000178	1	105	1247	system development	BP	0.0587	ARHGEF2,PRICKLE1,VEGFA,SIPA1L1,SULF1,SERPINH1,MYH9,NR1H4,HSPA5,SERPINE1,ACTN1,SOCS5,ETV6,SPARC,LTBP1,SGK1,PRKACB,PTPN14,ERRFI1,JCAD,CASP3,PGK1,BCAR3,PTPN11,AARS,FLNB,ENSCAFG00000032470,DPYSL3,HOOK3,IFRD1,TMED2,ILK,FHL2,PRCP,SMARCA4,TGFBR1,PTGS2,WDR1,PIK3C2A,CHSY1,MSN,HOXB7,KIF5B,HIF1A,SLC45A3,SLC20A1,THBS2,ACTG2,TNIK,NXN,UBA6,ZPR1,PTPN2,UCHL5,EIF2B1,EVI2B,NEO1,MTDH,ESM1,ACTA2,ADAMTS6,UST,CDH11,MAGED1,CTNNB1,ANLN,CRYAB,STARD13,TFRC,APOE,PLOD3,KLF5,LYN,STRAP,DDIT3,PDS5B,MMD,PLS3,ATF3,GSK3B,VLDLR,NEXN,MYO1E,SDC4,HTT,REST,MAPK10,PRMT1,SH2B3,EXT1,GLO1,DISC1,PAK2,CREB3L2,LRRTM2,INHBA,CAMK2D,BTK,SIX4,NRP1,SPINK2,ARF4,YWHAH,RTCB,HOMER1	down_VSAP_EC
GO:0009952	0.000183	1	4	66	anterior/posterior pattern specification	BP	0.0897	HOXC10,HOXA9,HOXB7,HOXA7	up_VFEM_EC
GO:0031333	0.000183612	0.99998	7	46	negative regulation of protein complex assembly	BP	0.3703682	TMOD1,SMAD6,PTGER4,CLSTN1,CLU,PRKCD,TUBB4A	up_ACOR_EC
GO:0010646	0.000185	1	108	945	regulation of cell communication	BP	0.0264	EPHB1,PLK2,TEK,HCLS1,IL7R,SELP,KIT,PRCP,CXCL8,PRKCH,ARHGEF15,FGD5,TNFAIP3,MAP3K11,TNIP1,AMOT,PDGFB,BAIAP2L1,RELN,FGD6,CXHXorf36,PDE4D,MEF2C,FERMT1,ATF3,CHD7,DLL4,RAPGEF1,TLR4,NLRC5,GPR4,GATA3,EDN1,APLNR,ARAP3,SNCAIP,HOMER3,EGFL7,FIGNL1,PELI1,PPP3CA,GUCY1A1,CASP8,NECTIN2,MTOR,ACPP,TSPAN14,PLEKHG1,MAP3K3,BMP2,HHEX,CHN2,CAV2,PREX2,BMP2K,ABCA1,PPP1R2,ITPKB,GDPD5,RIOK3,ARHGEF12,MADD,STMN1,PAK2,MCC,PRKD2,SH3GLB1,SMARCA4,TLR3,MFNG,AKAP12,APOD,NCK1,OTUD4,NET1,JAG1,GRM8,PTGS2,RAPGEF2,CTDSPL2,CREB1,BCL10,TAOK1,CTNNA1,CNOT7,HMGB1,PMAIP1,RIPK1,LSM14A,ITGA6,RGS2,SKIL,MCU,PDGFD,ARHGDIB,UFSP2,HIF1A,ARHGAP26,ALS2,ENSCAFG00000002015,TNFAIP1,IVNS1ABP,RGL2,JCAD,ZEB1,ARHGEF2,PEAR1,CDON	up_VCAV_EC
GO:0043009	0.000185	1	5	227	chordate embryonic development	BP	0.115	EYA1,HOXA9,TBX15,HOXA11,SHOX2	up_VFEM_VSMC
GO:0043009	0.000185	1	4	227	chordate embryonic development	BP	0.306	TBX15,EYA1,HOXA9,IRX5	up_AFEM_VSMC
GO:0007548	0.000185	1	4	68	sex differentiation	BP	0.149	HOXA11,HOXA9,SALL1,IRX5	up_VSAP_VSMC
GO:0022603	0.000188	1	44	301	regulation of anatomical structure morphogenesis	BP	0.0266	PLK2,HOXB7,TEK,KIT,CXCL8,IFRD1,TIE1,TNFAIP3,ADAMTS9,AMOT,RELN,CD34,MEF2C,DLL4,GPR4,GATA3,EDN1,RAB11A,APLNR,ARAP3,SMAD1,NTN4,NEDD9,PIK3C2A,PPP3CA,MTOR,MAP3K3,BMP2,HHEX,PDZD8,ANGPT2,PROX1,TSPAN12,PRKD2,TLR3,BTBD7,FES,RAPGEF2,FMNL3,HIF1A,ENSCAFG00000002015,VASH1,JCAD,ZMYM2	up_VCAV_EC
GO:0090330	0.000188	1	2	5	regulation of platelet aggregation	BP	0.16	LYN,PDPN	down_ACOR_EC

GO:0032502	0.000189	1	60	1661	developmental process	BP	0.509	TBX15,HOXA9,CMKLR1,IRX3,IRX5,HOXC10,LEP,LBH,XYLT1,GAS1,ANGPT4,HOXA7,COL8A2,CD40,DLL1,CRYAB,F3,VXN,PLXNA4,GPAM,NEFH,TWIST2,MAB21L1,THRB,LGI4,HCK,TMEM100,PLAGL2,GREM1,NFE2L1,OVOL1,CAMK2A,GREM2,HOXA6,CYLD,POSTN,LUZP1,MYBL1,RECK,PTHLH,TBXA2R,EVI2B,CNNM4,BVES,LGR6,PTPRB,AXIN2,ACACB,NAPEPLD,PPARGC1A,CDC42EP5,DMPK,ADAM17,CLIC4,LRRN2,HSPB2,GCNT4,FOSL2,KANK1,TNC	up_AFEM_EC
GO:2001251	0.000189	1	13	49	negative regulation of chromosome organization	BP	0.0166	ATAD2,ZW10,TRIP13,FBXO5,BIRC5,BRCA1,NOC2L,NDC80,ENSCAFG00000005299,BUB1,TTK,TINF2,GEN1	down_ACOR_VSMC
GO:0007186	0.000191	1	25	124	G protein-coupled receptor signaling pathway	BP	0.237	ADRA1B,ATRNL1,ACTN2,LGR6,PLCL1,ADGRD1,HTR2B,ABCA1,HRH1,GLRB,PTGIR,ADCY6,F2RL1,GNA14,APLP1,AGRN,SCG5,RGS2,SENP7,CCL5,EDN1,HPGD,USP20,GNAQ,INPP5K	up_ACOR_VSMC
GO:0043436	0.000194	1	49	303	oxoacid metabolic process	BP	0.494	PEX7,HNF4A,ACSM3,LIPC,INSIG2,ACAA1,ACACB,PEX2,GOT1,DLST,GLUL,ADHFE1,ENSCAFG00000013986,WARS2,C1QTNF2,ALDH6A1,HSD17B8,FAAH,PDK2,GALK1,PTGES,SUOX,HACD4,GCDH,ABAT,BCKDHB,ENSCAFG00000016140,AZIN2,DAGLB,MARC1,EHHADH,FARS2,LONP2,ACSS2,PC,DHRS9,ABHD16A,ENSCAFG00000002977,HPGD,QARS,ENSCAFG00000023820,IDH1,IDH3A,ENSCAFG00000006639,ASS1,CHST7,LARS2,DDIT4,IDO2	up_VSAP_EC
GO:0048856	0.000195	1	124	1533	anatomical structure development	BP	0.0619	ARHGEF2,PRICKLE1,VEGFA,SIPA1L1,SULF1,CELF1,SERPINH1,MYH9,TAGLN2,NR1H4,FMNL3,HSPA5,SERPINE1,ACTN1,SOCS5,ETV6,SPARC,LTBP1,SGK1,PRKACB,PTPN14,PTK7,ERRFI1,ITGA2,SEC24D,JCAD,CASP3,PGK1,AKAP2,BCAR3,PTPN11,EXOC5,FSCN1,AARS,FLNB,ENSCAFG00000032470,DPYSL3,HOOK3,IFRD1,TMED2,ILK,FHL2,PRCP,SMARCA4,TGFBR1,PTGS2,WDR1,PIK3C2A,CHSY1,MSN,HOXB7,KIF5B,HIF1A,FOXP4,SLC45A3,SLC20A1,THBS2,ACTG2,TNIK,NXN,UBA6,ZPR1,PTPN2,UCHL5,EIF2B1,EVI2B,NEO1,MTDH,ESM1,ADAM17,ACTA2,ADAMTS6,UST,CDH11,MAGED1,CTNNB1,ANLN,CRYAB,STARD13,ENSCAFG00000002015,TFRC,APOE,PLOD3,KLF5,LYN,BNIP3,STRAP,DDIT3,PDS5B,MMD,PLS3,ATF3,GSK3B,VLDLR,NEXN,MYO1E,SDC4,HTT,REST,RBBP8,MAPK10,PRMT1,SH2B3,EXT1,GLO1,DISC1,CDYL,PAK2,CREB3L2,LRRTM2,INHBA,CAMK2D,HIF1AN,BTK,SIX4,NRP1,SPINK2,ARF4,YWHAH,COL12A1,RTCB,KDM3A,MYBPC2,HOMER1	down_VSAP_EC
GO:0048869	0.000197	1	96	1119	cellular developmental process	BP	0.0619	ARHGEF2,PRICKLE1,VEGFA,SIPA1L1,SULF1,CELF1,SERPINH1,MYH9,TAGLN2,FMNL3,SERPINE1,ACTN1,SOCS5,ETV6,SPARC,SGK1,ERO1A,ENPP1,ERRFI1,ITGA2,CASP3,PGK1,AKAP2,NUAK1,PTPN11,FLNB,DPYSL3,HOOK3,TRIB3,IFRD1,ILK,FHL2,TGFBR1,PTGS2,WDR1,CHSY1,MSN,HOXB7,KIF5B,HIF1A,FOXP4,SLC45A3,TNIK,UBA6,ZPR1,PTPN2,EIF2B1,EVI2B,NEO1,ACTA2,UST,CDH11,CTNNB1,ANLN,ENSCAFG00000002015,TFRC,APOE,PLOD3,KLF5,LYN,BNIP3,STRAP,DDIT3,MMD,ATF3,GSK3B,VLDLR,NEXN,MYO1E,SDC4,REST,MAPK10,PRMT1,SH2B3,EXT1,GLO1,DISC1,CDYL,PAK2,CREB3L2,A2M,INHBA,CAMK2D,NR4A1,HIF1AN,BTK,SIX4,CDKN1A,NRP1,SPINK2,ARF4,YWHAH,COL12A1,KDM3A,MYBPC2,HOMER1	down_VSAP_EC
GO:0048731	0.000198	1	16	1247	system development	BP	0.16	ACVRL1,LYN,HOXA3,ACSL4,SPG11,PDPN,NRP2,HOXA7,ANPEP,SULF1,ELK3,HOXA1,CSGALNACT1,HOXC4,ASPN,NEDD4	down_ACOR_EC
GO:0009792	0.000199	1	4	231	embryo development ending in birth or egg	BP	0.306	TBX15,EYA1,HOXA9,IRX5	up_AFEM_VSMC
GO:0002684	0.0002	1	36	239	positive regulation of immune system process	BP	0.028	HCLS1,IL7R,GBP5,SELP,CXCL8,PRKCH,TNFAIP3,TNIP1,TMEM173,PDE4D,MEF2C,TLR4,NLRC5,EIF2B2,GATA3,PELI1,CASP8,NECTIN2,ITPKB,RIOK3,PRKD2,SWAP70,TLR3,NCK1,OTUD4,CD200,FES,JAG1,PLCG2,CREB1,BCL10,HMGB1,RIPK1,LSM14A,ATP11C,HIF1A	up_VCAV_EC

GO:0097028	0.000201	1	2	11	dendritic cell differentiation	BP	0.487	TMEM176A,TMEM176B	up_VPUL_VSMC
GO:0009792	0.000203	1	5	231	embryo development ending in birth or egg	BP	0.118	EYA1,HOXA9,TBX15,HOXA11,SHOX2	up_VFEM_VSMC
GO:0034613	0.000206	1	56	576	cellular protein localization	BP	0.0626	PRICKLE1,PLK3,HSPA5,HM13,TRAM1,PTPN14,SEC24D,PTPN11,EXOC5,SEC23A,MZT1,HOOK3,SESN2,SRP54,MYO1C,MSN,SEL1L,KIF5B,SSR1,IWS1,TNIK,ZPR1,STAM2,SMG7,XPOT,USO1,SRPRA,DDX39B,COPG1,AGRN,CTNNB1,KPNB1,COPB2,SRP72,APOE,KPNA4,DDIT3,TLN1,GSK3B,ARMCX3,GBP5,SH3GLB1,TAX1BP3,KPNA1,DISC1,KDELR2,AP3S1,CLTC,STX6,SIX4,CDKN1A,NRP1,ARF4,YWHAH,BLZF1,MID1	down_VSAP_EC
GO:1902531	0.000208	1	69	536	regulation of intracellular signal transduction	BP	0.0289	EPHB1,TEK,HCLS1,IL7R,SELP,KIT,ARHGEF15,FGD5,TNFAIP3,MAP3K11,TNIP1,AMOT,PDGFB,RELN,FGD6,PDE4D,ATF3,RAPGEF1,TLR4,GPR4,GATA3,EDN1,APLNR,ARAP3,HOMER3,FIGNL1,PELI1,PPP3CA,GUCY1A1,CASP8,MTOR,PLEKHG1,MAP3K3,BMP2,CHN2,PREX2,ABCA1,ITPKB,RIOK3,ARHGEF12,MADD,STMN1,PAK2,PRKD2,TLR3,AKAP12,NCK1,NET1,PTGS2,RAPGEF2,BCL10,TAOK1,CNOT7,HMGB1,PMAIP1,RIPK1,SKIL,PDGFD,ARHGDIB,HIF1A,ARHGAP26,ALS2,ENSCAFG00000002015,TNFAIP1,IVNS1ABP,RGL2,JCAD,ARHGEF2,CDON	up_VCAV_EC
GO:0034724	0.000208	1	6	12	DNA replication-independent nucleosome	BP	0.0181	OIP5,IPO4,CENPP,SUPT16H,HAT1,MIS18A	down_ACOR_VSMC
GO:0009954	0.000209	1	2	16	proximal/distal pattern formation	BP	0.306	HOXC10,HOXA9	up_AFEM_VSMC
GO:0003002	0.00021	1	4	118	regionalization	BP	0.118	HOXC10,HOXA9,EMX2,HOXA11	up_VFEM_VSMC
GO:0060914	0.000211	1	2	11	heart formation	BP	0.487	TBX5,GATA5	up_VPUL_VSMC
GO:0048513	0.000213	1	12	906	animal organ development	BP	0.152	HOXA11,TBX15,HOXA9,PITX1,EMX2,SALL1,HOXC10,IRX5,MAB21L1,CAV1,SHOX2,GJB5	up_VSAP_VSMC
GO:0048608	0.000213	1	5	129	reproductive structure development	BP	0.152	HOXA11,HOXA9,SALL1,IRX5,GJB5	up_VSAP_VSMC
GO:0072498	0.000213	1	2	8	embryonic skeletal joint development	BP	0.118	HOXA11,SHOX2	up_VFEM_VSMC
GO:0009952	0.000216	1	4	66	anterior/posterior pattern specification	BP	0.152	HOXA11,HOXA9,EMX2,HOXC10	up_VSAP_VSMC
GO:0048645	0.00022	1	2	33	animal organ formation	BP	0.64	HOXA3,TBX5	up_APUL_VSMC
GO:0030318	0.000224	1	5	8	melanocyte differentiation	BP	0.0309	EDNRB,KIT,ADAMTS9,MEF2C,RAB27A	up_VCAV_EC
GO:0060675	0.000226	1	3	29	ureteric bud morphogenesis	BP	0.107	EYA1,HOXB7,SIX1	up_VFEM_EC
GO:0061458	0.000227	1	5	131	reproductive system development	BP	0.152	HOXA11,HOXA9,SALL1,IRX5,GJB5	up_VSAP_VSMC
GO:0003002	0.000227	1	3	118	regionalization	BP	0.306	HOXC10,HOXA9,WNT7A	up_AORTRT_VSMC
GO:0070727	0.000234	1	56	579	cellular macromolecule localization	BP	0.0691	PRICKLE1,PLK3,HSPA5,HM13,TRAM1,PTPN14,SEC24D,PTPN11,EXOC5,SEC23A,MZT1,HOOK3,SESN2,SRP54,MYO1C,MSN,SEL1L,KIF5B,SSR1,IWS1,TNIK,ZPR1,STAM2,SMG7,XPOT,USO1,SRPRA,DDX39B,COPG1,AGRN,CTNNB1,KPNB1,COPB2,SRP72,APOE,KPNA4,DDIT3,TLN1,GSK3B,ARMCX3,GBP5,SH3GLB1,TAX1BP3,KPNA1,DISC1,KDELR2,AP3S1,CLTC,STX6,SIX4,CDKN1A,NRP1,ARF4,YWHAH,BLZF1,MID1	down_VSAP_EC
GO:0043586	0.000237	1	2	6	tongue development	BP	0.109	SIX1,CYP26B1	up_VFEM_EC
GO:0048598	0.000238	1	3	207	embryonic morphogenesis	BP	0.64	HOXA3,TBX5,HOXB5	up_APUL_VSMC
GO:0009790	0.000242	1	8	335	embryo development	BP	0.18	ACVRL1,HOXA3,ACSL4,NRP2,HOXA7,SULF1,HOXA1,HOXC4	down_ACOR_EC

GO:0032502	0.000243	1	132	1661	developmental process	BP	0.0691	ARHGEF2,PRICKLE1,VEGFA,SIPA1L1,SULF1,CELF1,SERPINH1,MYH9,TAGLN2,NR1H4,FMNL3,HSPA5,SERPINE1,ACTN1,SOCS5,ETV6,SPARC,LTBP1,SGK1,ERO1A,PRKACB,PTPN14,PTK7,ENPP1,ERRFI1,ITGA2,SEC24D,JCAD,CASP3,PGK1,AKAP2,BCAR3,NUAK1,PTPN11,EXOC5,FSCN1,AARS,FLNB,ENSCAFG00000032470,DPYSL3,HOOK3,TRIB3,IFRD1,TMED2,ILK,FHL2,PRCP,SMARCA4,TGFBR1,PTGS2,WDR1,PIK3C2A,CHSY1,MSN,HOXB7,KIF5B,HIF1A,FOXP4,SLC45A3,SLC20A1,THBS2,ACTG2,TNIK,NXN,UBA6,ZPR1,PTPN2,UCHL5,EIF2B1,EVI2B,NEO1,MTDH,ESM1,ADAM17,ACTA2,ADAMTS6,UST,CDH11,MAGED1,CTNNB1,ANLN,CRYAB,STARD13,ENSCAFG00000002015,TFRC,APOE,WWC3,PLOD3,KLF5,LYN,BNIP3,STRAP,DDIT3,PDS5B,MMD,PLS3,ATF3,GSK3B,VLDLR,NEXN,MYO1E,SDC4,HTT,REST,RBBP8,MAPK10,PRMT1,SH2B3,EXT1,GLO1,DISC1,CDYL,PAK2,CREB3L2,LRRTM2,A2M,INHBA,CAMK2D,NR4A1,HIF1AN,BTK,SIX4,CDKN1A,NRP1,SPINK2,ARF4,YWHAH,COL12A1,RTCB,KDM3A,MYBPC2,HOMER1	down_VSAP_EC
GO:0009653	0.000243	1	6	773	anatomical structure morphogenesis	BP	0.69	SLITRK5,LIMK1,GATA5,CA2,C6,TGFBI	up_VCAV_VSMC
GO:0051094	0.000245	1	7	388	positive regulation of developmental process	BP	0.493	TBX5,NR1H4,EVI2B,WIF1,CCL24,FLRT3,ARHGEF2	up_VPUL_EC
GO:0031960	0.000245	1	7	15	response to corticosteroid	BP	0.494	FAM107A,SLIT2,GOT1,ZFP36L1,CREBRF,ZFP36L2,DDIT4	up_VSAP_EC
GO:0051384	0.000245	1	7	15	response to glucocorticoid	BP	0.494	FAM107A,SLIT2,GOT1,ZFP36L1,CREBRF,ZFP36L2,DDIT4	up_VSAP_EC
GO:0030261	0.000246	1	5	8	chromosome condensation	BP	0.021	NCAPD2,SMC4,NCAPD3,NCAPH,SMC2	down_ACOR_VSMC
GO:0009653	0.000247	1	71	773	anatomical structure morphogenesis	BP	0.0691	ARHGEF2,PRICKLE1,VEGFA,SIPA1L1,SULF1,SERPINH1,MYH9,FMNL3,HSPA5,SERPINE1,ACTN1,SPARC,SGK1,PRKACB,PTPN14,PTK7,ERRFI1,ITGA2,JCAD,PGK1,AKAP2,BCAR3,PTPN11,EXOC5,FSCN1,FLNB,ENSCAFG00000032470,IFRD1,TMED2,ILK,FHL2,PRCP,TGFBR1,WDR1,PIK3C2A,CHSY1,MSN,HOXB7,KIF5B,HIF1A,FOXP4,THBS2,ACTG2,TNIK,UCHL5,NEO1,MTDH,ESM1,ADAM17,ACTA2,UST,CDH11,MAGED1,CTNNB1,CRYAB,STARD13,ENSCAFG00000002015,APOE,PLOD3,KLF5,BNIP3,VLDLR,MYO1E,SDC4,EXT1,INHBA,SIX4,NRP1,YWHAH,COL12A1,MYBPC2	down_VSAP_EC
GO:0044057	0.000248	1	2	110	regulation of system process	BP	0.438	JUP,GATA5	down_VFEM_VSMC
GO:0032956	0.000250732	0.99994	11	116	regulation of actin cytoskeleton organization	BP	0.44956164	TMOD1,ARHGAP6,SYNPO2,DIXDC1,ACTN2,ARHGAP28,PAK3,PTGER4,ARHGDIB,NRP1,PRKCD	up_ACOR_EC
GO:0045944	0.000254	1	43	287	positive regulation of transcription by RNA polymerase II	BP	0.0347	HCLS1,PPARG,TNIP1,PDGFB,TMEM173,MEF2C,ATF3,CHD7,TLR4,NLRC5,GATA3,EDN1,TCF4,ENSCAFG00000017103,IRF8,PPP3CA,YES1,BMP2,HHEX,CREM,PROX1,ARID4B,PRKD2,SMARCA4,TLR3,NCK1,LDB2,JAG1,CNOT7,HMGB1,AHR,RIPK1,ITGA6,MTA2,HIF1A,KDM3A,ATRX,NR4A1,ZEB1,TET2,ARHGEF2,CDON,ZNF609	up_VCAV_EC
GO:0072171	0.000254	1	3	30	mesonephric tubule morphogenesis	BP	0.114	EYA1,HOXB7,SIX1	up_VFEM_EC
GO:0006271	0.000256	1	4	5	DNA strand elongation involved in DNA	BP	0.0217	POLA1,POLE,FEN1,PCNA	down_ACOR_VSMC
GO:0043410	0.000263	1	28	144	positive regulation of MAPK cascade	BP	0.288	ADRA1B,LEP,MID1,HTR2B,RASSF2,BMP4,FGFR2,TLR3,SH3RF2,PDE5A,SASH1,FGF18,F2RL1,NRP1,PTPN22,NPNT,PRKCE,FRS2,APP,PSEN1,CCL5,DUSP22,EDN1,NOD2,SH3RF3,MAP3K7,ENSCAFG00000029553,CRKL	up_ACOR_VSMC
GO:0009896	0.000264	1	19	127	positive regulation of catabolic process	BP	0.0698	PRICKLE1,PLK3,SOCS5,TRIB3,SESN2,MSN,HIF1A,MTDH,CNOT7,APOE,BNIP3,GSK3B,CDC37,SH3GLB1,HTT,ABHD5,DISC1,RAD23A,TMTC3	down_VSAP_EC
GO:2001257	0.000268	1	3	30	regulation of cation channel activity	BP	0.173	NETO1,STIM1,CAV1	up_VSAP_VSMC

GO:0019752	0.00027	1	46	283	carboxylic acid metabolic process	BP	0.494	PEX7,HNF4A,ACSM3,LIPC,INSIG2,ACAA1,ACACB,PEX2,GOT1,DLST,GLUL,ADHFE1,ENSCAFG00000013986,WARS2,C1QTNF2,ALDH6A1,HSD17B8,FAAH,PDK2,GALK1,PTGES,HACD4,GCDH,ABAT,BCKDHB,ENSCAFG00000016140,AZIN2,DAGLB,EHHADH,FARS2,LONP2,ACSS2,PC,DHRS9,ABHD16A,ENSCAFG00000002977,HPGD,QARS,ENSCAFG00000023820,IDH1,IDH3A,ENSCAFG00000006639,ASS1,LARS2,DDIT4,IDO2	up_VSAP_EC
GO:0032502	0.000272	1	8	1661	developmental process	BP	0.69	HOXC6,SLITRK5,LIMK1,GATA5,CA2,LRRC17,C6,TGFBI	up_VCAV_VSMC
GO:0035480	0.000272	1	1	1	regulation of Notch signaling pathway involved in heart	BP	0.438	GATA5	down_VFEM_VSMC
GO:0035481	0.000272	1	1	1	positive regulation of Notch signaling pathway involved in heart	BP	0.438	GATA5	down_VFEM_VSMC
GO:0003137	0.000272	1	1	1	Notch signaling pathway involved in heart	BP	0.438	GATA5	down_VFEM_VSMC
GO:0061999	0.000272	1	1	1	regulation of cardiac endothelial to	BP	0.438	GATA5	down_VFEM_VSMC
GO:0062000	0.000272	1	1	1	positive regulation of cardiac endothelial to mesenchymal transition	BP	0.438	GATA5	down_VFEM_VSMC
GO:0090381	0.000272	1	1	1	regulation of heart induction	BP	0.438	GATA5	down_VFEM_VSMC
GO:1901321	0.000272	1	1	1	positive regulation of heart induction	BP	0.438	GATA5	down_VFEM_VSMC
GO:0007389	0.000274	1	4	137	pattern specification process	BP	0.788	HOXA3,BARX1,NKX2-5,CYP26B1	up_VPOR_VSMC
GO:0044085	0.000276	1	120	1076	cellular component biogenesis	BP	0.0231	ACSL3,FSCN1,HMGB1,IRAK1,ANLN,STXBP6,USP4,HMGA1,HMGCR,EDC3,SMC1A,UBE2S,RRM1,ZW10,CAPG,CENPA,SLC29A1,NDC1,CENPN,TRIP13,C1QTNF6,NOP56,SDC1,FBXO5,HSPA9,RAN,CFAP298,PSMD13,BIRC5,INSIG1,PRMT1,CAV1,RAD51,LIMA1,NF2,AURKB,TK1,STMN1,CENPO,HAUS8,GREM1,NEK2,FBL,RCC1,DHX33,DIS3,GMNN,CENPH,POC1A,NOC4L,WDR62,CENPI,FASTKD3,NOC2L,B4GALT1,FARSA,PREB,OIP5,IPO4,HIST1H1A,RAC1,NUP62,EMP2,DDX28,PDXP,FOSL1,PLK4,RSL1D1,IQGAP2,SAMM50,RRP1,HMGB2,CDC42EP4,HIST1H2BB,SMC3,CDK2,DDX39B,STAG1,ENSCAFG00000005299,EZR,SF3A2,SASS6,RBM14,CORO1A,CENPW,FOXRED1,CDC20,SIGMAR1,ENSCAFG00000019620,ASPM,ARPC1B,NOP14,CENPP,GRWD1,ENSCAFG00000010224,PRMT5,PSMC1,CTH,NOP58,TRAPPC6A,ENSCAFG00000015189,KIF11,HAT1,TINF2,EMG1,EXOSC5,GEMIN7,UBE2C,MIS18A,TBCC,SIX4,H3F3B,CEP83,PDSS1,TTLL1,TPBG,SF3A1,EIF3M,NOB1,KIF24	down_ACOR_VSMC
GO:1903010	0.000276	1	4	8	regulation of bone development	BP	0.446	NOTCH2,FBN1,GLI3,GREM1	down_VCAV_EC
GO:0048705	0.000276	1	3	95	skeletal system morphogenesis	BP	0.372	TBX15,EYA1,IRX5	up_AFEM_VSMC
GO:1900120	0.000277	1	2	7	regulation of receptor binding	BP	1	HOXA3,PLCL1	up_APUL_EC
GO:1904018	0.000278	1	15	69	positive regulation of vasculature development	BP	0.0371	PLK2,TEK,KIT,CXCL8,PDGFB,CD34,APLNR,SMAD1,PIK3C2A,MAP3K3,PRKD2,TLR3,RAPGEF2,HIF1A,JCAD	up_VCAV_EC
GO:0001501	0.00028	1	6	178	skeletal system development	BP	0.185	HOXA3,HOXA7,SULF1,HOXA1,CSGALNACT1,HOXC4	down_ACOR_EC
GO:0030198	0.000281	1	19	81	extracellular matrix organization	BP	0.288	FMOD,PRDX4,RECK,FERMT1,MFAP4,SPINT1,CTSS,ERO1B,NPNT,SPINT2,P4HA1,DAG1,APP,ECM2,DDR2,NID1,SERPINB5,KIF9,TNFRSF11B	up_ACOR_VSMC
GO:0006613	0.000281	1	5	12	cotranslational protein targeting to membrane	BP	0.0733	TRAM1,SRP54,SSR1,SRPRA,SRP72	down_VSAP_EC
GO:0003274	0.000281	1	1	2	endocardial cushion fusion	BP	0.517	GATA5	down_AFEM_VSMC
GO:0009888	0.000282	1	9	545	tissue development	BP	0.12	EYA1,TOX,HOXB7,HOXA7,SIX1,TIAM1,CYP26B1,MTSS1,CA9	up_VFEM_EC
GO:0048869	0.000283	1	13	1119	cellular developmental process	BP	0.12	HOXC10,EYA1,HOXA9,TOX,MME,HOXB7,HOXA7,SIX1,TIAM1,RADIL,CYP26B1,CDC42EP5,MTSS1	up_VFEM_EC
GO:0000380	0.000284	1	8	22	alternative mRNA splicing, via spliceosome	BP	0.0235	RNPS1,SRSF2,THRAP3,HNRNPL,RBM14,RBM17,WTAP,SF3A1	down_ACOR_VSMC

GO:0031536	0.000284	1	3	3	positive regulation of exit from mitosis	BP	0.0235	BIRC5,ENSCAFG00000005299,UBE2C	down_ACOR_VSMC
GO:0007154	0.000286	1	194	1622	cell communication	BP	0.288	SH3TC2,FGF13,RHOT1,ADRA1B,VEGFD,TLR1,SMAD6,ATRNL1,ARHGAP28,ACTN2,LGR6,PLCG2,IFNGR1,LEP,PLCL1,LBH,MAP3K8,MID1,GATA5,ADGRD1,CD83,EFNB2,ANGPT1,ENSCAFG00000016285,HTR2B,RASSF2,MBP,ZRANB1,FBXL20,C28H10orf71,RECK,SKP2,NACC2,PRKAA2,MLLT3,ITM2C,FERMT1,BMP4,TM7SF3,FGFR2,ABCA1,GJA1,DOCK3,ELF1,ATRX,MED14,HRH1,CASTOR1,TLR3,SH3RF2,LATS2,PDE5A,CITED2,TP53INP1,RERG,GLRB,ARHGAP29,ZNRF3,CD274,PLPP2,AR,PTGIR,SASH1,STX11,GPR155,TMEM108,FGF18,ARHGEF26,NDRG2,CHN2,CDKN2B,ADCY6,ITPR1,GRK7,LIMS2,BLNK,ANKRD1,F2RL1,ADGRL1,CAMK2D,PSEN2,PPP2R5E,GABARAPL1,DAPK1,ARHGAP5,BNIP3,EGLN1,CACNB3,NCEH1,SLC12A7,AKAP6,CDKN1A,JMY,GNA14,CRLF3,ENSCAFG00000006238,CLSTN1,LRRC8C,CREBRF,NRP1,UBR1,PDE1A,PTPN22,DMPK,NPNT,UNC5B,SMPD1,RCAN2,APLP1,SLITRK5,TBX20,SESN3,SH3RF1,TBX5,CCDC3,PRKCE,FZD3,FRS2,AGRN,RNF19A,RND1,SCG5,RGS2,DAG1,FUT8,PARP9,APP,PSEN1,TGFBR1,SYNJ2BP,CHD7,TESK2,PIK3C3,LAMP2,JUP,SIPA1L1,SENP7,CCL5,WWC2,PERP,PSKH1,RTKN2,DUSP22,SIAH1,TIRAP,RAB11B,EDN1,SESN1,HPGD,KCNC4,DUSP26,NOD2,LRRC4,A	up_ACOR_VSMC
GO:0050776	0.000286	1	28	173	regulation of immune response	BP	0.0378	IL7R,GBP5,KIT,PRKCH,TNFAIP3,TNIP1,TMEM173,PDE4D,MEF2C,TLR4,NLRC5,EIF2B2,GATA3,PELI1,ARG2,NECTIN2,RIOK3,PRKD2,THOC1,TLR3,OTUD4,CD200,FES,PLCG2,BCL10,CNOT7,HMGB1,LSM14A	up_VCAV_EC
GO:0034110	0.000286	1	2	6	regulation of homotypic cell-cell adhesion	BP	0.185	LYN,PDPN	down_ACOR_EC
GO:0140074	0.000288	1	1	2	cardiac endothelial to mesenchymal transition	BP	0.517	GATA5	down_AFEM_VSMC
GO:0006268	0.000289	1	4	5	DNA unwinding involved in DNA replication	BP	0.0238	MCM6,MCM4,MCM7,RAD51	down_ACOR_VSMC
GO:0046907	0.000291	1	52	536	intracellular transport	BP	0.0746	PRICKLE1,PLK3,HM13,ANKRD50,TRAM1,PTPN14,SEC24D,HYOU1,PTPN11,SEC23A,HOOK3,PPP1R12A,SRP54,TMEM87B,MYO1C,MSN,RAB6A,SEL1L,RPGR,KIF5B,LMAN1,HIF1A,DYNLL1,TRAK1,SSR1,IWS1,YIPF5,ZPR1,STAM2,SMG7,XPOT,USO1,SRPRA,DDX39B,COPG1,CTNNB1,KPNB1,COPB2,SRP72,CRYAB,KPNA4,LYN,GSK3B,KPNA1,HTT,AP3S1,CLTC,CREB3L2,STX6,CDKN1A,YWHAH,SEC13	down_VSAP_EC
GO:0061640	0.000291	1	8	23	cytoskeleton-dependent cytokinesis	BP	0.0238	ANLN,CENPA,BIRC5,AURKB,STMN1,NUP62,RAB35,ENSCAFG00000005299	down_ACOR_VSMC
GO:0030178	0.000294	1	3	52	negative regulation of Wnt signaling pathway	BP	0.788	BARX1,NKX2-5,ANKRD6	up_VPOR_VSMC
GO:0006275	0.000296	1	11	38	regulation of DNA replication	BP	0.0241	SMC1A,RFC2,RFC5,RFC3,RFC4,PCNA,GMNN,E2F8,SMC3,CDK2,GLI1	down_ACOR_VSMC
GO:0021545	0.0003	1	3	21	cranial nerve development	BP	0.186	HOXA3,NRP2,HOXA1	down_ACOR_EC
GO:0000281	0.000301	1	7	18	mitotic cytokinesis	BP	0.0244	ANLN,CENPA,BIRC5,STMN1,NUP62,RAB35,ENSCAFG00000005299	down_ACOR_VSMC
GO:0007267	0.000304	1	19	332	cell-cell signaling	BP	0.612	LEP,KCNC4,CRY2,DLL1,GPR1,CHRM2,SLC6A6,GREM1,HTR1D,FAM53B,CAMK2A,CYLD,RECK,LGR6,AXIN2,CCL5,DMPK,KANK1,TNC	up_AFEM_EC
GO:0007127	0.000306	1	9	28	meiosis I	BP	0.0246	ENSCAFG00000017264,NDC1,TRIP13,FBXO5,PSMD13,RAD51,MND1,CKS2,ESPL1	down_ACOR_VSMC
GO:0061982	0.000306	1	9	28	meiosis I cell cycle process	BP	0.0246	ENSCAFG00000017264,NDC1,TRIP13,FBXO5,PSMD13,RAD51,MND1,CKS2,ESPL1	down_ACOR_VSMC
GO:0048015	0.000312	1	13	48	phosphatidylinositol-mediated signaling	BP	0.0403	TEK,HCLS1,SELP,PDGFB,RELN,GATA3,EDN1,PIK3C2A,PREX2,PITPNM2,PDGFD,RGL2,PEAR1	up_VCAV_EC
GO:0048017	0.000312	1	13	48	inositol lipid-mediated signaling	BP	0.0403	TEK,HCLS1,SELP,PDGFB,RELN,GATA3,EDN1,PIK3C2A,PREX2,PITPNM2,PDGFD,RGL2,PEAR1	up_VCAV_EC
GO:0043009	0.000318	1	6	227	chordate embryonic development	BP	0.131	TBX15,EYA1,HOXA9,HOXB7,HOXA7,SIX1	up_VFEM_EC
GO:0016055	0.000322	1	4	134	Wnt signaling pathway	BP	0.788	BARX1,NKX2-5,BAMBI,ANKRD6	up_VPOR_VSMC
GO:0198738	0.000322	1	4	134	cell-cell signaling by wnt	BP	0.788	BARX1,NKX2-5,BAMBI,ANKRD6	up_VPOR_VSMC
GO:0032501	0.000325	1	17	1782	multicellular organismal process	BP	0.201	HOXA11,TBX15,HOXA9,PITX1,EMX2,NETO1,GATM,SALL1,HOXC10,IRX5,MAB21L1,TLR2,CAV1,SHOX2,F5,HAPLN1,GJB5	up_VSAP_VSMC

GO:0048333	0.000327	1	2	12	mesodermal cell differentiation	BP	0.176	EYA1,HOXA11	up_VFEM_VSMC
GO:0016192	0.000329	1	45	452	vesicle-mediated transport	BP	0.0805	VEGFA,MYH9,SERPINE1,ANKRD50,GOLT1B,SEC24D,ENSCAFG00000003477,HYOU1,EXOC5,SEC23A,HOOK3,TMEM87B,CANX,PIK3C2A,MSN,RAB6A,LMAN1,TRAK1,YIPF5,USO1,COPG1,CTNNB1,COPB2,TFRC,APOE,LYN,GPR107,GSK3B,MYO1E,SDC4,HTT,REST,CLINT1,SCAMP1,RAB11FIP1,AP3S1,CLTC,CREB3L2,LRRTM2,STX6,ICAM5,ARF4,BLZF1,SEC13,RINT1	down_VSAP_EC
GO:0001708	0.00033	1	2	20	cell fate specification	BP	0.39	HOXC10,EYA1	up_AFEM_VSMC
GO:0003008	0.000339	1	52	336	system process	BP	0.317	TNNT2,FGF13,ADRA1B,ACTN2,LEP,SLC26A7,KCNE3,GATA5,HTR2B,MBP,COCH,BMP4,NPPB,GJA1,HRH1,LRRN4,WASF3,PDE5A,GLRB,SLC2A5,GPR155,TMEM108,ADCY6,USP53,F2RL1,HRH2,CLSTN1,ABCA4,DMPK,NPNT,PTGDR,TBX20,RNLS,POMK,APP,PSEN1,CHD7,JUP,CLN8,EDN1,NOD2,FAM107A,INSYN1,JAM3,NCALD,GAA,INPP5K,WFS1,DOCK5,ERAP1,SLC8A1,BVES	up_ACOR_VSMC
GO:0048562	0.000339	1	3	102	embryonic organ morphogenesis	BP	0.39	TBX15,EYA1,IRX5	up_AFEM_VSMC
GO:0072078	0.000339	1	3	33	nephron tubule morphogenesis	BP	0.134	EYA1,HOXB7,SIX1	up_VFEM_EC
GO:0060351	0.000341	1	2	11	cartilage development involved in endochondral bone morphogenesis	BP	0.178	HOXA11,SHOX2	up_VFEM_VSMC
GO:0007275	0.000342	1	9	1370	multicellular organism development	BP	0.547	HOXA3,TBX5,GATA5,HOXA2,TMEM176A,TMEM176B,PHEX,HOXB5,SLIT2	up_VPUL_VSMC
GO:0048536	0.000342	1	2	12	spleen development	BP	0.788	BARX1,NKX2-5	up_VPOR_VSMC
GO:2000826	0.000342	1	2	14	regulation of heart morphogenesis	BP	0.547	TBX5,GATA5	up_VPUL_VSMC
GO:0007096	0.000345	1	4	6	regulation of exit from mitosis	BP	0.0275	ZW10,BIRC5,ENSCAFG00000005299,UBE2C	down_ACOR_VSMC
GO:0043933	0.000348	1	83	698	protein-containing complex subunit organization	BP	0.0277	ACSL3,FSCN1,HMGB1,IRAK1,STXBP6,USP4,HMGCR,EDC3,UBE2S,RRM1,ZW10,CAPG,SMC4,CENPA,SLC29A1,NDC1,CENPN,COLGALT1,C1QTNF6,FBXO5,PSMD13,INSIG1,PRMT1,CAV1,RAD51,LIMA1,TK1,STMN1,CENPO,GREM1,SMARCC1,RCC1,DHX33,GMNN,CENPH,CENPI,FASTKD3,EIF5A,B4GALT1,SMC2,FARSA,PREB,NDC80,OIP5,IPO4,HIST1H1A,RAC1,DDX28,PDXP,FOSL1,IQGAP2,SAMM50,HMGB2,CDC42EP4,HIST1H2BB,SMARCB1,DDX39B,SF3A2,CORO1A,CENPW,FOXRED1,SIGMAR1,ARPC1B,CENPP,GRWD1,PRMT5,PSMC1,CTH,ARHGEF2,ENSCAFG00000015189,SUPT16H,HAT1,SERPINH1,LIPC,GEMIN7,UBE2C,MIS18A,TBCC,H3F3B,PDSS1,SF3A1,EIF3M,KIF24	down_ACOR_VSMC
GO:0051241	0.000349	1	4	336	negative regulation of multicellular organismal	BP	0.38	HOXC10,HOXA9,WNT7A,ARG2	up_AORTRT_VSMC
GO:0009792	0.000351	1	6	231	embryo development ending in birth or egg	BP	0.134	TBX15,EYA1,HOXA9,HOXB7,HOXA7,SIX1	up_VFEM_EC
GO:0032502	0.000353	1	16	1661	developmental process	BP	0.134	HOXC10,TBX15,EYA1,HOXA9,TOX,MME,HOXB7,HOXA7,SIX1,MAB21L1,TIAM1,RADIL,CYP26B1,CDC42EP5,MTSS1,CA9	up_VFEM_EC
GO:0043009	0.000355	1	6	227	chordate embryonic development	BP	0.212	HOXA11,TBX15,HOXA9,IRX5,SHOX2,GJB5	up_VSAP_VSMC
GO:0007389	0.000355	1	3	137	pattern specification process	BP	0.38	HOXC10,HOXA9,WNT7A	up_AORTRT_VSMC
GO:0048608	0.000356	1	3	129	reproductive structure development	BP	0.38	HOXA9,WNT7A,ESR1	up_AORTRT_VSMC
GO:0009954	0.000358	1	7	16	proximal/distal pattern formation	BP	0.494	HOXA11,HOXA9,DLL1,OSR1,IRX3,HOXC10,LRP4	up_VSAP_EC
GO:0060795	0.000358	1	2	12	cell fate commitment involved in formation of	BP	0.181	EYA1,HOXA11	up_VFEM_VSMC
GO:0060284	0.000361	1	34	212	regulation of cell development	BP	0.0462	EDNRB,PRKCH,IFRD1,RELN,MEF2C,CHD7,DLL4,RAPGEF1,GATA3,EDN1,RAB11A,TCF4,NEDD9,PPP3CA,MTOR,HOOK3,BMP2,HMG20A,GDPD5,PROX1,NCK1,FES,JAG1,MMD,RAPGEF2,ZC4H2,CTNNA1,HIF1A,ENSCAFG00000002015,ZEB1,ARHGEF2,CDON,TJP2,ZNF609	up_VCAV_EC
GO:0051241	0.000361	1	5	336	negative regulation of multicellular organismal	BP	0.547	TBX5,GATA5,TMEM176A,TMEM176B,SLIT2	up_VPUL_VSMC

GO:0072359	0.000362	1	7	368	circulatory system development	BP	1	TBX5,HOXA3,ZDHHC16,SLITRK5,NTRK2,HOXA1,PDLIM3	up_APUL_EC
GO:2000136	0.000362	1	2	7	regulation of cell proliferation involved in	BP	0.134	EYA1,SIX1	up_VFEM_EC
GO:0048568	0.000365	1	5	148	embryonic organ development	BP	0.134	TBX15,EYA1,HOXB7,HOXA7,SIX1	up_VFEM_EC
GO:0072359	0.000366	1	50	368	circulatory system development	BP	0.0465	EPHB1,PLK2,TEK,ADGRF5,KIT,PRCP,CXCL8,ARHGEF15,EMCN,CLEC14A,CALCRL,TIE1,ADAMTS9,AMOT,PDGFB,CD34,MEF2C,CHD7,TIPARP,DLL4,RAPGEF1,GPR4,CDH5,GATA3,EDN1,APLNR,SMAD1,SOX18,PIK3C2A,PTPRB,EPHB4,MTOR,MAP3K3,BMP2,HHEX,ANGPT2,PROX1,ENSCAFG00000010596,TSPAN12,PRKD2,TLR3,JAG1,RAPGEF2,CREB1,ADIPOR2,HIF1A,LUZP1,VASH1,JCAD,ELK3	up_VCAV_EC
GO:0051216	0.000371	1	4	70	cartilage development	BP	0.206	HOXA3,SULF1,CSGALNACT1,HOXC4	down_ACOR_EC
GO:0061323	0.000373	1	2	7	cell proliferation involved in heart morphogenesis	BP	0.134	EYA1,SIX1	up_VFEM_EC
GO:0035136	0.000374	1	7	16	forelimb morphogenesis	BP	0.494	HOXA11,HOXA9,OSR2,OSR1,ZBTB16,RECK,SHOX2	up_VSAP_EC
GO:0006469	0.000377	1	4	78	negative regulation of protein kinase activity	BP	0.206	LYN,IGF1R,PRKAR2A,ASPN	down_ACOR_EC
GO:0061458	0.000377	1	3	131	reproductive system development	BP	0.38	HOXA9,WNT7A,ESR1	up_AORTRT_VSMC
GO:0030334	0.00038	1	7	267	regulation of cell migration	BP	0.206	ACVRL1,LYN,IGF1R,PDPN,HOXA7,SULF1,STX4	down_ACOR_EC
GO:0042058	0.000381	1	6	17	regulation of epidermal growth factor receptor	BP	0.0884	SOCS5,ERRFI1,PTPN2,ADAM17,CBLC,RHBDF2	down_VSAP_EC
GO:0008016	0.000383	1	13	48	regulation of heart contraction	BP	0.317	TNNT2,FGF13,ADRA1B,KCNE3,GJA1,PDE5A,DMPK,RNLS,JUP,EDN1,GAA,SLC8A1,BVES	up_ACOR_VSMC
GO:0032501	0.000386	1	19	1782	multicellular organismal process	BP	0.206	ACVRL1,LYN,HOXA3,IGF1R,ACSL4,SPG11,PDPN,NRP2,HIVEP2,HOXA7,ANPEP,SULF1,ELK3,HOXA1,CSGALNACT1,ACSL3,HOXC4,ASPN,NEDD4	down_ACOR_EC
GO:0035088	0.000388	1	5	12	establishment or maintenance of	BP	0.0884	FSCN1,ILK,WDR1,MSN,ARF4	down_VSAP_EC
GO:0061245	0.000388	1	5	12	establishment or maintenance of bipolar	BP	0.0884	FSCN1,ILK,WDR1,MSN,ARF4	down_VSAP_EC
GO:0097435	0.000390588	0.99988	16	230	supramolecular fiber organization	BP	0.61799576	MYOZ2,FMOD,TMOD1,ARHGAP6,SYNPO2,PDLIM3,PLS1,ACTN2,ARHGAP28,PAK3,PTGER4,FOXC2,NRP1,PRKCD,MFAP4,TUBB4A	up_ACOR_EC
GO:0045930	0.000391	1	19	97	negative regulation of mitotic cell cycle	BP	0.0308	ZW10,TRIP13,FBXO5,BIRC5,CDK1,CASP2,FANCI,TOPBP1,EZH2,MAP3K20,TRIM35,NDC80,ENSCAFG00000005299,CLSPN,BUB1,TTK,MTBP,ZWINT,GEN1	down_ACOR_VSMC
GO:0023052	0.000393	1	191	1602	signaling	BP	0.317	SH3TC2,FGF13,RHOT1,ADRA1B,VEGFD,TLR1,SMAD6,ATRNL1,ARHGAP28,ACTN2,LGR6,PLCG2,IFNGR1,LEP,PLCL1,LBH,MAP3K8,MID1,KCNE3,GATA5,ADGRD1,CD83,EFNB2,ANGPT1,ENSCAFG00000016285,HTR2B,RASSF2,MBP,ZRANB1,FBXL20,C28H10orf71,RECK,SKP2,NACC2,PRKAA2,MLLT3,ITM2C,FERMT1,BMP4,TM7SF3,FGFR2,ABCA1,GJA1,DOCK3,ELF1,ATRX,MED14,HRH1,CASTOR1,TLR3,SH3RF2,LATS2,PDE5A,CITED2,TP53INP1,RERG,GLRB,ARHGAP29,ZNRF3,CD274,PLPP2,AR,PTGIR,SASH1,STX11,GPR155,TMEM108,FGF18,ARHGEF26,NDRG2,CHN2,CDKN2B,ADCY6,ITPR1,GRK7,LIMS2,BLNK,ANKRD1,F2RL1,ADGRL1,CAMK2D,PSEN2,PPP2R5E,DAPK1,ARHGAP5,BNIP3,EGLN1,CACNB3,NCEH1,SLC12A7,AKAP6,CDKN1A,JMY,GNA14,CRLF3,CLSTN1,LRRC8C,CREBRF,NRP1,UBR1,PDE1A,PTPN22,DMPK,NPNT,UNC5B,SMPD1,RCAN2,APLP1,SLITRK5,TBX20,SESN3,SH3RF1,TBX5,CCDC3,PRKCE,FZD3,FRS2,AGRN,RNF19A,RND1,SCG5,RGS2,DAG1,FUT8,PARP9,APP,PSEN1,TGFBR1,SYNJ2BP,CHD7,TESK2,PIK3C3,JUP,SIPA1L1,SENP7,CCL5,WWC2,PERP,PSKH1,RTKN2,DUSP22,SIAH1,TIRAP,RAB11B,EDN1,SESN1,HPGD,KCNC4,DUSP26,NOD2,LRRC4,ARID5B,FAM107A,INSYN1,DDIT4,JAM	up_ACOR_VSMC
GO:0009792	0.000394	1	6	231	embryo development ending in birth or egg	BP	0.227	HOXA11,TBX15,HOXA9,IRX5,SHOX2,GJB5	up_VSAP_VSMC
GO:0048731	0.000395	1	7	1247	system development	BP	0.69	HOXC6,SLITRK5,LIMK1,GATA5,LRRC17,C6,TGFBI	up_VCAV_VSMC

GO:0051239	0.000396	1	36	859	regulation of multicellular organismal process	BP	0.632	HOXA9,CMKLR1,IRX3,IRX5,HOXC10,LEP,LBH,CRY2,ANGPT4,HOXA7,DLL1,F3,PLXNA4,BPI,GPAM,LMCD1,CHRM2,TWIST2,THRB,LGI4,TMEM100,GREM1,AVPR1A,CAMK2A,CYLD,PTHLH,TBXA2R,EVI2B,BVES,AXIN2,ACACB,PPARGC1A,DMPK,ADAM17,AGPAT2,KANK1	up_AFEM_EC
GO:0009888	0.000398	1	6	545	tissue development	BP	0.547	HOXA3,TBX5,GATA5,PHEX,HOXB5,SLIT2	up_VPUL_VSMC
GO:0051054	0.00041	1	17	81	positive regulation of DNA metabolic process	BP	0.0321	HMGB1,HNRNPD,SLC29A1,RFC2,RFC5,RFC3,TIMELESS,AURKB,GREM1,RFC4,NEK2,PCNA,E2F8,TFRC,CDK2,GLI1,CCT4	down_ACOR_VSMC
GO:1903522	0.000413	1	7	58	regulation of blood circulation	BP	0.632	IRX5,CHRM2,THRB,AVPR1A,TBXA2R,BVES,DMPK	up_AFEM_EC
GO:0006954	0.000415	1	24	147	inflammatory response	BP	0.0524	ACKR1,GBP5,SELP,KIT,PRCP,CXCL8,F8,IL6,TNFAIP3,TNIP1,BCL6B,TLR4,GATA3,PTGS1,SNAP23,BMP2,TLR3,APOD,PTGS2,HMGB1,NOS2,NR1D2,ENSCAFG00000025410,HIF1A	up_VCAV_EC
GO:0030878	0.000416	1	2	13	thyroid gland development	BP	0.839	HOXA3,NKX2-5	up_VPOR_VSMC
GO:0060429	0.000418	1	7	341	epithelium development	BP	0.14	EYA1,HOXB7,HOXA7,SIX1,CYP26B1,MTSS1,CA9	up_VFEM_EC
GO:0003129	0.000421	1	1	3	heart induction	BP	0.628	GATA5	down_AFEM_VSMC
GO:2001224	0.000422	1	2	5	positive regulation of neuron migration	BP	1	ARHGEF2,SEMA3A	up_VPOR_EC
GO:0061343	0.000428	1	1	3	cell adhesion involved in heart morphogenesis	BP	0.628	GATA5	down_AFEM_VSMC
GO:0032501	0.000429	1	10	1782	multicellular organismal process	BP	0.547	HOXA3,TBX5,GATA5,HOXA2,TMEM176A,TMEM176B,LRRN4,PHEX,HOXB5,SLIT2	up_VPUL_VSMC
GO:0072088	0.000431	1	3	36	nephron epithelium morphogenesis	BP	0.142	EYA1,HOXB7,SIX1	up_VFEM_EC
GO:0060065	0.000434	1	2	7	uterus development	BP	0.242	HOXA11,HOXA9	up_VSAP_VSMC
GO:0003197	0.00044	1	2	16	endocardial cushion development	BP	0.547	TBX5,GATA5	up_VPUL_VSMC
GO:0048704	0.000445	1	2	46	embryonic skeletal system morphogenesis	BP	0.898	HOXA3,HOXB5	up_APUL_VSMC
GO:0044843	0.000446	1	14	62	cell cycle G1/S phase transition	BP	0.0347	HINFP,POLE,CASP2,CDKN2C,EZH2,CDK4,PLK3,RCC1,CDK2,CCNA2,RBBP8,GLI1,CDKN3,MTBP	down_ACOR_VSMC
GO:0002768	0.000449	1	10	37	immune response-regulating cell surface receptor signaling	BP	0.0562	KIT,PRKCH,PDE4D,MEF2C,EIF2B2,GATA3,NECTIN2,PRKD2,PLCG2,BCL10	up_VCAV_EC
GO:0019935	0.000450587	0.99995	6	38	cyclic-nucleotide-mediated signaling	BP	0.61799576	SOX9,PTH1R,AQP1,PTGIR,PDE4A,GUCY1B1	up_ACOR_EC
GO:0035115	0.000457	1	6	12	embryonic forelimb morphogenesis	BP	0.494	HOXA11,HOXA9,OSR2,OSR1,RECK,SHOX2	up_VSAP_EC
GO:0050794	0.000459562	0.99973	104	3075	regulation of cellular process	BP	0.61799576	CNTNAP4,SH3TC2,CDH3,NR1H4,TMOD1,GATA4,BRSK1,TRPS1,PLCL1,PKIB,CALY,SOX9,RHOU,PKNOX2,ARHGAP6,PTH1R,RGS1,PCSK1N,NDRG2,AQP1,PORCN,ENSCAFG00000004331,SYNPO2,KCNB1,ADAMTS13,EDEM2,FZD5,CCDC3,AGRN,PDE1A,IFI6,SEMA6D,GNAO1,DUSP26,DIXDC1,RASL11A,PTGIR,NOD2,RGMA,CASP10,LHX9,PDE4A,INSYN1,SMAD6,KLHL25,PLCE1,PLS1,ACTN2,ZMYND8,ARHGAP28,ESM1,PAK3,MMP28,GUCY1B1,GRK7,BCL2,TNFRSF19,PTGER4,ARHGDIB,PREB,CST3,PIAS1,DCLK2,ADAMTSL2,MAPK8IP2,RAPGEF4,MAVS,FOXC2,FIGNL1,LRRC15,IFIT2,TBX5,LRP4,CLSTN1,SH3RF3,LRRC4,RBM38,CLU,SFMBT2,C23H3orf58,ZNF229,CHMP4B,TP53INP2,RARRES2,VPS16,GMEB2,RIOX2,PRKACA,RNF114,STK32C,NRP1,ARHGEF26,PRKCD,GNAZ,RIN2,PPP1R26,PM20D1,PYGO2,DBNDD2,EFEMP1,CCNYL1,ADGRL1,TUBB4A,TLE2	up_ACOR_EC
GO:0048568	0.000464	1	4	148	embryonic organ development	BP	0.227	EYA1,TBX15,HOXA11,SHOX2	up_VFEM_VSMC
GO:0006631	0.000466	1	20	93	fatty acid metabolic process	BP	0.494	PEX7,ACSM3,LIPC,INSIG2,ACAA1,ACACB,PEX2,C1QTNF2,HSD17B8,FAAH,PDK2,PTGES,HACD4,GCDH,DAGLB,EHHADH,LONP2,ABHD16A,HPGD,ENSCAFG00000023820	up_VSAP_EC
GO:2001014	0.000467	1	2	8	regulation of skeletal muscle cell differentiation	BP	0.143	SIX1,CYP26B1	up_VFEM_EC
GO:0051239	0.000468	1	11	859	regulation of multicellular organismal process	BP	0.143	HOXC10,EYA1,HOXA9,TOX,MAP2K6,AVPR1A,HOXB7,HOXA7,SIX1,TIAM1,CYP26B1	up_VFEM_EC
GO:0072028	0.000471	1	3	37	nephron morphogenesis	BP	0.143	EYA1,HOXB7,SIX1	up_VFEM_EC

GO:0060272	0.000473	1	2	7	embryonic skeletal joint morphogenesis	BP	0.246	HOXA11,SHOX2	up_VSAP_VSMC
GO:0016042	0.000475	1	18	80	lipid catabolic process	BP	0.494	PEX7,LIPC,ENPP2,PLBD1,ACAA1,ACACB,PEX2,HSD17B14,PAFAH2,FAAH,LPL,PLA2G6,GCDH,EHHADH,LONP2,ABHD16A,LIPE,IDH1	up_VSAP_EC
GO:0048584	0.000477	1	71	592	positive regulation of response to stimulus	BP	0.0592	TEK,HCLS1,IL7R,GBP5,SELP,KIT,CXCL8,PRKCH,TNFAIP3,MAP3K11,TNIP1,PDGFB,TMEM173,RELN,PDE4D,MEF2C,FERMT1,ATF3,DLL4,TLR4,NLRC5,GPR4,EIF2B2,GATA3,EDN1,PELI1,PPP3CA,GUCY1A1,ARG2,CASP8,NECTIN2,MTOR,ACPP,TSPAN14,MAP3K3,BMP2,HHEX,CAV2,BMP2K,ITPKB,RIOK3,MADD,PAK2,PRKD2,SH3GLB1,SWAP70,SMARCA4,TLR3,MFNG,AKAP12,NCK1,TIMELESS,OTUD4,JAG1,PTGS2,RAPGEF2,PLCG2,BCL10,TAOK1,CTNNA1,HMGB1,PMAIP1,RIPK1,LSM14A,SKIL,PDGFD,HIF1A,ENSCAFG00000002015,RGL2,JCAD,CDON	up_VCAV_EC
GO:0032268	0.000477	1	69	778	regulation of cellular protein metabolic process	BP	0.101	ARHGEF2,PRICKLE1,VEGFA,MYH9,PLK3,HSPA5,SERPINE1,SOCS5,ENPP1,ERRFI1,JCAD,SNX25,BCAR3,NUAK1,PTPN11,AARS,TRIB3,PUM3,PM20D2,ILK,PPP1R12A,SESN2,PTGS2,MSN,IWS1,DNAJC10,TNIK,NXN,PTPN2,UCHL5,SPOPL,YES1,PLCL1,ADAM17,EIF4EBP1,CNOT7,MAGED1,CTNNB1,CRYAB,APOE,LYN,STRAP,FAM162A,CCNYL1,EIF5,MMD,CBLC,ATF3,GSK3B,VLDLR,HTT,REST,EIF2S1,CALM1,DISC1,RAD23A,CDYL,PAK2,A2M,INHBA,CDKN1A,TMTC3,NRP1,SPINK2,SH3RF3,USP14,MADD,KDM3A,MID1	down_VSAP_EC
GO:0033044	0.000477	1	23	127	regulation of chromosome organization	BP	0.0367	SREBF1,ATAD2,HNRNPD,ZW10,TRIP13,FBXO5,BIRC5,AURKB,NEK2,FEN1,UHRF1,BRCA1,NOC2L,CDC6,NDC80,ENSCAFG00000005299,BUB1,TTK,TADA2A,TINF2,UBE2C,CCT4,GEN1	down_ACOR_VSMC
GO:0090103	0.00048	1	2	8	cochlea morphogenesis	BP	0.143	EYA1,SIX1	up_VFEM_EC
GO:0048468	0.000481	1	70	553	cell development	BP	0.0592	EPHB1,EDNRB,TEK,ADGRF5,KIT,PRKCH,IFRD1,FLI1,PDGFB,PODXL,RELN,PDE4D,MEF2C,CHD7,DLL4,RAPGEF1,TLR4,GPR4,STC1,EIF2B2,GATA3,EDN1,RAB11A,TCF4,NTN4,PSME4,NEDD9,SOX18,TMOD1,PPP3CA,NECTIN2,MTOR,HOOK3,BMP2,CDYL,HMG20A,CAV2,PREX2,GDPD5,PROX1,ENSCAFG00000010224,ARID4B,STMN1,FLNB,PAK2,APOD,NCK1,FES,JAG1,MMD,RAPGEF2,CREB1,ZC4H2,CTNNA1,SPTBN4,AFF4,SKIL,HIF1A,KDM3A,ALS2,ATRX,ENSCAFG00000002015,P2RX2,TMOD3,ZEB1,ARHGEF2,FBXO5,CDON,TJP2,ZNF609	up_VCAV_EC
GO:2000145	0.000484	1	7	279	regulation of cell motility	BP	0.227	ACVRL1,LYN,IGF1R,PDPN,HOXA7,SULF1,STX4	down_ACOR_EC
GO:0033673	0.000492	1	4	84	negative regulation of kinase activity	BP	0.227	LYN,IGF1R,PRKAR2A,ASPN	down_ACOR_EC
GO:0003231	0.000499	1	13	48	cardiac ventricle development	BP	0.361	TNNT2,SMAD6,BMP4,FGFR2,CITED2,LMO4,EGLN1,SMARCD3,TBX20,TBX5,FRS2,CHD7,AP2B1	up_ACOR_VSMC
GO:0009611	0.000504	1	22	122	response to wounding	BP	0.0616	SELP,PRCP,F8,IFRD1,PDGFB,VWF,CD34,KLKB1,TLR4,GPR4,RAB27A,TFPI,MTOR,MMRN1,APOD,ADIPOR2,PDGFD,HIF1A,ITGB3,VASH1,PEAR1,ELK3	up_VCAV_EC
GO:0014065	0.000509	1	11	39	phosphatidylinositol 3-kinase signaling	BP	0.0618	TEK,HCLS1,SELP,PDGFB,RELN,GATA3,EDN1,PREX2,PDGFD,RGL2,PEAR1	up_VCAV_EC
GO:0009653	0.00051	1	33	773	anatomical structure morphogenesis	BP	0.632	TBX15,HOXA9,IRX3,IRX5,HOXC10,LEP,ANGPT4,HOXA7,COL8A2,DLL1,CRYAB,F3,PLXNA4,THRB,TMEM100,GREM1,CAMK2A,GREM2,HOXA6,LUZP1,RECK,TBXA2R,CNNM4,BVES,PTPRB,AXIN2,CDC42EP5,ADAM17,CLIC4,LRRN2,GCNT4,KANK1,TNC	up_AFEM_EC
GO:0048598	0.000511	1	35	207	embryonic morphogenesis	BP	0.494	TBX15,HOXA11,FZD1,HOXA7,HOXA9,CDON,WNT11,HNF4A,OSR2,INSIG2,GJB5,LRIG3,IRX5,HOXD3,ABL1,DLL1,HOXA6,OSR1,FUZ,ZBTB16,RARG,HOXC4,RECK,PLPP3,ZFP36L1,IRX3,ZEB1,FBXW4,SHOX2,HOXC10,LRP4,KDM2B,TGFBR2,CITED2,LATS2	up_VSAP_EC
GO:0003279	0.000512	1	13	48	cardiac septum development	BP	0.361	SMAD6,GATA4,BMP4,FGFR2,CITED2,LMO4,EGLN1,NRP1,TBX20,TBX5,FRS2,CHD7,AP2B1	up_ACOR_VSMC
GO:0048663	0.000517	1	2	13	neuron fate commitment	BP	0.241	HOXC10,EYA1	up_VFEM_VSMC

GO:0016054	0.00052	1	15	61	organic acid catabolic process	BP	0.494	PEX7,ACAA1,ACACB,PEX2,GOT1,ADHFE1,ALDH6A1,FAAH,GCDH,BCKDHB,EHHADH,LONP2,ABHD16A,ENSCAFG00000002977,IDO2	up_VSAP_EC
GO:0046395	0.00052	1	15	61	carboxylic acid catabolic process	BP	0.494	PEX7,ACAA1,ACACB,PEX2,GOT1,ADHFE1,ALDH6A1,FAAH,GCDH,BCKDHB,EHHADH,LONP2,ABHD16A,ENSCAFG00000002977,IDO2	up_VSAP_EC
GO:1905114	0.000522	1	4	150	cell surface receptor signaling pathway involved in cell-cell	BP	0.935	BARX1,NKX2-5,BAMBI,ANKRD6	up_VPOR_VSMC
GO:0045165	0.000522	1	3	65	cell fate commitment	BP	0.241	HOXC10,EYA1,HOXA11	up_VFEM_VSMC
GO:0051962	0.00053	1	21	112	positive regulation of nervous system development	BP	0.0638	EPHB1,PRKCH,RELN,MEF2C,RAPGEF1,RAB11A,TCF4,MTOR,BMP2,GDPD5,PROX1,NCK1,FES,MMD,RAPGEF2,ZC4H2,HIF1A,ZEB1,ARHGEF2,CDON,ZNF609	up_VCAV_EC
GO:0006520	0.000531	1	17	114	cellular amino acid metabolic process	BP	0.11	P4HB,ASNS,CARS,PLOD2,NR1H4,ERO1A,P4HA1,SARS,AARS,NMNAT2,ALDH18A1,YARS,TARS,NARS,PLOD3,RAB11FIP1,BCAT1	down_VSAP_EC
GO:0008015	0.000534	1	10	120	blood circulation	BP	0.632	IRX5,F5,DLL1,CHRM2,THRB,HTR1D,AVPR1A,TBXA2R,BVES,DMPK	up_AFEM_EC
GO:0030220	0.000534	1	4	7	platelet formation	BP	0.11	MYH9,ACTN1,PTPN11,WDR1	down_VSAP_EC
GO:0060537	0.000539	1	21	117	muscle tissue development	BP	0.0645	EPHB1,IFRD1,ADAMTS9,MEF2C,ATF3,CHD7,TIPARP,DLL4,EDN1,FOXN2,PPP3CA,MTOR,BMP2,CAV2,PROX1,ENSCAFG00000010596,FLNB,CREB1,NR1D2,P2RX2,CDON	up_VCAV_EC
GO:0030198	0.00054	1	12	81	extracellular matrix organization	BP	0.583	ADAMTS2,FBLN2,LUM,LOX,RECK,CRISPLD2,B4GALT1,ADAMTSL4,LOXL3,DDR2,GREM1,ECM2	down_VCAV_EC
GO:0051301	0.000545	1	21	117	cell division	BP	0.0417	NCAPD2,ANLN,CENPA,RAN,BIRC5,AURKB,DSN1,STMN1,PLK3,CKS2,NCAPH,E2F8,CDC6,NUP62,PDXP,FGF5,RAB35,ENSCAFG00000005299,ASPM,ARHGEF2,CCNB3	down_ACOR_VSMC
GO:0030036	0.000549	1	34	223	actin cytoskeleton organization	BP	0.0651	TEK,HCLS1,KIT,ARHGEF15,ENSCAFG00000007112,AMOT,BAIAP2L1,MEF2C,EDN1,ARHGAP28,TMOD1,MTSS1,MTOR,NCKAP1,PROX1,IQGAP2,EPB41,ELMO1,STMN1,FLNB,PAK2,SWAP70,NCK1,FES,CTNNA1,FMNL3,ARPC5L,ARHGDIB,ARHGAP26,GAS2L3,TNFAIP1,TMOD3,ARHGEF2,FSCN1	up_VCAV_EC
GO:0009952	0.000549	1	3	66	anterior/posterior pattern specification	BP	1	HOXC10,HOXA9,HOXC6	up_AORTRT_EC
GO:0042127	0.000554	1	56	452	regulation of cell proliferation	BP	0.0653	EPHB1,HCLS1,IL7R,KIT,CXCL8,PRKCH,CALCRL,TNFAIP3,PDGFB,BCL6B,MEF2C,FERMT1,DLL4,RAPGEF1,TLR4,GATA3,EDN1,APLNR,PTGS1,SOX18,EGFL7,PELI1,MTSS1,ODC1,ARG2,MAP3K3,BMP2,HHEX,CAV2,CNOT6,CCAR1,PROX1,MCC,PRKD2,APOD,NCK1,FES,JAG1,PTGS2,RAPGEF2,CTNNA1,CNOT7,HMGB1,CRIP2,NRAS,PDGFD,CUL4A,BMX,HIF1A,NR4A1,HP1BP3,VASH1,JCAD,ZEB1,CDON,ZNF609	up_VCAV_EC
GO:0043062	0.000554	1	21	99	extracellular structure organization	BP	0.373	FMOD,PRDX4,RECK,FERMT1,LCAT,MFAP4,ABCA1,SPINT1,CTSS,ERO1B,NPNT,SPINT2,P4HA1,DAG1,APP,ECM2,DDR2,NID1,SERPINB5,KIF9,TNFRSF11B	up_ACOR_VSMC
GO:0048513	0.000561	1	6	906	animal organ development	BP	0.586	HOXC10,TBX15,EYA1,HOXA9,BEND2,IRX5	up_AFEM_VSMC
GO:1901228	0.000563	1	1	4	positive regulation of transcription from RNA polymerase II promoter involved in heart	BP	0.757	GATA5	down_AFEM_VSMC
GO:0003274	0.000564	1	1	2	endocardial cushion fusion	BP	0.61	GATA5	down_VFEM_VSMC
GO:0032101	0.000566	1	27	173	regulation of response to external stimulus	BP	0.0662	GBP5,SELP,CXCL8,CXCR4,TNFAIP3,TNIP1,PDGFB,BCL6B,TMEM173,CD34,KLKB1,TLR4,GATA3,MMP28,TFPI,ARG2,RIOK3,ANGPT2,PRKD2,SWAP70,TLR3,APOD,PTGS2,HMGB1,NOS2,NR1D2,ENSCAFG00000025410	up_VCAV_EC
GO:0007062	0.000566	1	9	29	sister chromatid cohesion	BP	0.0428	SMC1A,ENSCAFG00000017264,SGO1,FEN1,KIF22,HASPIN,SMC3,CDC20,SGO2	down_ACOR_VSMC
GO:2000026	0.000569	1	5	560	regulation of multicellular organismal development	BP	0.69	SLITRK5,LIMK1,GATA5,LRRC17,C6	up_VCAV_VSMC
GO:0003013	0.00057	1	10	121	circulatory system process	BP	0.632	IRX5,F5,DLL1,CHRM2,THRB,HTR1D,AVPR1A,TBXA2R,BVES,DMPK	up_AFEM_EC
GO:0042439	0.000573	1	2	2	ethanolamine-containing compound metabolic	BP	0.632	MOXD1,NAPEPLD	up_AFEM_EC

GO:0001819	0.000576	1	21	120	positive regulation of cytokine production	BP	0.0669	GBP5,TMEM173,CD34,PDE4D,FERMT1,TLR4,GATA3,IRF8,PELI1,MAPK13,RIOK3,PRKD2,TLR3,CD200,PLCG2,BCL10,HMGB1,RIPK1,LSM14A,HIF1A,ARHGEF2	up_VCAV_EC
GO:0043436	0.000579	1	33	303	oxoacid metabolic process	BP	0.115	P4HB,ASNS,CARS,PLOD2,NR1H4,ENSCAFG00000009211,ERO1A,ENPP1,P4HA1,SARS,PGK1,AARS,TPI1,ENSCAFG00000032470,TRIB3,PGAM1,ALDH1L2,SESN2,PTGS2,CEMIP2,CHSY1,HIF1A,SLC45A3,NMNAT2,ALDH18A1,YARS,TARS,NARS,ALDOC,PAPSS1,PLOD3,RAB11FIP1,BCAT1	down_VSAP_EC
GO:0048701	0.000581	1	2	25	embryonic cranial skeleton morphogenesis	BP	0.586	TBX15,IRX5	up_AFEM_VSMC
GO:0090383	0.000587	1	2	2	phagosome acidification	BP	0.632	RAB20,RAB7A	up_AFEM_EC
GO:0140074	0.000588	1	1	2	cardiac endothelial to mesenchymal transition	BP	0.61	GATA5	down_VFEM_VSMC
GO:0002252	0.00059	1	27	166	immune effector process	BP	0.0676	IL7R,KIT,IL6,MX2,TMEM173,TLR4,NLRC5,GATA3,RAB27A,SERINC5,NECTIN2,MTOR,SNAP23,RIOK3,THOC1,SWAP70,TLR3,MFNG,OAS3,FES,JAG1,PLCG2,BCL10,CNOT7,HMGB1,PMAIP1,LSM14A	up_VCAV_EC
GO:0030097	0.000596	1	36	244	hemopoiesis	BP	0.0678	HOXB7,HCLS1,IL7R,ADGRF5,KIT,IL6,FLI1,MEF2C,CHD7,TIPARP,DLL4,GATA3,HOXB8,ENSCAFG00000003530,IRF8,CASP8,MTOR,TENT2,HHEX,ITPKB,MFNG,FES,JAG1,PLCG2,CREB1,HMGB1,RIPK1,CRIP2,ATP11C,CUL4A,BMX,HIF1A,TMOD3,ZEB1,TET2,TJP2	up_VCAV_EC
GO:0003176	0.000596	1	5	9	aortic valve development	BP	0.385	SMAD6,GATA5,GATA4,BMP4,TBX20	up_ACOR_VSMC
GO:0045454	0.000597	1	8	35	cell redox homeostasis	BP	0.117	P4HB,ENSCAFG00000013214,ERO1A,ENSCAFG00000003477,DNAJC10,NXN,PDIA4,DDIT3	down_VSAP_EC
GO:0001938	0.000603	1	9	34	positive regulation of endothelial cell	BP	0.0681	PDGFB,APLNR,EGFL7,BMP2,PROX1,PRKD2,HMGB1,NRAS,JCAD	up_VCAV_EC
GO:0050767	0.000612	1	30	184	regulation of neurogenesis	BP	0.0683	EDNRB,PRKCH,IFRD1,RELN,MEF2C,CHD7,DLL4,RAPGEF1,GATA3,RAB11A,TCF4,PPP3CA,MTOR,HOOK3,BMP2,HMG20A,GDPD5,PROX1,NCK1,FES,JAG1,MMD,RAPGEF2,ZC4H2,CTNNA1,HIF1A,ZEB1,ARHGEF2,CDON,ZNF609	up_VCAV_EC
GO:0060037	0.000613	1	2	9	pharyngeal system development	BP	0.177	EYA1,SIX1	up_VFEM_EC
GO:0030517	0.000615	1	2	5	negative regulation of axon extension	BP	1	SEMA3A,RNF6	up_VPOR_EC
GO:0009967	0.000616	1	55	435	positive regulation of signal transduction	BP	0.0683	TEK,HCLS1,IL7R,SELP,KIT,PRKCH,MAP3K11,PDGFB,RELN,FERMT1,ATF3,DLL4,TLR4,NLRC5,GPR4,GATA3,EDN1,PELI1,PPP3CA,GUCY1A1,CASP8,NECTIN2,ACPP,TSPAN14,MAP3K3,BMP2,HHEX,CAV2,BMP2K,ITPKB,MADD,PAK2,PRKD2,SH3GLB1,SMARCA4,TLR3,MFNG,AKAP12,NCK1,JAG1,RAPGEF2,BCL10,TAOK1,CTNNA1,HMGB1,PMAIP1,RIPK1,LSM14A,SKIL,PDGFD,HIF1A,ENSCAFG00000002015,RGL2,JCAD,CDON	up_VCAV_EC
GO:1901343	0.000619	1	11	42	negative regulation of vasculature development	BP	0.0683	PLK2,TEK,TIE1,ADAMTS9,AMOT,PDGFB,DLL4,GPR4,HHEX,ANGPT2,VASH1	up_VCAV_EC
GO:0072498	0.000621	1	2	8	embryonic skeletal joint development	BP	0.313	HOXA11,SHOX2	up_VSAP_VSMC
GO:0032970	0.000621651	0.99984	11	130	regulation of actin filament-based process	BP	0.7009284	TMOD1,ARHGAP6,SYNPO2,DIXDC1,ACTN2,ARHGAP28,PAK3,PTGER4,ARHGDIB,NRP1,PRKCD	up_ACOR_EC
GO:0007186	0.000622	1	21	124	G protein-coupled receptor signaling pathway	BP	0.0683	GNGT2,EDNRB,RGS16,CXCL8,CALCRL,GPR4,EDN1,APLNR,ACPP,SPNS3,CAV2,PREX2,GNG11,ABCA1,ARHGEF12,IQGAP2,STMN1,AKAP12,GRM8,RAPGEF2,RGS2	up_VCAV_EC
GO:0048856	0.000626	1	17	1533	anatomical structure development	BP	0.258	ACVRL1,LYN,HOXA3,ACSL4,SPG11,PDPN,NRP2,HIVEP2,HOXA7,ANPEP,SULF1,ELK3,HOXA1,CSGALNACT1,HOXC4,ASPN,NEDD4	down_ACOR_EC
GO:0051348	0.000628	1	4	90	negative regulation of transferase activity	BP	0.258	LYN,IGF1R,PRKAR2A,ASPN	down_ACOR_EC

GO:0042221	0.000631	1	85	1012	response to chemical	BP	0.121	ARHGEF2,VEGFA,P4HB,SULF1,NR1H4,PLK3,HSPA5,SERPINE1,SOCS5,HM13,SPARC,ERO1A,HSP90B1,ENPP1,JCAD,SNX25,HYOU1,PGK1,PTPN11,FLNB,DPYSL3,TRIB3,ILK,FHL2,PPP1R12A,PRCP,SMARCA4,SESN2,SRP54,TGFBR1,PTGS2,MYO1C,MSN,SEL1L,KIF5B,HIF1A,CX3CL1,DNAJC10,ZPR1,PTPN2,YES1,NEO1,MTDH,ADAM17,AGRN,CNOT7,DNAJB9,GNAQ,CTNNB1,SRP72,CRYAB,ENSCAFG00000002015,TFRC,APOE,KPNA4,ZC3H15,KLF5,UBA5,MMP28,LYN,BNIP3,STRAP,DDIT3,FAM162A,BACH1,SLC7A11,ATF3,CDC37,GBP5,REST,EIF2S1,EXT1,CALM1,PAK2,CREB3L2,INHBA,NR4A1,ENSCAFG00000018360,HIF1AN,BTK,NRP1,USP14,YWHAH,KDM3A,HOMER1	down_VSAP_EC
GO:0071345	0.000631	1	5	210	cellular response to cytokine stimulus	BP	1	NR1H4,MRC1,CCL24,FLRT3,ARHGEF2	up_VPUL_EC
GO:0003179	0.000635	1	2	19	heart valve morphogenesis	BP	0.599	TBX5,GATA5	up_VPUL_VSMC
GO:0009954	0.000636	1	2	16	proximal/distal pattern formation	BP	1	HOXC10,HOXA9	up_AORTRT_EC
GO:0060039	0.000637	1	2	11	pericardium development	BP	1	TBX5,FLRT3	up_VPUL_EC
GO:0002159	0.000639	1	1	2	desmosome assembly	BP	0.61	JUP	down_VFEM_VSMC
GO:0086042	0.000639	1	1	2	cardiac muscle cell-cardiac muscle cell	BP	0.61	JUP	down_VFEM_VSMC
GO:0086073	0.000639	1	1	2	bundle of His cell-Purkinje myocyte adhesion involved in cell	BP	0.61	JUP	down_VFEM_VSMC
GO:0006082	0.000643	1	33	305	organic acid metabolic process	BP	0.122	P4HB,ASNS,CARS,PLOD2,NR1H4,ENSCAFG00000009211,ERO1A,ENPP1,P4HA1,SARS,PGK1,AARS,TPI1,ENSCAFG00000032470,TRIB3,PGAM1,ALDH1L2,SESN2,PTGS2,CEMIP2,CHSY1,HIF1A,SLC45A3,NMNAT2,ALDH18A1,YARS,TARS,NARS,ALDOC,PAPSS1,PLOD3,RAB11FIP1,BCAT1	down_VSAP_EC
GO:1901016	0.000644	1	2	9	regulation of potassium ion transmembrane transporter activity	BP	0.315	NETO1,CAV1	up_VSAP_VSMC
GO:0000381	0.000649	1	7	19	regulation of alternative mRNA splicing, via	BP	0.0483	RNPS1,SRSF2,THRAP3,HNRNPL,RBM14,WTAP,SF3A1	down_ACOR_VSMC
GO:0071599	0.000655	1	2	9	otic vesicle development	BP	0.178	EYA1,SIX1	up_VFEM_EC
GO:0060993	0.000662	1	3	42	kidney morphogenesis	BP	0.178	EYA1,HOXB7,SIX1	up_VFEM_EC
GO:0030162	0.000665	1	24	202	regulation of proteolysis	BP	0.122	PRICKLE1,MYH9,PLK3,SERPINE1,SOCS5,PRKACB,TRIB3,PTGS2,UCHL5,SPOPL,CRYAB,APOE,LYN,FAM162A,GSK3B,REST,SPOP,DISC1,RAD23A,PAK2,A2M,TMTC3,SPINK2,USP14	down_VSAP_EC
GO:0030879	0.000665	1	2	34	mammary gland development	BP	0.434	HOXA9,ESR1	up_AORTRT_VSMC
GO:0006928	0.000666	1	53	557	movement of cell or subcellular component	BP	0.122	ARHGEF2,VEGFA,SULF1,MYH9,FMNL3,HSPA5,SERPINE1,ACTN1,SPARC,ITGA2,JCAD,PTPN11,FSCN1,DPYSL3,ILK,PRCP,WDR1,MYO1C,PIK3C2A,MSN,RPGR,KIF5B,HIF1A,CX3CL1,DYNLL1,TRAK1,NEO1,SDC3,ADAM17,PALLD,CTNNB1,KPNB1,ANLN,STARD13,APOE,PTP4A1,MMP28,LYN,STRAP,PDS5B,CCNYL1,NEXN,SDC4,HTT,ZMYND8,EXT1,DISC1,AP3S1,PAK2,SIX4,NRP1,ARF4,TNFAIP1	down_VSAP_EC
GO:0045184	0.000666	1	50	532	establishment of protein localization	BP	0.122	PRICKLE1,MYH9,NR1H4,PLK3,HM13,TRAM1,PTPN14,SEC24D,PTPN11,SEC23A,SRP54,MYO1C,ENSCAFG00000031588,SEL1L,HIF1A,SSR1,IWS1,ZPR1,STAM2,SMG7,XPOT,USO1,SRPRA,DDX39B,COPG1,KPNB1,COPB2,SRP72,ENSCAFG00000002015,TFRC,APOE,KPNA4,LYN,DDIT3,GSK3B,GBP5,SH3GLB1,KPNA1,REST,SCAMP1,RAB11FIP1,KDELR2,AP3S1,CLTC,STX6,CDKN1A,RHBDF2,YWHAH,BLZF1,SEC13	down_VSAP_EC
GO:0051270	0.000668	1	7	296	regulation of cellular component movement	BP	0.258	ACVRL1,LYN,IGF1R,PDPN,HOXA7,SULF1,STX4	down_ACOR_EC
GO:0048534	0.00068	1	6	262	hematopoietic or lymphoid organ	BP	0.178	HOXA9,TOX,HOXB7,HOXA7,SIX1,CYP26B1	up_VFEM_EC
GO:0003007	0.000688	1	20	94	heart morphogenesis	BP	0.427	TNNT2,SMAD6,GATA5,GATA4,HTR2B,BMP4,FGFR2,GJA1,SEC24B,CITED2,ANKRD1,EGLN1,NRP1,SMARCD3,TBX20,TBX5,PSEN1,TGFBR1,CHD7,GAA	up_ACOR_VSMC

GO:0001945	0.000688	1	2	9	lymph vessel development	BP	0.258	ACVRL1,PDPN	down_ACOR_EC
GO:0001946	0.000688	1	2	9	lymphangiogenesis	BP	0.258	ACVRL1,PDPN	down_ACOR_EC
GO:0036303	0.000688	1	2	9	lymph vessel morphogenesis	BP	0.258	ACVRL1,PDPN	down_ACOR_EC
GO:0010389	0.00069	1	9	30	regulation of G2/M transition of mitotic cell	BP	0.0504	CDK1,FANCI,TOPBP1,CDK4,RNASEH2B,DTL,CDK2,CLSPN,CCNB3	down_ACOR_VSMC
GO:0010647	0.000691	1	57	460	positive regulation of cell communication	BP	0.0754	TEK,HCLS1,IL7R,SELP,KIT,PRKCH,MAP3K11,PDGFB,RELN,FERMT1,ATF3,DLL4,TLR4,NLRC5,GPR4,GATA3,EDN1,PELI1,PPP3CA,GUCY1A1,CASP8,NECTIN2,ACPP,TSPAN14,MAP3K3,BMP2,HHEX,CAV2,BMP2K,ITPKB,MADD,PAK2,PRKD2,SH3GLB1,SMARCA4,TLR3,MFNG,AKAP12,NCK1,JAG1,RAPGEF2,CREB1,BCL10,TAOK1,CTNNA1,HMGB1,PMAIP1,RIPK1,LSM14A,SKIL,MCU,PDGFD,HIF1A,ENSCAFG00000002015,RGL2,JCAD,CDON	up_VCAV_EC
GO:0001657	0.000694	1	3	42	ureteric bud development	BP	0.178	EYA1,HOXB7,SIX1	up_VFEM_EC
GO:0072163	0.000694	1	3	42	mesonephric epithelium development	BP	0.178	EYA1,HOXB7,SIX1	up_VFEM_EC
GO:0072164	0.000694	1	3	42	mesonephric tubule development	BP	0.178	EYA1,HOXB7,SIX1	up_VFEM_EC
GO:0006272	0.000698	1	3	3	leading strand elongation	BP	0.0508	POLA1,POLE,PCNA	down_ACOR_VSMC
GO:0003156	0.000698	1	2	20	regulation of animal organ formation	BP	0.599	TBX5,GATA5	up_VPUL_VSMC
GO:0010830	0.000703	1	2	16	regulation of myotube differentiation	BP	1	NKX2-5,CYP26B1	up_VPOR_VSMC
GO:0002009	0.000706	1	5	177	morphogenesis of an epithelium	BP	0.178	EYA1,HOXB7,SIX1,MTSS1,CA9	up_VFEM_EC
GO:0098609	0.000708	1	2	181	cell-cell adhesion	BP	0.635	JUP,GATA5	down_VFEM_VSMC
GO:0010614	0.000718	1	1	5	negative regulation of cardiac muscle	BP	0.827	GATA5	down_AFEM_VSMC
GO:0014741	0.000718	1	1	5	negative regulation of muscle hypertrophy	BP	0.827	GATA5	down_AFEM_VSMC
GO:0007275	0.000724	1	7	1370	multicellular organism development	BP	0.69	HOXC6,SLITRK5,LIMK1,GATA5,LRRC17,C6,TGFBI	up_VCAV_VSMC
GO:0065008	0.000726	1	41	1056	regulation of biological quality	BP	0.733	CMKLR1,IRX5,HOXC10,LEP,KCNC4,PRR16,CRY2,RAB20,F5,DLL1,CRYAB,F3,PLXNA4,GPAM,MLLT11,CHRM2,THRB,HTR1D,NFE2L1,AVPR1A,TENT5C,CAMK2A,CYLD,FXYD1,TBXA2R,MOXD1,CNNM4,TFPI2,BVES,RAB7A,AXIN2,ATP6V0B,ACACB,NAPEPLD,PPARGC1A,CCL5,CDC42EP5,DMPK,SAMD4B,GCNT4,KANK1	up_AFEM_EC
GO:0048706	0.000726	1	2	59	embryonic skeletal system development	BP	1	HOXA3,HOXB5	up_APUL_VSMC
GO:0007156	0.000733	1	9	26	homophilic cell adhesion via plasma membrane adhesion molecules	BP	0.0794	PCDH12,PCDH1,DSC2,CDH5,ESAM,NECTIN2,DSG2,CDH24,CD200	up_VCAV_EC
GO:0044282	0.000744	1	19	89	small molecule catabolic process	BP	0.667	PEX7,DHDH,ACAA1,ACACB,PEX2,GOT1,INPP5E,ADHFE1,ALDH6A1,FAAH,GALK1,GCDH,BCKDHB,EHHADH,LONP2,ABHD16A,ENSCAFG00000002977,PNP,IDO2	up_VSAP_EC
GO:0048856	0.000744	1	15	1533	anatomical structure development	BP	0.352	HOXA11,TBX15,HOXA9,PITX1,EMX2,GATM,SALL1,HOXC10,IRX5,MAB21L1,STIM1,CAV1,SHOX2,HAPLN1,GJB5	up_VSAP_VSMC
GO:0070887	0.000751	1	68	775	cellular response to chemical stimulus	BP	0.133	ARHGEF2,VEGFA,P4HB,SULF1,NR1H4,PLK3,HSPA5,SERPINE1,SOCS5,SPARC,ERO1A,ENPP1,JCAD,SNX25,HYOU1,PGK1,PTPN11,FLNB,DPYSL3,TRIB3,ILK,PPP1R12A,PRCP,SMARCA4,SESN2,TGFBR1,PTGS2,MYO1C,MSN,KIF5B,HIF1A,CX3CL1,ZPR1,PTPN2,YES1,NEO1,MTDH,ADAM17,AGRN,CNOT7,GNAQ,CTNNB1,ENSCAFG00000002015,TFRC,ZC3H15,KLF5,UBA5,MMP28,LYN,BNIP3,STRAP,DDIT3,FAM162A,BACH1,ATF3,CDC37,GBP5,REST,EIF2S1,PAK2,CREB3L2,NR4A1,ENSCAFG00000018360,HIF1AN,BTK,NRP1,YWHAH,KDM3A	down_VSAP_EC
GO:0120163	0.000754	1	2	17	negative regulation of cold-induced	BP	1	HOXC10,LNPEP	up_AORTRT_EC

GO:0023056	0.000758	1	57	462	positive regulation of signaling	BP	0.0814	TEK,HCLS1,IL7R,SELP,KIT,PRKCH,MAP3K11,PDGFB,RELN,FERMT1,ATF3,DLL4,TLR4,NLRC5,GPR4,GATA3,EDN1,PELI1,PPP3CA,GUCY1A1,CASP8,NECTIN2,ACPP,TSPAN14,MAP3K3,BMP2,HHEX,CAV2,BMP2K,ITPKB,MADD,PAK2,PRKD2,SH3GLB1,SMARCA4,TLR3,MFNG,AKAP12,NCK1,JAG1,RAPGEF2,CREB1,BCL10,TAOK1,CTNNA1,HMGB1,PMAIP1,RIPK1,LSM14A,SKIL,MCU,PDGFD,HIF1A,ENSCAFG00000002015,RGL2,JCAD,CDON	up_VCAV_EC
GO:0032501	0.00076	1	15	1782	multicellular organismal process	BP	1	NR1H4,TBX5,HOXA2,MAPK10,BPI,HTR7,HOXA3,PLCL1,PCSK1N,ZDHHC16,SLITRK5,NPNT,NTRK2,HOXA1,PDLIM3	up_APUL_EC
GO:0006334	0.00076	1	11	45	nucleosome assembly	BP	0.0545	CENPA,OIP5,IPO4,HIST1H1A,HMGB2,HIST1H2BB,CENPP,GRWD1,HAT1,MIS18A,H3F3B	down_ACOR_VSMC
GO:0014706	0.000761	1	20	112	striated muscle tissue development	BP	0.0814	EPHB1,IFRD1,ADAMTS9,MEF2C,ATF3,CHD7,DLL4,EDN1,FOXN2,PPP3CA,MTOR,BMP2,CAV2,PROX1,ENSCAFG00000010596,FLNB,CREB1,NR1D2,P2RX2,CDON	up_VCAV_EC
GO:0003218	0.000765	1	1	1	cardiac left ventricle formation	BP	1	TBX5	up_APUL_VSMC
GO:0040012	0.000772	1	7	303	regulation of locomotion	BP	0.276	ACVRL1,LYN,IGF1R,PDPN,HOXA7,SULF1,STX4	down_ACOR_EC
GO:0048705	0.00078	1	4	95	skeletal system morphogenesis	BP	0.352	HOXA11,TBX15,IRX5,SHOX2	up_VSAP_VSMC
GO:0007275	0.000786	1	14	1370	multicellular organism development	BP	0.352	HOXA11,TBX15,HOXA9,PITX1,EMX2,GATM,SALL1,HOXC10,IRX5,MAB21L1,CAV1,SHOX2,HAPLN1,GJB5	up_VSAP_VSMC
GO:0003158	0.000791	1	10	38	endothelium development	BP	0.084	PDE4D,DLL4,RAPGEF1,STC1,SOX18,PROX1,HOXB5,JAG1,RAPGEF2,ZEB1	up_VCAV_EC
GO:0090102	0.000793	1	2	10	cochlea development	BP	0.194	EYA1,SIX1	up_VFEM_EC
GO:0008284	0.000795	1	6	238	positive regulation of cell proliferation	BP	0.276	ACVRL1,LYN,HOXA3,IGF1R,SULF1,STX4	down_ACOR_EC
GO:0071840	0.000797	0.999	207	2059	cellular component organization or biogenesis	BP	0.0569	ACSL3,FSCN1,HMGB1,MCM6,SREBF1,MCM4,FLNB,POLE3,IRAK1,MCM7,RPA1,NCAPD2,ANLN,STXBP6,USP4,FMNL3,ATAD2,HMGA1,HMGCR,EDC3,HNRNPD,SMC1A,UBE2S,NEDD4,RRM1,ZW10,ENSCAFG00000016482,CAPG,SMC4,CENPA,SLC29A1,ENSCAFG00000017264,NDC1,CENPN,TOP3A,TRIP13,COLGALT1,SLC25A5,TIMM44,SF1,SGO1,GINS4,C1QTNF6,NOP56,SDC1,ARFGAP1,CTDNEP1,FBXO5,HSPA9,RAN,B3GNT2,CFAP298,PRELID1,PSMD13,BIRC5,INSIG1,PRMT1,CAV1,CDK1,RAD51,LIMA1,NF2,AURKB,MMP14,TK1,CDKN2C,DSN1,EZH2,STMN1,CENPO,HAUS8,GREM1,NCAPD3,MAP3K20,NEK2,CHCHD3,FBL,FEN1,SMARCC1,MND1,GAS2L3,PLK3,HMGB3,RCC1,DHX33,KIF22,UHRF1,CKS2,DIS3,GMNN,CDCA8,CENPH,DHCR24,NCAPH,POC1A,BRCA1,NOC4L,WDR62,CENPI,FASTKD3,NOC2L,EIF5A,B4GALT1,SMC2,FARSA,PREB,ESPL1,SPC25,CDC6,NDC80,OIP5,IPO4,HIST1H1A,RAC1,NUP62,EMP2,DDX28,PDXP,FOSL1,PLK4,IQGAP1,SUV39H1,HASPIN,RSL1D1,TOMM34,IQGAP2,SAMM50,RRP1,REEP4,HMGB2,DMAP1,CDC42EP4,HIST1H2BB,SMC3,CDK2,SMARCB1,DDX39B,CCNA2,RAB35,STAG1,ENSCAFG00000005299,EZR,SMC6,SF3A2,SASS6,BUB1,RBM14,CORO1A,NOLC1,CE	down_ACOR_VSMC
GO:0002886	0.000803	1	2	10	regulation of myeloid leukocyte mediated	BP	0.276	LYN,STX4	down_ACOR_EC
GO:0040011	0.000805	1	9	505	locomotion	BP	0.276	ACVRL1,LYN,IGF1R,PDPN,NRP2,HOXA7,SULF1,STX4,NEDD4	down_ACOR_EC
GO:0009653	0.000806	0.999	90	773	anatomical structure morphogenesis	BP	0.085	EPHB1,PLK2,HOXB7,TEK,IL7R,ADGRF5,KIT,PRCP,CXCL8,ARHGEF15,IFRD1,EMCN,CLEC14A,CALCRL,FLI1,TIE1,TNFAIP3,ADAMTS9,AMOT,PODXL,RELN,CD34,MEF2C,CHD7,TIPARP,DLL4,GPR4,STC1,GATA3,EDN1,HOXB8,RAB11A,APLNR,ARAP3,SMAD1,NTN4,NEDD9,SOX18,PIK3C2A,TMOD1,PPP3CA,PTPRB,MTSS1,EPHB4,NECTIN2,MTOR,MAP3K3,BMP2,HHEX,PREX2,PDZD8,ANGPT2,PROX1,ENSCAFG00000010224,MDFI,STMN1,FLNB,SNX2,TSPAN12,PRKD2,TLR3,BTBD7,EXOC5,TIMELESS,HOXB5,FES,JAG1,RAPGEF2,CREB1,BCL10,FMNL3,ADIPOR2,SPTBN4,ITGA6,SKIL,HIF1A,ALS2,ATRX,ALDH1A3,ENSCAFG00000002015,LUZP1,VASH1,JCAD,TMOD3,ZEB1,ARHGEF2,FSCN1,CDON,ELK3,ZMYM2	up_VCAV_EC

GO:0034123	0.00081	1	2	10	positive regulation of toll-like receptor signaling	BP	0.353	TLR2,CAV1	up_VSAP_VSMC
GO:0009887	0.000813	1	3	314	animal organ morphogenesis	BP	1	HOXA3,TBX5,HOXB5	up_APUL_VSMC
GO:0032715	0.000814	1	2	11	negative regulation of interleukin-6 production	BP	1	NR1H4,BPI	up_APUL_EC
GO:0050931	0.00082	1	5	10	pigment cell differentiation	BP	0.0858	EDNRB,KIT,ADAMTS9,MEF2C,RAB27A	up_VCAV_EC
GO:0030036	0.000823341	0.99973	15	223	actin cytoskeleton organization	BP	0.7009284	MYOZ2,TMOD1,ARHGAP6,AQP1,SYNPO2,DIXDC1,PDLIM3,PLS1,ACTN2,ARHGAP28,PAK3,PTGER4,ARHGDIB,NRP1,PRKCD	up_ACOR_EC
GO:2001224	0.000824	1	4	5	positive regulation of neuron migration	BP	0.0858	RELN,RAPGEF2,ARHGEF2,ZNF609	up_VCAV_EC
GO:0048856	0.000826	1	9	1533	anatomical structure development	BP	0.599	HOXA3,TBX5,GATA5,HOXA2,TMEM176A,TMEM176B,PHEX,HOXB5,SLIT2	up_VPUL_VSMC
GO:0007389	0.000829	1	3	137	pattern specification process	BP	0.777	HOXC10,EYA1,HOXA9	up_AFEM_VSMC
GO:0007338	0.000837	1	2	16	single fertilization	BP	0.375	HOXA9,HOXA11	up_VFEM_VSMC
GO:0048646	0.000838	1	47	349	anatomical structure formation involved in morphogenesis	BP	0.0858	EPHB1,PLK2,TEK,PRCP,CXCL8,EMCN,CLEC14A,CALCRL,TIE1,TNFAIP3,ADAMTS9,AMOT,PODXL,RELN,CD34,MEF2C,DLL4,GPR4,GATA3,EDN1,APLNR,SMAD1,SOX18,PIK3C2A,TMOD1,PTPRB,EPHB4,NECTIN2,MAP3K3,HHEX,ANGPT2,PROX1,TSPAN12,PRKD2,TLR3,BCL10,ADIPOR2,ITGA6,SKIL,HIF1A,ALDH1A3,LUZP1,VASH1,JCAD,TMOD3,CDON,ELK3	up_VCAV_EC
GO:0003129	0.000839	1	1	3	heart induction	BP	0.702	GATA5	down_VFEM_VSMC
GO:0001816	0.00084	1	29	194	cytokine production	BP	0.0858	ACKR1,GBP5,KIT,TNFAIP3,TMEM173,CD34,PDE4D,FERMT1,TLR4,GATA3,HOMER3,IRF8,PELI1,ARG2,ABCA1,MAPK13,RIOK3,PRKD2,TLR3,APOD,CD200,PLCG2,BCL10,HMGB1,NOS2,RIPK1,LSM14A,HIF1A,ARHGEF2	up_VCAV_EC
GO:0048731	0.00084	1	13	1247	system development	BP	0.198	HOXC10,TBX15,EYA1,HOXA9,TOX,MME,HOXB7,HOXA7,SIX1,MAB21L1,TIAM1,CYP26B1,MTSS1	up_VFEM_EC
GO:0035480	0.00084	1	1	1	regulation of Notch signaling pathway involved in heart	BP	1	GATA5	down_VSAP_VSMC
GO:0035481	0.00084	1	1	1	positive regulation of Notch signaling pathway involved in heart	BP	1	GATA5	down_VSAP_VSMC
GO:0003137	0.00084	1	1	1	Notch signaling pathway involved in heart	BP	1	GATA5	down_VSAP_VSMC
GO:0061999	0.00084	1	1	1	regulation of cardiac endothelial to	BP	1	GATA5	down_VSAP_VSMC
GO:0062000	0.00084	1	1	1	positive regulation of cardiac endothelial to mesenchymal transition	BP	1	GATA5	down_VSAP_VSMC
GO:0090381	0.00084	1	1	1	regulation of heart induction	BP	1	GATA5	down_VSAP_VSMC
GO:1901321	0.00084	1	1	1	positive regulation of heart induction	BP	1	GATA5	down_VSAP_VSMC
GO:0090190	0.000845	1	2	10	positive regulation of branching involved in ureteric bud	BP	0.198	HOXB7,SIX1	up_VFEM_EC
GO:0008283	0.000856	1	9	545	cell proliferation	BP	0.285	ACVRL1,LYN,HOXA3,IGF1R,PDPN,USP28,SULF1,WDR12,STX4	down_ACOR_EC
GO:0045446	0.00086	1	9	32	endothelial cell differentiation	BP	0.0873	PDE4D,RAPGEF1,STC1,SOX18,PROX1,HOXB5,JAG1,RAPGEF2,ZEB1	up_VCAV_EC
GO:0001763	0.00086	1	3	78	morphogenesis of a branching structure	BP	0.375	EYA1,HOXA11,SHOX2	up_VFEM_VSMC
GO:0001823	0.000865	1	3	45	mesonephros development	BP	0.199	EYA1,HOXB7,SIX1	up_VFEM_EC
GO:0010543	0.000865	1	2	10	regulation of platelet activation	BP	0.285	LYN,PDPN	down_ACOR_EC
GO:1904888	0.000866	1	2	31	cranial skeletal system development	BP	0.777	TBX15,IRX5	up_AFEM_VSMC
GO:0061343	0.00087	1	1	3	cell adhesion involved in heart morphogenesis	BP	0.702	GATA5	down_VFEM_VSMC
GO:0003128	0.000876	1	1	6	heart field specification	BP	0.942	GATA5	down_AFEM_VSMC
GO:0060297	0.000886	1	3	3	regulation of sarcomere organization	BP	0.0891	MEF2C,EDN1,PROX1	up_VCAV_EC
GO:0019932	0.000889	1	16	82	second-messenger-mediated signaling	BP	0.0891	EDNRB,SELP,CXCR4,CALCRL,PDE4D,EDN1,APLNR,HOMER3,PPP3CA,GUCY1A1,MTOR,ABCA1,GUCY1B1,RAPGEF2,NOS2,MCU	up_VCAV_EC
GO:0071470	0.000893	1	2	13	cellular response to osmotic stress	BP	1	PTGER2,ARHGEF2	up_VPUL_EC

GO:0002520	0.0009	1	39	279	immune system development	BP	0.0896	HOXB7,HCLS1,IL7R,ADGRF5,KIT,IL6,FLI1,TNFAIP3,MEF2C,CHD7,TIPARP,DLL4,GATA3,HOXB8,ENSCAFG00000003530,IRF8,CASP8,MTOR,TENT2,HHEX,ITPKB,THOC1,SWAP70,MFNG,FES,JAG1,PLCG2,CREB1,HMGB1,RIPK1,CRIP2,ATP11C,CUL4A,BMX,HIF1A,TMOD3,ZEB1,TET2,TJP2	up_VCAV_EC
GO:0060411	0.000901	1	10	33	cardiac septum morphogenesis	BP	0.485	SMAD6,GATA4,BMP4,FGFR2,CITED2,EGLN1,NRP1,TBX20,TBX5,CHD7	up_ACOR_VSMC
GO:0009792	0.000903	1	6	231	embryo development ending in birth or egg	BP	0.289	ACVRL1,HOXA3,ACSL4,HOXA7,SULF1,HOXA1	down_ACOR_EC
GO:0009952	0.000905	1	2	66	anterior/posterior pattern specification	BP	1	HOXA3,HOXB5	up_APUL_VSMC
GO:0060914	0.000914	1	2	11	heart formation	BP	0.208	EYA1,SIX1	up_VFEM_EC
GO:0036344	0.000916	1	4	8	platelet morphogenesis	BP	0.154	MYH9,ACTN1,PTPN11,WDR1	down_VSAP_EC
GO:0071356	0.000918	1	3	57	cellular response to tumor necrosis factor	BP	1	NR1H4,CCL24,ARHGEF2	up_VPUL_EC
GO:0010596	0.000919	1	7	21	negative regulation of endothelial cell migration	BP	0.0902	ADAMTS9,MEF2C,DLL4,STC1,ANGPT2,HMGB1,VASH1	up_VCAV_EC
GO:0061448	0.000919	1	4	89	connective tissue development	BP	0.289	HOXA3,SULF1,CSGALNACT1,HOXC4	down_ACOR_EC
GO:0002371	0.000922	1	3	3	dendritic cell cytokine production	BP	0.0902	KIT,TLR4,TLR3	up_VCAV_EC
GO:0050851	0.000927	1	9	34	antigen receptor-mediated signaling	BP	0.0902	PRKCH,PDE4D,MEF2C,EIF2B2,GATA3,NECTIN2,PRKD2,PLCG2,BCL10	up_VCAV_EC
GO:0002520	0.000935	1	6	279	immune system development	BP	0.21	HOXA9,TOX,HOXB7,HOXA7,SIX1,CYP26B1	up_VFEM_EC
GO:0003006	0.000948	1	3	180	developmental process involved in reproduction	BP	0.434	HOXA9,WNT7A,ESR1	up_AORTRT_VSMC
GO:0043408	0.000953	1	35	211	regulation of MAPK cascade	BP	0.485	ADRA1B,LEP,MID1,HTR2B,RASSF2,BMP4,FGFR2,TLR3,SH3RF2,PDE5A,SASH1,FGF18,NDRG2,F2RL1,NRP1,PTPN22,NPNT,SMPD1,SH3RF1,PRKCE,FRS2,DAG1,APP,PSEN1,SYNJ2BP,CCL5,DUSP22,EDN1,DUSP26,NOD2,SH3RF3,MAP3K7,INPP5K,ENSCAFG00000029553,CRKL	up_ACOR_VSMC
GO:0000910	0.000954	1	12	52	cytokinesis	BP	0.0663	ANLN,CENPA,BIRC5,AURKB,STMN1,PLK3,E2F8,CDC6,NUP62,PDXP,RAB35,ENSCAFG00000005299	down_ACOR_VSMC
GO:0035850	0.000961	1	8	25	epithelial cell differentiation involved in	BP	0.0926	PDGFB,PODXL,MEF2C,GPR4,GATA3,MTSS1,JAG1,BASP1	up_VCAV_EC
GO:0009790	0.000962	1	3	335	embryo development	BP	1	HOXA3,TBX5,HOXB5	up_APUL_VSMC
GO:0048518	0.000964	0.999	169	1676	positive regulation of biological process	BP	0.0926	EPHB1,PLK2,HOXB7,TEK,HCLS1,IL7R,GBP5,ADGRF5,SELP,KIT,SELE,PPP2R5A,CXCL8,PRKCH,ARHGEF15,DOCK4,CALCRL,TNFAIP3,ADAMTS9,PPARG,MAP3K11,ATP8A1,TNIP1,AMOT,PDGFB,PODXL,STK10,BAIAP2L1,TMEM173,RELN,CD34,PDE4D,MEF2C,FERMT1,ATF3,CHD7,KLKB1,TIPARP,DLL4,RAPGEF1,TLR4,NLRC5,GPR4,STC1,EIF2B2,GATA3,EDN1,RAB11A,RALB,APLNR,RAB27A,TCF4,SMAD1,FOXN2,TNFAIP8,ENSCAFG00000017103,NEDD9,IRF8,PIK3C2A,EGFL7,PELI1,PPP3CA,YES1,GUCY1A1,ODC1,SPTLC2,ARG2,CASP8,NECTIN2,MTOR,ACPP,TSPAN14,MAP3K3,BMP2,HHEX,CREM,CAV2,SLK,BMP2K,CNOT6,CCAR1,ABCA1,MAPK13,ITPKB,NCKAP1,GDPD5,RIOK3,PROX1,UHRF2,IQGAP2,MADD,EPB41,ARID4B,STMN1,HMBOX1,PAK2,PRKD2,THOC1,SH3GLB1,SWAP70,SMARCA4,TLR3,MFNG,AKAP12,NCK1,TIMELESS,OTUD4,LDB2,CD200,FES,JAG1,NSD1,HIST1H1A,PTGS2,MMD,RAPGEF2,PLCG2,CTDSPL2,CREB1,BCL10,PAN3,ZC4H2,TAOK1,CTNNA1,CNOT7,GCH1,ADIPOR2,HMGB1,AHR,NOS2,PMAIP1,ARPC5L,BLM,SPTBN4,RIPK1,CRIP2,NRAS,SMC5,LSM14A,ITGA6,RAD21,ATP11C,SKIL,GPBP1,MCU,PDGFD,MTA2,CUL4A,NR1D2,UBE2D1,KDM5A,HIF1A,KDM3A,ATRX,NR4A1,ALDH	up_VCAV_EC

GO:0022607	0.000969	0.999	107	969	cellular component assembly	BP	0.0671	ACSL3,FSCN1,HMGB1,IRAK1,ANLN,STXBP6,USP4,HMGA1,HMGCR,EDC3,SMC1A,UBE2S,RRM1,ZW10,CAPG,CENPA,SLC29A1,NDC1,CENPN,TRIP13,C1QTNF6,SDC1,FBXO5,HSPA9,CFAP298,PSMD13,BIRC5,INSIG1,PRMT1,CAV1,RAD51,LIMA1,NF2,AURKB,TK1,STMN1,CENPO,HAUS8,GREM1,NEK2,RCC1,DHX33,GMNN,CENPH,POC1A,WDR62,CENPI,FASTKD3,NOC2L,B4GALT1,FARSA,PREB,OIP5,IPO4,HIST1H1A,RAC1,NUP62,EMP2,DDX28,PDXP,FOSL1,PLK4,IQGAP2,SAMM50,HMGB2,CDC42EP4,HIST1H2BB,SMC3,CDK2,DDX39B,STAG1,ENSCAFG00000005299,EZR,SF3A2,SASS6,RBM14,CORO1A,CENPW,FOXRED1,CDC20,SIGMAR1,ASPM,ARPC1B,CENPP,GRWD1,ENSCAFG00000010224,PRMT5,PSMC1,CTH,TRAPPC6A,ENSCAFG00000015189,KIF11,HAT1,TINF2,GEMIN7,UBE2C,MIS18A,TBCC,SIX4,H3F3B,CEP83,PDSS1,TTLL1,TPBG,SF3A1,EIF3M,KIF24	down_ACOR_VSMC
GO:0032879	0.000969	1	32	767	regulation of localization	BP	0.919	CMKLR1,LEP,KCNC4,CRY2,RAB20,GAS1,ANGPT4,HOXA7,DLL1,CRYAB,KCND1,PLXNA4,GPAM,CHRM2,TWIST2,GREM1,CAMK2A,CYLD,FXYD1,LRRC15,RECK,TBXA2R,REEP2,BVES,LGR6,RAB7A,ACACB,CCL5,DMPK,ADAM17,CLIC4,KANK1	up_AFEM_EC
GO:0003006	0.000971	1	5	180	developmental process involved in reproduction	BP	0.387	HOXA11,HOXA9,SALL1,IRX5,GJB5	up_VSAP_VSMC
GO:0008584	0.000972	1	2	39	male gonad development	BP	0.434	HOXA9,ESR1	up_AORTRT_VSMC
GO:0046546	0.000972	1	2	39	development of primary male sexual	BP	0.434	HOXA9,ESR1	up_AORTRT_VSMC
GO:0003180	0.000975	1	1	7	aortic valve morphogenesis	BP	0.947	GATA5	down_AFEM_VSMC
GO:0046649	0.000986	1	26	162	lymphocyte activation	BP	0.0934	IL7R,KIT,IL6,TNFAIP3,MEF2C,CHD7,DLL4,TLR4,GATA3,RAB27A,PELI1,ARG2,MTOR,ELF4,HHEX,ITPKB,THOC1,SWAP70,MFNG,NCK1,PLCG2,BCL10,HMGB1,ATP11C,BMX,ZEB1	up_VCAV_EC
GO:0002064	0.00099	1	15	71	epithelial cell development	BP	0.0934	PDGFB,PODXL,PDE4D,RAPGEF1,GPR4,STC1,SOX18,TMOD1,PROX1,ARID4B,FLNB,JAG1,RAPGEF2,HIF1A,ATRX	up_VCAV_EC
GO:0001501	0.00099	1	3	178	skeletal system development	BP	0.434	HOXC10,HOXA9,WNT7A	up_AORTRT_VSMC
GO:0009653	0.000993	1	10	773	anatomical structure morphogenesis	BP	0.387	HOXA11,TBX15,HOXA9,PITX1,SALL1,HOXC10,IRX5,CAV1,SHOX2,GJB5	up_VSAP_VSMC
GO:0070527	0.000998	1	2	10	platelet aggregation	BP	0.302	LYN,PDPN	down_ACOR_EC
GO:0021983	0.000998	1	2	11	pituitary gland development	BP	0.387	PITX1,SALL1	up_VSAP_VSMC
GO:1901213	0.000998	1	1	7	regulation of transcription from RNA polymerase II promoter involved in	BP	0.947	GATA5	down_AFEM_VSMC
GO:0060351	0.001	1	2	11	cartilage development involved in endochondral bone morphogenesis	BP	0.387	HOXA11,SHOX2	up_VSAP_VSMC

GO:0048522	0.00101	0.999	152	1484	positive regulation of cellular process	BP	0.0945	EPHB1,PLK2,TEK,HCLS1,IL7R,GBP5,ADGRF5,SELP,KIT,SELE,PPP2R5A,CXCL8,PRKCH,ARHGEF15,DOCK4,CALCRL,TNFAIP3,ADAMTS9,PPARG,MAP3K11,ATP8A1,TNIP1,PDGFB,PODXL,STK10,BAIAP2L1,TMEM173,RELN,CD34,MEF2C,FERMT1,ATF3,CHD7,DLL4,RAPGEF1,TLR4,NLRC5,GPR4,GATA3,EDN1,RAB11A,RALB,APLNR,RAB27A,TCF4,FOXN2,TNFAIP8,ENSCAFG00000017103,NEDD9,IRF8,PIK3C2A,EGFL7,PELI1,PPP3CA,YES1,GUCY1A1,ODC1,SPTLC2,ARG2,CASP8,NECTIN2,MTOR,ACPP,TSPAN14,MAP3K3,BMP2,HHEX,CREM,CAV2,SLK,BMP2K,CNOT6,CCAR1,ITPKB,NCKAP1,GDPD5,PROX1,UHRF2,IQGAP2,MADD,ARID4B,STMN1,HMBOX1,PAK2,PRKD2,THOC1,SH3GLB1,SWAP70,SMARCA4,TLR3,MFNG,AKAP12,NCK1,TIMELESS,OTUD4,LDB2,CD200,FES,JAG1,NSD1,HIST1H1A,PTGS2,MMD,RAPGEF2,PLCG2,CREB1,BCL10,PAN3,ZC4H2,TAOK1,CTNNA1,CNOT7,HMGB1,AHR,NOS2,PMAIP1,ARPC5L,BLM,RIPK1,CRIP2,NRAS,SMC5,LSM14A,ITGA6,RAD21,ATP11C,SKIL,GPBP1,MCU,PDGFD,MTA2,CUL4A,NR1D2,UBE2D1,KDM5A,HIF1A,KDM3A,ATRX,NR4A1,ALDH1A3,ENSCAFG00000002015,PTP4A1,RGL2,JCAD,TMOD3,ZEB1,TET2,ARHGEF2,FSCN1,FBXO5,CDON,ZNF609	up_VCAV_EC
GO:0016239	0.00101	1	6	20	positive regulation of macroautophagy	BP	0.168	SESN2,HIF1A,BNIP3,CDC37,SH3GLB1,HTT	down_VSAP_EC
GO:0048568	0.00101	1	3	148	embryonic organ development	BP	0.786	TBX15,EYA1,IRX5	up_AFEM_VSMC
GO:0032412	0.00101	1	3	46	regulation of ion transmembrane	BP	0.387	NETO1,STIM1,CAV1	up_VSAP_VSMC
GO:0071606	0.00101	1	1	1	chemokine (C-C motif) ligand 4 production	BP	0.434	ARG2	up_AORTRT_VSMC
GO:0071608	0.00101	1	1	1	macrophage inflammatory protein-1	BP	0.434	ARG2	up_AORTRT_VSMC
GO:0071640	0.00101	1	1	1	regulation of macrophage inflammatory protein 1 alpha production	BP	0.434	ARG2	up_AORTRT_VSMC
GO:0071641	0.00101	1	1	1	negative regulation of macrophage inflammatory protein 1	BP	0.434	ARG2	up_AORTRT_VSMC
GO:0071643	0.00101	1	1	1	regulation of chemokine (C-C motif) ligand 4	BP	0.434	ARG2	up_AORTRT_VSMC
GO:0071644	0.00101	1	1	1	negative regulation of chemokine (C-C motif)	BP	0.434	ARG2	up_AORTRT_VSMC
GO:1905076	0.00101	1	1	1	regulation of interleukin-17 secretion	BP	0.434	ARG2	up_AORTRT_VSMC
GO:1905077	0.00101	1	1	1	negative regulation of interleukin-17 secretion	BP	0.434	ARG2	up_AORTRT_VSMC
GO:1905403	0.00101	1	1	1	negative regulation of activated CD8-positive, alpha-beta T cell	BP	0.434	ARG2	up_AORTRT_VSMC
GO:2000666	0.00101	1	1	1	negative regulation of interleukin-13 secretion	BP	0.434	ARG2	up_AORTRT_VSMC
GO:0051704	0.00102	0.999	58	476	multi-organism process	BP	0.0952	GBP5,KIT,LYZ,CXCL8,PTCHD1,TNFAIP3,ADAMTS9,TNIP1,MX2,BAIAP2L1,TMEM173,TSPAN8,PDE4D,MEF2C,CHD7,TLR4,NLRC5,GATA3,PSME4,IRF8,FIGNL1,PELI1,SERINC5,GUCY1A1,ODC1,ARG2,NECTIN2,MTOR,CDYL,CREM,ABCA1,RIOK3,PROX1,ARID4B,STMN1,THOC1,SMARCA4,TLR3,OAS3,NCK1,HIST1H1A,PTGS2,PLCG2,BCL10,CNOT7,GCH1,NOS2,PMAIP1,SPTBN4,LSM14A,AFF4,ARHGDIB,KDM3A,ATRX,IVNS1ABP,SPATA6,FBXO5,USP6NL	up_VCAV_EC
GO:0000086	0.00102	1	12	52	G2/M transition of mitotic cell cycle	BP	0.0701	BIRC5,CDK1,FANCI,TOPBP1,CDK4,RNASEH2B,DTL,CDK2,CCNA2,ENSCAFG00000005299,CLSPN,CCNB3	down_ACOR_VSMC
GO:1901184	0.00102	1	6	20	regulation of ERBB signaling pathway	BP	0.168	SOCS5,ERRFI1,PTPN2,ADAM17,CBLC,RHBDF2	down_VSAP_EC
GO:1904467	0.001026912	0.99996	3	8	regulation of tumor necrosis factor secretion	BP	0.7009284	NR1H4,FZD5,MAVS	up_ACOR_EC

GO:0048513	0.00103	0.999	101	906	animal organ development	BP	0.0952	EPHB1,EDNRB,HOXB7,HCLS1,PCDH12,IL7R,ADGRF5,KIT,CXCL8,PTCHD1,PRKCH,ARHGEF15,IL6,CALCRL,FLI1,TNFAIP3,ADAMTS9,PDGFB,PODXL,RELN,CD34,MEF2C,FERMT1,ATF3,CHD7,TIPARP,DLL4,GPR4,STC1,EIF2B2,GATA3,EDN1,HOXB8,APLNR,NTN4,FOXN2,IGSF3,ENSCAFG00000003530,IRF8,SOX18,TMOD1,PPP3CA,MTSS1,ODC1,ARG2,CASP8,MTOR,TENT2,HOOK3,BMP2,HHEX,CAV2,BMP2K,ITPKB,GDPD5,PROX1,ENSCAFG00000010224,MDFI,ENSCAFG00000010596,ARID4B,STMN1,FLNB,TSPAN12,SMARCA4,MFNG,APOD,BTBD7,TIMELESS,HOXB5,LDB2,SLC25A25,FES,JAG1,PTGS2,RAPGEF2,PLCG2,CREB1,BASP1,HMGB1,NUP133,RIPK1,CRIP2,ITGA6,ATP11C,SKIL,CUL4A,BMX,NR1D2,HIF1A,ATRX,ALDH1A3,P2RX2,LUZP1,RBBP6,VASH1,TMOD3,ZEB1,TET2,FBXO5,CDON,TJP2	up_VCAV_EC
GO:2000107	0.00103	1	6	16	negative regulation of leukocyte apoptotic	BP	0.0952	HCLS1,IL7R,ARG2,ITPKB,BCL10,HIF1A	up_VCAV_EC
GO:0061213	0.00103	1	2	11	positive regulation of mesonephros	BP	0.228	HOXB7,SIX1	up_VFEM_EC
GO:0002040	0.00104	1	11	46	sprouting angiogenesis	BP	0.0954	PLK2,TEK,CLEC14A,ADAMTS9,DLL4,APLNR,SMAD1,PIK3C2A,EPHB4,MAP3K3,JCAD	up_VCAV_EC
GO:0071398	0.00104	1	2	14	cellular response to fatty acid	BP	1	NR1H4,PTGER2	up_VPUL_EC
GO:0050900	0.00105	1	16	89	leukocyte migration	BP	0.0955	EDNRB,SELP,KIT,SELE,CXCL8,PDGFB,STK10,CD34,SELL,PDE4D,GATA3,MMP28,SWAP70,APOD,HMGB1,ITGA6	up_VCAV_EC
GO:0042474	0.00105	1	2	11	middle ear morphogenesis	BP	0.23	EYA1,SIX1	up_VFEM_EC
GO:0045167	0.00105	1	1	1	asymmetric protein localization involved in cell fate determination	BP	0.434	WNT7A	up_AORTRT_VSMC
GO:0061038	0.00105	1	1	1	uterus morphogenesis	BP	0.434	WNT7A	up_AORTRT_VSMC
GO:1904889	0.00105	1	1	1	regulation of excitatory synapse assembly	BP	0.434	WNT7A	up_AORTRT_VSMC
GO:1904891	0.00105	1	1	1	positive regulation of excitatory synapse	BP	0.434	WNT7A	up_AORTRT_VSMC
GO:0030099	0.00107	1	22	133	myeloid cell differentiation	BP	0.0967	HOXB7,HCLS1,ADGRF5,KIT,FLI1,MEF2C,GATA3,HOXB8,IRF8,CASP8,MTOR,HHEX,ITPKB,FES,JAG1,CREB1,RIPK1,CUL4A,HIF1A,TMOD3,TET2,TJP2	up_VCAV_EC
GO:0043300	0.00107	1	2	11	regulation of leukocyte degranulation	BP	0.315	LYN,STX4	down_ACOR_EC
GO:0065007	0.00109	0.999	375	3499	biological regulation	BP	0.515	SYNPO2L,EXPH5,TNNT2,SH3TC2,FGF13,RHOT1,ADRA1B,VEGFD,TMEM176B,TLR1,TMEM176A,DNAJC6,SMAD6,ATRNL1,ARHGAP28,ACTN2,LGR6,PLCG2,CASP10,IFNGR1,LEP,PLCL1,LBH,DCBLD2,MAP3K8,MID1,SLC26A7,KCNE3,GATA5,BCL2,GATA4,ADGRD1,CD83,EFNB2,CDC7,ANGPT1,PCGF5,ENSCAFG00000016285,HTR2B,RASSF2,TCAF1,MEIS2,USP6NL,FNDC5,MBP,ZRANB1,FBXL20,C28H10orf71,PRDX4,RECK,LIPT2,FEZ1,SKP2,NACC2,COCH,PRKAA2,MLLT3,ITM2C,FERMT1,BMP4,PNRC1,LCAT,ZNF331,TPC3,EVI5L,CRIM1,LNPK,SYNPO2,TUBB4A,TM7SF3,NPPB,CNNM2,FGFR2,TP53BP1,MX1,MFAP4,ABCA1,EIF4A2,GJA1,DOCK3,ELF1,SCARB2,ATRX,MED14,HRH1,CASTOR1,TRPS1,TLR3,SH3RF2,SEC24B,XIRP1,WASF3,PPARGC1A,LATS2,PDE5A,ZNF821,CITED2,TP53INP1,ATXN2,ZNF550,RERG,GLRB,PKP4,ARHGAP29,CCNG2,MAOA,ISG15,ZNRF3,ZNF782,CD274,ZNF365,PLPP2,MARCKSL1,AR,PTGIR,ZNF484,SARAF,SASH1,SLC2A5,STX11,GPR155,LMO4,GTF3C1,PTPN3,TMEM108,ZNF572,CLCN3,FGF18,FAM210B,ARHGEF26,NDRG2,ATRN,P4HTM,MICU2,TMX3,CHN2,CDKN2B,ADCY6,USP53,ZMYM2,ITPR1,GRK7,CST3,ZNF174,PLAC8B,KLHL20,LIMS2,BLNK,ANKRD1,PLEKHB2,F2RL1	up_ACOR_VSMC
GO:0007155	0.00109	0.999	29	324	cell adhesion	BP	0.928	GPAM,KIRREL1,CERCAM,FBLN2,ITGB5,PRNP,SDC4,FBN1,THBS3,TLN2,RHOD,ENSCAFG00000014978,TFE3,LAMA3,THY1,HAPLN3,VCAN,B4GALT1,GLI3,ZFP36L1,CDH2,EDIL3,LOXL3,CD164,SHC1,GREM1,RAC2,ZBTB16,ECM2	down_VCAV_EC
GO:0106106	0.00109	1	3	48	cold-induced thermogenesis	BP	0.396	GATM,HOXC10,CAV1	up_VSAP_VSMC

GO:0120161	0.00109	1	3	48	regulation of cold-induced thermogenesis	BP	0.396	GATM,HOXC10,CAV1	up_VSAP_VSMC
GO:0048731	0.0011	1	13	1247	system development	BP	0.396	HOXA11,TBX15,HOXA9,PITX1,EMX2,SALL1,HOXC10,IRX5,MAB21L1,CAV1,SHOX2,HAPLN1,GJB5	up_VSAP_VSMC
GO:0003170	0.0011	1	2	25	heart valve development	BP	0.599	TBX5,GATA5	up_VPUL_VSMC
GO:0098911	0.0011	1	1	4	regulation of ventricular cardiac muscle cell action	BP	0.773	JUP	down_VFEM_VSMC
GO:0007173	0.00111	1	7	27	epidermal growth factor receptor signaling	BP	0.18	SOCS5,ERRFI1,PTPN11,PTPN2,ADAM17,CBLC,RHBDF2	down_VSAP_EC
GO:0034097	0.00111	1	5	238	response to cytokine	BP	1	NR1H4,MRC1,CCL24,FLRT3,ARHGEF2	up_VPUL_EC
GO:1901228	0.00112	1	1	4	positive regulation of transcription from RNA polymerase II promoter involved in heart	BP	0.773	GATA5	down_VFEM_VSMC
GO:0031347	0.00113	1	23	144	regulation of defense response	BP	0.1	GBP5,TNFAIP3,TNIP1,BCL6B,TMEM173,MEF2C,TLR4,NLRC5,GATA3,PELI1,ARG2,NECTIN2,RIOK3,TLR3,APOD,OTUD4,PTGS2,CNOT7,HMGB1,NOS2,LSM14A,NR1D2,ENSCAFG00000025410	up_VCAV_EC
GO:0032970	0.00113	1	22	130	regulation of actin filament-based process	BP	0.1	TEK,HCLS1,ARHGEF15,BAIAP2L1,PDE4D,MEF2C,STC1,EDN1,ARHGAP28,TMOD1,MTOR,NCKAP1,PROX1,IQGAP2,STMN1,SWAP70,NCK1,FES,ARPC5L,ARHGDIB,TMOD3,FSCN1	up_VCAV_EC
GO:0007275	0.00114	0.999	109	1370	multicellular organism development	BP	0.18	ARHGEF2,PRICKLE1,VEGFA,SIPA1L1,SULF1,SERPINH1,MYH9,NR1H4,HSPA5,SERPINE1,ACTN1,SOCS5,ETV6,SPARC,LTBP1,SGK1,PRKACB,PTPN14,ERRFI1,ITGA2,SEC24D,JCAD,CASP3,PGK1,BCAR3,PTPN11,AARS,FLNB,ENSCAFG00000032470,DPYSL3,HOOK3,IFRD1,TMED2,ILK,FHL2,PRCP,SMARCA4,TGFBR1,PTGS2,WDR1,PIK3C2A,CHSY1,MSN,HOXB7,KIF5B,HIF1A,SLC45A3,SLC20A1,THBS2,ACTG2,TNIK,NXN,UBA6,ZPR1,PTPN2,UCHL5,EIF2B1,EVI2B,NEO1,MTDH,ESM1,ACTA2,ADAMTS6,UST,CDH11,MAGED1,CTNNB1,ANLN,CRYAB,STARD13,TFRC,APOE,PLOD3,KLF5,LYN,STRAP,DDIT3,PDS5B,MMD,PLS3,ATF3,GSK3B,VLDLR,NEXN,MYO1E,SDC4,HTT,REST,RBBP8,MAPK10,PRMT1,SH2B3,EXT1,GLO1,DISC1,PAK2,CREB3L2,LRRTM2,INHBA,CAMK2D,BTK,SIX4,NRP1,SPINK2,ARF4,YWHAH,COL12A1,RTCB,HOMER1	down_VSAP_EC
GO:0040011	0.00114	0.999	48	505	locomotion	BP	0.18	ARHGEF2,VEGFA,SULF1,MYH9,FMNL3,HSPA5,SERPINE1,SPARC,ITGA2,JCAD,PTPN11,FSCN1,DPYSL3,ILK,PRCP,WDR1,MYO1C,PIK3C2A,MSN,KIF5B,HIF1A,CX3CL1,PTPN2,NEO1,SDC3,ADAM17,PALLD,CTNNB1,ANLN,STARD13,APOE,PTP4A1,MMP28,LYN,STRAP,PDS5B,CCNYL1,NEXN,SDC4,ZMYND8,EXT1,DISC1,PAK2,SIX4,NRP1,USP14,ARF4,TNFAIP1	down_VSAP_EC
GO:0016525	0.00114	1	10	38	negative regulation of angiogenesis	BP	0.1	PLK2,TEK,TIE1,ADAMTS9,AMOT,DLL4,GPR4,HHEX,ANGPT2,VASH1	up_VCAV_EC
GO:2000181	0.00114	1	10	38	negative regulation of blood vessel	BP	0.1	PLK2,TEK,TIE1,ADAMTS9,AMOT,DLL4,GPR4,HHEX,ANGPT2,VASH1	up_VCAV_EC
GO:2001238	0.00114	1	7	23	positive regulation of extrinsic apoptotic	BP	0.1	ATF3,PAK2,BCL10,CTNNA1,PMAIP1,RIPK1,SKIL	up_VCAV_EC
GO:0010501	0.00114	1	6	15	RNA secondary structure unwinding	BP	0.0782	DDX39A,EIF4A3,DDX23,DDX51,DDX28,DDX39B	down_ACOR_VSMC
GO:0031032	0.001141497	0.9998	7	61	actomyosin structure organization	BP	0.7009284	MYOZ2,TMOD1,ARHGAP6,ACTN2,ARHGAP28,PTGER4,NRP1	up_ACOR_EC
GO:0051246	0.00115	0.999	71	832	regulation of protein metabolic process	BP	0.18	ARHGEF2,PRICKLE1,VEGFA,MYH9,PLK3,HSPA5,SERPINE1,SOCS5,PRKACB,ENPP1,ERRFI1,JCAD,SNX25,BCAR3,NUAK1,PTPN11,AARS,TRIB3,PUM3,PM20D2,ILK,PPP1R12A,SESN2,PTGS2,MSN,IWS1,DNAJC10,TNIK,NXN,PTPN2,UCHL5,SPOPL,YES1,PLCL1,ADAM17,EIF4EBP1,CNOT7,MAGED1,CTNNB1,CRYAB,APOE,LYN,STRAP,FAM162A,CCNYL1,EIF5,MMD,CBLC,ATF3,GSK3B,VLDLR,HTT,REST,EIF2S1,SPOP,CALM1,DISC1,RAD23A,CDYL,PAK2,A2M,INHBA,CDKN1A,TMTC3,NRP1,SPINK2,SH3RF3,USP14,MAD,KDM3A,MID1	down_VSAP_EC
GO:0048731	0.00115	1	8	1247	system development	BP	0.599	HOXA3,TBX5,GATA5,TMEM176A,TMEM176B,PHEX,HOXB5,SLIT2	up_VPUL_VSMC
GO:0022898	0.00115	1	3	48	regulation of transmembrane	BP	0.404	NETO1,STIM1,CAV1	up_VSAP_VSMC

GO:0032720	0.00115	1	2	13	negative regulation of tumor necrosis factor	BP	1	NR1H4,BPI	up_APUL_EC
GO:0086069	0.00115	1	1	4	bundle of His cell to Purkinje myocyte	BP	0.773	JUP	down_VFEM_VSMC
GO:0048704	0.00116	1	3	46	embryonic skeletal system morphogenesis	BP	0.335	HOXA3,HOXA7,HOXA1	down_ACOR_EC
GO:0048706	0.00117	1	14	59	embryonic skeletal system development	BP	0.803	TBX15,HOXA11,HOXA7,HOXA9,WNT11,OSR2,IRX5,HOXD3,HOXA6,OSR1,ZEB1,SULF2,SHOX2,TGFBR2	up_VSAP_EC
GO:0002429	0.00117	1	9	35	immune response-activating cell surface receptor signaling	BP	0.101	PRKCH,PDE4D,MEF2C,EIF2B2,GATA3,NECTIN2,PRKD2,PLCG2,BCL10	up_VCAV_EC
GO:0010633	0.00117	1	8	28	negative regulation of epithelial cell migration	BP	0.101	ADAMTS9,MEF2C,DLL4,STC1,ANGPT2,MCC,HMGB1,VASH1	up_VCAV_EC
GO:0060828	0.00117	1	3	82	regulation of canonical Wnt signaling pathway	BP	1	NKX2-5,BAMBI,ANKRD6	up_VPOR_VSMC
GO:0110053	0.001176249	0.99973	9	98	regulation of actin filament organization	BP	0.7009284	TMOD1,ARHGAP6,SYNPO2,ACTN2,ARHGAP28,PAK3,PTGER4,NRP1,PRKCD	up_ACOR_EC
GO:0080134	0.00118	0.999	50	386	regulation of response to stress	BP	0.101	EPHB1,PLK2,GBP5,SELP,TNFAIP3,MAP3K11,TNIP1,PDGFB,BCL6B,TMEM173,CD34,MEF2C,KLKB1,TLR4,NLRC5,GATA3,EDN1,TFPI,FIGNL1,PELI1,PPP3CA,ARG2,NECTIN2,MTOR,MAP3K3,BMP2,RIOK3,THOC1,TLR3,APOD,NCK1,TIMELESS,OTUD4,PTGS2,ETAA1,TAOK1,CNOT7,GCH1,HMGB1,NOS2,PMAIP1,RIPK1,LSM14A,SKIL,CUL4A,NR1D2,ENSCAFG00000025410,HIF1A,VASH1,ARHGEF2	up_VCAV_EC
GO:0071887	0.00118	1	9	34	leukocyte apoptotic process	BP	0.101	HCLS1,IL7R,MEF2C,ARG2,ITPKB,RAPGEF2,BCL10,RIPK1,HIF1A	up_VCAV_EC
GO:0043086	0.00119	1	5	170	negative regulation of catalytic activity	BP	0.336	LYN,IGF1R,PRKAR2A,MYO1D,ASPN	down_ACOR_EC
GO:0016477	0.0012	0.999	42	426	cell migration	BP	0.186	ARHGEF2,VEGFA,SULF1,MYH9,FMNL3,HSPA5,SERPINE1,SPARC,ITGA2,JCAD,PTPN11,FSCN1,DPYSL3,ILK,PRCP,WDR1,MYO1C,PIK3C2A,MSN,HIF1A,CX3CL1,SDC3,ADAM17,PALLD,CTNNB1,ANLN,STARD13,APOE,PTP4A1,MMP28,LYN,STRAP,PDS5B,NEXN,SDC4,ZMYND8,DISC1,PAK2,SIX4,NRP1,ARF4,TNFAIP1	down_VSAP_EC
GO:0006336	0.0012	1	5	11	DNA replication-independent nucleosome	BP	0.0819	OIP5,IPO4,CENPP,HAT1,MIS18A	down_ACOR_VSMC
GO:0044281	0.00121	0.999	83	622	small molecule metabolic process	BP	0.803	PEX7,NAPEPLD,HNF4A,ACSM3,DHDH,LIPC,INSIG2,ACAA1,ACACB,GDA,NADK,PEX2,GOT1,DLST,INPP5E,GLUL,ADHFE1,ENSCAFG00000013986,WARS2,C1QTNF2,COQ9,ALDH6A1,NDUFS1,ENSCAFG00000004260,HSD17B8,FAAH,LMF1,PPIP5K1,PDK2,GALK2,FXN,GALK1,RETSAT,ENTPD3,PTGES,SUOX,CTNS,HACD4,GCDH,ABAT,BCKDHB,ENSCAFG00000016140,CLYBL,NDUFS2,PASK,NT5C2,AZIN2,DAGLB,KHK,MARC1,EHHADH,FARS2,LCAT,PCBD1,LONP2,ACSS2,TKFC,PC,DHRS9,MVK,ABHD16A,LIPE,PNPO,DCAKD,ENSCAFG00000002977,HPGD,ENSCAFG00000013366,PNP,NCOA2,QARS,ENSCAFG00000023820,IDH1,IDH3A,CYB5R3,ENSCAFG00000006639,ASS1,NDUFV1,CHST7,LARS2,AHCYL1,DDIT4,SOAT1,IDO2	up_VSAP_EC
GO:0022610	0.00121	0.999	29	326	biological adhesion	BP	0.973	GPAM,KIRREL1,CERCAM,FBLN2,ITGB5,PRNP,SDC4,FBN1,THBS3,TLN2,RHOD,ENSCAFG00000014978,TFE3,LAMA3,THY1,HAPLN3,VCAN,B4GALT1,GLI3,ZFP36L1,CDH2,EDIL3,LOXL3,CD164,SHC1,GREM1,RAC2,ZBTB16,ECM2	down_VCAV_EC
GO:0072359	0.00121	1	4	368	circulatory system development	BP	0.69	SLITRK5,GATA5,C6,TGFBI	up_VCAV_VSMC
GO:0032330	0.00121	1	2	20	regulation of chondrocyte differentiation	BP	0.495	HOXA11,SHOX2	up_VFEM_VSMC
GO:0070542	0.00121	1	2	15	response to fatty acid	BP	1	NR1H4,PTGER2	up_VPUL_EC
GO:0002934	0.00121	1	1	4	desmosome organization	BP	0.773	JUP	down_VFEM_VSMC
GO:0003008	0.00122	0.999	44	336	system process	BP	0.104	EPHB1,PLK2,EDNRB,ADGRF5,KIT,PRCP,PTCHD1,DOCK4,FLI1,ATP8A1,PDGFB,RELN,CD34,PDE4D,MEF2C,CHD7,TLR4,STC1,GATA3,EDN1,HOXB8,HOMER3,PTGS1,TMOD1,PPP3CA,GUCY1A1,TMEM150C,ARG2,MTOR,NAV2,ACPP,MMP24,KCNJ2,PTGS2,CREB1,GCH1,NOS2,SPTBN4,HIF1A,ALDH1A3,DOP1B,P2RX2,ATP2A1,TMOD3	up_VCAV_EC

GO:0034612	0.00122	1	3	63	response to tumor necrosis factor	BP	1	NR1H4,CCL24,ARHGEF2	up_VPUL_EC
GO:0042496	0.00122	1	1	1	detection of diacyl bacterial lipopeptide	BP	0.786	TLR2	up_AFEM_VSMC
GO:0071724	0.00122	1	1	1	response to diacyl bacterial lipopeptide	BP	0.786	TLR2	up_AFEM_VSMC
GO:0071726	0.00122	1	1	1	cellular response to diacyl bacterial lipopeptide	BP	0.786	TLR2	up_AFEM_VSMC
GO:1903971	0.00122	1	1	1	positive regulation of response to macrophage colony-stimulating factor	BP	0.786	TLR2	up_AFEM_VSMC
GO:1903974	0.00122	1	1	1	positive regulation of cellular response to macrophage colony-	BP	0.786	TLR2	up_AFEM_VSMC
GO:0030326	0.00123	1	3	51	embryonic limb morphogenesis	BP	0.26	HOXC10,HOXA9,CYP26B1	up_VFEM_EC
GO:0035113	0.00123	1	3	51	embryonic appendage morphogenesis	BP	0.26	HOXC10,HOXA9,CYP26B1	up_VFEM_EC
GO:0030177	0.00123	1	3	51	positive regulation of Wnt signaling pathway	BP	0.415	SALL1,TLR2,CAV1	up_VSAP_VSMC
GO:0003156	0.00123	1	2	20	regulation of animal organ formation	BP	0.495	EYA1,HOXA11	up_VFEM_VSMC
GO:0032787	0.00124	0.999	26	144	monocarboxylic acid metabolic process	BP	0.803	PEX7,ACSM3,LIPC,INSIG2,ACAA1,ACACB,PEX2,C1QTNF2,HSD17B8,FAAH,PDK2,GALK1,PTGES,HACD4,GCDH,ABAT,DAGLB,EHHADH,LONP2,ACSS2,PC,DHRS9,ABHD16A,HPGD,ENSCAFG00000023820,DDIT4	up_VSAP_EC
GO:0007519	0.00124	1	3	49	skeletal muscle tissue development	BP	0.415	PITX1,CAV1,SHOX2	up_VSAP_VSMC
GO:0090189	0.00125	1	2	12	regulation of branching involved in ureteric bud	BP	0.262	HOXB7,SIX1	up_VFEM_EC
GO:0070972	0.00126	1	6	23	protein localization to endoplasmic reticulum	BP	0.193	HSPA5,TRAM1,SRP54,SRPRA,SRP72,KDELR2	down_VSAP_EC
GO:0046661	0.00126	1	2	45	male sex differentiation	BP	0.504	HOXA9,ESR1	up_AORTRT_VSMC
GO:0003176	0.00126	1	1	9	aortic valve development	BP	1	GATA5	down_AFEM_VSMC
GO:1990845	0.00127	1	3	51	adaptive thermogenesis	BP	0.415	GATM,HOXC10,CAV1	up_VSAP_VSMC
GO:0030513	0.00128	1	2	11	positive regulation of BMP signaling pathway	BP	0.353	ACVRL1,SULF1	down_ACOR_EC
GO:0060575	0.00128	1	1	9	intestinal epithelial cell differentiation	BP	1	GATA5	down_AFEM_VSMC
GO:0043535	0.00129	1	9	45	regulation of blood vessel endothelial cell migration	BP	0.193	VEGFA,JCAD,PRCP,PIK3C2A,HIF1A,ADAM17,STARD13,APOE,NRP1	down_VSAP_EC
GO:0016477	0.00129	1	8	426	cell migration	BP	0.353	ACVRL1,LYN,IGF1R,PDPN,NRP2,HOXA7,SULF1,STX4	down_ACOR_EC
GO:0010822	0.00129	1	7	31	positive regulation of mitochondrion	BP	0.193	HIF1A,BNIP3,FAM162A,GSK3B,CDC37,SH3GLB1,HTT	down_VSAP_EC
GO:0050819	0.00129	1	5	12	negative regulation of coagulation	BP	0.109	PDGFB,CD34,KLKB1,TFPI,PROCR	up_VCAV_EC
GO:0009083	0.00129	1	3	5	branched-chain amino acid catabolic process	BP	0.993	ENSCAFG00000002977,BCKDHB,ACAT1	down_VCAV_EC
GO:1901379	0.00129	1	2	12	regulation of potassium ion transmembrane	BP	0.415	NETO1,CAV1	up_VSAP_VSMC
GO:1900122	0.00129	1	1	3	positive regulation of receptor binding	BP	1	PLCL1	down_VFEM_EC
GO:0071680	0.00129	1	1	4	response to indole-3-methanol	BP	0.773	JUP	down_VFEM_VSMC
GO:0071681	0.00129	1	1	4	cellular response to indole-3-methanol	BP	0.773	JUP	down_VFEM_VSMC
GO:0050715	0.0013	1	9	29	positive regulation of cytokine secretion	BP	0.546	TLR1,HTR2B,MBP,FERMT1,CD274,F2RL1,PTPN22,NOD2,SCAMP5	up_ACOR_VSMC
GO:0010458	0.0013	1	5	12	exit from mitosis	BP	0.0872	UBE2S,ZW10,BIRC5,ENSCAFG00000005299,UBE2C	down_ACOR_VSMC
GO:0009952	0.00131	1	15	66	anterior/posterior pattern specification	BP	0.815	HOXA11,EMX2,HOXA7,HOXA9,CDON,AXIN2,HOXD3,DLL1,HOXA6,OSR1,ZBTB16,RARG,HOXC4,HOXC10,KDM2B	up_VSAP_EC
GO:2000146	0.00132	1	17	92	negative regulation of cell motility	BP	0.11	TIE1,ADAMTS9,MEF2C,DLL4,STC1,GATA3,MMP28,ARAP3,ANGPT2,ENSCAFG00000010224,MCC,APOD,JAG1,CTNNA1,HMGB1,ARHGDIB,VASH1	up_VCAV_EC
GO:0032508	0.00132	1	5	11	DNA duplex unwinding	BP	0.0879	MCM6,MCM4,MCM7,GINS4,RAD51	down_ACOR_VSMC
GO:0008608	0.00132	1	4	7	attachment of spindle microtubules to	BP	0.0879	DSN1,NEK2,NDC80,KNL1	down_ACOR_VSMC

GO:1902680	0.00133	0.999	54	415	positive regulation of RNA biosynthetic process	BP	0.11	HCLS1,PPARG,TNIP1,PDGFB,TMEM173,MEF2C,ATF3,CHD7,TLR4,NLRC5,GATA3,EDN1,TCF4,FOXN2,ENSCAFG00000017103,IRF8,PPP3CA,YES1,MTOR,BMP2,HHEX,CREM,CNOT6,PROX1,ARID4B,HMBOX1,PRKD2,THOC1,SMARCA4,TLR3,NCK1,LDB2,JAG1,NSD1,BCL10,CNOT7,HMGB1,AHR,BLM,RIPK1,ITGA6,GPBP1,MTA2,NR1D2,KDM5A,HIF1A,KDM3A,ATRX,NR4A1,ZEB1,TET2,ARHGEF2,CDON,ZNF609	up_VCAV_EC
GO:1903508	0.00133	0.999	54	415	positive regulation of nucleic acid-templated transcription	BP	0.11	HCLS1,PPARG,TNIP1,PDGFB,TMEM173,MEF2C,ATF3,CHD7,TLR4,NLRC5,GATA3,EDN1,TCF4,FOXN2,ENSCAFG00000017103,IRF8,PPP3CA,YES1,MTOR,BMP2,HHEX,CREM,CNOT6,PROX1,ARID4B,HMBOX1,PRKD2,THOC1,SMARCA4,TLR3,NCK1,LDB2,JAG1,NSD1,BCL10,CNOT7,HMGB1,AHR,BLM,RIPK1,ITGA6,GPBP1,MTA2,NR1D2,KDM5A,HIF1A,KDM3A,ATRX,NR4A1,ZEB1,TET2,ARHGEF2,CDON,ZNF609	up_VCAV_EC
GO:0016053	0.00134	1	14	97	organic acid biosynthetic process	BP	0.195	ASNS,PLOD2,NR1H4,PGK1,TPI1,TRIB3,PGAM1,PTGS2,HIF1A,SLC45A3,ALDH18A1,ALDOC,PLOD3,BCAT1	down_VSAP_EC
GO:0046394	0.00134	1	14	97	carboxylic acid biosynthetic process	BP	0.195	ASNS,PLOD2,NR1H4,PGK1,TPI1,TRIB3,PGAM1,PTGS2,HIF1A,SLC45A3,ALDH18A1,ALDOC,PLOD3,BCAT1	down_VSAP_EC
GO:1903556	0.00134	1	2	14	negative regulation of tumor necrosis factor superfamily cytokine	BP	1	NR1H4,BPI	up_APUL_EC
GO:0051050	0.001344794	0.99954	15	235	positive regulation of transport	BP	0.7009284	CDH3,NR1H4,CALY,AQP1,ENSCAFG00000004331,KCNB1,EDEM2,FZD5,NOD2,ACTN2,PTGER4,MAVS,RARRES2,AZIN2,PRKCD	up_ACOR_EC
GO:1902749	0.00136	1	10	39	regulation of cell cycle G2/M phase transition	BP	0.0893	CDK1,FANCI,TOPBP1,CDK4,RNASEH2B,DTL,CDK2,CLSPN,CDC25A,CCNB3	down_ACOR_VSMC
GO:0035909	0.00136	1	2	14	aorta morphogenesis	BP	0.281	EYA1,SIX1	up_VFEM_EC
GO:0048566	0.00136	1	2	13	embryonic digestive tract development	BP	0.429	SALL1,SHOX2	up_VSAP_VSMC
GO:0009888	0.00137	1	9	545	tissue development	BP	0.367	ACVRL1,HOXA3,PDPN,NRP2,HOXA7,SULF1,CSGALNACT1,HOXC4,ASPN	down_ACOR_EC
GO:0007267	0.00137	1	6	332	cell-cell signaling	BP	1	NR1H4,TBX5,HTR7,PLCL1,SLITRK5,NTRK2	up_APUL_EC
GO:0051239	0.00137	1	5	859	regulation of multicellular organismal process	BP	0.504	HOXC10,HOXA9,WNT7A,ARG2,ESR1	up_AORTRT_VSMC
GO:0086005	0.00137	1	1	5	ventricular cardiac muscle cell action potential	BP	0.773	JUP	down_VFEM_VSMC
GO:0048193	0.00138	1	16	116	Golgi vesicle transport	BP	0.198	SEC24D,HYOU1,EXOC5,SEC23A,RAB6A,LMAN1,YIPF5,USO1,COPB2,HTT,CREB3L2,STX6,ARF4,BLZF1,SEC13,RINT1	down_VSAP_EC
GO:0007186	0.001386572	0.99964	10	124	G protein-coupled receptor signaling	BP	0.7009284	PLCL1,CALY,PTH1R,RGS1,AGRN,GNAO1,PTGIR,ACTN2,PTGER4,GNAZ	up_ACOR_EC
GO:0048519	0.001389674	0.9992	59	1554	negative regulation of biological process	BP	0.7009284	CDH3,NR1H4,TMOD1,BRSK1,TRPS1,PLCL1,PKIB,SOX9,ARHGAP6,PTH1R,PCSK1N,FNDC4,NDRG2,AQP1,KCNB1,FZD5,CCDC3,IFI6,SEMA6D,DUSP26,DIXDC1,PTGIR,RGMA,SMAD6,ACTN2,ZMYND8,ARHGAP28,RNLS,MMP28,BCL2,TRIM8,PTGER4,ARHGDIB,CST3,PIAS1,DCLK2,ADAMTSL2,MAPK8IP2,MAVS,FOXC2,FIGNL1,LRRC15,TBX5,LRP4,CLSTN1,LRRC4,CLU,SFMBT2,CHMP4B,TP53INP2,RIOX2,NRP1,AZIN2,PRKCD,PPP1R26,PM20D1,DBNDD2,EFEMP1,TUBB4A	up_ACOR_EC
GO:0001817	0.00139	0.999	27	182	regulation of cytokine production	BP	0.114	ACKR1,GBP5,TNFAIP3,TMEM173,CD34,PDE4D,FERMT1,TLR4,GATA3,HOMER3,IRF8,PELI1,ARG2,MAPK13,RIOK3,PRKD2,TLR3,APOD,CD200,PLCG2,BCL10,HMGB1,NOS2,RIPK1,LSM14A,HIF1A,ARHGEF2	up_VCAV_EC
GO:0003281	0.0014	1	9	30	ventricular septum development	BP	0.546	SMAD6,BMP4,FGFR2,CITED2,LMO4,EGLN1,TBX5,FRS2,AP2B1	up_ACOR_VSMC
GO:1905330	0.0014	1	2	48	regulation of morphogenesis of an	BP	0.504	WNT7A,ESR1	up_AORTRT_VSMC
GO:0001905	0.0014	1	1	1	activation of membrane attack complex	BP	0.69	C6	up_VCAV_VSMC
GO:0001970	0.0014	1	1	1	positive regulation of activation of membrane	BP	0.69	C6	up_VCAV_VSMC
GO:0002922	0.0014	1	1	1	positive regulation of humoral immune	BP	0.69	C6	up_VCAV_VSMC
GO:0001969	0.0014	1	1	1	regulation of activation of membrane attack	BP	0.69	C6	up_VCAV_VSMC

GO:2000259	0.0014	1	1	1	positive regulation of protein activation cascade	BP	0.69	C6	up_VCAV_VSMC
GO:0045917	0.0014	1	1	1	positive regulation of complement activation	BP	0.69	C6	up_VCAV_VSMC
GO:0006351	0.00141	1	6	1069	transcription, DNA-templated	BP	0.795	TLR2,TBX15,EYA1,HOXA9,HOXA10,IRX5	up_AFEM_VSMC
GO:0048732	0.00141	1	4	116	gland development	BP	0.429	HOXA9,PITX1,SALL1,CAV1	up_VSAP_VSMC
GO:0060538	0.00141	1	3	51	skeletal muscle organ development	BP	0.429	PITX1,CAV1,SHOX2	up_VSAP_VSMC
GO:0048871	0.001417327	0.99963	10	120	multicellular organismal homeostasis	BP	0.7009284	CDH3,PLCL1,SOX9,PTH1R,AQP1,NOD2,FOXC2,CHMP4B,TP53INP2,PM20D1	up_ACOR_EC
GO:0035150	0.00143	1	10	36	regulation of tube size	BP	0.546	ADRA1B,HTR2B,NPPB,HRH1,ADCY6,F2RL1,HRH2,PTGDR,EDN1,DOCK5	up_ACOR_VSMC
GO:0050880	0.00143	1	10	36	regulation of blood vessel size	BP	0.546	ADRA1B,HTR2B,NPPB,HRH1,ADCY6,F2RL1,HRH2,PTGDR,EDN1,DOCK5	up_ACOR_VSMC
GO:0097659	0.00143	1	6	1072	nucleic acid-templated transcription	BP	0.795	TLR2,TBX15,EYA1,HOXA9,HOXA10,IRX5	up_AFEM_VSMC
GO:0019538	0.00144	0.999	128	1713	protein metabolic process	BP	0.202	ARHGEF2,PRICKLE1,VEGFA,P4HB,SULF1,SERPINH1,CARS,PLOD2,MYH9,NR1H4,PLK3,HSPA5,SERPINE1,SOCS5,HM13,SGK1,ERO1A,PRKACB,HSP90B1,ENPP1,ERRFI1,ENSCAFG00000005575,P4HA1,JCAD,SNX25,SARS,PGK1,WSB1,BCAR3,NUAK1,PTPN11,AARS,ENSCAFG00000032470,TRIB3,PUM3,PM20D2,ILK,PPP1R12A,PRCP,SESN2,PTGS2,PPIB,CHSY1,MSN,SEL1L,HIF1A,USP12,MRPL1,IWS1,DNAJC10,TNIK,GALNT1,NAA10,NXN,UBA6,PTPN2,UCHL5,MORF4L2,SPOPL,YARS,TARS,YES1,PLCL1,TTLL7,NARS,ADAM17,EIF4EBP1,CNOT7,MAGED1,DNAJB9,CTNNB1,CRYAB,CDK8,APOE,PLOD3,USP48,UBA5,LYN,BNIP3,STRAP,DDIT3,FAM162A,BACH1,ASPH,CCNYL1,STT3A,EIF5,MMD,CBLC,ATF3,GSK3B,VLDLR,GTF2H1,CUL2,HTT,REST,ZMYND8,MAPK10,EIF2S1,PRMT1,P3H4,EXT1,SPOP,CALM1,DISC1,RAD23A,CDYL,PAK2,A2M,INHBA,CAMK2D,HIF1AN,BTK,MSRB3,CDKN1A,TMTC3,RSL24D1,NRP1,SPINK2,SH3RF3,RHBDF2,USP14,ARF4,TNFAIP1,MADD,RPN1,KDM3A,MID1	down_VSAP_EC
GO:0050793	0.00144	0.999	64	731	regulation of developmental process	BP	0.202	ARHGEF2,PRICKLE1,VEGFA,SIPA1L1,SULF1,CELF1,MYH9,NR1H4,FMNL3,SERPINE1,SOCS5,SPARC,PRKACB,ENPP1,ERRFI1,JCAD,PGK1,AKAP2,NUAK1,PTPN11,DPYSL3,HOOK3,TRIB3,IFRD1,ILK,TGFBR1,PTGS2,WDR1,PIK3C2A,MSN,HOXB7,HIF1A,SLC45A3,THBS2,TNIK,ZPR1,PTPN2,EVI2B,MTDH,UST,MAGED1,CTNNB1,STARD13,ENSCAFG00000002015,TFRC,APOE,WWC3,KLF5,LYN,BNIP3,STRAP,DDIT3,MMD,VLDLR,REST,PRMT1,DISC1,LRRTM2,INHBA,HIF1AN,SIX4,NRP1,YWHAH,KDM3A	down_VSAP_EC
GO:0032774	0.00144	1	6	1073	RNA biosynthetic process	BP	0.795	TLR2,TBX15,EYA1,HOXA9,HOXA10,IRX5	up_AFEM_VSMC
GO:0010614	0.00144	1	1	5	negative regulation of cardiac muscle	BP	0.773	GATA5	down_VFEM_VSMC
GO:0014741	0.00144	1	1	5	negative regulation of muscle hypertrophy	BP	0.773	GATA5	down_VFEM_VSMC
GO:0006629	0.00145	0.999	55	382	lipid metabolic process	BP	0.868	PEX7,NAPEPLD,HNF4A,ACSM3,LIPC,INSIG2,ENPP2,PLBD1,ACAA1,ACACB,PEX2,SULT1A1,INPP5E,CERS4,HSD17B14,DHRS4,KDELC2,C1QTNF2,PAFAH2,ABHD15,HSD3B7,HSD17B8,PLPP3,ENSCAFG00000000071,FAAH,AMBRA1,RBL2,LMF1,LPL,PDK2,RETSAT,PIK3R1,PLA2G6,PIK3IP1,PTGES,HACD4,GCDH,GDPD5,DAGLB,EHHADH,LCAT,LONP2,DHRS9,MVK,ABHD16A,LIPE,FAXDC2,HPGD,ZFYVE21,ENSCAFG00000023820,IDH1,CYB5R3,PLPP5,B3GALT4,SOAT1	up_VSAP_EC
GO:0048731	0.00145	1	9	1247	system development	BP	1	CHL1,HOXA3,BARX1,NKX2-5,BAMBI,TRPC4,ANKRD6,CYP26B1,C3AR1	up_VPOR_VSMC
GO:0060562	0.00145	1	4	120	epithelial tube morphogenesis	BP	0.287	EYA1,HOXB7,SIX1,MTSS1	up_VFEM_EC
GO:0032409	0.00145	1	3	52	regulation of transporter activity	BP	0.434	NETO1,STIM1,CAV1	up_VSAP_VSMC
GO:0002933	0.00145	1	1	1	lipid hydroxylation	BP	0.69	CYP1A1	up_VCAV_VSMC

GO:0001932	0.001452741	0.99939	22	416	regulation of protein phosphorylation	BP	0.7009284	PLCL1,PKIB,SOX9,NDRG2,ENSCAFG00000004331,FZD5,DUSP26,NOD2,SMAD6,PAK3,TNFRSF19,MAPK8IP2,MAVS,LRRC15,LRP4,SH3RF3,LRRC4,RARRES2,NRP1,PRKCD,DBNDD2,CCNYL1	up_ACOR_EC
GO:0007015	0.001452856	0.99959	11	144	actin filament organization	BP	0.7009284	TMOD1,ARHGAP6,SYNPO2,PDLIM3,PLS1,ACTN2,ARHGAP28,PAK3,PTGER4,NRP1,PRKCD	up_ACOR_EC
GO:1903827	0.001459915	0.99956	12	164	regulation of cellular protein localization	BP	0.7009284	SH3TC2,KCNB1,EDEM2,PLS1,ACTN2,TRIM8,DCLK2,MAVS,LRRC15,LRP4,TP53INP2,PRKCD	up_ACOR_EC
GO:0044839	0.00146	1	13	61	cell cycle G2/M phase transition	BP	0.0927	BIRC5,CDK1,FANCI,TOPBP1,CDK4,RNASEH2B,DTL,CDK2,CCNA2,ENSCAFG00000005299,CLSPN,CDC25A,CCNB3	down_ACOR_VSMC
GO:0043618	0.00146	1	6	22	regulation of transcription from RNA polymerase II promoter in response to	BP	0.202	SESN2,HIF1A,DDIT3,BACH1,ATF3,HIF1AN	down_VSAP_EC
GO:0006614	0.00146	1	4	10	SRP-dependent cotranslational protein	BP	0.202	TRAM1,SRP54,SRPRA,SRP72	down_VSAP_EC
GO:0007268	0.00146	1	4	130	chemical synaptic transmission	BP	1	HTR7,PLCL1,SLITRK5,NTRK2	up_APUL_EC
GO:0098916	0.00146	1	4	130	anterograde trans-synaptic signaling	BP	1	HTR7,PLCL1,SLITRK5,NTRK2	up_APUL_EC
GO:1903706	0.00146	1	4	119	regulation of hemopoiesis	BP	0.287	HOXA9,TOX,HOXA7,CYP26B1	up_VFEM_EC
GO:0031055	0.00146	1	3	4	chromatin remodeling at centromere	BP	0.0927	OIP5,CENPP,MIS18A	down_ACOR_VSMC
GO:0034080	0.00146	1	3	4	CENP-A containing nucleosome assembly	BP	0.0927	OIP5,CENPP,MIS18A	down_ACOR_VSMC
GO:0061641	0.00146	1	3	4	CENP-A containing chromatin organization	BP	0.0927	OIP5,CENPP,MIS18A	down_ACOR_VSMC
GO:0045087	0.00147	0.999	25	161	innate immune response	BP	0.12	GBP5,TNFAIP3,TNIP1,MX2,TMEM173,TLR4,NLRC5,RAB27A,IRF8,PELI1,SERINC5,NECTIN2,ELF4,RIOK3,FLNB,TLR3,OTUD4,FES,BCL10,CNOT7,GCH1,HMGB1,LSM14A,BMX,ARHGEF2	up_VCAV_EC
GO:0003018	0.00147	1	11	42	vascular process in circulatory system	BP	0.546	ADRA1B,HTR2B,NPPB,HRH1,ADCY6,F2RL1,HRH2,CLSTN1,PTGDR,EDN1,DOCK5	up_ACOR_VSMC
GO:0048562	0.00147	1	4	102	embryonic organ morphogenesis	BP	0.388	HOXA3,HOXA7,HOXA1,HOXC4	down_ACOR_EC
GO:0061217	0.00147	1	2	13	regulation of mesonephros	BP	0.287	HOXB7,SIX1	up_VFEM_EC
GO:0035480	0.00147	1	1	1	regulation of Notch signaling pathway involved in heart	BP	0.69	GATA5	up_VCAV_VSMC
GO:0035481	0.00147	1	1	1	positive regulation of Notch signaling pathway involved in heart	BP	0.69	GATA5	up_VCAV_VSMC
GO:0048539	0.00147	1	1	1	bone marrow development	BP	0.69	LRRC17	up_VCAV_VSMC
GO:0003137	0.00147	1	1	1	Notch signaling pathway involved in heart	BP	0.69	GATA5	up_VCAV_VSMC
GO:0061999	0.00147	1	1	1	regulation of cardiac endothelial to	BP	0.69	GATA5	up_VCAV_VSMC
GO:0062000	0.00147	1	1	1	positive regulation of cardiac endothelial to mesenchymal transition	BP	0.69	GATA5	up_VCAV_VSMC
GO:0090381	0.00147	1	1	1	regulation of heart induction	BP	0.69	GATA5	up_VCAV_VSMC
GO:1901321	0.00147	1	1	1	positive regulation of heart induction	BP	0.69	GATA5	up_VCAV_VSMC
GO:0032847	0.00148	1	1	1	regulation of cellular pH reduction	BP	0.69	CA2	up_VCAV_VSMC
GO:0032849	0.00148	1	1	1	positive regulation of cellular pH reduction	BP	0.69	CA2	up_VCAV_VSMC
GO:0042938	0.00148	1	1	1	dipeptide transport	BP	0.69	CA2	up_VCAV_VSMC
GO:0090088	0.00148	1	1	1	regulation of oligopeptide transport	BP	0.69	CA2	up_VCAV_VSMC
GO:0090089	0.00148	1	1	1	regulation of dipeptide transport	BP	0.69	CA2	up_VCAV_VSMC
GO:2000878	0.00148	1	1	1	positive regulation of oligopeptide transport	BP	0.69	CA2	up_VCAV_VSMC
GO:0035442	0.00148	1	1	1	dipeptide transmembrane transport	BP	0.69	CA2	up_VCAV_VSMC
GO:2000880	0.00148	1	1	1	positive regulation of dipeptide transport	BP	0.69	CA2	up_VCAV_VSMC
GO:2001148	0.00148	1	1	1	regulation of dipeptide transmembrane transport	BP	0.69	CA2	up_VCAV_VSMC

GO:2001150	0.00148	1	1	1	positive regulation of dipeptide transmembrane	BP	0.69	CA2	up_VCAV_VSMC
GO:0051493	0.001484217	0.99953	13	188	regulation of cytoskeleton organization	BP	0.7009284	TMOD1,ARHGAP6,SYNPO2,DIXDC1,ACTN2,ARHGAP28,PAK3,PTGER4,ARHGDIB,CHMP4B,NRP1,PRKCD,TUBB4A	up_ACOR_EC
GO:0003179	0.001489276	0.99988	4	19	heart valve morphogenesis	BP	0.7009284	GATA4,SOX9,SMAD6,TBX5	up_ACOR_EC
GO:0099537	0.00149	1	4	131	trans-synaptic signaling	BP	1	HTR7,PLCL1,SLITRK5,NTRK2	up_APUL_EC
GO:0003002	0.00149	1	3	118	regionalization	BP	0.599	HOXA3,GATA5,HOXB5	up_VPUL_VSMC
GO:1990774	0.001494156	0.99994	3	9	tumor necrosis factor secretion	BP	0.7009284	NR1H4,FZD5,MAVS	up_ACOR_EC
GO:0051716	0.001497654	0.99911	72	2001	cellular response to stimulus	BP	0.7009284	SH3TC2,CDH3,NR1H4,BRSK1,PLCL1,CALY,SOX9,RHOU,ARHGAP6,PTH1R,RGS1,NDRG2,AQP1,PORCN,ENSCAFG00000004331,KCNB1,ADAMTS13,EDEM2,FZD5,CCDC3,AGRN,PDE1A,IFI6,GNAO1,DUSP26,DIXDC1,RASL11A,PTGIR,NOD2,RGMA,PDE4A,INSYN1,SMAD6,PLCE1,ACTN2,ARHGAP28,ESM1,PAK3,MMP28,GUCY1B1,GRK7,TNFRSF19,PTGER4,ARHGDIB,ELMO2,DCLK2,ADAMTSL2,MAPK8IP2,RAPGEF4,MAVS,FOXC2,FIGNL1,LRRC15,IFIT2,LRP4,CLSTN1,SH3RF3,LRRC4,CLU,C23H3orf58,RARRES2,PRKACA,STK32C,NRP1,HM13,ARHGEF26,PRKCD,GNAZ,RIN2,PYGO2,MFAP4,ADGRL1	up_ACOR_EC
GO:0051094	0.0015	1	4	388	positive regulation of developmental process	BP	0.69	SLITRK5,LIMK1,GATA5,C6	up_VCAV_VSMC
GO:0048598	0.0015	1	3	207	embryonic morphogenesis	BP	0.504	HOXC10,HOXA9,WNT7A	up_AORTRT_VSMC
GO:0001659	0.0015	1	3	54	temperature homeostasis	BP	0.44	GATM,HOXC10,CAV1	up_VSAP_VSMC
GO:0032231	0.001503254	0.99982	5	32	regulation of actin filament bundle assembly	BP	0.7009284	ARHGAP6,SYNPO2,ARHGAP28,PTGER4,NRP1	up_ACOR_EC
GO:0002460	0.00151	1	13	61	adaptive immune response based on somatic recombination of immune receptors built from immunoglobulin	BP	0.123	IL7R,IL6,TNFAIP3,MEF2C,GATA3,RAB27A,NECTIN2,MTOR,THOC1,SWAP70,JAG1,BCL10,HMGB1	up_VCAV_EC
GO:0048729	0.00151	1	5	209	tissue morphogenesis	BP	0.29	EYA1,HOXB7,SIX1,MTSS1,CA9	up_VFEM_EC
GO:0060350	0.00151	1	2	23	endochondral bone morphogenesis	BP	0.595	HOXA11,SHOX2	up_VFEM_VSMC
GO:0055074	0.00152	1	3	95	calcium ion homeostasis	BP	1	HOXA3,TRPC4,C3AR1	up_VPOR_VSMC
GO:0032502	0.00153	1	9	1661	developmental process	BP	0.599	HOXA3,TBX5,GATA5,HOXA2,TMEM176A,TMEM176B,PHEX,HOXB5,SLIT2	up_VPUL_VSMC
GO:0055081	0.00154	1	2	17	anion homeostasis	BP	1	HOXA3,NR1H4	up_VPUL_EC
GO:0003164	0.00154	1	1	2	His-Purkinje system development	BP	1	TBX5	up_APUL_VSMC
GO:0003166	0.00154	1	1	2	bundle of His development	BP	1	TBX5	up_APUL_VSMC
GO:0071702	0.00155	0.999	60	687	organic substance transport	BP	0.21	PRICKLE1,MYH9,NR1H4,PLK3,HM13,TRAM1,PTPN14,SLC7A5,ENPP1,SEC24D,PTPN11,SEC23A,SLC38A1,TRIB3,SESN2,SRP54,MYO1C,ENSCAFG00000031588,SEL1L,HIF1A,SSR1,IWS1,SLC2A1,OSBPL9,ZPR1,STAM2,YES1,SMG7,XPOT,USO1,SRPRA,DDX39B,COPG1,KPNB1,COPB2,SRP72,TFRC,APOE,KPNA4,LYN,SLC2A3,OSBPL6,SLC7A11,GSK3B,GBP5,KPNA1,REST,SCAMP1,RAB11FIP1,KDELR2,AP3S1,CLTC,INHBA,STX6,ESYT3,CDKN1A,RHBDF2,YWHAH,BLZF1,SEC13	down_VSAP_EC
GO:0009888	0.00155	1	6	545	tissue development	BP	1	HOXA3,BARX1,NKX2-5,BAMBI,ANKRD6,CYP26B1	up_VPOR_VSMC
GO:0034453	0.00155	1	2	8	microtubule anchoring	BP	1	HOOK3,GCC2	up_VPOR_EC
GO:0010159	0.00155	1	1	2	specification of animal organ position	BP	1	HOXA3	up_APUL_VSMC
GO:1901699	0.001554842	0.99953	12	164	cellular response to nitrogen compound	BP	0.7009284	NR1H4,SOX9,AQP1,ENSCAFG00000004331,AGRN,NOD2,ACTN2,GUCY1B1,MAVS,FOXC2,RARRES2,PRKCD	up_ACOR_EC

GO:0015031	0.00156	0.999	47	510	protein transport	BP	0.21	PRICKLE1,MYH9,NR1H4,PLK3,HM13,TRAM1,PTPN14,SEC24D,PTPN11,SEC23A,SRP54,MYO1C,ENSCAFG00000031588,SEL1L,HIF1A,SSR1,IWS1,ZPR1,STAM2,SMG7,XPOT,USO1,SRPRA,DDX39B,COPG1,KPNB1,COPB2,SRP72,TFRC,APOE,KPNA4,LYN,GSK3B,GBP5,KPNA1,REST,SCAMP1,RAB11FIP1,KDELR2,AP3S1,CLTC,STX6,CDKN1A,RHBDF2,YWHAH,BLZF1,SEC13	down_VSAP_EC
GO:0001508	0.00156	1	8	25	action potential	BP	0.546	FGF13,KCNE3,TPC3,SCARB2,PTPN3,USP53,JUP,GNAQ	up_ACOR_VSMC
GO:0003203	0.00156	1	1	11	endocardial cushion morphogenesis	BP	1	GATA5	down_AFEM_VSMC
GO:0055022	0.00156	1	1	2	negative regulation of cardiac muscle tissue	BP	1	TBX5	up_APUL_VSMC
GO:0060044	0.00156	1	1	2	negative regulation of cardiac muscle cell	BP	1	TBX5	up_APUL_VSMC
GO:0061117	0.00156	1	1	2	negative regulation of heart growth	BP	1	TBX5	up_APUL_VSMC
GO:0099536	0.00157	1	4	133	synaptic signaling	BP	1	HTR7,PLCL1,SLITRK5,NTRK2	up_APUL_EC
GO:0060040	0.00157	1	1	1	retinal bipolar neuron differentiation	BP	0.795	IRX5	up_AFEM_VSMC
GO:0002793	0.00158	1	13	54	positive regulation of peptide secretion	BP	0.546	EXPH5,TLR1,HTR2B,MBP,FERMT1,TM7SF3,CD274,ANKRD1,F2RL1,PTPN22,PRKCE,NOD2,SCAMP5	up_ACOR_VSMC
GO:0050714	0.00158	1	13	54	positive regulation of protein secretion	BP	0.546	EXPH5,TLR1,HTR2B,MBP,FERMT1,TM7SF3,CD274,ANKRD1,F2RL1,PTPN22,PRKCE,NOD2,SCAMP5	up_ACOR_VSMC
GO:2000826	0.00158	1	2	14	regulation of heart morphogenesis	BP	0.3	EYA1,SIX1	up_VFEM_EC
GO:0032502	0.00159	1	17	1661	developmental process	BP	0.415	ACVRL1,LYN,HOXA3,ACSL4,SPG11,PDPN,NRP2,HIVEP2,HOXA7,ANPEP,SULF1,ELK3,HOXA1,CSGALNACT1,HOXC4,ASPN,NEDD4	down_ACOR_EC
GO:0030029	0.001596185	0.99944	15	239	actin filament-based process	BP	0.7009284	MYOZ2,TMOD1,ARHGAP6,AQP1,SYNPO2,DIXDC1,PDLIM3,PLS1,ACTN2,ARHGAP28,PAK3,PTGER4,ARHGDIB,NRP1,PRKCD	up_ACOR_EC
GO:0048856	0.0016	0.999	53	1533	anatomical structure development	BP	1	TBX15,HOXA9,IRX3,IRX5,HOXC10,LEP,LBH,XYLT1,ANGPT4,HOXA7,COL8A2,CD40,DLL1,CRYAB,F3,VXN,PLXNA4,NEFH,MAB21L1,THRB,LGI4,TMEM100,PLAGL2,GREM1,NFE2L1,OVOL1,CAMK2A,GREM2,HOXA6,CYLD,POSTN,LUZP1,RECK,PTHLH,TBXA2R,EVI2B,CNNM4,BVES,LGR6,PTPRB,AXIN2,ACACB,PPARGC1A,CDC42EP5,DMPK,ADAM17,CLIC4,LRRN2,HSPB2,GCNT4,FOSL2,KANK1,TNC	up_AFEM_EC
GO:1903524	0.0016	1	5	11	positive regulation of blood circulation	BP	0.546	ADRA1B,HRH1,F2RL1,HRH2,EDN1	up_ACOR_VSMC
GO:0010611	0.0016	1	1	11	regulation of cardiac muscle hypertrophy	BP	1	GATA5	down_AFEM_VSMC
GO:0014743	0.0016	1	1	11	regulation of muscle hypertrophy	BP	1	GATA5	down_AFEM_VSMC
GO:0032879	0.001603155	0.99919	34	767	regulation of localization	BP	0.7009284	SH3TC2,CDH3,NR1H4,CALY,SOX9,AQP1,ENSCAFG00000004331,KCNB1,EDEM2,FZD5,SEMA6D,NOD2,PLS1,ACTN2,ZMYND8,MMP28,TRIM8,PTGER4,ARHGDIB,PREB,DCLK2,MAPK8IP2,MAVS,FOXC2,LRRC15,TBX5,LRP4,TP53INP2,RARRES2,VPS16,NRP1,AZIN2,PRKCD,PM20D1	up_ACOR_EC
GO:0021602	0.00161	1	2	16	cranial nerve morphogenesis	BP	1	HOXA3,HOXA1	up_APUL_EC
GO:0060914	0.00161	1	1	11	heart formation	BP	1	GATA5	down_AFEM_VSMC

GO:0051239	0.00162	0.999	107	859	regulation of multicellular organismal process	BP	0.934	HOXA11,FZD1,THRB,HOXA7,TWIST2,HOXA9,CDON,WNT11,HNF4A,CD274,AXIN2,C5AR2,WASF3,OSR2,AMIGO1,ENPP2,LRRN3,PPARGC1B,LIMS2,ACACB,IL6R,IRX5,SLIT2,ZHX3,HOXD3,SLC6A4,FGF7,ABL1,EBF2,BTG1,HDAC7,KLKB1,EMP2,KCNE3,CHRM1,DLL1,EPN2,OSR1,IL17RC,SAV1,ANXA4,HPSE,PLXNA4,ANKRD54,ZBTB16,TOX,RARG,CCBE1,STAT5A,STAT6,F2RL1,DDR2,MFN2,ZFP36L1,FXN,PIK3R1,TIAM1,OMD,MYC,IRX3,ZEB1,TBXA2R,SULF2,SHOX2,RTN4RL1,SASH1,AGTR1,HOXC10,MYD88,TFE3,LRP4,PASK,DPYSL2,GDPD5,PRKAB1,FBXO7,NTN4,LCAT,LIMD1,NLRX1,TKFC,CD83,KDM2B,RNF216,ZFP36L2,PNP,MAPKBP1,NFIB,ZNF580,CYBA,CYB5D2,PQBP1,TTL,ACVRL1,HTRA2,FLOT1,NCK1,MAVS,BGLAP,HMOX1,MAP2K2,TGFBR2,CITED2,TRADD,LBH,KEAP1,LATS2	up_VSAP_EC
GO:0030001	0.00162	0.999	29	172	metal ion transport	BP	0.546	FGF13,ACTN2,PLCG2,KCNE3,HTR2B,TPC3,NIPAL3,SARAF,PTPN3,MICU2,ITPR1,CACNB3,AKAP6,DMPK,ATP7A,KCNJ4,PRKCE,PSEN1,CHD7,RAB11B,KCNC4,LMTK2,B2M,FXYD1,INPP5K,WFS1,ATP2C1,SLC8A1,STC2	up_ACOR_VSMC
GO:0043266	0.00162	1	2	14	regulation of potassium ion transport	BP	0.467	NETO1,CAV1	up_VSAP_VSMC
GO:0044092	0.00163	1	6	270	negative regulation of molecular function	BP	0.416	LYN,IGF1R,PRKAR2A,MYO1D,ASPN,NEDD4	down_ACOR_EC
GO:0008015	0.00165	0.999	20	120	blood circulation	BP	0.133	EDNRB,PRCP,DOCK4,FLI1,PDGFB,CD34,PDE4D,CHD7,STC1,EDN1,PTGS1,GUCY1A1,MTOR,NAV2,KCNJ2,PTGS2,GCH1,NOS2,SPTBN4,P2RX2	up_VCAV_EC
GO:0050818	0.00165	1	7	24	regulation of coagulation	BP	0.133	SELP,PDGFB,CD34,KLKB1,TLR4,TFPI,PROCR	up_VCAV_EC
GO:0045595	0.00165	1	5	469	regulation of cell differentiation	BP	0.599	TBX5,GATA5,TMEM176A,TMEM176B,SLIT2	up_VPUL_VSMC
GO:0001658	0.00165	1	2	24	branching involved in ureteric bud	BP	0.628	EYA1,HOXA11	up_VFEM_VSMC
GO:0006616	0.00166	1	3	5	SRP-dependent cotranslational protein targeting to membrane,	BP	0.221	TRAM1,SRP54,SRP72	down_VSAP_EC
GO:0023051	0.00167	0.999	79	954	regulation of signaling	BP	0.221	ARHGEF2,PRICKLE1,VEGFA,P4HB,SIPA1L1,SULF1,NR1H4,HSPA5,SERPINE1,SOCS5,PRKACB,ENPP1,ERRFI1,JCAD,SNX25,HYOU1,PTPN11,TRIB3,FARP1,ILK,FHL2,PRCP,SMARCA4,SESN2,PTGS2,MYO1C,CHSY1,UACA,KIF5B,HIF1A,ENSCAFG00000000562,TNIK,NXN,RGS2,PTPN2,NEO1,PLCL1,MTDH,ESM1,ADAM17,CNOT7,CDH11,MAGED1,CTNNB1,ENSCAFG00000002015,APOE,WWC3,UBA5,LYN,BNIP3,STRAP,DDIT3,FAM162A,PLEKHG2,CBLC,ATF3,GSK3B,CDC37,SH3GLB1,TAX1BP3,HTT,REST,RDH11,ARFGEF3,CALM1,RAB11FIP1,DISC1,PAK2,LRRTM2,INHBA,HIF1AN,LMO7,NRP1,SH3RF3,RHBDF2,TNFAIP1,MADD,SEC13,MID1	down_VSAP_EC
GO:1901992	0.00168	1	7	23	positive regulation of mitotic cell cycle phase	BP	0.105	BIRC5,CDK1,CDK4,DTL,ENSCAFG00000005299,UBE2C,MTBP	down_ACOR_VSMC
GO:0009185	0.00169	1	6	23	ribonucleoside diphosphate metabolic	BP	0.221	PGK1,TPI1,PGAM1,HIF1A,ENTPD4,ALDOC	down_VSAP_EC
GO:0060842	0.00169	1	2	3	arterial endothelial cell differentiation	BP	1	DLL1,TMEM100	up_AFEM_EC
GO:0086065	0.00169	1	1	6	cell communication involved in cardiac	BP	0.829	JUP	down_VFEM_VSMC
GO:0006811	0.0017	0.999	48	330	ion transport	BP	0.546	FGF13,ACTN2,PLCG2,LEP,SLC26A7,KCNE3,HTR2B,TCAF1,TPC3,ABCA1,GJA1,NIPAL3,SARAF,PTPN3,CLCN3,MICU2,ITPR1,DAPK1,SLC17A5,CACNB3,AKAP6,LRRC8C,DMPK,ATP7A,SCP2,KCNJ4,SLCO1A2,PRKCE,PSEN1,CHD7,CLN8,ATP6V0E1,RAB11B,SLC25A20,KCNC4,LMTK2,B2M,PLIN2,FXYD1,CFI,INPP5K,WFS1,ATP2C1,SLC25A12,NDUFA4L2,PARK7,SLC8A1,STC2	up_ACOR_VSMC
GO:0009887	0.0017	1	6	314	animal organ morphogenesis	BP	0.307	TBX15,EYA1,HOXB7,HOXA7,SIX1,CYP26B1	up_VFEM_EC
GO:0048754	0.0017	1	3	57	branching morphogenesis of an epithelial tube	BP	0.307	EYA1,HOXB7,SIX1	up_VFEM_EC
GO:0003274	0.0017	1	1	2	endocardial cushion fusion	BP	1	GATA5	down_VSAP_VSMC
GO:0001776	0.00171	1	7	23	leukocyte homeostasis	BP	0.137	TNFAIP3,MEF2C,ITPKB,BCL10,HMGB1,SKIL,HIF1A	up_VCAV_EC

GO:0090184	0.00171	1	2	14	positive regulation of kidney development	BP	0.307	HOXB7,SIX1	up_VFEM_EC
GO:1902106	0.00171	1	2	31	negative regulation of leukocyte differentiation	BP	0.599	TMEM176A,TMEM176B	up_VPUL_VSMC
GO:0033554	0.00172	0.999	82	708	cellular response to stress	BP	0.107	HMGB1,SREBF1,USP1,MCM7,RPA1,DDX39A,CBX5,HMGA1,SREBF2,SMC1A,HINFP,NEDD4,WDR76,INTS7,MNDA,POLA1,TRIP13,PPP1R15B,PARPBP,GINS4,RAD54L,POLE,HM13,UBE2T,RFC2,INSIG1,CAV1,CDK1,RFC5,RFC3,RAD51,CASP2,FANCI,FANCD2,TIMELESS,TOPBP1,EZH2,MAP3K20,RFC4,FEN1,MND1,RNASEH2B,PLK3,PCNA,TONSL,ARG2,MCM10,RNF152,BRCA1,PPARD,POLH,NEIL3,UBE2J2,DTL,SUV39H1,RSL1D1,DMAP1,SMARCB1,DDX39B,ENSCAFG00000030087,RBBP8,EZR,CLSPN,SMC6,RBM14,MIOS,RBM17,RAD23A,SIGMAR1,MELK,SMARCAL1,TXNDC12,PSMC1,AIFM1,ARHGEF2,H2AFX,CDC5L,ATRIP,NUDT1,HMGN1,APEX1,GEN1	down_ACOR_VSMC
GO:0007010	0.00172	0.999	43	441	cytoskeleton organization	BP	0.221	ARHGEF2,SIPA1L1,MYH9,FMNL3,PLK3,ACTN1,VASP,TPM4,CORO1C,FSCN1,FLNB,MZT1,DPYSL3,HOOK3,PPP1R12A,WDR1,TUBB,TNIK,ZPR1,TMSB10,ACTR3,PALLD,CTNNB1,KPNB1,TWF1,PLEKHG2,TLN1,PLS3,GSK3B,NEXN,DAAM1,CCSER2,HTT,ENSCAFG00000017326,DISC1,PAK2,CAPG,SIX4,NRP1,ENSCAFG00000030508,TNFAIP1,MYBPC2,MID1	down_VSAP_EC
GO:0003205	0.00172	0.999	15	67	cardiac chamber development	BP	0.546	TNNT2,SMAD6,GATA4,BMP4,FGFR2,CITED2,LMO4,EGLN1,NRP1,SMARCD3,TBX20,TBX5,FRS2,CHD7,AP2B1	up_ACOR_VSMC
GO:2000106	0.00172	1	8	29	regulation of leukocyte apoptotic process	BP	0.137	HCLS1,IL7R,MEF2C,ARG2,ITPKB,RAPGEF2,BCL10,HIF1A	up_VCAV_EC
GO:0051093	0.00172	1	4	273	negative regulation of developmental process	BP	0.599	TBX5,TMEM176A,TMEM176B,SLIT2	up_VPUL_VSMC
GO:0007214	0.00172	1	1	4	gamma-aminobutyric acid signaling pathway	BP	1	PLCL1	down_VFEM_EC
GO:0018193	0.00173	0.999	37	368	peptidyl-amino acid modification	BP	0.221	ARHGEF2,VEGFA,P4HB,PLOD2,NR1H4,SOCS5,SGK1,ERRFI1,P4HA1,BCAR3,ILK,PTGS2,PPIB,IWS1,GALNT1,NAA10,PTPN2,MORF4L2,YES1,PLCL1,ADAM17,CNOT7,PLOD3,LYN,ASPH,STT3A,CBLC,GSK3B,PRMT1,P3H4,PAK2,CAMK2D,HIF1AN,BTK,NRP1,RPN1,KDM3A	down_VSAP_EC
GO:0002443	0.00173	0.999	14	71	leukocyte mediated immunity	BP	0.138	IL7R,KIT,TLR4,GATA3,RAB27A,NECTIN2,SNAP23,THOC1,SWAP70,TLR3,FES,JAG1,BCL10,HMGB1	up_VCAV_EC
GO:0050730	0.00173	1	11	66	regulation of peptidyl-tyrosine phosphorylation	BP	0.221	ARHGEF2,VEGFA,SOCS5,ERRFI1,PTPN2,YES1,ADAM17,CNOT7,LYN,CBLC,NRP1	down_VSAP_EC
GO:1905314	0.00173	1	1	12	semi-lunar valve development	BP	1	GATA5	down_AFEM_VSMC
GO:0140074	0.00173	1	1	2	cardiac endothelial to mesenchymal transition	BP	1	GATA5	down_VSAP_VSMC
GO:2000026	0.00174	1	8	560	regulation of multicellular organismal development	BP	0.308	EYA1,HOXA9,TOX,HOXB7,HOXA7,SIX1,TIAM1,CYP26B1	up_VFEM_EC
GO:0009566	0.00174	1	2	24	fertilization	BP	0.628	HOXA9,HOXA11	up_VFEM_VSMC
GO:0021953	0.00175	1	2	49	central nervous system neuron differentiation	BP	0.504	HOXC10,WNT7A	up_AORTRT_VSMC
GO:0050793	0.00176	0.999	93	731	regulation of developmental process	BP	0.98	HOXA11,FZD1,THRB,HOXA7,NAPEPLD,TWIST2,HOXA9,CDON,WNT11,HNF4A,CD274,AXIN2,WASF3,OSR2,AMIGO1,ENPP2,LRRN3,PPARGC1B,LIMS2,ACACB,DDHD1,SLIT2,ZHX3,HOXD3,SLC6A4,FGF7,ABL1,BTG1,HDAC7,EMP2,DLL1,EPN2,OSR1,MAP3K5,CDC42EP5,SAV1,HPSE,PLXNA4,ANKRD54,CDC42EP2,ZBTB16,TOX,RARG,CCBE1,STAT6,DDR2,ZFP36L1,FXN,PIK3R1,TIAM1,OMD,MYC,IRX3,ZEB1,TBXA2R,SULF2,SHOX2,RTN4RL1,SASH1,TFE3,LRP4,DPYSL2,GDPD5,ZADH2,FBXO7,NTN4,LIMD1,CDK9,CD83,KDM2B,FBLN1,ZFP36L2,PLEKHB2,YPEL3,PNP,TAF8,NFIB,CYB5D2,PQBP1,TTL,ACVRL1,HTRA2,FLOT1,NCK1,BGLAP,HMOX1,MAP2K2,TGFBR2,CITED2,TRADD,LBH,KEAP1,LATS2	up_VSAP_EC
GO:0016125	0.00176	1	10	41	sterol metabolic process	BP	0.109	SQLE,LSS,SREBF1,HMGCR,INSIG1,ENSCAFG00000018572,DHCR24,MVD,FDFT1,LIPC	down_ACOR_VSMC
GO:0048870	0.00176	1	8	451	cell motility	BP	0.438	ACVRL1,LYN,IGF1R,PDPN,NRP2,HOXA7,SULF1,STX4	down_ACOR_EC
GO:0051674	0.00176	1	8	451	localization of cell	BP	0.438	ACVRL1,LYN,IGF1R,PDPN,NRP2,HOXA7,SULF1,STX4	down_ACOR_EC

GO:0048513	0.00176	1	5	906	animal organ development	BP	0.504	HOXC10,HOXA9,WNT7A,ARG2,ESR1	up_AORTRT_VSMC
GO:0001707	0.00176	1	2	25	mesoderm formation	BP	0.628	EYA1,HOXA11	up_VFEM_VSMC
GO:0072001	0.00177	1	3	104	renal system development	BP	0.628	EYA1,EMX2,HOXA11	up_VFEM_VSMC
GO:0048762	0.00177	1	2	77	mesenchymal cell differentiation	BP	1	GATA5,TGFBR1	down_VSAP_VSMC
GO:1902903	0.001779217	0.99953	10	125	regulation of supramolecular fiber	BP	0.7009284	TMOD1,ARHGAP6,SYNPO2,ACTN2,ARHGAP28,PAK3,PTGER4,NRP1,PRKCD,TUBB4A	up_ACOR_EC
GO:0010811	0.00178	1	10	36	positive regulation of cell-substrate adhesion	BP	0.546	FERMT1,NRP1,NPNT,PRKCE,ECM2,JUP,NID1,DOCK5,RREB1,CRKL	up_ACOR_VSMC
GO:0032502	0.00179	0.999	15	1661	developmental process	BP	0.499	HOXA11,TBX15,HOXA9,PITX1,EMX2,GATM,SALL1,HOXC10,IRX5,MAB21L1,STIM1,CAV1,SHOX2,HAPLN1,GJB5	up_VSAP_VSMC
GO:0010508	0.00179	1	8	38	positive regulation of autophagy	BP	0.223	PLK3,SESN2,HIF1A,MTDH,BNIP3,CDC37,SH3GLB1,HTT	down_VSAP_EC
GO:0032835	0.00179	1	7	21	glomerulus development	BP	0.141	PDGFB,PODXL,MEF2C,GPR4,MTSS1,JAG1,BASP1	up_VCAV_EC
GO:0009653	0.00179	1	7	773	anatomical structure morphogenesis	BP	1	CHL1,HOXA3,NKX2-5,BAMBI,ANKRD6,CYP26B1,C3AR1	up_VPOR_VSMC
GO:0019471	0.00179	1	4	7	4-hydroxyproline metabolic process	BP	0.546	PRDX4,ERO1B,EGLN1,P4HA1	up_ACOR_VSMC
GO:0003002	0.00179	1	4	118	regionalization	BP	0.499	HOXA11,HOXA9,EMX2,HOXC10	up_VSAP_VSMC
GO:0061035	0.00179	1	2	24	regulation of cartilage development	BP	0.628	HOXA11,SHOX2	up_VFEM_VSMC
GO:0007586	0.001797952	0.99985	4	20	digestion	BP	0.7009284	SOX9,AQP1,NOD2,PLS1	up_ACOR_EC
GO:0022600	0.001797952	0.99985	4	20	digestive system process	BP	0.7009284	SOX9,AQP1,NOD2,PLS1	up_ACOR_EC
GO:0016043	0.0018	0.999	197	1973	cellular component organization	BP	0.11	ACSL3,FSCN1,HMGB1,MCM6,SREBF1,MCM4,FLNB,POLE3,IRAK1,MCM7,RPA1,NCAPD2,ANLN,STXBP6,USP4,FMNL3,ATAD2,HMGA1,HMGCR,EDC3,HNRNPD,SMC1A,UBE2S,NEDD4,RRM1,ZW10,ENSCAFG00000016482,CAPG,SMC4,CENPA,SLC29A1,ENSCAFG00000017264,NDC1,CENPN,TOP3A,TRIP13,COLGALT1,SLC25A5,TIMM44,SF1,SGO1,GINS4,C1QTNF6,SDC1,ARFGAP1,CTDNEP1,FBXO5,HSPA9,RAN,B3GNT2,CFAP298,PRELID1,PSMD13,BIRC5,INSIG1,PRMT1,CAV1,CDK1,RAD51,LIMA1,NF2,AURKB,MMP14,TK1,CDKN2C,DSN1,EZH2,STMN1,CENPO,HAUS8,GREM1,NCAPD3,MAP3K20,NEK2,CHCHD3,FBL,FEN1,SMARCC1,MND1,GAS2L3,PLK3,HMGB3,RCC1,DHX33,KIF22,UHRF1,CKS2,GMNN,CDCA8,CENPH,DHCR24,NCAPH,POC1A,BRCA1,WDR62,CENPI,FASTKD3,NOC2L,EIF5A,B4GALT1,SMC2,FARSA,PREB,ESPL1,SPC25,CDC6,NDC80,OIP5,IPO4,HIST1H1A,RAC1,NUP62,EMP2,DDX28,PDXP,FOSL1,PLK4,IQGAP1,SUV39H1,HASPIN,TOMM34,IQGAP2,SAMM50,REEP4,HMGB2,DMAP1,CDC42EP4,HIST1H2BB,SMC3,CDK2,SMARCB1,DDX39B,CCNA2,RAB35,STAG1,ENSCAFG00000005299,EZR,SMC6,SF3A2,SASS6,BUB1,RBM14,CORO1A,NOLC1,CENPW,FOXRED1,PHACTR4,CDC20,	down_ACOR_VSMC
GO:0010648	0.0018	0.999	40	407	negative regulation of cell communication	BP	0.223	ARHGEF2,PRICKLE1,SULF1,NR1H4,HSPA5,SERPINE1,SOCS5,PRKACB,ENPP1,ERRFI1,SNX25,HYOU1,PTPN11,FHL2,SMARCA4,SESN2,PTGS2,HIF1A,NXN,RGS2,PTPN2,ADAM17,CNOT7,CTNNB1,APOE,WWC3,LYN,STRAP,DDIT3,CBLC,ATF3,GSK3B,TAX1BP3,HTT,REST,CALM1,RAB11FIP1,HIF1AN,NRP1,TNFAIP1	down_VSAP_EC
GO:0048534	0.0018	0.999	36	262	hematopoietic or lymphoid organ development	BP	0.141	HOXB7,HCLS1,IL7R,ADGRF5,KIT,IL6,FLI1,MEF2C,CHD7,TIPARP,DLL4,GATA3,HOXB8,ENSCAFG00000003530,IRF8,CASP8,MTOR,TENT2,HHEX,ITPKB,MFNG,FES,JAG1,PLCG2,CREB1,HMGB1,RIPK1,CRIP2,ATP11C,CUL4A,BMX,HIF1A,TMOD3,ZEB1,TET2,TJP2	up_VCAV_EC
GO:0045197	0.0018	1	4	10	establishment or maintenance of epithelial cell apical/basal polarity	BP	0.223	ILK,WDR1,MSN,ARF4	down_VSAP_EC
GO:0048705	0.0018	1	2	95	skeletal system morphogenesis	BP	1	HOXA3,HOXB5	up_APUL_VSMC
GO:0003128	0.0018	1	1	6	heart field specification	BP	0.829	GATA5	down_VFEM_VSMC
GO:0019359	0.00181	1	6	23	nicotinamide nucleotide biosynthetic process	BP	0.223	PGK1,TPI1,PGAM1,HIF1A,NMNAT2,ALDOC	down_VSAP_EC

GO:0019363	0.00181	1	6	23	pyridine nucleotide biosynthetic process	BP	0.223	PGK1,TPI1,PGAM1,HIF1A,NMNAT2,ALDOC	down_VSAP_EC
GO:0001775	0.00181	1	3	219	cell activation	BP	0.504	HOXA9,WNT7A,ARG2	up_AORTRT_VSMC
GO:0061437	0.001812027	0.99992	3	10	renal system vasculature development	BP	0.7009284	AQP1,FOXC2,NRP1	up_ACOR_EC
GO:0061440	0.001812027	0.99992	3	10	kidney vasculature development	BP	0.7009284	AQP1,FOXC2,NRP1	up_ACOR_EC
GO:0003013	0.00182	0.999	20	121	circulatory system process	BP	0.142	EDNRB,PRCP,DOCK4,FLI1,PDGFB,CD34,PDE4D,CHD7,STC1,EDN1,PTGS1,GUCY1A1,MTOR,NAV2,KCNJ2,PTGS2,GCH1,NOS2,SPTBN4,P2RX2	up_VCAV_EC
GO:0046642	0.00182	1	1	2	negative regulation of alpha-beta T cell	BP	0.504	ARG2	up_AORTRT_VSMC
GO:1905397	0.00182	1	1	2	activated CD8-positive, alpha-beta T cell	BP	0.504	ARG2	up_AORTRT_VSMC
GO:1905402	0.00182	1	1	2	regulation of activated CD8-positive, alpha-beta T cell apoptotic process	BP	0.504	ARG2	up_AORTRT_VSMC
GO:2000562	0.00182	1	1	2	negative regulation of CD4-positive, alpha-beta T cell proliferation	BP	0.504	ARG2	up_AORTRT_VSMC
GO:0051017	0.001821495	0.99971	6	49	actin filament bundle assembly	BP	0.7009284	ARHGAP6,SYNPO2,PLS1,ARHGAP28,PTGER4,NRP1	up_ACOR_EC
GO:0045595	0.00183	0.999	57	469	regulation of cell differentiation	BP	0.142	EPHB1,EDNRB,HCLS1,IL7R,KIT,PRKCH,IFRD1,ADAMTS9,PPARG,PDGFB,BCL6B,RELN,CD34,MEF2C,CHD7,DLL4,RAPGEF1,GATA3,EDN1,HOXB8,RAB11A,TCF4,NEDD9,PPP3CA,CASP8,MTOR,HOOK3,BMP2,HMG20A,ITPKB,GDPD5,PROX1,ENSCAFG00000010596,NCK1,FES,JAG1,PTGS2,MMD,RAPGEF2,CREB1,ZC4H2,CTNNA1,HMGB1,RIPK1,ATP11C,SKIL,CUL4A,NR1D2,HIF1A,KDM3A,ENSCAFG00000002015,ZEB1,ARHGEF2,FBXO5,CDON,TJP2,ZNF609	up_VCAV_EC
GO:0032956	0.00183	0.999	20	116	regulation of actin cytoskeleton organization	BP	0.142	TEK,HCLS1,ARHGEF15,BAIAP2L1,MEF2C,EDN1,ARHGAP28,TMOD1,MTOR,NCKAP1,PROX1,IQGAP2,STMN1,SWAP70,NCK1,FES,ARPC5L,ARHGDIB,TMOD3,FSCN1	up_VCAV_EC
GO:0002683	0.00183	1	3	126	negative regulation of immune system process	BP	0.599	TMEM176A,TMEM176B,SLIT2	up_VPUL_VSMC
GO:0000165	0.00184	0.999	37	236	MAPK cascade	BP	0.551	FGF13,ADRA1B,LEP,MID1,HTR2B,RASSF2,MBP,BMP4,FGFR2,TLR3,SH3RF2,PDE5A,SASH1,FGF18,NDRG2,F2RL1,NRP1,PTPN22,NPNT,SMPD1,SH3RF1,PRKCE,FRS2,DAG1,APP,PSEN1,SYNJ2BP,CCL5,DUSP22,EDN1,DUSP26,NOD2,SH3RF3,MAP3K7,INPP5K,ENSCAFG00000029553,CRKL	up_ACOR_VSMC
GO:0048332	0.00184	1	2	26	mesoderm morphogenesis	BP	0.63	EYA1,HOXA11	up_VFEM_VSMC
GO:0001759	0.00185	1	1	13	organ induction	BP	1	GATA5	down_AFEM_VSMC
GO:0032880	0.001851663	0.99932	16	266	regulation of protein localization	BP	0.7009284	SH3TC2,NR1H4,KCNB1,EDEM2,FZD5,NOD2,PLS1,ACTN2,TRIM8,PTGER4,DCLK2,MAVS,LRRC15,LRP4,TP53INP2,PRKCD	up_ACOR_EC
GO:0055081	0.00187	1	2	17	anion homeostasis	BP	1	NR1H4,HOXA3	up_APUL_EC
GO:0043062	0.00188	1	4	99	extracellular structure organization	BP	0.446	PDPN,SULF1,CSGALNACT1,ACSL3	down_ACOR_EC
GO:0045668	0.00188	1	3	11	negative regulation of osteoblast differentiation	BP	1	TWIST2,GREM1,AXIN2	up_AFEM_EC
GO:0001953	0.00188	1	2	14	negative regulation of cell-matrix adhesion	BP	0.446	ACVRL1,HOXA7	down_ACOR_EC

GO:0050896	0.00189	0.999	261	2346	response to stimulus	BP	0.555	TNNT2,SH3TC2,FGF13,RHOT1,ADRA1B,VEGFD,SEMA5A,TLR1,SMAD6,ATRNL1,ARHGAP28,ACTN2,LGR6,PLCG2,IFNGR1,LEP,PLCL1,LBH,DCBLD2,AFF3,MAP3K8,MID1,GATA5,GATA4,ADGRD1,CD83,EFNB2,CDC7,ANGPT1,ENSCAFG00000016285,HTR2B,RASSF2,FNDC5,MBP,TMEM255A,ZRANB1,FBXL20,C28H10orf71,RECK,FEZ1,SKP2,NACC2,COCH,PRKAA2,MLLT3,ITM2C,FERMT1,BMP4,LNPK,TM7SF3,FGFR2,TP53BP1,MX1,MFAP4,ABCA1,EIF4A2,GJA1,DOCK3,ELF1,ATRX,MED14,HRH1,CASTOR1,TLR3,LRRN4,SH3RF2,PPARGC1A,LATS2,LPL,PDE5A,CITED2,TP53INP1,RERG,GLRB,ARHGAP29,ZBTB40,ISG15,ZNRF3,CD274,ZNF365,PLPP2,AR,PTGIR,SASH1,CTSS,SLC2A5,GPR155,TMEM108,FGF18,FAM210B,ARHGEF26,NDRG2,TMX3,CHN2,CDKN2B,ADCY6,ZBTB4,USP53,POLK,TINAGL1,ITPR1,GRK7,PLAC8B,KLHL20,LIMS2,BLNK,ANKRD1,F2RL1,MXRA5,HRH2,ADGRL1,FAM129A,CAMK2D,PSEN2,ENPP4,PPP2R5E,GABARAPL1,DAPK1,CTSB,ARHGAP5,RBM4B,BNIP3,EGLN1,SLC17A5,NCEH1,AKAP6,CDKN1A,JMY,C1S,GNA14,P2RX7,CRLF3,EDA,TNFSF10,CLSTN1,LRRC8C,CREBRF,NRP1,UBR1,PDE1A,PTPN22,GPM6A,CHD2,ULK2,DMPK,TCN2,NPNT,OS9,UNC5B,STT3B,EVL,SMPD1,RCAN2,APL	up_ACOR_VSMC
GO:0001894	0.001897603	0.99969	6	49	tissue homeostasis	BP	0.7009284	CDH3,SOX9,PTH1R,NOD2,CHMP4B,TP53INP2	up_ACOR_EC
GO:0009719	0.0019	1	6	395	response to endogenous stimulus	BP	1	NR1H4,PTGER2,FLRT3,ARHGEF2,NEO1,MXRA5	up_VPUL_EC
GO:0035107	0.0019	1	3	60	appendage morphogenesis	BP	0.33	HOXC10,HOXA9,CYP26B1	up_VFEM_EC
GO:0035108	0.0019	1	3	60	limb morphogenesis	BP	0.33	HOXC10,HOXA9,CYP26B1	up_VFEM_EC
GO:0098901	0.0019	1	1	7	regulation of cardiac muscle cell action	BP	0.829	JUP	down_VFEM_VSMC
GO:2000026	0.00191	0.999	74	560	regulation of multicellular organismal development	BP	1	HOXA11,FZD1,THRB,HOXA7,HOXA9,CDON,WNT11,HNF4A,CD274,AXIN2,WASF3,OSR2,AMIGO1,ENPP2,LRRN3,PPARGC1B,LIMS2,ACACB,SLIT2,HOXD3,FGF7,ABL1,BTG1,HDAC7,EMP2,DLL1,EPN2,OSR1,SAV1,HPSE,PLXNA4,ANKRD54,ZBTB16,TOX,RARG,CCBE1,STAT6,DDR2,ZFP36L1,PIK3R1,TIAM1,OMD,MYC,IRX3,ZEB1,TBXA2R,SULF2,SHOX2,RTN4RL1,SASH1,TFE3,LRP4,DPYSL2,GDPD5,FBXO7,NTN4,CD83,ZFP36L2,PNP,NFIB,CYB5D2,PQBP1,TTL,ACVRL1,FLOT1,NCK1,BGLAP,HMOX1,MAP2K2,TGFBR2,CITED2,TRADD,LBH,KEAP1	up_VSAP_EC
GO:0042059	0.00191	1	4	10	negative regulation of epidermal growth factor receptor signaling	BP	0.23	SOCS5,ERRFI1,PTPN2,CBLC	down_VSAP_EC
GO:0048870	0.00192	0.999	43	451	cell motility	BP	0.23	ARHGEF2,VEGFA,SULF1,MYH9,FMNL3,HSPA5,SERPINE1,SPARC,ITGA2,JCAD,PTPN11,FSCN1,DPYSL3,ILK,PRCP,WDR1,MYO1C,PIK3C2A,MSN,HIF1A,CX3CL1,SDC3,ADAM17,PALLD,CTNNB1,ANLN,STARD13,APOE,PTP4A1,MMP28,LYN,STRAP,PDS5B,CCNYL1,NEXN,SDC4,ZMYND8,DISC1,PAK2,SIX4,NRP1,ARF4,TNFAIP1	down_VSAP_EC
GO:0051674	0.00192	0.999	43	451	localization of cell	BP	0.23	ARHGEF2,VEGFA,SULF1,MYH9,FMNL3,HSPA5,SERPINE1,SPARC,ITGA2,JCAD,PTPN11,FSCN1,DPYSL3,ILK,PRCP,WDR1,MYO1C,PIK3C2A,MSN,HIF1A,CX3CL1,SDC3,ADAM17,PALLD,CTNNB1,ANLN,STARD13,APOE,PTP4A1,MMP28,LYN,STRAP,PDS5B,CCNYL1,NEXN,SDC4,ZMYND8,DISC1,PAK2,SIX4,NRP1,ARF4,TNFAIP1	down_VSAP_EC
GO:0006694	0.00192	1	10	42	steroid biosynthetic process	BP	0.117	SQLE,LSS,SREBF1,HMGCR,SF1,INSIG1,ENSCAFG00000018572,DHCR24,MVD,FDFT1	down_ACOR_VSMC
GO:0072507	0.00192	1	3	103	divalent inorganic cation homeostasis	BP	1	HOXA3,TRPC4,C3AR1	up_VPOR_VSMC
GO:0007155	0.00193	0.999	34	324	cell adhesion	BP	0.23	COL15A1,VEGFA,SULF1,MYH9,SERPINE1,ACTN1,SOCS5,ITGA2,FAM49B,NUAK1,PTPN11,FBLIM1,ILK,PPP1R12A,MSN,THBS2,PTPN2,PVR,ADAM17,CDH11,CTNNB1,ENSCAFG00000002015,TFRC,IGFBP7,LYN,CD44,TLN1,SLC7A11,GSK3B,ITGB3,SDC4,ICAM5,LMO7,NRP1	down_VSAP_EC

GO:0051960	0.00193	0.999	32	216	regulation of nervous system development	BP	0.147	EPHB1,EDNRB,PRKCH,ARHGEF15,IFRD1,RELN,MEF2C,CHD7,DLL4,RAPGEF1,GATA3,RAB11A,TCF4,PPP3CA,MTOR,HOOK3,BMP2,HMG20A,GDPD5,PROX1,NCK1,FES,JAG1,MMD,RAPGEF2,ZC4H2,CTNNA1,HIF1A,ZEB1,ARHGEF2,CDON,ZNF609	up_VCAV_EC
GO:0022603	0.00193	1	2	301	regulation of anatomical structure morphogenesis	BP	0.829	JUP,GATA5	down_VFEM_VSMC
GO:0003180	0.00193	1	1	7	aortic valve morphogenesis	BP	0.829	GATA5	down_VFEM_VSMC
GO:0086002	0.00193	1	1	7	cardiac muscle cell action potential involved in	BP	0.829	JUP	down_VFEM_VSMC
GO:0010646	0.00194	0.999	78	945	regulation of cell communication	BP	0.23	ARHGEF2,PRICKLE1,VEGFA,P4HB,SIPA1L1,SULF1,NR1H4,HSPA5,SERPINE1,SOCS5,PRKACB,ENPP1,ERRFI1,JCAD,SNX25,HYOU1,PTPN11,TRIB3,FARP1,ILK,FHL2,PRCP,SMARCA4,SESN2,PTGS2,MYO1C,CHSY1,UACA,KIF5B,HIF1A,ENSCAFG00000000562,TNIK,NXN,RGS2,PTPN2,NEO1,PLCL1,MTDH,ESM1,ADAM17,CNOT7,CDH11,MAGED1,CTNNB1,ENSCAFG00000002015,APOE,WWC3,UBA5,LYN,BNIP3,STRAP,DDIT3,FAM162A,PLEKHG2,CBLC,ATF3,GSK3B,CDC37,SH3GLB1,TAX1BP3,HTT,REST,RDH11,ARFGEF3,CALM1,RAB11FIP1,DISC1,PAK2,LRRTM2,INHBA,HIF1AN,NRP1,SH3RF3,RHBDF2,TNFAIP1,MADD,SEC13,MID1	down_VSAP_EC
GO:0003002	0.00194	1	9	118	regionalization	BP	1	HOXA9,IRX3,HOXC10,HOXA7,DLL1,GREM1,GREM2,HOXA6,AXIN2	up_AFEM_EC
GO:0048066	0.00194	1	5	12	developmental pigmentation	BP	0.148	EDNRB,KIT,ADAMTS9,MEF2C,RAB27A	up_VCAV_EC
GO:0051965	0.00194	1	2	17	positive regulation of synapse assembly	BP	1	SLITRK5,NTRK2	up_APUL_EC
GO:0071650	0.00194	1	1	2	negative regulation of chemokine (C-C motif)	BP	0.504	ARG2	up_AORTRT_VSMC
GO:0032102	0.001945554	0.99962	7	67	negative regulation of response to external	BP	0.7009284	NR1H4,FNDC4,RGMA,MMP28,PTGER4,NRP1,PRKCD	up_ACOR_EC
GO:0050793	0.00195	1	5	731	regulation of developmental process	BP	0.874	SLITRK5,LIMK1,GATA5,LRRC17,C6	up_VCAV_VSMC
GO:0032528	0.00196	1	4	9	microvillus organization	BP	0.231	PTPN11,FSCN1,TNIK,KLF5	down_VSAP_EC
GO:0072001	0.001967691	0.99951	9	104	renal system development	BP	0.7009284	TBX18,SOX9,AQP1,SMAD6,FOXC2,LRP4,CLSTN1,NRP1,PYGO2	up_ACOR_EC
GO:0009062	0.00197	1	9	31	fatty acid catabolic process	BP	1	PEX7,ACAA1,ACACB,PEX2,FAAH,GCDH,EHHADH,LONP2,ABHD16A	up_VSAP_EC
GO:0035480	0.00197	1	1	1	regulation of Notch signaling pathway involved in heart	BP	0.599	GATA5	up_VPUL_VSMC
GO:0035481	0.00197	1	1	1	positive regulation of Notch signaling pathway involved in heart	BP	0.599	GATA5	up_VPUL_VSMC
GO:0003137	0.00197	1	1	1	Notch signaling pathway involved in heart	BP	0.599	GATA5	up_VPUL_VSMC
GO:0061999	0.00197	1	1	1	regulation of cardiac endothelial to	BP	0.599	GATA5	up_VPUL_VSMC
GO:0062000	0.00197	1	1	1	positive regulation of cardiac endothelial to mesenchymal transition	BP	0.599	GATA5	up_VPUL_VSMC
GO:0090381	0.00197	1	1	1	regulation of heart induction	BP	0.599	GATA5	up_VPUL_VSMC
GO:1901321	0.00197	1	1	1	positive regulation of heart induction	BP	0.599	GATA5	up_VPUL_VSMC
GO:0023057	0.00198	0.999	40	409	negative regulation of signaling	BP	0.231	ARHGEF2,PRICKLE1,SULF1,NR1H4,HSPA5,SERPINE1,SOCS5,PRKACB,ENPP1,ERRFI1,SNX25,HYOU1,PTPN11,FHL2,SMARCA4,SESN2,PTGS2,HIF1A,NXN,RGS2,PTPN2,ADAM17,CNOT7,CTNNB1,APOE,WWC3,LYN,STRAP,DDIT3,CBLC,ATF3,GSK3B,TAX1BP3,HTT,REST,CALM1,RAB11FIP1,HIF1AN,NRP1,TNFAIP1	down_VSAP_EC
GO:0008406	0.00198	1	2	57	gonad development	BP	0.504	HOXA9,ESR1	up_AORTRT_VSMC
GO:0045137	0.00198	1	2	57	development of primary sexual characteristics	BP	0.504	HOXA9,ESR1	up_AORTRT_VSMC
GO:0048641	0.00198	1	2	16	regulation of skeletal muscle tissue	BP	0.34	SIX1,CYP26B1	up_VFEM_EC
GO:0000050	0.00198	1	1	2	urea cycle	BP	0.504	ARG2	up_AORTRT_VSMC
GO:0032696	0.00198	1	1	2	negative regulation of interleukin-13 production	BP	0.504	ARG2	up_AORTRT_VSMC

GO:0046777	0.00199	0.999	17	86	protein autophosphorylation	BP	0.151	EPHB1,TEK,KIT,MAP3K11,PDGFB,STK10,EPHB4,MTOR,MAP3K3,SLK,PAK2,PRKD2,FES,RIPK1,PDGFD,BMX,LMTK2	up_VCAV_EC
GO:0009887	0.00199	1	6	314	animal organ morphogenesis	BP	0.544	HOXA11,TBX15,SALL1,IRX5,CAV1,SHOX2	up_VSAP_VSMC
GO:0061572	0.001998061	0.99967	6	50	actin filament bundle organization	BP	0.7009284	ARHGAP6,SYNPO2,PLS1,ARHGAP28,PTGER4,NRP1	up_ACOR_EC
GO:2000826	0.00201	1	1	14	regulation of heart morphogenesis	BP	1	GATA5	down_AFEM_VSMC
GO:1901213	0.00201	1	1	7	regulation of transcription from RNA polymerase II promoter involved in	BP	0.829	GATA5	down_VFEM_VSMC
GO:0003218	0.00201	1	1	1	cardiac left ventricle formation	BP	0.599	TBX5	up_VPUL_VSMC
GO:0045893	0.00204	0.999	53	413	positive regulation of transcription, DNA-templated	BP	0.154	HCLS1,PPARG,TNIP1,PDGFB,TMEM173,MEF2C,ATF3,CHD7,TLR4,NLRC5,GATA3,EDN1,TCF4,FOXN2,ENSCAFG00000017103,IRF8,PPP3CA,YES1,MTOR,BMP2,HHEX,CREM,PROX1,ARID4B,HMBOX1,PRKD2,THOC1,SMARCA4,TLR3,NCK1,LDB2,JAG1,NSD1,BCL10,CNOT7,HMGB1,AHR,BLM,RIPK1,ITGA6,GPBP1,MTA2,NR1D2,KDM5A,HIF1A,KDM3A,ATRX,NR4A1,ZEB1,TET2,ARHGEF2,CDON,ZNF609	up_VCAV_EC
GO:0043534	0.00205	0.999	10	57	blood vessel endothelial cell migration	BP	0.235	VEGFA,MYH9,JCAD,PRCP,PIK3C2A,HIF1A,ADAM17,STARD13,APOE,NRP1	down_VSAP_EC
GO:0072615	0.00205	1	1	2	interleukin-17 secretion	BP	0.504	ARG2	up_AORTRT_VSMC
GO:0045087	0.00206	1	4	161	innate immune response	BP	1	NR1H4,MRC1,CCL24,ARHGEF2	up_VPUL_EC
GO:0048562	0.00206	1	2	102	embryonic organ morphogenesis	BP	1	HOXA3,HOXB5	up_APUL_VSMC
GO:0030155	0.00208	1	5	180	regulation of cell adhesion	BP	0.473	ACVRL1,LYN,PDPN,HOXA7,STX4	down_ACOR_EC
GO:0060054	0.00208	1	1	2	positive regulation of epithelial cell proliferation involved in wound healing	BP	0.504	WNT7A	up_AORTRT_VSMC
GO:0021615	0.00208	1	1	3	glossopharyngeal nerve morphogenesis	BP	1	HOXA3	up_APUL_VSMC
GO:0031133	0.00209	1	1	2	regulation of axon diameter	BP	0.504	WNT7A	up_AORTRT_VSMC
GO:0060068	0.00209	1	1	2	vagina development	BP	0.504	ESR1	up_AORTRT_VSMC
GO:0007275	0.0021	0.999	13	1370	multicellular organism development	BP	0.353	HOXC10,TBX15,EYA1,HOXA9,TOX,MME,HOXB7,HOXA7,SIX1,MAB21L1,TIAM1,CYP26B1,MTSS1	up_VFEM_EC
GO:0006928	0.0021	1	9	557	movement of cell or subcellular component	BP	0.473	ACVRL1,LYN,IGF1R,SPG11,PDPN,NRP2,HOXA7,SULF1,STX4	down_ACOR_EC
GO:0003002	0.0021	1	4	118	regionalization	BP	0.473	HOXA3,NRP2,HOXA7,HOXC4	down_ACOR_EC
GO:1900122	0.0021	1	1	3	positive regulation of receptor binding	BP	1	HOXA3	up_APUL_VSMC
GO:0042060	0.00211	1	4	103	wound healing	BP	0.473	ACVRL1,LYN,PDPN,ELK3	down_ACOR_EC
GO:0009952	0.00211	1	2	66	anterior/posterior pattern specification	BP	0.504	HOXC10,HOXA9	up_AORTRT_VSMC
GO:0001554	0.00211	1	1	1	luteolysis	BP	0.599	SLIT2	up_VPUL_VSMC
GO:0010593	0.00211	1	1	1	negative regulation of lamellipodium assembly	BP	0.599	SLIT2	up_VPUL_VSMC
GO:0021823	0.00211	1	1	1	cerebral cortex tangential migration using cell-cell interactions	BP	0.599	SLIT2	up_VPUL_VSMC
GO:0021827	0.00211	1	1	1	postnatal olfactory bulb interneuron migration	BP	0.599	SLIT2	up_VPUL_VSMC
GO:0021836	0.00211	1	1	1	chemorepulsion involved in postnatal olfactory bulb interneuron migration	BP	0.599	SLIT2	up_VPUL_VSMC
GO:0021966	0.00211	1	1	1	corticospinal neuron axon guidance	BP	0.599	SLIT2	up_VPUL_VSMC
GO:0021972	0.00211	1	1	1	corticospinal neuron axon guidance through spinal	BP	0.599	SLIT2	up_VPUL_VSMC
GO:0035385	0.00211	1	1	1	Roundabout signaling pathway	BP	0.599	SLIT2	up_VPUL_VSMC
GO:0050924	0.00211	1	1	1	positive regulation of negative chemotaxis	BP	0.599	SLIT2	up_VPUL_VSMC
GO:0050929	0.00211	1	1	1	induction of negative chemotaxis	BP	0.599	SLIT2	up_VPUL_VSMC
GO:0051414	0.00211	1	1	1	response to cortisol	BP	0.599	SLIT2	up_VPUL_VSMC

GO:0061364	0.00211	1	1	1	apoptotic process involved in luteolysis	BP	0.599	SLIT2	up_VPUL_VSMC
GO:0070099	0.00211	1	1	1	regulation of chemokine-mediated signaling	BP	0.599	SLIT2	up_VPUL_VSMC
GO:0070100	0.00211	1	1	1	negative regulation of chemokine-mediated	BP	0.599	SLIT2	up_VPUL_VSMC
GO:0071670	0.00211	1	1	1	smooth muscle cell chemotaxis	BP	0.599	SLIT2	up_VPUL_VSMC
GO:0071671	0.00211	1	1	1	regulation of smooth muscle cell chemotaxis	BP	0.599	SLIT2	up_VPUL_VSMC
GO:0071672	0.00211	1	1	1	negative regulation of smooth muscle cell	BP	0.599	SLIT2	up_VPUL_VSMC
GO:0090260	0.00211	1	1	1	negative regulation of retinal ganglion cell axon	BP	0.599	SLIT2	up_VPUL_VSMC
GO:0050896	0.002112158	0.99871	81	2346	response to stimulus	BP	0.72518906	C7,SH3TC2,CDH3,NR1H4,GATA4,BRSK1,PLCL1,CALY,SOX9,HMCN1,RHOU,ARHGAP6,PTH1R,RGS1,PCSK1N,FNDC4,NDRG2,AQP1,PORCN,ENSCAFG00000004331,KCNB1,ADAMTS13,EDEM2,FZD5,CCDC3,AGRN,PDE1A,IFI6,GNAO1,DUSP26,DIXDC1,RASL11A,PTGIR,NOD2,IL33,RGMA,PDE4A,INSYN1,SMAD6,PLCE1,ACTN2,ARHGAP28,ESM1,PAK3,MMP28,GUCY1B1,GRK7,TRIM8,TNFRSF19,PTGER4,ARHGDIB,ELMO2,DCLK2,ADAMTSL2,USP53,MAPK8IP2,RAPGEF4,MAVS,FOXC2,FIGNL1,LRRC15,IFIT2,LRP4,CLSTN1,SH3RF3,LRRC4,CLU,C23H3orf58,RARRES2,AGBL5,PRKACA,STK32C,NRP1,HM13,ARHGEF26,PRKCD,GNAZ,RIN2,PYGO2,MFAP4,ADGRL1	up_ACOR_EC
GO:1901655	0.00212	1	7	20	cellular response to ketone	BP	0.612	PRKAA2,ADCY6,PTGDR,PRKCE,NR3C1,DDIT4,PARK7	up_ACOR_VSMC
GO:0000082	0.00213	0.999	12	57	G1/S transition of mitotic cell cycle	BP	0.129	HINFP,POLE,CASP2,CDKN2C,EZH2,CDK4,PLK3,RCC1,CDK2,RBBP8,CDKN3,MTBP	down_ACOR_VSMC
GO:0031128	0.00214	1	1	15	developmental induction	BP	1	GATA5	down_AFEM_VSMC
GO:0022610	0.00215	0.999	34	326	biological adhesion	BP	0.243	COL15A1,VEGFA,SULF1,MYH9,SERPINE1,ACTN1,SOCS5,ITGA2,FAM49B,NUAK1,PTPN11,FBLIM1,ILK,PPP1R12A,MSN,THBS2,PTPN2,PVR,ADAM17,CDH11,CTNNB1,ENSCAFG00000002015,TFRC,IGFBP7,LYN,CD44,TLN1,SLC7A11,GSK3B,ITGB3,SDC4,ICAM5,LMO7,NRP1	down_VSAP_EC
GO:0036003	0.00215	1	4	10	positive regulation of transcription from RNA polymerase II promoter in response to stress	BP	0.243	SESN2,HIF1A,DDIT3,ATF3	down_VSAP_EC
GO:1902117	0.00215	1	2	32	positive regulation of organelle assembly	BP	1	GPSM2,FSCN1	down_AFEM_EC
GO:0097190	0.00218	0.999	24	217	apoptotic signaling pathway	BP	0.243	ARHGEF2,P4HB,SERPINE1,ERO1A,CASP3,HYOU1,PTGS2,UACA,HIF1A,DNAJC10,PTPN2,MAGED1,CTNNB1,BNIP3,DDIT3,FAM162A,ATF3,SH3GLB1,HTT,PAK2,INHBA,CDKN1A,NRP1,MADD	down_VSAP_EC
GO:0072525	0.00218	1	6	24	pyridine-containing compound biosynthetic	BP	0.243	PGK1,TPI1,PGAM1,HIF1A,NMNAT2,ALDOC	down_VSAP_EC
GO:0048643	0.0022	1	4	7	positive regulation of skeletal muscle tissue	BP	1	CDON,DLL1,SHOX2,FLOT1	up_VSAP_EC
GO:0050919	0.0022	1	2	10	negative chemotaxis	BP	1	SEMA3A,SEMA3D	up_VPOR_EC
GO:0010563	0.00221	1	5	203	negative regulation of phosphorus metabolic	BP	0.483	LYN,IGF1R,PRKAR2A,MYO1D,ASPN	down_ACOR_EC
GO:0045936	0.00221	1	5	203	negative regulation of phosphate metabolic	BP	0.483	LYN,IGF1R,PRKAR2A,MYO1D,ASPN	down_ACOR_EC
GO:1903670	0.00223	1	8	30	regulation of sprouting angiogenesis	BP	0.166	PLK2,ADAMTS9,DLL4,APLNR,SMAD1,PIK3C2A,MAP3K3,JCAD	up_VCAV_EC
GO:0060523	0.00223	1	1	2	prostate epithelial cord elongation	BP	0.504	ESR1	up_AORTRT_VSMC
GO:0003214	0.00223	1	1	3	cardiac left ventricle morphogenesis	BP	1	TBX5	up_APUL_VSMC
GO:0072513	0.00223	1	1	3	positive regulation of secondary heart field cardioblast proliferation	BP	1	TBX5	up_APUL_VSMC
GO:0086091	0.00224	1	1	8	regulation of heart rate by cardiac conduction	BP	0.829	JUP	down_VFEM_VSMC
GO:0021684	0.00224	1	1	2	cerebellar granular layer formation	BP	0.504	WNT7A	up_AORTRT_VSMC
GO:0021707	0.00224	1	1	2	cerebellar granule cell differentiation	BP	0.504	WNT7A	up_AORTRT_VSMC
GO:0009653	0.00225	1	5	773	anatomical structure morphogenesis	BP	0.851	HOXC10,TBX15,EYA1,HOXA9,IRX5	up_AFEM_VSMC

GO:0030326	0.00225	1	2	51	embryonic limb morphogenesis	BP	0.851	HOXC10,HOXA9	up_AFEM_VSMC
GO:0035113	0.00225	1	2	51	embryonic appendage morphogenesis	BP	0.851	HOXC10,HOXA9	up_AFEM_VSMC
GO:0006104	0.00225	1	1	1	succinyl-CoA metabolic process	BP	1	SUCLG2	down_AFEM_EC
GO:0006422	0.00226	1	1	1	aspartyl-tRNA aminoacylation	BP	1	DARS	down_AFEM_EC
GO:0051093	0.00227	0.999	29	273	negative regulation of developmental process	BP	0.251	PRICKLE1,SULF1,SERPINE1,SOCS5,SPARC,PRKACB,ENPP1,PGK1,DPYSL3,HOOK3,TRIB3,IFRD1,HIF1A,THBS2,PTPN2,CTNNB1,STARD13,APOE,WWC3,LYN,BNIP3,STRAP,DDIT3,REST,PRMT1,INHBA,SIX4,NRP1,YWHAH	down_VSAP_EC
GO:0030154	0.00227	0.999	11	1040	cell differentiation	BP	0.373	HOXC10,EYA1,HOXA9,TOX,HOXB7,HOXA7,SIX1,TIAM1,RADIL,CYP26B1,MTSS1	up_VFEM_EC
GO:0003197	0.00227	1	1	16	endocardial cushion development	BP	1	GATA5	down_AFEM_VSMC
GO:0035801	0.00227	1	1	1	adrenal cortex development	BP	1	CITED2	down_VPOR_EC
GO:0035802	0.00227	1	1	1	adrenal cortex formation	BP	1	CITED2	down_VPOR_EC
GO:0014719	0.00227	1	1	2	skeletal muscle satellite cell activation	BP	0.504	WNT7A	up_AORTRT_VSMC
GO:0071310	0.0023	0.999	56	634	cellular response to organic substance	BP	0.253	ARHGEF2,VEGFA,SULF1,NR1H4,HSPA5,SERPINE1,SOCS5,SPARC,ERO1A,ENPP1,JCAD,SNX25,PTPN11,FLNB,DPYSL3,TRIB3,ILK,PRCP,SMARCA4,SESN2,TGFBR1,MYO1C,MSN,KIF5B,HIF1A,CX3CL1,ZPR1,PTPN2,YES1,NEO1,MTDH,ADAM17,AGRN,CNOT7,GNAQ,CTNNB1,ENSCAFG00000002015,TFRC,ZC3H15,KLF5,UBA5,LYN,STRAP,DDIT3,ATF3,CDC37,GBP5,REST,PAK2,CREB3L2,NR4A1,ENSCAFG00000018360,BTK,NRP1,YWHAH,KDM3A	down_VSAP_EC
GO:0001974	0.0023	1	2	13	blood vessel remodeling	BP	0.488	ACVRL1,HOXA3	down_ACOR_EC
GO:0007155	0.0023	1	2	324	cell adhesion	BP	0.829	JUP,GATA5	down_VFEM_VSMC
GO:0014834	0.0023	1	1	2	skeletal muscle satellite cell maintenance involved in skeletal muscle	BP	0.504	WNT7A	up_AORTRT_VSMC
GO:0048729	0.00232	0.999	24	209	tissue morphogenesis	BP	0.253	PRICKLE1,SULF1,PRKACB,PTK7,ITGA2,EXOC5,ENSCAFG00000032470,TMED2,ILK,WDR1,MSN,HOXB7,HIF1A,ACTG2,ADAM17,ACTA2,MAGED1,CTNNB1,STARD13,PLOD3,SDC4,INHBA,SIX4,NRP1	down_VSAP_EC
GO:0051153	0.00232	1	2	28	regulation of striated muscle cell differentiation	BP	1	NKX2-5,CYP26B1	up_VPOR_VSMC
GO:0009968	0.00233	1	5	392	negative regulation of signal transduction	BP	1	BARX1,NKX2-5,BAMBI,ANKRD6,CYP26B1	up_VPOR_VSMC
GO:0001570	0.00233	1	2	37	vasculogenesis	BP	1	HDAC7,CITED2	down_VPOR_EC
GO:0022610	0.00233	1	2	326	biological adhesion	BP	0.829	JUP,GATA5	down_VFEM_VSMC
GO:0060675	0.00233	1	2	29	ureteric bud morphogenesis	BP	0.785	EYA1,HOXA11	up_VFEM_VSMC
GO:0043502	0.00233	1	1	16	regulation of muscle adaptation	BP	1	GATA5	down_AFEM_VSMC
GO:0051891	0.00233	1	1	3	positive regulation of cardioblast differentiation	BP	1	TBX5	up_APUL_VSMC
GO:0085020	0.00234	1	3	4	protein K6-linked ubiquitination	BP	0.141	UBE2S,UBE2T,BRCA1	down_ACOR_VSMC
GO:0070235	0.00234	1	2	2	regulation of activation-induced cell death of T	BP	1	GPAM,TSC22D3	down_VCAV_EC
GO:0070236	0.00234	1	2	2	negative regulation of activation-induced cell	BP	1	GPAM,TSC22D3	down_VCAV_EC
GO:0003197	0.00236	1	6	16	endocardial cushion development	BP	0.656	GATA5,GATA4,BMP4,CITED2,TBX20,TBX5	up_ACOR_VSMC
GO:0001937	0.00236	1	2	15	negative regulation of endothelial cell	BP	0.488	ACVRL1,SULF1	down_ACOR_EC
GO:0060017	0.00236	1	1	3	parathyroid gland development	BP	1	HOXA3	up_APUL_VSMC
GO:0060351	0.00237	1	5	11	cartilage development involved in endochondral bone morphogenesis	BP	1	HOXA11,AXIN2,RARG,SHOX2,TGFBR2	up_VSAP_EC
GO:0003008	0.00237	1	2	336	system process	BP	0.829	JUP,GATA5	down_VFEM_VSMC
GO:0051241	0.00239	1	2	336	negative regulation of multicellular organismal	BP	0.829	JUP,GATA5	down_VFEM_VSMC
GO:0120163	0.00239	1	2	17	negative regulation of cold-induced	BP	0.389	HOXC10,MAP2K6	up_VFEM_EC

GO:0030282	0.00239	1	2	37	bone mineralization	BP	0.666	HOXA3,PHEX	up_VPUL_VSMC
GO:0001503	0.002399262	0.99939	9	109	ossification	BP	0.7913958	SOX9,PTH1R,SMAD6,PTGER4,FOXC2,FIGNL1,LRP4,CLSTN1,TP53INP2	up_ACOR_EC
GO:0035296	0.0024	0.999	9	32	regulation of tube diameter	BP	0.656	ADRA1B,HTR2B,HRH1,ADCY6,F2RL1,HRH2,PTGDR,EDN1,DOCK5	up_ACOR_VSMC
GO:0097746	0.0024	0.999	9	32	regulation of blood vessel diameter	BP	0.656	ADRA1B,HTR2B,HRH1,ADCY6,F2RL1,HRH2,PTGDR,EDN1,DOCK5	up_ACOR_VSMC
GO:0042221	0.00241	0.998	107	1012	response to chemical	BP	0.178	ACKR1,EPHB1,EDNRB,HCLS1,GBP5,KIT,SELE,PRCP,CXCL8,LAPTM5,CXCR4,DOCK4,CALCRL,TIE1,TNFAIP3,PPARG,TNIP1,MX2,AMOT,PDGFB,P2RX7,BAIAP2L1,TMEM173,RELN,CD34,SELL,PDE4D,MEF2C,FERMT1,ATF3,CHD7,TIPARP,DLL4,RAPGEF1,TLR4,NLRC5,GATA3,MMP28,EDN1,RALB,SMAD1,TFPI,IRF8,PELI1,PPP3CA,YES1,CASP8,MTOR,BMP2,TJP1,HHEX,RALBP1,ST3GAL6,ABCA1,MAPK13,ITPKB,IFNGR1,RIOK3,ANGPT2,ENSCAFG00000010596,FLNB,PAK2,PRKD2,SWAP70,SMARCA4,TLR3,APOD,NCK1,TIMELESS,OTUD4,SLC25A25,FES,JAG1,GUCY1B1,PTGS2,TOP1,RAPGEF2,PLCG2,CTDSPL2,CREB1,BCL10,CTNNA1,CNOT7,GCH1,ADIPOR2,HMGB1,AHR,PMAIP1,BLM,RIPK1,LSM14A,USP13,SKIL,PDGFD,UFSP2,HIF1A,KDM3A,ATRX,NR4A1,HP1BP3,ARID5A,ENSCAFG00000002015,P2RX2,JCAD,ZEB1,ARHGEF2,TJP2	up_VCAV_EC
GO:0061418	0.00241	1	3	5	regulation of transcription from RNA polymerase II promoter in response to	BP	0.26	HIF1A,BACH1,HIF1AN	down_VSAP_EC
GO:0034109	0.00241	1	2	14	homotypic cell-cell adhesion	BP	0.488	LYN,PDPN	down_ACOR_EC
GO:0050793	0.00242	0.999	9	731	regulation of developmental process	BP	0.39	EYA1,HOXA9,TOX,HOXB7,HOXA7,SIX1,TIAM1,CYP26B1,CDC42EP5	up_VFEM_EC
GO:0060070	0.00242	1	3	105	canonical Wnt signaling pathway	BP	1	NKX2-5,BAMBI,ANKRD6	up_VPOR_VSMC
GO:0007338	0.00242	1	2	16	single fertilization	BP	0.652	HOXA11,HOXA9	up_VSAP_VSMC
GO:0007167	0.00243	0.999	29	274	enzyme linked receptor protein signaling pathway	BP	0.26	VEGFA,SIPA1L1,SULF1,NR1H4,HSPA5,SOCS5,ENPP1,ERRFI1,JCAD,SNX25,PTPN11,ILK,TGFBR1,MYO1C,HIF1A,PTPN2,NEO1,ESM1,ADAM17,CTNNB1,LYN,STRAP,CBLC,MYO1E,INHBA,ENSCAFG00000018360,BTK,NRP1,RHBDF2	down_VSAP_EC
GO:0032493	0.00243	1	1	2	response to bacterial lipoprotein	BP	0.851	TLR2	up_AFEM_VSMC
GO:0042494	0.00243	1	1	2	detection of bacterial lipoprotein	BP	0.851	TLR2	up_AFEM_VSMC
GO:0042495	0.00243	1	1	2	detection of triacyl bacterial lipopeptide	BP	0.851	TLR2	up_AFEM_VSMC
GO:0070339	0.00243	1	1	2	response to bacterial lipopeptide	BP	0.851	TLR2	up_AFEM_VSMC
GO:0070340	0.00243	1	1	2	detection of bacterial lipopeptide	BP	0.851	TLR2	up_AFEM_VSMC
GO:0071220	0.00243	1	1	2	cellular response to bacterial lipoprotein	BP	0.851	TLR2	up_AFEM_VSMC
GO:0071221	0.00243	1	1	2	cellular response to bacterial lipopeptide	BP	0.851	TLR2	up_AFEM_VSMC
GO:0071725	0.00243	1	1	2	response to triacyl bacterial lipopeptide	BP	0.851	TLR2	up_AFEM_VSMC
GO:0071727	0.00243	1	1	2	cellular response to triacyl bacterial lipopeptide	BP	0.851	TLR2	up_AFEM_VSMC
GO:0045165	0.00244	1	2	65	cell fate commitment	BP	0.504	HOXC10,WNT7A	up_AORTRT_VSMC
GO:0006468	0.002452682	0.99884	26	544	protein phosphorylation	BP	0.7913958	BRSK1,PLCL1,PKIB,SOX9,NDRG2,ENSCAFG00000004331,FZD5,DUSP26,NOD2,SMAD6,PAK3,GRK7,TNFRSF19,DCLK2,MAPK8IP2,MAVS,LRRC15,LRP4,SH3RF3,LRRC4,RARRES2,STK32C,NRP1,PRKCD,DBNDD2,CCNYL1	up_ACOR_EC
GO:0086001	0.00246	1	1	9	cardiac muscle cell action potential	BP	0.829	JUP	down_VFEM_VSMC
GO:0048736	0.00247	1	3	66	appendage development	BP	0.391	HOXC10,HOXA9,CYP26B1	up_VFEM_EC
GO:0060173	0.00247	1	3	66	limb development	BP	0.391	HOXC10,HOXA9,CYP26B1	up_VFEM_EC
GO:2001222	0.00248	1	2	11	regulation of neuron migration	BP	1	ARHGEF2,SEMA3A	up_VPOR_EC
GO:0061005	0.00249	0.999	8	28	cell differentiation involved in kidney	BP	0.182	PDGFB,PODXL,MEF2C,GPR4,GATA3,MTSS1,JAG1,BASP1	up_VCAV_EC

GO:0043620	0.00249	1	6	24	regulation of DNA-templated transcription in	BP	0.262	SESN2,HIF1A,DDIT3,BACH1,ATF3,HIF1AN	down_VSAP_EC
GO:0060272	0.00249	1	4	7	embryonic skeletal joint morphogenesis	BP	1	HOXA11,OSR2,OSR1,SHOX2	up_VSAP_EC
GO:0001655	0.00249	1	3	116	urogenital system development	BP	0.82	EYA1,EMX2,HOXA11	up_VFEM_VSMC
GO:0023014	0.0025	0.999	38	248	signal transduction by protein phosphorylation	BP	0.673	FGF13,ADRA1B,LEP,MAP3K8,MID1,HTR2B,RASSF2,MBP,BMP4,FGFR2,TLR3,SH3RF2,PDE5A,SASH1,FGF18,NDRG2,F2RL1,NRP1,PTPN22,NPNT,SMPD1,SH3RF1,PRKCE,FRS2,DAG1,APP,PSEN1,SYNJ2BP,CCL5,DUSP22,EDN1,DUSP26,NOD2,SH3RF3,MAP3K7,INPP5K,ENSCAFG00000029553,CRKL	up_ACOR_VSMC
GO:0044267	0.00251	0.998	118	1583	cellular protein metabolic process	BP	0.262	ARHGEF2,PRICKLE1,VEGFA,P4HB,CARS,PLOD2,MYH9,NR1H4,PLK3,HSPA5,SERPINE1,SOCS5,HM13,SGK1,ERO1A,HSP90B1,ENPP1,ERRFI1,P4HA1,JCAD,SNX25,SARS,WSB1,BCAR3,NUAK1,PTPN11,AARS,TRIB3,PUM3,PM20D2,ILK,PPP1R12A,SESN2,PTGS2,PPIB,MSN,SEL1L,USP12,MRPL1,IWS1,DNAJC10,TNIK,GALNT1,NAA10,NXN,UBA6,PTPN2,UCHL5,MORF4L2,SPOPL,YARS,TARS,YES1,PLCL1,TTLL7,NARS,ADAM17,EIF4EBP1,CNOT7,MAGED1,DNAJB9,CTNNB1,CRYAB,CDK8,APOE,PLOD3,USP48,UBA5,LYN,BNIP3,STRAP,DDIT3,FAM162A,BACH1,ASPH,CCNYL1,STT3A,EIF5,MMD,CBLC,ATF3,GSK3B,VLDLR,GTF2H1,CUL2,HTT,REST,ZMYND8,MAPK10,EIF2S1,PRMT1,P3H4,EXT1,SPOP,CALM1,DISC1,RAD23A,CDYL,PAK2,A2M,INHBA,CAMK2D,HIF1AN,BTK,MSRB3,CDKN1A,TMTC3,RSL24D1,NRP1,SPINK2,SH3RF3,USP14,ARF4,TNFAIP1,MADD,RPN1,KDM3A,MID1	down_VSAP_EC
GO:0014045	0.00251	1	1	2	establishment of endothelial blood-brain	BP	0.504	WNT7A	up_AORTRT_VSMC
GO:0022009	0.00251	1	1	2	central nervous system vasculogenesis	BP	0.504	WNT7A	up_AORTRT_VSMC
GO:0060066	0.00251	1	1	2	oviduct development	BP	0.504	WNT7A	up_AORTRT_VSMC
GO:0060856	0.00251	1	1	2	establishment of blood-brain barrier	BP	0.504	WNT7A	up_AORTRT_VSMC
GO:0009617	0.002512916	0.9993	10	133	response to bacterium	BP	0.7913958	NR1H4,HMCN1,RGS1,FZD5,PTGIR,NOD2,PTGER4,MAVS,RARRES2,PRKCD	up_ACOR_EC
GO:0009081	0.00252	1	3	6	branched-chain amino acid metabolic process	BP	1	ENSCAFG00000002977,BCKDHB,ACAT1	down_VCAV_EC
GO:0003176	0.00252	1	1	9	aortic valve development	BP	0.829	GATA5	down_VFEM_VSMC
GO:0015833	0.00253	0.999	47	521	peptide transport	BP	0.262	PRICKLE1,MYH9,NR1H4,PLK3,HM13,TRAM1,PTPN14,SEC24D,PTPN11,SEC23A,SRP54,MYO1C,ENSCAFG00000031588,SEL1L,HIF1A,SSR1,IWS1,ZPR1,STAM2,SMG7,XPOT,USO1,SRPRA,DDX39B,COPG1,KPNB1,COPB2,SRP72,TFRC,APOE,KPNA4,LYN,GSK3B,GBP5,KPNA1,REST,SCAMP1,RAB11FIP1,KDELR2,AP3S1,CLTC,STX6,CDKN1A,RHBDF2,YWHAH,BLZF1,SEC13	down_VSAP_EC
GO:0007176	0.00253	1	4	10	regulation of epidermal growth factor-activated	BP	0.262	SOCS5,ERRFI1,ADAM17,CBLC	down_VSAP_EC
GO:1904062	0.00253	1	3	62	regulation of cation transmembrane transport	BP	0.668	NETO1,STIM1,CAV1	up_VSAP_VSMC
GO:0072171	0.00254	1	2	30	mesonephric tubule morphogenesis	BP	0.82	EYA1,HOXA11	up_VFEM_VSMC
GO:0003129	0.00254	1	1	3	heart induction	BP	1	GATA5	down_VSAP_VSMC
GO:0009132	0.00256	1	6	25	nucleoside diphosphate metabolic process	BP	0.263	PGK1,TPI1,PGAM1,HIF1A,ENTPD4,ALDOC	down_VSAP_EC
GO:0007417	0.00257	1	5	235	central nervous system development	BP	0.668	PITX1,EMX2,SALL1,HOXC10,HAPLN1	up_VSAP_VSMC
GO:0060485	0.00257	1	2	93	mesenchyme development	BP	1	GATA5,TGFBR1	down_VSAP_VSMC
GO:0060575	0.00257	1	1	9	intestinal epithelial cell differentiation	BP	0.829	GATA5	down_VFEM_VSMC
GO:0003162	0.00257	1	1	1	atrioventricular node development	BP	1	NKX2-5	up_VPOR_VSMC
GO:0003165	0.00257	1	1	1	Purkinje myocyte development	BP	1	NKX2-5	up_VPOR_VSMC
GO:0003168	0.00257	1	1	1	Purkinje myocyte differentiation	BP	1	NKX2-5	up_VPOR_VSMC
GO:0003292	0.00257	1	1	1	cardiac septum cell differentiation	BP	1	NKX2-5	up_VPOR_VSMC

GO:0060922	0.00257	1	1	1	atrioventricular node cell differentiation	BP	1	NKX2-5	up_VPOR_VSMC
GO:0060927	0.00257	1	1	1	cardiac pacemaker cell fate commitment	BP	1	NKX2-5	up_VPOR_VSMC
GO:0060928	0.00257	1	1	1	atrioventricular node cell development	BP	1	NKX2-5	up_VPOR_VSMC
GO:0060929	0.00257	1	1	1	atrioventricular node cell fate commitment	BP	1	NKX2-5	up_VPOR_VSMC
GO:0060932	0.00257	1	1	1	His-Purkinje system cell differentiation	BP	1	NKX2-5	up_VPOR_VSMC
GO:0021545	0.00258	1	2	21	cranial nerve development	BP	1	HOXA3,HOXA1	up_APUL_EC
GO:0061343	0.00258	1	1	3	cell adhesion involved in heart morphogenesis	BP	1	GATA5	down_VSAP_VSMC
GO:0090130	0.00259	0.999	16	121	tissue migration	BP	0.265	VEGFA,MYH9,SPARC,JCAD,PTPN11,PRCP,PIK3C2A,HIF1A,ACTG2,ADAM17,ACTA2,ANLN,STARD13,APOE,STRAP,NRP1	down_VSAP_EC
GO:0045747	0.00259	1	1	18	positive regulation of Notch signaling pathway	BP	1	GATA5	down_AFEM_VSMC
GO:0070887	0.0026	0.998	85	775	cellular response to chemical stimulus	BP	0.19	ACKR1,EPHB1,EDNRB,HCLS1,GBP5,KIT,PRCP,CXCL8,LAPTM5,CXCR4,DOCK4,CALCRL,TNFAIP3,PPARG,TNIP1,PDGFB,BAIAP2L1,TMEM173,CD34,PDE4D,MEF2C,FERMT1,ATF3,TIPARP,DLL4,RAPGEF1,TLR4,NLRC5,GATA3,MMP28,RALB,SMAD1,TFPI,IRF8,PPP3CA,YES1,MTOR,BMP2,TJP1,HHEX,ST3GAL6,ABCA1,MAPK13,ITPKB,IFNGR1,RIOK3,ENSCAFG00000010596,FLNB,PAK2,PRKD2,SWAP70,SMARCA4,NCK1,TIMELESS,OTUD4,GUCY1B1,PTGS2,RAPGEF2,CTDSPL2,CREB1,CTNNA1,CNOT7,GCH1,ADIPOR2,HMGB1,AHR,PMAIP1,BLM,RIPK1,LSM14A,SKIL,PDGFD,UFSP2,HIF1A,KDM3A,ATRX,NR4A1,HP1BP3,ARID5A,ENSCAFG00000002015,P2RX2,JCAD,ZEB1,ARHGEF2,TJP2	up_VCAV_EC
GO:0002762	0.0026	1	2	15	negative regulation of myeloid leukocyte	BP	0.488	LYN,HOXA7	down_ACOR_EC
GO:0044319	0.0026	1	2	12	wound healing, spreading of cells	BP	0.488	ACVRL1,PDPN	down_ACOR_EC
GO:0090504	0.0026	1	2	12	epiboly	BP	0.488	ACVRL1,PDPN	down_ACOR_EC
GO:0090505	0.0026	1	2	12	epiboly involved in wound healing	BP	0.488	ACVRL1,PDPN	down_ACOR_EC
GO:0021563	0.0026	1	1	4	glossopharyngeal nerve development	BP	1	HOXA3	up_APUL_VSMC
GO:0001654	0.002602942	0.99933	9	110	eye development	BP	0.7913958	CPAMD8,TMOD1,SOX9,AQP1,FZD5,FOXC2,NRP1,PYGO2,EFEMP1	up_ACOR_EC
GO:0048880	0.002602942	0.99933	9	110	sensory system development	BP	0.7913958	CPAMD8,TMOD1,SOX9,AQP1,FZD5,FOXC2,NRP1,PYGO2,EFEMP1	up_ACOR_EC
GO:0150063	0.002602942	0.99933	9	110	visual system development	BP	0.7913958	CPAMD8,TMOD1,SOX9,AQP1,FZD5,FOXC2,NRP1,PYGO2,EFEMP1	up_ACOR_EC
GO:0045165	0.00261	1	3	65	cell fate commitment	BP	0.669	HOXA11,PITX1,HOXC10	up_VSAP_VSMC
GO:0048646	0.00261	1	2	349	anatomical structure formation involved in	BP	0.829	JUP,GATA5	down_VFEM_VSMC

GO:0051179	0.00263	0.998	172	1747	localization	BP	0.191	EPHB1,PLK2,EDNRB,TEK,HCLS1,GBP5,SELP,KIT,SELE,PRCP,PPP2R5A,CXCL8,CP,OSBPL9,DOCK4,CLEC14A,CALCRL,TIE1,TNFAIP3,ADAMTS9,ATP8A1,LRRC8D,MX2,AMOT,PDGFB,MALL,PODXL,STK10,TMEM173,ATP2B1,EXOC3L2,RELN,CD34,SELL,PDE4D,IWS1,MEF2C,FERMT1,CHD7,DLL4,SLC35A3,TLR4,QKI,TSPAN15,STC1,GATA3,MMP28,EDN1,RAB11A,KIF3C,APLNR,ARAP3,RAB27A,SLC39A8,EXOC6,CTTNBP2NL,SNCAIP,SLC49A3,ZDHHC22,NEDD9,IRF8,SLCO2A1,SOX18,PIK3C2A,PPP3CA,YES1,SLC38A1,ARG2,EPHB4,NECTIN2,MTOR,HOOK3,SNAP23,LRRC8C,TSPAN14,MAP3K3,BMP2,HHEX,RAB6A,SPNS3,CAV2,SLK,CEP68,BMP2K,RALBP1,SCAMP1,RSC1A1,PDZD8,ANKRD50,CCAR1,ABCA1,KCNJ3,MZT1,ANGPT2,PROX1,ENSCAFG00000010224,OSBPL6,EPB41,ELMO1,STMN1,SNX2,SLCO3A1,PAK2,MCC,PRKD2,THOC1,SH3GLB1,SWAP70,AKAP12,APOD,CENPC,SLC2A3,NCK1,KCNJ2,EXOC5,LDB2,CD200,SLC25A25,FES,JAG1,HIST1H1A,MMD,EHD4,RAPGEF2,PLCG2,CTDSPL2,CREB1,PAN3,GCC2,CTNNA1,FMNL3,STX11,SMG7,VPS54,PRAG1,HMGB1,TBC1D1,NOS2,PMAIP1,ARPC5L,NUP133,SPTBN4,RIPK1,CHIC2,ITGA6,RAD21,MCU,DNAJC13,PDGFD,ARHGDIB,ZW10,HIF1A,IPO11,ALS2,L	up_VCAV_EC
GO:0048382	0.00263	1	2	2	mesendoderm development	BP	1	BMPR1A,ZFP36L1	down_VCAV_EC
GO:0014896	0.00263	1	1	18	muscle hypertrophy	BP	1	GATA5	down_AFEM_VSMC
GO:0014897	0.00263	1	1	18	striated muscle hypertrophy	BP	1	GATA5	down_AFEM_VSMC
GO:0003300	0.00263	1	1	18	cardiac muscle hypertrophy	BP	1	GATA5	down_AFEM_VSMC
GO:0051271	0.00264	0.999	17	98	negative regulation of cellular component movement	BP	0.191	TIE1,ADAMTS9,MEF2C,DLL4,STC1,GATA3,MMP28,ARAP3,ANGPT2,ENSCAFG00000010224,MCC,APOD,JAG1,CTNNA1,HMGB1,ARHGDIB,VASH1	up_VCAV_EC
GO:0072359	0.00264	1	3	368	circulatory system development	BP	1	TGFBI,GATA5,TGFBR1	down_VSAP_VSMC
GO:0008300	0.00264	1	1	1	isoprenoid catabolic process	BP	1	CYP26B1	up_VPOR_VSMC
GO:0016103	0.00264	1	1	1	diterpenoid catabolic process	BP	1	CYP26B1	up_VPOR_VSMC
GO:0016115	0.00264	1	1	1	terpenoid catabolic process	BP	1	CYP26B1	up_VPOR_VSMC
GO:0035981	0.00264	1	1	1	tongue muscle cell differentiation	BP	1	CYP26B1	up_VPOR_VSMC
GO:0042363	0.00264	1	1	1	fat-soluble vitamin catabolic process	BP	1	CYP26B1	up_VPOR_VSMC
GO:0070268	0.00264	1	1	1	cornification	BP	1	CYP26B1	up_VPOR_VSMC
GO:1902809	0.00264	1	1	1	regulation of skeletal muscle fiber	BP	1	CYP26B1	up_VPOR_VSMC
GO:1902811	0.00264	1	1	1	positive regulation of skeletal muscle fiber	BP	1	CYP26B1	up_VPOR_VSMC
GO:2001035	0.00264	1	1	1	regulation of tongue muscle cell differentiation	BP	1	CYP26B1	up_VPOR_VSMC
GO:2001037	0.00264	1	1	1	positive regulation of tongue muscle cell	BP	1	CYP26B1	up_VPOR_VSMC
GO:0034653	0.00264	1	1	1	retinoic acid catabolic process	BP	1	CYP26B1	up_VPOR_VSMC
GO:0030857	0.00266	1	2	15	negative regulation of epithelial cell	BP	0.488	ACVRL1,HOXA7	down_ACOR_EC
GO:0110111	0.00266	1	2	17	negative regulation of animal organ	BP	0.488	SULF1,ASPN	down_ACOR_EC
GO:0050982	0.00266	1	1	9	detection of mechanical stimulus	BP	0.829	JUP	down_VFEM_VSMC
GO:0014068	0.00267	1	7	22	positive regulation of phosphatidylinositol 3-kinase signaling	BP	0.192	TEK,HCLS1,SELP,PDGFB,RELN,PDGFD,RGL2	up_VCAV_EC
GO:0003007	0.00267	1	2	94	heart morphogenesis	BP	1	GATA5,TGFBR1	down_VSAP_VSMC
GO:0048706	0.00268	1	6	59	embryonic skeletal system development	BP	1	TBX15,HOXA9,IRX5,XYLT1,HOXA7,HOXA6	up_AFEM_EC
GO:0006983	0.00268	1	3	5	ER overload response	BP	0.27	HSPA5,DDIT3,GSK3B	down_VSAP_EC
GO:0002444	0.00268	1	2	18	myeloid leukocyte mediated immunity	BP	0.488	LYN,STX4	down_ACOR_EC

GO:0050771	0.00268	1	2	11	negative regulation of axonogenesis	BP	1	SEMA3A,RNF6	up_VPOR_EC
GO:0060745	0.00268	1	1	2	mammary gland branching involved in	BP	0.504	ESR1	up_AORTRT_VSMC
GO:0035295	0.00269	1	2	356	tube development	BP	0.829	JUP,GATA5	down_VFEM_VSMC
GO:2001275	0.002697327	0.99986	3	11	positive regulation of glucose import in response to insulin	BP	0.7913958	NR1H4,ENSCAFG00000004331,RARRES2	up_ACOR_EC
GO:1902533	0.00271	0.999	36	271	positive regulation of intracellular signal transduction	BP	0.195	TEK,HCLS1,IL7R,SELP,KIT,MAP3K11,PDGFB,RELN,TLR4,GPR4,GATA3,EDN1,PELI1,PPP3CA,GUCY1A1,CASP8,MAP3K3,BMP2,ITPKB,MADD,PRKD2,TLR3,AKAP12,NCK1,RAPGEF2,BCL10,TAOK1,HMGB1,PMAIP1,RIPK1,SKIL,PDGFD,ENSCAFG00000002015,RGL2,JCAD,CDON	up_VCAV_EC
GO:0008406	0.00271	1	2	57	gonad development	BP	0.874	HOXA9,IRX5	up_AFEM_VSMC
GO:0045137	0.00271	1	2	57	development of primary sexual characteristics	BP	0.874	HOXA9,IRX5	up_AFEM_VSMC
GO:0032501	0.00273	0.999	15	1782	multicellular organismal process	BP	0.427	HOXC10,TBX15,EYA1,HOXA9,TOX,MAP2K6,MME,AVPR1A,HOXB7,HOXA7,SIX1,MAB21L1,TIAM1,CYP26B1,MTSS1	up_VFEM_EC
GO:0043299	0.00273	1	2	18	leukocyte degranulation	BP	0.488	LYN,STX4	down_ACOR_EC
GO:0086004	0.00274	1	1	10	regulation of cardiac muscle cell contraction	BP	0.829	JUP	down_VFEM_VSMC
GO:0030258	0.00275	0.999	15	71	lipid modification	BP	1	PEX7,ACAA1,ACACB,PEX2,INPP5E,C1QTNF2,PLPP3,PIK3R1,GCDH,EHHADH,LCAT,LONP2,ZFYVE21,PLPP5,SOAT1	up_VSAP_EC
GO:0034112	0.00275	1	1	1	positive regulation of homotypic cell-cell	BP	0.488	PDPN	down_ACOR_EC
GO:1901731	0.00275	1	1	1	positive regulation of platelet aggregation	BP	0.488	PDPN	down_ACOR_EC
GO:1904328	0.00275	1	1	1	regulation of myofibroblast contraction	BP	0.488	PDPN	down_ACOR_EC
GO:1905863	0.00275	1	1	1	invadopodium organization	BP	0.488	PDPN	down_ACOR_EC
GO:1990764	0.00275	1	1	1	myofibroblast contraction	BP	0.488	PDPN	down_ACOR_EC
GO:0010092	0.00275	1	1	19	specification of animal organ identity	BP	1	GATA5	down_AFEM_VSMC
GO:0044283	0.00276	0.999	22	201	small molecule biosynthetic process	BP	0.273	ASNS,PLOD2,NR1H4,ENPP1,PGK1,TPI1,TRIB3,PGAM1,SESN2,PTGS2,HIF1A,SLC45A3,PTPN2,ALDH18A1,ENTPD4,ALDOC,APOE,PLOD3,ATF3,REST,BCAT1,KDM3A	down_VSAP_EC
GO:0010648	0.00276	1	5	407	negative regulation of cell communication	BP	1	BARX1,NKX2-5,BAMBI,ANKRD6,CYP26B1	up_VPOR_VSMC
GO:0008627	0.00276	1	3	5	intrinsic apoptotic signaling pathway in response to osmotic	BP	0.273	ARHGEF2,CASP3,PTGS2	down_VSAP_EC
GO:0003263	0.00276	1	1	4	cardioblast proliferation	BP	1	TBX5	up_APUL_VSMC
GO:0009966	0.00277	0.998	72	868	regulation of signal transduction	BP	0.273	ARHGEF2,PRICKLE1,VEGFA,P4HB,SIPA1L1,SULF1,NR1H4,HSPA5,SERPINE1,SOCS5,PRKACB,ENPP1,ERRFI1,JCAD,SNX25,HYOU1,PTPN11,TRIB3,FARP1,ILK,FHL2,PRCP,SMARCA4,SESN2,PTGS2,MYO1C,CHSY1,UACA,HIF1A,ENSCAFG00000000562,TNIK,NXN,RGS2,PTPN2,NEO1,MTDH,ESM1,ADAM17,CNOT7,MAGED1,CTNNB1,ENSCAFG00000002015,APOE,WWC3,UBA5,LYN,BNIP3,STRAP,DDIT3,FAM162A,PLEKHG2,CBLC,ATF3,GSK3B,CDC37,SH3GLB1,TAX1BP3,HTT,RDH11,ARFGEF3,CALM1,DISC1,PAK2,INHBA,HIF1AN,NRP1,SH3RF3,RHBDF2,TNFAIP1,MADD,SEC13,MID1	down_VSAP_EC
GO:0010595	0.00277	0.999	11	52	positive regulation of endothelial cell migration	BP	0.197	PLK2,TEK,PDGFB,GATA3,PIK3C2A,MAP3K3,PROX1,PRKD2,HMGB1,HIF1A,JCAD	up_VCAV_EC
GO:0003179	0.00277	1	1	19	heart valve morphogenesis	BP	1	GATA5	down_AFEM_VSMC
GO:0086003	0.00277	1	1	10	cardiac muscle cell contraction	BP	0.829	JUP	down_VFEM_VSMC
GO:0003211	0.00278	1	1	4	cardiac ventricle formation	BP	1	TBX5	up_APUL_VSMC
GO:0042670	0.00279	1	2	2	retinal cone cell differentiation	BP	1	THY1,THRB	down_VCAV_EC
GO:0046549	0.00279	1	2	2	retinal cone cell development	BP	1	THY1,THRB	down_VCAV_EC

GO:0048863	0.00279	1	2	70	stem cell differentiation	BP	0.504	WNT7A,ESR1	up_AORTRT_VSMC
GO:0003002	0.0028	1	3	118	regionalization	BP	1	HOXC10,HOXA9,HOXC6	up_AORT_EC
GO:2000027	0.0028	1	2	72	regulation of animal organ morphogenesis	BP	0.504	WNT7A,ESR1	up_AORT_VSMC
GO:0003002	0.0028	1	2	118	regionalization	BP	1	HOXA3,HOXB5	up_APUL_VSMC
GO:0030154	0.00281	0.999	11	1040	cell differentiation	BP	0.707	HOXA11,HOXA9,PITX1,EMX2,SALL1,HOXC10,IRX5,STIM1,CAV1,SHOX2,GJB5	up_VSAP_VSMC
GO:1990009	0.00281	1	2	2	retinal cell apoptotic process	BP	1	AKT2,THRB	down_VCAV_EC
GO:2001233	0.00282	0.999	18	149	regulation of apoptotic signaling pathway	BP	0.275	ARHGEF2,P4HB,SERPINE1,HYOU1,PTGS2,HIF1A,PTPN2,MAGED1,CTNNB1,BNIP3,FAM162A,ATF3,SH3GLB1,HTT,PAK2,INHBA,NRP1,MADD	down_VSAP_EC
GO:0023057	0.00283	1	5	409	negative regulation of signaling	BP	1	BARX1,NKX2-5,BAMBI,ANKRD6,CYP26B1	up_VPOR_VSMC
GO:0060216	0.00283	1	1	3	definitive hemopoiesis	BP	0.504	HOXA9	up_AORT_VSMC
GO:0007275	0.00284	0.999	9	1370	multicellular organism development	BP	1	CHL1,HOXA3,BARX1,NKX2-5,BAMBI,TRPC4,ANKRD6,CYP26B1,C3AR1	up_VPOR_VSMC
GO:0045595	0.00284	0.999	7	469	regulation of cell differentiation	BP	0.44	EYA1,HOXA9,TOX,HOXA7,SIX1,TIAM1,CYP26B1	up_VFEM_EC
GO:0003008	0.00285	1	6	336	system process	BP	0.707	NETO1,GATM,HOXC10,IRX5,CAV1,F5	up_VSAP_VSMC
GO:0006970	0.00285	1	2	23	response to osmotic stress	BP	1	PTGER2,ARHGEF2	up_VPUL_EC
GO:0003156	0.00285	1	1	20	regulation of animal organ formation	BP	1	GATA5	down_AFEM_VSMC
GO:0022616	0.00286	1	4	8	DNA strand elongation	BP	0.17	POLA1,POLE,FEN1,PCNA	down_ACOR_VSMC
GO:0072359	0.00286	1	2	368	circulatory system development	BP	0.829	JUP,GATA5	down_VFEM_VSMC
GO:0009887	0.00287	1	4	314	animal organ morphogenesis	BP	0.771	HOXA3,TBX5,GATA5,HOXB5	up_VPUL_VSMC
GO:0071495	0.00287	1	2	365	cellular response to endogenous stimulus	BP	0.829	JUP,GATA5	down_VFEM_VSMC
GO:1901185	0.00288	1	4	11	negative regulation of ERBB signaling pathway	BP	0.279	SOCS5,ERRFI1,PTPN2,CBLC	down_VSAP_EC
GO:0034113	0.00288	1	1	10	heterotypic cell-cell adhesion	BP	0.829	JUP	down_VFEM_VSMC
GO:0010033	0.00289	0.998	65	772	response to organic substance	BP	0.279	ARHGEF2,VEGFA,SULF1,NR1H4,HSPA5,SERPINE1,SOCS5,HM13,SPARC,ERO1A,HSP90B1,ENPP1,JCAD,SNX25,PTPN11,FLNB,DPYSL3,TRIB3,ILK,FHL2,PRCP,SMARCA4,SESN2,TGFBR1,MYO1C,MSN,SEL1L,KIF5B,HIF1A,CX3CL1,DNAJC10,ZPR1,PTPN2,YES1,NEO1,MTDH,ADAM17,AGRN,CNOT7,DNAJB9,GNAQ,CTNNB1,ENSCAFG00000002015,TFRC,APOE,ZC3H15,KLF5,UBA5,LYN,STRAP,DDIT3,ATF3,CDC37,GBP5,REST,PAK2,CREB3L2,NR4A1,ENSCAFG00000018360,BTK,NRP1,USP14,YWHAH,KDM3A,HOMER1	down_VSAP_EC
GO:0043331	0.00289	1	6	20	response to dsRNA	BP	0.205	TMEM173,RALB,PELI1,RIOK3,TLR3,PMAIP1	up_VCAV_EC
GO:0140074	0.00289	1	1	2	cardiac endothelial to mesenchymal transition	BP	1	GATA5	up_VCAV_VSMC
GO:0007169	0.0029	0.999	21	178	transmembrane receptor protein tyrosine kinase signaling pathway	BP	0.279	VEGFA,SIPA1L1,SULF1,NR1H4,SOCS5,ENPP1,ERRFI1,JCAD,PTPN11,MYO1C,HIF1A,PTPN2,ESM1,ADAM17,CTNNB1,LYN,CBLC,MYO1E,BTK,NRP1,RHBDF2	down_VSAP_EC
GO:0035739	0.0029	1	1	3	CD4-positive, alpha-beta T cell proliferation	BP	0.504	ARG2	up_AORT_VSMC
GO:2000561	0.0029	1	1	3	regulation of CD4-positive, alpha-beta T cell	BP	0.504	ARG2	up_AORT_VSMC
GO:0051890	0.0029	1	1	4	regulation of cardioblast differentiation	BP	1	TBX5	up_APUL_VSMC
GO:0051254	0.00291	0.998	54	431	positive regulation of RNA metabolic process	BP	0.206	HCLS1,PPARG,TNIP1,PDGFB,TMEM173,MEF2C,ATF3,CHD7,TLR4,NLRC5,GATA3,EDN1,TCF4,FOXN2,ENSCAFG00000017103,IRF8,PPP3CA,YES1,MTOR,BMP2,HHEX,CREM,CNOT6,PROX1,ARID4B,HMBOX1,PRKD2,THOC1,SMARCA4,TLR3,NCK1,LDB2,JAG1,NSD1,BCL10,CNOT7,HMGB1,AHR,BLM,RIPK1,ITGA6,GPBP1,MTA2,NR1D2,KDM5A,HIF1A,KDM3A,ATRX,NR4A1,ZEB1,TET2,ARHGEF2,CDON,ZNF609	up_VCAV_EC
GO:0018158	0.00291	1	3	4	protein oxidation	BP	0.71	TMX3,SUMF1,PARK7	up_ACOR_VSMC

GO:0007548	0.00291	1	2	68	sex differentiation	BP	0.504	HOXA9,ESR1	up_AORT_VSMC
GO:0016576	0.00291	1	1	2	histone dephosphorylation	BP	0.917	EYA1	up_AFEM_VSMC
GO:0002275	0.00292	1	2	18	myeloid cell activation involved in immune	BP	0.507	LYN,STX4	down_ACOR_EC
GO:0003274	0.00292	1	1	2	endocardial cushion fusion	BP	1	GATA5	up_VCAV_VSMC
GO:0032989	0.00294	1	5	292	cellular component morphogenesis	BP	1	MYBPC2,CCL24,FLRT3,ARHGEF2,NEO1	up_VPUL_EC
GO:0032501	0.00296	0.999	10	1782	multicellular organismal process	BP	1	HOXC10,DKK2,HOXA9,HOXC6,LNPEP,SGCD,TAOK1,DUSP2,BPI,GPR155	up_AORT_EC
GO:0015669	0.00297	1	1	2	gas transport	BP	1	CA2	up_VCAV_VSMC
GO:0015670	0.00297	1	1	2	carbon dioxide transport	BP	1	CA2	up_VCAV_VSMC
GO:0008593	0.00299	0.999	8	33	regulation of Notch signaling pathway	BP	0.21	DLL4,EGFL7,TSPAN14,BMP2K,GDPD5,MFNG,JAG1,PEAR1	up_VCAV_EC
GO:1900120	0.00299	1	1	7	regulation of receptor binding	BP	1	PLCL1	down_VFEM_EC
GO:0006600	0.00299	1	1	1	creatine metabolic process	BP	0.83	GATM	up_VFEM_VSMC
GO:0006601	0.00299	1	1	1	creatine biosynthetic process	BP	0.83	GATM	up_VFEM_VSMC
GO:0014889	0.00299	1	1	1	muscle atrophy	BP	0.83	GATM	up_VFEM_VSMC
GO:0008203	0.003	0.999	9	37	cholesterol metabolic process	BP	0.177	SQLE,LSS,SREBF1,HMGCR,INSIG1,DHCR24,MVD,FDFT1,LIPC	down_ACOR_VSMC
GO:1905332	0.003	1	2	20	positive regulation of morphogenesis of an	BP	0.462	HOXB7,SIX1	up_VFEM_EC
GO:1900425	0.003	1	1	2	negative regulation of defense response to	BP	0.504	ARG2	up_AORT_VSMC
GO:0042692	0.00301	0.999	18	105	muscle cell differentiation	BP	0.21	EDNRB,KIT,IFRD1,PDGFB,MEF2C,EDN1,TMOD1,PPP3CA,MTOR,BMP2,CAV2,PROX1,ENSCAFG00000010596,ASF1A,P2RX2,TMOD3,ZEB1,CDON	up_VCAV_EC
GO:0018108	0.00301	0.999	12	79	peptidyl-tyrosine phosphorylation	BP	0.285	ARHGEF2,VEGFA,SOCS5,ERRFI1,PTPN2,YES1,ADAM17,CNOT7,LYN,CBLC,BTK,NRP1	down_VSAP_EC
GO:0006909	0.00301	1	5	42	phagocytosis	BP	1	LEP,RAB20,ICAM5,RAB7A,ANXA11	up_AFEM_EC
GO:0097009	0.00301	1	3	13	energy homeostasis	BP	1	DLL1,ACACB,PPARGC1A	up_AFEM_EC
GO:0071609	0.00301	1	1	3	chemokine (C-C motif) ligand 5 production	BP	0.504	ARG2	up_AORT_VSMC
GO:0071649	0.00301	1	1	3	regulation of chemokine (C-C motif) ligand 5	BP	0.504	ARG2	up_AORT_VSMC
GO:0071286	0.00301	1	1	1	cellular response to magnesium ion	BP	0.83	FBP1	up_VFEM_VSMC
GO:0019752	0.00302	0.998	29	283	carboxylic acid metabolic process	BP	0.285	P4HB,ASNS,CARS,PLOD2,NR1H4,ENSCAFG00000009211,ERO1A,P4HA1,SARS,PGK1,AARS,TPI1,TRIB3,PGAM1,ALDH1L2,SESN2,PTGS2,CEMIP2,HIF1A,SLC45A3,NMNAT2,ALDH18A1,YARS,TARS,NARS,ALDOC,PLOD3,RAB11FIP1,BCAT1	down_VSAP_EC
GO:0030168	0.00302	0.999	7	23	platelet activation	BP	0.21	SELP,F8,PDGFB,VWF,TLR4,ITGB3,PEAR1	up_VCAV_EC
GO:0035107	0.00302	1	2	60	appendage morphogenesis	BP	0.917	HOXC10,HOXA9	up_AFEM_VSMC
GO:0035108	0.00302	1	2	60	limb morphogenesis	BP	0.917	HOXC10,HOXA9	up_AFEM_VSMC
GO:0019627	0.00302	1	1	3	urea metabolic process	BP	0.504	ARG2	up_AORT_VSMC
GO:0007267	0.003022562	0.99876	18	332	cell-cell signaling	BP	0.8655851	CNTNAP4,CDH3,NR1H4,BRSK1,PLCL1,SOX9,AQP1,PORCN,KCNB1,FZD5,DIXDC1,INSYN1,MAPK8IP2,TBX5,LRP4,CLSTN1,LRRC4,PYGO2	up_ACOR_EC
GO:0014051	0.00303	1	1	1	gamma-aminobutyric acid secretion	BP	1	TRPC4	up_VPOR_VSMC
GO:0015812	0.00303	1	1	1	gamma-aminobutyric acid transport	BP	1	TRPC4	up_VPOR_VSMC
GO:1901292	0.00304	0.999	7	34	nucleoside phosphate catabolic process	BP	0.285	ENPP1,PGK1,TPI1,PGAM1,HIF1A,ENTPD4,ALDOC	down_VSAP_EC
GO:0030199	0.00305	1	5	21	collagen fibril organization	BP	1	ADAMTS2,LUM,LOX,DDR2,GREM1	down_VCAV_EC
GO:0007389	0.00305	1	4	137	pattern specification process	BP	0.708	HOXA11,HOXA9,EMX2,HOXC10	up_VSAP_VSMC
GO:0072078	0.00306	1	2	33	nephron tubule morphogenesis	BP	0.83	EYA1,HOXA11	up_VFEM_VSMC
GO:0043500	0.00306	1	1	21	muscle adaptation	BP	1	GATA5	down_AFEM_VSMC

GO:0050852	0.00308	0.999	7	26	T cell receptor signaling pathway	BP	0.214	PDE4D,EIF2B2,GATA3,NECTIN2,PRKD2,PLCG2,BCL10	up_VCAV_EC
GO:0048731	0.00308	1	6	1247	system development	BP	0.917	HOXC10,TBX15,EYA1,HOXA9,BEND2,IRX5	up_AFEM_VSMC
GO:0051094	0.00308	0.999	6	388	positive regulation of developmental process	BP	1	NR1H4,TBX5,SLITRK5,WIF1,NPNT,NTRK2	up_APUL_EC
GO:0042886	0.00309	0.998	47	527	amide transport	BP	0.286	PRICKLE1,MYH9,NR1H4,PLK3,HM13,TRAM1,PTPN14,SEC24D,PTPN11,SEC23A,SRP54,MYO1C,ENSCAFG00000031588,SEL1L,HIF1A,SSR1,IWS1,ZPR1,STAM2,SMG7,XPOT,USO1,SRPRA,DDX39B,COPG1,KPNB1,COPB2,SRP72,TFRC,APOE,KPNA4,LYN,GSK3B,GBP5,KPNA1,REST,SCAMP1,RAB11FIP1,KDELR2,AP3S1,CLTC,STX6,CDKN1A,RHBDF2,YWHAH,BLZF1,SEC13	down_VSAP_EC
GO:0042273	0.00309	1	3	35	ribosomal large subunit biogenesis	BP	1	RSL24D1,RPL10L,RRP15	up_VPOR_EC
GO:0048731	0.0031	0.998	44	1247	system development	BP	1	TBX15,HOXA9,IRX3,IRX5,HOXC10,LEP,LBH,XYLT1,ANGPT4,HOXA7,COL8A2,DLL1,CRYAB,F3,VXN,PLXNA4,NEFH,MAB21L1,THRB,LGI4,TMEM100,GREM1,NFE2L1,OVOL1,CAMK2A,HOXA6,CYLD,LUZP1,RECK,PTHLH,TBXA2R,EVI2B,CNNM4,BVES,PTPRB,AXIN2,ACACB,PPARGC1A,CLIC4,LRRN2,GCNT4,FOSL2,KANK1,TNC	up_AFEM_EC
GO:0009952	0.0031	1	3	66	anterior/posterior pattern specification	BP	0.526	HOXA3,HOXA7,HOXC4	down_ACOR_EC
GO:0048743	0.0031	1	1	1	positive regulation of skeletal muscle fiber	BP	0.83	SHOX2	up_VFEM_VSMC
GO:0038127	0.00311	0.999	7	32	ERBB signaling pathway	BP	0.286	SOCS5,ERRFI1,PTPN11,PTPN2,ADAM17,CBLC,RHBDF2	down_VSAP_EC
GO:0001708	0.00311	1	2	20	cell fate specification	BP	0.708	HOXA11,HOXC10	up_VSAP_VSMC
GO:0048872	0.00312	0.999	15	85	homeostasis of number of cells	BP	0.215	HCLS1,IL7R,ADGRF5,KIT,TNFAIP3,MEF2C,GATA3,ITPKB,EXOC5,BCL10,VPS54,HMGB1,SKIL,HIF1A,TMOD3	up_VCAV_EC
GO:0034654	0.00312	1	6	1236	nucleobase-containing compound biosynthetic	BP	0.917	TLR2,TBX15,EYA1,HOXA9,HOXA10,IRX5	up_AFEM_VSMC
GO:0060341	0.003126791	0.99883	15	255	regulation of cellular localization	BP	0.8655851	SH3TC2,CDH3,NR1H4,KCNB1,EDEM2,PLS1,ACTN2,TRIM8,PREB,DCLK2,MAVS,LRRC15,LRP4,TP53INP2,PRKCD	up_ACOR_EC
GO:0031175	0.00313	0.999	25	224	neuron projection development	BP	0.286	SIPA1L1,SGK1,PTPN11,DPYSL3,IFRD1,KIF5B,TNIK,UBA6,ZPR1,NEO1,UST,CDH11,CTNNB1,APOE,LYN,GSK3B,VLDLR,SDC4,PRMT1,EXT1,DISC1,PAK2,NRP1,ARF4,YWHAH	down_VSAP_EC
GO:0001782	0.00313	1	4	8	B cell homeostasis	BP	0.215	TNFAIP3,MEF2C,BCL10,HIF1A	up_VCAV_EC
GO:0003203	0.00314	1	1	11	endocardial cushion morphogenesis	BP	0.859	GATA5	down_VFEM_VSMC
GO:0042742	0.003149619	0.99955	5	39	defense response to bacterium	BP	0.8655851	NR1H4,NOD2,MAVS,RARRES2,PRKCD	up_ACOR_EC
GO:2000418	0.00315	1	1	1	positive regulation of eosinophil migration	BP	1	CCL24	up_VPUL_EC
GO:0006812	0.00316	0.998	34	222	cation transport	BP	0.71	FGF13,ACTN2,PLCG2,KCNE3,HTR2B,TPC3,NIPAL3,SARAF,PTPN3,MICU2,ITPR1,DAPK1,CACNB3,AKAP6,DMPK,ATP7A,KCNJ4,PRKCE,PSEN1,CHD7,ATP6V0E1,RAB11B,SLC25A20,KCNC4,LMTK2,B2M,FXYD1,INPP5K,WFS1,ATP2C1,NDUFA4L2,PARK7,SLC8A1,STC2	up_ACOR_VSMC
GO:0048640	0.00316	0.999	7	21	negative regulation of developmental growth	BP	0.71	BMP4,PLAC8B,NRP1,ULK2,TBX5,WWC2,STC2	up_ACOR_VSMC
GO:0014850	0.00316	1	3	4	response to muscle activity	BP	0.71	FNDC5,PRKAA2,PPARGC1A	up_ACOR_VSMC
GO:0051094	0.00316	1	2	388	positive regulation of developmental process	BP	0.859	JUP,GATA5	down_VFEM_VSMC
GO:0033002	0.003164747	0.99943	6	55	muscle cell proliferation	BP	0.8655851	NDRG2,PTGIR,FOXC2,TBX5,CLSTN1,C23H3orf58	up_ACOR_EC
GO:0072358	0.00317	0.998	28	268	cardiovascular system development	BP	0.287	PRICKLE1,VEGFA,SULF1,MYH9,SERPINE1,SPARC,LTBP1,PTPN14,ERRFI1,JCAD,PGK1,TMED2,PRCP,PIK3C2A,HIF1A,THBS2,NXN,MTDH,ESM1,ACTA2,ADAMTS6,CTNNB1,STARD13,APOE,KLF5,DDIT3,MYO1E,NRP1	down_VSAP_EC
GO:2001135	0.00317	1	3	4	regulation of endocytic recycling	BP	0.71	ACTN2,RAB11B,BVES	up_ACOR_VSMC
GO:1904468	0.00317	1	1	3	negative regulation of tumor necrosis factor	BP	0.504	ARG2	up_AORT_VSMC
GO:0035270	0.00318	1	2	35	endocrine system development	BP	1	HOXA3,NKX2-5	up_VPOR_VSMC
GO:0061034	0.00318	1	1	1	olfactory bulb mitral cell layer development	BP	0.708	SALL1	up_VSAP_VSMC

GO:0032874	0.00319	0.999	13	57	positive regulation of stress-activated MAPK cascade	BP	0.71	LEP,MID1,RASSF2,TLR3,SH3RF2,SASH1,F2RL1,APP,DUSP22,EDN1,NOD2,SH3RF3,MAP3K7	up_ACOR_VSMC
GO:1905114	0.00319	1	4	150	cell surface receptor signaling pathway involved in cell-cell	BP	0.708	NETO1,SALL1,TLR2,CAV1	up_VSAP_VSMC
GO:0038083	0.00319	1	2	16	peptidyl-tyrosine autophosphorylation	BP	0.528	LYN,IGF1R	down_ACOR_EC
GO:0021796	0.00319	1	1	1	cerebral cortex regionalization	BP	0.83	EMX2	up_VFEM_VSMC
GO:0048645	0.0032	1	2	33	animal organ formation	BP	0.83	EYA1,HOXA11	up_VFEM_VSMC
GO:0030097	0.00321	1	5	244	hemopoiesis	BP	0.488	HOXA9,TOX,HOXB7,HOXA7,CYP26B1	up_VFEM_EC
GO:0090385	0.00322	1	2	4	phagosome-lysosome fusion	BP	1	RAB20,RAB7A	up_AFEM_EC
GO:1903115	0.00322	1	1	12	regulation of actin filament-based movement	BP	0.859	JUP	down_VFEM_VSMC
GO:1902904	0.003221181	0.99954	5	38	negative regulation of supramolecular fiber	BP	0.86633664	TMOD1,ARHGAP6,ARHGAP28,PRKCD,TUBB4A	up_ACOR_EC
GO:0040013	0.00323	0.999	17	100	negative regulation of locomotion	BP	0.22	TIE1,ADAMTS9,MEF2C,DLL4,STC1,GATA3,MMP28,ARAP3,ANGPT2,ENSCAFG00000010224,MCC,APOD,JAG1,CTNNA1,HMGB1,ARHGDIB,VASH1	up_VCAV_EC
GO:0001547	0.00323	1	1	3	antral ovarian follicle growth	BP	0.504	ESR1	up_AORT_VSMC
GO:0071391	0.00323	1	1	3	cellular response to estrogen stimulus	BP	0.504	ESR1	up_AORT_VSMC
GO:0072132	0.00324	1	1	23	mesenchyme morphogenesis	BP	1	GATA5	down_AFEM_VSMC
GO:0072611	0.00324	1	1	3	interleukin-13 secretion	BP	0.504	ARG2	up_AORT_VSMC
GO:2000665	0.00324	1	1	3	regulation of interleukin-13 secretion	BP	0.504	ARG2	up_AORT_VSMC
GO:0061737	0.00324	1	1	1	leukotriene signaling pathway	BP	1	RGS1	up_VPUL_EC
GO:0003206	0.00325	0.999	12	51	cardiac chamber morphogenesis	BP	0.71	TNNT2,SMAD6,GATA4,BMP4,FGFR2,CITED2,EGLN1,NRP1,SMARCD3,TBX20,TBX5,CHD7	up_ACOR_VSMC
GO:0016054	0.00325	0.999	9	61	organic acid catabolic process	BP	1	AKT2,ACAA1,THNSL2,PEX5,ENSCAFG00000002977,LONP2,GLS2,BCKDHB,ACAT1	down_VCAV_EC
GO:0046395	0.00325	0.999	9	61	carboxylic acid catabolic process	BP	1	AKT2,ACAA1,THNSL2,PEX5,ENSCAFG00000002977,LONP2,GLS2,BCKDHB,ACAT1	down_VCAV_EC
GO:0002064	0.00325	1	2	71	epithelial cell development	BP	0.504	WNT7A,ESR1	up_AORT_VSMC
GO:0010033	0.00326	0.999	8	772	response to organic substance	BP	1	NR1H4,MRC1,CCL24,PTGER2,FLRT3,ARHGEF2,NEO1,MXRA5	up_VPUL_EC
GO:0032735	0.00326	1	4	9	positive regulation of interleukin-12 production	BP	0.221	TLR4,IRF8,TLR3,HMGB1	up_VCAV_EC
GO:0051239	0.00327	0.999	7	859	regulation of multicellular organismal process	BP	0.83	HOXC10,EYA1,GATM,HOXA9,NETO1,HOXA11,SHOX2	up_VFEM_VSMC
GO:0010611	0.00327	1	1	11	regulation of cardiac muscle hypertrophy	BP	0.859	GATA5	down_VFEM_VSMC
GO:0014743	0.00327	1	1	11	regulation of muscle hypertrophy	BP	0.859	GATA5	down_VFEM_VSMC
GO:0010819	0.00328	1	2	4	regulation of T cell chemotaxis	BP	1	CCL5,ADAM17	up_AFEM_EC
GO:0010820	0.00328	1	2	4	positive regulation of T cell chemotaxis	BP	1	CCL5,ADAM17	up_AFEM_EC
GO:0021533	0.00328	1	1	3	cell differentiation in hindbrain	BP	0.504	WNT7A	up_AORT_VSMC
GO:0060737	0.00328	1	1	3	prostate gland morphogenetic growth	BP	0.504	ESR1	up_AORT_VSMC
GO:0060525	0.00329	1	1	3	prostate glandular acinus development	BP	0.504	ESR1	up_AORT_VSMC
GO:0060526	0.00329	1	1	3	prostate glandular acinus morphogenesis	BP	0.504	ESR1	up_AORT_VSMC
GO:0060527	0.00329	1	1	3	prostate epithelial cord arborization involved in prostate glandular acinus	BP	0.504	ESR1	up_AORT_VSMC
GO:0010810	0.00331	1	3	60	regulation of cell-substrate adhesion	BP	0.528	ACVRL1,PDPN,HOXA7	down_ACOR_EC
GO:0060837	0.00331	1	2	4	blood vessel endothelial cell differentiation	BP	1	DLL1,TMEM100	up_AFEM_EC
GO:0060914	0.00331	1	1	11	heart formation	BP	0.859	GATA5	down_VFEM_VSMC
GO:1904861	0.00331	1	1	3	excitatory synapse assembly	BP	0.504	WNT7A	up_AORT_VSMC
GO:0034969	0.00333	1	2	2	histone arginine methylation	BP	0.294	NR1H4,PRMT1	down_VSAP_EC

GO:0048263	0.00333	1	2	4	determination of dorsal identity	BP	1	GREM1,GREM2	up_AFEM_EC
GO:0009888	0.00334	0.998	49	545	tissue development	BP	0.294	PRICKLE1,SULF1,SERPINH1,TAGLN2,NR1H4,SERPINE1,ACTN1,PRKACB,PTK7,ERRFI1,ITGA2,CASP3,PGK1,EXOC5,FLNB,ENSCAFG00000032470,IFRD1,TMED2,ILK,FHL2,TGFBR1,PTGS2,WDR1,CHSY1,MSN,HOXB7,HIF1A,SLC20A1,ACTG2,ADAM17,ACTA2,MAGED1,CTNNB1,STARD13,PLOD3,KLF5,STRAP,ATF3,GSK3B,NEXN,MYO1E,SDC4,EXT1,CREB3L2,INHBA,SIX4,NRP1,COL12A1,HOMER1	down_VSAP_EC
GO:0075525	0.00334	1	3	4	viral translational termination-reinitiation	BP	1	EIF3G,EIF3L,EIF3D	up_VSAP_EC
GO:0001656	0.00334	1	2	35	metanephros development	BP	0.83	EYA1,HOXA11	up_VFEM_VSMC
GO:0061333	0.00334	1	2	35	renal tubule morphogenesis	BP	0.83	EYA1,HOXA11	up_VFEM_VSMC
GO:0009719	0.00335	1	2	395	response to endogenous stimulus	BP	0.859	JUP,GATA5	down_VFEM_VSMC
GO:0003156	0.00335	1	2	20	regulation of animal organ formation	BP	0.505	EYA1,SIX1	up_VFEM_EC
GO:0021683	0.00335	1	1	3	cerebellar granular layer morphogenesis	BP	0.505	WNT7A	up_AORT_VSMC
GO:0055062	0.00335	1	1	5	phosphate ion homeostasis	BP	1	HOXA3	up_APUL_VSMC
GO:0072505	0.00335	1	1	5	divalent inorganic anion homeostasis	BP	1	HOXA3	up_APUL_VSMC
GO:0072506	0.00335	1	1	5	trivalent inorganic anion homeostasis	BP	1	HOXA3	up_APUL_VSMC
GO:0002366	0.00336	0.999	13	65	leukocyte activation involved in immune response	BP	0.226	KIT,IL6,TLR4,GATA3,RAB27A,MTOR,SNAP23,THOC1,SWAP70,MFNG,FES,PLCG2,HMGB1	up_VCAV_EC
GO:0014902	0.00336	1	2	34	myotube differentiation	BP	1	NKX2-5,CYP26B1	up_VPOR_VSMC
GO:0051024	0.00336	1	1	1	positive regulation of immunoglobulin secretion	BP	0.528	STX4	down_ACOR_EC
GO:0032501	0.00337	0.998	133	1782	multicellular organismal process	BP	0.295	ARHGEF2,PRICKLE1,VEGFA,SIPA1L1,SULF1,CELF1,SERPINH1,MYH9,NR1H4,HSPA5,SERPINE1,ACTN1,SOCS5,ETV6,SPARC,LTBP1,SGK1,PRKACB,PTPN14,ERRFI1,SLC1A4,ITGA2,SEC24D,JCAD,TPM4,CASP3,FAM49B,PGK1,BCAR3,PTPN11,EXOC5,AARS,FLNB,ENSCAFG00000032470,DPYSL3,HOOK3,IFRD1,TMED2,ILK,FHL2,BHLHE40,PRCP,SMARCA4,TGFBR1,PTGS2,WDR1,PIK3C2A,CHSY1,MSN,HOXB7,RPGR,KIF5B,HIF1A,HTR7,SLC45A3,SLC20A1,THBS2,ACTG2,TNIK,NXN,UBA6,ZPR1,PTPN2,UCHL5,PHC2,EIF2B1,EVI2B,NEO1,PLCL1,MTDH,ESM1,ADAM17,ACTA2,ADAMTS6,UST,CDH11,MAGED1,CTNNB1,ANLN,CRYAB,STARD13,TFRC,APOE,WWC3,PLOD3,KLF5,UBA5,LYN,STRAP,DDIT3,PDS5B,CCNYL1,MMD,PLS3,SLC7A11,ATF3,GSK3B,VLDLR,NEXN,ITGB3,GBP5,MYO1E,SDC4,HTT,REST,RBBP8,MAPK10,PRMT1,SH2B3,P3H4,EXT1,CALM1,RAB11FIP1,GLO1,DISC1,CDYL,PAK2,CREB3L2,LRRTM2,INHBA,CAMK2D,BTK,SIX4,NRP1,SPINK2,ARF4,YWHAH,COL12A1,RTCB,SNRPA1,KDM3A,MYBPC2,HOMER1	down_VSAP_EC
GO:0044087	0.00338	0.998	43	339	regulation of cellular component biogenesis	BP	0.226	EPHB1,PLK2,TEK,HCLS1,GBP5,SELP,PRKCH,ARHGEF15,PODXL,BAIAP2L1,MEF2C,RAPGEF1,TLR4,EDN1,RALB,SNCAIP,IRF8,ARHGAP28,TMOD1,MTOR,SAMD8,SLK,CNOT6,NCKAP1,RIOK3,PROX1,IQGAP2,STMN1,SH3GLB1,SWAP70,APOD,NCK1,LDB2,FES,RAPGEF2,SENP6,PAN3,HMGB1,PMAIP1,ARPC5L,ENSCAFG00000002015,TMOD3,FSCN1	up_VCAV_EC
GO:0051216	0.00339	1	3	70	cartilage development	BP	0.708	HOXA11,PITX1,SHOX2	up_VSAP_VSMC
GO:1901228	0.00339	1	1	4	positive regulation of transcription from RNA polymerase II promoter involved in heart	BP	1	GATA5	down_VSAP_VSMC
GO:0006808	0.0034	1	1	1	regulation of nitrogen utilization	BP	1	NR1H4	up_VPUL_EC
GO:0019676	0.0034	1	1	1	ammonia assimilation cycle	BP	1	NR1H4	up_VPUL_EC
GO:0019740	0.0034	1	1	1	nitrogen utilization	BP	1	NR1H4	up_VPUL_EC

GO:0034255	0.0034	1	1	1	regulation of urea metabolic process	BP	1	NR1H4	up_VPUL_EC
GO:0034971	0.0034	1	1	1	histone H3-R17 methylation	BP	1	NR1H4	up_VPUL_EC
GO:0038183	0.0034	1	1	1	bile acid signaling pathway	BP	1	NR1H4	up_VPUL_EC
GO:0038185	0.0034	1	1	1	intracellular bile acid receptor signaling	BP	1	NR1H4	up_VPUL_EC
GO:0045848	0.0034	1	1	1	positive regulation of nitrogen utilization	BP	1	NR1H4	up_VPUL_EC
GO:1903314	0.0034	1	1	1	regulation of nitrogen cycle metabolic process	BP	1	NR1H4	up_VPUL_EC
GO:1905693	0.0034	1	1	1	regulation of phosphatidic acid biosynthetic process	BP	1	NR1H4	up_VPUL_EC
GO:1905695	0.0034	1	1	1	positive regulation of phosphatidic acid biosynthetic process	BP	1	NR1H4	up_VPUL_EC
GO:2000211	0.0034	1	1	1	regulation of glutamate metabolic process	BP	1	NR1H4	up_VPUL_EC
GO:2000213	0.0034	1	1	1	positive regulation of glutamate metabolic	BP	1	NR1H4	up_VPUL_EC
GO:2001248	0.0034	1	1	1	regulation of ammonia assimilation cycle	BP	1	NR1H4	up_VPUL_EC
GO:2001250	0.0034	1	1	1	positive regulation of ammonia assimilation	BP	1	NR1H4	up_VPUL_EC
GO:0071380	0.00341	1	4	7	cellular response to prostaglandin E stimulus	BP	0.731	PRKAA2,ADCY6,PTGDR,PRKCE	up_ACOR_VSMC
GO:0071294	0.00342	1	2	4	cellular response to zinc ion	BP	1	MT2A,ENSCAFG00000009113	up_AFEM_EC
GO:0003218	0.00342	1	1	1	cardiac left ventricle formation	BP	1	TBX5	up_VPUL_EC
GO:0048562	0.00343	0.999	19	102	embryonic organ morphogenesis	BP	1	TBX15,HOXA11,HOXA7,WNT11,OSR2,INSIG2,LRIG3,IRX5,HOXD3,DLL1,HOXA6,OSR1,RARG,HOXC4,ZEB1,SHOX2,KDM2B,TGFBR2,CITED2	up_VSAP_EC
GO:0042118	0.00343	1	1	4	endothelial cell activation	BP	1	TGFBR1	down_VSAP_VSMC
GO:2000377	0.00344	0.999	9	41	regulation of reactive oxygen species metabolic	BP	0.228	PRCP,PDGFB,CD34,TLR4,RAB27A,ARG2,GCH1,RIPK1,HIF1A	up_VCAV_EC
GO:0019438	0.00344	1	6	1258	aromatic compound biosynthetic process	BP	0.926	TLR2,TBX15,EYA1,HOXA9,HOXA10,IRX5	up_AFEM_VSMC
GO:0061138	0.00344	1	3	73	morphogenesis of a branching epithelium	BP	0.507	EYA1,HOXB7,SIX1	up_VFEM_EC
GO:0021536	0.00344	1	2	21	diencephalon development	BP	0.708	PITX1,SALL1	up_VSAP_VSMC
GO:0032228	0.00344	1	1	8	regulation of synaptic transmission, GABAergic	BP	1	PLCL1	down_VFEM_EC
GO:1905314	0.00344	1	1	12	semi-lunar valve development	BP	0.867	GATA5	down_VFEM_VSMC
GO:2000027	0.00345	1	3	72	regulation of animal organ morphogenesis	BP	0.507	EYA1,HOXB7,SIX1	up_VFEM_EC
GO:0051704	0.003452041	0.9984	23	476	multi-organism process	BP	0.91320638	NR1H4,SOX9,HMCN1,RGS1,ATG16L2,AQP1,FZD5,PTGIR,NOD2,TRIM8,PTGER4,ARHGDIB,MAPK8IP2,MAVS,FIGNL1,LRRC15,IFIT2,CHMP4B,RARRES2,AGBL5,PRKCD,PYGO2,CCNYL1	up_ACOR_EC
GO:0019229	0.00346	1	5	12	regulation of vasoconstriction	BP	0.731	ADRA1B,HRH1,HRH2,EDN1,DOCK5	up_ACOR_VSMC
GO:0060749	0.00346	1	1	3	mammary gland alveolus development	BP	0.513	ESR1	up_AORT_VSMC
GO:0061377	0.00346	1	1	3	mammary gland lobule development	BP	0.513	ESR1	up_AORT_VSMC
GO:0032501	0.00347	1	7	1782	multicellular organismal process	BP	0.926	HOXC10,TLR2,TBX15,EYA1,HOXA9,BEND2,IRX5	up_AFEM_VSMC
GO:0009617	0.00348	0.998	20	133	response to bacterium	BP	0.228	GBP5,LYZ,CXCL8,TNFAIP3,ADAMTS9,TNIP1,BAIAP2L1,PDE4D,MEF2C,CHD7,TLR4,NLRC5,IRF8,PELI1,ARG2,ABCA1,PLCG2,BCL10,GCH1,NOS2	up_VCAV_EC
GO:0030282	0.00348	1	3	37	bone mineralization	BP	1	HOXA3,ASPN,ACVR2A	up_VPOR_EC
GO:0030010	0.00348	1	2	41	establishment of cell polarity	BP	1	GPSM2,FSCN1	down_AFEM_EC
GO:0051240	0.00349	1	4	489	positive regulation of multicellular organismal	BP	1	SLITRK5,LIMK1,GATA5,C6	up_VCAV_VSMC
GO:0007634	0.00349	1	1	1	optokinetic behavior	BP	0.528	HOXA1	down_ACOR_EC
GO:0021560	0.00349	1	1	1	abducens nerve development	BP	0.528	HOXA1	down_ACOR_EC
GO:0021598	0.00349	1	1	1	abducens nerve morphogenesis	BP	0.528	HOXA1	down_ACOR_EC

GO:0021599	0.00349	1	1	1	abducens nerve formation	BP	0.528	HOXA1	down_ACOR_EC
GO:0099537	0.0035	0.998	23	131	trans-synaptic signaling	BP	0.731	PLCL1,FBXL20,HRH1,GLRB,STX11,TMEM108,EGLN1,CACNB3,SLC12A7,DMPK,SLITRK5,PRKCE,RNF19A,DAG1,APP,PSEN1,EDN1,KCNC4,LRRC4,FAM107A,INSYN1,PREPL,PARK7	up_ACOR_VSMC
GO:0018130	0.0035	1	6	1261	heterocycle biosynthetic process	BP	0.926	TLR2,TBX15,EYA1,HOXA9,HOXA10,IRX5	up_AFEM_VSMC
GO:0003264	0.0035	1	1	5	regulation of cardioblast proliferation	BP	1	TBX5	up_APUL_VSMC
GO:0003266	0.0035	1	1	5	regulation of secondary heart field cardioblast	BP	1	TBX5	up_APUL_VSMC
GO:0071724	0.0035	1	1	1	response to diacyl bacterial lipopeptide	BP	0.708	TLR2	up_VSAP_VSMC
GO:0071726	0.0035	1	1	1	cellular response to diacyl bacterial lipopeptide	BP	0.708	TLR2	up_VSAP_VSMC
GO:1903971	0.0035	1	1	1	positive regulation of response to macrophage colony-stimulating factor	BP	0.708	TLR2	up_VSAP_VSMC
GO:1903974	0.0035	1	1	1	positive regulation of cellular response to macrophage colony-	BP	0.708	TLR2	up_VSAP_VSMC
GO:0042496	0.0035	1	1	1	detection of diacyl bacterial lipopeptide	BP	0.708	TLR2	up_VSAP_VSMC
GO:0044255	0.00351	0.998	43	293	cellular lipid metabolic process	BP	1	PEX7,NAPEPLD,ACSM3,LIPC,INSIG2,ENPP2,ACAA1,ACACB,PEX2,INPP5E,CERS4,DHRS4,KDELC2,C1QTNF2,ABHD15,HSD17B8,PLPP3,ENSCAFG00000000071,FAAH,AMBRA1,LMF1,PDK2,RETSAT,PIK3R1,PLA2G6,PIK3IP1,PTGES,HACD4,GCDH,DAGLB,EHHADH,LCAT,LONP2,DHRS9,MVK,ABHD16A,HPGD,ZFYVE21,ENSCAFG00000023820,IDH1,PLPP5,B3GALT4,SOAT1	up_VSAP_EC
GO:0090075	0.00351	1	4	8	relaxation of muscle	BP	0.23	PDE4D,GUCY1A1,KCNJ2,ATP2A1	up_VCAV_EC
GO:0072088	0.00352	1	2	36	nephron epithelium morphogenesis	BP	0.861	EYA1,HOXA11	up_VFEM_VSMC
GO:0003345	0.00352	1	1	1	proepicardium cell migration involved in pericardium	BP	1	FLRT3	up_VPUL_EC
GO:0006096	0.00353	1	5	18	glycolytic process	BP	0.302	PGK1,TPI1,PGAM1,HIF1A,ALDOC	down_VSAP_EC
GO:0006165	0.00353	1	5	18	nucleoside diphosphate phosphorylation	BP	0.302	PGK1,TPI1,PGAM1,HIF1A,ALDOC	down_VSAP_EC
GO:0006757	0.00353	1	5	18	ATP generation from ADP	BP	0.302	PGK1,TPI1,PGAM1,HIF1A,ALDOC	down_VSAP_EC
GO:0042866	0.00353	1	5	18	pyruvate biosynthetic process	BP	0.302	PGK1,TPI1,PGAM1,HIF1A,ALDOC	down_VSAP_EC
GO:0031214	0.00354	1	2	45	biomineral tissue development	BP	0.798	HOXA3,PHEX	up_VPUL_VSMC
GO:0002027	0.00354	1	2	20	regulation of heart rate	BP	0.708	IRX5,CAV1	up_VSAP_VSMC
GO:2001273	0.003547365	0.99979	3	12	regulation of glucose import in response to	BP	0.92328747	NR1H4,ENSCAFG00000004331,RARRES2	up_ACOR_EC
GO:0048705	0.00355	0.999	18	95	skeletal system morphogenesis	BP	1	TBX15,HOXA11,HOXA7,PEX7,AXIN2,OSR2,INSIG2,PPARGC1B,IRX5,HOXD3,HOXA6,OSR1,RARG,ZEB1,SHOX2,ZFAND5,TGFBR2,CITED2	up_VSAP_EC
GO:0034755	0.00355	1	3	4	iron ion transmembrane transport	BP	1	SLC25A28,SCARA5,STEAP3	up_VSAP_EC
GO:0003207	0.00355	1	1	5	cardiac chamber formation	BP	1	TBX5	up_APUL_VSMC
GO:0090183	0.00356	1	2	21	regulation of kidney development	BP	0.517	HOXB7,SIX1	up_VFEM_EC
GO:0045165	0.00357	1	2	65	cell fate commitment	BP	0.926	HOXC10,EYA1	up_AFEM_VSMC
GO:0048704	0.00359	0.999	11	46	embryonic skeletal system morphogenesis	BP	1	TBX15,HOXA11,HOXA7,OSR2,IRX5,HOXD3,HOXA6,OSR1,ZEB1,SHOX2,TGFBR2	up_VSAP_EC
GO:0061061	0.0036	0.998	26	179	muscle structure development	BP	0.234	EPHB1,EDNRB,KIT,IFRD1,PDGFB,MEF2C,ATF3,CHD7,DLL4,EDN1,FOXN2,TMOD1,PPP3CA,MTOR,BMP2,CAV2,PROX1,ENSCAFG00000010596,FLNB,CREB1,ASF1A,NR1D2,P2RX2,TMOD3,ZEB1,CDON	up_VCAV_EC
GO:0006351	0.0036	0.999	11	1069	transcription, DNA-templated	BP	0.708	HOXA11,TBX15,HOXA10,HOXA9,PITX1,EMX2,SALL1,IRX5,TLR2,CAV1,SHOX2	up_VSAP_VSMC
GO:0048736	0.0036	1	2	66	appendage development	BP	0.926	HOXC10,HOXA9	up_AFEM_VSMC
GO:0060173	0.0036	1	2	66	limb development	BP	0.926	HOXC10,HOXA9	up_AFEM_VSMC

GO:0032330	0.0036	1	2	20	regulation of chondrocyte differentiation	BP	0.708	HOXA11,SHOX2	up_VSAP_VSMC
GO:0010812	0.00361	1	2	19	negative regulation of cell-substrate adhesion	BP	0.528	ACVRL1,HOXA7	down_ACOR_EC
GO:0003170	0.00362	1	1	25	heart valve development	BP	1	GATA5	down_AFEM_VSMC
GO:0008380	0.00363	1	3	149	RNA splicing	BP	1	IVNS1ABP,SRSF6,TSEN15	down_AFEM_EC
GO:1903715	0.00363	1	2	2	regulation of aerobic respiration	BP	0.309	HIF1A,BNIP3	down_VSAP_EC
GO:0048598	0.00364	0.999	12	207	embryonic morphogenesis	BP	1	TBX15,HOXA9,IRX3,IRX5,HOXC10,HOXA7,DLL1,GREM1,GREM2,HOXA6,LUZP1,RECK	up_AFEM_EC
GO:0043536	0.00364	0.999	8	32	positive regulation of blood vessel endothelial	BP	0.236	PLK2,PDGFB,PIK3C2A,MAP3K3,PRKD2,HMGB1,HIF1A,JCAD	up_VCAV_EC
GO:0007389	0.00364	1	4	137	pattern specification process	BP	0.528	HOXA3,NRP2,HOXA7,HOXC4	down_ACOR_EC
GO:0048704	0.00364	1	2	46	embryonic skeletal system morphogenesis	BP	0.798	HOXA3,HOXB5	up_VPUL_VSMC
GO:0050793	0.00365	0.998	29	731	regulation of developmental process	BP	1	HOXA9,CMKLR1,IRX3,LEP,LBH,ANGPT4,HOXA7,DLL1,F3,PLXNA4,GPAM,TWIST2,THRB,LGI4,TMEM100,GREM1,CAMK2A,CYLD,PTHLH,TBXA2R,EVI2B,BVES,AXIN2,ACACB,NAPEPLD,PPARGC1A,CDC42EP5,DMPK,KANK1	up_AFEM_EC
GO:2000026	0.00365	1	5	560	regulation of multicellular organismal development	BP	0.798	TBX5,GATA5,TMEM176A,TMEM176B,SLIT2	up_VPUL_VSMC
GO:0048534	0.00365	1	4	262	hematopoietic or lymphoid organ	BP	1	HOXA3,BARX1,NKX2-5,CYP26B1	up_VPOR_VSMC
GO:0061156	0.00365	1	3	4	pulmonary artery morphogenesis	BP	0.735	BMP4,SEC24B,CITED2	up_ACOR_VSMC
GO:0010634	0.00366	0.999	13	68	positive regulation of epithelial cell migration	BP	0.236	PLK2,TEK,PDGFB,GATA3,RAB11A,PIK3C2A,MTOR,MAP3K3,PROX1,PRKD2,HMGB1,HIF1A,JCAD	up_VCAV_EC
GO:0006641	0.00367	0.999	7	25	triglyceride metabolic process	BP	0.212	SREBF1,CTDNEP1,INSIG1,CAV1,ABHD5,LIPC,GK5	down_ACOR_VSMC
GO:0051382	0.00367	1	4	9	kinetochore assembly	BP	0.212	CENPA,CENPN,CENPH,CENPW	down_ACOR_VSMC
GO:0055059	0.00367	1	1	1	asymmetric neuroblast division	BP	1	ARHGEF2	up_VPUL_EC
GO:0018212	0.00368	0.999	12	81	peptidyl-tyrosine modification	BP	0.311	ARHGEF2,VEGFA,SOCS5,ERRFI1,PTPN2,YES1,ADAM17,CNOT7,LYN,CBLC,BTK,NRP1	down_VSAP_EC
GO:0097659	0.00369	0.999	11	1072	nucleic acid-templated transcription	BP	0.714	HOXA11,TBX15,HOXA10,HOXA9,PITX1,EMX2,SALL1,IRX5,TLR2,CAV1,SHOX2	up_VSAP_VSMC
GO:1902652	0.00369	0.999	9	38	secondary alcohol metabolic process	BP	0.213	SQLE,LSS,SREBF1,HMGCR,INSIG1,DHCR24,MVD,FDFT1,LIPC	down_ACOR_VSMC
GO:0070304	0.00371	0.999	13	58	positive regulation of stress-activated protein kinase signaling cascade	BP	0.735	LEP,MID1,RASSF2,TLR3,SH3RF2,SASH1,F2RL1,APP,DUSP22,EDN1,NOD2,SH3RF3,MAP3K7	up_ACOR_VSMC
GO:0021520	0.00371	1	1	3	spinal cord motor neuron cell fate specification	BP	0.53	HOXC10	up_AORT_VSMC
GO:0019220	0.00372	0.998	61	525	regulation of phosphate metabolic process	BP	0.238	EPHB1,TEK,HCLS1,ADGRF5,KIT,PPP2R5A,CDA,TNFAIP3,MAP3K11,TNIP1,PDGFB,STK10,RELN,PDE4D,MEF2C,ATF3,RAPGEF1,TLR4,NLRC5,EDN1,RALB,APLNR,NEDD9,YES1,MTOR,MAP3K3,BMP2,HHEX,PREX2,SLK,SH3BP5,PPP1R2,ITPKB,PROX1,MADD,PAK2,PRKD2,SH3GLB1,SWAP70,TLR3,NCK1,LDB2,NSD1,PTGS2,MMD,RAPGEF2,TAOK1,CNOT7,HMGB1,NOS2,BLM,SPTBN4,RIPK1,ITGA6,RAD17,PDGFD,HIF1A,FKBP15,JCAD,ARHGEF2,CDON	up_VCAV_EC
GO:0051174	0.00372	0.998	61	525	regulation of phosphorus metabolic process	BP	0.238	EPHB1,TEK,HCLS1,ADGRF5,KIT,PPP2R5A,CDA,TNFAIP3,MAP3K11,TNIP1,PDGFB,STK10,RELN,PDE4D,MEF2C,ATF3,RAPGEF1,TLR4,NLRC5,EDN1,RALB,APLNR,NEDD9,YES1,MTOR,MAP3K3,BMP2,HHEX,PREX2,SLK,SH3BP5,PPP1R2,ITPKB,PROX1,MADD,PAK2,PRKD2,SH3GLB1,SWAP70,TLR3,NCK1,LDB2,NSD1,PTGS2,MMD,RAPGEF2,TAOK1,CNOT7,HMGB1,NOS2,BLM,SPTBN4,RIPK1,ITGA6,RAD17,PDGFD,HIF1A,FKBP15,JCAD,ARHGEF2,CDON	up_VCAV_EC
GO:0032774	0.00372	0.999	11	1073	RNA biosynthetic process	BP	0.714	HOXA11,TBX15,HOXA10,HOXA9,PITX1,EMX2,SALL1,IRX5,TLR2,CAV1,SHOX2	up_VSAP_VSMC
GO:0010463	0.00372	0.999	8	28	mesenchymal cell proliferation	BP	1	WNT11,FGF7,OSR1,ZEB1,FBXW4,SHOX2,NFIB,TGFBR2	up_VSAP_EC
GO:1903727	0.00372	1	2	16	positive regulation of phospholipid metabolic	BP	0.528	LYN,ACSL3	down_ACOR_EC
GO:0060750	0.00372	1	1	3	epithelial cell proliferation involved in mammary gland duct elongation	BP	0.53	ESR1	up_AORT_VSMC
GO:0060751	0.00372	1	1	3	branch elongation involved in mammary	BP	0.53	ESR1	up_AORT_VSMC

GO:0008299	0.00373	1	5	14	isoprenoid biosynthetic process	BP	0.214	HMGCR,ENSCAFG00000018572,MVD,NUS1,PDSS1	down_ACOR_VSMC
GO:1905314	0.00373	1	5	12	semi-lunar valve development	BP	0.735	SMAD6,GATA5,GATA4,BMP4,TBX20	up_ACOR_VSMC
GO:0032092	0.00373	1	2	38	positive regulation of protein binding	BP	1	HOXA3,BAMBI	up_VPOR_VSMC
GO:0035914	0.00373	1	2	22	skeletal muscle cell differentiation	BP	0.523	SIX1,CYP26B1	up_VFEM_EC
GO:0001759	0.00373	1	1	13	organ induction	BP	0.926	GATA5	down_VFEM_VSMC
GO:0051491	0.00375	1	3	6	positive regulation of filopodium assembly	BP	0.312	FSCN1,DPYSL3,NRP1	down_VSAP_EC
GO:0072028	0.00375	1	2	37	nephron morphogenesis	BP	0.903	EYA1,HOXA11	up_VFEM_VSMC
GO:0055026	0.00376	1	1	5	negative regulation of cardiac muscle tissue	BP	1	TBX5	up_APUL_VSMC
GO:0051973	0.00378	0.999	6	19	positive regulation of telomerase activity	BP	0.216	HNRNPD,SLC29A1,AURKB,GREM1,NEK2,CCT4	down_ACOR_VSMC
GO:0032501	0.00379	1	7	1782	multicellular organismal process	BP	1	HOXC6,SLITRK5,LIMK1,GATA5,LRRC17,C6,TGFBI	up_VCAV_VSMC
GO:0055090	0.00379	1	3	6	acylglycerol homeostasis	BP	0.312	NR1H4,SESN2,APOE	down_VSAP_EC
GO:0070328	0.00379	1	3	6	triglyceride homeostasis	BP	0.312	NR1H4,SESN2,APOE	down_VSAP_EC
GO:0051239	0.0038	1	5	859	regulation of multicellular organismal process	BP	0.926	HOXC10,TLR2,EYA1,HOXA9,IRX5	up_AFEM_VSMC
GO:0007423	0.00381	1	4	155	sensory organ development	BP	0.523	EYA1,SIX1,MAB21L1,CYP26B1	up_VFEM_EC
GO:0007548	0.00381	1	2	68	sex differentiation	BP	0.926	HOXA9,IRX5	up_AFEM_VSMC
GO:0007275	0.00382	0.998	47	1370	multicellular organism development	BP	1	TBX15,HOXA9,IRX3,IRX5,HOXC10,LEP,LBH,XYLT1,ANGPT4,HOXA7,COL8A2,CD40,DLL1,CRYAB,F3,VXN,PLXNA4,NEFH,MAB21L1,THRB,LGI4,TMEM100,PLAGL2,GREM1,NFE2L1,OVOL1,CAMK2A,GREM2,HOXA6,CYLD,LUZP1,RECK,PTHLH,TBXA2R,EVI2B,CNNM4,BVES,PTPRB,AXIN2,ACACB,PPARGC1A,CLIC4,LRRN2,GCNT4,FOSL2,KANK1,TNC	up_AFEM_EC
GO:0071384	0.00382	1	5	12	cellular response to corticosteroid stimulus	BP	1	FAM107A,ZFP36L1,CREBRF,ZFP36L2,DDIT4	up_VSAP_EC
GO:0071385	0.00382	1	5	12	cellular response to glucocorticoid stimulus	BP	1	FAM107A,ZFP36L1,CREBRF,ZFP36L2,DDIT4	up_VSAP_EC
GO:0045766	0.00384	0.999	12	63	positive regulation of angiogenesis	BP	0.244	PLK2,TEK,CXCL8,CD34,APLNR,SMAD1,PIK3C2A,MAP3K3,PRKD2,TLR3,HIF1A,JCAD	up_VCAV_EC
GO:0022008	0.00385	0.998	39	406	neurogenesis	BP	0.316	ARHGEF2,VEGFA,SIPA1L1,ETV6,SGK1,CASP3,PTPN11,DPYSL3,HOOK3,IFRD1,ILK,KIF5B,HIF1A,SLC45A3,TNIK,UBA6,ZPR1,EIF2B1,NEO1,UST,CDH11,CTNNB1,APOE,LYN,MMD,GSK3B,VLDLR,SDC4,REST,MAPK10,PRMT1,EXT1,DISC1,PAK2,INHBA,SIX4,NRP1,ARF4,YWHAH	down_VSAP_EC
GO:0051781	0.00385	0.999	6	18	positive regulation of cell division	BP	0.741	LBH,HTR2B,FGFR2,PKP4,PRKCE,CAT	up_ACOR_VSMC
GO:0030855	0.00387	0.998	26	183	epithelial cell differentiation	BP	0.244	PRKCH,PDGFB,PODXL,PDE4D,MEF2C,RAPGEF1,GPR4,STC1,GATA3,SOX18,TMOD1,MTSS1,BMP2,HHEX,PROX1,ARID4B,FLNB,HOXB5,JAG1,RAPGEF2,CREB1,BASP1,SKIL,HIF1A,ATRX,ZEB1	up_VCAV_EC
GO:0051932	0.00387	1	1	9	synaptic transmission, GABAergic	BP	1	PLCL1	down_VFEM_EC
GO:0045089	0.00388	0.999	13	70	positive regulation of innate immune response	BP	0.244	GBP5,TNFAIP3,TNIP1,TMEM173,TLR4,NLRC5,PELI1,NECTIN2,RIOK3,TLR3,OTUD4,HMGB1,LSM14A	up_VCAV_EC
GO:0034329	0.00389	0.999	10	60	cell junction assembly	BP	0.316	VEGFA,NR1H4,ACTN1,FSCN1,WDR1,MYO1C,CTNNB1,ENSCAFG00000002015,TLN1,NRP1	down_VSAP_EC
GO:0009611	0.00389	1	4	122	response to wounding	BP	0.528	ACVRL1,LYN,PDPN,ELK3	down_ACOR_EC
GO:0060429	0.00389	1	4	341	epithelium development	BP	0.798	TBX5,GATA5,HOXB5,SLIT2	up_VPUL_VSMC
GO:0030155	0.0039	0.998	26	180	regulation of cell adhesion	BP	0.244	TEK,IL7R,CXCL8,EMCN,PODXL,FERMT1,GATA3,NEDD9,PELI1,PPP3CA,ARG2,BMP2,SLK,ITPKB,ANGPT2,PRKD2,SWAP70,APOD,NCK1,FES,JAG1,BCL10,PPP1CB,HMGB1,ITGA6,ENSCAFG00000002015	up_VCAV_EC
GO:0032879	0.0039	0.999	10	767	regulation of localization	BP	0.528	ACVRL1,LYN,HOXA3,IGF1R,PDPN,HOXA7,SULF1,ACSL3,STX4,NEDD4	down_ACOR_EC
GO:0060042	0.00391	1	3	14	retina morphogenesis in camera-type eye	BP	1	IRX5,DLL1,THRB	up_AFEM_EC

GO:0070170	0.00391	1	1	1	regulation of tooth mineralization	BP	0.528	ASPN	down_ACOR_EC
GO:0070171	0.00391	1	1	1	negative regulation of tooth mineralization	BP	0.528	ASPN	down_ACOR_EC
GO:0050901	0.00392	1	3	5	leukocyte tethering or rolling	BP	0.244	SELP,SELE,SELL	up_VCAV_EC
GO:0061756	0.00392	1	3	5	leukocyte adhesion to vascular endothelial cell	BP	0.244	SELP,SELE,SELL	up_VCAV_EC
GO:0071695	0.00393	0.999	5	45	anatomical structure maturation	BP	1	IRX5,XYLT1,LGI4,GREM1,RECK	up_AFEM_EC
GO:0002765	0.00393	1	1	1	immune response-inhibiting signal	BP	0.528	LYN	down_ACOR_EC
GO:0002767	0.00393	1	1	1	immune response-inhibiting cell surface receptor signaling	BP	0.528	LYN	down_ACOR_EC
GO:0002774	0.00393	1	1	1	Fc receptor mediated inhibitory signaling	BP	0.528	LYN	down_ACOR_EC
GO:0034136	0.00393	1	1	1	negative regulation of toll-like receptor 2 signaling	BP	0.528	LYN	down_ACOR_EC
GO:0070667	0.00393	1	1	1	negative regulation of mast cell proliferation	BP	0.528	LYN	down_ACOR_EC
GO:0043269	0.00395	0.998	23	135	regulation of ion transport	BP	0.746	ACTN2,PLCG2,KCNE3,TCAF1,GJA1,SARAF,PTPN3,DAPK1,CACNB3,AKAP6,DMPK,KCNJ4,PRKCE,PSEN1,CHD7,RAB11B,KCNC4,B2M,FXYD1,INPP5K,WFS1,PARK7,STC2	up_ACOR_VSMC
GO:0009952	0.00396	1	2	66	anterior/posterior pattern specification	BP	0.926	HOXC10,HOXA9	up_AFEM_VSMC
GO:0002376	0.00397	0.999	7	622	immune system process	BP	1	HOXA3,NR1H4,EVI2B,IL18BP,MRC1,CCL24,ARHGEF2	up_VPUL_EC
GO:0055022	0.00397	1	1	2	negative regulation of cardiac muscle tissue	BP	0.798	TBX5	up_VPUL_VSMC
GO:0060044	0.00397	1	1	2	negative regulation of cardiac muscle cell	BP	0.798	TBX5	up_VPUL_VSMC
GO:0061117	0.00397	1	1	2	negative regulation of heart growth	BP	0.798	TBX5	up_VPUL_VSMC
GO:0043010	0.003971675	0.999	8	96	camera-type eye development	BP	0.96436359	TMOD1,SOX9,AQP1,FZD5,FOXC2,NRP1,PYGO2,EFEMP1	up_ACOR_EC
GO:0022414	0.00398	1	3	294	reproductive process	BP	0.53	HOXA9,WNT7A,ESR1	up_AORT_VSMC
GO:0034121	0.00398	1	2	22	regulation of toll-like receptor signaling	BP	0.739	TLR2,CAV1	up_VSAP_VSMC
GO:0003218	0.00398	1	1	1	cardiac left ventricle formation	BP	1	TBX5	up_APUL_EC
GO:0097195	0.00398	1	1	1	pilomotor reflex	BP	1	NPNT	up_APUL_EC
GO:0099551	0.00398	1	1	1	trans-synaptic signaling by neuropeptide, modulating synaptic transmission	BP	1	NTRK2	up_APUL_EC
GO:0006808	0.00398	1	1	1	regulation of nitrogen utilization	BP	1	NR1H4	up_APUL_EC
GO:0007634	0.00398	1	1	1	optokinetic behavior	BP	1	HOXA1	up_APUL_EC
GO:0019676	0.00398	1	1	1	ammonia assimilation cycle	BP	1	NR1H4	up_APUL_EC
GO:0019740	0.00398	1	1	1	nitrogen utilization	BP	1	NR1H4	up_APUL_EC
GO:0021560	0.00398	1	1	1	abducens nerve development	BP	1	HOXA1	up_APUL_EC
GO:0021598	0.00398	1	1	1	abducens nerve morphogenesis	BP	1	HOXA1	up_APUL_EC
GO:0021599	0.00398	1	1	1	abducens nerve formation	BP	1	HOXA1	up_APUL_EC
GO:0034255	0.00398	1	1	1	regulation of urea metabolic process	BP	1	NR1H4	up_APUL_EC
GO:0034971	0.00398	1	1	1	histone H3-R17 methylation	BP	1	NR1H4	up_APUL_EC
GO:0038183	0.00398	1	1	1	bile acid signaling pathway	BP	1	NR1H4	up_APUL_EC
GO:0038185	0.00398	1	1	1	intracellular bile acid receptor signaling	BP	1	NR1H4	up_APUL_EC
GO:0045848	0.00398	1	1	1	positive regulation of nitrogen utilization	BP	1	NR1H4	up_APUL_EC
GO:1903314	0.00398	1	1	1	regulation of nitrogen cycle metabolic process	BP	1	NR1H4	up_APUL_EC
GO:1905693	0.00398	1	1	1	regulation of phosphatidic acid biosynthetic process	BP	1	NR1H4	up_APUL_EC
GO:1905695	0.00398	1	1	1	positive regulation of phosphatidic acid biosynthetic process	BP	1	NR1H4	up_APUL_EC

GO:2000211	0.00398	1	1	1	regulation of glutamate metabolic process	BP	1	NR1H4	up_APUL_EC
GO:2000213	0.00398	1	1	1	positive regulation of glutamate metabolic	BP	1	NR1H4	up_APUL_EC
GO:2001248	0.00398	1	1	1	regulation of ammonia assimilation cycle	BP	1	NR1H4	up_APUL_EC
GO:2001250	0.00398	1	1	1	positive regulation of ammonia assimilation	BP	1	NR1H4	up_APUL_EC
GO:0009888	0.00399	1	3	545	tissue development	BP	1	HOXA3,TBX5,HOXB5	up_APUL_VSMC
GO:0010159	0.00399	1	1	2	specification of animal organ position	BP	0.798	HOXA3	up_VPUL_VSMC
GO:0003274	0.00399	1	1	2	endocardial cushion fusion	BP	0.798	GATA5	up_VPUL_VSMC
GO:0072329	0.004	0.999	9	34	monocarboxylic acid catabolic process	BP	1	PEX7,ACAA1,ACACB,PEX2,FAAH,GCDH,EHHADH,LONP2,ABHD16A	up_VSAP_EC
GO:0048598	0.004	0.999	5	207	embryonic morphogenesis	BP	0.529	HOXA3,HOXA7,SULF1,HOXA1,HOXC4	down_ACOR_EC
GO:0014821	0.004	1	3	5	phasic smooth muscle contraction	BP	0.248	EDNRB,EDN1,P2RX2	up_VCAV_EC
GO:0055114	0.00401	1	3	157	oxidation-reduction process	BP	1	ETFA,SUCLG2,NDUFA10	down_AFEM_EC
GO:0000003	0.00401	1	3	295	reproduction	BP	0.53	HOXA9,WNT7A,ESR1	up_AORT_VSMC
GO:0003164	0.00401	1	1	2	His-Purkinje system development	BP	0.798	TBX5	up_VPUL_VSMC
GO:0003166	0.00401	1	1	2	bundle of His development	BP	0.798	TBX5	up_VPUL_VSMC
GO:0007507	0.00402	0.998	31	194	heart development	BP	0.746	TNNT2,PDLIM3,SMAD6,ACTN2,GATA5,GATA4,HTR2B,BMP4,FGFR2,GJA1,SEC24B,XIRP1,CITED2,LMO4,NEBL,ANKRD1,EGLN1,NRP1,SMARCD3,TBX20,TBX5,FRS2,PSEN1,TGFBR1,CHD7,PSKH1,EDN1,AP2B1,GAA,BVES,CRKL	up_ACOR_VSMC
GO:1901362	0.00402	1	6	1293	organic cyclic compound biosynthetic process	BP	0.928	TLR2,TBX15,EYA1,HOXA9,HOXA10,IRX5	up_AFEM_VSMC
GO:0003179	0.00402	0.999	6	19	heart valve morphogenesis	BP	0.249	ADAMTS9,MEF2C,GATA3,MTOR,BMP2,JAG1	up_VCAV_EC
GO:0051239	0.00402	1	5	859	regulation of multicellular organismal process	BP	1	SLITRK5,LIMK1,GATA5,LRRC17,C6	up_VCAV_VSMC
GO:0046184	0.00402	1	3	6	aldehyde biosynthetic process	BP	0.323	TPI1,REST,KDM3A	down_VSAP_EC
GO:0008584	0.00403	1	2	39	male gonad development	BP	0.929	HOXA9,HOXA11	up_VFEM_VSMC
GO:0046546	0.00403	1	2	39	development of primary male sexual	BP	0.929	HOXA9,HOXA11	up_VFEM_VSMC
GO:1903707	0.00403	1	2	48	negative regulation of hemopoiesis	BP	0.798	TMEM176A,TMEM176B	up_VPUL_VSMC
GO:0046449	0.00403	1	1	1	creatinine metabolic process	BP	0.523	MME	up_VFEM_EC
GO:0071492	0.00403	1	1	1	cellular response to UV-A	BP	0.523	MME	up_VFEM_EC
GO:0072338	0.00403	1	1	1	cellular lactam metabolic process	BP	0.523	MME	up_VFEM_EC
GO:0032700	0.00404	1	1	4	negative regulation of interleukin-17 production	BP	0.53	ARG2	up_AORT_VSMC
GO:0050665	0.00404	1	1	3	hydrogen peroxide biosynthetic process	BP	1	CYP1A1	up_VCAV_VSMC
GO:0035544	0.00404	1	1	1	negative regulation of SNARE complex assembly	BP	0.523	TRIM9	up_VFEM_EC
GO:0140074	0.00405	1	1	2	cardiac endothelial to mesenchymal transition	BP	0.798	GATA5	up_VPUL_VSMC
GO:0060971	0.00406	1	1	2	embryonic heart tube left/right pattern	BP	1	NKX2-5	up_VPOR_VSMC
GO:1903364	0.00407	0.999	10	61	positive regulation of cellular protein catabolic	BP	0.325	PRICKLE1,PLK3,SOCS5,TRIB3,MSN,APOE,GSK3B,DISC1,RAD23A,TMTC3	down_VSAP_EC
GO:0007099	0.00407	0.999	6	19	centriole replication	BP	0.23	WDR62,NUP62,PLK4,CDK2,SASS6,RBM14	down_ACOR_VSMC
GO:0098534	0.00407	0.999	6	19	centriole assembly	BP	0.23	WDR62,NUP62,PLK4,CDK2,SASS6,RBM14	down_ACOR_VSMC
GO:0030193	0.00407	1	2	21	regulation of blood coagulation	BP	0.529	LYN,PDPN	down_ACOR_EC
GO:1900046	0.00407	1	2	21	regulation of hemostasis	BP	0.529	LYN,PDPN	down_ACOR_EC
GO:0071623	0.00407	1	1	2	negative regulation of granulocyte chemotaxis	BP	0.798	SLIT2	up_VPUL_VSMC
GO:0090024	0.00407	1	1	2	negative regulation of neutrophil chemotaxis	BP	0.798	SLIT2	up_VPUL_VSMC
GO:1902623	0.00407	1	1	2	negative regulation of neutrophil migration	BP	0.798	SLIT2	up_VPUL_VSMC

GO:0048583	0.004071257	0.99768	43	1095	regulation of response to stimulus	BP	0.96436359	C7,SH3TC2,CDH3,NR1H4,SOX9,RGS1,FNDC4,NDRG2,ENSCAFG00000004331,EDEM2,FZD5,CCDC3,IFI6,DUSP26,DIXDC1,PTGIR,NOD2,IL33,RGMA,PDE4A,SMAD6,ESM1,PAK3,MMP28,TNFRSF19,PTGER4,ARHGDIB,ADAMTSL2,MAPK8IP2,MAVS,FOXC2,FIGNL1,LRRC15,LRP4,CLSTN1,SH3RF3,LRRC4,CLU,C23H3orf58,RARRES2,NRP1,ARHGEF26,PRKCD	up_ACOR_EC
GO:0008284	0.00408	0.998	31	238	positive regulation of cell proliferation	BP	0.25	HCLS1,IL7R,KIT,PRKCH,CALCRL,TNFAIP3,PDGFB,MEF2C,DLL4,TLR4,EDN1,APLNR,EGFL7,PELI1,ODC1,MAP3K3,BMP2,CNOT6,CCAR1,PROX1,PRKD2,NCK1,CNOT7,HMGB1,CRIP2,NRAS,PDGFD,CUL4A,HIF1A,JCAD,CDON	up_VCAV_EC
GO:0045944	0.00409	1	3	287	positive regulation of transcription by RNA polymerase II	BP	0.53	HOXA9,WNT7A,ESR1	up_AORT_VSMC
GO:2001247	0.0041	1	1	1	positive regulation of phosphatidylcholine biosynthetic process	BP	0.53	ACSL3	down_ACOR_EC
GO:0030449	0.0041	1	1	3	regulation of complement activation	BP	1	C6	up_VCAV_VSMC
GO:2000257	0.0041	1	1	3	regulation of protein activation cascade	BP	1	C6	up_VCAV_VSMC
GO:0001655	0.004100579	0.99886	9	116	urogenital system development	BP	0.96436359	TBX18,SOX9,AQP1,SMAD6,FOXC2,LRP4,CLSTN1,NRP1,PYGO2	up_ACOR_EC
GO:0051303	0.00411	0.999	7	26	establishment of chromosome localization	BP	0.23	ZW10,BIRC5,KIF22,CDCA8,NDC80,NUP62,ENSCAFG00000005299	down_ACOR_VSMC
GO:0071941	0.00411	1	1	4	nitrogen cycle metabolic process	BP	0.53	ARG2	up_AORT_VSMC
GO:0043116	0.00411	1	1	2	negative regulation of vascular permeability	BP	0.798	SLIT2	up_VPUL_VSMC
GO:0071676	0.00411	1	1	2	negative regulation of mononuclear cell	BP	0.798	SLIT2	up_VPUL_VSMC
GO:0001763	0.00412	1	3	78	morphogenesis of a branching structure	BP	0.523	EYA1,HOXB7,SIX1	up_VFEM_EC
GO:2000826	0.00412	1	1	14	regulation of heart morphogenesis	BP	0.984	GATA5	down_VFEM_VSMC
GO:0061476	0.00412	1	1	2	response to anticoagulant	BP	0.798	SLIT2	up_VPUL_VSMC
GO:0071503	0.00412	1	1	2	response to heparin	BP	0.798	SLIT2	up_VPUL_VSMC
GO:0071504	0.00412	1	1	2	cellular response to heparin	BP	0.798	SLIT2	up_VPUL_VSMC
GO:1903672	0.00413	0.999	6	20	positive regulation of sprouting angiogenesis	BP	0.252	PLK2,APLNR,SMAD1,PIK3C2A,MAP3K3,JCAD	up_VCAV_EC
GO:0055083	0.00413	1	1	6	monovalent inorganic anion homeostasis	BP	1	HOXA3	up_APUL_VSMC
GO:0006857	0.00413	1	1	3	oligopeptide transport	BP	1	CA2	up_VCAV_VSMC
GO:0035672	0.00413	1	1	3	oligopeptide transmembrane transport	BP	1	CA2	up_VCAV_VSMC
GO:0060429	0.004130529	0.99825	18	341	epithelium development	BP	0.96436359	CDH3,TMOD1,GATA4,BRSK1,SOX9,AQP1,FZD5,SMAD6,DLX3,TNFRSF19,ADAMTSL2,FOXC2,TBX5,LRP4,CLSTN1,NRP1,ARHGEF26,PYGO2	up_ACOR_EC
GO:0019835	0.00414	1	2	2	cytolysis	BP	0.252	LYZ,MMD	up_VCAV_EC
GO:0020027	0.00415	1	2	2	hemoglobin metabolic process	BP	0.328	HIF1A,INHBA	down_VSAP_EC
GO:0042541	0.00415	1	2	2	hemoglobin biosynthetic process	BP	0.328	HIF1A,INHBA	down_VSAP_EC
GO:0021520	0.00415	1	1	3	spinal cord motor neuron cell fate specification	BP	0.944	HOXC10	up_AFEM_VSMC
GO:0048537	0.00416	1	1	1	mucosal-associated lymphoid tissue	BP	0.523	TOX	up_VFEM_EC
GO:0048541	0.00416	1	1	1	Peyer's patch development	BP	0.523	TOX	up_VFEM_EC
GO:0045899	0.00419	1	1	4	positive regulation of RNA polymerase II transcriptional	BP	0.53	ESR1	up_AORT_VSMC
GO:0003170	0.004195057	0.99953	4	25	heart valve development	BP	0.96436359	GATA4,SOX9,SMAD6,TBX5	up_ACOR_EC
GO:0001933	0.0042	1	4	146	negative regulation of protein phosphorylation	BP	0.538	LYN,IGF1R,PRKAR2A,ASPN	down_ACOR_EC
GO:0007498	0.0042	1	2	38	mesoderm development	BP	0.929	EYA1,HOXA11	up_VFEM_VSMC
GO:0007624	0.0042	1	1	1	ultradian rhythm	BP	0.523	ENOX1	up_VFEM_EC
GO:0034109	0.00421	1	1	14	homotypic cell-cell adhesion	BP	0.984	JUP	down_VFEM_VSMC

GO:0032616	0.00421	1	1	4	interleukin-13 production	BP	0.53	ARG2	up_AORT_VSMC
GO:0032656	0.00421	1	1	4	regulation of interleukin-13 production	BP	0.53	ARG2	up_AORT_VSMC
GO:1902744	0.00421	1	1	2	negative regulation of lamellipodium	BP	0.798	SLIT2	up_VPUL_VSMC
GO:0060349	0.00422	1	2	38	bone morphogenesis	BP	0.929	HOXA11,SHOX2	up_VFEM_VSMC
GO:0090259	0.00422	1	1	2	regulation of retinal ganglion cell axon	BP	0.798	SLIT2	up_VPUL_VSMC
GO:0034622	0.00423	0.997	50	421	cellular protein-containing complex assembly	BP	0.236	HMGB1,STXBP6,USP4,EDC3,UBE2S,CAPG,CENPA,SLC29A1,NDC1,CENPN,FBXO5,PSMD13,INSIG1,RAD51,STMN1,CENPO,DHX33,GMNN,CENPH,CENPI,FASTKD3,OIP5,IPO4,HIST1H1A,RAC1,DDX28,IQGAP2,SAMM50,HMGB2,CDC42EP4,HIST1H2BB,DDX39B,SF3A2,CORO1A,CENPW,FOXRED1,ARPC1B,CENPP,GRWD1,PRMT5,PSMC1,ENSCAFG00000015189,HAT1,GEMIN7,UBE2C,MIS18A,TBCC,H3F3B,SF3A1,EIF3M	down_ACOR_VSMC
GO:0050923	0.00423	1	1	2	regulation of negative chemotaxis	BP	0.798	SLIT2	up_VPUL_VSMC
GO:0019220	0.00424	0.997	47	525	regulation of phosphate metabolic process	BP	0.332	ARHGEF2,VEGFA,NR1H4,SOCS5,ENPP1,ERRFI1,JCAD,SNX25,BCAR3,NUAK1,PTPN11,TRIB3,PGAM1,ILK,PPP1R12A,SESN2,PTGS2,HIF1A,DYNLL1,DNAJC10,TNIK,PTPN2,YES1,PLCL1,ADAM17,CNOT7,MAGED1,CTNNB1,APOE,LYN,STRAP,CCNYL1,MMD,CBLC,ATF3,VLDLR,SH3GLB1,HTT,ARFGEF3,CALM1,PAK2,INHBA,CDKN1A,NRP1,SH3RF3,MADD,MID1	down_VSAP_EC
GO:0051174	0.00424	0.997	47	525	regulation of phosphorus metabolic process	BP	0.332	ARHGEF2,VEGFA,NR1H4,SOCS5,ENPP1,ERRFI1,JCAD,SNX25,BCAR3,NUAK1,PTPN11,TRIB3,PGAM1,ILK,PPP1R12A,SESN2,PTGS2,HIF1A,DYNLL1,DNAJC10,TNIK,PTPN2,YES1,PLCL1,ADAM17,CNOT7,MAGED1,CTNNB1,APOE,LYN,STRAP,CCNYL1,MMD,CBLC,ATF3,VLDLR,SH3GLB1,HTT,ARFGEF3,CALM1,PAK2,INHBA,CDKN1A,NRP1,SH3RF3,MADD,MID1	down_VSAP_EC
GO:0007389	0.00424	1	3	137	pattern specification process	BP	1	HOXC10,HOXA9,HOXC6	up_AORT_EC
GO:0099536	0.00425	0.998	23	133	synaptic signaling	BP	0.776	PLCL1,FBXL20,HRH1,GLRB,STX11,TMEM108,EGLN1,CACNB3,SLC12A7,DMPK,SLITRK5,PRKCE,RNF19A,DAG1,APP,PSEN1,EDN1,KCNC4,LRRC4,FAM107A,INSYN1,PREPL,PARK7	up_ACOR_VSMC
GO:0050678	0.00425	0.998	18	117	regulation of epithelial cell proliferation	BP	0.258	TNFAIP3,PDGFB,MEF2C,DLL4,GATA3,APLNR,EGFL7,MTSS1,BMP2,CAV2,PROX1,MCC,PRKD2,HMGB1,NRAS,NR4A1,VASH1,JCAD	up_VCAV_EC
GO:0051290	0.00425	1	4	9	protein heterotetramerization	BP	0.236	RRM1,RCC1,FARSA,PDSS1	down_ACOR_VSMC
GO:0048646	0.00425	1	4	349	anatomical structure formation involved in	BP	0.798	HOXA3,TBX5,GATA5,SLIT2	up_VPUL_VSMC
GO:0098900	0.00425	1	1	15	regulation of action potential	BP	0.984	JUP	down_VFEM_VSMC
GO:0060687	0.00425	1	1	4	regulation of branching involved in prostate gland	BP	0.53	ESR1	up_AORT_VSMC
GO:0090343	0.00425	1	1	4	positive regulation of cell aging	BP	0.53	ARG2	up_AORT_VSMC
GO:2000774	0.00425	1	1	4	positive regulation of cellular senescence	BP	0.53	ARG2	up_AORT_VSMC
GO:0032007	0.00428	0.999	8	27	negative regulation of TOR signaling	BP	0.776	PRKAA2,CASTOR1,UBR1,SESN3,SESN1,DDIT4,BMT2,SH3BP4	up_ACOR_VSMC
GO:0048878	0.00428	1	4	283	chemical homeostasis	BP	1	HOXA3,TRPC4,CYP26B1,C3AR1	up_VPOR_VSMC
GO:0010960	0.00428	1	1	6	magnesium ion homeostasis	BP	1	HOXA3	up_APUL_VSMC
GO:0048568	0.00429	1	2	148	embryonic organ development	BP	1	HOXA3,HOXB5	up_APUL_VSMC
GO:0042118	0.00429	1	1	4	endothelial cell activation	BP	0.53	HOXA9	up_AORT_VSMC
GO:0010614	0.0043	1	1	5	negative regulation of cardiac muscle	BP	1	GATA5	down_VSAP_VSMC
GO:0014741	0.0043	1	1	5	negative regulation of muscle hypertrophy	BP	1	GATA5	down_VSAP_VSMC
GO:0031128	0.00431	1	1	15	developmental induction	BP	0.984	GATA5	down_VFEM_VSMC

GO:0060429	0.00432	0.997	43	341	epithelium development	BP	0.261	HOXB7,PRKCH,PDGFB,PODXL,PDE4D,MEF2C,FERMT1,CHD7,DLL4,RAPGEF1,GPR4,STC1,GATA3,EDN1,NTN4,SOX18,TMOD1,MTSS1,ARG2,MTOR,BMP2,HHEX,PROX1,ARID4B,FLNB,BTBD7,EXOC5,TIMELESS,HOXB5,LDB2,JAG1,RAPGEF2,CREB1,BCL10,BASP1,NUP133,SKIL,HIF1A,ATRX,ALDH1A3,LUZP1,RBBP6,ZEB1	up_VCAV_EC
GO:0046398	0.00432	1	1	1	UDP-glucuronate metabolic process	BP	0.541	CSGALNACT1	down_ACOR_EC
GO:0050653	0.00432	1	1	1	chondroitin sulfate proteoglycan biosynthetic process, polysaccharide chain biosynthetic process	BP	0.541	CSGALNACT1	down_ACOR_EC
GO:0051716	0.00433	0.997	146	2001	cellular response to stimulus	BP	0.333	ARHGEF2,PRICKLE1,VEGFA,P4HB,SIPA1L1,SULF1,ARHGAP31,ASNS,NR1H4,PLK3,ENSCAFG00000013214,HSPA5,SERPINE1,SOCS5,HM13,SPARC,SGK1,ERO1A,PRKACB,HSP90B1,ENPP1,ERRFI1,ITGA2,JCAD,SNX25,CBX5,CASP3,ENSCAFG00000003477,HYOU1,PGK1,WSB1,BCAR3,NUAK1,PTPN11,FLNB,DPYSL3,LRRC8A,TRIB3,FARP1,ILK,FHL2,PPP1R12A,PRCP,SMARCA4,SESN2,TGFBR1,PTGS2,REM1,MYO1C,PIK3C2A,CHSY1,MSN,UACA,SEL1L,KIF5B,HIF1A,CX3CL1,ENSCAFG00000000562,DNAJC10,TNIK,NXN,UBA6,RGS2,ZPR1,PTPN2,EIF2B1,YES1,NEO1,PLCL1,MTDH,ESM1,DDX39B,ADAM17,AGRN,EIF4EBP1,SKAP2,PDIA4,CNOT7,ADGRE5,MAGED1,DNAJB9,GNAQ,CTNNB1,KPNB1,CRYAB,STARD13,ENSCAFG00000002015,TFRC,APOE,WWC3,PDE1A,ZC3H15,KLF5,UBA5,MMP28,LYN,BNIP3,STRAP,DDIT3,PDS5B,FAM162A,BACH1,PLEKHG2,TLN1,CBLC,ATF3,GSK3B,CDC37,GTF2H1,ITGB3,GBP5,SH3GLB1,TAX1BP3,MYO1E,KPNA1,HTT,REST,RBBP8,MAPK10,EIF2S1,SH2B3,RDH11,ARFGEF3,CALM1,DISC1,RAD23A,PAK2,CREB3L2,INHBA,CAMK2D,NR4A1,ENSCAFG00000018360,HIF1AN,BTK,CDKN1A,TMTC3,NRP1,SH3RF3,RHBDF2,USP14,YWHAH,TNFAIP1,MADD,SEC13,KDM3A,MID1	down_VSAP_EC
GO:0003044	0.00433	1	4	8	regulation of systemic arterial blood pressure mediated by a chemical	BP	0.777	ADRA1B,SLC2A5,F2RL1,EDN1	up_ACOR_VSMC
GO:0001704	0.00433	1	2	43	formation of primary germ layer	BP	0.929	EYA1,HOXA11	up_VFEM_VSMC
GO:0043537	0.00433	1	1	15	negative regulation of blood vessel endothelial	BP	0.984	JUP	down_VFEM_VSMC
GO:0002829	0.00433	1	1	4	negative regulation of type 2 immune response	BP	0.53	ARG2	up_AORT_VSMC
GO:0045054	0.00434	1	1	2	constitutive secretory pathway	BP	1	RAB11B	down_VPOR_EC
GO:0061343	0.00435	1	1	3	cell adhesion involved in heart morphogenesis	BP	1	GATA5	up_VCAV_VSMC
GO:0008150	0.004356435	0.99775	162	5646	biological_process	BP	0.96436359	C7,CNTNAP4,SH3TC2,MYOZ2,CA4,FMOD,CPAMD8,CDH3,NR1H4,TMOD1,CLIC3,GATA4,BRSK1,TRPS1,KCTD12,TBX18,PLCL1,PKIB,CALY,THNSL2,SOX9,HMCN1,NMNAT2,RHOU,PKNOX2,ARHGAP6,PTH1R,GALNT5,RGS1,PCSK1N,ENSCAFG00000002230,FNDC4,PHF20,ATG16L2,NDRG2,AQP1,PORCN,ENSCAFG00000004331,SYNPO2,KCNB1,ADAMTS13,SARDH,EDEM2,PI3,FZD5,CCDC3,AGRN,PDE1A,IFI6,SEMA6D,GNAO1,HSF4,DUSP26,DIXDC1,RASL11A,PTGIR,PDLIM3,NOD2,IL33,RGMA,CASP10,BTD,LHX9,ENSCAFG00000028814,PDE4A,INSYN1,SMAD6,B3GNT7,DYNC1I1,KLHL25,PLCE1,PLS1,ACTN2,ZMYND8,ARHGAP28,RNLS,ESM1,NIT2,PTGDS,PAK3,CFI,MMP28,IL11RA,DLX3,GUCY1B1,FMO3,GRK7,BCL2,TRIM8,TNFRSF19,PTGER4,ARHGDIB,ELMO2,PREB,CST3,PIAS1,SLC25A42,DCLK2,ADAMTSL2,USP53,CHPT1,MAPK8IP2,PDRG1,RAPGEF4,MAVS,FOXC2,FIGNL1,LRRC15,IFIT2,KLHDC8B,ARSK,TBX5,PIGS,LRP4,CLSTN1,SH3RF3,AK7,LRRC4,PRELP,RBM38,TAPBP,CLU,SLC9A9,SFMBT2,ALDOC,C23H3orf58,ZNF229,CHMP4B,TP53INP2,TMTC2,RARRES2,VPS16,GMEB2,AGBL5,RIOX2,ABCB9,PRKACA,RNF114,VCAM1,UBE2H,STK32C,NRP1,AZIN2,AGPAT2,HM13,FKBP11,ARHGEF26,PRKCD	up_ACOR_EC

GO:0017185	0.00436	1	3	6	peptidyl-lysine hydroxylation	BP	0.333	PLOD2,PLOD3,P3H4	down_VSAP_EC
GO:0060350	0.00436	1	2	23	endochondral bone morphogenesis	BP	0.739	HOXA11,SHOX2	up_VSAP_VSMC
GO:0019276	0.00436	1	1	2	UDP-N-acetylgalactosamine	BP	1	EXTL2	down_AFEM_EC
GO:0048513	0.00437	0.999	11	906	animal organ development	BP	0.542	ACVRL1,LYN,HOXA3,PDPN,NRP2,HOXA7,SULF1,HOXA1,CSGALNACT1,HOXC4,ASPN	down_ACOR_EC
GO:1902105	0.00437	1	3	80	regulation of leukocyte differentiation	BP	0.523	TOX,HOXA7,CYP26B1	up_VFEM_EC
GO:0002250	0.00439	0.998	13	68	adaptive immune response	BP	0.263	IL7R,IL6,TNFAIP3,MEF2C,GATA3,RAB27A,NECTIN2,MTOR,THOC1,SWAP70,JAG1,BCL10,HMGB1	up_VCAV_EC
GO:0060971	0.00439	1	1	2	embryonic heart tube left/right pattern	BP	1	CITED2	down_VPOR_EC
GO:0021681	0.00439	1	1	4	cerebellar granular layer development	BP	0.53	WNT7A	up_AORT_VSMC
GO:0003129	0.00439	1	1	3	heart induction	BP	1	GATA5	up_VCAV_VSMC
GO:0048513	0.0044	0.999	7	906	animal organ development	BP	0.929	HOXC10,EYA1,HOXA9,TBX15,EMX2,HOXA11,SHOX2	up_VFEM_VSMC
GO:1903969	0.0044	1	1	3	regulation of response to macrophage colony-stimulating factor	BP	0.972	TLR2	up_AFEM_VSMC
GO:1903972	0.0044	1	1	3	regulation of cellular response to macrophage colony-stimulating factor	BP	0.972	TLR2	up_AFEM_VSMC
GO:0032536	0.0044	1	1	4	regulation of cell projection size	BP	0.53	WNT7A	up_AORT_VSMC
GO:0003342	0.0044	1	1	2	proepicardium development	BP	1	NKX2-5	up_VPOR_VSMC
GO:0003343	0.0044	1	1	2	septum transversum development	BP	1	NKX2-5	up_VPOR_VSMC
GO:0030193	0.00441	0.999	6	21	regulation of blood coagulation	BP	0.263	SELP,PDGFB,CD34,KLKB1,TLR4,TFPI	up_VCAV_EC
GO:1900046	0.00441	0.999	6	21	regulation of hemostasis	BP	0.263	SELP,PDGFB,CD34,KLKB1,TLR4,TFPI	up_VCAV_EC
GO:0038107	0.00441	1	1	2	nodal signaling pathway involved in determination of left/right asymmetry	BP	1	CITED2	down_VPOR_EC
GO:1900094	0.00441	1	1	2	regulation of transcription from RNA polymerase II promoter involved in determination of	BP	1	CITED2	down_VPOR_EC
GO:1900164	0.00441	1	1	2	nodal signaling pathway involved in determination of lateral mesoderm	BP	1	CITED2	down_VPOR_EC
GO:0008300	0.00441	1	1	1	isoprenoid catabolic process	BP	0.523	CYP26B1	up_VFEM_EC
GO:0016103	0.00441	1	1	1	diterpenoid catabolic process	BP	0.523	CYP26B1	up_VFEM_EC
GO:0016115	0.00441	1	1	1	terpenoid catabolic process	BP	0.523	CYP26B1	up_VFEM_EC
GO:0035981	0.00441	1	1	1	tongue muscle cell differentiation	BP	0.523	CYP26B1	up_VFEM_EC
GO:0042363	0.00441	1	1	1	fat-soluble vitamin catabolic process	BP	0.523	CYP26B1	up_VFEM_EC
GO:0070268	0.00441	1	1	1	cornification	BP	0.523	CYP26B1	up_VFEM_EC
GO:1902809	0.00441	1	1	1	regulation of skeletal muscle fiber	BP	0.523	CYP26B1	up_VFEM_EC
GO:1902811	0.00441	1	1	1	positive regulation of skeletal muscle fiber	BP	0.523	CYP26B1	up_VFEM_EC
GO:2001035	0.00441	1	1	1	regulation of tongue muscle cell differentiation	BP	0.523	CYP26B1	up_VFEM_EC
GO:2001037	0.00441	1	1	1	positive regulation of tongue muscle cell	BP	0.523	CYP26B1	up_VFEM_EC
GO:0034653	0.00441	1	1	1	retinoic acid catabolic process	BP	0.523	CYP26B1	up_VFEM_EC
GO:0016202	0.00442	1	2	39	regulation of striated muscle tissue	BP	1	NKX2-5,CYP26B1	up_VPOR_VSMC
GO:0033555	0.00443	0.999	5	13	multicellular organismal response to stress	BP	0.263	RELN,MEF2C,PPP3CA,ALS2,P2RX2	up_VCAV_EC
GO:0060149	0.00443	1	1	4	negative regulation of posttranscriptional gene	BP	0.53	ESR1	up_AORT_VSMC
GO:0060965	0.00443	1	1	4	negative regulation of gene silencing by miRNA	BP	0.53	ESR1	up_AORT_VSMC
GO:0060967	0.00443	1	1	4	negative regulation of gene silencing by RNA	BP	0.53	ESR1	up_AORT_VSMC

GO:1903799	0.00443	1	1	4	negative regulation of production of miRNAs involved in gene silencing	BP	0.53	ESR1	up_AORT_VSMC
GO:0034097	0.00444	0.998	31	238	response to cytokine	BP	0.263	ACKR1,HCLS1,GBP5,KIT,SELE,CXCL8,LAPTM5,CXCR4,MX2,PDGFB,TMEM173,NLRC5,GATA3,IRF8,CASP8,ST3GAL6,MAPK13,IFNGR1,FLNB,OTUD4,CREB1,CNOT7,GCH1,ADIPOR2,RIPK1,LSM14A,HIF1A,KDM3A,ENSCAFG00000002015,ARHGEF2,TJP2	up_VCAV_EC
GO:0006040	0.00444	1	2	12	amino sugar metabolic process	BP	1	OGA,GNPNAT1	up_VPOR_EC
GO:2000729	0.00444	1	1	1	positive regulation of mesenchymal cell proliferation involved in	BP	0.523	SIX1	up_VFEM_EC
GO:2000015	0.00445	1	2	2	regulation of determination of dorsal	BP	0.337	CTNNB1,DDIT3	down_VSAP_EC
GO:0019755	0.00445	1	1	3	one-carbon compound transport	BP	1	CA2	up_VCAV_VSMC
GO:1903078	0.00448	1	2	19	positive regulation of protein localization to	BP	0.548	ACSL3,STX4	down_ACOR_EC
GO:0071310	0.00449	0.999	7	634	cellular response to organic substance	BP	1	NR1H4,MRC1,CCL24,PTGER2,FLRT3,ARHGEF2,NEO1	up_VPUL_EC
GO:0061326	0.00449	1	2	41	renal tubule development	BP	0.929	EYA1,HOXA11	up_VFEM_VSMC
GO:0072080	0.00449	1	2	41	nephron tubule development	BP	0.929	EYA1,HOXA11	up_VFEM_VSMC
GO:0030336	0.0045	0.998	15	86	negative regulation of cell migration	BP	0.266	TIE1,ADAMTS9,MEF2C,DLL4,STC1,MMP28,ARAP3,ANGPT2,ENSCAFG00000010224,MCC,APOD,JAG1,HMGB1,ARHGDIB,VASH1	up_VCAV_EC
GO:0043044	0.0045	0.999	5	14	ATP-dependent chromatin remodeling	BP	0.248	SMARCC1,OIP5,SMARCB1,CENPP,MIS18A	down_ACOR_VSMC
GO:0060920	0.0045	1	1	2	cardiac pacemaker cell differentiation	BP	1	NKX2-5	up_VPOR_VSMC
GO:0060926	0.0045	1	1	2	cardiac pacemaker cell development	BP	1	NKX2-5	up_VPOR_VSMC
GO:0035904	0.00451	1	2	25	aorta development	BP	0.523	EYA1,SIX1	up_VFEM_EC
GO:0023052	0.00452	0.997	120	1602	signaling	BP	0.339	ARHGEF2,PRICKLE1,VEGFA,P4HB,SIPA1L1,SULF1,ARHGAP31,NR1H4,HSPA5,SERPINE1,SOCS5,SGK1,ERO1A,PRKACB,ENPP1,ERRFI1,ITGA2,JCAD,SNX25,CASP3,HYOU1,WSB1,BCAR3,NUAK1,PTPN11,LRRC8A,TRIB3,FARP1,ILK,FHL2,PPP1R12A,PRCP,SMARCA4,SESN2,TGFBR1,PTGS2,REM1,MYO1C,PIK3C2A,CHSY1,UACA,KIF5B,HIF1A,HTR7,CX3CL1,ENSCAFG00000000562,DNAJC10,TNIK,NXN,RGS2,PTPN2,EIF2B1,NEO1,PLCL1,MTDH,ESM1,ADAM17,AGRN,EIF4EBP1,SKAP2,CNOT7,CDH11,ADGRE5,MAGED1,GNAQ,CTNNB1,KPNB1,STARD13,ENSCAFG00000002015,APOE,WWC3,PDE1A,ZC3H15,KLF5,UBA5,LYN,BNIP3,STRAP,DDIT3,FAM162A,PLEKHG2,TLN1,CBLC,ATF3,GSK3B,CDC37,ITGB3,SH3GLB1,TAX1BP3,MYO1E,KPNA1,HTT,REST,MAPK10,SH2B3,RDH11,ARFGEF3,CALM1,RAB11FIP1,DISC1,PAK2,CREB3L2,LRRTM2,INHBA,CAMK2D,ENSCAFG00000018360,HIF1AN,BTK,CDKN1A,LMO7,NRP1,SH3RF3,RHBDF2,USP14,YWHAH,TNFAIP1,MADD,SEC13,KDM3A,MID1	down_VSAP_EC
GO:0006955	0.00452	0.999	5	327	immune response	BP	1	NR1H4,IL18BP,MRC1,CCL24,ARHGEF2	up_VPUL_EC
GO:1905832	0.00452	1	1	2	positive regulation of spindle assembly	BP	1	GPSM2	down_AFEM_EC
GO:1904776	0.00452	1	1	2	regulation of protein localization to cell cortex	BP	1	GPSM2	down_AFEM_EC
GO:1904778	0.00452	1	1	2	positive regulation of protein localization to cell	BP	1	GPSM2	down_AFEM_EC
GO:0072513	0.00452	1	1	3	positive regulation of secondary heart field cardioblast proliferation	BP	0.983	EYA1	up_AFEM_VSMC
GO:0044381	0.004526718	0.9997	3	13	glucose import in response to insulin	BP	0.96436359	NR1H4,ENSCAFG00000004331,RARRES2	up_ACOR_EC
GO:0048699	0.00453	0.997	36	371	generation of neurons	BP	0.339	ARHGEF2,VEGFA,SIPA1L1,SGK1,CASP3,PTPN11,DPYSL3,HOOK3,IFRD1,KIF5B,HIF1A,SLC45A3,TNIK,UBA6,ZPR1,NEO1,UST,CDH11,CTNNB1,APOE,LYN,MMD,GSK3B,VLDLR,SDC4,REST,MAPK10,PRMT1,EXT1,DISC1,PAK2,INHBA,SIX4,NRP1,ARF4,YWHAH	down_VSAP_EC

GO:0120034	0.00454	0.999	6	28	positive regulation of plasma membrane bounded cell projection	BP	0.339	FSCN1,DPYSL3,DYNLL1,ANLN,HTT,NRP1	down_VSAP_EC
GO:0051445	0.00454	0.999	5	14	regulation of meiotic cell cycle	BP	0.25	FBXO5,CDK1,CDC20,TTK,ASPM	down_ACOR_VSMC
GO:0035295	0.00454	1	4	356	tube development	BP	0.842	HOXA3,TBX5,GATA5,SLIT2	up_VPUL_VSMC
GO:0051963	0.00455	1	2	26	regulation of synapse assembly	BP	1	SLITRK5,NTRK2	up_APUL_EC
GO:0046939	0.00456	0.999	5	19	nucleotide phosphorylation	BP	0.339	PGK1,TPI1,PGAM1,HIF1A,ALDOC	down_VSAP_EC
GO:0002520	0.00456	1	4	279	immune system development	BP	1	HOXA3,BARX1,NKX2-5,CYP26B1	up_VPOR_VSMC
GO:0061337	0.00456	1	1	16	cardiac conduction	BP	1	JUP	down_VFEM_VSMC
GO:0003197	0.00457	1	1	16	endocardial cushion development	BP	1	GATA5	down_VFEM_VSMC
GO:0032026	0.00457	1	1	2	response to magnesium ion	BP	0.933	FBP1	up_VFEM_VSMC
GO:1900078	0.004578934	0.99969	3	13	positive regulation of cellular response to	BP	0.96436359	NR1H4,ENSCAFG00000004331,RARRES2	up_ACOR_EC
GO:0001992	0.00458	1	1	1	regulation of systemic arterial blood pressure by	BP	0.528	AVPR1A	up_VFEM_EC
GO:0032502	0.00459	0.999	13	1661	developmental process	BP	1	NR1H4,TBX5,HOXA2,MAPK10,HOXA3,ARHGEF26,ZDHHC16,SLITRK5,WIF1,NPNT,NTRK2,HOXA1,PDLIM3	up_APUL_EC
GO:0003208	0.00459	0.999	7	24	cardiac ventricle morphogenesis	BP	0.27	MEF2C,CHD7,DLL4,GATA3,PROX1,JAG1,HIF1A	up_VCAV_EC
GO:0060040	0.0046	1	1	1	retinal bipolar neuron differentiation	BP	0.739	IRX5	up_VSAP_VSMC
GO:0042221	0.00461	0.997	121	1012	response to chemical	BP	0.799	TNNT2,VEGFD,SEMA5A,TLR1,SMAD6,ACTN2,PLCG2,IFNGR1,LEP,LBH,AFF3,GATA5,GATA4,CD83,HTR2B,MBP,FEZ1,SKP2,PRKAA2,FERMT1,BMP4,TM7SF3,FGFR2,ABCA1,EIF4A2,ATRX,MED14,HRH1,CASTOR1,TLR3,PPARGC1A,LATS2,LPL,CITED2,TP53INP1,RERG,GLRB,ISG15,CD274,AR,PTGIR,SASH1,SLC2A5,TMEM108,FGF18,FAM210B,CDKN2B,ADCY6,KLHL20,ANKRD1,F2RL1,MXRA5,DAPK1,CTSB,BNIP3,EGLN1,NCEH1,AKAP6,P2RX7,CREBRF,NRP1,UBR1,PTPN22,TCN2,NPNT,OS9,UNC5B,STT3B,EVL,APLP1,SLITRK5,PTGDR,SESN3,CCDC3,PRKCE,FZD3,FRS2,AGRN,DAG1,FUT8,PARP9,CHD6,APP,PSEN1,TGFBR1,CHD7,JUP,CCL5,CLN8,NR3C1,DUSP22,RAB11B,EDN1,SESN1,OXR1,HPGD,NOD2,LRRC4,ARID5B,FAM107A,DDIT4,JAM3,B2M,ENSCAFG00000018360,JKAMP,TNFRSF11B,HERPUD1,FEZ2,GNAQ,INPP5K,FAF2,PFKL,WFS1,SLC25A12,CAT,DZIP3,SH3BP4,ENSCAFG00000029553,PARK7,STC2,CRKL	up_ACOR_VSMC
GO:0045596	0.00461	0.998	11	186	negative regulation of cell differentiation	BP	1	HOXA9,IRX3,LBH,HOXA7,DLL1,TWIST2,THRB,GREM1,CYLD,AXIN2,KANK1	up_AFEM_EC
GO:0072498	0.00461	1	4	8	embryonic skeletal joint development	BP	1	HOXA11,OSR2,OSR1,SHOX2	up_VSAP_EC
GO:0003161	0.00461	1	1	6	cardiac conduction system development	BP	1	TBX5	up_APUL_VSMC
GO:0007188	0.004621907	0.99927	5	41	adenylate cyclase-modulating G protein-coupled receptor signaling	BP	0.96436359	PTH1R,GNAO1,PTGIR,PTGER4,GNAZ	up_ACOR_EC
GO:0007189	0.00463	0.999	7	22	adenylate cyclase-activating G protein-coupled receptor signaling	BP	0.799	ADRA1B,ADGRD1,ABCA1,PTGIR,ADCY6,APLP1,GNAQ	up_ACOR_VSMC
GO:0098543	0.00463	1	1	4	detection of other organism	BP	0.983	TLR2	up_AFEM_VSMC
GO:0016045	0.00463	1	1	4	detection of bacterium	BP	0.983	TLR2	up_AFEM_VSMC
GO:0070252	0.00464	1	1	17	actin-mediated cell contraction	BP	1	JUP	down_VFEM_VSMC

GO:0007165	0.00466	0.996	172	1496	signal transduction	BP	0.799	SH3TC2,FGF13,RHOT1,ADRA1B,VEGFD,TLR1,SMAD6,ATRNL1,ARHGAP28,ACTN2,LGR6,PLCG2,IFNGR1,LEP,PLCL1,LBH,MAP3K8,MID1,GATA5,ADGRD1,CD83,EFNB2,ANGPT1,ENSCAFG00000016285,HTR2B,RASSF2,MBP,ZRANB1,C28H10orf71,RECK,SKP2,NACC2,PRKAA2,MLLT3,ITM2C,FERMT1,BMP4,FGFR2,ABCA1,GJA1,DOCK3,ELF1,ATRX,MED14,HRH1,CASTOR1,TLR3,SH3RF2,LATS2,PDE5A,CITED2,TP53INP1,RERG,GLRB,ARHGAP29,ZNRF3,CD274,PLPP2,AR,PTGIR,SASH1,GPR155,TMEM108,FGF18,ARHGEF26,NDRG2,CHN2,CDKN2B,ADCY6,ITPR1,GRK7,LIMS2,BLNK,ANKRD1,F2RL1,ADGRL1,CAMK2D,PSEN2,PPP2R5E,DAPK1,ARHGAP5,BNIP3,NCEH1,AKAP6,CDKN1A,JMY,GNA14,CRLF3,CLSTN1,LRRC8C,CREBRF,NRP1,UBR1,PDE1A,PTPN22,DMPK,NPNT,UNC5B,SMPD1,RCAN2,APLP1,TBX20,SESN3,SH3RF1,CCDC3,PRKCE,FZD3,FRS2,AGRN,RND1,SCG5,RGS2,DAG1,FUT8,PARP9,APP,PSEN1,TGFBR1,SYNJ2BP,TESK2,PIK3C3,SIPA1L1,SENP7,CCL5,WWC2,PERP,PSKH1,RTKN2,DUSP22,SIAH1,TIRAP,EDN1,SESN1,HPGD,DUSP26,NOD2,LRRC4,ARID5B,INSYN1,DDIT4,ENSCAFG00000018360,DISC1,DEPDC7,RASGRP2,SH3RF3,SERINC3,TNFRSF11B,NCALD,PPP1R12B,USP20,HERPUD1,GNAQ,	up_ACOR_VSMC
GO:0001944	0.00466	0.998	27	263	vasculature development	BP	0.342	PRICKLE1,VEGFA,SULF1,MYH9,SERPINE1,SPARC,LTBP1,PTPN14,ERRFI1,JCAD,PGK1,TMED2,PRCP,PIK3C2A,HIF1A,THBS2,MTDH,ESM1,ACTA2,ADAMTS6,CTNNB1,STARD13,APOE,KLF5,DDIT3,MYO1E,NRP1	down_VSAP_EC
GO:0018208	0.00466	1	3	7	peptidyl-proline modification	BP	0.342	P4HB,P4HA1,PPIB	down_VSAP_EC
GO:0046947	0.00469	1	2	2	hydroxylysine biosynthetic process	BP	0.342	PLOD2,PLOD3	down_VSAP_EC
GO:0046946	0.00469	1	2	2	hydroxylysine metabolic process	BP	0.342	PLOD2,PLOD3	down_VSAP_EC
GO:0048634	0.00469	1	2	40	regulation of muscle organ development	BP	1	NKX2-5,CYP26B1	up_VPOR_VSMC
GO:0008593	0.0047	1	1	33	regulation of Notch signaling pathway	BP	1	GATA5	down_AFEM_VSMC
GO:0043502	0.00472	1	1	16	regulation of muscle adaptation	BP	1	GATA5	down_VFEM_VSMC
GO:0090066	0.00473	0.998	24	162	regulation of anatomical structure size	BP	0.276	EDNRB,HCLS1,IL7R,IFRD1,DOCK4,LRRC8D,BAIAP2L1,EDN1,RAB11A,TMOD1,GUCY1A1,MTOR,LRRC8C,SLC12A7,NCKAP1,IQGAP2,SWAP70,NCK1,CREB1,NOS2,ARPC5L,ALS2,HP1BP3,TMOD3	up_VCAV_EC
GO:0009952	0.00473	0.999	6	66	anterior/posterior pattern specification	BP	1	HOXA9,HOXC10,HOXA7,DLL1,HOXA6,AXIN2	up_AFEM_EC
GO:0048645	0.00473	1	1	33	animal organ formation	BP	1	GATA5	down_AFEM_VSMC
GO:0006525	0.00473	1	1	4	arginine metabolic process	BP	0.561	ARG2	up_AORT_VSMC
GO:0006182	0.00474	1	3	4	cGMP biosynthetic process	BP	0.276	NPR1,GUCY1A1,GUCY1B1	up_VCAV_EC
GO:0060216	0.00474	1	1	3	definitive hemopoiesis	BP	0.994	HOXA9	up_AFEM_VSMC
GO:0050778	0.00476	0.998	21	141	positive regulation of immune response	BP	0.277	GBP5,PRKCH,TNFAIP3,TNIP1,TMEM173,PDE4D,MEF2C,TLR4,NLRC5,EIF2B2,GATA3,PELI1,NECTIN2,RIOK3,PRKD2,TLR3,OTUD4,PLCG2,BCL10,HMGB1,LSM14A	up_VCAV_EC
GO:0048869	0.00477	1	5	1119	cellular developmental process	BP	0.562	HOXC10,HOXA9,WNT7A,ARG2,ESR1	up_AORT_VSMC
GO:0060993	0.00478	1	2	42	kidney morphogenesis	BP	0.943	EYA1,HOXA11	up_VFEM_VSMC
GO:0034629	0.0048	0.999	5	14	cellular protein-containing complex localization	BP	0.26	STXBP6,NDC1,HASPIN,EZR,MIOS	down_ACOR_VSMC
GO:1904262	0.00484	1	4	8	negative regulation of TORC1 signaling	BP	0.822	CASTOR1,SESN3,SESN1,BMT2	up_ACOR_VSMC
GO:0110110	0.00484	1	1	34	positive regulation of animal organ	BP	1	GATA5	down_AFEM_VSMC
GO:0051764	0.00485	1	3	6	actin crosslink formation	BP	0.349	ACTN1,DPYSL3,PLS3	down_VSAP_EC
GO:0010825	0.00486	1	3	5	positive regulation of centrosome duplication	BP	0.261	POC1A,NUP62,PLK4	down_ACOR_VSMC

GO:0007154	0.00487	0.997	121	1622	cell communication	BP	0.349	ARHGEF2,PRICKLE1,VEGFA,P4HB,SIPA1L1,SULF1,ARHGAP31,ASNS,NR1H4,HSPA5,SERPINE1,SOCS5,SGK1,ERO1A,PRKACB,ENPP1,ERRFI1,ITGA2,JCAD,SNX25,CASP3,HYOU1,WSB1,BCAR3,NUAK1,PTPN11,LRRC8A,TRIB3,FARP1,ILK,FHL2,PPP1R12A,PRCP,SMARCA4,SESN2,TGFBR1,PTGS2,REM1,MYO1C,PIK3C2A,CHSY1,UACA,KIF5B,HIF1A,HTR7,CX3CL1,ENSCAFG00000000562,DNAJC10,TNIK,NXN,RGS2,PTPN2,EIF2B1,NEO1,PLCL1,MTDH,ESM1,ADAM17,AGRN,EIF4EBP1,SKAP2,CNOT7,CDH11,ADGRE5,MAGED1,GNAQ,CTNNB1,KPNB1,STARD13,ENSCAFG00000002015,APOE,WWC3,PDE1A,ZC3H15,KLF5,UBA5,LYN,BNIP3,STRAP,DDIT3,FAM162A,PLEKHG2,TLN1,CBLC,ATF3,GSK3B,CDC37,ITGB3,SH3GLB1,TAX1BP3,MYO1E,KPNA1,HTT,REST,MAPK10,EIF2S1,SH2B3,RDH11,ARFGEF3,CALM1,RAB11FIP1,DISC1,PAK2,CREB3L2,LRRTM2,INHBA,CAMK2D,ENSCAFG00000018360,HIF1AN,BTK,CDKN1A,NRP1,SH3RF3,RHBDF2,USP14,YWHAH,TNFAIP1,MADD,SEC13,KDM3A,MID1	down_VSAP_EC
GO:0006284	0.00487	0.999	6	20	base-excision repair	BP	0.261	RPA1,HMGA1,POLE,FEN1,NEIL3,APEX1	down_ACOR_VSMC
GO:0044092	0.004871375	0.99808	15	270	negative regulation of molecular function	BP	0.96436359	NR1H4,PKIB,PCSK1N,IFI6,DUSP26,ACTN2,ARHGAP28,CST3,LRRC15,IFIT2,CLSTN1,LRRC4,PRKCD,PPP1R26,DBNDD2	up_ACOR_EC
GO:0019471	0.00488	1	3	7	4-hydroxyproline metabolic process	BP	0.349	P4HB,ERO1A,P4HA1	down_VSAP_EC
GO:0019221	0.00488	1	3	102	cytokine-mediated signaling pathway	BP	1	NR1H4,CCL24,FLRT3	up_VPUL_EC
GO:0001657	0.00488	1	2	42	ureteric bud development	BP	0.943	EYA1,HOXA11	up_VFEM_VSMC
GO:0072163	0.00488	1	2	42	mesonephric epithelium development	BP	0.943	EYA1,HOXA11	up_VFEM_VSMC
GO:0072164	0.00488	1	2	42	mesonephric tubule development	BP	0.943	EYA1,HOXA11	up_VFEM_VSMC
GO:0055065	0.00489	1	3	145	metal ion homeostasis	BP	1	HOXA3,TRPC4,C3AR1	up_VPOR_VSMC
GO:0042127	0.00491	0.999	7	452	regulation of cell proliferation	BP	0.595	ACVRL1,LYN,HOXA3,IGF1R,PDPN,SULF1,STX4	down_ACOR_EC
GO:1902532	0.00491	0.999	4	204	negative regulation of intracellular signal	BP	1	NR1H4,PTGER2,FLRT3,ARHGEF2	up_VPUL_EC
GO:0042482	0.00491	1	2	2	positive regulation of odontogenesis	BP	0.284	CD34,BMP2	up_VCAV_EC
GO:0001954	0.00491	1	1	17	positive regulation of cell-matrix adhesion	BP	1	JUP	down_VFEM_VSMC
GO:0016576	0.00491	1	1	2	histone dephosphorylation	BP	0.943	EYA1	up_VFEM_VSMC
GO:0071705	0.00492	0.997	50	582	nitrogen compound transport	BP	0.349	PRICKLE1,MYH9,NR1H4,PLK3,HM13,TRAM1,PTPN14,SLC7A5,SEC24D,PTPN11,SEC23A,SLC38A1,SRP54,MYO1C,ENSCAFG00000031588,SEL1L,HIF1A,SSR1,IWS1,ZPR1,STAM2,SMG7,XPOT,USO1,SRPRA,DDX39B,COPG1,KPNB1,COPB2,SRP72,TFRC,APOE,KPNA4,LYN,SLC7A11,GSK3B,GBP5,KPNA1,REST,SCAMP1,RAB11FIP1,KDELR2,AP3S1,CLTC,STX6,CDKN1A,RHBDF2,YWHAH,BLZF1,SEC13	down_VSAP_EC
GO:0048012	0.00493	1	3	7	hepatocyte growth factor receptor signaling	BP	0.349	PTPN2,ESM1,NRP1	down_VSAP_EC
GO:0090092	0.00494	1	3	72	regulation of transmembrane receptor protein serine/threonine	BP	0.595	ACVRL1,SULF1,ASPN	down_ACOR_EC
GO:0040013	0.004945831	0.99871	8	100	negative regulation of locomotion	BP	0.96436359	SEMA6D,ZMYND8,MMP28,TRIM8,PTGER4,ARHGDIB,TBX5,NRP1	up_ACOR_EC
GO:0090596	0.00496	1	3	84	sensory organ morphogenesis	BP	0.568	EYA1,SIX1,CYP26B1	up_VFEM_EC
GO:1901861	0.00496	1	2	41	regulation of muscle tissue development	BP	1	NKX2-5,CYP26B1	up_VPOR_VSMC
GO:0006600	0.00496	1	1	1	creatine metabolic process	BP	0.739	GATM	up_VSAP_VSMC
GO:0006601	0.00496	1	1	1	creatine biosynthetic process	BP	0.739	GATM	up_VSAP_VSMC
GO:0014889	0.00496	1	1	1	muscle atrophy	BP	0.739	GATM	up_VSAP_VSMC
GO:0021675	0.00498	1	2	29	nerve development	BP	1	HOXA3,HOXA1	up_APUL_EC
GO:2000136	0.00499	1	1	7	regulation of cell proliferation involved in	BP	1	TBX5	up_APUL_VSMC

GO:0001822	0.004993389	0.9987	8	98	kidney development	BP	0.96436359	SOX9,AQP1,SMAD6,FOXC2,LRP4,CLSTN1,NRP1,PYGO2	up_ACOR_EC
GO:1900120	0.00501	1	1	7	regulation of receptor binding	BP	1	HOXA3	up_APUL_VSMC
GO:0050684	0.00502	0.999	10	46	regulation of mRNA processing	BP	0.266	RNPS1,SRSF2,U2AF2,THRAP3,HNRNPL,SF3B4,RBM14,WTAP,JMJD6,SF3A1	down_ACOR_VSMC
GO:0006886	0.00503	0.997	32	332	intracellular protein transport	BP	0.354	PRICKLE1,PLK3,HM13,TRAM1,PTPN14,SEC24D,PTPN11,SEC23A,SRP54,MYO1C,SEL1L,SSR1,IWS1,ZPR1,STAM2,SMG7,XPOT,USO1,SRPRA,DDX39B,COPG1,KPNB1,COPB2,SRP72,KPNA4,GSK3B,KPNA1,AP3S1,CLTC,STX6,CDKN1A,YWHAH	down_VSAP_EC
GO:0010159	0.00503	1	1	2	specification of animal organ position	BP	1	HOXA3	up_VPOR_VSMC
GO:0051972	0.00504	0.999	6	20	regulation of telomerase activity	BP	0.266	HNRNPD,SLC29A1,AURKB,GREM1,NEK2,CCT4	down_ACOR_VSMC
GO:0051240	0.00504	1	2	489	positive regulation of multicellular organismal	BP	1	JUP,GATA5	down_VFEM_VSMC
GO:0009566	0.00504	1	2	24	fertilization	BP	0.739	HOXA11,HOXA9	up_VSAP_VSMC
GO:0043537	0.00505	0.999	5	15	negative regulation of blood vessel endothelial	BP	0.29	MEF2C,DLL4,ANGPT2,HMGB1,VASH1	up_VCAV_EC
GO:0050818	0.00505	1	2	24	regulation of coagulation	BP	0.604	LYN,PDPN	down_ACOR_EC
GO:0061323	0.00505	1	1	7	cell proliferation involved in heart morphogenesis	BP	1	TBX5	up_APUL_VSMC
GO:0048869	0.00506	0.999	11	1119	cellular developmental process	BP	0.739	HOXA11,HOXA9,PITX1,EMX2,SALL1,HOXC10,IRX5,STIM1,CAV1,SHOX2,GJB5	up_VSAP_VSMC
GO:0007519	0.00508	0.998	10	49	skeletal muscle tissue development	BP	0.291	EPHB1,MEF2C,ATF3,FOXN2,PPP3CA,CAV2,FLNB,NR1D2,P2RX2,CDON	up_VCAV_EC
GO:0048513	0.00508	0.999	9	906	animal organ development	BP	1	NR1H4,TBX5,HOXA3,ZDHHC16,SLITRK5,NPNT,NTRK2,HOXA1,PDLIM3	up_APUL_EC
GO:0007275	0.00508	0.999	6	1370	multicellular organism development	BP	1	HOXC10,TBX15,EYA1,HOXA9,BEND2,IRX5	up_AFEM_VSMC
GO:2000112	0.00509	0.999	11	1119	regulation of cellular macromolecule biosynthetic process	BP	0.739	HOXA11,HOXA10,HOXA9,PITX1,EMX2,SALL1,IRX5,PRR16,TLR2,CAV1,SHOX2	up_VSAP_VSMC
GO:0010959	0.00509	1	3	79	regulation of metal ion transport	BP	0.739	NETO1,STIM1,CAV1	up_VSAP_VSMC
GO:0072359	0.00511	0.999	4	368	circulatory system development	BP	0.936	HOXA3,TBX5,GATA5,SLIT2	up_VPUL_VSMC
GO:0032490	0.00512	1	1	4	detection of molecule of bacterial origin	BP	1	TLR2	up_AFEM_VSMC
GO:0003285	0.00513	1	1	2	septum secundum development	BP	1	NKX2-5	up_VPOR_VSMC
GO:0009968	0.00514	0.997	37	392	negative regulation of signal transduction	BP	0.359	ARHGEF2,PRICKLE1,SULF1,NR1H4,HSPA5,SERPINE1,SOCS5,PRKACB,ENPP1,ERRFI1,SNX25,HYOU1,FHL2,SMARCA4,SESN2,PTGS2,HIF1A,NXN,RGS2,PTPN2,ADAM17,CNOT7,CTNNB1,APOE,WWC3,LYN,STRAP,DDIT3,CBLC,ATF3,GSK3B,TAX1BP3,HTT,CALM1,HIF1AN,NRP1,TNFAIP1	down_VSAP_EC
GO:0071711	0.00514	1	4	8	basement membrane organization	BP	0.849	FERMT1,CTSS,SPINT2,DAG1	up_ACOR_VSMC
GO:0071696	0.00514	1	3	6	ectodermal placode development	BP	0.359	CTNNB1,SIX4,NRP1	down_VSAP_EC
GO:0010460	0.00514	1	2	2	positive regulation of heart rate	BP	0.293	EDN1,GCH1	up_VCAV_EC
GO:0048701	0.00514	1	2	25	embryonic cranial skeleton morphogenesis	BP	0.584	TBX15,SIX1	up_VFEM_EC
GO:0045898	0.00516	1	1	5	regulation of RNA polymerase II transcriptional	BP	0.604	ESR1	up_AORT_VSMC
GO:0010735	0.00518	1	1	2	positive regulation of transcription via serum response element binding	BP	1	NKX2-5	up_VPOR_VSMC
GO:0061035	0.0052	1	2	24	regulation of cartilage development	BP	0.739	HOXA11,SHOX2	up_VSAP_VSMC
GO:0045747	0.0052	1	1	18	positive regulation of Notch signaling pathway	BP	1	GATA5	down_VFEM_VSMC
GO:0008643	0.00521	0.999	8	45	carbohydrate transport	BP	0.361	NR1H4,ENPP1,PTPN11,TRIB3,SESN2,SLC2A1,YES1,SLC2A3	down_VSAP_EC
GO:0009111	0.00521	1	1	2	vitamin catabolic process	BP	1	CYP26B1	up_VPOR_VSMC
GO:0048743	0.00521	1	1	1	positive regulation of skeletal muscle fiber	BP	0.739	SHOX2	up_VSAP_VSMC
GO:0043009	0.00522	0.999	5	227	chordate embryonic development	BP	0.619	ACVRL1,HOXA3,HOXA7,SULF1,HOXA1	down_ACOR_EC
GO:0003128	0.00523	1	1	6	heart field specification	BP	1	GATA5	down_VSAP_VSMC

GO:0003164	0.00523	1	1	2	His-Purkinje system development	BP	1	NKX2-5	up_VPOR_VSMC
GO:0003166	0.00523	1	1	2	bundle of His development	BP	1	NKX2-5	up_VPOR_VSMC
GO:0050000	0.00524	0.999	7	27	chromosome localization	BP	0.273	ZW10,BIRC5,KIF22,CDCA8,NDC80,NUP62,ENSCAFG00000005299	down_ACOR_VSMC
GO:0010992	0.00524	1	2	2	ubiquitin recycling	BP	0.273	UBE2S,UBE2C	down_ACOR_VSMC
GO:0010994	0.00524	1	2	2	free ubiquitin chain polymerization	BP	0.273	UBE2S,UBE2C	down_ACOR_VSMC
GO:0009581	0.00524	1	1	18	detection of external stimulus	BP	1	JUP	down_VFEM_VSMC
GO:0009582	0.00524	1	1	18	detection of abiotic stimulus	BP	1	JUP	down_VFEM_VSMC
GO:0051130	0.00526	0.997	42	342	positive regulation of cellular component organization	BP	0.299	EPHB1,TEK,GBP5,SELE,ARHGEF15,ATP8A1,PDGFB,BAIAP2L1,RELN,RAPGEF1,TLR4,GATA3,EDN1,RAB11A,RALB,RAB27A,NEDD9,IRF8,PPP3CA,MTOR,CNOT6,NCKAP1,PROX1,IQGAP2,HMBOX1,PRKD2,SH3GLB1,SWAP70,NCK1,FES,HIST1H1A,RAPGEF2,PLCG2,PAN3,PMAIP1,ARPC5L,SMC5,RAD21,HIF1A,ATRX,ENSCAFG00000002015,FSCN1	up_VCAV_EC
GO:0007389	0.00529	0.998	9	137	pattern specification process	BP	1	HOXA9,IRX3,HOXC10,HOXA7,DLL1,GREM1,GREM2,HOXA6,AXIN2	up_AFEM_EC
GO:0048625	0.00529	1	1	1	myoblast fate commitment	BP	0.739	PITX1	up_VSAP_VSMC
GO:0001845	0.00531	1	2	5	phagolysosome assembly	BP	1	RAB20,RAB7A	up_AFEM_EC
GO:0046330	0.00532	0.998	11	47	positive regulation of JNK cascade	BP	0.849	RASSF2,TLR3,SH3RF2,SASH1,F2RL1,APP,DUSP22,EDN1,NOD2,SH3RF3,MAP3K7	up_ACOR_VSMC
GO:0140131	0.00532	1	2	5	positive regulation of lymphocyte chemotaxis	BP	1	CCL5,ADAM17	up_AFEM_EC
GO:0017000	0.00532	1	1	4	antibiotic biosynthetic process	BP	1	CYP1A1	up_VCAV_VSMC
GO:0043393	0.00533	0.998	15	77	regulation of protein binding	BP	0.849	PLCL1,BMP4,XIRP1,CLSTN1,LRPAP1,SMARCD3,CCPG1,APP,PSEN1,MITD1,AKTIP,B2M,DISC1,HERPUD1,PARK7	up_ACOR_VSMC
GO:0072070	0.00534	1	2	5	loop of Henle development	BP	1	IRX3,DLL1	up_AFEM_EC
GO:0003181	0.00534	1	1	7	atrioventricular valve morphogenesis	BP	1	TBX5	up_APUL_VSMC
GO:0065009	0.00535	0.996	91	735	regulation of molecular function	BP	0.849	TNNT2,ARHGAP28,ACTN2,PLCG2,LEP,PLCL1,MAP3K8,KCNE3,HTR2B,RASSF2,TCAF1,USP6NL,MBP,RECK,BMP4,EVI5L,FGFR2,EIF4A2,SCARB2,TLR3,SH3RF2,XIRP1,PPARGC1A,LATS2,PDE5A,PKP4,AR,SASH1,LMO4,PTPN3,FGF18,CHN2,CDKN2B,CST3,F2RL1,DAPK1,CTSB,EGLN1,CACNB3,AKAP6,CDKN1A,JMY,CLSTN1,LRPAP1,NPNT,ATP7A,SMARCD3,SMPD1,SH3RF1,CCNT1,MARF1,FRS2,CCPG1,PPP1R26,PARP9,APP,PSEN1,SGSM2,JUP,SPINK2,CCL5,DDR2,MITD1,PERP,DUSP22,EDN1,DYNC1I2,AKTIP,DUSP26,NOD2,LRRC4,ARID5B,PRKAG3,B2M,DISC1,FXYD1,DCUN1D4,HERPUD1,SBF2,MAP3K7,INPP5K,WFS1,SH3BP4,DYNC1I1,PARK7,GPATCH2,ZFYVE1,IL18R1,HMBOX1,BVES,LMF1	up_ACOR_VSMC
GO:0002065	0.00535	1	1	37	columnar/cuboidal epithelial cell	BP	1	GATA5	down_AFEM_VSMC
GO:0031399	0.00536	0.997	49	559	regulation of protein modification process	BP	0.366	ARHGEF2,PRICKLE1,VEGFA,HSPA5,SOCS5,ENPP1,ERRFI1,JCAD,SNX25,BCAR3,NUAK1,PTPN11,TRIB3,PUM3,ILK,PPP1R12A,SESN2,PTGS2,IWS1,DNAJC10,TNIK,NXN,PTPN2,SPOPL,YES1,PLCL1,ADAM17,CNOT7,MAGED1,CTNNB1,APOE,LYN,STRAP,CCNYL1,MMD,CBLC,ATF3,VLDLR,HTT,CALM1,CDYL,PAK2,INHBA,CDKN1A,NRP1,SH3RF3,MADD,KDM3A,MID1	down_VSAP_EC
GO:0014896	0.00536	1	1	18	muscle hypertrophy	BP	1	GATA5	down_VFEM_VSMC
GO:0014897	0.00536	1	1	18	striated muscle hypertrophy	BP	1	GATA5	down_VFEM_VSMC
GO:0003300	0.00536	1	1	18	cardiac muscle hypertrophy	BP	1	GATA5	down_VFEM_VSMC
GO:0007188	0.00537	0.998	10	41	adenylate cyclase-modulating G protein-coupled receptor signaling	BP	0.849	ADRA1B,ADGRD1,ABCA1,PTGIR,ADCY6,GNA14,APLP1,SENP7,EDN1,GNAQ	up_ACOR_VSMC
GO:0045814	0.00537	0.999	7	27	negative regulation of gene expression,	BP	0.278	ATAD2,EZH2,UHRF1,SUV39H1,HAT1,HIST2H2AC,H2AFX	down_ACOR_VSMC

GO:0061099	0.00537	0.999	4	13	negative regulation of protein tyrosine kinase	BP	0.366	SOCS5,ERRFI1,PTPN2,CBLC	down_VSAP_EC
GO:0045047	0.00537	0.999	4	14	protein targeting to ER	BP	0.366	TRAM1,SRP54,SRPRA,SRP72	down_VSAP_EC
GO:0072599	0.00537	0.999	4	14	establishment of protein localization to endoplasmic reticulum	BP	0.366	TRAM1,SRP54,SRPRA,SRP72	down_VSAP_EC
GO:0040012	0.00538	0.998	15	303	regulation of locomotion	BP	1	CMKLR1,ANGPT4,HOXA7,PLXNA4,TWIST2,HTR1D,CAMK2A,LRRC15,RECK,TBXA2R,BVES,LGR6,CCL5,ADAM17,KANK1	up_AFEM_EC
GO:0042472	0.00538	1	2	27	inner ear morphogenesis	BP	0.607	EYA1,SIX1	up_VFEM_EC
GO:0046661	0.00538	1	2	45	male sex differentiation	BP	1	HOXA9,HOXA11	up_VFEM_VSMC
GO:1900182	0.00539	0.999	6	33	positive regulation of protein localization to	BP	1	ENSCAFG00000014978,TRIM8,GLI3,PARK7,MAVS,GLIS2	down_VCAV_EC
GO:0045605	0.00539	1	2	5	negative regulation of epidermal cell	BP	1	HOXA7,DLL1	up_AFEM_EC
GO:0051240	0.0054	0.999	6	489	positive regulation of multicellular organismal	BP	1	TBX5,NR1H4,EVI2B,CCL24,FLRT3,ARHGEF2	up_VPUL_EC
GO:0008016	0.0054	0.999	5	48	regulation of heart contraction	BP	1	IRX5,CHRM2,THRB,BVES,DMPK	up_AFEM_EC
GO:0048585	0.0054	0.999	5	475	negative regulation of response to stimulus	BP	1	BARX1,NKX2-5,BAMBI,ANKRD6,CYP26B1	up_VPOR_VSMC
GO:0007399	0.00542	0.997	50	564	nervous system development	BP	0.367	ARHGEF2,PRICKLE1,VEGFA,SIPA1L1,SULF1,HSPA5,ETV6,SGK1,PRKACB,CASP3,PTPN11,AARS,DPYSL3,HOOK3,IFRD1,TMED2,ILK,KIF5B,HIF1A,SLC45A3,TNIK,UBA6,ZPR1,UCHL5,EIF2B1,NEO1,UST,CDH11,CTNNB1,APOE,PLOD3,LYN,PDS5B,MMD,GSK3B,VLDLR,SDC4,REST,MAPK10,PRMT1,EXT1,DISC1,PAK2,LRRTM2,INHBA,CAMK2D,SIX4,NRP1,ARF4,YWHAH	down_VSAP_EC
GO:0010818	0.00542	1	2	5	T cell chemotaxis	BP	1	CCL5,ADAM17	up_AFEM_EC
GO:0043010	0.00543	0.998	16	96	camera-type eye development	BP	0.307	EPHB1,ARHGEF15,CHD7,DLL4,GATA3,TMOD1,PROX1,TSPAN12,SMARCA4,SLC25A25,JAG1,SKIL,HIF1A,ALDH1A3,ZEB1,CDON	up_VCAV_EC
GO:0060736	0.00543	1	1	5	prostate gland growth	BP	0.629	ESR1	up_AORT_VSMC
GO:0046621	0.00543	1	1	8	negative regulation of organ growth	BP	1	TBX5	up_APUL_VSMC
GO:0045088	0.00546	0.998	14	81	regulation of innate immune response	BP	0.307	GBP5,TNFAIP3,TNIP1,TMEM173,TLR4,NLRC5,PELI1,NECTIN2,RIOK3,TLR3,OTUD4,CNOT7,HMGB1,LSM14A	up_VCAV_EC
GO:0003008	0.00546	1	3	336	system process	BP	0.629	HOXC10,WNT7A,ARG2	up_AORT_VSMC
GO:1904377	0.00546	1	2	21	positive regulation of protein localization to cell	BP	0.64	ACSL3,STX4	down_ACOR_EC
GO:0048701	0.00546	1	2	25	embryonic cranial skeleton morphogenesis	BP	0.739	TBX15,IRX5	up_VSAP_VSMC
GO:1904659	0.00547	0.999	7	36	glucose transmembrane transport	BP	0.368	NR1H4,ENPP1,PTPN11,TRIB3,SESN2,YES1,SLC2A3	down_VSAP_EC
GO:0072358	0.00548	1	3	268	cardiovascular system development	BP	1	SLITRK5,C6,TGFBI	up_VCAV_VSMC
GO:0048262	0.00549	1	2	5	determination of dorsal/ventral asymmetry	BP	1	GREM1,GREM2	up_AFEM_EC
GO:0060911	0.00549	1	1	2	cardiac cell fate commitment	BP	1	NKX2-5	up_VPOR_VSMC
GO:0060923	0.00549	1	1	2	cardiac muscle cell fate commitment	BP	1	NKX2-5	up_VPOR_VSMC
GO:0042325	0.005495518	0.99739	22	463	regulation of phosphorylation	BP	0.96436359	PLCL1,PKIB,SOX9,NDRG2,ENSCAFG00000004331,FZD5,DUSP26,NOD2,SMAD6,PAK3,TNFRSF19,MAPK8IP2,MAVS,LRRC15,LRP4,SH3RF3,LRRC4,RARRES2,NRP1,PRKCD,DBNDD2,CCNYL1	up_ACOR_EC
GO:0060056	0.00551	1	2	2	mammary gland involution	BP	0.284	CAV1,B4GALT1	down_ACOR_VSMC
GO:0044062	0.00551	1	2	2	regulation of excretion	BP	0.309	STC1,EDN1	up_VCAV_EC
GO:0021796	0.00552	1	1	1	cerebral cortex regionalization	BP	0.739	EMX2	up_VSAP_VSMC
GO:0045596	0.00553	1	3	186	negative regulation of cell differentiation	BP	0.961	TMEM176A,TMEM176B,SLIT2	up_VPUL_VSMC
GO:0003179	0.00554	1	1	19	heart valve morphogenesis	BP	1	GATA5	down_VFEM_VSMC
GO:0002920	0.00554	1	1	4	regulation of humoral immune response	BP	1	C6	up_VCAV_VSMC

GO:0006874	0.00555	0.998	17	91	cellular calcium ion homeostasis	BP	0.849	ADRA1B,PLCG2,HTR2B,MICU2,ITPR1,F2RL1,DMPK,PRKCE,APP,PSEN1,CHD7,EDN1,DISC1,HERPUD1,WFS1,ATP2C1,STC2	up_ACOR_VSMC
GO:0048565	0.00555	1	1	38	digestive tract development	BP	1	GATA5	down_AFEM_VSMC
GO:0042325	0.00557	0.997	42	463	regulation of phosphorylation	BP	0.373	ARHGEF2,VEGFA,SOCS5,ENPP1,ERRFI1,JCAD,SNX25,BCAR3,PTPN11,TRIB3,PGAM1,ILK,SESN2,PTGS2,HIF1A,DYNLL1,DNAJC10,TNIK,PTPN2,YES1,PLCL1,ADAM17,CNOT7,MAGED1,CTNNB1,APOE,LYN,STRAP,CCNYL1,MMD,CBLC,ATF3,VLDLR,SH3GLB1,HTT,PAK2,INHBA,CDKN1A,NRP1,SH3RF3,MADD,MID1	down_VSAP_EC
GO:0006936	0.00557	0.998	13	74	muscle contraction	BP	0.309	EDNRB,DOCK4,PDE4D,STC1,EDN1,TMOD1,GUCY1A1,ARG2,MTOR,KCNJ2,P2RX2,ATP2A1,TMOD3	up_VCAV_EC
GO:1903708	0.00557	0.998	12	65	positive regulation of hemopoiesis	BP	0.309	HCLS1,IL7R,GATA3,CASP8,ITPKB,FES,JAG1,CREB1,HMGB1,RIPK1,ATP11C,HIF1A	up_VCAV_EC
GO:0048568	0.00557	0.999	4	148	embryonic organ development	BP	0.646	HOXA3,HOXA7,HOXA1,HOXC4	down_ACOR_EC
GO:2000484	0.00557	1	1	4	positive regulation of interleukin-8 secretion	BP	1	TLR2	up_AFEM_VSMC
GO:0055005	0.00557	1	1	2	ventricular cardiac myofibril assembly	BP	1	NKX2-5	up_VPOR_VSMC
GO:0071379	0.00558	1	4	8	cellular response to prostaglandin stimulus	BP	0.849	PRKAA2,ADCY6,PTGDR,PRKCE	up_ACOR_VSMC
GO:0002682	0.00558	1	3	343	regulation of immune system process	BP	0.636	HOXA9,ARG2,ESR1	up_AORT_VSMC
GO:0106106	0.00558	1	2	48	cold-induced thermogenesis	BP	1	HOXC10,LNPEP	up_AORT_EC
GO:0120161	0.00558	1	2	48	regulation of cold-induced thermogenesis	BP	1	HOXC10,LNPEP	up_AORT_EC
GO:0003263	0.00558	1	1	4	cardioblast proliferation	BP	1	EYA1	up_AFEM_VSMC
GO:0000154	0.00559	0.999	5	15	rRNA modification	BP	0.288	NOP56,FBL,ENSCAFG00000019620,NOP58,EMG1	down_ACOR_VSMC
GO:0032649	0.0056	0.999	6	23	regulation of interferon-gamma production	BP	0.309	PDE4D,TLR4,GATA3,IRF8,TLR3,HMGB1	up_VCAV_EC
GO:0051147	0.0056	1	2	44	regulation of muscle cell differentiation	BP	1	NKX2-5,CYP26B1	up_VPOR_VSMC
GO:0048645	0.00561	1	2	33	animal organ formation	BP	1	TBX5,HOXA3	up_VPUL_EC
GO:0010092	0.00561	1	1	19	specification of animal organ identity	BP	1	GATA5	down_VFEM_VSMC
GO:0006817	0.00561	1	1	5	phosphate ion transport	BP	0.636	SLC20A2	up_AORT_VSMC
GO:0030195	0.00562	0.999	4	10	negative regulation of blood coagulation	BP	0.309	PDGFB,CD34,KLKB1,TFPI	up_VCAV_EC
GO:1900047	0.00562	0.999	4	10	negative regulation of hemostasis	BP	0.309	PDGFB,CD34,KLKB1,TFPI	up_VCAV_EC
GO:0002831	0.00564	0.998	10	42	regulation of response to biotic stimulus	BP	1	CD274,F2RL1,SASH1,NLRX1,TKFC,RNF216,MAPKBP1,CYBA,PQBP1,MAVS	up_VSAP_EC
GO:0090596	0.00564	1	2	84	sensory organ morphogenesis	BP	1	EYA1,IRX5	up_AFEM_VSMC
GO:0032469	0.00565	0.999	5	13	endoplasmic reticulum calcium ion homeostasis	BP	0.852	ITPR1,APP,PSEN1,HERPUD1,WFS1	up_ACOR_VSMC
GO:2000312	0.00565	1	1	2	regulation of kainate selective glutamate	BP	1	NETO1	up_VFEM_VSMC
GO:0007187	0.00565341	0.99906	5	43	G protein-coupled receptor signaling pathway, coupled to cyclic nucleotide second	BP	0.96436359	PTH1R,GNAO1,PTGIR,PTGER4,GNAZ	up_ACOR_EC
GO:0001767	0.00567	1	1	2	establishment of lymphocyte polarity	BP	1	CYP26B1	up_VPOR_VSMC
GO:0001768	0.00567	1	1	2	establishment of T cell polarity	BP	1	CYP26B1	up_VPOR_VSMC
GO:0098528	0.00567	1	1	2	skeletal muscle fiber differentiation	BP	1	CYP26B1	up_VPOR_VSMC
GO:2001016	0.00567	1	1	2	positive regulation of skeletal muscle cell	BP	1	CYP26B1	up_VPOR_VSMC
GO:0002027	0.00568	1	1	20	regulation of heart rate	BP	1	JUP	down_VFEM_VSMC
GO:2000172	0.00568	1	1	2	regulation of branching morphogenesis of a nerve	BP	1	SHOX2	up_VFEM_VSMC
GO:0031349	0.00569	0.998	15	90	positive regulation of defense response	BP	0.312	GBP5,TNFAIP3,TNIP1,TMEM173,MEF2C,TLR4,NLRC5,PELI1,NECTIN2,RIOK3,TLR3,OTUD4,PTGS2,HMGB1,LSM14A	up_VCAV_EC
GO:2001225	0.00569	1	1	4	regulation of chloride transport	BP	1	CA2	up_VCAV_VSMC
GO:0010644	0.00569	1	1	1	cell communication by electrical coupling	BP	0.739	CAV1	up_VSAP_VSMC

GO:0019065	0.00569	1	1	1	receptor-mediated endocytosis of virus by	BP	0.739	CAV1	up_VSAP_VSMC
GO:0033484	0.00569	1	1	1	nitric oxide homeostasis	BP	0.739	CAV1	up_VSAP_VSMC
GO:0044860	0.00569	1	1	1	protein localization to plasma membrane raft	BP	0.739	CAV1	up_VSAP_VSMC
GO:0048550	0.00569	1	1	1	negative regulation of pinocytosis	BP	0.739	CAV1	up_VSAP_VSMC
GO:0072584	0.00569	1	1	1	caveolin-mediated endocytosis	BP	0.739	CAV1	up_VSAP_VSMC
GO:0075509	0.00569	1	1	1	endocytosis involved in viral entry into host cell	BP	0.739	CAV1	up_VSAP_VSMC
GO:0086064	0.00569	1	1	1	cell communication by electrical coupling involved in cardiac	BP	0.739	CAV1	up_VSAP_VSMC
GO:0086098	0.00569	1	1	1	angiotensin-activated signaling pathway	BP	0.739	CAV1	up_VSAP_VSMC
GO:1900085	0.00569	1	1	1	negative regulation of peptidyl-tyrosine	BP	0.739	CAV1	up_VSAP_VSMC
GO:1901844	0.00569	1	1	1	regulation of cell communication by electrical coupling	BP	0.739	CAV1	up_VSAP_VSMC
GO:1901979	0.00569	1	1	1	regulation of inward rectifier potassium	BP	0.739	CAV1	up_VSAP_VSMC
GO:1903596	0.00569	1	1	1	regulation of gap junction assembly	BP	0.739	CAV1	up_VSAP_VSMC
GO:1903598	0.00569	1	1	1	positive regulation of gap junction assembly	BP	0.739	CAV1	up_VSAP_VSMC
GO:1903609	0.00569	1	1	1	negative regulation of inward rectifier potassium channel activity	BP	0.739	CAV1	up_VSAP_VSMC
GO:0010649	0.00569	1	1	1	regulation of cell communication by	BP	0.739	CAV1	up_VSAP_VSMC
GO:0072330	0.0057	0.998	10	68	monocarboxylic acid biosynthetic process	BP	0.378	ASNS,NR1H4,PGK1,TPI1,TRIB3,PGAM1,PTGS2,HIF1A,SLC45A3,ALDOC	down_VSAP_EC
GO:0001823	0.00571	1	2	45	mesonephros development	BP	1	EYA1,HOXA11	up_VFEM_VSMC
GO:0007275	0.00572	1	4	1370	multicellular organism development	BP	1	HOXA3,TBX5,HOXA2,HOXB5	up_APUL_VSMC
GO:0060374	0.00572	1	2	2	mast cell differentiation	BP	0.313	KIT,GATA3	up_VCAV_EC
GO:0003012	0.00574	0.998	15	90	muscle system process	BP	0.313	EDNRB,DOCK4,PDE4D,MEF2C,STC1,EDN1,TMOD1,PPP3CA,GUCY1A1,ARG2,MTOR,KCNJ2,P2RX2,ATP2A1,TMOD3	up_VCAV_EC
GO:2001243	0.00574	1	2	33	negative regulation of intrinsic apoptotic	BP	1	PTGER2,ARHGEF2	up_VPUL_EC
GO:0003156	0.00574	1	1	20	regulation of animal organ formation	BP	1	GATA5	down_VFEM_VSMC
GO:0070372	0.00575	0.998	16	85	regulation of ERK1 and ERK2 cascade	BP	0.859	HTR2B,BMP4,FGFR2,FGF18,NDRG2,F2RL1,NRP1,PTPN22,NPNT,FRS2,APP,SYNJ2BP,CCL5,DUSP26,ENSCAFG00000029553,CRKL	up_ACOR_VSMC
GO:0061550	0.00575	1	3	6	cranial ganglion development	BP	0.38	CTNNB1,SIX4,NRP1	down_VSAP_EC
GO:0051129	0.005750515	0.99786	13	221	negative regulation of cellular component organization	BP	0.96436359	TMOD1,ARHGAP6,RGMA,SMAD6,ARHGAP28,PTGER4,LRP4,CLSTN1,CLU,CHMP4B,NRP1,PRKCD,TUBB4A	up_ACOR_EC
GO:0009615	0.00577	0.998	14	83	response to virus	BP	0.314	MX2,TMEM173,NLRC5,GATA3,SERINC5,ODC1,RIOK3,STMN1,TLR3,OAS3,CNOT7,PMAIP1,LSM14A,IVNS1ABP	up_VCAV_EC
GO:0022027	0.00579	1	2	2	interkinetic nuclear migration	BP	0.314	HOOK3,HHEX	up_VCAV_EC
GO:0010556	0.00581	0.998	11	1137	regulation of macromolecule	BP	0.739	HOXA11,HOXA10,HOXA9,PITX1,EMX2,SALL1,IRX5,PRR16,TLR2,CAV1,SHOX2	up_VSAP_VSMC
GO:0006355	0.00581	0.998	10	974	regulation of transcription, DNA-	BP	0.739	HOXA11,HOXA10,HOXA9,PITX1,EMX2,SALL1,IRX5,TLR2,CAV1,SHOX2	up_VSAP_VSMC
GO:0038166	0.00581	1	1	4	angiotensin-activated signaling pathway	BP	1	CA2	up_VCAV_VSMC
GO:0009790	0.00583	1	3	335	embryo development	BP	0.653	HOXC10,HOXA9,WNT7A	up_AORT_VSMC
GO:0055123	0.00583	1	1	40	digestive system development	BP	1	GATA5	down_AFEM_VSMC
GO:1901228	0.00583	1	1	4	positive regulation of transcription from RNA polymerase II promoter involved in heart	BP	1	GATA5	up_VCAV_VSMC
GO:0060413	0.00585	1	1	8	atrial septum morphogenesis	BP	1	TBX5	up_APUL_VSMC
GO:0003283	0.00585	1	1	8	atrial septum development	BP	1	TBX5	up_APUL_VSMC

GO:0002683	0.00586	0.997	19	126	negative regulation of immune system process	BP	0.315	IL7R,ADGRF5,TNFAIP3,NLRC5,MMP28,HOXB8,PELI1,ARG2,ITPKB,RIOK3,THOC1,APOD,OTUD4,CD200,BCL10,CNOT7,HMGB1,CUL4A,TJP2	up_VCAV_EC
GO:0010628	0.00586	0.999	7	527	positive regulation of gene expression	BP	0.739	HOXA11,HOXA9,SALL1,PRR16,TLR2,CAV1,SHOX2	up_VSAP_VSMC
GO:0072009	0.00586	1	2	48	nephron epithelium development	BP	1	EYA1,HOXA11	up_VFEM_VSMC
GO:0055117	0.00586	1	1	21	regulation of cardiac muscle contraction	BP	1	JUP	down_VFEM_VSMC
GO:0042325	0.00587	0.996	54	463	regulation of phosphorylation	BP	0.315	EPHB1,TEK,HCLS1,KIT,PPP2R5A,TNFAIP3,MAP3K11,TNIP1,PDGFB,STK10,RELN,PDE4D,ATF3,RAPGEF1,TLR4,NLRC5,EDN1,RALB,APLNR,NEDD9,YES1,MTOR,MAP3K3,BMP2,HHEX,PREX2,SLK,SH3BP5,ITPKB,PROX1,MADD,PAK2,PRKD2,SH3GLB1,TLR3,NCK1,LDB2,NSD1,PTGS2,MMD,RAPGEF2,TAOK1,CNOT7,HMGB1,BLM,SPTBN4,RIPK1,ITGA6,RAD17,PDGFD,HIF1A,JCAD,ARHGEF2,CDON	up_VCAV_EC
GO:0045664	0.00587	0.997	22	144	regulation of neuron differentiation	BP	0.315	EDNRB,IFRD1,RELN,MEF2C,RAPGEF1,GATA3,RAB11A,TCF4,PPP3CA,BMP2,HMG20A,GDPD5,PROX1,NCK1,FES,JAG1,MMD,RAPGEF2,ZC4H2,ZEB1,ARHGEF2,CDON	up_VCAV_EC
GO:0042118	0.00587	1	1	4	endothelial cell activation	BP	1	HOXA9	up_AFEM_VSMC
GO:0030099	0.00588	0.998	16	133	myeloid cell differentiation	BP	0.385	VEGFA,MYH9,ACTN1,CASP3,PTPN11,WDR1,HOXB7,HIF1A,PTPN2,EVI2B,CTNNB1,TFRC,LYN,PRMT1,GLO1,INHBA	down_VSAP_EC
GO:0051216	0.00589	0.998	14	70	cartilage development	BP	1	HOXA11,AXIN2,OSR2,HOXD3,OSR1,ZBTB16,RARG,HOXC4,ZEB1,FBXW4,SULF2,SHOX2,NFIB,TGFBR2	up_VSAP_EC
GO:0032101	0.005891594	0.99801	11	173	regulation of response to external stimulus	BP	0.96436359	NR1H4,FNDC4,NOD2,IL33,RGMA,MMP28,PTGER4,MAVS,RARRES2,NRP1,PRKCD	up_ACOR_EC
GO:0003180	0.0059	1	1	7	aortic valve morphogenesis	BP	1	GATA5	down_VSAP_VSMC
GO:1905606	0.00591	1	1	5	regulation of presynapse assembly	BP	0.655	WNT7A	up_AORT_VSMC
GO:0060017	0.00591	1	1	3	parathyroid gland development	BP	0.961	HOXA3	up_VPUL_VSMC
GO:0090090	0.00593	1	2	44	negative regulation of canonical Wnt signaling	BP	1	NKX2-5,ANKRD6	up_VPOR_VSMC
GO:1900424	0.00593	1	1	5	regulation of defense response to bacterium	BP	0.655	ARG2	up_AORT_VSMC
GO:0048706	0.00594	1	2	59	embryonic skeletal system development	BP	0.961	HOXA3,HOXB5	up_VPUL_VSMC
GO:0045637	0.00595	0.998	13	73	regulation of myeloid cell differentiation	BP	0.318	HCLS1,MEF2C,HOXB8,CASP8,MTOR,ITPKB,FES,JAG1,CREB1,RIPK1,CUL4A,HIF1A,TJP2	up_VCAV_EC
GO:0060047	0.00595	0.998	12	57	heart contraction	BP	0.881	TNNT2,ADRA1B,KCNE3,GJA1,PDE5A,DMPK,RNLS,JUP,EDN1,GAA,SLC8A1,BVES	up_ACOR_VSMC
GO:1903506	0.00595	0.998	10	977	regulation of nucleic acid-templated transcription	BP	0.742	HOXA11,HOXA10,HOXA9,PITX1,EMX2,SALL1,IRX5,TLR2,CAV1,SHOX2	up_VSAP_VSMC
GO:0036005	0.00595	1	1	4	response to macrophage colony-stimulating factor	BP	1	TLR2	up_AFEM_VSMC
GO:0036006	0.00595	1	1	4	cellular response to macrophage colony-	BP	1	TLR2	up_AFEM_VSMC
GO:0051241	0.00596	0.998	16	336	negative regulation of multicellular organismal process	BP	1	HOXA9,CMKLR1,IRX3,HOXC10,CRY2,ANGPT4,HOXA7,DLL1,BPI,TWIST2,THRB,GREM1,CYLD,TBXA2R,AXIN2,KANK1	up_AFEM_EC
GO:0005985	0.00596	1	1	2	sucrose metabolic process	BP	1	FBP1	up_VFEM_VSMC
GO:0005986	0.00596	1	1	2	sucrose biosynthetic process	BP	1	FBP1	up_VFEM_VSMC
GO:0003129	0.00596	1	1	3	heart induction	BP	0.961	GATA5	up_VPUL_VSMC
GO:0042733	0.00598	1	2	27	embryonic digit morphogenesis	BP	0.742	HOXA11,SALL1	up_VSAP_VSMC
GO:0051891	0.00598	1	1	3	positive regulation of cardioblast differentiation	BP	0.961	TBX5	up_VPUL_VSMC
GO:0019220	0.005989672	0.99705	24	525	regulation of phosphate metabolic process	BP	0.96436359	NR1H4,PLCL1,PKIB,SOX9,NDRG2,ENSCAFG00000004331,FZD5,DUSP26,NOD2,SMAD6,PAK3,TNFRSF19,MAPK8IP2,MAVS,LRRC15,LRP4,SH3RF3,LRRC4,RARRES2,NRP1,PRKCD,PPP1R26,DBNDD2,CCNYL1	up_ACOR_EC
GO:0051174	0.005989672	0.99705	24	525	regulation of phosphorus metabolic process	BP	0.96436359	NR1H4,PLCL1,PKIB,SOX9,NDRG2,ENSCAFG00000004331,FZD5,DUSP26,NOD2,SMAD6,PAK3,TNFRSF19,MAPK8IP2,MAVS,LRRC15,LRP4,SH3RF3,LRRC4,RARRES2,NRP1,PRKCD,PPP1R26,DBNDD2,CCNYL1	up_ACOR_EC

GO:1901213	0.00599	1	1	7	regulation of transcription from RNA polymerase II promoter involved in	BP	1	GATA5	down_VSAP_VSMC
GO:0065009	0.006	0.996	79	735	regulation of molecular function	BP	0.318	TEK,HCLS1,KIT,SELE,PPP2R5A,PRKCH,ARHGEF15,TNFAIP3,MAP3K11,AMOT,PDGFB,STK10,TMEM173,RELN,FGD6,PDE4D,MEF2C,RAPGEF1,TLR4,NLRC5,GATA3,EDN1,RALB,CTTNBP2NL,HOMER3,NEDD9,ARHGAP28,PELI1,PPP3CA,CASP8,MTOR,MAP3K3,BMP2,HHEX,CHN2,PREX2,SLK,SH3BP5,CNOT6,PPP1R2,PROX1,IQGAP2,ENSCAFG00000010596,MADD,STMN1,HMBOX1,PAK2,PRKD2,SH3GLB1,SMARCA4,TLR3,MFNG,LDB2,CD200,NSD1,PTGS2,MMD,RAPGEF2,PLCG2,BCL10,GCH1,HMGB1,TBC1D1,NOS2,PMAIP1,BLM,RIPK1,ITGA6,PDGFD,KDM5A,HIF1A,ALS2,ENSCAFG00000002015,FKBP15,PSMB9,RABGAP1,ARHGEF2,CDON,USP6NL	up_VCAV_EC
GO:0003008	0.006	0.998	16	336	system process	BP	1	IRX5,HOXC10,LEP,CRY2,F5,DLL1,LMCD1,CHRM2,THRB,TMEM100,HTR1D,AVPR1A,TBXA2R,REEP2,BVES,DMPK	up_AFEM_EC
GO:1900122	0.00601	1	1	3	positive regulation of receptor binding	BP	0.961	HOXA3	up_VPUL_VSMC
GO:0002263	0.00602	0.998	13	68	cell activation involved in immune response	BP	0.318	KIT,IL6,TLR4,GATA3,RAB27A,MTOR,SNAP23,THOC1,SWAP70,MFNG,FES,PLCG2,HMGB1	up_VCAV_EC
GO:0031214	0.00602	1	3	45	biomineral tissue development	BP	1	HOXA3,ASPN,ACVR2A	up_VPOR_EC
GO:0090091	0.00602	1	1	3	positive regulation of extracellular matrix	BP	1	FSCN1	down_AFEM_EC
GO:0035637	0.00602	1	1	21	multicellular organismal signaling	BP	1	JUP	down_VFEM_VSMC
GO:0042326	0.00603	0.999	4	163	negative regulation of phosphorylation	BP	0.69	LYN,IGF1R,PRKAR2A,ASPN	down_ACOR_EC
GO:0001501	0.00603	1	2	178	skeletal system development	BP	1	HOXA3,HOXB5	up_APUL_VSMC
GO:0032675	0.00603	1	2	34	regulation of interleukin-6 production	BP	1	NR1H4,ARHGEF2	up_VPUL_EC
GO:0061343	0.00603	1	1	3	cell adhesion involved in heart morphogenesis	BP	0.961	GATA5	up_VPUL_VSMC
GO:1901605	0.00604	0.998	10	67	alpha-amino acid metabolic process	BP	0.39	P4HB,ASNS,PLOD2,NR1H4,ERO1A,P4HA1,NMNAT2,ALDH18A1,PLOD3,BCAT1	down_VSAP_EC
GO:0010596	0.00604	1	1	21	negative regulation of endothelial cell migration	BP	1	JUP	down_VFEM_VSMC
GO:2001141	0.00605	0.998	10	979	regulation of RNA biosynthetic process	BP	0.746	HOXA11,HOXA10,HOXA9,PITX1,EMX2,SALL1,IRX5,TLR2,CAV1,SHOX2	up_VSAP_VSMC
GO:0072513	0.00605	1	1	3	positive regulation of secondary heart field cardioblast proliferation	BP	0.961	TBX5	up_VPUL_VSMC
GO:0038084	0.00606	0.999	4	13	vascular endothelial growth factor signaling	BP	0.39	VEGFA,JCAD,MYO1C,NRP1	down_VSAP_EC
GO:0030516	0.00606	1	2	15	regulation of axon extension	BP	1	SEMA3A,RNF6	up_VPOR_EC
GO:0003214	0.00606	1	1	3	cardiac left ventricle morphogenesis	BP	0.961	TBX5	up_VPUL_VSMC
GO:0021615	0.00609	1	1	3	glossopharyngeal nerve morphogenesis	BP	0.961	HOXA3	up_VPUL_VSMC
GO:0045617	0.00611	1	1	3	negative regulation of keratinocyte	BP	1	SRSF6	down_AFEM_EC
GO:0021889	0.00611	1	1	3	olfactory bulb interneuron	BP	0.961	SLIT2	up_VPUL_VSMC
GO:0021891	0.00611	1	1	3	olfactory bulb interneuron development	BP	0.961	SLIT2	up_VPUL_VSMC
GO:0051093	0.00612	0.997	35	273	negative regulation of developmental process	BP	0.322	EPHB1,PLK2,EDNRB,TEK,ARHGEF15,IFRD1,TIE1,ADAMTS9,AMOT,PDGFB,FERMT1,DLL4,GPR4,GATA3,HOXB8,PPP3CA,HOOK3,MAP3K3,BMP2,HMG20A,HHEX,ITPKB,ANGPT2,THOC1,JAG1,RAPGEF2,CTNNA1,HMGB1,SKIL,ARHGDIB,CUL4A,HIF1A,VASH1,ZEB1,TJP2	up_VCAV_EC
GO:0009887	0.00612	0.999	5	314	animal organ morphogenesis	BP	1	TBX5,HOXA3,NPNT,NTRK2,HOXA1	up_APUL_EC
GO:0021957	0.00612	1	1	3	corticospinal tract morphogenesis	BP	0.961	SLIT2	up_VPUL_VSMC
GO:0022028	0.00613	1	1	3	tangential migration from the subventricular zone to the olfactory bulb	BP	0.961	SLIT2	up_VPUL_VSMC
GO:0050707	0.006138209	0.99896	5	44	regulation of cytokine secretion	BP	0.96436359	NR1H4,FZD5,NOD2,PTGER4,MAVS	up_ACOR_EC
GO:0032612	0.00614	0.999	6	22	interleukin-1 production	BP	0.322	GBP5,TNFAIP3,TLR4,ARG2,ABCA1,HMGB1	up_VCAV_EC

GO:0050923	0.00614	1	2	2	regulation of negative chemotaxis	BP	1	SLIT2,PLXNA4	up_VSAP_EC
GO:0048742	0.00615	1	1	2	regulation of skeletal muscle fiber development	BP	1	SHOX2	up_VFEM_VSMC
GO:1990845	0.00616	1	2	51	adaptive thermogenesis	BP	1	HOXC10,LNPEP	up_AORT_EC
GO:0045931	0.00617	0.998	10	49	positive regulation of mitotic cell cycle	BP	0.313	BIRC5,CDK1,CDK4,NDC80,NUP62,DTL,ENSCAFG00000005299,UBE2C,MTBP,GEN1	down_ACOR_VSMC
GO:0046640	0.00617	1	1	6	regulation of alpha-beta T cell proliferation	BP	0.674	ARG2	up_AORT_VSMC
GO:0051494	0.006182931	0.99895	5	44	negative regulation of cytoskeleton organization	BP	0.96436359	TMOD1,ARHGAP6,ARHGAP28,PRKCD,TUBB4A	up_ACOR_EC
GO:0106106	0.00619	1	2	48	cold-induced thermogenesis	BP	1	HOXC10,GATM	up_VFEM_VSMC
GO:0120161	0.00619	1	2	48	regulation of cold-induced thermogenesis	BP	1	HOXC10,GATM	up_VFEM_VSMC
GO:0098581	0.00619	1	1	5	detection of external biotic stimulus	BP	1	TLR2	up_AFEM_VSMC
GO:0060444	0.00619	1	1	5	branching involved in mammary gland duct	BP	0.674	ESR1	up_AORT_VSMC
GO:0043500	0.0062	1	1	21	muscle adaptation	BP	1	GATA5	down_VFEM_VSMC
GO:0048856	0.00622	0.999	9	1533	anatomical structure development	BP	1	CHL1,HOXA3,BARX1,NKX2-5,BAMBI,TRPC4,ANKRD6,CYP26B1,C3AR1	up_VPOR_VSMC
GO:0002260	0.00622	0.999	5	15	lymphocyte homeostasis	BP	0.326	TNFAIP3,MEF2C,BCL10,SKIL,HIF1A	up_VCAV_EC
GO:0060429	0.00622	1	3	341	epithelium development	BP	0.674	WNT7A,ARG2,ESR1	up_AORT_VSMC
GO:0051492	0.006238924	0.99921	4	28	regulation of stress fiber assembly	BP	0.96436359	ARHGAP6,ARHGAP28,PTGER4,NRP1	up_ACOR_EC
GO:0007517	0.00624	0.997	16	100	muscle organ development	BP	0.326	EPHB1,MEF2C,ATF3,CHD7,DLL4,EDN1,FOXN2,PPP3CA,BMP2,CAV2,PROX1,FLNB,CREB1,NR1D2,P2RX2,CDON	up_VCAV_EC
GO:0034695	0.00624	0.999	4	8	response to prostaglandin E	BP	0.896	PRKAA2,ADCY6,PTGDR,PRKCE	up_ACOR_VSMC
GO:0001942	0.00625738	0.99921	4	28	hair follicle development	BP	0.96436359	CDH3,SOX9,TNFRSF19,LRP4	up_ACOR_EC
GO:0022404	0.00625738	0.99921	4	28	molting cycle process	BP	0.96436359	CDH3,SOX9,TNFRSF19,LRP4	up_ACOR_EC
GO:0022405	0.00625738	0.99921	4	28	hair cycle process	BP	0.96436359	CDH3,SOX9,TNFRSF19,LRP4	up_ACOR_EC
GO:0051222	0.006271917	0.9983	8	103	positive regulation of protein transport	BP	0.96436359	NR1H4,KCNB1,EDEM2,FZD5,NOD2,PTGER4,MAVS,PRKCD	up_ACOR_EC
GO:0007015	0.00628	0.997	17	144	actin filament organization	BP	0.4	ARHGEF2,ACTN1,VASP,TPM4,FSCN1,DPYSL3,WDR1,TMSB10,ACTR3,TWF1,PLEKHG2,PLS3,CAPG,NRP1,ENSCAFG00000030508,TNFAIP1,MYBPC2	down_VSAP_EC
GO:0002062	0.00628	1	2	48	chondrocyte differentiation	BP	1	HOXA11,SHOX2	up_VFEM_VSMC
GO:0010002	0.00628	1	1	9	cardioblast differentiation	BP	1	TBX5	up_APUL_VSMC
GO:0003140	0.00629	1	1	3	determination of left/right asymmetry in	BP	1	CITED2	down_VPOR_EC
GO:0030182	0.0063	0.997	32	327	neuron differentiation	BP	0.4	ARHGEF2,SIPA1L1,SGK1,CASP3,PTPN11,DPYSL3,IFRD1,KIF5B,HIF1A,TNIK,UBA6,ZPR1,NEO1,UST,CDH11,CTNNB1,APOE,LYN,MMD,GSK3B,VLDLR,SDC4,REST,MAPK10,PRMT1,EXT1,DISC1,PAK2,INHBA,NRP1,ARF4,YWHAH	down_VSAP_EC
GO:0051480	0.0063	1	2	50	regulation of cytosolic calcium ion concentration	BP	1	TRPC4,C3AR1	up_VPOR_VSMC
GO:0031291	0.00632	1	1	3	Ran protein signal transduction	BP	1	GPSM2	down_AFEM_EC
GO:0044089	0.00634	0.997	24	170	positive regulation of cellular component biogenesis	BP	0.329	EPHB1,TEK,GBP5,ARHGEF15,BAIAP2L1,TLR4,EDN1,RALB,IRF8,MTOR,CNOT6,NCKAP1,PROX1,IQGAP2,SH3GLB1,SWAP70,NCK1,LDB2,FES,PAN3,PMAIP1,ARPC5L,ENSCAFG00000002015,FSCN1	up_VCAV_EC
GO:0030326	0.00637	1	2	51	embryonic limb morphogenesis	BP	1	HOXC10,HOXA9	up_AORT_EC
GO:0035113	0.00637	1	2	51	embryonic appendage morphogenesis	BP	1	HOXC10,HOXA9	up_AORT_EC
GO:0008645	0.00638	0.999	7	37	hexose transmembrane transport	BP	0.401	NR1H4,ENPP1,PTPN11,TRIB3,SESN2,YES1,SLC2A3	down_VSAP_EC
GO:0015749	0.00638	0.999	7	37	monosaccharide transmembrane transport	BP	0.401	NR1H4,ENPP1,PTPN11,TRIB3,SESN2,YES1,SLC2A3	down_VSAP_EC
GO:0034219	0.00638	0.999	7	37	carbohydrate transmembrane transport	BP	0.401	NR1H4,ENPP1,PTPN11,TRIB3,SESN2,YES1,SLC2A3	down_VSAP_EC
GO:0045653	0.00638	1	1	3	negative regulation of megakaryocyte	BP	1	GABPA	down_AFEM_EC

GO:0009617	0.0064	0.997	22	133	response to bacterium	BP	0.896	VEGFD,TLR1,PLCG2,TMEM255A,COCH,ABCA1,ISG15,PTGIR,SASH1,PLAC8B,ANKRD1,F2RL1,BNIP3,SLC17A5,GPM6A,PRKCE,CHD7,NOD2,NAALADL2,B2M,TNFRSF11B,WASL	up_ACOR_VSMC
GO:0032535	0.0064	0.997	20	128	regulation of cellular component size	BP	0.331	HCLS1,IL7R,IFRD1,LRRC8D,BAIAP2L1,EDN1,RAB11A,TMOD1,MTOR,LRRC8C,SLC12A7,NCKAP1,IQGAP2,SWAP70,NCK1,CREB1,ARPC5L,ALS2,HP1BP3,TMOD3	up_VCAV_EC
GO:0035519	0.0064	1	2	2	protein K29-linked ubiquitination	BP	0.323	UBE2S,UBE2T	down_ACOR_VSMC
GO:0031589	0.00641	0.999	3	93	cell-substrate adhesion	BP	0.713	HOXA7,TIAM1,RADIL	up_VFEM_EC
GO:0032388	0.006414809	0.99865	6	62	positive regulation of intracellular transport	BP	0.96436359	CDH3,KCNB1,EDEM2,ACTN2,MAVS,PRKCD	up_ACOR_EC
GO:0048856	0.00643	0.999	9	1533	anatomical structure development	BP	1	HOXC10,EYA1,GATM,HOXA9,SPINK5,TBX15,EMX2,HOXA11,SHOX2	up_VFEM_VSMC
GO:0043271	0.00643	1	2	28	negative regulation of ion transport	BP	0.718	HOXA3,NEDD4	down_ACOR_EC
GO:0060442	0.00645	1	1	6	branching involved in prostate gland	BP	0.681	ESR1	up_AORT_VSMC
GO:0071392	0.00645	1	1	6	cellular response to estradiol stimulus	BP	0.681	ESR1	up_AORT_VSMC
GO:0048245	0.00645	1	1	2	eosinophil chemotaxis	BP	1	CCL24	up_VPUL_EC
GO:0060675	0.00646	0.999	6	29	ureteric bud morphogenesis	BP	0.403	ENSCAFG00000032470,ILK,HOXB7,MAGED1,CTNNB1,SIX4	down_VSAP_EC
GO:0048752	0.00646	1	1	5	semicircular canal morphogenesis	BP	1	EYA1	up_AFEM_VSMC
GO:0070920	0.00646	1	1	6	regulation of production of small RNA involved in gene silencing by RNA	BP	0.681	ESR1	up_AORT_VSMC
GO:1903798	0.00646	1	1	6	regulation of production of miRNAs involved in gene silencing by miRNA	BP	0.681	ESR1	up_AORT_VSMC
GO:0030216	0.00647	1	2	29	keratinocyte differentiation	BP	0.715	HOXA7,CYP26B1	up_VFEM_EC
GO:0051865	0.00648	0.998	8	31	protein autoubiquitination	BP	0.896	SH3RF2,SASH1,MARCH5,SH3RF1,RNF141,SH3RF3,RNF181,CNOT4	up_ACOR_VSMC
GO:0072132	0.00648	1	1	23	mesenchyme morphogenesis	BP	1	GATA5	down_VFEM_VSMC
GO:0007268	0.00649	0.997	22	130	chemical synaptic transmission	BP	0.896	PLCL1,FBXL20,HRH1,GLRB,STX11,TMEM108,EGLN1,CACNB3,SLC12A7,DMPK,SLITRK5,PRKCE,RNF19A,APP,PSEN1,EDN1,KCNC4,LRRC4,FAM107A,INSYN1,PREPL,PARK7	up_ACOR_VSMC
GO:0098916	0.00649	0.997	22	130	anterograde trans-synaptic signaling	BP	0.896	PLCL1,FBXL20,HRH1,GLRB,STX11,TMEM108,EGLN1,CACNB3,SLC12A7,DMPK,SLITRK5,PRKCE,RNF19A,APP,PSEN1,EDN1,KCNC4,LRRC4,FAM107A,INSYN1,PREPL,PARK7	up_ACOR_VSMC
GO:0007175	0.0065	1	3	7	negative regulation of epidermal growth factor-activated receptor activity	BP	0.403	SOCS5,ERRFI1,CBLC	down_VSAP_EC
GO:2000272	0.0065	1	3	7	negative regulation of signaling receptor activity	BP	0.403	SOCS5,ERRFI1,CBLC	down_VSAP_EC
GO:0030048	0.00651	1	1	23	actin filament-based movement	BP	1	JUP	down_VFEM_VSMC
GO:0046326	0.006517214	0.99949	3	15	positive regulation of glucose import	BP	0.96436359	NR1H4,ENSCAFG00000004331,RARRES2	up_ACOR_EC
GO:0031334	0.00653	0.997	14	84	positive regulation of protein complex assembly	BP	0.335	GBP5,BAIAP2L1,TLR4,IRF8,MTOR,CNOT6,NCKAP1,IQGAP2,NCK1,FES,PAN3,PMAIP1,ARPC5L,FSCN1	up_VCAV_EC
GO:1903587	0.00654	1	3	5	regulation of blood vessel endothelial cell proliferation involved in sprouting angiogenesis	BP	0.335	DLL4,APLNR,JCAD	up_VCAV_EC
GO:1902202	0.00655	1	2	3	regulation of hepatocyte growth factor receptor	BP	0.404	PTPN2,ESM1	down_VSAP_EC
GO:0035295	0.00656	1	3	356	tube development	BP	0.688	WNT7A,ARG2,ESR1	up_AORT_VSMC
GO:0098742	0.00656	1	1	44	cell-cell adhesion via plasma-membrane	BP	1	GATA5	down_AFEM_VSMC
GO:0019722	0.00658	0.998	9	45	calcium-mediated signaling	BP	0.335	EDNRB,SELP,CXCR4,PDE4D,EDN1,HOMER3,PPP3CA,MTOR,MCU	up_VCAV_EC

GO:0048513	0.00659	0.996	73	906	animal organ development	BP	0.404	PRICKLE1,VEGFA,SULF1,SERPINH1,MYH9,NR1H4,HSPA5,SERPINE1,ACTN1,SOCS5,ETV6,SPARC,LTBP1,ERRFI1,CASP3,BCAR3,PTPN11,AARS,FLNB,ENSCAFG00000032470,HOOK3,TMED2,ILK,FHL2,SMARCA4,TGFBR1,PTGS2,WDR1,CHSY1,MSN,HOXB7,HIF1A,SLC20A1,ACTG2,UBA6,ZPR1,PTPN2,UCHL5,EVI2B,ACTA2,ADAMTS6,MAGED1,CTNNB1,ANLN,CRYAB,TFRC,PLOD3,KLF5,LYN,STRAP,PDS5B,PLS3,ATF3,GSK3B,VLDLR,NEXN,MYO1E,SDC4,HTT,REST,PRMT1,SH2B3,EXT1,GLO1,DISC1,CREB3L2,INHBA,BTK,SIX4,NRP1,SPINK2,RTCB,HOMER1	down_VSAP_EC
GO:0002009	0.00659	0.997	20	177	morphogenesis of an epithelium	BP	0.404	PRICKLE1,SULF1,PRKACB,PTK7,EXOC5,ENSCAFG00000032470,TMED2,ILK,WDR1,MSN,HOXB7,HIF1A,ADAM17,MAGED1,CTNNB1,STARD13,PLOD3,SDC4,SIX4,NRP1	down_VSAP_EC
GO:0061448	0.00659	0.999	3	89	connective tissue development	BP	0.781	HOXA11,PITX1,SHOX2	up_VSAP_VSMC
GO:0034654	0.0066	0.999	8	1236	nucleobase-containing compound biosynthetic	BP	1	EYA1,HOXA10,HOXA9,TBX15,FBP1,EMX2,HOXA11,SHOX2	up_VFEM_VSMC
GO:0021697	0.00661	1	1	6	cerebellar cortex formation	BP	0.688	WNT7A	up_AORT_VSMC
GO:0060017	0.00661	1	1	3	parathyroid gland development	BP	1	HOXA3	up_VPOR_VSMC
GO:0060538	0.00662	0.998	10	51	skeletal muscle organ development	BP	0.335	EPHB1,MEF2C,ATF3,FOXN2,PPP3CA,CAV2,FLNB,NR1D2,P2RX2,CDON	up_VCAV_EC
GO:0003148	0.00662	0.999	5	13	outflow tract septum morphogenesis	BP	0.898	SMAD6,BMP4,FGFR2,NRP1,TBX20	up_ACOR_VSMC
GO:0030201	0.00662	0.999	4	12	heparan sulfate proteoglycan metabolic	BP	0.405	SULF1,ENSCAFG00000032470,CTNNB1,EXT1	down_VSAP_EC
GO:2000026	0.00663	1	2	560	regulation of multicellular organismal development	BP	1	JUP,GATA5	down_VFEM_VSMC
GO:0006939	0.00663	1	2	32	smooth muscle contraction	BP	1	HTR7,NPNT	up_APUL_EC
GO:0048844	0.00663	1	2	30	artery morphogenesis	BP	0.727	EYA1,SIX1	up_VFEM_EC
GO:0045651	0.00664	1	3	6	positive regulation of macrophage	BP	0.335	HCLS1,CASP8,RIPK1	up_VCAV_EC
GO:0009888	0.00665	0.996	62	545	tissue development	BP	0.335	EPHB1,EDNRB,HOXB7,PRKCH,IFRD1,ADAMTS9,PDGFB,PODXL,PDE4D,MEF2C,FERMT1,ATF3,CHD7,TIPARP,DLL4,RAPGEF1,GPR4,STC1,GATA3,EDN1,NTN4,FOXN2,SOX18,TMOD1,PPP3CA,MTSS1,ARG2,MTOR,BMP2,HHEX,CAV2,BMP2K,PROX1,ENSCAFG00000010596,ARID4B,FLNB,APOD,BTBD7,EXOC5,TIMELESS,HOXB5,LDB2,SLC25A25,JAG1,PTGS2,RAPGEF2,CREB1,BCL10,BASP1,NUP133,ITGA6,SKIL,NR1D2,HIF1A,ATRX,ALDH1A3,P2RX2,LUZP1,RBBP6,ZEB1,FBXO5,CDON	up_VCAV_EC
GO:0072593	0.00665	0.998	12	68	reactive oxygen species metabolic process	BP	0.335	PRCP,PDGFB,CD34,TLR4,NOS3,RAB27A,ARG2,GCH1,NOS2,PMAIP1,RIPK1,HIF1A	up_VCAV_EC
GO:0007098	0.00665	0.998	11	56	centrosome cycle	BP	0.333	HAUS8,NEK2,POC1A,WDR62,NUP62,PLK4,CDK2,SASS6,RBM14,KIF11,GEN1	down_ACOR_VSMC
GO:0010768	0.00665	1	1	1	negative regulation of transcription from RNA polymerase II promoter in response to UV-induced DNA damage	BP	0.718	NEDD4	down_ACOR_EC
GO:0019089	0.00665	1	1	1	transmission of virus	BP	0.718	NEDD4	down_ACOR_EC
GO:0044007	0.00665	1	1	1	dissemination or transmission of symbiont	BP	0.718	NEDD4	down_ACOR_EC
GO:0044111	0.00665	1	1	1	development involved in symbiotic interaction	BP	0.718	NEDD4	down_ACOR_EC
GO:0051821	0.00665	1	1	1	dissemination or transmission of organism from other organism	BP	0.718	NEDD4	down_ACOR_EC
GO:0006355	0.00666	0.999	5	974	regulation of transcription, DNA-	BP	1	TLR2,EYA1,HOXA9,HOXA10,IRX5	up_AFEM_VSMC
GO:0010959	0.00666	0.999	3	79	regulation of metal ion transport	BP	0.718	LYN,HOXA3,NEDD4	down_ACOR_EC
GO:0045764	0.00666	1	1	2	positive regulation of cellular amino acid	BP	1	NR1H4	up_VPUL_EC

GO:0033043	0.00667	0.996	53	446	regulation of organelle organization	BP	0.333	FSCN1,SREBF1,STXBP6,ATAD2,HNRNPD,SMC1A,ZW10,CAPG,TRIP13,SLC25A5,SDC1,FBXO5,PRELID1,BIRC5,INSIG1,LIMA1,NF2,AURKB,STMN1,NEK2,FEN1,RCC1,UHRF1,POC1A,BRCA1,NOC2L,PREB,CDC6,NDC80,RAC1,NUP62,PDXP,PLK4,IQGAP2,CDC42EP4,SMC3,STAG1,ENSCAFG00000005299,EZR,BUB1,RBM14,CORO1A,CDC20,TTK,ARPC1B,ARHGEF2,KIF11,TADA2A,TINF2,UBE2C,CCT4,MTBP,GEN1	down_ACOR_VSMC
GO:0009887	0.00668	0.999	4	314	animal organ morphogenesis	BP	1	HOXA3,NKX2-5,ANKRD6,CYP26B1	up_VPOR_VSMC
GO:0007064	0.00669	0.999	5	15	mitotic sister chromatid cohesion	BP	0.333	SMC1A,ENSCAFG00000017264,SGO1,HASPIN,CDC20	down_ACOR_VSMC
GO:0044093	0.0067	0.996	51	437	positive regulation of molecular function	BP	0.336	TEK,HCLS1,KIT,SELE,PRKCH,MAP3K11,PDGFB,STK10,TMEM173,RELN,MEF2C,RAPGEF1,TLR4,GATA3,EDN1,RALB,NEDD9,PPP3CA,CASP8,MAP3K3,BMP2,SLK,CNOT6,PROX1,MADD,STMN1,HMBOX1,PAK2,PRKD2,SH3GLB1,SMARCA4,TLR3,MFNG,CD200,MMD,RAPGEF2,PLCG2,BCL10,GCH1,HMGB1,TBC1D1,NOS2,PMAIP1,RIPK1,ITGA6,PDGFD,ALS2,ENSCAFG00000002015,RABGAP1,ARHGEF2,USP6NL	up_VCAV_EC
GO:0051928	0.0067	1	2	28	positive regulation of calcium ion transport	BP	0.781	STIM1,CAV1	up_VSAP_VSMC
GO:0008643	0.006709349	0.99883	5	45	carbohydrate transport	BP	0.96436359	NR1H4,AQP1,ENSCAFG00000004331,PLS1,RARRES2	up_ACOR_EC
GO:0001767	0.00672	1	1	2	establishment of lymphocyte polarity	BP	0.727	CYP26B1	up_VFEM_EC
GO:0001768	0.00672	1	1	2	establishment of T cell polarity	BP	0.727	CYP26B1	up_VFEM_EC
GO:0034969	0.00672	1	1	2	histone arginine methylation	BP	1	NR1H4	up_VPUL_EC
GO:0050793	0.00673	0.999	4	731	regulation of developmental process	BP	0.697	HOXA9,WNT7A,ARG2,ESR1	up_AORT_VSMC
GO:0021602	0.00673	1	2	16	cranial nerve morphogenesis	BP	1	HOXA3,SEMA3A	up_VPOR_EC
GO:0097306	0.00673	1	1	22	cellular response to alcohol	BP	1	JUP	down_VFEM_VSMC
GO:0010959	0.00674	0.997	15	79	regulation of metal ion transport	BP	0.898	ACTN2,PLCG2,KCNE3,SARAF,PTPN3,CACNB3,AKAP6,DMPK,PRKCE,CHD7,B2M,FXYD1,INPP5K,WFS1,STC2	up_ACOR_VSMC
GO:0055022	0.00674	1	1	2	negative regulation of cardiac muscle tissue	BP	1	TBX5	up_VPUL_EC
GO:0060044	0.00674	1	1	2	negative regulation of cardiac muscle cell	BP	1	TBX5	up_VPUL_EC
GO:0061117	0.00674	1	1	2	negative regulation of heart growth	BP	1	TBX5	up_VPUL_EC
GO:0035909	0.00675	0.999	5	14	aorta morphogenesis	BP	0.337	ADAMTS9,CHD7,DLL4,PROX1,JAG1	up_VCAV_EC
GO:0042473	0.00675	1	1	5	outer ear morphogenesis	BP	1	EYA1	up_AFEM_VSMC
GO:1903506	0.00676	0.999	5	977	regulation of nucleic acid-templated transcription	BP	1	TLR2,EYA1,HOXA9,HOXA10,IRX5	up_AFEM_VSMC

GO:1901564	0.00677	0.995	144	2053	organonitrogen compound metabolic process	BP	0.411	ARHGEF2,PRICKLE1,VEGFA,P4HB,SULF1,ASNS,SERPINH1,CARS,PLOD2,MYH9,NR1H4,PLK3,HSPA5,SERPINE1,SOCS5,HM13,SGK1,ERO1A,PRKACB,HSP90B1,ENPP1,ERRFI1,ENSCAFG00000005575,P4HA1,JCAD,SNX25,SARS,PGK1,WSB1,BCAR3,NUAK1,PTPN11,AARS,TPI1,ENSCAFG00000032470,ENSCAFG00000028814,TRIB3,PGAM1,PUM3,UGCG,PM20D2,ILK,ALDH1L2,PPP1R12A,PRCP,SESN2,PTGS2,PPIB,CEMIP2,CHSY1,MSN,SEL1L,HIF1A,USP12,MRPL1,IWS1,NMNAT2,DNAJC10,TNIK,GALNT1,NAA10,NXN,UBA6,PTPN2,ALDH18A1,ENTPD4,UCHL5,MORF4L2,SPOPL,YARS,TARS,YES1,PLCL1,TTLL7,NARS,ADAM17,EIF4EBP1,CNOT7,MAGED1,DNAJB9,ALDOC,CTNNB1,PAPSS1,CRYAB,CDK8,APOE,PLOD3,USP48,UBA5,LYN,BNIP3,STRAP,DDIT3,FAM162A,BACH1,ASPH,CCNYL1,STT3A,EIF5,MMD,CBLC,ATF3,GSK3B,VLDLR,AK5,GTF2H1,CUL2,HTT,REST,ZMYND8,MAPK10,EIF2S1,PRMT1,P3H4,EXT1,SPOP,CALM1,RAB11FIP1,DISC1,RAD23A,CDYL,PAK2,A2M,INHBA,CAMK2D,HIF1AN,BTK,MSRB3,CDKN1A,TMTC3,RSL24D1,NRP1,SPINK2,SH3RF3,RHBDF2,USP14,ARF4,BCAT1,TNFAIP1,MADD,RPN1,KDM3A,MID1,CMPK1	down_VSAP_EC
GO:0072615	0.00677	1	1	2	interleukin-17 secretion	BP	1	NR1H4	up_VPUL_EC
GO:0032682	0.00678	1	1	7	negative regulation of chemokine production	BP	0.697	ARG2	up_AORT_VSMC
GO:0010159	0.00678	1	1	2	specification of animal organ position	BP	1	HOXA3	up_VPUL_EC
GO:0035556	0.00679	0.995	103	845	intracellular signal transduction	BP	0.898	FGF13,ADRA1B,PLCG2,LEP,PLCL1,MAP3K8,MID1,ADGRD1,ENSCAFG00000016285,HTR2B,RASSF2,MBP,C28H10orf71,NACC2,PRKAA2,BMP4,FGFR2,ABCA1,DOCK3,ATRX,CASTOR1,TLR3,SH3RF2,LATS2,PDE5A,ARHGAP29,PTGIR,SASH1,GPR155,FGF18,ARHGEF26,NDRG2,CHN2,ADCY6,ITPR1,BLNK,ANKRD1,F2RL1,CAMK2D,PSEN2,DAPK1,BNIP3,AKAP6,CDKN1A,JMY,CRLF3,NRP1,UBR1,PTPN22,DMPK,NPNT,UNC5B,SMPD1,RCAN2,APLP1,SESN3,SH3RF1,PRKCE,FRS2,RND1,DAG1,PARP9,APP,PSEN1,TGFBR1,SYNJ2BP,TESK2,PIK3C3,SIPA1L1,CCL5,WWC2,PERP,PSKH1,DUSP22,SIAH1,EDN1,SESN1,DUSP26,NOD2,LRRC4,DDIT4,DISC1,DEPDC7,RASGRP2,SH3RF3,SERINC3,NCALD,HERPUD1,GNAQ,MAP3K7,INPP5K,BMT2,WFS1,DOCK5,SH3BP4,ENSCAFG00000029553,PARK7,TEAD1,IL18R1,PAG1,CRKL,PIK3C2A,ZMAT3	up_ACOR_VSMC
GO:2000738	0.00679	1	1	9	positive regulation of stem cell differentiation	BP	1	TBX5	up_APUL_VSMC
GO:1990845	0.0068	1	2	51	adaptive thermogenesis	BP	1	HOXC10,GATM	up_VFEM_VSMC
GO:0010988	0.0068	1	1	2	regulation of low-density lipoprotein particle	BP	1	NR1H4	up_VPUL_EC
GO:0003164	0.00681	1	1	2	His-Purkinje system development	BP	1	TBX5	up_VPUL_EC
GO:0003166	0.00681	1	1	2	bundle of His development	BP	1	TBX5	up_VPUL_EC
GO:0002456	0.00682	0.999	6	23	T cell mediated immunity	BP	0.34	IL7R,GATA3,RAB27A,NECTIN2,JAG1,HMGB1	up_VCAV_EC
GO:0009887	0.00682	0.999	4	314	animal organ morphogenesis	BP	1	EYA1,TBX15,HOXA11,SHOX2	up_VFEM_VSMC
GO:2000416	0.00682	1	1	2	regulation of eosinophil migration	BP	1	CCL24	up_VPUL_EC
GO:2001141	0.00683	0.999	5	979	regulation of RNA biosynthetic process	BP	1	TLR2,EYA1,HOXA9,HOXA10,IRX5	up_AFEM_VSMC
GO:0030540	0.00684	1	1	5	female genitalia development	BP	0.699	ESR1	up_AORT_VSMC
GO:0009790	0.00685	0.999	4	335	embryo development	BP	1	HOXC10,HOXA9,HOXC6,DUSP2	up_AORT_EC
GO:1902959	0.00685	1	1	2	regulation of aspartic-type endopeptidase activity involved in amyloid precursor protein	BP	0.718	LYN	down_ACOR_EC

GO:1902961	0.00685	1	1	2	positive regulation of aspartic-type endopeptidase activity involved in amyloid	BP	0.718	LYN	down_ACOR_EC
GO:1905245	0.00685	1	1	2	regulation of aspartic-type peptidase activity	BP	0.718	LYN	down_ACOR_EC
GO:1905247	0.00685	1	1	2	positive regulation of aspartic-type peptidase	BP	0.718	LYN	down_ACOR_EC
GO:1902714	0.00685	1	1	2	negative regulation of interferon-gamma	BP	1	NR1H4	up_VPUL_EC
GO:0060688	0.00686	1	2	29	regulation of morphogenesis of a	BP	0.738	HOXB7,SIX1	up_VFEM_EC
GO:0007010	0.006870002	0.99673	21	441	cytoskeleton organization	BP	0.96436359	MYOZ2,TMOD1,BRSK1,RHOU,ARHGAP6,AQP1,SYNPO2,DIXDC1,PDLIM3,PLS1,ACTN2,ARHGAP28,PAK3,PTGER4,ARHGDIB,ELMO2,DCLK2,CHMP4B,NRP1,PRKCD,TUBB4A	up_ACOR_EC
GO:1905114	0.006879945	0.99777	10	150	cell surface receptor signaling pathway involved in cell-cell	BP	0.96436359	CDH3,SOX9,PORCN,FZD5,DIXDC1,INSYN1,MAPK8IP2,LRP4,CLSTN1,PYGO2	up_ACOR_EC
GO:0060214	0.00688	1	2	2	endocardium formation	BP	0.341	SOX18,PROX1	up_VCAV_EC
GO:0032680	0.00688	1	2	36	regulation of tumor necrosis factor production	BP	1	NR1H4,ARHGEF2	up_VPUL_EC
GO:0003015	0.00689	0.998	12	58	heart process	BP	0.898	TNNT2,ADRA1B,KCNE3,GJA1,PDE5A,DMPK,RNLS,JUP,EDN1,GAA,SLC8A1,BVES	up_ACOR_VSMC
GO:0042307	0.00689	1	1	23	positive regulation of protein import into	BP	1	JUP	down_VFEM_VSMC
GO:0070858	0.00691	1	1	2	negative regulation of bile acid biosynthetic process	BP	1	NR1H4	up_VPUL_EC
GO:1904252	0.00691	1	1	2	negative regulation of bile acid metabolic process	BP	1	NR1H4	up_VPUL_EC
GO:0006888	0.00692	0.998	8	49	ER to Golgi vesicle-mediated transport	BP	0.418	SEC24D,HYOU1,SEC23A,LMAN1,YIPF5,COPB2,CREB3L2,SEC13	down_VSAP_EC
GO:0014066	0.00693	0.998	7	26	regulation of phosphatidylinositol 3-	BP	0.343	TEK,HCLS1,SELP,PDGFB,RELN,PDGFD,RGL2	up_VCAV_EC
GO:0071447	0.00693	1	3	5	cellular response to hydroperoxide	BP	0.898	TP53INP1,DAPK1,OXR1	up_ACOR_VSMC
GO:0006972	0.00693	1	1	2	hyperosmotic response	BP	1	ARHGEF2	up_VPUL_EC
GO:0071474	0.00693	1	1	2	cellular hyperosmotic response	BP	1	ARHGEF2	up_VPUL_EC
GO:0065003	0.00694	0.995	68	616	protein-containing complex assembly	BP	0.342	ACSL3,FSCN1,HMGB1,IRAK1,STXBP6,USP4,HMGCR,EDC3,UBE2S,RRM1,ZW10,CAPG,CENPA,SLC29A1,NDC1,CENPN,C1QTNF6,FBXO5,PSMD13,INSIG1,PRMT1,CAV1,RAD51,TK1,STMN1,CENPO,RCC1,DHX33,GMNN,CENPH,CENPI,FASTKD3,B4GALT1,FARSA,PREB,OIP5,IPO4,HIST1H1A,RAC1,DDX28,FOSL1,IQGAP2,SAMM50,HMGB2,CDC42EP4,HIST1H2BB,DDX39B,SF3A2,CORO1A,CENPW,FOXRED1,SIGMAR1,ARPC1B,CENPP,GRWD1,PRMT5,PSMC1,CTH,ENSCAFG00000015189,HAT1,GEMIN7,UBE2C,MIS18A,TBCC,H3F3B,PDSS1,SF3A1,EIF3M	down_ACOR_VSMC
GO:0042743	0.00694	1	1	5	hydrogen peroxide metabolic process	BP	1	CYP1A1	up_VCAV_VSMC
GO:0043112	0.00695	0.998	12	57	receptor metabolic process	BP	0.898	PLCG2,HTR2B,ATXN2,ZNRF3,LRPAP1,FUT8,PSEN1,RAB11B,EDN1,LMTK2,AP2B1,BVES	up_ACOR_VSMC
GO:0070374	0.00695	0.998	12	57	positive regulation of ERK1 and ERK2 cascade	BP	0.898	HTR2B,BMP4,FGFR2,FGF18,F2RL1,NRP1,PTPN22,NPNT,APP,CCL5,ENSCAFG00000029553,CRKL	up_ACOR_VSMC
GO:0030182	0.00695	0.999	4	327	neuron differentiation	BP	1	HOXC10,EYA1,EMX2,SHOX2	up_VFEM_VSMC
GO:0001659	0.00695	1	2	54	temperature homeostasis	BP	1	HOXC10,LNPEP	up_AORT_EC
GO:0035356	0.00695	1	1	2	cellular triglyceride homeostasis	BP	1	NR1H4	up_VPUL_EC
GO:1904177	0.00695	1	1	2	regulation of adipose tissue development	BP	1	NR1H4	up_VPUL_EC
GO:1904179	0.00695	1	1	2	positive regulation of adipose tissue	BP	1	NR1H4	up_VPUL_EC
GO:0070391	0.00696	1	1	5	response to lipoteichoic acid	BP	1	TLR2	up_AFEM_VSMC
GO:0071223	0.00696	1	1	5	cellular response to lipoteichoic acid	BP	1	TLR2	up_AFEM_VSMC
GO:0031034	0.00696	1	1	2	myosin filament assembly	BP	1	MYBPC2	up_VPUL_EC
GO:0071688	0.00696	1	1	2	striated muscle myosin thick filament assembly	BP	1	MYBPC2	up_VPUL_EC

GO:0021520	0.00698	1	1	3	spinal cord motor neuron cell fate specification	BP	1	HOXC10	up_AORT_EC
GO:0032493	0.00698	1	1	2	response to bacterial lipoprotein	BP	0.781	TLR2	up_VSAP_VSMC
GO:0042494	0.00698	1	1	2	detection of bacterial lipoprotein	BP	0.781	TLR2	up_VSAP_VSMC
GO:0042495	0.00698	1	1	2	detection of triacyl bacterial lipopeptide	BP	0.781	TLR2	up_VSAP_VSMC
GO:0070339	0.00698	1	1	2	response to bacterial lipopeptide	BP	0.781	TLR2	up_VSAP_VSMC
GO:0070340	0.00698	1	1	2	detection of bacterial lipopeptide	BP	0.781	TLR2	up_VSAP_VSMC
GO:0071220	0.00698	1	1	2	cellular response to bacterial lipoprotein	BP	0.781	TLR2	up_VSAP_VSMC
GO:0071221	0.00698	1	1	2	cellular response to bacterial lipopeptide	BP	0.781	TLR2	up_VSAP_VSMC
GO:0071725	0.00698	1	1	2	response to triacyl bacterial lipopeptide	BP	0.781	TLR2	up_VSAP_VSMC
GO:0071727	0.00698	1	1	2	cellular response to triacyl bacterial lipopeptide	BP	0.781	TLR2	up_VSAP_VSMC
GO:0098722	0.00701	1	1	2	asymmetric stem cell division	BP	1	ARHGEF2	up_VPUL_EC
GO:0002474	0.00702	0.999	4	9	antigen processing and presentation of peptide antigen via MHC class I	BP	0.899	DLA88,B2M,ERAP1,TAPBPL	up_ACOR_VSMC
GO:0061061	0.00702	0.999	4	179	muscle structure development	BP	0.781	PITX1,STIM1,CAV1,SHOX2	up_VSAP_VSMC
GO:0050718	0.007027832	0.99981	2	5	positive regulation of interleukin-1 beta	BP	0.96436359	FZD5,NOD2	up_ACOR_EC
GO:0007144	0.00703	1	2	2	female meiosis I	BP	0.345	TRIP13,FBXO5	down_ACOR_VSMC
GO:0014826	0.00704	1	2	2	vein smooth muscle contraction	BP	0.347	EDNRB,EDN1	up_VCAV_EC
GO:0001932	0.00706	0.996	38	416	regulation of protein phosphorylation	BP	0.425	ARHGEF2,VEGFA,SOCS5,ENPP1,ERRFI1,JCAD,SNX25,BCAR3,PTPN11,TRIB3,ILK,SESN2,PTGS2,DNAJC10,TNIK,PTPN2,YES1,PLCL1,ADAM17,CNOT7,MAGED1,CTNNB1,APOE,LYN,STRAP,CCNYL1,MMD,CBLC,ATF3,VLDLR,HTT,PAK2,INHBA,CDKN1A,NRP1,SH3RF3,MADD,MID1	down_VSAP_EC
GO:2000482	0.00706	0.999	4	9	regulation of interleukin-8 secretion	BP	1	C5AR2,ANXA4,F2RL1,MAPKBP1	up_VSAP_EC
GO:0048856	0.00707	0.998	12	1533	anatomical structure development	BP	1	NR1H4,TBX5,HOXA2,MAPK10,HOXA3,ARHGEF26,ZDHHC16,SLITRK5,NPNT,NTRK2,HOXA1,PDLIM3	up_APUL_EC
GO:0043309	0.00707	1	1	2	regulation of eosinophil degranulation	BP	0.722	STX4	down_ACOR_EC
GO:0043311	0.00707	1	1	2	positive regulation of eosinophil degranulation	BP	0.722	STX4	down_ACOR_EC
GO:1902566	0.00707	1	1	2	regulation of eosinophil activation	BP	0.722	STX4	down_ACOR_EC
GO:1902568	0.00707	1	1	2	positive regulation of eosinophil activation	BP	0.722	STX4	down_ACOR_EC
GO:0051241	0.00708	0.999	4	336	negative regulation of multicellular organismal	BP	1	HOXC10,HOXA9,LNPEP,BPI	up_AORT_EC
GO:0019433	0.00708	1	2	2	triglyceride catabolic process	BP	0.346	ABHD5,LIPC	down_ACOR_VSMC
GO:0003264	0.00708	1	1	5	regulation of cardioblast proliferation	BP	1	EYA1	up_AFEM_VSMC
GO:0003266	0.00708	1	1	5	regulation of secondary heart field cardioblast	BP	1	EYA1	up_AFEM_VSMC
GO:0048665	0.0071	1	1	6	neuron fate specification	BP	0.721	HOXC10	up_AORT_VSMC
GO:0032230	0.0071	1	1	5	positive regulation of synaptic transmission,	BP	1	CA2	up_VCAV_VSMC
GO:1900122	0.0071	1	1	3	positive regulation of receptor binding	BP	1	HOXA3	up_VPOR_VSMC
GO:0055059	0.00711	1	1	1	asymmetric neuroblast division	BP	1	ARHGEF2	up_VPOR_EC
GO:0050715	0.007112369	0.99906	4	29	positive regulation of cytokine secretion	BP	0.96436359	FZD5,NOD2,PTGER4,MAVS	up_ACOR_EC
GO:0030855	0.00712	0.999	4	183	epithelial cell differentiation	BP	0.761	HOXA7,SIX1,CYP26B1,MTSS1	up_VFEM_EC
GO:1901653	0.007120284	0.99823	7	84	cellular response to peptide	BP	0.96436359	NR1H4,ENSCAFG00000004331,NOD2,ACTN2,FOXC2,RARRES2,PRKCD	up_ACOR_EC
GO:0098773	0.007133535	0.99906	4	29	skin epidermis development	BP	0.96436359	CDH3,SOX9,TNFRSF19,LRP4	up_ACOR_EC
GO:0071772	0.00715	1	1	50	response to BMP	BP	1	GATA5	down_AFEM_VSMC

GO:0071773	0.00715	1	1	50	cellular response to BMP stimulus	BP	1	GATA5	down_AFEM_VSMC
GO:0007501	0.00715	1	1	5	mesodermal cell fate specification	BP	1	EYA1	up_AFEM_VSMC
GO:0010614	0.00715	1	1	5	negative regulation of cardiac muscle	BP	1	GATA5	up_VCAV_VSMC
GO:0014741	0.00715	1	1	5	negative regulation of muscle hypertrophy	BP	1	GATA5	up_VCAV_VSMC
GO:0032635	0.00716	1	2	37	interleukin-6 production	BP	1	NR1H4,ARHGEF2	up_VPUL_EC
GO:2001245	0.00716	1	1	2	regulation of phosphatidylcholine	BP	0.725	ACSL3	down_ACOR_EC
GO:0003278	0.00718	1	1	3	apoptotic process involved in heart	BP	1	NKX2-5	up_VPOR_VSMC
GO:1904469	0.007188837	0.9998	2	5	positive regulation of tumor necrosis factor	BP	0.96436359	FZD5,MAVS	up_ACOR_EC
GO:0007252	0.00719	1	1	6	I-kappaB phosphorylation	BP	1	TLR2	up_AFEM_VSMC
GO:0006464	0.007194768	0.99574	44	1162	cellular protein modification process	BP	0.96436359	NR1H4,BRSK1,PLCL1,PKIB,SOX9,GALNT5,PHF20,NDRG2,PORCN,ENSCAFG00000004331,EDEM2,PI3,FZD5,DUSP26,NOD2,SMAD6,B3GNT7,KLHL25,ZMYND8,PAK3,GRK7,TNFRSF19,PIAS1,DCLK2,USP53,MAPK8IP2,MAVS,LRRC15,KLHDC8B,PIGS,LRP4,SH3RF3,LRRC4,RARRES2,AGBL5,RIOX2,RNF114,UBE2H,STK32C,NRP1,PRKCD,PYGO2,DBNDD2,CCNYL1	up_ACOR_EC
GO:0036211	0.007194768	0.99574	44	1162	protein modification process	BP	0.96436359	NR1H4,BRSK1,PLCL1,PKIB,SOX9,GALNT5,PHF20,NDRG2,PORCN,ENSCAFG00000004331,EDEM2,PI3,FZD5,DUSP26,NOD2,SMAD6,B3GNT7,KLHL25,ZMYND8,PAK3,GRK7,TNFRSF19,PIAS1,DCLK2,USP53,MAPK8IP2,MAVS,LRRC15,KLHDC8B,PIGS,LRP4,SH3RF3,LRRC4,RARRES2,AGBL5,RIOX2,RNF114,UBE2H,STK32C,NRP1,PRKCD,PYGO2,DBNDD2,CCNYL1	up_ACOR_EC
GO:0006775	0.0072	1	1	3	fat-soluble vitamin metabolic process	BP	1	CYP26B1	up_VPOR_VSMC
GO:1900076	0.007204173	0.99905	4	29	regulation of cellular response to insulin	BP	0.96436359	NR1H4,ENSCAFG00000004331,RARRES2,PRKCD	up_ACOR_EC
GO:0001508	0.00722	1	1	25	action potential	BP	1	JUP	down_VFEM_VSMC
GO:1903555	0.00723	1	2	37	regulation of tumor necrosis factor superfamily cytokine	BP	1	NR1H4,ARHGEF2	up_VPUL_EC
GO:0003170	0.00723	1	1	25	heart valve development	BP	1	GATA5	down_VFEM_VSMC
GO:0044242	0.00724	0.997	12	57	cellular lipid catabolic process	BP	1	PEX7,LIPC,ENPP2,ACAA1,ACACB,PEX2,FAAH,GCDH,EHHADH,LONP2,ABHD16A,IDH1	up_VSAP_EC
GO:0003008	0.00724	0.999	4	336	system process	BP	1	HOXC10,LNPEP,SGCD,GPR155	up_AORT_EC
GO:0007289	0.00725	1	3	5	spermatid nucleus differentiation	BP	0.354	PSME4,NECTIN2,KDM3A	up_VCAV_EC
GO:0099174	0.00726	1	1	6	regulation of presynapse organization	BP	0.727	WNT7A	up_AORT_VSMC
GO:0006924	0.00727	1	2	3	activation-induced cell death of T cells	BP	1	GPAM,TSC22D3	down_VCAV_EC
GO:0014823	0.00728	1	3	5	response to activity	BP	0.907	FNDC5,PRKAA2,PPARGC1A	up_ACOR_VSMC
GO:0032660	0.00728	1	1	7	regulation of interleukin-17 production	BP	0.727	ARG2	up_AORT_VSMC
GO:0043666	0.00729	0.999	4	14	regulation of phosphoprotein	BP	0.436	NUAK1,PPP1R12A,HTT,CALM1	down_VSAP_EC
GO:0007166	0.007290762	0.99611	29	686	cell surface receptor signaling pathway	BP	0.96436359	SH3TC2,CDH3,NR1H4,SOX9,PTH1R,PORCN,ENSCAFG00000004331,ADAMTS13,FZD5,CCDC3,IFI6,DIXDC1,PTGIR,RGMA,INSYN1,SMAD6,ESM1,ADAMTSL2,MAPK8IP2,MAVS,FOXC2,LRRC15,LRP4,CLSTN1,LRRC4,NRP1,PRKCD,PYGO2,ADGRL1	up_ACOR_EC

GO:0010033	0.0073	0.995	82	772	response to organic substance	BP	0.355	ACKR1,HCLS1,GBP5,KIT,SELE,PRCP,CXCL8,LAPTM5,CXCR4,CALCRL,TIE1,TNFAIP3,PPARG,TNIP1,MX2,PDGFB,P2RX7,BAIAP2L1,TMEM173,SELL,PDE4D,MEF2C,FERMT1,ATF3,TIPARP,DLL4,RAPGEF1,TLR4,NLRC5,GATA3,RALB,SMAD1,TFPI,IRF8,PELI1,YES1,CASP8,MTOR,BMP2,HHEX,ST3GAL6,ABCA1,MAPK13,IFNGR1,RIOK3,ENSCAFG00000010596,FLNB,PAK2,PRKD2,SMARCA4,TLR3,NCK1,TIMELESS,OTUD4,JAG1,RAPGEF2,PLCG2,CTDSPL2,CREB1,BCL10,CTNNA1,CNOT7,GCH1,ADIPOR2,PMAIP1,BLM,RIPK1,LSM14A,USP13,SKIL,PDGFD,UFSP2,HIF1A,KDM3A,ATRX,NR4A1,ENSCAFG00000002015,P2RX2,JCAD,ZEB1,ARHGEF2,TJP2	up_VCAV_EC
GO:0048645	0.0073	1	2	33	animal organ formation	BP	1	TBX5,HOXA3	up_APUL_EC
GO:0006942	0.0073	1	1	26	regulation of striated muscle contraction	BP	1	JUP	down_VFEM_VSMC
GO:0120163	0.00732	1	1	17	negative regulation of cold-induced	BP	1	PLCL1	down_VFEM_EC
GO:0060045	0.00733	1	1	11	positive regulation of cardiac muscle cell	BP	1	TBX5	up_APUL_VSMC
GO:1900424	0.00734	1	3	5	regulation of defense response to bacterium	BP	1	F2RL1,MAPKBP1,CYBA	up_VSAP_EC
GO:0034765	0.00734	0.999	3	91	regulation of ion transmembrane transport	BP	0.811	NETO1,STIM1,CAV1	up_VSAP_VSMC
GO:0030308	0.00735	0.999	3	49	negative regulation of cell growth	BP	1	SEMA3A,FBP1,RNF6	up_VPOR_EC
GO:0046633	0.00735	1	1	7	alpha-beta T cell proliferation	BP	0.727	ARG2	up_AORT_VSMC
GO:0021603	0.00735	1	1	2	cranial nerve formation	BP	1	HOXA1	up_APUL_EC
GO:0030326	0.00736	0.998	11	51	embryonic limb morphogenesis	BP	1	HOXA11,HOXA9,OSR2,OSR1,ZBTB16,RARG,RECK,FBXW4,SHOX2,HOXC10,LRP4	up_VSAP_EC
GO:0035113	0.00736	0.998	11	51	embryonic appendage morphogenesis	BP	1	HOXA11,HOXA9,OSR2,OSR1,ZBTB16,RARG,RECK,FBXW4,SHOX2,HOXC10,LRP4	up_VSAP_EC
GO:0007163	0.00736	1	2	60	establishment or maintenance of cell	BP	1	GPSM2,FSCN1	down_AFEM_EC
GO:0048387	0.00736	1	1	3	negative regulation of retinoic acid receptor	BP	1	CYP26B1	up_VPOR_VSMC
GO:0090049	0.00737	0.999	5	21	regulation of cell migration involved in sprouting angiogenesis	BP	0.438	VEGFA,JCAD,PIK3C2A,STARD13,NRP1	down_VSAP_EC
GO:0009952	0.00737	1	2	66	anterior/posterior pattern specification	BP	1	HOXA3,HOXB5	up_VPUL_VSMC
GO:0070662	0.00737	1	1	2	mast cell proliferation	BP	0.725	LYN	down_ACOR_EC
GO:0070666	0.00737	1	1	2	regulation of mast cell proliferation	BP	0.725	LYN	down_ACOR_EC
GO:0070668	0.00737	1	1	2	positive regulation of mast cell proliferation	BP	0.725	LYN	down_ACOR_EC
GO:0003350	0.00737	1	1	3	pulmonary myocardium development	BP	1	NKX2-5	up_VPOR_VSMC
GO:0097327	0.00738	1	1	25	response to antineoplastic agent	BP	1	JUP	down_VFEM_VSMC
GO:0072171	0.00739	0.998	6	30	mesonephric tubule morphogenesis	BP	0.438	ENSCAFG00000032470,ILK,HOXB7,MAGED1,CTNNB1,SIX4	down_VSAP_EC
GO:0003416	0.00739	0.999	3	9	endochondral bone growth	BP	1	THBS3,EVC,DDR2	down_VCAV_EC
GO:0006796	0.00739384	0.99578	37	938	phosphate-containing compound metabolic process	BP	0.96436359	NR1H4,BRSK1,PLCL1,PKIB,THNSL2,SOX9,NMNAT2,NDRG2,ENSCAFG00000004331,FZD5,DUSP26,NOD2,SMAD6,PAK3,CFI,GUCY1B1,GRK7,TNFRSF19,DCLK2,CHPT1,MAPK8IP2,MAVS,LRRC15,PIGS,LRP4,SH3RF3,AK7,LRRC4,ALDOC,RARRES2,STK32C,NRP1,AGPAT2,PRKCD,PPP1R26,DBNDD2,CCNYL1	up_ACOR_EC
GO:0061156	0.0074	1	1	4	pulmonary artery morphogenesis	BP	1	CITED2	down_VPOR_EC
GO:0006635	0.00741	0.999	5	25	fatty acid beta-oxidation	BP	1	AKT2,ACAA1,PEX5,LONP2,ACAT1	down_VCAV_EC
GO:0060670	0.00741	1	3	5	branching involved in labyrinthine layer	BP	0.907	FGFR2,SPINT1,SPINT2	up_ACOR_VSMC
GO:0000027	0.00741	1	2	16	ribosomal large subunit assembly	BP	1	RSL24D1,RPL10L	up_VPOR_EC
GO:0048702	0.00741	1	1	2	embryonic neurocranium morphogenesis	BP	0.725	HOXA1	down_ACOR_EC
GO:0021520	0.00741	1	1	3	spinal cord motor neuron cell fate specification	BP	1	HOXC10	up_VFEM_VSMC

GO:0071345	0.00742	0.996	22	210	cellular response to cytokine stimulus	BP	0.438	ARHGEF2,NR1H4,HSPA5,SOCS5,FLNB,DPYSL3,ILK,MYO1C,KIF5B,HIF1A,CX3CL1,PTPN2,ADAM17,CNOT7,ENSCAFG00000002015,TFRC,ZC3H15,KLF5,CDC37,GBP5,BTK,KDM3A	down_VSAP_EC
GO:0045595	0.00742	0.997	20	469	regulation of cell differentiation	BP	1	HOXA9,CMKLR1,IRX3,LEP,LBH,HOXA7,DLL1,PLXNA4,TWIST2,THRB,TMEM100,GREM1,CAMK2A,CYLD,PTHLH,EVI2B,AXIN2,NAPEPLD,DMPK,KANK1	up_AFEM_EC
GO:0019438	0.00743	0.998	8	1258	aromatic compound biosynthetic process	BP	1	EYA1,HOXA10,HOXA9,TBX15,FBP1,EMX2,HOXA11,SHOX2	up_VFEM_VSMC
GO:0050701	0.00743	0.999	5	16	interleukin-1 secretion	BP	0.36	GBP5,TLR4,ARG2,ABCA1,HMGB1	up_VCAV_EC
GO:0007187	0.00744	0.998	10	43	G protein-coupled receptor signaling pathway, coupled to cyclic nucleotide second	BP	0.907	ADRA1B,ADGRD1,ABCA1,PTGIR,ADCY6,GNA14,APLP1,SENP7,EDN1,GNAQ	up_ACOR_VSMC
GO:0030705	0.00744	0.998	9	56	cytoskeleton-dependent intracellular transport	BP	0.438	HOOK3,MYO1C,RPGR,KIF5B,HIF1A,DYNLL1,TRAK1,HTT,AP3S1	down_VSAP_EC
GO:0042110	0.00745	0.997	17	109	T cell activation	BP	0.36	IL7R,KIT,IL6,CHD7,DLL4,GATA3,RAB27A,PELI1,ARG2,MTOR,ELF4,ITPKB,NCK1,BCL10,HMGB1,BMX,ZEB1	up_VCAV_EC
GO:0045619	0.00745	0.998	10	44	regulation of lymphocyte differentiation	BP	1	ABL1,ZBTB16,TOX,ZFP36L1,ZEB1,FBXO7,CD83,ZFP36L2,PNP,TGFBR2	up_VSAP_EC
GO:1904591	0.00745	1	1	25	positive regulation of protein import	BP	1	JUP	down_VFEM_VSMC
GO:0060872	0.00745	1	1	6	semicircular canal development	BP	1	EYA1	up_AFEM_VSMC
GO:0001732	0.00746	0.999	4	9	formation of cytoplasmic translation initiation	BP	1	EIF3G,EIF3K,EIF3L,EIF3D	up_VSAP_EC
GO:0050877	0.00746	0.999	3	164	nervous system process	BP	1	HOXC10,GATM,NETO1	up_VFEM_VSMC
GO:0048598	0.00746	0.999	3	207	embryonic morphogenesis	BP	1	HOXA3,TBX5,HOXB5	up_VPUL_VSMC
GO:0022603	0.00747	0.999	3	301	regulation of anatomical structure morphogenesis	BP	1	LIMK1,GATA5,C6	up_VCAV_VSMC
GO:0090257	0.00747	1	1	52	regulation of muscle system process	BP	1	GATA5	down_AFEM_VSMC
GO:0071495	0.00748	0.999	5	365	cellular response to endogenous stimulus	BP	1	NR1H4,PTGER2,FLRT3,ARHGEF2,NEO1	up_VPUL_EC
GO:0051891	0.00748	1	1	3	positive regulation of cardioblast differentiation	BP	1	NKX2-5	up_VPOR_VSMC
GO:0032886	0.00749	0.997	14	81	regulation of microtubule-based process	BP	0.363	SMC1A,CFAP298,STMN1,NEK2,POC1A,RAC1,NUP62,PLK4,SMC3,STAG1,RBM14,ARHGEF2,KIF11,GEN1	down_ACOR_VSMC
GO:0045926	0.00749	0.997	14	72	negative regulation of growth	BP	0.907	DCBLD2,BMP4,GJA1,RERG,PLAC8B,CDKN1A,CRLF3,NRP1,ULK2,TBX5,WWC2,NAIF1,SH3BP4,STC2	up_ACOR_VSMC
GO:0071600	0.00749	1	1	5	otic vesicle morphogenesis	BP	1	EYA1	up_AFEM_VSMC
GO:0030177	0.00751	1	2	51	positive regulation of Wnt signaling pathway	BP	1	BAMBI,ANKRD6	up_VPOR_VSMC
GO:0120162	0.00751	1	2	31	positive regulation of cold-induced thermogenesis	BP	0.824	GATM,CAV1	up_VSAP_VSMC
GO:0060914	0.00752	1	1	11	heart formation	BP	1	TBX5	up_APUL_VSMC
GO:0048732	0.00754	1	2	116	gland development	BP	0.739	HOXA9,ESR1	up_AORT_VSMC
GO:0009595	0.00754	1	1	6	detection of biotic stimulus	BP	1	TLR2	up_AFEM_VSMC
GO:0002521	0.00755	0.996	22	149	leukocyte differentiation	BP	0.362	HCLS1,IL7R,KIT,IL6,MEF2C,CHD7,DLL4,GATA3,CASP8,MTOR,HHEX,ITPKB,MFNG,PLCG2,CREB1,HMGB1,RIPK1,ATP11C,CUL4A,BMX,ZEB1,TJP2	up_VCAV_EC
GO:0070507	0.00756	0.997	13	73	regulation of microtubule cytoskeleton organization	BP	0.364	SMC1A,STMN1,NEK2,POC1A,RAC1,NUP62,PLK4,SMC3,STAG1,RBM14,ARHGEF2,KIF11,GEN1	down_ACOR_VSMC
GO:0034308	0.00756	0.999	4	16	primary alcohol metabolic process	BP	1	CYP1B1,RETSAT,PARK7,NAPEPLD	down_VCAV_EC
GO:0070050	0.00756	1	1	3	neuron cellular homeostasis	BP	1	TAOK1	up_AORT_EC
GO:2000463	0.00756	1	1	5	positive regulation of excitatory postsynaptic	BP	0.739	WNT7A	up_AORT_VSMC

GO:0051240	0.00758	0.995	63	489	positive regulation of multicellular organismal process	BP	1	HOXA11,CDON,WNT11,CD274,AXIN2,WASF3,OSR2,AMIGO1,ENPP2,LRRN3,PPARGC1B,ACACB,IL6R,ZHX3,HOXD3,FGF7,ABL1,EBF2,BTG1,HDAC7,DLL1,OSR1,IL17RC,HPSE,ANKRD54,ZBTB16,TOX,CCBE1,STAT5A,STAT6,F2RL1,DDR2,MFN2,ZFP36L1,PIK3R1,IRX3,ZEB1,TBXA2R,SULF2,SHOX2,SASH1,MYD88,GDPD5,PRKAB1,NLRX1,TKFC,CD83,KDM2B,PNP,ZNF580,CYBA,CYB5D2,PQBP1,ACVRL1,FLOT1,NCK1,MAVS,HMOX1,MAP2K2,TGFBR2,CITED2,TRADD,LBH	up_VSAP_EC
GO:0060876	0.00758	1	1	2	semicircular canal formation	BP	0.725	HOXA1	down_ACOR_EC
GO:0032924	0.00758	1	1	9	activin receptor signaling pathway	BP	1	TGFBR1	down_VSAP_VSMC
GO:0051049	0.00759	0.995	62	479	regulation of transport	BP	0.907	EXPH5,SH3TC2,TLR1,DNAJC6,ACTN2,PLCG2,LEP,KCNE3,HTR2B,TCAF1,USP6NL,MBP,FBXL20,FERMT1,BMP4,EVI5L,TM7SF3,ABCA1,GJA1,SEC24B,ATXN2,CD274,SARAF,PTPN3,ANKRD1,F2RL1,DAPK1,CACNB3,AKAP6,CDKN1A,PTPN22,LRPAP1,DMPK,OS9,SCP2,KCNJ4,PRKCE,SCG5,PSEN1,SGSM2,SYNJ2BP,CHD7,JUP,CCL5,RAB11B,EDN1,GTF2A1L,KCNC4,NOD2,B2M,FXYD1,SCAMP5,INPP5K,RAB5C,PREPL,PFKL,WFS1,AZIN2,PARK7,STC2,BVES,PTPN14	up_ACOR_VSMC
GO:0018130	0.00759	0.998	8	1261	heterocycle biosynthetic process	BP	1	EYA1,HOXA10,HOXA9,TBX15,FBP1,EMX2,HOXA11,SHOX2	up_VFEM_VSMC
GO:0009063	0.00759	0.999	5	25	cellular amino acid catabolic process	BP	1	THNSL2,ENSCAFG00000002977,GLS2,BCKDHB,ACAT1	down_VCAV_EC
GO:0002576	0.00761	1	1	2	platelet degranulation	BP	0.725	LYN	down_ACOR_EC
GO:0031326	0.00762	0.998	11	1176	regulation of cellular biosynthetic process	BP	0.825	HOXA11,HOXA10,HOXA9,PITX1,EMX2,SALL1,IRX5,PRR16,TLR2,CAV1,SHOX2	up_VSAP_VSMC
GO:0001659	0.00762	1	2	54	temperature homeostasis	BP	1	HOXC10,GATM	up_VFEM_VSMC
GO:0003176	0.00762	1	1	9	aortic valve development	BP	1	GATA5	down_VSAP_VSMC
GO:0031572	0.00764	0.999	5	15	G2 DNA damage checkpoint	BP	0.366	CDK1,FANCI,TOPBP1,DTL,CLSPN	down_ACOR_VSMC
GO:0035239	0.00764	0.999	5	299	tube morphogenesis	BP	0.795	EYA1,HOXB7,HOXA7,SIX1,MTSS1	up_VFEM_EC
GO:0060158	0.00764	1	2	2	phospholipase C-activating dopamine receptor signaling	BP	0.907	GNA14,GNAQ	up_ACOR_VSMC
GO:0042483	0.00764	1	1	2	negative regulation of odontogenesis	BP	0.725	ASPN	down_ACOR_EC
GO:0030888	0.00765	0.999	4	13	regulation of B cell proliferation	BP	0.446	TFRC,LYN,BTK,CDKN1A	down_VSAP_EC
GO:0070925	0.00766	0.996	37	293	organelle assembly	BP	0.366	FSCN1,EDC3,SMC1A,CENPA,CENPN,SDC1,FBXO5,CFAP298,BIRC5,AURKB,HAUS8,NEK2,RCC1,CENPH,POC1A,WDR62,NUP62,DDX28,PLK4,SMC3,CDK2,STAG1,ENSCAFG00000005299,EZR,SASS6,RBM14,CORO1A,CENPW,CDC20,ASPM,TRAPPC6A,ENSCAFG00000015189,KIF11,SIX4,CEP83,TTLL1,KIF24	down_ACOR_VSMC
GO:0050865	0.00766	0.996	20	135	regulation of cell activation	BP	0.366	IL7R,ADGRF5,SELP,TNFAIP3,PDGFB,MEF2C,TLR4,GATA3,PELI1,ARG2,NECTIN2,ITPKB,THOC1,NCK1,CD200,FES,BCL10,HMGB1,ATP11C,ZEB1	up_VCAV_EC
GO:1902105	0.00766	0.997	15	80	regulation of leukocyte differentiation	BP	1	HOXA7,PPARGC1B,ABL1,ZBTB16,TOX,ZFP36L1,PIK3R1,MYC,ZEB1,TFE3,FBXO7,CD83,ZFP36L2,PNP,TGFBR2	up_VSAP_EC
GO:0006635	0.00766	0.998	7	25	fatty acid beta-oxidation	BP	1	PEX7,ACAA1,ACACB,PEX2,GCDH,EHHADH,LONP2	up_VSAP_EC
GO:0048585	0.007670502	0.99625	22	475	negative regulation of response to stimulus	BP	0.96436359	NR1H4,SOX9,FNDC4,NDRG2,CCDC3,IFI6,DUSP26,PTGIR,RGMA,SMAD6,MMP28,PTGER4,ADAMTSL2,MAPK8IP2,FIGNL1,LRRC15,LRP4,CLSTN1,LRRC4,CLU,NRP1,PRKCD	up_ACOR_EC
GO:0009135	0.00769	0.999	5	22	purine nucleoside diphosphate metabolic	BP	0.446	PGK1,TPI1,PGAM1,HIF1A,ALDOC	down_VSAP_EC
GO:0009179	0.00769	0.999	5	22	purine ribonucleoside diphosphate metabolic	BP	0.446	PGK1,TPI1,PGAM1,HIF1A,ALDOC	down_VSAP_EC
GO:0046031	0.00769	0.999	5	22	ADP metabolic process	BP	0.446	PGK1,TPI1,PGAM1,HIF1A,ALDOC	down_VSAP_EC
GO:0060575	0.00769	1	1	9	intestinal epithelial cell differentiation	BP	1	GATA5	down_VSAP_VSMC

GO:0002790	0.0077	0.996	22	133	peptide secretion	BP	0.907	EXPH5,TLR1,LEP,HTR2B,MBP,FERMT1,TM7SF3,ABCA1,CD274,ANKRD1,F2RL1,PTPN22,PRKCE,CHD7,CCL5,RAB11B,EDN1,NOD2,SCAMP5,PFKL,PARK7,LMF1	up_ACOR_VSMC
GO:0003171	0.0077	1	1	10	atrioventricular valve development	BP	1	TBX5	up_APUL_VSMC
GO:0032675	0.00771	1	2	34	regulation of interleukin-6 production	BP	1	NR1H4,BPI	up_APUL_EC
GO:1903045	0.00771	1	1	1	neural crest cell migration involved in sympathetic nervous system	BP	1	SEMA3A	up_VPOR_EC
GO:0003197	0.007713142	0.99935	3	16	endocardial cushion development	BP	0.96436359	GATA4,SOX9,TBX5	up_ACOR_EC
GO:2000117	0.00773	1	1	27	negative regulation of cysteine-type	BP	1	CST3	down_AORT_EC
GO:2000406	0.00774	1	2	6	positive regulation of T cell migration	BP	1	CCL5,ADAM17	up_AFEM_EC
GO:1904888	0.00774	1	2	31	cranial skeletal system development	BP	0.795	TBX15,SIX1	up_VFEM_EC
GO:0060969	0.00775	1	1	6	negative regulation of gene silencing	BP	0.744	ESR1	up_AORT_VSMC
GO:0050794	0.00776	0.997	23	3075	regulation of cellular process	BP	0.725	ACVRL1,YEATS2,LYN,HOXA3,IGF1R,PDPN,NRP2,YBX3,HIVEP2,HOXA7,PRKAR2A,USP28,IPO7,ATRIP,SULF1,ELK3,HOXA1,ACSL3,WDR12,STX4,MYO1D,ASPN,NEDD4	down_ACOR_EC
GO:0048729	0.00776	0.999	3	209	tissue morphogenesis	BP	1	TBX5,GATA5,SLIT2	up_VPUL_VSMC
GO:1905941	0.00776	1	1	4	positive regulation of gonad development	BP	1	CITED2	down_VPOR_EC
GO:2000018	0.00776	1	1	4	regulation of male gonad development	BP	1	CITED2	down_VPOR_EC
GO:2000020	0.00776	1	1	4	positive regulation of male gonad development	BP	1	CITED2	down_VPOR_EC
GO:0071468	0.00777	1	1	4	cellular response to acidic pH	BP	1	RAB11B	down_VPOR_EC
GO:0019276	0.00779	1	1	2	UDP-N-acetylgalactosamine	BP	0.725	CSGALNACT1	down_ACOR_EC
GO:0016310	0.0078	0.995	54	641	phosphorylation	BP	0.446	ARHGEF2,VEGFA,SOCS5,SGK1,ENPP1,ERRFI1,JCAD,SNX25,PGK1,BCAR3,NUAK1,PTPN11,TPI1,TRIB3,PGAM1,ILK,SESN2,PTGS2,HIF1A,DYNLL1,DNAJC10,TNIK,PTPN2,YES1,PLCL1,ADAM17,CNOT7,MAGED1,ALDOC,CTNNB1,CDK8,APOE,LYN,STRAP,CCNYL1,MMD,CBLC,ATF3,GSK3B,VLDLR,GTF2H1,SH3GLB1,HTT,MAPK10,EIF2S1,PAK2,INHBA,CAMK2D,BTK,CDKN1A,NRP1,SH3RF3,MADD,MID1	down_VSAP_EC
GO:0048147	0.0078	1	3	5	negative regulation of fibroblast proliferation	BP	1	PEX2,MORC3,FBXO4	up_VSAP_EC
GO:0030168	0.0078	1	2	23	platelet activation	BP	0.725	LYN,PDPN	down_ACOR_EC
GO:0060039	0.0078	1	1	11	pericardium development	BP	1	TBX5	up_APUL_VSMC
GO:0032872	0.00781	0.997	15	77	regulation of stress-activated MAPK cascade	BP	0.907	LEP,MID1,RASSF2,TLR3,SH3RF2,SASH1,F2RL1,PTPN22,SH3RF1,APP,DUSP22,EDN1,NOD2,SH3RF3,MAP3K7	up_ACOR_VSMC
GO:0030199	0.00781	0.999	5	21	collagen fibril organization	BP	0.446	SERPINH1,P4HA1,PLOD3,P3H4,COL12A1	down_VSAP_EC
GO:0048731	0.00782	0.999	5	1247	system development	BP	0.746	HOXC10,HOXA9,WNT7A,ARG2,ESR1	up_AORT_VSMC
GO:0030510	0.00782	1	2	27	regulation of BMP signaling pathway	BP	0.725	ACVRL1,SULF1	down_ACOR_EC
GO:0018197	0.00783	1	2	3	peptidyl-aspartic acid modification	BP	0.446	ASPH,HIF1AN	down_VSAP_EC
GO:0042264	0.00783	1	2	3	peptidyl-aspartic acid hydroxylation	BP	0.446	ASPH,HIF1AN	down_VSAP_EC
GO:0072513	0.00783	1	1	3	positive regulation of secondary heart field cardioblast proliferation	BP	1	EYA1	up_VFEM_VSMC
GO:0021615	0.00784	1	1	3	glossopharyngeal nerve morphogenesis	BP	1	HOXA3	up_VPOR_VSMC
GO:0032757	0.00785	0.999	5	14	positive regulation of interleukin-8 production	BP	0.907	TLR1,TLR3,F2RL1,NOD2,PARK7	up_ACOR_VSMC
GO:2001013	0.00785	1	1	2	epithelial cell proliferation involved in renal tubule morphogenesis	BP	0.795	MTSS1	up_VFEM_EC

GO:0032989	0.00786	0.996	29	292	cellular component morphogenesis	BP	0.446	ARHGEF2,VEGFA,SIPA1L1,MYH9,FMNL3,ACTN1,SPARC,SGK1,AKAP2,PTPN11,FLNB,IFRD1,ILK,WDR1,MSN,KIF5B,TNIK,NEO1,UST,CDH11,CTNNB1,ENSCAFG00000002015,PLOD3,VLDLR,EXT1,SIX4,NRP1,YWHAH,MYBPC2	down_VSAP_EC
GO:0048665	0.00786	1	2	6	neuron fate specification	BP	1	HOXC10,DLL1	up_AFEM_EC
GO:0006044	0.00787	1	3	5	N-acetylglucosamine metabolic process	BP	1	RENBP,CHST7,GNPDA1	up_VSAP_EC
GO:0019098	0.00787	1	2	6	reproductive behavior	BP	1	THRB,AVPR1A	up_AFEM_EC
GO:1901623	0.00787	1	2	6	regulation of lymphocyte chemotaxis	BP	1	CCL5,ADAM17	up_AFEM_EC
GO:0051090	0.00788	0.996	20	134	regulation of DNA-binding transcription factor activity	BP	0.375	HCLS1,KIT,PRKCH,TNFAIP3,TMEM173,RELN,NLRC5,EDN1,PELI1,PPP3CA,PROX1,ENSCAFG00000010596,PRKD2,SMARCA4,TLR3,CD200,BCL10,RIPK1,KDM5A,ARHGEF2	up_VCAV_EC
GO:1901215	0.00788	0.998	9	57	negative regulation of neuron death	BP	0.446	HYOU1,AARS,HIF1A,ZPR1,CTNNB1,APOE,GSK3B,SIX4,NRP1	down_VSAP_EC
GO:0016070	0.00788	0.999	6	1479	RNA metabolic process	BP	1	TLR2,TBX15,EYA1,HOXA9,HOXA10,IRX5	up_AFEM_VSMC
GO:2000721	0.00788	1	1	2	positive regulation of transcription from RNA polymerase II promoter involved in smooth muscle cell differentiation	BP	1	NPNT	up_APUL_EC
GO:0010828	0.007908453	0.99933	3	16	positive regulation of glucose transmembrane	BP	0.96436359	NR1H4,ENSCAFG00000004331,RARRES2	up_ACOR_EC
GO:0007143	0.00791	0.999	4	10	female meiotic nuclear division	BP	0.369	TRIP13,FBXO5,NCAPH,TTK	down_ACOR_VSMC
GO:0001835	0.00792	1	2	2	blastocyst hatching	BP	0.369	SMARCB1,RBBP8	down_ACOR_VSMC
GO:0035188	0.00792	1	2	2	hatching	BP	0.369	SMARCB1,RBBP8	down_ACOR_VSMC
GO:0071684	0.00792	1	2	2	organism emergence from protective structure	BP	0.369	SMARCB1,RBBP8	down_ACOR_VSMC
GO:0010606	0.00792	1	2	2	positive regulation of cytoplasmic mRNA	BP	0.375	CNOT6,PAN3	up_VCAV_EC
GO:0035356	0.00792	1	1	2	cellular triglyceride homeostasis	BP	1	NR1H4	up_APUL_EC
GO:1904177	0.00792	1	1	2	regulation of adipose tissue development	BP	1	NR1H4	up_APUL_EC
GO:1904179	0.00792	1	1	2	positive regulation of adipose tissue	BP	1	NR1H4	up_APUL_EC
GO:1901228	0.00793	1	1	4	positive regulation of transcription from RNA polymerase II promoter involved in heart	BP	1	GATA5	up_VPUL_VSMC
GO:0007166	0.00794	0.995	57	686	cell surface receptor signaling pathway	BP	0.446	ARHGEF2,PRICKLE1,VEGFA,SIPA1L1,SULF1,NR1H4,HSPA5,SERPINE1,SOCS5,PRKACB,ENPP1,ERRFI1,ITGA2,JCAD,SNX25,PTPN11,ILK,SMARCA4,TGFBR1,MYO1C,CHSY1,HIF1A,CX3CL1,NXN,PTPN2,EIF2B1,NEO1,MTDH,ESM1,ADAM17,CNOT7,ADGRE5,GNAQ,CTNNB1,ENSCAFG00000002015,APOE,ZC3H15,LYN,STRAP,DDIT3,TLN1,CBLC,ATF3,CDC37,ITGB3,TAX1BP3,MYO1E,HTT,DISC1,PAK2,INHBA,ENSCAFG00000018360,HIF1AN,BTK,NRP1,RHBDF2,MADD	down_VSAP_EC
GO:0030900	0.00794	0.999	3	101	forebrain development	BP	0.842	PITX1,EMX2,SALL1	up_VSAP_VSMC
GO:0007603	0.00794	1	2	3	phototransduction, visible light	BP	0.446	GNAQ,RDH11	down_VSAP_EC
GO:0070858	0.00794	1	1	2	negative regulation of bile acid biosynthetic process	BP	1	NR1H4	up_APUL_EC
GO:1904252	0.00794	1	1	2	negative regulation of bile acid metabolic process	BP	1	NR1H4	up_APUL_EC
GO:0048738	0.00795	0.997	13	64	cardiac muscle tissue development	BP	0.907	TNNT2,ACTN2,BMP4,FGFR2,NEBL,ANKRD1,EGLN1,TBX20,TBX5,FRS2,CHD7,EDN1,BVES	up_ACOR_VSMC
GO:0001501	0.00795	0.999	3	178	skeletal system development	BP	1	HOXC10,HOXA9,HOXC6	up_AORT_EC
GO:0003164	0.00795	1	1	2	His-Purkinje system development	BP	1	TBX5	up_APUL_EC
GO:0003166	0.00795	1	1	2	bundle of His development	BP	1	TBX5	up_APUL_EC
GO:0099183	0.00795	1	1	2	trans-synaptic signaling by BDNF, modulating	BP	1	NTRK2	up_APUL_EC
GO:0099191	0.00795	1	1	2	trans-synaptic signaling by BDNF	BP	1	NTRK2	up_APUL_EC

GO:0099538	0.00795	1	1	2	synaptic signaling via neuropeptide	BP	1	NTRK2	up_APUL_EC
GO:0099540	0.00795	1	1	2	trans-synaptic signaling by neuropeptide	BP	1	NTRK2	up_APUL_EC
GO:0010159	0.00795	1	1	2	specification of animal organ position	BP	1	HOXA3	up_APUL_EC
GO:0010988	0.00795	1	1	2	regulation of low-density lipoprotein particle	BP	1	NR1H4	up_APUL_EC
GO:0034969	0.00795	1	1	2	histone arginine methylation	BP	1	NR1H4	up_APUL_EC
GO:0045764	0.00795	1	1	2	positive regulation of cellular amino acid	BP	1	NR1H4	up_APUL_EC
GO:0048702	0.00795	1	1	2	embryonic neurocranium morphogenesis	BP	1	HOXA1	up_APUL_EC
GO:0048934	0.00795	1	1	2	peripheral nervous system neuron	BP	1	NTRK2	up_APUL_EC
GO:0048935	0.00795	1	1	2	peripheral nervous system neuron	BP	1	NTRK2	up_APUL_EC
GO:0055022	0.00795	1	1	2	negative regulation of cardiac muscle tissue	BP	1	TBX5	up_APUL_EC
GO:0060044	0.00795	1	1	2	negative regulation of cardiac muscle cell	BP	1	TBX5	up_APUL_EC
GO:0060876	0.00795	1	1	2	semicircular canal formation	BP	1	HOXA1	up_APUL_EC
GO:0061117	0.00795	1	1	2	negative regulation of heart growth	BP	1	TBX5	up_APUL_EC
GO:0072615	0.00795	1	1	2	interleukin-17 secretion	BP	1	NR1H4	up_APUL_EC
GO:1902714	0.00795	1	1	2	negative regulation of interferon-gamma	BP	1	NR1H4	up_APUL_EC
GO:0051241	0.00796	0.996	32	336	negative regulation of multicellular organismal process	BP	0.446	PRICKLE1,SULF1,NR1H4,SERPINE1,SOCS5,SPARC,PRKACB,PGK1,PTPN11,DPYSL3,HOOK3,IFRD1,CHSY1,HIF1A,THBS2,PTPN2,PLCL1,CTNNB1,STARD13,APOE,WWC3,LYN,STRAP,DDIT3,REST,PRMT1,RAB11FIP1,INHBA,BTK,SIX4,NRP1,YWHAH	down_VSAP_EC
GO:0044281	0.00796	0.999	5	622	small molecule metabolic process	BP	1	ETFA,EXTL2,DARS,SUCLG2,NDUFA10	down_AFEM_EC
GO:0030001	0.00796	0.999	3	172	metal ion transport	BP	1	HOXA3,NKX2-5,TRPC4	up_VPOR_VSMC
GO:0030098	0.00798	0.997	15	87	lymphocyte differentiation	BP	0.376	IL7R,KIT,IL6,CHD7,DLL4,GATA3,MTOR,HHEX,ITPKB,MFNG,PLCG2,HMGB1,ATP11C,BMX,ZEB1	up_VCAV_EC
GO:0007379	0.00798	1	2	6	segment specification	BP	1	IRX3,DLL1	up_AFEM_EC
GO:0007267	0.00799	0.995	46	332	cell-cell signaling	BP	0.907	ADRA1B,LGR6,LEP,PLCL1,ZRANB1,FBXL20,RECK,MLLT3,FERMT1,BMP4,TM7SF3,FGFR2,GJA1,HRH1,LATS2,GLRB,ZNRF3,STX11,TMEM108,EGLN1,CACNB3,SLC12A7,CLSTN1,DMPK,SLITRK5,TBX5,PRKCE,FZD3,RNF19A,SCG5,DAG1,APP,PSEN1,CHD7,CCL5,RAB11B,EDN1,KCNC4,LRRC4,FAM107A,INSYN1,DISC1,PREPL,PFKL,ERAP1,PARK7	up_ACOR_VSMC
GO:0045214	0.00799	0.999	4	13	sarcomere organization	BP	0.446	ACTN1,WDR1,SIX4,MYBPC2	down_VSAP_EC
GO:0060838	0.00799	1	1	2	lymphatic endothelial cell fate commitment	BP	0.728	PDPN	down_ACOR_EC
GO:0022414	0.008	0.999	5	294	reproductive process	BP	0.842	HOXA11,HOXA9,SALL1,IRX5,GJB5	up_VSAP_VSMC
GO:0018171	0.00801	1	2	2	peptidyl-cysteine oxidation	BP	0.907	TMX3,PARK7	up_ACOR_VSMC
GO:0055023	0.00801	1	1	12	positive regulation of cardiac muscle tissue	BP	1	TBX5	up_APUL_VSMC
GO:0002526	0.00802	0.999	4	17	acute inflammatory response	BP	1	CLSTN1,B4GALT1,PARK7,A2M	down_VCAV_EC
GO:0042983	0.00802	1	2	3	amyloid precursor protein biosynthetic process	BP	1	NECAB3,SOAT1	down_VCAV_EC
GO:0042984	0.00802	1	2	3	regulation of amyloid precursor protein	BP	1	NECAB3,SOAT1	down_VCAV_EC
GO:0032640	0.00802	1	2	39	tumor necrosis factor production	BP	1	NR1H4,ARHGEF2	up_VPUL_EC
GO:0051235	0.00803	0.997	12	90	maintenance of location	BP	0.447	HSPA5,ERO1A,ENPP1,PTPN2,APOE,LYN,DDIT3,TLN1,HTT,ABHD5,CALM1,KDELR2	down_VSAP_EC
GO:0001525	0.00803	0.997	10	173	angiogenesis	BP	1	LEP,ANGPT4,HOXA7,DLL1,F3,TMEM100,GREM1,TBXA2R,PTPRB,CLIC4	up_AFEM_EC
GO:0003207	0.00803	1	3	5	cardiac chamber formation	BP	0.907	SMARCD3,TBX20,TBX5	up_ACOR_VSMC
GO:0007611	0.00803	1	2	54	learning or memory	BP	1	GATM,NETO1	up_VFEM_VSMC

GO:0051389	0.00803	1	1	1	inactivation of MAPKK activity	BP	0.728	IGF1R	down_ACOR_EC
GO:0061077	0.00804	0.999	5	25	chaperone-mediated protein folding	BP	1	HSPB6,FKBP10,FKBP5,FKBP11,CRTAP	down_VCAV_EC
GO:0010633	0.00804	1	1	28	negative regulation of epithelial cell migration	BP	1	JUP	down_VFEM_VSMC
GO:0048169	0.00804	1	1	3	regulation of long-term neuronal synaptic	BP	1	NETO1	up_VFEM_VSMC
GO:0010646	0.008060643	0.99537	37	945	regulation of cell communication	BP	0.96436359	CNTNAP4,SH3TC2,CDH3,NR1H4,BRSK1,PLCL1,SOX9,RGS1,NDRG2,KCNB1,FZD5,CCDC3,IFI6,DUSP26,DIXDC1,PTGIR,NOD2,PDE4A,SMAD6,ESM1,PAK3,TNFRSF19,ARHGDIB,ADAMTSL2,MAPK8IP2,MAVS,FIGNL1,LRRC15,LRP4,CLSTN1,SH3RF3,LRRC4,CLU,C23H3orf58,NRP1,ARHGEF26,PRKCD	up_ACOR_EC
GO:0051890	0.00807	1	1	4	regulation of cardioblast differentiation	BP	1	TBX5	up_VPUL_VSMC
GO:0000724	0.00809	0.997	11	57	double-strand break repair via homologous	BP	0.375	RPA1,PARPBP,GINS4,RAD54L,RAD51,TONSL,BRCA1,RBBP8,SMC6,H2AFX,GEN1	down_ACOR_VSMC
GO:0007399	0.00809	0.998	7	564	nervous system development	BP	0.842	PITX1,EMX2,SALL1,HOXC10,IRX5,SHOX2,HAPLN1	up_VSAP_VSMC
GO:0010159	0.00809	1	1	2	specification of animal organ position	BP	0.728	HOXA3	down_ACOR_EC
GO:0070571	0.0081	1	2	3	negative regulation of neuron projection	BP	1	THY1,RGMA	down_VCAV_EC
GO:0051241	0.008103949	0.99641	17	336	negative regulation of multicellular organismal process	BP	0.96436359	CDH3,NR1H4,PLCL1,SOX9,NDRG2,DIXDC1,RGMA,SMAD6,RNLS,PTGER4,ARHGDIB,FOXC2,TBX5,LRP4,NRP1,PRKCD,EFEMP1	up_ACOR_EC
GO:0009416	0.00812	0.999	3	88	response to light stimulus	BP	0.728	USP28,HOXA1,NEDD4	down_ACOR_EC
GO:0009083	0.00812	1	3	5	branched-chain amino acid catabolic process	BP	1	ALDH6A1,BCKDHB,ENSCAFG00000002977	up_VSAP_EC
GO:0060216	0.00812	1	1	3	definitive hemopoiesis	BP	1	HOXA9	up_AORT_EC
GO:0000003	0.00813	0.999	5	295	reproduction	BP	0.842	HOXA11,HOXA9,SALL1,IRX5,GJB5	up_VSAP_VSMC
GO:0090076	0.00813	1	2	2	relaxation of skeletal muscle	BP	0.381	KCNJ2,ATP2A1	up_VCAV_EC
GO:0042303	0.008132091	0.99888	4	30	molting cycle	BP	0.96436359	CDH3,SOX9,TNFRSF19,LRP4	up_ACOR_EC
GO:0042633	0.008132091	0.99888	4	30	hair cycle	BP	0.96436359	CDH3,SOX9,TNFRSF19,LRP4	up_ACOR_EC
GO:0060298	0.00814	1	2	2	positive regulation of sarcomere organization	BP	0.381	EDN1,PROX1	up_VCAV_EC
GO:2001135	0.00814	1	1	4	regulation of endocytic recycling	BP	1	RAB11B	down_VPOR_EC
GO:0003211	0.00814	1	1	4	cardiac ventricle formation	BP	1	TBX5	up_VPUL_VSMC
GO:0002755	0.00815	1	1	6	MyD88-dependent toll-like receptor signaling	BP	1	TLR2	up_AFEM_VSMC
GO:0010832	0.00815	1	1	3	negative regulation of myotube differentiation	BP	1	NKX2-5	up_VPOR_VSMC
GO:0003263	0.00815	1	1	4	cardioblast proliferation	BP	1	TBX5	up_VPUL_VSMC
GO:0045907	0.00816	1	2	6	positive regulation of vasoconstriction	BP	1	AVPR1A,TBXA2R	up_AFEM_EC
GO:0003179	0.00817	0.998	6	19	heart valve morphogenesis	BP	0.916	SMAD6,GATA5,GATA4,BMP4,TBX20,TBX5	up_ACOR_VSMC
GO:0071804	0.00817	1	2	31	cellular potassium ion transport	BP	0.842	NETO1,CAV1	up_VSAP_VSMC
GO:0071805	0.00817	1	2	31	potassium ion transmembrane transport	BP	0.842	NETO1,CAV1	up_VSAP_VSMC
GO:0016310	0.00818	0.994	70	641	phosphorylation	BP	0.381	EPHB1,PLK2,TEK,HCLS1,KIT,PPP2R5A,PRKCH,TNFAIP3,MAP3K11,TNIP1,PDGFB,STK10,SH2D3C,RELN,PDE4D,MEF2C,ATF3,RAPGEF1,TLR4,NLRC5,EDN1,RALB,APLNR,NEDD9,YES1,EPHB4,MTOR,MAP3K3,BMP2,HHEX,PREX2,SLK,BMP2K,SH3BP5,ERC1,MAPK13,ITPKB,PROX1,ENSCAFG00000010596,MADD,PAK2,PRKD2,SH3GLB1,TLR3,ENSCAFG00000024323,NCK1,LDB2,FES,NSD1,PTGS2,MMD,TOP1,RAPGEF2,CREB1,TAOK1,CNOT7,HMGB1,BLM,SPTBN4,RIPK1,ITGA6,RAD17,IMPA1,PDGFD,BMX,HIF1A,LMTK2,JCAD,ARHGEF2,CDON	up_VCAV_EC
GO:0006103	0.00818	1	3	5	2-oxoglutarate metabolic process	BP	1	GOT1,ADHFE1,IDH1	up_VSAP_EC
GO:1902415	0.00819	1	3	5	regulation of mRNA binding	BP	1	LARP6,CDK9,EIF3D	up_VSAP_EC

GO:1902416	0.00819	1	3	5	positive regulation of mRNA binding	BP	1	LARP6,CDK9,EIF3D	up_VSAP_EC
GO:1904888	0.00819	1	2	31	cranial skeletal system development	BP	0.842	TBX15,IRX5	up_VSAP_VSMC
GO:0060136	0.00819	1	1	4	embryonic process involved in female	BP	1	CITED2	down_VPOR_EC
GO:0042713	0.00819	1	1	1	sperm ejaculation	BP	1	ACVR2A	up_VPOR_EC
GO:0043084	0.00819	1	1	1	penile erection	BP	1	ACVR2A	up_VPOR_EC
GO:2001016	0.00819	1	1	2	positive regulation of skeletal muscle cell	BP	0.795	CYP26B1	up_VFEM_EC
GO:0021563	0.00819	1	1	4	glossopharyngeal nerve development	BP	1	HOXA3	up_VPUL_VSMC
GO:0051259	0.0082	0.996	20	140	protein complex oligomerization	BP	0.381	TEK,GBP5,CDA,VWF,ACPP,LRRC8C,LNX2,RIOK3,SNX2,KCNJ2,EHD4,BCL10,GCH1,PMAIP1,BLM,RIPK1,MCU,ALDH1A3,P2RX2,TNFAIP1	up_VCAV_EC
GO:0000902	0.00821	0.996	27	266	cell morphogenesis	BP	0.454	ARHGEF2,VEGFA,SIPA1L1,MYH9,FMNL3,ACTN1,SPARC,SGK1,AKAP2,PTPN11,FLNB,IFRD1,ILK,WDR1,MSN,KIF5B,TNIK,NEO1,UST,CDH11,CTNNB1,ENSCAFG00000002015,PLOD3,VLDLR,EXT1,NRP1,YWHAH	down_VSAP_EC
GO:1904951	0.008212854	0.99766	8	108	positive regulation of establishment of protein localization	BP	0.96436359	NR1H4,KCNB1,EDEM2,FZD5,NOD2,PTGER4,MAVS,PRKCD	up_ACOR_EC
GO:0042462	0.00822	0.999	3	9	eye photoreceptor cell development	BP	1	NAGLU,THY1,THRB	down_VCAV_EC
GO:0051298	0.00823	0.998	8	35	centrosome duplication	BP	0.378	POC1A,WDR62,NUP62,PLK4,CDK2,SASS6,RBM14,GEN1	down_ACOR_VSMC
GO:0000731	0.00823	0.999	4	10	DNA synthesis involved in DNA repair	BP	0.378	POLE,PCNA,POLH,DTL	down_ACOR_VSMC
GO:0009887	0.00823	0.999	3	314	animal organ morphogenesis	BP	1	TBX15,EYA1,IRX5	up_AFEM_VSMC
GO:0071623	0.00823	1	2	2	negative regulation of granulocyte chemotaxis	BP	1	C5AR2,SLIT2	up_VSAP_EC
GO:0090024	0.00823	1	2	2	negative regulation of neutrophil chemotaxis	BP	1	C5AR2,SLIT2	up_VSAP_EC
GO:1902623	0.00823	1	2	2	negative regulation of neutrophil migration	BP	1	C5AR2,SLIT2	up_VSAP_EC
GO:0035754	0.00823	1	1	4	B cell chemotaxis	BP	1	HSD3B7	down_VPOR_EC
GO:0060261	0.00825	1	1	8	positive regulation of transcription initiation from RNA polymerase II	BP	0.775	ESR1	up_AORT_VSMC
GO:0002513	0.00827	1	1	2	tolerance induction to self antigen	BP	0.733	LYN	down_ACOR_EC
GO:0010715	0.00827	1	1	4	regulation of extracellular matrix disassembly	BP	1	FSCN1	down_AFEM_EC
GO:0002683	0.00828	1	2	126	negative regulation of immune system process	BP	0.775	HOXA9,ARG2	up_AORT_VSMC
GO:0031290	0.00828	1	1	4	retinal ganglion cell axon guidance	BP	1	SLIT2	up_VPUL_VSMC
GO:1902668	0.00829	1	1	4	negative regulation of axon guidance	BP	1	SLIT2	up_VPUL_VSMC
GO:0007399	0.0083	0.998	8	564	nervous system development	BP	0.733	LYN,HOXA3,ACSL4,SPG11,NRP2,SULF1,HOXA1,NEDD4	down_ACOR_EC
GO:0032620	0.00831	1	1	8	interleukin-17 production	BP	0.775	ARG2	up_AORT_VSMC
GO:0008625	0.00833	0.998	7	30	extrinsic apoptotic signaling pathway via death domain receptors	BP	0.386	TNFAIP3,ATF3,CASP8,MADD,PMAIP1,SKIL,ARHGEF2	up_VCAV_EC
GO:0002673	0.00834	1	1	6	regulation of acute inflammatory response	BP	1	C6	up_VCAV_VSMC
GO:0032825	0.00835	1	1	2	positive regulation of natural killer cell	BP	0.795	TOX	up_VFEM_EC
GO:0031324	0.00836	0.998	9	735	negative regulation of cellular metabolic process	BP	0.733	ACVRL1,LYN,IGF1R,HOXA7,PRKAR2A,ELK3,MYO1D,ASPN,NEDD4	down_ACOR_EC
GO:0002431	0.00836	1	1	2	Fc receptor mediated stimulatory signaling	BP	0.733	LYN	down_ACOR_EC
GO:0050873	0.00837	0.999	3	18	brown fat cell differentiation	BP	1	LEP,NAPEPLD,PPARGC1A	up_AFEM_EC
GO:2000515	0.00837	1	1	8	negative regulation of CD4-positive, alpha-beta T cell activation	BP	0.775	ARG2	up_AORT_VSMC
GO:0009166	0.00838	0.998	6	31	nucleotide catabolic process	BP	0.456	PGK1,TPI1,PGAM1,HIF1A,ENTPD4,ALDOC	down_VSAP_EC
GO:0032680	0.00838	1	2	36	regulation of tumor necrosis factor production	BP	1	NR1H4,BPI	up_APUL_EC

GO:0009653	0.00839	0.998	6	773	anatomical structure morphogenesis	BP	1	HOXC10,EYA1,HOXA9,TBX15,HOXA11,SHOX2	up_VFEM_VSMC
GO:0071706	0.00839	1	2	40	tumor necrosis factor superfamily cytokine	BP	1	NR1H4,ARHGEF2	up_VPUL_EC
GO:0021825	0.00839	1	1	4	substrate-dependent cerebral cortex tangential	BP	1	SLIT2	up_VPUL_VSMC
GO:0060603	0.0084	1	1	7	mammary gland duct morphogenesis	BP	0.775	ESR1	up_AORT_VSMC
GO:0050789	0.00841	0.997	21	3295	regulation of biological process	BP	0.795	HOXC10,EYA1,HOXA9,TOX,MAP2K6,AVPR1A,HOXA10,TRIM9,HOXB7,HOXA7,ENSCAFG00000018194,SIX1,YBX3,MAB21L1,TIAM1,RADIL,RASD2,CYP26B1,CDC42EP5,MTSS1,ADCY3	up_VFEM_EC
GO:0007416	0.00841	1	2	36	synapse assembly	BP	1	SLITRK5,NTRK2	up_APUL_EC
GO:0072006	0.00842	1	2	59	nephron development	BP	1	EYA1,HOXA11	up_VFEM_VSMC
GO:0007219	0.00842	1	1	59	Notch signaling pathway	BP	1	GATA5	down_AFEM_VSMC
GO:0098528	0.00842	1	1	2	skeletal muscle fiber differentiation	BP	0.795	CYP26B1	up_VFEM_EC
GO:0071605	0.00843	1	2	3	monocyte chemotactic protein-1 production	BP	0.456	NR1H4,SOCS5	down_VSAP_EC
GO:0071637	0.00843	1	2	3	regulation of monocyte chemotactic protein-1	BP	0.456	NR1H4,SOCS5	down_VSAP_EC
GO:0071638	0.00843	1	2	3	negative regulation of monocyte chemotactic	BP	0.456	NR1H4,SOCS5	down_VSAP_EC
GO:0007267	0.00844	0.999	4	332	cell-cell signaling	BP	1	BARX1,NKX2-5,BAMBI,ANKRD6	up_VPOR_VSMC
GO:0060048	0.00844	1	1	30	cardiac muscle contraction	BP	1	JUP	down_VFEM_VSMC
GO:0032609	0.00845	0.998	6	25	interferon-gamma production	BP	0.387	PDE4D,TLR4,GATA3,IRF8,TLR3,HMGB1	up_VCAV_EC
GO:0007043	0.00845	1	1	28	cell-cell junction assembly	BP	1	JUP	down_VFEM_VSMC
GO:0008582	0.00845	1	1	2	regulation of synaptic growth at neuromuscular	BP	0.795	SIX1	up_VFEM_EC
GO:0030910	0.00845	1	1	2	olfactory placode formation	BP	0.795	SIX1	up_VFEM_EC
GO:0031339	0.00845	1	1	2	negative regulation of vesicle fusion	BP	0.795	TRIM9	up_VFEM_EC
GO:0061055	0.00845	1	1	2	myotome development	BP	0.795	SIX1	up_VFEM_EC
GO:0071698	0.00845	1	1	2	olfactory placode development	BP	0.795	SIX1	up_VFEM_EC
GO:0071699	0.00845	1	1	2	olfactory placode morphogenesis	BP	0.795	SIX1	up_VFEM_EC
GO:0050673	0.00846	0.996	19	135	epithelial cell proliferation	BP	0.387	TNFAIP3,PDGFB,MEF2C,FERMT1,DLL4,GATA3,APLNR,EGFL7,MTSS1,BMP2,CAV2,PROX1,MCC,PRKD2,HMGB1,NRAS,NR4A1,VASH1,JCAD	up_VCAV_EC
GO:0009889	0.00846	0.997	11	1190	regulation of biosynthetic process	BP	0.853	HOXA11,HOXA10,HOXA9,PITX1,EMX2,SALL1,IRX5,PRR16,TLR2,CAV1,SHOX2	up_VSAP_VSMC
GO:0033566	0.00846	1	1	1	gamma-tubulin complex localization	BP	1	MZT1	up_VPOR_EC
GO:0009605	0.00847	0.998	6	538	response to external stimulus	BP	1	NR1H4,RGS1,MRC1,CCL24,FLRT3,NEO1	up_VPUL_EC
GO:0035107	0.00847	1	2	60	appendage morphogenesis	BP	1	HOXC10,HOXA9	up_AORT_EC
GO:0035108	0.00847	1	2	60	limb morphogenesis	BP	1	HOXC10,HOXA9	up_AORT_EC
GO:1901605	0.00847866	0.9981	6	67	alpha-amino acid metabolic process	BP	0.96436359	NR1H4,THNSL2,NMNAT2,SARDH,NIT2,AZIN2	up_ACOR_EC
GO:0055080	0.00848	0.999	3	176	cation homeostasis	BP	1	HOXA3,TRPC4,C3AR1	up_VPOR_VSMC
GO:0071310	0.00848	1	2	634	cellular response to organic substance	BP	1	JUP,GATA5	down_VFEM_VSMC
GO:0071495	0.00849	0.995	50	365	cellular response to endogenous stimulus	BP	0.934	SMAD6,ACTN2,LEP,LBH,GATA5,SKP2,PRKAA2,FERMT1,BMP4,FGFR2,MED14,CASTOR1,LATS2,CITED2,AR,TMEM108,FGF18,FAM210B,CDKN2B,ADCY6,ANKRD1,CTSB,AKAP6,CREBRF,UBR1,PTPN22,NPNT,APLP1,PTGDR,SESN3,PRKCE,FRS2,AGRN,FUT8,APP,PSEN1,TGFBR1,JUP,NR3C1,DUSP22,HPGD,NOD2,FAM107A,DDIT4,ENSCAFG00000018360,GNAQ,INPP5K,SH3BP4,PARK7,CRKL	up_ACOR_VSMC
GO:0016576	0.00849	1	1	2	histone dephosphorylation	BP	0.795	EYA1	up_VFEM_EC
GO:0048706	0.0085	0.997	11	59	embryonic skeletal system development	BP	0.387	EIF4A3,HOXA3,XYLT1,MMP14,HOXA7,HOXB7,HOXC6,HOXB5,TBX15,HOXA9,SIX4	down_ACOR_VSMC

GO:0007010	0.00852	0.995	53	441	cytoskeleton organization	BP	0.388	PLK2,TEK,HCLS1,KIT,ARHGEF15,ENSCAFG00000007112,AMOT,BAIAP2L1,MEF2C,EDN1,RAB11A,ARHGAP28,TMOD1,MTSS1,NECTIN2,MTOR,HOOK3,SLK,CEP68,PDZD8,NCKAP1,MZT1,PROX1,IQGAP2,ENSCAFG00000010224,EPB41,ELMO1,STMN1,FLNB,PAK2,SWAP70,NCK1,FES,SENP6,GCC2,TAOK1,CTNNA1,FMNL3,VPS54,ARPC5L,ARHGDIB,ZW10,ARHGAP26,ATRX,HAUS3,GAS2L3,TNFAIP1,TMOD3,ARHGEF2,FSCN1,FBXO5,ZMYM2,SASS6	up_VCAV_EC
GO:0006508	0.00852	0.995	42	483	proteolysis	BP	0.456	PRICKLE1,MYH9,PLK3,HSPA5,SERPINE1,SOCS5,HM13,PRKACB,HSP90B1,ENSCAFG00000005575,PGK1,TRIB3,PRCP,PTGS2,SEL1L,USP12,DNAJC10,UBA6,UCHL5,SPOPL,ADAM17,DNAJB9,CRYAB,APOE,USP48,LYN,DDIT3,FAM162A,CBLC,GSK3B,CUL2,REST,SPOP,DISC1,RAD23A,PAK2,A2M,TMTC3,SPINK2,RHBDF2,USP14,TNFAIP1	down_VSAP_EC
GO:0032269	0.00852	0.995	29	306	negative regulation of cellular protein metabolic process	BP	0.456	SERPINE1,SOCS5,ENPP1,ERRFI1,SNX25,TRIB3,SESN2,PTGS2,DNAJC10,NXN,PTPN2,UCHL5,SPOPL,EIF4EBP1,CNOT7,CRYAB,APOE,LYN,STRAP,CBLC,ATF3,EIF2S1,CDYL,PAK2,A2M,CDKN1A,SPINK2,USP14,KDM3A	down_VSAP_EC
GO:0051239	0.00852	0.998	9	859	regulation of multicellular organismal process	BP	0.854	HOXA11,HOXA9,NETO1,GATM,HOXC10,IRX5,TLR2,CAV1,SHOX2	up_VSAP_VSMC
GO:1901863	0.00852	0.998	7	25	positive regulation of muscle tissue	BP	0.934	BMP4,FGFR2,GJA1,PPARGC1A,TBX20,TBX5,EDN1	up_ACOR_VSMC
GO:0072520	0.00853	1	3	5	seminiferous tubule development	BP	0.934	ATRX,SPINK2,WDR48	up_ACOR_VSMC
GO:0008209	0.00854	1	1	8	androgen metabolic process	BP	0.78	ESR1	up_AORT_VSMC
GO:0061387	0.00855	1	2	18	regulation of extent of cell growth	BP	1	SEMA3A,RNF6	up_VPOR_EC
GO:0021756	0.00856	1	1	6	striatum development	BP	1	SLITRK5	up_VCAV_VSMC
GO:0016525	0.00857	0.998	7	38	negative regulation of angiogenesis	BP	0.456	SULF1,SERPINE1,SPARC,PGK1,THBS2,CTNNB1,STARD13	down_VSAP_EC
GO:2000181	0.00857	0.998	7	38	negative regulation of blood vessel	BP	0.456	SULF1,SERPINE1,SPARC,PGK1,THBS2,CTNNB1,STARD13	down_VSAP_EC
GO:0010453	0.00857	0.999	3	9	regulation of cell fate commitment	BP	1	BMPR1A,LMO4,LOXL3	down_VCAV_EC
GO:0071346	0.00857	1	2	41	cellular response to interferon-gamma	BP	1	MRC1,CCL24	up_VPUL_EC
GO:0035914	0.00858	0.998	6	22	skeletal muscle cell differentiation	BP	0.39	EPHB1,MEF2C,ATF3,FOXN2,NR1D2,CDON	up_VCAV_EC
GO:0003128	0.00858	1	1	6	heart field specification	BP	1	GATA5	up_VCAV_VSMC
GO:0070170	0.00858	1	1	1	regulation of tooth mineralization	BP	1	ASPN	up_VPOR_EC
GO:0070171	0.00858	1	1	1	negative regulation of tooth mineralization	BP	1	ASPN	up_VPOR_EC
GO:0030182	0.00859	0.999	3	327	neuron differentiation	BP	1	HOXC10,EYA1,IRX5	up_AFEM_VSMC
GO:0060689	0.00859	1	2	2	cell differentiation involved in salivary gland	BP	1	CLCN2,NFIB	up_VSAP_EC
GO:1901888	0.0086	0.998	7	29	regulation of cell junction assembly	BP	0.39	TEK,PRKCH,RAPGEF1,SLK,APOD,RAPGEF2,ENSCAFG00000002015	up_VCAV_EC
GO:0008406	0.0086	1	2	57	gonad development	BP	1	HOXA9,HOXA11	up_VFEM_VSMC
GO:0045137	0.0086	1	2	57	development of primary sexual characteristics	BP	1	HOXA9,HOXA11	up_VFEM_VSMC
GO:0003221	0.00861	1	1	3	right ventricular cardiac muscle tissue	BP	1	NKX2-5	up_VPOR_VSMC
GO:0055074	0.00862	0.996	17	95	calcium ion homeostasis	BP	0.934	ADRA1B,PLCG2,HTR2B,MICU2,ITPR1,F2RL1,DMPK,PRKCE,APP,PSEN1,CHD7,EDN1,DISC1,HERPUD1,WFS1,ATP2C1,STC2	up_ACOR_VSMC
GO:1904063	0.00862	0.999	5	17	negative regulation of cation transmembrane	BP	0.392	NEDD4,CAV1,SLC30A1,GSTO1,REM1	down_ACOR_VSMC
GO:0030154	0.00863	0.999	5	1040	cell differentiation	BP	1	HOXC10,EYA1,HOXA9,BEND2,IRX5	up_AFEM_VSMC
GO:0010954	0.00864	1	1	6	positive regulation of protein processing	BP	1	C6	up_VCAV_VSMC
GO:1903319	0.00864	1	1	6	positive regulation of protein maturation	BP	1	C6	up_VCAV_VSMC
GO:0042711	0.00864	1	1	2	maternal behavior	BP	0.795	AVPR1A	up_VFEM_EC
GO:0060746	0.00864	1	1	2	parental behavior	BP	0.795	AVPR1A	up_VFEM_EC

GO:0051444	0.00865	1	1	6	negative regulation of ubiquitin-protein	BP	1	LIMK1	up_VCAV_VSMC
GO:0045773	0.00866	1	1	6	positive regulation of axon extension	BP	1	LIMK1	up_VCAV_VSMC
GO:0007625	0.00867	1	1	6	grooming behavior	BP	1	SLITRK5	up_VCAV_VSMC
GO:0042471	0.00868	1	2	34	ear morphogenesis	BP	0.795	EYA1,SIX1	up_VFEM_EC
GO:1904385	0.00868	1	1	6	cellular response to angiotensin	BP	1	CA2	up_VCAV_VSMC
GO:1990776	0.00868	1	1	6	response to angiotensin	BP	1	CA2	up_VCAV_VSMC
GO:0031424	0.00868	1	1	3	keratinization	BP	1	CYP26B1	up_VPOR_VSMC
GO:0045576	0.00869	0.999	4	12	mast cell activation	BP	0.393	KIT,NECTIN2,SNAP23,FES	up_VCAV_EC
GO:0038092	0.00869	1	1	4	nodal signaling pathway	BP	1	CITED2	down_VPOR_EC
GO:0001659	0.00872	0.998	5	54	temperature homeostasis	BP	1	CMKLR1,HOXC10,LEP,NAPEPLD,PPARGC1A	up_AFEM_EC
GO:0071286	0.00873	1	1	1	cellular response to magnesium ion	BP	1	FBP1	up_VPOR_EC
GO:0006793	0.008735436	0.99496	37	948	phosphorus metabolic process	BP	0.96436359	NR1H4,BRSK1,PLCL1,PKIB,THNSL2,SOX9,NMNAT2,NDRG2,ENSCAFG00000004331,FZD5,DUSP26,NOD2,SMAD6,PAK3,CFI,GUCY1B1,GRK7,TNFRSF19,DCLK2,CHPT1,MAPK8IP2,MAVS,LRRC15,PIGS,LRP4,SH3RF3,AK7,LRRC4,ALDOC,RARRES2,STK32C,NRP1,AGPAT2,PRKCD,PPP1R26,DBNDD2,CCNYL1	up_ACOR_EC
GO:0070302	0.00874	0.996	15	78	regulation of stress-activated protein kinase signaling cascade	BP	0.934	LEP,MID1,RASSF2,TLR3,SH3RF2,SASH1,F2RL1,PTPN22,SH3RF1,APP,DUSP22,EDN1,NOD2,SH3RF3,MAP3K7	up_ACOR_VSMC
GO:0010896	0.00874	1	1	1	regulation of triglyceride catabolic process	BP	1	ABHD5	up_VPOR_EC
GO:0010898	0.00874	1	1	1	positive regulation of triglyceride catabolic	BP	1	ABHD5	up_VPOR_EC
GO:0045638	0.00875	1	2	33	negative regulation of myeloid cell	BP	0.795	HOXA9,HOXA7	up_VFEM_EC
GO:0048754	0.00875	1	2	57	branching morphogenesis of an epithelial tube	BP	1	EYA1,HOXA11	up_VFEM_VSMC
GO:0005984	0.00875	1	1	3	disaccharide metabolic process	BP	1	FBP1	up_VFEM_VSMC
GO:0046351	0.00875	1	1	3	disaccharide biosynthetic process	BP	1	FBP1	up_VFEM_VSMC
GO:0043062	0.00877	0.998	7	99	extracellular structure organization	BP	1	CTSS,MFAP4,PLAGL2,GREM1,POSTN,RECK,FAP	up_AFEM_EC
GO:0035115	0.00877	1	1	12	embryonic forelimb morphogenesis	BP	1	TBX5	up_APUL_VSMC
GO:0021978	0.00877	1	1	3	telencephalon regionalization	BP	1	EMX2	up_VFEM_VSMC
GO:0031126	0.00878	0.999	3	6	snoRNA 3'-end processing	BP	0.396	FBL,ENSCAFG00000019620,EXOSC5	down_ACOR_VSMC
GO:0006706	0.00878	1	2	3	steroid catabolic process	BP	0.463	APOE,YWHAH	down_VSAP_EC
GO:0007275	0.00879	0.997	11	1370	multicellular organism development	BP	1	NR1H4,TBX5,HOXA2,MAPK10,HOXA3,ZDHHC16,SLITRK5,NPNT,NTRK2,HOXA1,PDLIM3	up_APUL_EC
GO:0060349	0.0088	0.997	9	38	bone morphogenesis	BP	1	HOXA11,PEX7,AXIN2,OSR2,INSIG2,RARG,SHOX2,TGFBR2,CITED2	up_VSAP_EC
GO:0070141	0.0088	1	1	2	response to UV-A	BP	0.795	MME	up_VFEM_EC
GO:0048645	0.00881	1	2	33	animal organ formation	BP	0.795	EYA1,SIX1	up_VFEM_EC
GO:0009404	0.00882	1	1	6	toxin metabolic process	BP	1	CYP1A1	up_VCAV_VSMC
GO:0009111	0.00882	1	1	2	vitamin catabolic process	BP	0.795	CYP26B1	up_VFEM_EC
GO:1903350	0.00883	1	1	4	response to dopamine	BP	1	GABPA	down_AFEM_EC
GO:1903351	0.00883	1	1	4	cellular response to dopamine	BP	1	GABPA	down_AFEM_EC
GO:0031023	0.00884	0.997	11	58	microtubule organizing center organization	BP	0.397	HAUS8,NEK2,POC1A,WDR62,NUP62,PLK4,CDK2,SASS6,RBM14,KIF11,GEN1	down_ACOR_VSMC
GO:0007155	0.00884	0.998	6	324	cell adhesion	BP	0.763	ACVRL1,LYN,PDPN,HOXA7,SULF1,STX4	down_ACOR_EC
GO:0045926	0.00884	1	2	72	negative regulation of growth	BP	1	TBX5,SLIT2	up_VPUL_VSMC
GO:0048856	0.00885	1	4	1533	anatomical structure development	BP	1	HOXA3,TBX5,HOXA2,HOXB5	up_APUL_VSMC
GO:0042573	0.00885	1	1	4	retinoic acid metabolic process	BP	1	CYP26B1	up_VPOR_VSMC

GO:0090257	0.00886	0.997	11	52	regulation of muscle system process	BP	0.934	TNNT2,FGF13,ADRA1B,KCNE3,GATA5,PDE5A,DMPK,NPNT,JUP,EDN1,DOCK5	up_ACOR_VSMC
GO:1903555	0.00886	1	2	37	regulation of tumor necrosis factor superfamily cytokine	BP	1	NR1H4,BPI	up_APUL_EC
GO:0006828	0.00886	1	1	3	manganese ion transport	BP	1	TRPC4	up_VPOR_VSMC
GO:0032769	0.00886	1	1	2	negative regulation of monooxygenase activity	BP	0.857	CAV1	up_VSAP_VSMC
GO:0051001	0.00886	1	1	2	negative regulation of nitric-oxide synthase	BP	0.857	CAV1	up_VSAP_VSMC
GO:0086103	0.00886	1	1	2	G protein-coupled receptor signaling pathway involved in heart	BP	0.857	CAV1	up_VSAP_VSMC
GO:0072001	0.00887	0.999	3	104	renal system development	BP	0.857	HOXA11,EMX2,SALL1	up_VSAP_VSMC
GO:0042113	0.00888	0.997	12	64	B cell activation	BP	0.4	IL7R,KIT,TNFAIP3,MEF2C,TLR4,PELI1,HHEX,THOC1,SWAP70,MFNG,PLCG2,ATP11C	up_VCAV_EC
GO:0050673	0.00888	1	2	135	epithelial cell proliferation	BP	0.803	WNT7A,ESR1	up_AORT_VSMC
GO:0030038	0.00888093	0.99875	4	31	contractile actin filament bundle assembly	BP	0.96436359	ARHGAP6,ARHGAP28,PTGER4,NRP1	up_ACOR_EC
GO:0043149	0.00888093	0.99875	4	31	stress fiber assembly	BP	0.96436359	ARHGAP6,ARHGAP28,PTGER4,NRP1	up_ACOR_EC
GO:0072191	0.00889	1	1	2	ureter smooth muscle development	BP	0.795	SIX1	up_VFEM_EC
GO:0072193	0.00889	1	1	2	ureter smooth muscle cell differentiation	BP	0.795	SIX1	up_VFEM_EC
GO:0072198	0.00889	1	1	2	mesenchymal cell proliferation involved in	BP	0.795	SIX1	up_VFEM_EC
GO:0072199	0.00889	1	1	2	regulation of mesenchymal cell proliferation involved in	BP	0.795	SIX1	up_VFEM_EC
GO:0003151	0.0089	1	2	34	outflow tract morphogenesis	BP	0.795	EYA1,SIX1	up_VFEM_EC
GO:0009653	0.00892	0.999	4	773	anatomical structure morphogenesis	BP	0.803	HOXC10,HOXA9,WNT7A,ESR1	up_AORT_VSMC
GO:0043031	0.00893	1	1	4	negative regulation of macrophage activation	BP	1	BPI	up_AORT_EC
GO:2000027	0.00895	1	2	72	regulation of animal organ morphogenesis	BP	1	TBX5,GATA5	up_VPUL_VSMC
GO:0007010	0.00896	0.994	52	441	cytoskeleton organization	BP	0.402	FSCN1,FLNB,FMNL3,SMC1A,ZW10,CAPG,CENPA,FBXO5,BIRC5,CDK1,LIMA1,NF2,AURKB,STMN1,HAUS8,MAP3K20,NEK2,GAS2L3,PLK3,RCC1,POC1A,WDR62,ESPL1,SPC25,NDC80,RAC1,NUP62,EMP2,PDXP,PLK4,IQGAP2,CDC42EP4,SMC3,CDK2,STAG1,ENSCAFG00000005299,EZR,SASS6,RBM14,CORO1A,PHACTR4,CDC20,ASPM,MAST2,ARPC1B,ENSCAFG00000010224,ARHGEF2,KIF11,SIX4,TTLL1,GEN1,KIF24	down_ACOR_VSMC
GO:0001655	0.00896	1	2	116	urogenital system development	BP	0.803	ARG2,ESR1	up_AORT_VSMC
GO:0072697	0.00896	1	1	4	protein localization to cell cortex	BP	1	GPSM2	down_AFEM_EC
GO:0045765	0.00897	0.997	13	105	regulation of angiogenesis	BP	0.47	VEGFA,SULF1,SERPINE1,SPARC,JCAD,PGK1,PIK3C2A,HIF1A,THBS2,MTDH,CTNNB1,STARD13,NRP1	down_VSAP_EC
GO:1901362	0.00897	0.998	8	1293	organic cyclic compound biosynthetic process	BP	1	EYA1,HOXA10,HOXA9,TBX15,FBP1,EMX2,HOXA11,SHOX2	up_VFEM_VSMC
GO:0001656	0.00897	1	2	35	metanephros development	BP	0.795	EYA1,SIX1	up_VFEM_EC
GO:0051661	0.00897	1	1	4	maintenance of centrosome location	BP	1	GPSM2	down_AFEM_EC
GO:0001974	0.00897	1	1	13	blood vessel remodeling	BP	1	HOXA3	up_APUL_VSMC
GO:0072378	0.00898	1	2	2	blood coagulation, fibrin clot formation	BP	1	KLKB1,FBLN1	up_VSAP_EC
GO:0002042	0.00899	0.998	7	29	cell migration involved in sprouting angiogenesis	BP	0.404	PLK2,CLEC14A,DLL4,PIK3C2A,EPHB4,MAP3K3,JCAD	up_VCAV_EC
GO:0014902	0.00899	1	2	34	myotube differentiation	BP	0.863	STIM1,SHOX2	up_VSAP_VSMC
GO:1902626	0.00899	1	1	1	assembly of large subunit precursor of preribosome	BP	1	RSL24D1	up_VPOR_EC
GO:0032635	0.00901	1	2	37	interleukin-6 production	BP	1	NR1H4,BPI	up_APUL_EC
GO:0030035	0.00901	1	1	4	microspike assembly	BP	1	FSCN1	down_AFEM_EC
GO:0032534	0.00901	1	1	4	regulation of microvillus assembly	BP	1	FSCN1	down_AFEM_EC

GO:2001236	0.00902	0.997	11	59	regulation of extrinsic apoptotic signaling	BP	0.404	TNFAIP3,ATF3,MADD,PAK2,BCL10,CTNNA1,PMAIP1,RIPK1,ITGA6,SKIL,ARHGEF2	up_VCAV_EC
GO:0046824	0.00902	1	1	30	positive regulation of nucleocytoplasmic	BP	1	JUP	down_VFEM_VSMC
GO:0060216	0.00902	1	1	3	definitive hemopoiesis	BP	1	HOXA9	up_VFEM_VSMC
GO:0044271	0.00903	0.999	6	1501	cellular nitrogen compound biosynthetic	BP	1	TLR2,TBX15,EYA1,HOXA9,HOXA10,IRX5	up_AFEM_VSMC
GO:0099576	0.00903	1	2	2	regulation of protein catabolic process at postsynapse, modulating	BP	0.934	EGLN1,RNF19A	up_ACOR_VSMC
GO:0072073	0.00903	1	2	60	kidney epithelium development	BP	1	EYA1,HOXA11	up_VFEM_VSMC
GO:0032879	0.00904	0.994	62	767	regulation of localization	BP	0.471	ARHGEF2,VEGFA,SULF1,NR1H4,PLK3,HSPA5,SERPINE1,ACTN1,SPARC,ERO1A,PTPN14,ENPP1,JCAD,PTPN11,DPYSL3,TRIB3,PPP1R12A,PRCP,SESN2,REM1,WDR1,MYO1C,PIK3C2A,MSN,KIF5B,HIF1A,IWS1,YIPF5,ZPR1,PTPN2,YES1,ADAM17,CTNNB1,CRYAB,STARD13,APOE,PTP4A1,MMP28,LYN,STRAP,DDIT3,MMD,GSK3B,NKAIN1,NEXN,SH3GLB1,TAX1BP3,SDC4,HTT,REST,ABHD5,ZMYND8,CALM1,RAB11FIP1,LRRTM2,INHBA,SIX4,CDKN1A,NRP1,RHBDF2,YWHAH,HOMER1	down_VSAP_EC
GO:0090100	0.00904	1	2	31	positive regulation of transmembrane receptor protein serine/threonine kinase signaling pathway	BP	0.773	ACVRL1,SULF1	down_ACOR_EC
GO:0045056	0.00904	1	1	5	transcytosis	BP	1	RAB11B	down_VPOR_EC
GO:0072507	0.00905	0.996	18	103	divalent inorganic cation homeostasis	BP	0.934	ADRA1B,PLCG2,HTR2B,CNNM2,MICU2,ITPR1,F2RL1,DMPK,PRKCE,APP,PSEN1,CHD7,EDN1,DISC1,HERPUD1,WFS1,ATP2C1,STC2	up_ACOR_VSMC
GO:0022610	0.00906	0.998	6	326	biological adhesion	BP	0.773	ACVRL1,LYN,PDPN,HOXA7,SULF1,STX4	down_ACOR_EC
GO:0018198	0.00906	0.999	4	10	peptidyl-cysteine modification	BP	0.934	TMX3,OXR1,PARK7,ZDHHC21	up_ACOR_VSMC
GO:0098771	0.00907	0.999	3	180	inorganic ion homeostasis	BP	1	HOXA3,TRPC4,C3AR1	up_VPOR_VSMC
GO:0042306	0.00907	1	1	31	regulation of protein import into nucleus	BP	1	JUP	down_VFEM_VSMC
GO:0007165	0.00909	0.993	111	1496	signal transduction	BP	0.471	ARHGEF2,PRICKLE1,VEGFA,P4HB,SIPA1L1,SULF1,ARHGAP31,NR1H4,HSPA5,SERPINE1,SOCS5,SGK1,ERO1A,PRKACB,ENPP1,ERRFI1,ITGA2,JCAD,SNX25,CASP3,HYOU1,WSB1,BCAR3,NUAK1,PTPN11,LRRC8A,TRIB3,FARP1,ILK,FHL2,PPP1R12A,PRCP,SMARCA4,SESN2,TGFBR1,PTGS2,REM1,MYO1C,PIK3C2A,CHSY1,UACA,HIF1A,CX3CL1,ENSCAFG00000000562,DNAJC10,TNIK,NXN,RGS2,PTPN2,EIF2B1,NEO1,PLCL1,MTDH,ESM1,ADAM17,AGRN,EIF4EBP1,SKAP2,CNOT7,ADGRE5,MAGED1,GNAQ,CTNNB1,KPNB1,STARD13,ENSCAFG00000002015,APOE,WWC3,PDE1A,ZC3H15,UBA5,LYN,BNIP3,STRAP,DDIT3,FAM162A,PLEKHG2,TLN1,CBLC,ATF3,GSK3B,CDC37,ITGB3,SH3GLB1,TAX1BP3,MYO1E,KPNA1,HTT,MAPK10,SH2B3,RDH11,ARFGEF3,CALM1,DISC1,PAK2,CREB3L2,INHBA,CAMK2D,ENSCAFG00000018360,HIF1AN,BTK,CDKN1A,NRP1,SH3RF3,RHBDF2,YWHAH,TNFAIP1,MADD,SEC13,KDM3A,MID1	down_VSAP_EC
GO:0030033	0.00909	0.999	3	7	microvillus assembly	BP	0.471	FSCN1,TNIK,KLF5	down_VSAP_EC
GO:0051147	0.0091	0.997	9	44	regulation of muscle cell differentiation	BP	0.407	KIT,PDGFB,MEF2C,EDN1,MTOR,BMP2,PROX1,ENSCAFG00000010596,ZEB1	up_VCAV_EC
GO:0030325	0.0091	1	1	5	adrenal gland development	BP	1	CITED2	down_VPOR_EC
GO:0110020	0.009110875	0.9987	4	31	regulation of actomyosin structure organization	BP	0.96436359	ARHGAP6,ARHGAP28,PTGER4,NRP1	up_ACOR_EC
GO:0000725	0.00912	0.997	11	58	recombinational repair	BP	0.406	RPA1,PARPBP,GINS4,RAD54L,RAD51,TONSL,BRCA1,RBBP8,SMC6,H2AFX,GEN1	down_ACOR_VSMC
GO:0010466	0.00916	1	1	32	negative regulation of peptidase activity	BP	1	CST3	down_AORT_EC
GO:0010951	0.00916	1	1	32	negative regulation of endopeptidase activity	BP	1	CST3	down_AORT_EC
GO:0030154	0.00917	0.998	7	1040	cell differentiation	BP	1	HOXC10,EYA1,HOXA9,SPINK5,EMX2,HOXA11,SHOX2	up_VFEM_VSMC
GO:0048856	0.00919	0.999	6	1533	anatomical structure development	BP	1	HOXC10,TBX15,EYA1,HOXA9,BEND2,IRX5	up_AFEM_VSMC

GO:0060421	0.00919	1	1	14	positive regulation of heart growth	BP	1	TBX5	up_APUL_VSMC
GO:0048706	0.0092	1	2	59	embryonic skeletal system development	BP	1	HOXA9,HOXC6	up_AORT_EC
GO:0033572	0.00922	1	1	5	transferrin transport	BP	1	RAB11B	down_VPOR_EC
GO:0071375	0.009228706	0.9979	6	68	cellular response to peptide hormone stimulus	BP	0.96436359	NR1H4,ENSCAFG00000004331,ACTN2,FOXC2,RARRES2,PRKCD	up_ACOR_EC
GO:0010870	0.00923	1	2	2	positive regulation of receptor biosynthetic	BP	0.409	EDN1,HIF1A	up_VCAV_EC
GO:0072359	0.00924	0.995	34	368	circulatory system development	BP	0.474	PRICKLE1,VEGFA,SULF1,MYH9,SERPINE1,SPARC,LTBP1,PTPN14,ERRFI1,JCAD,PGK1,PTPN11,TMED2,ILK,FHL2,PRCP,TGFBR1,PIK3C2A,HIF1A,THBS2,NXN,MTDH,ESM1,ACTA2,ADAMTS6,CTNNB1,STARD13,APOE,KLF5,DDIT3,NEXN,MYO1E,REST,NRP1	down_VSAP_EC
GO:0071363	0.00924	0.996	20	188	cellular response to growth factor stimulus	BP	0.474	VEGFA,SULF1,HSPA5,SPARC,JCAD,SNX25,PTPN11,ILK,TGFBR1,MYO1C,HIF1A,ZPR1,YES1,NEO1,ADAM17,CTNNB1,ENSCAFG00000002015,STRAP,ENSCAFG00000018360,NRP1	down_VSAP_EC
GO:0046631	0.00924	0.997	8	35	alpha-beta T cell activation	BP	0.409	IL6,GATA3,ARG2,MTOR,ELF4,ITPKB,HMGB1,BMX	up_VCAV_EC
GO:0060429	0.00924	0.999	4	341	epithelium development	BP	1	BARX1,NKX2-5,ANKRD6,CYP26B1	up_VPOR_VSMC
GO:0032092	0.00924	1	2	38	positive regulation of protein binding	BP	1	HOXA3,PLCL1	up_APUL_EC
GO:0030336	0.00927	0.999	3	86	negative regulation of cell migration	BP	0.779	ACVRL1,HOXA7,SULF1	down_ACOR_EC
GO:0048585	0.00928	0.994	42	475	negative regulation of response to stimulus	BP	0.474	ARHGEF2,PRICKLE1,SULF1,NR1H4,HSPA5,SERPINE1,SOCS5,PRKACB,ENPP1,ERRFI1,SNX25,HYOU1,PTPN11,FHL2,SMARCA4,SESN2,PTGS2,HIF1A,NXN,RGS2,PTPN2,DDX39B,ADAM17,CNOT7,CTNNB1,APOE,WWC3,MMP28,LYN,STRAP,DDIT3,CBLC,ATF3,GSK3B,TAX1BP3,HTT,CALM1,A2M,HIF1AN,NRP1,USP14,TNFAIP1	down_VSAP_EC
GO:2000312	0.00928	1	1	2	regulation of kainate selective glutamate	BP	0.871	NETO1	up_VSAP_VSMC
GO:1905209	0.00929	1	1	13	positive regulation of cardiocyte differentiation	BP	1	TBX5	up_APUL_VSMC
GO:0031399	0.00931	0.994	62	559	regulation of protein modification process	BP	0.409	EPHB1,TEK,HCLS1,KIT,PPP2R5A,TNFAIP3,MAP3K11,TNIP1,PDGFB,STK10,RELN,PDE4D,IWS1,ATF3,RAPGEF1,TLR4,GATA3,EDN1,RALB,APLNR,NEDD9,PELI1,YES1,MTOR,MAP3K3,BMP2,CDYL,HMG20A,HHEX,PREX2,SLK,SH3BP5,PPP1R2,ITPKB,PROX1,MADD,PAK2,PRKD2,SWAP70,TLR3,NCK1,OTUD4,NSD1,PTGS2,MMD,RAPGEF2,BCL10,TAOK1,CNOT7,HMGB1,BLM,SPTBN4,RIPK1,PDGFD,UBE2D1,KDM5A,KDM3A,ATRX,IVNS1ABP,JCAD,ARHGEF2,CDON	up_VCAV_EC
GO:0051767	0.00931	1	2	2	nitric-oxide synthase biosynthetic process	BP	0.409	TLR4,EDN1	up_VCAV_EC
GO:0051769	0.00931	1	2	2	regulation of nitric-oxide synthase biosynthetic	BP	0.409	TLR4,EDN1	up_VCAV_EC
GO:0002224	0.00931	1	2	34	toll-like receptor signaling pathway	BP	0.871	TLR2,CAV1	up_VSAP_VSMC
GO:0097305	0.00931	1	1	31	response to alcohol	BP	1	JUP	down_VFEM_VSMC
GO:0034694	0.00933	0.999	4	9	response to prostaglandin	BP	0.953	PRKAA2,ADCY6,PTGDR,PRKCE	up_ACOR_VSMC
GO:0032793	0.00934	0.999	3	6	positive regulation of CREB transcription factor	BP	0.41	RELN,PRKD2,CD200	up_VCAV_EC
GO:0030879	0.00935	1	2	34	mammary gland development	BP	0.871	HOXA9,CAV1	up_VSAP_VSMC
GO:0045823	0.00935	1	1	4	positive regulation of heart contraction	BP	1	NKX2-5	up_VPOR_VSMC
GO:0051252	0.00936	0.999	5	1051	regulation of RNA metabolic process	BP	1	TLR2,EYA1,HOXA9,HOXA10,IRX5	up_AFEM_VSMC
GO:0034341	0.00936	1	2	43	response to interferon-gamma	BP	1	MRC1,CCL24	up_VPUL_EC
GO:0060317	0.00936	1	1	11	cardiac epithelial to mesenchymal transition	BP	1	TGFBR1	down_VSAP_VSMC
GO:1904385	0.009361005	0.99968	2	6	cellular response to angiotensin	BP	0.96436359	ACTN2,PRKCD	up_ACOR_EC
GO:1990776	0.009361005	0.99968	2	6	response to angiotensin	BP	0.96436359	ACTN2,PRKCD	up_ACOR_EC
GO:0030968	0.00937	0.998	6	33	endoplasmic reticulum unfolded protein	BP	0.476	HSPA5,ERO1A,PTPN2,DDIT3,ATF3,CREB3L2	down_VSAP_EC

GO:0018335	0.00937	1	2	2	protein succinylation	BP	1	DLST,DLD	up_VSAP_EC
GO:0106077	0.00937	1	2	2	histone succinylation	BP	1	DLST,DLD	up_VSAP_EC
GO:1990144	0.00938	1	2	3	intrinsic apoptotic signaling pathway in	BP	0.476	HYOU1,BNIP3	down_VSAP_EC
GO:0008593	0.00938	1	1	33	regulation of Notch signaling pathway	BP	1	GATA5	down_VFEM_VSMC
GO:0003203	0.00938	1	1	11	endocardial cushion morphogenesis	BP	1	GATA5	down_VSAP_VSMC
GO:0034645	0.00939	0.997	13	1546	cellular macromolecule biosynthetic process	BP	0.871	HOXA11,TBX15,HOXA10,HOXA9,PITX1,EMX2,SALL1,GALNT17,IRX5,PRR16,TLR2,CAV1,SHOX2	up_VSAP_VSMC
GO:0003057	0.00939	1	1	2	regulation of the force of heart contraction by	BP	0.871	CAV1	up_VSAP_VSMC
GO:0048662	0.009397963	0.99914	3	18	negative regulation of smooth muscle cell	BP	0.96436359	NDRG2,PTGIR,CLSTN1	up_ACOR_EC
GO:0007517	0.00941	0.999	3	100	muscle organ development	BP	0.871	PITX1,CAV1,SHOX2	up_VSAP_VSMC
GO:0023051	0.009417702	0.99453	37	954	regulation of signaling	BP	0.96436359	CNTNAP4,SH3TC2,CDH3,NR1H4,BRSK1,PLCL1,SOX9,RGS1,NDRG2,KCNB1,FZD5,CCDC3,IFI6,DUSP26,DIXDC1,PTGIR,NOD2,PDE4A,SMAD6,ESM1,PAK3,TNFRSF19,ARHGDIB,ADAMTSL2,MAPK8IP2,MAVS,FIGNL1,LRRC15,LRP4,CLSTN1,SH3RF3,LRRC4,CLU,C23H3orf58,NRP1,ARHGEF26,PRKCD	up_ACOR_EC
GO:0001501	0.00943	0.995	27	178	skeletal system development	BP	1	TBX15,HOXA11,HOXA7,PEX7,HOXA9,WNT11,AXIN2,OSR2,INSIG2,PPARGC1B,IRX5,HOXD3,HOXA6,OSR1,ZBTB16,RARG,HOXC4,ZEB1,FBXW4,SULF2,SHOX2,HOXC10,NFIB,ZFAND5,BGLAP,TGFBR2,CITED2	up_VSAP_EC
GO:1902903	0.00945	0.996	19	125	regulation of supramolecular fiber organization	BP	0.411	HCLS1,ARHGEF15,BAIAP2L1,MEF2C,EDN1,ARHGAP28,TMOD1,MTOR,NCKAP1,PROX1,IQGAP2,STMN1,SWAP70,NCK1,FES,TAOK1,ARPC5L,TMOD3,ARHGEF2	up_VCAV_EC
GO:0010594	0.00945	0.997	10	71	regulation of endothelial cell migration	BP	0.476	VEGFA,SPARC,JCAD,PRCP,PIK3C2A,HIF1A,ADAM17,STARD13,APOE,NRP1	down_VSAP_EC
GO:2000172	0.00945	1	1	2	regulation of branching morphogenesis of a nerve	BP	0.871	SHOX2	up_VSAP_VSMC
GO:0030279	0.00949	0.999	3	19	negative regulation of ossification	BP	1	TWIST2,GREM1,AXIN2	up_AFEM_EC
GO:0045843	0.00949	1	1	13	negative regulation of striated muscle tissue	BP	1	TBX5	up_APUL_VSMC
GO:0016358	0.0095	0.997	9	58	dendrite development	BP	0.476	SIPA1L1,TNIK,UBA6,APOE,VLDLR,PAK2,NRP1,ARF4,YWHAH	down_VSAP_EC
GO:0046113	0.0095	1	2	2	nucleobase catabolic process	BP	1	GDA,ALDH6A1	up_VSAP_EC
GO:0003263	0.0095	1	1	4	cardioblast proliferation	BP	1	EYA1	up_VFEM_VSMC
GO:0006468	0.00952	0.994	47	544	protein phosphorylation	BP	0.476	ARHGEF2,VEGFA,SOCS5,SGK1,ENPP1,ERRFI1,JCAD,SNX25,BCAR3,NUAK1,PTPN11,TRIB3,ILK,SESN2,PTGS2,DNAJC10,TNIK,PTPN2,YES1,PLCL1,ADAM17,CNOT7,MAGED1,CTNNB1,CDK8,APOE,LYN,STRAP,CCNYL1,MMD,CBLC,ATF3,GSK3B,VLDLR,GTF2H1,HTT,MAPK10,EIF2S1,PAK2,INHBA,CAMK2D,BTK,CDKN1A,NRP1,SH3RF3,MADD,MID1	down_VSAP_EC
GO:1902041	0.00953	0.998	6	23	regulation of extrinsic apoptotic signaling pathway via death	BP	0.411	TNFAIP3,ATF3,MADD,PMAIP1,SKIL,ARHGEF2	up_VCAV_EC
GO:0032400	0.00953	0.999	3	6	melanosome localization	BP	0.411	RAB11A,RAB27A,SHROOM2	up_VCAV_EC
GO:0032401	0.00953	0.999	3	6	establishment of melanosome localization	BP	0.411	RAB11A,RAB27A,SHROOM2	up_VCAV_EC
GO:0051875	0.00953	0.999	3	6	pigment granule localization	BP	0.411	RAB11A,RAB27A,SHROOM2	up_VCAV_EC
GO:0051905	0.00953	0.999	3	6	establishment of pigment granule localization	BP	0.411	RAB11A,RAB27A,SHROOM2	up_VCAV_EC
GO:0048645	0.00954	1	1	33	animal organ formation	BP	1	GATA5	down_VFEM_VSMC
GO:0001501	0.00956	0.997	10	178	skeletal system development	BP	1	TBX15,HOXA9,IRX5,HOXC10,XYLT1,HOXA7,GREM1,HOXA6,PTHLH,AXIN2	up_AFEM_EC
GO:0043009	0.00956	1	2	227	chordate embryonic development	BP	1	HOXA3,HOXB5	up_APUL_VSMC
GO:0051606	0.00956	1	1	33	detection of stimulus	BP	1	JUP	down_VFEM_VSMC
GO:0010611	0.00956	1	1	11	regulation of cardiac muscle hypertrophy	BP	1	GATA5	down_VSAP_VSMC
GO:0014743	0.00956	1	1	11	regulation of muscle hypertrophy	BP	1	GATA5	down_VSAP_VSMC

GO:0090131	0.00957	1	2	3	mesenchyme migration	BP	0.476	ACTG2,ACTA2	down_VSAP_EC
GO:0051563	0.00958	1	2	2	smooth endoplasmic reticulum calcium ion	BP	0.966	APP,PSEN1	up_ACOR_VSMC
GO:1905908	0.00958	1	2	2	positive regulation of amyloid fibril formation	BP	0.966	APP,PSEN1	up_ACOR_VSMC
GO:0007409	0.00958	1	2	109	axonogenesis	BP	1	SLITRK5,LIMK1	up_VCAV_VSMC
GO:0060043	0.00958	1	1	14	regulation of cardiac muscle cell proliferation	BP	1	TBX5	up_APUL_VSMC
GO:0110110	0.00959	1	2	34	positive regulation of animal organ	BP	0.841	HOXB7,SIX1	up_VFEM_EC
GO:0060914	0.00959	1	1	11	heart formation	BP	1	GATA5	down_VSAP_VSMC
GO:1904467	0.0096	1	1	8	regulation of tumor necrosis factor secretion	BP	0.856	ARG2	up_AORT_VSMC
GO:0018195	0.009604774	0.99966	2	6	peptidyl-arginine modification	BP	0.96436359	NR1H4,RIOX2	up_ACOR_EC
GO:0001568	0.00961	0.995	25	252	blood vessel development	BP	0.476	PRICKLE1,VEGFA,SULF1,MYH9,SERPINE1,SPARC,LTBP1,JCAD,PGK1,TMED2,PRCP,PIK3C2A,HIF1A,THBS2,MTDH,ESM1,ACTA2,ADAMTS6,CTNNB1,STARD13,APOE,KLF5,DDIT3,MYO1E,NRP1	down_VSAP_EC
GO:0000904	0.00961	0.995	20	181	cell morphogenesis involved in differentiation	BP	0.476	SIPA1L1,MYH9,ACTN1,PTPN11,FLNB,IFRD1,ILK,WDR1,KIF5B,TNIK,NEO1,UST,CDH11,CTNNB1,ENSCAFG00000002015,PLOD3,VLDLR,EXT1,NRP1,YWHAH	down_VSAP_EC
GO:0003430	0.00961	1	2	2	growth plate cartilage chondrocyte growth	BP	1	RARG,TGFBR2	up_VSAP_EC
GO:1904589	0.00963	1	1	33	regulation of protein import	BP	1	JUP	down_VFEM_VSMC
GO:0007369	0.00965	1	2	63	gastrulation	BP	1	EYA1,HOXA11	up_VFEM_VSMC
GO:0030335	0.00966	0.999	4	165	positive regulation of cell migration	BP	0.801	LYN,IGF1R,PDPN,STX4	down_ACOR_EC
GO:0034764	0.009668803	0.99815	5	50	positive regulation of transmembrane transport	BP	0.96436359	NR1H4,ENSCAFG00000004331,ACTN2,RARRES2,AZIN2	up_ACOR_EC
GO:0002218	0.00968	0.997	10	53	activation of innate immune response	BP	0.417	TNFAIP3,TNIP1,TMEM173,TLR4,PELI1,RIOK3,TLR3,OTUD4,HMGB1,LSM14A	up_VCAV_EC
GO:0002191	0.00968	1	2	2	cap-dependent translational initiation	BP	1	EIF3D,NCK1	up_VSAP_EC
GO:0006538	0.00968	1	2	2	glutamate catabolic process	BP	1	GOT1,ADHFE1	up_VSAP_EC
GO:0031646	0.00968	1	1	7	positive regulation of neurological system	BP	0.859	WNT7A	up_AORT_VSMC
GO:0001952	0.0097	1	1	34	regulation of cell-matrix adhesion	BP	1	JUP	down_VFEM_VSMC
GO:0042126	0.00971	1	2	2	nitrate metabolic process	BP	1	SUOX,MARC1	up_VSAP_EC
GO:0030198	0.00972	0.999	3	81	extracellular matrix organization	BP	0.801	PDPN,SULF1,CSGALNACT1	down_ACOR_EC
GO:0110110	0.00972	1	1	34	positive regulation of animal organ	BP	1	GATA5	down_VFEM_VSMC
GO:1900182	0.00973	1	1	33	positive regulation of protein localization to	BP	1	JUP	down_VFEM_VSMC
GO:0097191	0.00974	0.996	13	79	extrinsic apoptotic signaling pathway	BP	0.417	TNFAIP3,ATF3,CASP8,MADD,PAK2,TLR3,BCL10,CTNNA1,PMAIP1,RIPK1,ITGA6,SKIL,ARHGEF2	up_VCAV_EC
GO:0090287	0.00975	0.996	12	91	regulation of cellular response to growth factor	BP	0.478	VEGFA,SULF1,HSPA5,JCAD,SNX25,ILK,MYO1C,HIF1A,NEO1,ADAM17,CTNNB1,STRAP	down_VSAP_EC
GO:0071345	0.00976	0.995	27	210	cellular response to cytokine stimulus	BP	0.417	ACKR1,HCLS1,GBP5,KIT,CXCL8,LAPTM5,CXCR4,PDGFB,TMEM173,NLRC5,GATA3,IRF8,ST3GAL6,MAPK13,IFNGR1,FLNB,OTUD4,CREB1,CNOT7,ADIPOR2,RIPK1,LSM14A,HIF1A,KDM3A,ENSCAFG00000002015,ARHGEF2,TJP2	up_VCAV_EC
GO:1900426	0.00976	1	2	2	positive regulation of defense response to	BP	1	F2RL1,CYBA	up_VSAP_EC
GO:0043309	0.00976	1	2	2	regulation of eosinophil degranulation	BP	1	F2RL1,STX4	up_VSAP_EC
GO:0043311	0.00976	1	2	2	positive regulation of eosinophil degranulation	BP	1	F2RL1,STX4	up_VSAP_EC
GO:1902566	0.00976	1	2	2	regulation of eosinophil activation	BP	1	F2RL1,STX4	up_VSAP_EC
GO:1902568	0.00976	1	2	2	positive regulation of eosinophil activation	BP	1	F2RL1,STX4	up_VSAP_EC

GO:0048666	0.00977	0.995	26	258	neuron development	BP	0.478	SIPA1L1,SGK1,PTPN11,DPYSL3,IFRD1,KIF5B,TNIK,UBA6,ZPR1,NEO1,UST,CDH11,CTNNB1,APOE,LYN,GSK3B,VLDLR,SDC4,MAPK10,PRMT1,EXT1,DISC1,PAK2,NRP1,ARF4,YWHAH	down_VSAP_EC
GO:0008277	0.00977	0.998	7	31	regulation of G protein-coupled receptor signaling	BP	0.417	CXCL8,EDN1,APLNR,ACPP,CAV2,STMN1,RGS2	up_VCAV_EC
GO:0045667	0.00977	0.998	6	38	regulation of osteoblast differentiation	BP	1	BMPR1A,CLSTN1,GLI3,TWIST2,DDR2,GREM1	down_VCAV_EC
GO:0019933	0.009805067	0.99857	4	32	cAMP-mediated signaling	BP	0.96436359	SOX9,PTH1R,PTGIR,PDE4A	up_ACOR_EC
GO:0033135	0.00982	1	2	40	regulation of peptidyl-serine phosphorylation	BP	1	PLCL1,NTRK2	up_APUL_EC
GO:2000650	0.00982	1	1	2	negative regulation of sodium ion transmembrane	BP	0.801	NEDD4	down_ACOR_EC
GO:0032640	0.00983	1	2	39	tumor necrosis factor production	BP	1	NR1H4,BPI	up_APUL_EC
GO:0050793	0.00985	0.998	7	731	regulation of developmental process	BP	1	TBX5,NR1H4,EVI2B,WIF1,CCL24,FLRT3,ARHGEF2	up_VPUL_EC
GO:0042118	0.00985	1	1	4	endothelial cell activation	BP	1	HOXA9	up_AORT_EC
GO:0046622	0.00985	1	1	15	positive regulation of organ growth	BP	1	TBX5	up_APUL_VSMC
GO:0016070	0.00986	0.993	148	1479	RNA metabolic process	BP	0.436	ELK3,HMGB1,RNPS1,SREBF1,IRAK1,PTBP1,ILF3,DDX39A,CBX5,PPRC1,USP4,SF3B2,ATAD2,HMGA1,EIF4A3,HNRNPUL1,EDC3,HNRNPD,SREBF2,HINFP,VARS,NEDD4,SRSF2,MED17,U2AF2,INTS7,POLA1,MTDH,HEY1,SNRPB2,SF1,SRRT,NOP56,POLE,DDX23,NAB2,CDCA7L,FUS,BIRC5,MYBBP1A,INSIG1,PRMT1,CAV1,U2AF1,TIMELESS,THRAP3,TRUB2,FAM129B,TCEA1,EDC4,AURKB,NONO,HNRNPL,EZH2,GREM1,FBL,FEN1,SMARCC1,RNASEH2B,PLK3,SFSWAP,PCNA,DHX33,HOXA7,SMG9,SF3B4,PHF5A,UHRF1,CKS2,DIS3,GMNN,BRCA1,NOC4L,WDR62,FASTKD3,NOC2L,E2F8,FARSA,DDX51,ENSCAFG00000015065,PPARD,YBX3,HNRNPF,NCL,NUP62,DDX28,FOSL1,SUV39H1,RSL1D1,THUMPD2,RRP1,ZNF367,CACTIN,HMGB2,DMAP1,DHX15,CDK2,SMARCB1,DDX39B,CCNA2,ENSCAFG00000005299,EZR,SF3A2,DHX38,RBM14,RBM17,OSGEP,POLR3F,HOXA10,ERF,ENSCAFG00000019620,TCERG1,RARS,METTL1,SMARCAL1,NOP14,TBX15,ELF4,PRMT5,PSMC1,CTH,NOP58,ARHGEF2,ENSCAFG00000015189,SUPT16H,TADA2A,WTAP,HAT1,HIST2H2AC,HOXA9,GLI1,JMJD6,H2AFX,WDR33,ENSCAFG00000003222,CDC5L,EMG1,RNASEH2C,RTCB,EXOSC5,GEMIN7,HMGN1,SIX4,SF3A1,MED16	down_ACOR_VSMC
GO:0051240	0.00986	0.993	63	489	positive regulation of multicellular organismal process	BP	0.971	ADRA1B,VEGFD,TLR1,PLCG2,LEP,LBH,GATA5,CD83,HTR2B,MBP,FERMT1,BMP4,FGFR2,TP53BP1,GJA1,HRH1,TLR3,WASF3,PPARGC1A,PDE5A,CITED2,ISG15,CD274,ZNF365,SASH1,FGF18,FAM210B,PLAC8B,F2RL1,HRH2,ENPP4,CLSTN1,NRP1,PTPN22,NPNT,SMARCD3,SLITRK5,TBX20,TBX5,PRKCE,FZD3,FRS2,DAG1,APP,PSEN1,CHD7,JUP,DDR2,EDN1,NOD2,B2M,DISC1,SCAMP5,MAP3K7,INPP5K,DOCK5,ERAP1,PARK7,SLC8A1,IL18R1,RREB1,CRKL,PIK3C2A	up_ACOR_VSMC
GO:1901214	0.00986	0.996	11	81	regulation of neuron death	BP	0.481	HYOU1,AARS,HIF1A,ZPR1,CTNNB1,APOE,DDIT3,GSK3B,EIF2S1,SIX4,NRP1	down_VSAP_EC
GO:1905939	0.0099	1	1	5	regulation of gonad development	BP	1	CITED2	down_VPOR_EC
GO:0045637	0.00991	0.997	9	73	regulation of myeloid cell differentiation	BP	1	NOTCH2,FBN1,TFE3,CLSTN1,ZFP36L1,RAB7B,FOXO3,ZBTB16,ANKRD54	down_VCAV_EC
GO:0007100	0.00991	0.999	3	6	mitotic centrosome separation	BP	0.436	NEK2,NUP62,KIF11	down_ACOR_VSMC
GO:0009792	0.00991	1	2	231	embryo development ending in birth or egg	BP	1	HOXA3,HOXB5	up_APUL_VSMC
GO:0010804	0.009918494	0.99965	2	6	negative regulation of tumor necrosis factor-mediated signaling	BP	0.96436359	NR1H4,CCDC3	up_ACOR_EC
GO:0050706	0.00992	1	1	9	regulation of interleukin-1 beta secretion	BP	0.875	ARG2	up_AORT_VSMC
GO:1903706	0.00993	0.995	18	119	regulation of hemopoiesis	BP	0.422	HCLS1,IL7R,MEF2C,GATA3,HOXB8,CASP8,MTOR,ITPKB,FES,JAG1,CREB1,HMGB1,RIPK1,ATP11C,CUL4A,HIF1A,ZEB1,TJP2	up_VCAV_EC

GO:0045935	0.00994	0.993	57	491	positive regulation of nucleobase-containing compound metabolic process	BP	0.422	HCLS1,PPARG,TNIP1,PDGFB,TMEM173,MEF2C,ATF3,CHD7,TLR4,NLRC5,GATA3,EDN1,TCF4,FOXN2,ENSCAFG00000017103,IRF8,PPP3CA,YES1,MTOR,BMP2,HHEX,CREM,CNOT6,PROX1,ARID4B,HMBOX1,PRKD2,THOC1,SMARCA4,TLR3,NCK1,TIMELESS,OTUD4,LDB2,JAG1,NSD1,BCL10,CNOT7,HMGB1,AHR,NOS2,BLM,RIPK1,ITGA6,GPBP1,MTA2,NR1D2,KDM5A,HIF1A,KDM3A,ATRX,NR4A1,ZEB1,TET2,ARHGEF2,CDON,ZNF609	up_VCAV_EC
GO:0001656	0.00995	1	2	35	metanephros development	BP	0.898	HOXA11,SALL1	up_VSAP_VSMC
GO:0006468	0.00996	0.993	61	544	protein phosphorylation	BP	0.422	EPHB1,PLK2,TEK,HCLS1,KIT,PRKCH,TNFAIP3,MAP3K11,TNIP1,PDGFB,STK10,SH2D3C,RELN,PDE4D,MEF2C,ATF3,RAPGEF1,TLR4,EDN1,RALB,APLNR,NEDD9,YES1,EPHB4,MTOR,MAP3K3,BMP2,HHEX,PREX2,SLK,BMP2K,SH3BP5,ERC1,MAPK13,ITPKB,PROX1,ENSCAFG00000010596,MADD,PAK2,PRKD2,TLR3,NCK1,FES,NSD1,PTGS2,MMD,TOP1,RAPGEF2,CREB1,TAOK1,CNOT7,HMGB1,BLM,SPTBN4,RIPK1,PDGFD,BMX,LMTK2,JCAD,ARHGEF2,CDON	up_VCAV_EC
GO:0019725	0.00996	0.994	35	245	cellular homeostasis	BP	0.971	RHOT1,ADRA1B,PLCG2,SLC26A7,HTR2B,PRDX4,PRKAA2,CLCN3,MICU2,TMX3,ITPR1,F2RL1,ERO1B,EGLN1,SLC12A7,LRRC8C,DMPK,ATP7A,ORMDL3,PRKCE,APP,PSEN1,CHD7,LAMP2,DMXL1,RAB11B,EDN1,DISC1,HERPUD1,TXNDC5,GAA,WFS1,ATP2C1,SLC8A1,STC2	up_ACOR_VSMC
GO:0060708	0.00996	1	1	2	spongiotrophoblast differentiation	BP	0.898	GJB5	up_VSAP_VSMC
GO:0045333	0.00997	1	2	74	cellular respiration	BP	1	SUCLG2,NDUFA10	down_AFEM_EC
GO:0000028	0.00998	0.999	3	9	ribosomal small subunit assembly	BP	1	RPS10,MRPS11,RPS27	down_VCAV_EC
GO:0030878	0.00998	1	1	13	thyroid gland development	BP	1	HOXA3	up_APUL_VSMC
GO:0071605	0.00998	1	1	3	monocyte chemotactic protein-1 production	BP	1	NR1H4	up_VPUL_EC
GO:0071637	0.00998	1	1	3	regulation of monocyte chemotactic protein-1	BP	1	NR1H4	up_VPUL_EC
GO:0071638	0.00998	1	1	3	negative regulation of monocyte chemotactic	BP	1	NR1H4	up_VPUL_EC
GO:0019934	0.009981409	0.99964	2	6	cGMP-mediated signaling	BP	0.96436359	AQP1,GUCY1B1	up_ACOR_EC
GO:0002689	0.00999	1	1	5	negative regulation of leukocyte chemotaxis	BP	1	SLIT2	up_VPUL_VSMC
GO:0033627	0.01	0.998	5	22	cell adhesion mediated by integrin	BP	0.487	SERPINE1,ITGA2,PTPN11,ADAM17,LYN	down_VSAP_EC
GO:0002374	0.01	1	1	7	cytokine secretion involved in immune	BP	1	TLR2	up_AFEM_VSMC
GO:0048635	0.01	1	1	14	negative regulation of muscle organ	BP	1	TBX5	up_APUL_VSMC
GO:0060017	0.01	1	1	3	parathyroid gland development	BP	1	HOXA3	up_VPUL_EC
GO:0010614	0.01	1	1	5	negative regulation of cardiac muscle	BP	1	GATA5	up_VPUL_VSMC
GO:0014741	0.01	1	1	5	negative regulation of muscle hypertrophy	BP	1	GATA5	up_VPUL_VSMC
GO:0055026	0.01	1	1	5	negative regulation of cardiac muscle tissue	BP	1	TBX5	up_VPUL_VSMC
GO:0035295	0.0101	0.994	28	356	tube development	BP	1	ADAMTS2,HSPB6,PDGFRB,SMAD9,DCN,ANGPT1,SDC4,BMPR1A,LOX,LMO4,ANPEP,CLSTN1,SEMA3C,THY1,B4GALT1,GLI3,ZFP36L1,MYDGF,SASH1,IFT57,CDH2,LOXL3,SPRED1,SHC1,RDH10,GREM1,CLIC4,THRB	down_VCAV_EC
GO:0006066	0.0101	0.996	16	89	alcohol metabolic process	BP	1	NAPEPLD,LIPC,INSIG2,GOT1,INPP5E,LMF1,PPIP5K1,GALK1,RETSAT,LCAT,PCBD1,TKFC,MVK,LIPE,CYB5R3,SOAT1	up_VSAP_EC
GO:0051252	0.0101	0.997	10	1051	regulation of RNA metabolic process	BP	0.898	HOXA11,HOXA10,HOXA9,PITX1,EMX2,SALL1,IRX5,TLR2,CAV1,SHOX2	up_VSAP_VSMC
GO:0043473	0.0101	0.998	7	28	pigmentation	BP	0.423	EDNRB,KIT,ADAMTS9,MEF2C,RAB11A,RAB27A,SHROOM2	up_VCAV_EC
GO:1901224	0.0101	0.998	6	20	positive regulation of NIK/NF-kappaB signaling	BP	0.971	TLR3,SASH1,APP,NOD2,MAP3K7,IL18R1	up_ACOR_VSMC
GO:0048002	0.0101	0.999	4	10	antigen processing and presentation of peptide	BP	0.971	DLA88,B2M,ERAP1,TAPBPL	up_ACOR_VSMC
GO:0009617	0.0101	0.999	3	133	response to bacterium	BP	1	NR1H4,RGS1,MRC1	up_VPUL_EC

GO:0034644	0.0101	1	2	32	cellular response to UV	BP	0.801	USP28,NEDD4	down_ACOR_EC
GO:0048645	0.0101	1	2	33	animal organ formation	BP	0.801	HOXA3,SULF1	down_ACOR_EC
GO:0048736	0.0101	1	2	66	appendage development	BP	1	HOXC10,HOXA9	up_AORT_EC
GO:0060173	0.0101	1	2	66	limb development	BP	1	HOXC10,HOXA9	up_AORT_EC
GO:0042742	0.0101	1	2	39	defense response to bacterium	BP	1	NR1H4,BPI	up_APUL_EC
GO:0009952	0.0101	1	2	66	anterior/posterior pattern specification	BP	1	HOXA3,BARX1	up_VPOR_VSMC
GO:0002278	0.0101	1	1	3	eosinophil activation involved in immune	BP	0.801	STX4	down_ACOR_EC
GO:0002447	0.0101	1	1	3	eosinophil mediated immunity	BP	0.801	STX4	down_ACOR_EC
GO:0015884	0.0101	1	1	3	folic acid transport	BP	0.801	PDPN	down_ACOR_EC
GO:0043307	0.0101	1	1	3	eosinophil activation	BP	0.801	STX4	down_ACOR_EC
GO:0043308	0.0101	1	1	3	eosinophil degranulation	BP	0.801	STX4	down_ACOR_EC
GO:0046697	0.0101	1	1	5	decidualization	BP	1	CITED2	down_VPOR_EC
GO:0034134	0.0101	1	1	7	toll-like receptor 2 signaling pathway	BP	1	TLR2	up_AFEM_VSMC
GO:2000136	0.0101	1	1	7	regulation of cell proliferation involved in	BP	1	EYA1	up_AFEM_VSMC
GO:0021522	0.0101	1	1	8	spinal cord motor neuron differentiation	BP	0.883	HOXC10	up_AORT_VSMC
GO:0062009	0.0101	1	1	8	secondary palate development	BP	0.885	WNT7A	up_AORT_VSMC
GO:1901862	0.0101	1	1	14	negative regulation of muscle tissue	BP	1	TBX5	up_APUL_VSMC
GO:2000826	0.0101	1	1	14	regulation of heart morphogenesis	BP	1	TBX5	up_APUL_VSMC
GO:1901213	0.0101	1	1	7	regulation of transcription from RNA polymerase II promoter involved in	BP	1	GATA5	up_VCAV_VSMC
GO:0010759	0.0101	1	1	5	positive regulation of macrophage chemotaxis	BP	1	C3AR1	up_VPOR_VSMC
GO:0019627	0.0101	1	1	3	urea metabolic process	BP	1	NR1H4	up_VPUL_EC
GO:0072677	0.0101	1	1	3	eosinophil migration	BP	1	CCL24	up_VPUL_EC
GO:0003207	0.0101	1	1	5	cardiac chamber formation	BP	1	TBX5	up_VPUL_VSMC
GO:0060353	0.0101	1	1	2	regulation of cell adhesion molecule production	BP	0.898	CAV1	up_VSAP_VSMC
GO:0060355	0.0101	1	1	2	positive regulation of cell adhesion molecule	BP	0.898	CAV1	up_VSAP_VSMC
GO:0009719	0.0102	0.993	53	395	response to endogenous stimulus	BP	0.971	SMAD6,ACTN2,LEP,LBH,GATA5,SKP2,PRKAA2,FERMT1,BMP4,FGFR2,ABCA1,MED14,CASTOR1,LATS2,CITED2,RERG,AR,TMEM108,FGF18,FAM210B,CDKN2B,ADCY6,ANKRD1,MXRA5,CTSB,AKAP6,CREBRF,UBR1,PTPN22,NPNT,APLP1,PTGDR,SESN3,PRKCE,FRS2,AGRN,FUT8,APP,PSEN1,TGFBR1,JUP,NR3C1,DUSP22,HPGD,NOD2,FAM107A,DDIT4,ENSCAFG00000018360,GNAQ,INPP5K,SH3BP4,PARK7,CRKL	up_ACOR_VSMC
GO:0070997	0.0102	0.996	12	93	neuron death	BP	0.492	HYOU1,AARS,HIF1A,ZPR1,CTNNB1,APOE,DDIT3,FAM162A,GSK3B,EIF2S1,SIX4,NRP1	down_VSAP_EC
GO:0010810	0.0102	0.996	12	60	regulation of cell-substrate adhesion	BP	0.971	FERMT1,NRP1,NPNT,PRKCE,ECM2,JUP,DUSP22,NID1,FAM107A,DOCK5,RREB1,CRKL	up_ACOR_VSMC
GO:0045840	0.0102	0.999	4	12	positive regulation of mitotic nuclear division	BP	0.443	BIRC5,NUP62,ENSCAFG00000005299,UBE2C	down_ACOR_VSMC
GO:0030111	0.0102	0.999	3	108	regulation of Wnt signaling pathway	BP	0.898	SALL1,TLR2,CAV1	up_VSAP_VSMC
GO:0071363	0.0102	1	2	188	cellular response to growth factor stimulus	BP	1	GATA5,TGFBR1	down_VSAP_VSMC
GO:0097709	0.0102	1	2	2	connective tissue replacement	BP	0.427	PPP3CA,HIF1A	up_VCAV_EC
GO:0006941	0.0102	1	1	36	striated muscle contraction	BP	1	JUP	down_VFEM_VSMC
GO:0061323	0.0102	1	1	7	cell proliferation involved in heart morphogenesis	BP	1	EYA1	up_AFEM_VSMC
GO:0002828	0.0102	1	1	9	regulation of type 2 immune response	BP	0.888	ARG2	up_AORT_VSMC

GO:0003180	0.0102	1	1	7	aortic valve morphogenesis	BP	1	GATA5	up_VCAV_VSMC
GO:0010765	0.0102	1	1	5	positive regulation of sodium ion transport	BP	1	NKX2-5	up_VPOR_VSMC
GO:2000463	0.0102	1	1	5	positive regulation of excitatory postsynaptic	BP	1	NETO1	up_VFEM_VSMC
GO:0018216	0.0102	1	1	3	peptidyl-arginine methylation	BP	1	NR1H4	up_VPUL_EC
GO:0051891	0.0102	1	1	3	positive regulation of cardioblast differentiation	BP	1	TBX5	up_VPUL_EC
GO:1904468	0.0102	1	1	3	negative regulation of tumor necrosis factor	BP	1	NR1H4	up_VPUL_EC
GO:0007616	0.0102	1	1	5	long-term memory	BP	1	LRRN4	up_VPUL_VSMC
GO:0055062	0.0102	1	1	5	phosphate ion homeostasis	BP	1	HOXA3	up_VPUL_VSMC
GO:0003264	0.0102	1	1	5	regulation of cardioblast proliferation	BP	1	TBX5	up_VPUL_VSMC
GO:0003266	0.0102	1	1	5	regulation of secondary heart field cardioblast	BP	1	TBX5	up_VPUL_VSMC
GO:0072505	0.0102	1	1	5	divalent inorganic anion homeostasis	BP	1	HOXA3	up_VPUL_VSMC
GO:0072506	0.0102	1	1	5	trivalent inorganic anion homeostasis	BP	1	HOXA3	up_VPUL_VSMC
GO:0048548	0.0102	1	1	2	regulation of pinocytosis	BP	0.898	CAV1	up_VSAP_VSMC
GO:0050716	0.010261442	0.99963	2	6	positive regulation of interleukin-1 secretion	BP	0.96436359	FZD5,NOD2	up_ACOR_EC
GO:0051239	0.0103	0.993	103	859	regulation of multicellular organismal process	BP	0.971	TNNT2,FGF13,ADRA1B,VEGFD,TMEM176B,TLR1,TMEM176A,SMAD6,PLCG2,LEP,PLCL1,LBH,KCNE3,GATA5,CD83,HTR2B,RASSF2,MBP,PRDX4,MLLT3,ITM2C,FERMT1,BMP4,LCAT,LNPK,FGFR2,TP53BP1,GJA1,ELF1,HRH1,TLR3,SEC24B,WASF3,PPARGC1A,LATS2,PDE5A,CITED2,ISG15,ZNRF3,CD274,ZNF365,SASH1,TMEM108,FGF18,FAM210B,NDRG2,ATRN,P4HTM,PLAC8B,LIMS2,F2RL1,HRH2,ENPP4,EGLN1,CLSTN1,NRP1,PTPN22,LRPAP1,ULK2,DMPK,NPNT,SMARCD3,EVL,SLITRK5,TBX20,TBX5,PRKCE,FZD3,FRS2,RNLS,DAG1,APP,LAMA3,PSEN1,TGFBR1,SYNJ2BP,CHD7,JUP,SIPA1L1,DDR2,WWC2,SSH3,EDN1,NOD2,ASAP1,B2M,DISC1,SCAMP5,TNFRSF11B,GAA,MAP3K7,INPP5K,CNOT4,DOCK5,ERAP1,PARK7,SLC8A1,STC2,IL18R1,BVES,RREB1,CRKL,PIK3C2A	up_ACOR_VSMC
GO:0051188	0.0103	0.996	15	81	cofactor biosynthetic process	BP	1	ACACB,NADK,ENSCAFG00000013986,COQ9,PDK2,GALK1,MOCOS,PCBD1,ACSS2,PNPO,DCAKD,CPOX,PNP,ZNF205,DDIT4	up_VSAP_EC
GO:0002683	0.0103	0.996	13	126	negative regulation of immune system process	BP	1	GPAM,PRNP,SDC4,FBN1,THY1,GLI3,ZFP36L1,BPI,LOXL3,RAB7B,A2M,TSC22D3,ZBTB16	down_VCAV_EC
GO:0050663	0.0103	0.996	11	52	cytokine secretion	BP	0.971	TLR1,LEP,HTR2B,MBP,FERMT1,ABCA1,CD274,F2RL1,PTPN22,NOD2,SCAMP5	up_ACOR_VSMC
GO:2000026	0.0103	0.998	5	560	regulation of multicellular organismal development	BP	1	NKX2-5,BAMBI,ANKRD6,CYP26B1,C3AR1	up_VPOR_VSMC
GO:2000147	0.0103	0.998	4	169	positive regulation of cell motility	BP	0.801	LYN,IGF1R,PDPN,STX4	down_ACOR_EC
GO:0051057	0.0103	0.999	4	11	positive regulation of small GTPase mediated signal transduction	BP	0.427	RELN,GPR4,ITPKB,CDON	up_VCAV_EC
GO:0045597	0.0103	0.999	4	253	positive regulation of cell differentiation	BP	1	TBX5,EVI2B,WIF1,ARHGEF2	up_VPUL_EC
GO:0071679	0.0103	0.999	3	5	commissural neuron axon guidance	BP	0.971	NRP1,FZD3,DAG1	up_ACOR_VSMC
GO:0014706	0.0103	1	2	112	striated muscle tissue development	BP	1	EYA1,BEND2	up_AFEM_VSMC
GO:0071706	0.0103	1	2	40	tumor necrosis factor superfamily cytokine	BP	1	NR1H4,BPI	up_APUL_EC
GO:0033632	0.0103	1	2	2	regulation of cell-cell adhesion mediated by	BP	0.427	PODXL,SWAP70	up_VCAV_EC
GO:0051962	0.0103	1	2	112	positive regulation of nervous system	BP	1	SLITRK5,LIMK1	up_VCAV_VSMC
GO:0021545	0.0103	1	2	21	cranial nerve development	BP	1	HOXA3,SEMA3A	up_VPOR_EC
GO:0035089	0.0103	1	1	5	establishment of apical/basal cell polarity	BP	1	FSCN1	down_AFEM_EC
GO:1905314	0.0103	1	1	12	semi-lunar valve development	BP	1	GATA5	down_VSAP_VSMC
GO:0001710	0.0103	1	1	7	mesodermal cell fate commitment	BP	1	EYA1	up_AFEM_VSMC

GO:0055021	0.0103	1	1	15	regulation of cardiac muscle tissue growth	BP	1	TBX5	up_APUL_VSMC
GO:0003209	0.0103	1	1	14	cardiac atrium morphogenesis	BP	1	TBX5	up_APUL_VSMC
GO:0003230	0.0103	1	1	14	cardiac atrium development	BP	1	TBX5	up_APUL_VSMC
GO:0048384	0.0103	1	1	4	retinoic acid receptor signaling pathway	BP	1	CYP26B1	up_VPOR_VSMC
GO:0048385	0.0103	1	1	4	regulation of retinoic acid receptor signaling	BP	1	CYP26B1	up_VPOR_VSMC
GO:0003214	0.0103	1	1	3	cardiac left ventricle morphogenesis	BP	1	TBX5	up_VPUL_EC
GO:0031033	0.0103	1	1	3	myosin filament organization	BP	1	MYBPC2	up_VPUL_EC
GO:0033240	0.0103	1	1	3	positive regulation of cellular amine metabolic	BP	1	NR1H4	up_VPUL_EC
GO:0072513	0.0103	1	1	3	positive regulation of secondary heart field cardioblast proliferation	BP	1	TBX5	up_VPUL_EC
GO:1900122	0.0103	1	1	3	positive regulation of receptor binding	BP	1	HOXA3	up_VPUL_EC
GO:0048742	0.0103	1	1	2	regulation of skeletal muscle fiber development	BP	0.898	SHOX2	up_VSAP_VSMC
GO:0060056	0.0103	1	1	2	mammary gland involution	BP	0.898	CAV1	up_VSAP_VSMC
GO:0009059	0.0104	0.996	13	1563	macromolecule biosynthetic process	BP	0.898	HOXA11,TBX15,HOXA10,HOXA9,PITX1,EMX2,SALL1,GALNT17,IRX5,PRR16,TLR2,CAV1,SHOX2	up_VSAP_VSMC
GO:0034645	0.0104	0.998	6	1546	cellular macromolecule biosynthetic process	BP	1	TLR2,TBX15,EYA1,HOXA9,HOXA10,IRX5	up_AFEM_VSMC
GO:2000026	0.0104	0.998	6	560	regulation of multicellular organismal development	BP	1	TBX5,NR1H4,EVI2B,CCL24,FLRT3,ARHGEF2	up_VPUL_EC
GO:0090050	0.0104	0.999	4	15	positive regulation of cell migration involved in sprouting angiogenesis	BP	0.504	VEGFA,JCAD,PIK3C2A,NRP1	down_VSAP_EC
GO:0031347	0.0104	1	2	144	regulation of defense response	BP	0.9	ARG2,ESR1	up_AORT_VSMC
GO:0070428	0.0104	1	2	2	regulation of nucleotide-binding oligomerization domain containing 1	BP	0.43	TNFAIP3,TLR4	up_VCAV_EC
GO:2001013	0.0104	1	2	2	epithelial cell proliferation involved in renal tubule morphogenesis	BP	0.43	MEF2C,MTSS1	up_VCAV_EC
GO:0072088	0.0104	1	2	36	nephron epithelium morphogenesis	BP	0.898	HOXA11,SALL1	up_VSAP_VSMC
GO:2000027	0.0104	1	1	72	regulation of animal organ morphogenesis	BP	1	GATA5	down_AFEM_VSMC
GO:0007530	0.0104	1	1	5	sex determination	BP	1	CITED2	down_VPOR_EC
GO:0032402	0.0104	1	1	5	melanosome transport	BP	1	RAB11B	down_VPOR_EC
GO:0051904	0.0104	1	1	5	pigment granule transport	BP	1	RAB11B	down_VPOR_EC
GO:0042118	0.0104	1	1	4	endothelial cell activation	BP	1	HOXA9	up_VFEM_VSMC
GO:0000820	0.0104	1	1	3	regulation of glutamine family amino acid	BP	1	NR1H4	up_VPUL_EC
GO:1902667	0.0104	1	1	5	regulation of axon guidance	BP	1	SLIT2	up_VPUL_VSMC
GO:0072006	0.0105	0.997	9	59	nephron development	BP	0.506	SULF1,ENSCAFG00000032470,ILK,HOXB7,ACTA2,MAGED1,CTNNB1,MYO1E,SIX4	down_VSAP_EC
GO:0055002	0.0105	0.997	9	45	striated muscle cell development	BP	0.432	MEF2C,EDN1,TMOD1,PPP3CA,MTOR,CAV2,PROX1,P2RX2,TMOD3	up_VCAV_EC
GO:1901222	0.0105	0.997	8	32	regulation of NIK/NF-kappaB signaling	BP	0.974	RASSF2,TLR3,SASH1,PTPN22,APP,NOD2,MAP3K7,IL18R1	up_ACOR_VSMC
GO:0051272	0.0105	0.998	4	170	positive regulation of cellular component	BP	0.801	LYN,IGF1R,PDPN,STX4	down_ACOR_EC
GO:0034123	0.0105	0.999	4	10	positive regulation of toll-like receptor signaling	BP	0.974	TLR1,TLR3,F2RL1,PTPN22	up_ACOR_VSMC
GO:0009653	0.0105	0.999	3	773	anatomical structure morphogenesis	BP	1	HOXA3,TBX5,HOXB5	up_APUL_VSMC
GO:0060501	0.0105	1	1	5	positive regulation of epithelial cell proliferation involved in lung	BP	1	SRSF6	down_AFEM_EC
GO:0030259	0.0105	1	1	5	lipid glycosylation	BP	1	GBGT1	down_VPOR_EC
GO:0010811	0.0105	1	1	36	positive regulation of cell-substrate adhesion	BP	1	JUP	down_VFEM_VSMC
GO:0021615	0.0105	1	1	3	glossopharyngeal nerve morphogenesis	BP	1	HOXA3	up_APUL_EC

GO:0051890	0.0105	1	1	4	regulation of cardioblast differentiation	BP	1	NKX2-5	up_VPOR_VSMC
GO:1902713	0.0105	1	1	3	regulation of interferon-gamma secretion	BP	1	NR1H4	up_VPUL_EC
GO:0046794	0.0105	1	1	2	transport of virus	BP	0.899	CAV1	up_VSAP_VSMC
GO:0016310	0.010555302	0.99432	27	641	phosphorylation	BP	0.96436359	BRSK1,PLCL1,PKIB,SOX9,NDRG2,ENSCAFG00000004331,FZD5,DUSP26,NOD2,SMAD6,PAK3,GRK7,TNFRSF19,DCLK2,MAPK8IP2,MAVS,LRRC15,LRP4,SH3RF3,LRRC4,ALDOC,RARRES2,STK32C,NRP1,PRKCD,DBNDD2,CCNYL1	up_ACOR_EC
GO:0051186	0.0106	0.994	25	159	cofactor metabolic process	BP	1	ACSM3,ACACB,NADK,GOT1,ADHFE1,ENSCAFG00000013986,COQ9,PDK2,FXN,GALK1,CTNS,CLYBL,MOCOS,PCBD1,ACSS2,MVK,PNPO,DCAKD,CPOX,PNP,ZNF205,IDH1,HMOX1,ENSCAFG00000006478,DDIT4	up_VSAP_EC
GO:0046605	0.0106	0.997	7	30	regulation of centrosome cycle	BP	0.461	NEK2,POC1A,NUP62,PLK4,RBM14,KIF11,GEN1	down_ACOR_VSMC
GO:0035295	0.0106	0.998	5	356	tube development	BP	1	TBX5,HOXA3,NPNT,NTRK2,HOXA1	up_APUL_EC
GO:0030857	0.0106	0.998	5	15	negative regulation of epithelial cell	BP	1	HOXA7,DLL1,OSR1,ZEB1,ACVRL1	up_VSAP_EC
GO:0071363	0.0106	0.999	3	188	cellular response to growth factor stimulus	BP	1	FGF5,NKX2-5,BAMBI	up_VPOR_VSMC
GO:0018216	0.0106	1	2	3	peptidyl-arginine methylation	BP	0.51	NR1H4,PRMT1	down_VSAP_EC
GO:2000403	0.0106	1	2	7	positive regulation of lymphocyte migration	BP	1	CCL5,ADAM17	up_AFEM_EC
GO:0019371	0.0106	1	2	2	cyclooxygenase pathway	BP	0.435	PTGS1,PTGS2	up_VCAV_EC
GO:0035270	0.0106	1	2	35	endocrine system development	BP	0.903	PITX1,SALL1	up_VSAP_VSMC
GO:0045605	0.0106	1	1	5	negative regulation of epidermal cell	BP	1	SRSF6	down_AFEM_EC
GO:0060065	0.0106	1	1	7	uterus development	BP	1	HOXA9	up_AFEM_VSMC
GO:1990774	0.0106	1	1	9	tumor necrosis factor secretion	BP	0.907	ARG2	up_AORT_VSMC
GO:0051482	0.0106	1	1	5	positive regulation of cytosolic calcium ion concentration involved in phospholipase C-activating G protein-	BP	1	C3AR1	up_VPOR_VSMC
GO:0071624	0.0106	1	1	6	positive regulation of granulocyte chemotaxis	BP	1	C3AR1	up_VPOR_VSMC
GO:0090023	0.0106	1	1	6	positive regulation of neutrophil chemotaxis	BP	1	C3AR1	up_VPOR_VSMC
GO:0021615	0.0106	1	1	3	glossopharyngeal nerve morphogenesis	BP	1	HOXA3	up_VPUL_EC
GO:0046628	0.0106	1	1	3	positive regulation of insulin receptor signaling	BP	1	NR1H4	up_VPUL_EC
GO:0019932	0.0107	0.996	15	82	second-messenger-mediated signaling	BP	0.974	ADRA1B,ADGRD1,HTR2B,C28H10orf71,ABCA1,PDE5A,PTGIR,ADCY6,ITPR1,DMPK,RCAN2,APLP1,EDN1,NCALD,GNAQ	up_ACOR_VSMC
GO:0048699	0.0107	0.998	4	371	generation of neurons	BP	1	HOXC10,EYA1,EMX2,SHOX2	up_VFEM_VSMC
GO:0060485	0.0107	0.999	3	93	mesenchyme development	BP	0.801	ACVRL1,PDPN,NRP2	down_ACOR_EC
GO:0070848	0.0107	1	2	193	response to growth factor	BP	1	GATA5,TGFBR1	down_VSAP_VSMC
GO:0010767	0.0107	1	1	2	regulation of transcription from RNA polymerase II promoter in response to UV-induced DNA damage	BP	0.801	NEDD4	down_ACOR_EC
GO:0030388	0.0107	1	1	4	fructose 1,6-bisphosphate metabolic process	BP	1	FBP1	up_VFEM_VSMC

GO:0010033	0.0108	0.992	93	772	response to organic substance	BP	0.974	TLR1,SMAD6,ACTN2,PLCG2,IFNGR1,LEP,LBH,AFF3,GATA5,CD83,SKP2,PRKAA2,FERMT1,BMP4,TM7SF3,FGFR2,ABCA1,EIF4A2,ATRX,MED14,CASTOR1,TLR3,LATS2,LPL,CITED2,TP53INP1,RERG,GLRB,ISG15,CD274,AR,PTGIR,SASH1,SLC2A5,TMEM108,FGF18,FAM210B,CDKN2B,ADCY6,KLHL20,ANKRD1,F2RL1,MXRA5,DAPK1,CTSB,AKAP6,P2RX7,CREBRF,NRP1,UBR1,PTPN22,NPNT,OS9,STT3B,EVL,APLP1,PTGDR,SESN3,CCDC3,PRKCE,FRS2,AGRN,FUT8,PARP9,APP,PSEN1,TGFBR1,JUP,CCL5,NR3C1,DUSP22,RAB11B,HPGD,NOD2,LRRC4,ARID5B,FAM107A,DDIT4,B2M,ENSCAFG00000018360,JKAMP,TNFRSF11B,HERPUD1,GNAQ,INPP5K,FAF2,PFKL,WFS1,DZIP3,SH3BP4,PARK7,STC2,CRKL	up_ACOR_VSMC
GO:0009790	0.0108	0.993	45	335	embryo development	BP	1	TBX15,HOXA11,FZD1,HOXA7,HOXA9,CDON,WNT11,HNF4A,AXIN2,OSR2,INSIG2,GJB5,LRIG3,IRX5,HOXD3,ABL1,DLL1,HOXA6,OSR1,FUZ,ZBTB16,RARG,HOXC4,RECK,PLPP3,AMBRA1,MFN2,ZFP36L1,FXN,IRX3,ZEB1,FBXW4,SULF2,SHOX2,HOXC10,LRP4,KDM2B,TAF8,ZFAND5,ACVRL1,TPRA1,TGFBR2,CITED2,KEAP1,LATS2	up_VSAP_EC
GO:0072503	0.0108	0.995	17	97	cellular divalent inorganic cation homeostasis	BP	0.974	ADRA1B,PLCG2,HTR2B,MICU2,ITPR1,F2RL1,DMPK,PRKCE,APP,PSEN1,CHD7,EDN1,DISC1,HERPUD1,WFS1,ATP2C1,STC2	up_ACOR_VSMC
GO:0007159	0.0108	0.996	13	79	leukocyte cell-cell adhesion	BP	0.437	IL7R,SELP,SELE,TNIP1,STK10,SELL,GATA3,PELI1,ARG2,ITPKB,NCK1,BCL10,HMGB1	up_VCAV_EC
GO:0002221	0.0108	0.997	9	46	pattern recognition receptor signaling	BP	0.437	TNFAIP3,TNIP1,TLR4,PELI1,RIOK3,TLR3,OTUD4,HMGB1,LSM14A	up_VCAV_EC
GO:0002758	0.0108	0.997	9	46	innate immune response-activating signal	BP	0.437	TNFAIP3,TNIP1,TLR4,PELI1,RIOK3,TLR3,OTUD4,HMGB1,LSM14A	up_VCAV_EC
GO:0032611	0.0108	0.998	5	17	interleukin-1 beta production	BP	0.437	GBP5,TNFAIP3,TLR4,ARG2,ABCA1	up_VCAV_EC
GO:0045907	0.0108	0.999	3	6	positive regulation of vasoconstriction	BP	0.974	ADRA1B,HRH1,HRH2	up_ACOR_VSMC
GO:0071396	0.0108	0.999	3	134	cellular response to lipid	BP	1	NR1H4,MRC1,PTGER2	up_VPUL_EC
GO:0045638	0.0108	1	2	33	negative regulation of myeloid cell	BP	0.801	LYN,HOXA7	down_ACOR_EC
GO:2000404	0.0108	1	2	7	regulation of T cell migration	BP	1	CCL5,ADAM17	up_AFEM_EC
GO:0002467	0.0108	1	2	2	germinal center formation	BP	0.437	TNFAIP3,MEF2C	up_VCAV_EC
GO:0002634	0.0108	1	2	2	regulation of germinal center formation	BP	0.437	TNFAIP3,MEF2C	up_VCAV_EC
GO:0007411	0.0108	0.999	2	45	axon guidance	BP	1	FLRT3,NEO1	up_VPUL_EC
GO:0071222	0.0108	0.999	2	46	cellular response to lipopolysaccharide	BP	1	NR1H4,MRC1	up_VPUL_EC
GO:0097485	0.0108	0.999	2	45	neuron projection guidance	BP	1	FLRT3,NEO1	up_VPUL_EC
GO:0048565	0.0108	0.999	2	38	digestive tract development	BP	0.907	SALL1,SHOX2	up_VSAP_VSMC
GO:0060759	0.0108	0.999	2	37	regulation of response to cytokine stimulus	BP	0.907	TLR2,CAV1	up_VSAP_VSMC
GO:0071559	0.0108	1	1	75	response to transforming growth factor beta	BP	1	MXRA5	down_AORT_VSMC
GO:0002065	0.0108	1	1	37	columnar/cuboidal epithelial cell	BP	1	GATA5	down_VFEM_VSMC
GO:0071168	0.0108	1	1	9	protein localization to chromatin	BP	0.919	ESR1	up_AORT_VSMC
GO:0051926	0.0108	1	1	14	negative regulation of calcium ion transport	BP	1	HOXA3	up_APUL_VSMC
GO:0021602	0.0108	1	1	16	cranial nerve morphogenesis	BP	1	HOXA3	up_APUL_VSMC
GO:0055015	0.0108	1	1	5	ventricular cardiac muscle cell development	BP	1	NKX2-5	up_VPOR_VSMC
GO:0032237	0.0108	1	1	3	activation of store-operated calcium channel	BP	0.907	STIM1	up_VSAP_VSMC
GO:1901341	0.0108	1	1	3	positive regulation of store-operated calcium	BP	0.907	STIM1	up_VSAP_VSMC
GO:0009950	0.010839633	0.9996	2	6	dorsal/ventral axis specification	BP	0.96436359	SMAD6,LRP4	up_ACOR_EC

GO:0050896	0.0109	0.992	164	2346	response to stimulus	BP	0.514	ARHGEF2,PRICKLE1,VEGFA,P4HB,SIPA1L1,SULF1,ARHGAP31,ASNS,PLOD2,NR1H4,PLK3,ENSCAFG00000013214,HSPA5,SERPINE1,SOCS5,HM13,SPARC,SGK1,ERO1A,PRKACB,HSP90B1,ENPP1,ERRFI1,ITGA2,JCAD,SNX25,CBX5,CASP3,FAM49B,ENSCAFG00000003477,HYOU1,PGK1,WSB1,BCAR3,NUAK1,PTPN11,FLNB,COTL1,DPYSL3,LRRC8A,TRIB3,IFRD1,FARP1,ILK,FHL2,BHLHE40,PPP1R12A,PRCP,SMARCA4,SESN2,SRP54,TGFBR1,PTGS2,REM1,MYO1C,PIK3C2A,CHSY1,MSN,UACA,SEL1L,RPGR,KIF5B,HIF1A,CX3CL1,ENSCAFG00000000562,DNAJC10,TNIK,NXN,UBA6,RGS2,ZPR1,PTPN2,EIF2B1,PVR,YES1,NEO1,PLCL1,MTDH,ESM1,DDX39B,ADAM17,AGRN,ACTA2,EIF4EBP1,SKAP2,PDIA4,CNOT7,ADGRE5,MAGED1,DNAJB9,GNAQ,CTNNB1,KPNB1,SRP72,CRYAB,STARD13,ENSCAFG00000002015,TFRC,APOE,WWC3,KPNA4,PDE1A,ZC3H15,KLF5,UBA5,MMP28,LYN,BNIP3,STRAP,DDIT3,PDS5B,FAM162A,BACH1,PLEKHG2,TLN1,SLC7A11,CBLC,ATF3,GSK3B,CDC37,GTF2H1,ITGB3,GBP5,SH3GLB1,TAX1BP3,MYO1E,SDC4,KPNA1,HTT,REST,RBBP8,MAPK10,EIF2S1,SH2B3,RDH11,ARFGEF3,EXT1,CALM1,DISC1,RAD23A,PAK2,CREB3L2,A2M,INHBA,CAMK2D,NR4A1,ENSCAFG00000018360,HI	down_VSAP_EC
GO:1902533	0.0109	0.993	38	271	positive regulation of intracellular signal transduction	BP	0.974	ADRA1B,LEP,MID1,HTR2B,RASSF2,C28H10orf71,NACC2,BMP4,FGFR2,TLR3,SH3RF2,PDE5A,SASH1,FGF18,ANKRD1,F2RL1,CRLF3,NRP1,PTPN22,NPNT,UNC5B,PRKCE,FRS2,PARP9,APP,PSEN1,CCL5,DUSP22,SIAH1,EDN1,NOD2,SH3RF3,SERINC3,MAP3K7,ENSCAFG00000029553,PARK7,IL18R1,CRKL	up_ACOR_VSMC
GO:0016051	0.0109	0.997	9	63	carbohydrate biosynthetic process	BP	0.514	ENPP1,TPI1,ENSCAFG00000032470,PGAM1,SESN2,PTPN2,PPP1R3C,ATF3,EXT1	down_VSAP_EC
GO:0006937	0.0109	0.997	9	40	regulation of muscle contraction	BP	0.974	TNNT2,FGF13,ADRA1B,KCNE3,PDE5A,DMPK,NPNT,JUP,DOCK5	up_ACOR_VSMC
GO:0032502	0.0109	0.997	9	1661	developmental process	BP	1	CHL1,HOXA3,BARX1,NKX2-5,BAMBI,TRPC4,ANKRD6,CYP26B1,C3AR1	up_VPOR_VSMC
GO:0032637	0.0109	0.997	7	26	interleukin-8 production	BP	0.974	TLR1,LEP,TLR3,F2RL1,PTPN22,NOD2,PARK7	up_ACOR_VSMC
GO:0070231	0.0109	0.998	5	18	T cell apoptotic process	BP	0.437	IL7R,ARG2,BCL10,RIPK1,HIF1A	up_VCAV_EC
GO:0061045	0.0109	0.999	4	12	negative regulation of wound healing	BP	0.437	PDGFB,CD34,KLKB1,TFPI	up_VCAV_EC
GO:0090208	0.0109	0.999	3	6	positive regulation of triglyceride metabolic	BP	0.471	SREBF1,CTDNEP1,ABHD5	down_ACOR_VSMC
GO:1903725	0.0109	0.999	2	30	regulation of phospholipid metabolic process	BP	0.801	LYN,ACSL3	down_ACOR_EC
GO:0007507	0.0109	1	2	194	heart development	BP	1	GATA5,TGFBR1	down_VSAP_VSMC
GO:0090335	0.0109	1	2	7	regulation of brown fat cell differentiation	BP	1	LEP,NAPEPLD	up_AFEM_EC
GO:0008284	0.0109	1	2	238	positive regulation of cell proliferation	BP	1	HOXA3,TBX5	up_APUL_VSMC
GO:0006044	0.0109	1	1	5	N-acetylglucosamine metabolic process	BP	1	EXTL2	down_AFEM_EC
GO:0021563	0.0109	1	1	4	glossopharyngeal nerve development	BP	1	HOXA3	up_VPOR_VSMC
GO:0050920	0.011	0.997	8	53	regulation of chemotaxis	BP	0.517	VEGFA,SERPINE1,PTPN2,ADAM17,MMP28,LYN,NRP1,USP14	down_VSAP_EC
GO:0009059	0.011	0.998	6	1563	macromolecule biosynthetic process	BP	1	TLR2,TBX15,EYA1,HOXA9,HOXA10,IRX5	up_AFEM_VSMC
GO:0030511	0.011	0.999	4	10	positive regulation of transforming growth factor beta receptor	BP	0.974	FERMT1,CITED2,CDKN2B,NPNT	up_ACOR_VSMC
GO:1903846	0.011	0.999	4	10	positive regulation of cellular response to transforming growth	BP	0.974	FERMT1,CITED2,CDKN2B,NPNT	up_ACOR_VSMC
GO:0016558	0.011	0.999	4	10	protein import into peroxisome matrix	BP	1	PEX7,PEX2,LONP2,PEX6	up_VSAP_EC
GO:2000146	0.011	0.999	3	92	negative regulation of cell motility	BP	0.801	ACVRL1,HOXA7,SULF1	down_ACOR_EC
GO:0006029	0.011	0.999	2	29	proteoglycan metabolic process	BP	0.801	SULF1,CSGALNACT1	down_ACOR_EC
GO:0010712	0.011	1	2	7	regulation of collagen metabolic process	BP	1	MFAP4,FAP	up_AFEM_EC
GO:1902105	0.011	1	2	80	regulation of leukocyte differentiation	BP	1	TMEM176A,TMEM176B	up_VPUL_VSMC

GO:0072028	0.011	0.999	2	37	nephron morphogenesis	BP	0.908	HOXA11,SALL1	up_VSAP_VSMC
GO:0000463	0.011	1	1	3	maturation of LSU-rRNA from tricistronic rRNA transcript (SSU-rRNA, 5.8S	BP	0.801	WDR12	down_ACOR_EC
GO:0045671	0.011	1	1	8	negative regulation of osteoclast differentiation	BP	1	LRRC17	up_VCAV_VSMC
GO:0048752	0.011	1	1	5	semicircular canal morphogenesis	BP	1	EYA1	up_VFEM_VSMC
GO:0048755	0.011	1	1	4	branching morphogenesis of a nerve	BP	1	SHOX2	up_VFEM_VSMC
GO:0014707	0.011	1	1	2	branchiomeric skeletal muscle development	BP	0.908	PITX1	up_VSAP_VSMC
GO:2000286	0.011	1	1	2	receptor internalization involved in canonical Wnt signaling pathway	BP	0.908	CAV1	up_VSAP_VSMC
GO:1903829	0.011088393	0.99699	7	91	positive regulation of cellular protein	BP	0.96436359	KCNB1,EDEM2,PLS1,TRIM8,MAVS,LRP4,PRKCD	up_ACOR_EC
GO:0050789	0.0111	0.991	346	3295	regulation of biological process	BP	0.974	SYNPO2L,EXPH5,TNNT2,SH3TC2,FGF13,RHOT1,ADRA1B,VEGFD,TMEM176B,TLR1,TMEM176A,DNAJC6,SMAD6,ATRNL1,ARHGAP28,ACTN2,LGR6,PLCG2,CASP10,IFNGR1,LEP,PLCL1,LBH,DCBLD2,MAP3K8,MID1,KCNE3,GATA5,BCL2,GATA4,ADGRD1,CD83,EFNB2,CDC7,ANGPT1,PCGF5,ENSCAFG00000016285,HTR2B,RASSF2,TCAF1,MEIS2,USP6NL,FNDC5,MBP,ZRANB1,FBXL20,C28H10orf71,PRDX4,RECK,LIPT2,FEZ1,SKP2,NACC2,COCH,PRKAA2,MLLT3,ITM2C,FERMT1,BMP4,PNRC1,LCAT,ZNF331,EVI5L,CRIM1,LNPK,SYNPO2,TUBB4A,TM7SF3,FGFR2,TP53BP1,MX1,MFAP4,ABCA1,EIF4A2,GJA1,DOCK3,ELF1,ATRX,MED14,HRH1,CASTOR1,TRPS1,TLR3,SH3RF2,SEC24B,WASF3,PPARGC1A,LATS2,PDE5A,ZNF821,CITED2,TP53INP1,ATXN2,ZNF550,RERG,GLRB,PKP4,ARHGAP29,CCNG2,ISG15,ZNRF3,ZNF782,CD274,ZNF365,PLPP2,MARCKSL1,AR,PTGIR,ZNF484,SARAF,SASH1,GPR155,LMO4,GTF3C1,PTPN3,TMEM108,ZNF572,FGF18,FAM210B,ARHGEF26,NDRG2,ATRN,P4HTM,TMX3,CHN2,CDKN2B,ADCY6,ZMYM2,ITPR1,GRK7,CST3,ZNF174,PLAC8B,KLHL20,LIMS2,BLNK,ANKRD1,PLEKHB2,F2RL1,HRH2,ADGRL1,FAM129A,CAMK2D,MARCH5,PSEN2,ENPP4,PPP2R5E,ERO1B,DAPK1,PKNOX	up_ACOR_VSMC
GO:0009725	0.0111	0.995	20	188	response to hormone	BP	0.521	NR1H4,ENPP1,PTPN11,TRIB3,FHL2,PRCP,SMARCA4,SESN2,MYO1C,MSN,PTPN2,AGRN,GNAQ,CTNNB1,UBA5,LYN,REST,NR4A1,YWHAH,KDM3A	down_VSAP_EC
GO:0070371	0.0111	0.995	16	91	ERK1 and ERK2 cascade	BP	0.974	HTR2B,BMP4,FGFR2,FGF18,NDRG2,F2RL1,NRP1,PTPN22,NPNT,FRS2,APP,SYNJ2BP,CCL5,DUSP26,ENSCAFG00000029553,CRKL	up_ACOR_VSMC
GO:0032502	0.0111	0.997	9	1661	developmental process	BP	1	HOXC10,EYA1,GATM,HOXA9,SPINK5,TBX15,EMX2,HOXA11,SHOX2	up_VFEM_VSMC
GO:0006638	0.0111	0.997	7	30	neutral lipid metabolic process	BP	0.476	SREBF1,CTDNEP1,INSIG1,CAV1,ABHD5,LIPC,GK5	down_ACOR_VSMC
GO:0006639	0.0111	0.997	7	30	acylglycerol metabolic process	BP	0.476	SREBF1,CTDNEP1,INSIG1,CAV1,ABHD5,LIPC,GK5	down_ACOR_VSMC
GO:0006351	0.0111	0.997	7	1069	transcription, DNA-templated	BP	1	EYA1,HOXA10,HOXA9,TBX15,EMX2,HOXA11,SHOX2	up_VFEM_VSMC
GO:0034654	0.0111	0.999	4	1236	nucleobase-containing compound biosynthetic	BP	1	AK5,GATA5,TGFBR1,MEIS2	down_VSAP_VSMC
GO:1902106	0.0111	0.999	2	31	negative regulation of leukocyte differentiation	BP	0.801	LYN,HOXA7	down_ACOR_EC
GO:0071482	0.0111	0.999	2	34	cellular response to light stimulus	BP	0.801	USP28,NEDD4	down_ACOR_EC
GO:0071548	0.0111	1	2	7	response to dexamethasone	BP	1	FIBIN,NR3C1	up_AFEM_EC
GO:0002730	0.0111	1	2	2	regulation of dendritic cell cytokine production	BP	0.441	TLR4,TLR3	up_VCAV_EC
GO:0045359	0.0111	1	2	2	positive regulation of interferon-beta	BP	0.441	TLR4,TLR3	up_VCAV_EC
GO:0061314	0.0111	1	2	2	Notch signaling involved in heart development	BP	0.441	DLL4,JAG1	up_VCAV_EC
GO:0010771	0.0111	0.999	2	21	negative regulation of cell morphogenesis involved in differentiation	BP	1	SEMA3A,RNF6	up_VPOR_EC
GO:0048762	0.0111	1	1	77	mesenchymal cell differentiation	BP	1	GATA5	down_AFEM_VSMC
GO:0001759	0.0111	1	1	13	organ induction	BP	1	GATA5	down_VSAP_VSMC

GO:0006880	0.0111	1	1	4	intracellular sequestering of iron ion	BP	1	ENSCAFG00000005494	up_AORT_EC
GO:0097577	0.0111	1	1	4	sequestering of iron ion	BP	1	ENSCAFG00000005494	up_AORT_EC
GO:0032355	0.0111	1	1	10	response to estradiol	BP	0.931	ESR1	up_AORT_VSMC
GO:0060135	0.0111	1	1	10	maternal process involved in female pregnancy	BP	0.931	ESR1	up_AORT_VSMC
GO:0070233	0.0111	1	1	9	negative regulation of T cell apoptotic process	BP	0.931	ARG2	up_AORT_VSMC
GO:0042693	0.0111	1	1	4	muscle cell fate commitment	BP	1	NKX2-5	up_VPOR_VSMC
GO:2000096	0.0111	1	1	4	positive regulation of Wnt signaling pathway, planar cell polarity pathway	BP	1	ANKRD6	up_VPOR_VSMC
GO:0008356	0.0111	1	1	3	asymmetric cell division	BP	1	ARHGEF2	up_VPUL_EC
GO:0035690	0.01112454	0.99698	7	91	cellular response to drug	BP	0.96436359	SOX9,AQP1,AGRN,NOD2,PDE4A,GUCY1B1,PRKCD	up_ACOR_EC
GO:0034762	0.0112	0.995	20	122	regulation of transmembrane transport	BP	0.974	ACTN2,PLCG2,LEP,KCNE3,TCAF1,GJA1,PTPN3,DAPK1,CACNB3,AKAP6,KCNJ4,PRKCE,PSEN1,CHD7,EDN1,KCNC4,FXYD1,INPP5K,AZIN2,PARK7	up_ACOR_VSMC
GO:0060047	0.0112	0.998	5	57	heart contraction	BP	1	IRX5,CHRM2,THRB,BVES,DMPK	up_AFEM_EC
GO:1903599	0.0112	0.999	3	8	positive regulation of autophagy of	BP	0.521	HIF1A,CDC37,HTT	down_VSAP_EC
GO:0051452	0.0112	0.999	3	20	intracellular pH reduction	BP	1	RAB20,RAB7A,ATP6V0B	up_AFEM_EC
GO:0048646	0.0112	0.999	3	349	anatomical structure formation involved in	BP	1	GATA5,C6,TGFBI	up_VCAV_VSMC
GO:0045446	0.0112	0.999	2	32	endothelial cell differentiation	BP	0.801	ACVRL1,PDPN	down_ACOR_EC
GO:0048675	0.0112	0.999	2	26	axon extension	BP	0.801	SPG11,NRP2	down_ACOR_EC
GO:1903076	0.0112	0.999	2	32	regulation of protein localization to plasma	BP	0.801	ACSL3,STX4	down_ACOR_EC
GO:0010043	0.0112	1	2	7	response to zinc ion	BP	1	MT2A,ENSCAFG00000009113	up_AFEM_EC
GO:0060537	0.0112	1	2	117	muscle tissue development	BP	1	EYA1,BEND2	up_AFEM_VSMC
GO:0034144	0.0112	1	1	3	negative regulation of toll-like receptor 4 signaling	BP	0.801	LYN	down_ACOR_EC
GO:0048565	0.0112	1	1	38	digestive tract development	BP	1	GATA5	down_VFEM_VSMC
GO:0006956	0.0112	1	1	8	complement activation	BP	1	C6	up_VCAV_VSMC
GO:0043587	0.0112	1	1	4	tongue morphogenesis	BP	1	CYP26B1	up_VPOR_VSMC
GO:0003211	0.0112	1	1	4	cardiac ventricle formation	BP	1	NKX2-5	up_VPOR_VSMC
GO:0051260	0.0113	0.995	15	100	protein homooligomerization	BP	0.444	GBP5,CDA,VWF,ACPP,LNX2,RIOK3,KCNJ2,EHD4,BCL10,GCH1,BLM,RIPK1,ALDH1A3,P2RX2,TNFAIP1	up_VCAV_EC
GO:0032774	0.0113	0.997	7	1073	RNA biosynthetic process	BP	1	EYA1,HOXA10,HOXA9,TBX15,EMX2,HOXA11,SHOX2	up_VFEM_VSMC
GO:0097659	0.0113	0.997	7	1072	nucleic acid-templated transcription	BP	1	EYA1,HOXA10,HOXA9,TBX15,EMX2,HOXA11,SHOX2	up_VFEM_VSMC
GO:0051093	0.0113	0.998	5	273	negative regulation of developmental process	BP	0.801	ACVRL1,LYN,HOXA7,SULF1,ASPN	down_ACOR_EC
GO:0035773	0.0113	0.998	5	15	insulin secretion involved in cellular response to glucose stimulus	BP	1	RAB11B,RAF1,STX4,PPP3CB,RAB11FIP2	up_VSAP_EC
GO:0032330	0.0113	0.999	3	20	regulation of chondrocyte differentiation	BP	1	GREM1,PTHLH,AXIN2	up_AFEM_EC
GO:0072711	0.0113	0.999	3	5	cellular response to hydroxyurea	BP	0.444	TIMELESS,BLM,ATRX	up_VCAV_EC
GO:0046068	0.0113	0.999	3	5	cGMP metabolic process	BP	0.444	NPR1,GUCY1A1,GUCY1B1	up_VCAV_EC
GO:0050793	0.0113	1	2	731	regulation of developmental process	BP	1	JUP,GATA5	down_VFEM_VSMC
GO:0070602	0.0113	1	2	3	regulation of centromeric sister chromatid cohesion	BP	0.525	NAA10,CTNNB1	down_VSAP_EC
GO:0061564	0.0113	1	2	119	axon development	BP	1	SLITRK5,LIMK1	up_VCAV_VSMC
GO:0030593	0.0113	0.999	2	19	neutrophil chemotaxis	BP	1	C3AR1,ENSCAFG00000032731	up_VPOR_EC
GO:0090103	0.0113	1	1	8	cochlea morphogenesis	BP	1	EYA1	up_AFEM_VSMC

GO:0021522	0.0113	1	1	8	spinal cord motor neuron differentiation	BP	1	HOXC10	up_AFEM_VSMC
GO:0021515	0.0113	1	1	9	cell differentiation in spinal cord	BP	0.931	HOXC10	up_AORT_VSMC
GO:0000820	0.0113	1	1	3	regulation of glutamine family amino acid	BP	1	NR1H4	up_APUL_EC
GO:0035136	0.0113	1	1	16	forelimb morphogenesis	BP	1	TBX5	up_APUL_VSMC
GO:0021544	0.0113	1	1	8	subpallium development	BP	1	SLITRK5	up_VCAV_VSMC
GO:0032228	0.0113	1	1	8	regulation of synaptic transmission, GABAergic	BP	1	CA2	up_VCAV_VSMC
GO:0009891	0.0114	0.992	59	521	positive regulation of biosynthetic process	BP	0.447	HCLS1,ADGRF5,PPARG,TNIP1,PDGFB,TMEM173,MEF2C,ATF3,CHD7,TLR4,NLRC5,GATA3,EDN1,RAB27A,TCF4,FOXN2,ENSCAFG00000017103,IRF8,PPP3CA,YES1,MTOR,BMP2,HHEX,CREM,CNOT6,PROX1,ARID4B,HMBOX1,PRKD2,THOC1,SMARCA4,TLR3,NCK1,LDB2,JAG1,NSD1,PTGS2,CREB1,BCL10,CNOT7,HMGB1,AHR,NOS2,BLM,RIPK1,ITGA6,GPBP1,MTA2,NR1D2,KDM5A,HIF1A,KDM3A,ATRX,NR4A1,ZEB1,TET2,ARHGEF2,CDON,ZNF609	up_VCAV_EC
GO:0034765	0.0114	0.995	16	91	regulation of ion transmembrane transport	BP	0.976	ACTN2,PLCG2,KCNE3,TCAF1,GJA1,PTPN3,DAPK1,CACNB3,AKAP6,KCNJ4,PRKCE,PSEN1,CHD7,KCNC4,FXYD1,PARK7	up_ACOR_VSMC
GO:0042310	0.0114	0.998	6	21	vasoconstriction	BP	0.976	ADRA1B,HTR2B,HRH1,HRH2,EDN1,DOCK5	up_ACOR_VSMC
GO:0097756	0.0114	0.998	6	21	negative regulation of blood vessel diameter	BP	0.976	ADRA1B,HTR2B,HRH1,HRH2,EDN1,DOCK5	up_ACOR_VSMC
GO:0050793	0.0114	0.998	5	731	regulation of developmental process	BP	1	TBX5,GATA5,TMEM176A,TMEM176B,SLIT2	up_VPUL_VSMC
GO:0006826	0.0114	0.998	5	15	iron ion transport	BP	1	RAB11B,SLC25A28,SCARA5,STEAP3,FTL	up_VSAP_EC
GO:0090287	0.0114	0.999	3	91	regulation of cellular response to growth factor	BP	0.801	ACVRL1,SULF1,ASPN	down_ACOR_EC
GO:0042127	0.0114	0.999	3	452	regulation of cell proliferation	BP	0.931	WNT7A,ARG2,ESR1	up_AORT_VSMC
GO:0045893	0.0114	0.999	3	413	positive regulation of transcription, DNA-	BP	0.931	HOXA9,WNT7A,ESR1	up_AORT_VSMC
GO:0070848	0.0114	0.999	3	193	response to growth factor	BP	1	FGF5,NKX2-5,BAMBI	up_VPOR_VSMC
GO:0014706	0.0114	0.999	3	112	striated muscle tissue development	BP	0.984	EYA1,SIX1,CYP26B1	up_VFEM_EC
GO:0045176	0.0114	1	2	3	apical protein localization	BP	0.529	SHROOM2,ARF4	down_VSAP_EC
GO:0045656	0.0114	1	1	3	negative regulation of monocyte differentiation	BP	0.801	HOXA7	down_ACOR_EC
GO:0060017	0.0114	1	1	3	parathyroid gland development	BP	0.801	HOXA3	down_ACOR_EC
GO:0006937	0.0114	1	1	40	regulation of muscle contraction	BP	1	JUP	down_VFEM_VSMC
GO:0021696	0.0114	1	1	10	cerebellar cortex morphogenesis	BP	0.931	WNT7A	up_AORT_VSMC
GO:0046636	0.0114	1	1	10	negative regulation of alpha-beta T cell	BP	0.931	ARG2	up_AORT_VSMC
GO:0003229	0.0114	1	1	17	ventricular cardiac muscle tissue development	BP	1	TBX5	up_APUL_VSMC
GO:0060411	0.011496878	0.99824	4	33	cardiac septum morphogenesis	BP	0.96436359	GATA4,SMAD6,TBX5,NRP1	up_ACOR_EC
GO:0021700	0.0115	0.996	12	65	developmental maturation	BP	0.447	EDNRB,ARHGEF15,RELN,FERMT1,CDH5,GATA3,NTN4,SOX18,BMP2,SPTBN4,HIF1A,FBXO5	up_VCAV_EC
GO:0034654	0.0115	0.996	11	1236	nucleobase-containing compound biosynthetic	BP	0.919	HOXA11,TBX15,HOXA10,HOXA9,PITX1,EMX2,SALL1,IRX5,TLR2,CAV1,SHOX2	up_VSAP_VSMC
GO:0030154	0.0115	0.998	6	1040	cell differentiation	BP	1	TBX5,GATA5,TMEM176A,TMEM176B,HOXB5,SLIT2	up_VPUL_VSMC
GO:1902680	0.0115	0.999	3	415	positive regulation of RNA biosynthetic process	BP	0.931	HOXA9,WNT7A,ESR1	up_AORT_VSMC
GO:1903508	0.0115	0.999	3	415	positive regulation of nucleic acid-templated	BP	0.931	HOXA9,WNT7A,ESR1	up_AORT_VSMC
GO:0071356	0.0115	0.999	3	57	cellular response to tumor necrosis factor	BP	1	ARHGEF2,ENSCAFG00000002015,ENSCAFG00000032731	up_VPOR_EC
GO:0036257	0.0115	1	2	3	multivesicular body organization	BP	0.447	RAB11A,RAB27A	up_VCAV_EC
GO:0006813	0.0115	0.999	2	37	potassium ion transport	BP	0.919	NETO1,CAV1	up_VSAP_VSMC
GO:0010694	0.0115	1	1	3	positive regulation of alkaline phosphatase	BP	1	NPNT	up_APUL_EC

GO:1903052	0.0116	0.997	8	51	positive regulation of proteolysis involved in cellular protein catabolic	BP	0.531	PRICKLE1,PLK3,SOCS5,TRIB3,GSK3B,DISC1,RAD23A,TMTC3	down_VSAP_EC
GO:0007275	0.0116	0.997	8	1370	multicellular organism development	BP	1	HOXC10,EYA1,GATM,HOXA9,TBX15,EMX2,HOXA11,SHOX2	up_VFEM_VSMC
GO:0019395	0.0116	0.997	8	33	fatty acid oxidation	BP	1	PEX7,ACAA1,ACACB,PEX2,C1QTNF2,GCDH,EHHADH,LONP2	up_VSAP_EC
GO:0001658	0.0116	0.998	5	24	branching involved in ureteric bud	BP	0.531	ILK,HOXB7,MAGED1,CTNNB1,SIX4	down_VSAP_EC
GO:0060412	0.0116	0.998	5	15	ventricular septum morphogenesis	BP	1	FZD1,WNT11,SAV1,TGFBR2,CITED2	up_VSAP_EC
GO:0002011	0.0116	0.999	2	28	morphogenesis of an epithelial sheet	BP	0.804	ACVRL1,PDPN	down_ACOR_EC
GO:0048844	0.0116	0.999	2	30	artery morphogenesis	BP	0.804	ACVRL1,HOXA1	down_ACOR_EC
GO:1900748	0.0116	1	2	3	positive regulation of vascular endothelial growth factor signaling	BP	0.531	JCAD,MYO1C	down_VSAP_EC
GO:0048640	0.0116	0.999	2	21	negative regulation of developmental growth	BP	1	SEMA3A,RNF6	up_VPOR_EC
GO:0045165	0.0116	0.999	2	65	cell fate commitment	BP	1	NKX2-5,CYP26B1	up_VPOR_VSMC
GO:0035313	0.0116	1	1	7	wound healing, spreading of epidermal cells	BP	0.933	WNT7A	up_AORT_VSMC
GO:0055025	0.0116	1	1	17	positive regulation of cardiac muscle tissue	BP	1	TBX5	up_APUL_VSMC
GO:0070836	0.0116	1	1	2	caveola assembly	BP	0.919	CAV1	up_VSAP_VSMC
GO:0032652	0.011607369	0.99885	3	18	regulation of interleukin-1 production	BP	0.96436359	NR1H4,FZD5,NOD2	up_ACOR_EC
GO:0043254	0.011628727	0.9959	10	164	regulation of protein complex assembly	BP	0.96436359	TMOD1,SMAD6,PAK3,PTGER4,PREB,CLSTN1,CLU,VPS16,PRKCD,TUBB4A	up_ACOR_EC
GO:0003206	0.011681482	0.99766	5	51	cardiac chamber morphogenesis	BP	0.96436359	GATA4,SMAD6,FOXC2,TBX5,NRP1	up_ACOR_EC
GO:0010562	0.0117	0.993	38	312	positive regulation of phosphorus metabolic process	BP	0.448	TEK,HCLS1,ADGRF5,KIT,PPP2R5A,MAP3K11,PDGFB,STK10,RELN,MEF2C,TLR4,EDN1,RALB,APLNR,NEDD9,YES1,MTOR,MAP3K3,BMP2,SLK,PROX1,MADD,PAK2,PRKD2,SH3GLB1,TLR3,PTGS2,MMD,RAPGEF2,TAOK1,HMGB1,NOS2,RIPK1,ITGA6,PDGFD,JCAD,ARHGEF2,CDON	up_VCAV_EC
GO:0045937	0.0117	0.993	38	312	positive regulation of phosphate metabolic process	BP	0.448	TEK,HCLS1,ADGRF5,KIT,PPP2R5A,MAP3K11,PDGFB,STK10,RELN,MEF2C,TLR4,EDN1,RALB,APLNR,NEDD9,YES1,MTOR,MAP3K3,BMP2,SLK,PROX1,MADD,PAK2,PRKD2,SH3GLB1,TLR3,PTGS2,MMD,RAPGEF2,TAOK1,HMGB1,NOS2,RIPK1,ITGA6,PDGFD,JCAD,ARHGEF2,CDON	up_VCAV_EC
GO:0006310	0.0117	0.995	15	93	DNA recombination	BP	0.498	HMGB1,RPA1,TRIP13,PARPBP,GINS4,RAD54L,RAD51,MND1,TONSL,BRCA1,TFRC,RBBP8,SMC6,H2AFX,GEN1	down_ACOR_VSMC
GO:0002285	0.0117	0.996	10	50	lymphocyte activation involved in immune	BP	0.448	IL6,TLR4,GATA3,RAB27A,MTOR,THOC1,SWAP70,MFNG,PLCG2,HMGB1	up_VCAV_EC
GO:0043536	0.0117	0.997	6	32	positive regulation of blood vessel endothelial	BP	0.532	VEGFA,JCAD,PIK3C2A,HIF1A,ADAM17,NRP1	down_VSAP_EC
GO:0006094	0.0117	0.998	5	24	gluconeogenesis	BP	0.532	TPI1,PGAM1,SESN2,PTPN2,ATF3	down_VSAP_EC
GO:1900044	0.0117	0.999	3	5	regulation of protein K63-linked ubiquitination	BP	0.992	SASH1,PTPN22,NOD2	up_ACOR_VSMC
GO:1903305	0.0117	0.999	2	36	regulation of regulated secretory pathway	BP	0.808	LYN,STX4	down_ACOR_EC
GO:0015014	0.0117	1	2	3	heparan sulfate proteoglycan biosynthetic process, polysaccharide chain biosynthetic process	BP	0.532	ENSCAFG00000032470,EXT1	down_VSAP_EC
GO:0055123	0.0117	1	1	40	digestive system development	BP	1	GATA5	down_VFEM_VSMC
GO:0006749	0.0117	1	1	14	glutathione metabolic process	BP	1	ENSCAFG00000028814	down_VSAP_VSMC
GO:0001502	0.0117	1	1	8	cartilage condensation	BP	0.936	WNT7A	up_AORT_VSMC
GO:0098743	0.0117	1	1	8	cell aggregation	BP	0.936	WNT7A	up_AORT_VSMC
GO:0046628	0.0117	1	1	3	positive regulation of insulin receptor signaling	BP	1	NR1H4	up_APUL_EC
GO:1902713	0.0117	1	1	3	regulation of interferon-gamma secretion	BP	1	NR1H4	up_APUL_EC
GO:0086011	0.0117	1	1	2	membrane repolarization during action potential	BP	0.919	CAV1	up_VSAP_VSMC

GO:0098903	0.0117	1	1	2	regulation of membrane repolarization during action potential	BP	0.919	CAV1	up_VSAP_VSMC
GO:0043207	0.011719558	0.99541	12	216	response to external biotic stimulus	BP	0.96436359	NR1H4,HMCN1,RGS1,FZD5,PTGIR,NOD2,PTGER4,MAVS,IFIT2,RARRES2,AGBL5,PRKCD	up_ACOR_EC
GO:0051707	0.011719558	0.99541	12	216	response to other organism	BP	0.96436359	NR1H4,HMCN1,RGS1,FZD5,PTGIR,NOD2,PTGER4,MAVS,IFIT2,RARRES2,AGBL5,PRKCD	up_ACOR_EC
GO:0071310	0.0118	0.997	8	634	cellular response to organic substance	BP	0.808	ACVRL1,LYN,IGF1R,NRP2,SULF1,STX4,ASPN,NEDD4	down_ACOR_EC
GO:0006342	0.0118	0.997	6	24	chromatin silencing	BP	0.499	ATAD2,UHRF1,SUV39H1,HAT1,HIST2H2AC,H2AFX	down_ACOR_VSMC
GO:0007275	0.0118	0.999	5	1370	multicellular organism development	BP	0.936	HOXC10,HOXA9,WNT7A,ARG2,ESR1	up_AORT_VSMC
GO:0019438	0.0118	0.999	4	1258	aromatic compound biosynthetic process	BP	1	AK5,GATA5,TGFBR1,MEIS2	down_VSAP_VSMC
GO:0034035	0.0118	1	2	3	purine ribonucleoside bisphosphate metabolic	BP	0.532	ENPP1,PAPSS1	down_VSAP_EC
GO:0050427	0.0118	1	2	3	3'-phosphoadenosine 5'-phosphosulfate metabolic	BP	0.532	ENPP1,PAPSS1	down_VSAP_EC
GO:0003002	0.0118	0.999	2	118	regionalization	BP	1	HOXC6,GATA5	up_VCAV_VSMC
GO:0021603	0.0118	1	1	2	cranial nerve formation	BP	0.808	HOXA1	down_ACOR_EC
GO:0032400	0.0118	1	1	6	melanosome localization	BP	1	RAB11B	down_VPOR_EC
GO:0032401	0.0118	1	1	6	establishment of melanosome localization	BP	1	RAB11B	down_VPOR_EC
GO:0051875	0.0118	1	1	6	pigment granule localization	BP	1	RAB11B	down_VPOR_EC
GO:0051905	0.0118	1	1	6	establishment of pigment granule localization	BP	1	RAB11B	down_VPOR_EC
GO:0099550	0.0118	1	1	3	trans-synaptic signaling, modulating synaptic	BP	1	NTRK2	up_APUL_EC
GO:0033240	0.0118	1	1	3	positive regulation of cellular amine metabolic	BP	1	NR1H4	up_APUL_EC
GO:0003197	0.0118	1	1	16	endocardial cushion development	BP	1	TBX5	up_APUL_VSMC
GO:0055081	0.0118	1	1	17	anion homeostasis	BP	1	HOXA3	up_APUL_VSMC
GO:1903779	0.0118	1	1	6	regulation of cardiac conduction	BP	1	NKX2-5	up_VPOR_VSMC
GO:0006002	0.0118	1	1	5	fructose 6-phosphate metabolic process	BP	1	FBP1	up_VFEM_VSMC
GO:0003161	0.0118	1	1	6	cardiac conduction system development	BP	1	TBX5	up_VPUL_VSMC
GO:0032272	0.011828334	0.99882	3	18	negative regulation of protein polymerization	BP	0.96436359	TMOD1,PRKCD,TUBB4A	up_ACOR_EC
GO:0009743	0.0119	0.996	9	40	response to carbohydrate	BP	1	HNF4A,RAB11B,LPL,ZFP36L1,KCNB1,RAF1,STX4,PPP3CB,RAB11FIP2	up_VSAP_EC
GO:0048869	0.0119	0.998	5	1119	cellular developmental process	BP	1	HOXC10,EYA1,HOXA9,BEND2,IRX5	up_AFEM_VSMC
GO:0018130	0.0119	0.999	4	1261	heterocycle biosynthetic process	BP	1	AK5,GATA5,TGFBR1,MEIS2	down_VSAP_VSMC
GO:0035295	0.0119	0.999	3	356	tube development	BP	1	GATA5,C6,TGFBI	up_VCAV_VSMC
GO:0001952	0.0119	0.999	2	34	regulation of cell-matrix adhesion	BP	0.812	ACVRL1,HOXA7	down_ACOR_EC
GO:1902115	0.0119	0.999	2	77	regulation of organelle assembly	BP	1	GPSM2,FSCN1	down_AFEM_EC
GO:1901018	0.0119	1	2	2	positive regulation of potassium ion transmembrane	BP	1	ACTN2,AKAP6	up_ACOR_VSMC
GO:0007548	0.0119	0.999	2	68	sex differentiation	BP	1	HOXA9,HOXA11	up_VFEM_VSMC
GO:0050890	0.0119	0.999	2	67	cognition	BP	1	GATM,NETO1	up_VFEM_VSMC
GO:0008584	0.0119	0.999	2	39	male gonad development	BP	0.926	HOXA11,HOXA9	up_VSAP_VSMC
GO:0046546	0.0119	0.999	2	39	development of primary male sexual	BP	0.926	HOXA11,HOXA9	up_VSAP_VSMC
GO:0050704	0.0119	1	1	11	regulation of interleukin-1 secretion	BP	0.939	ARG2	up_AORT_VSMC
GO:0099054	0.0119	1	1	9	presynapse assembly	BP	0.939	WNT7A	up_AORT_VSMC
GO:0014047	0.0119	1	1	3	glutamate secretion	BP	1	NTRK2	up_APUL_EC
GO:0003214	0.0119	1	1	3	cardiac left ventricle morphogenesis	BP	1	TBX5	up_APUL_EC
GO:0018216	0.0119	1	1	3	peptidyl-arginine methylation	BP	1	NR1H4	up_APUL_EC

GO:0019627	0.0119	1	1	3	urea metabolic process	BP	1	NR1H4	up_APUL_EC
GO:0031115	0.0119	1	1	3	negative regulation of microtubule	BP	1	TUBB4A	up_APUL_EC
GO:0051891	0.0119	1	1	3	positive regulation of cardioblast differentiation	BP	1	TBX5	up_APUL_EC
GO:0060017	0.0119	1	1	3	parathyroid gland development	BP	1	HOXA3	up_APUL_EC
GO:0071605	0.0119	1	1	3	monocyte chemotactic protein-1 production	BP	1	NR1H4	up_APUL_EC
GO:0071637	0.0119	1	1	3	regulation of monocyte chemotactic protein-1	BP	1	NR1H4	up_APUL_EC
GO:0071638	0.0119	1	1	3	negative regulation of monocyte chemotactic	BP	1	NR1H4	up_APUL_EC
GO:0072513	0.0119	1	1	3	positive regulation of secondary heart field cardioblast proliferation	BP	1	TBX5	up_APUL_EC
GO:1904468	0.0119	1	1	3	negative regulation of tumor necrosis factor	BP	1	NR1H4	up_APUL_EC
GO:0042473	0.0119	1	1	5	outer ear morphogenesis	BP	1	EYA1	up_VFEM_VSMC
GO:0010648	0.011969521	0.99422	19	407	negative regulation of cell communication	BP	0.96436359	NR1H4,SOX9,NDRG2,KCNB1,CCDC3,IFI6,DUSP26,PTGIR,SMAD6,ADAMTSL2,MAPK8IP2,FIGNL1,LRRC15,LRP4,CLSTN1,LRRC4,CLU,NRP1,PRKCD	up_ACOR_EC
GO:0045834	0.012	0.997	7	41	positive regulation of lipid metabolic process	BP	0.534	NR1H4,PTGS2,SLC45A3,APOE,LYN,SH3GLB1,ABHD5	down_VSAP_EC
GO:0045639	0.012	0.997	7	33	positive regulation of myeloid cell	BP	0.459	HCLS1,CASP8,FES,JAG1,CREB1,RIPK1,HIF1A	up_VCAV_EC
GO:0030198	0.012	0.997	6	81	extracellular matrix organization	BP	1	CTSS,MFAP4,GREM1,POSTN,RECK,FAP	up_AFEM_EC
GO:0003015	0.012	0.998	5	58	heart process	BP	1	IRX5,CHRM2,THRB,BVES,DMPK	up_AFEM_EC
GO:0032502	0.012	0.999	4	1661	developmental process	BP	1	HOXA3,TBX5,HOXA2,HOXB5	up_APUL_VSMC
GO:0051307	0.012	0.999	3	6	meiotic chromosome separation	BP	0.504	NCAPH,ESPL1,TTK	down_ACOR_VSMC
GO:0048598	0.012	0.999	3	207	embryonic morphogenesis	BP	1	HOXC10,HOXA9,DUSP2	up_AORT_EC
GO:0050801	0.012	0.999	3	200	ion homeostasis	BP	1	HOXA3,TRPC4,C3AR1	up_VPOR_VSMC
GO:1900084	0.012	1	2	3	regulation of peptidyl-tyrosine	BP	0.504	CAV1,GREM1	down_ACOR_VSMC
GO:0003002	0.012	0.999	2	118	regionalization	BP	1	HOXC10,HOXA9	up_AFEM_VSMC
GO:0016202	0.012	0.999	2	39	regulation of striated muscle tissue	BP	1	SIX1,CYP26B1	up_VFEM_EC
GO:0048368	0.012	1	1	6	lateral mesoderm development	BP	1	CITED2	down_VPOR_EC
GO:1905207	0.012	1	1	17	regulation of cardiocyte differentiation	BP	1	TBX5	up_APUL_VSMC
GO:0033674	0.0121	0.994	21	144	positive regulation of kinase activity	BP	0.459	TEK,KIT,MAP3K11,PDGFB,STK10,RELN,TLR4,EDN1,RALB,NEDD9,MAP3K3,BMP2,SLK,PROX1,MADD,PAK2,SH3GLB1,MMD,RAPGEF2,RIPK1,PDGFD	up_VCAV_EC
GO:0045747	0.0121	0.998	5	18	positive regulation of Notch signaling pathway	BP	0.459	DLL4,TSPAN14,BMP2K,MFNG,JAG1	up_VCAV_EC
GO:0009066	0.0121	0.998	4	16	aspartate family amino acid metabolic process	BP	0.534	ASNS,PLOD2,NMNAT2,PLOD3	down_VSAP_EC
GO:0048699	0.0121	0.999	3	371	generation of neurons	BP	1	HOXC10,EYA1,IRX5	up_AFEM_VSMC
GO:0060349	0.0121	0.999	2	38	bone morphogenesis	BP	0.931	HOXA11,SHOX2	up_VSAP_VSMC
GO:0071467	0.0121	1	1	6	cellular response to pH	BP	1	RAB11B	down_VPOR_EC
GO:2000826	0.0121	1	1	14	regulation of heart morphogenesis	BP	1	GATA5	down_VSAP_VSMC
GO:0043403	0.0121	1	1	11	skeletal muscle tissue regeneration	BP	0.941	WNT7A	up_AORT_VSMC
GO:0061196	0.0121	1	1	3	fungiform papilla development	BP	1	SIX1	up_VFEM_EC
GO:0061197	0.0121	1	1	3	fungiform papilla morphogenesis	BP	1	SIX1	up_VFEM_EC
GO:0003264	0.0121	1	1	5	regulation of cardioblast proliferation	BP	1	EYA1	up_VFEM_VSMC
GO:0003266	0.0121	1	1	5	regulation of secondary heart field cardioblast	BP	1	EYA1	up_VFEM_VSMC
GO:0010960	0.0121	1	1	6	magnesium ion homeostasis	BP	1	HOXA3	up_VPUL_VSMC

GO:0048012	0.012100722	0.9995	2	7	hepatocyte growth factor receptor signaling	BP	0.96436359	ESM1,NRP1	up_ACOR_EC
GO:0070848	0.0122	0.994	20	193	response to growth factor	BP	0.534	VEGFA,SULF1,HSPA5,SPARC,JCAD,SNX25,PTPN11,ILK,TGFBR1,MYO1C,HIF1A,ZPR1,YES1,NEO1,ADAM17,CTNNB1,ENSCAFG00000002015,STRAP,ENSCAFG00000018360,NRP1	down_VSAP_EC
GO:0010631	0.0122	0.995	14	118	epithelial cell migration	BP	0.534	VEGFA,MYH9,SPARC,JCAD,PTPN11,PRCP,PIK3C2A,HIF1A,ADAM17,ANLN,STARD13,APOE,STRAP,NRP1	down_VSAP_EC
GO:0090132	0.0122	0.995	14	118	epithelium migration	BP	0.534	VEGFA,MYH9,SPARC,JCAD,PTPN11,PRCP,PIK3C2A,HIF1A,ADAM17,ANLN,STARD13,APOE,STRAP,NRP1	down_VSAP_EC
GO:0072078	0.0122	0.997	6	33	nephron tubule morphogenesis	BP	0.534	ENSCAFG00000032470,ILK,HOXB7,MAGED1,CTNNB1,SIX4	down_VSAP_EC
GO:0010464	0.0122	0.997	6	21	regulation of mesenchymal cell	BP	1	WNT11,ZEB1,FBXW4,SHOX2,NFIB,TGFBR2	up_VSAP_EC
GO:2000112	0.0122	0.998	5	1119	regulation of cellular macromolecule biosynthetic process	BP	1	TLR2,EYA1,HOXA9,HOXA10,IRX5	up_AFEM_VSMC
GO:0010894	0.0122	0.999	3	9	negative regulation of steroid biosynthetic	BP	0.534	NR1H4,APOE,REST	down_VSAP_EC
GO:0045939	0.0122	0.999	3	9	negative regulation of steroid metabolic process	BP	0.534	NR1H4,APOE,REST	down_VSAP_EC
GO:0001655	0.0122	0.999	3	116	urogenital system development	BP	0.931	HOXA11,EMX2,SALL1	up_VSAP_VSMC
GO:1904502	0.0122	1	2	3	regulation of lipophagy	BP	0.534	SESN2,HTT	down_VSAP_EC
GO:1904504	0.0122	1	2	3	positive regulation of lipophagy	BP	0.534	SESN2,HTT	down_VSAP_EC
GO:0051099	0.0122	0.999	2	70	positive regulation of binding	BP	1	HOXA3,BAMBI	up_VPOR_VSMC
GO:0055123	0.0122	0.999	2	40	digestive system development	BP	0.931	SALL1,SHOX2	up_VSAP_VSMC
GO:0045617	0.0122	1	1	3	negative regulation of keratinocyte	BP	0.813	HOXA7	down_ACOR_EC
GO:0097048	0.0122	1	1	2	dendritic cell apoptotic process	BP	0.813	LYN	down_ACOR_EC
GO:2000668	0.0122	1	1	2	regulation of dendritic cell apoptotic process	BP	0.813	LYN	down_ACOR_EC
GO:2000670	0.0122	1	1	2	positive regulation of dendritic cell apoptotic	BP	0.813	LYN	down_ACOR_EC
GO:0098815	0.0122	1	1	9	modulation of excitatory postsynaptic potential	BP	0.943	WNT7A	up_AORT_VSMC
GO:0060420	0.0122	1	1	18	regulation of heart growth	BP	1	TBX5	up_APUL_VSMC
GO:0061436	0.0122	1	1	5	establishment of skin barrier	BP	1	CYP26B1	up_VPOR_VSMC
GO:0003128	0.0122	1	1	6	heart field specification	BP	1	GATA5	up_VPUL_VSMC
GO:0055083	0.0122	1	1	6	monovalent inorganic anion homeostasis	BP	1	HOXA3	up_VPUL_VSMC
GO:0060038	0.012211164	0.99876	3	18	cardiac muscle cell proliferation	BP	0.96436359	FOXC2,TBX5,C23H3orf58	up_ACOR_EC
GO:0032527	0.012215222	0.99875	3	19	protein exit from endoplasmic reticulum	BP	0.96436359	EDEM2,PREB,HM13	up_ACOR_EC
GO:1901698	0.012217323	0.9948	14	266	response to nitrogen compound	BP	0.96436359	NR1H4,SOX9,AQP1,ENSCAFG00000004331,EDEM2,AGRN,NOD2,ACTN2,GUCY1B1,MAVS,FOXC2,RARRES2,HM13,PRKCD	up_ACOR_EC
GO:0050663	0.01223116	0.99752	5	52	cytokine secretion	BP	0.96436359	NR1H4,FZD5,NOD2,PTGER4,MAVS	up_ACOR_EC
GO:0042981	0.0123	0.999	3	471	regulation of apoptotic process	BP	0.945	WNT7A,ARG2,ESR1	up_AORT_VSMC
GO:0044057	0.0123	0.999	3	110	regulation of system process	BP	0.939	NETO1,IRX5,CAV1	up_VSAP_VSMC
GO:0030282	0.0123	0.999	2	37	bone mineralization	BP	0.813	HOXA3,ASPN	down_ACOR_EC
GO:0042092	0.0123	1	1	11	type 2 immune response	BP	0.945	ARG2	up_AORT_VSMC
GO:0001779	0.0123	1	1	3	natural killer cell differentiation	BP	1	TOX	up_VFEM_EC
GO:0021520	0.0123	1	1	3	spinal cord motor neuron cell fate specification	BP	1	HOXC10	up_VFEM_EC
GO:0031424	0.0123	1	1	3	keratinization	BP	1	CYP26B1	up_VFEM_EC
GO:0032823	0.0123	1	1	3	regulation of natural killer cell differentiation	BP	1	TOX	up_VFEM_EC

GO:0032268	0.012300452	0.99303	31	778	regulation of cellular protein metabolic process	BP	0.96436359	PLCL1,PKIB,SOX9,PCSK1N,NDRG2,ENSCAFG00000004331,EDEM2,FZD5,IFI6,DUSP26,NOD2,SMAD6,KLHL25,PAK3,TNFRSF19,CST3,PIAS1,MAPK8IP2,MAVS,LRRC15,LRP4,SH3RF3,LRRC4,CLU,RARRES2,RNF114,NRP1,PRKCD,PYGO2,DBNDD2,CCNYL1	up_ACOR_EC
GO:0071260	0.012337874	0.99874	3	19	cellular response to mechanical stimulus	BP	0.96436359	SOX9,AQP1,PTGER4	up_ACOR_EC
GO:0048469	0.0124	0.997	7	31	cell maturation	BP	0.47	EDNRB,GATA3,NTN4,SOX18,SPTBN4,HIF1A,FBXO5	up_VCAV_EC
GO:0007095	0.0124	0.998	4	11	mitotic G2 DNA damage checkpoint	BP	0.52	CDK1,FANCI,TOPBP1,CLSPN	down_ACOR_VSMC
GO:0071901	0.0124	0.999	2	37	negative regulation of protein serine/threonine	BP	0.813	LYN,PRKAR2A	down_ACOR_EC
GO:1902993	0.0124	1	1	3	positive regulation of amyloid precursor protein catabolic process	BP	0.813	LYN	down_ACOR_EC
GO:0060038	0.0124	1	1	18	cardiac muscle cell proliferation	BP	1	TBX5	up_APUL_VSMC
GO:0021800	0.0124	1	1	6	cerebral cortex tangential migration	BP	1	SLIT2	up_VPUL_VSMC
GO:0009987	0.0125	0.99	448	5086	cellular process	BP	0.524	ELK3,SCD,ACSL3,PRPSAP1,FSCN1,HMGB1,MCM6,RNPS1,SREBF1,ACLY,MCM4,FLNB,POLE3,USP1,IRAK1,MCM7,RPA1,NCAPD2,ANLN,PTBP1,ILF3,DDX39A,STXBP6,CBX5,ENSCAFG00000016095,PPRC1,USP4,FMNL3,RACGAP1,SF3B2,ACSL4,BCAT1,ATAD2,HMGA1,EIF4A3,HMGCR,HNRNPUL1,EDC3,NNT,TFPI,HNRNPD,SREBF2,MCM3,SMC1A,HINFP,VARS,KIF2A,UBE2S,NEDD4,SRSF2,RRM1,WDR76,THOP1,MED17,U2AF2,ZW10,INTS7,QSOX2,ENSCAFG00000016482,MNDA,CAPG,SMC4,CENPA,PSMD3,POLA1,PSMD7,SLC29A1,MTDH,HEY1,METAP2,HOXA3,ENSCAFG00000017264,NDC1,PPM1G,RRBP1,SNRPB2,CENPN,TOP3A,TRIP13,COLGALT1,SLC25A5,PPP1R15B,TIMM44,SF1,PARPBP,SGO1,POLA2,MPDU1,SRRT,GINS4,C1QTNF6,NOP56,SDC1,ARFGAP1,CTDNEP1,RAD54L,RANGAP1,POLE,FBXO5,DDX23,HSPA9,NAB2,SLC35C2,HNRNPH3,MRPS18B,RAN,B3GNT2,CFAP298,PRELID1,HM13,UBE2T,PSMD13,CDCA7L,FUS,RFC2,BIRC5,MYBBP1A,INSIG1,PRMT1,ENSCAFG00000030005,CAV1,CDK1,RFC5,CENPE,RFC3,RAD51,CASP2,FANCI,XYLT1,FANCD2,U2AF1,TIMELESS,THRAP3,LIMA1,TRUB2,ENSCAFG00000018572,FAM129B,TCEA1,TXLNA,ENSCAFG00000008236,NF2,ANPEP,PFAS,	down_ACOR_VSMC
GO:0042221	0.0125	0.993	35	1012	response to chemical	BP	1	CMKLR1,MT2A,LEP,LBH,CRY2,ENSCAFG00000009113,RAB20,GAS1,ANGPT4,CD40,DLL1,CRYAB,CPNE7,PLXNA4,GPAM,FIBIN,CHRM2,TMEM100,GREM1,NFE2L1,AVPR1A,CAMK2A,GREM2,CYLD,POSTN,LRRC15,NR3C1,TBXA2R,ACKR4,PPARGC1A,CCL5,ADAM17,KANK1,ANXA11,SOD1	up_AFEM_EC
GO:0048513	0.0125	0.993	32	906	animal organ development	BP	1	TBX15,HOXA9,IRX3,IRX5,HOXC10,LEP,LBH,XYLT1,HOXA7,COL8A2,DLL1,CRYAB,PLXNA4,MAB21L1,THRB,TMEM100,GREM1,NFE2L1,OVOL1,HOXA6,CYLD,LUZP1,PTHLH,EVI2B,CNNM4,BVES,AXIN2,ACACB,PPARGC1A,GCNT4,FOSL2,TNC	up_AFEM_EC
GO:0072001	0.0125	0.995	11	104	renal system development	BP	1	SMAD9,KANK2,CLSTN1,PKD1,GLI3,GLIS2,PRKCSH,RDH10,GREM1,ZBTB16,SEC61A1	down_VCAV_EC
GO:0051241	0.0125	0.998	5	336	negative regulation of multicellular organismal	BP	1	HOXC10,HOXA9,MAP2K6,HOXA7,SIX1	up_VFEM_EC
GO:0032486	0.0125	0.999	3	6	Rap protein signal transduction	BP	0.472	RAPGEF1,RAP1A,RAPGEF2	up_VCAV_EC
GO:0014706	0.0125	0.999	3	112	striated muscle tissue development	BP	0.943	PITX1,CAV1,SHOX2	up_VSAP_VSMC
GO:0010033	0.0125	1	2	772	response to organic substance	BP	1	JUP,GATA5	down_VFEM_VSMC
GO:0048634	0.0125	0.999	2	40	regulation of muscle organ development	BP	1	SIX1,CYP26B1	up_VFEM_EC
GO:0030336	0.0125	0.999	2	86	negative regulation of cell migration	BP	1	TBX5,SLIT2	up_VPUL_VSMC
GO:0042998	0.0125	1	1	3	positive regulation of Golgi to plasma membrane protein	BP	0.814	ACSL3	down_ACOR_EC
GO:0061162	0.0125	1	1	6	establishment of monopolar cell polarity	BP	1	FSCN1	down_AFEM_EC

GO:0061339	0.0125	1	1	6	establishment or maintenance of	BP	1	FSCN1	down_AFEM_EC
GO:2000482	0.0125	1	1	9	regulation of interleukin-8 secretion	BP	1	TLR2	up_AFEM_VSMC
GO:0001541	0.0125	1	1	12	ovarian follicle development	BP	0.95	ESR1	up_AORT_VSMC
GO:0001709	0.0125	1	1	11	cell fate determination	BP	0.95	WNT7A	up_AORT_VSMC
GO:0048846	0.0125	1	1	6	axon extension involved in axon guidance	BP	1	SLIT2	up_VPUL_VSMC
GO:1902284	0.0125	1	1	6	neuron projection extension involved in neuron projection	BP	1	SLIT2	up_VPUL_VSMC
GO:0023057	0.012574299	0.99389	19	409	negative regulation of signaling	BP	0.96436359	NR1H4,SOX9,NDRG2,KCNB1,CCDC3,IFI6,DUSP26,PTGIR,SMAD6,ADAMTSL2,MAPK8IP2,FIGNL1,LRRC15,LRP4,CLSTN1,LRRC4,CLU,NRP1,PRKCD	up_ACOR_EC
GO:0061351	0.0126	0.996	10	50	neural precursor cell proliferation	BP	0.473	EPHB1,DLL4,RAPGEF1,HOOK3,HHEX,PROX1,CTNNA1,HIF1A,ARHGEF2,CDON	up_VCAV_EC
GO:1903409	0.0126	0.997	6	24	reactive oxygen species biosynthetic process	BP	0.473	CD34,TLR4,NOS3,RAB27A,ARG2,NOS2	up_VCAV_EC
GO:0051254	0.0126	0.999	3	431	positive regulation of RNA metabolic process	BP	0.95	HOXA9,WNT7A,ESR1	up_AORT_VSMC
GO:0070887	0.0126	1	2	775	cellular response to chemical stimulus	BP	1	JUP,GATA5	down_VFEM_VSMC
GO:0060840	0.0126	0.999	2	42	artery development	BP	1	EYA1,SIX1	up_VFEM_EC
GO:0021515	0.0126	1	1	9	cell differentiation in spinal cord	BP	1	HOXC10	up_AFEM_VSMC
GO:0060260	0.0126	1	1	12	regulation of transcription initiation from RNA polymerase II promoter	BP	0.95	ESR1	up_AORT_VSMC
GO:0060872	0.0126	1	1	6	semicircular canal development	BP	1	EYA1	up_VFEM_VSMC
GO:0031399	0.012681148	0.99331	24	559	regulation of protein modification process	BP	0.96436359	PLCL1,PKIB,SOX9,NDRG2,ENSCAFG00000004331,FZD5,DUSP26,NOD2,SMAD6,PAK3,TNFRSF19,PIAS1,MAPK8IP2,MAVS,LRRC15,LRP4,SH3RF3,LRRC4,RARRES2,NRP1,PRKCD,PYGO2,DBNDD2,CCNYL1	up_ACOR_EC
GO:0060828	0.0127	0.997	6	82	regulation of canonical Wnt signaling pathway	BP	1	GREM1,FAM53B,CYLD,RECK,AXIN2,KANK1	up_AFEM_EC
GO:0008652	0.0127	0.998	5	24	cellular amino acid biosynthetic process	BP	0.546	ASNS,PLOD2,ALDH18A1,PLOD3,BCAT1	down_VSAP_EC
GO:1901607	0.0127	0.998	5	24	alpha-amino acid biosynthetic process	BP	0.546	ASNS,PLOD2,ALDH18A1,PLOD3,BCAT1	down_VSAP_EC
GO:0003008	0.0127	0.997	5	336	system process	BP	1	HOXC10,MAP2K6,MME,AVPR1A,SIX1	up_VFEM_EC
GO:0055086	0.0127	0.999	3	237	nucleobase-containing small molecule metabolic	BP	1	EXTL2,SUCLG2,NDUFA10	down_AFEM_EC
GO:0006935	0.0127	0.999	3	143	chemotaxis	BP	1	CCL24,FLRT3,NEO1	up_VPUL_EC
GO:0042330	0.0127	0.999	3	143	taxis	BP	1	CCL24,FLRT3,NEO1	up_VPUL_EC
GO:0000394	0.0127	1	1	6	RNA splicing, via endonucleolytic cleavage	BP	1	TSEN15	down_AFEM_EC
GO:0006388	0.0127	1	1	6	tRNA splicing, via endonucleolytic cleavage	BP	1	TSEN15	down_AFEM_EC
GO:1903055	0.0127	1	1	6	positive regulation of extracellular matrix	BP	1	FSCN1	down_AFEM_EC
GO:2000794	0.0127	1	1	6	regulation of epithelial cell proliferation involved in lung morphogenesis	BP	1	SRSF6	down_AFEM_EC
GO:0007252	0.0127	1	1	6	I-kappaB phosphorylation	BP	1	ERC1	up_AORT_EC
GO:0000578	0.0127	1	1	11	embryonic axis specification	BP	0.95	WNT7A	up_AORT_VSMC
GO:0021846	0.0127	1	1	11	cell proliferation in forebrain	BP	0.95	WNT7A	up_AORT_VSMC
GO:0051932	0.0127	1	1	9	synaptic transmission, GABAergic	BP	1	CA2	up_VCAV_VSMC
GO:0051093	0.0128	0.995	13	273	negative regulation of developmental process	BP	1	HOXA9,IRX3,LBH,ANGPT4,HOXA7,DLL1,TWIST2,THRB,GREM1,CYLD,TBXA2R,AXIN2,KANK1	up_AFEM_EC
GO:0009108	0.0128	0.995	13	68	coenzyme biosynthetic process	BP	1	ACACB,NADK,ENSCAFG00000013986,COQ9,PDK2,GALK1,MOCOS,PCBD1,ACSS2,PNPO,DCAKD,PNP,DDIT4	up_VSAP_EC
GO:0030534	0.0128	0.996	8	33	adult behavior	BP	1	LEP,GLRB,SLITRK5,APP,CHD7,CLN8,OXR1,PARK7	up_ACOR_VSMC
GO:0006612	0.0128	0.997	6	35	protein targeting to membrane	BP	0.546	TRAM1,SRP54,MYO1C,SSR1,SRPRA,SRP72	down_VSAP_EC

GO:0000902	0.0128	0.998	4	266	cell morphogenesis	BP	1	CCL24,FLRT3,ARHGEF2,NEO1	up_VPUL_EC
GO:0098868	0.0128	0.999	3	11	bone growth	BP	1	THBS3,EVC,DDR2	down_VCAV_EC
GO:0060537	0.0128	0.999	3	117	muscle tissue development	BP	1	EYA1,SIX1,CYP26B1	up_VFEM_EC
GO:0009653	0.0128	1	2	773	anatomical structure morphogenesis	BP	1	JUP,GATA5	down_VFEM_VSMC
GO:0035507	0.0128	1	2	3	regulation of myosin-light-chain-phosphatase	BP	0.546	NUAK1,PPP1R12A	down_VSAP_EC
GO:0071219	0.0128	0.999	2	50	cellular response to molecule of bacterial	BP	1	NR1H4,MRC1	up_VPUL_EC
GO:0031128	0.0128	1	1	15	developmental induction	BP	1	GATA5	down_VSAP_VSMC
GO:2000144	0.0128	1	1	12	positive regulation of DNA-templated transcription,	BP	0.95	ESR1	up_AORT_VSMC
GO:0051248	0.0129	0.993	29	316	negative regulation of protein metabolic process	BP	0.549	SERPINE1,SOCS5,ENPP1,ERRFI1,SNX25,TRIB3,SESN2,PTGS2,DNAJC10,NXN,PTPN2,UCHL5,SPOPL,EIF4EBP1,CNOT7,CRYAB,APOE,LYN,STRAP,CBLC,ATF3,EIF2S1,CDYL,PAK2,A2M,CDKN1A,SPINK2,USP14,KDM3A	down_VSAP_EC
GO:1902107	0.0129	0.996	9	47	positive regulation of leukocyte differentiation	BP	0.483	HCLS1,IL7R,GATA3,CASP8,ITPKB,CREB1,HMGB1,RIPK1,ATP11C	up_VCAV_EC
GO:0007080	0.0129	0.998	5	18	mitotic metaphase plate congression	BP	0.536	ZW10,KIF22,CDCA8,NDC80,NUP62	down_ACOR_VSMC
GO:0048639	0.0129	0.998	4	39	positive regulation of developmental growth	BP	1	LEP,DLL1,GPAM,ACACB	up_AFEM_EC
GO:0042310	0.0129	0.999	3	21	vasoconstriction	BP	1	HTR1D,AVPR1A,TBXA2R	up_AFEM_EC
GO:0045851	0.0129	0.999	3	21	pH reduction	BP	1	RAB20,RAB7A,ATP6V0B	up_AFEM_EC
GO:0097756	0.0129	0.999	3	21	negative regulation of blood vessel diameter	BP	1	HTR1D,AVPR1A,TBXA2R	up_AFEM_EC
GO:0060037	0.0129	1	1	9	pharyngeal system development	BP	1	EYA1	up_AFEM_VSMC
GO:0071493	0.0129	1	1	3	cellular response to UV-B	BP	1	MME	up_VFEM_EC
GO:0072095	0.0129	1	1	3	regulation of branch elongation involved in ureteric bud branching	BP	1	SIX1	up_VFEM_EC
GO:0021520	0.0129	1	1	3	spinal cord motor neuron cell fate specification	BP	0.968	HOXC10	up_VSAP_VSMC
GO:0003180	0.012982199	0.99944	2	7	aortic valve morphogenesis	BP	0.96436359	GATA4,SMAD6	up_ACOR_EC
GO:0008150	0.013	0.991	353	5646	biological_process	BP	0.552	ARHGEF2,COL15A1,PRICKLE1,VEGFA,P4HB,SIPA1L1,SULF1,ARHGAP31,CELF1,ASNS,SERPINH1,CARS,PLOD2,MYH9,SLC39A7,SHROOM2,TAGLN2,NR1H4,FMNL3,PLK3,C1QTNF6,ENSCAFG00000013214,HSPA5,SERPINE1,ACTN1,SOCS5,ETV6,HM13,SPARC,VASP,ANKRD50,GOLT1B,TRAM1,ENSCAFG00000009211,MEIS2,LTBP1,SGK1,ERO1A,PRKACB,HSP90B1,PTPN14,PTK7,SLC7A5,ENPP1,ERRFI1,SLC1A4,ENSCAFG00000005575,ITGA2,P4HA1,SEC24D,JCAD,TPM4,SNX25,CBX5,CASP3,FAM49B,SARS,ENSCAFG00000003477,HYOU1,PGK1,AKAP2,WSB1,BCAR3,NUAK1,PTPN11,EXOC5,ATP2B1,CORO1C,FSCN1,AARS,FLNB,SEC23A,MZT1,COTL1,TPI1,ENSCAFG00000032470,DPYSL3,ENSCAFG00000028814,LRRC8A,HOOK3,SLC38A1,TRIB3,PGAM1,IFRD1,FARP1,ENSCAFG00000007260,TMED2,PUM3,FKBP11,UGCG,PM20D2,FBLIM1,ILK,FHL2,BHLHE40,ALDH1L2,PPP1R12A,PRCP,SMARCA4,ANKRA2,SESN2,SRP54,TGFBR1,PTGS2,TMEM87B,REM1,WDR1,MYO1C,TMTC2,ENSCAFG00000031588,CANX,PPIB,CEMIP2,PIK3C2A,CHSY1,MSN,RAB6A,UACA,HOXB7,SEL1L,RPGR,KIF5B,ENSCAFG00000008716,LMAN1,HIF1A,HTR7,NDUFA4L2,FKBP10,CX3CL1,ENSCAFG00000000562,DYNLL1,TRA	down_VSAP_EC
GO:0009790	0.013	0.994	15	335	embryo development	BP	1	TBX15,HOXA9,IRX3,IRX5,HOXC10,XYLT1,HOXA7,DLL1,TMEM100,GREM1,GREM2,HOXA6,LUZP1,RECK,AXIN2	up_AFEM_EC
GO:0009749	0.013	0.996	8	34	response to glucose	BP	1	HNF4A,RAB11B,LPL,KCNB1,RAF1,STX4,PPP3CB,RAB11FIP2	up_VSAP_EC
GO:0019932	0.013	0.997	6	82	second-messenger-mediated signaling	BP	1	CMKLR1,LMCD1,TMEM100,PTHLH,TBXA2R,DMPK	up_AFEM_EC
GO:0040017	0.013	0.998	4	180	positive regulation of locomotion	BP	0.835	LYN,IGF1R,PDPN,STX4	down_ACOR_EC

GO:0015985	0.013	0.998	4	13	energy coupled proton transport, down	BP	0.537	CYC1,ATP5F1A,ENSCAFG00000013366,ATP5F1D	down_ACOR_VSMC
GO:0015986	0.013	0.998	4	13	ATP synthesis coupled proton transport	BP	0.537	CYC1,ATP5F1A,ENSCAFG00000013366,ATP5F1D	down_ACOR_VSMC
GO:0051271	0.013	0.999	3	98	negative regulation of cellular component	BP	0.835	ACVRL1,HOXA7,SULF1	down_ACOR_EC
GO:0043067	0.013	0.999	3	479	regulation of programmed cell death	BP	0.964	WNT7A,ARG2,ESR1	up_AORT_VSMC
GO:0007015	0.013	0.999	3	144	actin filament organization	BP	1	MYBPC2,CCL24,ARHGEF2	up_VPUL_EC
GO:0003012	0.013	1	1	90	muscle system process	BP	1	GATA5	down_AFEM_VSMC
GO:0043535	0.013	1	1	45	regulation of blood vessel endothelial cell migration	BP	1	JUP	down_VFEM_VSMC
GO:0045216	0.013	1	1	44	cell-cell junction organization	BP	1	JUP	down_VFEM_VSMC
GO:0060575	0.013	1	1	9	intestinal epithelial cell differentiation	BP	1	GATA5	up_VCAV_VSMC
GO:1905523	0.013	1	1	6	positive regulation of macrophage migration	BP	1	C3AR1	up_VPOR_VSMC
GO:0030279	0.01308022	0.99863	3	19	negative regulation of ossification	BP	0.96436359	SOX9,SMAD6,LRP4	up_ACOR_EC
GO:0003181	0.013091447	0.99943	2	7	atrioventricular valve morphogenesis	BP	0.96436359	SMAD6,TBX5	up_ACOR_EC
GO:0034641	0.0131	0.999	5	2171	cellular nitrogen compound metabolic	BP	1	AK5,GATA5,TGFBR1,MEIS2,ENSCAFG00000028814	down_VSAP_VSMC
GO:0010556	0.0131	0.998	5	1137	regulation of macromolecule	BP	1	TLR2,EYA1,HOXA9,HOXA10,IRX5	up_AFEM_VSMC
GO:1901362	0.0131	0.999	4	1293	organic cyclic compound biosynthetic process	BP	1	AK5,GATA5,TGFBR1,MEIS2	down_VSAP_VSMC
GO:0046425	0.0131	0.999	2	39	regulation of JAK-STAT cascade	BP	0.835	LYN,ASPN	down_ACOR_EC
GO:0061196	0.0131	1	2	3	fungiform papilla development	BP	0.552	CTNNB1,SIX4	down_VSAP_EC
GO:0061197	0.0131	1	2	3	fungiform papilla morphogenesis	BP	0.552	CTNNB1,SIX4	down_VSAP_EC
GO:0034379	0.0131	1	1	3	very-low-density lipoprotein particle	BP	0.835	ACSL3	down_ACOR_EC
GO:0048025	0.0131	1	1	6	negative regulation of mRNA splicing, via	BP	1	SRSF6	down_AFEM_EC
GO:0051491	0.0131	1	1	6	positive regulation of filopodium assembly	BP	1	FSCN1	down_AFEM_EC
GO:1901071	0.0131	1	1	6	glucosamine-containing compound metabolic	BP	1	EXTL2	down_AFEM_EC
GO:0055017	0.0131	1	1	19	cardiac muscle tissue growth	BP	1	TBX5	up_APUL_VSMC
GO:0003176	0.0131	1	1	9	aortic valve development	BP	1	GATA5	up_VCAV_VSMC
GO:0019438	0.0132	0.996	11	1258	aromatic compound biosynthetic process	BP	0.986	HOXA11,TBX15,HOXA10,HOXA9,PITX1,EMX2,SALL1,IRX5,TLR2,CAV1,SHOX2	up_VSAP_VSMC
GO:0070887	0.0132	0.997	7	775	cellular response to chemical stimulus	BP	1	NR1H4,MRC1,CCL24,PTGER2,FLRT3,ARHGEF2,NEO1	up_VPUL_EC
GO:0043124	0.0132	0.998	5	17	negative regulation of I-kappaB kinase/NF-kappaB	BP	0.489	TNFAIP3,TNIP1,CASP8,RIOK3,RIPK1	up_VCAV_EC
GO:0048729	0.0132	0.999	3	209	tissue morphogenesis	BP	1	EYA1,HOXA11,SHOX2	up_VFEM_VSMC
GO:0060284	0.0132	0.999	3	212	regulation of cell development	BP	1	EYA1,HOXA11,SHOX2	up_VFEM_VSMC
GO:0061041	0.0132	0.999	2	34	regulation of wound healing	BP	0.835	LYN,PDPN	down_ACOR_EC
GO:1905477	0.0132	0.999	2	32	positive regulation of protein localization to	BP	0.835	ACSL3,STX4	down_ACOR_EC
GO:0032504	0.0132	0.999	2	152	multicellular organism reproduction	BP	0.965	HOXA9,ESR1	up_AORT_VSMC
GO:0048609	0.0132	0.999	2	152	multicellular organismal reproductive process	BP	0.965	HOXA9,ESR1	up_AORT_VSMC
GO:1901861	0.0132	0.999	2	41	regulation of muscle tissue development	BP	1	SIX1,CYP26B1	up_VFEM_EC
GO:0021563	0.0132	1	1	4	glossopharyngeal nerve development	BP	1	HOXA3	up_APUL_EC
GO:0071955	0.0132	1	1	2	recycling endosome to Golgi transport	BP	1	GCC2	up_VPOR_EC
GO:0048387	0.0132	1	1	3	negative regulation of retinoic acid receptor	BP	1	CYP26B1	up_VFEM_EC
GO:0006775	0.0132	1	1	3	fat-soluble vitamin metabolic process	BP	1	CYP26B1	up_VFEM_EC
GO:0050727	0.0133	0.995	12	74	regulation of inflammatory response	BP	0.491	GBP5,TNFAIP3,TNIP1,BCL6B,TLR4,GATA3,TLR3,APOD,PTGS2,NOS2,NR1D2,ENSCAFG00000025410	up_VCAV_EC

GO:0002274	0.0133	0.996	8	40	myeloid leukocyte activation	BP	0.491	ADGRF5,KIT,CXCL8,TLR4,NECTIN2,SNAP23,CD200,FES	up_VCAV_EC
GO:0006720	0.0133	0.997	6	25	isoprenoid metabolic process	BP	0.547	HMGCR,ENSCAFG00000018572,MVD,FDFT1,NUS1,PDSS1	down_ACOR_VSMC
GO:0046173	0.0133	0.997	5	19	polyol biosynthetic process	BP	0.491	SPTLC2,ITPKB,PLCG2,GCH1,IMPA1	up_VCAV_EC
GO:0048871	0.0133	0.999	3	120	multicellular organismal homeostasis	BP	1	HOXC10,MAP2K6,CYP26B1	up_VFEM_EC
GO:0051216	0.0133	0.999	2	70	cartilage development	BP	1	HOXA11,SHOX2	up_VFEM_VSMC
GO:0098742	0.0133	1	1	44	cell-cell adhesion via plasma-membrane	BP	1	GATA5	down_VFEM_VSMC
GO:0071599	0.0133	1	1	9	otic vesicle development	BP	1	EYA1	up_AFEM_VSMC
GO:0030812	0.0133	1	1	5	negative regulation of nucleotide catabolic	BP	1	FBP1	up_VFEM_VSMC
GO:0045820	0.0133	1	1	5	negative regulation of glycolytic process	BP	1	FBP1	up_VFEM_VSMC
GO:0051195	0.0133	1	1	5	negative regulation of cofactor metabolic	BP	1	FBP1	up_VFEM_VSMC
GO:0051198	0.0133	1	1	5	negative regulation of coenzyme metabolic	BP	1	FBP1	up_VFEM_VSMC
GO:0071600	0.0133	1	1	5	otic vesicle morphogenesis	BP	1	EYA1	up_VFEM_VSMC
GO:0032879	0.0134	0.99	92	767	regulation of localization	BP	1	EXPH5,SH3TC2,FGF13,VEGFD,TLR1,DNAJC6,ACTN2,LGR6,PLCG2,LEP,KCNE3,HTR2B,TCAF1,USP6NL,MBP,FBXL20,RECK,PRKAA2,FERMT1,BMP4,EVI5L,TM7SF3,ABCA1,GJA1,SH3RF2,SEC24B,TP53INP1,ATXN2,CD274,AR,SARAF,SASH1,LMO4,PTPN3,FGF18,ITPR1,ANKRD1,F2RL1,DAPK1,CACNB3,AKAP6,CDKN1A,NRP1,PTPN22,LRPAP1,DMPK,OS9,SCP2,EVL,SPINT2,KCNJ4,TBX5,PRKCE,SCG5,DAG1,PARP9,APP,LAMA3,PSEN1,SEMA6D,SGSM2,SYNJ2BP,CHD7,JUP,CCL5,DDR2,DUSP22,RAB11B,EDN1,GTF2A1L,KCNC4,NOD2,FAM107A,JAM3,B2M,FXYD1,SCAMP5,INPP5K,RAB5C,PREPL,PFKL,WFS1,AZIN2,WASL,DOCK5,PARK7,STC2,BVES,RREB1,CRKL,PIK3C2A,PTPN14	up_ACOR_VSMC
GO:0002521	0.0134	0.993	23	149	leukocyte differentiation	BP	1	HOXA7,PPARGC1B,ABL1,DLL1,ZBTB16,TOX,STAT6,PATZ1,F2RL1,ZFP36L1,PIK3R1,BATF2,MYC,ZEB1,TFE3,FBXO7,CD83,ZFP36L2,PNP,PPP3CB,TGFBR2,CITED2,PIR	up_VSAP_EC
GO:0098542	0.0134	0.994	15	100	defense response to other organism	BP	0.491	LYZ,MX2,TMEM173,TLR4,NLRC5,IRF8,SERINC5,ARG2,RIOK3,TLR3,OAS3,CNOT7,NOS2,PMAIP1,LSM14A	up_VCAV_EC
GO:0030154	0.0134	0.995	11	1040	cell differentiation	BP	0.835	ACVRL1,LYN,ACSL4,SPG11,PDPN,NRP2,HOXA7,ANPEP,SULF1,ELK3,NEDD4	down_ACOR_EC
GO:0048731	0.0134	0.996	10	1247	system development	BP	1	NR1H4,TBX5,MAPK10,HOXA3,ZDHHC16,SLITRK5,NPNT,NTRK2,HOXA1,PDLIM3	up_APUL_EC
GO:1901998	0.0134	0.998	4	16	toxin transport	BP	0.558	HSPA5,COPB2,BNIP3,NRP1	down_VSAP_EC
GO:0000414	0.0134	1	2	2	regulation of histone H3-K36 methylation	BP	0.491	IWS1,NSD1	up_VCAV_EC
GO:0090091	0.0134	1	1	3	positive regulation of extracellular matrix	BP	0.835	PDPN	down_ACOR_EC
GO:0060485	0.0134	1	1	93	mesenchyme development	BP	1	GATA5	down_AFEM_VSMC
GO:0060602	0.0134	1	1	12	branch elongation of an epithelium	BP	0.977	ESR1	up_AORT_VSMC
GO:0007512	0.0134	1	1	6	adult heart development	BP	1	NKX2-5	up_VPOR_VSMC
GO:1902624	0.0134	1	1	7	positive regulation of neutrophil migration	BP	1	C3AR1	up_VPOR_VSMC
GO:0030854	0.0134	1	1	4	positive regulation of granulocyte	BP	1	EVI2B	up_VPUL_EC
GO:0001649	0.0134894	0.99719	5	54	osteoblast differentiation	BP	0.96436359	PTH1R,SMAD6,FIGNL1,CLSTN1,TP53INP2	up_ACOR_EC
GO:0010468	0.0135	0.995	11	1270	regulation of gene expression	BP	0.993	HOXA11,HOXA10,HOXA9,PITX1,EMX2,SALL1,IRX5,PRR16,TLR2,CAV1,SHOX2	up_VSAP_VSMC
GO:0018130	0.0135	0.995	11	1261	heterocycle biosynthetic process	BP	0.993	HOXA11,TBX15,HOXA10,HOXA9,PITX1,EMX2,SALL1,IRX5,TLR2,CAV1,SHOX2	up_VSAP_VSMC
GO:0010035	0.0135	0.996	7	107	response to inorganic substance	BP	1	MT2A,ENSCAFG00000009113,CRYAB,CPNE7,FIBIN,ANXA11,SOD1	up_AFEM_EC
GO:0009653	0.0135	0.997	7	773	anatomical structure morphogenesis	BP	1	TBX5,HOXA3,MYBPC2,CCL24,FLRT3,ARHGEF2,NEO1	up_VPUL_EC
GO:1902117	0.0135	0.997	6	32	positive regulation of organelle assembly	BP	0.562	FSCN1,MSN,DYNLL1,SH3GLB1,SDC4,HTT	down_VSAP_EC

GO:1901701	0.0135	0.998	4	271	cellular response to oxygen-containing	BP	1	NR1H4,MRC1,PTGER2,ARHGEF2	up_VPUL_EC
GO:0040013	0.0135	0.999	3	100	negative regulation of locomotion	BP	0.835	ACVRL1,HOXA7,SULF1	down_ACOR_EC
GO:0032530	0.0135	1	1	6	regulation of microvillus organization	BP	1	FSCN1	down_AFEM_EC
GO:0071803	0.0135	1	1	6	positive regulation of podosome assembly	BP	1	FSCN1	down_AFEM_EC
GO:0009612	0.0135	1	1	47	response to mechanical stimulus	BP	1	JUP	down_VFEM_VSMC
GO:0033598	0.0135	1	1	11	mammary gland epithelial cell proliferation	BP	0.977	ESR1	up_AORT_VSMC
GO:0051123	0.0135	1	1	14	RNA polymerase II preinitiation complex	BP	0.977	ESR1	up_AORT_VSMC
GO:0003179	0.0135	1	1	19	heart valve morphogenesis	BP	1	TBX5	up_APUL_VSMC
GO:0021545	0.0135	1	1	21	cranial nerve development	BP	1	HOXA3	up_APUL_VSMC
GO:0048168	0.0135	1	1	5	regulation of neuronal synaptic plasticity	BP	1	NETO1	up_VFEM_VSMC
GO:0071941	0.0135	1	1	4	nitrogen cycle metabolic process	BP	1	NR1H4	up_VPUL_EC
GO:0098543	0.0135	1	1	4	detection of other organism	BP	0.993	TLR2	up_VSAP_VSMC
GO:0016045	0.0135	1	1	4	detection of bacterium	BP	0.993	TLR2	up_VSAP_VSMC
GO:0048569	0.013509381	0.9994	2	7	post-embryonic animal organ development	BP	0.96436359	FZD5,EFEMP1	up_ACOR_EC
GO:0006654	0.013569774	0.9994	2	7	phosphatidic acid biosynthetic process	BP	0.96436359	NR1H4,AGPAT2	up_ACOR_EC
GO:0046473	0.013569774	0.9994	2	7	phosphatidic acid metabolic process	BP	0.96436359	NR1H4,AGPAT2	up_ACOR_EC
GO:0030277	0.013587612	0.9994	2	7	maintenance of gastrointestinal	BP	0.96436359	SOX9,NOD2	up_ACOR_EC
GO:0045862	0.0136	0.994	13	112	positive regulation of proteolysis	BP	0.563	PRICKLE1,MYH9,PLK3,SOCS5,TRIB3,APOE,LYN,FAM162A,GSK3B,REST,DISC1,RAD23A,TMTC3	down_VSAP_EC
GO:0031032	0.0136	0.995	11	61	actomyosin structure organization	BP	0.497	ARHGEF15,MEF2C,EDN1,ARHGAP28,TMOD1,MTOR,PROX1,EPB41,STMN1,TNFAIP1,TMOD3	up_VCAV_EC
GO:0021514	0.0136	1	2	4	ventral spinal cord interneuron	BP	1	LMO4,GLI3	down_VCAV_EC
GO:0048608	0.0136	0.999	2	129	reproductive structure development	BP	1	HOXA9,IRX5	up_AFEM_VSMC
GO:0048704	0.0136	0.999	2	46	embryonic skeletal system morphogenesis	BP	1	HOXA3,HOXA1	up_APUL_EC
GO:0015682	0.0136	1	1	7	ferric iron transport	BP	1	RAB11B	down_VPOR_EC
GO:0072512	0.0136	1	1	7	trivalent inorganic cation transport	BP	1	RAB11B	down_VPOR_EC
GO:0003197	0.0136	1	1	16	endocardial cushion development	BP	1	GATA5	down_VSAP_VSMC
GO:0003161	0.0136	1	1	6	cardiac conduction system development	BP	1	NKX2-5	up_VPOR_VSMC
GO:0055062	0.0136	1	1	5	phosphate ion homeostasis	BP	1	HOXA3	up_VPOR_VSMC
GO:0072505	0.0136	1	1	5	divalent inorganic anion homeostasis	BP	1	HOXA3	up_VPOR_VSMC
GO:0072506	0.0136	1	1	5	trivalent inorganic anion homeostasis	BP	1	HOXA3	up_VPOR_VSMC
GO:0045617	0.0136	1	1	3	negative regulation of keratinocyte	BP	1	HOXA7	up_VFEM_EC
GO:0006521	0.0136	1	1	4	regulation of cellular amino acid metabolic	BP	1	NR1H4	up_VPUL_EC
GO:0032731	0.013637571	0.99939	2	7	positive regulation of interleukin-1 beta	BP	0.96436359	FZD5,NOD2	up_ACOR_EC
GO:0032232	0.013656058	0.99939	2	7	negative regulation of actin filament bundle	BP	0.96436359	ARHGAP6,ARHGAP28	up_ACOR_EC
GO:0051497	0.013656058	0.99939	2	7	negative regulation of stress fiber assembly	BP	0.96436359	ARHGAP6,ARHGAP28	up_ACOR_EC
GO:0048519	0.0137	0.995	14	1554	negative regulation of biological process	BP	0.835	ACVRL1,LYN,HOXA3,IGF1R,PDPN,HOXA7,PRKAR2A,USP28,ATRIP,SULF1,ELK3,MYO1D,ASPN,NEDD4	down_ACOR_EC
GO:0043542	0.0137	0.995	11	86	endothelial cell migration	BP	0.564	VEGFA,MYH9,SPARC,JCAD,PRCP,PIK3C2A,HIF1A,ADAM17,STARD13,APOE,NRP1	down_VSAP_EC
GO:0032675	0.0137	0.996	8	34	regulation of interleukin-6 production	BP	1	C5AR2,IL6R,IL17RC,F2RL1,MYD88,NLRX1,CYBA,MAVS	up_VSAP_EC
GO:0048869	0.0137	0.997	7	1119	cellular developmental process	BP	1	HOXC10,EYA1,HOXA9,SPINK5,EMX2,HOXA11,SHOX2	up_VFEM_VSMC

GO:0019219	0.0137	0.998	5	1151	regulation of nucleobase-containing compound metabolic process	BP	1	TLR2,EYA1,HOXA9,HOXA10,IRX5	up_AFEM_VSMC
GO:0051094	0.0137	0.998	4	388	positive regulation of developmental process	BP	1	NKX2-5,BAMBI,CYP26B1,C3AR1	up_VPOR_VSMC
GO:0031145	0.0137	0.999	3	7	anaphase-promoting complex-dependent	BP	0.557	UBE2S,CDC20,UBE2C	down_ACOR_VSMC
GO:0043486	0.0137	0.999	3	7	histone exchange	BP	0.557	OIP5,CENPP,MIS18A	down_ACOR_VSMC
GO:0061548	0.0137	0.999	3	8	ganglion development	BP	0.564	CTNNB1,SIX4,NRP1	down_VSAP_EC
GO:1904892	0.0137	0.999	2	40	regulation of STAT cascade	BP	0.835	LYN,ASPN	down_ACOR_EC
GO:1903530	0.0137	0.999	2	165	regulation of secretion by cell	BP	0.977	WNT7A,ARG2	up_AORT_VSMC
GO:0061138	0.0137	0.999	2	73	morphogenesis of a branching epithelium	BP	1	EYA1,HOXA11	up_VFEM_VSMC
GO:2000027	0.0137	0.999	2	72	regulation of animal organ morphogenesis	BP	1	EYA1,HOXA11	up_VFEM_VSMC
GO:0071229	0.0137	0.999	2	51	cellular response to acid chemical	BP	1	NR1H4,PTGER2	up_VPUL_EC
GO:0021649	0.0137	1	1	2	vestibulocochlear nerve structural organization	BP	0.835	NRP2	down_ACOR_EC
GO:0061552	0.0137	1	1	2	ganglion morphogenesis	BP	0.835	NRP2	down_ACOR_EC
GO:0097374	0.0137	1	1	2	sensory neuron axon guidance	BP	0.835	NRP2	down_ACOR_EC
GO:1904835	0.0137	1	1	2	dorsal root ganglion morphogenesis	BP	0.835	NRP2	down_ACOR_EC
GO:0003007	0.0137	1	1	94	heart morphogenesis	BP	1	GATA5	down_AFEM_VSMC
GO:0008016	0.0137	1	1	48	regulation of heart contraction	BP	1	JUP	down_VFEM_VSMC
GO:0048103	0.0137	1	1	10	somatic stem cell division	BP	0.977	WNT7A	up_AORT_VSMC
GO:0003207	0.0137	1	1	5	cardiac chamber formation	BP	1	NKX2-5	up_VPOR_VSMC
GO:2000052	0.0137	1	1	5	positive regulation of non-canonical Wnt signaling	BP	1	ANKRD6	up_VPOR_VSMC
GO:0045656	0.0137	1	1	3	negative regulation of monocyte differentiation	BP	1	HOXA7	up_VFEM_EC
GO:0034383	0.0137	1	1	4	low-density lipoprotein particle clearance	BP	1	NR1H4	up_VPUL_EC
GO:0051890	0.0137	1	1	4	regulation of cardioblast differentiation	BP	1	TBX5	up_VPUL_EC
GO:1903969	0.0137	1	1	3	regulation of response to macrophage colony-stimulating factor	BP	0.993	TLR2	up_VSAP_VSMC
GO:1903972	0.0137	1	1	3	regulation of cellular response to macrophage colony-stimulating factor	BP	0.993	TLR2	up_VSAP_VSMC
GO:0002062	0.0138	0.995	10	48	chondrocyte differentiation	BP	1	HOXA11,AXIN2,OSR2,OSR1,ZBTB16,RARG,SULF2,SHOX2,NFIB,TGFBR2	up_VSAP_EC
GO:0030111	0.0138	0.996	7	108	regulation of Wnt signaling pathway	BP	1	GREM1,FAM53B,CYLD,RECK,LGR6,AXIN2,KANK1	up_AFEM_EC
GO:0032502	0.0138	0.998	6	1661	developmental process	BP	1	HOXC10,TBX15,EYA1,HOXA9,BEND2,IRX5	up_AFEM_VSMC
GO:0006970	0.0138	0.997	5	23	response to osmotic stress	BP	0.567	ARHGEF2,PLK3,CASP3,LRRC8A,PTGS2	down_VSAP_EC
GO:0043502	0.0138	1	1	16	regulation of muscle adaptation	BP	1	GATA5	down_VSAP_VSMC
GO:2000514	0.0138	1	1	13	regulation of CD4-positive, alpha-beta T cell	BP	0.977	ARG2	up_AORT_VSMC
GO:0090191	0.0138	1	1	3	negative regulation of branching involved in ureteric bud	BP	1	SIX1	up_VFEM_EC
GO:0071073	0.0138	1	1	4	positive regulation of phospholipid biosynthetic	BP	1	NR1H4	up_VPUL_EC
GO:0003180	0.0138	1	1	7	aortic valve morphogenesis	BP	1	GATA5	up_VPUL_VSMC
GO:1900120	0.0138	1	1	7	regulation of receptor binding	BP	1	HOXA3	up_VPUL_VSMC
GO:0048169	0.0138	1	1	3	regulation of long-term neuronal synaptic	BP	0.993	NETO1	up_VSAP_VSMC
GO:0048523	0.0139	0.995	13	1399	negative regulation of cellular process	BP	0.835	ACVRL1,LYN,IGF1R,PDPN,HOXA7,PRKAR2A,USP28,ATRIP,SULF1,ELK3,MYO1D,ASPN,NEDD4	down_ACOR_EC
GO:0060442	0.0139	0.999	3	6	branching involved in prostate gland	BP	1	BMP4,FGFR2,FRS2	up_ACOR_VSMC

GO:0048598	0.0139	0.999	3	207	embryonic morphogenesis	BP	1	HOXA3,NKX2-5,CYP26B1	up_VPOR_VSMC
GO:0050678	0.0139	0.998	3	117	regulation of epithelial cell proliferation	BP	1	EYA1,SIX1,MTSS1	up_VFEM_EC
GO:0048871	0.0139	0.998	3	120	multicellular organismal homeostasis	BP	0.993	GATM,HOXC10,CAV1	up_VSAP_VSMC
GO:0060537	0.0139	0.998	3	117	muscle tissue development	BP	0.993	PITX1,CAV1,SHOX2	up_VSAP_VSMC
GO:0019896	0.0139	1	2	3	axonal transport of mitochondrion	BP	0.569	HIF1A,TRAK1	down_VSAP_EC
GO:1902306	0.0139	1	1	3	negative regulation of sodium ion	BP	0.835	NEDD4	down_ACOR_EC
GO:0060216	0.0139	1	1	3	definitive hemopoiesis	BP	1	HOXA9	up_VFEM_EC
GO:0003211	0.0139	1	1	4	cardiac ventricle formation	BP	1	TBX5	up_VPUL_EC
GO:0003263	0.0139	1	1	4	cardioblast proliferation	BP	1	TBX5	up_VPUL_EC
GO:0070857	0.0139	1	1	4	regulation of bile acid biosynthetic process	BP	1	NR1H4	up_VPUL_EC
GO:1904251	0.0139	1	1	4	regulation of bile acid metabolic process	BP	1	NR1H4	up_VPUL_EC
GO:0003181	0.0139	1	1	7	atrioventricular valve morphogenesis	BP	1	TBX5	up_VPUL_VSMC
GO:0072170	0.013971849	0.99937	2	7	metanephric tubule development	BP	0.96436359	SOX9,AQP1	up_ACOR_EC
GO:0072207	0.013971849	0.99937	2	7	metanephric epithelium development	BP	0.96436359	SOX9,AQP1	up_ACOR_EC
GO:0072234	0.013971849	0.99937	2	7	metanephric nephron tubule development	BP	0.96436359	SOX9,AQP1	up_ACOR_EC
GO:0072243	0.013971849	0.99937	2	7	metanephric nephron epithelium development	BP	0.96436359	SOX9,AQP1	up_ACOR_EC
GO:0035556	0.01398193	0.99187	33	845	intracellular signal transduction	BP	0.96436359	NR1H4,BRSK1,PLCL1,SOX9,RHOU,PTH1R,NDRG2,AQP1,ENSCAFG00000004331,FZD5,DUSP26,PTGIR,NOD2,PDE4A,PLCE1,PAK3,GUCY1B1,TNFRSF19,ARHGDIB,DCLK2,MAPK8IP2,RAPGEF4,FIGNL1,LRRC15,SH3RF3,LRRC4,CLU,C23H3orf58,PRKACA,STK32C,NRP1,ARHGEF26,PRKCD	up_ACOR_EC
GO:0050790	0.014	0.991	58	532	regulation of catalytic activity	BP	0.509	TEK,KIT,SELE,PPP2R5A,ARHGEF15,TNFAIP3,MAP3K11,AMOT,PDGFB,STK10,RELN,FGD6,MEF2C,RAPGEF1,TLR4,NLRC5,EDN1,RALB,NEDD9,CASP8,MTOR,MAP3K3,BMP2,HHEX,CHN2,PREX2,SLK,SH3BP5,PPP1R2,PROX1,IQGAP2,MADD,HMBOX1,PAK2,PRKD2,SH3GLB1,LDB2,PTGS2,MMD,RAPGEF2,BCL10,GCH1,HMGB1,TBC1D1,NOS2,PMAIP1,BLM,RIPK1,ITGA6,PDGFD,KDM5A,HIF1A,ALS2,ENSCAFG00000002015,FKBP15,PSMB9,RABGAP1,USP6NL	up_VCAV_EC
GO:0043603	0.014	0.992	29	372	cellular amide metabolic process	BP	1	GPAM,PRNP,UBA52,LARP6,COA1,SAMD4B,RPL23,RPL13,EIF3K,MRPL37,PARK7,RPL27A,EEF2,ZFP36L1,SEC11C,RNPEP,RPL27,MRPS11,ORMDL3,RPS27,ENSCAFG00000017708,ACSS2,PCSK6,ENSCAFG00000015122,PYM1,NAPEPLD,FOXO3,EIF4EBP1,ACAT1	down_VCAV_EC
GO:0006816	0.014	0.994	15	84	calcium ion transport	BP	1	PLCG2,HTR2B,TPC3,SARAF,MICU2,ITPR1,CACNB3,PRKCE,PSEN1,CHD7,INPP5K,WFS1,ATP2C1,SLC8A1,STC2	up_ACOR_VSMC
GO:0022898	0.014	0.995	10	48	regulation of transmembrane	BP	1	ACTN2,PLCG2,KCNE3,TCAF1,PTPN3,DAPK1,CACNB3,AKAP6,FXYD1,PARK7	up_ACOR_VSMC
GO:0055001	0.014	0.995	9	47	muscle cell development	BP	0.509	MEF2C,EDN1,TMOD1,PPP3CA,MTOR,CAV2,PROX1,P2RX2,TMOD3	up_VCAV_EC
GO:0034405	0.014	0.998	4	11	response to fluid shear stress	BP	0.509	MEF2C,MTSS1,ABCA1,PTGS2	up_VCAV_EC
GO:0031639	0.014	0.999	3	9	plasminogen activation	BP	0.569	SERPINE1,ENSCAFG00000005575,PGK1	down_VSAP_EC
GO:0051704	0.014	0.999	3	476	multi-organism process	BP	0.977	HOXA9,ARG2,ESR1	up_AORT_VSMC
GO:0061458	0.014	0.999	2	131	reproductive system development	BP	1	HOXA9,IRX5	up_AFEM_VSMC
GO:0007268	0.014	0.999	2	130	chemical synaptic transmission	BP	1	SLITRK5,CA2	up_VCAV_VSMC
GO:0098916	0.014	0.999	2	130	anterograde trans-synaptic signaling	BP	1	SLITRK5,CA2	up_VCAV_VSMC
GO:0060993	0.014	0.999	2	42	kidney morphogenesis	BP	0.993	HOXA11,SALL1	up_VSAP_VSMC
GO:0010592	0.014	1	1	7	positive regulation of lamellipodium assembly	BP	1	FSCN1	down_AFEM_EC

GO:0001829	0.014	1	1	7	trophectodermal cell differentiation	BP	1	CITED2	down_VPOR_EC
GO:0048665	0.014	1	1	6	neuron fate specification	BP	1	HOXC10	up_AORT_EC
GO:0032651	0.014	1	1	13	regulation of interleukin-1 beta production	BP	0.977	ARG2	up_AORT_VSMC
GO:0048663	0.014	1	1	13	neuron fate commitment	BP	0.977	HOXC10	up_AORT_VSMC
GO:0010667	0.014	1	1	5	negative regulation of cardiac muscle cell	BP	1	NKX2-5	up_VPOR_VSMC
GO:0043114	0.014	1	1	7	regulation of vascular permeability	BP	1	SLIT2	up_VPUL_VSMC
GO:1901213	0.014	1	1	7	regulation of transcription from RNA polymerase II promoter involved in	BP	1	GATA5	up_VPUL_VSMC
GO:0035635	0.014	1	1	3	entry of bacterium into host cell	BP	0.993	CAV1	up_VSAP_VSMC
GO:2000535	0.014	1	1	3	regulation of entry of bacterium into host cell	BP	0.993	CAV1	up_VSAP_VSMC
GO:0007167	0.014068952	0.99391	14	274	enzyme linked receptor protein signaling pathway	BP	0.96436359	SH3TC2,CDH3,NR1H4,SOX9,ENSCAFG00000004331,PTGIR,RGMA,SMAD6,ESM1,ADAMTSL2,FOXC2,LRP4,NRP1,PRKCD	up_ACOR_EC
GO:0051172	0.0141	0.996	8	663	negative regulation of nitrogen compound	BP	0.835	ACVRL1,LYN,IGF1R,HOXA7,PRKAR2A,ELK3,ASPN,NEDD4	down_ACOR_EC
GO:0009067	0.0141	0.999	3	9	aspartate family amino acid biosynthetic process	BP	0.569	ASNS,PLOD2,PLOD3	down_VSAP_EC
GO:0048489	0.0141	0.999	2	38	synaptic vesicle transport	BP	0.835	SPG11,STX4	down_ACOR_EC
GO:0097480	0.0141	0.999	2	38	establishment of synaptic vesicle localization	BP	0.835	SPG11,STX4	down_ACOR_EC
GO:1904375	0.0141	0.999	2	35	regulation of protein localization to cell	BP	0.835	ACSL3,STX4	down_ACOR_EC
GO:0042659	0.0141	1	2	4	regulation of cell fate specification	BP	1	BMPR1A,LMO4	down_VCAV_EC
GO:0048263	0.0141	1	2	4	determination of dorsal identity	BP	0.569	CTNNB1,DDIT3	down_VSAP_EC
GO:0034123	0.0141	1	1	10	positive regulation of toll-like receptor signaling	BP	1	TLR2	up_AFEM_VSMC
GO:1901099	0.0141	1	1	10	negative regulation of signal transduction in	BP	1	EYA1	up_AFEM_VSMC
GO:2001240	0.0141	1	1	10	negative regulation of extrinsic apoptotic signaling pathway in	BP	1	EYA1	up_AFEM_VSMC
GO:0048806	0.0141	1	1	10	genitalia development	BP	0.977	ESR1	up_AORT_VSMC
GO:0099172	0.0141	1	1	11	presynapse organization	BP	0.977	WNT7A	up_AORT_VSMC
GO:0061323	0.0141	1	1	7	cell proliferation involved in heart morphogenesis	BP	1	TBX5	up_VPUL_VSMC
GO:0043086	0.014110048	0.99488	10	170	negative regulation of catalytic activity	BP	0.96436359	PKIB,PCSK1N,IFI6,DUSP26,CST3,LRRC15,LRRC4,PRKCD,PPP1R26,DBNDD2	up_ACOR_EC
GO:0002793	0.014112616	0.99703	5	54	positive regulation of peptide secretion	BP	0.96436359	NR1H4,FZD5,NOD2,PTGER4,MAVS	up_ACOR_EC
GO:0050714	0.014112616	0.99703	5	54	positive regulation of protein secretion	BP	0.96436359	NR1H4,FZD5,NOD2,PTGER4,MAVS	up_ACOR_EC
GO:1901701	0.014151493	0.99387	14	271	cellular response to oxygen-containing compound	BP	0.96436359	NR1H4,SOX9,AQP1,ENSCAFG00000004331,KCNB1,AGRN,NOD2,ACTN2,GUCY1B1,PTGER4,FOXC2,RARRES2,PRKACA,PRKCD	up_ACOR_EC
GO:0045927	0.0142	0.997	5	61	positive regulation of growth	BP	1	LEP,DLL1,GPAM,ACACB,ADAM17	up_AFEM_EC
GO:0097028	0.0142	0.998	4	11	dendritic cell differentiation	BP	1	TMEM176B,TMEM176A,F2RL1,PSEN1	up_ACOR_VSMC
GO:0044060	0.0142	0.999	3	9	regulation of endocrine process	BP	0.573	PTPN11,RAB11FIP1,INHBA	down_VSAP_EC
GO:0016486	0.0142	0.999	3	6	peptide hormone processing	BP	1	ERO1B,SCG5,PCSK6	up_ACOR_VSMC
GO:0000423	0.0142	0.999	3	6	mitophagy	BP	1	AMBRA1,MFN2,HTRA2	up_VSAP_EC
GO:0099537	0.0142	0.999	2	131	trans-synaptic signaling	BP	1	SLITRK5,CA2	up_VCAV_VSMC
GO:0014846	0.0142	1	1	2	esophagus smooth muscle contraction	BP	0.835	SULF1	down_ACOR_EC
GO:0090316	0.0142	1	1	48	positive regulation of intracellular protein	BP	1	JUP	down_VFEM_VSMC
GO:2000136	0.0142	1	1	7	regulation of cell proliferation involved in	BP	1	TBX5	up_VPUL_VSMC
GO:0055017	0.014224288	0.99846	3	19	cardiac muscle tissue growth	BP	0.96436359	FOXC2,TBX5,C23H3orf58	up_ACOR_EC
GO:0019220	0.0143	0.996	7	525	regulation of phosphate metabolic process	BP	0.835	ACVRL1,LYN,IGF1R,PRKAR2A,ACSL3,MYO1D,ASPN	down_ACOR_EC

GO:0051174	0.0143	0.996	7	525	regulation of phosphorus metabolic process	BP	0.835	ACVRL1,LYN,IGF1R,PRKAR2A,ACSL3,MYO1D,ASPN	down_ACOR_EC
GO:1901343	0.0143	0.996	7	42	negative regulation of vasculature development	BP	0.576	SULF1,SERPINE1,SPARC,PGK1,THBS2,CTNNB1,STARD13	down_VSAP_EC
GO:0048534	0.0143	0.999	3	262	hematopoietic or lymphoid organ	BP	1	HOXA3,TMEM176A,TMEM176B	up_VPUL_VSMC
GO:0006399	0.0143	0.999	2	87	tRNA metabolic process	BP	1	DARS,TSEN15	down_AFEM_EC
GO:2000146	0.0143	0.999	2	92	negative regulation of cell motility	BP	1	TBX5,SLIT2	up_VPUL_VSMC
GO:0061307	0.0143	1	1	7	cardiac neural crest cell differentiation involved in heart development	BP	1	CITED2	down_VPOR_EC
GO:0061308	0.0143	1	1	7	cardiac neural crest cell development involved in heart development	BP	1	CITED2	down_VPOR_EC
GO:0071772	0.0143	1	1	50	response to BMP	BP	1	GATA5	down_VFEM_VSMC
GO:0071773	0.0143	1	1	50	cellular response to BMP stimulus	BP	1	GATA5	down_VFEM_VSMC
GO:0042551	0.0143	1	1	10	neuron maturation	BP	1	IRX5	up_AFEM_VSMC
GO:0090102	0.0143	1	1	10	cochlea development	BP	1	EYA1	up_AFEM_VSMC
GO:0032720	0.0143	1	1	13	negative regulation of tumor necrosis factor	BP	0.979	ARG2	up_AORT_VSMC
GO:0009605	0.0144	0.99	67	538	response to external stimulus	BP	1	VEGFD,SEMA5A,TLR1,PLCG2,LEP,TMEM255A,FEZ1,COCH,PRKAA2,MX1,ABCA1,GJA1,HRH1,CASTOR1,TLR3,PPARGC1A,GLRB,ISG15,PTGIR,SASH1,FGF18,CDKN2B,USP53,PLAC8B,ANKRD1,F2RL1,ENPP4,GABARAPL1,RBM4B,BNIP3,SLC17A5,CDKN1A,P2RX7,NRP1,PTPN22,GPM6A,ULK2,NPNT,UNC5B,SESN3,PRKCE,FZD3,DAG1,PARP9,APP,CHD7,PIK3C3,LAMP2,JUP,CCL5,SESN1,NOD2,FAM107A,DDIT4,JAM3,NAALADL2,B2M,SERINC3,TNFRSF11B,FEZ2,GNAQ,BMT2,WASL,ENSCAFG00000029553,PARK7,SLC8A1,ZFYVE1	up_ACOR_VSMC
GO:0009887	0.0144	0.991	42	314	animal organ morphogenesis	BP	1	TBX15,HOXA11,FZD1,THRB,HOXA7,PEX7,CDON,WNT11,AXIN2,OSR2,INSIG2,PPARGC1B,LIMS2,LRIG3,IRX5,HOXD3,SLC6A4,FGF7,ABL1,DLL1,HOXA6,OSR1,SAV1,RARG,STAT6,HOXC4,DLX3,MFN2,IRX3,ZEB1,SULF2,SHOX2,LRP4,E2F4,NTN4,KDM2B,NFIB,ZFAND5,ACVRL1,MAP2K2,TGFBR2,CITED2	up_VSAP_EC
GO:0048646	0.0144	0.992	32	349	anatomical structure formation involved in morphogenesis	BP	0.577	PRICKLE1,VEGFA,SULF1,MYH9,SERPINE1,ACTN1,SPARC,PRKACB,PTPN14,ITGA2,JCAD,PGK1,PTPN11,ENSCAFG00000032470,TMED2,FHL2,PRCP,WDR1,PIK3C2A,HIF1A,THBS2,MTDH,ESM1,CTNNB1,STARD13,KLF5,SDC4,INHBA,SIX4,NRP1,COL12A1,MYBPC2	down_VSAP_EC
GO:1903532	0.0144	0.994	14	78	positive regulation of secretion by cell	BP	1	EXPH5,TLR1,HTR2B,MBP,FERMT1,TM7SF3,CD274,ANKRD1,F2RL1,PTPN22,PRKCE,EDN1,NOD2,SCAMP5	up_ACOR_VSMC
GO:0006418	0.0144	0.997	6	31	tRNA aminoacylation for protein translation	BP	0.577	CARS,SARS,AARS,YARS,TARS,NARS	down_VSAP_EC
GO:0070167	0.0144	0.997	5	29	regulation of biomineral tissue development	BP	1	BMPR1A,ASPN,DDR2,OMD,GREM1	down_VCAV_EC
GO:0007178	0.0144	0.998	3	107	transmembrane receptor protein serine/threonine kinase signaling pathway	BP	0.841	ACVRL1,SULF1,ASPN	down_ACOR_EC
GO:0043144	0.0144	0.999	3	7	snoRNA processing	BP	0.582	FBL,ENSCAFG00000019620,EXOSC5	down_ACOR_VSMC
GO:0016074	0.0144	0.999	3	7	snoRNA metabolic process	BP	0.582	FBL,ENSCAFG00000019620,EXOSC5	down_ACOR_VSMC
GO:0016482	0.0144	0.998	3	63	cytosolic transport	BP	1	HOOK3,GCC2,TMEM87B	up_VPOR_EC
GO:0090280	0.0144	1	2	3	positive regulation of calcium ion import	BP	0.52	PDGFB,STC1	up_VCAV_EC
GO:0048640	0.0144	1	1	21	negative regulation of developmental growth	BP	1	TBX5	up_APUL_VSMC
GO:1903361	0.0144	1	1	3	protein localization to basolateral plasma	BP	1	CAV1	up_VSAP_VSMC

GO:0048519	0.0145	0.989	150	1554	negative regulation of biological process	BP	0.521	EPHB1,PLK2,EDNRB,TEK,HCLS1,IL7R,ADGRF5,KIT,PPP2R5A,CXCL8,PRKCH,ARHGEF15,IFRD1,DOCK4,TIE1,CDA,TNFAIP3,ADAMTS9,TNIP1,AMOT,PDGFB,PODXL,CD34,PDE4D,MOV10,MEF2C,FERMT1,ATF3,CHD7,KLKB1,TIPARP,DLL4,RAPGEF1,WWP1,TLR4,NLRC5,GPR4,STC1,GATA3,MMP28,EDN1,HOXB8,APLNR,ARAP3,CTTNBP2NL,HOMER3,TFPI,IRF8,ARHGAP28,SOX18,TMOD1,EGFL7,FIGNL1,PELI1,PPP3CA,FAM3C,MTSS1,GUCY1A1,ARG2,CASP8,MTOR,TENT2,HOOK3,MAP3K3,BMP2,CDYL,HMG20A,HHEX,CREM,CAV2,PREX2,TUT7,SH3BP5,CNOT6,RSC1A1,ENC1,ITPKB,GDPD5,RIOK3,ANGPT2,PROX1,UHRF2,ENSCAFG00000010224,MDFI,ENSCAFG00000010596,STMN1,HMBOX1,PAK2,MCC,THOC1,SWAP70,SMARCA4,APOD,PROCR,NCK1,TIMELESS,OTUD4,CD200,JAG1,PTGS2,ETAA1,RAPGEF2,TCERG1,PLCG2,CTDSPL2,CREB1,BCL10,BASP1,PAN3,USP28,TAOK1,CTNNA1,CNOT7,SMG7,ENSCAFG00000002897,HMGB1,BLM,SPTBN4,RIPK1,ITGA6,RAD21,RAD17,RGS2,SKIL,MTA2,ARHGDIB,CUL4A,ZW10,KDM5A,HIF1A,KDM3A,ATRX,NR4A1,HIST2H2AC,ARID5A,TOPBP1,ENSCAFG00000002015,VASH1,DCP2,TNFAIP1,IVNS1ABP,FKBP15,RGL2,TMOD3,ZEB1,ARHGEF2,PEAR1,FBXO5,	up_VCAV_EC
GO:0051091	0.0145	0.994	14	87	positive regulation of DNA-binding transcription factor activity	BP	0.52	HCLS1,KIT,PRKCH,TMEM173,RELN,EDN1,PPP3CA,PRKD2,SMARCA4,TLR3,CD200,BCL10,RIPK1,ARHGEF2	up_VCAV_EC
GO:0034333	0.0145	0.997	5	25	adherens junction assembly	BP	0.581	VEGFA,ACTN1,CTNNB1,ENSCAFG00000002015,NRP1	down_VSAP_EC
GO:0009653	0.0145	0.997	5	773	anatomical structure morphogenesis	BP	1	HOXA3,TBX5,GATA5,HOXB5,SLIT2	up_VPUL_VSMC
GO:0090382	0.0145	0.999	2	8	phagosome maturation	BP	1	RAB20,RAB7A	up_AFEM_EC
GO:0043393	0.0145	0.999	2	77	regulation of protein binding	BP	1	HOXA3,BAMBI	up_VPOR_VSMC
GO:0045619	0.0145	0.999	2	44	regulation of lymphocyte differentiation	BP	1	TOX,CYP26B1	up_VFEM_EC
GO:0060485	0.0145	0.999	2	93	mesenchyme development	BP	1	TBX5,GATA5	up_VPUL_VSMC
GO:2000736	0.0145	1	1	21	regulation of stem cell differentiation	BP	1	TBX5	up_APUL_VSMC
GO:0021563	0.0145	1	1	4	glossopharyngeal nerve development	BP	1	HOXA3	up_VPUL_EC
GO:0018108	0.0146	0.994	13	79	peptidyl-tyrosine phosphorylation	BP	0.522	HCLS1,KIT,PDGFB,RELN,NEDD9,YES1,MTOR,SH3BP5,FES,CNOT7,PDGFD,BMX,ARHGEF2	up_VCAV_EC
GO:1903829	0.0146	0.995	10	91	positive regulation of cellular protein	BP	1	LRP1,PRNP,CLIP3,ENSCAFG00000014978,AKT2,TRIM8,GLI3,PARK7,MAVS,GLIS2	down_VCAV_EC
GO:0022008	0.0146	0.998	4	406	neurogenesis	BP	1	HOXC10,EYA1,EMX2,SHOX2	up_VFEM_VSMC
GO:0099536	0.0146	0.999	2	133	synaptic signaling	BP	1	SLITRK5,CA2	up_VCAV_VSMC
GO:0045652	0.0146	1	1	7	regulation of megakaryocyte	BP	1	GABPA	down_AFEM_EC
GO:1900180	0.0146	1	1	50	regulation of protein localization to nucleus	BP	1	JUP	down_VFEM_VSMC
GO:0021978	0.0146	1	1	3	telencephalon regionalization	BP	1	EMX2	up_VSAP_VSMC
GO:0055093	0.0146	1	1	3	response to hyperoxia	BP	1	CAV1	up_VSAP_VSMC
GO:0071455	0.0146	1	1	3	cellular response to hyperoxia	BP	1	CAV1	up_VSAP_VSMC
GO:0007350	0.014631301	0.99933	2	7	blastoderm segmentation	BP	0.96436359	FZD5,NRP1	up_ACOR_EC
GO:0051271	0.014669661	0.99579	7	98	negative regulation of cellular component	BP	0.96436359	SEMA6D,ZMYND8,MMP28,PTGER4,ARHGDIB,TBX5,NRP1	up_ACOR_EC
GO:0003170	0.0147	0.997	6	25	heart valve development	BP	0.522	ADAMTS9,MEF2C,GATA3,MTOR,BMP2,JAG1	up_VCAV_EC
GO:0098801	0.0147	0.999	3	8	regulation of renal system process	BP	0.522	PDGFB,STC1,EDN1	up_VCAV_EC
GO:1901071	0.0147	0.999	3	6	glucosamine-containing compound metabolic	BP	1	RENBP,CHST7,GNPDA1	up_VSAP_EC
GO:0048305	0.0147	1	1	4	immunoglobulin secretion	BP	0.842	STX4	down_ACOR_EC
GO:0051023	0.0147	1	1	4	regulation of immunoglobulin secretion	BP	0.842	STX4	down_ACOR_EC
GO:0090386	0.0147	1	1	1	phagosome maturation involved in apoptotic cell	BP	0.842	SPG11	down_ACOR_EC

GO:0090387	0.0147	1	1	1	phagolysosome assembly involved in apoptotic cell clearance	BP	0.842	SPG11	down_ACOR_EC
GO:0090389	0.0147	1	1	1	phagosome-lysosome fusion involved in apoptotic cell clearance	BP	0.842	SPG11	down_ACOR_EC
GO:0003156	0.0147	1	1	20	regulation of animal organ formation	BP	1	TBX5	up_APUL_VSMC
GO:0048538	0.0147	1	1	20	thymus development	BP	1	HOXA3	up_APUL_VSMC
GO:0021871	0.0147	1	1	6	forebrain regionalization	BP	1	EMX2	up_VFEM_VSMC
GO:0071417	0.014754978	0.99494	9	142	cellular response to organonitrogen	BP	0.96436359	NR1H4,SOX9,AQP1,ENSCAFG00000004331,NOD2,ACTN2,FOXC2,RARRES2,PRKCD	up_ACOR_EC
GO:0048583	0.0148	0.989	83	1095	regulation of response to stimulus	BP	0.588	ARHGEF2,PRICKLE1,VEGFA,P4HB,SIPA1L1,SULF1,NR1H4,HSPA5,SERPINE1,SOCS5,PRKACB,ENPP1,ERRFI1,JCAD,SNX25,FAM49B,HYOU1,NUAK1,PTPN11,TRIB3,FARP1,ILK,FHL2,PRCP,SMARCA4,SESN2,PTGS2,MYO1C,CHSY1,UACA,HIF1A,ENSCAFG00000000562,TNIK,NXN,RGS2,PTPN2,EIF2B1,PVR,NEO1,MTDH,ESM1,DDX39B,ADAM17,SKAP2,CNOT7,MAGED1,CTNNB1,ENSCAFG00000002015,TFRC,APOE,WWC3,UBA5,MMP28,LYN,BNIP3,STRAP,DDIT3,FAM162A,PLEKHG2,CBLC,ATF3,GSK3B,CDC37,GBP5,SH3GLB1,TAX1BP3,HTT,RDH11,ARFGEF3,CALM1,DISC1,PAK2,A2M,INHBA,HIF1AN,NRP1,SH3RF3,RHBDF2,USP14,TNFAIP1,MADD,SEC13,MID1	down_VSAP_EC
GO:0031589	0.0148	0.998	3	93	cell-substrate adhesion	BP	0.842	ACVRL1,PDPN,HOXA7	down_ACOR_EC
GO:0046607	0.0148	0.999	3	7	positive regulation of centrosome cycle	BP	0.594	POC1A,NUP62,PLK4	down_ACOR_VSMC
GO:1900122	0.0148	1	1	3	positive regulation of receptor binding	BP	0.842	HOXA3	down_ACOR_EC
GO:0050710	0.0148	1	1	13	negative regulation of cytokine secretion	BP	0.987	ARG2	up_AORT_VSMC
GO:0070232	0.0148	1	1	13	regulation of T cell apoptotic process	BP	0.987	ARG2	up_AORT_VSMC
GO:0033561	0.0148	1	1	6	regulation of water loss via skin	BP	1	CYP26B1	up_VPOR_VSMC
GO:0060347	0.0148	1	1	6	heart trabecula formation	BP	1	NKX2-5	up_VPOR_VSMC
GO:0006000	0.0148	1	1	5	fructose metabolic process	BP	1	FBP1	up_VFEM_VSMC
GO:0032490	0.0148	1	1	4	detection of molecule of bacterial origin	BP	1	TLR2	up_VSAP_VSMC
GO:1901339	0.0148	1	1	4	regulation of store-operated calcium channel	BP	1	STIM1	up_VSAP_VSMC
GO:0043085	0.0149	0.991	38	323	positive regulation of catalytic activity	BP	0.527	TEK,KIT,SELE,MAP3K11,PDGFB,STK10,RELN,MEF2C,RAPGEF1,TLR4,EDN1,RALB,NEDD9,CASP8,MAP3K3,BMP2,SLK,PROX1,MADD,HMBOX1,PAK2,PRKD2,SH3GLB1,MMD,RAPGEF2,BCL10,GCH1,HMGB1,TBC1D1,NOS2,PMAIP1,RIPK1,ITGA6,PDGFD,ALS2,ENSCAFG00000002015,RABGAP1,USP6NL	up_VCAV_EC
GO:0030855	0.0149	0.992	27	183	epithelial cell differentiation	BP	1	FZD1,THRB,HOXA7,WNT11,HNF4A,ABL1,BTG1,DLL1,OSR1,ADAMTSL4,SAV1,KLF15,ANXA4,RILPL2,RARG,F2RL1,DLX3,ZFP36L1,ZEB1,MYD88,FRMD6,E2F4,NFIB,ACVRL1,LBH,KEAP1,LATS2	up_VSAP_EC
GO:0034766	0.0149	0.997	5	19	negative regulation of ion transmembrane transport	BP	0.599	NEDD4,CAV1,SLC30A1,GSTO1,REM1	down_ACOR_VSMC
GO:0033209	0.0149	0.997	5	24	tumor necrosis factor-mediated signaling	BP	0.59	NR1H4,ILK,PTPN2,ADAM17,ENSCAFG00000002015	down_VSAP_EC
GO:0006690	0.0149	0.997	5	16	icosanoid metabolic process	BP	1	PTGES,DAGLB,ABHD16A,HPGD,ENSCAFG00000023820	up_VSAP_EC
GO:0050768	0.0149	0.998	3	66	negative regulation of neurogenesis	BP	1	HOOK3,SEMA3A,RNF6	up_VPOR_EC
GO:0007379	0.0149	0.999	3	6	segment specification	BP	1	DLL1,OSR1,IRX3	up_VSAP_EC
GO:0003413	0.0149	0.999	3	6	chondrocyte differentiation involved in endochondral bone	BP	1	AXIN2,RARG,TGFBR2	up_VSAP_EC
GO:0032101	0.0149	0.999	2	173	regulation of response to external stimulus	BP	0.987	ARG2,ESR1	up_AORT_VSMC
GO:0150018	0.0149	1	1	2	basal dendrite development	BP	1	SEMA3A	up_VPOR_EC
GO:0150019	0.0149	1	1	2	basal dendrite morphogenesis	BP	1	SEMA3A	up_VPOR_EC

GO:0150020	0.0149	1	1	2	basal dendrite arborization	BP	1	SEMA3A	up_VPOR_EC
GO:0051124	0.0149	1	1	4	synaptic growth at neuromuscular junction	BP	1	SIX1	up_VFEM_EC
GO:0060352	0.0149	1	1	3	cell adhesion molecule production	BP	1	CAV1	up_VSAP_VSMC
GO:0034762	0.014913371	0.99526	8	122	regulation of transmembrane transport	BP	0.96436359	NR1H4,ENSCAFG00000004331,KCNB1,ACTN2,MAPK8IP2,RARRES2,AZIN2,PM20D1	up_ACOR_EC
GO:0008347	0.015	0.998	3	11	glial cell migration	BP	1	NDN,CSPG4,GLI3	down_VCAV_EC
GO:0032689	0.015	0.999	3	8	negative regulation of interferon-gamma	BP	0.53	TLR4,GATA3,HMGB1	up_VCAV_EC
GO:0048514	0.015	0.998	3	214	blood vessel morphogenesis	BP	1	HOXA3,NKX2-5,C3AR1	up_VPOR_VSMC
GO:1905214	0.015	0.999	3	6	regulation of RNA binding	BP	1	LARP6,CDK9,EIF3D	up_VSAP_EC
GO:1905216	0.015	0.999	3	6	positive regulation of RNA binding	BP	1	LARP6,CDK9,EIF3D	up_VSAP_EC
GO:0018158	0.015	0.999	2	4	protein oxidation	BP	1	PARK7,LOXL3	down_VCAV_EC
GO:0050776	0.015	0.999	2	173	regulation of immune response	BP	0.987	ARG2,ESR1	up_AORT_VSMC
GO:0050877	0.015	0.999	2	164	nervous system process	BP	0.987	HOXC10,WNT7A	up_AORT_VSMC
GO:0051046	0.015	0.999	2	174	regulation of secretion	BP	0.987	WNT7A,ARG2	up_AORT_VSMC
GO:0060348	0.015	0.999	2	78	bone development	BP	1	HOXA11,SHOX2	up_VFEM_VSMC
GO:0003007	0.015	0.999	2	94	heart morphogenesis	BP	1	TBX5,GATA5	up_VPUL_VSMC
GO:0048705	0.015	0.999	2	95	skeletal system morphogenesis	BP	1	HOXA3,HOXB5	up_VPUL_VSMC
GO:0042130	0.015	1	1	13	negative regulation of T cell proliferation	BP	0.987	ARG2	up_AORT_VSMC
GO:0060419	0.015	1	1	22	heart growth	BP	1	TBX5	up_APUL_VSMC
GO:1900120	0.015	1	1	7	regulation of receptor binding	BP	1	HOXA3	up_VPOR_VSMC
GO:0006907	0.015	1	1	3	pinocytosis	BP	1	CAV1	up_VSAP_VSMC
GO:0035239	0.0151	0.991	28	299	tube morphogenesis	BP	0.592	PRICKLE1,VEGFA,SULF1,MYH9,SERPINE1,SPARC,PRKACB,JCAD,PGK1,ENSCAFG00000032470,TMED2,ILK,PRCP,PIK3C2A,HOXB7,HIF1A,THBS2,MTDH,ESM1,MAGED1,CTNNB1,STARD13,APOE,KLF5,MYO1E,SDC4,SIX4,NRP1	down_VSAP_EC
GO:0070887	0.0151	0.992	28	775	cellular response to chemical stimulus	BP	1	CMKLR1,MT2A,LEP,LBH,CRY2,ENSCAFG00000009113,RAB20,GAS1,ANGPT4,DLL1,CPNE7,PLXNA4,CHRM2,TMEM100,GREM1,NFE2L1,AVPR1A,CAMK2A,GREM2,CYLD,POSTN,LRRC15,NR3C1,PPARGC1A,CCL5,ADAM17,KANK1,SOD1	up_AFEM_EC
GO:0016052	0.0151	0.996	6	34	carbohydrate catabolic process	BP	0.592	PGK1,TPI1,PGAM1,HIF1A,ALDOC,GALE	down_VSAP_EC
GO:0031326	0.0151	0.998	5	1176	regulation of cellular biosynthetic process	BP	1	TLR2,EYA1,HOXA9,HOXA10,IRX5	up_AFEM_VSMC
GO:0031400	0.0151	0.997	4	205	negative regulation of protein modification	BP	0.847	LYN,IGF1R,PRKAR2A,ASPN	down_ACOR_EC
GO:0009081	0.0151	0.999	3	6	branched-chain amino acid metabolic process	BP	1	ALDH6A1,BCKDHB,ENSCAFG00000002977	up_VSAP_EC
GO:0006486	0.0151	0.999	2	91	protein glycosylation	BP	1	MGAT4B,GBGT1	down_VPOR_EC
GO:0043413	0.0151	0.999	2	91	macromolecule glycosylation	BP	1	MGAT4B,GBGT1	down_VPOR_EC
GO:0048705	0.0151	0.999	2	95	skeletal system morphogenesis	BP	1	CITED2,TBX15	down_VPOR_EC
GO:0071229	0.0151	0.999	2	51	cellular response to acid chemical	BP	1	NR1H4,NTRK2	up_APUL_EC
GO:0022602	0.0151	1	1	13	ovulation cycle process	BP	0.987	ESR1	up_AORT_VSMC
GO:1903556	0.0151	1	1	14	negative regulation of tumor necrosis factor superfamily cytokine	BP	0.987	ARG2	up_AORT_VSMC
GO:0033631	0.0151	1	1	4	cell-cell adhesion mediated by integrin	BP	1	NPNT	up_APUL_EC
GO:0043031	0.0151	1	1	4	negative regulation of macrophage activation	BP	1	BPI	up_APUL_EC
GO:0014855	0.0151	1	1	22	striated muscle cell proliferation	BP	1	TBX5	up_APUL_VSMC

GO:0032026	0.0151	1	1	2	response to magnesium ion	BP	1	FBP1	up_VPOR_EC
GO:0000188	0.0151	1	1	3	inactivation of MAPK activity	BP	1	CAV1	up_VSAP_VSMC
GO:0060216	0.0151	1	1	3	definitive hemopoiesis	BP	1	HOXA9	up_VSAP_VSMC
GO:0048598	0.0152	0.993	18	207	embryonic morphogenesis	BP	1	SDC4,FBN1,BMPR1A,LMO4,RECK,COL6A1,NAGLU,TBX15,HOXC4,LAMA3,TLX2,GLI3,ZFP36L1,IFT57,IRX5,RDH10,GREM1,ZBTB16	down_VCAV_EC
GO:0019362	0.0152	0.996	6	35	pyridine nucleotide metabolic process	BP	0.592	PGK1,TPI1,PGAM1,HIF1A,NMNAT2,ALDOC	down_VSAP_EC
GO:0046496	0.0152	0.996	6	35	nicotinamide nucleotide metabolic process	BP	0.592	PGK1,TPI1,PGAM1,HIF1A,NMNAT2,ALDOC	down_VSAP_EC
GO:0032677	0.0152	0.997	6	22	regulation of interleukin-8 production	BP	1	C5AR2,ANXA4,F2RL1,MAPKBP1,ZNF580,MAVS	up_VSAP_EC
GO:0048008	0.0152	0.997	5	24	platelet-derived growth factor receptor signaling	BP	0.592	VEGFA,PTPN11,PTPN2,MYO1E,NRP1	down_VSAP_EC
GO:0002183	0.0152	0.997	5	16	cytoplasmic translational initiation	BP	1	EIF3G,EIF3K,EIF3L,EIF3D,NCK1	up_VSAP_EC
GO:0001954	0.0152	0.998	4	17	positive regulation of cell-matrix adhesion	BP	0.592	VEGFA,ENSCAFG00000002015,GSK3B,NRP1	down_VSAP_EC
GO:0010592	0.0152	0.999	3	7	positive regulation of lamellipodium assembly	BP	0.533	MTOR,NCKAP1,FSCN1	up_VCAV_EC
GO:0060914	0.0152	1	1	11	heart formation	BP	1	EYA1	up_AFEM_VSMC
GO:0007214	0.0152	1	1	4	gamma-aminobutyric acid signaling pathway	BP	1	PLCL1	up_APUL_EC
GO:0071073	0.0152	1	1	4	positive regulation of phospholipid biosynthetic	BP	1	NR1H4	up_APUL_EC
GO:1904396	0.0152	1	1	4	regulation of neuromuscular junction	BP	1	SIX1	up_VFEM_EC
GO:0045665	0.015218598	0.99673	5	55	negative regulation of neuron differentiation	BP	0.96436359	SOX9,DIXDC1,RGMA,LRP4,NRP1	up_ACOR_EC
GO:0046324	0.015260998	0.9983	3	20	regulation of glucose import	BP	0.96436359	NR1H4,ENSCAFG00000004331,RARRES2	up_ACOR_EC
GO:0002790	0.0153	0.992	21	133	peptide secretion	BP	1	HNF4A,CD274,C5AR2,SLC8B1,ABL1,IL17RC,ANXA4,F2RL1,LMF1,RAB11B,PASK,STEAP3,KCNB1,RAF1,PNP,MAPKBP1,STX4,PPP3CB,MAVS,NAGPA,RAB11FIP2	up_VSAP_EC
GO:0007219	0.0153	0.995	10	59	Notch signaling pathway	BP	0.533	DLL4,EGFL7,TSPAN14,BMP2,HHEX,BMP2K,GDPD5,MFNG,JAG1,PEAR1	up_VCAV_EC
GO:0007229	0.0153	0.996	6	32	integrin-mediated signaling pathway	BP	0.592	ITGA2,PTPN11,ILK,TLN1,ITGB3,NRP1	down_VSAP_EC
GO:0072384	0.0153	0.997	5	24	organelle transport along microtubule	BP	0.592	KIF5B,HIF1A,TRAK1,HTT,AP3S1	down_VSAP_EC
GO:0002376	0.0153	0.997	5	622	immune system process	BP	1	HOXA3,BARX1,NKX2-5,CYP26B1,C3AR1	up_VPOR_VSMC
GO:2000249	0.0153	0.998	4	12	regulation of actin cytoskeleton	BP	0.533	TEK,BAIAP2L1,FES,ARHGDIB	up_VCAV_EC
GO:0003128	0.0153	0.999	3	6	heart field specification	BP	1	GATA5,BMP4,SMARCD3	up_ACOR_VSMC
GO:0034612	0.0153	0.998	3	63	response to tumor necrosis factor	BP	1	ARHGEF2,ENSCAFG00000002015,ENSCAFG00000032731	up_VPOR_EC
GO:0002274	0.0153	0.999	2	40	myeloid leukocyte activation	BP	0.848	LYN,STX4	down_ACOR_EC
GO:1990791	0.0153	1	2	3	dorsal root ganglion development	BP	0.592	CTNNB1,NRP1	down_VSAP_EC
GO:0010959	0.0153	0.999	2	79	regulation of metal ion transport	BP	1	HOXA3,NKX2-5	up_VPOR_VSMC
GO:0050855	0.0153	1	1	4	regulation of B cell receptor signaling	BP	0.848	LYN	down_ACOR_EC
GO:0050861	0.0153	1	1	4	positive regulation of B cell receptor signaling	BP	0.848	LYN	down_ACOR_EC
GO:0033539	0.0153	1	1	7	fatty acid beta-oxidation using acyl-CoA	BP	1	ETFA	down_AFEM_EC
GO:0070229	0.0153	1	1	13	negative regulation of lymphocyte apoptotic	BP	0.993	ARG2	up_AORT_VSMC
GO:0006521	0.0153	1	1	4	regulation of cellular amino acid metabolic	BP	1	NR1H4	up_APUL_EC
GO:0030809	0.0153	1	1	6	negative regulation of nucleotide biosynthetic	BP	1	FBP1	up_VFEM_VSMC
GO:1900372	0.0153	1	1	6	negative regulation of purine nucleotide	BP	1	FBP1	up_VFEM_VSMC
GO:2001170	0.0153	1	1	6	negative regulation of ATP biosynthetic process	BP	1	FBP1	up_VFEM_VSMC
GO:0001667	0.0154	0.993	16	146	ameboidal-type cell migration	BP	0.594	VEGFA,MYH9,SPARC,JCAD,PTPN11,ILK,PRCP,PIK3C2A,HIF1A,ADAM17,ANLN,STARD13,APOE,STRAP,SDC4,NRP1	down_VSAP_EC
GO:0009142	0.0154	0.996	7	46	nucleoside triphosphate biosynthetic process	BP	0.594	PGK1,TPI1,PGAM1,HIF1A,ALDOC,AK5,CMPK1	down_VSAP_EC

GO:0090304	0.0154	0.997	6	1694	nucleic acid metabolic process	BP	1	TLR2,TBX15,EYA1,HOXA9,HOXA10,IRX5	up_AFEM_VSMC
GO:0045597	0.0154	0.997	4	253	positive regulation of cell differentiation	BP	1	TBX5,WIF1,NPNT,NTRK2	up_APUL_EC
GO:0006812	0.0154	0.998	3	222	cation transport	BP	1	HOXA3,NKX2-5,TRPC4	up_VPOR_VSMC
GO:0008015	0.0154	0.998	3	120	blood circulation	BP	1	IRX5,CAV1,F5	up_VSAP_VSMC
GO:0003158	0.0154	0.999	2	38	endothelium development	BP	0.849	ACVRL1,PDPN	down_ACOR_EC
GO:0045747	0.0154	1	1	18	positive regulation of Notch signaling pathway	BP	1	GATA5	down_VSAP_VSMC
GO:0060443	0.0154	1	1	13	mammary gland morphogenesis	BP	0.997	ESR1	up_AORT_VSMC
GO:0055024	0.0154	1	1	22	regulation of cardiac muscle tissue	BP	1	TBX5	up_APUL_VSMC
GO:0072376	0.0154	1	1	11	protein activation cascade	BP	1	C6	up_VCAV_VSMC
GO:0051960	0.0155	0.992	22	216	regulation of nervous system development	BP	0.594	ARHGEF2,SIPA1L1,PRKACB,DPYSL3,HOOK3,IFRD1,HIF1A,SLC45A3,TNIK,ZPR1,UST,CTNNB1,APOE,LYN,MMD,VLDLR,REST,DISC1,LRRTM2,SIX4,NRP1,YWHAH	down_VSAP_EC
GO:0048638	0.0155	0.993	15	82	regulation of developmental growth	BP	1	LEP,BMP4,FGFR2,LATS2,ATRN,PLAC8B,NRP1,ULK2,TBX20,TBX5,APP,CHD7,WWC2,EDN1,STC2	up_ACOR_VSMC
GO:0009746	0.0155	0.995	8	35	response to hexose	BP	1	HNF4A,RAB11B,LPL,KCNB1,RAF1,STX4,PPP3CB,RAB11FIP2	up_VSAP_EC
GO:0034284	0.0155	0.995	8	35	response to monosaccharide	BP	1	HNF4A,RAB11B,LPL,KCNB1,RAF1,STX4,PPP3CB,RAB11FIP2	up_VSAP_EC
GO:0045893	0.0155	0.998	4	413	positive regulation of transcription, DNA-	BP	1	EYA1,HOXA9,HOXA11,SHOX2	up_VFEM_VSMC
GO:0022008	0.0155	0.998	3	406	neurogenesis	BP	1	HOXC10,EYA1,IRX5	up_AFEM_VSMC
GO:0007602	0.0155	0.999	2	4	phototransduction	BP	0.596	GNAQ,RDH11	down_VSAP_EC
GO:0048667	0.0155	0.999	2	140	cell morphogenesis involved in neuron	BP	1	SLITRK5,LIMK1	up_VCAV_VSMC
GO:0031646	0.0155	1	1	7	positive regulation of neurological system	BP	1	NETO1	up_VFEM_VSMC
GO:0008286	0.015565218	0.99738	4	36	insulin receptor signaling pathway	BP	0.96436359	NR1H4,ENSCAFG00000004331,FOXC2,PRKCD	up_ACOR_EC
GO:0048585	0.0156	0.99	53	475	negative regulation of response to stimulus	BP	0.541	PLK2,IL7R,CXCL8,TNFAIP3,TNIP1,PDGFB,CD34,PDE4D,FERMT1,ATF3,KLKB1,DLL4,RAPGEF1,TLR4,NLRC5,GATA3,MMP28,APLNR,ARAP3,HOMER3,TFPI,EGFL7,FIGNL1,ARG2,CASP8,MTOR,MAP3K3,BMP2,HHEX,PREX2,GDPD5,RIOK3,ANGPT2,STMN1,MCC,THOC1,SMARCA4,APOD,NCK1,OTUD4,PTGS2,CTDSPL2,CTNNA1,CNOT7,RIPK1,ITGA6,RGS2,SKIL,HIF1A,TNFAIP1,IVNS1ABP,ARHGEF2,PEAR1	up_VCAV_EC
GO:0046328	0.0156	0.994	12	61	regulation of JNK cascade	BP	1	RASSF2,TLR3,SH3RF2,SASH1,F2RL1,SH3RF1,APP,DUSP22,EDN1,NOD2,SH3RF3,MAP3K7	up_ACOR_VSMC
GO:0032502	0.0156	0.995	11	1661	developmental process	BP	1	TBX5,HOXA3,NR1H4,EVI2B,HOXA2,MYBPC2,WIF1,CCL24,FLRT3,ARHGEF2,NEO1	up_VPUL_EC
GO:0048593	0.0156	0.997	4	41	camera-type eye morphogenesis	BP	1	IRX5,COL8A2,DLL1,THRB	up_AFEM_EC
GO:0032501	0.0156	0.999	4	1782	multicellular organismal process	BP	1	HOXA3,TBX5,HOXA2,HOXB5	up_APUL_VSMC
GO:0060219	0.0156	0.999	2	4	camera-type eye photoreceptor cell	BP	1	THY1,THRB	down_VCAV_EC
GO:0051239	0.0156	1	2	859	regulation of multicellular organismal process	BP	1	JUP,GATA5	down_VFEM_VSMC
GO:0051146	0.0156	0.999	2	76	striated muscle cell differentiation	BP	1	NKX2-5,CYP26B1	up_VPOR_VSMC
GO:0051657	0.0156	1	1	7	maintenance of organelle location	BP	1	GPSM2	down_AFEM_EC
GO:1990126	0.0156	1	1	8	retrograde transport, endosome to plasma	BP	1	RAB11B	down_VPOR_EC
GO:0014896	0.0156	1	1	18	muscle hypertrophy	BP	1	GATA5	down_VSAP_VSMC
GO:0014897	0.0156	1	1	18	striated muscle hypertrophy	BP	1	GATA5	down_VSAP_VSMC
GO:0003300	0.0156	1	1	18	cardiac muscle hypertrophy	BP	1	GATA5	down_VSAP_VSMC
GO:0070897	0.0156	1	1	16	transcription preinitiation complex assembly	BP	1	ESR1	up_AORT_VSMC
GO:0003263	0.0156	1	1	4	cardioblast proliferation	BP	1	TBX5	up_APUL_EC
GO:0010611	0.0156	1	1	11	regulation of cardiac muscle hypertrophy	BP	1	GATA5	up_VCAV_VSMC

GO:0014743	0.0156	1	1	11	regulation of muscle hypertrophy	BP	1	GATA5	up_VCAV_VSMC
GO:0048643	0.0156	1	1	7	positive regulation of skeletal muscle tissue	BP	1	SHOX2	up_VFEM_VSMC
GO:1903044	0.0156	1	1	3	protein localization to membrane raft	BP	1	CAV1	up_VSAP_VSMC
GO:0038061	0.0157	0.995	8	34	NIK/NF-kappaB signaling	BP	1	RASSF2,TLR3,SASH1,PTPN22,APP,NOD2,MAP3K7,IL18R1	up_ACOR_VSMC
GO:0070059	0.0157	0.997	5	25	intrinsic apoptotic signaling pathway in response to endoplasmic	BP	0.6	ERO1A,HYOU1,DNAJC10,PTPN2,DDIT3	down_VSAP_EC
GO:0035295	0.0157	0.997	5	356	tube development	BP	1	EYA1,HOXB7,HOXA7,SIX1,MTSS1	up_VFEM_EC
GO:0008285	0.0157	0.997	4	201	negative regulation of cell proliferation	BP	0.865	ACVRL1,LYN,PDPN,SULF1	down_ACOR_EC
GO:0003013	0.0157	0.998	3	121	circulatory system process	BP	1	IRX5,CAV1,F5	up_VSAP_VSMC
GO:0034433	0.0157	0.999	2	4	steroid esterification	BP	1	LCAT,SOAT1	down_VCAV_EC
GO:0034434	0.0157	0.999	2	4	sterol esterification	BP	1	LCAT,SOAT1	down_VCAV_EC
GO:0034435	0.0157	0.999	2	4	cholesterol esterification	BP	1	LCAT,SOAT1	down_VCAV_EC
GO:0007389	0.0157	0.999	2	137	pattern specification process	BP	1	HOXC6,GATA5	up_VCAV_VSMC
GO:0046661	0.0157	0.999	2	45	male sex differentiation	BP	1	HOXA11,HOXA9	up_VSAP_VSMC
GO:0030033	0.0157	1	1	7	microvillus assembly	BP	1	FSCN1	down_AFEM_EC
GO:0071801	0.0157	1	1	7	regulation of podosome assembly	BP	1	FSCN1	down_AFEM_EC
GO:0032717	0.0157	1	1	7	negative regulation of interleukin-8 production	BP	1	BPI	up_AORT_EC
GO:0003211	0.0157	1	1	4	cardiac ventricle formation	BP	1	TBX5	up_APUL_EC
GO:0034383	0.0157	1	1	4	low-density lipoprotein particle clearance	BP	1	NR1H4	up_APUL_EC
GO:0046548	0.0157	1	1	4	retinal rod cell development	BP	1	NTRK2	up_APUL_EC
GO:0060914	0.0157	1	1	11	heart formation	BP	1	GATA5	up_VCAV_VSMC
GO:0098722	0.0157	1	1	2	asymmetric stem cell division	BP	1	ARHGEF2	up_VPOR_EC
GO:0010960	0.0157	1	1	6	magnesium ion homeostasis	BP	1	HOXA3	up_VPOR_VSMC
GO:0060841	0.0157	1	1	6	venous blood vessel development	BP	1	NKX2-5	up_VPOR_VSMC
GO:0090399	0.0157	1	1	4	replicative senescence	BP	1	MME	up_VFEM_EC
GO:0050829	0.0157	1	1	8	defense response to Gram-negative bacterium	BP	1	IL22RA1	up_VPUL_VSMC
GO:0016264	0.0157	1	1	3	gap junction assembly	BP	1	CAV1	up_VSAP_VSMC
GO:0032680	0.01571387	0.99734	4	36	regulation of tumor necrosis factor production	BP	0.96436359	NR1H4,FZD5,NOD2,MAVS	up_ACOR_EC
GO:0051128	0.015746715	0.9909	31	790	regulation of cellular component organization	BP	0.96436359	CNTNAP4,TMOD1,BRSK1,PKIB,CALY,RHOU,ARHGAP6,SYNPO2,FZD5,DIXDC1,RGMA,SMAD6,PLS1,ACTN2,ARHGAP28,ESM1,PAK3,PTGER4,ARHGDIB,PREB,MAVS,LRP4,CLSTN1,LRRC4,CLU,CHMP4B,VPS16,NRP1,PRKCD,PYGO2,TUBB4A	up_ACOR_EC
GO:0055114	0.0158	0.992	24	157	oxidation-reduction process	BP	1	PEX7,ACAA1,ACACB,PEX2,DLST,C1QTNF2,COQ9,NDUFS1,ENSCAFG00000004260,SLC25A12,PHKA2,FXN,GCDH,NDUFS2,PASK,EHHADH,LONP2,MTFR1L,ENSCAFG00000001866,IDH1,IDH3A,ENSCAFG00000006639,NDUFV1,HMOX1	up_VSAP_EC
GO:0040011	0.0158	0.992	20	505	locomotion	BP	1	CMKLR1,ANGPT4,HOXA7,PLXNA4,TWIST2,HCK,GREM1,HTR1D,CAMK2A,LRRC15,RECK,TBXA2R,BVES,LGR6,ACKR4,RAB7A,CCL5,FAP,ADAM17,KANK1	up_AFEM_EC
GO:0071695	0.0158	0.995	9	45	anatomical structure maturation	BP	0.545	EDNRB,FERMT1,CDH5,GATA3,NTN4,BMP2,SPTBN4,HIF1A,FBXO5	up_VCAV_EC
GO:0055024	0.0158	0.996	6	22	regulation of cardiac muscle tissue	BP	1	BMP4,FGFR2,TBX20,TBX5,FRS2,EDN1	up_ACOR_VSMC
GO:1902680	0.0158	0.998	4	415	positive regulation of RNA biosynthetic process	BP	1	EYA1,HOXA9,HOXA11,SHOX2	up_VFEM_VSMC
GO:1903508	0.0158	0.998	4	415	positive regulation of nucleic acid-templated	BP	1	EYA1,HOXA9,HOXA11,SHOX2	up_VFEM_VSMC

GO:0045668	0.0158	0.998	4	11	negative regulation of osteoblast differentiation	BP	1	TWIST2,AXIN2,HDAC7,LIMD1	up_VSAP_EC
GO:0002043	0.0158	0.999	3	7	blood vessel endothelial cell proliferation involved in sprouting angiogenesis	BP	0.545	DLL4,APLNR,JCAD	up_VCAV_EC
GO:0034755	0.0158	0.999	2	4	iron ion transmembrane transport	BP	1	SCARA5,ARHGAP1	down_VCAV_EC
GO:0044057	0.0158	1	1	110	regulation of system process	BP	1	GATA5	down_AFEM_VSMC
GO:0046822	0.0158	1	1	53	regulation of nucleocytoplasmic	BP	1	JUP	down_VFEM_VSMC
GO:0009593	0.0158	1	1	11	detection of chemical stimulus	BP	1	TLR2	up_AFEM_VSMC
GO:0048333	0.0158	1	1	12	mesodermal cell differentiation	BP	1	EYA1	up_AFEM_VSMC
GO:0060964	0.0158	1	1	13	regulation of gene silencing by miRNA	BP	1	ESR1	up_AORT_VSMC
GO:0051890	0.0158	1	1	4	regulation of cardioblast differentiation	BP	1	TBX5	up_APUL_EC
GO:0070857	0.0158	1	1	4	regulation of bile acid biosynthetic process	BP	1	NR1H4	up_APUL_EC
GO:0071941	0.0158	1	1	4	nitrogen cycle metabolic process	BP	1	NR1H4	up_APUL_EC
GO:1904251	0.0158	1	1	4	regulation of bile acid metabolic process	BP	1	NR1H4	up_APUL_EC
GO:0043254	0.0159	0.992	22	164	regulation of protein complex assembly	BP	0.545	HCLS1,GBP5,SELP,BAIAP2L1,TLR4,IRF8,TMOD1,MTOR,CNOT6,NCKAP1,RIOK3,IQGAP2,STMN1,NCK1,FES,SENP6,PAN3,HMGB1,PMAIP1,ARPC5L,TMOD3,FSCN1	up_VCAV_EC
GO:0048568	0.0159	0.992	20	148	embryonic organ development	BP	0.545	HOXB7,PCDH12,KIT,CXCL8,PDGFB,MEF2C,CHD7,GATA3,EDN1,HOXB8,SOX18,HHEX,PROX1,MDFI,HOXB5,HIF1A,ALDH1A3,RBBP6,VASH1,ZEB1	up_VCAV_EC
GO:0030098	0.0159	0.993	15	87	lymphocyte differentiation	BP	1	ABL1,DLL1,ZBTB16,TOX,STAT6,PATZ1,ZFP36L1,PIK3R1,ZEB1,FBXO7,CD83,ZFP36L2,PNP,PP3CB,TGFBR2	up_VSAP_EC
GO:0006401	0.0159	0.993	14	88	RNA catabolic process	BP	0.629	RNPS1,EIF4A3,EDC3,HNRNPD,THRAP3,EDC4,FEN1,RNASEH2B,SMG9,DIS3,FASTKD3,ENSCAFG00000015189,RNASEH2C,EXOSC5	down_ACOR_VSMC
GO:0110020	0.0159	0.996	7	31	regulation of actomyosin structure organization	BP	0.546	ARHGEF15,MEF2C,EDN1,ARHGAP28,MTOR,PROX1,STMN1	up_VCAV_EC
GO:0010038	0.0159	0.997	5	62	response to metal ion	BP	1	MT2A,ENSCAFG00000009113,CPNE7,FIBIN,ANXA11	up_AFEM_EC
GO:0060004	0.0159	0.999	3	6	reflex	BP	1	GJA1,GLRB,NPNT	up_ACOR_VSMC
GO:0002761	0.0159	0.999	2	36	regulation of myeloid leukocyte differentiation	BP	0.87	LYN,HOXA7	down_ACOR_EC
GO:0060740	0.0159	1	1	13	prostate gland epithelium morphogenesis	BP	1	ESR1	up_AORT_VSMC
GO:0021517	0.0159	1	1	12	ventral spinal cord development	BP	1	HOXC10	up_AORT_VSMC
GO:0003208	0.0159	1	1	24	cardiac ventricle morphogenesis	BP	1	TBX5	up_APUL_VSMC
GO:0003203	0.0159	1	1	11	endocardial cushion morphogenesis	BP	1	GATA5	up_VCAV_VSMC
GO:0021542	0.0159	1	1	6	dentate gyrus development	BP	1	EMX2	up_VFEM_VSMC
GO:1903828	0.015995754	0.99727	4	38	negative regulation of cellular protein	BP	0.96436359	ACTN2,DCLK2,LRRC15,TP53INP2	up_ACOR_EC
GO:0046903	0.016	0.99	35	255	secretion	BP	1	EXPH5,TLR1,LEP,SLC26A7,HTR2B,MBP,FBXL20,FERMT1,SYT16,TM7SF3,ABCA1,GJA1,CD274,STX11,ANKRD1,F2RL1,HRH2,PTPN22,PRKCE,SCG5,PSEN1,PCSK6,CHD7,CCL5,RAB11B,EDN1,KCNC4,NOD2,SCAMP5,SCRN2,CFI,PREPL,PFKL,PARK7,LMF1	up_ACOR_VSMC
GO:0030278	0.016	0.995	8	66	regulation of ossification	BP	1	BMPR1A,CLSTN1,GLI3,TWIST2,DDR2,OMD,GREM1,ZBTB16	down_VCAV_EC
GO:0032677	0.016	0.996	6	22	regulation of interleukin-8 production	BP	1	TLR1,TLR3,F2RL1,PTPN22,NOD2,PARK7	up_ACOR_VSMC
GO:0009889	0.016	0.997	5	1190	regulation of biosynthetic process	BP	1	TLR2,EYA1,HOXA9,HOXA10,IRX5	up_AFEM_VSMC
GO:0032663	0.016	0.997	5	19	regulation of interleukin-2 production	BP	0.546	TNFAIP3,PDE4D,GATA3,HOMER3,PRKD2	up_VCAV_EC
GO:0003002	0.016	0.997	4	118	regionalization	BP	1	BARX1,HOXA3,SEMA3A,ACVR2A	up_VPOR_EC
GO:0010769	0.016	0.998	3	69	regulation of cell morphogenesis involved	BP	1	ENSCAFG00000002015,SEMA3A,RNF6	up_VPOR_EC
GO:0034762	0.016	0.998	3	122	regulation of transmembrane transport	BP	1	NETO1,STIM1,CAV1	up_VSAP_VSMC

GO:0071674	0.016	0.999	2	23	mononuclear cell migration	BP	1	C3AR1,ENSCAFG00000032731	up_VPOR_EC
GO:0009913	0.016	0.999	2	47	epidermal cell differentiation	BP	1	HOXA7,CYP26B1	up_VFEM_EC
GO:0002089	0.016	1	1	8	lens morphogenesis in camera-type eye	BP	1	CITED2	down_VPOR_EC
GO:0021517	0.016	1	1	12	ventral spinal cord development	BP	1	HOXC10	up_AFEM_VSMC
GO:0050702	0.016	1	1	13	interleukin-1 beta secretion	BP	1	ARG2	up_AORT_VSMC
GO:0006972	0.016	1	1	2	hyperosmotic response	BP	1	ARHGEF2	up_VPOR_EC
GO:0010891	0.016	1	1	2	negative regulation of sequestering of	BP	1	ABHD5	up_VPOR_EC
GO:0071474	0.016	1	1	2	cellular hyperosmotic response	BP	1	ARHGEF2	up_VPOR_EC
GO:0060413	0.016	1	1	8	atrial septum morphogenesis	BP	1	TBX5	up_VPUL_VSMC
GO:0003283	0.016	1	1	8	atrial septum development	BP	1	TBX5	up_VPUL_VSMC
GO:0044854	0.016	1	1	3	plasma membrane raft assembly	BP	1	CAV1	up_VSAP_VSMC
GO:0044857	0.016	1	1	3	plasma membrane raft organization	BP	1	CAV1	up_VSAP_VSMC
GO:0001932	0.0161	0.99	47	416	regulation of protein phosphorylation	BP	0.546	EPHB1,TEK,HCLS1,KIT,TNFAIP3,MAP3K11,TNIP1,PDGFB,STK10,RELN,PDE4D,ATF3,RAPGEF1,TLR4,EDN1,RALB,APLNR,NEDD9,YES1,MTOR,MAP3K3,BMP2,HHEX,PREX2,SLK,SH3BP5,ITPKB,PROX1,MADD,PAK2,PRKD2,TLR3,NCK1,NSD1,PTGS2,MMD,RAPGEF2,TAOK1,CNOT7,HMGB1,BLM,SPTBN4,RIPK1,PDGFD,JCAD,ARHGEF2,CDON	up_VCAV_EC
GO:0014070	0.0161	0.991	23	172	response to organic cyclic compound	BP	0.546	PDGFB,P2RX7,TMEM173,SELL,PDE4D,MEF2C,TIPARP,RAPGEF1,RALB,TFPI,PELI1,BMP2,RIOK3,PAK2,SMARCA4,TLR3,RAPGEF2,CTNNA1,PMAIP1,BLM,UFSP2,KDM3A,P2RX2	up_VCAV_EC
GO:0032102	0.0161	0.994	11	67	negative regulation of response to external	BP	0.546	TNFAIP3,PDGFB,CD34,KLKB1,GATA3,MMP28,TFPI,ARG2,RIOK3,ANGPT2,APOD	up_VCAV_EC
GO:1903523	0.0161	0.998	4	10	negative regulation of blood circulation	BP	1	KCNE3,PDE5A,RNLS,DOCK5	up_ACOR_VSMC
GO:0007252	0.0161	0.999	3	6	I-kappaB phosphorylation	BP	0.546	TLR4,ERC1,TLR3	up_VCAV_EC
GO:0034447	0.0161	0.999	2	4	very-low-density lipoprotein particle	BP	0.613	APOE,VLDLR	down_VSAP_EC
GO:0048675	0.0161	0.999	2	26	axon extension	BP	1	SEMA3A,RNF6	up_VPOR_EC
GO:0051271	0.0161	0.999	2	98	negative regulation of cellular component	BP	1	TBX5,SLIT2	up_VPUL_VSMC
GO:0032703	0.0161	1	1	8	negative regulation of interleukin-2 production	BP	1	HDAC7	down_VPOR_EC
GO:0060047	0.0161	1	1	57	heart contraction	BP	1	JUP	down_VFEM_VSMC
GO:0072606	0.0161	1	1	12	interleukin-8 secretion	BP	1	TLR2	up_AFEM_VSMC
GO:0042698	0.0161	1	1	14	ovulation cycle	BP	1	ESR1	up_AORT_VSMC
GO:0055083	0.0161	1	1	6	monovalent inorganic anion homeostasis	BP	1	HOXA3	up_VPOR_VSMC
GO:0072102	0.0161	1	1	4	glomerulus morphogenesis	BP	1	MTSS1	up_VFEM_EC
GO:1903817	0.0161	1	1	3	negative regulation of voltage-gated potassium	BP	1	CAV1	up_VSAP_VSMC
GO:0001501	0.0162	0.992	16	178	skeletal system development	BP	1	NOTCH2,SMAD9,NAB2,FBN1,THBS3,BMPR1A,TBX15,HOXC4,HAPLN3,VCAN,GLI3,ENSCAFG00000017708,IRX5,RDH10,GREM1,ZBTB16	down_VCAV_EC
GO:0032092	0.0162	1	1	38	positive regulation of protein binding	BP	1	PLCL1	down_VFEM_EC
GO:0060071	0.0162	1	1	14	Wnt signaling pathway, planar cell polarity	BP	1	WNT7A	up_AORT_VSMC
GO:0014912	0.0162	1	1	8	negative regulation of smooth muscle cell	BP	1	SLIT2	up_VPUL_VSMC
GO:0046621	0.0162	1	1	8	negative regulation of organ growth	BP	1	TBX5	up_VPUL_VSMC
GO:1902105	0.0163	0.993	13	80	regulation of leukocyte differentiation	BP	0.549	HCLS1,IL7R,GATA3,CASP8,MTOR,ITPKB,CREB1,HMGB1,RIPK1,ATP11C,CUL4A,ZEB1,TJP2	up_VCAV_EC
GO:0019219	0.0163	0.996	7	1151	regulation of nucleobase-containing compound metabolic process	BP	1	EYA1,HOXA10,HOXA9,FBP1,EMX2,HOXA11,SHOX2	up_VFEM_VSMC

GO:0048869	0.0163	0.997	6	1119	cellular developmental process	BP	1	TBX5,GATA5,TMEM176A,TMEM176B,HOXB5,SLIT2	up_VPUL_VSMC
GO:0070085	0.0163	0.999	2	95	glycosylation	BP	1	MGAT4B,GBGT1	down_VPOR_EC
GO:0007160	0.0163	1	1	56	cell-matrix adhesion	BP	1	JUP	down_VFEM_VSMC
GO:0007338	0.0163	1	1	16	single fertilization	BP	1	HOXA9	up_AORT_VSMC
GO:0043586	0.0163	1	1	6	tongue development	BP	1	CYP26B1	up_VPOR_VSMC
GO:0032332	0.0163	1	1	6	positive regulation of chondrocyte	BP	1	HOXA11	up_VFEM_VSMC
GO:1901362	0.0164	0.994	11	1293	organic cyclic compound biosynthetic process	BP	1	HOXA11,TBX15,HOXA10,HOXA9,PITX1,EMX2,SALL1,IRX5,TLR2,CAV1,SHOX2	up_VSAP_VSMC
GO:0019319	0.0164	0.997	5	26	hexose biosynthetic process	BP	0.621	TPI1,PGAM1,SESN2,PTPN2,ATF3	down_VSAP_EC
GO:0007259	0.0164	0.999	2	42	JAK-STAT cascade	BP	0.891	LYN,ASPN	down_ACOR_EC
GO:0002221	0.0164	0.999	2	46	pattern recognition receptor signaling	BP	1	TLR2,CAV1	up_VSAP_VSMC
GO:0002758	0.0164	0.999	2	46	innate immune response-activating signal	BP	1	TLR2,CAV1	up_VSAP_VSMC
GO:0001893	0.0164	1	1	8	maternal placenta development	BP	1	CITED2	down_VPOR_EC
GO:0003015	0.0164	1	1	58	heart process	BP	1	JUP	down_VFEM_VSMC
GO:0010092	0.0164	1	1	19	specification of animal organ identity	BP	1	GATA5	down_VSAP_VSMC
GO:0003179	0.0164	1	1	19	heart valve morphogenesis	BP	1	GATA5	down_VSAP_VSMC
GO:0060795	0.0164	1	1	12	cell fate commitment involved in formation of	BP	1	EYA1	up_AFEM_VSMC
GO:0061028	0.0164	1	1	14	establishment of endothelial barrier	BP	1	WNT7A	up_AORT_VSMC
GO:0045844	0.0164	1	1	24	positive regulation of striated muscle tissue	BP	1	TBX5	up_APUL_VSMC
GO:0048636	0.0164	1	1	24	positive regulation of muscle organ	BP	1	TBX5	up_APUL_VSMC
GO:0007320	0.0164	1	1	2	insemination	BP	1	ACVR2A	up_VPOR_EC
GO:0007620	0.0164	1	1	2	copulation	BP	1	ACVR2A	up_VPOR_EC
GO:2000484	0.0164	1	1	4	positive regulation of interleukin-8 secretion	BP	1	TLR2	up_VSAP_VSMC
GO:0098542	0.01640875	0.99518	7	100	defense response to other organism	BP	0.96436359	NR1H4,NOD2,MAVS,IFIT2,RARRES2,AGBL5,PRKCD	up_ACOR_EC
GO:0009719	0.0165	0.996	6	395	response to endogenous stimulus	BP	0.891	ACVRL1,LYN,IGF1R,SULF1,ASPN,NEDD4	down_ACOR_EC
GO:0007031	0.0165	0.997	4	20	peroxisome organization	BP	1	SCP2,PEX5,MAVS,LONP2	down_VCAV_EC
GO:0045746	0.0165	0.997	4	14	negative regulation of Notch signaling pathway	BP	0.555	DLL4,EGFL7,GDPD5,PEAR1	up_VCAV_EC
GO:0044089	0.0165	0.999	2	170	positive regulation of cellular component	BP	1	WNT7A,ESR1	up_AORT_VSMC
GO:0007596	0.0165	0.999	2	47	blood coagulation	BP	1	CAV1,F5	up_VSAP_VSMC
GO:0034135	0.0165	1	1	4	regulation of toll-like receptor 2 signaling	BP	0.891	LYN	down_ACOR_EC
GO:0010447	0.0165	1	1	8	response to acidic pH	BP	1	RAB11B	down_VPOR_EC
GO:0043534	0.0165	1	1	57	blood vessel endothelial cell migration	BP	1	JUP	down_VFEM_VSMC
GO:1903522	0.0165	1	1	58	regulation of blood circulation	BP	1	JUP	down_VFEM_VSMC
GO:0042474	0.0165	1	1	11	middle ear morphogenesis	BP	1	EYA1	up_AFEM_VSMC
GO:0022603	0.0166	0.991	28	301	regulation of anatomical structure morphogenesis	BP	0.628	VEGFA,SIPA1L1,SULF1,MYH9,FMNL3,SERPINE1,SPARC,JCAD,PGK1,AKAP2,IFRD1,WDR1,PIK3C2A,MSN,HOXB7,HIF1A,THBS2,TNIK,MTDH,UST,MAGED1,CTNNB1,STARD13,ENSCAFG00000002015,BNIP3,SIX4,NRP1,YWHAH	down_VSAP_EC
GO:0055085	0.0166	0.991	23	284	transmembrane transport	BP	1	PRNP,FOLR2,FXYD1,CLIP3,ENSCAFG00000014978,AKT2,SLC39A6,CACNB3,ATP5MC1,THY1,PEX5,MFSD3,ATP6V0C,PARK7,SLC17A9,SCARA5,SLC2A10,CYBA,SPNS1,SEC61B,ARHGAP1,LONP2,CLIC4	down_VCAV_EC

GO:0050877	0.0166	0.991	22	164	nervous system process	BP	0.556	EPHB1,PLK2,KIT,PTCHD1,ATP8A1,RELN,MEF2C,CHD7,HOXB8,HOMER3,PPP3CA,TMEM150C,NAV2,ACPP,MMP24,CREB1,GCH1,SPTBN4,HIF1A,ALDH1A3,DOP1B,P2RX2	up_VCAV_EC
GO:0001568	0.0166	0.993	12	252	blood vessel development	BP	1	LEP,ANGPT4,HOXA7,DLL1,F3,TMEM100,GREM1,LUZP1,RECK,TBXA2R,PTPRB,CLIC4	up_AFEM_EC
GO:0034440	0.0166	0.995	8	35	lipid oxidation	BP	1	PEX7,ACAA1,ACACB,PEX2,C1QTNF2,GCDH,EHHADH,LONP2	up_VSAP_EC
GO:0046928	0.0166	0.998	3	23	regulation of neurotransmitter	BP	1	KCNC4,CHRM2,CAMK2A	up_AFEM_EC
GO:0045649	0.0166	0.998	3	8	regulation of macrophage differentiation	BP	0.556	HCLS1,CASP8,RIPK1	up_VCAV_EC
GO:0006167	0.0166	1	1	2	AMP biosynthetic process	BP	0.891	TNRC6B	down_ACOR_EC
GO:0044208	0.0166	1	1	2	'de novo' AMP biosynthetic process	BP	0.891	TNRC6B	down_ACOR_EC
GO:0032927	0.0166	1	1	2	positive regulation of activin receptor signaling	BP	1	ACVR2A	up_VPOR_EC
GO:0060011	0.0166	1	1	2	Sertoli cell proliferation	BP	1	ACVR2A	up_VPOR_EC
GO:0006617	0.0166	1	1	2	SRP-dependent cotranslational protein targeting to membrane,	BP	1	SRP54	up_VPOR_EC
GO:0014841	0.0166	1	1	4	skeletal muscle satellite cell proliferation	BP	1	SIX1	up_VFEM_EC
GO:0014842	0.0166	1	1	4	regulation of skeletal muscle satellite cell	BP	1	SIX1	up_VFEM_EC
GO:0014856	0.0166	1	1	4	skeletal muscle cell proliferation	BP	1	SIX1	up_VFEM_EC
GO:0014857	0.0166	1	1	4	regulation of skeletal muscle cell proliferation	BP	1	SIX1	up_VFEM_EC
GO:0031295	0.0166	1	1	3	T cell costimulation	BP	1	CAV1	up_VSAP_VSMC
GO:0009968	0.016654157	0.99183	18	392	negative regulation of signal transduction	BP	0.96436359	NR1H4,SOX9,NDRG2,CCDC3,IFI6,DUSP26,PTGIR,SMAD6,ADAMTSL2,MAPK8IP2,FIGNL1,LRRC15,LRP4,CLSTN1,LRRC4,CLU,NRP1,PRKCD	up_ACOR_EC
GO:0008285	0.0167	0.991	20	201	negative regulation of cell proliferation	BP	0.628	SULF1,SPARC,PTPN14,ILK,PTPN2,SKAP2,CNOT7,MAGED1,CTNNB1,APOE,LYN,STRAP,PDS5B,TAX1BP3,SDC4,REST,ENSCAFG00000017326,INHBA,BTK,CDKN1A	down_VSAP_EC
GO:0042221	0.0167	0.995	8	1012	response to chemical	BP	1	NR1H4,MRC1,CCL24,PTGER2,FLRT3,ARHGEF2,NEO1,MXRA5	up_VPUL_EC
GO:0035270	0.0167	0.995	8	35	endocrine system development	BP	1	WNT11,HNF4A,IL6R,HOXD3,DLL1,RAF1,MAP2K2,CITED2	up_VSAP_EC
GO:0007157	0.0167	0.998	4	12	heterophilic cell-cell adhesion via plasma membrane cell adhesion	BP	0.559	SELP,SELE,NECTIN2,CD200	up_VCAV_EC
GO:0090190	0.0167	0.998	3	10	positive regulation of branching involved in ureteric bud	BP	0.629	HOXB7,MAGED1,SIX4	down_VSAP_EC
GO:0044703	0.0167	0.999	2	172	multi-organism reproductive process	BP	1	HOXA9,ESR1	up_AORT_VSMC
GO:0040013	0.0167	0.999	2	100	negative regulation of locomotion	BP	1	TBX5,SLIT2	up_VPUL_VSMC
GO:0030851	0.0167	1	1	8	granulocyte differentiation	BP	1	CITED2	down_VPOR_EC
GO:0007219	0.0167	1	1	59	Notch signaling pathway	BP	1	GATA5	down_VFEM_VSMC
GO:0045793	0.0167	1	1	4	positive regulation of cell size	BP	1	PRR16	up_VSAP_VSMC
GO:0006469	0.016711957	0.99564	6	78	negative regulation of protein kinase activity	BP	0.96436359	PKIB,DUSP26,LRRC15,LRRC4,PRKCD,DBNDD2	up_ACOR_EC
GO:0051902	0.016718356	1	1	1	negative regulation of mitochondrial	BP	0.96436359	IFI6	up_ACOR_EC
GO:1904180	0.016718356	1	1	1	negative regulation of membrane depolarization	BP	0.96436359	IFI6	up_ACOR_EC
GO:0010758	0.016746609	0.99914	2	8	regulation of macrophage chemotaxis	BP	0.96436359	MMP28,RARRES2	up_ACOR_EC
GO:0099072	0.016774117	0.99914	2	8	regulation of postsynaptic membrane neurotransmitter	BP	0.96436359	CALY,PORCN	up_ACOR_EC
GO:0009887	0.0168	0.993	14	314	animal organ morphogenesis	BP	1	TBX15,IRX3,IRX5,HOXA7,COL8A2,DLL1,THRB,TMEM100,GREM1,HOXA6,CNNM4,AXIN2,GCNT4,TNC	up_AFEM_EC
GO:0050730	0.0168	0.994	11	66	regulation of peptidyl-tyrosine phosphorylation	BP	0.559	HCLS1,KIT,PDGFB,RELN,NEDD9,YES1,MTOR,SH3BP5,CNOT7,PDGFD,ARHGEF2	up_VCAV_EC
GO:0051240	0.0168	0.996	6	489	positive regulation of multicellular organismal	BP	1	HOXA11,NETO1,GATM,TLR2,CAV1,SHOX2	up_VSAP_VSMC

GO:0010941	0.0168	0.998	3	524	regulation of cell death	BP	1	WNT7A,ARG2,ESR1	up_AORT_VSMC
GO:2001214	0.0168	1	2	3	positive regulation of vasculogenesis	BP	0.559	CD34,RAPGEF2	up_VCAV_EC
GO:0003002	0.0168	1	1	118	regionalization	BP	1	GATA5	down_AFEM_VSMC
GO:0022027	0.0168	1	1	2	interkinetic nuclear migration	BP	1	HOOK3	up_VPOR_EC
GO:0010664	0.0168	1	1	6	negative regulation of striated muscle cell	BP	1	NKX2-5	up_VPOR_VSMC
GO:0034162	0.0168	1	1	5	toll-like receptor 9 signaling pathway	BP	1	NR1H4	up_VPUL_EC
GO:1903555	0.016867718	0.99708	4	37	regulation of tumor necrosis factor superfamily cytokine	BP	0.96436359	NR1H4,FZD5,NOD2,MAVS	up_ACOR_EC
GO:0045860	0.0169	0.992	19	132	positive regulation of protein kinase activity	BP	0.559	KIT,MAP3K11,PDGFB,STK10,RELN,TLR4,EDN1,RALB,NEDD9,MAP3K3,BMP2,SLK,PROX1,MADD,PAK2,MMD,RAPGEF2,RIPK1,PDGFD	up_VCAV_EC
GO:0030155	0.0169	0.992	16	180	regulation of cell adhesion	BP	1	GPAM,FBLN2,PRNP,SDC4,RHOD,ENSCAFG00000014978,TFE3,LAMA3,THY1,GLI3,EDIL3,LOXL3,GREM1,RAC2,ZBTB16,ECM2	down_VCAV_EC
GO:1901342	0.0169	0.993	13	114	regulation of vasculature development	BP	0.633	VEGFA,SULF1,SERPINE1,SPARC,JCAD,PGK1,PIK3C2A,HIF1A,THBS2,MTDH,CTNNB1,STARD13,NRP1	down_VSAP_EC
GO:0002224	0.0169	0.995	7	34	toll-like receptor signaling pathway	BP	0.559	TNFAIP3,TNIP1,TLR4,PELI1,TLR3,OTUD4,HMGB1	up_VCAV_EC
GO:0051299	0.0169	0.998	3	7	centrosome separation	BP	0.659	NEK2,NUP62,KIF11	down_ACOR_VSMC
GO:0048513	0.0169	0.999	3	906	animal organ development	BP	1	HOXA3,TBX5,HOXB5	up_APUL_VSMC
GO:0002520	0.0169	0.998	3	279	immune system development	BP	1	HOXA3,TMEM176A,TMEM176B	up_VPUL_VSMC
GO:1905330	0.0169	0.999	2	48	regulation of morphogenesis of an	BP	1	HOXB7,SIX1	up_VFEM_EC
GO:0042996	0.0169	1	1	4	regulation of Golgi to plasma membrane	BP	0.9	ACSL3	down_ACOR_EC
GO:0017145	0.0169	1	1	13	stem cell division	BP	1	WNT7A	up_AORT_VSMC
GO:0060512	0.0169	1	1	14	prostate gland morphogenesis	BP	1	ESR1	up_AORT_VSMC
GO:1905314	0.0169	1	1	12	semi-lunar valve development	BP	1	GATA5	up_VCAV_VSMC
GO:0010159	0.0169	1	1	2	specification of animal organ position	BP	1	HOXA3	up_VPOR_EC
GO:2000095	0.0169	1	1	6	regulation of Wnt signaling pathway, planar cell polarity pathway	BP	1	ANKRD6	up_VPOR_VSMC
GO:1900084	0.0169	1	1	3	regulation of peptidyl-tyrosine	BP	1	CAV1	up_VSAP_VSMC
GO:0032331	0.01695558	0.99912	2	8	negative regulation of chondrocyte	BP	0.96436359	SOX9,EFEMP1	up_ACOR_EC
GO:0061037	0.01695558	0.99912	2	8	negative regulation of cartilage development	BP	0.96436359	SOX9,EFEMP1	up_ACOR_EC
GO:0032620	0.016969193	0.99912	2	8	interleukin-17 production	BP	0.96436359	NR1H4,NOD2	up_ACOR_EC
GO:0051094	0.017	0.989	50	388	positive regulation of developmental process	BP	1	HOXA11,NAPEPLD,CDON,CD274,AXIN2,WASF3,OSR2,AMIGO1,ENPP2,LRRN3,PPARGC1B,ACACB,DDHD1,ZHX3,HOXD3,FGF7,ABL1,BTG1,HDAC7,DLL1,OSR1,MAP3K5,SAV1,HPSE,ANKRD54,ZBTB16,TOX,CCBE1,STAT6,DDR2,ZFP36L1,IRX3,ZEB1,SHOX2,SASH1,GDPD5,CD83,KDM2B,YPEL3,PNP,CYB5D2,ACVRL1,FLOT1,NCK1,HMOX1,MAP2K2,TGFBR2,CITED2,TRADD,LBH	up_VSAP_EC
GO:0002694	0.017	0.992	18	126	regulation of leukocyte activation	BP	0.559	IL7R,ADGRF5,TNFAIP3,MEF2C,TLR4,GATA3,PELI1,ARG2,NECTIN2,ITPKB,THOC1,NCK1,CD200,FES,BCL10,HMGB1,ATP11C,ZEB1	up_VCAV_EC
GO:0051607	0.017	0.994	10	58	defense response to virus	BP	0.559	MX2,TMEM173,NLRC5,SERINC5,RIOK3,TLR3,OAS3,CNOT7,PMAIP1,LSM14A	up_VCAV_EC
GO:0061333	0.017	0.996	6	35	renal tubule morphogenesis	BP	0.635	ENSCAFG00000032470,ILK,HOXB7,MAGED1,CTNNB1,SIX4	down_VSAP_EC
GO:0010972	0.017	0.997	5	18	negative regulation of G2/M transition of mitotic	BP	0.559	TAOK1,BLM,RAD21,RAD17,TOPBP1	up_VCAV_EC
GO:0003073	0.017	0.997	5	20	regulation of systemic arterial blood pressure	BP	0.559	PRCP,PDGFB,EDN1,NAV2,P2RX2	up_VCAV_EC
GO:1900026	0.017	0.998	4	11	positive regulation of substrate adhesion-dependent cell spreading	BP	1	NRP1,DOCK5,RREB1,CRKL	up_ACOR_VSMC
GO:0070206	0.017	0.998	4	11	protein trimerization	BP	1	C1QTNF2,SCARA5,CLYBL,HTRA2	up_VSAP_EC

GO:0003184	0.017	0.999	3	6	pulmonary valve morphogenesis	BP	1	SMAD6,BMP4,TBX20	up_ACOR_VSMC
GO:0097696	0.017	0.999	2	43	STAT cascade	BP	0.903	LYN,ASPN	down_ACOR_EC
GO:0060850	0.017	1	2	3	regulation of transcription involved in cell fate	BP	0.559	PPARG,PROX1	up_VCAV_EC
GO:0033135	0.017	1	1	40	regulation of peptidyl-serine phosphorylation	BP	1	PLCL1	down_VFEM_EC
GO:0002832	0.017	1	1	15	negative regulation of response to biotic	BP	1	ARG2	up_AORT_VSMC
GO:0060997	0.017	1	1	14	dendritic spine morphogenesis	BP	1	WNT7A	up_AORT_VSMC
GO:0019433	0.017	1	1	2	triglyceride catabolic process	BP	1	ABHD5	up_VPOR_EC
GO:0034454	0.017	1	1	2	microtubule anchoring at centrosome	BP	1	HOOK3	up_VPOR_EC
GO:0051006	0.017	1	1	2	positive regulation of lipoprotein lipase activity	BP	1	ABHD5	up_VPOR_EC
GO:0061365	0.017	1	1	2	positive regulation of triglyceride lipase activity	BP	1	ABHD5	up_VPOR_EC
GO:0032816	0.017	1	1	4	positive regulation of natural killer cell	BP	1	TOX	up_VFEM_EC
GO:0055026	0.017	1	1	5	negative regulation of cardiac muscle tissue	BP	1	TBX5	up_VPUL_EC
GO:1903706	0.0171	0.992	19	119	regulation of hemopoiesis	BP	1	HOXA7,HOXA9,PPARGC1B,ABL1,DLL1,ANKRD54,ZBTB16,TOX,RARG,ZFP36L1,PIK3R1,MYC,ZEB1,TFE3,FBXO7,CD83,ZFP36L2,PNP,TGFBR2	up_VSAP_EC
GO:0045740	0.0171	0.997	4	12	positive regulation of DNA replication	BP	0.667	PCNA,E2F8,CDK2,GLI1	down_ACOR_VSMC
GO:0048562	0.0171	0.999	2	102	embryonic organ morphogenesis	BP	1	CITED2,TBX15	down_VPOR_EC
GO:0106106	0.0171	0.999	2	48	cold-induced thermogenesis	BP	1	HOXC10,MAP2K6	up_VFEM_EC
GO:0120161	0.0171	0.999	2	48	regulation of cold-induced thermogenesis	BP	1	HOXC10,MAP2K6	up_VFEM_EC
GO:0003156	0.0171	1	1	20	regulation of animal organ formation	BP	1	GATA5	down_VSAP_VSMC
GO:0043171	0.0171	1	1	8	peptide catabolic process	BP	1	LNPEP	up_AORT_EC
GO:1901863	0.0171	1	1	25	positive regulation of muscle tissue	BP	1	TBX5	up_APUL_VSMC
GO:0006171	0.0171	1	1	5	cAMP biosynthetic process	BP	1	ADCY3	up_VFEM_EC
GO:0046058	0.0171	1	1	5	cAMP metabolic process	BP	1	ADCY3	up_VFEM_EC
GO:0072643	0.0171	1	1	5	interferon-gamma secretion	BP	1	NR1H4	up_VPUL_EC
GO:0009306	0.0172	0.991	20	127	protein secretion	BP	1	EXPH5,TLR1,LEP,HTR2B,MBP,FERMT1,TM7SF3,ABCA1,CD274,ANKRD1,F2RL1,PTPN22,PRKCE,CCL5,RAB11B,NOD2,SCAMP5,PFKL,PARK7,LMF1	up_ACOR_VSMC
GO:0032720	0.0172	0.997	4	13	negative regulation of tumor necrosis factor	BP	0.564	TNFAIP3,CD34,TLR4,ARG2	up_VCAV_EC
GO:0097479	0.0172	0.999	2	41	synaptic vesicle localization	BP	0.905	SPG11,STX4	down_ACOR_EC
GO:0019221	0.0172	0.999	2	102	cytokine-mediated signaling pathway	BP	1	IL22RA1,SLIT2	up_VPUL_VSMC
GO:0072009	0.0172	0.999	2	48	nephron epithelium development	BP	1	HOXA11,SALL1	up_VSAP_VSMC
GO:0010810	0.0172	1	1	60	regulation of cell-substrate adhesion	BP	1	JUP	down_VFEM_VSMC
GO:0044319	0.0172	1	1	12	wound healing, spreading of cells	BP	1	WNT7A	up_AORT_VSMC
GO:0003401	0.0172	1	1	15	axis elongation	BP	1	ESR1	up_AORT_VSMC
GO:0090504	0.0172	1	1	12	epiboly	BP	1	WNT7A	up_AORT_VSMC
GO:0090505	0.0172	1	1	12	epiboly involved in wound healing	BP	1	WNT7A	up_AORT_VSMC
GO:0042483	0.0172	1	1	2	negative regulation of odontogenesis	BP	1	ASPN	up_VPOR_EC
GO:0006699	0.0172	1	1	5	bile acid biosynthetic process	BP	1	NR1H4	up_VPUL_EC
GO:0018212	0.0173	0.993	13	81	peptidyl-tyrosine modification	BP	0.564	HCLS1,KIT,PDGFB,RELN,NEDD9,YES1,MTOR,SH3BP5,FES,CNOT7,PDGFD,BMX,ARHGEF2	up_VCAV_EC
GO:0001678	0.0173	0.995	7	29	cellular glucose homeostasis	BP	1	RAB11B,PIK3R1,KCNB1,RAF1,STX4,PPP3CB,RAB11FIP2	up_VSAP_EC
GO:0001885	0.0173	0.997	5	19	endothelial cell development	BP	0.564	PDE4D,RAPGEF1,STC1,SOX18,RAPGEF2	up_VCAV_EC

GO:0018401	0.0173	0.999	2	4	peptidyl-proline hydroxylation to 4-	BP	0.644	P4HB,P4HA1	down_VSAP_EC
GO:0048562	0.0173	0.999	2	102	embryonic organ morphogenesis	BP	1	HOXA3,HOXB5	up_VPUL_VSMC
GO:0007599	0.0173	0.999	2	48	hemostasis	BP	1	CAV1,F5	up_VSAP_VSMC
GO:2000480	0.0173	1	1	4	negative regulation of cAMP-dependent protein	BP	0.905	PRKAR2A	down_ACOR_EC
GO:0033119	0.0173	1	1	8	negative regulation of RNA splicing	BP	1	SRSF6	down_AFEM_EC
GO:2000772	0.0173	1	1	15	regulation of cellular senescence	BP	1	ARG2	up_AORT_VSMC
GO:0005985	0.0173	1	1	2	sucrose metabolic process	BP	1	FBP1	up_VPOR_EC
GO:0005986	0.0173	1	1	2	sucrose biosynthetic process	BP	1	FBP1	up_VPOR_EC
GO:0060681	0.0173	1	1	4	branch elongation involved in ureteric bud	BP	1	SIX1	up_VFEM_EC
GO:0072106	0.0173	1	1	4	regulation of ureteric bud formation	BP	1	SIX1	up_VFEM_EC
GO:0072107	0.0173	1	1	4	positive regulation of ureteric bud formation	BP	1	SIX1	up_VFEM_EC
GO:1905278	0.0173	1	1	4	positive regulation of epithelial tube formation	BP	1	SIX1	up_VFEM_EC
GO:2000136	0.0173	1	1	7	regulation of cell proliferation involved in	BP	1	EYA1	up_VFEM_VSMC
GO:0003264	0.0173	1	1	5	regulation of cardioblast proliferation	BP	1	TBX5	up_VPUL_EC
GO:0003207	0.0173	1	1	5	cardiac chamber formation	BP	1	TBX5	up_VPUL_EC
GO:0003266	0.0173	1	1	5	regulation of secondary heart field cardioblast	BP	1	TBX5	up_VPUL_EC
GO:1901617	0.0174	0.994	10	60	organic hydroxy compound biosynthetic	BP	0.564	GATA3,SPTLC2,BMP2,ITPKB,PROX1,RAPGEF2 ,PLCG2,GCH1,LBR,IMPA1	up_VCAV_EC
GO:0032501	0.0174	0.995	9	1782	multicellular organismal process	BP	1	CHL1,HOXA3,BARX1,NKX2-5,BAMBI,TRPC4,ANKRD6,CYP26B1,C3AR1	up_VPOR_VSMC
GO:0001503	0.0174	0.998	3	109	ossification	BP	0.905	HOXA3,CSGALNACT1,ASPN	down_ACOR_EC
GO:0010557	0.0174	0.998	3	492	positive regulation of macromolecule biosynthetic process	BP	1	HOXA9,WNT7A,ESR1	up_AORT_VSMC
GO:0016339	0.0174	0.999	3	6	calcium-dependent cell-cell adhesion via plasma membrane cell adhesion	BP	0.564	PCDH12,SELP,SELL	up_VCAV_EC
GO:0060055	0.0174	0.998	3	8	angiogenesis involved in wound healing	BP	0.564	PRCP,GPR4,ADIPOR2	up_VCAV_EC
GO:0048513	0.0174	1	2	906	animal organ development	BP	1	JUP,GATA5	down_VFEM_VSMC
GO:0043931	0.0174	0.999	2	9	ossification involved in bone maturation	BP	1	XYLT1,GREM1	up_AFEM_EC
GO:0070977	0.0174	0.999	2	9	bone maturation	BP	1	XYLT1,GREM1	up_AFEM_EC
GO:0060136	0.0174	1	1	4	embryonic process involved in female	BP	0.905	ACSL4	down_ACOR_EC
GO:0010837	0.0174	1	1	8	regulation of keratinocyte proliferation	BP	1	SRSF6	down_AFEM_EC
GO:0070986	0.0174	1	1	9	left/right axis specification	BP	1	CITED2	down_VPOR_EC
GO:0003222	0.0174	1	1	6	ventricular trabecula myocardium	BP	1	NKX2-5	up_VPOR_VSMC
GO:0042118	0.0174	1	1	4	endothelial cell activation	BP	1	HOXA9	up_VFEM_EC
GO:0048384	0.0174	1	1	4	retinoic acid receptor signaling pathway	BP	1	CYP26B1	up_VFEM_EC
GO:0048385	0.0174	1	1	4	regulation of retinoic acid receptor signaling	BP	1	CYP26B1	up_VFEM_EC
GO:0055062	0.0174	1	1	5	phosphate ion homeostasis	BP	1	HOXA3	up_VPUL_EC
GO:0072505	0.0174	1	1	5	divalent inorganic anion homeostasis	BP	1	HOXA3	up_VPUL_EC
GO:0072506	0.0174	1	1	5	trivalent inorganic anion homeostasis	BP	1	HOXA3	up_VPUL_EC
GO:0055002	0.0175	0.995	7	45	striated muscle cell development	BP	0.649	ACTN1,FHL2,WDR1,NEXN,SIX4,MYBPC2,HOMER1	down_VSAP_EC
GO:0097327	0.0175	0.996	6	25	response to antineoplastic agent	BP	0.564	PDGFB,MAPK13,TIMELESS,CTNNA1,BLM,ATRX	up_VCAV_EC
GO:0099518	0.0175	0.997	4	17	vesicle cytoskeletal trafficking	BP	0.649	MYO1C,KIF5B,HTT,AP3S1	down_VSAP_EC
GO:0001881	0.0175	0.997	4	11	receptor recycling	BP	1	PSEN1,RAB11B,LMTK2,BVES	up_ACOR_VSMC

GO:0009952	0.0175	0.998	3	66	anterior/posterior pattern specification	BP	1	BARX1,HOXA3,ACVR2A	up_VPOR_EC
GO:0030856	0.0175	0.999	2	41	regulation of epithelial cell differentiation	BP	0.905	ACVRL1,HOXA7	down_ACOR_EC
GO:1903707	0.0175	0.999	2	48	negative regulation of hemopoiesis	BP	1	HOXA9,HOXA7	up_VFEM_EC
GO:0050830	0.0175	1	1	12	defense response to Gram-positive bacterium	BP	1	TLR2	up_AFEM_VSMC
GO:0035115	0.0175	1	1	12	embryonic forelimb morphogenesis	BP	1	HOXA9	up_AFEM_VSMC
GO:0060147	0.0175	1	1	14	regulation of posttranscriptional gene	BP	1	ESR1	up_AORT_VSMC
GO:0060966	0.0175	1	1	14	regulation of gene silencing by RNA	BP	1	ESR1	up_AORT_VSMC
GO:0048752	0.0175	1	1	5	semicircular canal morphogenesis	BP	1	HOXA1	up_APUL_EC
GO:0010758	0.0175	1	1	8	regulation of macrophage chemotaxis	BP	1	C3AR1	up_VPOR_VSMC
GO:0090022	0.0175	1	1	9	regulation of neutrophil chemotaxis	BP	1	SLIT2	up_VPUL_VSMC
GO:0042118	0.0175	1	1	4	endothelial cell activation	BP	1	HOXA9	up_VSAP_VSMC
GO:0032869	0.017552243	0.99608	5	57	cellular response to insulin stimulus	BP	0.96436359	NR1H4,ENSCAFG00000004331,FOXC2,RARRES2,PRKCD	up_ACOR_EC
GO:1903362	0.0176	0.993	12	100	regulation of cellular protein catabolic process	BP	0.65	PRICKLE1,PLK3,SOCS5,TRIB3,MSN,UCHL5,APOE,GSK3B,DISC1,RAD23A,TMTC3,USP14	down_VSAP_EC
GO:0032640	0.0176	0.995	8	39	tumor necrosis factor production	BP	0.564	TNFAIP3,CD34,TLR4,ARG2,TLR3,HMGB1,RIPK1,ARHGEF2	up_VCAV_EC
GO:0071241	0.0176	0.995	8	42	cellular response to inorganic substance	BP	0.564	MEF2C,TJP1,ITPKB,TIMELESS,GUCY1B1,CREB1,BLM,ATRX	up_VCAV_EC
GO:0071322	0.0176	0.995	7	29	cellular response to carbohydrate stimulus	BP	1	RAB11B,ZFP36L1,KCNB1,RAF1,STX4,PPP3CB,RAB11FIP2	up_VSAP_EC
GO:0007405	0.0176	0.996	6	21	neuroblast proliferation	BP	1	FGFR2,ACSL6,SMARCD3,FZD3,FRS2,DISC1	up_ACOR_VSMC
GO:0002027	0.0176	0.996	5	20	regulation of heart rate	BP	0.564	PDE4D,EDN1,KCNJ2,GCH1,SPTBN4	up_VCAV_EC
GO:1902914	0.0176	0.997	4	11	regulation of protein polyubiquitination	BP	1	SKP2,SASH1,PTPN22,NOD2	up_ACOR_VSMC
GO:0045935	0.0176	0.998	3	491	positive regulation of nucleobase-containing compound metabolic	BP	1	HOXA9,WNT7A,ESR1	up_AORT_VSMC
GO:1990126	0.0176	1	1	8	retrograde transport, endosome to plasma	BP	1	ANKRD50	down_AFEM_EC
GO:0048596	0.0176	1	1	9	embryonic camera-type eye morphogenesis	BP	1	CITED2	down_VPOR_EC
GO:0006048	0.0176	1	1	2	UDP-N-acetylglucosamine biosynthetic process	BP	1	GNPNAT1	up_VPOR_EC
GO:0046349	0.0176	1	1	2	amino sugar biosynthetic process	BP	1	GNPNAT1	up_VPOR_EC
GO:0090022	0.0176	1	1	9	regulation of neutrophil chemotaxis	BP	1	C3AR1	up_VPOR_VSMC
GO:0048557	0.0176	1	1	7	embryonic digestive tract morphogenesis	BP	1	SHOX2	up_VFEM_VSMC
GO:0043412	0.017638181	0.9889	44	1225	macromolecule modification	BP	0.96436359	NR1H4,BRSK1,PLCL1,PKIB,SOX9,GALNT5,PHF20,NDRG2,PORCN,ENSCAFG00000004331,EDEM2,PI3,FZD5,DUSP26,NOD2,SMAD6,B3GNT7,KLHL25,ZMYND8,PAK3,GRK7,TNFRSF19,PIAS1,DCLK2,USP53,MAPK8IP2,MAVS,LRRC15,KLHDC8B,PIGS,LRP4,SH3RF3,LRRC4,RARRES2,AGBL5,RIOX2,RNF114,UBE2H,STK32C,NRP1,PRKCD,PYGO2,DBNDD2,CCNYL1	up_ACOR_EC
GO:0022604	0.0177	0.992	14	122	regulation of cell morphogenesis	BP	0.65	VEGFA,SIPA1L1,MYH9,FMNL3,SPARC,AKAP2,IFRD1,WDR1,MSN,TNIK,UST,ENSCAFG00000002015,NRP1,YWHAH	down_VSAP_EC
GO:0034329	0.0177	0.993	11	60	cell junction assembly	BP	0.566	TEK,PRKCH,OCLN,RAPGEF1,TJP1,SLK,APOD,RAPGEF2,CTNNA1,ENSCAFG00000002015,FSCN1	up_VCAV_EC
GO:0032501	0.0177	0.995	9	1782	multicellular organismal process	BP	1	HOXC10,EYA1,GATM,HOXA9,NETO1,TBX15,EMX2,HOXA11,SHOX2	up_VFEM_VSMC
GO:2000060	0.0177	0.995	7	45	positive regulation of ubiquitin-dependent protein catabolic process	BP	0.65	PRICKLE1,PLK3,SOCS5,TRIB3,GSK3B,DISC1,RAD23A	down_VSAP_EC
GO:0034381	0.0177	0.998	3	10	plasma lipoprotein particle clearance	BP	0.65	NR1H4,APOE,VLDLR	down_VSAP_EC
GO:0048532	0.0177	0.999	2	9	anatomical structure arrangement	BP	1	DLL1,PLXNA4	up_AFEM_EC
GO:0072611	0.0177	1	2	3	interleukin-13 secretion	BP	0.564	GATA3,ARG2	up_VCAV_EC
GO:2000665	0.0177	1	2	3	regulation of interleukin-13 secretion	BP	0.564	GATA3,ARG2	up_VCAV_EC

GO:1990266	0.0177	0.999	2	24	neutrophil migration	BP	1	C3AR1,ENSCAFG00000032731	up_VPOR_EC
GO:0048839	0.0177	0.999	2	50	inner ear development	BP	1	EYA1,SIX1	up_VFEM_EC
GO:0014904	0.0177	1	1	12	myotube cell development	BP	1	BEND2	up_AFEM_VSMC
GO:0048741	0.0177	1	1	12	skeletal muscle fiber development	BP	1	BEND2	up_AFEM_VSMC
GO:0099560	0.0177	1	1	5	synaptic membrane adhesion	BP	1	FLRT3	up_VPUL_EC
GO:0051246	0.017732955	0.98957	32	832	regulation of protein metabolic process	BP	0.96436359	PLCL1,PKIB,SOX9,PCSK1N,NDRG2,ENSCAFG00000004331,EDEM2,FZD5,IFI6,DUSP26,NOD2,SMAD6,KLHL25,PAK3,TNFRSF19,CST3,PIAS1,MAPK8IP2,MAVS,LRRC15,LRP4,SH3RF3,LRRC4,CLU,RARRES2,RNF114,NRP1,AZIN2,PRKCD,PYGO2,DBNDD2,CCNYL1	up_ACOR_EC
GO:0032228	0.017776087	0.99906	2	8	regulation of synaptic transmission, GABAergic	BP	0.96436359	CNTNAP4,PLCL1	up_ACOR_EC
GO:0045165	0.0178	0.993	11	65	cell fate commitment	BP	0.566	IL6,PPARG,MEF2C,DLL4,GATA3,SOX18,MTOR,BMP2,PROX1,ARHGEF2,CDON	up_VCAV_EC
GO:0007610	0.0178	0.998	3	146	behavior	BP	1	SLITRK5,NTRK2,HOXA1	up_APUL_EC
GO:0034329	0.0178	1	1	60	cell junction assembly	BP	1	JUP	down_VFEM_VSMC
GO:0060065	0.0178	1	1	7	uterus development	BP	1	HOXA9	up_AORT_EC
GO:0090175	0.0178	1	1	15	regulation of establishment of planar	BP	1	WNT7A	up_AORT_VSMC
GO:0061323	0.0178	1	1	7	cell proliferation involved in heart morphogenesis	BP	1	EYA1	up_VFEM_VSMC
GO:0003176	0.0178	1	1	9	aortic valve development	BP	1	GATA5	up_VPUL_VSMC
GO:0009888	0.0179	0.991	21	545	tissue development	BP	1	IRX3,LEP,LBH,HOXA7,DLL1,THRB,TMEM100,GREM1,OVOL1,POSTN,LUZP1,PTHLH,CNNM4,BVES,LGR6,AXIN2,PPARGC1A,ADAM17,GCNT4,FOSL2,TNC	up_AFEM_EC
GO:0006090	0.0179	0.996	5	26	pyruvate metabolic process	BP	0.654	PGK1,TPI1,PGAM1,HIF1A,ALDOC	down_VSAP_EC
GO:0060914	0.0179	0.997	4	11	heart formation	BP	1	GATA5,BMP4,SMARCD3,TBX5	up_ACOR_VSMC
GO:0070229	0.0179	0.997	4	13	negative regulation of lymphocyte apoptotic	BP	0.57	IL7R,ARG2,BCL10,HIF1A	up_VCAV_EC
GO:1903034	0.0179	0.999	2	40	regulation of response to wounding	BP	0.905	LYN,PDPN	down_ACOR_EC
GO:0030855	0.0179	0.999	2	183	epithelial cell differentiation	BP	1	WNT7A,ESR1	up_AORT_VSMC
GO:0048812	0.0179	0.999	2	151	neuron projection morphogenesis	BP	1	SLITRK5,LIMK1	up_VCAV_VSMC
GO:0014831	0.0179	1	1	3	gastro-intestinal system smooth muscle	BP	0.905	SULF1	down_ACOR_EC
GO:0071800	0.0179	1	1	8	podosome assembly	BP	1	FSCN1	down_AFEM_EC
GO:0003170	0.0179	1	1	25	heart valve development	BP	1	TBX5	up_APUL_VSMC
GO:0060575	0.0179	1	1	9	intestinal epithelial cell differentiation	BP	1	GATA5	up_VPUL_VSMC
GO:2000738	0.0179	1	1	9	positive regulation of stem cell differentiation	BP	1	TBX5	up_VPUL_VSMC
GO:0009888	0.017941488	0.99035	23	545	tissue development	BP	0.96436359	CDH3,NR1H4,TMOD1,GATA4,BRSK1,SOX9,PTH1R,AQP1,FZD5,SMAD6,ACTN2,DLX3,TNFRSF19,ADAMTSL2,FOXC2,TBX5,LRP4,CLSTN1,C23H3orf58,NRP1,ARHGEF26,PYGO2,EFEMP1	up_ACOR_EC
GO:0030239	0.017997635	0.99785	3	21	myofibril assembly	BP	0.96436359	MYOZ2,TMOD1,ACTN2	up_ACOR_EC
GO:0044249	0.018	0.993	14	1851	cellular biosynthetic process	BP	1	HOXA11,TBX15,HOXA10,HOXA9,PITX1,EMX2,GATM,SALL1,GALNT17,IRX5,PRR16,TLR2,CAV1,SHOX2	up_VSAP_VSMC
GO:0050867	0.018	0.992	13	83	positive regulation of cell activation	BP	0.571	IL7R,SELP,MEF2C,TLR4,GATA3,PELI1,NECTIN2,ITPKB,NCK1,CD200,BCL10,HMGB1,ATP11C	up_VCAV_EC
GO:0000212	0.018	0.998	3	7	meiotic spindle organization	BP	0.695	FBXO5,ESPL1,ASPM	down_ACOR_VSMC
GO:0050680	0.018	0.999	2	44	negative regulation of epithelial cell proliferation	BP	0.905	ACVRL1,SULF1	down_ACOR_EC
GO:2001204	0.018	0.999	2	5	regulation of osteoclast development	BP	1	NOTCH2,FBN1	down_VCAV_EC
GO:0002176	0.018	0.999	2	3	male germ cell proliferation	BP	1	PRDX4,SPINK2	up_ACOR_VSMC

GO:0036093	0.018	0.999	2	3	germ cell proliferation	BP	1	PRDX4,SPINK2	up_ACOR_VSMC
GO:0046850	0.018	0.999	2	9	regulation of bone remodeling	BP	1	LEP,GREM1	up_AFEM_EC
GO:0060839	0.018	1	1	4	endothelial cell fate commitment	BP	0.905	PDPN	down_ACOR_EC
GO:0072148	0.018	1	1	4	epithelial cell fate commitment	BP	0.905	PDPN	down_ACOR_EC
GO:0006833	0.018	1	1	5	water transport	BP	0.905	PDPN	down_ACOR_EC
GO:0045766	0.018	1	1	63	positive regulation of angiogenesis	BP	1	JUP	down_VFEM_VSMC
GO:0055003	0.018	1	1	7	cardiac myofibril assembly	BP	1	NKX2-5	up_VPOR_VSMC
GO:0098581	0.018	1	1	5	detection of external biotic stimulus	BP	1	TLR2	up_VSAP_VSMC
GO:0044087	0.018001466	0.99149	16	339	regulation of cellular component biogenesis	BP	0.96436359	TMOD1,ARHGAP6,SYNPO2,FZD5,SMAD6,ARHGAP28,PAK3,PTGER4,PREB,CLSTN1,CLU,CHMP4B,VPS16,NRP1,PRKCD,TUBB4A	up_ACOR_EC
GO:0043039	0.0181	0.995	6	33	tRNA aminoacylation	BP	0.66	CARS,SARS,AARS,YARS,TARS,NARS	down_VSAP_EC
GO:0051254	0.0181	0.997	4	431	positive regulation of RNA metabolic process	BP	1	EYA1,HOXA9,HOXA11,SHOX2	up_VFEM_VSMC
GO:0016055	0.0181	0.998	3	134	Wnt signaling pathway	BP	1	SALL1,TLR2,CAV1	up_VSAP_VSMC
GO:0198738	0.0181	0.998	3	134	cell-cell signaling by wnt	BP	1	SALL1,TLR2,CAV1	up_VSAP_VSMC
GO:0031214	0.0181	0.999	2	45	biomineral tissue development	BP	0.905	HOXA3,ASPN	down_ACOR_EC
GO:0045683	0.0181	0.999	2	9	negative regulation of epidermis development	BP	1	HOXA7,DLL1	up_AFEM_EC
GO:0002062	0.0181	0.999	2	48	chondrocyte differentiation	BP	1	HOXA11,SHOX2	up_VSAP_VSMC
GO:0043500	0.0181	1	1	21	muscle adaptation	BP	1	GATA5	down_VSAP_VSMC
GO:1903426	0.0181	1	1	17	regulation of reactive oxygen species	BP	1	ARG2	up_AORT_VSMC
GO:0010002	0.0181	1	1	9	cardioblast differentiation	BP	1	TBX5	up_VPUL_VSMC
GO:0009719	0.0182	0.989	35	395	response to endogenous stimulus	BP	0.66	ARHGEF2,SULF1,NR1H4,HSPA5,SOCS5,ENPP1,SNX25,PTPN11,TRIB3,ILK,FHL2,PRCP,SMARCA4,SESN2,TGFBR1,MYO1C,MSN,ZPR1,PTPN2,YES1,NEO1,ADAM17,AGRN,GNAQ,CTNNB1,ENSCAFG00000002015,APOE,UBA5,LYN,STRAP,REST,NR4A1,ENSCAFG00000018360,YWHAH,KDM3A	down_VSAP_EC
GO:0031589	0.0182	0.992	16	93	cell-substrate adhesion	BP	1	SMAD6,ACTN2,FERMT1,NRP1,NPNT,PRKCE,ECM2,JUP,DUSP22,NID1,FAM107A,JAM3,DOCK5,BVES,RREB1,CRKL	up_ACOR_VSMC
GO:0021700	0.0182	0.996	5	65	developmental maturation	BP	1	IRX5,XYLT1,LGI4,GREM1,RECK	up_AFEM_EC
GO:0048519	0.0182	0.997	5	1554	negative regulation of biological process	BP	1	HOXC10,HOXA9,WNT7A,ARG2,ESR1	up_AORT_VSMC
GO:0046825	0.0182	0.997	4	16	regulation of protein export from nucleus	BP	0.66	PTPN14,PTPN11,IWS1,GSK3B	down_VSAP_EC
GO:0002021	0.0182	0.999	2	9	response to dietary excess	BP	1	LEP,PPARGC1A	up_AFEM_EC
GO:0042246	0.0182	1	1	17	tissue regeneration	BP	1	WNT7A	up_AORT_VSMC
GO:2000107	0.0182	1	1	16	negative regulation of leukocyte apoptotic	BP	1	ARG2	up_AORT_VSMC
GO:0008356	0.0182	1	1	3	asymmetric cell division	BP	1	ARHGEF2	up_VPOR_EC
GO:0042573	0.0182	1	1	4	retinoic acid metabolic process	BP	1	CYP26B1	up_VFEM_EC
GO:0072189	0.0182	1	1	4	ureter development	BP	1	SIX1	up_VFEM_EC
GO:0036005	0.0182	1	1	4	response to macrophage colony-stimulating factor	BP	1	TLR2	up_VSAP_VSMC
GO:0036006	0.0182	1	1	4	cellular response to macrophage colony-	BP	1	TLR2	up_VSAP_VSMC
GO:0042532	0.0182	1	1	4	negative regulation of tyrosine phosphorylation	BP	1	CAV1	up_VSAP_VSMC
GO:0022603	0.0183	0.988	40	301	regulation of anatomical structure morphogenesis	BP	1	HOXA11,FZD1,THRB,WNT11,HNF4A,WASF3,ENPP2,LIMS2,DDHD1,SLIT2,FGF7,ABL1,BTG1,HDAC7,EMP2,DLL1,EPN2,OSR1,CDC42EP5,PLXNA4,CDC42EP2,CCBE1,TBXA2R,SULF2,SHOX2,SASH1,LRP4,DPYSL2,NTN4,LIMD1,FBLN1,NFIB,PQBP1,TTL,ACVRL1,FLOT1,HMOX1,MAP2K2,TGFBR2,CITED2	up_VSAP_EC

GO:0034220	0.0183	0.99	27	188	ion transmembrane transport	BP	1	ACTN2,PLCG2,SLC26A7,KCNE3,HTR2B,TCAF1,GJA1,PTPN3,CLCN3,MICU2,ITPR1,DAPK1,CACNB3,AKAP6,LRRC8C,KCNJ4,PRKCE,PSEN1,CHD7,CLN8,ATP6V0E1,KCNC4,FXYD1,SLC25A12,NDUFA4L2,PARK7,SLC8A1	up_ACOR_VSMC
GO:0031323	0.0183	0.993	15	1788	regulation of cellular metabolic process	BP	0.905	ACVRL1,YEATS2,LYN,IGF1R,YBX3,HIVEP2,HOXA7,PRKAR2A,SULF1,ELK3,HOXA1,ACSL3,MYO1D,ASPN,NEDD4	down_ACOR_EC
GO:0009062	0.0183	0.996	5	31	fatty acid catabolic process	BP	1	AKT2,ACAA1,PEX5,LONP2,ACAT1	down_VCAV_EC
GO:0007187	0.0183	0.997	4	43	G protein-coupled receptor signaling pathway, coupled to cyclic nucleotide second	BP	1	GPR1,CHRM2,HTR1D,PTHLH	up_AFEM_EC
GO:0098901	0.0183	0.998	3	7	regulation of cardiac muscle cell action	BP	1	FGF13,KCNE3,JUP	up_ACOR_VSMC
GO:0032388	0.0183	1	1	62	positive regulation of intracellular transport	BP	1	JUP	down_VFEM_VSMC
GO:0021522	0.0183	1	1	8	spinal cord motor neuron differentiation	BP	1	HOXC10	up_AORT_EC
GO:2000142	0.0183	1	1	17	regulation of DNA-templated transcription,	BP	1	ESR1	up_AORT_VSMC
GO:0061218	0.0183	1	1	4	negative regulation of mesonephros	BP	1	SIX1	up_VFEM_EC
GO:1902204	0.018362751	1	1	1	positive regulation of hepatocyte growth factor receptor signaling	BP	0.96436359	ESM1	up_ACOR_EC
GO:0045058	0.0184	0.997	4	12	T cell selection	BP	0.582	IL6,GATA3,MTOR,ITPKB	up_VCAV_EC
GO:0008284	0.0184	0.997	4	238	positive regulation of cell proliferation	BP	1	EYA1,SIX1,MAB21L1,TIAM1	up_VFEM_EC
GO:0009584	0.0184	0.999	2	4	detection of visible light	BP	0.666	GNAQ,RDH11	down_VSAP_EC
GO:0002931	0.0184	0.999	2	9	response to ischemia	BP	1	CAMK2A,BVES	up_AFEM_EC
GO:0010766	0.0184	1	1	4	negative regulation of sodium ion transport	BP	0.905	NEDD4	down_ACOR_EC
GO:0007026	0.0184	1	1	8	negative regulation of microtubule	BP	1	TAOK1	up_AORT_EC
GO:0050772	0.0184	1	1	13	positive regulation of axonogenesis	BP	1	LIMK1	up_VCAV_VSMC
GO:0048755	0.0184	1	1	4	branching morphogenesis of a nerve	BP	1	SHOX2	up_VSAP_VSMC
GO:0050804	0.0185	0.992	15	87	modulation of chemical synaptic transmission	BP	1	PLCL1,FBXL20,HRH1,TMEM108,EGLN1,DMPK,PRKCE,RNF19A,APP,PSEN1,EDN1,KCNC4,LRRC4,FAM107A,PREPL	up_ACOR_VSMC
GO:0099177	0.0185	0.992	15	87	regulation of trans-synaptic signaling	BP	1	PLCL1,FBXL20,HRH1,TMEM108,EGLN1,DMPK,PRKCE,RNF19A,APP,PSEN1,EDN1,KCNC4,LRRC4,FAM107A,PREPL	up_ACOR_VSMC
GO:0019219	0.0185	0.994	10	1151	regulation of nucleobase-containing compound metabolic process	BP	1	HOXA11,HOXA10,HOXA9,PITX1,EMX2,SALL1,IRX5,TLR2,CAV1,SHOX2	up_VSAP_VSMC
GO:0006081	0.0185	0.996	5	27	cellular aldehyde metabolic process	BP	0.667	TPI1,PGAM1,REST,RDH11,KDM3A	down_VSAP_EC
GO:0072710	0.0185	0.998	3	6	response to hydroxyurea	BP	0.583	TIMELESS,BLM,ATRX	up_VCAV_EC
GO:0097329	0.0185	0.998	3	6	response to antimetabolite	BP	0.583	TIMELESS,BLM,ATRX	up_VCAV_EC
GO:0030099	0.0185	0.998	3	133	myeloid cell differentiation	BP	1	HOXA9,HOXB7,HOXA7	up_VFEM_EC
GO:0007519	0.0185	0.999	2	49	skeletal muscle tissue development	BP	1	SIX1,CYP26B1	up_VFEM_EC
GO:1902745	0.0185	1	1	9	positive regulation of lamellipodium	BP	1	FSCN1	down_AFEM_EC
GO:0051238	0.0185	1	1	7	sequestering of metal ion	BP	1	ENSCAFG00000005494	up_AORT_EC
GO:2001224	0.0185	1	1	5	positive regulation of neuron migration	BP	1	ARHGEF2	up_VPUL_EC
GO:0051354	0.0185	1	1	4	negative regulation of oxidoreductase activity	BP	1	CAV1	up_VSAP_VSMC
GO:0098911	0.0185	1	1	4	regulation of ventricular cardiac muscle cell action	BP	1	CAV1	up_VSAP_VSMC
GO:2000463	0.0185	1	1	5	positive regulation of excitatory postsynaptic	BP	1	NETO1	up_VSAP_VSMC
GO:0031326	0.0186	0.995	7	1176	regulation of cellular biosynthetic process	BP	1	EYA1,HOXA10,HOXA9,FBP1,EMX2,HOXA11,SHOX2	up_VFEM_VSMC
GO:0042632	0.0186	0.995	6	27	cholesterol homeostasis	BP	0.715	SREBF2,RALY,INSIG1,CAV1,LIPC,NUS1	down_ACOR_VSMC
GO:0006661	0.0186	0.995	6	23	phosphatidylinositol biosynthetic process	BP	1	PIP5K1B,PLCG2,HTR2B,PIGN,PIGS,PIGG	up_ACOR_VSMC

GO:0006337	0.0186	0.998	3	7	nucleosome disassembly	BP	0.715	SMARCC1,SMARCB1,GRWD1	down_ACOR_VSMC
GO:0032986	0.0186	0.998	3	7	protein-DNA complex disassembly	BP	0.715	SMARCC1,SMARCB1,GRWD1	down_ACOR_VSMC
GO:0032754	0.0186	0.999	2	3	positive regulation of interleukin-5 production	BP	0.584	PDE4D,GATA3	up_VCAV_EC
GO:0006816	0.0186	0.999	2	84	calcium ion transport	BP	1	HOXA3,TRPC4	up_VPOR_VSMC
GO:0021575	0.0186	1	1	15	hindbrain morphogenesis	BP	1	WNT7A	up_AORT_VSMC
GO:0021587	0.0186	1	1	15	cerebellum morphogenesis	BP	1	WNT7A	up_AORT_VSMC
GO:0035116	0.0186	1	1	15	embryonic hindlimb morphogenesis	BP	1	WNT7A	up_AORT_VSMC
GO:0120163	0.0186	1	1	17	negative regulation of cold-induced	BP	1	HOXC10	up_AORT_VSMC
GO:0021675	0.0186	1	1	29	nerve development	BP	1	HOXA3	up_APUL_VSMC
GO:0035640	0.0186	1	1	7	exploration behavior	BP	1	CHL1	up_VPOR_VSMC
GO:0006805	0.0186	1	1	8	xenobiotic metabolic process	BP	1	CYP26B1	up_VPOR_VSMC
GO:0008045	0.0186	1	1	9	motor neuron axon guidance	BP	1	SLIT2	up_VPUL_VSMC
GO:0038166	0.0186	1	1	4	angiotensin-activated signaling pathway	BP	1	CAV1	up_VSAP_VSMC
GO:0003283	0.018642737	0.99899	2	8	atrial septum development	BP	0.96436359	GATA4,TBX5	up_ACOR_EC
GO:0060413	0.018642737	0.99899	2	8	atrial septum morphogenesis	BP	0.96436359	GATA4,TBX5	up_ACOR_EC
GO:0043066	0.0187	0.99	24	253	negative regulation of apoptotic process	BP	0.673	ARHGEF2,VEGFA,ASNS,NR1H4,PLK3,HSPA5,SERPINE1,HYOU1,AARS,FHL2,PTGS2,HIF1A,ZPR1,MTDH,CTNNB1,CRYAB,BNIP3,GSK3B,HTT,GLO1,PAK2,SIX4,NRP1,ARF4	down_VSAP_EC
GO:0009306	0.0187	0.99	20	127	protein secretion	BP	1	HNF4A,CD274,C5AR2,SLC8B1,ABL1,IL17RC,ANXA4,F2RL1,LMF1,RAB11B,STEAP3,KCNB1,RAF1,PNP,MAPKBP1,STX4,PPP3CB,MAVS,NAGPA,RAB11FIP2	up_VSAP_EC
GO:0051049	0.0187	0.991	19	479	regulation of transport	BP	1	LEP,KCNC4,CRY2,RAB20,GAS1,DLL1,CRYAB,KCND1,GPAM,CHRM2,GREM1,CAMK2A,FXYD1,REEP2,BVES,RAB7A,CCL5,DMPK,CLIC4	up_AFEM_EC
GO:0046890	0.0187	0.994	9	51	regulation of lipid biosynthetic process	BP	0.584	ADGRF5,PDGFB,MTOR,SAMD8,BMP2,PROX1,ENSCAFG00000010596,PTGS2,CREB1	up_VCAV_EC
GO:1901342	0.0187	0.994	7	114	regulation of vasculature development	BP	1	LEP,ANGPT4,DLL1,F3,TMEM100,GREM1,TBXA2R	up_AFEM_EC
GO:1901880	0.0187	0.996	5	19	negative regulation of protein depolymerization	BP	0.584	TMOD1,SWAP70,TAOK1,TMOD3,ARHGEF2	up_VCAV_EC
GO:0072358	0.0187	0.997	4	268	cardiovascular system development	BP	1	HOXA3,SLITRK5,NTRK2,HOXA1	up_APUL_EC
GO:0015012	0.0187	0.998	3	9	heparan sulfate proteoglycan biosynthetic	BP	0.672	ENSCAFG00000032470,CTNNB1,EXT1	down_VSAP_EC
GO:0061035	0.0187	0.998	3	24	regulation of cartilage development	BP	1	GREM1,PTHLH,AXIN2	up_AFEM_EC
GO:0000290	0.0187	0.998	3	8	deadenylation-dependent decapping of nuclear-transcribed mRNA	BP	0.584	PAN3,CNOT7,DCP2	up_VCAV_EC
GO:0008284	0.0187	0.998	3	238	positive regulation of cell proliferation	BP	1	HOXA3,NKX2-5,BAMBI	up_VPOR_VSMC
GO:0045596	0.0187	0.999	2	186	negative regulation of cell differentiation	BP	1	HOXA9,WNT7A	up_AORT_VSMC
GO:0007623	0.0187	0.999	2	55	circadian rhythm	BP	1	HTR7,NTRK2	up_APUL_EC
GO:0050803	0.0187	0.999	2	55	regulation of synapse structure or activity	BP	1	SLITRK5,NTRK2	up_APUL_EC
GO:0050807	0.0187	0.999	2	55	regulation of synapse organization	BP	1	SLITRK5,NTRK2	up_APUL_EC
GO:1903589	0.0187	0.999	2	3	positive regulation of blood vessel endothelial cell proliferation involved in sprouting angiogenesis	BP	0.584	APLNR,JCAD	up_VCAV_EC
GO:0035019	0.0187	1	1	16	somatic stem cell population maintenance	BP	1	WNT7A	up_AORT_VSMC
GO:0050701	0.0187	1	1	16	interleukin-1 secretion	BP	1	ARG2	up_AORT_VSMC
GO:0001759	0.0187	1	1	13	organ induction	BP	1	GATA5	up_VCAV_VSMC
GO:0019748	0.0187	1	1	13	secondary metabolic process	BP	1	CYP1A1	up_VCAV_VSMC
GO:0048752	0.0187	1	1	5	semicircular canal morphogenesis	BP	1	EYA1	up_VFEM_EC

GO:0033483	0.0187	1	1	4	gas homeostasis	BP	1	CAV1	up_VSAP_VSMC
GO:0042391	0.018762353	0.99498	6	81	regulation of membrane potential	BP	0.96436359	KCNB1,IFI6,INSYN1,ACTN2,USP53,MAPK8IP2	up_ACOR_EC
GO:0016050	0.0188	0.991	17	119	vesicle organization	BP	0.584	ATP8A1,RAB11A,RAB27A,NECTIN2,HOOK3,SNAP23,CAV2,ABCA1,SNX2,CREB1,STX11,TBC1D1,DNAJC13,ALS2,RABGAP1,FBXO5,USP6NL	up_VCAV_EC
GO:0006302	0.0188	0.991	15	97	double-strand break repair	BP	0.721	RPA1,POLA1,TRIP13,PARPBP,GINS4,RAD54L,RAD51,TIMELESS,MND1,TONSL,BRCA1,RBBP8,SMC6,H2AFX,GEN1	down_ACOR_VSMC
GO:1903035	0.0188	0.997	4	14	negative regulation of response to wounding	BP	0.584	PDGFB,CD34,KLKB1,TFPI	up_VCAV_EC
GO:1990845	0.0188	0.999	2	51	adaptive thermogenesis	BP	1	HOXC10,MAP2K6	up_VFEM_EC
GO:0071216	0.0188	0.999	2	61	cellular response to biotic stimulus	BP	1	NR1H4,MRC1	up_VPUL_EC
GO:0010572	0.0188	1	1	4	positive regulation of platelet activation	BP	0.905	PDPN	down_ACOR_EC
GO:0034505	0.0188	1	1	4	tooth mineralization	BP	0.905	ASPN	down_ACOR_EC
GO:0035909	0.0188	1	1	14	aorta morphogenesis	BP	1	EYA1	up_AFEM_VSMC
GO:0051128	0.0189	0.987	62	790	regulation of cellular component organization	BP	0.673	ARHGEF2,VEGFA,SIPA1L1,MYH9,FMNL3,SERPINE1,SPARC,ENPP1,AKAP2,PTPN11,FSCN1,DPYSL3,IFRD1,FBLIM1,SMARCA4,ANKRA2,SESN2,WDR1,MYO1C,MSN,LMAN1,HIF1A,DYNLL1,TUBB,IWS1,TNIK,NAA10,ESM1,ADAM17,UST,ACTR3,CTNNB1,ANLN,CRYAB,ENSCAFG00000002015,APOE,IGFBP7,KLF5,LYN,BNIP3,TWF1,FAM162A,PLEKHG2,GSK3B,VLDLR,NEXN,CDC37,GBP5,SH3GLB1,SDC4,HTT,DISC1,LRRTM2,CAPG,INHBA,SIX4,CDKN1A,NRP1,ARF4,YWHAH,KDM3A,MID1	down_VSAP_EC
GO:0031328	0.0189	0.987	57	512	positive regulation of cellular biosynthetic process	BP	0.584	HCLS1,ADGRF5,PPARG,TNIP1,PDGFB,TMEM173,MEF2C,ATF3,CHD7,TLR4,NLRC5,GATA3,EDN1,TCF4,FOXN2,ENSCAFG00000017103,IRF8,PPP3CA,YES1,MTOR,BMP2,HHEX,CREM,CNOT6,PROX1,ARID4B,HMBOX1,PRKD2,THOC1,SMARCA4,TLR3,NCK1,LDB2,JAG1,NSD1,PTGS2,BCL10,CNOT7,HMGB1,AHR,NOS2,BLM,RIPK1,ITGA6,GPBP1,MTA2,NR1D2,KDM5A,HIF1A,KDM3A,ATRX,NR4A1,ZEB1,TET2,ARHGEF2,CDON,ZNF609	up_VCAV_EC
GO:0061041	0.0189	0.995	7	34	regulation of wound healing	BP	0.584	SELP,PDGFB,CD34,KLKB1,TLR4,TFPI,MTOR	up_VCAV_EC
GO:0061097	0.0189	0.996	5	27	regulation of protein tyrosine kinase activity	BP	0.673	SOCS5,ERRFI1,PTPN2,ADAM17,CBLC	down_VSAP_EC
GO:0010469	0.0189	0.996	5	26	regulation of signaling receptor activity	BP	0.673	SERPINE1,SOCS5,ERRFI1,ADAM17,CBLC	down_VSAP_EC
GO:0048856	0.0189	0.997	5	1533	anatomical structure development	BP	1	HOXC10,HOXA9,WNT7A,ARG2,ESR1	up_AORT_VSMC
GO:0006915	0.0189	0.998	3	550	apoptotic process	BP	1	WNT7A,ARG2,ESR1	up_AORT_VSMC
GO:1990138	0.0189	0.999	2	35	neuron projection extension	BP	0.905	SPG11,NRP2	down_ACOR_EC
GO:2001242	0.0189	0.999	2	62	regulation of intrinsic apoptotic signaling	BP	1	PTGER2,ARHGEF2	up_VPUL_EC
GO:0001886	0.0189	1	1	5	endothelial cell morphogenesis	BP	1	ARHGEF26	up_APUL_EC
GO:2000209	0.0189	1	1	5	regulation of anoikis	BP	1	NTRK2	up_APUL_EC
GO:2000811	0.0189	1	1	5	negative regulation of anoikis	BP	1	NTRK2	up_APUL_EC
GO:0016331	0.019	0.993	9	66	morphogenesis of embryonic epithelium	BP	0.673	PRICKLE1,SULF1,PRKACB,ENSCAFG00000032470,TMED2,HIF1A,CTNNB1,SDC4,SIX4	down_VSAP_EC
GO:0034620	0.019	0.995	6	38	cellular response to unfolded protein	BP	0.673	HSPA5,ERO1A,PTPN2,DDIT3,ATF3,CREB3L2	down_VSAP_EC
GO:0060306	0.019	0.998	3	7	regulation of membrane repolarization	BP	1	KCNE3,GJA1,AKAP6	up_ACOR_VSMC
GO:0086009	0.019	0.998	3	7	membrane repolarization	BP	1	KCNE3,GJA1,AKAP6	up_ACOR_VSMC
GO:0001516	0.019	0.998	3	7	prostaglandin biosynthetic process	BP	0.585	EDN1,PTGS1,PTGS2	up_VCAV_EC
GO:0046457	0.019	0.998	3	7	prostanoid biosynthetic process	BP	0.585	EDN1,PTGS1,PTGS2	up_VCAV_EC
GO:0007423	0.019	0.999	2	155	sensory organ development	BP	1	EYA1,IRX5	up_AFEM_VSMC
GO:0050817	0.019	0.999	2	50	coagulation	BP	1	CAV1,F5	up_VSAP_VSMC

GO:1902991	0.019	1	1	5	regulation of amyloid precursor protein	BP	0.905	LYN	down_ACOR_EC
GO:0042473	0.019	1	1	5	outer ear morphogenesis	BP	1	HOXA1	up_APUL_EC
GO:0048523	0.019047841	0.98772	49	1399	negative regulation of cellular process	BP	0.96436359	NR1H4,TMOD1,BRSK1,TRPS1,PKIB,SOX9,ARHGAP6,PTH1R,PCSK1N,NDRG2,AQP1,KCNB1,FZD5,CCDC3,IFI6,SEMA6D,DUSP26,DIXDC1,PTGIR,RGMA,SMAD6,ACTN2,ZMYND8,ARHGAP28,MMP28,BCL2,PTGER4,ARHGDIB,CST3,PIAS1,ADAMTSL2,MAPK8IP2,FOXC2,FIGNL1,LRRC15,TBX5,LRP4,CLSTN1,LRRC4,CLU,CHMP4B,RIOX2,NRP1,PRKCD,PPP1R26,PM20D1,DBNDD2,EFEMP1,TUBB4A	up_ACOR_EC
GO:0009966	0.019093011	0.98862	33	868	regulation of signal transduction	BP	0.96436359	SH3TC2,CDH3,NR1H4,SOX9,RGS1,NDRG2,FZD5,CCDC3,IFI6,DUSP26,DIXDC1,PTGIR,NOD2,PDE4A,SMAD6,ESM1,PAK3,TNFRSF19,ARHGDIB,ADAMTSL2,MAPK8IP2,MAVS,FIGNL1,LRRC15,LRP4,CLSTN1,SH3RF3,LRRC4,CLU,C23H3orf58,NRP1,ARHGEF26,PRKCD	up_ACOR_EC
GO:0065008	0.0191	0.985	121	1056	regulation of biological quality	BP	1	FGF13,RHOT1,ADRA1B,ACTN2,PLCG2,LEP,PLCL1,SLC26A7,KCNE3,CDC7,HTR2B,RASSF2,MBP,FBXL20,PRDX4,PRKAA2,BMP4,LCAT,TPC3,LNPK,TM7SF3,NPPB,CNNM2,ABCA1,GJA1,SCARB2,ATRX,HRH1,WASF3,PPARGC1A,CITED2,MAOA,ISG15,ZNF365,SLC2A5,STX11,PTPN3,TMEM108,CLCN3,FAM210B,P4HTM,MICU2,TMX3,ADCY6,USP53,ITPR1,PLAC8B,F2RL1,HRH2,STN1,ENPP4,ERO1B,BNIP3,EGLN1,SLC12A7,AKAP6,JMY,CLSTN1,LRRC8C,NRP1,ULK2,DMPK,OS9,ATP7A,ORMDL3,EVL,SLITRK5,PTGDR,SESN3,MOXD1,PRKCE,DKKL1,SCG5,POLR2M,RNLS,DAG1,APP,PSEN1,PCSK6,CHD7,LAMP2,JUP,DMXL1,CCL5,CLN8,RAB11B,SSH3,EDN1,KCNC4,NOD2,LRRC4,FAM107A,INSYN1,JAM3,B2M,DISC1,AP2B1,PLIN2,FXYD1,NCALD,HERPUD1,TXNDC5,GAA,SUN1,GNAQ,INPP5K,PREPL,PFKL,WFS1,WDR48,WASL,ATP2C1,NAIF1,DOCK5,ERAP1,PARK7,SLC8A1,STC2,IL18R1,HMBOX1,BVES	up_ACOR_VSMC
GO:0065007	0.0191	0.993	21	3499	biological regulation	BP	1	HOXC10,EYA1,HOXA9,TOX,MAP2K6,AVPR1A,HOXA10,TRIM9,HOXB7,HOXA7,ENSCAFG00000018194,SIX1,YBX3,MAB21L1,TIAM1,RADIL,RASD2,CYP26B1,CDC42EP5,MTSS1,ADCY3	up_VFEM_EC
GO:0008202	0.0191	0.992	14	80	steroid metabolic process	BP	1	LIPC,INSIG2,ACAA1,SULT1A1,HSD17B14,HSD3B7,HSD17B8,LMF1,LCAT,DHRS9,MVK,LIPE,CYB5R3,SOAT1	up_VSAP_EC
GO:0035148	0.0191	0.993	9	66	tube formation	BP	0.673	PRICKLE1,VEGFA,PRKACB,ENSCAFG00000032470,TMED2,HIF1A,CTNNB1,SDC4,SIX4	down_VSAP_EC
GO:0010821	0.0191	0.994	7	49	regulation of mitochondrion	BP	0.673	HIF1A,BNIP3,FAM162A,GSK3B,CDC37,SH3GLB1,HTT	down_VSAP_EC
GO:0030514	0.0191	0.998	3	12	negative regulation of BMP signaling pathway	BP	1	FBN1,CHRDL1,GREM1	down_VCAV_EC
GO:0140115	0.0191	0.999	2	3	export across plasma membrane	BP	1	KCNE3,FXYD1	up_ACOR_VSMC
GO:0002009	0.0191	0.999	2	177	morphogenesis of an epithelium	BP	1	WNT7A,ESR1	up_AORT_VSMC
GO:0008016	0.0191	0.999	2	48	regulation of heart contraction	BP	1	IRX5,CAV1	up_VSAP_VSMC
GO:0002888	0.0191	1	1	5	positive regulation of myeloid leukocyte	BP	0.905	STX4	down_ACOR_EC
GO:0021615	0.0191	1	1	3	glossopharyngeal nerve morphogenesis	BP	0.905	HOXA3	down_ACOR_EC
GO:0030889	0.0191	1	1	4	negative regulation of B cell proliferation	BP	0.905	LYN	down_ACOR_EC
GO:2000116	0.0191	1	1	67	regulation of cysteine-type endopeptidase	BP	1	CST3	down_AORT_EC
GO:0032722	0.0191	1	1	14	positive regulation of chemokine production	BP	1	TLR2	up_AFEM_VSMC
GO:0048643	0.0191	1	1	7	positive regulation of skeletal muscle tissue	BP	1	CYP26B1	up_VPOR_VSMC

GO:0048523	0.0192	0.986	102	1399	negative regulation of cellular process	BP	0.673	ARHGEF2,PRICKLE1,VEGFA,SULF1,ASNS,MYH9,NR1H4,PLK3,HSPA5,SERPINE1,ACTN1,SOCS5,ETV6,SPARC,ERO1A,PRKACB,PTPN14,ENPP1,ERRFI1,SNX25,CBX5,HYOU1,PTPN11,AARS,DPYSL3,HOOK3,TRIB3,IFRD1,ILK,FHL2,SMARCA4,SESN2,PTGS2,HIF1A,DYNLL1,DNAJC10,NAA10,NXN,RGS2,ZPR1,PTPN2,UCHL5,MORF4L2,SPOPL,PEG3,MTDH,DDX39B,ADAM17,EIF4EBP1,SKAP2,CNOT7,MAGED1,CTNNB1,CRYAB,STARD13,ENSCAFG00000002015,APOE,WWC3,KLF5,MMP28,LYN,BNIP3,STRAP,DDIT3,TWF1,PDS5B,CBLC,ATF3,GSK3B,VLDLR,TAX1BP3,SDC4,HTT,REST,ENSCAFG00000017326,ZMYND8,EIF2S1,PRMT1,ARFGEF3,CALM1,RAB11FIP1,GLO1,CDYL,PAK2,LRRTM2,A2M,CAPG,INHBA,NR4A1,HIF1AN,BTK,SIX4,CDKN1A,NRP1,SPINK2,RHBDF2,USP14,ARF4,YWHAH,TNFAIP1,KDM3A,MID1	down_VSAP_EC
GO:0001503	0.0192	0.992	11	109	ossification	BP	1	NAB2,THBS3,BMPR1A,CLSTN1,ASPN,GLI3,TWIST2,DDR2,OMD,GREM1,ZBTB16	down_VCAV_EC
GO:0048593	0.0192	0.994	8	41	camera-type eye morphogenesis	BP	0.587	EPHB1,PROX1,TSPAN12,JAG1,HIF1A,ALDH1A3,ZEB1,CDON	up_VCAV_EC
GO:0071706	0.0192	0.994	8	40	tumor necrosis factor superfamily cytokine	BP	0.587	TNFAIP3,CD34,TLR4,ARG2,TLR3,HMGB1,RIPK1,ARHGEF2	up_VCAV_EC
GO:0034332	0.0192	0.996	5	32	adherens junction organization	BP	1	NUMBL,RHOD,RASSF8,THY1,GREM1	down_VCAV_EC
GO:0001754	0.0192	0.998	3	12	eye photoreceptor cell differentiation	BP	1	NAGLU,THY1,THRB	down_VCAV_EC
GO:2000251	0.0192	0.998	3	7	positive regulation of actin cytoskeleton	BP	0.587	TEK,BAIAP2L1,FES	up_VCAV_EC
GO:0038089	0.0192	0.999	2	4	positive regulation of cell migration by vascular endothelial growth factor	BP	0.673	VEGFA,MYO1C	down_VSAP_EC
GO:0010715	0.0192	1	1	4	regulation of extracellular matrix disassembly	BP	0.905	PDPN	down_ACOR_EC
GO:0001732	0.0192	1	1	9	formation of cytoplasmic translation initiation	BP	1	EIF3M	down_AFEM_EC
GO:0045616	0.0192	1	1	9	regulation of keratinocyte differentiation	BP	1	SRSF6	down_AFEM_EC
GO:0032924	0.0192	1	1	9	activin receptor signaling pathway	BP	1	CITED2	down_VPOR_EC
GO:0060042	0.0192	1	1	14	retina morphogenesis in camera-type eye	BP	1	IRX5	up_AFEM_VSMC
GO:0010692	0.0192	1	1	5	regulation of alkaline phosphatase activity	BP	1	NPNT	up_APUL_EC
GO:0010659	0.0192	1	1	7	cardiac muscle cell apoptotic process	BP	1	NKX2-5	up_VPOR_VSMC
GO:0010665	0.0192	1	1	7	regulation of cardiac muscle cell apoptotic	BP	1	NKX2-5	up_VPOR_VSMC
GO:0060712	0.0192	1	1	4	spongiotrophoblast layer development	BP	1	GJB5	up_VSAP_VSMC
GO:0007160	0.0193	0.994	8	56	cell-matrix adhesion	BP	0.673	VEGFA,SERPINE1,ACTN1,ILK,CTNNB1,ENSCAFG00000002015,GSK3B,NRP1	down_VSAP_EC
GO:0072524	0.0193	0.995	6	37	pyridine-containing compound metabolic	BP	0.673	PGK1,TPI1,PGAM1,HIF1A,NMNAT2,ALDOC	down_VSAP_EC
GO:0046364	0.0193	0.996	5	27	monosaccharide biosynthetic process	BP	0.673	TPI1,PGAM1,SESN2,PTPN2,ATF3	down_VSAP_EC
GO:0042461	0.0193	0.998	3	12	photoreceptor cell development	BP	1	NAGLU,THY1,THRB	down_VCAV_EC
GO:0008283	0.0193	0.998	3	545	cell proliferation	BP	1	WNT7A,ARG2,ESR1	up_AORT_VSMC
GO:0031328	0.0193	0.998	3	512	positive regulation of cellular biosynthetic	BP	1	HOXA9,WNT7A,ESR1	up_AORT_VSMC
GO:0010721	0.0193	0.998	3	73	negative regulation of cell development	BP	1	HOOK3,SEMA3A,RNF6	up_VPOR_EC
GO:0007135	0.0193	0.999	2	3	meiosis II	BP	0.728	DSN1,SGO2	down_ACOR_VSMC
GO:0045144	0.0193	0.999	2	3	meiotic sister chromatid segregation	BP	0.728	DSN1,SGO2	down_ACOR_VSMC
GO:0061983	0.0193	0.999	2	3	meiosis II cell cycle process	BP	0.728	DSN1,SGO2	down_ACOR_VSMC
GO:0042481	0.0193	1	1	5	regulation of odontogenesis	BP	0.905	ASPN	down_ACOR_EC
GO:0032652	0.0193	1	1	18	regulation of interleukin-1 production	BP	1	ARG2	up_AORT_VSMC
GO:0051965	0.0193	1	1	17	positive regulation of synapse assembly	BP	1	WNT7A	up_AORT_VSMC
GO:0090342	0.0193	1	1	17	regulation of cell aging	BP	1	ARG2	up_AORT_VSMC

GO:0055062	0.0193	1	1	5	phosphate ion homeostasis	BP	1	HOXA3	up_APUL_EC
GO:0072505	0.0193	1	1	5	divalent inorganic anion homeostasis	BP	1	HOXA3	up_APUL_EC
GO:0072506	0.0193	1	1	5	trivalent inorganic anion homeostasis	BP	1	HOXA3	up_APUL_EC
GO:0051154	0.0193	1	1	7	negative regulation of striated muscle cell	BP	1	NKX2-5	up_VPOR_VSMC
GO:0055065	0.0194	0.99	22	145	metal ion homeostasis	BP	1	ADRA1B,PLCG2,HTR2B,CNNM2,MICU2,ITPR1,F2RL1,EGLN1,DMPK,ATP7A,PRKCE,APP,PSEN1,CHD7,EDN1,B2M,DISC1,HERPUD1,WFS1,ATP2C1,SLC8A1,STC2	up_ACOR_VSMC
GO:0050808	0.0194	0.991	16	93	synapse organization	BP	1	WASF3,GLRB,ZNF365,TMEM108,EGLN1,CACNB3,NRP1,DMPK,SLITRK5,DAG1,APP,PSEN1,LRRC4,DISC1,WASL,CRKL	up_ACOR_VSMC
GO:1903311	0.0194	0.992	12	72	regulation of mRNA metabolic process	BP	0.728	RNPS1,HNRNPD,SRSF2,U2AF2,THRAP3,HNRNPL,SF3B4,FASTKD3,RBM14,WTAP,JMJD6,SF3A1	down_ACOR_VSMC
GO:0048513	0.0194	0.996	6	906	animal organ development	BP	1	HOXA3,BARX1,NKX2-5,BAMBI,ANKRD6,CYP26B1	up_VPOR_VSMC
GO:0060350	0.0194	0.995	6	23	endochondral bone morphogenesis	BP	1	HOXA11,PEX7,AXIN2,RARG,SHOX2,TGFBR2	up_VSAP_EC
GO:0045143	0.0194	0.997	4	13	homologous chromosome segregation	BP	0.728	ENSCAFG00000017264,NDC1,TRIP13,ESPL1	down_ACOR_VSMC
GO:0006953	0.0194	0.998	3	7	acute-phase response	BP	0.591	F8,IL6,PTGS2	up_VCAV_EC
GO:0060685	0.0194	1	1	4	regulation of prostatic bud formation	BP	0.905	SULF1	down_ACOR_EC
GO:0060686	0.0194	1	1	4	negative regulation of prostatic bud formation	BP	0.905	SULF1	down_ACOR_EC
GO:0060502	0.0194	1	1	9	epithelial cell proliferation involved in lung	BP	1	SRSF6	down_AFEM_EC
GO:0030511	0.0194	1	1	10	positive regulation of transforming growth factor beta receptor	BP	1	CITED2	down_VPOR_EC
GO:1903846	0.0194	1	1	10	positive regulation of cellular response to transforming growth	BP	1	CITED2	down_VPOR_EC
GO:0072132	0.0194	1	1	23	mesenchyme morphogenesis	BP	1	GATA5	down_VSAP_VSMC
GO:0032945	0.0194	1	1	17	negative regulation of mononuclear cell	BP	1	ARG2	up_AORT_VSMC
GO:0050672	0.0194	1	1	17	negative regulation of lymphocyte proliferation	BP	1	ARG2	up_AORT_VSMC
GO:0043584	0.0194	1	1	5	nose development	BP	1	SIX1	up_VFEM_EC
GO:0010033	0.019424129	0.98867	30	772	response to organic substance	BP	0.96436359	NR1H4,SOX9,FNDC4,AQP1,ENSCAFG00000004331,KCNB1,EDEM2,FZD5,CCDC3,AGRN,PTGIR,NOD2,RGMA,SMAD6,ACTN2,PAK3,PTGER4,ADAMTSL2,MAVS,FOXC2,LRRC15,IFIT2,LRP4,LRRC4,CLU,RARRES2,PRKACA,NRP1,HM13,PRKCD	up_ACOR_EC
GO:0065008	0.0195	0.985	105	1056	regulation of biological quality	BP	0.593	EPHB1,PLK2,EDNRB,HCLS1,IL7R,ADGRF5,SELP,KIT,PRCP,F8,ARHGEF15,IFRD1,CP,DOCK4,TNFAIP3,ATP8A1,LRRC8D,PDGFB,BAIAP2L1,ATP2B1,RELN,VWF,CD34,PDE4D,MEF2C,CHD7,KLKB1,TIPARP,TLR4,NOS3,GPR4,STC1,GATA3,EDN1,RAB11A,APLNR,ARAP3,RAB27A,SLC39A8,SNCAIP,PTGS1,TFPI,TMOD1,PPP3CA,GUCY1A1,MTOR,NAV2,TENT2,SNAP23,MMRN1,LRRC8C,BMP2,SLC12A7,PDZD8,ABCA1,ITPKB,NCKAP1,IQGAP2,STMN1,HMBOX1,SH3GLB1,SWAP70,SMARCA4,NCK1,KCNJ2,EXOC5,LDB2,PDIA5,FES,PTGS2,PLCG2,CREB1,BCL10,USP28,TAOK1,FMNL3,STX11,SMG7,GCH1,VPS54,ADIPOR2,HMGB1,NOS2,PMAIP1,ARPC5L,BLM,SPTBN4,SMC5,USP13,SKIL,MCU,PDGFD,NR1D2,HIF1A,KDM3A,ALS2,ITGB3,ATRX,ALDH1A3,HP1BP3,P2RX2,DCP2,ATP2A1,TMOD3,PEAR1	up_VCAV_EC
GO:0051785	0.0195	0.997	4	14	positive regulation of nuclear division	BP	0.728	BIRC5,NUP62,ENSCAFG00000005299,UBE2C	down_ACOR_VSMC
GO:0044314	0.0195	0.999	2	3	protein K27-linked ubiquitination	BP	0.728	UBE2S,UBE2T	down_ACOR_VSMC
GO:0010888	0.0195	0.999	2	4	negative regulation of lipid storage	BP	0.677	PTPN2,ABHD5	down_VSAP_EC
GO:0120039	0.0195	0.999	2	158	plasma membrane bounded cell projection	BP	1	SLITRK5,LIMK1	up_VCAV_VSMC
GO:0045683	0.0195	1	1	9	negative regulation of epidermis development	BP	1	SRSF6	down_AFEM_EC

GO:0032757	0.0195	1	1	14	positive regulation of interleukin-8 production	BP	1	TLR2	up_AFEM_VSMC
GO:0048146	0.0195	1	1	18	positive regulation of fibroblast proliferation	BP	1	ESR1	up_AORT_VSMC
GO:0003264	0.0195	1	1	5	regulation of cardioblast proliferation	BP	1	TBX5	up_APUL_EC
GO:0003266	0.0195	1	1	5	regulation of secondary heart field cardioblast	BP	1	TBX5	up_APUL_EC
GO:0090103	0.0195	1	1	8	cochlea morphogenesis	BP	1	EYA1	up_VFEM_VSMC
GO:0098815	0.0195	1	1	9	modulation of excitatory postsynaptic potential	BP	1	NETO1	up_VFEM_VSMC
GO:0048736	0.0196	0.992	12	66	appendage development	BP	1	HOXA11,HOXA9,OSR2,OSR1,ZBTB16,RARG,RECK,FBXW4,SHOX2,HOXC10,LRP4,PRKAB1	up_VSAP_EC
GO:0060173	0.0196	0.992	12	66	limb development	BP	1	HOXA11,HOXA9,OSR2,OSR1,ZBTB16,RARG,RECK,FBXW4,SHOX2,HOXC10,LRP4,PRKAB1	up_VSAP_EC
GO:0009892	0.0196	0.993	9	835	negative regulation of metabolic process	BP	0.905	ACVRL1,LYN,IGF1R,HOXA7,PRKAR2A,ELK3,MYO1D,ASPN,NEDD4	down_ACOR_EC
GO:0072088	0.0196	0.995	6	36	nephron epithelium morphogenesis	BP	0.677	ENSCAFG00000032470,ILK,HOXB7,MAGED1,CTNNB1,SIX4	down_VSAP_EC
GO:0006879	0.0196	0.995	6	23	cellular iron ion homeostasis	BP	1	FXN,MYC,SCARA5,FTL,HMOX1,NCOA4	up_VSAP_EC
GO:0032875	0.0196	0.998	3	7	regulation of DNA endoreduplication	BP	0.728	SMC1A,E2F8,SMC3	down_ACOR_VSMC
GO:0010803	0.0196	0.998	3	10	regulation of tumor necrosis factor-mediated signaling pathway	BP	0.677	NR1H4,PTPN2,ADAM17	down_VSAP_EC
GO:0060338	0.0196	0.998	3	10	regulation of type I interferon-mediated	BP	0.679	PTPN2,CNOT7,CDC37	down_VSAP_EC
GO:0021675	0.0196	0.999	2	29	nerve development	BP	1	HOXA3,SEMA3A	up_VPOR_EC
GO:0021824	0.0196	1	1	3	cerebral cortex tangential migration using cell-axon interactions	BP	0.905	NRP2	down_ACOR_EC
GO:0021828	0.0196	1	1	3	gonadotrophin-releasing hormone neuronal migration to the	BP	0.905	NRP2	down_ACOR_EC
GO:0021856	0.0196	1	1	3	hypothalamic tangential migration using cell-axon interactions	BP	0.905	NRP2	down_ACOR_EC
GO:0021886	0.0196	1	1	3	hypothalamus gonadotrophin-releasing hormone neuron	BP	0.905	NRP2	down_ACOR_EC
GO:0021888	0.0196	1	1	3	hypothalamus gonadotrophin-releasing hormone neuron	BP	0.905	NRP2	down_ACOR_EC
GO:0021979	0.0196	1	1	3	hypothalamus cell differentiation	BP	0.905	NRP2	down_ACOR_EC
GO:0035290	0.0196	1	1	3	trunk segmentation	BP	0.905	NRP2	down_ACOR_EC
GO:0036484	0.0196	1	1	3	trunk neural crest cell migration	BP	0.905	NRP2	down_ACOR_EC
GO:0036486	0.0196	1	1	3	ventral trunk neural crest cell migration	BP	0.905	NRP2	down_ACOR_EC
GO:0097490	0.0196	1	1	3	sympathetic neuron projection extension	BP	0.905	NRP2	down_ACOR_EC
GO:0097491	0.0196	1	1	3	sympathetic neuron projection guidance	BP	0.905	NRP2	down_ACOR_EC
GO:1901166	0.0196	1	1	3	neural crest cell migration involved in autonomic nervous system	BP	0.905	NRP2	down_ACOR_EC
GO:1903375	0.0196	1	1	3	facioacoustic ganglion development	BP	0.905	NRP2	down_ACOR_EC
GO:0007389	0.0196	1	1	137	pattern specification process	BP	1	GATA5	down_AFEM_VSMC
GO:0048821	0.0196	1	1	10	erythrocyte development	BP	1	CITED2	down_VPOR_EC
GO:0003207	0.0196	1	1	5	cardiac chamber formation	BP	1	TBX5	up_APUL_EC
GO:0072643	0.0196	1	1	5	interferon-gamma secretion	BP	1	NR1H4	up_APUL_EC
GO:0032612	0.019699196	0.99756	3	22	interleukin-1 production	BP	0.96436359	NR1H4,FZD5,NOD2	up_ACOR_EC
GO:0030032	0.0197	0.996	5	19	lamellipodium assembly	BP	0.599	KIT,MTOR,NCKAP1,NCK1,FSCN1	up_VCAV_EC
GO:0060986	0.0197	0.998	3	10	endocrine hormone secretion	BP	0.68	PTPN11,RAB11FIP1,INHBA	down_VSAP_EC
GO:0051961	0.0197	0.997	3	73	negative regulation of nervous system	BP	1	HOOK3,SEMA3A,RNF6	up_VPOR_EC

GO:0045834	0.0197	0.999	2	41	positive regulation of lipid metabolic process	BP	0.905	LYN,ACSL3	down_ACOR_EC
GO:0048858	0.0197	0.999	2	159	cell projection morphogenesis	BP	1	SLITRK5,LIMK1	up_VCAV_VSMC
GO:0001503	0.0197	0.999	2	109	ossification	BP	1	HOXA3,PHEX	up_VPUL_VSMC
GO:1904018	0.0197	1	1	69	positive regulation of vasculature development	BP	1	JUP	down_VFEM_VSMC
GO:0032613	0.0197	1	1	14	interleukin-10 production	BP	1	TLR2	up_AFEM_VSMC
GO:0006699	0.0197	1	1	5	bile acid biosynthetic process	BP	1	NR1H4	up_APUL_EC
GO:0034162	0.0197	1	1	5	toll-like receptor 9 signaling pathway	BP	1	NR1H4	up_APUL_EC
GO:0021522	0.0197	1	1	8	spinal cord motor neuron differentiation	BP	1	HOXC10	up_VFEM_VSMC
GO:0003171	0.0197	1	1	10	atrioventricular valve development	BP	1	TBX5	up_VPUL_VSMC
GO:0048522	0.0198	0.994	10	1484	positive regulation of cellular process	BP	1	TBX5,HOXA3,NR1H4,EVI2B,WIF1,CCL24,PTGER2,FLRT3,ARHGEF2,NEO1	up_VPUL_EC
GO:0090288	0.0198	0.995	6	44	negative regulation of cellular response to growth factor stimulus	BP	1	DCN,VASN,FBN1,CHRDL1,ASPN,GREM1	down_VCAV_EC
GO:0006811	0.0198	0.996	5	330	ion transport	BP	0.906	LYN,HOXA3,PDPN,ACSL3,NEDD4	down_ACOR_EC
GO:1904738	0.0198	0.998	3	6	vascular associated smooth muscle cell	BP	0.599	DOCK4,PDGFB,MEF2C	up_VCAV_EC
GO:1904752	0.0198	0.998	3	6	regulation of vascular associated smooth muscle	BP	0.599	DOCK4,PDGFB,MEF2C	up_VCAV_EC
GO:0060019	0.0198	0.999	2	5	radial glial cell differentiation	BP	1	GLI3,CDH2	down_VCAV_EC
GO:1903011	0.0198	0.999	2	5	negative regulation of bone development	BP	1	FBN1,GREM1	down_VCAV_EC
GO:0003160	0.0198	0.999	2	3	endocardium morphogenesis	BP	0.599	SOX18,PROX1	up_VCAV_EC
GO:0006606	0.0198	1	1	67	protein import into nucleus	BP	1	JUP	down_VFEM_VSMC
GO:0055026	0.0198	1	1	5	negative regulation of cardiac muscle tissue	BP	1	TBX5	up_APUL_EC
GO:0021559	0.0198	1	1	3	trigeminal nerve development	BP	1	SEMA3A	up_VPOR_EC
GO:0021636	0.0198	1	1	3	trigeminal nerve morphogenesis	BP	1	SEMA3A	up_VPOR_EC
GO:0021637	0.0198	1	1	3	trigeminal nerve structural organization	BP	1	SEMA3A	up_VPOR_EC
GO:0021785	0.0198	1	1	3	branchiomotor neuron axon guidance	BP	1	SEMA3A	up_VPOR_EC
GO:1902287	0.0198	1	1	3	semaphorin-plexin signaling pathway involved in axon guidance	BP	1	SEMA3A	up_VPOR_EC
GO:0055012	0.0198	1	1	8	ventricular cardiac muscle cell differentiation	BP	1	NKX2-5	up_VPOR_VSMC
GO:0060261	0.0198	1	1	8	positive regulation of transcription initiation from RNA polymerase II	BP	1	NKX2-5	up_VPOR_VSMC
GO:1900449	0.0198	1	1	9	regulation of glutamate receptor signaling	BP	1	NETO1	up_VFEM_VSMC
GO:0009607	0.019847977	0.99163	12	232	response to biotic stimulus	BP	0.96436359	NR1H4,HMCN1,RGS1,FZD5,PTGIR,NOD2,PTGER4,MAVS,IFIT2,RARRES2,AGBL5,PRKCD	up_ACOR_EC
GO:0050789	0.0199	0.992	23	3295	regulation of biological process	BP	0.907	ACVRL1,YEATS2,LYN,HOXA3,IGF1R,PDPN,NRP2,YBX3,HIVEP2,HOXA7,PRKAR2A,USP28,IPO7,ATRIP,SULF1,ELK3,HOXA1,ACSL3,WDR12,STX4,MYO1D,ASPN,NEDD4	down_ACOR_EC
GO:0070527	0.0199	0.998	3	10	platelet aggregation	BP	0.685	LYN,SLC7A11,ITGB3	down_VSAP_EC
GO:1901223	0.0199	1	1	10	negative regulation of NIK/NF-kappaB signaling	BP	1	HDAC7	down_VPOR_EC
GO:2001239	0.0199	1	1	14	regulation of extrinsic apoptotic signaling pathway in absence of	BP	1	EYA1	up_AFEM_VSMC
GO:0061551	0.0199	1	1	5	trigeminal ganglion development	BP	1	SIX1	up_VFEM_EC
GO:0023052	0.02	0.992	14	1602	signaling	BP	0.907	ACVRL1,LYN,IGF1R,SPG11,PDPN,NRP2,HIVEP2,USP28,IPO7,SULF1,WDR12,STX4,ASPN,NEDD4	down_ACOR_EC
GO:0034641	0.02	0.995	9	2171	cellular nitrogen compound metabolic	BP	1	IVNS1ABP,EXTL2,DARS,SUCLG2,EIF3M,SRSF6,GABPA,NDUFA10,TSEN15	down_AFEM_EC
GO:0009889	0.02	0.995	7	1190	regulation of biosynthetic process	BP	1	EYA1,HOXA10,HOXA9,FBP1,EMX2,HOXA11,SHOX2	up_VFEM_VSMC

GO:0051894	0.02	0.998	3	10	positive regulation of focal adhesion assembly	BP	0.685	VEGFA,ENSCAFG00000002015,NRP1	down_VSAP_EC
GO:0009891	0.02	0.998	3	521	positive regulation of biosynthetic process	BP	1	HOXA9,WNT7A,ESR1	up_AORT_VSMC
GO:0000289	0.02	0.998	3	7	nuclear-transcribed mRNA poly(A) tail shortening	BP	0.602	CNOT6,PAN3,CNOT7	up_VCAV_EC
GO:0060538	0.02	0.999	2	51	skeletal muscle organ development	BP	1	SIX1,CYP26B1	up_VFEM_EC
GO:0048535	0.02	1	1	5	lymph node development	BP	0.907	PDPN	down_ACOR_EC
GO:0071867	0.02	1	1	9	response to monoamine	BP	1	GABPA	down_AFEM_EC
GO:0071868	0.02	1	1	9	cellular response to monoamine stimulus	BP	1	GABPA	down_AFEM_EC
GO:0071869	0.02	1	1	9	response to catecholamine	BP	1	GABPA	down_AFEM_EC
GO:0071870	0.02	1	1	9	cellular response to catecholamine stimulus	BP	1	GABPA	down_AFEM_EC
GO:0060135	0.02	1	1	10	maternal process involved in female pregnancy	BP	1	CITED2	down_VPOR_EC
GO:0018195	0.02	1	1	6	peptidyl-arginine modification	BP	1	NR1H4	up_VPUL_EC
GO:0010638	0.0201	0.989	26	207	positive regulation of organelle organization	BP	0.604	TEK,ARHGEF15,PDGFB,BAIAP2L1,GATA3,EDN1,RALB,MTOR,CNOT6,NCKAP1,PROX1,IQGAP2,HMBOX1,PRKD2,SH3GLB1,SWAP70,NCK1,FES,PAN3,PMAIP1,ARPC5L,SMC5,RAD21,HIF1A,ATRX,FSCN1	up_VCAV_EC
GO:0060428	0.0201	0.996	5	17	lung epithelium development	BP	1	THRB,FGF7,SAV1,NFIB,MAP2K2	up_VSAP_EC
GO:0060669	0.0201	0.997	4	12	embryonic placenta morphogenesis	BP	1	FGFR2,SPINT1,ENSCAFG00000006238,SPINT2	up_ACOR_VSMC
GO:0032611	0.0201	1	1	17	interleukin-1 beta production	BP	1	ARG2	up_AORT_VSMC
GO:2000826	0.0201	1	1	14	regulation of heart morphogenesis	BP	1	GATA5	up_VCAV_VSMC
GO:0010575	0.0201	1	1	8	positive regulation of vascular endothelial growth factor production	BP	1	C3AR1	up_VPOR_VSMC
GO:0010804	0.0201	1	1	6	negative regulation of tumor necrosis factor-mediated signaling	BP	1	NR1H4	up_VPUL_EC
GO:0003161	0.0201	1	1	6	cardiac conduction system development	BP	1	TBX5	up_VPUL_EC
GO:0043434	0.020102501	0.99454	6	80	response to peptide hormone	BP	0.96436359	NR1H4,ENSCAFG00000004331,ACTN2,FOXC2,RARRES2,PRKCD	up_ACOR_EC
GO:0001819	0.0202	0.99	19	120	positive regulation of cytokine production	BP	1	WNT11,CD274,IL6R,ABL1,IL17RC,HPSE,CCBE1,F2RL1,PIK3R1,SULF2,MYD88,NLRX1,TKFC,CD83,ZNF580,CYBA,PQBP1,FLOT1,MAVS	up_VSAP_EC
GO:0070838	0.0202	0.99	16	96	divalent metal ion transport	BP	1	PLCG2,HTR2B,TPC3,NIPAL3,SARAF,MICU2,ITPR1,CACNB3,PRKCE,PSEN1,CHD7,INPP5K,WFS1,ATP2C1,SLC8A1,STC2	up_ACOR_VSMC
GO:0051258	0.0202	0.991	14	94	protein polymerization	BP	0.606	HCLS1,MX2,BAIAP2L1,TMOD1,MTOR,NCKAP1,MZT1,IQGAP2,STMN1,NCK1,FES,ARPC5L,TMOD3,FBXO5	up_VCAV_EC
GO:0046165	0.0202	0.994	8	42	alcohol biosynthetic process	BP	0.739	LSS,SREBF1,HMGCR,INSIG1,DHCR24,MVD,FDFT1,NUS1	down_ACOR_VSMC
GO:1901800	0.0202	0.994	7	46	positive regulation of proteasomal protein catabolic process	BP	0.692	PRICKLE1,PLK3,SOCS5,TRIB3,GSK3B,RAD23A,TMTC3	down_VSAP_EC
GO:0055025	0.0202	0.996	5	17	positive regulation of cardiac muscle tissue	BP	1	BMP4,FGFR2,TBX20,TBX5,EDN1	up_ACOR_VSMC
GO:0016572	0.0202	0.997	4	13	histone phosphorylation	BP	0.739	CDK1,AURKB,HASPIN,CCNA2	down_ACOR_VSMC
GO:0071384	0.0202	0.997	4	12	cellular response to corticosteroid stimulus	BP	1	CREBRF,NR3C1,FAM107A,DDIT4	up_ACOR_VSMC
GO:0071385	0.0202	0.997	4	12	cellular response to glucocorticoid stimulus	BP	1	CREBRF,NR3C1,FAM107A,DDIT4	up_ACOR_VSMC
GO:0012501	0.0202	0.998	3	561	programmed cell death	BP	1	WNT7A,ARG2,ESR1	up_AORT_VSMC
GO:0006355	0.0202	1	2	974	regulation of transcription, DNA-	BP	1	JUP,GATA5	down_VFEM_VSMC
GO:0071073	0.0202	1	1	4	positive regulation of phospholipid biosynthetic	BP	0.913	ACSL3	down_ACOR_EC
GO:0032528	0.0202	1	1	9	microvillus organization	BP	1	FSCN1	down_AFEM_EC
GO:0043124	0.0202	1	1	17	negative regulation of I-kappaB kinase/NF-kappaB	BP	1	ESR1	up_AORT_VSMC
GO:0060079	0.0202	1	1	15	excitatory postsynaptic potential	BP	1	WNT7A	up_AORT_VSMC

GO:0070664	0.0202	1	1	18	negative regulation of leukocyte proliferation	BP	1	ARG2	up_AORT_VSMC
GO:0046620	0.0202	1	1	30	regulation of organ growth	BP	1	TBX5	up_APUL_VSMC
GO:0048246	0.0202	1	1	9	macrophage chemotaxis	BP	1	C3AR1	up_VPOR_VSMC
GO:0060413	0.0202	1	1	8	atrial septum morphogenesis	BP	1	NKX2-5	up_VPOR_VSMC
GO:0003283	0.0202	1	1	8	atrial septum development	BP	1	NKX2-5	up_VPOR_VSMC
GO:0097120	0.0202	1	1	9	receptor localization to synapse	BP	1	NETO1	up_VFEM_VSMC
GO:0010984	0.0202	1	1	6	regulation of lipoprotein particle clearance	BP	1	NR1H4	up_VPUL_EC
GO:0006929	0.0202	1	1	10	substrate-dependent cell migration	BP	1	SLIT2	up_VPUL_VSMC
GO:0007169	0.020218582	0.99222	10	178	transmembrane receptor protein tyrosine kinase	BP	0.96436359	SH3TC2,CDH3,NR1H4,SOX9,ENSCAFG00000004331,PTGIR,ESM1,FOXC2,NRP1,PRKCD	up_ACOR_EC
GO:0001936	0.0203	0.994	7	49	regulation of endothelial cell proliferation	BP	0.694	VEGFA,SULF1,SPARC,JCAD,TGFBR1,ADAM17,APOE	down_VSAP_EC
GO:1903506	0.0203	1	2	977	regulation of nucleic acid-templated transcription	BP	1	JUP,GATA5	down_VFEM_VSMC
GO:0045861	0.0203	1	1	71	negative regulation of proteolysis	BP	1	CST3	down_AORT_EC
GO:0051170	0.0203	1	1	69	import into nucleus	BP	1	JUP	down_VFEM_VSMC
GO:0030852	0.0203	1	1	6	regulation of granulocyte differentiation	BP	1	EVI2B	up_VPUL_EC
GO:0032692	0.0203	1	1	6	negative regulation of interleukin-1 production	BP	1	NR1H4	up_VPUL_EC
GO:0055090	0.0203	1	1	6	acylglycerol homeostasis	BP	1	NR1H4	up_VPUL_EC
GO:0070328	0.0203	1	1	6	triglyceride homeostasis	BP	1	NR1H4	up_VPUL_EC
GO:0021884	0.0203	1	1	10	forebrain neuron development	BP	1	SLIT2	up_VPUL_VSMC
GO:0030325	0.0203	1	1	5	adrenal gland development	BP	1	SALL1	up_VSAP_VSMC
GO:0070391	0.0203	1	1	5	response to lipoteichoic acid	BP	1	TLR2	up_VSAP_VSMC
GO:0071223	0.0203	1	1	5	cellular response to lipoteichoic acid	BP	1	TLR2	up_VSAP_VSMC
GO:0007045	0.020382812	0.99743	3	23	cell-substrate adherens junction assembly	BP	0.96436359	ARHGAP6,ACTN2,NRP1	up_ACOR_EC
GO:0048041	0.020382812	0.99743	3	23	focal adhesion assembly	BP	0.96436359	ARHGAP6,ACTN2,NRP1	up_ACOR_EC
GO:0009792	0.0204	0.988	32	231	embryo development ending in birth or egg hatching	BP	1	TBX15,HOXA11,FZD1,HOXA7,HOXA9,WNT11,AXIN2,OSR2,GJB5,IRX5,HOXD3,ABL1,DLL1,HOXA6,OSR1,FUZ,RARG,AMBRA1,MFN2,ZFP36L1,FXN,ZEB1,SULF2,SHOX2,KDM2B,TAF8,ZFAND5,ACVRL1,TGFBR2,CITED2,KEAP1,LATS2	up_VSAP_EC
GO:0090087	0.0204	0.988	27	185	regulation of peptide transport	BP	1	EXPH5,SH3TC2,TLR1,LEP,HTR2B,TCAF1,MBP,FERMT1,BMP4,TM7SF3,CD274,ANKRD1,F2RL1,CDKN1A,PTPN22,OS9,PRKCE,PSEN1,CHD7,JUP,CCL5,NOD2,SCAMP5,INPP5K,PFKL,PARK7,PTPN14	up_ACOR_VSMC
GO:0006636	0.0204	0.997	4	14	unsaturated fatty acid biosynthetic process	BP	0.612	EDN1,PTGS1,ELOVL7,PTGS2	up_VCAV_EC
GO:0045926	0.0204	0.997	3	72	negative regulation of growth	BP	1	SEMA3A,FBP1,RNF6	up_VPOR_EC
GO:0030509	0.0204	0.999	2	47	BMP signaling pathway	BP	0.922	ACVRL1,SULF1	down_ACOR_EC
GO:2001141	0.0204	1	2	979	regulation of RNA biosynthetic process	BP	1	JUP,GATA5	down_VFEM_VSMC
GO:0033629	0.0204	0.999	2	4	negative regulation of cell adhesion mediated by	BP	0.695	SERPINE1,PTPN11	down_VSAP_EC
GO:0106106	0.0204	1	1	48	cold-induced thermogenesis	BP	1	PLCL1	down_VFEM_EC
GO:0120161	0.0204	1	1	48	regulation of cold-induced thermogenesis	BP	1	PLCL1	down_VFEM_EC
GO:0010594	0.0204	1	1	71	regulation of endothelial cell migration	BP	1	JUP	down_VFEM_VSMC
GO:2000826	0.0204	1	1	14	regulation of heart morphogenesis	BP	1	EYA1	up_AFEM_VSMC
GO:0006749	0.0204	1	1	14	glutathione metabolic process	BP	1	ENSCAFG00000028814	up_VCAV_VSMC
GO:1905521	0.0204	1	1	9	regulation of macrophage migration	BP	1	C3AR1	up_VPOR_VSMC

GO:0035774	0.0204	1	1	6	positive regulation of insulin secretion involved in cellular response to	BP	1	NR1H4	up_VPUL_EC
GO:0044271	0.0205	0.992	12	1501	cellular nitrogen compound biosynthetic	BP	1	HOXA11,TBX15,HOXA10,HOXA9,PITX1,EMX2,SALL1,IRX5,PRR16,TLR2,CAV1,SHOX2	up_VSAP_VSMC
GO:0006890	0.0205	0.996	4	18	retrograde vesicle-mediated transport, Golgi	BP	0.698	RAB6A,COPB2,HTT,ARF4	down_VSAP_EC
GO:0007628	0.0205	0.997	4	11	adult walking behavior	BP	1	GLRB,CHD7,CLN8,OXR1	up_ACOR_VSMC
GO:1901863	0.0205	0.997	3	25	positive regulation of muscle tissue	BP	1	DLL1,GREM1,PPARGC1A	up_AFEM_EC
GO:0050673	0.0205	0.997	3	135	epithelial cell proliferation	BP	1	EYA1,SIX1,MTSS1	up_VFEM_EC
GO:0015980	0.0205	0.999	2	106	energy derivation by oxidation of organic	BP	1	SUCLG2,NDUFA10	down_AFEM_EC
GO:0021515	0.0205	1	1	9	cell differentiation in spinal cord	BP	1	HOXC10	up_AORT_EC
GO:0070231	0.0205	1	1	18	T cell apoptotic process	BP	1	ARG2	up_AORT_VSMC
GO:0060788	0.0205	1	1	5	ectodermal placode formation	BP	1	SIX1	up_VFEM_EC
GO:0071697	0.0205	1	1	5	ectodermal placode morphogenesis	BP	1	SIX1	up_VFEM_EC
GO:0043549	0.0206	0.988	30	244	regulation of kinase activity	BP	0.614	TEK,KIT,PPP2R5A,TNFAIP3,MAP3K11,PDGFB,STK10,RELN,TLR4,NLRC5,EDN1,RALB,NEDD9,MTOR,MAP3K3,BMP2,HHEX,PREX2,SLK,SH3BP5,PROX1,MADD,PAK2,SH3GLB1,LDB2,MMD,RAPGEF2,BLM,RIPK1,PDGFD	up_VCAV_EC
GO:0006875	0.0206	0.989	20	129	cellular metal ion homeostasis	BP	1	ADRA1B,PLCG2,HTR2B,MICU2,ITPR1,F2RL1,EGLN1,DMPK,ATP7A,PRKCE,APP,PSEN1,CHD7,EDN1,DISC1,HERPUD1,WFS1,ATP2C1,SLC8A1,STC2	up_ACOR_VSMC
GO:1901576	0.0206	0.992	14	1877	organic substance biosynthetic process	BP	1	HOXA11,TBX15,HOXA10,HOXA9,PITX1,EMX2,GATM,SALL1,GALNT17,IRX5,PRR16,TLR2,CAV1,SHOX2	up_VSAP_VSMC
GO:0009451	0.0206	0.992	10	59	RNA modification	BP	0.748	NOP56,TRUB2,FBL,THUMPD2,OSGEP,ENSCAFG00000019620,METTL1,NOP58,WTAP,EMG1	down_ACOR_VSMC
GO:0008203	0.0206	0.993	8	37	cholesterol metabolic process	BP	1	LIPC,INSIG2,LMF1,LCAT,MVK,LIPE,CYB5R3,SOAT1	up_VSAP_EC
GO:1903556	0.0206	0.997	4	14	negative regulation of tumor necrosis factor superfamily cytokine	BP	0.614	TNFAIP3,CD34,TLR4,ARG2	up_VCAV_EC
GO:0032703	0.0206	0.998	3	8	negative regulation of interleukin-2 production	BP	0.614	TNFAIP3,GATA3,HOMER3	up_VCAV_EC
GO:0036022	0.0206	0.999	2	4	limb joint morphogenesis	BP	0.698	SULF1,CTNNB1	down_VSAP_EC
GO:0006874	0.0206	0.999	2	91	cellular calcium ion homeostasis	BP	1	TRPC4,C3AR1	up_VPOR_VSMC
GO:0043271	0.0206	1	1	28	negative regulation of ion transport	BP	1	HOXA3	up_APUL_VSMC
GO:0042473	0.0206	1	1	5	outer ear morphogenesis	BP	1	EYA1	up_VFEM_EC
GO:0032640	0.020606327	0.9962	4	39	tumor necrosis factor production	BP	0.96436359	NR1H4,FZD5,NOD2,MAVS	up_ACOR_EC
GO:0019932	0.020614546	0.99436	6	82	second-messenger-mediated signaling	BP	0.96436359	SOX9,PTH1R,AQP1,PTGIR,PDE4A,GUCY1B1	up_ACOR_EC
GO:0050770	0.020619748	0.9962	4	39	regulation of axonogenesis	BP	0.96436359	BRSK1,PAK3,LRP4,NRP1	up_ACOR_EC
GO:0031329	0.0207	0.989	23	241	regulation of cellular catabolic process	BP	0.699	PRICKLE1,PLK3,SOCS5,TRIB3,PGAM1,SESN2,MSN,HIF1A,UCHL5,SMG7,MTDH,CNOT7,APOE,BNIP3,GSK3B,CDC37,SH3GLB1,HTT,ABHD5,DISC1,RAD23A,TMTC3,USP14	down_VSAP_EC
GO:0002791	0.0207	0.99	17	104	regulation of peptide secretion	BP	1	EXPH5,TLR1,LEP,HTR2B,MBP,FERMT1,TM7SF3,CD274,ANKRD1,F2RL1,PTPN22,PRKCE,CHD7,CCL5,NOD2,SCAMP5,PFKL	up_ACOR_VSMC
GO:0022603	0.0207	0.998	3	301	regulation of anatomical structure morphogenesis	BP	1	TBX5,GATA5,SLIT2	up_VPUL_VSMC
GO:0018216	0.0207	0.999	2	3	peptidyl-arginine methylation	BP	0.749	PRMT1,PRMT5	down_ACOR_VSMC
GO:0031998	0.0207	0.999	2	5	regulation of fatty acid beta-oxidation	BP	1	AKT2,LONP2	down_VCAV_EC
GO:0060346	0.0207	0.999	2	5	bone trabecula formation	BP	1	THBS3,GREM1	down_VCAV_EC
GO:0031114	0.0207	1	1	9	regulation of microtubule depolymerization	BP	1	TAOK1	up_AORT_EC
GO:0050829	0.0207	1	1	8	defense response to Gram-negative bacterium	BP	1	BPI	up_AORT_EC
GO:0050919	0.0207	1	1	10	negative chemotaxis	BP	1	SLIT2	up_VPUL_VSMC

GO:0032760	0.020761855	0.99737	3	22	positive regulation of tumor necrosis factor	BP	0.96436359	FZD5,NOD2,MAVS	up_ACOR_EC
GO:1903557	0.020761855	0.99737	3	22	positive regulation of tumor necrosis factor superfamily cytokine	BP	0.96436359	FZD5,NOD2,MAVS	up_ACOR_EC
GO:0009880	0.020799091	0.99737	3	22	embryonic pattern specification	BP	0.96436359	FZD5,SMAD6,NRP1	up_ACOR_EC
GO:2000058	0.0208	0.993	9	68	regulation of ubiquitin-dependent protein catabolic process	BP	0.701	PRICKLE1,PLK3,SOCS5,TRIB3,UCHL5,GSK3B,DISC1,RAD23A,USP14	down_VSAP_EC
GO:0010628	0.0208	0.998	3	527	positive regulation of gene expression	BP	1	HOXA9,WNT7A,ESR1	up_AORT_VSMC
GO:0001936	0.0208	0.999	2	49	regulation of endothelial cell proliferation	BP	0.926	ACVRL1,SULF1	down_ACOR_EC
GO:0048771	0.0208	0.999	2	43	tissue remodeling	BP	0.926	ACVRL1,HOXA3	down_ACOR_EC
GO:0071356	0.0208	0.999	2	57	cellular response to tumor necrosis factor	BP	1	NR1H4,NPNT	up_APUL_EC
GO:0060429	0.0208	0.999	2	341	epithelium development	BP	1	TBX5,HOXB5	up_APUL_VSMC
GO:1990791	0.0208	1	1	3	dorsal root ganglion development	BP	0.926	NRP2	down_ACOR_EC
GO:0009268	0.0208	1	1	10	response to pH	BP	1	RAB11B	down_VPOR_EC
GO:0060872	0.0208	1	1	6	semicircular canal development	BP	1	HOXA1	up_APUL_EC
GO:0010960	0.0208	1	1	6	magnesium ion homeostasis	BP	1	HOXA3	up_VPUL_EC
GO:0055083	0.0208	1	1	6	monovalent inorganic anion homeostasis	BP	1	HOXA3	up_VPUL_EC
GO:0042473	0.0208	1	1	5	outer ear morphogenesis	BP	1	SALL1	up_VSAP_VSMC
GO:0010468	0.0209	0.996	5	1270	regulation of gene expression	BP	1	TLR2,EYA1,HOXA9,HOXA10,IRX5	up_AFEM_VSMC
GO:0002052	0.0209	0.998	3	9	positive regulation of neuroblast proliferation	BP	0.701	HIF1A,CTNNB1,DISC1	down_VSAP_EC
GO:0033194	0.0209	0.998	3	7	response to hydroperoxide	BP	1	TP53INP1,DAPK1,OXR1	up_ACOR_VSMC
GO:0007423	0.0209	0.997	3	155	sensory organ development	BP	1	ZDHHC16,NTRK2,HOXA1	up_APUL_EC
GO:0061724	0.0209	0.999	2	4	lipophagy	BP	0.701	SESN2,HTT	down_VSAP_EC
GO:0061448	0.0209	0.999	2	89	connective tissue development	BP	1	HOXA11,SHOX2	up_VFEM_VSMC
GO:0045603	0.0209	1	1	5	positive regulation of endothelial cell	BP	0.926	ACVRL1	down_ACOR_EC
GO:0016579	0.0209	1	1	49	protein deubiquitination	BP	1	USP12	down_VFEM_EC
GO:0003344	0.0209	1	1	6	pericardium morphogenesis	BP	1	FLRT3	up_VPUL_EC
GO:0006796	0.021	0.984	93	938	phosphate-containing compound metabolic process	BP	0.621	EPHB1,PLK2,TEK,HCLS1,NPR1,ADGRF5,KIT,PPP2R5A,PANK3,PRKCH,CDA,TNFAIP3,MAP3K11,TNIP1,PDGFB,STK10,SH2D3C,RELN,PDE4D,MEF2C,ATF3,RAPGEF1,TLR4,NLRC5,GK5,EDN1,RALB,APLNR,CTTNBP2NL,PIPOX,NEDD9,SERINC5,ADSS,YES1,GUCY1A1,SPTLC2,EPHB4,MTOR,ACPP,SAMD8,MAP3K3,BMP2,HHEX,PREX2,SLK,BMP2K,SH3BP5,PPP1R2,ERC1,MAPK13,ITPKB,PROX1,ENSCAFG00000010596,MADD,PAK2,PRKD2,SH3GLB1,SWAP70,ELOVL7,TLR3,ENSCAFG00000024323,NCK1,LDB2,SLC25A25,FES,NSD1,GUCY1B1,PTGS2,MMD,TOP1,RAPGEF2,PLCG2,CREB1,TAOK1,CNOT7,DCK,HMGB1,NOS2,BLM,SPTBN4,RIPK1,ITGA6,RAD17,IMPA1,PDGFD,PIP5K1B,BMX,HIF1A,LMTK2,FKBP15,JCAD,ARHGEF2,CDON	up_VCAV_EC

GO:0006464	0.021	0.985	86	1162	cellular protein modification process	BP	0.701	ARHGEF2,PRICKLE1,VEGFA,P4HB,PLOD2,NR1H4,HSPA5,SOCS5,SGK1,ERO1A,ENPP1,ERRFI1,P4HA1,JCAD,SNX25,WSB1,BCAR3,NUAK1,PTPN11,TRIB3,PUM3,ILK,PPP1R12A,SESN2,PTGS2,PPIB,USP12,IWS1,DNAJC10,TNIK,GALNT1,NAA10,NXN,PTPN2,UCHL5,MORF4L2,SPOPL,YES1,PLCL1,TTLL7,ADAM17,CNOT7,MAGED1,CTNNB1,CDK8,APOE,PLOD3,USP48,UBA5,LYN,STRAP,BACH1,ASPH,CCNYL1,STT3A,MMD,CBLC,ATF3,GSK3B,VLDLR,GTF2H1,CUL2,HTT,REST,ZMYND8,MAPK10,EIF2S1,PRMT1,P3H4,EXT1,CALM1,CDYL,PAK2,INHBA,CAMK2D,HIF1AN,BTK,CDKN1A,NRP1,SH3RF3,USP14,ARF4,MADD,RPN1,KDM3A,MID1	down_VSAP_EC
GO:0036211	0.021	0.985	86	1162	protein modification process	BP	0.701	ARHGEF2,PRICKLE1,VEGFA,P4HB,PLOD2,NR1H4,HSPA5,SOCS5,SGK1,ERO1A,ENPP1,ERRFI1,P4HA1,JCAD,SNX25,WSB1,BCAR3,NUAK1,PTPN11,TRIB3,PUM3,ILK,PPP1R12A,SESN2,PTGS2,PPIB,USP12,IWS1,DNAJC10,TNIK,GALNT1,NAA10,NXN,PTPN2,UCHL5,MORF4L2,SPOPL,YES1,PLCL1,TTLL7,ADAM17,CNOT7,MAGED1,CTNNB1,CDK8,APOE,PLOD3,USP48,UBA5,LYN,STRAP,BACH1,ASPH,CCNYL1,STT3A,MMD,CBLC,ATF3,GSK3B,VLDLR,GTF2H1,CUL2,HTT,REST,ZMYND8,MAPK10,EIF2S1,PRMT1,P3H4,EXT1,CALM1,CDYL,PAK2,INHBA,CAMK2D,HIF1AN,BTK,CDKN1A,NRP1,SH3RF3,USP14,ARF4,MADD,RPN1,KDM3A,MID1	down_VSAP_EC
GO:2000026	0.021	0.986	46	560	regulation of multicellular organismal development	BP	0.701	ARHGEF2,PRICKLE1,VEGFA,SIPA1L1,SULF1,NR1H4,SERPINE1,SOCS5,SPARC,PRKACB,ERRFI1,JCAD,PGK1,DPYSL3,HOOK3,IFRD1,TGFBR1,PIK3C2A,HOXB7,HIF1A,SLC45A3,THBS2,TNIK,ZPR1,PTPN2,EVI2B,MTDH,UST,MAGED1,CTNNB1,STARD13,TFRC,APOE,LYN,STRAP,DDIT3,MMD,VLDLR,REST,PRMT1,DISC1,LRRTM2,INHBA,SIX4,NRP1,YWHAH	down_VSAP_EC
GO:0033962	0.021	0.997	4	12	cytoplasmic mRNA processing body assembly	BP	0.621	CNOT6,PAN3,CNOT7,LSM14A	up_VCAV_EC
GO:0006613	0.021	0.998	3	12	cotranslational protein targeting to membrane	BP	1	SSR2,SSR3,SEC61B	down_VCAV_EC
GO:0006273	0.021	0.999	2	3	lagging strand elongation	BP	0.758	POLA1,FEN1	down_ACOR_VSMC
GO:0032055	0.021	0.999	2	4	negative regulation of translation in response to	BP	0.701	SESN2,EIF2S1	down_VSAP_EC
GO:0097527	0.021	0.999	2	3	necroptotic signaling pathway	BP	0.621	TLR3,RIPK1	up_VCAV_EC
GO:1903053	0.021	1	1	10	regulation of extracellular matrix organization	BP	1	FSCN1	down_AFEM_EC
GO:2000027	0.021	1	1	72	regulation of animal organ morphogenesis	BP	1	GATA5	down_VFEM_VSMC
GO:0055007	0.021	1	1	31	cardiac muscle cell differentiation	BP	1	TBX5	up_APUL_VSMC
GO:0061436	0.021	1	1	5	establishment of skin barrier	BP	1	CYP26B1	up_VFEM_EC
GO:0007252	0.021	1	1	6	I-kappaB phosphorylation	BP	1	TLR2	up_VSAP_VSMC
GO:0072012	0.021071517	0.99874	2	9	glomerulus vasculature development	BP	0.96436359	AQP1,FOXC2	up_ACOR_EC
GO:0010628	0.0211	0.985	58	527	positive regulation of gene expression	BP	0.621	HCLS1,IL7R,KIT,PPARG,TNIP1,PDGFB,TMEM173,MEF2C,ATF3,CHD7,DLL4,TLR4,NLRC5,GATA3,EDN1,RAB27A,TCF4,FOXN2,ENSCAFG00000017103,IRF8,PPP3CA,YES1,MTOR,BMP2,HHEX,CREM,PROX1,ARID4B,HMBOX1,PRKD2,THOC1,SMARCA4,TLR3,NCK1,LDB2,JAG1,NSD1,BCL10,CNOT7,HMGB1,AHR,BLM,RIPK1,ITGA6,GPBP1,MTA2,NR1D2,KDM5A,HIF1A,KDM3A,ATRX,NR4A1,ENSCAFG00000002015,ZEB1,TET2,ARHGEF2,CDON,ZNF609	up_VCAV_EC
GO:0001654	0.0211	0.99	16	110	eye development	BP	0.621	EPHB1,ARHGEF15,CHD7,DLL4,GATA3,TMOD1,PROX1,TSPAN12,SMARCA4,SLC25A25,JAG1,SKIL,HIF1A,ALDH1A3,ZEB1,CDON	up_VCAV_EC
GO:0048880	0.0211	0.99	16	110	sensory system development	BP	0.621	EPHB1,ARHGEF15,CHD7,DLL4,GATA3,TMOD1,PROX1,TSPAN12,SMARCA4,SLC25A25,JAG1,SKIL,HIF1A,ALDH1A3,ZEB1,CDON	up_VCAV_EC
GO:0150063	0.0211	0.99	16	110	visual system development	BP	0.621	EPHB1,ARHGEF15,CHD7,DLL4,GATA3,TMOD1,PROX1,TSPAN12,SMARCA4,SLC25A25,JAG1,SKIL,HIF1A,ALDH1A3,ZEB1,CDON	up_VCAV_EC

GO:0001822	0.0211	0.992	10	98	kidney development	BP	1	SMAD9,KANK2,CLSTN1,PKD1,GLI3,GLIS2,RDH10,GREM1,ZBTB16,SEC61A1	down_VCAV_EC
GO:0050863	0.0211	0.993	8	69	regulation of T cell activation	BP	1	GPAM,PRNP,SDC4,THY1,GLI3,LOXL3,RAC2,ZBTB16	down_VCAV_EC
GO:0043038	0.0211	0.994	6	34	amino acid activation	BP	0.701	CARS,SARS,AARS,YARS,TARS,NARS	down_VSAP_EC
GO:0010562	0.0211	0.996	4	312	positive regulation of phosphorus metabolic	BP	1	NR1H4,CCL24,PTGER2,ARHGEF2	up_VPUL_EC
GO:0045937	0.0211	0.996	4	312	positive regulation of phosphate metabolic	BP	1	NR1H4,CCL24,PTGER2,ARHGEF2	up_VPUL_EC
GO:0098662	0.0211	0.997	3	131	inorganic cation transmembrane transport	BP	1	NETO1,STIM1,CAV1	up_VSAP_VSMC
GO:0090288	0.0211	0.999	2	44	negative regulation of cellular response to growth factor stimulus	BP	0.926	SULF1,ASPN	down_ACOR_EC
GO:0051186	0.0211	0.999	2	159	cofactor metabolic process	BP	1	CYP1A1,ENSCAFG00000028814	up_VCAV_VSMC
GO:2000010	0.0211	1	1	5	positive regulation of protein localization to cell	BP	0.926	STX4	down_ACOR_EC
GO:0030516	0.0211	1	1	15	regulation of axon extension	BP	1	LIMK1	up_VCAV_VSMC
GO:0002762	0.0211	1	1	15	negative regulation of myeloid leukocyte	BP	1	LRRC17	up_VCAV_VSMC
GO:0050732	0.0212	0.996	4	18	negative regulation of peptidyl-tyrosine	BP	0.702	SOCS5,ERRFI1,PTPN2,CBLC	down_VSAP_EC
GO:0001659	0.0212	0.999	2	54	temperature homeostasis	BP	1	HOXC10,MAP2K6	up_VFEM_EC
GO:0061502	0.0212	1	1	3	early endosome to recycling endosome	BP	0.926	MYO1D	down_ACOR_EC
GO:0001881	0.0212	1	1	11	receptor recycling	BP	1	RAB11B	down_VPOR_EC
GO:0006936	0.0212	1	1	74	muscle contraction	BP	1	JUP	down_VFEM_VSMC
GO:0035196	0.0212	1	1	16	production of miRNAs involved in gene silencing	BP	1	ESR1	up_AORT_VSMC
GO:0032501	0.02128002	0.98578	60	1782	multicellular organismal process	BP	0.96436359	SH3TC2,CPAMD8,CDH3,NR1H4,TMOD1,GATA4,BRSK1,TBX18,PLCL1,SOX9,PTH1R,PCSK1N,NDRG2,AQP1,ENSCAFG00000004331,FZD5,SEMA6D,GNAO1,DIXDC1,PDLIM3,NOD2,IL33,RGMA,LHX9,PDE4A,INSYN1,SMAD6,PLS1,ACTN2,RNLS,ESM1,PTGDS,PAK3,DLX3,TRIM8,TNFRSF19,PTGER4,ARHGDIB,DCLK2,ADAMTSL2,USP53,MAPK8IP2,MAVS,FOXC2,FIGNL1,TBX5,LRP4,CLSTN1,C23H3orf58,CHMP4B,TP53INP2,RARRES2,NRP1,AGPAT2,PRKCD,PM20D1,PYGO2,DBNDD2,EFEMP1,CCNYL1	up_ACOR_EC
GO:1901698	0.0213	0.987	33	266	response to nitrogen compound	BP	0.624	TNFAIP3,PPARG,P2RX7,BAIAP2L1,TMEM173,SELL,PDE4D,MEF2C,RAPGEF1,TLR4,RALB,PELI1,MTOR,ABCA1,MAPK13,RIOK3,TLR3,NCK1,TIMELESS,JAG1,GUCY1B1,RAPGEF2,CREB1,CTNNA1,PMAIP1,BLM,USP13,PDGFD,ATRX,NR4A1,P2RX2,ZEB1,ARHGEF2	up_VCAV_EC
GO:0010467	0.0213	0.996	6	1787	gene expression	BP	1	TLR2,TBX15,EYA1,HOXA9,HOXA10,IRX5	up_AFEM_VSMC
GO:0055013	0.0213	0.996	5	17	cardiac muscle cell development	BP	1	ACTN2,BMP4,NEBL,EDN1,BVES	up_ACOR_VSMC
GO:0072606	0.0213	0.997	4	12	interleukin-8 secretion	BP	1	C5AR2,ANXA4,F2RL1,MAPKBP1	up_VSAP_EC
GO:0048646	0.0213	0.999	2	349	anatomical structure formation involved in	BP	1	HOXA3,TBX5	up_APUL_VSMC
GO:0036022	0.0213	1	1	4	limb joint morphogenesis	BP	0.928	SULF1	down_ACOR_EC
GO:0045668	0.0213	1	1	11	negative regulation of osteoblast differentiation	BP	1	HDAC7	down_VPOR_EC
GO:0034499	0.0213	1	1	3	late endosome to Golgi transport	BP	1	GCC2	up_VPOR_EC
GO:0014855	0.021307998	0.99728	3	22	striated muscle cell proliferation	BP	0.96436359	FOXC2,TBX5,C23H3orf58	up_ACOR_EC
GO:0061448	0.0214	0.99	15	89	connective tissue development	BP	1	HOXA11,AXIN2,OSR2,HOXD3,EBF2,OSR1,ZBTB16,RARG,HOXC4,ZEB1,FBXW4,SULF2,SHOX2,NFIB,TGFBR2	up_VSAP_EC
GO:0001838	0.0214	0.993	8	56	embryonic epithelial tube formation	BP	0.709	PRICKLE1,PRKACB,ENSCAFG00000032470,TMED2,HIF1A,CTNNB1,SDC4,SIX4	down_VSAP_EC
GO:0001935	0.0214	0.999	2	50	endothelial cell proliferation	BP	0.928	ACVRL1,SULF1	down_ACOR_EC
GO:0035860	0.0214	1	1	4	glial cell-derived neurotrophic factor	BP	0.928	SULF1	down_ACOR_EC
GO:1902803	0.0214	1	1	20	regulation of synaptic vesicle transport	BP	1	WNT7A	up_AORT_VSMC

GO:2000300	0.0214	1	1	20	regulation of synaptic vesicle exocytosis	BP	1	WNT7A	up_AORT_VSMC
GO:1902622	0.0214	1	1	11	regulation of neutrophil migration	BP	1	SLIT2	up_VPUL_VSMC
GO:2000209	0.0214	1	1	5	regulation of anoikis	BP	1	CAV1	up_VSAP_VSMC
GO:2000811	0.0214	1	1	5	negative regulation of anoikis	BP	1	CAV1	up_VSAP_VSMC
GO:0048246	0.021406736	0.99871	2	9	macrophage chemotaxis	BP	0.96436359	MMP28,RARRES2	up_ACOR_EC
GO:1905521	0.021442726	0.99871	2	9	regulation of macrophage migration	BP	0.96436359	MMP28,RARRES2	up_ACOR_EC
GO:0050679	0.0215	0.992	10	62	positive regulation of epithelial cell proliferation	BP	0.628	TNFAIP3,PDGFB,APLNR,EGFL7,BMP2,PROX1,PRKD2,HMGB1,NRAS,JCAD	up_VCAV_EC
GO:0045646	0.0215	0.996	4	19	regulation of erythrocyte differentiation	BP	0.71	HIF1A,LYN,PRMT1,INHBA	down_VSAP_EC
GO:0050702	0.0215	0.997	4	13	interleukin-1 beta secretion	BP	0.627	GBP5,TLR4,ARG2,ABCA1	up_VCAV_EC
GO:0072604	0.0215	0.997	4	12	interleukin-6 secretion	BP	1	C5AR2,IL17RC,F2RL1,MAVS	up_VSAP_EC
GO:0009653	0.0215	0.998	3	773	anatomical structure morphogenesis	BP	1	TGFBI,GATA5,TGFBR1	down_VSAP_VSMC
GO:0042221	0.0215	1	2	1012	response to chemical	BP	1	JUP,GATA5	down_VFEM_VSMC
GO:0048705	0.0215	0.999	2	95	skeletal system morphogenesis	BP	1	HOXA3,CYP26B1	up_VPOR_VSMC
GO:0001101	0.0215	0.999	2	65	response to acid chemical	BP	1	NR1H4,PTGER2	up_VPUL_EC
GO:0002218	0.0215	0.999	2	53	activation of innate immune response	BP	1	TLR2,CAV1	up_VSAP_VSMC
GO:0009225	0.0215	1	1	10	nucleotide-sugar metabolic process	BP	1	EXTL2	down_AFEM_EC
GO:0003170	0.0215	1	1	25	heart valve development	BP	1	GATA5	down_VSAP_VSMC
GO:0035567	0.0215	1	1	19	non-canonical Wnt signaling pathway	BP	1	WNT7A	up_AORT_VSMC
GO:0003281	0.0215	1	1	30	ventricular septum development	BP	1	TBX5	up_APUL_VSMC
GO:0045670	0.0215	1	1	16	regulation of osteoclast differentiation	BP	1	LRRC17	up_VCAV_VSMC
GO:0010831	0.0215	1	1	9	positive regulation of myotube differentiation	BP	1	CYP26B1	up_VPOR_VSMC
GO:2000050	0.0215	1	1	8	regulation of non-canonical Wnt signaling	BP	1	ANKRD6	up_VPOR_VSMC
GO:0099173	0.021511039	0.99598	4	40	postsynapse organization	BP	0.96436359	PAK3,LRP4,LRRC4,NRP1	up_ACOR_EC
GO:0060419	0.021525195	0.99724	3	22	heart growth	BP	0.96436359	FOXC2,TBX5,C23H3orf58	up_ACOR_EC
GO:0051094	0.0216	0.99	16	388	positive regulation of developmental process	BP	1	CMKLR1,IRX3,LEP,LBH,ANGPT4,DLL1,F3,GPAM,TMEM100,GREM1,CYLD,EVI2B,AXIN2,ACACB,NAPEPLD,PPARGC1A	up_AFEM_EC
GO:0043269	0.0216	0.997	3	135	regulation of ion transport	BP	1	NETO1,STIM1,CAV1	up_VSAP_VSMC
GO:0045055	0.0216	0.999	2	52	regulated exocytosis	BP	0.933	LYN,STX4	down_ACOR_EC
GO:0048799	0.0216	0.999	2	10	animal organ maturation	BP	1	XYLT1,GREM1	up_AFEM_EC
GO:0003007	0.0216	0.999	2	94	heart morphogenesis	BP	1	EYA1,SHOX2	up_VFEM_VSMC
GO:0050847	0.0216	1	1	2	progesterone receptor signaling pathway	BP	0.934	NEDD4	down_ACOR_EC
GO:0043588	0.0216	1	1	74	skin development	BP	1	JUP	down_VFEM_VSMC
GO:0031128	0.0216	1	1	15	developmental induction	BP	1	GATA5	up_VCAV_VSMC
GO:0072075	0.0216	1	1	5	metanephric mesenchyme	BP	1	SIX1	up_VFEM_EC
GO:0036445	0.0216	1	1	6	neuronal stem cell division	BP	1	ARHGEF2	up_VPUL_EC
GO:0055057	0.0216	1	1	6	neuroblast division	BP	1	ARHGEF2	up_VPUL_EC
GO:0031294	0.0216	1	1	4	lymphocyte costimulation	BP	1	CAV1	up_VSAP_VSMC
GO:0019732	0.021673752	1	1	1	antifungal humoral response	BP	0.96436359	RARRES2	up_ACOR_EC
GO:0061760	0.021673752	1	1	1	antifungal innate immune response	BP	0.96436359	RARRES2	up_ACOR_EC

GO:0023051	0.0217	0.984	110	954	regulation of signaling	BP	1	SH3TC2,ADRA1B,TLR1,SMAD6,LGR6,LEP,PLCL1,LBH,MID1,GATA5,HTR2B,RASSF2,ZRANB1,FBXL20,C28H10orf71,RECK,SKP2,NACC2,PRKAA2,MLLT3,ITM2C,FERMT1,BMP4,TM7SF3,FGFR2,ABCA1,ELF1,HRH1,CASTOR1,TLR3,SH3RF2,LATS2,PDE5A,CITED2,TP53INP1,ZNRF3,AR,PTGIR,SASH1,TMEM108,FGF18,ARHGEF26,NDRG2,CHN2,CDKN2B,ITPR1,LIMS2,ANKRD1,F2RL1,DAPK1,BNIP3,EGLN1,CRLF3,CLSTN1,CREBRF,NRP1,UBR1,PTPN22,DMPK,NPNT,UNC5B,SMPD1,APLP1,TBX20,SESN3,SH3RF1,CCDC3,PRKCE,FRS2,RNF19A,SCG5,RGS2,DAG1,PARP9,APP,PSEN1,SYNJ2BP,CHD7,SIPA1L1,CCL5,WWC2,DUSP22,SIAH1,RAB11B,EDN1,SESN1,KCNC4,DUSP26,NOD2,LRRC4,LMTK2,FAM107A,DDIT4,DISC1,SH3RF3,SERINC3,USP20,HERPUD1,MAP3K7,INPP5K,PREPL,BMT2,PFKL,WFS1,SH3BP4,ENSCAFG00000029553,PARK7,IL18R1,BVES,CRKL	up_ACOR_VSMC
GO:0022610	0.0217	0.99	14	326	biological adhesion	BP	1	LEP,CDH18,HOXA7,DLL1,GPAM,GREM1,CYLD,POSTN,LRRC15,ICAM5,BVES,ADAM17,KANK1,TNC	up_AFEM_EC
GO:0055001	0.0217	0.993	7	47	muscle cell development	BP	0.713	ACTN1,FHL2,WDR1,NEXN,SIX4,MYBPC2,HOMER1	down_VSAP_EC
GO:0055072	0.0217	0.994	7	30	iron ion homeostasis	BP	1	FXN,SLC25A28,MYC,SCARA5,FTL,HMOX1,NCOA4	up_VSAP_EC
GO:0071838	0.0217	0.999	2	3	cell proliferation in bone marrow	BP	0.633	MEF2C,MAP3K3	up_VCAV_EC
GO:1990845	0.0217	1	1	51	adaptive thermogenesis	BP	1	PLCL1	down_VFEM_EC
GO:0007501	0.0217	1	1	5	mesodermal cell fate specification	BP	1	EYA1	up_VFEM_EC
GO:0045605	0.0217	1	1	5	negative regulation of epidermal cell	BP	1	HOXA7	up_VFEM_EC
GO:0009595	0.0217	1	1	6	detection of biotic stimulus	BP	1	TLR2	up_VSAP_VSMC
GO:0001765	0.0217	1	1	4	membrane raft assembly	BP	1	CAV1	up_VSAP_VSMC
GO:0060429	0.0218	0.986	44	341	epithelium development	BP	1	HOXA11,FZD1,THRB,HOXA7,WNT11,HNF4A,AXIN2,SLIT2,FGF7,ABL1,BTG1,DLL1,OSR1,FUZ,ADAMTSL4,SAV1,KLF15,ANXA4,HPSE,RILPL2,RARG,STAT6,AMBRA1,F2RL1,DLX3,ZFP36L1,IRX3,ZEB1,MYD88,LRP4,FRMD6,E2F4,NTN4,KDM2B,NFIB,ARHGAP12,ACVRL1,MAP2K2,TGFBR2,CITED2,TRADD,LBH,KEAP1,LATS2	up_VSAP_EC
GO:0030278	0.0218	0.991	12	66	regulation of ossification	BP	1	TWIST2,AXIN2,OSR2,ZHX3,HDAC7,OSR1,ZBTB16,DDR2,OMD,LRP4,LIMD1,BGLAP	up_VSAP_EC
GO:0016192	0.0218	0.994	6	452	vesicle-mediated transport	BP	0.938	LYN,SPG11,ACSL3,STX4,MYO1D,NEDD4	down_ACOR_EC
GO:0050873	0.0218	0.995	5	18	brown fat cell differentiation	BP	1	LEP,FNDC5,PPARGC1A,PLAC8B,BNIP3	up_ACOR_VSMC
GO:0019722	0.0218	0.996	4	45	calcium-mediated signaling	BP	1	CMKLR1,LMCD1,TMEM100,DMPK	up_AFEM_EC
GO:0031214	0.0218	0.996	4	45	biomineral tissue development	BP	1	GREM1,PTHLH,CNNM4,AXIN2	up_AFEM_EC
GO:0045663	0.0218	0.998	3	7	positive regulation of myoblast differentiation	BP	1	CDON,BTG1,MAP3K5	up_VSAP_EC
GO:0045056	0.0218	0.999	2	5	transcytosis	BP	1	LRP1,LRPAP1	down_VCAV_EC
GO:0045657	0.0218	0.999	2	5	positive regulation of monocyte differentiation	BP	1	CLSTN1,ZFP36L1	down_VCAV_EC
GO:0048706	0.0218	0.999	2	59	embryonic skeletal system development	BP	1	HOXA3,HOXA1	up_APUL_EC
GO:0032990	0.0218	0.999	2	167	cell part morphogenesis	BP	1	SLITRK5,LIMK1	up_VCAV_VSMC
GO:0043616	0.0218	1	1	10	keratinocyte proliferation	BP	1	SRSF6	down_AFEM_EC
GO:2001293	0.0218	1	1	1	malonyl-CoA metabolic process	BP	1	ACACB	up_AFEM_EC
GO:2001295	0.0218	1	1	1	malonyl-CoA biosynthetic process	BP	1	ACACB	up_AFEM_EC
GO:0021615	0.0218	1	1	3	glossopharyngeal nerve morphogenesis	BP	1	HOXA3	up_VPOR_EC
GO:0003203	0.0218	1	1	11	endocardial cushion morphogenesis	BP	1	GATA5	up_VPUL_VSMC
GO:0007501	0.0218	1	1	5	mesodermal cell fate specification	BP	1	HOXA11	up_VSAP_VSMC

GO:0045595	0.0219	0.985	58	469	regulation of cell differentiation	BP	1	HOXA11,THRB,HOXA7,NAPEPLD,TWIST2,HOXA9,CDON,AXIN2,PPARGC1B,SLIT2,ZHX3,HOXD3,ABL1,BTG1,HDAC7,EMP2,DLL1,OSR1,MAP3K5,SAV1,PLXNA4,ANKRD54,ZBTB16,TOX,RARG,DDR2,ZFP36L1,PIK3R1,TIAM1,MYC,IRX3,ZEB1,SHOX2,RTN4RL1,TFE3,LRP4,DPYSL2,GDPD5,ZADH2,FBXO7,LIMD1,CDK9,CD83,FBLN1,ZFP36L2,PLEKHB2,PNP,TAF8,CYB5D2,PQBP1,TTL,ACVRL1,FLOT1,NCK1,MAP2K2,TGFBR2,LBH,KEAP1	up_VSAP_EC
GO:0006936	0.0219	0.991	13	74	muscle contraction	BP	1	TNNT2,ADRA1B,ACTN2,KCNE3,HTR2B,PDE5A,DMPK,NPNT,TBX20,JUP,EDN1,GAA,DOCK5	up_ACOR_VSMC
GO:0032963	0.0219	0.996	4	18	collagen metabolic process	BP	0.714	SERPINH1,HIF1A,PLOD3,P3H4	down_VSAP_EC
GO:0019752	0.0219	0.997	3	283	carboxylic acid metabolic process	BP	1	ETFA,DARS,SUCLG2	down_AFEM_EC
GO:0007283	0.0219	0.999	2	91	spermatogenesis	BP	1	HOXA9,HOXA11	up_VFEM_VSMC
GO:0021648	0.0219	1	1	3	vestibulocochlear nerve morphogenesis	BP	0.938	NRP2	down_ACOR_EC
GO:0003407	0.0219	1	1	15	neural retina development	BP	1	IRX5	up_AFEM_VSMC
GO:0060872	0.0219	1	1	6	semicircular canal development	BP	1	EYA1	up_VFEM_EC
GO:0071600	0.0219	1	1	5	otic vesicle morphogenesis	BP	1	EYA1	up_VFEM_EC
GO:0021515	0.0219	1	1	9	cell differentiation in spinal cord	BP	1	HOXC10	up_VFEM_VSMC
GO:0003176	0.021938141	0.99866	2	9	aortic valve development	BP	0.96436359	GATA4,SMAD6	up_ACOR_EC
GO:0071706	0.021967522	0.99587	4	40	tumor necrosis factor superfamily cytokine	BP	0.96436359	NR1H4,FZD5,NOD2,MAVS	up_ACOR_EC
GO:0006518	0.022	0.988	23	290	peptide metabolic process	BP	1	PRNP,UBA52,LARP6,COA1,SAMD4B,RPL23,RPL13,EIF3K,MRPL37,PARK7,RPL27A,EEF2,ZFP36L1,SEC11C,RNPEP,RPL27,MRPS11,RPS27,PCSK6,ENSCAFG00000015122,PYM1,FOXO3,EIF4EBP1	down_VCAV_EC
GO:0045732	0.022	0.992	10	78	positive regulation of protein catabolic process	BP	0.714	PRICKLE1,PLK3,SOCS5,TRIB3,MSN,APOE,GSK3B,DISC1,RAD23A,TMTC3	down_VSAP_EC
GO:0032635	0.022	0.993	8	37	interleukin-6 production	BP	1	C5AR2,IL6R,IL17RC,F2RL1,MYD88,NLRX1,CYBA,MAVS	up_VSAP_EC
GO:0048262	0.022	0.999	2	5	determination of dorsal/ventral asymmetry	BP	0.714	CTNNB1,DDIT3	down_VSAP_EC
GO:0030889	0.022	0.999	2	4	negative regulation of B cell proliferation	BP	0.714	LYN,BTK	down_VSAP_EC
GO:0033144	0.022	0.999	2	10	negative regulation of intracellular steroid hormone receptor	BP	1	LBH,CRY2	up_AFEM_EC
GO:0060739	0.022	1	1	1	mesenchymal-epithelial cell signaling involved in prostate gland	BP	1	TNC	up_AFEM_EC
GO:0010573	0.022	1	1	9	vascular endothelial growth factor production	BP	1	C3AR1	up_VPOR_VSMC
GO:0010574	0.022	1	1	9	regulation of vascular endothelial growth factor	BP	1	C3AR1	up_VPOR_VSMC
GO:0010658	0.022	1	1	8	striated muscle cell apoptotic process	BP	1	NKX2-5	up_VPOR_VSMC
GO:0010662	0.022	1	1	8	regulation of striated muscle cell apoptotic	BP	1	NKX2-5	up_VPOR_VSMC
GO:0006044	0.022	1	1	5	N-acetylglucosamine metabolic process	BP	1	CHST7	up_VFEM_EC
GO:1901016	0.022	1	1	9	regulation of potassium ion transmembrane transporter activity	BP	1	NETO1	up_VFEM_VSMC
GO:0034329	0.022054863	0.99477	5	60	cell junction assembly	BP	0.96436359	NR1H4,ARHGAP6,FZD5,ACTN2,NRP1	up_ACOR_EC
GO:0072073	0.022098453	0.99476	5	60	kidney epithelium development	BP	0.96436359	SOX9,AQP1,SMAD6,FOXC2,CLSTN1	up_ACOR_EC
GO:0048608	0.0221	0.989	20	129	reproductive structure development	BP	1	LEP,GATA4,PRDX4,BMP4,FGFR2,ATRX,CITED2,SPINT1,CTSB,EGLN1,ENSCAFG00000006238,SPINT2,FRS2,CHD7,SPINK2,ARID5B,DMC1,SERPINB5,LHX9,WDR48	up_ACOR_VSMC
GO:0001935	0.0221	0.993	7	50	endothelial cell proliferation	BP	0.714	VEGFA,SULF1,SPARC,JCAD,TGFBR1,ADAM17,APOE	down_VSAP_EC
GO:0072028	0.0221	0.994	6	37	nephron morphogenesis	BP	0.714	ENSCAFG00000032470,ILK,HOXB7,MAGED1,CTNNB1,SIX4	down_VSAP_EC
GO:0097306	0.0221	0.994	6	22	cellular response to alcohol	BP	1	PRKAA2,TP53INP1,ADCY6,PTGDR,PRKCE,JUP	up_ACOR_VSMC

GO:0007044	0.0221	0.995	5	27	cell-substrate junction assembly	BP	0.714	VEGFA,ACTN1,ENSCAFG00000002015,TLN1, NRP1	down_VSAP_EC
GO:0051239	0.0221	0.996	5	859	regulation of multicellular organismal process	BP	1	TBX5,GATA5,TMEM176A,TMEM176B,SLIT2	up_VPUL_VSMC
GO:0061213	0.0221	0.997	3	11	positive regulation of mesonephros	BP	0.714	HOXB7,MAGED1,SIX4	down_VSAP_EC
GO:0009888	0.0221	0.998	3	545	tissue development	BP	1	WNT7A,ARG2,ESR1	up_AORT_VSMC
GO:0009101	0.0221	0.999	2	113	glycoprotein biosynthetic process	BP	1	MGAT4B,GBGT1	down_VPOR_EC
GO:0036037	0.0221	0.999	2	3	CD8-positive, alpha-beta T cell activation	BP	1	CD274,PTPN22	up_ACOR_VSMC
GO:2001185	0.0221	0.999	2	3	regulation of CD8-positive, alpha-beta T cell	BP	1	CD274,PTPN22	up_ACOR_VSMC
GO:0071621	0.0221	0.998	2	27	granulocyte chemotaxis	BP	1	C3AR1,ENSCAFG00000032731	up_VPOR_EC
GO:0060485	0.0221	0.999	2	93	mesenchyme development	BP	1	NKX2-5,BAMBI	up_VPOR_VSMC
GO:0001822	0.0221	0.999	2	98	kidney development	BP	1	EYA1,HOXA11	up_VFEM_VSMC
GO:0021960	0.0221	0.999	2	3	anterior commissure morphogenesis	BP	1	PLXNA4,NFIB	up_VSAP_EC
GO:0001885	0.0221	1	1	19	endothelial cell development	BP	1	WNT7A	up_AORT_VSMC
GO:0031050	0.0221	1	1	17	dsRNA processing	BP	1	ESR1	up_AORT_VSMC
GO:0070918	0.0221	1	1	17	production of small RNA involved in gene silencing	BP	1	ESR1	up_AORT_VSMC
GO:0050922	0.0221	1	1	11	negative regulation of chemotaxis	BP	1	SLIT2	up_VPUL_VSMC
GO:0060039	0.0221	1	1	11	pericardium development	BP	1	TBX5	up_VPUL_VSMC
GO:0048869	0.0222	0.992	11	1119	cellular developmental process	BP	0.945	ACVRL1,LYN,ACSL4,SPG11,PDPN,NRP2,HOXA 7,ANPEP,SULF1,ELK3,NEDD4	down_ACOR_EC
GO:0045833	0.0222	0.994	6	29	negative regulation of lipid metabolic process	BP	0.645	PPP2R5A,PDGFB,BMP2,PROX1,ENSCAFG000 00010596,APOD	up_VCAV_EC
GO:0050777	0.0222	0.994	6	28	negative regulation of immune response	BP	0.644	IL7R,TNFAIP3,NLRC5,ARG2,THOC1,CNOT7	up_VCAV_EC
GO:0044271	0.0222	0.998	4	1501	cellular nitrogen compound biosynthetic	BP	1	AK5,GATA5,TGFBR1,MEIS2	down_VSAP_VSMC
GO:0006952	0.0222	0.996	4	319	defense response	BP	1	NR1H4,MRC1,CCL24,ARHGEF2	up_VPUL_EC
GO:0001765	0.0222	0.999	2	4	membrane raft assembly	BP	0.781	CAV1,EMP2	down_ACOR_VSMC
GO:0042551	0.0222	0.999	2	10	neuron maturation	BP	1	IRX5,LGI4	up_AFEM_EC
GO:0046320	0.0222	0.999	2	10	regulation of fatty acid oxidation	BP	1	ACACB,PPARGC1A	up_AFEM_EC
GO:0072678	0.0222	0.999	2	10	T cell migration	BP	1	CCL5,ADAM17	up_AFEM_EC
GO:0051346	0.0222	1	1	78	negative regulation of hydrolase activity	BP	1	CST3	down_AORT_EC
GO:0048762	0.0222	1	1	77	mesenchymal cell differentiation	BP	1	GATA5	down_VFEM_VSMC
GO:0045446	0.0222	1	1	32	endothelial cell differentiation	BP	1	HOXB5	up_APUL_VSMC
GO:0021824	0.0222	1	1	3	cerebral cortex tangential migration using cell-axon interactions	BP	1	SEMA3A	up_VPOR_EC
GO:0021828	0.0222	1	1	3	gonadotrophin-releasing hormone neuronal migration to the	BP	1	SEMA3A	up_VPOR_EC
GO:0021856	0.0222	1	1	3	hypothalamic tangential migration using cell-axon interactions	BP	1	SEMA3A	up_VPOR_EC
GO:0021886	0.0222	1	1	3	hypothalamus gonadotrophin-releasing hormone neuron	BP	1	SEMA3A	up_VPOR_EC
GO:0021888	0.0222	1	1	3	hypothalamus gonadotrophin-releasing hormone neuron	BP	1	SEMA3A	up_VPOR_EC
GO:0021979	0.0222	1	1	3	hypothalamus cell differentiation	BP	1	SEMA3A	up_VPOR_EC
GO:0035290	0.0222	1	1	3	trunk segmentation	BP	1	SEMA3A	up_VPOR_EC
GO:0036484	0.0222	1	1	3	trunk neural crest cell migration	BP	1	SEMA3A	up_VPOR_EC
GO:0036486	0.0222	1	1	3	ventral trunk neural crest cell migration	BP	1	SEMA3A	up_VPOR_EC

GO:0097490	0.0222	1	1	3	sympathetic neuron projection extension	BP	1	SEMA3A	up_VPOR_EC
GO:0097491	0.0222	1	1	3	sympathetic neuron projection guidance	BP	1	SEMA3A	up_VPOR_EC
GO:1901166	0.0222	1	1	3	neural crest cell migration involved in autonomic nervous system	BP	1	SEMA3A	up_VPOR_EC
GO:1903375	0.0222	1	1	3	facioacoustic ganglion development	BP	1	SEMA3A	up_VPOR_EC
GO:1902622	0.0222	1	1	11	regulation of neutrophil migration	BP	1	C3AR1	up_VPOR_VSMC
GO:0010611	0.0222	1	1	11	regulation of cardiac muscle hypertrophy	BP	1	GATA5	up_VPUL_VSMC
GO:0014743	0.0222	1	1	11	regulation of muscle hypertrophy	BP	1	GATA5	up_VPUL_VSMC
GO:0051240	0.0223	0.988	19	489	positive regulation of multicellular organismal process	BP	1	CMKLR1,IRX3,LEP,LBH,ANGPT4,DLL1,F3,GPAM,TMEM100,GREM1,AVPR1A,CYLD,TBXA2R,EVI2B,AXIN2,ACACB,PPARGC1A,ADAM17,AGPAT2	up_AFEM_EC
GO:0051222	0.0223	0.989	17	103	positive regulation of protein transport	BP	1	EXPH5,TLR1,LEP,HTR2B,TCAF1,MBP,FERMT1,TM7SF3,CD274,ANKRD1,F2RL1,PTPN22,PRKCE,PSEN1,JUP,NOD2,SCAMP5	up_ACOR_VSMC
GO:0072511	0.0223	0.989	16	97	divalent inorganic cation transport	BP	1	PLCG2,HTR2B,TPC3,NIPAL3,SARAF,MICU2,ITPR1,CACNB3,PRKCE,PSEN1,CHD7,INPP5K,WFS1,ATP2C1,SLC8A1,STC2	up_ACOR_VSMC
GO:0006575	0.0223	0.992	10	53	cellular modified amino acid metabolic process	BP	1	PRDX4,ERO1B,EGLN1,ENSCAFG00000028814,P4HA1,SARDH,PCYOX1,SERINC3,SERINC1,PARK7	up_ACOR_VSMC
GO:0090150	0.0223	0.992	8	64	establishment of protein localization to membrane	BP	0.717	TRAM1,SRP54,MYO1C,SSR1,SRPRA,SRP72,SH3GLB1,BLZF1	down_VSAP_EC
GO:0002063	0.0223	0.995	5	18	chondrocyte development	BP	1	AXIN2,RARG,SULF2,SHOX2,TGFBR2	up_VSAP_EC
GO:0051926	0.0223	0.996	4	14	negative regulation of calcium ion transport	BP	0.781	HOXA3,SLC30A1,GSTO1,REM1	down_ACOR_VSMC
GO:0035926	0.0223	0.999	2	3	chemokine (C-C motif) ligand 2 secretion	BP	1	MBP,F2RL1	up_ACOR_VSMC
GO:2000725	0.0223	0.999	2	10	regulation of cardiac muscle cell differentiation	BP	1	DLL1,GREM1	up_AFEM_EC
GO:0021644	0.0223	1	1	1	vagus nerve morphogenesis	BP	1	PLXNA4	up_AFEM_EC
GO:0021793	0.0223	1	1	1	chemorepulsion of branchiomotor axon	BP	1	PLXNA4	up_AFEM_EC
GO:0070228	0.0223	1	1	20	regulation of lymphocyte apoptotic process	BP	1	ARG2	up_AORT_VSMC
GO:0010960	0.0223	1	1	6	magnesium ion homeostasis	BP	1	HOXA3	up_APUL_EC
GO:0060037	0.0223	1	1	9	pharyngeal system development	BP	1	EYA1	up_VFEM_VSMC
GO:0021772	0.0223	1	1	11	olfactory bulb development	BP	1	SLIT2	up_VPUL_VSMC
GO:0021988	0.0223	1	1	11	olfactory lobe development	BP	1	SLIT2	up_VPUL_VSMC
GO:0060045	0.0223	1	1	11	positive regulation of cardiac muscle cell	BP	1	TBX5	up_VPUL_VSMC
GO:0001944	0.0224	0.99	12	263	vasculature development	BP	1	LEP,ANGPT4,HOXA7,DLL1,F3,TMEM100,GREM1,LUZP1,RECK,TBXA2R,PTPRB,CLIC4	up_AFEM_EC
GO:0035051	0.0224	0.993	8	41	cardiocyte differentiation	BP	0.649	MEF2C,EDN1,SOX18,MTOR,BMP2,PROX1,ENSCAFG00000010596,JAG1	up_VCAV_EC
GO:0002833	0.0224	0.996	4	12	positive regulation of response to biotic	BP	1	CD274,F2RL1,SASH1,CYBA	up_VSAP_EC
GO:0006357	0.0224	0.998	3	547	regulation of transcription by RNA polymerase II	BP	1	HOXA9,WNT7A,ESR1	up_AORT_VSMC
GO:0002699	0.0224	0.998	2	46	positive regulation of immune effector process	BP	0.95	LYN,STX4	down_ACOR_EC
GO:0022618	0.0224	0.999	2	111	ribonucleoprotein complex assembly	BP	1	EIF3M,SRSF6	down_AFEM_EC
GO:0035295	0.0224	0.999	2	356	tube development	BP	1	HOXA3,TBX5	up_APUL_VSMC
GO:0035136	0.0224	1	1	16	forelimb morphogenesis	BP	1	HOXA9	up_AFEM_VSMC
GO:2001014	0.0224	1	1	8	regulation of skeletal muscle cell differentiation	BP	1	CYP26B1	up_VPOR_VSMC
GO:0048535	0.0224	1	1	5	lymph node development	BP	1	TOX	up_VFEM_EC
GO:0051047	0.022401141	0.99375	6	84	positive regulation of secretion	BP	0.96436359	NR1H4,AQP1,FZD5,NOD2,PTGER4,MAVS	up_ACOR_EC
GO:0007154	0.0225	0.991	14	1622	cell communication	BP	0.95	ACVRL1,LYN,IGF1R,SPG11,PDPN,NRP2,HIVEP2,USP28,IPO7,SULF1,WDR12,STX4,ASPN,NEDD4	down_ACOR_EC

GO:0030182	0.0225	0.99	14	327	neuron differentiation	BP	1	IRX3,IRX5,HOXC10,LEP,DLL1,VXN,PLXNA4,NEFH,THRB,LGI4,CAMK2A,LRRN2,KANK1,TNC	up_AFEM_EC
GO:0055092	0.0225	0.994	6	28	sterol homeostasis	BP	0.785	SREBF2,RALY,INSIG1,CAV1,LIPC,NUS1	down_ACOR_VSMC
GO:0071772	0.0225	0.998	2	50	response to BMP	BP	0.95	ACVRL1,SULF1	down_ACOR_EC
GO:0071773	0.0225	0.998	2	50	cellular response to BMP stimulus	BP	0.95	ACVRL1,SULF1	down_ACOR_EC
GO:0010803	0.0225	0.999	2	10	regulation of tumor necrosis factor-mediated signaling pathway	BP	1	CYLD,ADAM17	up_AFEM_EC
GO:0048014	0.0225	0.999	2	3	Tie signaling pathway	BP	0.652	TEK,ANGPT2	up_VCAV_EC
GO:0007160	0.0225	0.998	2	56	cell-matrix adhesion	BP	1	HOXA7,TIAM1	up_VFEM_EC
GO:0031644	0.0225	1	1	19	regulation of neurological system process	BP	1	WNT7A	up_AORT_VSMC
GO:0060078	0.0225	1	1	17	regulation of postsynaptic membrane potential	BP	1	WNT7A	up_AORT_VSMC
GO:0099565	0.0225	1	1	17	chemical synaptic transmission, postsynaptic	BP	1	WNT7A	up_AORT_VSMC
GO:0050771	0.0225	1	1	11	negative regulation of axonogenesis	BP	1	SLIT2	up_VPUL_VSMC
GO:0034141	0.0225	1	1	5	positive regulation of toll-like receptor 3 signaling	BP	1	CAV1	up_VSAP_VSMC
GO:0061737	0.022519107	1	1	1	leukotriene signaling pathway	BP	0.96436359	RGS1	up_ACOR_EC
GO:0051932	0.022534655	0.99861	2	9	synaptic transmission, GABAergic	BP	0.96436359	CNTNAP4,PLCL1	up_ACOR_EC
GO:0006625	0.0226	0.996	4	12	protein targeting to peroxisome	BP	1	PEX7,PEX2,LONP2,PEX6	up_VSAP_EC
GO:0015919	0.0226	0.996	4	12	peroxisomal membrane transport	BP	1	PEX7,PEX2,LONP2,PEX6	up_VSAP_EC
GO:0043574	0.0226	0.996	4	12	peroxisomal transport	BP	1	PEX7,PEX2,LONP2,PEX6	up_VSAP_EC
GO:0072662	0.0226	0.996	4	12	protein localization to peroxisome	BP	1	PEX7,PEX2,LONP2,PEX6	up_VSAP_EC
GO:0072663	0.0226	0.996	4	12	establishment of protein localization to peroxisome	BP	1	PEX7,PEX2,LONP2,PEX6	up_VSAP_EC
GO:0071402	0.0226	0.997	3	10	cellular response to lipoprotein particle	BP	0.725	SOCS5,ADAM17,APOE	down_VSAP_EC
GO:0060840	0.0226	0.998	2	42	artery development	BP	0.95	ACVRL1,HOXA1	down_ACOR_EC
GO:0051660	0.0226	0.999	2	3	establishment of centrosome localization	BP	0.788	EZR,CEP83	down_ACOR_VSMC
GO:0072608	0.0226	0.999	2	3	interleukin-10 secretion	BP	1	CD274,F2RL1	up_ACOR_VSMC
GO:0006576	0.0226	0.999	2	10	cellular biogenic amine metabolic process	BP	1	MOXD1,NAPEPLD	up_AFEM_EC
GO:0007611	0.0226	0.998	2	54	learning or memory	BP	1	NETO1,GATM	up_VSAP_VSMC
GO:0043502	0.0226	1	1	16	regulation of muscle adaptation	BP	1	GATA5	up_VCAV_VSMC
GO:0060600	0.0226	1	1	3	dichotomous subdivision of an epithelial terminal	BP	1	SEMA3A	up_VPOR_EC
GO:0060666	0.0226	1	1	3	dichotomous subdivision of terminal units involved in salivary gland	BP	1	SEMA3A	up_VPOR_EC
GO:0010656	0.0226	1	1	8	negative regulation of muscle cell apoptotic	BP	1	NKX2-5	up_VPOR_VSMC
GO:2000738	0.0226	1	1	9	positive regulation of stem cell differentiation	BP	1	NKX2-5	up_VPOR_VSMC
GO:0048168	0.0226	1	1	5	regulation of neuronal synaptic plasticity	BP	1	NETO1	up_VSAP_VSMC
GO:0007423	0.022604646	0.99166	9	155	sensory organ development	BP	0.96436359	CPAMD8,TMOD1,SOX9,AQP1,FZD5,FOXC2,NRP1,PYGO2,EFEMP1	up_ACOR_EC
GO:2000778	0.0227	0.998	3	7	positive regulation of interleukin-6 secretion	BP	1	IL17RC,F2RL1,MAVS	up_VSAP_EC
GO:0048589	0.0227	0.999	2	188	developmental growth	BP	1	WNT7A,ESR1	up_AORT_VSMC
GO:0035137	0.0227	1	1	18	hindlimb morphogenesis	BP	1	WNT7A	up_AORT_VSMC
GO:0051415	0.0227	1	1	3	microtubule nucleation by interphase microtubule organizing center	BP	1	MZT1	up_VPOR_EC
GO:0051418	0.0227	1	1	3	microtubule nucleation by microtubule organizing	BP	1	MZT1	up_VPOR_EC
GO:0006110	0.0227	1	1	9	regulation of glycolytic process	BP	1	FBP1	up_VFEM_VSMC

GO:0030811	0.0227	1	1	9	regulation of nucleotide catabolic process	BP	1	FBP1	up_VFEM_VSMC
GO:0043470	0.0227	1	1	9	regulation of carbohydrate catabolic	BP	1	FBP1	up_VFEM_VSMC
GO:0099601	0.0227	1	1	10	regulation of neurotransmitter	BP	1	NETO1	up_VFEM_VSMC
GO:0032732	0.022790746	0.99858	2	9	positive regulation of interleukin-1 production	BP	0.96436359	FZD5,NOD2	up_ACOR_EC
GO:0034404	0.0228	0.993	7	50	nucleobase-containing small molecule	BP	0.729	ENPP1,PGK1,TPI1,PGAM1,HIF1A,ENTPD4,ALDOC	down_VSAP_EC
GO:1901606	0.0228	0.996	4	22	alpha-amino acid catabolic process	BP	1	THNSL2,ENSCAFG00000002977,GLS2,ACAT1	down_VCAV_EC
GO:0001523	0.0228	0.997	3	12	retinoid metabolic process	BP	1	CYP1B1,RETSAT,RDH10	down_VCAV_EC
GO:0052548	0.0228	1	1	80	regulation of endopeptidase activity	BP	1	CST3	down_AORT_EC
GO:0033059	0.0228	1	1	12	cellular pigmentation	BP	1	RAB11B	down_VPOR_EC
GO:0043276	0.0228	1	1	6	anoikis	BP	1	NTRK2	up_APUL_EC
GO:0060070	0.022870972	0.99281	7	105	canonical Wnt signaling pathway	BP	0.96436359	CDH3,SOX9,PORCN,FZD5,DIXDC1,LRP4,PYGO2	up_ACOR_EC
GO:0009653	0.0229	0.993	7	773	anatomical structure morphogenesis	BP	1	TBX5,HOXA3,ARHGEF26,SLITRK5,NPNT,NTRK2,HOXA1	up_APUL_EC
GO:1902117	0.0229	0.993	7	32	positive regulation of organelle assembly	BP	0.654	EDN1,RALB,CNOT6,PROX1,SH3GLB1,PAN3,FSCN1	up_VCAV_EC
GO:0001568	0.0229	0.997	3	252	blood vessel development	BP	1	HOXA3,NKX2-5,C3AR1	up_VPOR_VSMC
GO:0009084	0.0229	0.998	3	7	glutamine family amino acid biosynthetic process	BP	1	GLUL,ENSCAFG00000016140,ASS1	up_VSAP_EC
GO:0008356	0.0229	0.999	2	3	asymmetric cell division	BP	0.796	ASPM,ARHGEF2	down_ACOR_VSMC
GO:0034137	0.0229	0.999	2	3	positive regulation of toll-like receptor 2 signaling	BP	1	TLR1,F2RL1	up_ACOR_VSMC
GO:0045357	0.0229	0.999	2	3	regulation of interferon-beta biosynthetic process	BP	0.654	TLR4,TLR3	up_VCAV_EC
GO:0036462	0.0229	0.999	2	4	TRAIL-activated apoptotic signaling pathway	BP	0.654	ATF3,CASP8	up_VCAV_EC
GO:0045350	0.0229	0.999	2	3	interferon-beta biosynthetic process	BP	0.654	TLR4,TLR3	up_VCAV_EC
GO:0060760	0.0229	1	1	17	positive regulation of response to cytokine	BP	1	TLR2	up_AFEM_VSMC
GO:0060411	0.0229	1	1	33	cardiac septum morphogenesis	BP	1	TBX5	up_APUL_VSMC
GO:1901652	0.022940365	0.99278	7	105	response to peptide	BP	0.96436359	NR1H4,ENSCAFG00000004331,NOD2,ACTN2,FOXC2,RARRES2,PRKCD	up_ACOR_EC
GO:0014902	0.023	0.993	7	34	myotube differentiation	BP	0.654	MEF2C,PPP3CA,MTOR,CAV2,ENSCAFG00000010596,P2RX2,CDON	up_VCAV_EC
GO:0001913	0.023	0.997	3	10	T cell mediated cytotoxicity	BP	0.654	IL7R,RAB27A,NECTIN2	up_VCAV_EC
GO:0051694	0.023	0.999	2	4	pointed-end actin filament capping	BP	0.654	TMOD1,TMOD3	up_VCAV_EC
GO:0044089	0.023	0.999	2	170	positive regulation of cellular component	BP	1	SLITRK5,LIMK1	up_VCAV_VSMC
GO:0021855	0.023	1	1	4	hypothalamus cell migration	BP	0.959	NRP2	down_ACOR_EC
GO:0001659	0.023	1	1	54	temperature homeostasis	BP	1	PLCL1	down_VFEM_EC
GO:0021695	0.023	1	1	20	cerebellar cortex development	BP	1	WNT7A	up_AORT_VSMC
GO:0003197	0.023	1	1	16	endocardial cushion development	BP	1	GATA5	up_VCAV_VSMC
GO:0060677	0.023	1	1	6	ureteric bud elongation	BP	1	SIX1	up_VFEM_EC
GO:0045019	0.023	1	1	5	negative regulation of nitric oxide biosynthetic	BP	1	CAV1	up_VSAP_VSMC
GO:1901017	0.023	1	1	5	negative regulation of potassium ion transmembrane	BP	1	CAV1	up_VSAP_VSMC
GO:1901380	0.023	1	1	5	negative regulation of potassium ion	BP	1	CAV1	up_VSAP_VSMC
GO:1904406	0.023	1	1	5	negative regulation of nitric oxide metabolic	BP	1	CAV1	up_VSAP_VSMC
GO:0006541	0.023079055	0.99855	2	9	glutamine metabolic process	BP	0.96436359	NR1H4,NIT2	up_ACOR_EC
GO:0043271	0.0231	0.994	6	28	negative regulation of ion transport	BP	0.801	NEDD4,HOXA3,CAV1,SLC30A1,GSTO1,REM1	down_ACOR_VSMC
GO:0032755	0.0231	0.994	6	24	positive regulation of interleukin-6 production	BP	1	IL6R,IL17RC,F2RL1,MYD88,CYBA,MAVS	up_VSAP_EC

GO:0001974	0.0231	0.996	4	13	blood vessel remodeling	BP	0.656	MEF2C,CHD7,DLL4,JAG1	up_VCAV_EC
GO:0021615	0.0231	0.999	2	3	glossopharyngeal nerve morphogenesis	BP	1	HOXD3,PLXNA4	up_VSAP_EC
GO:0051924	0.0231	0.998	2	53	regulation of calcium ion transport	BP	1	STIM1,CAV1	up_VSAP_VSMC
GO:0021503	0.0231	1	1	1	neural fold bending	BP	1	LUZP1	up_AFEM_EC
GO:1903524	0.0231	1	1	11	positive regulation of blood circulation	BP	1	NKX2-5	up_VPOR_VSMC
GO:1905517	0.0231	1	1	10	macrophage migration	BP	1	C3AR1	up_VPOR_VSMC
GO:0010831	0.0231	1	1	9	positive regulation of myotube differentiation	BP	1	SHOX2	up_VFEM_VSMC
GO:2000738	0.023101052	0.99855	2	9	positive regulation of stem cell differentiation	BP	0.96436359	SOX9,TBX5	up_ACOR_EC
GO:0033673	0.023127166	0.9935	6	84	negative regulation of kinase activity	BP	0.96436359	PKIB,DUSP26,LRRC15,LRRC4,PRKCD,DBNDD2	up_ACOR_EC
GO:0090399	0.0232	0.999	2	4	replicative senescence	BP	0.735	SERPINE1,CDKN1A	down_VSAP_EC
GO:0048232	0.0232	0.998	2	95	male gamete generation	BP	1	HOXA9,HOXA11	up_VFEM_VSMC
GO:0045605	0.0232	1	1	5	negative regulation of epidermal cell	BP	0.966	HOXA7	down_ACOR_EC
GO:0008343	0.0232	1	1	1	adult feeding behavior	BP	1	LEP	up_AFEM_EC
GO:1904478	0.0232	1	1	1	regulation of intestinal absorption	BP	1	LEP	up_AFEM_EC
GO:1904729	0.0232	1	1	1	regulation of intestinal lipid absorption	BP	1	LEP	up_AFEM_EC
GO:0030300	0.0232	1	1	1	regulation of intestinal cholesterol absorption	BP	1	LEP	up_AFEM_EC
GO:0032817	0.0232	1	1	1	regulation of natural killer cell proliferation	BP	1	LEP	up_AFEM_EC
GO:0070093	0.0232	1	1	1	negative regulation of glucagon secretion	BP	1	LEP	up_AFEM_EC
GO:0060004	0.0232	1	1	6	reflex	BP	1	NPNT	up_APUL_EC
GO:0021756	0.0232	1	1	6	striatum development	BP	1	SLITRK5	up_APUL_EC
GO:0055083	0.0232	1	1	6	monovalent inorganic anion homeostasis	BP	1	HOXA3	up_APUL_EC
GO:0035542	0.0232	1	1	6	regulation of SNARE complex assembly	BP	1	TRIM9	up_VFEM_EC
GO:0032682	0.0232	1	1	7	negative regulation of chemokine production	BP	1	NR1H4	up_VPUL_EC
GO:0051270	0.0233	0.99	13	296	regulation of cellular component movement	BP	1	CMKLR1,ANGPT4,HOXA7,PLXNA4,TWIST2,CAMK2A,LRRC15,RECK,TBXA2R,LGR6,CCL5,ADAM17,KANK1	up_AFEM_EC
GO:0050707	0.0233	0.992	9	44	regulation of cytokine secretion	BP	1	TLR1,HTR2B,MBP,FERMT1,CD274,F2RL1,PTPN22,NOD2,SCAMP5	up_ACOR_VSMC
GO:0043619	0.0233	0.999	2	4	regulation of transcription from RNA polymerase II promoter in response to	BP	0.738	SESN2,HIF1A	down_VSAP_EC
GO:0072503	0.0233	0.998	2	97	cellular divalent inorganic cation homeostasis	BP	1	TRPC4,C3AR1	up_VPOR_VSMC
GO:1903706	0.0233	0.998	2	119	regulation of hemopoiesis	BP	1	TMEM176A,TMEM176B	up_VPUL_VSMC
GO:0042391	0.0233	1	1	81	regulation of membrane potential	BP	1	JUP	down_VFEM_VSMC
GO:0030850	0.0233	1	1	20	prostate gland development	BP	1	ESR1	up_AORT_VSMC
GO:0016486	0.0233	1	1	6	peptide hormone processing	BP	1	PCSK1N	up_APUL_EC
GO:0048843	0.0233	1	1	3	negative regulation of axon extension involved in axon guidance	BP	1	SEMA3A	up_VPOR_EC
GO:0035239	0.0234	0.987	23	299	tube morphogenesis	BP	1	HSPB6,PDGFRB,DCN,ANGPT1,SDC4,BMPR1A,LOX,LMO4,ANPEP,CLSTN1,THY1,B4GALT1,GLI3,ZFP36L1,MYDGF,SASH1,IFT57,CDH2,SPRED1,SHC1,RDH10,GREM1,CLIC4	down_VCAV_EC
GO:0044271	0.0234	0.993	8	1501	cellular nitrogen compound biosynthetic	BP	1	EYA1,HOXA10,HOXA9,TBX15,FBP1,EMX2,HOXA11,SHOX2	up_VFEM_VSMC
GO:0048871	0.0234	0.993	7	120	multicellular organismal homeostasis	BP	1	CMKLR1,HOXC10,LEP,DLL1,ACACB,NAPEPLD,PPARGC1A	up_AFEM_EC
GO:0045639	0.0234	0.995	5	33	positive regulation of myeloid cell	BP	1	CLSTN1,ZFP36L1,RAB7B,FOXO3,ANKRD54	down_VCAV_EC
GO:1903393	0.0234	0.997	3	11	positive regulation of adherens junction	BP	0.738	VEGFA,ENSCAFG00000002015,NRP1	down_VSAP_EC

GO:1903707	0.0234	0.998	2	48	negative regulation of hemopoiesis	BP	0.966	LYN,HOXA7	down_ACOR_EC
GO:0052547	0.0234	1	1	82	regulation of peptidase activity	BP	1	CST3	down_AORT_EC
GO:0072659	0.0234	1	1	82	protein localization to plasma membrane	BP	1	JUP	down_VFEM_VSMC
GO:0071307	0.0234	1	1	1	cellular response to vitamin K	BP	1	POSTN	up_AFEM_EC
GO:2000393	0.0234	1	1	1	negative regulation of lamellipodium	BP	1	KANK1	up_AFEM_EC
GO:0035624	0.0234	1	1	1	receptor transactivation	BP	1	ADAM17	up_AFEM_EC
GO:0051088	0.0234	1	1	1	PMA-inducible membrane protein ectodomain	BP	1	ADAM17	up_AFEM_EC
GO:0071403	0.0234	1	1	1	cellular response to high density lipoprotein	BP	1	ADAM17	up_AFEM_EC
GO:1901098	0.0234	1	1	1	positive regulation of autophagosome	BP	1	CLEC16A	up_AFEM_EC
GO:1904766	0.0234	1	1	1	negative regulation of macroautophagy by TORC1 signaling	BP	1	CLEC16A	up_AFEM_EC
GO:0001708	0.0234	1	1	20	cell fate specification	BP	1	HOXC10	up_AORT_VSMC
GO:0007625	0.0234	1	1	6	grooming behavior	BP	1	SLITRK5	up_APUL_EC
GO:0060385	0.0234	1	1	3	axonogenesis involved in innervation	BP	1	SEMA3A	up_VPOR_EC
GO:0010002	0.0234	1	1	9	cardioblast differentiation	BP	1	NKX2-5	up_VPOR_VSMC
GO:1901164	0.023417454	1	1	1	negative regulation of trophoblast cell migration	BP	0.96436359	ARHGDIB	up_ACOR_EC
GO:0050706	0.023461357	0.99852	2	9	regulation of interleukin-1 beta secretion	BP	0.96436359	FZD5,NOD2	up_ACOR_EC
GO:0033365	0.0235	0.986	33	276	protein localization to organelle	BP	0.814	SREBF1,RPA1,NEDD4,ZW10,CENPA,TIMM44,POLA2,CTDNEP1,RAN,INSIG1,CDK1,NF2,AURKB,EZH2,ENSCAFG00000011598,TRAM1,NDC80,IPO4,NUP62,NUP188,HASPIN,TOMM34,SAMM50,DMAP1,RAB35,EZR,TTK,KNL1,PMPCA,TINF2,CCT4,SIX4,MTBP	down_ACOR_VSMC
GO:2000026	0.0235	0.987	21	560	regulation of multicellular organismal development	BP	1	HOXA9,IRX3,LEP,LBH,ANGPT4,HOXA7,DLL1,F3,PLXNA4,THRB,LGI4,TMEM100,GREM1,CAMK2A,CYLD,PTHLH,TBXA2R,EVI2B,AXIN2,ACACB,KANK1	up_AFEM_EC
GO:0070838	0.0235	0.989	14	96	divalent metal ion transport	BP	0.663	CALCRL,PDGFB,ATP2B1,PDE4D,CHD7,STC1,APLNR,SLC39A8,PPP3CA,KCNJ2,SLC25A25,PLCG2,MCU,ATP2A1	up_VCAV_EC
GO:0097581	0.0235	0.994	6	26	lamellipodium organization	BP	0.662	KIT,MTOR,NCKAP1,SNX2,NCK1,FSCN1	up_VCAV_EC
GO:0045859	0.0235	0.996	4	219	regulation of protein kinase activity	BP	0.966	LYN,IGF1R,PRKAR2A,ASPN	down_ACOR_EC
GO:0033619	0.0235	0.996	4	19	membrane protein proteolysis	BP	0.738	MYH9,HM13,ADAM17,APOE	down_VSAP_EC
GO:0048704	0.0235	0.995	4	46	embryonic skeletal system morphogenesis	BP	1	TBX15,IRX5,HOXA7,HOXA6	up_AFEM_EC
GO:0015682	0.0235	0.998	3	7	ferric iron transport	BP	1	RAB11B,LMTK2,B2M	up_ACOR_VSMC
GO:0072512	0.0235	0.998	3	7	trivalent inorganic cation transport	BP	1	RAB11B,LMTK2,B2M	up_ACOR_VSMC
GO:0045597	0.0235	0.997	3	253	positive regulation of cell differentiation	BP	1	NKX2-5,BAMBI,CYP26B1	up_VPOR_VSMC
GO:0051252	0.0235	1	2	1051	regulation of RNA metabolic process	BP	1	JUP,GATA5	down_VFEM_VSMC
GO:1902946	0.0235	0.999	2	4	protein localization to early endosome	BP	0.738	MSN,NRP1	down_VSAP_EC
GO:2001199	0.0235	0.999	2	3	negative regulation of dendritic cell	BP	1	TMEM176B,TMEM176A	up_ACOR_VSMC
GO:0072008	0.0235	0.999	2	4	glomerular mesangial cell differentiation	BP	0.662	PDGFB,GPR4	up_VCAV_EC
GO:0072144	0.0235	0.999	2	4	glomerular mesangial cell development	BP	0.662	PDGFB,GPR4	up_VCAV_EC
GO:0070838	0.0235	0.998	2	96	divalent metal ion transport	BP	1	HOXA3,TRPC4	up_VPOR_VSMC
GO:1900151	0.0235	0.999	2	3	regulation of nuclear-transcribed mRNA catabolic process,	BP	1	ZFP36L2,TNRC6C	up_VSAP_EC
GO:1900153	0.0235	0.999	2	3	positive regulation of nuclear-transcribed mRNA catabolic process, deadenylation-dependent	BP	1	ZFP36L2,TNRC6C	up_VSAP_EC

GO:0060513	0.0235	1	1	5	prostatic bud formation	BP	0.966	SULF1	down_ACOR_EC
GO:0034330	0.0235	1	1	80	cell junction organization	BP	1	JUP	down_VFEM_VSMC
GO:0060244	0.0235	1	1	1	negative regulation of cell proliferation involved in contact inhibition	BP	1	FAP	up_AFEM_EC
GO:1902362	0.0235	1	1	1	melanocyte apoptotic process	BP	1	FAP	up_AFEM_EC
GO:0010710	0.0235	1	1	1	regulation of collagen catabolic process	BP	1	FAP	up_AFEM_EC
GO:0010716	0.0235	1	1	1	negative regulation of extracellular matrix	BP	1	FAP	up_AFEM_EC
GO:0016925	0.0235	1	1	16	protein sumoylation	BP	1	EYA1	up_AFEM_VSMC
GO:0035774	0.0235	1	1	6	positive regulation of insulin secretion involved in cellular response to	BP	1	NR1H4	up_APUL_EC
GO:0140059	0.0235	1	1	3	dendrite arborization	BP	1	SEMA3A	up_VPOR_EC
GO:0061550	0.0235	1	1	6	cranial ganglion development	BP	1	SIX1	up_VFEM_EC
GO:0071599	0.0235	1	1	9	otic vesicle development	BP	1	EYA1	up_VFEM_VSMC
GO:0003181	0.0235	1	1	7	atrioventricular valve morphogenesis	BP	1	TBX5	up_VPUL_EC
GO:0086005	0.0235	1	1	5	ventricular cardiac muscle cell action potential	BP	1	CAV1	up_VSAP_VSMC
GO:0002690	0.0236	0.997	3	26	positive regulation of leukocyte chemotaxis	BP	1	CMKLR1,CCL5,ADAM17	up_AFEM_EC
GO:2000026	0.0236	0.997	3	560	regulation of multicellular organismal development	BP	1	HOXA9,WNT7A,ESR1	up_AORT_VSMC
GO:0098780	0.0236	0.998	3	7	response to mitochondrial depolarisation	BP	1	AMBRA1,MFN2,HTRA2	up_VSAP_EC
GO:0046688	0.0236	0.999	2	5	response to copper ion	BP	1	PRNP,PARK7	down_VCAV_EC
GO:0035907	0.0236	1	1	5	dorsal aorta development	BP	0.966	ACVRL1	down_ACOR_EC
GO:0035912	0.0236	1	1	5	dorsal aorta morphogenesis	BP	0.966	ACVRL1	down_ACOR_EC
GO:0001757	0.0236	1	1	1	somite specification	BP	1	DLL1	up_AFEM_EC
GO:0002085	0.0236	1	1	1	inhibition of neuroepithelial cell	BP	1	DLL1	up_AFEM_EC
GO:1901860	0.0236	1	1	1	positive regulation of mitochondrial DNA metabolic process	BP	1	PPARGC1A	up_AFEM_EC
GO:0007386	0.0236	1	1	1	compartment pattern specification	BP	1	DLL1	up_AFEM_EC
GO:0014807	0.0236	1	1	1	regulation of somitogenesis	BP	1	DLL1	up_AFEM_EC
GO:0021688	0.0236	1	1	1	cerebellar molecular layer formation	BP	1	DLL1	up_AFEM_EC
GO:0021693	0.0236	1	1	1	cerebellar Purkinje cell layer structural	BP	1	DLL1	up_AFEM_EC
GO:0021698	0.0236	1	1	1	cerebellar cortex structural organization	BP	1	DLL1	up_AFEM_EC
GO:0045168	0.0236	1	1	1	cell-cell signaling involved in cell fate commitment	BP	1	DLL1	up_AFEM_EC
GO:0045608	0.0236	1	1	1	negative regulation of inner ear auditory receptor cell	BP	1	DLL1	up_AFEM_EC
GO:0045632	0.0236	1	1	1	negative regulation of mechanoreceptor	BP	1	DLL1	up_AFEM_EC
GO:0046331	0.0236	1	1	1	lateral inhibition	BP	1	DLL1	up_AFEM_EC
GO:0048630	0.0236	1	1	1	skeletal muscle tissue growth	BP	1	DLL1	up_AFEM_EC
GO:0048631	0.0236	1	1	1	regulation of skeletal muscle tissue growth	BP	1	DLL1	up_AFEM_EC
GO:0048633	0.0236	1	1	1	positive regulation of skeletal muscle tissue	BP	1	DLL1	up_AFEM_EC
GO:0060853	0.0236	1	1	1	Notch signaling pathway involved in arterial endothelial cell fate	BP	1	DLL1	up_AFEM_EC
GO:0097102	0.0236	1	1	1	endothelial tip cell fate specification	BP	1	DLL1	up_AFEM_EC
GO:2000981	0.0236	1	1	1	negative regulation of inner ear receptor cell	BP	1	DLL1	up_AFEM_EC

GO:0010984	0.0236	1	1	6	regulation of lipoprotein particle clearance	BP	1	NR1H4	up_APUL_EC
GO:0032692	0.0236	1	1	6	negative regulation of interleukin-1 production	BP	1	NR1H4	up_APUL_EC
GO:0055090	0.0236	1	1	6	acylglycerol homeostasis	BP	1	NR1H4	up_APUL_EC
GO:0070328	0.0236	1	1	6	triglyceride homeostasis	BP	1	NR1H4	up_APUL_EC
GO:0051496	0.0236	1	1	17	positive regulation of stress fiber assembly	BP	1	LIMK1	up_VCAV_VSMC
GO:0030217	0.0237	0.99	11	65	T cell differentiation	BP	0.665	IL7R,KIT,IL6,CHD7,DLL4,GATA3,MTOR,ITPKB, HMGB1,BMX,ZEB1	up_VCAV_EC
GO:0007166	0.0237	0.995	5	686	cell surface receptor signaling pathway	BP	1	FGF5,BARX1,NKX2-5,BAMBI,ANKRD6	up_VPOR_VSMC
GO:0002521	0.0237	0.997	3	149	leukocyte differentiation	BP	1	TOX,HOXA7,CYP26B1	up_VFEM_EC
GO:0016321	0.0237	0.999	2	3	female meiosis chromosome segregation	BP	0.814	NCAPH,TTK	down_ACOR_VSMC
GO:0097421	0.0237	0.999	2	3	liver regeneration	BP	0.814	EZH2,GLI1	down_ACOR_VSMC
GO:0043587	0.0237	0.999	2	4	tongue morphogenesis	BP	0.74	CTNNB1,SIX4	down_VSAP_EC
GO:0001921	0.0237	0.999	2	3	positive regulation of receptor recycling	BP	1	PSEN1,BVES	up_ACOR_VSMC
GO:1902259	0.0237	0.999	2	3	regulation of delayed rectifier potassium	BP	1	KCNE3,AKAP6	up_ACOR_VSMC
GO:0006120	0.0237	1	1	12	mitochondrial electron transport, NADH to	BP	1	NDUFA10	down_AFEM_EC
GO:2000008	0.0237	1	1	12	regulation of protein localization to cell surface	BP	1	RAB11B	down_VPOR_EC
GO:0006589	0.0237	1	1	1	octopamine biosynthetic process	BP	1	MOXD1	up_AFEM_EC
GO:0014722	0.0237	1	1	1	regulation of skeletal muscle contraction by calcium ion signaling	BP	1	DMPK	up_AFEM_EC
GO:0014852	0.0237	1	1	1	regulation of skeletal muscle contraction by neural stimulation via	BP	1	DMPK	up_AFEM_EC
GO:0014853	0.0237	1	1	1	regulation of excitatory postsynaptic membrane potential involved in skeletal muscle	BP	1	DMPK	up_AFEM_EC
GO:0046333	0.0237	1	1	1	octopamine metabolic process	BP	1	MOXD1	up_AFEM_EC
GO:0051823	0.0237	1	1	1	regulation of synapse structural plasticity	BP	1	DMPK	up_AFEM_EC
GO:0007095	0.0237	1	1	11	mitotic G2 DNA damage checkpoint	BP	1	TAOK1	up_AORT_EC
GO:0061180	0.0237	1	1	20	mammary gland epithelium development	BP	1	ESR1	up_AORT_VSMC
GO:0032642	0.0237	1	1	22	regulation of chemokine production	BP	1	ARG2	up_AORT_VSMC
GO:0010804	0.0237	1	1	6	negative regulation of tumor necrosis factor-mediated signaling	BP	1	NR1H4	up_APUL_EC
GO:0018195	0.0237	1	1	6	peptidyl-arginine modification	BP	1	NR1H4	up_APUL_EC
GO:0003161	0.0237	1	1	6	cardiac conduction system development	BP	1	TBX5	up_APUL_EC
GO:0002526	0.0237	1	1	17	acute inflammatory response	BP	1	C6	up_VCAV_VSMC
GO:1900120	0.0237	1	1	7	regulation of receptor binding	BP	1	HOXA3	up_VPUL_EC
GO:0035115	0.0237	1	1	12	embryonic forelimb morphogenesis	BP	1	TBX5	up_VPUL_VSMC
GO:0040008	0.0238	0.986	26	179	regulation of growth	BP	1	LEP,DCBLD2,BMP4,CRIM1,FGFR2,GJA1,LATS2 ,RERG,ATRN,PLAC8B,CDKN1A,CRLF3,NRP1,U LK2,TBX20,TBX5,APP,CHD7,WWC2,EDN1,FA M107A,IGFBP7,WFS1,NAIF1,SH3BP4,STC2	up_ACOR_VSMC
GO:0035107	0.0238	0.99	11	60	appendage morphogenesis	BP	1	HOXA11,HOXA9,OSR2,OSR1,ZBTB16,RARG,R ECK,FBXW4,SHOX2,HOXC10,LRP4	up_VSAP_EC
GO:0035108	0.0238	0.99	11	60	limb morphogenesis	BP	1	HOXA11,HOXA9,OSR2,OSR1,ZBTB16,RARG,R ECK,FBXW4,SHOX2,HOXC10,LRP4	up_VSAP_EC
GO:0035384	0.0238	0.996	4	14	thioester biosynthetic process	BP	0.814	ACLY,ELOVL1,ELOVL6,PDHA1	down_ACOR_VSMC
GO:0071616	0.0238	0.996	4	14	acyl-CoA biosynthetic process	BP	0.814	ACLY,ELOVL1,ELOVL6,PDHA1	down_ACOR_VSMC
GO:2001222	0.0238	0.996	4	11	regulation of neuron migration	BP	0.666	RELN,RAPGEF2,ARHGEF2,ZNF609	up_VCAV_EC

GO:0072359	0.0238	0.999	2	368	circulatory system development	BP	1	HOXA3,TBX5	up_APUL_VSMC
GO:0022617	0.0238	1	1	11	extracellular matrix disassembly	BP	1	FSCN1	down_AFEM_EC
GO:0002541	0.0238	1	1	1	activation of plasma proteins involved in acute inflammatory response	BP	1	F3	up_AFEM_EC
GO:0010641	0.0238	1	1	1	positive regulation of platelet-derived growth factor receptor signaling	BP	1	F3	up_AFEM_EC
GO:0006478	0.0238	1	1	1	peptidyl-tyrosine sulfation	BP	1	TPST2	up_AFEM_EC
GO:0007338	0.0238	1	1	16	single fertilization	BP	1	HOXA9	up_AFEM_VSMC
GO:0010889	0.0238	1	1	3	regulation of sequestering of triglyceride	BP	1	ABHD5	up_VPOR_EC
GO:0006654	0.0238	1	1	7	phosphatidic acid biosynthetic process	BP	1	NR1H4	up_VPUL_EC
GO:0046473	0.0238	1	1	7	phosphatidic acid metabolic process	BP	1	NR1H4	up_VPUL_EC
GO:1905314	0.0238	1	1	12	semi-lunar valve development	BP	1	GATA5	up_VPUL_VSMC
GO:0006950	0.0239	0.982	109	1102	response to stress	BP	0.666	ACKR1,EPHB1,PLK2,GBP5,SELP,KIT,LYZ,PRCP,CXCL8,F8,IFRD1,CXCR4,IL6,TNFAIP3,MAP3K11,TNIP1,LRRC8D,MX2,PDGFB,BCL6B,STK10,SH2D3C,TMEM173,RELN,VWF,CD34,MEF2C,ATF3,KLKB1,TLR4,NLRC5,GPR4,GATA3,EDN1,RALB,RAB27A,PTGS1,TFPI,PSME4,IRF8,FIGNL1,PELI1,PPP3CA,SERINC5,MTSS1,ARG2,NECTIN2,MTOR,SNAP23,MMRN1,ELF4,MAP3K3,BMP2,SLK,ABCA1,MAPK13,RIOK3,FLNB,PAK2,THOC1,TLR3,OAS3,APOD,NCK1,TIMELESS,OTUD4,PDIA5,FES,GUCY1B1,PTGS2,ETAA1,BCL10,USP28,TAOK1,CNOT7,GCH1,ENSCAFG00000002897,ASF1A,ADIPOR2,HMGB1,NOS2,PMAIP1,BLM,RIPK1,SMC5,LSM14A,USP13,RAD21,RAD17,SKIL,PDGFD,CUL4A,BMX,NR1D2,ENSCAFG00000025410,HIF1A,ALS2,ITGB3,ATRX,HP1BP3,TOPBP1,P2RX2,RBBP6,VASH1,ATP2A1,ARHGEF2,PEAR1,ELK3,GINS4	up_VCAV_EC
GO:0032434	0.0239	0.992	8	59	regulation of proteasomal ubiquitin-dependent protein catabolic process	BP	0.744	PRICKLE1,PLK3,SOCS5,TRIB3,UCHL5,GSK3B,RAD23A,USP14	down_VSAP_EC
GO:0009966	0.0239	0.993	7	868	regulation of signal transduction	BP	1	NR1H4,RGS1,CCL24,PTGER2,FLRT3,ARHGEF2,NEO1	up_VPUL_EC
GO:1904018	0.0239	0.994	5	69	positive regulation of vasculature development	BP	1	ANGPT4,DLL1,F3,TMEM100,GREM1	up_AFEM_EC
GO:0032757	0.0239	0.996	4	14	positive regulation of interleukin-8 production	BP	0.666	PRKD2,TLR3,BCL10,RIPK1	up_VCAV_EC
GO:1901575	0.0239	0.999	2	643	organic substance catabolic process	BP	1	PLCL1,USP12	down_VFEM_EC
GO:0097475	0.0239	0.999	2	4	motor neuron migration	BP	0.744	VEGFA,NRP1	down_VSAP_EC
GO:0003129	0.0239	0.999	2	3	heart induction	BP	1	GATA5,BMP4	up_ACOR_VSMC
GO:0042487	0.0239	0.999	2	3	regulation of odontogenesis of dentin-	BP	1	BMP4,TNFRSF11B	up_ACOR_VSMC
GO:0000132	0.0239	1	1	11	establishment of mitotic spindle orientation	BP	1	GPSM2	down_AFEM_EC
GO:0051294	0.0239	1	1	11	establishment of spindle orientation	BP	1	GPSM2	down_AFEM_EC
GO:0015739	0.0239	1	1	1	sialic acid transport	BP	1	SLC17A5	up_AFEM_EC
GO:0099148	0.0239	1	1	1	regulation of synaptic vesicle docking	BP	1	CAMK2A	up_AFEM_EC
GO:0106020	0.0239	1	1	1	regulation of vesicle docking	BP	1	CAMK2A	up_AFEM_EC
GO:1990443	0.0239	1	1	1	peptidyl-threonine autophosphorylation	BP	1	CAMK2A	up_AFEM_EC
GO:0007619	0.0239	1	1	1	courtship behavior	BP	1	THRB	up_AFEM_EC
GO:0007621	0.0239	1	1	1	negative regulation of female receptivity	BP	1	THRB	up_AFEM_EC
GO:0008050	0.0239	1	1	1	female courtship behavior	BP	1	THRB	up_AFEM_EC
GO:0016081	0.0239	1	1	1	synaptic vesicle docking	BP	1	CAMK2A	up_AFEM_EC
GO:0034378	0.0239	1	1	1	chylomicron assembly	BP	1	PLAGL2	up_AFEM_EC

GO:0042480	0.0239	1	1	1	negative regulation of eye photoreceptor cell	BP	1	THRB	up_AFEM_EC
GO:0045924	0.0239	1	1	1	regulation of female receptivity	BP	1	THRB	up_AFEM_EC
GO:0060040	0.0239	1	1	1	retinal bipolar neuron differentiation	BP	1	IRX5	up_AFEM_EC
GO:0060180	0.0239	1	1	1	female mating behavior	BP	1	THRB	up_AFEM_EC
GO:0097474	0.0239	1	1	1	retinal cone cell apoptotic process	BP	1	THRB	up_AFEM_EC
GO:0034121	0.0239	1	1	22	regulation of toll-like receptor signaling	BP	1	ESR1	up_AORT_VSMC
GO:1900543	0.0239	1	1	9	negative regulation of purine nucleotide	BP	1	FBP1	up_VFEM_VSMC
GO:1903579	0.0239	1	1	9	negative regulation of ATP metabolic process	BP	1	FBP1	up_VFEM_VSMC
GO:0033238	0.0239	1	1	7	regulation of cellular amine metabolic process	BP	1	NR1H4	up_VPUL_EC
GO:0060973	0.0239	1	1	7	cell migration involved in heart development	BP	1	FLRT3	up_VPUL_EC
GO:0061323	0.0239	1	1	7	cell proliferation involved in heart morphogenesis	BP	1	TBX5	up_VPUL_EC
GO:0002686	0.0239	1	1	12	negative regulation of leukocyte migration	BP	1	SLIT2	up_VPUL_VSMC
GO:0030030	0.024	0.985	33	377	cell projection organization	BP	0.744	SIPA1L1,SGK1,PTPN11,EXOC5,FSCN1,DPYSL3,IFRD1,ILK,RPGR,KIF5B,DYNLL1,TNIK,UBA6,ZPR1,NEO1,UST,CDH11,CTNNB1,ANLN,APOE,KLF5,LYN,GSK3B,VLDLR,SDC4,HTT,PRMT1,EXT1,DISC1,PAK2,NRP1,ARF4,YWHAH	down_VSAP_EC
GO:0009058	0.024	0.991	14	1910	biosynthetic process	BP	1	HOXA11,TBX15,HOXA10,HOXA9,PITX1,EMX2,GATM,SALL1,GALNT17,IRX5,PRR16,TLR2,CAV1,SHOX2	up_VSAP_VSMC
GO:0032409	0.024	0.991	10	52	regulation of transporter activity	BP	1	ACTN2,PLCG2,KCNE3,TCAF1,PTPN3,DAPK1,CACNB3,AKAP6,FXYD1,PARK7	up_ACOR_VSMC
GO:1902652	0.024	0.992	8	38	secondary alcohol metabolic process	BP	1	LIPC,INSIG2,LMF1,LCAT,MVK,LIPE,CYB5R3,SOAT1	up_VSAP_EC
GO:0060487	0.024	0.998	3	7	lung epithelial cell differentiation	BP	1	THRB,SAV1,NFIB	up_VSAP_EC
GO:0090647	0.024	0.999	2	3	modulation of age-related behavioral decline	BP	1	APP,PSEN1	up_ACOR_VSMC
GO:0071242	0.024	1	1	11	cellular response to ammonium ion	BP	1	GABPA	down_AFEM_EC
GO:0002118	0.024	1	1	1	aggressive behavior	BP	1	GCNT4	up_AFEM_EC
GO:0002121	0.024	1	1	1	inter-male aggressive behavior	BP	1	GCNT4	up_AFEM_EC
GO:0034769	0.024	1	1	1	basement membrane disassembly	BP	1	CTSS	up_AFEM_EC
GO:0060037	0.024	1	1	9	pharyngeal system development	BP	1	NKX2-5	up_VPOR_VSMC
GO:0010591	0.024	1	1	12	regulation of lamellipodium assembly	BP	1	SLIT2	up_VPUL_VSMC
GO:0048731	0.0241	0.994	7	1247	system development	BP	1	HOXC10,EYA1,HOXA9,TBX15,EMX2,HOXA11,SHOX2	up_VFEM_VSMC
GO:0055008	0.0241	0.994	6	24	cardiac muscle tissue morphogenesis	BP	1	TNNT2,FGFR2,ANKRD1,EGLN1,TBX20,CHD7	up_ACOR_VSMC
GO:0032069	0.0241	0.997	3	8	regulation of nuclease activity	BP	0.822	HMGB1,PCNA,HMGB2	down_ACOR_VSMC
GO:0001504	0.0241	0.998	3	7	neurotransmitter uptake	BP	1	PSEN1,CLN8,PARK7	up_ACOR_VSMC
GO:0072511	0.0241	0.998	2	97	divalent inorganic cation transport	BP	1	HOXA3,TRPC4	up_VPOR_VSMC
GO:0033157	0.0241	1	1	82	regulation of intracellular protein transport	BP	1	JUP	down_VFEM_VSMC
GO:0001992	0.0241	1	1	1	regulation of systemic arterial blood pressure by	BP	1	AVPR1A	up_AFEM_EC
GO:0055089	0.0241	1	1	7	fatty acid homeostasis	BP	1	NR1H4	up_VPUL_EC
GO:0060546	0.0241	1	1	7	negative regulation of necroptotic process	BP	1	ARHGEF2	up_VPUL_EC
GO:0060547	0.0241	1	1	7	negative regulation of necrotic cell death	BP	1	ARHGEF2	up_VPUL_EC
GO:2000136	0.0241	1	1	7	regulation of cell proliferation involved in	BP	1	TBX5	up_VPUL_EC
GO:0120033	0.0241	1	1	12	negative regulation of plasma membrane bounded cell projection	BP	1	SLIT2	up_VPUL_VSMC
GO:0046328	0.024157669	0.99413	5	61	regulation of JNK cascade	BP	0.96436359	FZD5,NOD2,TNFRSF19,MAPK8IP2,SH3RF3	up_ACOR_EC

GO:0015791	0.024182689	1	1	1	polyol transport	BP	0.96436359	AQP1	up_ACOR_EC
GO:0015793	0.024182689	1	1	1	glycerol transport	BP	0.96436359	AQP1	up_ACOR_EC
GO:0035377	0.024182689	1	1	1	transepithelial water transport	BP	0.96436359	AQP1	up_ACOR_EC
GO:0035378	0.024182689	1	1	1	carbon dioxide transmembrane transport	BP	0.96436359	AQP1	up_ACOR_EC
GO:0046878	0.024182689	1	1	1	positive regulation of saliva secretion	BP	0.96436359	AQP1	up_ACOR_EC
GO:0051458	0.024182689	1	1	1	corticotropin secretion	BP	0.96436359	AQP1	up_ACOR_EC
GO:0071288	0.024182689	1	1	1	cellular response to mercury ion	BP	0.96436359	AQP1	up_ACOR_EC
GO:0072019	0.024182689	1	1	1	proximal convoluted tubule development	BP	0.96436359	AQP1	up_ACOR_EC
GO:0072020	0.024182689	1	1	1	proximal straight tubule development	BP	0.96436359	AQP1	up_ACOR_EC
GO:0072022	0.024182689	1	1	1	descending thin limb development	BP	0.96436359	AQP1	up_ACOR_EC
GO:0072032	0.024182689	1	1	1	proximal convoluted tubule segment 2	BP	0.96436359	AQP1	up_ACOR_EC
GO:0072220	0.024182689	1	1	1	metanephric descending thin limb development	BP	0.96436359	AQP1	up_ACOR_EC
GO:0072229	0.024182689	1	1	1	metanephric proximal convoluted tubule	BP	0.96436359	AQP1	up_ACOR_EC
GO:0072230	0.024182689	1	1	1	metanephric proximal straight tubule	BP	0.96436359	AQP1	up_ACOR_EC
GO:0072232	0.024182689	1	1	1	metanephric proximal convoluted tubule	BP	0.96436359	AQP1	up_ACOR_EC
GO:0072236	0.024182689	1	1	1	metanephric loop of Henle development	BP	0.96436359	AQP1	up_ACOR_EC
GO:0072237	0.024182689	1	1	1	metanephric proximal tubule development	BP	0.96436359	AQP1	up_ACOR_EC
GO:0085018	0.024182689	1	1	1	maintenance of symbiont-containing vacuole by	BP	0.96436359	AQP1	up_ACOR_EC
GO:0070207	0.0242	0.997	3	7	protein homotrimerization	BP	1	SCARA5,CLYBL,HTRA2	up_VSAP_EC
GO:0090521	0.0242	0.999	2	3	glomerular visceral epithelial cell migration	BP	0.823	ANLN,NUP188	down_ACOR_VSMC
GO:0001525	0.0242	0.998	2	173	angiogenesis	BP	1	C6,TGFBI	up_VCAV_VSMC
GO:0003163	0.0242	1	1	1	sinoatrial node development	BP	1	BVES	up_AFEM_EC
GO:0060931	0.0242	1	1	1	sinoatrial node cell development	BP	1	BVES	up_AFEM_EC
GO:0007174	0.0242	1	1	1	epidermal growth factor catabolic process	BP	1	RAB7A	up_AFEM_EC
GO:0060921	0.0242	1	1	1	sinoatrial node cell differentiation	BP	1	BVES	up_AFEM_EC
GO:0051965	0.0242	1	1	17	positive regulation of synapse assembly	BP	1	SLITRK5	up_VCAV_VSMC
GO:1900122	0.0242	1	1	3	positive regulation of receptor binding	BP	1	HOXA3	up_VPOR_EC
GO:0071696	0.0242	1	1	6	ectodermal placode development	BP	1	SIX1	up_VFEM_EC
GO:0060548	0.024207215	0.98875	14	294	negative regulation of cell death	BP	0.96436359	NR1H4,SOX9,AQP1,IFI6,SMAD6,BCL2,MAPK8IP2,FOXC2,FIGNL1,CLU,CHMP4B,NRP1,PRKCD,PM20D1	up_ACOR_EC
GO:2001236	0.0243	0.992	8	59	regulation of extrinsic apoptotic signaling	BP	0.753	ARHGEF2,SERPINE1,ATF3,HTT,PAK2,INHBA,NRP1,MADD	down_VSAP_EC
GO:0045661	0.0243	0.995	5	18	regulation of myoblast differentiation	BP	1	CDON,BTG1,DLL1,MAP3K5,ZFP36L1	up_VSAP_EC
GO:0006351	0.0243	1	2	1069	transcription, DNA-templated	BP	1	JUP,GATA5	down_VFEM_VSMC
GO:0055118	0.0243	0.999	2	3	negative regulation of cardiac muscle	BP	1	KCNE3,PDE5A	up_ACOR_VSMC
GO:0001101	0.0243	0.998	2	65	response to acid chemical	BP	1	NR1H4,NTRK2	up_APUL_EC
GO:0070167	0.0243	0.998	2	29	regulation of biomineral tissue development	BP	1	ASPN,ACVR2A	up_VPOR_EC
GO:0006376	0.0243	1	1	11	mRNA splice site selection	BP	1	SRSF6	down_AFEM_EC
GO:0032596	0.0243	1	1	1	protein transport into membrane raft	BP	1	REEP2	up_AFEM_EC
GO:0050913	0.0243	1	1	1	sensory perception of bitter taste	BP	1	REEP2	up_AFEM_EC
GO:0050916	0.0243	1	1	1	sensory perception of sweet taste	BP	1	REEP2	up_AFEM_EC

GO:0051729	0.0243	1	1	1	germline cell cycle switching, mitotic to	BP	1	OVOL1	up_AFEM_EC
GO:0048874	0.0243	1	1	1	host-mediated regulation of intestinal microbiota	BP	1	NAPEPLD	up_AFEM_EC
GO:0051728	0.0243	1	1	1	cell cycle switching, mitotic to meiotic cell	BP	1	OVOL1	up_AFEM_EC
GO:0060184	0.0243	1	1	1	cell cycle switching	BP	1	OVOL1	up_AFEM_EC
GO:0070291	0.0243	1	1	1	N-acylethanolamine metabolic process	BP	1	NAPEPLD	up_AFEM_EC
GO:0070292	0.0243	1	1	1	N-acylphosphatidylethanola	BP	1	NAPEPLD	up_AFEM_EC
GO:0022603	0.0244	0.984	40	301	regulation of anatomical structure morphogenesis	BP	1	VEGFD,LEP,GATA5,CDC7,MBP,ZRANB1,MLLT3,BMP4,FGFR2,TLR3,SEC24B,WASF3,CITED2,ZNRF3,SASH1,FGF18,ZMYM2,LIMS2,MARCH5,BNIP3,EGLN1,NRP1,ULK2,TBX5,FZD3,FRS2,DAG1,PSEN1,SYNJ2BP,JUP,SIPA1L1,SSH3,EDN1,TNFRSF11B,DOCK5,ERAP1,BVES,RREB1,CRKL,PIK3C2A	up_ACOR_VSMC
GO:0051153	0.0244	0.993	6	28	regulation of striated muscle cell differentiation	BP	0.677	MEF2C,EDN1,MTOR,BMP2,PROX1,ENSCAFG00000010596	up_VCAV_EC
GO:0072604	0.0244	0.996	4	12	interleukin-6 secretion	BP	1	LEP,MBP,F2RL1,PTPN22	up_ACOR_VSMC
GO:0032467	0.0244	0.997	3	8	positive regulation of cytokinesis	BP	0.826	AURKB,CDC6,NUP62	down_ACOR_VSMC
GO:0050920	0.0244	0.998	2	53	regulation of chemotaxis	BP	0.994	LYN,STX4	down_ACOR_EC
GO:0032774	0.0244	1	2	1073	RNA biosynthetic process	BP	1	JUP,GATA5	down_VFEM_VSMC
GO:0097659	0.0244	1	2	1072	nucleic acid-templated transcription	BP	1	JUP,GATA5	down_VFEM_VSMC
GO:0036376	0.0244	1	1	1	sodium ion export across plasma membrane	BP	1	FXYD1	up_AFEM_EC
GO:1903276	0.0244	1	1	1	regulation of sodium ion export across plasma	BP	1	FXYD1	up_AFEM_EC
GO:1903278	0.0244	1	1	1	positive regulation of sodium ion export across	BP	1	FXYD1	up_AFEM_EC
GO:0120163	0.0244	1	1	17	negative regulation of cold-induced	BP	1	HOXC10	up_AFEM_VSMC
GO:0072393	0.0244	1	1	3	microtubule anchoring at microtubule organizing	BP	1	HOOK3	up_VPOR_EC
GO:0055023	0.0244	1	1	12	positive regulation of cardiac muscle tissue	BP	1	TBX5	up_VPUL_VSMC
GO:0002790	0.024431716	0.99148	8	133	peptide secretion	BP	0.96436359	NR1H4,AQP1,KCNB1,FZD5,NOD2,PTGER4,PREB,MAVS	up_ACOR_EC
GO:0023056	0.0245	0.983	57	462	positive regulation of signaling	BP	1	ADRA1B,TLR1,LGR6,LEP,MID1,GATA5,HTR2B,RASSF2,ZRANB1,C28H10orf71,SKP2,NACC2,MLLT3,ITM2C,FERMT1,BMP4,TM7SF3,FGFR2,TLR3,SH3RF2,PDE5A,CITED2,TP53INP1,SASH1,TMEM108,FGF18,CDKN2B,LIMS2,ANKRD1,F2RL1,BNIP3,CRLF3,CREBRF,NRP1,PTPN22,NPNT,UNC5B,PRKCE,FRS2,PARP9,APP,PSEN1,CCL5,DUSP22,SIAH1,EDN1,NOD2,FAM107A,DISC1,SH3RF3,SERINC3,MAP3K7,ENSCAFG00000029553,PARK7,IL18R1,BVES,CRKL	up_ACOR_VSMC
GO:0030334	0.0245	0.989	12	267	regulation of cell migration	BP	1	CMKLR1,ANGPT4,HOXA7,TWIST2,CAMK2A,LRRC15,RECK,TBXA2R,LGR6,CCL5,ADAM17,KANK1	up_AFEM_EC
GO:0032534	0.0245	0.999	2	4	regulation of microvillus assembly	BP	0.756	FSCN1,KLF5	down_VSAP_EC
GO:0007525	0.0245	1	1	1	somatic muscle development	BP	1	HSPB2	up_AFEM_EC
GO:0002158	0.0245	1	1	1	osteoclast proliferation	BP	1	GREM1	up_AFEM_EC
GO:0002174	0.0245	1	1	1	mammary stem cell proliferation	BP	1	LBH	up_AFEM_EC
GO:0038098	0.0245	1	1	1	sequestering of BMP from receptor via BMP binding	BP	1	GREM2	up_AFEM_EC
GO:0046851	0.0245	1	1	1	negative regulation of bone remodeling	BP	1	GREM1	up_AFEM_EC
GO:0060300	0.0245	1	1	1	regulation of cytokine activity	BP	1	GREM2	up_AFEM_EC
GO:0090289	0.0245	1	1	1	regulation of osteoclast proliferation	BP	1	GREM1	up_AFEM_EC
GO:0090291	0.0245	1	1	1	negative regulation of osteoclast proliferation	BP	1	GREM1	up_AFEM_EC
GO:1904674	0.0245	1	1	1	positive regulation of somatic stem cell	BP	1	LBH	up_AFEM_EC

GO:1904675	0.0245	1	1	1	regulation of somatic stem cell division	BP	1	LBH	up_AFEM_EC
GO:1904677	0.0245	1	1	1	positive regulation of somatic stem cell division	BP	1	LBH	up_AFEM_EC
GO:2000035	0.0245	1	1	1	regulation of stem cell division	BP	1	LBH	up_AFEM_EC
GO:2000101	0.0245	1	1	1	regulation of mammary stem cell proliferation	BP	1	LBH	up_AFEM_EC
GO:2000103	0.0245	1	1	1	positive regulation of mammary stem cell	BP	1	LBH	up_AFEM_EC
GO:1901099	0.0245	1	1	10	negative regulation of signal transduction in	BP	1	EYA1	up_VFEM_VSMC
GO:2001240	0.0245	1	1	10	negative regulation of extrinsic apoptotic signaling pathway in	BP	1	EYA1	up_VFEM_VSMC
GO:0002755	0.0245	1	1	6	MyD88-dependent toll-like receptor signaling	BP	1	TLR2	up_VSAP_VSMC
GO:0050770	0.0246	0.992	8	39	regulation of axonogenesis	BP	1	SLIT2,ABL1,PLXNA4,SHOX2,LRP4,DPYSL2,TTL,MAP2K2	up_VSAP_EC
GO:0032623	0.0246	0.994	5	21	interleukin-2 production	BP	0.68	TNFAIP3,PDE4D,GATA3,HOMER3,PRKD2	up_VCAV_EC
GO:0060284	0.0246	0.994	5	212	regulation of cell development	BP	1	HOOK3,ARHGEF2,ENSCAFG00000002015,SEMA3A,RNF6	up_VPOR_EC
GO:0006984	0.0246	0.996	4	19	ER-nucleus signaling pathway	BP	0.759	HSPA5,PTPN2,DDIT3,GSK3B	down_VSAP_EC
GO:0060055	0.0246	0.997	3	8	angiogenesis involved in wound healing	BP	0.829	ADIPOR2,B4GALT1,PRCP	down_ACOR_VSMC
GO:0042255	0.0246	0.998	2	29	ribosome assembly	BP	1	RSL24D1,RPL10L	up_VPOR_EC
GO:0045602	0.0246	1	1	4	negative regulation of endothelial cell	BP	1	ACVRL1	down_ACOR_EC
GO:0050794	0.0247	0.98	321	3075	regulation of cellular process	BP	1	SYNPO2L,EXPH5,SH3TC2,FGF13,RHOT1,ADRA1B,VEGFD,TMEM176B,TLR1,TMEM176A,DNAJC6,SMAD6,ATRNL1,ARHGAP28,ACTN2,LGR6,PLCG2,CASP10,IFNGR1,LEP,PLCL1,LBH,DCBLD2,MAP3K8,MID1,KCNE3,GATA5,BCL2,GATA4,ADGRD1,CD83,EFNB2,CDC7,ANGPT1,PCGF5,ENSCAFG00000016285,HTR2B,RASSF2,TCAF1,MEIS2,USP6NL,FNDC5,MBP,ZRANB1,FBXL20,C28H10orf71,PRDX4,RECK,LIPT2,FEZ1,SKP2,NACC2,PRKAA2,MLLT3,ITM2C,FERMT1,BMP4,LCAT,ZNF331,EVI5L,CRIM1,LNPK,SYNPO2,TUBB4A,TM7SF3,FGFR2,TP53BP1,ABCA1,EIF4A2,GJA1,DOCK3,ELF1,ATRX,MED14,HRH1,CASTOR1,TRPS1,TLR3,SH3RF2,SEC24B,WASF3,PPARGC1A,LATS2,PDE5A,ZNF821,CITED2,TP53INP1,ATXN2,ZNF550,RERG,GLRB,PKP4,ARHGAP29,CCNG2,ISG15,ZNRF3,ZNF782,CD274,ZNF365,PLPP2,MARCKSL1,AR,PTGIR,ZNF484,SASH1,GPR155,LMO4,GTF3C1,PTPN3,TMEM108,ZNF572,FGF18,FAM210B,ARHGEF26,NDRG2,P4HTM,TMX3,CHN2,CDKN2B,ADCY6,ZMYM2,ITPR1,GRK7,CST3,ZNF174,PLAC8B,KLHL20,LIMS2,BLNK,ANKRD1,PLEKHB2,F2RL1,ADGRL1,FAM129A,CAMK2D,MARCH5,PSEN2,PPP2R5E,ERO1B,DAPK1,PKNOX2,ARHGAP5,RBM4B,HKR1,BNIP3,EGLN1,NCEH1,AKAP6,CDK	up_ACOR_VSMC
GO:0006364	0.0247	0.989	13	87	rRNA processing	BP	0.831	NOP56,FBL,DIS3,NOC4L,RSL1D1,RRP1,ENSCAFG00000019620,NOP14,NOP58,ENSCAFG00000015189,EMG1,EXOSC5,NOB1	down_ACOR_VSMC
GO:0019395	0.0247	0.994	5	33	fatty acid oxidation	BP	1	AKT2,ACAA1,PEX5,LONP2,ACAT1	down_VCAV_EC
GO:0050709	0.0247	0.994	5	28	negative regulation of protein secretion	BP	0.761	NR1H4,PTPN11,REST,RAB11FIP1,RHBDF2	down_VSAP_EC
GO:0030154	0.0247	0.997	4	1040	cell differentiation	BP	1	HOXC10,HOXA9,WNT7A,ESR1	up_AORT_VSMC
GO:1903729	0.0247	0.997	3	7	regulation of plasma membrane organization	BP	1	AR,ASAP1,WASL	up_ACOR_VSMC
GO:0019400	0.0247	0.997	3	7	alditol metabolic process	BP	1	GOT1,GALK1,TKFC	up_VSAP_EC
GO:0002703	0.0247	0.998	2	47	regulation of leukocyte mediated immunity	BP	1	LYN,STX4	down_ACOR_EC
GO:1902991	0.0247	0.999	2	5	regulation of amyloid precursor protein	BP	0.761	APOE,LYN	down_VSAP_EC
GO:0007517	0.0247	0.998	2	100	muscle organ development	BP	1	NKX2-5,CYP26B1	up_VPOR_VSMC
GO:1902459	0.0247	0.999	2	3	positive regulation of stem cell population	BP	1	KDM2B,LBH	up_VSAP_EC
GO:0072006	0.0247	0.998	2	59	nephron development	BP	1	HOXA11,SALL1	up_VSAP_VSMC

GO:0048048	0.0247	1	1	13	embryonic eye morphogenesis	BP	1	CITED2	down_VPOR_EC
GO:0043542	0.0247	1	1	86	endothelial cell migration	BP	1	JUP	down_VFEM_VSMC
GO:0001657	0.024749809	0.99516	4	42	ureteric bud development	BP	0.96436359	SOX9,SMAD6,FOXC2,CLSTN1	up_ACOR_EC
GO:0072163	0.024749809	0.99516	4	42	mesonephric epithelium development	BP	0.96436359	SOX9,SMAD6,FOXC2,CLSTN1	up_ACOR_EC
GO:0072164	0.024749809	0.99516	4	42	mesonephric tubule development	BP	0.96436359	SOX9,SMAD6,FOXC2,CLSTN1	up_ACOR_EC
GO:0072111	0.0248	0.997	3	9	cell proliferation involved in kidney development	BP	0.682	PDGFB,GATA3,BMP2	up_VCAV_EC
GO:0030730	0.0248	0.999	2	4	sequestering of triglyceride	BP	0.761	ENPP1,ABHD5	down_VSAP_EC
GO:0043583	0.0248	0.998	2	59	ear development	BP	1	EYA1,SIX1	up_VFEM_EC
GO:0010591	0.0248	1	1	12	regulation of lamellipodium assembly	BP	1	FSCN1	down_AFEM_EC
GO:0030336	0.0248	1	1	86	negative regulation of cell migration	BP	1	JUP	down_VFEM_VSMC
GO:0032612	0.0248	1	1	22	interleukin-1 production	BP	1	ARG2	up_AORT_VSMC
GO:0097061	0.0248	1	1	21	dendritic spine organization	BP	1	WNT7A	up_AORT_VSMC
GO:0032602	0.0248	1	1	23	chemokine production	BP	1	ARG2	up_AORT_VSMC
GO:0051047	0.0249	0.989	14	84	positive regulation of secretion	BP	1	EXPH5,TLR1,HTR2B,MBP,FERMT1,TM7SF3,CD274,ANKRD1,F2RL1,PTPN22,PRKCE,EDN1,NOD2,SCAMP5	up_ACOR_VSMC
GO:0030308	0.0249	0.992	7	49	negative regulation of cell growth	BP	0.761	ENPP1,IFRD1,SMARCA4,SESN2,INHBA,CDKN1A,NRP1	down_VSAP_EC
GO:0051145	0.0249	0.994	5	21	smooth muscle cell differentiation	BP	0.685	EDNRB,KIT,PDGFB,MEF2C,ZEB1	up_VCAV_EC
GO:0072606	0.0249	0.996	4	12	interleukin-8 secretion	BP	1	TLR1,LEP,F2RL1,PTPN22	up_ACOR_VSMC
GO:0098660	0.0249	0.997	3	141	inorganic ion transmembrane transport	BP	1	NETO1,STIM1,CAV1	up_VSAP_VSMC
GO:0071826	0.0249	0.998	2	117	ribonucleoprotein complex subunit	BP	1	EIF3M,SRSF6	down_AFEM_EC
GO:0001501	0.0249	0.998	2	178	skeletal system development	BP	1	HOXC6,LRRC17	up_VCAV_VSMC
GO:0048144	0.0249	1	1	23	fibroblast proliferation	BP	1	ESR1	up_AORT_VSMC
GO:0048145	0.0249	1	1	23	regulation of fibroblast proliferation	BP	1	ESR1	up_AORT_VSMC
GO:0043276	0.0249	1	1	6	anoikis	BP	1	CAV1	up_VSAP_VSMC
GO:0015865	0.024950347	1	1	1	purine nucleotide transport	BP	0.96436359	SLC25A42	up_ACOR_EC
GO:0015866	0.024950347	1	1	1	ADP transport	BP	0.96436359	SLC25A42	up_ACOR_EC
GO:0015868	0.024950347	1	1	1	purine ribonucleotide transport	BP	0.96436359	SLC25A42	up_ACOR_EC
GO:0015880	0.024950347	1	1	1	coenzyme A transport	BP	0.96436359	SLC25A42	up_ACOR_EC
GO:0035349	0.024950347	1	1	1	coenzyme A transmembrane transport	BP	0.96436359	SLC25A42	up_ACOR_EC
GO:0051503	0.024950347	1	1	1	adenine nucleotide transport	BP	0.96436359	SLC25A42	up_ACOR_EC
GO:0071106	0.024950347	1	1	1	adenosine 3',5'-bisphosphate	BP	0.96436359	SLC25A42	up_ACOR_EC
GO:0072530	0.024950347	1	1	1	purine-containing compound	BP	0.96436359	SLC25A42	up_ACOR_EC
GO:0006915	0.025	0.984	44	550	apoptotic process	BP	0.763	ARHGEF2,VEGFA,P4HB,ASNS,NR1H4,PLK3,HSPA5,SERPINE1,ETV6,ERO1A,CASP3,HYOU1,EXOC5,AARS,FHL2,PTGS2,UACA,HIF1A,DNAJC10,TNIK,ZPR1,PTPN2,MTDH,MAGED1,CTNNB1,CRYAB,LYN,BNIP3,DDIT3,FAM162A,ATF3,GSK3B,SH3GLB1,HTT,REST,GLO1,PAK2,INHBA,SIX4,CDKN1A,NRP1,SPINK2,ARF4,MADD	down_VSAP_EC
GO:0048644	0.025	0.993	7	31	muscle organ morphogenesis	BP	1	TNNT2,FGFR2,ANKRD1,EGLN1,TBX20,CHD7,ARID5B	up_ACOR_VSMC
GO:0090101	0.025	0.994	5	34	negative regulation of transmembrane receptor protein serine/threonine kinase signaling pathway	BP	1	VASN,FBN1,CHRDL1,ASPN,GREM1	down_VCAV_EC
GO:0045595	0.025	0.997	3	469	regulation of cell differentiation	BP	1	LIMK1,GATA5,LRRC17	up_VCAV_VSMC

GO:0048536	0.025	1	1	12	spleen development	BP	1	CITED2	down_VPOR_EC
GO:0071380	0.025	1	1	7	cellular response to prostaglandin E stimulus	BP	1	PTGER2	up_VPUL_EC
GO:0048665	0.025	1	1	6	neuron fate specification	BP	1	HOXC10	up_VSAP_VSMC
GO:0032092	0.0251	0.992	8	38	positive regulation of protein binding	BP	1	PLCL1,BMP4,APP,PSEN1,AKTIP,B2M,HERPUD1,PARK7	up_ACOR_VSMC
GO:0007519	0.0251	0.992	7	49	skeletal muscle tissue development	BP	0.763	ACTN1,FLNB,CTNNB1,KLF5,ATF3,SIX4,HOMER1	down_VSAP_EC
GO:0042572	0.0251	0.999	2	5	retinol metabolic process	BP	1	CYP1B1,RETSAT	down_VCAV_EC
GO:0001696	0.0251	0.999	2	3	gastric acid secretion	BP	1	SLC26A7,HRH2	up_ACOR_VSMC
GO:0034612	0.0251	0.998	2	63	response to tumor necrosis factor	BP	1	NR1H4,NPNT	up_APUL_EC
GO:0090385	0.0251	1	1	4	phagosome-lysosome fusion	BP	1	SPG11	down_ACOR_EC
GO:0007565	0.0251	1	1	23	female pregnancy	BP	1	ESR1	up_AORT_VSMC
GO:0044314	0.0251	1	1	3	protein K27-linked ubiquitination	BP	1	RNF6	up_VPOR_EC
GO:0090102	0.0251	1	1	10	cochlea development	BP	1	EYA1	up_VFEM_VSMC
GO:0048856	0.0252	0.992	10	1533	anatomical structure development	BP	1	TBX5,HOXA3,NR1H4,EVI2B,HOXA2,MYBPC2,CCL24,FLRT3,ARHGEF2,NEO1	up_VPUL_EC
GO:0048584	0.0252	0.997	3	592	positive regulation of response to stimulus	BP	1	WNT7A,ARG2,ESR1	up_AORT_VSMC
GO:0046085	0.0252	0.999	2	5	adenosine metabolic process	BP	1	NT5C2,ADK	down_VCAV_EC
GO:0019336	0.0252	0.999	2	3	phenol-containing compound catabolic	BP	1	MAOA,MOXD1	up_ACOR_VSMC
GO:0019614	0.0252	0.999	2	3	catechol-containing compound catabolic	BP	1	MAOA,MOXD1	up_ACOR_VSMC
GO:0042420	0.0252	0.999	2	3	dopamine catabolic process	BP	1	MAOA,MOXD1	up_ACOR_VSMC
GO:0042424	0.0252	0.999	2	3	catecholamine catabolic process	BP	1	MAOA,MOXD1	up_ACOR_VSMC
GO:1900122	0.0252	0.999	2	3	positive regulation of receptor binding	BP	1	PLCL1,B2M	up_ACOR_VSMC
GO:0000904	0.0252	0.998	2	181	cell morphogenesis involved in differentiation	BP	1	SLITRK5,LIMK1	up_VCAV_VSMC
GO:0061387	0.0252	1	1	18	regulation of extent of cell growth	BP	1	LIMK1	up_VCAV_VSMC
GO:0019098	0.0252	1	1	6	reproductive behavior	BP	1	AVPR1A	up_VFEM_EC
GO:0071622	0.0252	1	1	13	regulation of granulocyte chemotaxis	BP	1	SLIT2	up_VPUL_VSMC
GO:0019058	0.025240474	0.99379	5	64	viral life cycle	BP	0.96436359	ATG16L2,TRIM8,MAVS,LRRC15,CHMP4B	up_ACOR_EC
GO:0034330	0.0253	0.99	10	80	cell junction organization	BP	0.766	VEGFA,NR1H4,ACTN1,FSCN1,WDR1,MYO1C,CTNNB1,ENSCAFG00000002015,TLN1,NRP1	down_VSAP_EC
GO:0045844	0.0253	0.993	6	24	positive regulation of striated muscle tissue	BP	1	BMP4,FGFR2,GJA1,TBX20,TBX5,EDN1	up_ACOR_VSMC
GO:0048636	0.0253	0.993	6	24	positive regulation of muscle organ	BP	1	BMP4,FGFR2,GJA1,TBX20,TBX5,EDN1	up_ACOR_VSMC
GO:0048705	0.0253	0.993	6	95	skeletal system morphogenesis	BP	1	TBX15,IRX5,HOXA7,GREM1,HOXA6,AXIN2	up_AFEM_EC
GO:0051639	0.0253	0.999	2	4	actin filament network formation	BP	0.766	ACTN1,PLS3	down_VSAP_EC
GO:1901985	0.0253	1	1	11	positive regulation of protein acetylation	BP	1	TAOK1	up_AORT_EC
GO:0014896	0.0253	1	1	18	muscle hypertrophy	BP	1	GATA5	up_VCAV_VSMC
GO:0014897	0.0253	1	1	18	striated muscle hypertrophy	BP	1	GATA5	up_VCAV_VSMC
GO:0003300	0.0253	1	1	18	cardiac muscle hypertrophy	BP	1	GATA5	up_VCAV_VSMC
GO:0003215	0.0253	1	1	9	cardiac right ventricle morphogenesis	BP	1	NKX2-5	up_VPOR_VSMC
GO:0001990	0.0253	1	1	6	regulation of systemic arterial blood pressure by	BP	1	AVPR1A	up_VFEM_EC
GO:1905114	0.0254	0.991	8	150	cell surface receptor signaling pathway involved in cell-cell	BP	1	GREM1,FAM53B,CYLD,RECK,LGR6,AXIN2,DMPK,KANK1	up_AFEM_EC
GO:0009611	0.0254	0.992	7	122	response to wounding	BP	1	F5,F3,TFPI2,LGR6,ADAM17,KANK1,TNC	up_AFEM_EC
GO:0033993	0.0254	0.997	3	186	response to lipid	BP	1	NR1H4,MRC1,PTGER2	up_VPUL_EC

GO:0071472	0.0254	0.999	2	3	cellular response to salt stress	BP	1	ZFP36L1,SLC12A6	up_VSAP_EC
GO:0034143	0.0254	1	1	6	regulation of toll-like receptor 4 signaling	BP	1	LYN	down_ACOR_EC
GO:0046928	0.0254	1	1	23	regulation of neurotransmitter	BP	1	WNT7A	up_AORT_VSMC
GO:0035270	0.0254	1	1	35	endocrine system development	BP	1	HOXA3	up_APUL_VSMC
GO:0006821	0.0254	1	1	18	chloride transport	BP	1	CA2	up_VCAV_VSMC
GO:0033561	0.0254	1	1	6	regulation of water loss via skin	BP	1	CYP26B1	up_VFEM_EC
GO:0072358	0.0255	0.989	12	268	cardiovascular system development	BP	1	LEP,ANGPT4,HOXA7,DLL1,F3,TMEM100,GREM1,LUZP1,RECK,TBXA2R,PTPRB,CLIC4	up_AFEM_EC
GO:0023061	0.0255	0.993	6	95	signal release	BP	1	LEP,KCNC4,CRY2,CHRM2,CAMK2A,CCL5	up_AFEM_EC
GO:0032642	0.0255	0.994	5	22	regulation of chemokine production	BP	0.699	ACKR1,TLR4,ARG2,TLR3,APOD	up_VCAV_EC
GO:0072350	0.0255	0.994	5	18	tricarboxylic acid metabolic process	BP	1	DLST,IDH1,IDH3A,ENSCAFG00000006639,ASS1	up_VSAP_EC
GO:0048729	0.0255	0.995	4	209	tissue morphogenesis	BP	1	ACVRL1,PDPN,NRP2,SULF1	down_ACOR_EC
GO:0016049	0.0255	0.996	3	124	cell growth	BP	1	ACVRL1,SPG11,NRP2	down_ACOR_EC
GO:0008219	0.0255	0.997	3	611	cell death	BP	1	WNT7A,ARG2,ESR1	up_AORT_VSMC
GO:0032504	0.0255	0.996	3	152	multicellular organism reproduction	BP	1	HOXA9,AVPR1A,CYP26B1	up_VFEM_EC
GO:0048609	0.0255	0.996	3	152	multicellular organismal reproductive process	BP	1	HOXA9,AVPR1A,CYP26B1	up_VFEM_EC
GO:0098655	0.0255	0.996	3	142	cation transmembrane transport	BP	1	NETO1,STIM1,CAV1	up_VSAP_VSMC
GO:0043409	0.0255	0.998	2	54	negative regulation of MAPK cascade	BP	1	LYN,IGF1R	down_ACOR_EC
GO:0003006	0.0255	0.998	2	180	developmental process involved in reproduction	BP	1	HOXA9,IRX5	up_AFEM_VSMC
GO:0032940	0.0255	0.998	2	231	secretion by cell	BP	1	WNT7A,ARG2	up_AORT_VSMC
GO:0051091	0.0255	1	1	87	positive regulation of DNA-binding transcription factor activity	BP	1	JUP	down_VFEM_VSMC
GO:0046889	0.025542109	0.99648	3	24	positive regulation of lipid biosynthetic process	BP	0.96436359	NR1H4,CCDC3,PRKCD	up_ACOR_EC
GO:0010970	0.0256	0.992	7	48	transport along microtubule	BP	0.767	RPGR,KIF5B,HIF1A,DYNLL1,TRAK1,HTT,AP3S1	down_VSAP_EC
GO:0099111	0.0256	0.992	7	48	microtubule-based transport	BP	0.767	RPGR,KIF5B,HIF1A,DYNLL1,TRAK1,HTT,AP3S1	down_VSAP_EC
GO:0010827	0.0256	0.994	5	28	regulation of glucose transmembrane transport	BP	0.767	NR1H4,ENPP1,PTPN11,TRIB3,YES1	down_VSAP_EC
GO:0035249	0.0256	0.996	4	13	synaptic transmission, glutamatergic	BP	0.7	RELN,MEF2C,GRM8,ALS2	up_VCAV_EC
GO:0001842	0.0256	0.999	2	3	neural fold formation	BP	0.7	HIF1A,LUZP1	up_VCAV_EC
GO:0048562	0.0256	0.998	2	102	embryonic organ morphogenesis	BP	1	HOXA3,NKX2-5	up_VPOR_VSMC
GO:0042044	0.0256	1	1	6	fluid transport	BP	1	PDPN	down_ACOR_EC
GO:0006040	0.0256	1	1	12	amino sugar metabolic process	BP	1	EXTL2	down_AFEM_EC
GO:0060972	0.0256	1	1	13	left/right pattern formation	BP	1	CITED2	down_VPOR_EC
GO:0032715	0.0256	1	1	11	negative regulation of interleukin-6 production	BP	1	BPI	up_AORT_EC
GO:0045747	0.0256	1	1	18	positive regulation of Notch signaling pathway	BP	1	GATA5	up_VCAV_VSMC
GO:0030878	0.0256	1	1	13	thyroid gland development	BP	1	HOXA3	up_VPUL_VSMC
GO:0007595	0.0256	1	1	6	lactation	BP	1	CAV1	up_VSAP_VSMC

GO:0048519	0.0257	0.981	111	1554	negative regulation of biological process	BP	0.767	ARHGEF2,PRICKLE1,VEGFA,SULF1,ASNS,MYH9,NR1H4,PLK3,HSPA5,SERPINE1,ACTN1,SOCS5,ETV6,SPARC,ERO1A,PRKACB,PTPN14,ENPP1,ERRFI1,SNX25,CBX5,HYOU1,PGK1,PTPN11,AARS,DPYSL3,HOOK3,TRIB3,IFRD1,ILK,FHL2,BHLHE40,SMARCA4,SESN2,PTGS2,REM1,CHSY1,HIF1A,DYNLL1,THBS2,DNAJC10,NAA10,NXN,RGS2,ZPR1,PTPN2,UCHL5,MORF4L2,SPOPL,PLCL1,PEG3,SMG7,MTDH,DDX39B,ADAM17,EIF4EBP1,SKAP2,CNOT7,MAGED1,CTNNB1,CRYAB,STARD13,SND1,ENSCAFG00000002015,APOE,WWC3,KLF5,MMP28,LYN,BNIP3,STRAP,DDIT3,TWF1,PDS5B,CBLC,ATF3,GSK3B,VLDLR,TAX1BP3,SDC4,HTT,REST,ABHD5,ENSCAFG00000017326,ZMYND8,EIF2S1,PRMT1,ARFGEF3,CALM1,RAB11FIP1,GLO1,CDYL,PAK2,LRRTM2,A2M,CAPG,INHBA,NR4A1,HIF1AN,BTK,SIX4,CDKN1A,NRP1,SPINK2,RHBDF2,USP14,ARF4,YWHAH,TNFAIP1,KDM3A,MID1	down_VSAP_EC
GO:0001568	0.0257	0.986	20	252	blood vessel development	BP	1	HSPB6,PDGFRB,DCN,ANGPT1,BMPR1A,LOX,RECK,ANPEP,SEMA3C,THY1,B4GALT1,GLI3,ZFP36L1,MYDGF,SASH1,CDH2,SPRED1,SHC1,GREM1,CLIC4	down_VCAV_EC
GO:0051403	0.0257	0.988	15	89	stress-activated MAPK cascade	BP	1	LEP,MID1,RASSF2,TLR3,SH3RF2,SASH1,F2RL1,PTPN22,SH3RF1,APP,DUSP22,EDN1,NOD2,SH3RF3,MAP3K7	up_ACOR_VSMC
GO:0051146	0.0257	0.989	12	76	striated muscle cell differentiation	BP	0.7	MEF2C,EDN1,TMOD1,PPP3CA,MTOR,BMP2,CAV2,PROX1,ENSCAFG00000010596,P2RX2,TMOD3,CDON	up_VCAV_EC
GO:0010721	0.0257	0.991	8	73	negative regulation of cell development	BP	1	FBN1,BMPR1A,THY1,TLX2,GLI3,PRKCSH,THRB,RGMA	down_VCAV_EC
GO:0051241	0.0257	0.994	5	336	negative regulation of multicellular organismal	BP	1	ACVRL1,LYN,HOXA7,SULF1,ASPN	down_ACOR_EC
GO:0090049	0.0257	0.994	5	21	regulation of cell migration involved in sprouting angiogenesis	BP	0.701	PLK2,DLL4,PIK3C2A,MAP3K3,JCAD	up_VCAV_EC
GO:0055023	0.0257	0.996	4	12	positive regulation of cardiac muscle tissue	BP	1	FGFR2,TBX20,TBX5,EDN1	up_ACOR_VSMC
GO:0001944	0.0257	0.997	3	263	vasculature development	BP	1	HOXA3,NKX2-5,C3AR1	up_VPOR_VSMC
GO:0035089	0.0257	0.999	2	5	establishment of apical/basal cell polarity	BP	0.767	FSCN1,MSN	down_VSAP_EC
GO:0043302	0.0257	1	1	6	positive regulation of leukocyte degranulation	BP	1	STX4	down_ACOR_EC
GO:0044539	0.0257	1	1	5	long-chain fatty acid import	BP	1	ACSL3	down_ACOR_EC
GO:0005984	0.0257	1	1	3	disaccharide metabolic process	BP	1	FBP1	up_VPOR_EC
GO:0046351	0.0257	1	1	3	disaccharide biosynthetic process	BP	1	FBP1	up_VPOR_EC
GO:0008542	0.0257	1	1	13	visual learning	BP	1	LRRN4	up_VPUL_VSMC
GO:0071675	0.0257	1	1	13	regulation of mononuclear cell	BP	1	SLIT2	up_VPUL_VSMC
GO:0021871	0.0257	1	1	6	forebrain regionalization	BP	1	EMX2	up_VSAP_VSMC
GO:0072511	0.0258	0.988	14	97	divalent inorganic cation transport	BP	0.701	CALCRL,PDGFB,ATP2B1,PDE4D,CHD7,STC1,APLNR,SLC39A8,PPP3CA,KCNJ2,SLC25A25,PLCG2,MCU,ATP2A1	up_VCAV_EC
GO:0032413	0.0258	0.995	4	15	negative regulation of ion transmembrane transporter activity	BP	0.863	NEDD4,CAV1,GSTO1,REM1	down_ACOR_VSMC
GO:0060544	0.0258	0.997	3	8	regulation of necroptotic process	BP	0.701	PELI1,RIPK1,ARHGEF2	up_VCAV_EC
GO:0048729	0.0258	0.998	2	209	tissue morphogenesis	BP	1	WNT7A,ESR1	up_AORT_VSMC
GO:0043270	0.0258	0.998	2	56	positive regulation of ion transport	BP	1	STIM1,CAV1	up_VSAP_VSMC
GO:0035088	0.0258	1	1	12	establishment or maintenance of	BP	1	FSCN1	down_AFEM_EC
GO:0061245	0.0258	1	1	12	establishment or maintenance of bipolar	BP	1	FSCN1	down_AFEM_EC
GO:0031111	0.0258	1	1	11	negative regulation of microtubule polymerization or	BP	1	TAOK1	up_AORT_EC
GO:0060017	0.0258	1	1	3	parathyroid gland development	BP	1	HOXA3	up_VPOR_EC
GO:0001523	0.0258	1	1	12	retinoid metabolic process	BP	1	CYP26B1	up_VPOR_VSMC

GO:0071622	0.0258	1	1	13	regulation of granulocyte chemotaxis	BP	1	C3AR1	up_VPOR_VSMC
GO:0060914	0.0258	1	1	11	heart formation	BP	1	EYA1	up_VFEM_VSMC
GO:0001759	0.0258	1	1	13	organ induction	BP	1	GATA5	up_VPUL_VSMC
GO:0031345	0.0259	0.993	6	46	negative regulation of cell projection organization	BP	1	PRNP,THY1,TLX2,PRKCSH,EVI5L,RGMA	down_VCAV_EC
GO:0099003	0.0259	0.998	2	50	vesicle-mediated transport in synapse	BP	1	SPG11,STX4	down_ACOR_EC
GO:0021562	0.0259	1	1	4	vestibulocochlear nerve development	BP	1	NRP2	down_ACOR_EC
GO:1990778	0.0259	1	1	91	protein localization to cell periphery	BP	1	JUP	down_VFEM_VSMC
GO:0060561	0.0259	1	1	11	apoptotic process involved in	BP	1	NKX2-5	up_VPOR_VSMC
GO:0045843	0.0259	1	1	13	negative regulation of striated muscle tissue	BP	1	TBX5	up_VPUL_VSMC
GO:1905209	0.0259	1	1	13	positive regulation of cardiocyte differentiation	BP	1	TBX5	up_VPUL_VSMC
GO:0034333	0.025920972	0.99639	3	25	adherens junction assembly	BP	0.96436359	ARHGAP6,ACTN2,NRP1	up_ACOR_EC
GO:0035262	0.025922746	1	1	1	gonad morphogenesis	BP	0.96436359	LHX9	up_ACOR_EC
GO:0090360	0.025937111	1	1	1	platelet-derived growth factor production	BP	0.96436359	NDRG2	up_ACOR_EC
GO:0090361	0.025937111	1	1	1	regulation of platelet-derived growth factor	BP	0.96436359	NDRG2	up_ACOR_EC
GO:0061458	0.026	0.986	20	131	reproductive system development	BP	1	LEP,GATA4,PRDX4,BMP4,FGFR2,ATRX,CITED2,SPINT1,CTSB,EGLN1,ENSCAFG00000006238,SPINT2,FRS2,CHD7,SPINK2,ARID5B,DMC1,SERPINB5,LHX9,WDR48	up_ACOR_VSMC
GO:0048705	0.026	0.988	14	95	skeletal system morphogenesis	BP	0.87	EIF4A3,HOXA3,NAB2,INSIG1,MMP14,GREM1,HOXA7,HOXB7,POC1A,HOXB5,SGPL1,TBX15,SERPINH1,SIX4	down_ACOR_VSMC
GO:0061028	0.026	0.995	4	14	establishment of endothelial barrier	BP	0.703	PDE4D,RAPGEF1,SOX18,RAPGEF2	up_VCAV_EC
GO:0007389	0.026	0.995	4	137	pattern specification process	BP	1	BARX1,HOXA3,SEMA3A,ACVR2A	up_VPOR_EC
GO:0060977	0.026	0.998	2	6	coronary vasculature morphogenesis	BP	1	PDGFRB,SPRED1	down_VCAV_EC
GO:0036023	0.026	0.999	2	3	embryonic skeletal limb joint morphogenesis	BP	1	OSR2,OSR1	up_VSAP_EC
GO:0072073	0.026	0.998	2	60	kidney epithelium development	BP	1	HOXA11,SALL1	up_VSAP_VSMC
GO:0046033	0.026	1	1	5	AMP metabolic process	BP	1	TNRC6B	down_ACOR_EC
GO:0009880	0.026	1	1	22	embryonic pattern specification	BP	1	WNT7A	up_AORT_VSMC
GO:0022602	0.026	1	1	13	ovulation cycle process	BP	1	SLIT2	up_VPUL_VSMC
GO:0046360	0.026031548	1	1	1	2-oxobutyrate biosynthetic process	BP	0.96436359	THNSL2	up_ACOR_EC
GO:0046361	0.026031548	1	1	1	2-oxobutyrate metabolic process	BP	0.96436359	THNSL2	up_ACOR_EC
GO:1901617	0.0261	0.99	10	60	organic hydroxy compound biosynthetic	BP	0.87	SQLE,LSS,SREBF1,HMGCR,INSIG1,ENSCAFG00000018572,DHCR24,MVD,FDFT1,NUS1	down_ACOR_VSMC
GO:0044249	0.0261	0.995	6	1851	cellular biosynthetic process	BP	1	TLR2,TBX15,EYA1,HOXA9,HOXA10,IRX5	up_AFEM_VSMC
GO:0006355	0.0261	0.993	6	974	regulation of transcription, DNA-	BP	1	EYA1,HOXA10,HOXA9,EMX2,HOXA11,SHOX2	up_VFEM_VSMC
GO:0060525	0.0261	0.999	2	3	prostate glandular acinus development	BP	1	FGFR2,FRS2	up_ACOR_VSMC
GO:0060526	0.0261	0.999	2	3	prostate glandular acinus morphogenesis	BP	1	FGFR2,FRS2	up_ACOR_VSMC
GO:0060527	0.0261	0.999	2	3	prostate epithelial cord arborization involved in prostate glandular acinus	BP	1	FGFR2,FRS2	up_ACOR_VSMC
GO:0040001	0.0261	1	1	12	establishment of mitotic spindle localization	BP	1	GPSM2	down_AFEM_EC
GO:0017158	0.0261	1	1	24	regulation of calcium ion-dependent exocytosis	BP	1	WNT7A	up_AORT_VSMC
GO:0035710	0.0261	1	1	22	CD4-positive, alpha-beta T cell activation	BP	1	ARG2	up_AORT_VSMC
GO:0048639	0.0261	1	1	39	positive regulation of developmental growth	BP	1	TBX5	up_APUL_VSMC
GO:0009226	0.0261	1	1	3	nucleotide-sugar biosynthetic process	BP	1	GNPNAT1	up_VPOR_EC
GO:0051004	0.0261	1	1	3	regulation of lipoprotein lipase activity	BP	1	ABHD5	up_VPOR_EC

GO:2000271	0.0261	1	1	3	positive regulation of fibroblast apoptotic	BP	1	STK17B	up_VPOR_EC
GO:0030336	0.026124203	0.99245	6	86	negative regulation of cell migration	BP	0.96436359	SEMA6D,ZMYND8,MMP28,PTGER4,ARHGDIB,TBX5	up_ACOR_EC
GO:0040008	0.0262	0.99	9	179	regulation of growth	BP	1	MT2A,LEP,ENSCAFG00000009113,DLL1,PLXNA4,GPAM,DCUN1D3,ACACB,ADAM17	up_AFEM_EC
GO:0043010	0.0262	0.992	6	96	camera-type eye development	BP	1	IRX5,COL8A2,DLL1,CRYAB,MAB21L1,THRB	up_AFEM_EC
GO:0051209	0.0262	0.994	5	28	release of sequestered calcium ion into cytosol	BP	0.776	ERO1A,LYN,DDIT3,HTT,CALM1	down_VSAP_EC
GO:0051283	0.0262	0.994	5	28	negative regulation of sequestering of calcium	BP	0.776	ERO1A,LYN,DDIT3,HTT,CALM1	down_VSAP_EC
GO:0035239	0.0262	0.995	4	299	tube morphogenesis	BP	1	HOXA3,NPNT,NTRK2,HOXA1	up_APUL_EC
GO:0043436	0.0262	0.997	3	303	oxoacid metabolic process	BP	1	ETFA,DARS,SUCLG2	down_AFEM_EC
GO:0030513	0.0262	0.997	3	11	positive regulation of BMP signaling pathway	BP	0.776	SULF1,ILK,NEO1	down_VSAP_EC
GO:0035162	0.0262	0.997	3	8	embryonic hemopoiesis	BP	0.707	KIT,GATA3,HIF1A	up_VCAV_EC
GO:0060509	0.0262	0.999	2	3	type I pneumocyte differentiation	BP	1	THRB,NFIB	up_VSAP_EC
GO:0021517	0.0262	1	1	12	ventral spinal cord development	BP	1	HOXC10	up_AORT_EC
GO:0034754	0.0262	1	1	24	cellular hormone metabolic process	BP	1	ESR1	up_AORT_VSMC
GO:0099175	0.0262	1	1	21	regulation of postsynapse organization	BP	1	WNT7A	up_AORT_VSMC
GO:0001974	0.0262	1	1	13	blood vessel remodeling	BP	1	HOXA3	up_VPUL_VSMC
GO:0043267	0.0262	1	1	6	negative regulation of potassium ion transport	BP	1	CAV1	up_VSAP_VSMC
GO:0009605	0.026239257	0.98554	22	538	response to external stimulus	BP	0.96436359	NR1H4,SOX9,HMCN1,RGS1,PCSK1N,FNDC4,AQP1,FZD5,PTGIR,NOD2,IL33,RGMA,MMP28,PTGER4,ELMO2,USP53,MAVS,IFIT2,RARRES2,AGBL5,NRP1,PRKCD	up_ACOR_EC
GO:0060429	0.0263	0.984	30	341	epithelium development	BP	0.776	PRICKLE1,SULF1,TAGLN2,SERPINE1,PRKACB,PTK7,ERRFI1,CASP3,PGK1,EXOC5,FLNB,ENSCAFG00000032470,TMED2,ILK,WDR1,MSN,HOXB7,HIF1A,ADAM17,ACTA2,MAGED1,CTNNB1,STARD13,PLOD3,KLF5,MYO1E,SDC4,INHBA,SIX4,NRP1	down_VSAP_EC
GO:0032501	0.0263	0.991	11	1782	multicellular organismal process	BP	1	TBX5,HOXA3,NR1H4,EVI2B,HOXA2,MYBPC2,CCL24,HTR7,FLRT3,ARHGEF2,NEO1	up_VPUL_EC
GO:0032502	0.0263	0.996	5	1661	developmental process	BP	1	HOXC10,HOXA9,WNT7A,ARG2,ESR1	up_AORT_VSMC
GO:0002685	0.0263	0.998	2	55	regulation of leukocyte migration	BP	1	LYN,HOXA7	down_ACOR_EC
GO:0034975	0.0263	0.999	2	5	protein folding in endoplasmic reticulum	BP	0.776	ERO1A,DNAJC10	down_VSAP_EC
GO:0050727	0.0263	0.998	2	74	regulation of inflammatory response	BP	1	NR1H4,CCL24	up_VPUL_EC
GO:0003158	0.0263	1	1	38	endothelium development	BP	1	HOXB5	up_APUL_VSMC
GO:0030282	0.0263	1	1	37	bone mineralization	BP	1	HOXA3	up_APUL_VSMC
GO:0003179	0.0263	1	1	19	heart valve morphogenesis	BP	1	GATA5	up_VCAV_VSMC
GO:0021952	0.0263	1	1	13	central nervous system projection neuron	BP	1	SLIT2	up_VPUL_VSMC
GO:0050729	0.026388797	0.9963	3	25	positive regulation of inflammatory response	BP	0.96436359	NOD2,IL33,PTGER4	up_ACOR_EC
GO:0042327	0.0264	0.984	34	289	positive regulation of phosphorylation	BP	0.709	TEK,HCLS1,KIT,MAP3K11,PDGFB,STK10,RELN,TLR4,EDN1,RALB,APLNR,NEDD9,YES1,MTOR,MAP3K3,BMP2,SLK,PROX1,MADD,PAK2,PRKD2,SH3GLB1,TLR3,PTGS2,MMD,RAPGEF2,TAOK1,HMGB1,RIPK1,ITGA6,PDGFD,JCAD,ARHGEF2,CDON	up_VCAV_EC
GO:0051223	0.0264	0.985	26	181	regulation of protein transport	BP	1	EXPH5,SH3TC2,TLR1,LEP,HTR2B,TCAF1,MBP,FERMT1,BMP4,TM7SF3,CD274,ANKRD1,F2RL1,CDKN1A,PTPN22,OS9,PRKCE,PSEN1,JUP,CCL5,NOD2,SCAMP5,INPP5K,PFKL,PARK7,PTPN14	up_ACOR_VSMC
GO:0045926	0.0264	0.99	9	72	negative regulation of growth	BP	0.776	ENPP1,IFRD1,SMARCA4,SESN2,HIF1A,WWC3,INHBA,CDKN1A,NRP1	down_VSAP_EC
GO:0106106	0.0264	0.995	4	48	cold-induced thermogenesis	BP	1	CMKLR1,HOXC10,LEP,PPARGC1A	up_AFEM_EC
GO:0120161	0.0264	0.995	4	48	regulation of cold-induced thermogenesis	BP	1	CMKLR1,HOXC10,LEP,PPARGC1A	up_AFEM_EC

GO:0034145	0.0264	0.999	2	3	positive regulation of toll-like receptor 4 signaling	BP	1	F2RL1,PTPN22	up_ACOR_VSMC
GO:0071493	0.0264	0.999	2	3	cellular response to UV-B	BP	1	MFAP4,CDKN1A	up_ACOR_VSMC
GO:2001212	0.0264	0.999	2	4	regulation of vasculogenesis	BP	0.709	CD34,RAPGEF2	up_VCAV_EC
GO:0006936	0.0264	0.998	2	74	muscle contraction	BP	1	MYBPC2,HTR7	up_VPUL_EC
GO:0030855	0.0264	1	1	183	epithelial cell differentiation	BP	1	GATA5	down_AFEM_VSMC
GO:0098609	0.0264	1	1	181	cell-cell adhesion	BP	1	GATA5	down_AFEM_VSMC
GO:0014910	0.0264	1	1	13	regulation of smooth muscle cell migration	BP	1	SLIT2	up_VPUL_VSMC
GO:0006793	0.0265	0.98	93	948	phosphorus metabolic process	BP	0.709	EPHB1,PLK2,TEK,HCLS1,NPR1,ADGRF5,KIT,PPP2R5A,PANK3,PRKCH,CDA,TNFAIP3,MAP3K11,TNIP1,PDGFB,STK10,SH2D3C,RELN,PDE4D,MEF2C,ATF3,RAPGEF1,TLR4,NLRC5,GK5,EDN1,RALB,APLNR,CTTNBP2NL,PIPOX,NEDD9,SERINC5,ADSS,YES1,GUCY1A1,SPTLC2,EPHB4,MTOR,ACPP,SAMD8,MAP3K3,BMP2,HHEX,PREX2,SLK,BMP2K,SH3BP5,PPP1R2,ERC1,MAPK13,ITPKB,PROX1,ENSCAFG00000010596,MADD,PAK2,PRKD2,SH3GLB1,SWAP70,ELOVL7,TLR3,ENSCAFG00000024323,NCK1,LDB2,SLC25A25,FES,NSD1,GUCY1B1,PTGS2,MMD,TOP1,RAPGEF2,PLCG2,CREB1,TAOK1,CNOT7,DCK,HMGB1,NOS2,BLM,SPTBN4,RIPK1,ITGA6,RAD17,IMPA1,PDGFD,PIP5K1B,BMX,HIF1A,LMTK2,FKBP15,JCAD,ARHGEF2,CDON	up_VCAV_EC
GO:0043009	0.0265	0.984	31	227	chordate embryonic development	BP	1	TBX15,HOXA11,FZD1,HOXA7,HOXA9,WNT11,AXIN2,OSR2,GJB5,IRX5,HOXD3,ABL1,DLL1,HOXA6,OSR1,FUZ,RARG,AMBRA1,MFN2,ZFP36L1,ZEB1,SULF2,SHOX2,KDM2B,TAF8,ZFAND5,ACVRL1,TGFBR2,CITED2,KEAP1,LATS2	up_VSAP_EC
GO:0034767	0.0265	0.992	7	31	positive regulation of ion transmembrane transport	BP	1	ACTN2,PLCG2,TCAF1,AKAP6,PSEN1,FXYD1,PARK7	up_ACOR_VSMC
GO:1903506	0.0265	0.993	6	977	regulation of nucleic acid-templated transcription	BP	1	EYA1,HOXA10,HOXA9,EMX2,HOXA11,SHOX2	up_VFEM_VSMC
GO:0106049	0.0265	0.999	2	4	regulation of cellular response to osmotic	BP	0.776	ARHGEF2,PTGS2	down_VSAP_EC
GO:1902218	0.0265	0.999	2	4	regulation of intrinsic apoptotic signaling pathway in response to	BP	0.776	ARHGEF2,PTGS2	down_VSAP_EC
GO:1902219	0.0265	0.999	2	4	negative regulation of intrinsic apoptotic signaling pathway in	BP	0.776	ARHGEF2,PTGS2	down_VSAP_EC
GO:0055062	0.0265	1	1	5	phosphate ion homeostasis	BP	1	HOXA3	down_ACOR_EC
GO:0072505	0.0265	1	1	5	divalent inorganic anion homeostasis	BP	1	HOXA3	down_ACOR_EC
GO:0072506	0.0265	1	1	5	trivalent inorganic anion homeostasis	BP	1	HOXA3	down_ACOR_EC
GO:2000392	0.0265	1	1	4	regulation of lamellipodium	BP	1	PDPN	down_ACOR_EC
GO:1903829	0.0265	1	1	91	positive regulation of cellular protein	BP	1	JUP	down_VFEM_VSMC
GO:2000146	0.0265	1	1	92	negative regulation of cell motility	BP	1	JUP	down_VFEM_VSMC
GO:0032717	0.0265	1	1	7	negative regulation of interleukin-8 production	BP	1	BPI	up_APUL_EC
GO:1901071	0.0265	1	1	6	glucosamine-containing compound metabolic	BP	1	CHST7	up_VFEM_EC
GO:0051049	0.026561879	0.98577	20	479	regulation of transport	BP	0.96436359	SH3TC2,CDH3,NR1H4,CALY,AQP1,ENSCAFG00000004331,KCNB1,EDEM2,FZD5,NOD2,ACTN2,PTGER4,PREB,MAPK8IP2,MAVS,RARRES2,VPS16,AZIN2,PRKCD,PM20D1	up_ACOR_EC
GO:1905517	0.026578702	0.99817	2	10	macrophage migration	BP	0.96436359	MMP28,RARRES2	up_ACOR_EC
GO:0071333	0.026587631	0.99626	3	25	cellular response to glucose stimulus	BP	0.96436359	NR1H4,KCNB1,PRKACA	up_ACOR_EC
GO:0003171	0.026588257	0.99816	2	10	atrioventricular valve development	BP	0.96436359	SMAD6,TBX5	up_ACOR_EC
GO:0016055	0.026598006	0.99058	8	134	Wnt signaling pathway	BP	0.96436359	CDH3,SOX9,PORCN,FZD5,DIXDC1,LRP4,CLSTN1,PYGO2	up_ACOR_EC
GO:0198738	0.026598006	0.99058	8	134	cell-cell signaling by wnt	BP	0.96436359	CDH3,SOX9,PORCN,FZD5,DIXDC1,LRP4,CLSTN1,PYGO2	up_ACOR_EC

GO:0043067	0.0266	0.983	39	479	regulation of programmed cell death	BP	0.778	ARHGEF2,VEGFA,P4HB,ASNS,NR1H4,PLK3,HSPA5,SERPINE1,CASP3,HYOU1,AARS,FHL2,PTGS2,HIF1A,TNIK,ZPR1,PTPN2,MTDH,MAGED1,CTNNB1,CRYAB,LYN,BNIP3,DDIT3,FAM162A,ATF3,GSK3B,SH3GLB1,HTT,REST,GLO1,PAK2,INHBA,SIX4,CDKN1A,NRP1,SPINK2,ARF4,MADD	down_VSAP_EC
GO:1902751	0.0266	0.995	4	14	positive regulation of cell cycle G2/M phase	BP	0.883	CDK1,CDK4,DTL,CDC25A	down_ACOR_VSMC
GO:1903707	0.0266	0.995	4	48	negative regulation of hemopoiesis	BP	1	HOXA9,HOXA7,DLL1,CYLD	up_AFEM_EC
GO:0007350	0.0266	0.997	3	7	blastoderm segmentation	BP	1	NRP1,FRS2,PCSK6	up_ACOR_VSMC
GO:2000112	0.0266	1	2	1119	regulation of cellular macromolecule biosynthetic process	BP	1	JUP,GATA5	down_VFEM_VSMC
GO:0019731	0.0266	0.999	2	3	antibacterial humoral response	BP	1	PLA2G6,JCHAIN	up_VSAP_EC
GO:0051451	0.0266	1	1	6	myoblast migration	BP	1	SIX1	up_VFEM_EC
GO:0060676	0.0266	1	1	6	ureteric bud formation	BP	1	SIX1	up_VFEM_EC
GO:0032689	0.0266	1	1	8	negative regulation of interferon-gamma	BP	1	NR1H4	up_VPUL_EC
GO:0021542	0.0266	1	1	6	dentate gyrus development	BP	1	EMX2	up_VSAP_VSMC
GO:1904385	0.0266	1	1	6	cellular response to angiotensin	BP	1	CAV1	up_VSAP_VSMC
GO:1990776	0.0266	1	1	6	response to angiotensin	BP	1	CAV1	up_VSAP_VSMC
GO:0003400	0.026642364	1	1	1	regulation of COPII vesicle coating	BP	0.96436359	PREB	up_ACOR_EC
GO:0090113	0.026642364	1	1	1	regulation of ER to Golgi vesicle-mediated transport by GTP	BP	0.96436359	PREB	up_ACOR_EC
GO:0009967	0.0267	0.982	54	435	positive regulation of signal transduction	BP	1	ADRA1B,TLR1,LGR6,LEP,MID1,GATA5,HTR2B,RASSF2,ZRANB1,C28H10orf71,SKP2,NACC2,MLLT3,ITM2C,FERMT1,BMP4,FGFR2,TLR3,SH3RF2,PDE5A,CITED2,TP53INP1,SASH1,TMEM108,FGF18,CDKN2B,LIMS2,ANKRD1,F2RL1,BNIP3,CRLF3,CREBRF,NRP1,PTPN22,NPNT,UNC5B,PRKCE,FRS2,PARP9,APP,PSEN1,CCL5,DUSP22,SIAH1,EDN1,NOD2,DISC1,SH3RF3,SERINC3,MAP3K7,ENSCAFG00000029553,PARK7,IL18R1,CRKL	up_ACOR_VSMC
GO:0034097	0.0267	0.985	22	238	response to cytokine	BP	0.779	ARHGEF2,NR1H4,HSPA5,SOCS5,FLNB,DPYSL3,ILK,MYO1C,KIF5B,HIF1A,CX3CL1,PTPN2,ADAM17,CNOT7,ENSCAFG00000002015,TFRC,ZC3H15,KLF5,CDC37,GBP5,BTK,KDM3A	down_VSAP_EC
GO:0016458	0.0267	0.99	10	59	gene silencing	BP	0.888	ATAD2,SRRT,RAN,UHRF1,SUV39H1,CDK2,RBM14,HAT1,HIST2H2AC,H2AFX	down_ACOR_VSMC
GO:0042306	0.0267	0.992	7	31	regulation of protein import into nucleus	BP	1	LEP,FERMT1,BMP4,CDKN1A,PTPN22,PSEN1,JUP	up_ACOR_VSMC
GO:0045595	0.0267	0.995	4	469	regulation of cell differentiation	BP	1	EYA1,HOXA9,HOXA11,SHOX2	up_VFEM_VSMC
GO:0006082	0.0267	0.996	3	305	organic acid metabolic process	BP	1	ETFA,DARS,SUCLG2	down_AFEM_EC
GO:0001945	0.0267	0.997	3	9	lymph vessel development	BP	0.71	SOX18,PROX1,VASH1	up_VCAV_EC
GO:0001946	0.0267	0.997	3	9	lymphangiogenesis	BP	0.71	SOX18,PROX1,VASH1	up_VCAV_EC
GO:0036303	0.0267	0.997	3	9	lymph vessel morphogenesis	BP	0.71	SOX18,PROX1,VASH1	up_VCAV_EC
GO:0048588	0.0267	0.998	2	44	developmental cell growth	BP	1	SPG11,NRP2	down_ACOR_EC
GO:0048255	0.0267	0.998	2	11	mRNA stabilization	BP	1	TENT5C,AXIN2	up_AFEM_EC
GO:0001818	0.0267	0.998	2	65	negative regulation of cytokine production	BP	1	NR1H4,BPI	up_APUL_EC
GO:0050890	0.0267	0.998	2	67	cognition	BP	1	NTRK2,HOXA1	up_APUL_EC
GO:0034137	0.0267	0.999	2	3	positive regulation of toll-like receptor 2 signaling	BP	1	F2RL1,CYBA	up_VSAP_EC
GO:0042473	0.0267	1	1	5	outer ear morphogenesis	BP	1	HOXA1	down_ACOR_EC
GO:0043030	0.0267	1	1	12	regulation of macrophage activation	BP	1	BPI	up_AORT_EC
GO:0021846	0.0267	1	1	11	cell proliferation in forebrain	BP	1	EMX2	up_VFEM_VSMC

GO:0030097	0.0268	0.985	23	244	hemopoiesis	BP	0.779	VEGFA,MYH9,ACTN1,SOCS5,ETV6,CASP3,PTPN11,WDR1,HOXB7,HIF1A,PTPN2,EVI2B,CTNNB1,ANLN,TFRC,LYN,MYO1E,REST,PRMT1,SH2B3,GLO1,INHBA,BTK	down_VSAP_EC
GO:0071559	0.0268	0.988	13	75	response to transforming growth factor beta	BP	1	SMAD6,FERMT1,CITED2,CDKN2B,ANKRD1,MXRA5,NPNT,FUT8,TGFBR1,NR3C1,DUSP22,HPGD,CRKL	up_ACOR_VSMC
GO:0072009	0.0268	0.992	7	48	nephron epithelium development	BP	0.779	ENSCAFG00000032470,ILK,HOXB7,MAGED1,CTNNB1,MYO1E,SIX4	down_VSAP_EC
GO:2001141	0.0268	0.993	6	979	regulation of RNA biosynthetic process	BP	1	EYA1,HOXA10,HOXA9,EMX2,HOXA11,SHOX2	up_VFEM_VSMC
GO:0009888	0.0268	0.993	6	545	tissue development	BP	1	HOXA11,PITX1,SALL1,CAV1,SHOX2,GJB5	up_VSAP_VSMC
GO:1901535	0.0268	0.999	2	3	regulation of DNA demethylation	BP	0.712	GATA3,OTUD4	up_VCAV_EC
GO:0035690	0.0268	1	1	91	cellular response to drug	BP	1	JUP	down_VFEM_VSMC
GO:0060485	0.0268	1	1	93	mesenchyme development	BP	1	GATA5	down_VFEM_VSMC
GO:0009064	0.0268	1	1	23	glutamine family amino acid metabolic process	BP	1	ARG2	up_AORT_VSMC
GO:0021563	0.0268	1	1	4	glossopharyngeal nerve development	BP	1	HOXA3	up_VPOR_EC
GO:0030851	0.0268	1	1	8	granulocyte differentiation	BP	1	EVI2B	up_VPUL_EC
GO:0032620	0.0268	1	1	8	interleukin-17 production	BP	1	NR1H4	up_VPUL_EC
GO:0071071	0.0268	1	1	8	regulation of phospholipid biosynthetic process	BP	1	NR1H4	up_VPUL_EC
GO:0035556	0.0269	0.981	65	845	intracellular signal transduction	BP	0.779	ARHGEF2,VEGFA,P4HB,SIPA1L1,ARHGAP31,NR1H4,SOCS5,SGK1,ERO1A,JCAD,CASP3,HYOU1,WSB1,BCAR3,NUAK1,PTPN11,TRIB3,FARP1,ILK,FHL2,SESN2,TGFBR1,PTGS2,PIK3C2A,UACA,HIF1A,DNAJC10,TNIK,PTPN2,PLCL1,MTDH,EIF4EBP1,CNOT7,MAGED1,GNAQ,CTNNB1,KPNB1,ENSCAFG00000002015,APOE,WWC3,LYN,BNIP3,DDIT3,PLEKHG2,CBLC,ATF3,GSK3B,TAX1BP3,HTT,MAPK10,SH2B3,ARFGEF3,CALM1,DISC1,PAK2,INHBA,CAMK2D,BTK,CDKN1A,NRP1,SH3RF3,TNFAIP1,MADD,SEC13,MID1	down_VSAP_EC
GO:0048732	0.0269	0.986	18	116	gland development	BP	1	HOXA9,WNT11,HNF4A,LIMS2,HOXD3,FGF7,ABL1,RARG,STAT6,SULF2,CLCN2,NTN4,RAF1,NFIB,MAP2K2,TGFBR2,CITED2,LBH	up_VSAP_EC
GO:0051171	0.0269	0.989	14	1697	regulation of nitrogen compound metabolic process	BP	1	ACVRL1,YEATS2,LYN,IGF1R,YBX3,HIVEP2,HOXA7,PRKAR2A,SULF1,ELK3,HOXA1,ACSL3,ASPN,NEDD4	down_ACOR_EC
GO:0072329	0.0269	0.993	5	34	monocarboxylic acid catabolic process	BP	1	AKT2,ACAA1,PEX5,LONP2,ACAT1	down_VCAV_EC
GO:0006366	0.0269	0.997	3	583	transcription by RNA polymerase II	BP	1	HOXA9,WNT7A,ESR1	up_AORT_VSMC
GO:0099170	0.0269	0.999	2	3	postsynaptic modulation of chemical synaptic	BP	1	EGLN1,RNF19A	up_ACOR_VSMC
GO:0050650	0.0269	0.998	2	11	chondroitin sulfate proteoglycan biosynthetic	BP	1	XYLT1,CSGALNACT2	up_AFEM_EC
GO:0006574	0.0269	0.999	2	3	valine catabolic process	BP	1	ALDH6A1,ENSCAFG00000002977	up_VSAP_EC
GO:0032693	0.0269	0.999	2	3	negative regulation of interleukin-10 production	BP	1	CD274,DLL1	up_VSAP_EC
GO:0072608	0.0269	0.999	2	3	interleukin-10 secretion	BP	1	CD274,F2RL1	up_VSAP_EC
GO:0070646	0.0269	1	1	63	protein modification by small protein removal	BP	1	USP12	down_VFEM_EC
GO:0009566	0.0269	1	1	24	fertilization	BP	1	HOXA9	up_AORT_VSMC
GO:0033238	0.0269	1	1	7	regulation of cellular amine metabolic process	BP	1	NR1H4	up_APUL_EC
GO:0009312	0.0269	1	1	9	oligosaccharide biosynthetic process	BP	1	FBP1	up_VFEM_VSMC
GO:2001169	0.0269	1	1	11	regulation of ATP biosynthetic process	BP	1	FBP1	up_VFEM_VSMC
GO:0031953	0.0269	1	1	6	negative regulation of protein	BP	1	CAV1	up_VSAP_VSMC
GO:0044249	0.027	0.992	9	1851	cellular biosynthetic process	BP	1	EYA1,GATM,HOXA10,HOXA9,TBX15,FBP1,EMX2,HOXA11,SHOX2	up_VFEM_VSMC
GO:1901654	0.027	0.992	7	31	response to ketone	BP	1	PRKAA2,ADCY6,PTGDR,PRKCE,NR3C1,DDIT4,PARK7	up_ACOR_VSMC
GO:0006809	0.027	0.995	4	14	nitric oxide biosynthetic process	BP	0.713	CD34,TLR4,NOS3,NOS2	up_VCAV_EC

GO:0046209	0.027	0.995	4	14	nitric oxide metabolic process	BP	0.713	CD34,TLR4,NOS3,NOS2	up_VCAV_EC
GO:0050854	0.027	0.995	4	16	regulation of antigen receptor-mediated	BP	0.713	PRKCH,NECTIN2,PRKD2,BCL10	up_VCAV_EC
GO:0043269	0.027	0.996	3	135	regulation of ion transport	BP	1	LYN,HOXA3,NEDD4	down_ACOR_EC
GO:0051924	0.027	0.998	2	53	regulation of calcium ion transport	BP	1	LYN,HOXA3	down_ACOR_EC
GO:0003415	0.027	0.999	2	3	chondrocyte hypertrophy	BP	1	RARG,TGFBR2	up_VSAP_EC
GO:0060841	0.027	1	1	6	venous blood vessel development	BP	1	ACVRL1	down_ACOR_EC
GO:0031589	0.027	1	1	93	cell-substrate adhesion	BP	1	JUP	down_VFEM_VSMC
GO:0010092	0.027	1	1	19	specification of animal organ identity	BP	1	GATA5	up_VCAV_VSMC
GO:1902285	0.027	1	1	4	semaphorin-plexin signaling pathway involved in neuron	BP	1	SEMA3A	up_VPOR_EC
GO:0006808	0.027052226	1	1	1	regulation of nitrogen utilization	BP	0.96436359	NR1H4	up_ACOR_EC
GO:0019676	0.027052226	1	1	1	ammonia assimilation cycle	BP	0.96436359	NR1H4	up_ACOR_EC
GO:0019740	0.027052226	1	1	1	nitrogen utilization	BP	0.96436359	NR1H4	up_ACOR_EC
GO:0034255	0.027052226	1	1	1	regulation of urea metabolic process	BP	0.96436359	NR1H4	up_ACOR_EC
GO:0034971	0.027052226	1	1	1	histone H3-R17 methylation	BP	0.96436359	NR1H4	up_ACOR_EC
GO:0038183	0.027052226	1	1	1	bile acid signaling pathway	BP	0.96436359	NR1H4	up_ACOR_EC
GO:0038185	0.027052226	1	1	1	intracellular bile acid receptor signaling	BP	0.96436359	NR1H4	up_ACOR_EC
GO:0045848	0.027052226	1	1	1	positive regulation of nitrogen utilization	BP	0.96436359	NR1H4	up_ACOR_EC
GO:1903314	0.027052226	1	1	1	regulation of nitrogen cycle metabolic process	BP	0.96436359	NR1H4	up_ACOR_EC
GO:1905693	0.027052226	1	1	1	regulation of phosphatidic acid biosynthetic process	BP	0.96436359	NR1H4	up_ACOR_EC
GO:1905695	0.027052226	1	1	1	positive regulation of phosphatidic acid biosynthetic process	BP	0.96436359	NR1H4	up_ACOR_EC
GO:2000211	0.027052226	1	1	1	regulation of glutamate metabolic process	BP	0.96436359	NR1H4	up_ACOR_EC
GO:2000213	0.027052226	1	1	1	positive regulation of glutamate metabolic	BP	0.96436359	NR1H4	up_ACOR_EC
GO:2001248	0.027052226	1	1	1	regulation of ammonia assimilation cycle	BP	0.96436359	NR1H4	up_ACOR_EC
GO:2001250	0.027052226	1	1	1	positive regulation of ammonia assimilation	BP	0.96436359	NR1H4	up_ACOR_EC
GO:0098629	0.027061226	1	1	1	trans-Golgi network membrane organization	BP	0.96436359	AZIN2	up_ACOR_EC
GO:0003290	0.027067801	1	1	1	atrial septum secundum morphogenesis	BP	0.96436359	GATA4	up_ACOR_EC
GO:1902310	0.027067801	1	1	1	positive regulation of peptidyl-serine	BP	0.96436359	DUSP26	up_ACOR_EC
GO:0003275	0.027091778	1	1	1	apoptotic process involved in outflow tract	BP	0.96436359	FOXC2	up_ACOR_EC
GO:0035470	0.027091778	1	1	1	positive regulation of vascular wound healing	BP	0.96436359	FOXC2	up_ACOR_EC
GO:0048342	0.027091778	1	1	1	paraxial mesodermal cell differentiation	BP	0.96436359	FOXC2	up_ACOR_EC
GO:0048343	0.027091778	1	1	1	paraxial mesodermal cell fate commitment	BP	0.96436359	FOXC2	up_ACOR_EC
GO:0072011	0.027091778	1	1	1	glomerular endothelium development	BP	0.96436359	FOXC2	up_ACOR_EC
GO:1902256	0.027091778	1	1	1	regulation of apoptotic process involved in outflow tract	BP	0.96436359	FOXC2	up_ACOR_EC
GO:1902257	0.027091778	1	1	1	negative regulation of apoptotic process involved in outflow tract	BP	0.96436359	FOXC2	up_ACOR_EC
GO:0007538	0.027096764	1	1	1	primary sex determination	BP	0.96436359	SOX9	up_ACOR_EC
GO:0007542	0.027096764	1	1	1	primary sex determination, germ-line	BP	0.96436359	SOX9	up_ACOR_EC
GO:0014034	0.027096764	1	1	1	neural crest cell fate commitment	BP	0.96436359	SOX9	up_ACOR_EC
GO:0014036	0.027096764	1	1	1	neural crest cell fate specification	BP	0.96436359	SOX9	up_ACOR_EC

GO:0018992	0.027096764	1	1	1	germ-line sex determination	BP	0.96436359	SOX9	up_ACOR_EC
GO:0019100	0.027096764	1	1	1	male germ-line sex determination	BP	0.96436359	SOX9	up_ACOR_EC
GO:0060221	0.027096764	1	1	1	retinal rod cell differentiation	BP	0.96436359	SOX9	up_ACOR_EC
GO:0060516	0.027096764	1	1	1	primary prostatic bud elongation	BP	0.96436359	SOX9	up_ACOR_EC
GO:0060517	0.027096764	1	1	1	epithelial cell proliferation involved in prostatic bud elongation	BP	0.96436359	SOX9	up_ACOR_EC
GO:0060784	0.027096764	1	1	1	regulation of cell proliferation involved in	BP	0.96436359	SOX9	up_ACOR_EC
GO:0072034	0.027096764	1	1	1	renal vesicle induction	BP	0.96436359	SOX9	up_ACOR_EC
GO:2000741	0.027096764	1	1	1	positive regulation of mesenchymal stem cell differentiation	BP	0.96436359	SOX9	up_ACOR_EC
GO:0007165	0.0271	0.989	13	1496	signal transduction	BP	1	ACVRL1,LYN,IGF1R,PDPN,NRP2,HIVEP2,USP28,IPO7,SULF1,WDR12,STX4,ASPN,NEDD4	down_ACOR_EC
GO:0040011	0.0271	0.994	5	505	locomotion	BP	1	TBX5,CCL24,FLRT3,ARHGEF2,NEO1	up_VPUL_EC
GO:0072358	0.0271	0.996	3	268	cardiovascular system development	BP	1	HOXA3,NKX2-5,C3AR1	up_VPOR_VSMC
GO:0003418	0.0271	0.999	2	3	growth plate cartilage chondrocyte	BP	1	RARG,TGFBR2	up_VSAP_EC
GO:0003431	0.0271	0.999	2	3	growth plate cartilage chondrocyte development	BP	1	RARG,TGFBR2	up_VSAP_EC
GO:0140115	0.0271	0.999	2	3	export across plasma membrane	BP	1	KCNE3,FXYD1	up_VSAP_EC
GO:0051180	0.0271	1	1	7	vitamin transport	BP	1	PDPN	down_ACOR_EC
GO:0071363	0.0271	1	1	188	cellular response to growth factor stimulus	BP	1	GATA5	down_AFEM_VSMC
GO:0001825	0.0271	1	1	14	blastocyst formation	BP	1	CITED2	down_VPOR_EC
GO:1901224	0.0271	1	1	20	positive regulation of NIK/NF-kappaB signaling	BP	1	TLR2	up_AFEM_VSMC
GO:0032092	0.0271	1	1	38	positive regulation of protein binding	BP	1	HOXA3	up_APUL_VSMC
GO:0060343	0.0271	1	1	11	trabecula formation	BP	1	NKX2-5	up_VPOR_VSMC
GO:0045980	0.0271	1	1	10	negative regulation of nucleotide metabolic	BP	1	FBP1	up_VFEM_VSMC
GO:0003218	0.027138217	1	1	1	cardiac left ventricle formation	BP	0.96436359	TBX5	up_ACOR_EC
GO:0071612	0.027144544	1	1	1	IP-10 production	BP	0.96436359	MAVS	up_ACOR_EC
GO:0071651	0.027144544	1	1	1	positive regulation of chemokine (C-C motif)	BP	0.96436359	MAVS	up_ACOR_EC
GO:0071658	0.027144544	1	1	1	regulation of IP-10 production	BP	0.96436359	MAVS	up_ACOR_EC
GO:0071660	0.027144544	1	1	1	positive regulation of IP-10 production	BP	0.96436359	MAVS	up_ACOR_EC
GO:0001655	0.0272	0.989	11	116	urogenital system development	BP	1	SMAD9,KANK2,CLSTN1,PKD1,GLI3,GLIS2,PRKCSH,RDH10,GREM1,ZBTB16,SEC61A1	down_VCAV_EC
GO:0009887	0.0272	0.998	2	314	animal organ morphogenesis	BP	1	GATA5,TGFBR1	down_VSAP_VSMC
GO:0002756	0.0272	0.999	2	3	MyD88-independent toll-like receptor signaling	BP	0.717	TLR4,TLR3	up_VCAV_EC
GO:0002645	0.0272	0.999	2	3	positive regulation of tolerance induction	BP	1	CD274,TGFBR2	up_VSAP_EC
GO:0035739	0.0272	0.999	2	3	CD4-positive, alpha-beta T cell proliferation	BP	1	CD274,TGFBR2	up_VSAP_EC
GO:0042983	0.0272	0.999	2	3	amyloid precursor protein biosynthetic process	BP	1	NECAB3,SOAT1	up_VSAP_EC
GO:0042984	0.0272	0.999	2	3	regulation of amyloid precursor protein	BP	1	NECAB3,SOAT1	up_VSAP_EC
GO:2000561	0.0272	0.999	2	3	regulation of CD4-positive, alpha-beta T cell	BP	1	CD274,TGFBR2	up_VSAP_EC
GO:0045656	0.0272	0.999	2	3	negative regulation of monocyte differentiation	BP	1	HOXA7,MYC	up_VSAP_EC
GO:0001709	0.0272	1	1	11	cell fate determination	BP	1	CYP26B1	up_VPOR_VSMC
GO:0032703	0.0272	1	1	8	negative regulation of interleukin-2 production	BP	1	NR1H4	up_VPUL_EC
GO:0070098	0.0272	1	1	14	chemokine-mediated signaling pathway	BP	1	SLIT2	up_VPUL_VSMC
GO:0031646	0.0272	1	1	7	positive regulation of neurological system	BP	1	NETO1	up_VSAP_VSMC

GO:0010669	0.027231185	0.9981	2	10	epithelial structure maintenance	BP	0.96436359	SOX9,NOD2	up_ACOR_EC
GO:0007517	0.0273	0.987	16	100	muscle organ development	BP	1	TNNT2,BMP4,FGFR2,GJA1,CITED2,ANKRD1,EGLN1,CHD2,TBX20,TBX5,FRS2,CHD7,EDN1,ARID5B,ID3,BVES	up_ACOR_VSMC
GO:0010632	0.0273	0.989	11	95	regulation of epithelial cell migration	BP	0.785	VEGFA,SPARC,JCAD,PRCP,PIK3C2A,HIF1A,ADAM17,STARD13,APOE,STRAP,NRP1	down_VSAP_EC
GO:0032729	0.0273	0.995	4	15	positive regulation of interferon-gamma	BP	0.717	PDE4D,TLR4,IRF8,TLR3	up_VCAV_EC
GO:0008284	0.0273	0.998	2	238	positive regulation of cell proliferation	BP	1	WNT7A,ESR1	up_AORT_VSMC
GO:0016053	0.0273	0.998	2	97	organic acid biosynthetic process	BP	1	GATM,FBP1	up_VFEM_VSMC
GO:0046394	0.0273	0.998	2	97	carboxylic acid biosynthetic process	BP	1	GATM,FBP1	up_VFEM_VSMC
GO:0034370	0.0273	0.999	2	3	triglyceride-rich lipoprotein particle	BP	1	LIPC,LCAT	up_VSAP_EC
GO:0034372	0.0273	0.999	2	3	very-low-density lipoprotein particle	BP	1	LIPC,LCAT	up_VSAP_EC
GO:0002278	0.0273	0.999	2	3	eosinophil activation involved in immune	BP	1	F2RL1,STX4	up_VSAP_EC
GO:0002447	0.0273	0.999	2	3	eosinophil mediated immunity	BP	1	F2RL1,STX4	up_VSAP_EC
GO:0060216	0.0273	0.999	2	3	definitive hemopoiesis	BP	1	HOXA9,ZFP36L2	up_VSAP_EC
GO:0072047	0.0273	0.999	2	3	proximal/distal pattern formation involved in	BP	1	OSR1,IRX3	up_VSAP_EC
GO:0072081	0.0273	0.999	2	3	specification of nephron tubule identity	BP	1	OSR1,IRX3	up_VSAP_EC
GO:0072177	0.0273	0.999	2	3	mesonephric duct development	BP	1	WNT11,OSR1	up_VSAP_EC
GO:0043307	0.0273	0.999	2	3	eosinophil activation	BP	1	F2RL1,STX4	up_VSAP_EC
GO:0043308	0.0273	0.999	2	3	eosinophil degranulation	BP	1	F2RL1,STX4	up_VSAP_EC
GO:0051895	0.0273	1	1	7	negative regulation of focal adhesion assembly	BP	1	ACVRL1	down_ACOR_EC
GO:1903392	0.0273	1	1	7	negative regulation of adherens junction	BP	1	ACVRL1	down_ACOR_EC
GO:2000243	0.0273	1	1	14	positive regulation of reproductive process	BP	1	CITED2	down_VPOR_EC
GO:0010632	0.0273	1	1	95	regulation of epithelial cell migration	BP	1	JUP	down_VFEM_VSMC
GO:0046634	0.0273	1	1	24	regulation of alpha-beta T cell activation	BP	1	ARG2	up_AORT_VSMC
GO:0061323	0.0273	1	1	7	cell proliferation involved in heart morphogenesis	BP	1	TBX5	up_APUL_EC
GO:2000136	0.0273	1	1	7	regulation of cell proliferation involved in	BP	1	TBX5	up_APUL_EC
GO:0007140	0.0273	1	1	10	male meiotic nuclear division	BP	1	CYP26B1	up_VPOR_VSMC
GO:0051926	0.0273	1	1	14	negative regulation of calcium ion transport	BP	1	HOXA3	up_VPUL_VSMC
GO:0048643	0.0273	1	1	7	positive regulation of skeletal muscle tissue	BP	1	SHOX2	up_VSAP_VSMC
GO:0086065	0.0273	1	1	6	cell communication involved in cardiac	BP	1	CAV1	up_VSAP_VSMC
GO:0045234	0.02730132	1	1	1	protein palmitoleylation	BP	0.96436359	PORCN	up_ACOR_EC
GO:0060061	0.027338152	1	1	1	Spemann organizer formation	BP	0.96436359	FZD5	up_ACOR_EC
GO:0060715	0.027338152	1	1	1	syncytiotrophoblast cell differentiation involved in labyrinthine layer	BP	0.96436359	FZD5	up_ACOR_EC
GO:0097360	0.027338152	1	1	1	chorionic trophoblast cell proliferation	BP	0.96436359	FZD5	up_ACOR_EC
GO:1901382	0.027338152	1	1	1	regulation of chorionic trophoblast cell	BP	0.96436359	FZD5	up_ACOR_EC
GO:0036509	0.027384624	1	1	1	trimming of terminal mannose on B branch	BP	0.96436359	EDEM2	up_ACOR_EC
GO:1904380	0.027384624	1	1	1	endoplasmic reticulum mannose trimming	BP	0.96436359	EDEM2	up_ACOR_EC
GO:0043605	0.027398898	1	1	1	cellular amide catabolic process	BP	0.96436359	PM20D1	up_ACOR_EC
GO:0035295	0.0274	0.983	31	356	tube development	BP	0.785	PRICKLE1,VEGFA,SULF1,MYH9,SERPINE1,SPARC,PRKACB,ERRFI1,JCAD,PGK1,ENSCAFG00000032470,TMED2,ILK,PRCP,PIK3C2A,HOXB7,HIF1A,THBS2,MTDH,ESM1,MAGED1,CTNNB1,STARD13,APOE,PLOD3,KLF5,MYO1E,SDC4,SIX4,NRP1,SPINK2	down_VSAP_EC

GO:0097193	0.0274	0.988	12	109	intrinsic apoptotic signaling pathway	BP	0.785	ARHGEF2,P4HB,ERO1A,CASP3,HYOU1,PTGS2,HIF1A,DNAJC10,PTPN2,BNIP3,DDIT3,CDKN1A	down_VSAP_EC
GO:0090189	0.0274	0.996	3	12	regulation of branching involved in ureteric bud	BP	0.785	HOXB7,MAGED1,SIX4	down_VSAP_EC
GO:0009790	0.0274	0.996	3	335	embryo development	BP	1	HOXA3,TBX5,HOXB5	up_VPUL_VSMC
GO:0032700	0.0274	0.999	2	4	negative regulation of interleukin-17 production	BP	0.717	TLR4,ARG2	up_VCAV_EC
GO:0060047	0.0274	0.998	2	57	heart contraction	BP	1	IRX5,CAV1	up_VSAP_VSMC
GO:0070168	0.0274	1	1	6	negative regulation of biomineral tissue	BP	1	ASPN	down_ACOR_EC
GO:0090169	0.0274	1	1	13	regulation of spindle assembly	BP	1	GPSM2	down_AFEM_EC
GO:0003007	0.0274	1	1	94	heart morphogenesis	BP	1	GATA5	down_VFEM_VSMC
GO:0010717	0.0274	1	1	32	regulation of epithelial to mesenchymal transition	BP	1	TGFBR1	down_VSAP_VSMC
GO:0021510	0.0274	1	1	23	spinal cord development	BP	1	HOXC10	up_AORT_VSMC
GO:0006654	0.0274	1	1	7	phosphatidic acid biosynthetic process	BP	1	NR1H4	up_APUL_EC
GO:0046473	0.0274	1	1	7	phosphatidic acid metabolic process	BP	1	NR1H4	up_APUL_EC
GO:0055089	0.0274	1	1	7	fatty acid homeostasis	BP	1	NR1H4	up_APUL_EC
GO:0021517	0.0274	1	1	12	ventral spinal cord development	BP	1	HOXC10	up_VFEM_VSMC
GO:0032940	0.0275	0.984	27	231	secretion by cell	BP	0.717	GBP5,KIT,EXOC3L2,MEF2C,FERMT1,CHD7,TLR4,GATA3,EDN1,RAB11A,RAB27A,EXOC6,SNCAIP,ARG2,SNAP23,SCAMP1,RSC1A1,ABCA1,EXOC5,CD200,FES,CREB1,STX11,HMGB1,NOS2,MCU,HIF1A	up_VCAV_EC
GO:0072503	0.0275	0.987	14	97	cellular divalent inorganic cation homeostasis	BP	0.717	ATP2B1,PDE4D,CHD7,GPR4,STC1,EDN1,APLNR,SLC39A8,PDZD8,SWAP70,PLCG2,HMGB1,MCU,ATP2A1	up_VCAV_EC
GO:0030258	0.0275	0.99	8	71	lipid modification	BP	1	ENSCAFG00000014978,AKT2,LCAT,ACAA1,PEX5,LONP2,SOAT1,ACAT1	down_VCAV_EC
GO:0060041	0.0275	0.991	8	41	retina development in camera-type eye	BP	0.717	ARHGEF15,CHD7,DLL4,PROX1,TSPAN12,SMARCA4,HIF1A,CDON	up_VCAV_EC
GO:0003008	0.0275	0.996	3	336	system process	BP	1	GATA5,LRRN4,SLIT2	up_VPUL_VSMC
GO:0010556	0.0275	1	2	1137	regulation of macromolecule	BP	1	JUP,GATA5	down_VFEM_VSMC
GO:1903524	0.0275	0.998	2	11	positive regulation of blood circulation	BP	1	AVPR1A,TBXA2R	up_AFEM_EC
GO:0035774	0.0275	1	1	6	positive regulation of insulin secretion involved in cellular response to	BP	1	STX4	down_ACOR_EC
GO:0017038	0.0275	1	1	96	protein import	BP	1	JUP	down_VFEM_VSMC
GO:0008585	0.0275	1	1	24	female gonad development	BP	1	ESR1	up_AORT_VSMC
GO:0046545	0.0275	1	1	24	development of primary female sexual	BP	1	ESR1	up_AORT_VSMC
GO:0045987	0.0275	1	1	7	positive regulation of smooth muscle	BP	1	NPNT	up_APUL_EC
GO:0003283	0.0275	1	1	8	atrial septum development	BP	1	TBX5	up_VPUL_EC
GO:0060413	0.0275	1	1	8	atrial septum morphogenesis	BP	1	TBX5	up_VPUL_EC
GO:0032332	0.0275	1	1	6	positive regulation of chondrocyte	BP	1	HOXA11	up_VSAP_VSMC
GO:0009653	0.0276	0.979	89	773	anatomical structure morphogenesis	BP	1	TBX15,HOXA11,FZD1,THRB,HOXA7,PEX7,HOXA9,CDON,WNT11,HNF4A,ACKR3,AXIN2,WASF3,OSR2,INSIG2,ENPP2,RADIL,PPARGC1B,GJB5,LIMS2,LRIG3,DDHD1,IRX5,SLIT2,HOXD3,SLC6A4,FGF7,ABL1,BTG1,HDAC7,EMP2,DLL1,EPN2,HOXA6,OSR1,CDC42EP5,FUZ,WARS2,SAV1,HPSE,PLXNA4,RILPL2,CDC42EP2,ZBTB16,RARG,CCBE1,STAT6,HOXC4,RECK,PLPP3,DLX3,MFN2,ZFP36L1,IRX3,ZEB1,FBXW4,TBXA2R,SULF2,SHOX2,SASH1,HOXC10,POLDIP2,LOX,LRP4,DPYSL2,FRMD6,E2F4,NTN4,LIMD1,KDM2B,FBLN1,HPGD,NFIB,ZFAND5,ANPEP,PQBP1,TTL,ARHGAP12,ACVRL1,FLOT1,PPP3CB,ULK1,HMOX1,MAP2K2,TGFBR2,NPRL3,CITED2,OGN,LATS2	up_VSAP_EC

GO:0071333	0.0276	0.993	6	25	cellular response to glucose stimulus	BP	1	RAB11B,KCNB1,RAF1,STX4,PPP3CB,RAB11FIP2	up_VSAP_EC
GO:0006935	0.0276	0.994	4	143	chemotaxis	BP	1	C3AR1,SEMA3A,SEMA3D,ENSCAFG00000032731	up_VPOR_EC
GO:0042330	0.0276	0.994	4	143	taxis	BP	1	C3AR1,SEMA3A,SEMA3D,ENSCAFG00000032731	up_VPOR_EC
GO:0051656	0.0276	0.996	3	129	establishment of organelle localization	BP	1	LYN,SPG11,STX4	down_ACOR_EC
GO:2001014	0.0276	0.997	3	8	regulation of skeletal muscle cell differentiation	BP	0.717	EPHB1,MEF2C,NR1D2	up_VCAV_EC
GO:1903522	0.0276	0.998	2	58	regulation of blood circulation	BP	1	IRX5,CAV1	up_VSAP_VSMC
GO:0003181	0.0276	1	1	7	atrioventricular valve morphogenesis	BP	1	TBX5	up_APUL_EC
GO:0032682	0.0276	1	1	7	negative regulation of chemokine production	BP	1	NR1H4	up_APUL_EC
GO:0016202	0.0276	1	1	39	regulation of striated muscle tissue	BP	1	TBX5	up_APUL_VSMC
GO:0007026	0.0276	1	1	8	negative regulation of microtubule	BP	1	ARHGEF2	up_VPUL_EC
GO:0060544	0.0276	1	1	8	regulation of necroptotic process	BP	1	ARHGEF2	up_VPUL_EC
GO:1904467	0.0276	1	1	8	regulation of tumor necrosis factor secretion	BP	1	NR1H4	up_VPUL_EC
GO:0007632	0.0276	1	1	14	visual behavior	BP	1	LRRN4	up_VPUL_VSMC
GO:1902742	0.0276	1	1	14	apoptotic process involved in development	BP	1	SLIT2	up_VPUL_VSMC
GO:0032570	0.0276	1	1	6	response to progesterone	BP	1	CAV1	up_VSAP_VSMC
GO:0051490	0.027648686	1	1	1	negative regulation of filopodium assembly	BP	0.96436359	PRKCD	up_ACOR_EC
GO:0060697	0.027648686	1	1	1	positive regulation of phospholipid catabolic	BP	0.96436359	PRKCD	up_ACOR_EC
GO:0110112	0.027648686	1	1	1	regulation of lipid transporter activity	BP	0.96436359	PRKCD	up_ACOR_EC
GO:0110113	0.027648686	1	1	1	positive regulation of lipid transporter activity	BP	0.96436359	PRKCD	up_ACOR_EC
GO:1900161	0.027648686	1	1	1	regulation of phospholipid scramblase activity	BP	0.96436359	PRKCD	up_ACOR_EC
GO:1900163	0.027648686	1	1	1	positive regulation of phospholipid scramblase	BP	0.96436359	PRKCD	up_ACOR_EC
GO:2000752	0.027648686	1	1	1	regulation of glucosylceramide	BP	0.96436359	PRKCD	up_ACOR_EC
GO:2000753	0.027648686	1	1	1	positive regulation of glucosylceramide catabolic process	BP	0.96436359	PRKCD	up_ACOR_EC
GO:2000754	0.027648686	1	1	1	regulation of sphingomyelin catabolic	BP	0.96436359	PRKCD	up_ACOR_EC
GO:2000755	0.027648686	1	1	1	positive regulation of sphingomyelin catabolic	BP	0.96436359	PRKCD	up_ACOR_EC
GO:0021884	0.027693558	0.99805	2	10	forebrain neuron development	BP	0.96436359	DCLK2,NRP1	up_ACOR_EC
GO:0010810	0.0277	0.991	7	60	regulation of cell-substrate adhesion	BP	1	FBLN2,RHOD,THY1,EDIL3,GREM1,RAC2,ECM2	down_VCAV_EC
GO:0048524	0.0277	0.993	5	30	positive regulation of viral process	BP	0.791	P4HB,SMARCA4,CNOT7,APOE,RAD23A	down_VSAP_EC
GO:0001937	0.0277	0.995	4	15	negative regulation of endothelial cell	BP	0.718	MEF2C,DLL4,CAV2,VASH1	up_VCAV_EC
GO:0030033	0.0277	0.997	3	7	microvillus assembly	BP	0.718	PODXL,RAPGEF2,FSCN1	up_VCAV_EC
GO:0050857	0.0277	0.997	3	9	positive regulation of antigen receptor-mediated signaling	BP	0.718	PRKCH,NECTIN2,PRKD2	up_VCAV_EC
GO:0007596	0.0277	0.998	2	47	blood coagulation	BP	1	LYN,PDPN	down_ACOR_EC
GO:2001234	0.0277	0.998	2	75	negative regulation of apoptotic signaling	BP	1	PTGER2,ARHGEF2	up_VPUL_EC
GO:0006656	0.0277	1	1	7	phosphatidylcholine biosynthetic process	BP	1	ACSL3	down_ACOR_EC
GO:0090398	0.0277	1	1	24	cellular senescence	BP	1	ARG2	up_AORT_VSMC
GO:0071225	0.0277	1	1	8	cellular response to muramyl dipeptide	BP	1	ARHGEF2	up_VPUL_EC
GO:0035608	0.027791732	1	1	1	protein deglutamylation	BP	0.96436359	AGBL5	up_ACOR_EC
GO:0035611	0.027791732	1	1	1	protein branching point deglutamylation	BP	0.96436359	AGBL5	up_ACOR_EC
GO:0034121	0.0278	0.994	5	22	regulation of toll-like receptor signaling	BP	0.718	TNFAIP3,PELI1,TLR3,OTUD4,HMGB1	up_VCAV_EC

GO:0043488	0.0278	0.996	3	28	regulation of mRNA stability	BP	1	TENT5C,AXIN2,SAMD4B	up_AFEM_EC
GO:1990440	0.0278	0.998	2	5	positive regulation of transcription from RNA polymerase II promoter in response to endoplasmic reticulum stress	BP	0.792	DDIT3,ATF3	down_VSAP_EC
GO:0070848	0.0278	1	1	193	response to growth factor	BP	1	GATA5	down_AFEM_VSMC
GO:0007164	0.0278	1	1	23	establishment of tissue polarity	BP	1	WNT7A	up_AORT_VSMC
GO:0001736	0.0278	1	1	23	establishment of planar polarity	BP	1	WNT7A	up_AORT_VSMC
GO:0070227	0.0278	1	1	25	lymphocyte apoptotic process	BP	1	ARG2	up_AORT_VSMC
GO:0035051	0.0278	1	1	41	cardiocyte differentiation	BP	1	TBX5	up_APUL_VSMC
GO:0045907	0.0278	1	1	6	positive regulation of vasoconstriction	BP	1	AVPR1A	up_VFEM_EC
GO:0046621	0.0278	1	1	8	negative regulation of organ growth	BP	1	TBX5	up_VPUL_EC
GO:0003209	0.0278	1	1	14	cardiac atrium morphogenesis	BP	1	TBX5	up_VPUL_VSMC
GO:0003230	0.0278	1	1	14	cardiac atrium development	BP	1	TBX5	up_VPUL_VSMC
GO:0032771	0.027888604	1	1	1	regulation of monophenol monooxygenase activity	BP	0.96436359	CDH3	up_ACOR_EC
GO:0032773	0.027888604	1	1	1	positive regulation of monophenol monooxygenase activity	BP	0.96436359	CDH3	up_ACOR_EC
GO:0032912	0.027888604	1	1	1	negative regulation of transforming growth factor beta2 production	BP	0.96436359	CDH3	up_ACOR_EC
GO:0048023	0.027888604	1	1	1	positive regulation of melanin biosynthetic	BP	0.96436359	CDH3	up_ACOR_EC
GO:0051796	0.027888604	1	1	1	negative regulation of timing of catagen	BP	0.96436359	CDH3	up_ACOR_EC
GO:0060901	0.027888604	1	1	1	regulation of hair cycle by canonical Wnt signaling	BP	0.96436359	CDH3	up_ACOR_EC
GO:1900378	0.027888604	1	1	1	positive regulation of secondary metabolite	BP	0.96436359	CDH3	up_ACOR_EC
GO:1902908	0.027888604	1	1	1	regulation of melanosome transport	BP	0.96436359	CDH3	up_ACOR_EC
GO:1902910	0.027888604	1	1	1	positive regulation of melanosome transport	BP	0.96436359	CDH3	up_ACOR_EC
GO:0051128	0.0279	0.979	81	790	regulation of cellular component organization	BP	0.719	EPHB1,PLK2,TEK,HCLS1,GBP5,SELP,KIT,SELE,PRKCH,ARHGEF15,IFRD1,CDA,ATP8A1,PDGFB,PODXL,BAIAP2L1,RELN,IWS1,MEF2C,RAPGEF1,TLR4,GATA3,EDN1,RAB11A,RALB,ARAP3,RAB27A,SNCAIP,NEDD9,IRF8,ARHGAP28,TMOD1,PPP3CA,MTOR,SLK,BMP2K,CNOT6,PDZD8,NCKAP1,RIOK3,PROX1,UHRF2,IQGAP2,STMN1,HMBOX1,PRKD2,SH3GLB1,SWAP70,SMARCA4,APOD,NCK1,FES,NSD1,HIST1H1A,RAPGEF2,PLCG2,SENP6,PAN3,TAOK1,FMNL3,HMGB1,TBC1D1,PMAIP1,ARPC5L,SMC5,RAD21,ARHGDIB,ZW10,KDM5A,HIF1A,KDM3A,ATRX,ENSCAFG00000002015,DCP2,TMOD3,RABGAP1,ARHGEF2,FSCN1,FBXO5,USP6NL,ZMYM2	up_VCAV_EC
GO:0051239	0.0279	0.981	55	859	regulation of multicellular organismal process	BP	1	GPAM,NOTCH2,HSPB6,LRP1,DCN,PRNP,LUM,VASN,FBN1,GLIPR2,BMPR1A,TRPV2,NUMBL,CHID1,LCAT,TFE3,SYDE1,LAMA3,CLSTN1,PLCL1,SEMA3C,ASPN,LRPAP1,THY1,RNF216,PEX5,TLX2,B4GALT1,GLI3,PARK7,MAST2,ZFP36L1,MYDGF,SASH1,BPI,CYBA,MAVS,CDH2,LOXL3,SPRED1,RAB7B,IRX5,TSC22D3,UST,TWIST2,PRKCSH,DDR2,OMD,GREM1,FOXO3,THRB,ZBTB16,ANKRD54,RGMA,BRSK1	down_VCAV_EC
GO:0072359	0.0279	0.986	15	368	circulatory system development	BP	1	LEP,ANGPT4,HOXA7,DLL1,F3,TMEM100,GREM1,LUZP1,RECK,TBXA2R,BVES,PTPRB,AXIN2,ACACB,CLIC4	up_AFEM_EC
GO:0009060	0.0279	0.991	7	31	aerobic respiration	BP	1	DLST,ENSCAFG00000004260,FXN,MTFR1L,IDH1,IDH3A,ENSCAFG00000006639	up_VSAP_EC
GO:0006942	0.0279	0.992	6	26	regulation of striated muscle contraction	BP	1	FGF13,ADRA1B,KCNE3,PDE5A,DMPK,JUP	up_ACOR_VSMC
GO:0051153	0.0279	0.996	3	28	regulation of striated muscle cell differentiation	BP	1	DLL1,GREM1,DMPK	up_AFEM_EC

GO:0070848	0.0279	0.996	3	193	response to growth factor	BP	1	FLRT3,NEO1,MXRA5	up_VPUL_EC
GO:0070848	0.0279	1	1	193	response to growth factor	BP	1	MXRA5	down_AORT_VSMC
GO:0008593	0.0279	1	1	33	regulation of Notch signaling pathway	BP	1	GATA5	down_VSAP_VSMC
GO:0030837	0.0279	1	1	14	negative regulation of actin filament	BP	1	SLIT2	up_VPUL_VSMC
GO:1901862	0.0279	1	1	14	negative regulation of muscle tissue	BP	1	TBX5	up_VPUL_VSMC
GO:0032060	0.0279	1	1	6	bleb assembly	BP	1	EMP1	up_VSAP_VSMC
GO:0050804	0.027920086	0.9918	6	87	modulation of chemical synaptic transmission	BP	0.96436359	CNTNAP4,BRSK1,PLCL1,KCNB1,MAPK8IP2,LRRC4	up_ACOR_EC
GO:0099177	0.027920086	0.9918	6	87	regulation of trans-synaptic signaling	BP	0.96436359	CNTNAP4,BRSK1,PLCL1,KCNB1,MAPK8IP2,LRRC4	up_ACOR_EC
GO:0055006	0.028	0.994	5	18	cardiac cell development	BP	1	ACTN2,BMP4,NEBL,EDN1,BVES	up_ACOR_VSMC
GO:0045823	0.028	0.999	2	4	positive regulation of heart contraction	BP	0.72	EDN1,GCH1	up_VCAV_EC
GO:0002507	0.028	1	1	7	tolerance induction	BP	1	LYN	down_ACOR_EC
GO:0098693	0.028	1	1	25	regulation of synaptic vesicle cycle	BP	1	WNT7A	up_AORT_VSMC
GO:0021825	0.028	1	1	4	substrate-dependent cerebral cortex tangential	BP	1	SEMA3A	up_VPOR_EC
GO:0016101	0.028	1	1	13	diterpenoid metabolic process	BP	1	CYP26B1	up_VPOR_VSMC
GO:0042698	0.028	1	1	14	ovulation cycle	BP	1	SLIT2	up_VPUL_VSMC
GO:0048635	0.028	1	1	14	negative regulation of muscle organ	BP	1	TBX5	up_VPUL_VSMC
GO:0046958	0.028063966	1	1	1	nonassociative learning	BP	0.96436359	MAPK8IP2	up_ACOR_EC
GO:1901576	0.0281	0.995	6	1877	organic substance biosynthetic process	BP	1	TLR2,TBX15,EYA1,HOXA9,HOXA10,IRX5	up_AFEM_VSMC
GO:0051291	0.0281	0.993	5	21	protein heterooligomerization	BP	0.929	RRM1,C1QTNF6,RCC1,FARSA,PDSS1	down_ACOR_VSMC
GO:0030279	0.0281	0.994	5	19	negative regulation of ossification	BP	1	TWIST2,AXIN2,HDAC7,LRP4,LIMD1	up_VSAP_EC
GO:0045744	0.0281	0.995	4	15	negative regulation of G protein-coupled receptor signaling pathway	BP	0.72	CXCL8,APLNR,STMN1,RGS2	up_VCAV_EC
GO:0060536	0.0281	0.999	2	3	cartilage morphogenesis	BP	0.72	MEF2C,STC1	up_VCAV_EC
GO:0044090	0.0281	0.999	2	4	positive regulation of vacuole organization	BP	0.72	RALB,SH3GLB1	up_VCAV_EC
GO:2000786	0.0281	0.999	2	4	positive regulation of autophagosome assembly	BP	0.72	RALB,SH3GLB1	up_VCAV_EC
GO:0010977	0.0281	0.998	2	33	negative regulation of neuron projection	BP	1	SEMA3A,RNF6	up_VPOR_EC
GO:0007266	0.0281	0.998	2	64	Rho protein signal transduction	BP	1	TIAM1,CDC42EP5	up_VFEM_EC
GO:0010720	0.0281	0.998	2	110	positive regulation of cell development	BP	1	HOXA11,SHOX2	up_VFEM_VSMC
GO:0000028	0.0281	1	1	9	ribosomal small subunit assembly	BP	1	RPSA	down_ACOR_EC
GO:0043162	0.0281	1	1	7	ubiquitin-dependent protein catabolic process via the multivesicular body sorting pathway	BP	1	NEDD4	down_ACOR_EC
GO:0031076	0.0281	1	1	14	embryonic camera-type eye development	BP	1	CITED2	down_VPOR_EC
GO:0048645	0.0281	1	1	33	animal organ formation	BP	1	GATA5	down_VSAP_VSMC
GO:0048747	0.0281	1	1	20	muscle fiber development	BP	1	BEND2	up_AFEM_VSMC
GO:0016079	0.0281	1	1	27	synaptic vesicle exocytosis	BP	1	WNT7A	up_AORT_VSMC
GO:0048634	0.0281	1	1	40	regulation of muscle organ development	BP	1	TBX5	up_APUL_VSMC
GO:0048841	0.0281	1	1	4	regulation of axon extension involved in	BP	1	SEMA3A	up_VPOR_EC
GO:0048643	0.0281	1	1	7	positive regulation of skeletal muscle tissue	BP	1	CYP26B1	up_VFEM_EC
GO:0038060	0.028101498	1	1	1	nitric oxide-cGMP-mediated signaling	BP	0.96436359	GUCY1B1	up_ACOR_EC
GO:1900454	0.028122114	1	1	1	positive regulation of long-term synaptic depression	BP	0.96436359	KCNB1	up_ACOR_EC

GO:0032498	0.028131274	1	1	1	detection of muramyl dipeptide	BP	0.96436359	NOD2	up_ACOR_EC
GO:0032499	0.028131274	1	1	1	detection of peptidoglycan	BP	0.96436359	NOD2	up_ACOR_EC
GO:0032889	0.028164414	1	1	1	regulation of vacuole fusion, non-autophagic	BP	0.96436359	VPS16	up_ACOR_EC
GO:0010803	0.028186101	0.99799	2	10	regulation of tumor necrosis factor-mediated signaling pathway	BP	0.96436359	NR1H4,CCDC3	up_ACOR_EC
GO:0043069	0.0282	0.984	24	262	negative regulation of programmed cell death	BP	0.795	ARHGEF2,VEGFA,ASNS,NR1H4,PLK3,HSPA5,SERPINE1,HYOU1,AARS,FHL2,PTGS2,HIF1A,ZPR1,MTDH,CTNNB1,CRYAB,BNIP3,GSK3B,HTT,GLO1,PAK2,SIX4,NRP1,ARF4	down_VSAP_EC
GO:0071345	0.0282	0.993	5	210	cellular response to cytokine stimulus	BP	1	ARHGEF2,ENSCAFG00000002015,ASPN,ENSCAFG00000032731,GNPNAT1	up_VPOR_EC
GO:0051588	0.0282	0.996	3	28	regulation of neurotransmitter	BP	1	KCNC4,CHRM2,CAMK2A	up_AFEM_EC
GO:0007423	0.0282	0.996	3	155	sensory organ development	BP	1	SALL1,IRX5,MAB21L1	up_VSAP_VSMC
GO:0019219	0.0282	1	2	1151	regulation of nucleobase-containing compound metabolic process	BP	1	JUP,GATA5	down_VFEM_VSMC
GO:0140059	0.0282	0.999	2	3	dendrite arborization	BP	1	ZNF365,NRP1	up_ACOR_VSMC
GO:0032496	0.0282	0.998	2	77	response to lipopolysaccharide	BP	1	NR1H4,MRC1	up_VPUL_EC
GO:0006189	0.0282	1	1	4	'de novo' IMP biosynthetic process	BP	1	TNRC6B	down_ACOR_EC
GO:0031629	0.0282	1	1	8	synaptic vesicle fusion to presynaptic active zone membrane	BP	1	STX4	down_ACOR_EC
GO:0099500	0.0282	1	1	8	vesicle fusion to plasma membrane	BP	1	STX4	down_ACOR_EC
GO:0007507	0.0282	1	1	194	heart development	BP	1	GATA5	down_AFEM_VSMC
GO:0051271	0.0282	1	1	98	negative regulation of cellular component	BP	1	JUP	down_VFEM_VSMC
GO:0051452	0.0282	1	1	20	intracellular pH reduction	BP	1	CA2	up_VCAV_VSMC
GO:0009190	0.0282	1	1	8	cyclic nucleotide biosynthetic process	BP	1	ADCY3	up_VFEM_EC
GO:0052652	0.0282	1	1	8	cyclic purine nucleotide metabolic process	BP	1	ADCY3	up_VFEM_EC
GO:0071379	0.0282	1	1	8	cellular response to prostaglandin stimulus	BP	1	PTGER2	up_VPUL_EC
GO:0060043	0.0282	1	1	14	regulation of cardiac muscle cell proliferation	BP	1	TBX5	up_VPUL_VSMC
GO:1901052	0.028206196	1	1	1	sarcosine metabolic process	BP	0.96436359	SARDH	up_ACOR_EC
GO:1901053	0.028206196	1	1	1	sarcosine catabolic process	BP	0.96436359	SARDH	up_ACOR_EC
GO:0038190	0.028214623	1	1	1	VEGF-activated neuropilin signaling pathway	BP	0.96436359	NRP1	up_ACOR_EC
GO:0048842	0.028214623	1	1	1	positive regulation of axon extension involved in axon guidance	BP	0.96436359	NRP1	up_ACOR_EC
GO:0061441	0.028214623	1	1	1	renal artery morphogenesis	BP	0.96436359	NRP1	up_ACOR_EC
GO:1902336	0.028214623	1	1	1	positive regulation of retinal ganglion cell axon	BP	0.96436359	NRP1	up_ACOR_EC
GO:1902378	0.028214623	1	1	1	VEGF-activated neuropilin signaling pathway involved in axon guidance	BP	0.96436359	NRP1	up_ACOR_EC
GO:1902669	0.028214623	1	1	1	positive regulation of axon guidance	BP	0.96436359	NRP1	up_ACOR_EC
GO:1905040	0.028214623	1	1	1	otic placode development	BP	0.96436359	NRP1	up_ACOR_EC
GO:2000821	0.028214778	1	1	1	regulation of grooming behavior	BP	0.96436359	CNTNAP4	up_ACOR_EC
GO:0051695	0.028217394	1	1	1	actin filament uncapping	BP	0.96436359	ACTN2	up_ACOR_EC
GO:0086097	0.028217394	1	1	1	phospholipase C-activating angiotensin-activated signaling	BP	0.96436359	ACTN2	up_ACOR_EC
GO:2001137	0.028217394	1	1	1	positive regulation of endocytic recycling	BP	0.96436359	ACTN2	up_ACOR_EC
GO:0033624	0.028268997	1	1	1	negative regulation of integrin activation	BP	0.96436359	PTGER4	up_ACOR_EC
GO:0072682	0.028268997	1	1	1	eosinophil extravasation	BP	0.96436359	PTGER4	up_ACOR_EC

GO:2000417	0.028268997	1	1	1	negative regulation of eosinophil migration	BP	0.96436359	PTGER4	up_ACOR_EC
GO:2000419	0.028268997	1	1	1	regulation of eosinophil extravasation	BP	0.96436359	PTGER4	up_ACOR_EC
GO:2000420	0.028268997	1	1	1	negative regulation of eosinophil extravasation	BP	0.96436359	PTGER4	up_ACOR_EC
GO:0042221	0.0283	0.987	14	1012	response to chemical	BP	1	C3AR1,ARHGEF2,ENSCAFG00000002015,SLC7A11,SEMA3A,ASPN,ACVR2A,FBP1,MTDH,SEMA3D,SRP54,ENSCAFG00000032731,GNPNAT1,RNF6	up_VPOR_EC
GO:0007186	0.0283	0.991	7	124	G protein-coupled receptor signaling	BP	1	GPR1,CHRM2,HTR1D,CAMK2A,PTHLH,LGR6,CCL5	up_AFEM_EC
GO:0035967	0.0283	0.992	6	42	cellular response to topologically incorrect	BP	0.795	HSPA5,ERO1A,PTPN2,DDIT3,ATF3,CREB3L2	down_VSAP_EC
GO:0048675	0.0283	0.992	6	26	axon extension	BP	1	SLIT2,ABL1,PLXNA4,DPYSL2,TTL,ULK1	up_VSAP_EC
GO:0051353	0.0283	0.995	4	13	positive regulation of oxidoreductase activity	BP	1	HTR2B,ATP7A,EDN1,PARK7	up_ACOR_VSMC
GO:0045935	0.0283	0.995	4	491	positive regulation of nucleobase-containing compound metabolic	BP	1	EYA1,HOXA9,HOXA11,SHOX2	up_VFEM_VSMC
GO:0031498	0.0283	0.997	3	8	chromatin disassembly	BP	0.933	SMARCC1,SMARCB1,GRWD1	down_ACOR_VSMC
GO:0045824	0.0283	0.996	3	11	negative regulation of innate immune response	BP	0.795	PTPN2,CNOT7,A2M	down_VSAP_EC
GO:0050922	0.0283	0.996	3	11	negative regulation of chemotaxis	BP	0.795	PTPN2,MMP28,NRP1	down_VSAP_EC
GO:0070432	0.0283	0.999	2	3	regulation of nucleotide-binding oligomerization domain containing 2	BP	0.722	TNFAIP3,TLR4	up_VCAV_EC
GO:0070424	0.0283	0.999	2	3	regulation of nucleotide-binding oligomerization domain containing	BP	0.722	TNFAIP3,TLR4	up_VCAV_EC
GO:0051146	0.0283	0.998	2	76	striated muscle cell differentiation	BP	1	TBX5,MYBPC2	up_VPUL_EC
GO:0003015	0.0283	0.998	2	58	heart process	BP	1	IRX5,CAV1	up_VSAP_VSMC
GO:0031958	0.0283	1	1	5	corticosteroid receptor signaling pathway	BP	1	NEDD4	down_ACOR_EC
GO:0042921	0.0283	1	1	5	glucocorticoid receptor signaling pathway	BP	1	NEDD4	down_ACOR_EC
GO:0051293	0.0283	1	1	13	establishment of spindle localization	BP	1	GPSM2	down_AFEM_EC
GO:0015698	0.0283	1	1	24	inorganic anion transport	BP	1	SLC20A2	up_AORT_VSMC
GO:0106027	0.0283	1	1	24	neuron projection organization	BP	1	WNT7A	up_AORT_VSMC
GO:0032814	0.0283	1	1	7	regulation of natural killer cell activation	BP	1	TOX	up_VFEM_EC
GO:0071310	0.028326535	0.98369	25	634	cellular response to organic substance	BP	0.96436359	NR1H4,SOX9,AQP1,ENSCAFG00000004331,KCNB1,EDEM2,CCDC3,AGRN,NOD2,RGMA,SMAD6,ACTN2,PAK3,PTGER4,ADAMTSL2,MAVS,FOXC2,LRRC15,IFIT2,LRP4,LRRC4,RARRES2,PRKACA,NRP1,PRKCD	up_ACOR_EC
GO:1903756	0.028326724	1	1	1	regulation of transcription from RNA polymerase II promoter by histone	BP	0.96436359	ZMYND8	up_ACOR_EC
GO:1903758	0.028326724	1	1	1	negative regulation of transcription from RNA polymerase II promoter by histone modification	BP	0.96436359	ZMYND8	up_ACOR_EC
GO:0010557	0.0284	0.98	54	492	positive regulation of macromolecule biosynthetic process	BP	0.722	HCLS1,PPARG,TNIP1,PDGFB,TMEM173,MEF2C,ATF3,CHD7,TLR4,NLRC5,GATA3,EDN1,TCF4,FOXN2,ENSCAFG00000017103,IRF8,PPP3CA,YES1,MTOR,BMP2,HHEX,CREM,CNOT6,PROX1,ARID4B,HMBOX1,PRKD2,THOC1,SMARCA4,TLR3,NCK1,LDB2,JAG1,NSD1,BCL10,CNOT7,HMGB1,AHR,BLM,RIPK1,ITGA6,GPBP1,MTA2,NR1D2,KDM5A,HIF1A,KDM3A,ATRX,NR4A1,ZEB1,TET2,ARHGEF2,CDON,ZNF609	up_VCAV_EC
GO:0006366	0.0284	0.991	7	583	transcription by RNA polymerase II	BP	1	ACVRL1,HIVEP2,HOXA7,SULF1,ELK3,HOXA1,NEDD4	down_ACOR_EC
GO:0060688	0.0284	0.993	5	29	regulation of morphogenesis of a	BP	0.795	SULF1,HOXB7,MAGED1,CTNNB1,SIX4	down_VSAP_EC
GO:0019511	0.0284	0.998	2	5	peptidyl-proline hydroxylation	BP	0.795	P4HB,P4HA1	down_VSAP_EC
GO:0021825	0.0284	1	1	4	substrate-dependent cerebral cortex tangential	BP	1	NRP2	down_ACOR_EC

GO:0051642	0.0284	1	1	13	centrosome localization	BP	1	GPSM2	down_AFEM_EC
GO:0061842	0.0284	1	1	13	microtubule organizing center localization	BP	1	GPSM2	down_AFEM_EC
GO:0002027	0.0284	1	1	20	regulation of heart rate	BP	1	IRX5	up_AFEM_VSMC
GO:0060421	0.0284	1	1	14	positive regulation of heart growth	BP	1	TBX5	up_VPUL_VSMC
GO:0006139	0.0285	0.994	6	1909	nucleobase-containing compound metabolic	BP	1	TLR2,TBX15,EYA1,HOXA9,HOXA10,IRX5	up_AFEM_VSMC
GO:0045669	0.0285	0.994	4	24	positive regulation of osteoblast differentiation	BP	1	BMPR1A,CLSTN1,GLI3,DDR2	down_VCAV_EC
GO:0007599	0.0285	0.998	2	48	hemostasis	BP	1	LYN,PDPN	down_ACOR_EC
GO:0048265	0.0285	0.999	2	3	response to pain	BP	0.725	RELN,P2RX2	up_VCAV_EC
GO:0032836	0.0285	1	1	4	glomerular basement membrane development	BP	1	SULF1	down_ACOR_EC
GO:0060601	0.0285	1	1	6	lateral sprouting from an epithelium	BP	1	SULF1	down_ACOR_EC
GO:0034695	0.0285	1	1	8	response to prostaglandin E	BP	1	PTGER2	up_VPUL_EC
GO:0072359	0.0286	0.981	47	368	circulatory system development	BP	1	TNNT2,PDLIM3,VEGFD,SMAD6,ACTN2,LEP,GATA5,GATA4,ANGPT1,HTR2B,RECK,BMP4,FGFR2,GJA1,TLR3,SEC24B,XIRP1,CITED2,SPINT1,SASH1,LMO4,FGF18,NEBL,ANKRD1,EGLN1,NRP1,SMARCD3,SLITRK5,TBX20,TBX5,FRS2,PSEN1,TGFBR1,SYNJ2BP,CHD7,JUP,PSKH1,EDN1,HPGD,JAM3,AP2B1,GAA,ERAP1,BVES,CRKL,PIK3C2A,PTPN14	up_ACOR_VSMC
GO:0007050	0.0286	0.989	9	53	cell cycle arrest	BP	0.94	SLC29A1,PPM1G,CASP2,CDKN2C,MAP3K20,CDK4,BRCA1,PRR11,CDKN3	down_ACOR_VSMC
GO:0019935	0.0286	0.99	8	38	cyclic-nucleotide-mediated signaling	BP	1	ADRA1B,ADGRD1,HTR2B,ABCA1,PTGIR,ADCY6,APLP1,GNAQ	up_ACOR_VSMC
GO:0071900	0.0286	0.996	3	131	regulation of protein serine/threonine kinase	BP	1	LYN,IGF1R,PRKAR2A	down_ACOR_EC
GO:0016101	0.0286	0.996	3	13	diterpenoid metabolic process	BP	1	CYP1B1,RETSAT,RDH10	down_VCAV_EC
GO:0002376	0.0286	0.997	3	622	immune system process	BP	1	HOXA9,ARG2,ESR1	up_AORT_VSMC
GO:1990138	0.0286	0.998	2	35	neuron projection extension	BP	1	SEMA3A,RNF6	up_VPOR_EC
GO:0001938	0.0286	1	1	34	positive regulation of endothelial cell	BP	1	TGFBR1	down_VSAP_VSMC
GO:0009308	0.0286	1	1	20	amine metabolic process	BP	1	CYP1A1	up_VCAV_VSMC
GO:0003156	0.0286	1	1	20	regulation of animal organ formation	BP	1	GATA5	up_VCAV_VSMC
GO:0010646	0.0287	0.978	108	945	regulation of cell communication	BP	1	SH3TC2,ADRA1B,TLR1,SMAD6,LGR6,LEP,PLCL1,LBH,MID1,GATA5,HTR2B,RASSF2,ZRANB1,FBXL20,C28H10orf71,RECK,SKP2,NACC2,PRKAA2,MLLT3,ITM2C,FERMT1,BMP4,TM7SF3,FGFR2,ABCA1,ELF1,HRH1,CASTOR1,TLR3,SH3RF2,LATS2,PDE5A,CITED2,TP53INP1,ZNRF3,AR,PTGIR,SASH1,TMEM108,FGF18,ARHGEF26,NDRG2,CHN2,CDKN2B,ITPR1,LIMS2,ANKRD1,F2RL1,DAPK1,BNIP3,EGLN1,CRLF3,ENSCAFG00000006238,CLSTN1,CREBRF,NRP1,UBR1,PTPN22,DMPK,NPNT,UNC5B,SMPD1,APLP1,TBX20,SESN3,SH3RF1,CCDC3,PRKCE,FRS2,RNF19A,SCG5,RGS2,DAG1,PARP9,APP,PSEN1,SYNJ2BP,CHD7,SIPA1L1,CCL5,WWC2,DUSP22,SIAH1,EDN1,SESN1,KCNC4,DUSP26,NOD2,LRRC4,FAM107A,DDIT4,DISC1,SH3RF3,SERINC3,USP20,HERPUD1,MAP3K7,INPP5K,PREPL,BMT2,PFKL,WFS1,SH3BP4,ENSCAFG00000029553,PARK7,IL18R1,CRKL	up_ACOR_VSMC
GO:0051094	0.0287	0.982	33	388	positive regulation of developmental process	BP	0.798	ARHGEF2,VEGFA,CELF1,NR1H4,SERPINE1,SOCS5,JCAD,DPYSL3,ILK,PTGS2,PIK3C2A,HOXB7,HIF1A,EVI2B,MTDH,MAGED1,CTNNB1,ENSCAFG00000002015,TFRC,APOE,KLF5,LYN,BNIP3,MMD,VLDLR,REST,PRMT1,DISC1,LRRTM2,INHBA,HIF1AN,SIX4,NRP1	down_VSAP_EC
GO:0032412	0.0287	0.989	9	46	regulation of ion transmembrane	BP	1	ACTN2,PLCG2,KCNE3,TCAF1,PTPN3,DAPK1,CACNB3,AKAP6,FXYD1	up_ACOR_VSMC
GO:0002761	0.0287	0.991	7	36	regulation of myeloid leukocyte differentiation	BP	0.727	HCLS1,CASP8,MTOR,CREB1,RIPK1,CUL4A,TJP2	up_VCAV_EC
GO:0071346	0.0287	0.992	6	41	cellular response to interferon-gamma	BP	0.798	FLNB,MYO1C,KIF5B,PTPN2,CDC37,GBP5	down_VSAP_EC

GO:0030239	0.0287	0.993	5	21	myofibril assembly	BP	0.727	MEF2C,EDN1,TMOD1,PROX1,TMOD3	up_VCAV_EC
GO:0019233	0.0287	0.995	4	16	sensory perception of pain	BP	0.727	EPHB1,HOXB8,ACPP,MMP24	up_VCAV_EC
GO:0042023	0.0287	0.997	3	8	DNA endoreduplication	BP	0.94	SMC1A,E2F8,SMC3	down_ACOR_VSMC
GO:0098801	0.0287	0.997	3	8	regulation of renal system process	BP	1	F2RL1,EDN1,INPP5K	up_ACOR_VSMC
GO:0035437	0.0287	0.998	2	5	maintenance of protein localization in endoplasmic reticulum	BP	0.798	HSPA5,KDELR2	down_VSAP_EC
GO:0050686	0.0287	1	1	13	negative regulation of mRNA processing	BP	1	SRSF6	down_AFEM_EC
GO:0035107	0.0288	0.991	7	60	appendage morphogenesis	BP	1	BMPR1A,RECK,SEMA3C,GLI3,RDH10,GREM1,ZBTB16	down_VCAV_EC
GO:0035108	0.0288	0.991	7	60	limb morphogenesis	BP	1	BMPR1A,RECK,SEMA3C,GLI3,RDH10,GREM1,ZBTB16	down_VCAV_EC
GO:0072527	0.0288	0.993	5	23	pyrimidine-containing compound metabolic	BP	0.727	CDA,MTOR,ACPP,DCK,TET2	up_VCAV_EC
GO:0046638	0.0288	0.995	4	13	positive regulation of alpha-beta T cell	BP	1	ZBTB16,CD83,PNP,TGFBR2	up_VSAP_EC
GO:0048048	0.0288	0.995	4	13	embryonic eye morphogenesis	BP	1	RARG,ZEB1,KDM2B,CITED2	up_VSAP_EC
GO:0009583	0.0288	0.998	2	5	detection of light stimulus	BP	0.799	GNAQ,RDH11	down_VSAP_EC
GO:0016311	0.0288	0.998	2	106	dephosphorylation	BP	1	EYA1,FBP1	up_VFEM_VSMC
GO:0040013	0.0288	1	1	100	negative regulation of locomotion	BP	1	JUP	down_VFEM_VSMC
GO:0110110	0.0288	1	1	34	positive regulation of animal organ	BP	1	GATA5	down_VSAP_VSMC
GO:0065009	0.028829351	0.98288	28	735	regulation of molecular function	BP	0.97940892	CDH3,NR1H4,PLCL1,PKIB,PCSK1N,ENSCAFG00000004331,FZD5,IFI6,DUSP26,NOD2,DYNC1I1,ACTN2,ARHGAP28,PAK3,CST3,MAPK8IP2,MAVS,LRRC15,IFIT2,CLSTN1,LRRC4,CLU,PRKCD,PPP1R26,PM20D1,PYGO2,DBNDD2,CCNYL1	up_ACOR_EC
GO:0055088	0.0289	0.991	7	37	lipid homeostasis	BP	0.946	SREBF2,RALY,INSIG1,CAV1,LIPC,NUS1,THADA	down_ACOR_VSMC
GO:0001570	0.0289	0.991	7	37	vasculogenesis	BP	0.727	AMOT,CD34,TIPARP,APLNR,SOX18,HHEX,RAPGEF2	up_VCAV_EC
GO:0003208	0.0289	0.992	6	24	cardiac ventricle morphogenesis	BP	1	TNNT2,FGFR2,SMARCD3,TBX20,TBX5,CHD7	up_ACOR_VSMC
GO:0060042	0.0289	0.996	3	14	retina morphogenesis in camera-type eye	BP	1	THY1,IRX5,THRB	down_VCAV_EC
GO:0017157	0.0289	0.998	2	58	regulation of exocytosis	BP	1	LYN,STX4	down_ACOR_EC
GO:0015813	0.0289	0.999	2	3	L-glutamate transmembrane transport	BP	1	PSEN1,CLN8	up_ACOR_VSMC
GO:0051938	0.0289	0.999	2	3	L-glutamate import	BP	1	PSEN1,CLN8	up_ACOR_VSMC
GO:0098712	0.0289	0.999	2	3	L-glutamate import across plasma membrane	BP	1	PSEN1,CLN8	up_ACOR_VSMC
GO:0061209	0.0289	0.999	2	4	cell proliferation involved in mesonephros	BP	0.727	GATA3,BMP2	up_VCAV_EC
GO:1903409	0.0289	1	1	24	reactive oxygen species biosynthetic process	BP	1	ARG2	up_AORT_VSMC
GO:1901861	0.0289	1	1	41	regulation of muscle tissue development	BP	1	TBX5	up_APUL_VSMC
GO:0002682	0.029	0.996	3	343	regulation of immune system process	BP	1	TMEM176A,TMEM176B,SLIT2	up_VPUL_VSMC
GO:0032836	0.029	0.999	2	4	glomerular basement membrane development	BP	0.803	SULF1,MYO1E	down_VSAP_EC
GO:0042692	0.029	0.998	2	105	muscle cell differentiation	BP	1	NKX2-5,CYP26B1	up_VPOR_VSMC
GO:0002902	0.029	1	1	6	regulation of B cell apoptotic process	BP	1	LYN	down_ACOR_EC
GO:0032233	0.029	1	1	21	positive regulation of actin filament bundle	BP	1	LIMK1	up_VCAV_VSMC
GO:1900027	0.029	1	1	6	regulation of ruffle assembly	BP	1	CAV1	up_VSAP_VSMC
GO:2000026	0.0291	0.981	38	560	regulation of multicellular organismal development	BP	1	NOTCH2,HSPB6,DCN,VASN,FBN1,GLIPR2,BMPR1A,TRPV2,NUMBL,TFE3,SYDE1,LAMA3,CLSTN1,SEMA3C,ASPN,THY1,TLX2,B4GALT1,GLI3,ZFP36L1,MYDGF,SASH1,CDH2,LOXL3,SPRED1,RAB7B,TSC22D3,UST,PRKCSH,DDR2,OMD,GREM1,FOXO3,THRB,ZBTB16,ANKRD54,RGMA,BRSK1	down_VCAV_EC

GO:0030154	0.0291	0.982	34	1040	cell differentiation	BP	1	HOXA9,CMKLR1,IRX3,IRX5,HOXC10,LEP,LBH,GAS1,HOXA7,DLL1,VXN,PLXNA4,NEFH,TWIST2,THRB,LGI4,HCK,TMEM100,GREM1,NFE2L1,CAMK2A,CYLD,MYBL1,PTHLH,EVI2B,BVES,AXIN2,NAPEPLD,PPARGC1A,DMPK,LRRN2,FOSL2,KANK1,TNC	up_AFEM_EC
GO:0031346	0.0291	0.989	9	73	positive regulation of cell projection organization	BP	0.803	FSCN1,DPYSL3,DYNLL1,ANLN,APOE,LYN,VLDLR,HTT,NRP1	down_VSAP_EC
GO:0055007	0.0291	0.991	7	31	cardiac muscle cell differentiation	BP	1	ACTN2,BMP4,NEBL,TBX5,FRS2,EDN1,BVES	up_ACOR_VSMC
GO:1902895	0.0291	0.995	4	15	positive regulation of pri-miRNA transcription by RNA polymerase II	BP	0.731	PDGFB,BMP2,SMARCA4,HIF1A	up_VCAV_EC
GO:0072079	0.0291	0.996	3	12	nephron tubule formation	BP	0.803	ENSCAFG00000032470,CTNNB1,SIX4	down_VSAP_EC
GO:0001845	0.0291	1	1	5	phagolysosome assembly	BP	1	SPG11	down_ACOR_EC
GO:0021854	0.0291	1	1	6	hypothalamus development	BP	1	NRP2	down_ACOR_EC
GO:0060968	0.0291	1	1	23	regulation of gene silencing	BP	1	ESR1	up_AORT_VSMC
GO:0044706	0.0291	1	1	27	multi-multicellular organism process	BP	1	ESR1	up_AORT_VSMC
GO:1902668	0.0291	1	1	4	negative regulation of axon guidance	BP	1	SEMA3A	up_VPOR_EC
GO:2000144	0.0291	1	1	12	positive regulation of DNA-templated transcription,	BP	1	NKX2-5	up_VPOR_VSMC
GO:0060546	0.0291	1	1	7	negative regulation of necroptotic process	BP	1	CAV1	up_VSAP_VSMC
GO:0060547	0.0291	1	1	7	negative regulation of necrotic cell death	BP	1	CAV1	up_VSAP_VSMC
GO:1901631	0.02912899	1	1	1	positive regulation of presynaptic membrane	BP	0.98132464	LRP4	up_ACOR_EC
GO:1903911	0.02912899	1	1	1	positive regulation of receptor clustering	BP	0.98132464	LRP4	up_ACOR_EC
GO:1904395	0.02912899	1	1	1	positive regulation of skeletal muscle acetylcholine-gated	BP	0.98132464	LRP4	up_ACOR_EC
GO:1904398	0.02912899	1	1	1	positive regulation of neuromuscular junction development	BP	0.98132464	LRP4	up_ACOR_EC
GO:0009063	0.0292	0.992	6	25	cellular amino acid catabolic process	BP	1	GOT1,ADHFE1,ALDH6A1,BCKDHB,ENSCAFG00000002977,IDO2	up_VSAP_EC
GO:0002792	0.0292	0.993	5	29	negative regulation of peptide secretion	BP	0.804	NR1H4,PTPN11,REST,RAB11FIP1,RHBDF2	down_VSAP_EC
GO:0010499	0.0292	0.999	2	3	proteasomal ubiquitin-independent protein catabolic process	BP	0.731	PSME4,ENC1	up_VCAV_EC
GO:0050891	0.0292	1	1	12	multicellular organismal water homeostasis	BP	1	CYP26B1	up_VPOR_VSMC
GO:0060260	0.0292	1	1	12	regulation of transcription initiation from RNA polymerase II promoter	BP	1	NKX2-5	up_VPOR_VSMC
GO:0035265	0.0293	0.988	10	54	organ growth	BP	1	ACACB,SLC6A4,FGF7,DLL1,SAV1,RARG,DDR2,FXN,TGFBR2,LATS2	up_VSAP_EC
GO:0090050	0.0293	0.995	4	15	positive regulation of cell migration involved in sprouting angiogenesis	BP	0.731	PLK2,PIK3C2A,MAP3K3,JCAD	up_VCAV_EC
GO:0010557	0.0293	0.994	4	492	positive regulation of macromolecule biosynthetic process	BP	1	EYA1,HOXA9,HOXA11,SHOX2	up_VFEM_VSMC
GO:0046456	0.0293	0.997	3	8	icosanoid biosynthetic process	BP	0.731	EDN1,PTGS1,PTGS2	up_VCAV_EC
GO:0007178	0.0293	0.998	2	107	transmembrane receptor protein serine/threonine kinase signaling pathway	BP	1	NKX2-5,BAMBI	up_VPOR_VSMC
GO:0007019	0.0293	1	1	13	microtubule depolymerization	BP	1	TAOK1	up_AORT_EC
GO:0032386	0.029333462	0.98941	8	137	regulation of intracellular transport	BP	0.98615433	SH3TC2,CDH3,KCNB1,EDEM2,ACTN2,PREB,MAVS,PRKCD	up_ACOR_EC
GO:0001503	0.0294	0.985	17	109	ossification	BP	1	FZD1,PEX7,TWIST2,WNT11,AXIN2,OSR2,PPARGC1B,ZHX3,HDAC7,OSR1,ZBTB16,DDR2,OMD,SHOX2,LRP4,LIMD1,BGLAP	up_VSAP_EC
GO:0050731	0.0294	0.99	8	44	positive regulation of peptidyl-tyrosine	BP	0.731	HCLS1,KIT,PDGFB,RELN,NEDD9,YES1,MTOR,ARHGEF2	up_VCAV_EC
GO:0045778	0.0294	0.993	5	35	positive regulation of ossification	BP	1	BMPR1A,CLSTN1,GLI3,DDR2,ZBTB16	down_VCAV_EC
GO:0002720	0.0294	0.995	4	13	positive regulation of cytokine production involved in immune	BP	1	F2RL1,B2M,MAP3K7,IL18R1	up_ACOR_VSMC

GO:0097150	0.0294	0.995	4	13	neuronal stem cell population maintenance	BP	0.731	HOOK3,PROX1,MMP24,JAG1	up_VCAV_EC
GO:0071498	0.0294	0.997	3	8	cellular response to fluid shear stress	BP	0.731	MEF2C,MTSS1,PTGS2	up_VCAV_EC
GO:0031326	0.0294	1	2	1176	regulation of cellular biosynthetic process	BP	1	JUP,GATA5	down_VFEM_VSMC
GO:0007155	0.0294	0.998	2	324	cell adhesion	BP	1	TGFBI,GATA5	down_VSAP_VSMC
GO:0061438	0.0294	0.999	2	3	renal system vasculature morphogenesis	BP	1	BMP4,NRP1	up_ACOR_VSMC
GO:0061439	0.0294	0.999	2	3	kidney vasculature morphogenesis	BP	1	BMP4,NRP1	up_ACOR_VSMC
GO:0061551	0.0294	1	1	5	trigeminal ganglion development	BP	1	NRP2	down_ACOR_EC
GO:0090050	0.0294	1	1	15	positive regulation of cell migration involved in sprouting angiogenesis	BP	1	HDAC7	down_VPOR_EC
GO:0003156	0.0294	1	1	20	regulation of animal organ formation	BP	1	EYA1	up_AFEM_VSMC
GO:0050806	0.0294	1	1	25	positive regulation of synaptic transmission	BP	1	WNT7A	up_AORT_VSMC
GO:0106049	0.0294	1	1	4	regulation of cellular response to osmotic	BP	1	ARHGEF2	up_VPOR_EC
GO:1902218	0.0294	1	1	4	regulation of intrinsic apoptotic signaling pathway in response to	BP	1	ARHGEF2	up_VPOR_EC
GO:1902219	0.0294	1	1	4	negative regulation of intrinsic apoptotic signaling pathway in	BP	1	ARHGEF2	up_VPOR_EC
GO:0032940	0.0295	0.982	31	231	secretion by cell	BP	1	EXPH5,TLR1,LEP,HTR2B,MBP,FBXL20,FERMT1,SYT16,TM7SF3,ABCA1,CD274,STX11,ANKRD1,F2RL1,PTPN22,PRKCE,SCG5,PSEN1,PCSK6,CHD7,CCL5,RAB11B,EDN1,KCNC4,NOD2,SCAMP5,SCRN2,PREPL,PFKL,PARK7,LMF1	up_ACOR_VSMC
GO:0010605	0.0295	0.99	8	745	negative regulation of macromolecule metabolic	BP	1	ACVRL1,LYN,IGF1R,HOXA7,PRKAR2A,ELK3,ASPN,NEDD4	down_ACOR_EC
GO:0002376	0.0295	0.991	7	622	immune system process	BP	1	LYN,HOXA3,IGF1R,PDPN,HOXA7,IPO7,STX4	down_ACOR_EC
GO:0002042	0.0295	0.993	5	29	cell migration involved in sprouting angiogenesis	BP	0.81	VEGFA,JCAD,PIK3C2A,STARD13,NRP1	down_VSAP_EC
GO:1902043	0.0295	0.996	3	9	positive regulation of extrinsic apoptotic signaling pathway via	BP	0.731	ATF3,PMAIP1,SKIL	up_VCAV_EC
GO:0060836	0.0295	0.998	2	4	lymphatic endothelial cell differentiation	BP	0.731	SOX18,PROX1	up_VCAV_EC
GO:0050905	0.0295	1	1	26	neuromuscular process	BP	1	HOXC10	up_AORT_VSMC
GO:0051148	0.0295	1	1	12	negative regulation of muscle cell differentiation	BP	1	NKX2-5	up_VPOR_VSMC
GO:0000045	0.02955579	0.99386	4	46	autophagosome assembly	BP	0.98664651	ATG16L2,CHMP4B,TP53INP2,ENSCAFG00000010493	up_ACOR_EC
GO:0060249	0.029584643	0.99018	7	110	anatomical structure homeostasis	BP	0.98664651	CDH3,PKIB,SOX9,PTH1R,NOD2,CHMP4B,TP53INP2	up_ACOR_EC
GO:0071326	0.02959267	0.99565	3	26	cellular response to monosaccharide stimulus	BP	0.98664651	NR1H4,KCNB1,PRKACA	up_ACOR_EC
GO:0071331	0.02959267	0.99565	3	26	cellular response to hexose stimulus	BP	0.98664651	NR1H4,KCNB1,PRKACA	up_ACOR_EC

GO:0032501	0.0296	0.976	194	1782	multicellular organismal process	BP	1	EXPH5,TNNT2,SH3TC2,PDLIM3,FGF13,ADRA1B,VEGFD,TMEM176B,TLR1,TMEM176A,SMAD6,ACTN2,PLCG2,LEP,PLCL1,LBH,AFF3,SLC26A7,KCNE3,GATA5,GATA4,CD83,ANGPT1,EPYC,HTR2B,RASSF2,MBP,FBXL20,PRDX4,RECK,SHROOM3,FEZ1,COCH,MLLT3,ITM2C,FERMT1,BMP4,LCAT,LNPK,TBX18,NPPB,FGFR2,TP53BP1,ABCA1,GJA1,ELF1,ATRX,MED14,HRH1,TLR3,LRRN4,SEC24B,XIRP1,WASF3,PPARGC1A,LATS2,PDE5A,CITED2,SPINT1,GLRB,ISG15,ACSL6,ZNRF3,CD274,ZNF365,SASH1,SLC2A5,GPR155,LMO4,TMEM108,FGF18,FAM210B,NDRG2,ATRN,P4HTM,NEBL,CDKN2B,ADCY6,USP53,EBF4,ZMYM2,ITPR1,PLAC8B,LIMS2,ANKRD1,F2RL1,HRH2,CAMK2D,ENPP4,CTSB,RBM4B,EGLN1,ENSCAFG00000006238,CLSTN1,CREBRF,ABCA4,NRP1,PTPN22,GPM6A,CHD2,LRPAP1,ULK2,DMPK,NPNT,UNC5B,SMARCD3,EVL,SPINT2,SLITRK5,PTGDR,TBX20,BBX,TBX5,PRKCE,MARF1,FZD3,AFF4,DKKL1,FRS2,CPAMD8,RND1,RNLS,DAG1,FUT8,POMK,APP,LAMA3,PSEN1,SEMA6D,TGFBR1,PCSK6,SYNJ2BP,CHD7,JUP,SPINK2,SIPA1L1,DDR2,CLN8,WWC2,FOSL2,PERP,PSKH1,RTKN2,SIAH1,SSH3,C10H2orf49,EDN1,OXR1,NID1,HPGD,NOD2,ARID5B,ASAP1,FAM107A,DMC1,INSYN1,SERPINB5,JAM3,	up_ACOR_VSMC
GO:0051489	0.0296	1	1	14	regulation of filopodium assembly	BP	1	FSCN1	down_AFEM_EC
GO:0003283	0.0296	1	1	8	atrial septum development	BP	1	TBX5	up_APUL_EC
GO:0060413	0.0296	1	1	8	atrial septum morphogenesis	BP	1	TBX5	up_APUL_EC
GO:0043500	0.0296	1	1	21	muscle adaptation	BP	1	GATA5	up_VCAV_VSMC
GO:0006357	0.0297	0.979	59	547	regulation of transcription by RNA polymerase II	BP	0.733	EDNRB,HCLS1,FLI1,PPARG,TNIP1,PDGFB,TMEM173,MEF2C,ATF3,CHD7,DLL4,TLR4,NLRC5,GATA3,EDN1,HOXB8,TCF4,SMAD1,ENSCAFG00000017103,IRF8,SOX18,PPP3CA,YES1,ELF4,BMP2,HMG20A,HHEX,CREM,PROX1,ARID4B,HMBOX1,PRKD2,SMARCA4,TLR3,NCK1,TIMELESS,LDB2,JAG1,TCERG1,CTDSPL2,CNOT7,HMGB1,AHR,RIPK1,ITGA6,SKIL,MTA2,KDM5A,HIF1A,KDM3A,ATRX,NR4A1,ARID5A,ZEB1,TET2,ARHGEF2,CDON,ELK3,ZNF609	up_VCAV_EC
GO:1901566	0.0297	0.98	41	598	organonitrogen compound biosynthetic process	BP	1	GPAM,UBA52,ADCY6,LARP6,COA1,KRTCAP2,CLIP3,LCAT,SAMD4B,RPL23,RPL13,DDOST,UROS,ATP5MC1,GFPT2,PIGS,EIF3K,NECAB3,ADK,WDR45,MRPL37,B4GALT1,PARK7,RPL27A,EEF2,ZFP36L1,RPL27,AMPD2,MRPS11,ORMDL3,RPS27,FUT8,ACSS2,ENSCAFG00000015122,PYM1,RPN2,GLS2,FOXO3,SOAT1,EIF4EBP1,ACAT1	down_VCAV_EC
GO:0031345	0.0297	0.989	9	46	negative regulation of cell projection organization	BP	1	ITM2C,EVI5L,ZNF365,NRP1,ULK2,EVL,PSEN1,ASAP1,B2M	up_ACOR_VSMC
GO:0016485	0.0297	0.989	8	73	protein processing	BP	1	ADAMTS2,RHBDL1,PCSK7,GLI3,SEC11C,A2M,PCSK6,LONP2	down_VCAV_EC
GO:0007275	0.0297	0.992	7	1370	multicellular organism development	BP	1	HOXC10,DKK2,HOXA9,HOXC6,SGCD,TAOK1,DUSP2	up_AORT_EC
GO:0030182	0.0297	0.993	5	327	neuron differentiation	BP	1	LYN,ACSL4,SPG11,NRP2,NEDD4	down_ACOR_EC
GO:0042100	0.0297	0.994	4	19	B cell proliferation	BP	0.811	TFRC,LYN,BTK,CDKN1A	down_VSAP_EC
GO:1903672	0.0297	0.994	4	20	positive regulation of sprouting angiogenesis	BP	0.811	VEGFA,JCAD,PIK3C2A,NRP1	down_VSAP_EC
GO:0022610	0.0297	0.998	2	326	biological adhesion	BP	1	TGFBI,GATA5	down_VSAP_VSMC
GO:0034380	0.0297	0.999	2	3	high-density lipoprotein particle assembly	BP	1	LCAT,ABCA1	up_ACOR_VSMC
GO:0021563	0.0297	1	1	4	glossopharyngeal nerve development	BP	1	HOXA3	down_ACOR_EC
GO:0051963	0.0297	1	1	26	regulation of synapse assembly	BP	1	WNT7A	up_AORT_VSMC
GO:0006959	0.0297	1	1	21	humoral immune response	BP	1	C6	up_VCAV_VSMC
GO:0045851	0.0297	1	1	21	pH reduction	BP	1	CA2	up_VCAV_VSMC
GO:0001960	0.0297	1	1	15	negative regulation of cytokine-mediated	BP	1	SLIT2	up_VPUL_VSMC

GO:0031960	0.0297	1	1	15	response to corticosteroid	BP	1	SLIT2	up_VPUL_VSMC
GO:0051384	0.0297	1	1	15	response to glucocorticoid	BP	1	SLIT2	up_VPUL_VSMC
GO:0060761	0.0297	1	1	15	negative regulation of response to cytokine	BP	1	SLIT2	up_VPUL_VSMC
GO:0031128	0.0297	1	1	15	developmental induction	BP	1	GATA5	up_VPUL_VSMC
GO:0060707	0.0297	1	1	7	trophoblast giant cell differentiation	BP	1	GJB5	up_VSAP_VSMC
GO:0032870	0.029728892	0.98848	9	162	cellular response to hormone stimulus	BP	0.98710931	NR1H4,AQP1,ENSCAFG00000004331,AGRN, ACTN2,PTGER4,FOXC2,RARRES2,PRKCD	up_ACOR_EC
GO:0007204	0.0298	0.99	8	46	positive regulation of cytosolic calcium ion	BP	0.735	PDE4D,CHD7,GPR4,EDN1,APLNR,SWAP70,PL CG2,HMGB1	up_VCAV_EC
GO:0050793	0.0298	0.993	5	731	regulation of developmental process	BP	1	NKX2-5,BAMBI,ANKRD6,CYP26B1,C3AR1	up_VPOR_VSMC
GO:0034394	0.0298	0.993	5	19	protein localization to cell surface	BP	1	WNT11,FGF7,EMP2,RAB11B,STX4	up_VSAP_EC
GO:0009411	0.0298	0.998	2	56	response to UV	BP	1	USP28,NEDD4	down_ACOR_EC
GO:0021537	0.0298	0.998	2	73	telencephalon development	BP	1	SLITRK5,NTRK2	up_APUL_EC
GO:0051099	0.0298	0.998	2	70	positive regulation of binding	BP	1	HOXA3,PLCL1	up_APUL_EC
GO:0051099	0.0298	1	1	70	positive regulation of binding	BP	1	PLCL1	down_VFEM_EC
GO:0048771	0.0298	1	1	43	tissue remodeling	BP	1	HOXA3	up_APUL_VSMC
GO:2000649	0.0298	1	1	11	regulation of sodium ion transmembrane transporter activity	BP	1	NETO1	up_VFEM_VSMC
GO:0034139	0.0298	1	1	7	regulation of toll-like receptor 3 signaling	BP	1	CAV1	up_VSAP_VSMC
GO:1901576	0.0299	0.991	9	1877	organic substance biosynthetic process	BP	1	EYA1,GATM,HOXA10,HOXA9,TBX15,FBP1,EM X2,HOXA11,SHOX2	up_VFEM_VSMC
GO:0006487	0.0299	0.993	5	34	protein N-linked glycosylation	BP	1	DDOST,GFPT2,B4GALT1,FUT8,RPN2	down_VCAV_EC
GO:0050865	0.0299	0.996	3	135	regulation of cell activation	BP	1	LYN,PDPN,STX4	down_ACOR_EC
GO:1905953	0.0299	0.996	3	12	negative regulation of lipid localization	BP	0.813	PTPN11,PTPN2,ABHD5	down_VSAP_EC
GO:0045944	0.0299	0.996	3	287	positive regulation of transcription by RNA polymerase II	BP	1	EYA1,HOXA9,SHOX2	up_VFEM_VSMC
GO:0050817	0.0299	0.998	2	50	coagulation	BP	1	LYN,PDPN	down_ACOR_EC
GO:0002756	0.0299	0.999	2	3	MyD88-independent toll-like receptor signaling	BP	1	TLR3,PRKCE	up_ACOR_VSMC
GO:0085020	0.0299	1	1	4	protein K6-linked ubiquitination	BP	1	RNF6	up_VPOR_EC
GO:0030278	0.029970569	0.99227	5	66	regulation of ossification	BP	0.9930905	SOX9,SMAD6,PTGER4,LRP4,CLSTN1	up_ACOR_EC
GO:0003170	0.03	0.992	6	25	heart valve development	BP	1	SMAD6,GATA5,GATA4,BMP4,TBX20,TBX5	up_ACOR_VSMC
GO:0050773	0.03	0.993	5	29	regulation of dendrite development	BP	0.813	SIPA1L1,TNIK,APOE,VLDLR,YWHAH	down_VSAP_EC
GO:0051208	0.03	0.993	5	29	sequestering of calcium ion	BP	0.813	ERO1A,LYN,DDIT3,HTT,CALM1	down_VSAP_EC
GO:0051282	0.03	0.993	5	29	regulation of sequestering of calcium ion	BP	0.813	ERO1A,LYN,DDIT3,HTT,CALM1	down_VSAP_EC
GO:0051240	0.03	0.994	4	489	positive regulation of multicellular organismal	BP	1	NKX2-5,BAMBI,CYP26B1,C3AR1	up_VPOR_VSMC
GO:0009889	0.03	1	2	1190	regulation of biosynthetic process	BP	1	JUP,GATA5	down_VFEM_VSMC
GO:0071361	0.03	0.999	2	3	cellular response to ethanol	BP	1	TP53INP1,PRKCE	up_ACOR_VSMC
GO:1901381	0.03	0.999	2	3	positive regulation of potassium ion	BP	1	ACTN2,AKAP6	up_ACOR_VSMC
GO:1902523	0.03	0.999	2	3	positive regulation of protein K63-linked	BP	1	PTPN22,NOD2	up_ACOR_VSMC
GO:0045765	0.03	1	1	105	regulation of angiogenesis	BP	1	JUP	down_VFEM_VSMC
GO:0061036	0.03	1	1	11	positive regulation of cartilage development	BP	1	HOXA11	up_VFEM_VSMC
GO:0048869	0.0301	0.99	8	1119	cellular developmental process	BP	1	TBX5,EVI2B,MYBPC2,WIF1,CCL24,FLRT3,ARH GEF2,NEO1	up_VPUL_EC
GO:0006612	0.0301	0.992	5	35	protein targeting to membrane	BP	1	PRNP,AKT2,SSR2,SSR3,SEC61B	down_VCAV_EC
GO:0086003	0.0301	0.996	3	10	cardiac muscle cell contraction	BP	0.739	PDE4D,STC1,KCNJ2	up_VCAV_EC

GO:0006906	0.0301	0.998	2	55	vesicle fusion	BP	1	SPG11,STX4	down_ACOR_EC
GO:0015849	0.0301	0.998	2	56	organic acid transport	BP	1	PDPN,ACSL3	down_ACOR_EC
GO:0046942	0.0301	0.998	2	56	carboxylic acid transport	BP	1	PDPN,ACSL3	down_ACOR_EC
GO:0002673	0.0301	0.998	2	6	regulation of acute inflammatory response	BP	1	PARK7,A2M	down_VCAV_EC
GO:0007213	0.0301	0.998	2	5	G protein-coupled acetylcholine receptor	BP	0.813	AGRN,GNAQ	down_VSAP_EC
GO:1903525	0.0301	0.999	2	3	regulation of membrane tubulation	BP	1	ASAP1,WASL	up_ACOR_VSMC
GO:0021602	0.0301	1	1	16	cranial nerve morphogenesis	BP	1	CITED2	down_VPOR_EC
GO:0035115	0.0301	1	1	12	embryonic forelimb morphogenesis	BP	1	HOXA9	up_AORT_EC
GO:0051588	0.0301	1	1	28	regulation of neurotransmitter	BP	1	WNT7A	up_AORT_VSMC
GO:0042474	0.0301	1	1	11	middle ear morphogenesis	BP	1	EYA1	up_VFEM_VSMC
GO:0010894	0.0301	1	1	9	negative regulation of steroid biosynthetic	BP	1	NR1H4	up_VPUL_EC
GO:0045939	0.0301	1	1	9	negative regulation of steroid metabolic process	BP	1	NR1H4	up_VPUL_EC
GO:0007254	0.0302	0.987	12	67	JNK cascade	BP	1	RASSF2,TLR3,SH3RF2,SASH1,F2RL1,SH3RF1,APP,DUSP22,EDN1,NOD2,SH3RF3,MAP3K7	up_ACOR_VSMC
GO:0051240	0.0302	0.994	4	489	positive regulation of multicellular organismal	BP	1	GATM,NETO1,HOXA11,SHOX2	up_VFEM_VSMC
GO:0050710	0.0302	0.995	4	13	negative regulation of cytokine secretion	BP	1	C5AR2,ANXA4,F2RL1,MAPKBP1	up_VSAP_EC
GO:0007167	0.0302	0.996	3	274	enzyme linked receptor protein signaling pathway	BP	1	FGF5,NKX2-5,BAMBI	up_VPOR_VSMC
GO:0051222	0.0302	1	1	103	positive regulation of protein transport	BP	1	JUP	down_VFEM_VSMC
GO:0032677	0.0302	1	1	22	regulation of interleukin-8 production	BP	1	TLR2	up_AFEM_VSMC
GO:0021879	0.0302	1	1	15	forebrain neuron differentiation	BP	1	SLIT2	up_VPUL_VSMC
GO:0055021	0.0302	1	1	15	regulation of cardiac muscle tissue growth	BP	1	TBX5	up_VPUL_VSMC
GO:0045907	0.0302	1	1	6	positive regulation of vasoconstriction	BP	1	CAV1	up_VSAP_VSMC
GO:0040012	0.030214797	0.98553	14	303	regulation of locomotion	BP	0.99913151	SOX9,AQP1,SEMA6D,ZMYND8,MMP28,TRIM8,PTGER4,ARHGDIB,FOXC2,LRRC15,TBX5,CHMP4B,RARRES2,NRP1	up_ACOR_EC
GO:0010927	0.0303	0.991	6	28	cellular component assembly involved in	BP	0.742	MEF2C,EDN1,TMOD1,NECTIN2,PROX1,TMOD3	up_VCAV_EC
GO:0001922	0.0303	0.999	2	3	B-1 B cell homeostasis	BP	0.742	TNFAIP3,HIF1A	up_VCAV_EC
GO:0035773	0.0303	1	1	15	insulin secretion involved in cellular response to glucose stimulus	BP	1	RAB11B	down_VPOR_EC
GO:0060996	0.0303	1	1	26	dendritic spine development	BP	1	WNT7A	up_AORT_VSMC
GO:0046621	0.0303	1	1	8	negative regulation of organ growth	BP	1	TBX5	up_APUL_EC
GO:0060079	0.0303	1	1	15	excitatory postsynaptic potential	BP	1	NETO1	up_VFEM_VSMC
GO:2000738	0.0303	1	1	9	positive regulation of stem cell differentiation	BP	1	TBX5	up_VPUL_EC
GO:0014909	0.0303	1	1	15	smooth muscle cell migration	BP	1	SLIT2	up_VPUL_VSMC
GO:0034134	0.0303	1	1	7	toll-like receptor 2 signaling pathway	BP	1	TLR2	up_VSAP_VSMC
GO:0048557	0.0303	1	1	7	embryonic digestive tract morphogenesis	BP	1	SHOX2	up_VSAP_VSMC
GO:0048534	0.0304	0.982	24	262	hematopoietic or lymphoid organ development	BP	0.813	VEGFA,MYH9,ACTN1,SOCS5,ETV6,CASP3,PTPN11,WDR1,HOXB7,HIF1A,PTPN2,EVI2B,CTNNB1,ANLN,TFRC,LYN,MYO1E,REST,PRMT1,SH2B3,GLO1,INHBA,BTK,SIX4	down_VSAP_EC
GO:0051493	0.0304	0.983	24	188	regulation of cytoskeleton organization	BP	0.742	PLK2,TEK,HCLS1,ARHGEF15,BAIAP2L1,MEF2C,EDN1,ARHGAP28,TMOD1,MTOR,NCKAP1,PROX1,IQGAP2,STMN1,SWAP70,NCK1,FES,SENP6,TAOK1,ARPC5L,ARHGDIB,TMOD3,ARHGEF2,FSCN1	up_VCAV_EC
GO:0001525	0.0304	0.984	17	173	angiogenesis	BP	0.813	VEGFA,SULF1,MYH9,SERPINE1,SPARC,JCAD,PGK1,PRCP,PIK3C2A,HIF1A,THBS2,MTDH,ESM1,CTNNB1,STARD13,KLF5,NRP1	down_VSAP_EC

GO:0050708	0.0304	0.985	16	101	regulation of protein secretion	BP	1	EXPH5,TLR1,LEP,HTR2B,MBP,FERMT1,TM7SF3,CD274,ANKRD1,F2RL1,PTPN22,PRKCE,CCL5,NOD2,SCAMP5,PFKL	up_ACOR_VSMC
GO:1904062	0.0304	0.987	11	62	regulation of cation transmembrane transport	BP	1	ACTN2,PLCG2,KCNE3,PTPN3,DAPK1,CACNB3,AKAP6,PRKCE,CHD7,FXYD1,PARK7	up_ACOR_VSMC
GO:0032436	0.0304	0.991	6	40	positive regulation of proteasomal ubiquitin-dependent protein	BP	0.813	PRICKLE1,PLK3,SOCS5,TRIB3,GSK3B,RAD23A	down_VSAP_EC
GO:0010812	0.0304	0.993	5	19	negative regulation of cell-substrate adhesion	BP	1	HOXA7,FAM107A,PIK3R1,FBLN1,ACVRL1	up_VSAP_EC
GO:0030166	0.0304	0.994	4	19	proteoglycan biosynthetic process	BP	0.813	ENSCAFG00000032470,CHSY1,CTNNB1,EXT1	down_VSAP_EC
GO:0050801	0.0304	0.996	3	200	ion homeostasis	BP	1	HOXA3,NR1H4,NEO1	up_VPUL_EC
GO:0055083	0.0304	1	1	6	monovalent inorganic anion homeostasis	BP	1	HOXA3	down_ACOR_EC
GO:1902204	0.0304	1	1	1	positive regulation of hepatocyte growth factor receptor signaling	BP	0.813	ESM1	down_VSAP_EC
GO:0021544	0.0304	1	1	8	subpallium development	BP	1	SLITRK5	up_APUL_EC
GO:0001710	0.0304	1	1	7	mesodermal cell fate commitment	BP	1	EYA1	up_VFEM_EC
GO:0021955	0.0304	1	1	15	central nervous system neuron axonogenesis	BP	1	SLIT2	up_VPUL_VSMC
GO:0046622	0.0304	1	1	15	positive regulation of organ growth	BP	1	TBX5	up_VPUL_VSMC
GO:0002374	0.0304	1	1	7	cytokine secretion involved in immune	BP	1	TLR2	up_VSAP_VSMC
GO:1903779	0.0304	1	1	6	regulation of cardiac conduction	BP	1	CAV1	up_VSAP_VSMC
GO:0007212	0.030423479	0.9977	2	11	dopamine receptor signaling pathway	BP	1	CALY,GNAO1	up_ACOR_EC
GO:0060538	0.0305	0.99	7	51	skeletal muscle organ development	BP	0.814	ACTN1,FLNB,CTNNB1,KLF5,ATF3,SIX4,HOMER1	down_VSAP_EC
GO:0042221	0.0305	0.992	6	1012	response to chemical	BP	1	CHL1,FGF5,NKX2-5,BAMBI,CYP26B1,C3AR1	up_VPOR_VSMC
GO:0038180	0.0305	0.999	2	3	nerve growth factor signaling pathway	BP	0.743	RAPGEF1,RAPGEF2	up_VCAV_EC
GO:0014816	0.0305	0.999	2	3	skeletal muscle satellite cell differentiation	BP	0.743	EPHB1,CDON	up_VCAV_EC
GO:0090025	0.0305	1	1	7	regulation of monocyte chemotaxis	BP	1	LYN	down_ACOR_EC
GO:0048729	0.0305	1	1	209	tissue morphogenesis	BP	1	GATA5	down_AFEM_VSMC
GO:0038034	0.0305	1	1	21	signal transduction in absence of ligand	BP	1	EYA1	up_AFEM_VSMC
GO:0097192	0.0305	1	1	21	extrinsic apoptotic signaling pathway in	BP	1	EYA1	up_AFEM_VSMC
GO:0032228	0.0305	1	1	8	regulation of synaptic transmission, GABAergic	BP	1	PLCL1	up_APUL_EC
GO:1904467	0.0305	1	1	8	regulation of tumor necrosis factor secretion	BP	1	NR1H4	up_APUL_EC
GO:0071675	0.0305	1	1	13	regulation of mononuclear cell	BP	1	C3AR1	up_VPOR_VSMC
GO:0009058	0.0306	0.994	6	1910	biosynthetic process	BP	1	TLR2,TBX15,EYA1,HOXA9,HOXA10,IRX5	up_AFEM_VSMC
GO:0051055	0.0306	0.994	4	21	negative regulation of lipid biosynthetic process	BP	0.814	NR1H4,TRIB3,APOE,REST	down_VSAP_EC
GO:0048646	0.0306	0.994	4	349	anatomical structure formation involved in	BP	1	TBX5,HOXA3,MYBPC2,CCL24	up_VPUL_EC
GO:0072337	0.0306	1	1	7	modified amino acid transport	BP	1	PDPN	down_ACOR_EC
GO:1902285	0.0306	1	1	4	semaphorin-plexin signaling pathway involved in neuron	BP	1	NRP2	down_ACOR_EC
GO:0060359	0.0306	1	1	14	response to ammonium ion	BP	1	GABPA	down_AFEM_EC
GO:0006721	0.0306	1	1	14	terpenoid metabolic process	BP	1	CYP26B1	up_VPOR_VSMC
GO:0040012	0.0307	0.98	39	303	regulation of locomotion	BP	1	HOXA7,TWIST2,WNT11,FAM107A,C5AR2,ENPP2,SLC8B1,SLIT2,CDH1,FGF7,ABL1,HDAC7,EMP2,CHRM1,FUZ,PDGFD,PLXNA4,CCBE1,STAT5A,RECK,F2RL1,DDR2,PIK3R1,TIAM1,TBXA2R,CFAP69,SASH1,MYD88,FBXO7,PC,FBLN1,ZNF580,STX4,ACVRL1,NCK1,HMOX1,MAP2K2,TGFBR2,BAG4	up_VSAP_EC
GO:0002376	0.0307	0.994	4	622	immune system process	BP	1	HOXA3,TMEM176A,TMEM176B,SLIT2	up_VPUL_VSMC

GO:0032733	0.0307	0.996	3	9	positive regulation of interleukin-10 production	BP	0.744	CD34,TLR4,HMGB1	up_VCAV_EC
GO:0071478	0.0307	0.997	2	58	cellular response to radiation	BP	1	USP28,NEDD4	down_ACOR_EC
GO:0046903	0.0307	0.998	2	255	secretion	BP	1	WNT7A,ARG2	up_AORT_VSMC
GO:0006826	0.0307	1	1	15	iron ion transport	BP	1	RAB11B	down_VPOR_EC
GO:0030730	0.0307	1	1	4	sequestering of triglyceride	BP	1	ABHD5	up_VPOR_EC
GO:0007215	0.0307	1	1	13	glutamate receptor signaling pathway	BP	1	NETO1	up_VFEM_VSMC
GO:0010002	0.0307	1	1	9	cardioblast differentiation	BP	1	TBX5	up_VPUL_EC
GO:0010939	0.0307	1	1	9	regulation of necrotic cell death	BP	1	ARHGEF2	up_VPUL_EC
GO:0043648	0.030707469	0.99542	3	26	dicarboxylic acid metabolic process	BP	1	NR1H4,NMNAT2,NIT2	up_ACOR_EC
GO:0043122	0.0308	0.988	10	62	regulation of I-kappaB kinase/NF-kappaB	BP	0.744	TNFAIP3,TNIP1,TLR4,PELI1,CASP8,MAP3K3,RIOK3,TLR3,BCL10,RIPK1	up_VCAV_EC
GO:0090659	0.0308	0.995	4	12	walking behavior	BP	1	GLRB,CHD7,CLN8,OXR1	up_ACOR_VSMC
GO:0014829	0.0308	0.996	3	8	vascular smooth muscle contraction	BP	0.744	EDNRB,DOCK4,EDN1	up_VCAV_EC
GO:0090174	0.0308	0.997	2	56	organelle membrane fusion	BP	1	SPG11,STX4	down_ACOR_EC
GO:1900221	0.0308	0.998	2	6	regulation of amyloid-beta clearance	BP	1	LRP1,LRPAP1	down_VCAV_EC
GO:0034616	0.0308	0.999	2	3	response to laminar fluid shear stress	BP	1	SMAD6,ABCA1	up_ACOR_VSMC
GO:0060412	0.0308	1	1	15	ventricular septum morphogenesis	BP	1	CITED2	down_VPOR_EC
GO:0071071	0.0308	1	1	8	regulation of phospholipid biosynthetic process	BP	1	NR1H4	up_APUL_EC
GO:0006541	0.0308	1	1	9	glutamine metabolic process	BP	1	NR1H4	up_VPUL_EC
GO:0001710	0.0308	1	1	7	mesodermal cell fate commitment	BP	1	HOXA11	up_VSAP_VSMC
GO:0001823	0.030880334	0.99351	4	45	mesonephros development	BP	1	SOX9,SMAD6,FOXC2,CLSTN1	up_ACOR_EC
GO:0048514	0.0309	0.987	10	214	blood vessel morphogenesis	BP	1	LEP,ANGPT4,HOXA7,DLL1,F3,TMEM100,GREM1,TBXA2R,PTPRB,CLIC4	up_AFEM_EC
GO:0072175	0.0309	0.989	8	60	epithelial tube formation	BP	0.818	PRICKLE1,PRKACB,ENSCAFG00000032470,TMED2,HIF1A,CTNNB1,SDC4,SIX4	down_VSAP_EC
GO:0001843	0.0309	0.989	8	38	neural tube closure	BP	1	SHROOM3,BMP4,SEC24B,CITED2,SPINT1,LMO4,SPINT2,FZD3	up_ACOR_VSMC
GO:0043242	0.0309	0.993	5	21	negative regulation of protein complex	BP	0.744	TMOD1,SWAP70,TAOK1,TMOD3,ARHGEF2	up_VCAV_EC
GO:0009314	0.0309	0.995	3	143	response to radiation	BP	1	USP28,HOXA1,NEDD4	down_ACOR_EC
GO:1904646	0.0309	0.998	2	6	cellular response to amyloid-beta	BP	1	LRP1,PRNP	down_VCAV_EC
GO:0045723	0.0309	0.998	2	5	positive regulation of fatty acid biosynthetic process	BP	0.818	PTGS2,SLC45A3	down_VSAP_EC
GO:1905906	0.0309	0.998	2	5	regulation of amyloid fibril formation	BP	0.818	CRYAB,APOE	down_VSAP_EC
GO:0048511	0.0309	0.997	2	72	rhythmic process	BP	1	HTR7,NTRK2	up_APUL_EC
GO:0014706	0.0309	0.998	2	112	striated muscle tissue development	BP	1	NKX2-5,CYP26B1	up_VPOR_VSMC
GO:0045785	0.0309	1	1	107	positive regulation of cell adhesion	BP	1	JUP	down_VFEM_VSMC
GO:0021855	0.0309	1	1	4	hypothalamus cell migration	BP	1	SEMA3A	up_VPOR_EC
GO:0071300	0.0309	1	1	12	cellular response to retinoic acid	BP	1	CYP26B1	up_VPOR_VSMC
GO:0043113	0.0309	1	1	12	receptor clustering	BP	1	LHFPL4	up_VPOR_VSMC
GO:1990774	0.0309	1	1	9	tumor necrosis factor secretion	BP	1	NR1H4	up_VPUL_EC
GO:0120035	0.031	0.984	16	160	regulation of plasma membrane bounded cell projection organization	BP	0.819	SIPA1L1,FSCN1,DPYSL3,IFRD1,DYNLL1,TNIK,UST,ANLN,APOE,KLF5,LYN,VLDLR,HTT,DISC1,NRP1,YWHAH	down_VSAP_EC
GO:0008361	0.031	0.988	9	50	regulation of cell size	BP	0.744	IL7R,IFRD1,LRRC8D,EDN1,RAB11A,MTOR,LRRC8C,SLC12A7,CREB1	up_VCAV_EC
GO:0006986	0.031	0.991	6	42	response to unfolded protein	BP	0.819	HSPA5,ERO1A,PTPN2,DDIT3,ATF3,CREB3L2	down_VSAP_EC
GO:1903055	0.031	1	1	6	positive regulation of extracellular matrix	BP	1	PDPN	down_ACOR_EC

GO:0034121	0.031	1	1	22	regulation of toll-like receptor signaling	BP	1	TLR2	up_AFEM_VSMC
GO:0032720	0.031	1	1	13	negative regulation of tumor necrosis factor	BP	1	BPI	up_AORT_EC
GO:0050777	0.031	1	1	28	negative regulation of immune response	BP	1	ARG2	up_AORT_VSMC
GO:0045933	0.031	1	1	8	positive regulation of muscle contraction	BP	1	NPNT	up_APUL_EC
GO:0090103	0.031	1	1	8	cochlea morphogenesis	BP	1	HOXA1	up_APUL_EC
GO:0051926	0.031	1	1	14	negative regulation of calcium ion transport	BP	1	HOXA3	up_VPOR_VSMC
GO:0060065	0.031	1	1	7	uterus development	BP	1	HOXA9	up_VFEM_EC
GO:1901379	0.031	1	1	12	regulation of potassium ion transmembrane	BP	1	NETO1	up_VFEM_VSMC
GO:0019222	0.0311	0.994	6	1934	regulation of metabolic process	BP	1	HOXC10,TLR2,EYA1,HOXA9,HOXA10,IRX5	up_AFEM_VSMC
GO:0051084	0.0311	0.998	2	5	'de novo' posttranslational protein	BP	0.819	ERO1A,SH3GLB1	down_VSAP_EC
GO:0032656	0.0311	0.998	2	4	regulation of interleukin-13 production	BP	0.744	GATA3,ARG2	up_VCAV_EC
GO:0032616	0.0311	0.998	2	4	interleukin-13 production	BP	0.744	GATA3,ARG2	up_VCAV_EC
GO:0042733	0.0311	1	1	27	embryonic digit morphogenesis	BP	1	WNT7A	up_AORT_VSMC
GO:0031114	0.0311	1	1	9	regulation of microtubule depolymerization	BP	1	ARHGEF2	up_VPUL_EC
GO:0042981	0.0312	0.98	38	471	regulation of apoptotic process	BP	0.819	ARHGEF2,VEGFA,P4HB,ASNS,NR1H4,PLK3,HSPA5,SERPINE1,CASP3,HYOU1,AARS,FHL2,PTGS2,HIF1A,TNIK,ZPR1,PTPN2,MTDH,MAGED1,CTNNB1,CRYAB,LYN,BNIP3,DDIT3,FAM162A,ATF3,GSK3B,SH3GLB1,HTT,REST,GLO1,PAK2,INHBA,SIX4,NRP1,SPINK2,ARF4,MADD	down_VSAP_EC
GO:0002573	0.0312	0.987	11	71	myeloid leukocyte differentiation	BP	0.744	HCLS1,KIT,MEF2C,GATA3,CASP8,MTOR,HHEX,CREB1,RIPK1,CUL4A,TJP2	up_VCAV_EC
GO:0032602	0.0312	0.992	5	23	chemokine production	BP	0.744	ACKR1,TLR4,ARG2,TLR3,APOD	up_VCAV_EC
GO:0019220	0.0312	0.992	5	525	regulation of phosphate metabolic process	BP	1	NR1H4,CCL24,PTGER2,FLRT3,ARHGEF2	up_VPUL_EC
GO:0051174	0.0312	0.992	5	525	regulation of phosphorus metabolic process	BP	1	NR1H4,CCL24,PTGER2,FLRT3,ARHGEF2	up_VPUL_EC
GO:0051966	0.0312	0.996	3	8	regulation of synaptic transmission,	BP	0.744	RELN,MEF2C,GRM8	up_VCAV_EC
GO:0050877	0.0312	0.995	3	164	nervous system process	BP	1	HOXC10,MME,SIX1	up_VFEM_EC
GO:0032703	0.0312	1	1	8	negative regulation of interleukin-2 production	BP	1	NR1H4	up_APUL_EC
GO:0044703	0.0313	0.982	22	172	multi-organism reproductive process	BP	1	ACSL4,NDC1,TRIP13,CTDNEP1,FBXO5,AAAS,NCAPH,POC1A,B4GALT1,PPARD,HIST1H1A,EMP2,FOSL1,HMGB2,SGPL1,TTK,ASPM,HOXA9,GLI1,H2AFX,CCT4,TTLL1	down_ACOR_VSMC
GO:0001649	0.0313	0.988	10	54	osteoblast differentiation	BP	1	FZD1,TWIST2,WNT11,AXIN2,ZHX3,HDAC7,DDR2,SHOX2,LIMD1,BGLAP	up_VSAP_EC
GO:0006139	0.0313	0.991	8	1909	nucleobase-containing compound metabolic	BP	1	IVNS1ABP,EXTL2,DARS,SUCLG2,SRSF6,GABPA,NDUFA10,TSEN15	down_AFEM_EC
GO:0034440	0.0313	0.992	5	35	lipid oxidation	BP	1	AKT2,ACAA1,PEX5,LONP2,ACAT1	down_VCAV_EC
GO:1902931	0.0313	0.998	2	5	negative regulation of alcohol biosynthetic	BP	0.819	APOE,REST	down_VSAP_EC
GO:1903961	0.0313	0.999	2	3	positive regulation of anion transmembrane	BP	1	TCAF1,PSEN1	up_ACOR_VSMC
GO:0048871	0.0313	0.997	2	120	multicellular organismal homeostasis	BP	1	HOXC10,LNPEP	up_AORT_EC
GO:0050709	0.0313	1	1	28	negative regulation of protein secretion	BP	1	ARG2	up_AORT_VSMC
GO:0032620	0.0313	1	1	8	interleukin-17 production	BP	1	NR1H4	up_APUL_EC
GO:0044070	0.0313	1	1	22	regulation of anion transport	BP	1	CA2	up_VCAV_VSMC
GO:0072172	0.0313	1	1	7	mesonephric tubule formation	BP	1	SIX1	up_VFEM_EC
GO:1990868	0.0313	1	1	16	response to chemokine	BP	1	SLIT2	up_VPUL_VSMC
GO:1990869	0.0313	1	1	16	cellular response to chemokine	BP	1	SLIT2	up_VPUL_VSMC
GO:0046323	0.031320192	0.9953	3	26	glucose import	BP	1	NR1H4,ENSCAFG00000004331,RARRES2	up_ACOR_EC

GO:0051348	0.031329055	0.99054	6	90	negative regulation of transferase activity	BP	1	PKIB,DUSP26,LRRC15,LRRC4,PRKCD,DBNDD2	up_ACOR_EC
GO:0050768	0.031376335	0.99181	5	66	negative regulation of neurogenesis	BP	1	SOX9,DIXDC1,RGMA,LRP4,NRP1	up_ACOR_EC
GO:0016477	0.0314	0.981	30	426	cell migration	BP	1	PDGFRB,DCN,NDN,SDC4,GLIPR2,KANK2,BMPR1A,LMO4,RECK,RHOD,AKT2,DOCK10,SYDE1,HSD3B7,LAMA3,CSPG4,SEMA3C,THY1,PEX5,B4GALT1,GLI3,SASH1,CDH2,FUT8,SPRED1,TWIST2,DDR2,GREM1,RAC2,FOXO3	down_VCAV_EC
GO:0002449	0.0314	0.988	9	53	lymphocyte mediated immunity	BP	0.746	IL7R,GATA3,RAB27A,NECTIN2,THOC1,SWAP70,JAG1,BCL10,HMGB1	up_VCAV_EC
GO:0001510	0.0314	0.992	5	22	RNA methylation	BP	1	FBL,THUMPD2,METTL1,WTAP,EMG1	down_ACOR_VSMC
GO:1901570	0.0314	0.994	4	16	fatty acid derivative biosynthetic process	BP	0.746	EDN1,PTGS1,ELOVL7,PTGS2	up_VCAV_EC
GO:0045047	0.0314	0.996	3	14	protein targeting to ER	BP	1	SSR3,SEC61B,SEC61A1	down_VCAV_EC
GO:0072599	0.0314	0.996	3	14	establishment of protein localization to endoplasmic reticulum	BP	1	SSR3,SEC61B,SEC61A1	down_VCAV_EC
GO:0032689	0.0314	1	1	8	negative regulation of interferon-gamma	BP	1	NR1H4	up_APUL_EC
GO:0050829	0.0314	1	1	8	defense response to Gram-negative bacterium	BP	1	BPI	up_APUL_EC
GO:0021561	0.0314	1	1	8	facial nerve development	BP	1	SIX1	up_VFEM_EC
GO:0021610	0.0314	1	1	8	facial nerve morphogenesis	BP	1	SIX1	up_VFEM_EC
GO:0035909	0.0314	1	1	14	aorta morphogenesis	BP	1	EYA1	up_VFEM_VSMC
GO:0001934	0.0315	0.98	32	274	positive regulation of protein phosphorylation	BP	0.746	TEK,HCLS1,KIT,MAP3K11,PDGFB,STK10,RELN,TLR4,EDN1,RALB,APLNR,NEDD9,YES1,MTOR,MAP3K3,BMP2,SLK,PROX1,MADD,PAK2,PRKD2,TLR3,PTGS2,MMD,RAPGEF2,TAOK1,HMGB1,RIPK1,PDGFD,JCAD,ARHGEF2,CDON	up_VCAV_EC
GO:0035690	0.0315	0.985	15	91	cellular response to drug	BP	1	PRKAA2,ATRX,CASTOR1,TP53INP1,ADCY6,ANKRD1,PTPN22,PRKCE,AGRN,JUP,NR3C1,NOD2,DDIT4,GNAQ,PARK7	up_ACOR_VSMC
GO:0002761	0.0315	0.992	5	36	regulation of myeloid leukocyte differentiation	BP	1	NOTCH2,FBN1,TFE3,CLSTN1,ZFP36L1	down_VCAV_EC
GO:1905332	0.0315	0.994	4	20	positive regulation of morphogenesis of an	BP	0.819	HOXB7,MAGED1,CTNNB1,SIX4	down_VSAP_EC
GO:0080111	0.0315	0.996	3	9	DNA demethylation	BP	0.747	GATA3,OTUD4,TET2	up_VCAV_EC
GO:0007420	0.0315	0.995	3	169	brain development	BP	1	PITX1,EMX2,SALL1	up_VSAP_VSMC
GO:0034103	0.0315	0.998	2	12	regulation of tissue remodeling	BP	1	LEP,GREM1	up_AFEM_EC
GO:0032642	0.0315	1	1	22	regulation of chemokine production	BP	1	TLR2	up_AFEM_VSMC
GO:0060972	0.0315	1	1	13	left/right pattern formation	BP	1	NKX2-5	up_VPOR_VSMC
GO:0016579	0.0316	0.988	9	49	protein deubiquitination	BP	0.747	TNFAIP3,TNIP1,USP33,ENSCAFG00000010893,OTUD4,USP28,USP13,USP38,USP37	up_VCAV_EC
GO:0046483	0.0316	0.994	6	1948	heterocycle metabolic process	BP	1	TLR2,TBX15,EYA1,HOXA9,HOXA10,IRX5	up_AFEM_VSMC
GO:1901976	0.0316	0.992	5	21	regulation of cell cycle checkpoint	BP	1	WDR76,RNASEH2B,NDC80,DDX39B,GEN1	down_ACOR_VSMC
GO:0033692	0.0316	0.994	4	20	cellular polysaccharide biosynthetic process	BP	0.819	ENPP1,ENSCAFG00000032470,PPP1R3C,EXT1	down_VSAP_EC
GO:0009100	0.0316	0.997	2	139	glycoprotein metabolic process	BP	1	MGAT4B,GBGT1	down_VPOR_EC
GO:1902373	0.0316	0.998	2	12	negative regulation of mRNA catabolic process	BP	1	TENT5C,AXIN2	up_AFEM_EC
GO:0034134	0.0316	1	1	7	toll-like receptor 2 signaling pathway	BP	1	LYN	down_ACOR_EC
GO:1904951	0.0316	1	1	108	positive regulation of establishment of protein localization	BP	1	JUP	down_VFEM_VSMC
GO:0021549	0.0316	1	1	26	cerebellum development	BP	1	WNT7A	up_AORT_VSMC
GO:0048566	0.0316	1	1	13	embryonic digestive tract development	BP	1	SHOX2	up_VFEM_VSMC
GO:0023061	0.0317	0.985	15	95	signal release	BP	1	LEP,FBXL20,TM7SF3,STX11,PRKCE,SCG5,PSEN1,CHD7,CCL5,RAB11B,EDN1,KCNC4,PREPL,PFKL,PARK7	up_ACOR_VSMC
GO:0002696	0.0317	0.986	12	80	positive regulation of leukocyte activation	BP	0.749	IL7R,MEF2C,TLR4,GATA3,PELI1,NECTIN2,ITPKB,NCK1,CD200,BCL10,HMGB1,ATP11C	up_VCAV_EC

GO:0050727	0.0317	0.988	9	74	regulation of inflammatory response	BP	0.819	NR1H4,SERPINE1,SOCS5,PTGS2,PTPN2,APOE,LYN,GBP5,A2M	down_VSAP_EC
GO:0051310	0.0317	0.992	5	22	metaphase plate congression	BP	1	ZW10,KIF22,CDCA8,NDC80,NUP62	down_ACOR_VSMC
GO:0007043	0.0317	0.992	5	28	cell-cell junction assembly	BP	0.819	NR1H4,FSCN1,WDR1,MYO1C,CTNNB1	down_VSAP_EC
GO:0071709	0.0317	0.996	3	8	membrane assembly	BP	1	EMP2,LRP4,FLOT1	up_VSAP_EC
GO:0019229	0.0317	0.998	2	12	regulation of vasoconstriction	BP	1	AVPR1A,TBXA2R	up_AFEM_EC
GO:0030514	0.0317	0.998	2	12	negative regulation of BMP signaling pathway	BP	1	GREM1,GREM2	up_AFEM_EC
GO:0043066	0.0317	0.998	2	253	negative regulation of apoptotic process	BP	1	WNT7A,ARG2	up_AORT_VSMC
GO:0097530	0.0317	0.997	2	33	granulocyte migration	BP	1	C3AR1,ENSCAFG00000032731	up_VPOR_EC
GO:0002065	0.0317	1	1	37	columnar/cuboidal epithelial cell	BP	1	GATA5	down_VSAP_VSMC
GO:0060688	0.0317	1	1	29	regulation of morphogenesis of a	BP	1	ESR1	up_AORT_VSMC
GO:0030104	0.0317	1	1	13	water homeostasis	BP	1	CYP26B1	up_VPOR_VSMC
GO:0061384	0.0317	1	1	12	heart trabecula morphogenesis	BP	1	NKX2-5	up_VPOR_VSMC
GO:0034694	0.0317	1	1	9	response to prostaglandin	BP	1	PTGER2	up_VPUL_EC
GO:0035136	0.0317	1	1	16	forelimb morphogenesis	BP	1	TBX5	up_VPUL_VSMC
GO:0032868	0.031742494	0.99169	5	66	response to insulin	BP	1	NR1H4,ENSCAFG00000004331,FOXC2,RARRES2,PRKCD	up_ACOR_EC
GO:0045596	0.0318	0.983	18	186	negative regulation of cell differentiation	BP	0.819	PRICKLE1,SOCS5,ENPP1,DPYSL3,HOOK3,TRIB3,IFRD1,PTPN2,CTNNB1,APOE,LYN,STRAP,DDIT3,REST,PRMT1,SIX4,NRP1,YWHAH	down_VSAP_EC
GO:0062012	0.0318	0.986	11	100	regulation of small molecule metabolic	BP	0.819	NR1H4,ENPP1,TRIB3,PGAM1,SESN2,PTGS2,HIF1A,SLC45A3,PTPN2,APOE,REST	down_VSAP_EC
GO:0060576	0.0318	0.998	2	5	intestinal epithelial cell development	BP	0.819	HIF1A,KLF5	down_VSAP_EC
GO:0014706	0.0318	0.997	2	112	striated muscle tissue development	BP	1	EYA1,SHOX2	up_VFEM_VSMC
GO:0030857	0.0318	1	1	15	negative regulation of epithelial cell	BP	1	SRSF6	down_AFEM_EC
GO:0060236	0.0318	1	1	15	regulation of mitotic spindle organization	BP	1	GPSM2	down_AFEM_EC
GO:0031214	0.0318	1	1	45	biomineral tissue development	BP	1	HOXA3	up_APUL_VSMC
GO:0140058	0.0318	1	1	4	neuron projection arborization	BP	1	SEMA3A	up_VPOR_EC
GO:0061548	0.0318	1	1	8	ganglion development	BP	1	SIX1	up_VFEM_EC
GO:1903427	0.0318	1	1	7	negative regulation of reactive oxygen species biosynthetic process	BP	1	CAV1	up_VSAP_VSMC
GO:1905037	0.031807644	0.99323	4	47	autophagosome organization	BP	1	ATG16L2,CHMP4B,TP53INP2,ENSCAFG00000010493	up_ACOR_EC
GO:1903670	0.0319	0.992	5	30	regulation of sprouting angiogenesis	BP	0.819	VEGFA,JCAD,PIK3C2A,STARD13,NRP1	down_VSAP_EC
GO:0010092	0.0319	0.992	5	19	specification of animal organ identity	BP	1	GATA5,BMP4,FGFR2,SMARCD3,FRS2	up_ACOR_VSMC
GO:1905475	0.0319	0.997	2	52	regulation of protein localization to membrane	BP	1	ACSL3,STX4	down_ACOR_EC
GO:0010559	0.0319	0.998	2	6	regulation of glycoprotein biosynthetic process	BP	1	NECAB3,SOAT1	down_VCAV_EC
GO:1903018	0.0319	0.998	2	6	regulation of glycoprotein metabolic process	BP	1	NECAB3,SOAT1	down_VCAV_EC
GO:0033006	0.0319	1	1	7	regulation of mast cell activation involved in	BP	1	LYN	down_ACOR_EC
GO:0043304	0.0319	1	1	7	regulation of mast cell degranulation	BP	1	LYN	down_ACOR_EC
GO:0048663	0.0319	1	1	13	neuron fate commitment	BP	1	HOXC10	up_AORT_EC
GO:0001738	0.0319	1	1	27	morphogenesis of a polarized epithelium	BP	1	WNT7A	up_AORT_VSMC
GO:0050868	0.0319	1	1	28	negative regulation of T cell activation	BP	1	ARG2	up_AORT_VSMC
GO:0042398	0.0319	1	1	12	cellular modified amino acid biosynthetic process	BP	1	GATM	up_VFEM_VSMC
GO:0031579	0.0319	1	1	6	membrane raft organization	BP	1	CAV1	up_VSAP_VSMC
GO:0002026	0.0319	1	1	8	regulation of the force of heart contraction	BP	1	CAV1	up_VSAP_VSMC

GO:0007249	0.032	0.986	11	71	I-kappaB kinase/NF-kappaB signaling	BP	0.751	TNFAIP3,TNIP1,TLR4,PELI1,CASP8,MAP3K3,ERC1,RIOK3,TLR3,BCL10,RIPK1	up_VCAV_EC
GO:0060236	0.032	0.994	4	15	regulation of mitotic spindle organization	BP	1	SMC1A,NUP62,SMC3,STAG1	down_ACOR_VSMC
GO:0045185	0.032	0.993	4	25	maintenance of protein location	BP	1	FBN1,TLN2,PARK7,ARL2BP	down_VCAV_EC
GO:0035924	0.032	0.994	4	21	cellular response to vascular endothelial growth factor stimulus	BP	0.819	VEGFA,JCAD,MYO1C,NRP1	down_VSAP_EC
GO:1990845	0.032	0.993	4	51	adaptive thermogenesis	BP	1	CMKLR1,HOXC10,LEP,PPARGC1A	up_AFEM_EC
GO:1902916	0.032	0.996	3	8	positive regulation of protein polyubiquitination	BP	1	SKP2,PTPN22,NOD2	up_ACOR_VSMC
GO:0048070	0.032	0.998	2	4	regulation of developmental	BP	0.751	KIT,ADAMTS9	up_VCAV_EC
GO:0014904	0.032	1	1	12	myotube cell development	BP	1	SHOX2	up_VFEM_VSMC
GO:0048741	0.032	1	1	12	skeletal muscle fiber development	BP	1	SHOX2	up_VFEM_VSMC
GO:0043502	0.032	1	1	16	regulation of muscle adaptation	BP	1	GATA5	up_VPUL_VSMC
GO:1902743	0.032	1	1	16	regulation of lamellipodium	BP	1	SLIT2	up_VPUL_VSMC
GO:0072006	0.0321	0.987	10	59	nephron development	BP	0.751	HOXB7,PDGFB,PODXL,MEF2C,GPR4,GATA3,MTSS1,BMP2,JAG1,BASP1	up_VCAV_EC
GO:0035710	0.0321	0.992	5	22	CD4-positive, alpha-beta T cell activation	BP	0.751	IL6,GATA3,ARG2,MTOR,HMGB1	up_VCAV_EC
GO:0090183	0.0321	0.994	4	21	regulation of kidney development	BP	0.819	HOXB7,MAGED1,CTNNB1,SIX4	down_VSAP_EC
GO:0042592	0.0321	0.994	4	522	homeostatic process	BP	1	HOXC10,ENSCAFG00000005494,LNPEP,TAOK1	up_AORT_EC
GO:0010628	0.0321	0.996	3	527	positive regulation of gene expression	BP	1	TLR2,EYA1,HOXA9	up_AFEM_VSMC
GO:0002279	0.0321	0.996	3	10	mast cell activation involved in immune	BP	0.751	KIT,SNAP23,FES	up_VCAV_EC
GO:0010939	0.0321	0.996	3	9	regulation of necrotic cell death	BP	0.751	PELI1,RIPK1,ARHGEF2	up_VCAV_EC
GO:0002448	0.0321	0.996	3	10	mast cell mediated immunity	BP	0.751	KIT,SNAP23,FES	up_VCAV_EC
GO:0043303	0.0321	0.996	3	10	mast cell degranulation	BP	0.751	KIT,SNAP23,FES	up_VCAV_EC
GO:0035115	0.0321	0.997	2	12	embryonic forelimb morphogenesis	BP	1	HOXA9,RECK	up_AFEM_EC
GO:0072079	0.0321	0.997	2	12	nephron tubule formation	BP	1	IRX3,GREM1	up_AFEM_EC
GO:0021510	0.0321	1	1	23	spinal cord development	BP	1	HOXC10	up_AFEM_VSMC
GO:1905941	0.0321	1	1	4	positive regulation of gonad development	BP	1	SEMA3A	up_VPOR_EC
GO:2000018	0.0321	1	1	4	regulation of male gonad development	BP	1	SEMA3A	up_VPOR_EC
GO:2000020	0.0321	1	1	4	positive regulation of male gonad development	BP	1	SEMA3A	up_VPOR_EC
GO:0045880	0.0321	1	1	14	positive regulation of smoothened signaling	BP	1	SHOX2	up_VFEM_VSMC
GO:0021602	0.0321	1	1	16	cranial nerve morphogenesis	BP	1	HOXA3	up_VPUL_VSMC
GO:0002040	0.032129687	0.99315	4	46	sprouting angiogenesis	BP	1	ENSCAFG00000004331,ESM1,FOXC2,NRP1	up_ACOR_EC
GO:0080090	0.0322	0.986	14	1734	regulation of primary metabolic process	BP	1	ACVRL1,YEATS2,LYN,IGF1R,YBX3,HIVEP2,HOXA7,PRKAR2A,SULF1,ELK3,HOXA1,ACSL3,ASPN,NEDD4	down_ACOR_EC
GO:0030326	0.0322	0.993	4	51	embryonic limb morphogenesis	BP	1	HOXA9,HOXC10,GREM1,RECK	up_AFEM_EC
GO:0035113	0.0322	0.993	4	51	embryonic appendage morphogenesis	BP	1	HOXA9,HOXC10,GREM1,RECK	up_AFEM_EC
GO:0048015	0.0322	0.997	2	48	phosphatidylinositol-mediated signaling	BP	1	IGF1R,NEDD4	down_ACOR_EC
GO:0048017	0.0322	0.997	2	48	inositol lipid-mediated signaling	BP	1	IGF1R,NEDD4	down_ACOR_EC
GO:0032488	0.0322	0.998	2	5	Cdc42 protein signal transduction	BP	0.819	APOE,NRP1	down_VSAP_EC
GO:0032489	0.0322	0.998	2	5	regulation of Cdc42 protein signal	BP	0.819	APOE,NRP1	down_VSAP_EC
GO:0051036	0.0322	0.998	2	4	regulation of endosome size	BP	0.751	RAB11A,ALS2	up_VCAV_EC
GO:0006725	0.0323	0.994	6	1956	cellular aromatic compound metabolic	BP	1	TLR2,TBX15,EYA1,HOXA9,HOXA10,IRX5	up_AFEM_VSMC
GO:0061647	0.0323	0.994	4	13	histone H3-K9 modification	BP	0.751	GATA3,ARID4B,KDM3A,ATRX	up_VCAV_EC

GO:0007179	0.0323	0.997	2	57	transforming growth factor beta receptor	BP	1	ACVRL1,ASPN	down_ACOR_EC
GO:0034654	0.0323	1	2	1236	nucleobase-containing compound biosynthetic	BP	1	JUP,GATA5	down_VFEM_VSMC
GO:1903307	0.0323	1	1	8	positive regulation of regulated secretory	BP	1	STX4	down_ACOR_EC
GO:0033194	0.0323	1	1	7	response to hydroperoxide	BP	1	STX4	down_ACOR_EC
GO:0021522	0.0323	1	1	8	spinal cord motor neuron differentiation	BP	1	HOXC10	up_VFEM_EC
GO:0010647	0.0324	0.977	56	460	positive regulation of cell communication	BP	1	ADRA1B,TLR1,LGR6,LEP,MID1,GATA5,HTR2B,RASSF2,ZRANB1,C28H10orf71,SKP2,NACC2,MLLT3,ITM2C,FERMT1,BMP4,TM7SF3,FGFR2,TLR3,SH3RF2,PDE5A,CITED2,TP53INP1,SASH1,TMEM108,FGF18,CDKN2B,LIMS2,ANKRD1,F2RL1,BNIP3,CRLF3,CREBRF,NRP1,PTPN22,NPNT,UNC5B,PRKCE,FRS2,PARP9,APP,PSEN1,CCL5,DUSP22,SIAH1,EDN1,NOD2,FAM107A,DISC1,SH3RF3,SERINC3,MAP3K7,ENSCAFG00000029553,PARK7,IL18R1,CRKL	up_ACOR_VSMC
GO:0022603	0.0324	0.99	6	301	regulation of anatomical structure morphogenesis	BP	1	C3AR1,ENSCAFG00000002015,SEMA3A,ASPN,MTDH,RNF6	up_VPOR_EC
GO:0032634	0.0324	0.998	2	4	interleukin-5 production	BP	0.751	PDE4D,GATA3	up_VCAV_EC
GO:0032674	0.0324	0.998	2	4	regulation of interleukin-5 production	BP	0.751	PDE4D,GATA3	up_VCAV_EC
GO:0034504	0.0324	1	1	111	protein localization to nucleus	BP	1	JUP	down_VFEM_VSMC
GO:0034505	0.0324	1	1	4	tooth mineralization	BP	1	ASPN	up_VPOR_EC
GO:0071498	0.0324	1	1	8	cellular response to fluid shear stress	BP	1	MTSS1	up_VFEM_EC
GO:0006575	0.0325	0.989	7	53	cellular modified amino acid metabolic process	BP	0.825	P4HB,PLOD2,ERO1A,P4HA1,ENSCAFG00000028814,ALDH1L2,PLOD3	down_VSAP_EC
GO:0010960	0.0325	1	1	6	magnesium ion homeostasis	BP	1	HOXA3	down_ACOR_EC
GO:0061549	0.0325	1	1	5	sympathetic ganglion development	BP	1	NRP2	down_ACOR_EC
GO:0003197	0.0325	1	1	16	endocardial cushion development	BP	1	CITED2	down_VPOR_EC
GO:1901342	0.0325	1	1	114	regulation of vasculature development	BP	1	JUP	down_VFEM_VSMC
GO:0050772	0.0325	1	1	13	positive regulation of axonogenesis	BP	1	SHOX2	up_VFEM_VSMC
GO:1902305	0.0325	1	1	12	regulation of sodium ion transmembrane transport	BP	1	NETO1	up_VFEM_VSMC
GO:0008584	0.0326	0.989	8	39	male gonad development	BP	1	GATA4,PRDX4,ATRX,CITED2,SPINK2,ARID5B,LHX9,WDR48	up_ACOR_VSMC
GO:0046546	0.0326	0.989	8	39	development of primary male sexual	BP	1	GATA4,PRDX4,ATRX,CITED2,SPINK2,ARID5B,LHX9,WDR48	up_ACOR_VSMC
GO:0032465	0.0326	0.991	6	29	regulation of cytokinesis	BP	1	AURKB,PLK3,E2F8,CDC6,NUP62,PDXP	down_ACOR_VSMC
GO:0070266	0.0326	0.994	4	14	necroptotic process	BP	0.756	PELI1,TLR3,RIPK1,ARHGEF2	up_VCAV_EC
GO:0009056	0.0326	0.999	2	755	catabolic process	BP	1	PLCL1,USP12	down_VFEM_EC
GO:0086002	0.0326	1	1	7	cardiac muscle cell action potential involved in	BP	1	CAV1	up_VSAP_VSMC
GO:0072657	0.032613916	0.98797	8	144	protein localization to membrane	BP	1	KCNB1,AGRN,PLS1,ACTN2,LRRC15,LRP4,LRRC4,CHMP4B	up_ACOR_EC
GO:0032103	0.032677836	0.99137	5	69	positive regulation of response to external	BP	1	NOD2,IL33,PTGER4,RARRES2,NRP1	up_ACOR_EC
GO:0031344	0.0327	0.983	16	161	regulation of cell projection organization	BP	0.827	SIPA1L1,FSCN1,DPYSL3,IFRD1,DYNLL1,TNIK,UST,ANLN,APOE,KLF5,LYN,VLDLR,HTT,DISC1,NRP1,YWHAH	down_VSAP_EC
GO:0019933	0.0327	0.99	7	32	cAMP-mediated signaling	BP	1	ADRA1B,ADGRD1,ABCA1,PTGIR,ADCY6,APLP1,GNAQ	up_ACOR_VSMC
GO:0043524	0.0327	0.99	6	39	negative regulation of neuron apoptotic process	BP	0.827	HYOU1,AARS,HIF1A,ZPR1,SIX4,NRP1	down_VSAP_EC
GO:0032637	0.0327	0.991	6	26	interleukin-8 production	BP	1	C5AR2,ANXA4,F2RL1,MAPKBP1,ZNF580,MAVS	up_VSAP_EC
GO:0070168	0.0327	0.998	2	6	negative regulation of biomineral tissue	BP	1	ASPN,GREM1	down_VCAV_EC
GO:0006099	0.0327	1	1	15	tricarboxylic acid cycle	BP	1	SUCLG2	down_AFEM_EC
GO:0051653	0.0327	1	1	15	spindle localization	BP	1	GPSM2	down_AFEM_EC
GO:0043393	0.0327	1	1	77	regulation of protein binding	BP	1	PLCL1	down_VFEM_EC

GO:0048565	0.0327	1	1	38	digestive tract development	BP	1	GATA5	down_VSAP_VSMC
GO:0002792	0.0327	1	1	29	negative regulation of peptide secretion	BP	1	ARG2	up_AORT_VSMC
GO:0060544	0.0327	1	1	8	regulation of necroptotic process	BP	1	CAV1	up_VSAP_VSMC
GO:0060561	0.032780476	0.99742	2	11	apoptotic process involved in	BP	1	FZD5,FOXC2	up_ACOR_EC
GO:0048513	0.0328	0.975	104	906	animal organ development	BP	1	EXPH5,TNNT2,PDLIM3,FGF13,TMEM176B,TMEM176A,SMAD6,ACTN2,PLCG2,LEP,LBH,GATA5,GATA4,CD83,HTR2B,RASSF2,PRDX4,MLLT3,FERMT1,BMP4,LNPK,FGFR2,GJA1,ATRX,SEC24B,XIRP1,PPARGC1A,LATS2,CITED2,SPINT1,ISG15,ZNRF3,ZNF365,LMO4,TMEM108,FGF18,FAM210B,NDRG2,ATRN,P4HTM,NEBL,CDKN2B,LIMS2,ANKRD1,F2RL1,CTSB,EGLN1,ENSCAFG00000006238,CLSTN1,NRP1,PTPN22,GPM6A,CHD2,NPNT,SMARCD3,SPINT2,SLITRK5,TBX20,BBX,TBX5,FZD3,FRS2,CPAMD8,DAG1,POMK,APP,PSEN1,SEMA6D,TGFBR1,CHD7,JUP,SPINK2,DDR2,CLN8,FOSL2,PERP,PSKH1,RTKN2,EDN1,NID1,ARID5B,DMC1,SERPINB5,JAM3,B2M,DISC1,AP2B1,TNFRSF11B,TACC1,GAA,SUN1,CNOT4,LHX9,WFS1,WDR48,ID3,SLC8A1,TEAD1,ENSCAFG00000003530,IL18R1,BVES,RREB1,ZDHHC21,CRKL	up_ACOR_VSMC
GO:1900076	0.0328	0.992	5	29	regulation of cellular response to insulin	BP	0.827	NR1H4,ENPP1,PTPN11,MYO1C,PTPN2	down_VSAP_EC
GO:0061014	0.0328	0.996	3	8	positive regulation of mRNA catabolic process	BP	1	ZFP36L1,ZFP36L2,TNRC6C	up_VSAP_EC
GO:0062009	0.0328	0.996	3	8	secondary palate development	BP	1	FZD1,WNT11,TGFBR2	up_VSAP_EC
GO:0035929	0.0328	0.998	2	5	steroid hormone secretion	BP	0.827	PTPN11,INHBA	down_VSAP_EC
GO:0008015	0.0328	0.997	2	120	blood circulation	BP	1	LNPEP,SGCD	up_AORT_EC
GO:1902743	0.0328	1	1	16	regulation of lamellipodium	BP	1	FSCN1	down_AFEM_EC
GO:0031572	0.0328	1	1	15	G2 DNA damage checkpoint	BP	1	TAOK1	up_AORT_EC
GO:0042592	0.0329	0.976	63	522	homeostatic process	BP	1	RHOT1,ADRA1B,PLCG2,LEP,PLCL1,SLC26A7,HTR2B,RASSF2,PRDX4,PRKAA2,BMP4,LCAT,CNNM2,ABCA1,GJA1,ATRX,PPARGC1A,CITED2,ISG15,ZNF365,PTPN3,CLCN3,FAM210B,P4HTM,MICU2,TMX3,ITPR1,PLAC8B,F2RL1,STN1,ERO1B,EGLN1,SLC12A7,LRRC8C,DMPK,ATP7A,ORMDL3,SESN3,PRKCE,APP,PSEN1,CHD7,LAMP2,DMXL1,CLN8,RAB11B,EDN1,NOD2,JAM3,B2M,DISC1,HERPUD1,TXNDC5,GAA,INPP5K,WFS1,WDR48,ATP2C1,PARK7,SLC8A1,STC2,IL18R1,HMBOX1	up_ACOR_VSMC
GO:1904951	0.0329	0.983	17	108	positive regulation of establishment of protein localization	BP	1	EXPH5,TLR1,LEP,HTR2B,TCAF1,MBP,FERMT1,TM7SF3,CD274,ANKRD1,F2RL1,PTPN22,PRKCE,PSEN1,JUP,NOD2,SCAMP5	up_ACOR_VSMC
GO:0019222	0.0329	0.99	9	1934	regulation of metabolic process	BP	1	HOXC10,EYA1,GATM,HOXA10,HOXA9,FBP1,EMX2,HOXA11,SHOX2	up_VFEM_VSMC
GO:0034138	0.0329	0.996	3	9	toll-like receptor 3 signaling pathway	BP	0.762	TNFAIP3,PELI1,TLR3	up_VCAV_EC
GO:0006936	0.0329	0.997	2	74	muscle contraction	BP	1	HTR7,NPNT	up_APUL_EC
GO:0032602	0.0329	1	1	23	chemokine production	BP	1	TLR2	up_AFEM_VSMC
GO:0032755	0.0329	1	1	24	positive regulation of interleukin-6 production	BP	1	TLR2	up_AFEM_VSMC
GO:0048864	0.0329	1	1	29	stem cell development	BP	1	WNT7A	up_AORT_VSMC
GO:2000145	0.033	0.985	12	279	regulation of cell motility	BP	1	CMKLR1,ANGPT4,HOXA7,TWIST2,CAMK2A,LRRC15,RECK,TBXA2R,LGR6,CCL5,ADAM17,KANK1	up_AFEM_EC
GO:0048731	0.033	1	2	1247	system development	BP	1	JUP,GATA5	down_VFEM_VSMC
GO:0060339	0.033	0.998	2	5	negative regulation of type I interferon-mediated signaling	BP	0.83	PTPN2,CNOT7	down_VSAP_EC
GO:2000106	0.033	1	1	29	regulation of leukocyte apoptotic process	BP	1	ARG2	up_AORT_VSMC
GO:0003231	0.033	1	1	48	cardiac ventricle development	BP	1	TBX5	up_APUL_VSMC

GO:0006520	0.033039386	0.98876	7	114	cellular amino acid metabolic process	BP	1	NR1H4,THNSL2,NMNAT2,SARDH,NIT2,AZIN2,PM20D1	up_ACOR_EC
GO:0001816	0.033048463	0.98621	10	194	cytokine production	BP	1	CDH3,NR1H4,NDRG2,FZD5,NOD2,IL33,PTGER4,MAVS,AGPAT2,PRKCD	up_ACOR_EC
GO:0031345	0.033081653	0.99288	4	46	negative regulation of cell projection organization	BP	1	RGMA,LRP4,NRP1,PRKCD	up_ACOR_EC
GO:0120036	0.0331	0.979	32	373	plasma membrane bounded cell projection organization	BP	0.832	SIPA1L1,SGK1,PTPN11,EXOC5,FSCN1,DPYSL3,IFRD1,RPGR,KIF5B,DYNLL1,TNIK,UBA6,ZPR1,NEO1,UST,CDH11,CTNNB1,ANLN,APOE,KLF5,LYN,GSK3B,VLDLR,SDC4,HTT,PRMT1,EXT1,DISC1,PAK2,NRP1,ARF4,YWHAH	down_VSAP_EC
GO:0031109	0.0331	0.997	2	36	microtubule polymerization or	BP	1	MZT1,ARHGEF2	up_VPOR_EC
GO:0046660	0.0331	1	1	28	female sex differentiation	BP	1	ESR1	up_AORT_VSMC
GO:0072132	0.0331	1	1	23	mesenchyme morphogenesis	BP	1	GATA5	up_VCAV_VSMC
GO:1905209	0.0331	1	1	13	positive regulation of cardiocyte differentiation	BP	1	NKX2-5	up_VPOR_VSMC
GO:0060306	0.0331	1	1	7	regulation of membrane repolarization	BP	1	CAV1	up_VSAP_VSMC
GO:0086009	0.0331	1	1	7	membrane repolarization	BP	1	CAV1	up_VSAP_VSMC
GO:0051704	0.0332	0.982	18	476	multi-organism process	BP	1	HOXA9,LEP,CD40,BPI,GPAM,CHRM2,SLC17A5,THRB,OVOL1,LRRC15,RECK,TBXA2R,RAB7A,NAPEPLD,SPATA6,CCL5,ADAM17,GCNT4	up_AFEM_EC
GO:0046621	0.0332	0.996	3	8	negative regulation of organ growth	BP	1	SLC6A4,SAV1,FXN	up_VSAP_EC
GO:0050877	0.0332	0.995	3	164	nervous system process	BP	1	NETO1,GATM,HOXC10	up_VSAP_VSMC
GO:0022037	0.0332	1	1	27	metencephalon development	BP	1	WNT7A	up_AORT_VSMC
GO:0003279	0.0332	1	1	48	cardiac septum development	BP	1	TBX5	up_APUL_VSMC
GO:0030388	0.0332	1	1	4	fructose 1,6-bisphosphate metabolic process	BP	1	FBP1	up_VPOR_EC
GO:0003171	0.0332	1	1	10	atrioventricular valve development	BP	1	TBX5	up_VPUL_EC
GO:0050769	0.0333	0.986	11	95	positive regulation of neurogenesis	BP	0.834	ARHGEF2,DPYSL3,HIF1A,CTNNB1,APOE,LYN,MMD,VLDLR,REST,DISC1,NRP1	down_VSAP_EC
GO:0002367	0.0333	0.991	6	26	cytokine production involved in immune	BP	1	TLR3,F2RL1,NOD2,B2M,MAP3K7,IL18R1	up_ACOR_VSMC
GO:0071326	0.0333	0.991	6	26	cellular response to monosaccharide stimulus	BP	1	RAB11B,KCNB1,RAF1,STX4,PPP3CB,RAB11FIP2	up_VSAP_EC
GO:0071331	0.0333	0.991	6	26	cellular response to hexose stimulus	BP	1	RAB11B,KCNB1,RAF1,STX4,PPP3CB,RAB11FIP2	up_VSAP_EC
GO:0048010	0.0333	0.993	4	20	vascular endothelial growth factor receptor	BP	0.834	VEGFA,SULF1,HIF1A,NRP1	down_VSAP_EC
GO:0031328	0.0333	0.993	4	512	positive regulation of cellular biosynthetic	BP	1	EYA1,HOXA9,HOXA11,SHOX2	up_VFEM_VSMC
GO:0061469	0.0333	0.998	2	4	regulation of type B pancreatic cell	BP	1	BIRC5,ENSCAFG00000005299	down_ACOR_VSMC
GO:0048646	0.0333	0.998	2	349	anatomical structure formation involved in	BP	1	TGFBI,GATA5	down_VSAP_VSMC
GO:0003013	0.0333	0.997	2	121	circulatory system process	BP	1	LNPEP,SGCD	up_AORT_EC
GO:0014819	0.0333	0.998	2	4	regulation of skeletal muscle contraction	BP	0.766	KCNJ2,ATP2A1	up_VCAV_EC
GO:1901889	0.0333	1	1	8	negative regulation of cell junction assembly	BP	1	ACVRL1	down_ACOR_EC
GO:0035904	0.0333	1	1	25	aorta development	BP	1	EYA1	up_AFEM_VSMC
GO:0010888	0.0333	1	1	4	negative regulation of lipid storage	BP	1	ABHD5	up_VPOR_EC
GO:0070887	0.033363611	0.97976	29	775	cellular response to chemical stimulus	BP	1	NR1H4,SOX9,AQP1,ENSCAFG00000004331,KCNB1,EDEM2,CCDC3,AGRN,NOD2,RGMA,PDE4A,SMAD6,ACTN2,PAK3,MMP28,GUCY1B1,PTGER4,ELMO2,ADAMTSL2,MAVS,FOXC2,LRRC15,IFIT2,LRP4,LRRC4,RARRES2,PRKACA,NRP1,PRKCD	up_ACOR_EC

GO:0048523	0.0334	0.974	133	1399	negative regulation of cellular process	BP	0.766	EPHB1,PLK2,EDNRB,HCLS1,IL7R,ADGRF5,KIT,PPP2R5A,CXCL8,PRKCH,ARHGEF15,IFRD1,TIE1,CDA,TNFAIP3,ADAMTS9,TNIP1,PDGFB,PODXL,CD34,PDE4D,MEF2C,FERMT1,ATF3,CHD7,DLL4,RAPGEF1,WWP1,TLR4,NLRC5,STC1,GATA3,MMP28,EDN1,HOXB8,APLNR,ARAP3,HOMER3,ARHGAP28,SOX18,TMOD1,EGFL7,FIGNL1,PELI1,PPP3CA,FAM3C,MTSS1,ARG2,CASP8,MTOR,TENT2,HOOK3,MAP3K3,BMP2,CDYL,HMG20A,HHEX,CREM,CAV2,PREX2,SH3BP5,RSC1A1,ENC1,ITPKB,GDPD5,RIOK3,ANGPT2,PROX1,UHRF2,ENSCAFG00000010224,MDFI,ENSCAFG00000010596,STMN1,HMBOX1,PAK2,MCC,THOC1,SWAP70,SMARCA4,APOD,NCK1,TIMELESS,OTUD4,CD200,JAG1,PTGS2,ETAA1,RAPGEF2,TCERG1,PLCG2,CTDSPL2,CREB1,BCL10,BASP1,USP28,TAOK1,CTNNA1,CNOT7,ENSCAFG00000002897,HMGB1,BLM,RIPK1,ITGA6,RAD21,RAD17,RGS2,SKIL,MTA2,ARHGDIB,CUL4A,ZW10,KDM5A,HIF1A,KDM3A,ATRX,NR4A1,HIST2H2AC,ARID5A,TOPBP1,ENSCAFG00000002015,VASH1,DCP2,TNFAIP1,IVNS1ABP,FKBP15,RGL2,TMOD3,ZEB1,ARHGEF2,PEAR1,FBXO5,ELK3,TJP2	up_VCAV_EC
GO:0003002	0.0334	0.982	18	118	regionalization	BP	1	HOXA11,EMX2,HOXA7,HOXA9,CDON,AXIN2,HOXD3,DLL1,HOXA6,OSR1,ZBTB16,RARG,HOXC4,IRX3,HOXC10,LRP4,KDM2B,CITED2	up_VSAP_EC
GO:0023052	0.0334	0.988	10	1602	signaling	BP	1	TBX5,NR1H4,RGS1,STK32B,CCL24,HTR7,PTGER2,FLRT3,ARHGEF2,NEO1	up_VPUL_EC
GO:0009058	0.0334	0.99	9	1910	biosynthetic process	BP	1	EYA1,GATM,HOXA10,HOXA9,TBX15,FBP1,EMX2,HOXA11,SHOX2	up_VFEM_VSMC
GO:0010033	0.0334	0.988	8	772	response to organic substance	BP	1	ACVRL1,LYN,IGF1R,NRP2,SULF1,STX4,ASPN,NEDD4	down_ACOR_EC
GO:0048645	0.0334	0.989	7	33	animal organ formation	BP	1	GATA5,BMP4,FGFR2,CITED2,SMARCD3,TBX5,FRS2	up_ACOR_VSMC
GO:0035282	0.0334	0.995	3	30	segmentation	BP	1	IRX3,DLL1,AXIN2	up_AFEM_EC
GO:0010575	0.0334	0.996	3	8	positive regulation of vascular endothelial growth factor production	BP	1	HPSE,CCBE1,SULF2	up_VSAP_EC
GO:0019438	0.0334	1	2	1258	aromatic compound biosynthetic process	BP	1	JUP,GATA5	down_VFEM_VSMC
GO:0048729	0.0334	0.997	2	209	tissue morphogenesis	BP	1	GATA5,CA2	up_VCAV_VSMC
GO:0000470	0.0334	1	1	9	maturation of LSU-rRNA	BP	1	WDR12	down_ACOR_EC
GO:0048247	0.0334	1	1	16	lymphocyte chemotaxis	BP	1	HSD3B7	down_VPOR_EC
GO:0003002	0.0334	1	1	118	regionalization	BP	1	GATA5	down_VFEM_VSMC
GO:0051196	0.0334	1	1	13	regulation of coenzyme metabolic process	BP	1	FBP1	up_VFEM_VSMC
GO:0098901	0.0334	1	1	7	regulation of cardiac muscle cell action	BP	1	CAV1	up_VSAP_VSMC
GO:0003008	0.033471567	0.98331	15	336	system process	BP	1	TMOD1,BRSK1,SOX9,AQP1,GNAO1,NOD2,PDE4A,INSYN1,PLS1,ACTN2,RNLS,USP53,MAPK8IP2,FOXC2,CLSTN1	up_ACOR_EC
GO:0009792	0.0335	0.98	28	231	embryo development ending in birth or egg hatching	BP	1	ACSL4,EIF4A3,HINFP,HOXA3,GINS4,PRMT1,XYLT1,MMP14,SLC30A1,NEK2,RNASEH2B,HOXA7,HOXB7,BRCA1,E2F8,FOSL1,PLK4,HOXC6,SMARCB1,HOXB5,RBBP8,NOLC1,PHACTR4,TBX15,HOXA9,EMG1,RTCB,SIX4	down_ACOR_VSMC
GO:0045005	0.0335	0.992	5	21	DNA-dependent DNA replication maintenance	BP	1	POLE,RAD51,TONSL,SMARCAL1,GEN1	down_ACOR_VSMC
GO:0050848	0.0335	0.995	3	30	regulation of calcium-mediated signaling	BP	1	CMKLR1,LMCD1,TMEM100	up_AFEM_EC
GO:0018130	0.0335	1	2	1261	heterocycle biosynthetic process	BP	1	JUP,GATA5	down_VFEM_VSMC
GO:1904590	0.0335	0.998	2	5	negative regulation of protein import	BP	0.766	FERMT1,APOD	up_VCAV_EC
GO:0042308	0.0335	0.998	2	5	negative regulation of protein import into	BP	0.766	FERMT1,APOD	up_VCAV_EC
GO:0030219	0.0335	1	1	16	megakaryocyte differentiation	BP	1	GABPA	down_AFEM_EC
GO:0044818	0.0335	0.999	1	15	mitotic G2/M transition checkpoint	BP	1	TAOK1	up_AORT_EC
GO:1903409	0.0335	1	1	24	reactive oxygen species biosynthetic process	BP	1	CYP1A1	up_VCAV_VSMC

GO:0060562	0.0336	0.984	13	120	epithelial tube morphogenesis	BP	0.84	PRICKLE1,PRKACB,ENSCAFG00000032470,TMED2,ILK,HOXB7,HIF1A,MAGED1,CTNNB1,STARD13,SDC4,SIX4,NRP1	down_VSAP_EC
GO:0002237	0.0336	0.985	12	85	response to molecule of bacterial origin	BP	0.766	CXCL8,TNFAIP3,TNIP1,PDE4D,MEF2C,TLR4,IRF8,PELI1,ABCA1,PLCG2,BCL10,GCH1	up_VCAV_EC
GO:0048704	0.0336	0.988	8	46	embryonic skeletal system morphogenesis	BP	1	EIF4A3,HOXA3,MMP14,HOXA7,HOXB7,HOXB5,TBX15,SIX4	down_ACOR_VSMC
GO:0008344	0.0336	0.991	6	25	adult locomotory behavior	BP	1	GLRB,APP,CHD7,CLN8,OXR1,PARK7	up_ACOR_VSMC
GO:0048598	0.0336	0.995	3	207	embryonic morphogenesis	BP	1	TBX5,HOXA3,FLRT3	up_VPUL_EC
GO:0060900	0.0336	0.998	2	4	embryonic camera-type eye formation	BP	0.766	PROX1,ALDH1A3	up_VCAV_EC
GO:0034381	0.0336	1	1	10	plasma lipoprotein particle clearance	BP	1	NR1H4	up_VPUL_EC
GO:0023014	0.0337	0.979	29	248	signal transduction by protein phosphorylation	BP	0.766	EPHB1,TEK,KIT,MAP3K11,TNIP1,PDGFB,STK10,SH2D3C,MEF2C,ATF3,RAPGEF1,TLR4,EDN1,MAP3K3,BMP2,SLK,MAPK13,ITPKB,MADD,PAK2,PRKD2,TLR3,RAPGEF2,TAOK1,HMGB1,RIPK1,PDGFD,JCAD,CDON	up_VCAV_EC
GO:1903706	0.0337	0.985	11	119	regulation of hemopoiesis	BP	1	NOTCH2,FBN1,TFE3,CLSTN1,GLI3,ZFP36L1,LOXL3,RAB7B,FOXO3,ZBTB16,ANKRD54	down_VCAV_EC
GO:0032635	0.0337	0.989	7	37	interleukin-6 production	BP	0.766	TNFAIP3,TLR4,MAPK13,TLR3,HMGB1,NOS2,ARHGEF2	up_VCAV_EC
GO:0060537	0.0337	0.997	2	117	muscle tissue development	BP	1	NKX2-5,CYP26B1	up_VPOR_VSMC
GO:0006367	0.0337	1	1	32	transcription initiation from RNA polymerase II	BP	1	ESR1	up_AORT_VSMC
GO:0017156	0.0337	1	1	32	calcium ion regulated exocytosis	BP	1	WNT7A	up_AORT_VSMC
GO:0015698	0.0337	1	1	24	inorganic anion transport	BP	1	CA2	up_VCAV_VSMC
GO:0003205	0.033742723	0.99102	5	67	cardiac chamber development	BP	1	GATA4,SMAD6,FOXC2,TBX5,NRP1	up_ACOR_EC
GO:0050890	0.0338	0.997	2	67	cognition	BP	1	NETO1,GATM	up_VSAP_VSMC
GO:0048752	0.0338	1	1	5	semicircular canal morphogenesis	BP	1	HOXA1	down_ACOR_EC
GO:0042462	0.0338	1	1	9	eye photoreceptor cell development	BP	1	NTRK2	up_APUL_EC
GO:0032925	0.0338	1	1	4	regulation of activin receptor signaling	BP	1	ACVR2A	up_VPOR_EC
GO:0006047	0.0338	1	1	4	UDP-N-acetylglucosamine metabolic process	BP	1	GNPNAT1	up_VPOR_EC
GO:0051645	0.0338	1	1	4	Golgi localization	BP	1	HOOK3	up_VPOR_EC
GO:1902742	0.0338	0.999	1	14	apoptotic process involved in development	BP	1	NKX2-5	up_VPOR_VSMC
GO:0010803	0.0338	1	1	10	regulation of tumor necrosis factor-mediated signaling pathway	BP	1	NR1H4	up_VPUL_EC
GO:0021522	0.0338	1	1	8	spinal cord motor neuron differentiation	BP	1	HOXC10	up_VSAP_VSMC
GO:0046434	0.0339	0.989	7	52	organophosphate catabolic process	BP	0.84	ENPP1,PGK1,TPI1,PGAM1,HIF1A,ENTPD4,ALDOC	down_VSAP_EC
GO:0034341	0.0339	0.99	6	43	response to interferon-gamma	BP	0.84	FLNB,MYO1C,KIF5B,PTPN2,CDC37,GBP5	down_VSAP_EC
GO:0060337	0.0339	0.995	3	12	type I interferon signaling pathway	BP	0.84	PTPN2,CNOT7,CDC37	down_VSAP_EC
GO:0071357	0.0339	0.995	3	12	cellular response to type I interferon	BP	0.84	PTPN2,CNOT7,CDC37	down_VSAP_EC
GO:0002726	0.0339	0.996	3	8	positive regulation of T cell cytokine production	BP	1	B2M,MAP3K7,IL18R1	up_ACOR_VSMC
GO:0006091	0.0339	0.997	2	139	generation of precursor metabolites and energy	BP	1	SUCLG2,NDUFA10	down_AFEM_EC
GO:0007417	0.0339	0.997	2	235	central nervous system development	BP	1	HOXC10,WNT7A	up_AORT_VSMC
GO:0002237	0.0339	0.997	2	85	response to molecule of bacterial origin	BP	1	NR1H4,MRC1	up_VPUL_EC
GO:0090224	0.0339	0.999	1	16	regulation of spindle organization	BP	1	GPSM2	down_AFEM_EC
GO:0001658	0.0339	0.999	1	24	branching involved in ureteric bud	BP	1	EYA1	up_AFEM_VSMC
GO:1903556	0.0339	0.999	1	14	negative regulation of tumor necrosis factor superfamily cytokine	BP	1	BPI	up_AORT_EC
GO:1902667	0.0339	1	1	5	regulation of axon guidance	BP	1	SEMA3A	up_VPOR_EC
GO:1905207	0.0339	0.999	1	17	regulation of cardiocyte differentiation	BP	1	TBX5	up_VPUL_VSMC

GO:1901800	0.033940422	0.99264	4	46	positive regulation of proteasomal protein catabolic process	BP	1	EDEM2,PIAS1,CLU,RNF114	up_ACOR_EC
GO:0022008	0.034	0.977	46	406	neurogenesis	BP	0.766	EPHB1,EDNRB,PRKCH,IFRD1,RELN,MEF2C,CHD7,DLL4,RAPGEF1,TLR4,EIF2B2,GATA3,RAB11A,TCF4,NTN4,PPP3CA,MTOR,NAV2,HOOK3,BMP2,HMG20A,PREX2,GDPD5,PROX1,ENSCAFG00000010224,STMN1,MMP24,PAK2,APOD,NCK1,FES,JAG1,MMD,RAPGEF2,CREB1,ZC4H2,CTNNA1,NUP133,SPTBN4,SKIL,HIF1A,ALS2,ZEB1,ARHGEF2,CDON,ZNF609	up_VCAV_EC
GO:1902905	0.034	0.985	11	72	positive regulation of supramolecular fiber	BP	0.766	ARHGEF15,BAIAP2L1,EDN1,MTOR,NCKAP1,PROX1,IQGAP2,SWAP70,NCK1,FES,ARPC5L	up_VCAV_EC
GO:0007275	0.034	0.988	9	1370	multicellular organism development	BP	1	TBX5,HOXA3,NR1H4,EVI2B,HOXA2,CCL24,FLRT3,ARHGEF2,NEO1	up_VPUL_EC
GO:0070887	0.034	0.988	8	775	cellular response to chemical stimulus	BP	1	ACVRL1,LYN,IGF1R,NRP2,SULF1,STX4,ASPN,NEDD4	down_ACOR_EC
GO:2001235	0.034	0.988	8	69	positive regulation of apoptotic signaling	BP	0.84	PTPN2,MAGED1,BNIP3,FAM162A,ATF3,SH3GLB1,PAK2,INHBA	down_VSAP_EC
GO:0019751	0.034	0.99	6	33	polyol metabolic process	BP	0.766	GK5,SPTLC2,ITPKB,PLCG2,GCH1,IMPA1	up_VCAV_EC
GO:0035024	0.034	0.996	3	8	negative regulation of Rho protein signal	BP	0.766	ARAP3,STMN1,TNFAIP1	up_VCAV_EC
GO:0048596	0.034	0.996	3	9	embryonic camera-type eye morphogenesis	BP	0.766	PROX1,ALDH1A3,ZEB1	up_VCAV_EC
GO:0061318	0.034	0.996	3	8	renal filtration cell differentiation	BP	0.766	PODXL,JAG1,BASP1	up_VCAV_EC
GO:0072010	0.034	0.996	3	8	glomerular epithelium development	BP	0.766	PODXL,JAG1,BASP1	up_VCAV_EC
GO:0072112	0.034	0.996	3	8	glomerular visceral epithelial cell	BP	0.766	PODXL,JAG1,BASP1	up_VCAV_EC
GO:0072311	0.034	0.996	3	8	glomerular epithelial cell differentiation	BP	0.766	PODXL,JAG1,BASP1	up_VCAV_EC
GO:0010572	0.034	0.998	2	4	positive regulation of platelet activation	BP	0.766	SELP,TLR4	up_VCAV_EC
GO:0021783	0.034	0.999	1	9	preganglionic parasympathetic fiber	BP	1	SIX1	up_VFEM_EC
GO:0003044	0.034	1	1	8	regulation of systemic arterial blood pressure mediated by a chemical	BP	1	AVPR1A	up_VFEM_EC
GO:0072074	0.034	1	1	8	kidney mesenchyme development	BP	1	SIX1	up_VFEM_EC
GO:0008542	0.034	0.999	1	13	visual learning	BP	1	NETO1	up_VFEM_VSMC
GO:0055081	0.034	0.999	1	17	anion homeostasis	BP	1	HOXA3	up_VPUL_VSMC
GO:0007044	0.034008766	0.99471	3	27	cell-substrate junction assembly	BP	1	ARHGAP6,ACTN2,NRP1	up_ACOR_EC
GO:0000578	0.034089891	0.99726	2	11	embryonic axis specification	BP	1	FZD5,SMAD6	up_ACOR_EC
GO:0019081	0.0341	0.996	3	8	viral translation	BP	1	EIF3G,EIF3L,EIF3D	up_VSAP_EC
GO:0006729	0.0341	0.998	2	4	tetrahydrobiopterin biosynthetic process	BP	1	PCBD1,PTS	up_ACOR_VSMC
GO:0046146	0.0341	0.998	2	4	tetrahydrobiopterin metabolic process	BP	1	PCBD1,PTS	up_ACOR_VSMC
GO:0043069	0.0341	0.997	2	262	negative regulation of programmed cell death	BP	1	WNT7A,ARG2	up_AORT_VSMC
GO:1901653	0.0341	0.997	2	84	cellular response to peptide	BP	1	NR1H4,ARHGEF2	up_VPUL_EC
GO:0002183	0.0341	0.999	1	16	cytoplasmic translational initiation	BP	1	EIF3M	down_AFEM_EC
GO:0016042	0.0341	1	1	80	lipid catabolic process	BP	1	PLCL1	down_VFEM_EC
GO:0010631	0.0341	1	1	118	epithelial cell migration	BP	1	JUP	down_VFEM_VSMC
GO:0090132	0.0341	1	1	118	epithelium migration	BP	1	JUP	down_VFEM_VSMC
GO:0055025	0.0341	0.999	1	17	positive regulation of cardiac muscle tissue	BP	1	TBX5	up_VPUL_VSMC
GO:0098815	0.0341	0.999	1	9	modulation of excitatory postsynaptic potential	BP	1	NETO1	up_VSAP_VSMC
GO:0022613	0.0342	0.98	25	208	ribonucleoprotein complex biogenesis	BP	1	USP4,EDC3,SLC29A1,NOP56,RAN,FBL,DIS3,NOC4L,NOC2L,DDX28,RSL1D1,RRP1,DDX39B,SF3A2,ENSCAFG00000019620,NOP14,PRMT5,NOP58,ENSCAFG00000015189,EMG1,EXOSC5,GEMIN7,SF3A1,EIF3M,NOB1	down_ACOR_VSMC
GO:0010468	0.0342	1	2	1270	regulation of gene expression	BP	1	JUP,GATA5	down_VFEM_VSMC

GO:0048871	0.0342	0.997	2	120	multicellular organismal homeostasis	BP	1	HOXC10,GATM	up_VFEM_VSMC
GO:0060562	0.0342	0.997	2	120	epithelial tube morphogenesis	BP	1	EYA1,HOXA11	up_VFEM_VSMC
GO:0051438	0.0342	0.999	1	24	regulation of ubiquitin-protein transferase	BP	1	LIMK1	up_VCAV_VSMC
GO:0003229	0.0342	0.999	1	17	ventricular cardiac muscle tissue development	BP	1	TBX5	up_VPUL_VSMC
GO:0051129	0.0343	0.979	30	221	negative regulation of cellular component organization	BP	1	FGF13,SMAD6,ARHGAP28,MID1,FEZ1,ITM2C,BMP4,EVI5L,TUBB4A,ATRX,ATXN2,ZNF365,AR,LMO4,BNIP3,CLSTN1,NRP1,LRPAP1,ULK2,EVL,TBX20,PSEN1,DUSP22,ASAP1,FAM107A,B2M,SCAMP5,FEZ2,INPP5K,WASL	up_ACOR_VSMC
GO:1902531	0.0343	0.991	5	536	regulation of intracellular signal transduction	BP	1	NR1H4,CCL24,PTGER2,FLRT3,ARHGEF2	up_VPUL_EC
GO:0051457	0.0343	0.996	3	8	maintenance of protein location in nucleus	BP	1	MORC3,CIZ1,TAF8	up_VSAP_EC
GO:1904668	0.0343	0.998	2	4	positive regulation of ubiquitin protein ligase	BP	1	UBE2S,CDC20	down_ACOR_VSMC
GO:0048568	0.0343	0.997	2	148	embryonic organ development	BP	1	CITED2,TBX15	down_VPOR_EC
GO:0060537	0.0343	0.997	2	117	muscle tissue development	BP	1	EYA1,SHOX2	up_VFEM_VSMC
GO:0008015	0.0343	1	1	120	blood circulation	BP	1	JUP	down_VFEM_VSMC
GO:0055123	0.0343	1	1	40	digestive system development	BP	1	GATA5	down_VSAP_VSMC
GO:0048745	0.0343	0.999	1	8	smooth muscle tissue development	BP	1	SIX1	up_VFEM_EC
GO:0001759	0.0343	0.999	1	13	organ induction	BP	1	HOXA11	up_VFEM_VSMC
GO:0043266	0.0343	0.999	1	14	regulation of potassium ion transport	BP	1	NETO1	up_VFEM_VSMC
GO:0050704	0.03430161	0.99724	2	11	regulation of interleukin-1 secretion	BP	1	FZD5,NOD2	up_ACOR_EC
GO:0050922	0.034302473	0.99724	2	11	negative regulation of chemotaxis	BP	1	MMP28,NRP1	up_ACOR_EC
GO:0012501	0.0344	0.977	44	561	programmed cell death	BP	0.846	ARHGEF2,VEGFA,P4HB,ASNS,NR1H4,PLK3,HSPA5,SERPINE1,ETV6,ERO1A,CASP3,HYOU1,EXOC5,AARS,FHL2,PTGS2,UACA,HIF1A,DNAJC10,TNIK,ZPR1,PTPN2,MTDH,MAGED1,CTNNB1,CRYAB,LYN,BNIP3,DDIT3,FAM162A,ATF3,GSK3B,SH3GLB1,HTT,REST,GLO1,PAK2,INHBA,SIX4,CDKN1A,NRP1,SPINK2,ARF4,MADD	down_VSAP_EC
GO:0006874	0.0344	0.984	13	91	cellular calcium ion homeostasis	BP	0.771	ATP2B1,PDE4D,CHD7,GPR4,STC1,EDN1,APLNR,PDZD8,SWAP70,PLCG2,HMGB1,MCU,ATP2A1	up_VCAV_EC
GO:0009725	0.0344	0.986	9	188	response to hormone	BP	1	LEP,LBH,CRY2,FIBIN,CHRM2,AVPR1A,CAMK2A,NR3C1,KANK1	up_AFEM_EC
GO:0061217	0.0344	0.995	3	13	regulation of mesonephros	BP	0.846	HOXB7,MAGED1,SIX4	down_VSAP_EC
GO:0035295	0.0344	0.998	2	356	tube development	BP	1	TGFBI,GATA5	down_VSAP_VSMC
GO:0071071	0.0344	0.999	1	8	regulation of phospholipid biosynthetic process	BP	1	ACSL3	down_ACOR_EC
GO:0051932	0.0344	0.999	1	9	synaptic transmission, GABAergic	BP	1	PLCL1	up_APUL_EC
GO:1990774	0.0344	0.999	1	9	tumor necrosis factor secretion	BP	1	NR1H4	up_APUL_EC
GO:0007254	0.034464514	0.99077	5	67	JNK cascade	BP	1	FZD5,NOD2,TNFRSF19,MAPK8IP2,SH3RF3	up_ACOR_EC
GO:0030072	0.0345	0.987	9	48	peptide hormone secretion	BP	1	LEP,TM7SF3,PRKCE,CHD7,CCL5,RAB11B,EDN1,PFKL,PARK7	up_ACOR_VSMC
GO:0045638	0.0345	0.989	7	33	negative regulation of myeloid cell	BP	1	HOXA7,HOXA9,DLL1,ZBTB16,ZFP36L1,PIK3R1,MYC	up_VSAP_EC
GO:0019221	0.0345	0.989	6	102	cytokine-mediated signaling pathway	BP	1	LEP,GREM2,CYLD,LRRC15,CCL5,ADAM17	up_AFEM_EC
GO:0090257	0.0345	0.992	4	52	regulation of muscle system process	BP	1	LMCD1,CHRM2,TBXA2R,DMPK	up_AFEM_EC
GO:2000772	0.0345	0.993	4	15	regulation of cellular senescence	BP	0.771	PLK2,ARG2,MAP3K3,VASH1	up_VCAV_EC
GO:0032332	0.0345	0.998	2	6	positive regulation of chondrocyte	BP	1	GLI3,ZBTB16	down_VCAV_EC
GO:1903038	0.0345	0.999	1	30	negative regulation of leukocyte cell-cell	BP	1	ARG2	up_AORT_VSMC
GO:0006541	0.0345	0.999	1	9	glutamine metabolic process	BP	1	NR1H4	up_APUL_EC

GO:0019216	0.0346	0.983	14	101	regulation of lipid metabolic process	BP	0.771	TEK,ADGRF5,PPP2R5A,PDGFB,MTOR,SAMD8,BMP2,PROX1,ENSCAFG00000010596,SH3GLB1,APOD,PTGS2,CREB1,NR1D2	up_VCAV_EC
GO:0031175	0.0346	0.993	4	224	neuron projection development	BP	1	LYN,SPG11,NRP2,NEDD4	down_ACOR_EC
GO:0071604	0.0346	0.995	3	10	transforming growth factor beta production	BP	0.771	CD34,CD200,HIF1A	up_VCAV_EC
GO:0071634	0.0346	0.995	3	10	regulation of transforming growth factor beta	BP	0.771	CD34,CD200,HIF1A	up_VCAV_EC
GO:0048568	0.0346	0.997	2	148	embryonic organ development	BP	1	HOXA3,HOXB5	up_VPUL_VSMC
GO:0003013	0.0346	1	1	121	circulatory system process	BP	1	JUP	down_VFEM_VSMC
GO:0009566	0.0346	0.999	1	24	fertilization	BP	1	HOXA9	up_AFEM_VSMC
GO:0001974	0.0346	0.999	1	13	blood vessel remodeling	BP	1	HOXA3	up_VPOR_VSMC
GO:0003209	0.0346	0.999	1	14	cardiac atrium morphogenesis	BP	1	NKX2-5	up_VPOR_VSMC
GO:0003230	0.0346	0.999	1	14	cardiac atrium development	BP	1	NKX2-5	up_VPOR_VSMC
GO:0010677	0.0346	0.999	1	14	negative regulation of cellular carbohydrate	BP	1	FBP1	up_VFEM_VSMC
GO:0048103	0.0346	0.999	1	10	somatic stem cell division	BP	1	ARHGEF2	up_VPUL_EC
GO:0008202	0.0347	0.984	12	80	steroid metabolic process	BP	1	SQLE,LSS,SREBF1,HMGCR,SF1,INSIG1,ENSCAFG00000018572,DHCR24,MVD,FDFT1,SGPL1,LIPC	down_ACOR_VSMC
GO:0007268	0.0347	0.988	7	130	chemical synaptic transmission	BP	1	KCNC4,GPR1,CHRM2,SLC6A6,HTR1D,CAMK2A,DMPK	up_AFEM_EC
GO:0098916	0.0347	0.988	7	130	anterograde trans-synaptic signaling	BP	1	KCNC4,GPR1,CHRM2,SLC6A6,HTR1D,CAMK2A,DMPK	up_AFEM_EC
GO:0072160	0.0347	0.999	1	9	nephron tubule epithelial cell differentiation	BP	1	MTSS1	up_VFEM_EC
GO:0042092	0.034704212	0.99719	2	11	type 2 immune response	BP	1	NOD2,IL33	up_ACOR_EC
GO:1901700	0.034732295	0.98219	16	364	response to oxygen-containing compound	BP	1	NR1H4,SOX9,AQP1,ENSCAFG00000004331,KCNB1,EDEM2,AGRN,PTGIR,NOD2,ACTN2,GUCY1B1,PTGER4,FOXC2,RARRES2,PRKACA,PRKCD	up_ACOR_EC
GO:0030218	0.0348	0.99	6	42	erythrocyte differentiation	BP	0.853	CASP3,HIF1A,PTPN2,LYN,PRMT1,INHBA	down_VSAP_EC
GO:0006721	0.0348	0.995	3	14	terpenoid metabolic process	BP	1	CYP1B1,RETSAT,RDH10	down_VCAV_EC
GO:0043101	0.0348	0.998	2	6	purine-containing compound salvage	BP	1	ADK,AMPD2	down_VCAV_EC
GO:0048732	0.0348	0.997	2	116	gland development	BP	1	HOXA3,NKX2-5	up_VPOR_VSMC
GO:0040037	0.0348	1	1	6	negative regulation of fibroblast growth factor receptor signaling	BP	1	SULF1	down_ACOR_EC
GO:1900120	0.0348	0.999	1	7	regulation of receptor binding	BP	1	HOXA3	down_ACOR_EC
GO:0007422	0.0348	0.999	1	18	peripheral nervous system development	BP	1	CITED2	down_VPOR_EC
GO:0032637	0.0348	0.999	1	26	interleukin-8 production	BP	1	TLR2	up_AFEM_VSMC
GO:0003170	0.0348	0.999	1	25	heart valve development	BP	1	GATA5	up_VCAV_VSMC
GO:0006536	0.0348	0.999	1	10	glutamate metabolic process	BP	1	NR1H4	up_VPUL_EC
GO:0043207	0.0349	0.979	29	216	response to external biotic stimulus	BP	1	VEGFD,TLR1,PLCG2,TMEM255A,COCH,MX1,ABCA1,TLR3,ISG15,PTGIR,SASH1,PLAC8B,ANKRD1,F2RL1,BNIP3,SLC17A5,PTPN22,GPM6A,PRKCE,PARP9,CHD7,CCL5,NOD2,DDIT4,NAALADL2,B2M,SERINC3,TNFRSF11B,WASL	up_ACOR_VSMC
GO:0051707	0.0349	0.979	29	216	response to other organism	BP	1	VEGFD,TLR1,PLCG2,TMEM255A,COCH,MX1,ABCA1,TLR3,ISG15,PTGIR,SASH1,PLAC8B,ANKRD1,F2RL1,BNIP3,SLC17A5,PTPN22,GPM6A,PRKCE,PARP9,CHD7,CCL5,NOD2,DDIT4,NAALADL2,B2M,SERINC3,TNFRSF11B,WASL	up_ACOR_VSMC
GO:0003073	0.0349	0.991	5	20	regulation of systemic arterial blood pressure	BP	1	ADRA1B,SLC2A5,F2RL1,EDN1,NCALD	up_ACOR_VSMC
GO:1903426	0.0349	0.993	4	17	regulation of reactive oxygen species	BP	0.771	CD34,TLR4,RAB27A,ARG2	up_VCAV_EC
GO:0060479	0.0349	0.996	3	8	lung cell differentiation	BP	1	THRB,SAV1,NFIB	up_VSAP_EC

GO:0046006	0.0349	0.998	2	6	regulation of activated T cell proliferation	BP	1	GPAM,PRNP	down_VCAV_EC
GO:0097152	0.0349	0.998	2	5	mesenchymal cell apoptotic process	BP	0.853	ETV6,HIF1A	down_VSAP_EC
GO:0006101	0.0349	0.999	1	16	citrate metabolic process	BP	1	SUCLG2	down_AFEM_EC
GO:0090130	0.0349	1	1	121	tissue migration	BP	1	JUP	down_VFEM_VSMC
GO:0010002	0.0349	0.999	1	9	cardioblast differentiation	BP	1	TBX5	up_APUL_EC
GO:0003206	0.0349	1	1	51	cardiac chamber morphogenesis	BP	1	TBX5	up_APUL_VSMC
GO:0060078	0.0349	0.999	1	17	regulation of postsynaptic membrane potential	BP	1	NETO1	up_VFEM_VSMC
GO:0099565	0.0349	0.999	1	17	chemical synaptic transmission, postsynaptic	BP	1	NETO1	up_VFEM_VSMC
GO:2001239	0.0349	0.999	1	14	regulation of extrinsic apoptotic signaling pathway in absence of	BP	1	EYA1	up_VFEM_VSMC
GO:2000146	0.034987276	0.98915	6	92	negative regulation of cell motility	BP	1	SEMA6D,ZMYND8,MMP28,PTGER4,ARHGDIB,TBX5	up_ACOR_EC
GO:0030334	0.035	0.98	24	267	regulation of cell migration	BP	0.853	ARHGEF2,VEGFA,SULF1,HSPA5,SERPINE1,SPARC,JCAD,DPYSL3,PRCP,MYO1C,PIK3C2A,MSN,HIF1A,ADAM17,STARD13,APOE,PTP4A1,MMP28,LYN,STRAP,NEXN,SDC4,ZMYND8,NRP1	down_VSAP_EC
GO:0006793	0.035	0.987	9	948	phosphorus metabolic process	BP	1	ACVRL1,LYN,IGF1R,PRKAR2A,CSGALNACT1,ACSL3,MYO1D,TNRC6B,ASPN	down_ACOR_EC
GO:0032680	0.035	0.989	7	36	regulation of tumor necrosis factor production	BP	0.771	TNFAIP3,CD34,TLR4,ARG2,TLR3,RIPK1,ARHGEF2	up_VCAV_EC
GO:0042060	0.035	0.989	6	103	wound healing	BP	1	F5,F3,TFPI2,LGR6,ADAM17,KANK1	up_AFEM_EC
GO:0002027	0.035	0.991	5	20	regulation of heart rate	BP	1	KCNE3,RNLS,JUP,EDN1,BVES	up_ACOR_VSMC
GO:0043549	0.035	0.992	4	244	regulation of kinase activity	BP	1	LYN,IGF1R,PRKAR2A,ASPN	down_ACOR_EC
GO:0018279	0.035	0.993	4	14	protein N-linked glycosylation via	BP	1	ST3GAL5,MAGT1,STT3B,FUT8	up_ACOR_VSMC
GO:0070233	0.035	0.995	3	9	negative regulation of T cell apoptotic process	BP	0.771	IL7R,ARG2,HIF1A	up_VCAV_EC
GO:0006817	0.035	0.998	2	5	phosphate ion transport	BP	0.853	SLC20A1,SLC20A2	down_VSAP_EC
GO:0000466	0.035	0.999	1	8	maturation of 5.8S rRNA from tricistronic rRNA transcript (SSU-rRNA, 5.8S	BP	1	WDR12	down_ACOR_EC
GO:0050729	0.035	0.999	1	25	positive regulation of inflammatory response	BP	1	TLR2	up_AFEM_VSMC
GO:0010894	0.035	0.999	1	9	negative regulation of steroid biosynthetic	BP	1	NR1H4	up_APUL_EC
GO:0045939	0.035	0.999	1	9	negative regulation of steroid metabolic process	BP	1	NR1H4	up_APUL_EC
GO:0071709	0.035	0.999	1	8	membrane assembly	BP	1	CAV1	up_VSAP_VSMC
GO:1900449	0.035	0.999	1	9	regulation of glutamate receptor signaling	BP	1	NETO1	up_VSAP_VSMC
GO:0046483	0.0351	0.99	8	1948	heterocycle metabolic process	BP	1	IVNS1ABP,EXTL2,DARS,SUCLG2,SRSF6,GABPA,NDUFA10,TSEN15	down_AFEM_EC
GO:0065009	0.0351	0.99	6	735	regulation of molecular function	BP	1	HOXA3,NR1H4,CCL24,PTGER2,FLRT3,ARHGEF2	up_VPUL_EC
GO:0003156	0.0351	0.991	5	20	regulation of animal organ formation	BP	1	GATA5,BMP4,CITED2,TBX5,FRS2	up_ACOR_VSMC
GO:0030500	0.0351	0.993	4	25	regulation of bone mineralization	BP	1	BMPR1A,DDR2,OMD,GREM1	down_VCAV_EC
GO:0046189	0.0351	0.995	3	9	phenol-containing compound biosynthetic	BP	0.771	GATA3,RAPGEF2,GCH1	up_VCAV_EC
GO:0031018	0.0351	0.996	3	8	endocrine pancreas development	BP	1	HNF4A,IL6R,DLL1	up_VSAP_EC
GO:0042532	0.0351	0.998	2	4	negative regulation of tyrosine phosphorylation	BP	1	CAV1,NF2	down_ACOR_VSMC
GO:0050770	0.0351	0.997	2	39	regulation of axonogenesis	BP	1	SEMA3A,RNF6	up_VPOR_EC
GO:0002021	0.0351	0.999	1	9	response to dietary excess	BP	1	PCSK1N	up_APUL_EC
GO:0050806	0.0351	0.999	1	25	positive regulation of synaptic transmission	BP	1	CA2	up_VCAV_VSMC
GO:0098657	0.0352	0.979	25	181	import into cell	BP	1	DNAJC6,PLCG2,LEP,CDC7,HTR2B,ABCA1,ATXN2,SLC2A5,TMEM108,F2RL1,LRPAP1,APP,PSEN1,SYNJ2BP,PIK3C3,CLN8,GTF2A1L,B2M,AP2B1,SCAMP5,TXNDC5,RAB5C,PARK7,SLC8A1,PIK3C2A	up_ACOR_VSMC

GO:0032091	0.0352	0.989	7	34	negative regulation of protein binding	BP	1	XIRP1,CLSTN1,LRPAP1,MITD1,B2M,DISC1,PARK7	up_ACOR_VSMC
GO:1901700	0.0352	0.993	4	364	response to oxygen-containing compound	BP	1	NR1H4,MRC1,PTGER2,ARHGEF2	up_VPUL_EC
GO:1901362	0.0352	1	2	1293	organic cyclic compound biosynthetic process	BP	1	JUP,GATA5	down_VFEM_VSMC
GO:0001916	0.0352	0.998	2	6	positive regulation of T cell mediated cytotoxicity	BP	0.856	FAM49B,PVR	down_VSAP_EC
GO:0043393	0.0352	0.997	2	77	regulation of protein binding	BP	1	HOXA3,PLCL1	up_APUL_EC
GO:0002418	0.0352	0.998	2	5	immune response to tumor cell	BP	0.771	NECTIN2,HMGB1	up_VCAV_EC
GO:0002834	0.0352	0.998	2	5	regulation of response to tumor cell	BP	0.771	NECTIN2,HMGB1	up_VCAV_EC
GO:0002837	0.0352	0.998	2	5	regulation of immune response to tumor cell	BP	0.771	NECTIN2,HMGB1	up_VCAV_EC
GO:0032352	0.0352	0.998	2	4	positive regulation of hormone metabolic	BP	0.771	GATA3,HIF1A	up_VCAV_EC
GO:0072007	0.0352	0.998	2	5	mesangial cell differentiation	BP	0.771	PDGFB,GPR4	up_VCAV_EC
GO:0072143	0.0352	0.998	2	5	mesangial cell development	BP	0.771	PDGFB,GPR4	up_VCAV_EC
GO:1904238	0.0352	0.998	2	5	pericyte cell differentiation	BP	0.771	PDGFB,GPR4	up_VCAV_EC
GO:0021537	0.0352	0.997	2	73	telencephalon development	BP	1	EMX2,SALL1	up_VSAP_VSMC
GO:0001707	0.0352	0.999	1	25	mesoderm formation	BP	1	EYA1	up_AFEM_VSMC
GO:0031099	0.0352	0.999	1	30	regeneration	BP	1	WNT7A	up_AORT_VSMC
GO:0006880	0.0352	1	1	4	intracellular sequestering of iron ion	BP	1	ENSCAFG00000005494	up_VPOR_EC
GO:0097577	0.0352	1	1	4	sequestering of iron ion	BP	1	ENSCAFG00000005494	up_VPOR_EC
GO:2000826	0.0352	0.999	1	14	regulation of heart morphogenesis	BP	1	EYA1	up_VFEM_VSMC
GO:0002068	0.0353	0.996	3	8	glandular epithelial cell development	BP	1	HNF4A,DLL1,RARG	up_VSAP_EC
GO:1901857	0.0353	0.998	2	4	positive regulation of cellular respiration	BP	1	PRELID1,CDK1	down_ACOR_VSMC
GO:0030279	0.0353	0.999	1	19	negative regulation of ossification	BP	1	HDAC7	down_VPOR_EC
GO:2000738	0.0353	0.999	1	9	positive regulation of stem cell differentiation	BP	1	TBX5	up_APUL_EC
GO:0006805	0.0353	0.999	1	8	xenobiotic metabolic process	BP	1	CYP26B1	up_VFEM_EC
GO:0060606	0.0354	0.987	8	39	tube closure	BP	1	SHROOM3,BMP4,SEC24B,CITED2,SPINT1,LMO4,SPINT2,FZD3	up_ACOR_VSMC
GO:0060021	0.0354	0.988	7	34	roof of mouth development	BP	1	FZD1,WNT11,OSR2,INSIG2,OSR1,TGFBR2,NPRL3	up_VSAP_EC
GO:0045648	0.0354	0.995	3	13	positive regulation of erythrocyte	BP	0.857	HIF1A,PRMT1,INHBA	down_VSAP_EC
GO:0048254	0.0354	0.998	2	4	snoRNA localization	BP	1	FBL,NOP58	down_ACOR_VSMC
GO:0002521	0.0354	0.997	2	149	leukocyte differentiation	BP	1	TMEM176A,TMEM176B	up_VPUL_VSMC
GO:0001783	0.0354	0.999	1	8	B cell apoptotic process	BP	1	LYN	down_ACOR_EC
GO:0070613	0.0354	0.999	1	25	regulation of protein processing	BP	1	C6	up_VCAV_VSMC
GO:1903317	0.0354	0.999	1	25	regulation of protein maturation	BP	1	C6	up_VCAV_VSMC
GO:0048675	0.0354	0.999	1	26	axon extension	BP	1	LIMK1	up_VCAV_VSMC
GO:0047484	0.0354	1	1	5	regulation of response to osmotic stress	BP	1	ARHGEF2	up_VPOR_EC
GO:0045992	0.0355	0.995	3	12	negative regulation of embryonic development	BP	0.857	SULF1,PRKACB,CTNNB1	down_VSAP_EC
GO:0050858	0.0355	0.997	2	6	negative regulation of antigen receptor-mediated signaling	BP	1	PRNP,THY1	down_VCAV_EC
GO:0050860	0.0355	0.997	2	6	negative regulation of T cell receptor signaling	BP	1	PRNP,THY1	down_VCAV_EC
GO:0007028	0.0355	0.998	2	5	cytoplasm organization	BP	0.857	ETV6,KIF5B	down_VSAP_EC
GO:0001775	0.0355	0.997	2	219	cell activation	BP	1	TLR2,HOXA9	up_AFEM_VSMC
GO:0051960	0.0355	0.997	2	216	regulation of nervous system development	BP	1	SLITRK5,LIMK1	up_VCAV_VSMC

GO:0051961	0.0356	0.986	9	73	negative regulation of nervous system	BP	0.857	PRKACB,DPYSL3,HOOK3,IFRD1,CTNNB1,APOE,REST,NRP1,YWHAH	down_VSAP_EC
GO:0009891	0.0356	0.993	4	521	positive regulation of biosynthetic process	BP	1	EYA1,HOXA9,HOXA11,SHOX2	up_VFEM_VSMC
GO:0048468	0.0356	0.995	3	553	cell development	BP	1	EYA1,BEND2,IRX5	up_AFEM_VSMC
GO:0060412	0.0356	0.999	1	15	ventricular septum morphogenesis	BP	1	NKX2-5	up_VPOR_VSMC
GO:0002115	0.0356	0.999	1	8	store-operated calcium entry	BP	1	STIM1	up_VSAP_VSMC
GO:0048729	0.0357	0.978	29	209	tissue morphogenesis	BP	1	TNNT2,GATA5,SHROOM3,MLLT3,BMP4,FGFR2,GJA1,SEC24B,CITED2,SPINT1,ZNRF3,LMO4,ANKRD1,EGLN1,ENSCAFG00000006238,CLSTN1,NRP1,NPNT,SPINT2,TBX20,TBX5,FZD3,FRS2,DAG1,PSEN1,CHD7,EDN1,SERPINB5,RREB1	up_ACOR_VSMC
GO:0006935	0.0357	0.994	3	143	chemotaxis	BP	1	LYN,NRP2,STX4	down_ACOR_EC
GO:0042330	0.0357	0.994	3	143	taxis	BP	1	LYN,NRP2,STX4	down_ACOR_EC
GO:0035239	0.0357	0.995	3	299	tube morphogenesis	BP	1	EYA1,HOXA11,SHOX2	up_VFEM_VSMC
GO:0080009	0.0357	0.998	2	4	mRNA methylation	BP	0.779	WTAP,ZC3H13	up_VCAV_EC
GO:0048514	0.0357	0.997	2	214	blood vessel morphogenesis	BP	1	C6,TGFBI	up_VCAV_VSMC
GO:0045747	0.0357	0.999	1	18	positive regulation of Notch signaling pathway	BP	1	GATA5	up_VPUL_VSMC
GO:0032272	0.0357	0.999	1	18	negative regulation of protein polymerization	BP	1	SLIT2	up_VPUL_VSMC
GO:0031667	0.0358	0.982	16	102	response to nutrient levels	BP	1	LEP,PRKAA2,CASTOR1,PPARGC1A,CDKN2B,GABARAPL1,CDKN1A,P2RX7,ULK2,SESN3,PIK3C3,LAMP2,SESN1,FAM107A,BMT2,ZFYVE1	up_ACOR_VSMC
GO:0006338	0.0358	0.987	8	45	chromatin remodeling	BP	1	HMGA1,SMARCC1,OIP5,DMAP1,SMARCB1,CENPP,TADA2A,MIS18A	down_ACOR_VSMC
GO:0007266	0.0358	0.987	8	64	Rho protein signal transduction	BP	0.861	ARHGEF2,FARP1,APOE,PLEKHG2,TAX1BP3,PAK2,NRP1,TNFAIP1	down_VSAP_EC
GO:0099537	0.0358	0.988	7	131	trans-synaptic signaling	BP	1	KCNC4,GPR1,CHRM2,SLC6A6,HTR1D,CAMK2A,DMPK	up_AFEM_EC
GO:0018193	0.0358	0.992	4	368	peptidyl-amino acid modification	BP	1	NR1H4,STK32B,PTGER2,ARHGEF2	up_VPUL_EC
GO:0007160	0.0358	0.997	2	56	cell-matrix adhesion	BP	1	ACVRL1,HOXA7	down_ACOR_EC
GO:0046601	0.0358	0.998	2	4	positive regulation of centriole replication	BP	1	NUP62,PLK4	down_ACOR_VSMC
GO:0006335	0.0358	0.998	2	4	DNA replication-dependent nucleosome	BP	0.779	NASP,ASF1A	up_VCAV_EC
GO:0034723	0.0358	0.998	2	4	DNA replication-dependent nucleosome	BP	0.779	NASP,ASF1A	up_VCAV_EC
GO:0002707	0.0358	0.998	2	4	negative regulation of lymphocyte mediated	BP	0.779	IL7R,THOC1	up_VCAV_EC
GO:0072529	0.0358	0.998	2	4	pyrimidine-containing compound catabolic	BP	0.779	CDA,TET2	up_VCAV_EC
GO:0050435	0.0358	0.999	1	8	amyloid-beta metabolic process	BP	1	MME	up_VFEM_EC
GO:0009612	0.035867914	0.99208	4	47	response to mechanical stimulus	BP	1	SOX9,AQP1,PTGER4,USP53	up_ACOR_EC
GO:0006725	0.0359	0.99	8	1956	cellular aromatic compound metabolic	BP	1	IVNS1ABP,EXTL2,DARS,SUCLG2,SRSF6,GABPA,NDUFA10,TSEN15	down_AFEM_EC
GO:0006986	0.0359	0.987	8	42	response to unfolded protein	BP	1	TM7SF3,CREBRF,STT3B,JKAMP,HERPUD1,WFS1,DZIP3,STC2	up_ACOR_VSMC
GO:0007032	0.0359	0.991	5	22	endosome organization	BP	0.779	RAB11A,RAB27A,HOOK3,DNAJC13,ALS2	up_VCAV_EC
GO:0006468	0.0359	0.991	5	544	protein phosphorylation	BP	1	STK32B,CCL24,PTGER2,FLRT3,ARHGEF2	up_VPUL_EC
GO:0022603	0.0359	0.992	4	301	regulation of anatomical structure morphogenesis	BP	1	EYA1,HOXB7,SIX1,CDC42EP5	up_VFEM_EC
GO:0055007	0.0359	0.994	3	31	cardiac muscle cell differentiation	BP	1	DLL1,GREM1,BVES	up_AFEM_EC
GO:0022603	0.0359	0.994	3	301	regulation of anatomical structure morphogenesis	BP	1	EYA1,HOXA11,SHOX2	up_VFEM_VSMC
GO:0045214	0.0359	0.999	1	13	sarcomere organization	BP	1	NKX2-5	up_VPOR_VSMC
GO:0001666	0.036	0.985	10	67	response to hypoxia	BP	0.779	CXCR4,CD34,EDN1,MTOR,BMP2,PTGS2,PMAIP1,HIF1A,HP1BP3,P2RX2	up_VCAV_EC
GO:0036293	0.036	0.985	10	67	response to decreased oxygen levels	BP	0.779	CXCR4,CD34,EDN1,MTOR,BMP2,PTGS2,PMAIP1,HIF1A,HP1BP3,P2RX2	up_VCAV_EC
GO:0072359	0.036	0.992	4	368	circulatory system development	BP	1	TBX5,HOXA3,CCL24,FLRT3	up_VPUL_EC

GO:0120162	0.036	0.994	3	31	positive regulation of cold-induced thermogenesis	BP	1	CMKLR1,LEP,PPARGC1A	up_AFEM_EC
GO:0033003	0.036	0.999	1	8	regulation of mast cell activation	BP	1	LYN	down_ACOR_EC
GO:0045604	0.036	0.999	1	17	regulation of epidermal cell differentiation	BP	1	SRSF6	down_AFEM_EC
GO:0050905	0.036	0.999	1	26	neuromuscular process	BP	1	HOXC10	up_AFEM_VSMC
GO:0007269	0.036	0.999	1	34	neurotransmitter secretion	BP	1	WNT7A	up_AORT_VSMC
GO:0099643	0.036	0.999	1	34	signal release from synapse	BP	1	WNT7A	up_AORT_VSMC
GO:0030326	0.036	0.999	1	51	embryonic limb morphogenesis	BP	1	TBX5	up_APUL_VSMC
GO:0035113	0.036	0.999	1	51	embryonic appendage morphogenesis	BP	1	TBX5	up_APUL_VSMC
GO:0009187	0.036	0.999	1	10	cyclic nucleotide metabolic process	BP	1	ADCY3	up_VFEM_EC
GO:0050771	0.036089422	0.99701	2	11	negative regulation of axonogenesis	BP	1	LRP4,NRP1	up_ACOR_EC
GO:0051495	0.0361	0.984	12	82	positive regulation of cytoskeleton organization	BP	0.78	TEK,ARHGEF15,BAIAP2L1,EDN1,MTOR,NCKAP1,PROX1,IQGAP2,SWAP70,NCK1,FES,ARPC5L	up_VCAV_EC
GO:0007154	0.0361	0.987	10	1622	cell communication	BP	1	TBX5,NR1H4,RGS1,STK32B,CCL24,HTR7,PTGER2,FLRT3,ARHGEF2,NEO1	up_VPUL_EC
GO:0061326	0.0361	0.989	6	41	renal tubule development	BP	0.865	ENSCAFG00000032470,ILK,HOXB7,MAGED1,CTNNB1,SIX4	down_VSAP_EC
GO:0072080	0.0361	0.989	6	41	nephron tubule development	BP	0.865	ENSCAFG00000032470,ILK,HOXB7,MAGED1,CTNNB1,SIX4	down_VSAP_EC
GO:0032501	0.0361	0.994	5	1782	multicellular organismal process	BP	1	HOXC10,HOXA9,WNT7A,ARG2,ESR1	up_AORT_VSMC
GO:0051653	0.0361	0.993	4	15	spindle localization	BP	1	ZW10,CENPA,NDC80,ASPM	down_ACOR_VSMC
GO:0048592	0.0361	0.992	4	53	eye morphogenesis	BP	1	IRX5,COL8A2,DLL1,THRB	up_AFEM_EC
GO:2000401	0.0361	0.997	2	13	regulation of lymphocyte migration	BP	1	CCL5,ADAM17	up_AFEM_EC
GO:0001976	0.0361	0.998	2	4	neurological system process involved in regulation of systemic	BP	0.78	NAV2,P2RX2	up_VCAV_EC
GO:1902904	0.0361	0.997	2	38	negative regulation of supramolecular fiber	BP	1	ARHGEF2,TWF1	up_VPOR_EC
GO:0046847	0.0361	0.999	1	17	filopodium assembly	BP	1	FSCN1	down_AFEM_EC
GO:0048332	0.0361	0.999	1	26	mesoderm morphogenesis	BP	1	EYA1	up_AFEM_VSMC
GO:0034502	0.0361	0.999	1	30	protein localization to chromosome	BP	1	ESR1	up_AORT_VSMC
GO:0014896	0.0361	0.999	1	18	muscle hypertrophy	BP	1	GATA5	up_VPUL_VSMC
GO:0014897	0.0361	0.999	1	18	striated muscle hypertrophy	BP	1	GATA5	up_VPUL_VSMC
GO:0003300	0.0361	0.999	1	18	cardiac muscle hypertrophy	BP	1	GATA5	up_VPUL_VSMC
GO:0060038	0.0361	0.999	1	18	cardiac muscle cell proliferation	BP	1	TBX5	up_VPUL_VSMC
GO:0000187	0.0362	0.989	6	28	activation of MAPK activity	BP	0.78	KIT,MAP3K11,TLR4,BMP2,MADD,RIPK1	up_VCAV_EC
GO:0006029	0.0362	0.991	5	29	proteoglycan metabolic process	BP	0.866	SULF1,ENSCAFG00000032470,CHSY1,CTNNB1,EXT1	down_VSAP_EC
GO:0006493	0.0362	0.991	5	20	protein O-linked glycosylation	BP	1	GALNT3,GALNT10,B3GALNT2,POMK,EOGT	up_ACOR_VSMC
GO:1903428	0.0362	0.995	3	8	positive regulation of reactive oxygen species biosynthetic process	BP	1	CYBA,ZNF205,ASS1	up_VSAP_EC
GO:0045739	0.0362	0.999	1	26	positive regulation of DNA repair	BP	1	EYA1	up_AFEM_VSMC
GO:0006766	0.0362	0.999	1	16	vitamin metabolic process	BP	1	CYP26B1	up_VPOR_VSMC
GO:0003209	0.0362	0.999	1	14	cardiac atrium morphogenesis	BP	1	SHOX2	up_VFEM_VSMC
GO:0003230	0.0362	0.999	1	14	cardiac atrium development	BP	1	SHOX2	up_VFEM_VSMC
GO:0060420	0.0362	0.999	1	18	regulation of heart growth	BP	1	TBX5	up_VPUL_VSMC
GO:0043066	0.036219561	0.9833	12	253	negative regulation of apoptotic process	BP	1	NR1H4,SOX9,AQP1,IFI6,SMAD6,BCL2,MAPK8IP2,FOXC2,FIGNL1,CLU,NRP1,PRKCD	up_ACOR_EC
GO:0071902	0.0363	0.984	12	79	positive regulation of protein serine/threonine	BP	0.781	KIT,MAP3K11,PDGFB,TLR4,EDN1,RALB,BMP2,PROX1,MADD,RAPGEF2,RIPK1,PDGFD	up_VCAV_EC

GO:0016202	0.0363	0.988	7	39	regulation of striated muscle tissue	BP	0.78	EPHB1,MEF2C,EDN1,BMP2,CREB1,NR1D2,CDON	up_VCAV_EC
GO:0010646	0.0363	0.989	7	945	regulation of cell communication	BP	1	NR1H4,RGS1,CCL24,PTGER2,FLRT3,ARHGEF2,NEO1	up_VPUL_EC
GO:0009889	0.0363	0.994	4	1190	regulation of biosynthetic process	BP	1	HOXA9,WNT7A,ARG2,ESR1	up_AORT_VSMC
GO:0007507	0.0363	0.994	3	194	heart development	BP	1	TBX5,ZDHHC16,PDLIM3	up_APUL_EC
GO:0002903	0.0363	0.998	2	4	negative regulation of B cell apoptotic process	BP	1	AURKB,NOC2L	down_ACOR_VSMC
GO:0071495	0.0363	0.997	2	365	cellular response to endogenous stimulus	BP	1	GATA5,TGFBR1	down_VSAP_VSMC
GO:0055119	0.0363	0.998	2	4	relaxation of cardiac muscle	BP	0.78	PDE4D,KCNJ2	up_VCAV_EC
GO:0045089	0.0363	0.997	2	70	positive regulation of innate immune response	BP	1	TLR2,CAV1	up_VSAP_VSMC
GO:0002367	0.0363	0.999	1	26	cytokine production involved in immune	BP	1	TLR2	up_AFEM_VSMC
GO:0009798	0.0363	0.999	1	31	axis specification	BP	1	WNT7A	up_AORT_VSMC
GO:0021515	0.0363	0.999	1	9	cell differentiation in spinal cord	BP	1	HOXC10	up_VFEM_EC
GO:0044257	0.0364	0.978	28	327	cellular protein catabolic process	BP	0.868	PRICKLE1,PLK3,HSPA5,SOCS5,HM13,HSP90B1,TRIB3,MSN,SEL1L,USP12,DNAJC10,UBA6,UCHL5,SPOPL,DNAJB9,APOE,USP48,BNIP3,DDIT3,CBLC,GSK3B,CUL2,SPOP,DISC1,RAD23A,TMTC3,USP14,TNFAIP1	down_VSAP_EC
GO:0034764	0.0364	0.989	6	50	positive regulation of transmembrane transport	BP	1	FXYD1,CLIP3,ENSCAFG00000014978,AKT2,THY1,PARK7	down_VCAV_EC
GO:0051240	0.0364	0.991	5	489	positive regulation of multicellular organismal	BP	1	NR1H4,TBX5,SLITRK5,NPNT,NTRK2	up_APUL_EC
GO:1902692	0.0364	0.995	3	11	regulation of neuroblast proliferation	BP	0.868	HIF1A,CTNNB1,DISC1	down_VSAP_EC
GO:0061550	0.0364	0.999	1	6	cranial ganglion development	BP	1	NRP2	down_ACOR_EC
GO:0035265	0.0364	0.999	1	54	organ growth	BP	1	TBX5	up_APUL_VSMC
GO:0086091	0.0364	0.999	1	8	regulation of heart rate by cardiac conduction	BP	1	CAV1	up_VSAP_VSMC
GO:0010647	0.0365	0.976	37	460	positive regulation of cell communication	BP	0.868	VEGFA,SULF1,NR1H4,JCAD,PTPN11,ILK,SMARCA4,MYO1C,CHSY1,KIF5B,HIF1A,TNIK,PTPN2,NEO1,MTDH,ESM1,ADAM17,MAGED1,CTNNB1,ENSCAFG00000002015,APOE,LYN,BNIP3,FAM162A,ATF3,SH3GLB1,HTT,CALM1,DISC1,PAK2,LRRTM2,INHBA,NRP1,SH3RF3,MADD,SEC13,MID1	down_VSAP_EC
GO:1901615	0.0365	0.98	19	128	organic hydroxy compound metabolic process	BP	1	NAPEPLD,LIPC,INSIG2,ACAA1,SULT1A1,GOT1,INPP5E,LMF1,PPIP5K1,GALK1,RETSAT,LCAT,PCBD1,TKFC,MVK,LIPE,PNPO,CYB5R3,SOAT1	up_VSAP_EC
GO:0051179	0.0365	0.984	14	1747	localization	BP	1	ACVRL1,LYN,HOXA3,IGF1R,SPG11,PDPN,NRP2,HOXA7,IPO7,SULF1,ACSL3,STX4,MYO1D,NEDD4	down_ACOR_EC
GO:0051650	0.0365	0.986	8	65	establishment of vesicle localization	BP	0.868	MYH9,SHROOM2,MYO1C,KIF5B,CTNNB1,GSK3B,HTT,AP3S1	down_VSAP_EC
GO:0065003	0.0365	0.993	4	616	protein-containing complex assembly	BP	1	EIF3M,SRSF6,NDUFA10,FSCN1	down_AFEM_EC
GO:0042592	0.0365	0.993	4	522	homeostatic process	BP	1	HOXA3,TRPC4,CYP26B1,C3AR1	up_VPOR_VSMC
GO:0033120	0.0365	0.995	3	9	positive regulation of RNA splicing	BP	1	U2AF2,THRAP3,SF3B4	down_ACOR_VSMC
GO:0060413	0.0365	0.995	3	8	atrial septum morphogenesis	BP	1	GATA4,TBX20,TBX5	up_ACOR_VSMC
GO:0003283	0.0365	0.995	3	8	atrial septum development	BP	1	GATA4,TBX20,TBX5	up_ACOR_VSMC
GO:0045597	0.0365	1	1	253	positive regulation of cell differentiation	BP	1	GATA5	down_AFEM_VSMC
GO:0006913	0.0365	1	1	124	nucleocytoplasmic transport	BP	1	JUP	down_VFEM_VSMC
GO:0051169	0.0365	1	1	124	nuclear transport	BP	1	JUP	down_VFEM_VSMC
GO:0042472	0.0365	0.999	1	27	inner ear morphogenesis	BP	1	EYA1	up_AFEM_VSMC
GO:0003148	0.0365	0.999	1	13	outflow tract septum morphogenesis	BP	1	NKX2-5	up_VPOR_VSMC
GO:0097120	0.0365	0.999	1	9	receptor localization to synapse	BP	1	NETO1	up_VSAP_VSMC
GO:0032271	0.0366	0.984	11	73	regulation of protein polymerization	BP	0.784	HCLS1,BAIAP2L1,TMOD1,MTOR,NCKAP1,IQGAP2,STMN1,NCK1,FES,ARPC5L,TMOD3	up_VCAV_EC

GO:0010595	0.0366	0.988	7	52	positive regulation of endothelial cell migration	BP	0.868	VEGFA,SPARC,JCAD,PIK3C2A,HIF1A,ADAM17,NRP1	down_VSAP_EC
GO:1904589	0.0366	0.988	7	33	regulation of protein import	BP	1	LEP,FERMT1,BMP4,CDKN1A,PTPN22,PSEN1,JUP	up_ACOR_VSMC
GO:0030282	0.0366	0.99	5	37	bone mineralization	BP	1	BMPR1A,ASPN,DDR2,OMD,GREM1	down_VCAV_EC
GO:0070887	0.0366	0.991	5	775	cellular response to chemical stimulus	BP	1	FGF5,NKX2-5,BAMBI,CYP26B1,C3AR1	up_VPOR_VSMC
GO:0044818	0.0366	0.993	4	15	mitotic G2/M transition checkpoint	BP	1	CDK1,FANCI,TOPBP1,CLSPN	down_ACOR_VSMC
GO:0043207	0.0366	0.994	3	216	response to external biotic stimulus	BP	1	NR1H4,RGS1,MRC1	up_VPUL_EC
GO:0051707	0.0366	0.994	3	216	response to other organism	BP	1	NR1H4,RGS1,MRC1	up_VPUL_EC
GO:0002683	0.0366	0.997	2	126	negative regulation of immune system process	BP	1	HOXA9,BPI	up_AORT_EC
GO:0060059	0.0366	0.998	2	3	embryonic retina morphogenesis in camera-	BP	0.784	PROX1,CDON	up_VCAV_EC
GO:0061549	0.0366	0.999	1	5	sympathetic ganglion development	BP	1	SEMA3A	up_VPOR_EC
GO:0030593	0.0366	0.999	1	19	neutrophil chemotaxis	BP	1	SLIT2	up_VPUL_VSMC
GO:0030154	0.0367	0.99	6	1040	cell differentiation	BP	1	CHL1,BARX1,NKX2-5,BAMBI,TRPC4,CYP26B1	up_VPOR_VSMC
GO:0010628	0.0367	0.992	4	527	positive regulation of gene expression	BP	1	EYA1,HOXA9,HOXA11,SHOX2	up_VFEM_VSMC
GO:0010543	0.0367	0.995	3	10	regulation of platelet activation	BP	0.784	SELP,PDGFB,TLR4	up_VCAV_EC
GO:0032989	0.0367	0.994	3	292	cellular component morphogenesis	BP	1	CHL1,NKX2-5,BAMBI	up_VPOR_VSMC
GO:0060982	0.0367	0.998	2	3	coronary artery morphogenesis	BP	1	SEC24B,NRP1	up_ACOR_VSMC
GO:0042127	0.0367	0.998	2	452	regulation of cell proliferation	BP	1	HOXA3,TBX5	up_APUL_VSMC
GO:0071636	0.0367	0.998	2	5	positive regulation of transforming growth factor beta production	BP	0.784	CD34,CD200	up_VCAV_EC
GO:0002011	0.0367	0.999	1	28	morphogenesis of an epithelial sheet	BP	1	WNT7A	up_AORT_VSMC
GO:0045638	0.0367	0.999	1	33	negative regulation of myeloid cell	BP	1	HOXA9	up_AORT_VSMC
GO:0051963	0.0367	0.999	1	26	regulation of synapse assembly	BP	1	SLITRK5	up_VCAV_VSMC
GO:0097435	0.0368	0.978	28	230	supramolecular fiber organization	BP	0.784	HCLS1,ARHGEF15,BAIAP2L1,MEF2C,EDN1,RAB11A,ARHGAP28,TMOD1,MTOR,HOOK3,SLK,NCKAP1,MZT1,PROX1,IQGAP2,STMN1,SWAP70,NCK1,FES,TAOK1,CTNNA1,ARPC5L,RIPK1,TNFAIP1,TMOD3,ARHGEF2,FSCN1,FBXO5	up_VCAV_EC
GO:0031272	0.0368	0.995	3	8	regulation of pseudopodium assembly	BP	1	CDC42EP5,CDC42EP2,F2RL1	up_VSAP_EC
GO:0031274	0.0368	0.995	3	8	positive regulation of pseudopodium assembly	BP	1	CDC42EP5,CDC42EP2,F2RL1	up_VSAP_EC
GO:0043489	0.0368	0.997	2	13	RNA stabilization	BP	1	TENT5C,AXIN2	up_AFEM_EC
GO:0060428	0.0368	0.999	1	17	lung epithelium development	BP	1	SRSF6	down_AFEM_EC
GO:0002224	0.0368	0.999	1	34	toll-like receptor signaling pathway	BP	1	ESR1	up_AORT_VSMC
GO:0015893	0.0368	0.999	1	26	drug transport	BP	1	CA2	up_VCAV_VSMC
GO:0030808	0.0368	0.999	1	15	regulation of nucleotide biosynthetic process	BP	1	FBP1	up_VFEM_VSMC
GO:1900371	0.0368	0.999	1	15	regulation of purine nucleotide biosynthetic	BP	1	FBP1	up_VFEM_VSMC
GO:0070372	0.0369	0.983	12	85	regulation of ERK1 and ERK2 cascade	BP	0.784	EPHB1,TEK,TNIP1,PDGFB,ATF3,RAPGEF1,TLR4,BMP2,PRKD2,RAPGEF2,HMGB1,PDGFD	up_VCAV_EC
GO:0010977	0.0369	0.988	7	33	negative regulation of neuron projection	BP	1	ITM2C,ZNF365,NRP1,ULK2,PSEN1,ASAP1,B2M	up_ACOR_VSMC
GO:0002763	0.0369	0.992	4	17	positive regulation of myeloid leukocyte	BP	0.784	HCLS1,CASP8,CREB1,RIPK1	up_VCAV_EC
GO:0009888	0.0369	0.995	3	545	tissue development	BP	1	GATA5,CA2,LRRC17	up_VCAV_VSMC
GO:0045351	0.0369	0.998	2	4	type I interferon biosynthetic process	BP	0.784	TLR4,TLR3	up_VCAV_EC
GO:0010831	0.0369	0.999	1	9	positive regulation of myotube differentiation	BP	1	CYP26B1	up_VFEM_EC
GO:1905276	0.0369	0.999	1	9	regulation of epithelial tube formation	BP	1	SIX1	up_VFEM_EC

GO:0050709	0.036983733	0.99404	3	28	negative regulation of protein secretion	BP	1	NR1H4,KCNB1,PTGER4	up_ACOR_EC
GO:0048514	0.037	0.98	17	214	blood vessel morphogenesis	BP	1	HSPB6,PDGFRB,DCN,ANGPT1,BMPR1A,LOX,ANPEP,THY1,B4GALT1,ZFP36L1,MYDGF,SASH1,CDH2,SPRED1,SHC1,GREM1,CLIC4	down_VCAV_EC
GO:0030900	0.037	0.982	15	101	forebrain development	BP	0.786	PTCHD1,RELN,CHD7,HOOK3,BMP2,HHEX,GDPD5,PROX1,ENSCAFG00000010224,RAPGEF2,CREB1,HIF1A,ATRX,ALDH1A3,CDON	up_VCAV_EC
GO:0048646	0.037	0.982	14	349	anatomical structure formation involved in morphogenesis	BP	1	IRX3,LEP,ANGPT4,HOXA7,DLL1,F3,TMEM100,GREM1,LUZP1,TBXA2R,CNNM4,PTPRB,AXIN2,CLIC4	up_AFEM_EC
GO:0030072	0.037	0.986	9	48	peptide hormone secretion	BP	1	HNF4A,SLC8B1,RAB11B,PASK,KCNB1,RAF1,STX4,PPP3CB,RAB11FIP2	up_VSAP_EC
GO:0008211	0.037	0.998	2	5	glucocorticoid metabolic process	BP	0.871	REST,YWHAH	down_VSAP_EC
GO:0007193	0.037	0.997	2	13	adenylate cyclase-inhibiting G protein-coupled receptor signaling	BP	1	CHRM2,HTR1D	up_AFEM_EC
GO:0050804	0.037	1	1	87	modulation of chemical synaptic transmission	BP	1	PLCL1	down_VFEM_EC
GO:0099177	0.037	1	1	87	regulation of trans-synaptic signaling	BP	1	PLCL1	down_VFEM_EC
GO:1900003	0.037	1	1	1	regulation of serine-type endopeptidase activity	BP	0.871	SPINK2	down_VSAP_EC
GO:1900004	0.037	1	1	1	negative regulation of serine-type	BP	0.871	SPINK2	down_VSAP_EC
GO:1902571	0.037	1	1	1	regulation of serine-type peptidase activity	BP	0.871	SPINK2	down_VSAP_EC
GO:1902572	0.037	1	1	1	negative regulation of serine-type peptidase	BP	0.871	SPINK2	down_VSAP_EC
GO:0045446	0.037	0.999	1	32	endothelial cell differentiation	BP	1	WNT7A	up_AORT_VSMC
GO:0007413	0.037	0.999	1	5	axonal fasciculation	BP	1	SEMA3A	up_VPOR_EC
GO:0106030	0.037	0.999	1	5	neuron projection fasciculation	BP	1	SEMA3A	up_VPOR_EC
GO:0006111	0.037	0.999	1	15	regulation of gluconeogenesis	BP	1	FBP1	up_VFEM_VSMC
GO:0007632	0.037	0.999	1	14	visual behavior	BP	1	NETO1	up_VFEM_VSMC
GO:0043627	0.037	0.999	1	8	response to estrogen	BP	1	CAV1	up_VSAP_VSMC
GO:0043412	0.0371	0.972	88	1225	macromolecule modification	BP	0.871	ARHGEF2,PRICKLE1,VEGFA,P4HB,PLOD2,NR1H4,HSPA5,SOCS5,SGK1,ERO1A,ENPP1,ERRFI1,P4HA1,JCAD,SNX25,WSB1,BCAR3,NUAK1,PTPN11,AARS,TRIB3,PUM3,ILK,PPP1R12A,SESN2,PTGS2,PPIB,USP12,MRPL1,IWS1,DNAJC10,TNIK,GALNT1,NAA10,NXN,PTPN2,UCHL5,MORF4L2,SPOPL,YES1,PLCL1,TTLL7,ADAM17,CNOT7,MAGED1,CTNNB1,CDK8,APOE,PLOD3,USP48,UBA5,LYN,STRAP,BACH1,ASPH,CCNYL1,STT3A,MMD,CBLC,ATF3,GSK3B,VLDLR,GTF2H1,CUL2,HTT,REST,ZMYND8,MAPK10,EIF2S1,PRMT1,P3H4,EXT1,CALM1,CDYL,PAK2,INHBA,CAMK2D,HIF1AN,BTK,CDKN1A,NRP1,SH3RF3,USP14,ARF4,MADD,RPN1,KDM3A,MID1	down_VSAP_EC
GO:0016125	0.0371	0.987	8	41	sterol metabolic process	BP	1	LIPC,INSIG2,LMF1,LCAT,MVK,LIPE,CYB5R3,SOAT1	up_VSAP_EC
GO:0051252	0.0371	0.99	6	1051	regulation of RNA metabolic process	BP	1	EYA1,HOXA10,HOXA9,EMX2,HOXA11,SHOX2	up_VFEM_VSMC
GO:0050920	0.0371	0.992	4	53	regulation of chemotaxis	BP	1	CMKLR1,PLXNA4,CCL5,ADAM17	up_AFEM_EC
GO:0045721	0.0371	0.998	2	4	negative regulation of gluconeogenesis	BP	0.786	FAM3C,ENSCAFG00000010596	up_VCAV_EC
GO:0010660	0.0371	0.999	1	14	regulation of muscle cell apoptotic process	BP	1	NKX2-5	up_VPOR_VSMC
GO:0002053	0.0371	0.999	1	15	positive regulation of mesenchymal cell	BP	1	SHOX2	up_VFEM_VSMC
GO:0006644	0.037172504	0.98702	7	118	phospholipid metabolic process	BP	1	NR1H4,ENSCAFG00000004331,CFI,CHPT1,PIGS,AGPAT2,PRKCD	up_ACOR_EC

GO:1901576	0.0372	0.97	176	1877	organic substance biosynthetic process	BP	1	ELK3,SCD,ACSL3,PRPSAP1,SQLE,HMGB1,MCM6,LSS,SREBF1,ACLY,MCM4,IRAK1,MCM7,RPA1,ILF3,CBX5,PPRC1,BCAT1,ATAD2,HMGA1,EIF4A3,HMGCR,HNRNPD,SREBF2,MCM3,SMC1A,HINFP,VARS,NEDD4,RRM1,MED17,POLA1,SLC29A1,MTDH,HEY1,RRBP1,PPP1R15B,SF1,POLA2,MPDU1,SRRT,GINS4,CTDNEP1,POLE,NAB2,SLC35C2,MRPS18B,B3GNT2,CDCA7L,FUS,RFC2,BIRC5,MYBBP1A,INSIG1,PRMT1,CAV1,RFC5,RFC3,RAD51,XYLT1,TIMELESS,THRAP3,TRUB2,ENSCAFG00000018572,FAM129B,TCEA1,PFAS,AURKB,NONO,TK1,TOPBP1,EZH2,GREM1,RFC4,NEK2,FEN1,CDK4,SMARCC1,PLK3,PCNA,DHX33,HOXA7,TONSL,ARG2,MCM10,UHRF1,CKS2,TKT,GMNN,DHCR24,BRCA1,MVD,FASTKD3,NOC2L,EIF5A,E2F8,B4GALT1,ELOVL1,FARSA,PPARD,CDC6,POLH,YBX3,NCL,NUP62,UGDH,MRPL37,DTL,VAC14,FOSL1,SUV39H1,ZNF367,CACTIN,HMGB2,DMAP1,SMC3,CDK2,SMARCB1,DDX39B,CCNA2,ENSCAFG00000005299,EZR,ELOVL6,FDFT1,RBM14,CYC1,NOLC1,POLR3F,HOXA10,ERF,TCERG1,RARS,ATP5F1A,PUM3,CDC45,MAST2,SMARCAL1,PRPS1,ENSCAFG00000013366,TBX15,GRWD1,ELF4,PRMT5,PSMC1,CTH,ARHGEF2,SUPT16H,PDHA1,TADA2A,HAT1,HIST2H2AC,HOXA9,GL	down_ACOR_VSMC
GO:0019725	0.0372	0.979	22	245	cellular homeostasis	BP	0.872	P4HB,SLC39A7,NR1H4,ENSCAFG00000013214,ERO1A,ENPP1,ENSCAFG00000003477,ATP2B1,LRRC8A,SMARCA4,HIF1A,DNAJC10,NXN,PDIA4,TFRC,APOE,LYN,DDIT3,CLCN3,HTT,CALM1,DISC1	down_VSAP_EC
GO:2000576	0.0372	0.998	2	4	positive regulation of microtubule motor	BP	1	DYNC1I2,DYNC1I1	up_ACOR_VSMC
GO:2000580	0.0372	0.998	2	4	regulation of ATP-dependent microtubule motor activity, plus-end-	BP	1	DYNC1I2,DYNC1I1	up_ACOR_VSMC
GO:2000582	0.0372	0.998	2	4	positive regulation of ATP-dependent microtubule motor activity, plus-end-	BP	1	DYNC1I2,DYNC1I1	up_ACOR_VSMC
GO:0048663	0.0372	0.997	2	13	neuron fate commitment	BP	1	HOXC10,DLL1	up_AFEM_EC
GO:0021545	0.0372	0.999	1	21	cranial nerve development	BP	1	CITED2	down_VPOR_EC
GO:0035195	0.0372	0.999	1	28	gene silencing by miRNA	BP	1	ESR1	up_AORT_VSMC
GO:0070201	0.0373	0.978	26	187	regulation of establishment of protein localization	BP	1	EXPH5,SH3TC2,TLR1,LEP,HTR2B,TCAF1,MBP,FERMT1,BMP4,TM7SF3,CD274,ANKRD1,F2RL1,CDKN1A,PTPN22,OS9,PRKCE,PSEN1,JUP,CCL5,NOD2,SCAMP5,INPP5K,PFKL,PARK7,PTPN14	up_ACOR_VSMC
GO:0019222	0.0373	0.984	15	1934	regulation of metabolic process	BP	1	ACVRL1,YEATS2,LYN,IGF1R,YBX3,HIVEP2,HOXA7,PRKAR2A,SULF1,ELK3,HOXA1,ACSL3,MYO1D,ASPN,NEDD4	down_ACOR_EC
GO:0043523	0.0373	0.987	7	52	regulation of neuron apoptotic process	BP	0.872	HYOU1,AARS,HIF1A,ZPR1,DDIT3,SIX4,NRP1	down_VSAP_EC
GO:0016241	0.0373	0.989	6	41	regulation of macroautophagy	BP	0.872	SESN2,HIF1A,BNIP3,CDC37,SH3GLB1,HTT	down_VSAP_EC
GO:0021522	0.0373	0.995	3	8	spinal cord motor neuron differentiation	BP	1	LMO4,TBX20,CLN8	up_ACOR_VSMC
GO:0035239	0.0373	0.994	3	299	tube morphogenesis	BP	1	HOXA3,NKX2-5,C3AR1	up_VPOR_VSMC
GO:0034377	0.0373	0.999	1	7	plasma lipoprotein particle assembly	BP	1	ACSL3	down_ACOR_EC
GO:0065005	0.0373	0.999	1	7	protein-lipid complex assembly	BP	1	ACSL3	down_ACOR_EC
GO:0034394	0.0373	0.999	1	19	protein localization to cell surface	BP	1	RAB11B	down_VPOR_EC
GO:0010224	0.0373	0.999	1	9	response to UV-B	BP	1	MME	up_VFEM_EC
GO:0031272	0.0373	0.999	1	8	regulation of pseudopodium assembly	BP	1	CDC42EP5	up_VFEM_EC
GO:0031274	0.0373	0.999	1	8	positive regulation of pseudopodium assembly	BP	1	CDC42EP5	up_VFEM_EC
GO:0034138	0.0373	0.999	1	9	toll-like receptor 3 signaling pathway	BP	1	CAV1	up_VSAP_VSMC
GO:0046330	0.037309666	0.99166	4	47	positive regulation of JNK cascade	BP	1	FZD5,NOD2,TNFRSF19,SH3RF3	up_ACOR_EC

GO:0050793	0.0374	0.972	85	731	regulation of developmental process	BP	1	VEGFD,TMEM176B,TMEM176A,SMAD6,LEP,LBH,GATA5,CD83,CDC7,RASSF2,FNDC5,MBP,ZRANB1,MLLT3,ITM2C,FERMT1,BMP4,LNPK,FGFR2,TP53BP1,GJA1,TLR3,SEC24B,WASF3,PPARGC1A,LATS2,PDE5A,CITED2,TP53INP1,ISG15,ZNRF3,CD274,ZNF365,SASH1,LMO4,FGF18,FAM210B,ATRN,P4HTM,ZMYM2,PLAC8B,LIMS2,PLEKHB2,MARCH5,BNIP3,EGLN1,CLSTN1,NRP1,ULK2,DMPK,NPNT,SMARCD3,SLITRK5,TBX20,TBX5,CCDC3,FZD3,DKKL1,FRS2,DAG1,APP,LAMA3,PSEN1,TGFBR1,SYNJ2BP,CHD7,JUP,SIPA1L1,DDR2,WWC2,SSH3,EDN1,ASAP1,B2M,DISC1,TNFRSF11B,CNOT4,DOCK5,ERAP1,SLC8A1,STC2,BVES,RREB1,CRKL,PIK3C2A	up_ACOR_VSMC
GO:0045595	0.0374	0.975	38	469	regulation of cell differentiation	BP	0.874	ARHGEF2,PRICKLE1,SIPA1L1,SERPINE1,SOCS5,ENPP1,ERRFI1,DPYSL3,HOOK3,TRIB3,IFRD1,ILK,TGFBR1,PTGS2,HIF1A,SLC45A3,TNIK,PTPN2,EVI2B,UST,CTNNB1,ENSCAFG00000002015,APOE,KLF5,LYN,STRAP,DDIT3,MMD,VLDLR,REST,PRMT1,DISC1,INHBA,HIF1AN,SIX4,NRP1,YWHAH,KDM3A	down_VSAP_EC
GO:1901360	0.0374	0.992	6	2016	organic cyclic compound metabolic process	BP	1	TLR2,TBX15,EYA1,HOXA9,HOXA10,IRX5	up_AFEM_VSMC
GO:0060042	0.0374	0.993	4	14	retina morphogenesis in camera-type eye	BP	1	THRB,CDON,IRX5,DLL1	up_VSAP_EC
GO:0043588	0.0374	0.997	2	74	skin development	BP	1	HOXA7,CYP26B1	up_VFEM_EC
GO:0061448	0.0374	0.997	2	89	connective tissue development	BP	1	HOXA3,NR1H4	up_VPUL_EC
GO:0032663	0.0374	0.999	1	19	regulation of interleukin-2 production	BP	1	HDAC7	down_VPOR_EC
GO:0006826	0.0374	0.999	1	15	iron ion transport	BP	1	ENSCAFG00000005494	up_AORT_EC
GO:0042129	0.0374	0.999	1	35	regulation of T cell proliferation	BP	1	ARG2	up_AORT_VSMC
GO:0021515	0.0374	0.999	1	9	cell differentiation in spinal cord	BP	1	HOXC10	up_VSAP_VSMC
GO:0055117	0.0375	0.991	5	21	regulation of cardiac muscle contraction	BP	1	FGF13,ADRA1B,KCNE3,PDE5A,JUP	up_ACOR_VSMC
GO:0042118	0.0375	0.998	2	4	endothelial cell activation	BP	1	PRMT5,HOXA9	down_ACOR_VSMC
GO:0007600	0.0375	0.997	2	73	sensory perception	BP	1	MME,SIX1	up_VFEM_EC
GO:0032970	0.0375	1	1	130	regulation of actin filament-based process	BP	1	JUP	down_VFEM_VSMC
GO:0045912	0.0375	0.999	1	15	negative regulation of carbohydrate metabolic	BP	1	FBP1	up_VFEM_VSMC
GO:0030513	0.0375	0.999	1	11	positive regulation of BMP signaling pathway	BP	1	NEO1	up_VPUL_EC
GO:0070670	0.0375	0.999	1	11	response to interleukin-4	BP	1	MRC1	up_VPUL_EC
GO:0071353	0.0375	0.999	1	11	cellular response to interleukin-4	BP	1	MRC1	up_VPUL_EC
GO:1902042	0.0375	0.999	1	11	negative regulation of extrinsic apoptotic signaling pathway via	BP	1	ARHGEF2	up_VPUL_EC
GO:0060070	0.0376	0.988	6	105	canonical Wnt signaling pathway	BP	1	GREM1,FAM53B,CYLD,RECK,AXIN2,KANK1	up_AFEM_EC
GO:0032330	0.0376	0.991	5	20	regulation of chondrocyte differentiation	BP	1	HOXA11,AXIN2,ZBTB16,RARG,SHOX2	up_VSAP_EC
GO:0002551	0.0376	0.997	2	5	mast cell chemotaxis	BP	0.793	KIT,SWAP70	up_VCAV_EC
GO:0097531	0.0376	0.997	2	5	mast cell migration	BP	0.793	KIT,SWAP70	up_VCAV_EC
GO:0032456	0.0376	0.999	1	19	endocytic recycling	BP	1	RAB11B	down_VPOR_EC
GO:0030593	0.0376	0.999	1	19	neutrophil chemotaxis	BP	1	C3AR1	up_VPOR_VSMC
GO:0031111	0.0376	0.999	1	11	negative regulation of microtubule polymerization or	BP	1	ARHGEF2	up_VPUL_EC
GO:2001275	0.0376	0.999	1	11	positive regulation of glucose import in response to insulin	BP	1	NR1H4	up_VPUL_EC
GO:0030032	0.0376	0.999	1	19	lamellipodium assembly	BP	1	SLIT2	up_VPUL_VSMC
GO:0050767	0.0377	0.98	18	184	regulation of neurogenesis	BP	0.874	ARHGEF2,SIPA1L1,DPYSL3,HOOK3,IFRD1,HIF1A,SLC45A3,TNIK,UST,CTNNB1,APOE,LYN,MMD,VLDLR,REST,DISC1,NRP1,YWHAH	down_VSAP_EC

GO:1903050	0.0377	0.984	10	88	regulation of proteolysis involved in cellular protein catabolic process	BP	0.874	PRICKLE1,PLK3,SOCS5,TRIB3,UCHL5,GSK3B,DISC1,RAD23A,TMTC3,USP14	down_VSAP_EC
GO:0048593	0.0377	0.986	8	41	camera-type eye morphogenesis	BP	1	THRB,CDON,IRX5,DLL1,MFN2,ZEB1,KDM2B,CITED2	up_VSAP_EC
GO:1900180	0.0377	0.988	6	50	regulation of protein localization to nucleus	BP	1	ENSCAFG00000014978,TRIM8,GLI3,PARK7,MAVS,GLIS2	down_VCAV_EC
GO:0031175	0.0377	0.997	2	224	neuron projection development	BP	1	SLITRK5,LIMK1	up_VCAV_VSMC
GO:0071407	0.0377	1	1	129	cellular response to organic cyclic compound	BP	1	JUP	down_VFEM_VSMC
GO:0030206	0.0377	0.999	1	9	chondroitin sulfate biosynthetic process	BP	1	CHST7	up_VFEM_EC
GO:0032715	0.0377	0.999	1	11	negative regulation of interleukin-6 production	BP	1	NR1H4	up_VPUL_EC
GO:0010827	0.037765516	0.99386	3	28	regulation of glucose transmembrane transport	BP	1	NR1H4,ENSCAFG00000004331,RARRES2	up_ACOR_EC
GO:0051402	0.0378	0.986	8	64	neuron apoptotic process	BP	0.874	HYOU1,AARS,HIF1A,ZPR1,DDIT3,FAM162A,SIX4,NRP1	down_VSAP_EC
GO:0007399	0.0378	0.995	3	564	nervous system development	BP	1	HOXC10,EYA1,IRX5	up_AFEM_VSMC
GO:0090382	0.0378	0.999	1	8	phagosome maturation	BP	1	SPG11	down_ACOR_EC
GO:0021604	0.0378	0.999	1	6	cranial nerve structural organization	BP	1	NRP2	down_ACOR_EC
GO:0021612	0.0378	0.999	1	6	facial nerve structural organization	BP	1	NRP2	down_ACOR_EC
GO:0008627	0.0378	0.999	1	5	intrinsic apoptotic signaling pathway in response to osmotic	BP	1	ARHGEF2	up_VPOR_EC
GO:0060043	0.0378	0.999	1	14	regulation of cardiac muscle cell proliferation	BP	1	NKX2-5	up_VPOR_VSMC
GO:0061337	0.0378	0.999	1	16	cardiac conduction	BP	1	NKX2-5	up_VPOR_VSMC
GO:0009954	0.0378	0.999	1	16	proximal/distal pattern formation	BP	1	CYP26B1	up_VPOR_VSMC
GO:0060914	0.0378	0.999	1	11	heart formation	BP	1	TBX5	up_VPUL_EC
GO:2000482	0.0378	0.999	1	9	regulation of interleukin-8 secretion	BP	1	TLR2	up_VSAP_VSMC
GO:0001944	0.0379	0.979	20	263	vasculature development	BP	1	HSPB6,PDGFRB,DCN,ANGPT1,BMPR1A,LOX,RECK,ANPEP,SEMA3C,THY1,B4GALT1,GLI3,ZFP36L1,MYDGF,SASH1,CDH2,SPRED1,SHC1,GREM1,CLIC4	down_VCAV_EC
GO:0044282	0.0379	0.985	9	89	small molecule catabolic process	BP	1	AKT2,ACAA1,THNSL2,PEX5,ENSCAFG00000002977,LONP2,GLS2,BCKDHB,ACAT1	down_VCAV_EC
GO:1903555	0.0379	0.987	7	37	regulation of tumor necrosis factor superfamily cytokine	BP	0.795	TNFAIP3,CD34,TLR4,ARG2,TLR3,RIPK1,ARHGEF2	up_VCAV_EC
GO:0021675	0.0379	0.99	5	29	nerve development	BP	0.874	SULF1,ILK,CTNNB1,SIX4,NRP1	down_VSAP_EC
GO:0032655	0.0379	0.992	4	16	regulation of interleukin-12 production	BP	0.795	TLR4,IRF8,TLR3,HMGB1	up_VCAV_EC
GO:0022603	0.0379	0.994	3	301	regulation of anatomical structure morphogenesis	BP	1	BAMBI,ANKRD6,C3AR1	up_VPOR_VSMC
GO:0060788	0.0379	0.997	2	5	ectodermal placode formation	BP	0.874	CTNNB1,SIX4	down_VSAP_EC
GO:0071697	0.0379	0.997	2	5	ectodermal placode morphogenesis	BP	0.874	CTNNB1,SIX4	down_VSAP_EC
GO:0030517	0.0379	0.997	2	5	negative regulation of axon extension	BP	0.874	IFRD1,NRP1	down_VSAP_EC
GO:0061551	0.0379	0.999	1	5	trigeminal ganglion development	BP	1	SEMA3A	up_VPOR_EC
GO:1905331	0.0379	0.999	1	9	negative regulation of morphogenesis of an	BP	1	SIX1	up_VFEM_EC
GO:0010092	0.0379	0.999	1	19	specification of animal organ identity	BP	1	GATA5	up_VPUL_VSMC
GO:0010562	0.037997047	0.98122	14	312	positive regulation of phosphorus metabolic process	BP	1	NR1H4,SOX9,ENSCAFG00000004331,FZD5,DUSP26,NOD2,PAK3,TNFRSF19,MAVS,LRP4,SH3RF3,RARRES2,NRP1,PRKCD	up_ACOR_EC
GO:0045937	0.037997047	0.98122	14	312	positive regulation of phosphate metabolic process	BP	1	NR1H4,SOX9,ENSCAFG00000004331,FZD5,DUSP26,NOD2,PAK3,TNFRSF19,MAVS,LRP4,SH3RF3,RARRES2,NRP1,PRKCD	up_ACOR_EC

GO:0071310	0.038	0.972	65	634	cellular response to organic substance	BP	0.797	ACKR1,HCLS1,GBP5,KIT,PRCP,CXCL8,LAPTM5,CXCR4,CALCRL,TNFAIP3,PPARG,TNIP1,PDGFB,BAIAP2L1,TMEM173,PDE4D,MEF2C,FERMT1,ATF3,TIPARP,DLL4,RAPGEF1,NLRC5,GATA3,RALB,SMAD1,TFPI,IRF8,YES1,MTOR,BMP2,HHEX,ST3GAL6,ABCA1,MAPK13,IFNGR1,RIOK3,ENSCAFG00000010596,FLNB,PAK2,PRKD2,SMARCA4,NCK1,TIMELESS,OTUD4,RAPGEF2,CTDSPL2,CREB1,CTNNA1,CNOT7,ADIPOR2,BLM,RIPK1,LSM14A,SKIL,PDGFD,UFSP2,HIF1A,KDM3A,NR4A1,ENSCAFG00000002015,JCAD,ZEB1,ARHGEF2,TJP2	up_VCAV_EC
GO:0023051	0.038	0.988	7	954	regulation of signaling	BP	1	NR1H4,RGS1,CCL24,PTGER2,FLRT3,ARHGEF2,NEO1	up_VPUL_EC
GO:0034332	0.038	0.99	5	32	adherens junction organization	BP	0.874	VEGFA,ACTN1,CTNNB1,ENSCAFG00000002015,NRP1	down_VSAP_EC
GO:1904062	0.038	0.996	2	62	regulation of cation transmembrane transport	BP	1	LYN,NEDD4	down_ACOR_EC
GO:0008584	0.038	0.996	2	39	male gonad development	BP	1	SEMA3A,ACVR2A	up_VPOR_EC
GO:0046546	0.038	0.996	2	39	development of primary male sexual	BP	1	SEMA3A,ACVR2A	up_VPOR_EC
GO:0031349	0.038	0.997	2	90	positive regulation of defense response	BP	1	NR1H4,CCL24	up_VPUL_EC
GO:0003012	0.038	0.997	2	90	muscle system process	BP	1	MYBPC2,HTR7	up_VPUL_EC
GO:0036230	0.038	0.999	1	9	granulocyte activation	BP	1	STX4	down_ACOR_EC
GO:0006536	0.038	0.999	1	10	glutamate metabolic process	BP	1	NR1H4	up_APUL_EC
GO:0033002	0.038	0.999	1	55	muscle cell proliferation	BP	1	TBX5	up_APUL_VSMC
GO:0051924	0.038	0.999	1	53	regulation of calcium ion transport	BP	1	HOXA3	up_APUL_VSMC
GO:0008206	0.038	0.999	1	11	bile acid metabolic process	BP	1	NR1H4	up_VPUL_EC
GO:0060045	0.038	0.999	1	11	positive regulation of cardiac muscle cell	BP	1	TBX5	up_VPUL_EC
GO:0021591	0.038067282	0.99671	2	12	ventricular system development	BP	1	AQP1,SEMA6D	up_ACOR_EC
GO:0030856	0.0381	0.986	8	41	regulation of epithelial cell differentiation	BP	1	HOXA7,BTG1,DLL1,OSR1,ZFP36L1,ZEB1,ACVRL1,KEAP1	up_VSAP_EC
GO:2000177	0.0381	0.989	6	26	regulation of neural precursor cell	BP	0.797	DLL4,RAPGEF1,PROX1,CTNNA1,HIF1A,CDON	up_VCAV_EC
GO:0003407	0.0381	0.994	3	15	neural retina development	BP	1	THY1,IRX5,THRB	down_VCAV_EC
GO:0045637	0.0381	0.996	2	73	regulation of myeloid cell differentiation	BP	1	HOXA9,HOXA7	up_VFEM_EC
GO:0035136	0.0381	0.999	1	16	forelimb morphogenesis	BP	1	HOXA9	up_AORT_EC
GO:0045683	0.0381	0.999	1	9	negative regulation of epidermis development	BP	1	HOXA7	up_VFEM_EC
GO:0055017	0.0381	0.999	1	19	cardiac muscle tissue growth	BP	1	TBX5	up_VPUL_VSMC
GO:0010498	0.0382	0.979	18	194	proteasomal protein catabolic process	BP	0.874	PRICKLE1,PLK3,SOCS5,HM13,HSP90B1,TRIB3,SEL1L,DNAJC10,UCHL5,SPOPL,DNAJB9,DDIT3,GSK3B,SPOP,RAD23A,TMTC3,USP14,TNFAIP1	down_VSAP_EC
GO:0006486	0.0382	0.981	14	91	protein glycosylation	BP	1	GALNT3,GALNT10,ST3GAL5,B3GALNT2,B3GNT7,CCDC126,MAGT1,STT3B,FUT11,FUT8,POMK,PSEN1,EOGT,LMF1	up_ACOR_VSMC
GO:0043413	0.0382	0.981	14	91	macromolecule glycosylation	BP	1	GALNT3,GALNT10,ST3GAL5,B3GALNT2,B3GNT7,CCDC126,MAGT1,STT3B,FUT11,FUT8,POMK,PSEN1,EOGT,LMF1	up_ACOR_VSMC
GO:0036035	0.0382	0.997	2	7	osteoclast development	BP	1	NOTCH2,FBN1	down_VCAV_EC
GO:0051093	0.0382	0.997	2	273	negative regulation of developmental process	BP	1	HOXA9,WNT7A	up_AORT_VSMC
GO:0050869	0.0382	0.999	1	8	negative regulation of B cell activation	BP	1	LYN	down_ACOR_EC
GO:0048486	0.0382	0.999	1	10	parasympathetic nervous system development	BP	1	SIX1	up_VFEM_EC
GO:0046580	0.0382	0.999	1	18	negative regulation of Ras protein signal	BP	1	FBP1	up_VFEM_VSMC
GO:0048641	0.0382	0.999	1	16	regulation of skeletal muscle tissue	BP	1	SHOX2	up_VFEM_VSMC
GO:1901888	0.038211713	0.99375	3	29	regulation of cell junction assembly	BP	1	ARHGAP6,FZD5,NRP1	up_ACOR_EC
GO:0002686	0.038245924	0.99669	2	12	negative regulation of leukocyte migration	BP	1	MMP28,PTGER4	up_ACOR_EC

GO:2000249	0.038294772	0.99668	2	12	regulation of actin cytoskeleton	BP	1	ARHGDIB,NRP1	up_ACOR_EC
GO:0045785	0.0383	0.984	10	107	positive regulation of cell adhesion	BP	1	GPAM,FBLN2,RHOD,ENSCAFG00000014978,TFE3,THY1,GLI3,EDIL3,ZBTB16,ECM2	down_VCAV_EC
GO:0097529	0.0383	0.986	8	52	myeloid leukocyte migration	BP	0.799	EDNRB,KIT,CXCL8,PDGFB,PDE4D,MMP28,SWAP70,HMGB1	up_VCAV_EC
GO:0010824	0.0383	0.99	5	23	regulation of centrosome duplication	BP	1	POC1A,NUP62,PLK4,RBM14,GEN1	down_ACOR_VSMC
GO:0007031	0.0383	0.99	5	20	peroxisome organization	BP	1	PEX7,PEX2,LONP2,PEX6,MAVS	up_VSAP_EC
GO:0050688	0.0383	0.99	5	20	regulation of defense response to virus	BP	1	NLRX1,TKFC,RNF216,PQBP1,MAVS	up_VSAP_EC
GO:0090343	0.0383	0.998	2	4	positive regulation of cell aging	BP	1	HMGA1,ARG2	down_ACOR_VSMC
GO:2000774	0.0383	0.998	2	4	positive regulation of cellular senescence	BP	1	HMGA1,ARG2	down_ACOR_VSMC
GO:0002265	0.0383	0.998	2	3	astrocyte activation involved in immune	BP	1	APP,PSEN1	up_ACOR_VSMC
GO:0098742	0.0383	0.999	1	44	cell-cell adhesion via plasma-membrane	BP	1	GATA5	down_VSAP_VSMC
GO:0007140	0.0383	0.999	1	10	male meiotic nuclear division	BP	1	CYP26B1	up_VFEM_EC
GO:0021872	0.0383	0.999	1	19	forebrain generation of neurons	BP	1	SLIT2	up_VPUL_VSMC
GO:0010939	0.0383	0.999	1	9	regulation of necrotic cell death	BP	1	CAV1	up_VSAP_VSMC
GO:0051302	0.0384	0.986	8	47	regulation of cell division	BP	1	AURKB,PLK3,E2F8,CDC6,NUP62,PDXP,FGF5,ASPM	down_ACOR_VSMC
GO:0032870	0.0384	0.985	8	162	cellular response to hormone stimulus	BP	1	LEP,LBH,CRY2,CHRM2,AVPR1A,CAMK2A,NR3C1,KANK1	up_AFEM_EC
GO:0099536	0.0384	0.986	7	133	synaptic signaling	BP	1	KCNC4,GPR1,CHRM2,SLC6A6,HTR1D,CAMK2A,DMPK	up_AFEM_EC
GO:0010389	0.0384	0.989	6	30	regulation of G2/M transition of mitotic cell	BP	0.799	RAB11A,TAOK1,BLM,RAD21,RAD17,TOPBP1	up_VCAV_EC
GO:0009967	0.0384	0.99	5	435	positive regulation of signal transduction	BP	1	NETO1,SALL1,TLR2,CAV1,SHOX2	up_VSAP_VSMC
GO:0051241	0.0384	0.992	4	336	negative regulation of multicellular organismal	BP	1	NR1H4,TBX5,BPI,PLCL1	up_APUL_EC
GO:0045670	0.0384	0.994	3	16	regulation of osteoclast differentiation	BP	1	NOTCH2,FBN1,TFE3	down_VCAV_EC
GO:0060338	0.0384	0.995	3	10	regulation of type I interferon-mediated	BP	0.799	NLRC5,CNOT7,LSM14A	up_VCAV_EC
GO:0060340	0.0384	0.998	2	4	positive regulation of type I interferon-mediated signaling pathway	BP	0.799	NLRC5,LSM14A	up_VCAV_EC
GO:0007350	0.0384	0.999	1	7	blastoderm segmentation	BP	1	NRP2	down_ACOR_EC
GO:1903305	0.0384	0.999	1	36	regulation of regulated secretory pathway	BP	1	WNT7A	up_AORT_VSMC
GO:0015800	0.0384	0.999	1	10	acidic amino acid transport	BP	1	NTRK2	up_APUL_EC
GO:0043010	0.0385	0.981	15	96	camera-type eye development	BP	1	THRB,CDON,OSR2,MAB21L1,IRX5,DLL1,RARG,MFN2,ZEB1,CTNS,CLCN2,KDM2B,ACVRL1,TGFBR2,CITED2	up_VSAP_EC
GO:0043087	0.0385	0.982	13	83	regulation of GTPase activity	BP	0.799	AMOT,FGD6,RAPGEF1,MTOR,CHN2,IQGAP2,RAPGEF2,TBC1D1,ITGA6,ALS2,ENSCAFG00000002015,RABGAP1,USP6NL	up_VCAV_EC
GO:0003008	0.0385	0.992	4	336	system process	BP	1	HTR7,NPNT,NTRK2,HOXA1	up_APUL_EC
GO:0070988	0.0385	0.992	4	14	demethylation	BP	0.799	GATA3,OTUD4,KDM3A,TET2	up_VCAV_EC
GO:0003433	0.0385	0.998	2	4	chondrocyte development involved in endochondral bone morphogenesis	BP	1	POC1A,SERPINH1	down_ACOR_VSMC
GO:0042987	0.0385	0.999	1	10	amyloid precursor protein catabolic process	BP	1	LYN	down_ACOR_EC
GO:0071887	0.0385	0.999	1	34	leukocyte apoptotic process	BP	1	ARG2	up_AORT_VSMC
GO:0030511	0.0385	0.999	1	10	positive regulation of transforming growth factor beta receptor	BP	1	NPNT	up_APUL_EC
GO:1903846	0.0385	0.999	1	10	positive regulation of cellular response to transforming growth	BP	1	NPNT	up_APUL_EC
GO:0060071	0.0385	0.999	1	14	Wnt signaling pathway, planar cell polarity	BP	1	ANKRD6	up_VPOR_VSMC

GO:0023056	0.0386	0.975	37	462	positive regulation of signaling	BP	0.874	VEGFA,SULF1,NR1H4,JCAD,PTPN11,ILK,SMARCA4,MYO1C,CHSY1,KIF5B,HIF1A,TNIK,PTPN2,NEO1,MTDH,ESM1,ADAM17,MAGED1,CTNNB1,ENSCAFG00000002015,APOE,LYN,BNIP3,FAM162A,ATF3,SH3GLB1,HTT,CALM1,DISC1,PAK2,LRRTM2,INHBA,NRP1,SH3RF3,MADD,SEC13,MID1	down_VSAP_EC
GO:1901890	0.0386	0.994	3	13	positive regulation of cell junction assembly	BP	0.874	VEGFA,ENSCAFG00000002015,NRP1	down_VSAP_EC
GO:0009142	0.0386	0.999	1	46	nucleoside triphosphate biosynthetic process	BP	1	AK5	down_VSAP_VSMC
GO:0009410	0.0386	0.999	1	27	response to xenobiotic stimulus	BP	1	SLITRK5	up_VCAV_VSMC
GO:0051193	0.0386	0.999	1	15	regulation of cofactor metabolic process	BP	1	FBP1	up_VFEM_VSMC
GO:0042127	0.0387	0.973	55	452	regulation of cell proliferation	BP	1	CDON,WNT11,HNF4A,CD274,AXIN2,C5AR2,OSR2,PTGFR,MAB21L1,LIMS2,IL6R,CRIP2,FGF7,ABL1,PEX2,BTG1,EMP2,DLL1,OSR1,PDGFD,SAV1,HPSE,ZBTB16,RARG,STAT5A,STAT6,AMBRA1,MORC3,DDR2,MFN2,ZFP36L1,TIAM1,MYC,PTGES,ZEB1,FBXW4,SULF2,SHOX2,FBXO4,MYD88,E2F4,KDM2B,FBLN1,RAF1,PNP,NFIB,ZNF580,STX4,SMARCA2,ACVRL1,NCK1,HMOX1,TGFBR2,LBH,DPT	up_VSAP_EC
GO:0045765	0.0387	0.988	6	105	regulation of angiogenesis	BP	1	LEP,ANGPT4,DLL1,F3,GREM1,TBXA2R	up_AFEM_EC
GO:1900227	0.0387	0.998	2	4	positive regulation of NLRP3 inflammasome	BP	0.802	GBP5,TLR4	up_VCAV_EC
GO:0042147	0.0387	0.996	2	38	retrograde transport, endosome to Golgi	BP	1	GCC2,TMEM87B	up_VPOR_EC
GO:0051098	0.0387	0.996	2	129	regulation of binding	BP	1	HOXA3,BAMBI	up_VPOR_VSMC
GO:0072009	0.038788876	0.99121	4	48	nephron epithelium development	BP	1	SOX9,AQP1,FOXC2,CLSTN1	up_ACOR_EC
GO:0030178	0.0388	0.988	6	52	negative regulation of Wnt signaling pathway	BP	1	CLSTN1,GLI3,CDH2,GREM1,NPHP4,FOXO3	down_VCAV_EC
GO:0007492	0.0388	0.991	4	27	endoderm development	BP	1	BMPR1A,COL6A1,LAMA3,ZFP36L1	down_VCAV_EC
GO:0006875	0.0388	0.996	2	129	cellular metal ion homeostasis	BP	1	TRPC4,C3AR1	up_VPOR_VSMC
GO:0008544	0.0388	0.996	2	75	epidermis development	BP	1	HOXA7,CYP26B1	up_VFEM_EC
GO:0007389	0.0388	1	1	137	pattern specification process	BP	1	GATA5	down_VFEM_VSMC
GO:0090102	0.0388	0.999	1	10	cochlea development	BP	1	HOXA1	up_APUL_EC
GO:0051492	0.0388	0.999	1	28	regulation of stress fiber assembly	BP	1	LIMK1	up_VCAV_VSMC
GO:0001657	0.0389	0.988	6	42	ureteric bud development	BP	0.874	ENSCAFG00000032470,ILK,HOXB7,MAGED1,CTNNB1,SIX4	down_VSAP_EC
GO:0072163	0.0389	0.988	6	42	mesonephric epithelium development	BP	0.874	ENSCAFG00000032470,ILK,HOXB7,MAGED1,CTNNB1,SIX4	down_VSAP_EC
GO:0072164	0.0389	0.988	6	42	mesonephric tubule development	BP	0.874	ENSCAFG00000032470,ILK,HOXB7,MAGED1,CTNNB1,SIX4	down_VSAP_EC
GO:0001649	0.0389	0.991	4	54	osteoblast differentiation	BP	1	TWIST2,GREM1,PTHLH,AXIN2	up_AFEM_EC
GO:0010107	0.0389	0.997	2	5	potassium ion import	BP	0.803	KCNJ3,KCNJ2	up_VCAV_EC
GO:0051090	0.0389	1	1	134	regulation of DNA-binding transcription factor	BP	1	JUP	down_VFEM_VSMC
GO:0010803	0.0389	0.999	1	10	regulation of tumor necrosis factor-mediated signaling pathway	BP	1	NR1H4	up_APUL_EC
GO:0034381	0.0389	0.999	1	10	plasma lipoprotein particle clearance	BP	1	NR1H4	up_APUL_EC
GO:0048546	0.0389	0.999	1	16	digestive tract morphogenesis	BP	1	SHOX2	up_VFEM_VSMC
GO:0044766	0.0389	0.999	1	9	multi-organism transport	BP	1	CAV1	up_VSAP_VSMC
GO:1902579	0.0389	0.999	1	9	multi-organism localization	BP	1	CAV1	up_VSAP_VSMC
GO:0001953	0.039	0.992	4	14	negative regulation of cell-matrix adhesion	BP	1	HOXA7,FAM107A,PIK3R1,ACVRL1	up_VSAP_EC
GO:0060394	0.039	0.997	2	6	negative regulation of pathway-restricted SMAD protein phosphorylation	BP	0.874	SNX25,STRAP	down_VSAP_EC
GO:0090103	0.039	0.999	1	8	cochlea morphogenesis	BP	1	HOXA1	down_ACOR_EC
GO:0072350	0.039	0.999	1	18	tricarboxylic acid metabolic process	BP	1	SUCLG2	down_AFEM_EC

GO:0045655	0.039	0.999	1	9	regulation of monocyte differentiation	BP	1	HOXA7	up_VFEM_EC
GO:0010927	0.039085368	0.99356	3	28	cellular component assembly involved in	BP	1	MYOZ2,TMOD1,ACTN2	up_ACOR_EC
GO:2001057	0.0391	0.992	4	16	reactive nitrogen species metabolic process	BP	0.807	CD34,TLR4,NOS3,NOS2	up_VCAV_EC
GO:0030238	0.0391	0.998	2	4	male sex determination	BP	1	SF1,SIX4	down_ACOR_VSMC
GO:0006607	0.0391	0.997	2	5	NLS-bearing protein import into nucleus	BP	0.874	KPNB1,KPNA1	down_VSAP_EC
GO:1903672	0.0391	0.999	1	20	positive regulation of sprouting angiogenesis	BP	1	HDAC7	down_VPOR_EC
GO:0050715	0.0391	0.999	1	29	positive regulation of cytokine secretion	BP	1	TLR2	up_AFEM_VSMC
GO:0016441	0.0391	0.999	1	30	posttranscriptional gene silencing	BP	1	ESR1	up_AORT_VSMC
GO:0035194	0.0391	0.999	1	30	posttranscriptional gene silencing by RNA	BP	1	ESR1	up_AORT_VSMC
GO:0003171	0.0391	0.999	1	10	atrioventricular valve development	BP	1	TBX5	up_APUL_EC
GO:0055062	0.0391	0.999	1	5	phosphate ion homeostasis	BP	1	HOXA3	up_VPOR_EC
GO:0072505	0.0391	0.999	1	5	divalent inorganic anion homeostasis	BP	1	HOXA3	up_VPOR_EC
GO:0072506	0.0391	0.999	1	5	trivalent inorganic anion homeostasis	BP	1	HOXA3	up_VPOR_EC
GO:0045616	0.0391	0.999	1	9	regulation of keratinocyte differentiation	BP	1	HOXA7	up_VFEM_EC
GO:0016055	0.0392	0.986	7	134	Wnt signaling pathway	BP	1	GREM1,FAM53B,CYLD,RECK,LGR6,AXIN2,KANK1	up_AFEM_EC
GO:0198738	0.0392	0.986	7	134	cell-cell signaling by wnt	BP	1	GREM1,FAM53B,CYLD,RECK,LGR6,AXIN2,KANK1	up_AFEM_EC
GO:0009954	0.0392	0.992	4	16	proximal/distal pattern formation	BP	1	GREM1,HOXC10,HOXA9,GLI1	down_ACOR_VSMC
GO:0003177	0.0392	0.995	3	8	pulmonary valve development	BP	1	SMAD6,BMP4,TBX20	up_ACOR_VSMC
GO:1901564	0.0393	0.97	119	2053	organonitrogen compound metabolic process	BP	1	PCOLCE,CST3,ADAMTS2,GPAM,NOTCH2,PDGFRB,KIRREL1,SMAD9,LRP1,DCN,MAOA,CFD,PRNP,UBA52,ADCY6,GLIPR2,BGN,LARP6,COA1,BMPR1A,KRTCAP2,VKORC1,RECK,CLIP3,NUMBL,CHID1,AKT1S1,TIMP2,AKT2,PSMF1,LCAT,RHBDL1,SAMD4B,EEF2K,H6PD,HR,RPL23,RPL13,PCSK7,PLCL1,DDOST,CSPG4,PRMT6,UROS,NT5C2,THNSL2,PGLS,ATP5MC1,GFPT2,UQCR10,ASPN,PIGS,HS3ST3B1,EIF3K,THY1,NECAB3,RNF216,ADK,WDR45,MRPL37,B4GALT1,GLI3,PARK7,RPL27A,EEF2,MAST2,ZFP36L1,MYDGF,SASH1,SEC11C,CLEC16A,RNPEP,RPL27,COPS7A,ARL2BP,AMPD2,IFT57,MAVS,MRPS11,CDH2,ENSCAFG00000002977,LOXL3,ORMDL3,GSDME,HPDL,CAMK2D,RPS27,FUT8,ENSCAFG00000017708,SPRED1,A2M,SEC61B,ACSS2,MAPRE3,CDKL2,HM13,PRKCSH,DDR2,SHC1,CRTAP,PCSK6,ENSCAFG00000015122,PYM1,FBH1,GREM1,RPN2,KLHL29,ATOX1,LONP2,NAPEPLD,GLS2,FOXO3,SOAT1,PACSIN3,EIF4EBP1,BCKDHB,ACAT1,BRSK1,CDK14	down_VCAV_EC
GO:0010562	0.0393	0.974	40	312	positive regulation of phosphorus metabolic process	BP	1	ADRA1B,LEP,MAP3K8,MID1,HTR2B,RASSF2,BMP4,FGFR2,TLR3,SH3RF2,PPARGC1A,PDE5A,AR,SASH1,LMO4,FGF18,F2RL1,FAM129A,NRP1,PTPN22,NPNT,SMPD1,PRKCE,CCNT1,FRS2,PARP9,APP,PSEN1,CCL5,DDR2,DUSP22,EDN1,AKTIP,DUSP26,NOD2,SH3RF3,MAP3K7,ENSCAFG00000029553,PARK7,CRKL	up_ACOR_VSMC
GO:0045937	0.0393	0.974	40	312	positive regulation of phosphate metabolic process	BP	1	ADRA1B,LEP,MAP3K8,MID1,HTR2B,RASSF2,BMP4,FGFR2,TLR3,SH3RF2,PPARGC1A,PDE5A,AR,SASH1,LMO4,FGF18,F2RL1,FAM129A,NRP1,PTPN22,NPNT,SMPD1,PRKCE,CCNT1,FRS2,PARP9,APP,PSEN1,CCL5,DDR2,DUSP22,EDN1,AKTIP,DUSP26,NOD2,SH3RF3,MAP3K7,ENSCAFG00000029553,PARK7,CRKL	up_ACOR_VSMC
GO:0007389	0.0393	0.978	20	137	pattern specification process	BP	1	HOXA11,EMX2,HOXA7,HOXA9,CDON,AXIN2,HOXD3,DLL1,HOXA6,OSR1,ZBTB16,RARG,HOXC4,IRX3,ZEB1,HOXC10,LRP4,KDM2B,TGFBR2,CITED2	up_VSAP_EC
GO:0010720	0.0393	0.982	12	110	positive regulation of cell development	BP	0.874	ARHGEF2,DPYSL3,HIF1A,CTNNB1,ENSCAFG00000002015,APOE,LYN,MMD,VLDLR,REST,DISC1,NRP1	down_VSAP_EC

GO:0006939	0.0393	0.993	3	32	smooth muscle contraction	BP	1	CHRM2,HTR1D,TBXA2R	up_AFEM_EC
GO:0061013	0.0393	0.993	3	32	regulation of mRNA catabolic process	BP	1	TENT5C,AXIN2,SAMD4B	up_AFEM_EC
GO:0001933	0.039329639	0.98495	8	146	negative regulation of protein phosphorylation	BP	1	PKIB,NDRG2,DUSP26,SMAD6,LRRC15,LRRC4,PRKCD,DBNDD2	up_ACOR_EC
GO:0071322	0.039393046	0.99348	3	29	cellular response to carbohydrate stimulus	BP	1	NR1H4,KCNB1,PRKACA	up_ACOR_EC
GO:0010629	0.0394	0.972	54	502	negative regulation of gene expression	BP	1	ELK3,HMGB1,RNPS1,SREBF1,ILF3,CBX5,ATAD2,HMGA1,EIF4A3,EDC3,HNRNPD,SREBF2,NEDD4,U2AF2,MTDH,SRRT,NAB2,SLC35C2,RAN,BIRC5,CAV1,TIMELESS,THRAP3,FAM129B,EDC4,AURKB,EZH2,GREM1,RNASEH2B,PLK3,HOXA7,SMG9,UHRF1,GMNN,FASTKD3,NOC2L,PPARD,NCL,SUV39H1,HMGB2,DMAP1,CDK2,ENSCAFG00000005299,EZR,RBM14,ERF,TCERG1,ENSCAFG00000015189,HAT1,HIST2H2AC,H2AFX,EXOSC5,SIX4,ANXA7	down_ACOR_VSMC
GO:0030308	0.0394	0.985	9	49	negative regulation of cell growth	BP	1	DCBLD2,GJA1,RERG,CDKN1A,CRLF3,NRP1,ULK2,NAIF1,SH3BP4	up_ACOR_VSMC
GO:0051239	0.0394	0.987	7	859	regulation of multicellular organismal process	BP	1	NR1H4,TBX5,BPI,PLCL1,SLITRK5,NPNT,NTRK2	up_APUL_EC
GO:0060993	0.0394	0.988	6	42	kidney morphogenesis	BP	0.874	ENSCAFG00000032470,ILK,HOXB7,MAGED1,CTNNB1,SIX4	down_VSAP_EC
GO:0086001	0.0394	0.995	3	9	cardiac muscle cell action potential	BP	1	FGF13,KCNE3,JUP	up_ACOR_VSMC
GO:0030032	0.0394	0.999	1	19	lamellipodium assembly	BP	1	FSCN1	down_AFEM_EC
GO:0007200	0.0394	0.999	1	18	phospholipase C-activating G protein-coupled receptor signaling	BP	1	C3AR1	up_VPOR_VSMC
GO:0031128	0.0394	0.999	1	15	developmental induction	BP	1	HOXA11	up_VFEM_VSMC
GO:0048538	0.0394	0.999	1	20	thymus development	BP	1	HOXA3	up_VPUL_VSMC
GO:1905314	0.039471593	0.99652	2	12	semi-lunar valve development	BP	1	GATA4,SMAD6	up_ACOR_EC
GO:2000379	0.0395	0.99	5	20	positive regulation of reactive oxygen species	BP	1	F2RL1,CYBA,ZNF205,ASS1,TGFBR2	up_VSAP_EC
GO:0009892	0.0395	0.998	2	835	negative regulation of metabolic process	BP	1	PLCL1,MOSPD1	down_VFEM_EC
GO:0006002	0.0395	0.999	1	5	fructose 6-phosphate metabolic process	BP	1	FBP1	up_VPOR_EC
GO:2001222	0.0395	0.999	1	11	regulation of neuron migration	BP	1	ARHGEF2	up_VPUL_EC
GO:0010831	0.0395	0.999	1	9	positive regulation of myotube differentiation	BP	1	SHOX2	up_VSAP_VSMC
GO:0010942	0.0396	0.978	18	200	positive regulation of cell death	BP	0.874	CASP3,PTPN2,MAGED1,CTNNB1,LYN,BNIP3,DDIT3,FAM162A,ATF3,GSK3B,SH3GLB1,HTT,REST,EIF2S1,PAK2,INHBA,CDKN1A,SPINK2	down_VSAP_EC
GO:0031076	0.0396	0.992	4	14	embryonic camera-type eye development	BP	1	RARG,ZEB1,KDM2B,CITED2	up_VSAP_EC
GO:0035296	0.0396	0.993	3	32	regulation of tube diameter	BP	1	HTR1D,AVPR1A,TBXA2R	up_AFEM_EC
GO:0097746	0.0396	0.993	3	32	regulation of blood vessel diameter	BP	1	HTR1D,AVPR1A,TBXA2R	up_AFEM_EC
GO:0030001	0.0396	0.993	3	172	metal ion transport	BP	1	NETO1,STIM1,CAV1	up_VSAP_VSMC
GO:0045616	0.0396	0.999	1	9	regulation of keratinocyte differentiation	BP	1	HOXA7	down_ACOR_EC
GO:0032386	0.0396	1	1	137	regulation of intracellular transport	BP	1	JUP	down_VFEM_VSMC
GO:0055010	0.0396	0.999	1	15	ventricular cardiac muscle tissue morphogenesis	BP	1	NKX2-5	up_VPOR_VSMC

GO:0006807	0.0397	0.967	290	3221	nitrogen compound metabolic process	BP	1	ELK3,ACSL3,PRPSAP1,HMGB1,MCM6,RNPS1,SREBF1,ACLY,MCM4,POLE3,USP1,IRAK1,MCM7,RPA1,PTBP1,ILF3,DDX39A,CBX5,ENSCAFG00000016095,PPRC1,USP4,SF3B2,BCAT1,ATAD2,HMGA1,EIF4A3,HMGCR,HNRNPUL1,EDC3,NNT,HNRNPD,SREBF2,MCM3,SMC1A,HINFP,VARS,UBE2S,NEDD4,SRSF2,RRM1,THOP1,MED17,U2AF2,INTS7,PSMD3,POLA1,PSMD7,SLC29A1,MTDH,HEY1,METAP2,PPM1G,RRBP1,SNRPB2,TOP3A,TRIP13,PPP1R15B,SF1,PARPBP,POLA2,MPDU1,SRRT,GINS4,NOP56,RAD54L,POLE,DDX23,NAB2,SLC35C2,MRPS18B,RAN,B3GNT2,PRELID1,HM13,UBE2T,PSMD13,CDCA7L,FUS,RFC2,BIRC5,MYBBP1A,INSIG1,PRMT1,CAV1,CDK1,RFC5,RFC3,RAD51,CASP2,FANCI,XYLT1,FANCD2,U2AF1,TIMELESS,THRAP3,TRUB2,FAM129B,TCEA1,NF2,PFAS,EDC4,AURKB,NONO,MMP14,HNRNPL,TK1,CDKN2C,TOPBP1,EZH2,PPP2R5D,GREM1,MAP3K20,RFC4,NEK2,FBL,FEN1,CDK4,SMARCC1,MND1,RNASEH2B,PLK3,SFSWAP,PCNA,DHX33,HOXA7,SMG9,TONSL,ARG2,MCM10,SF3B4,PHF5A,UHRF1,CKS2,PDCD2,RNF152,DIS3,GMNN,DHCR24,BRCA1,NOC4L,MVD,WDR62,FASTKD3,NOC2L,EIF5A,E2F8,B4GALT1,PRCP,ELOVL1,FARSA,TFRC,DDX51,ENSCAFG00000015065,PPA	down_ACOR_VSMC
GO:1901861	0.0397	0.986	8	41	regulation of muscle tissue development	BP	1	BMP4,FGFR2,GJA1,PPARGC1A,TBX20,TBX5,FRS2,EDN1	up_ACOR_VSMC
GO:0006928	0.0397	0.99	5	557	movement of cell or subcellular component	BP	1	TBX5,CCL24,FLRT3,ARHGEF2,NEO1	up_VPUL_EC
GO:0043009	0.0397	0.997	2	227	chordate embryonic development	BP	1	HOXC6,C6	up_VCAV_VSMC
GO:0007041	0.0397	0.996	2	41	lysosomal transport	BP	1	HOOK3,GCC2	up_VPOR_EC
GO:0007618	0.0397	0.999	1	5	mating	BP	1	ACVR2A	up_VPOR_EC
GO:0007613	0.0397	0.999	1	20	memory	BP	1	LRRN4	up_VPUL_VSMC
GO:0050999	0.0397	0.999	1	9	regulation of nitric-oxide synthase activity	BP	1	CAV1	up_VSAP_VSMC
GO:0099601	0.0397	0.999	1	10	regulation of neurotransmitter	BP	1	NETO1	up_VSAP_VSMC
GO:0090316	0.039739463	0.99093	4	48	positive regulation of intracellular protein	BP	1	KCNB1,EDEM2,MAVS,PRKCD	up_ACOR_EC
GO:0006066	0.0398	0.981	14	89	alcohol metabolic process	BP	1	PLCG2,LEP,LCAT,PCBD1,ABCA1,PPIP5K1,LIPE,MOXD1,APP,CLN8,INPP5K,PTS,PARK7,LMF1	up_ACOR_VSMC
GO:0016070	0.0398	0.984	11	1479	RNA metabolic process	BP	1	HOXA11,TBX15,HOXA10,HOXA9,PITX1,EMX2,SALL1,IRX5,TLR2,CAV1,SHOX2	up_VSAP_VSMC
GO:0030239	0.0398	0.991	4	21	myofibril assembly	BP	0.874	ACTN1,WDR1,SIX4,MYBPC2	down_VSAP_EC
GO:0090066	0.0398	0.991	4	162	regulation of anatomical structure size	BP	1	HTR7,SEMA3A,TWF1,RNF6	up_VPOR_EC
GO:0007275	0.0398	1	2	1370	multicellular organism development	BP	1	JUP,GATA5	down_VFEM_VSMC
GO:0051781	0.0398	0.999	1	18	positive regulation of cell division	BP	1	FGF5	up_VPOR_VSMC
GO:0003279	0.039812668	0.99091	4	48	cardiac septum development	BP	1	GATA4,SMAD6,TBX5,NRP1	up_ACOR_EC
GO:0043113	0.039878561	0.99647	2	12	receptor clustering	BP	1	AGRN,LRP4	up_ACOR_EC
GO:0072001	0.0399	0.98	15	104	renal system development	BP	0.822	HOXB7,PDGFB,PODXL,MEF2C,TIPARP,GPR4,GATA3,MTSS1,ODC1,ARG2,BMP2,PROX1,JAG1,BASP1,ITGA6	up_VCAV_EC
GO:0097194	0.0399	0.99	5	23	execution phase of apoptosis	BP	0.822	CASP8,MADD,PAK2,ENSCAFG00000005099,TAOK1	up_VCAV_EC
GO:0006355	0.0399	0.995	3	974	regulation of transcription, DNA-	BP	1	GATA5,TGFBR1,MEIS2	down_VSAP_VSMC
GO:0006335	0.0399	0.998	2	4	DNA replication-dependent nucleosome	BP	1	IPO4,HAT1	down_ACOR_VSMC
GO:0034723	0.0399	0.998	2	4	DNA replication-dependent nucleosome	BP	1	IPO4,HAT1	down_ACOR_VSMC
GO:0001678	0.039952582	0.99335	3	29	cellular glucose homeostasis	BP	1	NR1H4,KCNB1,PRKACA	up_ACOR_EC

GO:1902531	0.04	0.973	43	536	regulation of intracellular signal transduction	BP	0.874	ARHGEF2,VEGFA,P4HB,SIPA1L1,NR1H4,SOCS5,JCAD,HYOU1,PTPN11,TRIB3,FARP1,ILK,FHL2,SESN2,PTGS2,UACA,HIF1A,TNIK,PTPN2,MTDH,CNOT7,MAGED1,CTNNB1,ENSCAFG00000002015,APOE,WWC3,LYN,DDIT3,PLEKHG2,CBLC,ATF3,GSK3B,HTT,ARFGEF3,CALM1,PAK2,INHBA,NRP1,SH3RF3,TNFAIP1,MADD,SEC13,MID1	down_VSAP_EC
GO:0098660	0.04	0.978	20	141	inorganic ion transmembrane transport	BP	1	ACTN2,PLCG2,SLC26A7,KCNE3,HTR2B,PTPN3,CLCN3,MICU2,ITPR1,CACNB3,AKAP6,KCNJ4,PRKCE,CHD7,ATP6V0E1,KCNC4,FXYD1,NDUFA4L2,PARK7,SLC8A1	up_ACOR_VSMC
GO:0043603	0.04	0.994	3	372	cellular amide metabolic process	BP	1	DARS,SUCLG2,EIF3M	down_AFEM_EC
GO:0001706	0.04	0.999	1	20	endoderm formation	BP	1	DUSP2	up_AORT_EC
GO:0009953	0.04	0.999	1	34	dorsal/ventral pattern formation	BP	1	WNT7A	up_AORT_VSMC
GO:0010657	0.04	0.999	1	15	muscle cell apoptotic process	BP	1	NKX2-5	up_VPOR_VSMC
GO:0065008	0.0401	0.97	77	1056	regulation of biological quality	BP	0.874	VEGFA,P4HB,MYH9,SLC39A7,NR1H4,FMNL3,ENSCAFG00000013214,HSPA5,SERPINE1,ERO1A,ENPP1,CASP3,ENSCAFG00000003477,AKAP2,PTPN11,EXOC5,ATP2B1,AARS,LRRC8A,IFRD1,PRCP,SMARCA4,SESN2,PTGS2,WDR1,TMTC2,MSN,KIF5B,HIF1A,HTR7,TUBB,DNAJC10,NXN,PTPN2,NEO1,PLCL1,SMG7,ACTA2,PDIA4,ACTR3,GNAQ,CTNNB1,CRYAB,TFRC,APOE,PLOD3,LYN,BNIP3,STRAP,DDIT3,TWF1,PLEKHG2,TLN1,CLCN3,SLC7A11,GSK3B,CDC37,ITGB3,SH3GLB1,HTT,REST,ABHD5,PRMT1,CALM1,RAB11FIP1,DISC1,RAD23A,KDELR2,LRRTM2,CAPG,INHBA,SIX4,NRP1,ARF4,YWHAH,KDM3A,HOMER1	down_VSAP_EC
GO:0003006	0.0401	0.976	25	180	developmental process involved in reproduction	BP	1	LEP,GATA4,PRDX4,BMP4,FGFR2,ATRX,PDE5A,CITED2,SPINT1,CTSB,EGLN1,ENSCAFG00000006238,SPINT2,PTGDR,MARF1,AFF4,FRS2,CHD7,SPINK2,ARID5B,DMC1,SERPINB5,JAM3,LHX9,WDR48	up_ACOR_VSMC
GO:0010959	0.0401	0.984	9	79	regulation of metal ion transport	BP	0.874	REM1,KIF5B,CTNNB1,LYN,NKAIN1,HTT,CALM1,YWHAH,HOMER1	down_VSAP_EC
GO:0061311	0.0401	0.994	3	9	cell surface receptor signaling pathway involved in heart	BP	0.824	DLL4,BMP2,JAG1	up_VCAV_EC
GO:0048284	0.0401	0.996	2	66	organelle fusion	BP	1	SPG11,STX4	down_ACOR_EC
GO:0036010	0.0401	0.997	2	6	protein localization to endosome	BP	0.874	MSN,NRP1	down_VSAP_EC
GO:0006941	0.0401	0.999	1	36	striated muscle contraction	BP	1	ARG2	up_AORT_VSMC
GO:0003382	0.0401	0.999	1	11	epithelial cell morphogenesis	BP	1	ARHGEF26	up_APUL_EC
GO:0010770	0.0401	0.999	1	29	positive regulation of cell morphogenesis involved in differentiation	BP	1	LIMK1	up_VCAV_VSMC
GO:0071466	0.0401	0.999	1	17	cellular response to xenobiotic stimulus	BP	1	CYP26B1	up_VPOR_VSMC
GO:0032024	0.0401	0.999	1	12	positive regulation of insulin secretion	BP	1	NR1H4	up_VPUL_EC
GO:0035115	0.0401	0.999	1	12	embryonic forelimb morphogenesis	BP	1	TBX5	up_VPUL_EC
GO:0061178	0.0401	0.999	1	12	regulation of insulin secretion involved in cellular response to	BP	1	NR1H4	up_VPUL_EC
GO:0090277	0.0401	0.999	1	12	positive regulation of peptide hormone	BP	1	NR1H4	up_VPUL_EC
GO:0021795	0.0401	0.999	1	20	cerebral cortex cell migration	BP	1	SLIT2	up_VPUL_VSMC
GO:0071560	0.0402	0.982	12	72	cellular response to transforming growth factor beta stimulus	BP	1	SMAD6,FERMT1,CITED2,CDKN2B,ANKRD1,NPNT,FUT8,TGFBR1,NR3C1,DUSP22,HPGD,CRKL	up_ACOR_VSMC
GO:0050866	0.0402	0.985	8	48	negative regulation of cell activation	BP	0.824	ADGRF5,TNFAIP3,PDGFB,PELI1,ARG2,THOC1,CD200,HMGB1	up_VCAV_EC
GO:0030324	0.0402	0.986	7	64	lung development	BP	1	ADAMTS2,BMPR1A,LOX,GLI3,LOXL3,RDH10,THRB	down_VCAV_EC
GO:0044818	0.0402	0.992	4	15	mitotic G2/M transition checkpoint	BP	0.824	TAOK1,BLM,RAD17,TOPBP1	up_VCAV_EC
GO:0032613	0.0402	0.992	4	14	interleukin-10 production	BP	1	CD274,DLL1,F2RL1,CD83	up_VSAP_EC

GO:1903506	0.0402	0.995	3	977	regulation of nucleic acid-templated transcription	BP	1	GATA5,TGFBR1,MEIS2	down_VSAP_VSMC
GO:0045822	0.0402	0.995	3	8	negative regulation of heart contraction	BP	1	KCNE3,PDE5A,RNLS	up_ACOR_VSMC
GO:0048731	0.0402	0.996	3	1247	system development	BP	1	HOXA3,TBX5,HOXB5	up_APUL_VSMC
GO:0051482	0.0402	0.997	2	5	positive regulation of cytosolic calcium ion concentration involved in phospholipase C-activating G protein-	BP	0.824	GPR4,EDN1	up_VCAV_EC
GO:0021563	0.0402	0.998	2	4	glossopharyngeal nerve development	BP	1	HOXD3,PLXNA4	up_VSAP_EC
GO:0045840	0.0403	0.994	3	12	positive regulation of mitotic nuclear division	BP	0.824	PDGFB,EDN1,SMC5	up_VCAV_EC
GO:0071214	0.0403	0.996	2	93	cellular response to abiotic stimulus	BP	1	PTGER2,ARHGEF2	up_VPUL_EC
GO:0104004	0.0403	0.996	2	93	cellular response to environmental stimulus	BP	1	PTGER2,ARHGEF2	up_VPUL_EC
GO:0060039	0.0403	0.999	1	11	pericardium development	BP	1	TBX5	up_APUL_EC
GO:0090161	0.0403	0.999	1	5	Golgi ribbon formation	BP	1	GCC2	up_VPOR_EC
GO:0010830	0.0403	0.999	1	16	regulation of myotube differentiation	BP	1	SHOX2	up_VFEM_VSMC
GO:0002042	0.040311793	0.99326	3	29	cell migration involved in sprouting angiogenesis	BP	1	ENSCAFG00000004331,FOXC2,NRP1	up_ACOR_EC
GO:0023051	0.0404	0.975	31	954	regulation of signaling	BP	1	CMKLR1,LEP,KCNC4,LBH,CRY2,GAS1,DLL1,F3,MLLT11,LMCD1,CHRM2,TMEM100,SLC6A6,PLAGL2,GREM1,FAM53B,CAMK2A,GREM2,CLEC16A,CYLD,POSTN,LRRC15,RECK,BVES,LGR6,RAB7A,AXIN2,CCL5,DMPK,ADAM17,KANK1	up_AFEM_EC
GO:0046632	0.0404	0.988	6	29	alpha-beta T cell differentiation	BP	0.825	IL6,GATA3,MTOR,ITPKB,HMGB1,BMX	up_VCAV_EC
GO:0002688	0.0404	0.989	5	34	regulation of leukocyte chemotaxis	BP	0.874	VEGFA,SERPINE1,ADAM17,MMP28,LYN	down_VSAP_EC
GO:0034123	0.0404	0.994	3	10	positive regulation of toll-like receptor signaling	BP	0.824	PELI1,TLR3,HMGB1	up_VCAV_EC
GO:0033327	0.0404	0.998	2	4	Leydig cell differentiation	BP	1	SF1,SGPL1	down_ACOR_VSMC
GO:0097242	0.0404	0.997	2	7	amyloid-beta clearance	BP	1	LRP1,LRPAP1	down_VCAV_EC
GO:0009225	0.0404	0.999	1	10	nucleotide-sugar metabolic process	BP	1	CSGALNACT1	down_ACOR_EC
GO:0050857	0.0404	0.999	1	9	positive regulation of antigen receptor-mediated signaling	BP	1	LYN	down_ACOR_EC
GO:0045655	0.0404	0.999	1	9	regulation of monocyte differentiation	BP	1	HOXA7	down_ACOR_EC
GO:0003156	0.0404	0.999	1	20	regulation of animal organ formation	BP	1	CITED2	down_VPOR_EC
GO:0048844	0.0404	0.999	1	30	artery morphogenesis	BP	1	EYA1	up_AFEM_VSMC
GO:0060675	0.0404	0.999	1	29	ureteric bud morphogenesis	BP	1	EYA1	up_AFEM_VSMC
GO:0007338	0.0404	0.999	1	16	single fertilization	BP	1	HOXA9	up_AORT_EC
GO:0042116	0.0404	0.999	1	18	macrophage activation	BP	1	BPI	up_AORT_EC
GO:1905939	0.0404	0.999	1	5	regulation of gonad development	BP	1	SEMA3A	up_VPOR_EC
GO:0051094	0.0405	0.972	48	388	positive regulation of developmental process	BP	1	VEGFD,LEP,LBH,GATA5,CD83,FNDC5,BMP4,FGFR2,TP53BP1,GJA1,TLR3,WASF3,PPARGC1A,PDE5A,CITED2,ISG15,CD274,ZNF365,SASH1,FGF18,FAM210B,MARCH5,BNIP3,CLSTN1,NRP1,NPNT,SMARCD3,SLITRK5,TBX20,TBX5,CCDC3,FZD3,DKKL1,FRS2,DAG1,APP,PSEN1,CHD7,JUP,DDR2,EDN1,DISC1,DOCK5,ERAP1,SLC8A1,RREB1,CRKL,PIK3C2A	up_ACOR_VSMC
GO:0090224	0.0405	0.992	4	16	regulation of spindle organization	BP	1	SMC1A,NUP62,SMC3,STAG1	down_ACOR_VSMC
GO:0045665	0.0405	0.991	4	55	negative regulation of neuron differentiation	BP	1	IRX3,DLL1,THRB,KANK1	up_AFEM_EC
GO:2001141	0.0405	0.995	3	979	regulation of RNA biosynthetic process	BP	1	GATA5,TGFBR1,MEIS2	down_VSAP_VSMC
GO:0090596	0.0405	0.996	2	84	sensory organ morphogenesis	BP	1	NTRK2,HOXA1	up_APUL_EC

GO:0070427	0.0405	0.998	2	4	nucleotide-binding oligomerization domain containing 1 signaling	BP	0.826	TNFAIP3,TLR4	up_VCAV_EC
GO:0048538	0.0405	0.999	1	20	thymus development	BP	1	CITED2	down_VPOR_EC
GO:0072657	0.0405	1	1	144	protein localization to membrane	BP	1	JUP	down_VFEM_VSMC
GO:0030155	0.0406	0.976	25	180	regulation of cell adhesion	BP	1	LEP,CD83,MBP,FERMT1,BMP4,PDE5A,CITED2,CD274,NRP1,PTPN22,NPNT,SPINT2,PRKCE,RND1,ECM2,LAMA3,JUP,DUSP22,NID1,DUSP26,FAM107A,DOCK5,RREB1,PAG1,CRKL	up_ACOR_VSMC
GO:0002532	0.0406	0.989	5	25	production of molecular mediator involved in inflammatory response	BP	0.826	GBP5,TLR4,SNAP23,APOD,NOS2	up_VCAV_EC
GO:0090049	0.0406	0.999	1	21	regulation of cell migration involved in sprouting angiogenesis	BP	1	HDAC7	down_VPOR_EC
GO:0002792	0.040696596	0.99317	3	29	negative regulation of peptide secretion	BP	1	NR1H4,KCNB1,PTGER4	up_ACOR_EC
GO:0060322	0.0407	0.975	26	193	head development	BP	1	EMX2,PCDH19,SEZ6L,CDON,OSR2,SLIT2,SLC6A4,ABL1,DLL1,PLXNA4,RARG,CTNS,RTN4RL1,DPYSL2,GDPD5,SMG9,MCPH1,KIF27,KDM2B,RAF1,NFIB,ZFAND5,QARS,HTRA2,MAP2K2,TGFBR2	up_VSAP_EC
GO:0030217	0.0407	0.982	11	65	T cell differentiation	BP	1	ABL1,ZBTB16,STAT6,PATZ1,ZFP36L1,ZEB1,CD83,ZFP36L2,PNP,PPP3CB,TGFBR2	up_VSAP_EC
GO:0021537	0.0407	0.984	9	73	telencephalon development	BP	0.874	HIF1A,UBA6,UCHL5,CTNNB1,GSK3B,EXT1,DISC1,INHBA,NRP1	down_VSAP_EC
GO:0060307	0.0407	0.998	2	4	regulation of ventricular cardiac muscle cell membrane repolarization	BP	1	KCNE3,GJA1	up_ACOR_VSMC
GO:0099622	0.0407	0.998	2	4	cardiac muscle cell membrane repolarization	BP	1	KCNE3,GJA1	up_ACOR_VSMC
GO:0099623	0.0407	0.998	2	4	regulation of cardiac muscle cell membrane	BP	1	KCNE3,GJA1	up_ACOR_VSMC
GO:0099625	0.0407	0.998	2	4	ventricular cardiac muscle cell membrane	BP	1	KCNE3,GJA1	up_ACOR_VSMC
GO:0007276	0.0407	0.996	2	130	gamete generation	BP	1	HOXA9,HOXA11	up_VFEM_VSMC
GO:0010972	0.0407	0.999	1	18	negative regulation of G2/M transition of mitotic	BP	1	TAOK1	up_AORT_EC
GO:0055021	0.0407	0.999	1	15	regulation of cardiac muscle tissue growth	BP	1	NKX2-5	up_VPOR_VSMC
GO:0018105	0.0408	0.979	16	116	peptidyl-serine phosphorylation	BP	0.83	PLK2,HCLS1,PRKCH,PDGFB,PDE4D,MTOR,MAPK13,PAK2,PRKD2,NCK1,NSD1,PTGS2,TOP1,SPTBN4,RIPK1,LMTK2	up_VCAV_EC
GO:0071495	0.0408	0.989	5	365	cellular response to endogenous stimulus	BP	1	ACVRL1,IGF1R,SULF1,ASPN,NEDD4	down_ACOR_EC
GO:0001837	0.0408	0.999	1	48	epithelial to mesenchymal transition	BP	1	TGFBR1	down_VSAP_VSMC
GO:0045927	0.0408	0.999	1	61	positive regulation of growth	BP	1	TBX5	up_APUL_VSMC
GO:0042088	0.0408	0.999	1	12	T-helper 1 type immune response	BP	1	IL18BP	up_VPUL_EC
GO:0036295	0.0408	0.999	1	9	cellular response to increased oxygen levels	BP	1	CAV1	up_VSAP_VSMC
GO:0036296	0.0408	0.999	1	9	response to increased oxygen levels	BP	1	CAV1	up_VSAP_VSMC
GO:0010941	0.0409	0.972	41	524	regulation of cell death	BP	0.874	ARHGEF2,VEGFA,P4HB,ASNS,NR1H4,PLK3,HSPA5,SERPINE1,CASP3,HYOU1,AARS,FHL2,PTGS2,HIF1A,TNIK,ZPR1,PTPN2,MTDH,MAGED1,CTNNB1,CRYAB,APOE,LYN,BNIP3,DDIT3,FAM162A,ATF3,GSK3B,SH3GLB1,HTT,REST,EIF2S1,GLO1,PAK2,INHBA,SIX4,CDKN1A,NRP1,SPINK2,ARF4,MADD	down_VSAP_EC
GO:0030334	0.0409	0.977	20	267	regulation of cell migration	BP	1	PDGFRB,DCN,SDC4,GLIPR2,BMPR1A,LMO4,RECK,RHOD,AKT2,DOCK10,SYDE1,LAMA3,SEMA3C,THY1,SASH1,SPRED1,TWIST2,DDR2,RAC2,FOXO3	down_VCAV_EC
GO:0010833	0.0409	0.988	6	30	telomere maintenance via telomere lengthening	BP	1	SLC29A1,RAD51,AURKB,NEK2,TINF2,CCT4	down_ACOR_VSMC
GO:0032410	0.0409	0.991	4	17	negative regulation of transporter activity	BP	1	NEDD4,CAV1,GSTO1,REM1	down_ACOR_VSMC
GO:0042982	0.0409	0.994	3	15	amyloid precursor protein metabolic process	BP	1	PRNP,NECAB3,SOAT1	down_VCAV_EC
GO:0030036	0.0409	0.993	3	223	actin cytoskeleton organization	BP	1	MYBPC2,CCL24,ARHGEF2	up_VPUL_EC
GO:0000077	0.0409	0.996	2	60	DNA damage checkpoint	BP	1	USP28,ATRIP	down_ACOR_EC

GO:0031579	0.0409	0.997	2	6	membrane raft organization	BP	0.83	MALL,CAV2	up_VCAV_EC
GO:0006188	0.0409	0.999	1	7	IMP biosynthetic process	BP	1	TNRC6B	down_ACOR_EC
GO:0021602	0.0409	0.999	1	16	cranial nerve morphogenesis	BP	1	HOXA3	up_VPOR_VSMC
GO:2001273	0.0409	0.999	1	12	regulation of glucose import in response to	BP	1	NR1H4	up_VPUL_EC
GO:0001932	0.041	0.974	29	416	regulation of protein phosphorylation	BP	1	NOTCH2,PDGFRB,KIRREL1,DCN,PRNP,GLIPR2,BGN,BMPR1A,CLIP3,AKT1S1,AKT2,EEF2K,PLCL1,CSPG4,ASPN,THY1,PARK7,MYDGF,SASH1,ARL2BP,MAVS,CDH2,GSDME,SPRED1,MAPRE3,DDR2,SHC1,FBH1,GREM1	down_VCAV_EC
GO:0009792	0.041	0.996	2	231	embryo development ending in birth or egg	BP	1	HOXC6,C6	up_VCAV_VSMC
GO:0050878	0.041	0.996	2	75	regulation of body fluid levels	BP	1	CAV1,F5	up_VSAP_VSMC
GO:0043001	0.041	0.999	1	11	Golgi to plasma membrane protein	BP	1	ACSL3	down_ACOR_EC
GO:0000381	0.041	0.999	1	19	regulation of alternative mRNA splicing, via	BP	1	SRSF6	down_AFEM_EC
GO:0030812	0.041	0.999	1	5	negative regulation of nucleotide catabolic	BP	1	FBP1	up_VPOR_EC
GO:0045820	0.041	0.999	1	5	negative regulation of glycolytic process	BP	1	FBP1	up_VPOR_EC
GO:0048846	0.041	0.999	1	6	axon extension involved in axon guidance	BP	1	SEMA3A	up_VPOR_EC
GO:0051195	0.041	0.999	1	5	negative regulation of cofactor metabolic	BP	1	FBP1	up_VPOR_EC
GO:0051198	0.041	0.999	1	5	negative regulation of coenzyme metabolic	BP	1	FBP1	up_VPOR_EC
GO:1902284	0.041	0.999	1	6	neuron projection extension involved in neuron projection	BP	1	SEMA3A	up_VPOR_EC
GO:0003007	0.041011962	0.98679	6	94	heart morphogenesis	BP	1	GATA4,SOX9,SMAD6,FOXC2,TBX5,NRP1	up_ACOR_EC
GO:0046887	0.0411	0.991	4	18	positive regulation of hormone secretion	BP	0.833	EDN1,CREB1,MCU,HIF1A	up_VCAV_EC
GO:0001936	0.0411	0.999	1	49	regulation of endothelial cell proliferation	BP	1	TGFBR1	down_VSAP_VSMC
GO:0002931	0.0411	0.999	1	9	response to ischemia	BP	1	CAV1	up_VSAP_VSMC
GO:0060415	0.0412	0.988	6	27	muscle tissue morphogenesis	BP	1	TNNT2,FGFR2,ANKRD1,EGLN1,TBX20,CHD7	up_ACOR_VSMC
GO:0045682	0.0412	0.988	6	27	regulation of epidermis development	BP	1	HOXA7,DLL1,HPSE,ZFP36L1,TRADD,KEAP1	up_VSAP_EC
GO:0000271	0.0412	0.991	4	22	polysaccharide biosynthetic process	BP	0.874	ENPP1,ENSCAFG00000032470,PPP1R3C,EXT1	down_VSAP_EC
GO:0032623	0.0412	0.999	1	21	interleukin-2 production	BP	1	HDAC7	down_VPOR_EC
GO:0070227	0.0413	0.989	5	25	lymphocyte apoptotic process	BP	0.833	IL7R,ARG2,BCL10,RIPK1,HIF1A	up_VCAV_EC
GO:0019915	0.0413	0.991	4	22	lipid storage	BP	0.874	ENPP1,PTPN2,APOE,ABHD5	down_VSAP_EC
GO:0043299	0.0413	0.991	4	18	leukocyte degranulation	BP	0.833	KIT,RAB27A,SNAP23,FES	up_VCAV_EC
GO:0061551	0.0413	0.997	2	5	trigeminal ganglion development	BP	0.874	SIX4,NRP1	down_VSAP_EC
GO:0048608	0.0413	0.996	2	129	reproductive structure development	BP	1	HOXA9,HOXA11	up_VFEM_VSMC
GO:0006044	0.0413	0.999	1	5	N-acetylglucosamine metabolic process	BP	1	OGA	up_VPOR_EC
GO:2000142	0.0413	0.999	1	17	regulation of DNA-templated transcription,	BP	1	NKX2-5	up_VPOR_VSMC
GO:0006620	0.041373906	0.99957	1	2	posttranslational protein targeting to endoplasmic reticulum membrane	BP	1	CHMP4B	up_ACOR_EC
GO:0050801	0.0414	0.975	27	200	ion homeostasis	BP	1	ADRA1B,PLCG2,SLC26A7,HTR2B,CNNM2,ABCA1,GJA1,PTPN3,MICU2,ITPR1,F2RL1,EGLN1,DMPK,ATP7A,PRKCE,APP,PSEN1,CHD7,DMXL1,EDN1,B2M,DISC1,HERPUD1,WFS1,ATP2C1,SLC8A1,STC2	up_ACOR_VSMC
GO:1902904	0.0414	0.986	7	38	negative regulation of supramolecular fiber	BP	0.833	ARHGAP28,TMOD1,STMN1,SWAP70,TAOK1,TMOD3,ARHGEF2	up_VCAV_EC
GO:0048522	0.0414	0.991	5	1484	positive regulation of cellular process	BP	1	SLITRK5,LIMK1,GATA5,CA2,C6	up_VCAV_VSMC
GO:0030890	0.0414	0.994	3	9	positive regulation of B cell proliferation	BP	0.833	MEF2C,TLR4,PELI1	up_VCAV_EC
GO:0072567	0.0414	0.997	2	4	chemokine (C-X-C motif) ligand 2 production	BP	1	MBP,F2RL1	up_ACOR_VSMC

GO:0001782	0.0414	0.999	1	8	B cell homeostasis	BP	1	LYN	down_ACOR_EC
GO:0001947	0.0414	0.999	1	21	heart looping	BP	1	CITED2	down_VPOR_EC
GO:0061371	0.0414	0.999	1	21	determination of heart left/right asymmetry	BP	1	CITED2	down_VPOR_EC
GO:0008206	0.0414	0.999	1	11	bile acid metabolic process	BP	1	NR1H4	up_APUL_EC
GO:0036445	0.0414	0.999	1	6	neuronal stem cell division	BP	1	ARHGEF2	up_VPOR_EC
GO:0055057	0.0414	0.999	1	6	neuroblast division	BP	1	ARHGEF2	up_VPOR_EC
GO:0090175	0.0414	0.999	1	15	regulation of establishment of planar	BP	1	ANKRD6	up_VPOR_VSMC
GO:0055023	0.0414	0.999	1	12	positive regulation of cardiac muscle tissue	BP	1	TBX5	up_VPUL_EC
GO:0044262	0.0415	0.982	10	89	cellular carbohydrate metabolic process	BP	0.874	ENPP1,TPI1,ENSCAFG00000032470,PGAM1, SESN2,SLC45A3,PTPN2,PPP1R3C,GSK3B,EXT1	down_VSAP_EC
GO:0007263	0.0415	0.994	3	8	nitric oxide mediated signal transduction	BP	0.833	GUCY1A1,GUCY1B1,NOS2	up_VCAV_EC
GO:0071863	0.0415	0.997	2	4	regulation of cell proliferation in bone	BP	0.833	MEF2C,MAP3K3	up_VCAV_EC
GO:0071864	0.0415	0.997	2	4	positive regulation of cell proliferation in bone	BP	0.833	MEF2C,MAP3K3	up_VCAV_EC
GO:0051146	0.0415	0.996	2	76	striated muscle cell differentiation	BP	1	STIM1,SHOX2	up_VSAP_VSMC
GO:0008306	0.0415	0.999	1	21	associative learning	BP	1	LRRN4	up_VPUL_VSMC
GO:0010596	0.0415	0.999	1	21	negative regulation of endothelial cell migration	BP	1	SLIT2	up_VPUL_VSMC
GO:2000027	0.0416	0.981	12	72	regulation of animal organ morphogenesis	BP	1	GATA5,MLLT3,BMP4,FGFR2,SEC24B,CITED2, ZNRF3,LIMS2,TBX5,FZD3,FRS2,TNFRSF11B	up_ACOR_VSMC
GO:0030326	0.0416	0.987	6	51	embryonic limb morphogenesis	BP	1	BMPR1A,RECK,GLI3,RDH10,GREM1,ZBTB16	down_VCAV_EC
GO:0035113	0.0416	0.987	6	51	embryonic appendage morphogenesis	BP	1	BMPR1A,RECK,GLI3,RDH10,GREM1,ZBTB16	down_VCAV_EC
GO:0002685	0.0416	0.99	4	55	regulation of leukocyte migration	BP	1	CMKLR1,HOXA7,CCL5,ADAM17	up_AFEM_EC
GO:0050853	0.0416	0.994	3	10	B cell receptor signaling pathway	BP	0.834	PRKCH,MEF2C,PLCG2	up_VCAV_EC
GO:0022417	0.0416	0.997	2	4	protein maturation by protein folding	BP	1	PRDX4,ERO1B	up_ACOR_VSMC
GO:0060548	0.0416	0.996	2	294	negative regulation of cell death	BP	1	WNT7A,ARG2	up_AORT_VSMC
GO:0060291	0.0416	0.999	1	11	long-term synaptic potentiation	BP	1	NTRK2	up_APUL_EC
GO:0035107	0.0416	0.999	1	60	appendage morphogenesis	BP	1	TBX5	up_APUL_VSMC
GO:0035108	0.0416	0.999	1	60	limb morphogenesis	BP	1	TBX5	up_APUL_VSMC
GO:0031268	0.0416	0.999	1	9	pseudopodium organization	BP	1	CDC42EP5	up_VFEM_EC
GO:0031269	0.0416	0.999	1	9	pseudopodium assembly	BP	1	CDC42EP5	up_VFEM_EC
GO:0035725	0.0416	0.999	1	16	sodium ion transmembrane transport	BP	1	NETO1	up_VFEM_VSMC
GO:0071360	0.0416	0.999	1	9	cellular response to exogenous dsRNA	BP	1	CAV1	up_VSAP_VSMC
GO:0032501	0.0417	0.968	103	1782	multicellular organismal process	BP	1	ADAMTS2,GPAM,NOTCH2,HSPB6,PDGFRB,KIRREL1,SMAD9,LRP1,DCN,NAB2,NDN,ANGPT1,PRNP,LUM,SDC4,VASN,FBN1,ADCY6,GLIPR2,KANK2,THBS3,BMPR1A,LOX,VKORC1,LMO4,EVC,RECK,TRPV2,ANPEP,COL6A1,NUMBL,CHID1,NAGLU,CHRDL1,TBX15,AKT2,LCAT,TFE3,ENSCAFG00000018277,SYDE1,HOXC4,LAMA3,CLSTN1,PLCL1,CSPG4,TRIM8,PKD1,ENSCAFG00000013404,SEMA3C,ASPN,LRPAP1,THY1,RNF216,TEAD1,MEA1,HAPLN3,PEX5,TLX2,VCAN,B4GALT1,GLI3,PARK7,EEF2,MAST2,ZFP36L1,MYDGF,SASH1,NCDN,BPI,RNPEP,CYBA,IFT57,MAVS,CDH2,LOXL3,GSDME,CAMK2D,FUT8,ENSCAFG00000017708,SPRED1,RAB7B,IRX5,TSC22D3,UST,GLIS2,TWIST2,PRKCSH,DDR2,SHC1,RDH10,PCSK6,OMD,GREM1,NAPEPLD,CLIC4,FOXO3,SOAT1,THRB,ZBTB16,ANKRD54,RGMA,SEC61A1,BRSK1	down_VCAV_EC

GO:0032870	0.0417	0.976	23	162	cellular response to hormone stimulus	BP	1	ACTN2,LEP,LBH,SKP2,PRKAA2,BMP4,MED14,LATS2,AR,FAM210B,ADCY6,CTSB,CREBRF,PTGDR,SESN3,PRKCE,AGRN,NR3C1,FAM107A,DDIT4,GNAQ,INPP5K,PARK7	up_ACOR_VSMC
GO:2000027	0.0417	0.981	12	72	regulation of animal organ morphogenesis	BP	1	HOXA11,FZD1,THRB,WNT11,LIMS2,FGF7,ABL1,SULF2,NTN4,NFIB,TGFBR2,CITED2	up_VSAP_EC
GO:0048738	0.0417	0.982	10	64	cardiac muscle tissue development	BP	0.834	ADAMTS9,MEF2C,CHD7,DLL4,EDN1,MTOR,BMP2,PROX1,ENSCAFG00000010596,CREB1	up_VCAV_EC
GO:0042982	0.0417	0.991	4	15	amyloid precursor protein metabolic process	BP	1	ITM2C,PSEN2,APP,PSEN1	up_ACOR_VSMC
GO:0006396	0.0417	0.993	3	368	RNA processing	BP	1	IVNS1ABP,SRSF6,TSEN15	down_AFEM_EC
GO:0060676	0.0417	0.997	2	6	ureteric bud formation	BP	0.874	ENSCAFG00000032470,SIX4	down_VSAP_EC
GO:0061162	0.0417	0.997	2	6	establishment of monopolar cell polarity	BP	0.874	FSCN1,MSN	down_VSAP_EC
GO:0061339	0.0417	0.997	2	6	establishment or maintenance of	BP	0.874	FSCN1,MSN	down_VSAP_EC
GO:0034122	0.0417	0.999	1	9	negative regulation of toll-like receptor signaling	BP	1	LYN	down_ACOR_EC
GO:2000279	0.0417	0.999	1	9	negative regulation of DNA biosynthetic process	BP	1	ACVRL1	down_ACOR_EC
GO:0021800	0.0417	0.999	1	6	cerebral cortex tangential migration	BP	1	NRP2	down_ACOR_EC
GO:0015718	0.0417	0.999	1	30	monocarboxylic acid transport	BP	1	SLC16A12	up_AFEM_VSMC
GO:0007569	0.0417	0.999	1	36	cell aging	BP	1	ARG2	up_AORT_VSMC
GO:0042742	0.0417	0.999	1	39	defense response to bacterium	BP	1	ARG2	up_AORT_VSMC
GO:0006616	0.0417	0.999	1	5	SRP-dependent cotranslational protein targeting to membrane,	BP	1	SRP54	up_VPOR_EC
GO:0046461	0.0417	0.999	1	5	neutral lipid catabolic process	BP	1	ABHD5	up_VPOR_EC
GO:0046464	0.0417	0.999	1	5	acylglycerol catabolic process	BP	1	ABHD5	up_VPOR_EC
GO:1903034	0.0418	0.986	7	40	regulation of response to wounding	BP	0.834	SELP,PDGFB,CD34,KLKB1,TLR4,TFPI,MTOR	up_VCAV_EC
GO:0090184	0.0418	0.993	3	14	positive regulation of kidney development	BP	0.874	HOXB7,MAGED1,SIX4	down_VSAP_EC
GO:0090306	0.0418	0.997	2	4	spindle assembly involved in meiosis	BP	1	FBXO5,ASPM	down_ACOR_VSMC
GO:0006940	0.0418	0.996	2	14	regulation of smooth muscle contraction	BP	1	CHRM2,TBXA2R	up_AFEM_EC
GO:0043269	0.0418	0.996	2	135	regulation of ion transport	BP	1	HOXA3,NKX2-5	up_VPOR_VSMC
GO:0043500	0.0418	0.999	1	21	muscle adaptation	BP	1	GATA5	up_VPUL_VSMC
GO:2000736	0.0418	0.999	1	21	regulation of stem cell differentiation	BP	1	TBX5	up_VPUL_VSMC
GO:0002528	0.04185417	0.99956	1	2	regulation of vascular permeability involved in acute inflammatory	BP	1	CLSTN1	up_ACOR_EC
GO:0065008	0.041889943	0.97273	37	1056	regulation of biological quality	BP	1	CNTNAP4,CDH3,NR1H4,TMOD1,BRSK1,PLCL1,PKIB,CALY,SOX9,RHOU,PTH1R,PCSK1N,AQP1,PORCN,KCNB1,IFI6,NOD2,INSYN1,PLS1,ACTN2,RNLS,PAK3,USP53,MAPK8IP2,FOXC2,LRP4,CLSTN1,LRRC4,CLU,SLC9A9,CHMP4B,TP53INP2,TMTC2,PRKACA,NRP1,PRKCD,PM20D1	up_ACOR_EC
GO:0016072	0.0419	0.979	14	102	rRNA metabolic process	BP	1	NOP56,FBL,DIS3,NOC4L,NCL,RSL1D1,RRP1,ENSCAFG00000019620,NOP14,NOP58,ENSCAFG00000015189,EMG1,EXOSC5,NOB1	down_ACOR_VSMC
GO:0050678	0.0419	0.98	12	117	regulation of epithelial cell proliferation	BP	0.874	VEGFA,SULF1,SPARC,JCAD,TGFBR1,ADAM17,MAGED1,CTNNB1,APOE,STRAP,NR4A1,SIX4	down_VSAP_EC
GO:1902905	0.0419	0.981	12	72	positive regulation of supramolecular fiber	BP	1	SYNPO2L,ACTN2,SYNPO2,F2RL1,JMY,NRP1,EVL,PRKCE,APP,PSEN1,EDN1,WASL	up_ACOR_VSMC
GO:0000723	0.0419	0.983	9	54	telomere maintenance	BP	1	RPA1,HNRNPD,SLC29A1,RAD51,AURKB,NEK2,SMC6,TINF2,CCT4	down_ACOR_VSMC
GO:0032200	0.0419	0.983	9	54	telomere organization	BP	1	RPA1,HNRNPD,SLC29A1,RAD51,AURKB,NEK2,SMC6,TINF2,CCT4	down_ACOR_VSMC
GO:2001237	0.0419	0.986	7	33	negative regulation of extrinsic apoptotic	BP	1	BMP4,AR,DAPK1,NRP1,UNC5B,SH3RF1,PARK7	up_ACOR_VSMC
GO:0048534	0.0419	0.99	4	262	hematopoietic or lymphoid organ	BP	1	LYN,HOXA3,PDPN,HOXA7	down_ACOR_EC
GO:0050879	0.0419	0.991	4	16	multicellular organismal movement	BP	0.834	MTOR,KCNJ2,VPS54,ATP2A1	up_VCAV_EC

GO:0050881	0.0419	0.991	4	16	musculoskeletal movement	BP	0.834	MTOR,KCNJ2,VPS54,ATP2A1	up_VCAV_EC
GO:0080134	0.0419	0.991	4	386	regulation of response to stress	BP	1	NR1H4,CCL24,PTGER2,ARHGEF2	up_VPUL_EC
GO:1902745	0.0419	0.994	3	9	positive regulation of lamellipodium	BP	0.834	MTOR,NCKAP1,FSCN1	up_VCAV_EC
GO:0060612	0.0419	0.996	2	14	adipose tissue development	BP	1	LEP,PPARGC1A	up_AFEM_EC
GO:0045944	0.0419	1	1	287	positive regulation of transcription by RNA polymerase II	BP	1	GATA5	down_AFEM_VSMC
GO:0001889	0.0419	0.999	1	22	liver development	BP	1	CITED2	down_VPOR_EC
GO:0071417	0.0419	1	1	142	cellular response to organonitrogen	BP	1	JUP	down_VFEM_VSMC
GO:0001935	0.0419	0.999	1	50	endothelial cell proliferation	BP	1	TGFBR1	down_VSAP_VSMC
GO:0072171	0.0419	0.999	1	30	mesonephric tubule morphogenesis	BP	1	EYA1	up_AFEM_VSMC
GO:0021800	0.0419	0.999	1	6	cerebral cortex tangential migration	BP	1	SEMA3A	up_VPOR_EC
GO:0016925	0.0419	0.999	1	16	protein sumoylation	BP	1	EYA1	up_VFEM_VSMC
GO:0014812	0.0419	0.999	1	21	muscle cell migration	BP	1	SLIT2	up_VPUL_VSMC
GO:0007178	0.042	0.982	10	107	transmembrane receptor protein serine/threonine kinase signaling pathway	BP	1	SMAD9,VASN,FBN1,BMPR1A,CHRDL1,ASPN,FUT8,ENSCAFG00000017708,GREM1,RGMA	down_VCAV_EC
GO:0042127	0.042	0.989	5	452	regulation of cell proliferation	BP	1	EYA1,SIX1,MAB21L1,TIAM1,MTSS1	up_VFEM_EC
GO:0051851	0.042	0.991	4	23	modification by host of symbiont morphology or	BP	0.874	SMARCA4,APOE,REST,CALM1	down_VSAP_EC
GO:0031570	0.042	0.996	2	61	DNA integrity checkpoint	BP	1	USP28,ATRIP	down_ACOR_EC
GO:0021563	0.042	0.997	2	4	glossopharyngeal nerve development	BP	1	HOXA3,NAV2	down_ACOR_VSMC
GO:0009719	0.042	0.997	2	395	response to endogenous stimulus	BP	1	GATA5,TGFBR1	down_VSAP_VSMC
GO:0098911	0.042	0.997	2	4	regulation of ventricular cardiac muscle cell action	BP	1	KCNE3,JUP	up_ACOR_VSMC
GO:0001667	0.042	1	1	146	ameboidal-type cell migration	BP	1	JUP	down_VFEM_VSMC
GO:0031111	0.042	0.999	1	11	negative regulation of microtubule polymerization or	BP	1	TUBB4A	up_APUL_EC
GO:0055013	0.042	0.999	1	17	cardiac muscle cell development	BP	1	NKX2-5	up_VPOR_VSMC
GO:1901099	0.042	0.999	1	10	negative regulation of signal transduction in	BP	1	EYA1	up_VFEM_EC
GO:2001240	0.042	0.999	1	10	negative regulation of extrinsic apoptotic signaling pathway in	BP	1	EYA1	up_VFEM_EC
GO:0010771	0.042	0.999	1	21	negative regulation of cell morphogenesis involved in differentiation	BP	1	SLIT2	up_VPUL_VSMC
GO:0048640	0.042	0.999	1	21	negative regulation of developmental growth	BP	1	TBX5	up_VPUL_VSMC
GO:0072507	0.0421	0.979	14	103	divalent inorganic cation homeostasis	BP	0.836	ATP2B1,PDE4D,CHD7,GPR4,STC1,EDN1,APLNR,SLC39A8,PDZD8,SWAP70,PLCG2,HMGB1,MCU,ATP2A1	up_VCAV_EC
GO:0060512	0.0421	0.991	4	14	prostate gland morphogenesis	BP	1	BMP4,FGFR2,FRS2,SERPINB5	up_ACOR_VSMC
GO:0018195	0.0421	0.997	2	6	peptidyl-arginine modification	BP	0.874	NR1H4,PRMT1	down_VSAP_EC
GO:0046888	0.0421	0.996	2	14	negative regulation of hormone secretion	BP	1	LEP,CRY2	up_AFEM_EC
GO:0060872	0.0421	0.999	1	6	semicircular canal development	BP	1	HOXA1	down_ACOR_EC
GO:0001570	0.0421	0.999	1	37	vasculogenesis	BP	1	WNT7A	up_AORT_VSMC
GO:0060045	0.0421	0.999	1	11	positive regulation of cardiac muscle cell	BP	1	TBX5	up_APUL_EC
GO:0048641	0.0421	0.999	1	16	regulation of skeletal muscle tissue	BP	1	CYP26B1	up_VPOR_VSMC
GO:0042157	0.0422	0.983	9	51	lipoprotein metabolic process	BP	1	LCAT,ABCA1,PIGN,PIGS,PIGG,APOL6,LYPLAL1,PIK3C3,ZDHHC21	up_ACOR_VSMC
GO:0034968	0.0422	0.986	7	34	histone lysine methylation	BP	0.836	IWS1,GATA3,ARID4B,NSD1,KDM3A,ATRX,TET2	up_VCAV_EC
GO:0033198	0.0422	0.994	3	10	response to ATP	BP	0.836	P2RX7,SELL,P2RX2	up_VCAV_EC

GO:0010452	0.0422	0.998	2	3	histone H3-K36 methylation	BP	0.836	IWS1,NSD1	up_VCAV_EC
GO:0010575	0.0422	0.999	1	8	positive regulation of vascular endothelial growth factor production	BP	1	SULF1	down_ACOR_EC
GO:0035914	0.0422	0.999	1	22	skeletal muscle cell differentiation	BP	1	CITED2	down_VPOR_EC
GO:0071772	0.0422	0.999	1	50	response to BMP	BP	1	GATA5	down_VSAP_VSMC
GO:0071773	0.0422	0.999	1	50	cellular response to BMP stimulus	BP	1	GATA5	down_VSAP_VSMC
GO:2001275	0.0422	0.999	1	11	positive regulation of glucose import in response to insulin	BP	1	NR1H4	up_APUL_EC
GO:0031397	0.0422	0.999	1	30	negative regulation of protein ubiquitination	BP	1	LIMK1	up_VCAV_VSMC
GO:0002703	0.0423	0.984	8	47	regulation of leukocyte mediated immunity	BP	0.836	IL7R,TLR4,GATA3,NECTIN2,THOC1,TLR3,FES,HMGB1	up_VCAV_EC
GO:0009888	0.0423	0.991	4	545	tissue development	BP	1	EYA1,SPINK5,HOXA11,SHOX2	up_VFEM_VSMC
GO:0043487	0.0423	0.993	3	33	regulation of RNA stability	BP	1	TENT5C,AXIN2,SAMD4B	up_AFEM_EC
GO:0047497	0.0423	0.997	2	5	mitochondrion transport along microtubule	BP	0.874	HIF1A,TRAK1	down_VSAP_EC
GO:0034643	0.0423	0.997	2	5	establishment of mitochondrion localization, microtubule-	BP	0.874	HIF1A,TRAK1	down_VSAP_EC
GO:1901857	0.0423	0.997	2	4	positive regulation of cellular respiration	BP	1	PPARGC1A,PARK7	up_ACOR_VSMC
GO:0061458	0.0423	0.996	2	131	reproductive system development	BP	1	HOXA9,HOXA11	up_VFEM_VSMC
GO:0022012	0.0423	1	1	1	subpallium cell proliferation in forebrain	BP	1	GLI3	down_VCAV_EC
GO:0022018	0.0423	1	1	1	lateral ganglionic eminence cell	BP	1	GLI3	down_VCAV_EC
GO:0001869	0.0423	1	1	1	negative regulation of complement activation, lectin pathway	BP	1	A2M	down_VCAV_EC
GO:0035582	0.0423	1	1	1	sequestering of BMP in extracellular matrix	BP	1	FBN1	down_VCAV_EC
GO:0035583	0.0423	1	1	1	sequestering of TGFbeta in extracellular matrix	BP	1	FBN1	down_VCAV_EC
GO:0040009	0.0423	1	1	1	regulation of growth rate	BP	1	NOTCH2	down_VCAV_EC
GO:0045967	0.0423	1	1	1	negative regulation of growth rate	BP	1	NOTCH2	down_VCAV_EC
GO:0002921	0.0423	1	1	1	negative regulation of humoral immune	BP	1	A2M	down_VCAV_EC
GO:0060366	0.0423	1	1	1	lambdoid suture morphogenesis	BP	1	GLI3	down_VCAV_EC
GO:0060367	0.0423	1	1	1	sagittal suture morphogenesis	BP	1	GLI3	down_VCAV_EC
GO:0060981	0.0423	1	1	1	cell migration involved in coronary angiogenesis	BP	1	PDGFRB	down_VCAV_EC
GO:0071692	0.0423	1	1	1	protein localization to extracellular region	BP	1	FBN1	down_VCAV_EC
GO:0071694	0.0423	1	1	1	maintenance of protein location in extracellular	BP	1	FBN1	down_VCAV_EC
GO:0097118	0.0423	1	1	1	neuroligin clustering involved in postsynaptic	BP	1	CDH2	down_VCAV_EC
GO:0001868	0.0423	1	1	1	regulation of complement activation, lectin pathway	BP	1	A2M	down_VCAV_EC
GO:0045916	0.0423	1	1	1	negative regulation of complement activation	BP	1	A2M	down_VCAV_EC
GO:0060873	0.0423	1	1	1	anterior semicircular canal development	BP	1	GLI3	down_VCAV_EC
GO:0060875	0.0423	1	1	1	lateral semicircular canal development	BP	1	GLI3	down_VCAV_EC
GO:0070444	0.0423	1	1	1	oligodendrocyte progenitor proliferation	BP	1	CDH2	down_VCAV_EC
GO:0070445	0.0423	1	1	1	regulation of oligodendrocyte	BP	1	CDH2	down_VCAV_EC
GO:2000258	0.0423	1	1	1	negative regulation of protein activation cascade	BP	1	A2M	down_VCAV_EC
GO:0048489	0.0423	0.999	1	38	synaptic vesicle transport	BP	1	WNT7A	up_AORT_VSMC
GO:0097480	0.0423	0.999	1	38	establishment of synaptic vesicle localization	BP	1	WNT7A	up_AORT_VSMC
GO:0042481	0.0423	0.999	1	5	regulation of odontogenesis	BP	1	ASPN	up_VPOR_EC

GO:0021545	0.0423	0.999	1	21	cranial nerve development	BP	1	HOXA3	up_VPUL_VSMC
GO:0003337	0.0423	0.999	1	10	mesenchymal to epithelial transition involved in metanephros	BP	1	SALL1	up_VSAP_VSMC
GO:0007155	0.0424	0.979	13	324	cell adhesion	BP	1	LEP,CDH18,HOXA7,DLL1,GPAM,GREM1,CYLD,POSTN,ICAM5,BVES,ADAM17,KANK1,TNC	up_AFEM_EC
GO:0050768	0.0424	0.985	7	66	negative regulation of neurogenesis	BP	1	BMPR1A,THY1,TLX2,GLI3,PRKCSH,THRB,RGMA	down_VCAV_EC
GO:0042481	0.0424	0.997	2	5	regulation of odontogenesis	BP	0.838	CD34,BMP2	up_VCAV_EC
GO:0046631	0.0424	0.999	1	35	alpha-beta T cell activation	BP	1	ARG2	up_AORT_VSMC
GO:0007416	0.0424	0.999	1	36	synapse assembly	BP	1	WNT7A	up_AORT_VSMC
GO:0016999	0.0424	0.999	1	30	antibiotic metabolic process	BP	1	CYP1A1	up_VCAV_VSMC
GO:0030316	0.0424	0.999	1	31	osteoclast differentiation	BP	1	LRRC17	up_VCAV_VSMC
GO:0048709	0.0424	0.999	1	17	oligodendrocyte differentiation	BP	1	TRPC4	up_VPOR_VSMC
GO:0086001	0.0424	0.999	1	9	cardiac muscle cell action potential	BP	1	CAV1	up_VSAP_VSMC
GO:0071772	0.042431914	0.99009	4	50	response to BMP	BP	1	SOX9,RGMA,SMAD6,LRP4	up_ACOR_EC
GO:0071773	0.042431914	0.99009	4	50	cellular response to BMP stimulus	BP	1	SOX9,RGMA,SMAD6,LRP4	up_ACOR_EC
GO:0002376	0.0425	0.97	48	622	immune system process	BP	0.874	ARHGEF2,VEGFA,MYH9,NR1H4,SERPINE1,ACTN1,SOCS5,ETV6,ENPP1,CASP3,FAM49B,PTPN11,FLNB,WDR1,MYO1C,MSN,HOXB7,KIF5B,HIF1A,CX3CL1,PTPN2,EIF2B1,EVI2B,PVR,ADAM17,SKAP2,CNOT7,CTNNB1,ANLN,TFRC,MMP28,LYN,BNIP3,CDC37,GBP5,MYO1E,SDC4,REST,PRMT1,SH2B3,GLO1,A2M,INHBA,BTK,SIX4,CDKN1A,TNFAIP1,DEFB124	down_VSAP_EC
GO:0051656	0.0425	0.979	13	129	establishment of organelle localization	BP	0.874	MYH9,SHROOM2,HOOK3,MYO1C,KIF5B,HIF1A,TRAK1,CTNNB1,KPNB1,LYN,GSK3B,HTT,AP3S1	down_VSAP_EC
GO:0042886	0.0425	0.987	6	527	amide transport	BP	1	LYN,PDPN,IPO7,ACSL3,STX4,NEDD4	down_ACOR_EC
GO:0031047	0.0425	0.999	1	33	gene silencing by RNA	BP	1	ESR1	up_AORT_VSMC
GO:0060914	0.0425	0.999	1	11	heart formation	BP	1	TBX5	up_APUL_EC
GO:0051058	0.0425	0.999	1	21	negative regulation of small GTPase mediated signal transduction	BP	1	SLIT2	up_VPUL_VSMC
GO:1901360	0.0426	0.987	8	2016	organic cyclic compound metabolic process	BP	1	IVNS1ABP,EXTL2,DARS,SUCLG2,SRSF6,GABPA,NDUFA10,TSEN15	down_AFEM_EC
GO:0048634	0.0426	0.985	7	40	regulation of muscle organ development	BP	0.838	EPHB1,MEF2C,EDN1,BMP2,CREB1,NR1D2,CDON	up_VCAV_EC
GO:0090190	0.0426	0.994	3	10	positive regulation of branching involved in ureteric bud	BP	1	GREM1,HOXB7,SIX4	down_ACOR_VSMC
GO:0051453	0.0426	0.993	3	33	regulation of intracellular pH	BP	1	RAB20,RAB7A,ATP6V0B	up_AFEM_EC
GO:1905952	0.0426	0.993	3	33	regulation of lipid localization	BP	1	LEP,CRY2,ACACB	up_AFEM_EC
GO:0010869	0.0426	0.997	2	4	regulation of receptor biosynthetic process	BP	0.838	EDN1,HIF1A	up_VCAV_EC
GO:0032800	0.0426	0.997	2	4	receptor biosynthetic process	BP	0.838	EDN1,HIF1A	up_VCAV_EC
GO:0051482	0.0426	0.999	1	5	positive regulation of cytosolic calcium ion concentration involved in phospholipase C-activating G protein-	BP	1	C3AR1	up_VPOR_EC
GO:0002063	0.0426	0.999	1	18	chondrocyte development	BP	1	SHOX2	up_VFEM_VSMC
GO:0061061	0.0427	0.977	17	179	muscle structure development	BP	0.874	PRICKLE1,ACTN1,FLNB,IFRD1,FHL2,WDR1,CTNNB1,CRYAB,KLF5,DDIT3,ATF3,NEXN,REST,HIF1AN,SIX4,MYBPC2,HOMER1	down_VSAP_EC
GO:0032870	0.0427	0.978	16	162	cellular response to hormone stimulus	BP	0.874	NR1H4,ENPP1,PTPN11,TRIB3,PRCP,SMARCA4,MYO1C,MSN,PTPN2,AGRN,GNAQ,UBA5,REST,NR4A1,YWHAH,KDM3A	down_VSAP_EC
GO:0035136	0.0427	0.993	3	16	forelimb morphogenesis	BP	1	RECK,RDH10,ZBTB16	down_VCAV_EC
GO:1904645	0.0427	0.996	2	7	response to amyloid-beta	BP	1	LRP1,PRNP	down_VCAV_EC

GO:0060348	0.0427	0.996	2	78	bone development	BP	1	HOXA11,SHOX2	up_VSAP_VSMC
GO:0002279	0.0427	0.999	1	10	mast cell activation involved in immune	BP	1	LYN	down_ACOR_EC
GO:0002448	0.0427	0.999	1	10	mast cell mediated immunity	BP	1	LYN	down_ACOR_EC
GO:0043303	0.0427	0.999	1	10	mast cell degranulation	BP	1	LYN	down_ACOR_EC
GO:0006000	0.0427	0.999	1	5	fructose metabolic process	BP	1	FBP1	up_VPOR_EC
GO:0042551	0.0427	0.999	1	10	neuron maturation	BP	1	IRX5	up_VSAP_VSMC
GO:0007399	0.0428	0.969	60	564	nervous system development	BP	0.841	EPHB1,EDNRB,PCDH12,PTCHD1,PRKCH,ARHGEF15,IFRD1,MALL,RELN,MEF2C,CHD7,DLL4,RAPGEF1,TLR4,EIF2B2,GATA3,HOXB8,RAB11A,TCF4,NTN4,PPP3CA,SERINC5,B3GNT5,MTOR,NAV2,HOOK3,BMP2,HMG20A,HHEX,PREX2,ENC1,GDPD5,PROX1,ENSCAFG00000010224,STMN1,MMP24,PAK2,APOD,NCK1,FES,JAG1,MMD,RAPGEF2,CREB1,BCL10,ZC4H2,TAOK1,CTNNA1,NUP133,SPTBN4,SKIL,HIF1A,ALS2,ATRX,ALDH1A3,LUZP1,ZEB1,ARHGEF2,CDON,ZNF609	up_VCAV_EC
GO:1903708	0.0428	0.985	7	65	positive regulation of hemopoiesis	BP	1	CLSTN1,GLI3,ZFP36L1,RAB7B,FOXO3,ZBTB16,ANKRD54	down_VCAV_EC
GO:0018212	0.0428	0.988	5	81	peptidyl-tyrosine modification	BP	1	LEP,HCK,GREM1,TPST2,ADAM17	up_AFEM_EC
GO:0016322	0.0428	0.997	2	4	neuron remodeling	BP	1	RND1,APP	up_ACOR_VSMC
GO:1902902	0.0428	0.997	2	4	negative regulation of autophagosome assembly	BP	1	FEZ1,FEZ2	up_ACOR_VSMC
GO:0040007	0.0428	0.996	2	276	growth	BP	1	WNT7A,ESR1	up_AORT_VSMC
GO:0032680	0.0428	0.999	1	36	regulation of tumor necrosis factor production	BP	1	ARG2	up_AORT_VSMC
GO:0046717	0.0428	0.999	1	17	acid secretion	BP	1	TRPC4	up_VPOR_VSMC
GO:1903530	0.0429	0.983	8	165	regulation of secretion by cell	BP	1	LEP,KCNC4,CRY2,GPAM,CHRM2,CAMK2A,RAB7A,CCL5	up_AFEM_EC
GO:0097553	0.0429	0.988	5	32	calcium ion transmembrane import	BP	0.874	ERO1A,LYN,DDIT3,HTT,CALM1	down_VSAP_EC
GO:0048641	0.0429	0.991	4	16	regulation of skeletal muscle tissue	BP	0.842	EPHB1,MEF2C,NR1D2,CDON	up_VCAV_EC
GO:0045638	0.0429	0.993	3	33	negative regulation of myeloid cell	BP	1	HOXA9,HOXA7,DLL1	up_AFEM_EC
GO:0045683	0.0429	0.999	1	9	negative regulation of epidermis development	BP	1	HOXA7	down_ACOR_EC
GO:0071960	0.0429	1	1	1	maintenance of mitotic sister chromatid cohesion,	BP	0.874	NAA10	down_VSAP_EC
GO:1903570	0.0429	1	1	1	regulation of protein kinase D signaling	BP	0.874	VEGFA	down_VSAP_EC
GO:1903572	0.0429	1	1	1	positive regulation of protein kinase D signaling	BP	0.874	VEGFA	down_VSAP_EC
GO:2000718	0.0429	1	1	1	regulation of maintenance of mitotic sister chromatid cohesion, centromeric	BP	0.874	NAA10	down_VSAP_EC
GO:2000719	0.0429	1	1	1	negative regulation of maintenance of mitotic sister chromatid cohesion,	BP	0.874	NAA10	down_VSAP_EC
GO:0051250	0.0429	0.999	1	38	negative regulation of lymphocyte activation	BP	1	ARG2	up_AORT_VSMC
GO:0110020	0.0429	0.999	1	31	regulation of actomyosin structure organization	BP	1	LIMK1	up_VCAV_VSMC
GO:0010759	0.0429	0.999	1	5	positive regulation of macrophage chemotaxis	BP	1	C3AR1	up_VPOR_EC
GO:0032386	0.043	0.976	20	137	regulation of intracellular transport	BP	1	SH3TC2,ACTN2,LEP,TCAF1,FBXL20,FERMT1,BMP4,SEC24B,CDKN1A,PTPN22,OS9,SCP2,PSEN1,JUP,RAB11B,INPP5K,PREPL,PARK7,BVES,PTPN14	up_ACOR_VSMC
GO:0048844	0.043	0.987	6	30	artery morphogenesis	BP	0.842	ADGRF5,ADAMTS9,CHD7,DLL4,PROX1,JAG1	up_VCAV_EC
GO:0045947	0.043	0.997	2	6	negative regulation of translational initiation	BP	0.874	EIF4EBP1,EIF2S1	down_VSAP_EC
GO:0030038	0.043	0.999	1	31	contractile actin filament bundle assembly	BP	1	LIMK1	up_VCAV_VSMC
GO:0043149	0.043	0.999	1	31	stress fiber assembly	BP	1	LIMK1	up_VCAV_VSMC
GO:0021772	0.043	0.999	1	11	olfactory bulb development	BP	1	SALL1	up_VSAP_VSMC

GO:0021988	0.043	0.999	1	11	olfactory lobe development	BP	1	SALL1	up_VSAP_VSMC
GO:0030335	0.0431	0.99	4	165	positive regulation of cell migration	BP	1	C3AR1,ARHGEF2,SEMA3A,CCAR1	up_VPOR_EC
GO:0019395	0.0431	0.993	3	33	fatty acid oxidation	BP	1	LEP,ACACB,PPARGC1A	up_AFEM_EC
GO:0047484	0.0431	0.997	2	5	regulation of response to osmotic stress	BP	0.874	ARHGEF2,PTGS2	down_VSAP_EC
GO:0018401	0.0431	0.997	2	4	peptidyl-proline hydroxylation to 4-	BP	1	EGLN1,P4HA1	up_ACOR_VSMC
GO:0001763	0.0431	0.996	2	78	morphogenesis of a branching structure	BP	1	HOXA11,SHOX2	up_VSAP_VSMC
GO:1901222	0.0431	0.999	1	32	regulation of NIK/NF-kappaB signaling	BP	1	TLR2	up_AFEM_VSMC
GO:0034405	0.0431	0.999	1	11	response to fluid shear stress	BP	1	MTSS1	up_VFEM_EC
GO:0090185	0.0431	0.999	1	10	negative regulation of kidney development	BP	1	SIX1	up_VFEM_EC
GO:0060627	0.0432	0.983	8	165	regulation of vesicle-mediated transport	BP	1	RAB20,GAS1,DLL1,CHRM2,GREM1,CAMK2A,BVES,RAB7A	up_AFEM_EC
GO:0007045	0.0432	0.99	4	23	cell-substrate adherens junction assembly	BP	0.874	VEGFA,ACTN1,ENSCAFG00000002015,NRP1	down_VSAP_EC
GO:0048041	0.0432	0.99	4	23	focal adhesion assembly	BP	0.874	VEGFA,ACTN1,ENSCAFG00000002015,NRP1	down_VSAP_EC
GO:0072698	0.0432	0.993	3	13	protein localization to microtubule cytoskeleton	BP	0.874	HOOK3,GSK3B,MID1	down_VSAP_EC
GO:0051489	0.0432	0.993	3	14	regulation of filopodium assembly	BP	0.874	FSCN1,DPYSL3,NRP1	down_VSAP_EC
GO:1905331	0.0432	0.999	1	9	negative regulation of morphogenesis of an	BP	1	SULF1	down_ACOR_EC
GO:1901016	0.0432	0.999	1	9	regulation of potassium ion transmembrane transporter activity	BP	1	NEDD4	down_ACOR_EC
GO:1902106	0.0432	0.999	1	31	negative regulation of leukocyte differentiation	BP	1	LRRC17	up_VCAV_VSMC
GO:0030878	0.0432	0.999	1	13	thyroid gland development	BP	1	HOXA3	up_VPUL_EC
GO:0048518	0.0433	0.982	12	1676	positive regulation of biological process	BP	1	HOXA11,HOXA9,NETO1,GATM,SALL1,MAB21L1,PRR16,STIM1,TLR2,CAV1,SHOX2,FGF5	up_VSAP_VSMC
GO:0045778	0.0433	0.985	7	35	positive regulation of ossification	BP	1	BMP4,GJA1,ISG15,CLSTN1,NPNT,DDR2,SLC8A1	up_ACOR_VSMC
GO:0003151	0.0433	0.985	7	34	outflow tract morphogenesis	BP	1	SMAD6,BMP4,FGFR2,SEC24B,CITED2,NRP1,TBX20	up_ACOR_VSMC
GO:0044786	0.0433	0.989	5	23	cell cycle DNA replication	BP	1	SMC1A,RAD51,GMNN,E2F8,SMC3	down_ACOR_VSMC
GO:0021795	0.0433	0.989	5	20	cerebral cortex cell migration	BP	1	FGF13,NRP1,PSEN1,DISC1,SUN1	up_ACOR_VSMC
GO:0051289	0.0433	0.988	5	25	protein homotetramerization	BP	0.847	GBP5,CDA,ACPP,KCNJ2,ALDH1A3	up_VCAV_EC
GO:0048598	0.0433	0.993	3	207	embryonic morphogenesis	BP	1	TBX5,HOXA3,HOXA1	up_APUL_EC
GO:0048846	0.0433	0.999	1	6	axon extension involved in axon guidance	BP	1	NRP2	down_ACOR_EC
GO:1902284	0.0433	0.999	1	6	neuron projection extension involved in neuron projection	BP	1	NRP2	down_ACOR_EC
GO:1904377	0.0433	0.999	1	21	positive regulation of protein localization to cell	BP	1	GPSM2	down_AFEM_EC
GO:0048469	0.0433	0.999	1	31	cell maturation	BP	1	IRX5	up_AFEM_VSMC
GO:0120163	0.0433	0.999	1	17	negative regulation of cold-induced	BP	1	HOXC10	up_VFEM_VSMC
GO:0001817	0.0434	0.974	25	182	regulation of cytokine production	BP	1	WNT11,CD274,C5AR2,IL6R,ABL1,HDAC7,DLL1,IL17RC,ANXA4,HPSE,CCBE1,F2RL1,PIK3R1,SULF2,MYD88,NLRX1,TKFC,CD83,RNF216,MAPKBP1,ZNF580,CYBA,PQBP1,FLOT1,MAVS	up_VSAP_EC
GO:0043009	0.0434	0.981	10	227	chordate embryonic development	BP	1	TBX15,HOXA9,IRX5,XYLT1,HOXA7,DLL1,TMEM100,HOXA6,LUZP1,AXIN2	up_AFEM_EC
GO:0007159	0.0434	0.983	8	79	leukocyte cell-cell adhesion	BP	1	GPAM,PRNP,SDC4,THY1,GLI3,LOXL3,RAC2,ZBTB16	down_VCAV_EC
GO:0030323	0.0434	0.985	7	65	respiratory tube development	BP	1	ADAMTS2,BMPR1A,LOX,GLI3,LOXL3,RDH10,THRB	down_VCAV_EC
GO:1905475	0.0434	0.986	6	52	regulation of protein localization to membrane	BP	1	LRP1,PRNP,CLIP3,ENSCAFG00000014978,AKT2,CDH2	down_VCAV_EC
GO:0071398	0.0434	0.991	4	14	cellular response to fatty acid	BP	1	PRKAA2,ADCY6,PTGDR,PRKCE	up_ACOR_VSMC
GO:0060571	0.0434	0.993	3	13	morphogenesis of an epithelial fold	BP	0.874	SULF1,HIF1A,CTNNB1	down_VSAP_EC

GO:0035520	0.0434	0.997	2	4	monoubiquitinated protein deubiquitination	BP	1	USP1,BAP1	down_ACOR_VSMC
GO:0031953	0.0434	0.997	2	6	negative regulation of protein	BP	0.874	ENPP1,ERRFI1	down_VSAP_EC
GO:0003143	0.0434	0.999	1	22	embryonic heart tube morphogenesis	BP	1	CITED2	down_VPOR_EC
GO:0030307	0.0434	0.999	1	31	positive regulation of cell growth	BP	1	LIMK1	up_VCAV_VSMC
GO:0045428	0.0434	0.999	1	10	regulation of nitric oxide biosynthetic process	BP	1	CAV1	up_VSAP_VSMC
GO:0061136	0.0435	0.982	9	78	regulation of proteasomal protein catabolic process	BP	0.874	PRICKLE1,PLK3,SOCS5,TRIB3,UCHL5,GSK3B,RAD23A,TMTC3,USP14	down_VSAP_EC
GO:0046631	0.0435	0.985	7	35	alpha-beta T cell activation	BP	1	CD274,ABL1,ZBTB16,STAT6,CD83,PNP,TGFBR2	up_VSAP_EC
GO:1901673	0.0435	0.994	3	10	regulation of mitotic spindle assembly	BP	1	SMC1A,SMC3,STAG1	down_ACOR_VSMC
GO:0010804	0.0435	0.997	2	6	negative regulation of tumor necrosis factor-mediated signaling	BP	0.874	NR1H4,PTPN2	down_VSAP_EC
GO:0034135	0.0435	0.997	2	4	regulation of toll-like receptor 2 signaling	BP	1	TLR1,F2RL1	up_ACOR_VSMC
GO:0030206	0.0435	0.999	1	9	chondroitin sulfate biosynthetic process	BP	1	CSGALNACT1	down_ACOR_EC
GO:0003158	0.0435	0.999	1	38	endothelium development	BP	1	WNT7A	up_AORT_VSMC
GO:0051058	0.0435	0.999	1	21	negative regulation of small GTPase mediated signal transduction	BP	1	FBP1	up_VFEM_VSMC
GO:0006950	0.0436	0.967	80	1102	response to stress	BP	0.874	ARHGEF2,VEGFA,P4HB,ASNS,PLOD2,NR1H4,PLK3,ENSCAFG00000013214,HSPA5,SERPINE1,SOCS5,HM13,SGK1,ERO1A,HSP90B1,CBX5,CASP3,ENSCAFG00000003477,HYOU1,PGK1,NUAK1,PTPN11,FLNB,COTL1,DPYSL3,LRRC8A,IFRD1,PRCP,SESN2,PTGS2,MYO1C,SEL1L,KIF5B,HIF1A,DNAJC10,TNIK,UBA6,PTPN2,PVR,DDX39B,ADAM17,PDIA4,CNOT7,DNAJB9,CTNNB1,CRYAB,APOE,KPNA4,UBA5,LYN,BNIP3,DDIT3,PDS5B,FAM162A,BACH1,SLC7A11,ATF3,GSK3B,CDC37,GTF2H1,ITGB3,GBP5,SDC4,REST,RBBP8,MAPK10,EIF2S1,RAD23A,PAK2,CREB3L2,A2M,HIF1AN,BTK,MSRB3,CDKN1A,TMTC3,SH3RF3,USP14,DEFB124,MID1	down_VSAP_EC
GO:0035051	0.0436	0.984	8	41	cardiocyte differentiation	BP	1	ACTN2,BMP4,CITED2,NEBL,TBX5,FRS2,EDN1,BVES	up_ACOR_VSMC
GO:1903037	0.0436	0.984	7	65	regulation of leukocyte cell-cell adhesion	BP	1	GPAM,PRNP,SDC4,THY1,GLI3,LOXL3,ZBTB16	down_VCAV_EC
GO:0060341	0.0436	0.99	4	255	regulation of cellular localization	BP	1	LYN,ACSL3,STX4,NEDD4	down_ACOR_EC
GO:0007162	0.0436	0.996	2	66	negative regulation of cell adhesion	BP	1	ACVRL1,HOXA7	down_ACOR_EC
GO:0046639	0.0436	0.996	2	7	negative regulation of alpha-beta T cell	BP	1	GLI3,LOXL3	down_VCAV_EC
GO:0022603	0.0436	1	1	301	regulation of anatomical structure morphogenesis	BP	1	GATA5	down_AFEM_VSMC
GO:2001237	0.0436	0.999	1	33	negative regulation of extrinsic apoptotic	BP	1	EYA1	up_AFEM_VSMC
GO:1903555	0.0436	0.999	1	37	regulation of tumor necrosis factor superfamily cytokine	BP	1	ARG2	up_AORT_VSMC
GO:0031669	0.0437	0.98	12	72	cellular response to nutrient levels	BP	1	PRKAA2,CASTOR1,CDKN2B,GABARAPL1,CDKN1A,SESN3,PIK3C3,LAMP2,SESN1,FAM107A,BMT2,ZFYVE1	up_ACOR_VSMC
GO:0048518	0.0437	0.984	10	1676	positive regulation of biological process	BP	1	TBX5,HOXA3,NR1H4,EVI2B,WIF1,CCL24,PTGER2,FLRT3,ARHGEF2,NEO1	up_VPUL_EC
GO:0043624	0.0437	0.985	7	39	cellular protein complex disassembly	BP	1	CAPG,LIMA1,STMN1,EIF5A,PDXP,ARHGEF2,KIF24	down_ACOR_VSMC
GO:0032386	0.0437	0.984	7	137	regulation of intracellular transport	BP	1	LEP,GAS1,CRYAB,CHRM2,CAMK2A,REEP2,BVES	up_AFEM_EC
GO:0098900	0.0437	0.991	4	15	regulation of action potential	BP	1	FGF13,KCNE3,PTPN3,JUP	up_ACOR_VSMC
GO:0009968	0.0437	0.99	4	392	negative regulation of signal transduction	BP	1	NR1H4,PTGER2,FLRT3,ARHGEF2	up_VPUL_EC
GO:0097028	0.0437	0.999	1	11	dendritic cell differentiation	BP	1	LYN	down_ACOR_EC

GO:0071495	0.0438	0.972	31	365	cellular response to endogenous stimulus	BP	0.874	ARHGEF2,SULF1,NR1H4,HSPA5,ENPP1,SNX25,PTPN11,TRIB3,ILK,PRCP,SMARCA4,SESN2,TGFBR1,MYO1C,MSN,ZPR1,PTPN2,YES1,NEO1,ADAM17,AGRN,GNAQ,CTNNB1,ENSCAFG00000002015,UBA5,STRAP,REST,NR4A1,ENSCAFG00000018360,YWHAH,KDM3A	down_VSAP_EC
GO:0048638	0.0438	0.987	5	82	regulation of developmental growth	BP	1	LEP,DLL1,PLXNA4,GPAM,ACACB	up_AFEM_EC
GO:0060627	0.0438	0.992	3	165	regulation of vesicle-mediated transport	BP	1	LYN,ACSL3,STX4	down_ACOR_EC
GO:0095500	0.0438	0.997	2	6	acetylcholine receptor signaling pathway	BP	0.874	AGRN,GNAQ	down_VSAP_EC
GO:1903831	0.0438	0.997	2	6	signal transduction involved in cellular response to ammonium	BP	0.874	AGRN,GNAQ	down_VSAP_EC
GO:1905144	0.0438	0.997	2	6	response to acetylcholine	BP	0.874	AGRN,GNAQ	down_VSAP_EC
GO:1905145	0.0438	0.997	2	6	cellular response to acetylcholine	BP	0.874	AGRN,GNAQ	down_VSAP_EC
GO:0061008	0.0438	0.999	1	23	hepaticobiliary system development	BP	1	CITED2	down_VPOR_EC
GO:1901880	0.0438	0.999	1	19	negative regulation of protein depolymerization	BP	1	TAOK1	up_AORT_EC
GO:0045843	0.0438	0.999	1	13	negative regulation of striated muscle tissue	BP	1	TBX5	up_VPUL_EC
GO:0055024	0.0438	0.999	1	22	regulation of cardiac muscle tissue	BP	1	TBX5	up_VPUL_VSMC
GO:0048568	0.0439	0.975	21	148	embryonic organ development	BP	1	TBX15,HOXA11,HOXA7,WNT11,OSR2,INSIG2,GJB5,LRIG3,IRX5,HOXD3,DLL1,HOXA6,OSR1,RARG,HOXC4,ZFP36L1,ZEB1,SHOX2,KDM2B,TGFBR2,CITED2	up_VSAP_EC
GO:0006950	0.0439	0.982	10	1102	response to stress	BP	1	ACVRL1,LYN,IGF1R,PDPN,USP28,IPO7,ATRIP,ELK3,STX4,NEDD4	down_ACOR_EC
GO:0009411	0.0439	0.982	9	56	response to UV	BP	1	USP1,NEDD4,AURKB,PCNA,NOC2L,POLH,DTL,CDC25A,HMGN1	down_ACOR_VSMC
GO:0002718	0.0439	0.988	5	21	regulation of cytokine production involved in	BP	1	TLR3,F2RL1,B2M,MAP3K7,IL18R1	up_ACOR_VSMC
GO:1900015	0.0439	0.991	4	17	regulation of cytokine production involved in inflammatory response	BP	0.855	GBP5,TLR4,APOD,NOS2	up_VCAV_EC
GO:0060560	0.0439	0.996	2	58	developmental growth involved in	BP	1	SPG11,NRP2	down_ACOR_EC
GO:2001198	0.0439	0.997	2	4	regulation of dendritic cell differentiation	BP	1	TMEM176B,TMEM176A	up_ACOR_VSMC
GO:0044070	0.0439	0.999	1	22	regulation of anion transport	BP	1	RAB11B	down_VPOR_EC
GO:0060021	0.0439	0.999	1	34	roof of mouth development	BP	1	WNT7A	up_AORT_VSMC
GO:0048738	0.0439	0.999	1	64	cardiac muscle tissue development	BP	1	TBX5	up_APUL_VSMC
GO:0021604	0.0439	0.999	1	6	cranial nerve structural organization	BP	1	SEMA3A	up_VPOR_EC
GO:0021612	0.0439	0.999	1	6	facial nerve structural organization	BP	1	SEMA3A	up_VPOR_EC
GO:1905209	0.0439	0.999	1	13	positive regulation of cardiocyte differentiation	BP	1	TBX5	up_VPUL_EC
GO:0006816	0.044	0.979	12	84	calcium ion transport	BP	0.856	CALCRL,PDGFB,ATP2B1,PDE4D,CHD7,STC1,APLNR,PPP3CA,SLC25A25,PLCG2,MCU,ATP2A1	up_VCAV_EC
GO:0009607	0.044	0.992	3	232	response to biotic stimulus	BP	1	NR1H4,RGS1,MRC1	up_VPUL_EC
GO:0070563	0.044	1	1	1	negative regulation of vitamin D receptor	BP	1	KANK2	down_VCAV_EC
GO:0072594	0.044	1	1	155	establishment of protein localization to organelle	BP	1	JUP	down_VFEM_VSMC
GO:0090257	0.044	0.999	1	52	regulation of muscle system process	BP	1	GATA5	down_VSAP_VSMC
GO:0055081	0.044	0.999	1	17	anion homeostasis	BP	1	HOXA3	up_VPOR_VSMC
GO:1905207	0.044	0.999	1	17	regulation of cardiocyte differentiation	BP	1	NKX2-5	up_VPOR_VSMC
GO:0032720	0.044	0.999	1	13	negative regulation of tumor necrosis factor	BP	1	NR1H4	up_VPUL_EC
GO:0050710	0.044	0.999	1	13	negative regulation of cytokine secretion	BP	1	NR1H4	up_VPUL_EC
GO:0014855	0.044	0.999	1	22	striated muscle cell proliferation	BP	1	TBX5	up_VPUL_VSMC
GO:0060419	0.044	0.999	1	22	heart growth	BP	1	TBX5	up_VPUL_VSMC

GO:2000416	0.044026705	0.99951	1	2	regulation of eosinophil migration	BP	1	PTGER4	up_ACOR_EC
GO:0001952	0.0441	0.988	5	34	regulation of cell-matrix adhesion	BP	0.874	VEGFA,SERPINE1,ENSCAFG00000002015,GSK3B,NRP1	down_VSAP_EC
GO:0016601	0.0441	0.999	1	11	Rac protein signal transduction	BP	1	TIAM1	up_VFEM_EC
GO:0065007	0.0442	0.98	23	3499	biological regulation	BP	1	ACVRL1,YEATS2,LYN,HOXA3,IGF1R,PDPN,NRP2,YBX3,HIVEP2,HOXA7,PRKAR2A,USP28,IPO7,ATRIP,SULF1,ELK3,HOXA1,ACSL3,WDR12,STX4,MYO1D,ASPN,NEDD4	down_ACOR_EC
GO:0005975	0.0442	0.976	17	183	carbohydrate metabolic process	BP	0.874	ENSCAFG00000009211,ENPP1,PGK1,TPI1,ENSCAFG00000032470,PGAM1,SESN2,HIF1A,SLC45A3,PTPN2,MOGS,ALDOC,PPP1R3C,ATF3,GSK3B,GALE,EXT1	down_VSAP_EC
GO:0032755	0.0442	0.988	5	24	positive regulation of interleukin-6 production	BP	0.86	TLR4,MAPK13,TLR3,HMGB1,ARHGEF2	up_VCAV_EC
GO:0018196	0.0442	0.991	4	15	peptidyl-asparagine modification	BP	1	ST3GAL5,MAGT1,STT3B,FUT8	up_ACOR_VSMC
GO:0007399	0.0442	0.99	4	564	nervous system development	BP	1	HOXC10,EYA1,EMX2,SHOX2	up_VFEM_VSMC
GO:0021564	0.0442	1	1	2	vagus nerve development	BP	1	PLXNA4	up_AFEM_EC
GO:0032231	0.0442	0.999	1	32	regulation of actin filament bundle assembly	BP	1	LIMK1	up_VCAV_VSMC
GO:0044381	0.0442	0.999	1	13	glucose import in response to insulin	BP	1	NR1H4	up_VPUL_EC
GO:0044091	0.0442	0.999	1	10	membrane biogenesis	BP	1	CAV1	up_VSAP_VSMC
GO:1903725	0.044243365	0.99231	3	30	regulation of phospholipid metabolic process	BP	1	NR1H4,ENSCAFG00000004331,PRKCD	up_ACOR_EC
GO:0071496	0.0443	0.977	15	97	cellular response to external stimulus	BP	1	PRKAA2,CASTOR1,TLR3,CDKN2B,ANKRD1,GABARAPL1,BNIP3,CDKN1A,SESN3,PIK3C3,LAMP2,SESN1,FAM107A,BMT2,ZFYVE1	up_ACOR_VSMC
GO:0043062	0.0443	0.98	11	99	extracellular structure organization	BP	0.874	SULF1,SERPINH1,ERO1A,P4HA1,FSCN1,APOE,PLOD3,MYO1E,SDC4,P3H4,COL12A1	down_VSAP_EC
GO:0060412	0.0443	0.991	4	15	ventricular septum morphogenesis	BP	1	BMP4,FGFR2,CITED2,EGLN1	up_ACOR_VSMC
GO:0010828	0.0443	0.993	3	16	positive regulation of glucose transmembrane	BP	1	CLIP3,ENSCAFG00000014978,AKT2	down_VCAV_EC
GO:0060322	0.0443	0.992	3	193	head development	BP	1	PITX1,EMX2,SALL1	up_VSAP_VSMC
GO:0051155	0.0443	0.999	1	18	positive regulation of striated muscle cell	BP	1	SHOX2	up_VFEM_VSMC
GO:0048592	0.0444	0.986	6	53	eye morphogenesis	BP	1	FBN1,NAGLU,THY1,GLI3,IRX5,THRB	down_VCAV_EC
GO:0042221	0.0444	0.992	4	1012	response to chemical	BP	1	SLITRK5,CYP1A1,GATA5,CA2	up_VCAV_VSMC
GO:0045932	0.0444	0.994	3	8	negative regulation of muscle contraction	BP	1	KCNE3,PDE5A,DOCK5	up_ACOR_VSMC
GO:0071359	0.0444	0.993	3	11	cellular response to dsRNA	BP	0.861	TMEM173,RALB,RIOK3	up_VCAV_EC
GO:0097435	0.0444	0.992	3	230	supramolecular fiber organization	BP	1	MYBPC2,CCL24,ARHGEF2	up_VPUL_EC
GO:0060973	0.0444	0.996	2	7	cell migration involved in heart development	BP	1	PDGFRB,SEMA3C	down_VCAV_EC
GO:0006575	0.0444	0.999	1	53	cellular modified amino acid metabolic process	BP	1	ENSCAFG00000028814	down_VSAP_VSMC
GO:0017145	0.0444	0.999	1	13	stem cell division	BP	1	ARHGEF2	up_VPUL_EC

GO:0009058	0.0445	0.964	178	1910	biosynthetic process	BP	1	ELK3,SCD,ACSL3,PRPSAP1,SQLE,HMGB1,MCM6,LSS,SREBF1,ACLY,MCM4,IRAK1,MCM7,RPA1,ILF3,CBX5,PPRC1,BCAT1,ATAD2,HMGA1,EIF4A3,HMGCR,HNRNPD,SREBF2,MCM3,SMC1A,HINFP,VARS,NEDD4,RRM1,MED17,POLA1,SLC29A1,MTDH,HEY1,RRBP1,PPP1R15B,SF1,POLA2,MPDU1,SRRT,GINS4,CTDNEP1,POLE,NAB2,SLC35C2,MRPS18B,B3GNT2,CDCA7L,FUS,RFC2,BIRC5,MYBBP1A,INSIG1,PRMT1,CAV1,RFC5,RFC3,RAD51,XYLT1,TIMELESS,THRAP3,TRUB2,ENSCAFG00000018572,FAM129B,TCEA1,PFAS,AURKB,NONO,TK1,TOPBP1,EZH2,GREM1,RFC4,NEK2,FEN1,CDK4,SMARCC1,PLK3,PCNA,DHX33,HOXA7,TONSL,ARG2,MCM10,UHRF1,CKS2,TKT,GMNN,DHCR24,BRCA1,MVD,FASTKD3,NOC2L,EIF5A,E2F8,B4GALT1,ELOVL1,FARSA,PPARD,CDC6,POLH,YBX3,NCL,NUP62,UGDH,MRPL37,DTL,VAC14,FOSL1,SUV39H1,AADAT,ZNF367,CACTIN,HMGB2,DMAP1,SMC3,CDK2,SMARCB1,DDX39B,CCNA2,ENSCAFG00000005299,EZR,ELOVL6,FDFT1,RBM14,CYC1,NOLC1,POLR3F,HOXA10,ERF,TCERG1,RARS,ATP5F1A,PUM3,CDC45,MAST2,SMARCAL1,PRPS1,ENSCAFG00000013366,TBX15,GRWD1,ELF4,PRMT5,PSMC1,CTH,ARHGEF2,SUPT16H,PDHA1,TADA2A,HAT1,HIST2H2AC,HO	down_ACOR_VSMC
GO:0048878	0.0445	0.971	36	283	chemical homeostasis	BP	1	ADRA1B,PLCG2,LEP,SLC26A7,HTR2B,PRKAA2,LCAT,CNNM2,ABCA1,GJA1,PTPN3,MICU2,ITPR1,F2RL1,ERO1B,EGLN1,DMPK,ATP7A,ORMDL3,SESN3,PRKCE,APP,PSEN1,CHD7,DMXL1,RAB11B,EDN1,B2M,DISC1,HERPUD1,INPP5K,WFS1,ATP2C1,PARK7,SLC8A1,STC2	up_ACOR_VSMC
GO:0055085	0.0445	0.973	24	284	transmembrane transport	BP	0.874	SLC39A7,NR1H4,TRAM1,ERO1A,ENPP1,PTPN11,LRRC8A,SLC38A1,TRIB3,SESN2,SRP54,REM1,WDR1,NDUFA4L2,YES1,SRP72,LYN,DDIT3,SLC2A3,CLCN3,HTT,CALM1,YWHAH,HOMER1	down_VSAP_EC
GO:0072358	0.0445	0.975	20	268	cardiovascular system development	BP	1	HSPB6,PDGFRB,DCN,ANGPT1,BMPR1A,LOX,RECK,ANPEP,SEMA3C,THY1,B4GALT1,GLI3,ZFP36L1,MYDGF,SASH1,CDH2,SPRED1,SHC1,GREM1,CLIC4	down_VCAV_EC
GO:1902105	0.0445	0.983	8	80	regulation of leukocyte differentiation	BP	1	NOTCH2,FBN1,TFE3,CLSTN1,GLI3,ZFP36L1,LOXL3,ZBTB16	down_VCAV_EC
GO:0061549	0.0445	0.997	2	5	sympathetic ganglion development	BP	0.874	CTNNB1,NRP1	down_VSAP_EC
GO:0003159	0.0445	0.999	1	9	morphogenesis of an endothelium	BP	1	ACVRL1	down_ACOR_EC
GO:0061154	0.0445	0.999	1	9	endothelial tube morphogenesis	BP	1	ACVRL1	down_ACOR_EC
GO:0007354	0.0445	1	1	1	zygotic determination of anterior/posterior axis,	BP	1	PCSK6	down_VCAV_EC
GO:0032898	0.0445	1	1	1	neurotrophin production	BP	1	PCSK6	down_VCAV_EC
GO:0032902	0.0445	1	1	1	nerve growth factor production	BP	1	PCSK6	down_VCAV_EC
GO:0050923	0.0445	0.999	1	2	regulation of negative chemotaxis	BP	1	PLXNA4	up_AFEM_EC
GO:0071674	0.0445	0.999	1	23	mononuclear cell migration	BP	1	SLIT2	up_VPUL_VSMC
GO:1902186	0.044539597	0.99576	2	13	regulation of viral release from host cell	BP	1	TRIM8,CHMP4B	up_ACOR_EC
GO:0048736	0.0446	0.984	7	66	appendage development	BP	1	BMPR1A,RECK,SEMA3C,GLI3,RDH10,GREM1,ZBTB16	down_VCAV_EC
GO:0060173	0.0446	0.984	7	66	limb development	BP	1	BMPR1A,RECK,SEMA3C,GLI3,RDH10,GREM1,ZBTB16	down_VCAV_EC
GO:0071542	0.0446	0.996	2	6	dopaminergic neuron differentiation	BP	0.874	HIF1A,CTNNB1	down_VSAP_EC
GO:0051138	0.0446	0.997	2	4	positive regulation of NK T cell differentiation	BP	1	ZBTB16,TGFBR2	up_VSAP_EC
GO:1900078	0.0446	0.999	1	13	positive regulation of cellular response to	BP	1	NR1H4	up_VPUL_EC
GO:0043009	0.0447	0.972	27	227	chordate embryonic development	BP	1	EIF4A3,HINFP,HOXA3,GINS4,PRMT1,XYLT1,MMP14,SLC30A1,NEK2,RNASEH2B,HOXA7,HOXB7,BRCA1,E2F8,FOSL1,PLK4,HOXC6,SMARCB1,HOXB5,RBBP8,NOLC1,PHACTR4,TBX15,HOXA9,EMG1,RTCB,SIX4	down_ACOR_VSMC
GO:0048583	0.0447	0.987	6	1095	regulation of response to stimulus	BP	1	BARX1,NKX2-5,BAMBI,ANKRD6,CYP26B1,C3AR1	up_VPOR_VSMC
GO:0006084	0.0447	0.99	4	15	acetyl-CoA metabolic process	BP	1	ACACB,PDK2,ACSS2,MVK	up_VSAP_EC

GO:1902236	0.0447	0.993	3	9	negative regulation of endoplasmic reticulum stress-induced intrinsic apoptotic signaling	BP	1	HERPUD1,WFS1,PARK7	up_ACOR_VSMC
GO:0042551	0.0447	0.993	3	10	neuron maturation	BP	0.867	EDNRB,NTN4,SPTBN4	up_VCAV_EC
GO:0006811	0.0447	0.993	3	330	ion transport	BP	1	HOXA3,NKX2-5,TRPC4	up_VPOR_VSMC
GO:0009150	0.0447	0.996	2	161	purine ribonucleotide metabolic process	BP	1	SUCLG2,NDUFA10	down_AFEM_EC
GO:0050804	0.0447	0.995	2	87	modulation of chemical synaptic transmission	BP	1	PLCL1,NTRK2	up_APUL_EC
GO:0099177	0.0447	0.995	2	87	regulation of trans-synaptic signaling	BP	1	PLCL1,NTRK2	up_APUL_EC
GO:0061515	0.0447	0.999	1	24	myeloid cell development	BP	1	CITED2	down_VPOR_EC
GO:0001974	0.0447	0.999	1	13	blood vessel remodeling	BP	1	HOXA3	up_VPUL_EC
GO:0035282	0.044760611	0.99219	3	30	segmentation	BP	1	FZD5,FOXC2,NRP1	up_ACOR_EC
GO:0016477	0.0448	0.976	16	426	cell migration	BP	1	CMKLR1,ANGPT4,HOXA7,TWIST2,HCK,GREM1,CAMK2A,LRRC15,RECK,TBXA2R,BVES,LGR6,CCL5,FAP,ADAM17,KANK1	up_AFEM_EC
GO:0043368	0.0448	0.993	3	9	positive T cell selection	BP	0.867	IL6,MTOR,ITPKB	up_VCAV_EC
GO:0048011	0.0448	0.996	2	7	neurotrophin TRK receptor signaling	BP	1	NDN,AKT1S1	down_VCAV_EC
GO:0030194	0.0448	0.999	1	9	positive regulation of blood coagulation	BP	1	PDPN	down_ACOR_EC
GO:1900048	0.0448	0.999	1	9	positive regulation of hemostasis	BP	1	PDPN	down_ACOR_EC
GO:2000377	0.0448	0.999	1	41	regulation of reactive oxygen species metabolic	BP	1	ARG2	up_AORT_VSMC
GO:0030324	0.0448	0.999	1	64	lung development	BP	1	TBX5	up_APUL_VSMC
GO:0061550	0.0448	0.999	1	6	cranial ganglion development	BP	1	SEMA3A	up_VPOR_EC
GO:0007019	0.0448	0.999	1	13	microtubule depolymerization	BP	1	ARHGEF2	up_VPUL_EC
GO:0045214	0.0448	0.999	1	13	sarcomere organization	BP	1	MYBPC2	up_VPUL_EC
GO:0030003	0.0449	0.974	22	157	cellular cation homeostasis	BP	1	ADRA1B,PLCG2,SLC26A7,HTR2B,MICU2,ITPR1,F2RL1,EGLN1,DMPK,ATP7A,PRKCE,APP,PSEN1,CHD7,DMXL1,EDN1,DISC1,HERPUD1,WFS1,ATP2C1,SLC8A1,STC2	up_ACOR_VSMC
GO:0002376	0.0449	0.986	6	622	immune system process	BP	1	HOXA9,TOX,HOXB7,HOXA7,SIX1,CYP26B1	up_VFEM_EC
GO:0034097	0.0449	0.987	5	238	response to cytokine	BP	1	ARHGEF2,ENSCAFG00000002015,ASPN,ENSCAFG00000032731,GNPNAT1	up_VPOR_EC
GO:0031100	0.0449	0.997	2	4	animal organ regeneration	BP	1	EZH2,GLI1	down_ACOR_VSMC
GO:0035774	0.0449	0.996	2	6	positive regulation of insulin secretion involved in cellular response to	BP	0.874	NR1H4,HIF1A	down_VSAP_EC
GO:0048588	0.0449	0.995	2	44	developmental cell growth	BP	1	SEMA3A,RNF6	up_VPOR_EC
GO:0009247	0.0449	0.999	1	22	glycolipid biosynthetic process	BP	1	GBGT1	down_VPOR_EC
GO:0002028	0.0449	0.999	1	19	regulation of sodium ion transport	BP	1	NKX2-5	up_VPOR_VSMC
GO:0055006	0.0449	0.999	1	18	cardiac cell development	BP	1	NKX2-5	up_VPOR_VSMC
GO:1900180	0.044922604	0.98932	4	50	regulation of protein localization to nucleus	BP	1	TRIM8,DCLK2,MAVS,PRKCD	up_ACOR_EC
GO:0016053	0.045	0.977	15	97	organic acid biosynthetic process	BP	1	ACSM3,LIPC,INSIG2,ACACB,GOT1,GLUL,ENSCAFG00000013986,HSD17B8,GALK1,PTGES,HACD4,ENSCAFG00000016140,DHRS9,ASS1,DDIT4	up_VSAP_EC
GO:0046394	0.045	0.977	15	97	carboxylic acid biosynthetic process	BP	1	ACSM3,LIPC,INSIG2,ACACB,GOT1,GLUL,ENSCAFG00000013986,HSD17B8,GALK1,PTGES,HACD4,ENSCAFG00000016140,DHRS9,ASS1,DDIT4	up_VSAP_EC
GO:0001906	0.045	0.99	4	20	cell killing	BP	0.867	IL7R,LYZ,RAB27A,NECTIN2	up_VCAV_EC
GO:0033628	0.045	0.993	3	14	regulation of cell adhesion mediated by integrin	BP	0.874	SERPINE1,PTPN11,LYN	down_VSAP_EC
GO:0043031	0.045	0.997	2	4	negative regulation of macrophage activation	BP	0.867	ADGRF5,CD200	up_VCAV_EC
GO:2000647	0.045	0.997	2	4	negative regulation of stem cell proliferation	BP	0.867	FERMT1,CTNNA1	up_VCAV_EC

GO:2000241	0.045	0.999	1	40	regulation of reproductive process	BP	1	ESR1	up_AORT_VSMC
GO:0003229	0.045	0.999	1	17	ventricular cardiac muscle tissue development	BP	1	NKX2-5	up_VPOR_VSMC
GO:0072132	0.045	0.999	1	23	mesenchyme morphogenesis	BP	1	GATA5	up_VPUL_VSMC
GO:0050727	0.04505981	0.98699	5	74	regulation of inflammatory response	BP	1	NR1H4,FNDC4,NOD2,IL33,PTGER4	up_ACOR_EC
GO:2000725	0.0451	0.993	3	10	regulation of cardiac muscle cell differentiation	BP	0.867	MEF2C,EDN1,BMP2	up_VCAV_EC
GO:0046653	0.0451	0.997	2	5	tetrahydrofolate metabolic process	BP	0.867	PIPOX,GCH1	up_VCAV_EC
GO:2000649	0.0451	0.999	1	11	regulation of sodium ion transmembrane transporter activity	BP	1	NEDD4	down_ACOR_EC
GO:1903531	0.0451	0.999	1	41	negative regulation of secretion by cell	BP	1	ARG2	up_AORT_VSMC
GO:0001754	0.0451	0.999	1	12	eye photoreceptor cell differentiation	BP	1	NTRK2	up_APUL_EC
GO:0006959	0.0452	0.988	5	21	humoral immune response	BP	1	PLA2G6,TFE3,CFD,CD83,JCHAIN	up_VSAP_EC
GO:0045087	0.0452	0.992	3	161	innate immune response	BP	1	LYN,IPO7,STX4	down_ACOR_EC
GO:1903115	0.0452	0.999	1	12	regulation of actin filament-based movement	BP	1	PDPN	down_ACOR_EC
GO:0038061	0.0452	0.999	1	34	NIK/NF-kappaB signaling	BP	1	TLR2	up_AFEM_VSMC
GO:0042461	0.0452	0.999	1	12	photoreceptor cell development	BP	1	NTRK2	up_APUL_EC
GO:0051155	0.0452	0.999	1	18	positive regulation of striated muscle cell	BP	1	CYP26B1	up_VPOR_VSMC
GO:0021846	0.0452	0.999	1	11	cell proliferation in forebrain	BP	1	EMX2	up_VSAP_VSMC
GO:0051962	0.0453	0.979	12	112	positive regulation of nervous system	BP	0.874	ARHGEF2,DPYSL3,HIF1A,CTNNB1,APOE,LYN,MMD,VLDLR,REST,DISC1,LRRTM2,NRP1	down_VSAP_EC
GO:2001258	0.0453	0.993	3	11	negative regulation of cation channel activity	BP	1	CAV1,GSTO1,REM1	down_ACOR_VSMC
GO:0002757	0.0453	0.995	2	78	immune response-activating signal	BP	1	TLR2,CAV1	up_VSAP_VSMC
GO:0061298	0.0453	0.999	1	8	retina vasculature development in camera-	BP	1	ACVRL1	down_ACOR_EC
GO:1901098	0.0453	1	1	1	positive regulation of autophagosome	BP	1	CLEC16A	down_VCAV_EC
GO:1904766	0.0453	1	1	1	negative regulation of macroautophagy by TORC1 signaling	BP	1	CLEC16A	down_VCAV_EC
GO:0072673	0.0454	0.999	1	8	lamellipodium morphogenesis	BP	1	PDPN	down_ACOR_EC
GO:0009887	0.0454	1	1	314	animal organ morphogenesis	BP	1	GATA5	down_AFEM_VSMC
GO:0043030	0.0454	0.999	1	12	regulation of macrophage activation	BP	1	BPI	up_APUL_EC
GO:0010721	0.045447501	0.98685	5	73	negative regulation of cell development	BP	1	SOX9,DIXDC1,RGMA,LRP4,NRP1	up_ACOR_EC
GO:0061138	0.04547428	0.98684	5	73	morphogenesis of a branching epithelium	BP	1	SOX9,FZD5,FOXC2,CLSTN1,NRP1	up_ACOR_EC
GO:0014033	0.045490839	0.99201	3	30	neural crest cell differentiation	BP	1	SOX9,FOXC2,NRP1	up_ACOR_EC
GO:0032409	0.045493626	0.98912	4	52	regulation of transporter activity	BP	1	ACTN2,MAPK8IP2,PRKCD,PM20D1	up_ACOR_EC
GO:0045859	0.0455	0.974	20	219	regulation of protein kinase activity	BP	0.874	VEGFA,SOCS5,ERRFI1,PTPN11,TRIB3,SESN2,TNIK,PTPN2,ADAM17,MAGED1,APOE,LYN,CCNYL1,MMD,CBLC,VLDLR,HTT,PAK2,CDKN1A,MADD	down_VSAP_EC
GO:1902041	0.0455	0.99	4	23	regulation of extrinsic apoptotic signaling pathway via death	BP	0.874	ARHGEF2,SERPINE1,ATF3,MADD	down_VSAP_EC
GO:1900272	0.0455	0.997	2	4	negative regulation of long-term synaptic	BP	1	APP,FAM107A	up_ACOR_VSMC
GO:0007565	0.0455	0.999	1	23	female pregnancy	BP	1	CITED2	down_VPOR_EC
GO:0030323	0.0455	0.999	1	65	respiratory tube development	BP	1	TBX5	up_APUL_VSMC
GO:0048736	0.0455	0.999	1	66	appendage development	BP	1	TBX5	up_APUL_VSMC
GO:0060173	0.0455	0.999	1	66	limb development	BP	1	TBX5	up_APUL_VSMC
GO:0006216	0.0455	1	1	1	cytidine catabolic process	BP	0.871	CDA	up_VCAV_EC

GO:0009972	0.0455	1	1	1	cytidine deamination	BP	0.871	CDA	up_VCAV_EC
GO:0046087	0.0455	1	1	1	cytidine metabolic process	BP	0.871	CDA	up_VCAV_EC
GO:0051961	0.045581227	0.98681	5	73	negative regulation of nervous system	BP	1	SOX9,DIXDC1,RGMA,LRP4,NRP1	up_ACOR_EC
GO:0006468	0.0456	0.97	36	544	protein phosphorylation	BP	1	NOTCH2,PDGFRB,KIRREL1,SMAD9,DCN,PRNP,GLIPR2,BGN,BMPR1A,CLIP3,AKT1S1,AKT2,EEF2K,PLCL1,CSPG4,ASPN,THY1,PARK7,MAST2,ZFP36L1,MYDGF,SASH1,ARL2BP,MAVS,CDH2,GSDME,CAMK2D,SPRED1,MAPRE3,CDKL2,DDR2,SHC1,FBH1,GREM1,BRSK1,CDK14	down_VCAV_EC
GO:0031098	0.0456	0.977	15	109	stress-activated protein kinase signaling cascade	BP	0.873	EPHB1,MAP3K11,STK10,SH2D3C,TLR4,EDN1,MAP3K3,BMP2,SLK,MAPK13,PAK2,TLR3,TAOK1,HMGB1,RIPK1	up_VCAV_EC
GO:0019222	0.0456	0.992	5	1934	regulation of metabolic process	BP	1	HOXC10,HOXA9,WNT7A,ARG2,ESR1	up_AORT_VSMC
GO:0071470	0.0456	0.993	3	13	cellular response to osmotic stress	BP	0.874	ARHGEF2,CASP3,PTGS2	down_VSAP_EC
GO:1900078	0.0456	0.993	3	13	positive regulation of cellular response to	BP	0.874	NR1H4,PTPN11,MYO1C	down_VSAP_EC
GO:0061025	0.0456	0.995	2	69	membrane fusion	BP	1	SPG11,STX4	down_ACOR_EC
GO:1901722	0.0456	0.997	2	5	regulation of cell proliferation involved in	BP	0.873	PDGFB,GATA3	up_VCAV_EC
GO:0010573	0.0456	0.999	1	9	vascular endothelial growth factor production	BP	1	SULF1	down_ACOR_EC
GO:0010574	0.0456	0.999	1	9	regulation of vascular endothelial growth factor	BP	1	SULF1	down_ACOR_EC
GO:0015734	0.0456	0.999	1	2	taurine transport	BP	1	SLC6A6	up_AFEM_EC
GO:0031999	0.0456	0.999	1	2	negative regulation of fatty acid beta-oxidation	BP	1	ACACB	up_AFEM_EC
GO:0070091	0.0456	0.999	1	2	glucagon secretion	BP	1	LEP	up_AFEM_EC
GO:0070092	0.0456	0.999	1	2	regulation of glucagon secretion	BP	1	LEP	up_AFEM_EC
GO:1902744	0.0456	0.999	1	2	negative regulation of lamellipodium	BP	1	KANK1	up_AFEM_EC
GO:0030534	0.0456	0.999	1	33	adult behavior	BP	1	SLITRK5	up_VCAV_VSMC
GO:0051262	0.0457	0.984	7	40	protein tetramerization	BP	1	HMGCR,RRM1,TK1,RCC1,FARSA,CTH,PDSS1	down_ACOR_VSMC
GO:0031960	0.0457	0.99	4	15	response to corticosteroid	BP	1	CREBRF,NR3C1,FAM107A,DDIT4	up_ACOR_VSMC
GO:0051384	0.0457	0.99	4	15	response to glucocorticoid	BP	1	CREBRF,NR3C1,FAM107A,DDIT4	up_ACOR_VSMC
GO:0072160	0.0457	0.993	3	9	nephron tubule epithelial cell differentiation	BP	0.874	MEF2C,GATA3,MTSS1	up_VCAV_EC
GO:0045602	0.0457	0.997	2	4	negative regulation of endothelial cell	BP	1	ZEB1,ACVRL1	up_VSAP_EC
GO:0021784	0.0457	0.999	1	2	postganglionic parasympathetic fiber	BP	1	PLXNA4	up_AFEM_EC
GO:0061643	0.0457	0.999	1	2	chemorepulsion of axon	BP	1	PLXNA4	up_AFEM_EC
GO:0003205	0.0457	0.999	1	67	cardiac chamber development	BP	1	TBX5	up_APUL_VSMC
GO:0043069	0.045745039	0.97814	12	262	negative regulation of programmed cell death	BP	1	NR1H4,SOX9,AQP1,IFI6,SMAD6,BCL2,MAPK8IP2,FOXC2,FIGNL1,CLU,NRP1,PRKCD	up_ACOR_EC
GO:0030073	0.0458	0.983	8	42	insulin secretion	BP	1	HNF4A,SLC8B1,RAB11B,KCNB1,RAF1,STX4,PPP3CB,RAB11FIP2	up_VSAP_EC
GO:0006733	0.0458	0.985	6	45	oxidoreduction coenzyme metabolic process	BP	0.874	PGK1,TPI1,PGAM1,HIF1A,NMNAT2,ALDOC	down_VSAP_EC
GO:2000026	0.0458	0.99	4	560	regulation of multicellular organismal development	BP	1	EYA1,HOXA9,HOXA11,SHOX2	up_VFEM_VSMC
GO:0051984	0.0458	0.993	3	10	positive regulation of chromosome segregation	BP	1	FEN1,CDC6,SMC6	down_ACOR_VSMC
GO:0045893	0.0458	0.996	2	413	positive regulation of transcription, DNA-	BP	1	GATA5,TGFBR1	down_VSAP_VSMC
GO:0006543	0.0458	1	1	1	glutamine catabolic process	BP	1	GLS2	down_VCAV_EC
GO:0042478	0.0458	0.999	1	2	regulation of eye photoreceptor cell	BP	1	THRB	up_AFEM_EC
GO:0045741	0.0458	0.999	1	2	positive regulation of epidermal growth factor-activated receptor activity	BP	1	ADAM17	up_AFEM_EC
GO:0006749	0.0458	0.999	1	14	glutathione metabolic process	BP	1	ENSCAFG00000028814	up_VPUL_EC

GO:1902235	0.0459	0.99	4	15	regulation of endoplasmic reticulum stress-induced intrinsic apoptotic	BP	1	SERINC3,HERPUD1,WFS1,PARK7	up_ACOR_VSMC
GO:0043534	0.0459	0.989	4	57	blood vessel endothelial cell migration	BP	1	ANGPT4,GREM1,TBXA2R,ADAM17	up_AFEM_EC
GO:0035239	0.0459	0.996	2	299	tube morphogenesis	BP	1	WNT7A,ESR1	up_AORT_VSMC
GO:0061548	0.0459	0.999	1	8	ganglion development	BP	1	NRP2	down_ACOR_EC
GO:0014012	0.0459	0.999	1	2	peripheral nervous system axon regeneration	BP	1	TNC	up_AFEM_EC
GO:1990051	0.0459	0.999	1	2	activation of protein kinase C activity	BP	1	LEP	up_AFEM_EC
GO:0032640	0.0459	0.999	1	39	tumor necrosis factor production	BP	1	ARG2	up_AORT_VSMC
GO:0031644	0.0459	0.999	1	19	regulation of neurological system process	BP	1	NETO1	up_VFEM_VSMC
GO:0022029	0.0459	0.999	1	23	telencephalon cell migration	BP	1	SLIT2	up_VPUL_VSMC
GO:0010737	0.045981809	0.99555	2	13	protein kinase A signaling	BP	1	PDE4A,PRKACA	up_ACOR_EC
GO:0031396	0.046	0.977	14	90	regulation of protein ubiquitination	BP	1	SKP2,SH3RF2,ISG15,SASH1,PTPN22,PRKCE,PSEN1,NOD2,FAM107A,DCUN1D4,HERPUD1,WFS1,WDR48,PARK7	up_ACOR_VSMC
GO:0030641	0.046	0.992	3	34	regulation of cellular pH	BP	1	RAB20,RAB7A,ATP6V0B	up_AFEM_EC
GO:0061448	0.046	0.995	2	89	connective tissue development	BP	1	NR1H4,HOXA3	up_APUL_EC
GO:0003203	0.046	0.999	1	11	endocardial cushion morphogenesis	BP	1	ACVRL1	down_ACOR_EC
GO:0072078	0.046	0.999	1	33	nephron tubule morphogenesis	BP	1	EYA1	up_AFEM_VSMC
GO:0001708	0.046	0.999	1	20	cell fate specification	BP	1	HOXC10	up_AORT_EC
GO:0055023	0.046	0.999	1	12	positive regulation of cardiac muscle tissue	BP	1	TBX5	up_APUL_EC
GO:2001273	0.046	0.999	1	12	regulation of glucose import in response to	BP	1	NR1H4	up_APUL_EC
GO:0001766	0.046	1	1	1	membrane raft polarization	BP	0.877	MALL	up_VCAV_EC
GO:0031580	0.046	1	1	1	membrane raft distribution	BP	0.877	MALL	up_VCAV_EC
GO:0010960	0.046	0.999	1	6	magnesium ion homeostasis	BP	1	HOXA3	up_VPOR_EC
GO:0051926	0.046	0.999	1	14	negative regulation of calcium ion transport	BP	1	HOXA3	up_VPUL_EC
GO:0042417	0.0461	0.993	3	9	dopamine metabolic process	BP	1	MAOA,MOXD1,PARK7	up_ACOR_VSMC
GO:0090101	0.0461	0.992	3	34	negative regulation of transmembrane receptor protein serine/threonine kinase signaling pathway	BP	1	GREM1,GREM2,ADAM17	up_AFEM_EC
GO:0034377	0.0461	0.996	2	7	plasma lipoprotein particle assembly	BP	1	LCAT,SOAT1	down_VCAV_EC
GO:0065005	0.0461	0.996	2	7	protein-lipid complex assembly	BP	1	LCAT,SOAT1	down_VCAV_EC
GO:0001525	0.0461	0.995	2	173	angiogenesis	BP	1	HOXA3,SLIT2	up_VPUL_VSMC
GO:2000483	0.0461	0.997	2	4	negative regulation of interleukin-8 secretion	BP	1	ANXA4,MAPKBP1	up_VSAP_EC
GO:0043570	0.0461	0.999	1	2	maintenance of DNA repeat elements	BP	1	AXIN2	up_AFEM_EC
GO:0018345	0.0461	0.999	1	12	protein palmitoylation	BP	1	ZDHHC16	up_APUL_EC
GO:0051453	0.0461	0.999	1	33	regulation of intracellular pH	BP	1	CA2	up_VCAV_VSMC
GO:0001709	0.0461	0.999	1	11	cell fate determination	BP	1	CYP26B1	up_VFEM_EC
GO:0035493	0.0461	0.999	1	11	SNARE complex assembly	BP	1	TRIM9	up_VFEM_EC
GO:0070098	0.0461	0.999	1	14	chemokine-mediated signaling pathway	BP	1	CCL24	up_VPUL_EC
GO:0033137	0.0461	0.999	1	10	negative regulation of peptidyl-serine	BP	1	CAV1	up_VSAP_VSMC
GO:0032651	0.046101946	0.99553	2	13	regulation of interleukin-1 beta production	BP	1	FZD5,NOD2	up_ACOR_EC
GO:0001654	0.0462	0.985	6	110	eye development	BP	1	IRX5,COL8A2,DLL1,CRYAB,MAB21L1,THRB	up_AFEM_EC
GO:0150063	0.0462	0.985	6	110	visual system development	BP	1	IRX5,COL8A2,DLL1,CRYAB,MAB21L1,THRB	up_AFEM_EC

GO:0048880	0.0462	0.985	6	110	sensory system development	BP	1	IRX5,COL8A2,DLL1,CRYAB,MAB21L1,THRB	up_AFEM_EC
GO:1901016	0.0462	0.993	3	9	regulation of potassium ion transmembrane transporter activity	BP	1	ACTN2,KCNE3,AKAP6	up_ACOR_VSMC
GO:1902680	0.0462	0.996	2	415	positive regulation of RNA biosynthetic process	BP	1	GATA5,TGFBR1	down_VSAP_VSMC
GO:1903508	0.0462	0.996	2	415	positive regulation of nucleic acid-templated	BP	1	GATA5,TGFBR1	down_VSAP_VSMC
GO:1901097	0.0462	0.999	1	2	negative regulation of autophagosome	BP	1	CLEC16A	up_AFEM_EC
GO:0051606	0.0462	0.999	1	33	detection of stimulus	BP	1	TLR2	up_AFEM_VSMC
GO:0035115	0.0462	0.999	1	12	embryonic forelimb morphogenesis	BP	1	TBX5	up_APUL_EC
GO:0010092	0.0462	0.999	1	19	specification of animal organ identity	BP	1	HOXA11	up_VFEM_VSMC
GO:1990266	0.0462	0.999	1	24	neutrophil migration	BP	1	SLIT2	up_VPUL_VSMC
GO:0040011	0.046238292	0.97364	20	505	locomotion	BP	1	SOX9,AQP1,ENSCAFG00000004331,SEMA6D,DIXDC1,ZMYND8,PAK3,MMP28,TRIM8,PTGER4,ARHGDIB,ELMO2,FOXC2,LRRC15,TBX5,CHMP4B,RARRES2,NRP1,PRKCD,CCNYL1	up_ACOR_EC
GO:0055074	0.0463	0.977	13	95	calcium ion homeostasis	BP	0.88	ATP2B1,PDE4D,CHD7,GPR4,STC1,EDN1,APLNR,PDZD8,SWAP70,PLCG2,HMGB1,MCU,ATP2A1	up_VCAV_EC
GO:0016070	0.0463	1	2	1479	RNA metabolic process	BP	1	JUP,GATA5	down_VFEM_VSMC
GO:0090102	0.0463	0.999	1	10	cochlea development	BP	1	HOXA1	down_ACOR_EC
GO:0001787	0.0463	0.999	1	2	natural killer cell proliferation	BP	1	LEP	up_AFEM_EC
GO:0003151	0.0463	0.999	1	34	outflow tract morphogenesis	BP	1	EYA1	up_AFEM_VSMC
GO:0045638	0.0463	0.999	1	33	negative regulation of myeloid cell	BP	1	HOXA9	up_AFEM_VSMC
GO:0051048	0.0463	0.999	1	42	negative regulation of secretion	BP	1	ARG2	up_AORT_VSMC
GO:0086003	0.0463	0.999	1	10	cardiac muscle cell contraction	BP	1	CAV1	up_VSAP_VSMC
GO:0051480	0.0464	0.982	8	50	regulation of cytosolic calcium ion concentration	BP	0.881	PDE4D,CHD7,GPR4,EDN1,APLNR,SWAP70,PLCG2,HMGB1	up_VCAV_EC
GO:0035116	0.0464	0.99	4	15	embryonic hindlimb morphogenesis	BP	1	OSR2,OSR1,ZBTB16,RARG	up_VSAP_EC
GO:0014902	0.0464	0.999	1	34	myotube differentiation	BP	1	BEND2	up_AFEM_VSMC
GO:0042471	0.0464	0.999	1	34	ear morphogenesis	BP	1	EYA1	up_AFEM_VSMC
GO:0002831	0.0464	0.999	1	42	regulation of response to biotic stimulus	BP	1	ARG2	up_AORT_VSMC
GO:0097479	0.0464	0.999	1	41	synaptic vesicle localization	BP	1	WNT7A	up_AORT_VSMC
GO:0061178	0.0464	0.999	1	12	regulation of insulin secretion involved in cellular response to	BP	1	NR1H4	up_APUL_EC
GO:1903321	0.0464	0.999	1	33	negative regulation of protein modification by small protein conjugation	BP	1	LIMK1	up_VCAV_VSMC
GO:0050650	0.0464	0.999	1	11	chondroitin sulfate proteoglycan biosynthetic	BP	1	CHST7	up_VFEM_EC
GO:0060713	0.0464	0.999	1	10	labyrinthine layer morphogenesis	BP	1	GJB5	up_VSAP_VSMC
GO:0071804	0.046453521	0.99176	3	31	cellular potassium ion transport	BP	1	KCNB1,ACTN2,SLC9A9	up_ACOR_EC
GO:0071805	0.046453521	0.99176	3	31	potassium ion transmembrane transport	BP	1	KCNB1,ACTN2,SLC9A9	up_ACOR_EC
GO:0022607	0.0465	0.988	5	969	cellular component assembly	BP	1	GPSM2,EIF3M,SRSF6,NDUFA10,FSCN1	down_AFEM_EC
GO:0044249	0.0465	0.994	4	1851	cellular biosynthetic process	BP	1	AK5,GATA5,TGFBR1,MEIS2	down_VSAP_VSMC
GO:1990000	0.0465	0.996	2	6	amyloid fibril formation	BP	0.874	CRYAB,APOE	down_VSAP_EC
GO:0032099	0.0465	0.999	1	2	negative regulation of appetite	BP	1	LEP	up_AFEM_EC
GO:0038108	0.0465	0.999	1	2	negative regulation of appetite by leptin-mediated signaling	BP	1	LEP	up_AFEM_EC
GO:0060242	0.0465	0.999	1	2	contact inhibition	BP	1	FAP	up_AFEM_EC

GO:0032095	0.0465	0.999	1	2	regulation of response to food	BP	1	LEP	up_AFEM_EC
GO:0032096	0.0465	0.999	1	2	negative regulation of response to food	BP	1	LEP	up_AFEM_EC
GO:0032098	0.0465	0.999	1	2	regulation of appetite	BP	1	LEP	up_AFEM_EC
GO:1904684	0.0465	0.999	1	2	negative regulation of metalloendopeptidase	BP	1	RECK	up_AFEM_EC
GO:1905049	0.0465	0.999	1	2	negative regulation of metallopeptidase activity	BP	1	RECK	up_AFEM_EC
GO:0045638	0.0465	0.999	1	33	negative regulation of myeloid cell	BP	1	LRRC17	up_VCAV_VSMC
GO:0006096	0.0465	0.999	1	18	glycolytic process	BP	1	FBP1	up_VFEM_VSMC
GO:0006165	0.0465	0.999	1	18	nucleoside diphosphate phosphorylation	BP	1	FBP1	up_VFEM_VSMC
GO:0006757	0.0465	0.999	1	18	ATP generation from ADP	BP	1	FBP1	up_VFEM_VSMC
GO:0042866	0.0465	0.999	1	18	pyruvate biosynthetic process	BP	1	FBP1	up_VFEM_VSMC
GO:0050710	0.046568864	0.99546	2	13	negative regulation of cytokine secretion	BP	1	NR1H4,PTGER4	up_ACOR_EC
GO:0002286	0.0466	0.987	5	24	T cell activation involved in immune response	BP	0.881	IL6,GATA3,RAB27A,MTOR,HMGB1	up_VCAV_EC
GO:2000147	0.0466	0.989	4	169	positive regulation of cell motility	BP	1	C3AR1,ARHGEF2,SEMA3A,CCAR1	up_VPOR_EC
GO:0010971	0.0466	0.993	3	10	positive regulation of G2/M transition of mitotic	BP	1	CDK1,CDK4,DTL	down_ACOR_VSMC
GO:0007269	0.0466	0.992	3	34	neurotransmitter secretion	BP	1	KCNC4,CHRM2,CAMK2A	up_AFEM_EC
GO:0099643	0.0466	0.992	3	34	signal release from synapse	BP	1	KCNC4,CHRM2,CAMK2A	up_AFEM_EC
GO:0000083	0.0466	0.996	2	6	regulation of transcription involved in G1/S transition of mitotic cell	BP	0.874	ZPR1,BACH1	down_VSAP_EC
GO:0010990	0.0466	0.997	2	4	regulation of SMAD protein complex assembly	BP	1	SMAD6,TBX20	up_ACOR_VSMC
GO:0010991	0.0466	0.997	2	4	negative regulation of SMAD protein complex	BP	1	SMAD6,TBX20	up_ACOR_VSMC
GO:1901858	0.0466	0.999	1	2	regulation of mitochondrial DNA	BP	1	PPARGC1A	up_AFEM_EC
GO:0032675	0.0466	0.999	1	34	regulation of interleukin-6 production	BP	1	TLR2	up_AFEM_VSMC
GO:0032024	0.0466	0.999	1	12	positive regulation of insulin secretion	BP	1	NR1H4	up_APUL_EC
GO:0090277	0.0466	0.999	1	12	positive regulation of peptide hormone	BP	1	NR1H4	up_APUL_EC
GO:0048048	0.046674953	0.99544	2	13	embryonic eye morphogenesis	BP	1	FZD5,EFEMP1	up_ACOR_EC
GO:0000079	0.0467	0.987	5	26	regulation of cyclin-dependent protein serine/threonine kinase	BP	0.881	TNFAIP3,PDGFB,HHEX,PROX1,BLM	up_VCAV_EC
GO:0031952	0.0467	0.992	3	15	regulation of protein autophosphorylation	BP	0.874	VEGFA,ENPP1,ERRFI1	down_VSAP_EC
GO:0008284	0.0467	0.992	3	238	positive regulation of cell proliferation	BP	1	TBX5,HOXA3,CCL24	up_VPUL_EC
GO:0001501	0.0467	0.995	2	178	skeletal system development	BP	1	CITED2,TBX15	down_VPOR_EC
GO:1900409	0.0467	0.997	2	4	positive regulation of cellular response to	BP	0.881	TLR4,RIPK1	up_VCAV_EC
GO:1902884	0.0467	0.997	2	4	positive regulation of response to oxidative	BP	0.881	TLR4,RIPK1	up_VCAV_EC
GO:1903209	0.0467	0.997	2	4	positive regulation of oxidative stress-induced	BP	0.881	TLR4,RIPK1	up_VCAV_EC
GO:0045664	0.0467	0.995	2	144	regulation of neuron differentiation	BP	1	EYA1,SHOX2	up_VFEM_VSMC
GO:0070235	0.0467	0.999	1	2	regulation of activation-induced cell death of T	BP	1	GPAM	up_AFEM_EC
GO:0070236	0.0467	0.999	1	2	negative regulation of activation-induced cell	BP	1	GPAM	up_AFEM_EC
GO:1903265	0.0467	0.999	1	2	positive regulation of tumor necrosis factor-mediated signaling	BP	1	ADAM17	up_AFEM_EC
GO:0043901	0.0467	0.999	1	44	negative regulation of multi-organism process	BP	1	ARG2	up_AORT_VSMC
GO:0071706	0.0467	0.999	1	40	tumor necrosis factor superfamily cytokine	BP	1	ARG2	up_AORT_VSMC
GO:0048645	0.0467	0.999	1	33	animal organ formation	BP	1	GATA5	up_VCAV_VSMC

GO:0021517	0.0467	0.999	1	12	ventral spinal cord development	BP	1	HOXC10	up_VFEM_EC
GO:0042327	0.0468	0.969	37	289	positive regulation of phosphorylation	BP	1	ADRA1B,LEP,MAP3K8,MID1,HTR2B,RASSF2,BMP4,FGFR2,TLR3,SH3RF2,PDE5A,AR,SASH1,LMO4,FGF18,F2RL1,FAM129A,NRP1,PTPN22,NPNT,PRKCE,CCNT1,FRS2,PARP9,APP,PSEN1,CCL5,DDR2,DUSP22,EDN1,AKTIP,NOD2,SH3RF3,MAP3K7,ENSCAFG00000029553,PARK7,CRKL	up_ACOR_VSMC
GO:0050868	0.0468	0.985	6	28	negative regulation of T cell activation	BP	1	BMP4,PDE5A,CD274,PTPN22,DUSP22,PAG1	up_ACOR_VSMC
GO:0044271	0.0468	1	2	1501	cellular nitrogen compound biosynthetic	BP	1	JUP,GATA5	down_VFEM_VSMC
GO:0070365	0.0468	0.996	2	5	hepatocyte differentiation	BP	0.881	HHEX,PROX1	up_VCAV_EC
GO:0097325	0.0468	0.999	1	2	melanocyte proliferation	BP	1	FAP	up_AFEM_EC
GO:1990108	0.0468	0.999	1	2	protein linear deubiquitination	BP	1	CYLD	up_AFEM_EC
GO:0032571	0.0468	0.999	1	2	response to vitamin K	BP	1	POSTN	up_AFEM_EC
GO:0035581	0.0468	0.999	1	2	sequestering of extracellular ligand from	BP	1	GREM2	up_AFEM_EC
GO:0060844	0.0468	0.999	1	2	arterial endothelial cell fate commitment	BP	1	DLL1	up_AFEM_EC
GO:0060846	0.0468	0.999	1	2	blood vessel endothelial cell fate commitment	BP	1	DLL1	up_AFEM_EC
GO:0060847	0.0468	0.999	1	2	endothelial cell fate specification	BP	1	DLL1	up_AFEM_EC
GO:0097101	0.0468	0.999	1	2	blood vessel endothelial cell fate specification	BP	1	DLL1	up_AFEM_EC
GO:1900115	0.0468	0.999	1	2	extracellular regulation of signal transduction	BP	1	GREM2	up_AFEM_EC
GO:1900116	0.0468	0.999	1	2	extracellular negative regulation of signal	BP	1	GREM2	up_AFEM_EC
GO:1903054	0.0468	0.999	1	2	negative regulation of extracellular matrix	BP	1	FAP	up_AFEM_EC
GO:0008593	0.0468	0.999	1	33	regulation of Notch signaling pathway	BP	1	GATA5	up_VCAV_VSMC
GO:0048513	0.0469	0.963	101	906	animal organ development	BP	1	TBX15,HOXA11,FZD1,THRB,EMX2,HOXA7,PEX7,PCDH19,HOXA9,SEZ6L,CDON,WNT11,HNF4A,AXIN2,OSR2,INSIG2,PPARGC1B,MAB21L1,GJB5,LIMS2,LRIG3,ACACB,IL6R,IRX5,SLIT2,CRIP2,HOXD3,SLC6A4,FGF7,ABL1,EBF2,DLL1,HOXA6,OSR1,SAV1,KLF15,HPSE,PLXNA4,ANKRD54,CLDN1,ZBTB16,TOX,RARG,CCBE1,STAT6,HOXC4,PATZ1,GTF3C5,F2RL1,DLX3,GLIS2,DDR2,MFN2,ZFP36L1,PIK3R1,TIAM1,BATF2,OMD,MYC,IRX3,ZEB1,FBXW4,CTNS,SULF2,SHOX2,EEF2,RTN4RL1,HOXC10,LOX,TFE3,LRP4,DPYSL2,GDPD5,CLCN2,E2F4,SMG9,MCPH1,KIF27,FBXO7,NTN4,CD83,KDM2B,ZFP36L2,RAF1,PNP,NFIB,ZFAND5,QARS,ACVRL1,HTRA2,FLOT1,PPP3CB,BGLAP,MAP2K2,TGFBR2,NPRL3,CITED2,TRADD,PIR,LBH,LATS2	up_VSAP_EC
GO:0014020	0.0469	0.982	8	41	primary neural tube formation	BP	1	SHROOM3,BMP4,SEC24B,CITED2,SPINT1,LMO4,SPINT2,FZD3	up_ACOR_VSMC
GO:0003231	0.0469	0.982	8	48	cardiac ventricle development	BP	0.881	MEF2C,CHD7,DLL4,GATA3,PROX1,JAG1,HIF1A,LUZP1	up_VCAV_EC
GO:0006584	0.0469	0.993	3	11	catecholamine metabolic process	BP	0.881	GATA3,SNCAIP,GCH1	up_VCAV_EC
GO:0009712	0.0469	0.993	3	11	catechol-containing compound metabolic	BP	0.881	GATA3,SNCAIP,GCH1	up_VCAV_EC
GO:0003012	0.0469	0.995	2	90	muscle system process	BP	1	HTR7,NPNT	up_APUL_EC
GO:0001957	0.0469	0.999	1	2	intramembranous ossification	BP	1	AXIN2	up_AFEM_EC
GO:0032423	0.0469	0.999	1	2	regulation of mismatch repair	BP	1	AXIN2	up_AFEM_EC
GO:0036072	0.0469	0.999	1	2	direct ossification	BP	1	AXIN2	up_AFEM_EC
GO:0090208	0.0469	0.999	1	6	positive regulation of triglyceride metabolic	BP	1	ABHD5	up_VPOR_EC
GO:0060359	0.047	0.992	3	14	response to ammonium ion	BP	0.874	AGRN,GNAQ,HOMER1	down_VSAP_EC
GO:0002688	0.047	0.992	3	34	regulation of leukocyte chemotaxis	BP	1	CMKLR1,CCL5,ADAM17	up_AFEM_EC
GO:0022603	0.047	0.996	2	301	regulation of anatomical structure morphogenesis	BP	1	WNT7A,ESR1	up_AORT_VSMC

GO:0051494	0.047	0.995	2	44	negative regulation of cytoskeleton organization	BP	1	ARHGEF2,TWF1	up_VPOR_EC
GO:0021561	0.047	0.999	1	8	facial nerve development	BP	1	NRP2	down_ACOR_EC
GO:0021610	0.047	0.999	1	8	facial nerve morphogenesis	BP	1	NRP2	down_ACOR_EC
GO:0021692	0.047	0.999	1	2	cerebellar Purkinje cell layer morphogenesis	BP	1	DLL1	up_AFEM_EC
GO:0042711	0.047	0.999	1	2	maternal behavior	BP	1	AVPR1A	up_AFEM_EC
GO:0060746	0.047	0.999	1	2	parental behavior	BP	1	AVPR1A	up_AFEM_EC
GO:1903556	0.047	0.999	1	14	negative regulation of tumor necrosis factor superfamily cytokine	BP	1	NR1H4	up_VPUL_EC
GO:0086004	0.047	0.999	1	10	regulation of cardiac muscle cell contraction	BP	1	CAV1	up_VSAP_VSMC
GO:0050728	0.047031036	0.99161	3	31	negative regulation of inflammatory response	BP	1	NR1H4,FNDC4,PTGER4	up_ACOR_EC
GO:0033559	0.0471	0.987	5	21	unsaturated fatty acid metabolic process	BP	1	PTGES,DAGLB,ABHD16A,HPGD,ENSCAFG00000023820	up_VSAP_EC
GO:0010573	0.0471	0.993	3	9	vascular endothelial growth factor production	BP	1	HPSE,CCBE1,SULF2	up_VSAP_EC
GO:0010574	0.0471	0.993	3	9	regulation of vascular endothelial growth factor	BP	1	HPSE,CCBE1,SULF2	up_VSAP_EC
GO:0071560	0.0471	0.995	2	72	cellular response to transforming growth	BP	1	ACVRL1,ASPN	down_ACOR_EC
GO:0032402	0.0471	0.996	2	5	melanosome transport	BP	0.881	RAB11A,RAB27A	up_VCAV_EC
GO:0051904	0.0471	0.996	2	5	pigment granule transport	BP	0.881	RAB11A,RAB27A	up_VCAV_EC
GO:0036022	0.0471	0.997	2	4	limb joint morphogenesis	BP	1	OSR2,OSR1	up_VSAP_EC
GO:0002764	0.0471	0.995	2	80	immune response-regulating signaling	BP	1	TLR2,CAV1	up_VSAP_VSMC
GO:0000380	0.0471	0.999	1	22	alternative mRNA splicing, via spliceosome	BP	1	SRSF6	down_AFEM_EC
GO:0044771	0.0471	0.999	1	2	meiotic cell cycle phase transition	BP	1	OVOL1	up_AFEM_EC
GO:1901993	0.0471	0.999	1	2	regulation of meiotic cell cycle phase transition	BP	1	OVOL1	up_AFEM_EC
GO:1901994	0.0471	0.999	1	2	negative regulation of meiotic cell cycle phase	BP	1	OVOL1	up_AFEM_EC
GO:2001171	0.0471	0.999	1	2	positive regulation of ATP biosynthetic process	BP	1	PPARGC1A	up_AFEM_EC
GO:0021679	0.0471	0.999	1	2	cerebellar molecular layer development	BP	1	DLL1	up_AFEM_EC
GO:0021687	0.0471	0.999	1	2	cerebellar molecular layer morphogenesis	BP	1	DLL1	up_AFEM_EC
GO:0045607	0.0471	0.999	1	2	regulation of inner ear auditory receptor cell	BP	1	DLL1	up_AFEM_EC
GO:0045631	0.0471	0.999	1	2	regulation of mechanoreceptor	BP	1	DLL1	up_AFEM_EC
GO:0061181	0.0471	0.999	1	2	regulation of chondrocyte development	BP	1	AXIN2	up_AFEM_EC
GO:2000980	0.0471	0.999	1	2	regulation of inner ear receptor cell	BP	1	DLL1	up_AFEM_EC
GO:0002028	0.0471	0.999	1	19	regulation of sodium ion transport	BP	1	NETO1	up_VFEM_VSMC
GO:0034142	0.0471	0.999	1	14	toll-like receptor 4 signaling pathway	BP	1	NR1H4	up_VPUL_EC
GO:0060706	0.0471	0.999	1	11	cell differentiation involved in embryonic	BP	1	GJB5	up_VSAP_VSMC
GO:0040012	0.0472	0.971	26	303	regulation of locomotion	BP	0.874	ARHGEF2,VEGFA,SULF1,HSPA5,SERPINE1,SPARC,JCAD,DPYSL3,PRCP,MYO1C,PIK3C2A,MSN,HIF1A,PTPN2,ADAM17,STARD13,APOE,PTP4A1,MMP28,LYN,STRAP,NEXN,SDC4,ZMYND8,NRP1,USP14	down_VSAP_EC
GO:0032652	0.0472	0.989	4	18	regulation of interleukin-1 production	BP	0.881	TNFAIP3,TLR4,ARG2,HMGB1	up_VCAV_EC
GO:0060687	0.0472	0.997	2	4	regulation of branching involved in prostate gland	BP	1	BMP4,FGFR2	up_ACOR_VSMC
GO:0046823	0.0472	0.996	2	6	negative regulation of nucleocytoplasmic	BP	0.881	FERMT1,APOD	up_VCAV_EC
GO:0006083	0.0472	1	1	1	acetate metabolic process	BP	1	ACSS2	down_VCAV_EC
GO:0019427	0.0472	1	1	1	acetyl-CoA biosynthetic process from acetate	BP	1	ACSS2	down_VCAV_EC
GO:0006477	0.0472	0.999	1	2	protein sulfation	BP	1	TPST2	up_AFEM_EC

GO:0010663	0.0472	0.999	1	2	positive regulation of striated muscle cell	BP	1	CAMK2A	up_AFEM_EC
GO:0010666	0.0472	0.999	1	2	positive regulation of cardiac muscle cell	BP	1	CAMK2A	up_AFEM_EC
GO:0042415	0.0472	0.999	1	2	norepinephrine metabolic process	BP	1	MOXD1	up_AFEM_EC
GO:0042421	0.0472	0.999	1	2	norepinephrine biosynthetic process	BP	1	MOXD1	up_AFEM_EC
GO:0046813	0.0472	0.999	1	2	receptor-mediated virion attachment to host cell	BP	1	LRRC15	up_AFEM_EC
GO:1905907	0.0472	0.999	1	2	negative regulation of amyloid fibril formation	BP	1	CRYAB	up_AFEM_EC
GO:0048645	0.0472	0.999	1	33	animal organ formation	BP	1	EYA1	up_AFEM_VSMC
GO:0099173	0.0472	0.999	1	40	postsynapse organization	BP	1	WNT7A	up_AORT_VSMC
GO:0055083	0.0472	0.999	1	6	monovalent inorganic anion homeostasis	BP	1	HOXA3	up_VPOR_EC
GO:0003230	0.0472	0.999	1	14	cardiac atrium development	BP	1	TBX5	up_VPUL_EC
GO:0003209	0.0472	0.999	1	14	cardiac atrium morphogenesis	BP	1	TBX5	up_VPUL_EC
GO:1901862	0.0472	0.999	1	14	negative regulation of muscle tissue	BP	1	TBX5	up_VPUL_EC
GO:1903052	0.047291178	0.98856	4	51	positive regulation of proteolysis involved in cellular protein catabolic	BP	1	EDEM2,PIAS1,CLU,RNF114	up_ACOR_EC
GO:0007167	0.0473	0.989	4	274	enzyme linked receptor protein signaling pathway	BP	1	ACVRL1,LYN,SULF1,ASPN	down_ACOR_EC
GO:0030182	0.0473	0.989	4	327	neuron differentiation	BP	1	EMX2,HOXC10,IRX5,SHOX2	up_VSAP_VSMC
GO:0006954	0.0473	0.995	2	147	inflammatory response	BP	1	CYP26B1,C3AR1	up_VPOR_VSMC
GO:0007197	0.0473	0.999	1	2	adenylate cyclase-inhibiting G protein-coupled acetylcholine	BP	1	CHRM2	up_AFEM_EC
GO:0007207	0.0473	0.999	1	2	phospholipase C-activating G protein-coupled acetylcholine	BP	1	CHRM2	up_AFEM_EC
GO:0042670	0.0473	0.999	1	2	retinal cone cell differentiation	BP	1	THRB	up_AFEM_EC
GO:0046549	0.0473	0.999	1	2	retinal cone cell development	BP	1	THRB	up_AFEM_EC
GO:0072014	0.0473	0.999	1	2	proximal tubule development	BP	1	DLL1	up_AFEM_EC
GO:0072086	0.0473	0.999	1	2	specification of loop of Henle identity	BP	1	IRX3	up_AFEM_EC
GO:0030879	0.0473	0.999	1	34	mammary gland development	BP	1	HOXA9	up_AFEM_VSMC
GO:0048635	0.0473	0.999	1	14	negative regulation of muscle organ	BP	1	TBX5	up_VPUL_EC
GO:2000826	0.0473	0.999	1	14	regulation of heart morphogenesis	BP	1	TBX5	up_VPUL_EC
GO:0050778	0.0474	0.973	20	141	positive regulation of immune response	BP	1	TLR1,PLCG2,COCH,TP53BP1,ELF1,TLR3,CD274,F2RL1,C1S,PTPN22,C7,PRKCE,PARP9,PSEN1,DUSP22,NOD2,B2M,MAP3K7,PARK7,IL18R1	up_ACOR_VSMC
GO:0003012	0.0474	0.976	14	90	muscle system process	BP	1	TNNT2,ADRA1B,ACTN2,KCNE3,GATA5,HTR2B,PDE5A,DMPK,NPNT,TBX20,JUP,EDN1,GAA,DOCK5	up_ACOR_VSMC
GO:0006941	0.0474	0.983	7	36	striated muscle contraction	BP	1	TNNT2,ADRA1B,KCNE3,PDE5A,DMPK,JUP,GAA	up_ACOR_VSMC
GO:0006081	0.0474	0.989	4	27	cellular aldehyde metabolic process	BP	1	H6PD,PGLS,PARK7,RDH10	down_VCAV_EC
GO:0003416	0.0474	0.993	3	9	endochondral bone growth	BP	1	RARG,DDR2,TGFBR2	up_VSAP_EC
GO:0048596	0.0474	0.993	3	9	embryonic camera-type eye morphogenesis	BP	1	ZEB1,KDM2B,CITED2	up_VSAP_EC
GO:0009259	0.0474	0.995	2	166	ribonucleotide metabolic process	BP	1	SUCLG2,NDUFA10	down_AFEM_EC
GO:1990535	0.0474	0.997	2	4	neuron projection maintenance	BP	1	APP,PSEN1	up_ACOR_VSMC
GO:0015732	0.0474	0.997	2	4	prostaglandin transport	BP	0.883	SLCO3A1,NOS2	up_VCAV_EC
GO:0001764	0.0474	0.995	2	45	neuron migration	BP	1	ARHGEF2,SEMA3A	up_VPOR_EC
GO:0030035	0.0474	0.997	2	4	microspike assembly	BP	1	ABL1,FGD4	up_VSAP_EC
GO:0045088	0.0474	0.995	2	81	regulation of innate immune response	BP	1	TLR2,CAV1	up_VSAP_VSMC

GO:1990009	0.0474	0.999	1	2	retinal cell apoptotic process	BP	1	THRB	up_AFEM_EC
GO:0042098	0.0474	0.999	1	43	T cell proliferation	BP	1	ARG2	up_AORT_VSMC
GO:0036438	0.047470776	0.99943	1	2	maintenance of lens transparency	BP	1	CHMP4B	up_ACOR_EC
GO:0048869	0.0475	0.969	35	1119	cellular developmental process	BP	1	HOXA9,CMKLR1,IRX3,IRX5,HOXC10,LEP,LBH,GAS1,HOXA7,DLL1,VXN,PLXNA4,NEFH,TWIST2,THRB,LGI4,HCK,TMEM100,GREM1,NFE2L1,CAMK2A,CYLD,MYBL1,PTHLH,EVI2B,BVES,AXIN2,NAPEPLD,PPARGC1A,CDC42EP5,DMPK,LRRN2,FOSL2,KANK1,TNC	up_AFEM_EC
GO:0034101	0.0475	0.985	6	45	erythrocyte homeostasis	BP	0.874	CASP3,HIF1A,PTPN2,LYN,PRMT1,INHBA	down_VSAP_EC
GO:0051272	0.0475	0.988	4	170	positive regulation of cellular component	BP	1	C3AR1,ARHGEF2,SEMA3A,CCAR1	up_VPOR_EC
GO:0030516	0.0475	0.99	4	15	regulation of axon extension	BP	1	ABL1,PLXNA4,DPYSL2,TTL	up_VSAP_EC
GO:0002366	0.0475	0.995	2	65	leukocyte activation involved in immune	BP	1	LYN,STX4	down_ACOR_EC
GO:0043094	0.0475	0.996	2	7	cellular metabolic compound salvage	BP	1	ADK,AMPD2	down_VCAV_EC
GO:0007155	0.0475	1	1	324	cell adhesion	BP	1	GATA5	down_AFEM_VSMC
GO:0060926	0.0475	0.999	1	2	cardiac pacemaker cell development	BP	1	BVES	up_AFEM_EC
GO:0060920	0.0475	0.999	1	2	cardiac pacemaker cell differentiation	BP	1	BVES	up_AFEM_EC
GO:0021517	0.0475	0.999	1	12	ventral spinal cord development	BP	1	HOXC10	up_VSAP_VSMC
GO:1905475	0.047504543	0.98848	4	52	regulation of protein localization to membrane	BP	1	KCNB1,PLS1,LRRC15,LRP4	up_ACOR_EC
GO:0030154	0.0476	0.991	4	1040	cell differentiation	BP	1	SLITRK5,LIMK1,GATA5,LRRC17	up_VCAV_VSMC
GO:0002684	0.0476	0.992	3	239	positive regulation of immune system process	BP	1	NR1H4,EVI2B,CCL24	up_VPUL_EC
GO:0045988	0.0476	0.997	2	4	negative regulation of striated muscle	BP	1	KCNE3,PDE5A	up_ACOR_VSMC
GO:0006935	0.0476	0.995	2	143	chemotaxis	BP	1	CHL1,C3AR1	up_VPOR_VSMC
GO:0042330	0.0476	0.995	2	143	taxis	BP	1	CHL1,C3AR1	up_VPOR_VSMC
GO:1903827	0.0476	0.999	1	164	regulation of cellular protein localization	BP	1	JUP	down_VFEM_VSMC
GO:0045777	0.0476	0.999	1	2	positive regulation of blood pressure	BP	1	TBXA2R	up_AFEM_EC
GO:0030641	0.0476	0.999	1	34	regulation of cellular pH	BP	1	CA2	up_VCAV_VSMC
GO:1990138	0.0476	0.999	1	35	neuron projection extension	BP	1	LIMK1	up_VCAV_VSMC
GO:0055117	0.0476	0.999	1	21	regulation of cardiac muscle contraction	BP	1	NKX2-5	up_VPOR_VSMC
GO:0048333	0.0476	0.999	1	12	mesodermal cell differentiation	BP	1	EYA1	up_VFEM_EC
GO:0008585	0.0476	0.999	1	24	female gonad development	BP	1	SLIT2	up_VPUL_VSMC
GO:0046545	0.0476	0.999	1	24	development of primary female sexual	BP	1	SLIT2	up_VPUL_VSMC
GO:0009593	0.0476	0.999	1	11	detection of chemical stimulus	BP	1	TLR2	up_VSAP_VSMC
GO:0010466	0.047635885	0.99144	3	32	negative regulation of peptidase activity	BP	1	PCSK1N,IFI6,CST3	up_ACOR_EC
GO:0010951	0.047635885	0.99144	3	32	negative regulation of endopeptidase activity	BP	1	PCSK1N,IFI6,CST3	up_ACOR_EC
GO:0046635	0.0477	0.99	4	15	positive regulation of alpha-beta T cell	BP	1	ZBTB16,CD83,PNP,TGFBR2	up_VSAP_EC
GO:0010453	0.0477	0.993	3	9	regulation of cell fate commitment	BP	1	BMP4,FGFR2,LMO4	up_ACOR_VSMC
GO:0002483	0.0477	0.997	2	4	antigen processing and presentation of endogenous peptide	BP	1	B2M,ERAP1	up_ACOR_VSMC
GO:0019885	0.0477	0.997	2	4	antigen processing and presentation of endogenous peptide	BP	1	B2M,ERAP1	up_ACOR_VSMC
GO:0090196	0.0477	0.997	2	4	regulation of chemokine secretion	BP	1	MBP,F2RL1	up_ACOR_VSMC
GO:0098810	0.0477	0.997	2	4	neurotransmitter reuptake	BP	1	CLN8,PARK7	up_ACOR_VSMC
GO:0051181	0.0477	0.999	1	11	cofactor transport	BP	1	PDPN	down_ACOR_EC

GO:0010042	0.0477	0.999	1	2	response to manganese ion	BP	1	FIBIN	up_AFEM_EC
GO:0086036	0.0477	0.999	1	2	regulation of cardiac muscle cell membrane	BP	1	FXYD1	up_AFEM_EC
GO:1903542	0.0477	0.999	1	2	negative regulation of exosomal secretion	BP	1	RAB7A	up_AFEM_EC
GO:0009409	0.0477	0.999	1	13	response to cold	BP	1	PCSK1N	up_APUL_EC
GO:0007020	0.0477	0.999	1	6	microtubule nucleation	BP	1	MZT1	up_VPOR_EC
GO:0021885	0.0477	0.999	1	24	forebrain cell migration	BP	1	SLIT2	up_VPUL_VSMC
GO:0048333	0.0477	0.999	1	12	mesodermal cell differentiation	BP	1	HOXA11	up_VSAP_VSMC
GO:1901861	0.0478	0.983	7	41	regulation of muscle tissue development	BP	0.884	EPHB1,MEF2C,EDN1,BMP2,CREB1,NR1D2,CDON	up_VCAV_EC
GO:0010977	0.0478	0.986	5	33	negative regulation of neuron projection	BP	0.874	DPYSL3,IFRD1,APOE,NRP1,YWHAH	down_VSAP_EC
GO:1901135	0.0478	0.992	3	390	carbohydrate derivative metabolic process	BP	1	EXTL2,SUCLG2,NDUFA10	down_AFEM_EC
GO:0033363	0.0478	0.993	3	10	secretory granule organization	BP	0.884	NECTIN2,ABCA1,CREB1	up_VCAV_EC
GO:0048863	0.0478	0.995	2	70	stem cell differentiation	BP	1	NRP2,HOXA7	down_ACOR_EC
GO:0010657	0.0478	0.995	2	15	muscle cell apoptotic process	BP	1	CAMK2A,DMPK	up_AFEM_EC
GO:1900744	0.0478	0.995	2	15	regulation of p38MAPK cascade	BP	1	LEP,CYLD	up_AFEM_EC
GO:1901725	0.0478	0.997	2	4	regulation of histone deacetylase activity	BP	0.884	PRKD2,KDM5A	up_VCAV_EC
GO:0010862	0.0478	0.999	1	12	positive regulation of pathway-restricted SMAD protein phosphorylation	BP	1	ACVRL1	down_ACOR_EC
GO:0022610	0.0478	1	1	326	biological adhesion	BP	1	GATA5	down_AFEM_VSMC
GO:0043242	0.0478	0.999	1	21	negative regulation of protein complex	BP	1	TAOK1	up_AORT_EC
GO:0060038	0.0478	0.999	1	18	cardiac muscle cell proliferation	BP	1	NKX2-5	up_VPOR_VSMC
GO:0045844	0.0478	0.999	1	24	positive regulation of striated muscle tissue	BP	1	TBX5	up_VPUL_VSMC
GO:0048636	0.0478	0.999	1	24	positive regulation of muscle organ	BP	1	TBX5	up_VPUL_VSMC
GO:0009792	0.0479	0.979	10	231	embryo development ending in birth or egg	BP	1	TBX15,HOXA9,IRX5,XYLT1,HOXA7,DLL1,TMEM100,HOXA6,LUZP1,AXIN2	up_AFEM_EC
GO:0050920	0.0479	0.981	8	53	regulation of chemotaxis	BP	0.884	CXCL8,CXCR4,PDGFB,MMP28,ANGPT2,PRKD2,SWAP70,HMGB1	up_VCAV_EC
GO:0010647	0.0479	0.987	5	460	positive regulation of cell communication	BP	1	NETO1,SALL1,TLR2,CAV1,SHOX2	up_VSAP_VSMC
GO:0070306	0.0479	0.992	3	11	lens fiber cell differentiation	BP	0.884	TMOD1,PROX1,SKIL	up_VCAV_EC
GO:0045655	0.0479	0.993	3	9	regulation of monocyte differentiation	BP	1	HOXA7,ZFP36L1,MYC	up_VSAP_EC
GO:0051650	0.0479	0.995	2	65	establishment of vesicle localization	BP	1	SPG11,STX4	down_ACOR_EC
GO:2000027	0.0479	0.995	2	72	regulation of animal organ morphogenesis	BP	1	SULF1,ASPN	down_ACOR_EC
GO:0070601	0.0479	0.996	2	6	centromeric sister chromatid cohesion	BP	0.874	NAA10,CTNNB1	down_VSAP_EC
GO:0030857	0.0479	0.995	2	15	negative regulation of epithelial cell	BP	1	HOXA7,DLL1	up_AFEM_EC
GO:0034104	0.0479	0.999	1	2	negative regulation of tissue remodeling	BP	1	GREM1	up_AFEM_EC
GO:0048813	0.0479	0.999	1	39	dendrite morphogenesis	BP	1	WNT7A	up_AORT_VSMC
GO:0099504	0.0479	0.999	1	42	synaptic vesicle cycle	BP	1	WNT7A	up_AORT_VSMC
GO:0016242	0.0479	0.999	1	13	negative regulation of macroautophagy	BP	1	LZTS1	up_APUL_EC
GO:0060043	0.0479	0.999	1	14	regulation of cardiac muscle cell proliferation	BP	1	TBX5	up_VPUL_EC
GO:0046689	0.047916513	0.99942	1	2	response to mercury ion	BP	1	AQP1	up_ACOR_EC
GO:0009798	0.047945215	0.99138	3	31	axis specification	BP	1	FZD5,SMAD6,LRP4	up_ACOR_EC
GO:0055080	0.048	0.971	24	176	cation homeostasis	BP	1	ADRA1B,PLCG2,SLC26A7,HTR2B,CNNM2,MICU2,ITPR1,F2RL1,EGLN1,DMPK,ATP7A,PRKCE,APP,PSEN1,CHD7,DMXL1,EDN1,B2M,DISC1,HERPUD1,WFS1,ATP2C1,SLC8A1,STC2	up_ACOR_VSMC

GO:0050731	0.048	0.984	6	44	positive regulation of peptidyl-tyrosine	BP	0.874	ARHGEF2,VEGFA,YES1,ADAM17,LYN,NRP1	down_VSAP_EC
GO:0044264	0.048	0.986	5	32	cellular polysaccharide metabolic process	BP	0.874	ENPP1,ENSCAFG00000032470,PPP1R3C,GSK3B,EXT1	down_VSAP_EC
GO:0060421	0.048	0.99	4	14	positive regulation of heart growth	BP	1	FGFR2,TBX20,TBX5,EDN1	up_ACOR_VSMC
GO:0051043	0.048	0.996	2	7	regulation of membrane protein ectodomain	BP	1	TIMP2,PACSIN3	down_VCAV_EC
GO:0009713	0.048	0.996	2	5	catechol-containing compound biosynthetic	BP	0.884	GATA3,GCH1	up_VCAV_EC
GO:0042423	0.048	0.996	2	5	catecholamine biosynthetic process	BP	0.884	GATA3,GCH1	up_VCAV_EC
GO:1900154	0.048	0.999	1	2	regulation of bone trabecula formation	BP	1	GREM1	up_AFEM_EC
GO:1900155	0.048	0.999	1	2	negative regulation of bone trabecula formation	BP	1	GREM1	up_AFEM_EC
GO:0001656	0.048	0.999	1	35	metanephros development	BP	1	EYA1	up_AFEM_VSMC
GO:0002224	0.048	0.999	1	34	toll-like receptor signaling pathway	BP	1	TLR2	up_AFEM_VSMC
GO:0060612	0.048	0.999	1	14	adipose tissue development	BP	1	NR1H4	up_VPUL_EC
GO:0003208	0.048	0.999	1	24	cardiac ventricle morphogenesis	BP	1	TBX5	up_VPUL_VSMC
GO:0050702	0.048009948	0.99524	2	13	interleukin-1 beta secretion	BP	1	FZD5,NOD2	up_ACOR_EC
GO:0007160	0.0481	0.98	9	56	cell-matrix adhesion	BP	0.884	TEK,ADAMTS9,CD34,FERMT1,SLK,APOD,JAG1,ITGA6,ENSCAFG00000002015	up_VCAV_EC
GO:0035966	0.0481	0.984	6	47	response to topologically incorrect protein	BP	0.874	HSPA5,ERO1A,PTPN2,DDIT3,ATF3,CREB3L2	down_VSAP_EC
GO:0006939	0.0481	0.985	6	32	smooth muscle contraction	BP	0.884	EDNRB,DOCK4,PDE4D,EDN1,GUCY1A1,P2RX2	up_VCAV_EC
GO:0071360	0.0481	0.993	3	9	cellular response to exogenous dsRNA	BP	1	PQBP1,FLOT1,MAVS	up_VSAP_EC
GO:0006952	0.0481	0.996	2	319	defense response	BP	1	ARG2,ESR1	up_AORT_VSMC
GO:0070970	0.0481	0.997	2	4	interleukin-2 secretion	BP	1	ABL1,PNP	up_VSAP_EC
GO:0099563	0.0481	0.997	2	4	modification of synaptic structure	BP	1	WASF3,ABL1	up_VSAP_EC
GO:0001974	0.0481	0.999	1	13	blood vessel remodeling	BP	1	HOXA3	up_APUL_EC
GO:0000165	0.0482	0.97	27	236	MAPK cascade	BP	0.884	EPHB1,TEK,KIT,MAP3K11,TNIP1,PDGFB,SH2D3C,MEF2C,ATF3,RAPGEF1,TLR4,EDN1,MAP3K3,BMP2,MAPK13,ITPKB,MADD,PAK2,PRKD2,TLR3,RAPGEF2,TAOK1,HMGB1,RIPK1,PDGFD,JCAD,CDON	up_VCAV_EC
GO:0006913	0.0482	0.976	13	124	nucleocytoplasmic transport	BP	0.874	PRICKLE1,PTPN14,PTPN11,PPP1R12A,IWS1,ZPR1,SMG7,DDX39B,KPNB1,KPNA4,GSK3B,KPNA1,CDKN1A	down_VSAP_EC
GO:0051169	0.0482	0.976	13	124	nuclear transport	BP	0.874	PRICKLE1,PTPN14,PTPN11,PPP1R12A,IWS1,ZPR1,SMG7,DDX39B,KPNB1,KPNA4,GSK3B,KPNA1,CDKN1A	down_VSAP_EC
GO:2000112	0.0482	0.986	6	1119	regulation of cellular macromolecule biosynthetic process	BP	1	EYA1,HOXA10,HOXA9,EMX2,HOXA11,SHOX2	up_VFEM_VSMC
GO:0048699	0.0482	0.986	5	371	generation of neurons	BP	1	LYN,ACSL4,SPG11,NRP2,NEDD4	down_ACOR_EC
GO:0120036	0.0482	0.986	5	373	plasma membrane bounded cell projection	BP	1	LYN,SPG11,PDPN,NRP2,NEDD4	down_ACOR_EC
GO:0050654	0.0482	0.995	2	15	chondroitin sulfate proteoglycan metabolic	BP	1	XYLT1,CSGALNACT2	up_AFEM_EC
GO:0001568	0.0482	0.995	2	252	blood vessel development	BP	1	C6,TGFBI	up_VCAV_VSMC
GO:0050853	0.0482	0.999	1	10	B cell receptor signaling pathway	BP	1	LYN	down_ACOR_EC
GO:0010257	0.0482	0.999	1	25	NADH dehydrogenase complex assembly	BP	1	NDUFA10	down_AFEM_EC
GO:0032981	0.0482	0.999	1	25	mitochondrial respiratory chain complex I assembly	BP	1	NDUFA10	down_AFEM_EC
GO:0043086	0.0482	0.999	1	170	negative regulation of catalytic activity	BP	1	CST3	down_AORT_EC
GO:1901699	0.0482	0.999	1	164	cellular response to nitrogen compound	BP	1	JUP	down_VFEM_VSMC
GO:1900086	0.0482	0.999	1	2	positive regulation of peptidyl-tyrosine	BP	1	GREM1	up_AFEM_EC
GO:1900157	0.0482	0.999	1	2	regulation of bone mineralization involved in	BP	1	GREM1	up_AFEM_EC

GO:1900158	0.0482	0.999	1	2	negative regulation of bone mineralization involved in bone	BP	1	GREM1	up_AFEM_EC
GO:0031113	0.0482	0.999	1	13	regulation of microtubule polymerization	BP	1	TUBB4A	up_APUL_EC
GO:0032526	0.0482	0.999	1	19	response to retinoic acid	BP	1	CYP26B1	up_VPOR_VSMC
GO:0040019	0.0482	0.999	1	12	positive regulation of embryonic development	BP	1	SIX1	up_VFEM_EC
GO:0071300	0.0482	0.999	1	12	cellular response to retinoic acid	BP	1	CYP26B1	up_VFEM_EC
GO:0070266	0.0482	0.999	1	14	necroptotic process	BP	1	ARHGEF2	up_VPUL_EC
GO:0031077	0.048216136	0.99941	1	2	post-embryonic camera-type eye development	BP	1	FZD5	up_ACOR_EC
GO:0044238	0.0483	0.986	11	3383	primary metabolic process	BP	1	ETFA,IVNS1ABP,EXTL2,DARS,SUCLG2,EIF3M,SRSF6,USP33,GABPA,NDUFA10,TSEN15	down_AFEM_EC
GO:0034138	0.0483	0.993	3	9	toll-like receptor 3 signaling pathway	BP	1	TLR3,F2RL1,PTPN22	up_ACOR_VSMC
GO:0043537	0.0483	0.995	2	15	negative regulation of blood vessel endothelial	BP	1	ANGPT4,TBXA2R	up_AFEM_EC
GO:0003407	0.0483	0.995	2	15	neural retina development	BP	1	IRX5,THRB	up_AFEM_EC
GO:1902369	0.0483	0.995	2	15	negative regulation of RNA catabolic process	BP	1	TENT5C,AXIN2	up_AFEM_EC
GO:0061158	0.0483	0.997	2	4	3'-UTR-mediated mRNA destabilization	BP	1	ZFP36L1,ZFP36L2	up_VSAP_EC
GO:0042391	0.0483	0.995	2	81	regulation of membrane potential	BP	1	NETO1,CAV1	up_VSAP_VSMC
GO:0061333	0.0483	0.999	1	35	renal tubule morphogenesis	BP	1	EYA1	up_AFEM_VSMC
GO:0110110	0.0483	0.999	1	34	positive regulation of animal organ	BP	1	GATA5	up_VCAV_VSMC
GO:0021854	0.0483	0.999	1	6	hypothalamus development	BP	1	SEMA3A	up_VPOR_EC
GO:0048538	0.0483	0.999	1	20	thymus development	BP	1	HOXA3	up_VPOR_VSMC
GO:0042063	0.0484	0.982	7	67	gliogenesis	BP	1	LRP1,NAB2,NDN,AKT2,CSPG4,GLI3,CDH2	down_VCAV_EC
GO:0048514	0.0484	0.991	3	214	blood vessel morphogenesis	BP	1	HOXA3,NTRK2,HOXA1	up_APUL_EC
GO:0030029	0.0484	0.991	3	239	actin filament-based process	BP	1	MYBPC2,CCL24,ARHGEF2	up_VPUL_EC
GO:0048713	0.0484	0.996	2	6	regulation of oligodendrocyte	BP	0.874	SLC45A3,CTNNB1	down_VSAP_EC
GO:0031960	0.0484	0.995	2	15	response to corticosteroid	BP	1	FIBIN,NR3C1	up_AFEM_EC
GO:0051384	0.0484	0.995	2	15	response to glucocorticoid	BP	1	FIBIN,NR3C1	up_AFEM_EC
GO:0010454	0.0484	1	1	1	negative regulation of cell fate commitment	BP	1	LOXL3	down_VCAV_EC
GO:0018057	0.0484	1	1	1	peptidyl-lysine oxidation	BP	1	LOXL3	down_VCAV_EC
GO:1905590	0.0484	1	1	1	fibronectin fibril organization	BP	1	LOXL3	down_VCAV_EC
GO:2000328	0.0484	1	1	1	regulation of T-helper 17 cell lineage commitment	BP	1	LOXL3	down_VCAV_EC
GO:2000329	0.0484	1	1	1	negative regulation of T-helper 17 cell lineage	BP	1	LOXL3	down_VCAV_EC
GO:1902626	0.0484	1	1	1	assembly of large subunit precursor of preribosome	BP	0.874	RSL24D1	down_VSAP_EC
GO:0007179	0.0484	0.999	1	57	transforming growth factor beta receptor	BP	1	TGFBR1	down_VSAP_VSMC
GO:0030902	0.0484	0.999	1	41	hindbrain development	BP	1	WNT7A	up_AORT_VSMC
GO:0060420	0.0484	0.999	1	18	regulation of heart growth	BP	1	NKX2-5	up_VPOR_VSMC
GO:0072283	0.0484	0.999	1	12	metanephric renal vesicle morphogenesis	BP	1	SALL1	up_VSAP_VSMC
GO:0015669	0.048445871	0.9994	1	2	gas transport	BP	1	AQP1	up_ACOR_EC
GO:0015670	0.048445871	0.9994	1	2	carbon dioxide transport	BP	1	AQP1	up_ACOR_EC
GO:0044089	0.0485	0.974	16	170	positive regulation of cellular component biogenesis	BP	0.874	VEGFA,FSCN1,DPYSL3,MSN,DYNLL1,ACTR3,CTNNB1,ANLN,ENSCAFG00000002015,GSK3B,GBP5,SH3GLB1,SDC4,HTT,LRRTM2,NRP1	down_VSAP_EC
GO:0001558	0.0485	0.991	3	101	regulation of cell growth	BP	1	SEMA3A,FBP1,RNF6	up_VPOR_EC

GO:0002443	0.0485	0.995	2	71	leukocyte mediated immunity	BP	1	LYN,STX4	down_ACOR_EC
GO:0045597	0.0485	0.995	2	253	positive regulation of cell differentiation	BP	1	LIMK1,GATA5	up_VCAV_VSMC
GO:0001501	0.0485	0.995	2	178	skeletal system development	BP	1	HOXA3,HOXB5	up_VPUL_VSMC
GO:0003008	0.0485	1	1	336	system process	BP	1	GATA5	down_AFEM_VSMC
GO:0030809	0.0485	0.999	1	6	negative regulation of nucleotide biosynthetic	BP	1	FBP1	up_VPOR_EC
GO:1900372	0.0485	0.999	1	6	negative regulation of purine nucleotide	BP	1	FBP1	up_VPOR_EC
GO:2001170	0.0485	0.999	1	6	negative regulation of ATP biosynthetic process	BP	1	FBP1	up_VPOR_EC
GO:0007613	0.0485	0.999	1	20	memory	BP	1	NETO1	up_VFEM_VSMC
GO:0021766	0.0485	0.999	1	20	hippocampus development	BP	1	EMX2	up_VFEM_VSMC
GO:0003148	0.048501708	0.99516	2	13	outflow tract septum morphogenesis	BP	1	SMAD6,NRP1	up_ACOR_EC
GO:0010035	0.0486	0.975	14	107	response to inorganic substance	BP	0.884	TNFAIP3,MEF2C,PPP3CA,TJP1,MAPK13,ITPKB,TIMELESS,GUCY1B1,CREB1,GCH1,BLM,RIPK1,HIF1A,ATRX	up_VCAV_EC
GO:0043902	0.0486	0.984	6	45	positive regulation of multi-organism process	BP	0.874	P4HB,SMARCA4,CNOT7,APOE,RAD23A,INHBA	down_VSAP_EC
GO:0032101	0.0486	0.991	3	173	regulation of response to external stimulus	BP	1	LYN,PDPN,STX4	down_ACOR_EC
GO:0072102	0.0486	0.997	2	4	glomerulus morphogenesis	BP	0.884	MEF2C,MTSS1	up_VCAV_EC
GO:0002009	0.0486	0.995	2	177	morphogenesis of an epithelium	BP	1	TBX5,SLIT2	up_VPUL_VSMC
GO:0051241	0.0486	1	1	336	negative regulation of multicellular organismal	BP	1	GATA5	down_AFEM_VSMC
GO:0048863	0.0486	0.999	1	70	stem cell differentiation	BP	1	TBX5	up_APUL_VSMC
GO:0070509	0.0486	0.999	1	21	calcium ion import	BP	1	TRPC4	up_VPOR_VSMC
GO:0071407	0.0487	0.974	17	129	cellular response to organic cyclic compound	BP	0.884	PDGFB,TMEM173,PDE4D,MEF2C,TIPARP,RAPGEF1,RALB,TFPI,BMP2,RIOK3,PAK2,SMARCA4,RAPGEF2,CTNNA1,BLM,UFSP2,KDM3A	up_VCAV_EC
GO:0002244	0.0487	0.983	7	35	hematopoietic progenitor cell differentiation	BP	1	BMP4,CITED2,CHD2,PSEN1,JAM3,ENSCAFG00000003530,BVES	up_ACOR_VSMC
GO:0023056	0.0487	0.986	5	462	positive regulation of signaling	BP	1	NETO1,SALL1,TLR2,CAV1,SHOX2	up_VSAP_VSMC
GO:0050868	0.0487	0.988	4	28	negative regulation of T cell activation	BP	1	PRNP,SDC4,GLI3,LOXL3	down_VCAV_EC
GO:0060976	0.0487	0.989	4	22	coronary vasculature development	BP	0.874	PRICKLE1,LTBP1,ADAMTS6,NRP1	down_VSAP_EC
GO:0071260	0.0487	0.989	4	19	cellular response to mechanical stimulus	BP	0.884	TMEM150C,CASP8,TLR3,BCL10	up_VCAV_EC
GO:0051252	0.0487	0.994	3	1051	regulation of RNA metabolic process	BP	1	GATA5,TGFBR1,MEIS2	down_VSAP_VSMC
GO:0043649	0.0487	0.997	2	4	dicarboxylic acid catabolic process	BP	1	GOT1,ADHFE1	up_VSAP_EC
GO:0050820	0.0487	0.999	1	10	positive regulation of coagulation	BP	1	PDPN	down_ACOR_EC
GO:0071333	0.0487	0.999	1	25	cellular response to glucose stimulus	BP	1	RAB11B	down_VPOR_EC
GO:0010835	0.0487	0.999	1	6	regulation of protein ADP-ribosylation	BP	1	PUM3	up_VPOR_EC
GO:0060421	0.0487	0.999	1	14	positive regulation of heart growth	BP	1	TBX5	up_VPUL_EC
GO:0072606	0.0487	0.999	1	12	interleukin-8 secretion	BP	1	TLR2	up_VSAP_VSMC
GO:0098884	0.04875227	0.99939	1	2	postsynaptic neurotransmitter	BP	1	CALY	up_ACOR_EC
GO:0099590	0.04875227	0.99939	1	2	neurotransmitter receptor internalization	BP	1	CALY	up_ACOR_EC
GO:0140239	0.04875227	0.99939	1	2	postsynaptic endocytosis	BP	1	CALY	up_ACOR_EC
GO:0034332	0.048771537	0.99116	3	32	adherens junction organization	BP	1	ARHGAP6,ACTN2,NRP1	up_ACOR_EC
GO:0090398	0.0488	0.987	5	24	cellular senescence	BP	0.884	PLK2,ARG2,MAP3K3,SMC5,VASH1	up_VCAV_EC
GO:1901576	0.0488	0.993	4	1877	organic substance biosynthetic process	BP	1	AK5,GATA5,TGFBR1,MEIS2	down_VSAP_VSMC
GO:0071461	0.0488	1	1	1	cellular response to redox state	BP	1	VASN	down_VCAV_EC

GO:0051216	0.0488	0.999	1	70	cartilage development	BP	1	HOXA3	up_APUL_VSMC
GO:0050996	0.0488	0.999	1	6	positive regulation of lipid catabolic process	BP	1	ABHD5	up_VPOR_EC
GO:0060795	0.0488	0.999	1	12	cell fate commitment involved in formation of	BP	1	HOXA11	up_VSAP_VSMC
GO:0034644	0.0489	0.984	6	32	cellular response to UV	BP	1	NEDD4,AURKB,PCNA,NOC2L,POLH,CDC25A	down_ACOR_VSMC
GO:0090342	0.0489	0.989	4	17	regulation of cell aging	BP	0.884	PLK2,ARG2,MAP3K3,VASH1	up_VCAV_EC
GO:0040020	0.0489	0.992	3	10	regulation of meiotic nuclear division	BP	1	FBXO5,CDC20,TTK	down_ACOR_VSMC
GO:0003215	0.0489	0.993	3	9	cardiac right ventricle morphogenesis	BP	0.884	CHD7,GATA3,JAG1	up_VCAV_EC
GO:0050808	0.0489	0.995	2	93	synapse organization	BP	1	SLITRK5,NTRK2	up_APUL_EC
GO:0048666	0.0489	0.995	2	258	neuron development	BP	1	SLITRK5,LIMK1	up_VCAV_VSMC
GO:0006040	0.0489	0.999	1	12	amino sugar metabolic process	BP	1	CSGALNACT1	down_ACOR_EC
GO:1900180	0.049	0.98	9	50	regulation of protein localization to nucleus	BP	1	LEP,FERMT1,BMP4,CDKN1A,PTPN22,PARP9,PSEN1,JUP,PARK7	up_ACOR_VSMC
GO:0051046	0.049	0.991	3	174	regulation of secretion	BP	1	LYN,ACSL3,STX4	down_ACOR_EC
GO:0001945	0.049	0.993	3	9	lymph vessel development	BP	1	CCBE1,ACVRL1,PPP3CB	up_VSAP_EC
GO:0001946	0.049	0.993	3	9	lymphangiogenesis	BP	1	CCBE1,ACVRL1,PPP3CB	up_VSAP_EC
GO:0036303	0.049	0.993	3	9	lymph vessel morphogenesis	BP	1	CCBE1,ACVRL1,PPP3CB	up_VSAP_EC
GO:0002313	0.049	0.997	2	4	mature B cell differentiation involved in	BP	0.884	MFNG,PLCG2	up_VCAV_EC
GO:0002335	0.049	0.997	2	4	mature B cell differentiation	BP	0.884	MFNG,PLCG2	up_VCAV_EC
GO:0032534	0.049	0.997	2	4	regulation of microvillus assembly	BP	0.884	PODXL,FSCN1	up_VCAV_EC
GO:1900272	0.049	0.997	2	4	negative regulation of long-term synaptic	BP	1	FAM107A,ABL1	up_VSAP_EC
GO:0090596	0.049	0.995	2	84	sensory organ morphogenesis	BP	1	SALL1,IRX5	up_VSAP_VSMC
GO:1902305	0.049	0.999	1	12	regulation of sodium ion transmembrane transport	BP	1	NEDD4	down_ACOR_EC
GO:0018105	0.049	0.999	1	116	peptidyl-serine phosphorylation	BP	1	PLCL1	down_VFEM_EC
GO:0070168	0.049	0.999	1	6	negative regulation of biomineral tissue	BP	1	ASPN	up_VPOR_EC
GO:0001959	0.0491	0.986	5	33	regulation of cytokine-mediated signaling	BP	0.874	NR1H4,PTPN2,ADAM17,CNOT7,CDC37	down_VSAP_EC
GO:0034138	0.0491	0.993	3	9	toll-like receptor 3 signaling pathway	BP	1	F2RL1,UNC93B1,FLOT1	up_VSAP_EC
GO:0061430	0.0491	0.995	2	8	bone trabecula morphogenesis	BP	1	THBS3,GREM1	down_VCAV_EC
GO:0071674	0.0491	0.999	1	23	mononuclear cell migration	BP	1	C3AR1	up_VPOR_VSMC
GO:0046939	0.0491	0.999	1	19	nucleotide phosphorylation	BP	1	FBP1	up_VFEM_VSMC
GO:0051781	0.0492	0.989	4	18	positive regulation of cell division	BP	1	AURKB,CDC6,NUP62,FGF5	down_ACOR_VSMC
GO:0006509	0.0492	0.989	4	15	membrane protein ectodomain proteolysis	BP	1	PTPN3,DAG1,PSEN1,ERAP1	up_ACOR_VSMC
GO:0010648	0.0492	0.988	4	407	negative regulation of cell communication	BP	1	NR1H4,PTGER2,FLRT3,ARHGEF2	up_VPUL_EC
GO:0045123	0.0492	0.992	3	11	cellular extravasation	BP	0.884	SELP,SELE,SELL	up_VCAV_EC
GO:0040008	0.0492	0.995	2	179	regulation of growth	BP	1	TBX5,SLIT2	up_VPUL_VSMC
GO:0000165	0.049299175	0.977	11	236	MAPK cascade	BP	1	SOX9,NDRG2,FZD5,DUSP26,NOD2,PAK3,TNFRSF19,MAPK8IP2,SH3RF3,NRP1,PRKCD	up_ACOR_EC
GO:0071363	0.0493	0.974	15	188	cellular response to growth factor stimulus	BP	1	PDGFRB,SMAD9,DCN,NDN,VASN,FBN1,AKT1S1,CHRDL1,NR3C1,ASPN,ZFP36L1,FUT8,SHC1,GREM1,RGMA	down_VCAV_EC
GO:0007405	0.0493	0.989	4	21	neuroblast proliferation	BP	0.874	ARHGEF2,HIF1A,CTNNB1,DISC1	down_VSAP_EC
GO:0060379	0.0493	0.996	2	6	cardiac muscle cell myoblast differentiation	BP	0.874	PRICKLE1,REST	down_VSAP_EC
GO:0001880	0.0493	1	1	1	Mullerian duct regression	BP	1	BMPR1A	down_VCAV_EC
GO:0048352	0.0493	1	1	1	paraxial mesoderm structural organization	BP	1	BMPR1A	down_VCAV_EC

GO:0060897	0.0493	1	1	1	neural plate regionalization	BP	1	BMPR1A	down_VCAV_EC
GO:1904412	0.0493	1	1	1	regulation of cardiac ventricle development	BP	1	BMPR1A	down_VCAV_EC
GO:1904414	0.0493	1	1	1	positive regulation of cardiac ventricle	BP	1	BMPR1A	down_VCAV_EC
GO:1905285	0.0493	1	1	1	fibrous ring of heart morphogenesis	BP	1	BMPR1A	down_VCAV_EC
GO:0021998	0.0493	1	1	1	neural plate mediolateral regionalization	BP	1	BMPR1A	down_VCAV_EC
GO:0048338	0.0493	1	1	1	mesoderm structural organization	BP	1	BMPR1A	down_VCAV_EC
GO:0060896	0.0493	1	1	1	neural plate pattern specification	BP	1	BMPR1A	down_VCAV_EC
GO:0072676	0.0493	0.999	1	25	lymphocyte migration	BP	1	HSD3B7	down_VPOR_EC
GO:1900078	0.0493	0.999	1	13	positive regulation of cellular response to	BP	1	NR1H4	up_APUL_EC
GO:0033690	0.0494	0.997	2	4	positive regulation of osteoblast proliferation	BP	1	ABL1,HPSE	up_VSAP_EC
GO:0060429	0.0494	1	1	341	epithelium development	BP	1	GATA5	down_AFEM_VSMC
GO:0008626	0.0494	1	1	1	granzyme-mediated apoptotic signaling	BP	0.874	BNIP3	down_VSAP_EC
GO:0050710	0.0494	0.999	1	13	negative regulation of cytokine secretion	BP	1	NR1H4	up_APUL_EC
GO:1901700	0.0495	0.975	14	364	response to oxygen-containing compound	BP	1	LEP,CRY2,CD40,CRYAB,GPAM,FIBIN,CHRM2,AVPR1A,CAMK2A,NR3C1,TBXA2R,ADAM17,KANK1,SOD1	up_AFEM_EC
GO:0046822	0.0495	0.982	7	53	regulation of nucleocytoplasmic	BP	0.874	PTPN14,PTPN11,PPP1R12A,IWS1,ZPR1,GSK3B,CDKN1A	down_VSAP_EC
GO:0002724	0.0495	0.992	3	9	regulation of T cell cytokine production	BP	1	B2M,MAP3K7,IL18R1	up_ACOR_VSMC
GO:0051254	0.0495	0.996	2	431	positive regulation of RNA metabolic process	BP	1	GATA5,TGFBR1	down_VSAP_VSMC
GO:0001525	0.0495	0.999	1	173	angiogenesis	BP	1	JUP	down_VFEM_VSMC
GO:0072088	0.0495	0.999	1	36	nephron epithelium morphogenesis	BP	1	EYA1	up_AFEM_VSMC
GO:0001657	0.0495	0.999	1	42	ureteric bud development	BP	1	ARG2	up_AORT_VSMC
GO:0072163	0.0495	0.999	1	42	mesonephric epithelium development	BP	1	ARG2	up_AORT_VSMC
GO:0072164	0.0495	0.999	1	42	mesonephric tubule development	BP	1	ARG2	up_AORT_VSMC
GO:0060795	0.0495	0.999	1	12	cell fate commitment involved in formation of	BP	1	EYA1	up_VFEM_EC
GO:0043500	0.0495	0.999	1	21	muscle adaptation	BP	1	GATM	up_VFEM_VSMC
GO:0022410	0.049585252	0.99937	1	2	circadian sleep/wake cycle process	BP	1	PTGDS	up_ACOR_EC
GO:0042745	0.049585252	0.99937	1	2	circadian sleep/wake cycle	BP	1	PTGDS	up_ACOR_EC
GO:0042749	0.049585252	0.99937	1	2	regulation of circadian sleep/wake cycle	BP	1	PTGDS	up_ACOR_EC
GO:0045187	0.049585252	0.99937	1	2	regulation of circadian sleep/wake cycle, sleep	BP	1	PTGDS	up_ACOR_EC
GO:0050802	0.049585252	0.99937	1	2	circadian sleep/wake cycle, sleep	BP	1	PTGDS	up_ACOR_EC
GO:0009952	0.0496	0.982	7	66	anterior/posterior pattern specification	BP	1	BMPR1A,HOXC4,CLSTN1,SEMA3C,GLI3,PCSK6,ZBTB16	down_VCAV_EC
GO:0061077	0.0496	0.988	4	25	chaperone-mediated protein folding	BP	0.874	ERO1A,FKBP11,PPIB,FKBP10	down_VSAP_EC
GO:0035809	0.0496	0.997	2	4	regulation of urine volume	BP	1	EDN1,INPP5K	up_ACOR_VSMC
GO:0035810	0.0496	0.997	2	4	positive regulation of urine volume	BP	1	EDN1,INPP5K	up_ACOR_VSMC
GO:0060572	0.0496	0.999	1	11	morphogenesis of an epithelial bud	BP	1	SULF1	down_ACOR_EC
GO:0014070	0.0496	0.999	1	172	response to organic cyclic compound	BP	1	JUP	down_VFEM_VSMC
GO:0007219	0.0496	0.999	1	59	Notch signaling pathway	BP	1	GATA5	down_VSAP_VSMC
GO:0031348	0.0496	0.999	1	46	negative regulation of defense response	BP	1	ARG2	up_AORT_VSMC
GO:1901071	0.0496	0.999	1	6	glucosamine-containing compound metabolic	BP	1	OGA	up_VPOR_EC
GO:1903524	0.0496	0.999	1	11	positive regulation of blood circulation	BP	1	AVPR1A	up_VFEM_EC

GO:0045596	0.0497	0.97	25	186	negative regulation of cell differentiation	BP	1	THRB,HOXA7,TWIST2,HOXA9,AXIN2,SLIT2,HDAC7,DLL1,OSR1,ZBTB16,RARG,ZFP36L1,PIK3R1,MYC,IRX3,ZEB1,RTN4RL1,LRP4,ZADH2,FBXO7,LIMD1,FBLN1,ZFP36L2,ACVRL1,LBH	up_VSAP_EC
GO:0048232	0.0497	0.976	13	95	male gamete generation	BP	0.884	KIT,TSPAN8,PSME4,FIGNL1,NECTIN2,CDYL,CREM,ARID4B,HIST1H1A,AFF4,KDM3A,ATRX,SPATA6	up_VCAV_EC
GO:0070646	0.0497	0.978	10	63	protein modification by small protein removal	BP	0.884	TNFAIP3,TNIP1,USP33,ENSCAFG00000010893,OTUD4,SENP6,USP28,USP13,USP38,USP37	up_VCAV_EC
GO:2000482	0.0497	0.992	3	9	regulation of interleukin-8 secretion	BP	1	TLR1,F2RL1,PTPN22	up_ACOR_VSMC
GO:0048856	0.0497	1	2	1533	anatomical structure development	BP	1	JUP,GATA5	down_VFEM_VSMC
GO:0003149	0.0497	0.997	2	4	membranous septum morphogenesis	BP	1	BMP4,FGFR2	up_ACOR_VSMC
GO:0030098	0.0497	0.995	2	87	lymphocyte differentiation	BP	1	TOX,CYP26B1	up_VFEM_EC
GO:0002695	0.0497	0.999	1	44	negative regulation of leukocyte activation	BP	1	ARG2	up_AORT_VSMC
GO:0050707	0.0497	0.999	1	44	regulation of cytokine secretion	BP	1	ARG2	up_AORT_VSMC
GO:0002761	0.0497	0.999	1	36	regulation of myeloid leukocyte differentiation	BP	1	LRRC17	up_VCAV_VSMC
GO:0048747	0.0497	0.999	1	20	muscle fiber development	BP	1	SHOX2	up_VFEM_VSMC
GO:0042327	0.0498	0.971	21	289	positive regulation of phosphorylation	BP	1	NOTCH2,PDGFRB,PRNP,GLIPR2,BMPR1A,LMO4,CLIP3,AKT2,CSPG4,PARK7,MYDGF,SASH1,ARL2BP,MAVS,CDH2,GSDME,MAPRE3,DDR2,SHC1,FBH1,GREM1	down_VCAV_EC
GO:0046661	0.0498	0.995	2	45	male sex differentiation	BP	1	SEMA3A,ACVR2A	up_VPOR_EC
GO:0006835	0.0498	0.999	1	13	dicarboxylic acid transport	BP	1	NTRK2	up_APUL_EC
GO:0044381	0.0498	0.999	1	13	glucose import in response to insulin	BP	1	NR1H4	up_APUL_EC
GO:0051099	0.0498	0.999	1	70	positive regulation of binding	BP	1	HOXA3	up_APUL_VSMC
GO:2000027	0.0498	0.999	1	72	regulation of animal organ morphogenesis	BP	1	TBX5	up_APUL_VSMC
GO:1990266	0.0498	0.999	1	24	neutrophil migration	BP	1	C3AR1	up_VPOR_VSMC
GO:0002686	0.0498	0.999	1	12	negative regulation of leukocyte migration	BP	1	HOXA7	up_VFEM_EC
GO:0019229	0.0498	0.999	1	12	regulation of vasoconstriction	BP	1	AVPR1A	up_VFEM_EC
GO:1901863	0.0498	0.999	1	25	positive regulation of muscle tissue	BP	1	TBX5	up_VPUL_VSMC
GO:0061036	0.0498	0.999	1	11	positive regulation of cartilage development	BP	1	HOXA11	up_VSAP_VSMC
GO:0050994	0.049811322	0.99491	2	14	regulation of lipid catabolic process	BP	1	RARRES2,PRKCD	up_ACOR_EC
GO:0044281	0.0499	0.966	41	622	small molecule metabolic process	BP	1	GPAM,DCN,ADCY6,VKORC1,AKT2,LCAT,ACAA1,ME3,H6PD,CYB5R3,UROS,NT5C2,EPHX1,ENTPD3,THNSL2,PGLS,POFUT1,ATP5MC1,GFPT2,UQCR10,SUOX,CYP1B1,ADK,AHCYL1,PEX5,B4GALT1,RETSAT,PARK7,AMPD2,ENSCAFG00000002977,HPDL,FUT8,ACSS2,RDH10,CRTAP,LONP2,NAPEPLD,GLS2,SOAT1,BCKDHB,ACAT1	down_VCAV_EC
GO:0048588	0.0499	0.981	8	44	developmental cell growth	BP	1	SLIT2,ABL1,PLXNA4,RARG,DPYSL2,TTL,ULK1,TGFBR2	up_VSAP_EC
GO:0030030	0.0499	0.986	5	377	cell projection organization	BP	1	LYN,SPG11,PDPN,NRP2,NEDD4	down_ACOR_EC
GO:0051702	0.0499	0.988	4	24	interaction with symbiont	BP	0.874	SMARCA4,APOE,REST,CALM1	down_VSAP_EC
GO:0023057	0.0499	0.988	4	409	negative regulation of signaling	BP	1	NR1H4,PTGER2,FLRT3,ARHGEF2	up_VPUL_EC
GO:0009790	0.0499	0.991	3	335	embryo development	BP	1	HOXA3,NKX2-5,CYP26B1	up_VPOR_VSMC
GO:0034220	0.0499	0.991	3	188	ion transmembrane transport	BP	1	NETO1,STIM1,CAV1	up_VSAP_VSMC
GO:0034645	0.0499	1	2	1546	cellular macromolecule biosynthetic process	BP	1	JUP,GATA5	down_VFEM_VSMC
GO:1904683	0.0499	0.997	2	4	regulation of metalloendopeptidase	BP	1	MBP,RECK	up_ACOR_VSMC
GO:0051489	0.049959504	0.99489	2	14	regulation of filopodium assembly	BP	1	NRP1,PRKCD	up_ACOR_EC

GO:1905477	0.049971887	0.99085	3	32	positive regulation of protein localization to	BP	1	KCNB1,PLS1,LRP4	up_ACOR_EC
GO:0001822	0.05	0.975	14	98	kidney development	BP	0.884	HOXB7,PDGFB,PODXL,MEF2C,TIPARP,GPR4,GATA3,MTSS1,ODC1,ARG2,BMP2,PROX1,JAG1,BASP1	up_VCAV_EC
GO:0000082	0.05	0.982	7	57	G1/S transition of mitotic cell cycle	BP	0.874	PLK3,ZPR1,EIF4EBP1,BACH1,RBBP8,INHBA,CDKN1A	down_VSAP_EC
GO:0034440	0.05	0.991	3	35	lipid oxidation	BP	1	LEP,ACACB,PPARGC1A	up_AFEM_EC
GO:0060429	0.05	0.991	3	341	epithelium development	BP	1	EYA1,SPINK5,HOXA11	up_VFEM_VSMC
GO:0030949	0.05	0.996	2	6	positive regulation of vascular endothelial growth factor receptor	BP	0.874	VEGFA,HIF1A	down_VSAP_EC
GO:0006836	0.05	0.999	1	47	neurotransmitter transport	BP	1	WNT7A	up_AORT_VSMC
GO:0035773	0.05	0.999	1	15	insulin secretion involved in cellular response to glucose stimulus	BP	1	NR1H4	up_VPUL_EC
GO:0071359	0.05	0.999	1	11	cellular response to dsRNA	BP	1	CAV1	up_VSAP_VSMC
GO:0030970	0.050079277	0.99487	2	14	retrograde protein transport, ER to cytosol	BP	1	EDEM2,HM13	up_ACOR_EC
GO:1903513	0.050079277	0.99487	2	14	endoplasmic reticulum to cytosol transport	BP	1	EDEM2,HM13	up_ACOR_EC
GO:0051056	0.0501	0.975	13	87	regulation of small GTPase mediated signal transduction	BP	1	CDON,ARHGEF5,ARHGEF6,SLIT2,ABL1,F2RL1,MFN2,TIAM1,DLC1,FGD4,PSD4,ARHGAP12,FLOT1	up_VSAP_EC
GO:0050768	0.0501	0.98	8	66	negative regulation of neurogenesis	BP	0.874	DPYSL3,HOOK3,IFRD1,CTNNB1,APOE,REST,NRP1,YWHAH	down_VSAP_EC
GO:0046907	0.0501	0.984	6	536	intracellular transport	BP	1	LYN,SPG11,IPO7,STX4,MYO1D,NEDD4	down_ACOR_EC
GO:0055007	0.0501	0.984	6	31	cardiac muscle cell differentiation	BP	0.884	MEF2C,EDN1,MTOR,BMP2,PROX1,ENSCAFG00000010596	up_VCAV_EC
GO:0060502	0.0501	0.992	3	9	epithelial cell proliferation involved in lung	BP	1	FGF7,NFIB,MAP2K2	up_VSAP_EC
GO:0008610	0.0501	0.995	2	177	lipid biosynthetic process	BP	1	HSD3B7,GBGT1	down_VPOR_EC
GO:0055078	0.0501	0.997	2	4	sodium ion homeostasis	BP	1	EDN1,SLC8A1	up_ACOR_VSMC
GO:0007610	0.0501	0.995	2	146	behavior	BP	1	GATM,NETO1	up_VFEM_VSMC
GO:0048701	0.0501	0.999	1	25	embryonic cranial skeleton morphogenesis	BP	1	TBX15	down_VPOR_EC
GO:0032635	0.0501	0.999	1	37	interleukin-6 production	BP	1	TLR2	up_AFEM_VSMC
GO:0030101	0.0501	0.999	1	12	natural killer cell activation	BP	1	TOX	up_VFEM_EC
GO:0033043	0.050103074	0.97203	18	446	regulation of organelle organization	BP	1	TMOD1,PKIB,ARHGAP6,SYNPO2,DIXDC1,ACTN2,ARHGAP28,PAK3,PTGER4,ARHGDIB,PREB,MAVS,CHMP4B,VPS16,NRP1,PRKCD,PYGO2,TUBB4A	up_ACOR_EC
GO:0022406	0.0502	0.991	3	35	membrane docking	BP	1	CAMK2A,BVES,RAB7A	up_AFEM_EC
GO:0071248	0.0502	0.991	3	35	cellular response to metal ion	BP	1	MT2A,ENSCAFG00000009113,CPNE7	up_AFEM_EC
GO:0007351	0.0502	0.997	2	4	tripartite regional subdivision	BP	1	FRS2,PCSK6	up_ACOR_VSMC
GO:0008595	0.0502	0.997	2	4	anterior/posterior axis specification, embryo	BP	1	FRS2,PCSK6	up_ACOR_VSMC
GO:2000484	0.0502	0.997	2	4	positive regulation of interleukin-8 secretion	BP	1	TLR1,F2RL1	up_ACOR_VSMC
GO:0003149	0.0502	0.997	2	4	membranous septum morphogenesis	BP	1	FZD1,TGFBR2	up_VSAP_EC
GO:0048505	0.0502	0.997	2	4	regulation of timing of cell differentiation	BP	1	DLL1,GDPD5	up_VSAP_EC
GO:0002221	0.0502	0.999	1	46	pattern recognition receptor signaling	BP	1	ESR1	up_AORT_VSMC
GO:0002758	0.0502	0.999	1	46	innate immune response-activating signal	BP	1	ESR1	up_AORT_VSMC
GO:0007416	0.0502	0.999	1	36	synapse assembly	BP	1	SLITRK5	up_VCAV_VSMC
GO:0001960	0.0502	0.999	1	15	negative regulation of cytokine-mediated	BP	1	NR1H4	up_VPUL_EC
GO:0060761	0.0502	0.999	1	15	negative regulation of response to cytokine	BP	1	NR1H4	up_VPUL_EC
GO:0006862	0.050229759	0.99936	1	2	nucleotide transport	BP	1	SLC25A42	up_ACOR_EC
GO:1901679	0.050229759	0.99936	1	2	nucleotide transmembrane transport	BP	1	SLC25A42	up_ACOR_EC

GO:0019355	0.050291033	0.99935	1	2	nicotinamide nucleotide biosynthetic process from aspartate	BP	1	NMNAT2	up_ACOR_EC
GO:0034627	0.050291033	0.99935	1	2	'de novo' NAD biosynthetic process	BP	1	NMNAT2	up_ACOR_EC
GO:0034628	0.050291033	0.99935	1	2	'de novo' NAD biosynthetic process from	BP	1	NMNAT2	up_ACOR_EC
GO:0048731	0.0503	0.962	74	1247	system development	BP	1	ADAMTS2,NOTCH2,HSPB6,PDGFRB,SMAD9,LRP1,DCN,NAB2,NDN,ANGPT1,SDC4,VASN,FBN1,GLIPR2,KANK2,THBS3,BMPR1A,LOX,LMO4,RECK,TRPV2,ANPEP,NUMBL,NAGLU,CHRDL1,TBX15,AKT2,TFE3,ENSCAFG00000018277,HOXC4,CLSTN1,CSPG4,PKD1,SEMA3C,ASPN,THY1,TEAD1,HAPLN3,PEX5,TLX2,VCAN,B4GALT1,GLI3,EEF2,ZFP36L1,MYDGF,SASH1,NCDN,IFT57,CDH2,LOXL3,GSDME,CAMK2D,ENSCAFG00000017708,SPRED1,RAB7B,IRX5,TSC22D3,UST,GLIS2,PRKCSH,DDR2,SHC1,RDH10,OMD,GREM1,CLIC4,FOXO3,THRB,ZBTB16,ANKRD54,RGMA,SEC61A1,BRSK1	down_VCAV_EC
GO:0048869	0.0503	0.985	6	1119	cellular developmental process	BP	1	CHL1,BARX1,NKX2-5,BAMBI,TRPC4,CYP26B1	up_VPOR_VSMC
GO:0051193	0.0503	0.989	4	15	regulation of cofactor metabolic process	BP	1	PDK2,CLYBL,ZNF205,DDIT4	up_VSAP_EC
GO:0003008	0.0503	0.991	3	336	system process	BP	1	HOXC10,GATM,NETO1	up_VFEM_VSMC
GO:0033622	0.0503	0.996	2	6	integrin activation	BP	0.874	FBLIM1,TLN1	down_VSAP_EC
GO:0034135	0.0503	0.997	2	4	regulation of toll-like receptor 2 signaling	BP	1	F2RL1,CYBA	up_VSAP_EC
GO:0032801	0.0503	0.999	1	10	receptor catabolic process	BP	1	NEDD4	down_ACOR_EC
GO:0045576	0.0503	0.999	1	12	mast cell activation	BP	1	LYN	down_ACOR_EC
GO:0046040	0.0503	0.999	1	9	IMP metabolic process	BP	1	TNRC6B	down_ACOR_EC
GO:0000413	0.0503	1	1	1	protein peptidyl-prolyl isomerization	BP	0.874	PPIB	down_VSAP_EC
GO:0030004	0.0503	0.999	1	36	cellular monovalent inorganic cation	BP	1	CA2	up_VCAV_VSMC
GO:0046326	0.0503	0.999	1	15	positive regulation of glucose import	BP	1	NR1H4	up_VPUL_EC
GO:0051270	0.0504	0.966	37	296	regulation of cellular component movement	BP	1	HOXA7,TWIST2,WNT11,FAM107A,C5AR2,ENPP2,SLC8B1,SLIT2,CDH1,FGF7,ABL1,HDAC7,EMP2,KCNE3,FUZ,PDGFD,PLXNA4,CCBE1,STAT5A,RECK,F2RL1,DDR2,PIK3R1,TIAM1,TBXA2R,CFAP69,SASH1,MYD88,FBLN1,ZNF580,STX4,ACVRL1,NCK1,HMOX1,MAP2K2,TGFBR2,BAG4	up_VSAP_EC
GO:0001649	0.0504	0.983	6	54	osteoblast differentiation	BP	1	BMPR1A,CLSTN1,GLI3,TWIST2,DDR2,GREM1	down_VCAV_EC
GO:0035510	0.0504	0.992	3	10	DNA dealkylation	BP	0.884	GATA3,OTUD4,TET2	up_VCAV_EC
GO:1903053	0.0504	0.999	1	10	regulation of extracellular matrix organization	BP	1	PDPN	down_ACOR_EC
GO:0051224	0.0504	0.999	1	47	negative regulation of protein transport	BP	1	ARG2	up_AORT_VSMC
GO:1904950	0.0504	0.999	1	47	negative regulation of establishment of protein localization	BP	1	ARG2	up_AORT_VSMC
GO:1905209	0.0504	0.999	1	13	positive regulation of cardiocyte differentiation	BP	1	TBX5	up_APUL_EC
GO:0035637	0.0504	0.999	1	21	multicellular organismal signaling	BP	1	NKX2-5	up_VPOR_VSMC
GO:0008284	0.0505	0.967	31	238	positive regulation of cell proliferation	BP	1	CDON,C5AR2,OSR2,PTGFR,MAB21L1,IL6R,CRIP2,FGF7,ABL1,EMP2,DLL1,OSR1,PDGFD,HPSE,RARG,STAT5A,DDR2,MFN2,TIAM1,MYC,FBXW4,SHOX2,MYD88,PNP,ZNF580,STX4,ACVRL1,NCK1,HMOX1,TGFBR2,LBH	up_VSAP_EC
GO:0007286	0.0505	0.984	6	34	spermatid development	BP	0.884	KIT,PSME4,NECTIN2,CDYL,AFF4,KDM3A	up_VCAV_EC
GO:0019693	0.0505	0.995	2	172	ribose phosphate metabolic process	BP	1	SUCLG2,NDUFA10	down_AFEM_EC
GO:0006573	0.0505	0.997	2	4	valine metabolic process	BP	1	ALDH6A1,ENSCAFG00000002977	up_VSAP_EC
GO:0000245	0.0505	0.999	1	24	spliceosomal complex assembly	BP	1	SRSF6	down_AFEM_EC
GO:0045843	0.0505	0.999	1	13	negative regulation of striated muscle tissue	BP	1	TBX5	up_APUL_EC

GO:0060602	0.0505	0.999	1	12	branch elongation of an epithelium	BP	1	SIX1	up_VFEM_EC
GO:1904211	0.050524297	0.99935	1	2	membrane protein proteolysis involved in retrograde protein	BP	1	HM13	up_ACOR_EC
GO:0009306	0.05054773	0.98112	7	127	protein secretion	BP	1	NR1H4,KCNB1,FZD5,NOD2,PTGER4,PREB,MAVS	up_ACOR_EC
GO:0046877	0.050599406	0.99935	1	2	regulation of saliva secretion	BP	1	AQP1	up_ACOR_EC
GO:0046632	0.0506	0.984	6	29	alpha-beta T cell differentiation	BP	1	ABL1,ZBTB16,STAT6,CD83,PNP,TGFBR2	up_VSAP_EC
GO:0002573	0.0506	0.995	2	71	myeloid leukocyte differentiation	BP	1	LYN,HOXA7	down_ACOR_EC
GO:0045601	0.0506	0.999	1	10	regulation of endothelial cell differentiation	BP	1	ACVRL1	down_ACOR_EC
GO:0048646	0.0506	1	1	349	anatomical structure formation involved in	BP	1	GATA5	down_AFEM_VSMC
GO:0048871	0.0506	0.999	1	120	multicellular organismal homeostasis	BP	1	PLCL1	down_VFEM_EC
GO:0030878	0.0506	0.999	1	13	thyroid gland development	BP	1	HOXA3	up_APUL_EC
GO:0006355	0.0507	0.98	9	974	regulation of transcription, DNA-	BP	1	ACVRL1,YEATS2,YBX3,HIVEP2,HOXA7,SULF1,ELK3,HOXA1,NEDD4	down_ACOR_EC
GO:0030001	0.0507	0.991	3	172	metal ion transport	BP	1	LYN,HOXA3,NEDD4	down_ACOR_EC
GO:0046530	0.0507	0.991	3	17	photoreceptor cell differentiation	BP	1	NAGLU,THY1,THRB	down_VCAV_EC
GO:0001773	0.0507	0.996	2	4	myeloid dendritic cell activation	BP	1	BATF2,TGFBR2	up_VSAP_EC
GO:0043011	0.0507	0.996	2	4	myeloid dendritic cell differentiation	BP	1	BATF2,TGFBR2	up_VSAP_EC
GO:2001046	0.0507	0.996	2	4	positive regulation of integrin-mediated	BP	1	LIMS2,EMP2	up_VSAP_EC
GO:0006816	0.0507	0.994	2	84	calcium ion transport	BP	1	STIM1,CAV1	up_VSAP_VSMC
GO:0071326	0.0507	0.999	1	26	cellular response to monosaccharide stimulus	BP	1	RAB11B	down_VPOR_EC
GO:0071331	0.0507	0.999	1	26	cellular response to hexose stimulus	BP	1	RAB11B	down_VPOR_EC
GO:0022408	0.0507	0.999	1	44	negative regulation of cell-cell adhesion	BP	1	ARG2	up_AORT_VSMC
GO:0045926	0.0507	0.999	1	72	negative regulation of growth	BP	1	TBX5	up_APUL_VSMC
GO:1905523	0.0507	0.999	1	6	positive regulation of macrophage migration	BP	1	C3AR1	up_VPOR_EC
GO:0055017	0.0507	0.999	1	19	cardiac muscle tissue growth	BP	1	NKX2-5	up_VPOR_VSMC
GO:0010464	0.0507	0.999	1	21	regulation of mesenchymal cell	BP	1	SHOX2	up_VFEM_VSMC
GO:0045764	0.050716629	0.99934	1	2	positive regulation of cellular amino acid	BP	1	NR1H4	up_ACOR_EC
GO:0060456	0.050781662	0.99934	1	2	positive regulation of digestive system process	BP	1	AQP1	up_ACOR_EC
GO:0072488	0.050781662	0.99934	1	2	ammonium transmembrane transport	BP	1	AQP1	up_ACOR_EC
GO:0006898	0.0508	0.981	7	57	receptor-mediated endocytosis	BP	0.874	VEGFA,SERPINE1,CANX,TFRC,APOE,GPR107,LRRTM2	down_VSAP_EC
GO:0051924	0.0508	0.983	6	53	regulation of calcium ion transport	BP	1	PRNP,TRPV2,CACNB3,THY1,CYBA,PACSIN3	down_VCAV_EC
GO:0048519	0.0508	1	2	1554	negative regulation of biological process	BP	1	JUP,GATA5	down_VFEM_VSMC
GO:0009314	0.0508	0.995	2	143	response to radiation	BP	1	EYA1,NETO1	up_VFEM_VSMC
GO:0003433	0.0508	0.996	2	4	chondrocyte development involved in endochondral bone morphogenesis	BP	1	RARG,TGFBR2	up_VSAP_EC
GO:0072567	0.0508	0.996	2	4	chemokine (C-X-C motif) ligand 2 production	BP	1	F2RL1,MYD88	up_VSAP_EC
GO:1904646	0.0508	0.999	1	6	cellular response to amyloid-beta	BP	1	IGF1R	down_ACOR_EC
GO:0060541	0.0508	0.999	1	73	respiratory system development	BP	1	TBX5	up_APUL_VSMC
GO:0030334	0.050877139	0.97527	12	267	regulation of cell migration	BP	1	SOX9,AQP1,SEMA6D,ZMYND8,MMP28,PTGER4,ARHGDIB,FOXC2,LRRC15,TBX5,RARRES2,NRP1	up_ACOR_EC
GO:0045664	0.0509	0.971	20	144	regulation of neuron differentiation	BP	1	THRB,CDON,SLIT2,HOXD3,ABL1,DLL1,PLXNA4,IRX3,ZEB1,SHOX2,RTN4RL1,LRP4,DPYSL2,GDPD5,FBXO7,CYB5D2,PQBP1,TTL,NCK1,MAP2K2	up_VSAP_EC

GO:0009100	0.0509	0.975	12	139	glycoprotein metabolic process	BP	1	DCN,KRTCAP2,DDOST,GFPT2,NECAB3,B4GALT1,PARK7,FUT8,PRKCSH,PCSK6,RPN2,SOAT1	down_VCAV_EC
GO:0045861	0.0509	0.98	8	71	negative regulation of proteolysis	BP	0.874	SERPINE1,PTGS2,UCHL5,CRYAB,PAK2,A2M,SPINK2,USP14	down_VSAP_EC
GO:0034249	0.0509	0.985	5	41	negative regulation of cellular amide metabolic	BP	1	PRNP,SAMD4B,ZFP36L1,ORMDL3,EIF4EBP1	down_VCAV_EC
GO:1902750	0.0509	0.986	5	24	negative regulation of cell cycle G2/M phase	BP	0.884	TAOK1,BLM,RAD21,RAD17,TOPBP1	up_VCAV_EC
GO:0090317	0.0509	0.989	4	16	negative regulation of intracellular protein	BP	1	FERMT1,OS9,INPP5K,PARK7	up_ACOR_VSMC
GO:0007399	0.0509	0.988	4	564	nervous system development	BP	1	CHL1,HOXA3,NKX2-5,TRPC4	up_VPOR_VSMC
GO:0006351	0.0509	0.993	3	1069	transcription, DNA-templated	BP	1	GATA5,TGFBR1,MEIS2	down_VSAP_VSMC
GO:0060712	0.0509	0.996	2	4	spongiotrophoblast layer development	BP	1	GJB5,ZFP36L1	up_VSAP_EC
GO:0071941	0.0509	0.996	2	4	nitrogen cycle metabolic process	BP	1	SUOX,ASS1	up_VSAP_EC
GO:0050650	0.0509	0.999	1	11	chondroitin sulfate proteoglycan biosynthetic	BP	1	CSGALNACT1	down_ACOR_EC
GO:0021849	0.0509	1	1	1	neuroblast division in subventricular zone	BP	1	NUMBL	down_VCAV_EC
GO:0042445	0.0509	0.999	1	46	hormone metabolic process	BP	1	ESR1	up_AORT_VSMC
GO:0050891	0.0509	0.999	1	12	multicellular organismal water homeostasis	BP	1	CYP26B1	up_VFEM_EC
GO:0051347	0.051	0.97	22	182	positive regulation of transferase activity	BP	0.884	TEK,KIT,MAP3K11,PDGFB,STK10,RELN,TLR4,EDN1,RALB,NEDD9,MAP3K3,BMP2,SLK,PROX1,MADD,HMBOX1,PAK2,SH3GLB1,MMD,RAPGEF2,RIPK1,PDGFD	up_VCAV_EC
GO:0051648	0.051	0.98	8	69	vesicle localization	BP	0.874	MYH9,SHROOM2,MYO1C,KIF5B,CTNNB1,GSK3B,HTT,AP3S1	down_VSAP_EC
GO:0018022	0.051	0.982	7	36	peptidyl-lysine methylation	BP	0.884	IWS1,GATA3,ARID4B,NSD1,KDM3A,ATRX,TET2	up_VCAV_EC
GO:0001823	0.051	0.983	6	45	mesonephros development	BP	0.874	ENSCAFG00000032470,ILK,HOXB7,MAGED1,CTNNB1,SIX4	down_VSAP_EC
GO:0002702	0.051	0.984	6	28	positive regulation of production of molecular mediator of immune	BP	1	TP53BP1,F2RL1,PTPN22,B2M,MAP3K7,IL18R1	up_ACOR_VSMC
GO:0008104	0.051	0.992	3	763	protein localization	BP	1	WNT7A,ARG2,ESR1	up_AORT_VSMC
GO:0044089	0.051	0.995	2	170	positive regulation of cellular component	BP	1	GPSM2,FSCN1	down_AFEM_EC
GO:0009059	0.051	1	2	1563	macromolecule biosynthetic process	BP	1	JUP,GATA5	down_VFEM_VSMC
GO:0042692	0.051	0.994	2	105	muscle cell differentiation	BP	1	TBX5,MYBPC2	up_VPUL_EC
GO:0030855	0.051	0.995	2	183	epithelial cell differentiation	BP	1	GATA5,HOXB5	up_VPUL_VSMC
GO:0001765	0.051	0.996	2	4	membrane raft assembly	BP	1	EMP2,FLOT1	up_VSAP_EC
GO:0015693	0.051	0.996	2	4	magnesium ion transport	BP	1	NIPAL3,NIPAL1	up_VSAP_EC
GO:0034433	0.051	0.996	2	4	steroid esterification	BP	1	LCAT,SOAT1	up_VSAP_EC
GO:0034434	0.051	0.996	2	4	sterol esterification	BP	1	LCAT,SOAT1	up_VSAP_EC
GO:0034435	0.051	0.996	2	4	cholesterol esterification	BP	1	LCAT,SOAT1	up_VSAP_EC
GO:0002088	0.051	0.999	1	26	lens development in camera-type eye	BP	1	CITED2	down_VPOR_EC
GO:0032677	0.051	0.999	1	22	regulation of interleukin-8 production	BP	1	BPI	up_AORT_EC
GO:0032495	0.051	0.999	1	15	response to muramyl dipeptide	BP	1	ARHGEF2	up_VPUL_EC
GO:2000649	0.051	0.999	1	11	regulation of sodium ion transmembrane transporter activity	BP	1	NETO1	up_VSAP_VSMC
GO:2000275	0.051054914	0.99933	1	2	regulation of oxidative phosphorylation uncoupler activity	BP	1	PM20D1	up_ACOR_EC
GO:0030185	0.051087603	0.99933	1	2	nitric oxide transport	BP	1	AQP1	up_ACOR_EC
GO:0009607	0.0511	0.967	30	232	response to biotic stimulus	BP	1	VEGFD,TLR1,PLCG2,TMEM255A,COCH,MX1,ABCA1,TLR3,ISG15,CD274,PTGIR,SASH1,PLAC8B,ANKRD1,F2RL1,BNIP3,SLC17A5,PTPN22,GPM6A,PRKCE,PARP9,CHD7,CCL5,NOD2,DDIT4,NAALADL2,B2M,SERINC3,TNFRSF11B,WASL	up_ACOR_VSMC

GO:0140058	0.0511	0.996	2	4	neuron projection arborization	BP	1	ZNF365,NRP1	up_ACOR_VSMC
GO:0071294	0.0511	0.996	2	4	cellular response to zinc ion	BP	1	ENSCAFG00000009113,MT2A	up_VSAP_EC
GO:0072028	0.0511	0.999	1	37	nephron morphogenesis	BP	1	EYA1	up_AFEM_VSMC
GO:0050770	0.0511	0.999	1	39	regulation of axonogenesis	BP	1	WNT7A	up_AORT_VSMC
GO:0032768	0.0511	0.999	1	12	regulation of monooxygenase activity	BP	1	CAV1	up_VSAP_VSMC
GO:0072014	0.051197048	0.99933	1	2	proximal tubule development	BP	1	AQP1	up_ACOR_EC
GO:0033554	0.0512	0.963	54	708	cellular response to stress	BP	0.874	ARHGEF2,VEGFA,P4HB,ASNS,PLK3,ENSCAFG00000013214,HSPA5,SOCS5,HM13,SGK1,ERO1A,HSP90B1,CBX5,CASP3,ENSCAFG00000003477,HYOU1,PGK1,NUAK1,PTPN11,SESN2,PTGS2,SEL1L,HIF1A,DNAJC10,TNIK,UBA6,PTPN2,DDX39B,PDIA4,DNAJB9,CTNNB1,UBA5,LYN,BNIP3,DDIT3,PDS5B,FAM162A,BACH1,ATF3,GSK3B,CDC37,GTF2H1,RBBP8,MAPK10,EIF2S1,RAD23A,PAK2,CREB3L2,HIF1AN,CDKN1A,TMTC3,SH3RF3,USP14,MID1	down_VSAP_EC
GO:0046903	0.0512	0.968	28	255	secretion	BP	0.884	GBP5,KIT,EXOC3L2,MEF2C,FERMT1,CHD7,TLR4,STC1,GATA3,EDN1,RAB11A,RAB27A,EXOC6,SNCAIP,ARG2,SNAP23,SCAMP1,RSC1A1,ABCA1,EXOC5,CD200,FES,CREB1,STX11,HMGB1,NOS2,MCU,HIF1A	up_VCAV_EC
GO:0018345	0.0512	0.991	3	12	protein palmitoylation	BP	0.884	ZDHHC22,HHAT,ZDHHC6	up_VCAV_EC
GO:0031268	0.0512	0.992	3	9	pseudopodium organization	BP	1	CDC42EP5,CDC42EP2,F2RL1	up_VSAP_EC
GO:0031269	0.0512	0.992	3	9	pseudopodium assembly	BP	1	CDC42EP5,CDC42EP2,F2RL1	up_VSAP_EC
GO:0003211	0.0512	0.996	2	4	cardiac ventricle formation	BP	1	SMARCD3,TBX5	up_ACOR_VSMC
GO:0048568	0.0512	0.995	2	148	embryonic organ development	BP	1	HOXA3,NKX2-5	up_VPOR_VSMC
GO:1901652	0.0512	0.994	2	105	response to peptide	BP	1	NR1H4,ARHGEF2	up_VPUL_EC
GO:0001502	0.0512	0.999	1	8	cartilage condensation	BP	1	SULF1	down_ACOR_EC
GO:0098743	0.0512	0.999	1	8	cell aggregation	BP	1	SULF1	down_ACOR_EC
GO:0042493	0.0512	0.999	1	178	response to drug	BP	1	JUP	down_VFEM_VSMC
GO:0060759	0.0512	0.999	1	37	regulation of response to cytokine stimulus	BP	1	TLR2	up_AFEM_VSMC
GO:0006940	0.0512	0.999	1	14	regulation of smooth muscle contraction	BP	1	NPNT	up_APUL_EC
GO:0055021	0.0512	0.999	1	15	regulation of cardiac muscle tissue growth	BP	1	TBX5	up_VPUL_EC
GO:0050708	0.05121385	0.98256	6	101	regulation of protein secretion	BP	1	NR1H4,KCNB1,FZD5,NOD2,PTGER4,MAVS	up_ACOR_EC
GO:2001234	0.05122669	0.98468	5	75	negative regulation of apoptotic signaling	BP	1	IFI6,MAPK8IP2,FIGNL1,CLU,NRP1	up_ACOR_EC
GO:0001816	0.0513	0.968	26	194	cytokine production	BP	1	WNT11,CD274,C5AR2,IL6R,ABL1,HDAC7,DLL1,IL17RC,ANXA4,HPSE,CCBE1,F2RL1,PIK3R1,SULF2,MYD88,NLRX1,TKFC,CD83,RNF216,PNP,MAPKBP1,ZNF580,CYBA,PQBP1,FLOT1,MAVS	up_VSAP_EC
GO:1903506	0.0513	0.98	9	977	regulation of nucleic acid-templated transcription	BP	1	ACVRL1,YEATS2,YBX3,HIVEP2,HOXA7,SULF1,ELK3,HOXA1,NEDD4	down_ACOR_EC
GO:0040014	0.0513	0.984	6	27	regulation of multicellular organism growth	BP	1	FGFR2,ATRN,PLAC8B,APP,CHD7,STC2	up_ACOR_VSMC
GO:0044260	0.0513	1	3	2593	cellular macromolecule metabolic process	BP	1	PLCL1,MOSPD1,USP12	down_VFEM_EC
GO:0001937	0.0513	0.991	3	15	negative regulation of endothelial cell	BP	0.874	SULF1,SPARC,APOE	down_VSAP_EC
GO:0097659	0.0513	0.993	3	1072	nucleic acid-templated transcription	BP	1	GATA5,TGFBR1,MEIS2	down_VSAP_VSMC
GO:0009887	0.0513	0.995	2	314	animal organ morphogenesis	BP	1	WNT7A,ESR1	up_AORT_VSMC
GO:0048705	0.0513	0.994	2	95	skeletal system morphogenesis	BP	1	HOXA3,HOXA1	up_APUL_EC
GO:0048872	0.0514	0.985	5	85	homeostasis of number of cells	BP	1	GPAM,NFE2L1,CYLD,NAPEPLD,GCNT4	up_AFEM_EC
GO:0048666	0.0514	0.987	4	258	neuron development	BP	1	LYN,SPG11,NRP2,NEDD4	down_ACOR_EC
GO:0032774	0.0514	0.993	3	1073	RNA biosynthetic process	BP	1	GATA5,TGFBR1,MEIS2	down_VSAP_VSMC

GO:0006163	0.0514	0.995	2	174	purine nucleotide metabolic process	BP	1	SUCLG2,NDUFA10	down_AFEM_EC
GO:0002091	0.0514	0.996	2	4	negative regulation of receptor internalization	BP	1	ATXN2,LRPAP1	up_ACOR_VSMC
GO:0030878	0.0514	0.999	1	13	thyroid gland development	BP	1	HOXA3	down_ACOR_EC
GO:0032024	0.0514	0.999	1	12	positive regulation of insulin secretion	BP	1	STX4	down_ACOR_EC
GO:0090277	0.0514	0.999	1	12	positive regulation of peptide hormone	BP	1	STX4	down_ACOR_EC
GO:0001544	0.0514	1	1	1	initiation of primordial ovarian follicle growth	BP	1	FOXO3	down_VCAV_EC
GO:0007043	0.0514	0.999	1	28	cell-cell junction assembly	BP	1	HDAC7	down_VPOR_EC
GO:0021675	0.0514	0.999	1	29	nerve development	BP	1	CITED2	down_VPOR_EC
GO:0097581	0.0514	0.999	1	26	lamellipodium organization	BP	1	SLIT2	up_VPUL_VSMC
GO:0050794	0.0515	0.977	18	3075	regulation of cellular process	BP	1	EYA1,HOXA9,TOX,MAP2K6,HOXA10,TRIM9,HOXA7,ENSCAFG00000018194,SIX1,YBX3,MAB21L1,TIAM1,RADIL,RASD2,CYP26B1,CDC42EP5,MTSS1,ADCY3	up_VFEM_EC
GO:0035239	0.0515	0.975	12	299	tube morphogenesis	BP	1	IRX3,LEP,ANGPT4,HOXA7,DLL1,F3,TMEM100,GREM1,LUZP1,TBXA2R,PTPRB,CLIC4	up_AFEM_EC
GO:0040029	0.0515	0.976	11	74	regulation of gene expression, epigenetic	BP	1	ATAD2,SRRT,RAN,EZH2,UHRF1,BRCA1,SUV39H1,RBM14,HAT1,HIST2H2AC,H2AFX	down_ACOR_VSMC
GO:0031330	0.0515	0.985	5	85	negative regulation of cellular catabolic process	BP	1	LEP,TENT5C,CLEC16A,AXIN2,ACACB	up_AFEM_EC
GO:0007219	0.0515	0.987	4	59	Notch signaling pathway	BP	1	ANGPT4,DLL1,TMEM100,POSTN	up_AFEM_EC
GO:0007267	0.0515	0.987	4	332	cell-cell signaling	BP	1	NETO1,SALL1,TLR2,CAV1	up_VSAP_VSMC
GO:0009309	0.0515	0.996	2	4	amine biosynthetic process	BP	1	MOXD1,AZIN2	up_ACOR_VSMC
GO:0042401	0.0515	0.996	2	4	cellular biogenic amine biosynthetic process	BP	1	MOXD1,AZIN2	up_ACOR_VSMC
GO:0006955	0.0515	0.995	2	327	immune response	BP	1	ARG2,ESR1	up_AORT_VSMC
GO:0045602	0.0515	0.996	2	4	negative regulation of endothelial cell	BP	0.884	JAG1,ZEB1	up_VCAV_EC
GO:0000902	0.0515	0.995	2	266	cell morphogenesis	BP	1	SLITRK5,LIMK1	up_VCAV_VSMC
GO:0035295	0.0515	1	1	356	tube development	BP	1	GATA5	down_AFEM_VSMC
GO:0046474	0.0516	0.98	8	44	glycerophospholipid biosynthetic process	BP	1	PIP5K1B,PLCG2,HTR2B,LCAT,PIGN,PIGS,PIGG,CRLS1	up_ACOR_VSMC
GO:0001952	0.0516	0.983	6	34	regulation of cell-matrix adhesion	BP	0.884	TEK,FERMT1,SLK,APOD,JAG1,ENSCAFG00000002015	up_VCAV_EC
GO:0007338	0.0516	0.988	4	16	single fertilization	BP	1	GLRB,DKKL1,SPINK2,WDR48	up_ACOR_VSMC
GO:0060421	0.0516	0.999	1	14	positive regulation of heart growth	BP	1	TBX5	up_APUL_EC
GO:0071398	0.0516	0.999	1	14	cellular response to fatty acid	BP	1	NR1H4	up_APUL_EC
GO:0035567	0.0516	0.999	1	19	non-canonical Wnt signaling pathway	BP	1	ANKRD6	up_VPOR_VSMC
GO:0030961	0.051650571	0.99932	1	2	peptidyl-arginine hydroxylation	BP	1	RIOX2	up_ACOR_EC
GO:0051223	0.051658373	0.97783	9	181	regulation of protein transport	BP	1	SH3TC2,NR1H4,KCNB1,EDEM2,FZD5,NOD2,PTGER4,MAVS,PRKCD	up_ACOR_EC
GO:0051149	0.0517	0.985	5	25	positive regulation of muscle cell differentiation	BP	0.884	KIT,MEF2C,EDN1,MTOR,PROX1	up_VCAV_EC
GO:0002934	0.0517	0.996	2	4	desmosome organization	BP	1	JUP,PERP	up_ACOR_VSMC
GO:0008283	0.0517	0.996	2	545	cell proliferation	BP	1	HOXA3,TBX5	up_APUL_VSMC
GO:0060291	0.0517	0.999	1	11	long-term synaptic potentiation	BP	1	STX4	down_ACOR_EC
GO:0030155	0.0517	0.999	1	180	regulation of cell adhesion	BP	1	JUP	down_VFEM_VSMC
GO:0048209	0.051728485	0.99932	1	2	regulation of vesicle targeting, to, from or	BP	1	PREB	up_ACOR_EC
GO:0006972	0.05176157	0.99932	1	2	hyperosmotic response	BP	1	AQP1	up_ACOR_EC
GO:0071474	0.05176157	0.99932	1	2	cellular hyperosmotic response	BP	1	AQP1	up_ACOR_EC
GO:0010760	0.051770463	0.99932	1	2	negative regulation of macrophage chemotaxis	BP	1	MMP28	up_ACOR_EC
GO:1905522	0.051770463	0.99932	1	2	negative regulation of macrophage migration	BP	1	MMP28	up_ACOR_EC

GO:0042045	0.051793512	0.99932	1	2	epithelial fluid transport	BP	1	AQP1	up_ACOR_EC
GO:0001934	0.0518	0.966	35	274	positive regulation of protein phosphorylation	BP	1	ADRA1B,LEP,MAP3K8,MID1,HTR2B,RASSF2,BMP4,FGFR2,TLR3,SH3RF2,PDE5A,SASH1,FGF18,F2RL1,FAM129A,NRP1,PTPN22,NPNT,PRKCE,CCNT1,FRS2,PARP9,APP,PSEN1,CCL5,DDR2,DUSP22,EDN1,AKTIP,NOD2,SH3RF3,MAP3K7,ENSCAFG00000029553,PARK7,CRKL	up_ACOR_VSMC
GO:0051129	0.0518	0.968	26	221	negative regulation of cellular component organization	BP	1	HMGB1,ATAD2,ZW10,CAPG,TRIP13,SLC25A5,FBXO5,PRELID1,BIRC5,INSIG1,CAV1,LIMA1,STMN1,NEK2,BRCA1,NOC2L,NDC80,ENSCAFG00000005299,BUB1,RBM14,CORO1A,TTK,ARHGEF2,TINF2,MTBP,GEN1	down_ACOR_VSMC
GO:2001141	0.0518	0.979	9	979	regulation of RNA biosynthetic process	BP	1	ACVRL1,YEATS2,YBX3,HIVEP2,HOXA7,SULF1,ELK3,HOXA1,NEDD4	down_ACOR_EC
GO:0009058	0.0518	0.993	4	1910	biosynthetic process	BP	1	AK5,GATA5,TGFBR1,MEIS2	down_VSAP_VSMC
GO:1903727	0.0518	0.988	4	16	positive regulation of phospholipid metabolic	BP	0.884	TEK,ADGRF5,PDGFB,SH3GLB1	up_VCAV_EC
GO:0019221	0.0518	0.99	3	102	cytokine-mediated signaling pathway	BP	1	ENSCAFG00000002015,ASPN,ENSCAFG00000032731	up_VPOR_EC
GO:0071621	0.0518	0.999	1	27	granulocyte chemotaxis	BP	1	SLIT2	up_VPUL_VSMC
GO:0071383	0.0519	0.977	10	58	cellular response to steroid hormone stimulus	BP	1	LBH,SKP2,BMP4,MED14,AR,CREBRF,NR3C1,FAM107A,DDIT4,PARK7	up_ACOR_VSMC
GO:0007292	0.0519	0.983	6	32	female gamete generation	BP	1	TRIP13,FBXO5,NCAPH,FOSL1,TTK,ASPM	down_ACOR_VSMC
GO:0010556	0.0519	0.985	6	1137	regulation of macromolecule	BP	1	EYA1,HOXA10,HOXA9,EMX2,HOXA11,SHOX2	up_VFEM_VSMC
GO:0032528	0.0519	0.992	3	9	microvillus organization	BP	0.884	PODXL,RAPGEF2,FSCN1	up_VCAV_EC
GO:0042986	0.0519	1	1	1	positive regulation of amyloid precursor protein biosynthetic process	BP	1	SOAT1	down_VCAV_EC
GO:0043473	0.0519	0.999	1	28	pigmentation	BP	1	RAB11B	down_VPOR_EC
GO:0002065	0.0519	0.999	1	37	columnar/cuboidal epithelial cell	BP	1	GATA5	up_VCAV_VSMC
GO:2001259	0.0519	0.999	1	13	positive regulation of cation channel activity	BP	1	STIM1	up_VSAP_VSMC
GO:0051146	0.051944499	0.9844	5	76	striated muscle cell differentiation	BP	1	MYOZ2,TMOD1,ACTN2,TBX5,RBM38	up_ACOR_EC
GO:1904154	0.051959737	0.99931	1	2	positive regulation of retrograde protein	BP	1	EDEM2	up_ACOR_EC
GO:0033043	0.052	0.963	48	446	regulation of organelle organization	BP	0.884	PLK2,TEK,HCLS1,ARHGEF15,PDGFB,BAIAP2L1,IWS1,MEF2C,GATA3,EDN1,RALB,ARHGAP28,TMOD1,MTOR,CNOT6,NCKAP1,PROX1,UHRF2,IQGAP2,STMN1,HMBOX1,PRKD2,SH3GLB1,SWAP70,NCK1,FES,NSD1,SENP6,PAN3,TAOK1,TBC1D1,PMAIP1,ARPC5L,SMC5,RAD21,ARHGDIB,ZW10,KDM5A,HIF1A,KDM3A,ATRX,DCP2,TMOD3,RABGAP1,ARHGEF2,FSCN1,FBXO5,USP6NL	up_VCAV_EC
GO:0002520	0.052	0.987	4	279	immune system development	BP	1	LYN,HOXA3,PDPN,HOXA7	down_ACOR_EC
GO:0110111	0.052	0.991	3	17	negative regulation of animal organ	BP	1	ASPN,GREM1,THRB	down_VCAV_EC
GO:0006956	0.052	0.995	2	8	complement activation	BP	1	CFD,A2M	down_VCAV_EC
GO:0001944	0.052	0.995	2	263	vasculature development	BP	1	C6,TGFBI	up_VCAV_VSMC
GO:0042128	0.052	1	1	1	nitrate assimilation	BP	1	SUOX	down_VCAV_EC
GO:0050679	0.052	0.999	1	62	positive regulation of epithelial cell proliferation	BP	1	TGFBR1	down_VSAP_VSMC
GO:0051146	0.052	0.999	1	76	striated muscle cell differentiation	BP	1	TBX5	up_APUL_VSMC
GO:0072079	0.052	0.999	1	12	nephron tubule formation	BP	1	SIX1	up_VFEM_EC
GO:0046622	0.052	0.999	1	15	positive regulation of organ growth	BP	1	TBX5	up_VPUL_EC
GO:0048675	0.052	0.999	1	26	axon extension	BP	1	SLIT2	up_VPUL_VSMC
GO:0051182	0.052004021	0.99931	1	2	coenzyme transport	BP	1	SLC25A42	up_ACOR_EC
GO:0072009	0.0521	0.98	8	48	nephron epithelium development	BP	0.884	HOXB7,PODXL,MEF2C,GATA3,MTSS1,BMP2,JAG1,BASP1	up_VCAV_EC
GO:0002753	0.0521	0.988	4	17	cytoplasmic pattern recognition receptor	BP	0.884	TNFAIP3,TLR4,RIOK3,LSM14A	up_VCAV_EC

GO:0021515	0.0521	0.992	3	9	cell differentiation in spinal cord	BP	1	LMO4,TBX20,CLN8	up_ACOR_VSMC
GO:0002673	0.0521	0.995	2	6	regulation of acute inflammatory response	BP	0.874	PTGS2,A2M	down_VSAP_EC
GO:0010718	0.0521	0.999	1	21	positive regulation of epithelial to mesenchymal	BP	1	BAMBI	up_VPOR_VSMC
GO:0035115	0.0521	0.999	1	12	embryonic forelimb morphogenesis	BP	1	HOXA9	up_VFEM_EC
GO:0021954	0.0521	0.999	1	26	central nervous system neuron development	BP	1	SLIT2	up_VPUL_VSMC
GO:1902750	0.0522	0.985	5	24	negative regulation of cell cycle G2/M phase	BP	1	CDK1,FANCI,TOPBP1,DTL,CLSPN	down_ACOR_VSMC
GO:0051240	0.0522	0.985	5	489	positive regulation of multicellular organismal	BP	1	TOX,AVPR1A,HOXB7,SIX1,CYP26B1	up_VFEM_EC
GO:0002534	0.0522	0.988	4	18	cytokine production involved in inflammatory	BP	0.884	GBP5,TLR4,APOD,NOS2	up_VCAV_EC
GO:0033631	0.0522	0.996	2	4	cell-cell adhesion mediated by integrin	BP	0.884	PODXL,SWAP70	up_VCAV_EC
GO:0061178	0.0522	0.999	1	12	regulation of insulin secretion involved in cellular response to	BP	1	STX4	down_ACOR_EC
GO:0051223	0.0522	0.999	1	181	regulation of protein transport	BP	1	JUP	down_VFEM_VSMC
GO:0048592	0.052242551	0.98693	4	53	eye morphogenesis	BP	1	SOX9,AQP1,FZD5,EFEMP1	up_ACOR_EC
GO:1902742	0.052270454	0.99451	2	14	apoptotic process involved in development	BP	1	FZD5,FOXC2	up_ACOR_EC
GO:0001817	0.052277134	0.97751	9	182	regulation of cytokine production	BP	1	CDH3,NR1H4,NDRG2,FZD5,NOD2,IL33,PTGER4,MAVS,AGPAT2	up_ACOR_EC
GO:0016567	0.0523	0.966	30	232	protein ubiquitination	BP	1	PCGF5,SKP2,SH3RF2,ISG15,SASH1,KLHL20,MARCH5,PTPN22,OS9,UBE2H,SH3RF1,RNF24,PRKCE,RNF19A,PSEN1,SHPRH,RNF141,NOD2,FAM107A,SH3RF3,DCUN1D4,RNF181,HERPUD1,CNOT4,KLHDC8B,WFS1,KLHL36,WDR48,DZIP3,PARK7	up_ACOR_VSMC
GO:0090596	0.0523	0.974	13	84	sensory organ morphogenesis	BP	1	THRB,CDON,OSR2,INSIG2,LRIG3,IRX5,DLL1,OSR1,RARG,MFN2,ZEB1,KDM2B,CITED2	up_VSAP_EC
GO:0001932	0.0523	0.988	4	416	regulation of protein phosphorylation	BP	1	CCL24,PTGER2,FLRT3,ARHGEF2	up_VPUL_EC
GO:0006099	0.0523	0.988	4	15	tricarboxylic acid cycle	BP	1	DLST,IDH1,IDH3A,ENSCAFG00000006639	up_VSAP_EC
GO:0009190	0.0523	0.992	3	8	cyclic nucleotide biosynthetic process	BP	0.884	NPR1,GUCY1A1,GUCY1B1	up_VCAV_EC
GO:0052652	0.0523	0.992	3	8	cyclic purine nucleotide metabolic process	BP	0.884	NPR1,GUCY1A1,GUCY1B1	up_VCAV_EC
GO:0019367	0.0523	0.996	2	5	fatty acid elongation, saturated fatty acid	BP	1	ELOVL1,ELOVL6	down_ACOR_VSMC
GO:0019368	0.0523	0.996	2	5	fatty acid elongation, unsaturated fatty acid	BP	1	ELOVL1,ELOVL6	down_ACOR_VSMC
GO:0034625	0.0523	0.996	2	5	fatty acid elongation, monounsaturated fatty	BP	1	ELOVL1,ELOVL6	down_ACOR_VSMC
GO:0034626	0.0523	0.996	2	5	fatty acid elongation, polyunsaturated fatty acid	BP	1	ELOVL1,ELOVL6	down_ACOR_VSMC
GO:0035338	0.0523	0.996	2	5	long-chain fatty-acyl-CoA biosynthetic process	BP	1	ELOVL1,ELOVL6	down_ACOR_VSMC
GO:0030855	0.0523	0.999	1	183	epithelial cell differentiation	BP	1	GATA5	down_VFEM_VSMC
GO:0001823	0.0523	0.999	1	45	mesonephros development	BP	1	ARG2	up_AORT_VSMC
GO:0060231	0.0523	0.999	1	12	mesenchymal to epithelial transition	BP	1	SALL1	up_VSAP_VSMC
GO:1903035	0.052311495	0.99451	2	14	negative regulation of response to wounding	BP	1	RGMA,PRKCD	up_ACOR_EC
GO:0046459	0.052379949	0.9993	1	2	short-chain fatty acid metabolic process	BP	1	THNSL2	up_ACOR_EC
GO:0051790	0.052379949	0.9993	1	2	short-chain fatty acid biosynthetic process	BP	1	THNSL2	up_ACOR_EC
GO:0048856	0.0524	0.984	7	1533	anatomical structure development	BP	1	HOXC10,DKK2,HOXA9,HOXC6,SGCD,TAOK1,DUSP2	up_AORT_EC
GO:0045665	0.0524	0.982	6	55	negative regulation of neuron differentiation	BP	1	THY1,TLX2,GLI3,PRKCSH,THRB,RGMA	down_VCAV_EC
GO:0030168	0.0524	0.988	4	23	platelet activation	BP	0.874	APOE,LYN,SLC7A11,ITGB3	down_VSAP_EC
GO:0006139	0.0524	0.993	4	1909	nucleobase-containing compound metabolic	BP	1	AK5,GATA5,TGFBR1,MEIS2	down_VSAP_VSMC
GO:0032506	0.0524	0.995	2	8	cytokinetic process	BP	1	SNX18,ANXA11	down_VCAV_EC
GO:0048749	0.0524	1	1	1	compound eye development	BP	1	CHRDL1	down_VCAV_EC

GO:0007186	0.0524	0.999	1	124	G protein-coupled receptor signaling	BP	1	PLCL1	down_VFEM_EC
GO:0048247	0.0524	0.999	1	16	lymphocyte chemotaxis	BP	1	CCL24	up_VPUL_EC
GO:0060443	0.0524	0.999	1	13	mammary gland morphogenesis	BP	1	CAV1	up_VSAP_VSMC
GO:0006928	0.0525	0.961	66	557	movement of cell or subcellular component	BP	1	TNNT2,FGF13,RHOT1,VEGFD,LGR6,DNAH7,KCNE3,HTR2B,TCAF1,ZRANB1,RECK,FEZ1,FERMT1,BMP4,GJA1,HRH1,SH3RF2,TP53INP1,SASH1,LMO4,TMEM108,FGF18,IFT43,KIF1A,F2RL1,NRP1,PTPN22,GPM6A,UNC5B,EVL,SPINT2,TBX20,TBX5,PRKCE,FZD3,MYO1B,DAG1,FUT8,POMK,APP,LAMA3,PSEN1,SEMA6D,SYNJ2BP,JUP,CCL5,DDR2,DUSP22,EDN1,DYNC1I2,ARID5B,FAM107A,JAM3,DISC1,KIF9,FEZ2,SUN1,WASL,DOCK5,ENSCAFG00000029553,DYNC1I1,ENSCAFG00000013536,BVES,RREB1,CRKL,PIK3C2A	up_ACOR_VSMC
GO:0001958	0.0525	0.999	1	11	endochondral ossification	BP	1	CSGALNACT1	down_ACOR_EC
GO:0036075	0.0525	0.999	1	11	replacement ossification	BP	1	CSGALNACT1	down_ACOR_EC
GO:0071612	0.0525	1	1	1	IP-10 production	BP	1	MAVS	down_VCAV_EC
GO:0071651	0.0525	1	1	1	positive regulation of chemokine (C-C motif)	BP	1	MAVS	down_VCAV_EC
GO:0071658	0.0525	1	1	1	regulation of IP-10 production	BP	1	MAVS	down_VCAV_EC
GO:0071660	0.0525	1	1	1	positive regulation of IP-10 production	BP	1	MAVS	down_VCAV_EC
GO:0002905	0.0525	1	1	1	regulation of mature B cell apoptotic process	BP	0.884	BCL10	up_VCAV_EC
GO:0002906	0.0525	1	1	1	negative regulation of mature B cell apoptotic	BP	0.884	BCL10	up_VCAV_EC
GO:0045414	0.0525	1	1	1	regulation of interleukin-8 biosynthetic process	BP	0.884	BCL10	up_VCAV_EC
GO:0045416	0.0525	1	1	1	positive regulation of interleukin-8 biosynthetic	BP	0.884	BCL10	up_VCAV_EC
GO:0002901	0.0525	1	1	1	mature B cell apoptotic process	BP	0.884	BCL10	up_VCAV_EC
GO:0048565	0.0525	0.999	1	38	digestive tract development	BP	1	GATA5	up_VCAV_VSMC
GO:0061383	0.0525	0.999	1	20	trabecula morphogenesis	BP	1	NKX2-5	up_VPOR_VSMC
GO:0034969	0.052551194	0.99929	1	2	histone arginine methylation	BP	1	NR1H4	up_ACOR_EC
GO:0006528	0.052599696	0.99929	1	2	asparagine metabolic process	BP	1	NIT2	up_ACOR_EC
GO:0045785	0.0526	0.972	16	107	positive regulation of cell adhesion	BP	1	LEP,CD83,FERMT1,CITED2,CD274,NRP1,PTPN22,NPNT,PRKCE,ECM2,JUP,NID1,DUSP26,DOCK5,RREB1,CRKL	up_ACOR_VSMC
GO:0060840	0.0526	0.98	8	42	artery development	BP	1	SMAD6,BMP4,SEC24B,CITED2,NRP1,CHD7,HPGD,AP2B1	up_ACOR_VSMC
GO:0006754	0.0526	0.985	5	36	ATP biosynthetic process	BP	0.874	PGK1,TPI1,PGAM1,HIF1A,ALDOC	down_VSAP_EC
GO:0044380	0.0526	0.991	3	14	protein localization to cytoskeleton	BP	0.874	HOOK3,GSK3B,MID1	down_VSAP_EC
GO:0045637	0.0526	0.994	2	73	regulation of myeloid cell differentiation	BP	1	LYN,HOXA7	down_ACOR_EC
GO:0021510	0.0526	0.999	1	23	spinal cord development	BP	1	HOXC10	up_AORT_EC
GO:0003209	0.0526	0.999	1	14	cardiac atrium morphogenesis	BP	1	TBX5	up_APUL_EC
GO:0003230	0.0526	0.999	1	14	cardiac atrium development	BP	1	TBX5	up_APUL_EC
GO:0071624	0.0526	0.999	1	6	positive regulation of granulocyte chemotaxis	BP	1	C3AR1	up_VPOR_EC
GO:0090023	0.0526	0.999	1	6	positive regulation of neutrophil chemotaxis	BP	1	C3AR1	up_VPOR_EC
GO:0006040	0.0526	0.999	1	12	amino sugar metabolic process	BP	1	CHST7	up_VFEM_EC
GO:0055022	0.052689359	0.99929	1	2	negative regulation of cardiac muscle tissue	BP	1	TBX5	up_ACOR_EC
GO:0060044	0.052689359	0.99929	1	2	negative regulation of cardiac muscle cell	BP	1	TBX5	up_ACOR_EC
GO:0061117	0.052689359	0.99929	1	2	negative regulation of heart growth	BP	1	TBX5	up_ACOR_EC
GO:0022407	0.0527	0.974	13	96	regulation of cell-cell adhesion	BP	0.884	IL7R,PODXL,GATA3,PELI1,ARG2,BMP2,ITPKB,SWAP70,NCK1,JAG1,BCL10,HMGB1,ITGA6	up_VCAV_EC

GO:0051926	0.0527	0.999	1	14	negative regulation of calcium ion transport	BP	1	HOXA3	down_ACOR_EC
GO:0018209	0.0527	0.999	1	125	peptidyl-serine modification	BP	1	PLCL1	down_VFEM_EC
GO:0060612	0.0527	0.999	1	14	adipose tissue development	BP	1	NR1H4	up_APUL_EC
GO:0072077	0.0527	0.999	1	13	renal vesicle morphogenesis	BP	1	SALL1	up_VSAP_VSMC
GO:0072273	0.0527	0.999	1	13	metanephric nephron morphogenesis	BP	1	SALL1	up_VSAP_VSMC
GO:0032757	0.052754214	0.99444	2	14	positive regulation of interleukin-8 production	BP	1	NOD2,MAVS	up_ACOR_EC
GO:0001941	0.052787819	0.99443	2	14	postsynaptic membrane organization	BP	1	LRP4,LRRC4	up_ACOR_EC
GO:0009968	0.0528	0.964	42	392	negative regulation of signal transduction	BP	0.884	CXCL8,TNFAIP3,TNIP1,PDE4D,FERMT1,ATF3,DLL4,RAPGEF1,TLR4,NLRC5,GATA3,APLNR,ARAP3,HOMER3,EGFL7,FIGNL1,CASP8,MTOR,BMP2,HHEX,PREX2,GDPD5,RIOK3,STMN1,MCC,SMARCA4,APOD,NCK1,OTUD4,PTGS2,CTDSPL2,CTNNA1,CNOT7,RIPK1,ITGA6,RGS2,SKIL,HIF1A,TNFAIP1,IVNS1ABP,ARHGEF2,PEAR1	up_VCAV_EC
GO:2000179	0.0528	0.988	4	14	positive regulation of neural precursor cell	BP	0.884	DLL4,PROX1,HIF1A,CDON	up_VCAV_EC
GO:0086004	0.0528	0.991	3	10	regulation of cardiac muscle cell contraction	BP	1	FGF13,KCNE3,JUP	up_ACOR_VSMC
GO:0060592	0.0528	0.996	2	4	mammary gland formation	BP	1	BMP4,FGFR2	up_ACOR_VSMC
GO:0006549	0.0528	1	1	1	isoleucine metabolic process	BP	1	ACAT1	down_VCAV_EC
GO:0006550	0.0528	1	1	1	isoleucine catabolic process	BP	1	ACAT1	down_VCAV_EC
GO:0016480	0.0528	1	1	1	negative regulation of transcription by RNA polymerase III	BP	1	NAB2	down_VCAV_EC
GO:0046952	0.0528	1	1	1	ketone body catabolic process	BP	1	ACAT1	down_VCAV_EC
GO:1901373	0.0528	1	1	1	lipid hydroperoxide transport	BP	1	SCP2	down_VCAV_EC
GO:1902858	0.0528	1	1	1	propionyl-CoA metabolic process	BP	1	ACAT1	down_VCAV_EC
GO:1902860	0.0528	1	1	1	propionyl-CoA biosynthetic process	BP	1	ACAT1	down_VCAV_EC
GO:0006957	0.0528	1	1	1	complement activation, alternative pathway	BP	1	CFD	down_VCAV_EC
GO:0097194	0.0528	0.999	1	23	execution phase of apoptosis	BP	1	TAOK1	up_AORT_EC
GO:0043433	0.0528	0.999	1	49	negative regulation of DNA-binding transcription factor activity	BP	1	ESR1	up_AORT_VSMC
GO:0001947	0.0528	0.999	1	21	heart looping	BP	1	NKX2-5	up_VPOR_VSMC
GO:0061371	0.0528	0.999	1	21	determination of heart left/right asymmetry	BP	1	NKX2-5	up_VPOR_VSMC
GO:0061043	0.052877902	0.99929	1	2	regulation of vascular wound healing	BP	1	FOXC2	up_ACOR_EC
GO:0080134	0.052893481	0.97134	16	386	regulation of response to stress	BP	1	NR1H4,FNDC4,EDEM2,FZD5,NOD2,IL33,RGMA,TNFRSF19,PTGER4,MAPK8IP2,MAVS,FOXC2,FIGNL1,SH3RF3,CLU,PRKCD	up_ACOR_EC
GO:0001701	0.0529	0.971	17	134	in utero embryonic development	BP	0.884	PCDH12,TIE1,AMOT,PDGFB,CHD7,GATA3,EDN1,SOX18,BMP2,HHEX,MDFI,SKIL,CUL4A,HIF1A,RBBP6,VASH1,GINS4	up_VCAV_EC
GO:0071478	0.0529	0.978	9	58	cellular response to radiation	BP	1	NEDD4,INTS7,RAD51,AURKB,MAP3K20,PCNA,NOC2L,POLH,CDC25A	down_ACOR_VSMC
GO:0031345	0.0529	0.994	2	46	negative regulation of cell projection organization	BP	1	SEMA3A,RNF6	up_VPOR_EC
GO:0060425	0.0529	0.999	1	25	lung morphogenesis	BP	1	SRSF6	down_AFEM_EC
GO:0005988	0.0529	1	1	1	lactose metabolic process	BP	1	B4GALT1	down_VCAV_EC
GO:0005989	0.0529	1	1	1	lactose biosynthetic process	BP	1	B4GALT1	down_VCAV_EC
GO:0060057	0.0529	1	1	1	apoptotic process involved in mammary	BP	1	B4GALT1	down_VCAV_EC
GO:0060058	0.0529	1	1	1	positive regulation of apoptotic process involved in mammary	BP	1	B4GALT1	down_VCAV_EC
GO:1903519	0.0529	1	1	1	regulation of mammary gland involution	BP	1	B4GALT1	down_VCAV_EC

GO:1903521	0.0529	1	1	1	positive regulation of mammary gland	BP	1	B4GALT1	down_VCAV_EC
GO:0007619	0.0529	1	1	1	courtship behavior	BP	1	THRB	down_VCAV_EC
GO:0007621	0.0529	1	1	1	negative regulation of female receptivity	BP	1	THRB	down_VCAV_EC
GO:0008050	0.0529	1	1	1	female courtship behavior	BP	1	THRB	down_VCAV_EC
GO:0042480	0.0529	1	1	1	negative regulation of eye photoreceptor cell	BP	1	THRB	down_VCAV_EC
GO:0045924	0.0529	1	1	1	regulation of female receptivity	BP	1	THRB	down_VCAV_EC
GO:0060180	0.0529	1	1	1	female mating behavior	BP	1	THRB	down_VCAV_EC
GO:0097474	0.0529	1	1	1	retinal cone cell apoptotic process	BP	1	THRB	down_VCAV_EC
GO:0050770	0.0529	0.999	1	39	regulation of axonogenesis	BP	1	LIMK1	up_VCAV_VSMC
GO:0008306	0.0529	0.999	1	21	associative learning	BP	1	NETO1	up_VFEM_VSMC
GO:0060548	0.053	0.967	25	294	negative regulation of cell death	BP	0.874	ARHGEF2,VEGFA,ASNS,NR1H4,PLK3,HSPA5,SERPINE1,HYOU1,AARS,FHL2,PTGS2,HIF1A,ZPR1,MTDH,CTNNB1,CRYAB,APOE,BNIP3,GSK3B,HTT,GLO1,PAK2,SIX4,NRP1,ARF4	down_VSAP_EC
GO:0070085	0.053	0.973	14	95	glycosylation	BP	1	GALNT3,GALNT10,ST3GAL5,B3GALNT2,B3GNT7,CCDC126,MAGT1,STT3B,FUT11,FUT8,POMK,PSEN1,EOGT,LMF1	up_ACOR_VSMC
GO:0022612	0.053	0.979	8	44	gland morphogenesis	BP	1	LIMS2,FGF7,RARG,STAT6,SULF2,NTN4,NFIB,TGFBR2	up_VSAP_EC
GO:0030004	0.053	0.99	3	36	cellular monovalent inorganic cation	BP	1	RAB20,RAB7A,ATP6V0B	up_AFEM_EC
GO:0043691	0.053	0.996	2	4	reverse cholesterol transport	BP	1	LCAT,ABCA1	up_ACOR_VSMC
GO:0048532	0.053	0.999	1	9	anatomical structure arrangement	BP	1	NRP2	down_ACOR_EC
GO:0030644	0.053	1	1	1	cellular chloride ion homeostasis	BP	1	ENSCAFG00000018277	down_VCAV_EC
GO:0055064	0.053	1	1	1	chloride ion homeostasis	BP	1	ENSCAFG00000018277	down_VCAV_EC
GO:0060040	0.053	1	1	1	retinal bipolar neuron differentiation	BP	1	IRX5	down_VCAV_EC
GO:0001824	0.053	0.999	1	27	blastocyst development	BP	1	CITED2	down_VPOR_EC
GO:0008584	0.053	0.999	1	39	male gonad development	BP	1	HOXA9	up_AFEM_VSMC
GO:0046546	0.053	0.999	1	39	development of primary male sexual	BP	1	HOXA9	up_AFEM_VSMC
GO:0032944	0.053	0.999	1	48	regulation of mononuclear cell	BP	1	ARG2	up_AORT_VSMC
GO:0050670	0.053	0.999	1	48	regulation of lymphocyte proliferation	BP	1	ARG2	up_AORT_VSMC
GO:0001934	0.0531	0.969	20	274	positive regulation of protein phosphorylation	BP	1	NOTCH2,PDGFRB,PRNP,GLIPR2,BMPR1A,CLIP3,AKT2,CSPG4,PARK7,MYDGF,SASH1,ARL2BP,MAVS,CDH2,GSDME,MAPRE3,DDR2,SHC1,FBH1,GREM1	down_VCAV_EC
GO:0098660	0.0531	0.974	12	141	inorganic ion transmembrane transport	BP	1	PRNP,FXYD1,SLC39A6,CACNB3,ATP5MC1,THY1,ATP6V0C,PARK7,SCARA5,CYBA,ARHGAP1,CLIC4	down_VCAV_EC
GO:0010975	0.0531	0.975	11	103	regulation of neuron projection development	BP	0.874	SIPA1L1,DPYSL3,IFRD1,TNIK,UST,APOE,LYN,VLDLR,DISC1,NRP1,YWHAH	down_VSAP_EC
GO:0030336	0.0531	0.984	5	86	negative regulation of cell migration	BP	1	ANGPT4,HOXA7,RECK,TBXA2R,KANK1	up_AFEM_EC
GO:0044728	0.0531	0.988	4	17	DNA methylation or demethylation	BP	0.884	GATA3,OTUD4,MTA2,TET2	up_VCAV_EC
GO:0071495	0.0531	1	1	365	cellular response to endogenous stimulus	BP	1	GATA5	down_AFEM_VSMC
GO:0016056	0.0531	1	1	1	rhodopsin mediated signaling pathway	BP	0.874	RDH11	down_VSAP_EC
GO:0036367	0.0531	1	1	1	light adaption	BP	0.874	RDH11	down_VSAP_EC
GO:0016062	0.0531	1	1	1	adaptation of rhodopsin mediated signaling	BP	0.874	RDH11	down_VSAP_EC
GO:0022612	0.0531	0.999	1	44	gland morphogenesis	BP	1	ESR1	up_AORT_VSMC
GO:0060026	0.0531	0.999	1	7	convergent extension	BP	1	PTK7	up_VPOR_EC
GO:1990868	0.0531	0.999	1	16	response to chemokine	BP	1	CCL24	up_VPUL_EC
GO:1990869	0.0531	0.999	1	16	cellular response to chemokine	BP	1	CCL24	up_VPUL_EC

GO:1903524	0.0531	0.999	1	11	positive regulation of blood circulation	BP	1	CAV1	up_VSAP_VSMC
GO:0048681	0.053126613	0.99928	1	2	negative regulation of axon regeneration	BP	1	RGMA	up_ACOR_EC
GO:0090154	0.053127462	0.99928	1	2	positive regulation of sphingolipid biosynthetic	BP	1	PRKCD	up_ACOR_EC
GO:2000304	0.053127462	0.99928	1	2	positive regulation of ceramide biosynthetic	BP	1	PRKCD	up_ACOR_EC
GO:2000145	0.0532	0.965	35	279	regulation of cell motility	BP	1	HOXA7,TWIST2,WNT11,FAM107A,C5AR2,ENPP2,SLC8B1,SLIT2,CDH1,FGF7,ABL1,HDAC7,EMP2,FUZ,PDGFD,CCBE1,STAT5A,RECK,F2RL1,DDR2,PIK3R1,TIAM1,TBXA2R,CFAP69,SASH1,MYD88,FBLN1,ZNF580,STX4,ACVRL1,NCK1,HMOX1,MAP2K2,TGFBR2,BAG4	up_VSAP_EC
GO:0042254	0.0532	0.971	16	128	ribosome biogenesis	BP	1	NOP56,RAN,FBL,DIS3,NOC4L,NOC2L,DDX28,RSL1D1,RRP1,ENSCAFG00000019620,NOP14,NOP58,ENSCAFG00000015189,EMG1,EXOSC5,NOB1	down_ACOR_VSMC
GO:0035107	0.0532	0.987	4	60	appendage morphogenesis	BP	1	HOXA9,HOXC10,GREM1,RECK	up_AFEM_EC
GO:0035108	0.0532	0.987	4	60	limb morphogenesis	BP	1	HOXA9,HOXC10,GREM1,RECK	up_AFEM_EC
GO:0042304	0.0532	0.991	3	15	regulation of fatty acid biosynthetic process	BP	0.874	TRIB3,PTGS2,SLC45A3	down_VSAP_EC
GO:0060462	0.0532	0.996	2	4	lung lobe development	BP	1	FGFR2,SEC24B	up_ACOR_VSMC
GO:0060463	0.0532	0.996	2	4	lung lobe morphogenesis	BP	1	FGFR2,SEC24B	up_ACOR_VSMC
GO:1905063	0.0532	0.996	2	5	regulation of vascular smooth muscle cell	BP	0.884	KIT,PDGFB	up_VCAV_EC
GO:0035313	0.0532	0.999	1	7	wound healing, spreading of epidermal cells	BP	1	ACVRL1	down_ACOR_EC
GO:1901379	0.0532	0.999	1	12	regulation of potassium ion transmembrane	BP	1	NEDD4	down_ACOR_EC
GO:0006169	0.0532	1	1	1	adenosine salvage	BP	1	ADK	down_VCAV_EC
GO:0006175	0.0532	1	1	1	dATP biosynthetic process	BP	1	ADK	down_VCAV_EC
GO:0009153	0.0532	1	1	1	purine deoxyribonucleotide	BP	1	ADK	down_VCAV_EC
GO:0009202	0.0532	1	1	1	deoxyribonucleoside triphosphate biosynthetic	BP	1	ADK	down_VCAV_EC
GO:0018400	0.0532	1	1	1	peptidyl-proline hydroxylation to 3-	BP	1	CRTAP	down_VCAV_EC
GO:0046086	0.0532	1	1	1	adenosine biosynthetic process	BP	1	ADK	down_VCAV_EC
GO:0009216	0.0532	1	1	1	purine deoxyribonucleoside	BP	1	ADK	down_VCAV_EC
GO:0070170	0.0532	1	1	1	regulation of tooth mineralization	BP	1	ASPN	down_VCAV_EC
GO:0070171	0.0532	1	1	1	negative regulation of tooth mineralization	BP	1	ASPN	down_VCAV_EC
GO:0090107	0.0532	1	1	1	regulation of high-density lipoprotein particle	BP	1	LCAT	down_VCAV_EC
GO:0014031	0.0532	0.999	1	27	mesenchymal cell development	BP	1	CITED2	down_VPOR_EC
GO:0014032	0.0532	0.999	1	27	neural crest cell development	BP	1	CITED2	down_VPOR_EC
GO:0006707	0.0532	1	1	1	cholesterol catabolic process	BP	0.874	APOE	down_VSAP_EC
GO:0010873	0.0532	1	1	1	positive regulation of cholesterol esterification	BP	0.874	APOE	down_VSAP_EC
GO:0010877	0.0532	1	1	1	lipid transport involved in lipid storage	BP	0.874	APOE	down_VSAP_EC
GO:0032802	0.0532	1	1	1	low-density lipoprotein particle receptor catabolic	BP	0.874	APOE	down_VSAP_EC
GO:0032803	0.0532	1	1	1	regulation of low-density lipoprotein particle receptor catabolic process	BP	0.874	APOE	down_VSAP_EC
GO:0032805	0.0532	1	1	1	positive regulation of low-density lipoprotein particle receptor catabolic	BP	0.874	APOE	down_VSAP_EC
GO:0090731	0.0532	1	1	1	cellular response to very-low-density lipoprotein particle stimulus	BP	0.874	APOE	down_VSAP_EC
GO:0097113	0.0532	1	1	1	AMPA glutamate receptor clustering	BP	0.874	APOE	down_VSAP_EC
GO:0097688	0.0532	1	1	1	glutamate receptor clustering	BP	0.874	APOE	down_VSAP_EC

GO:1901630	0.0532	1	1	1	negative regulation of presynaptic membrane	BP	0.874	APOE	down_VSAP_EC
GO:1902952	0.0532	1	1	1	positive regulation of dendritic spine	BP	0.874	APOE	down_VSAP_EC
GO:1902994	0.0532	1	1	1	regulation of phospholipid efflux	BP	0.874	APOE	down_VSAP_EC
GO:1902995	0.0532	1	1	1	positive regulation of phospholipid efflux	BP	0.874	APOE	down_VSAP_EC
GO:1903000	0.0532	1	1	1	regulation of lipid transport across blood	BP	0.874	APOE	down_VSAP_EC
GO:1903002	0.0532	1	1	1	positive regulation of lipid transport across blood brain barrier	BP	0.874	APOE	down_VSAP_EC
GO:1905853	0.0532	1	1	1	regulation of heparan sulfate binding	BP	0.874	APOE	down_VSAP_EC
GO:1905855	0.0532	1	1	1	positive regulation of heparan sulfate binding	BP	0.874	APOE	down_VSAP_EC
GO:1905858	0.0532	1	1	1	regulation of heparan sulfate proteoglycan	BP	0.874	APOE	down_VSAP_EC
GO:1905860	0.0532	1	1	1	positive regulation of heparan sulfate	BP	0.874	APOE	down_VSAP_EC
GO:1905890	0.0532	1	1	1	regulation of cellular response to very-low-density lipoprotein	BP	0.874	APOE	down_VSAP_EC
GO:1990379	0.0532	1	1	1	lipid transport across blood brain barrier	BP	0.874	APOE	down_VSAP_EC
GO:0016127	0.0532	1	1	1	sterol catabolic process	BP	0.874	APOE	down_VSAP_EC
GO:0008038	0.0532	0.999	1	7	neuron recognition	BP	1	SEMA3A	up_VPOR_EC
GO:0072615	0.053219172	0.99928	1	2	interleukin-17 secretion	BP	1	NR1H4	up_ACOR_EC
GO:0072173	0.053299059	0.99927	1	2	metanephric tubule morphogenesis	BP	1	SOX9	up_ACOR_EC
GO:0072174	0.053299059	0.99927	1	2	metanephric tubule formation	BP	1	SOX9	up_ACOR_EC
GO:0072282	0.053299059	0.99927	1	2	metanephric nephron tubule morphogenesis	BP	1	SOX9	up_ACOR_EC
GO:0072289	0.053299059	0.99927	1	2	metanephric nephron tubule formation	BP	1	SOX9	up_ACOR_EC
GO:0030833	0.0533	0.978	9	59	regulation of actin filament polymerization	BP	0.884	HCLS1,BAIAP2L1,TMOD1,MTOR,NCKAP1,IQGAP2,NCK1,ARPC5L,TMOD3	up_VCAV_EC
GO:0048731	0.0533	0.981	8	1247	system development	BP	1	TBX5,HOXA3,NR1H4,EVI2B,CCL24,FLRT3,ARHGEF2,NEO1	up_VPUL_EC
GO:0072172	0.0533	0.995	2	7	mesonephric tubule formation	BP	0.874	ENSCAFG00000032470,SIX4	down_VSAP_EC
GO:0072359	0.0533	1	1	368	circulatory system development	BP	1	GATA5	down_AFEM_VSMC
GO:0060979	0.0533	1	1	1	vasculogenesis involved in coronary vascular	BP	1	SPRED1	down_VCAV_EC
GO:0071484	0.0533	1	1	1	cellular response to light intensity	BP	1	AKT2	down_VCAV_EC
GO:0071486	0.0533	1	1	1	cellular response to high light intensity	BP	1	AKT2	down_VCAV_EC
GO:0097473	0.0533	1	1	1	retinal rod cell apoptotic process	BP	1	AKT2	down_VCAV_EC
GO:2001136	0.0533	1	1	1	negative regulation of endocytic recycling	BP	1	ARHGAP1	down_VCAV_EC
GO:0010918	0.0533	1	1	1	positive regulation of mitochondrial membrane potential	BP	1	AKT2	down_VCAV_EC
GO:0021527	0.0533	1	1	1	spinal cord association neuron differentiation	BP	1	LMO4	down_VCAV_EC
GO:0042078	0.0533	1	1	1	germ-line stem cell division	BP	1	ZBTB16	down_VCAV_EC
GO:0048133	0.0533	1	1	1	male germ-line stem cell asymmetric division	BP	1	ZBTB16	down_VCAV_EC
GO:0098728	0.0533	1	1	1	germline stem cell asymmetric division	BP	1	ZBTB16	down_VCAV_EC
GO:0090087	0.0533	0.999	1	185	regulation of peptide transport	BP	1	JUP	down_VFEM_VSMC
GO:0030239	0.0533	0.999	1	21	myofibril assembly	BP	1	NKX2-5	up_VPOR_VSMC
GO:0007215	0.0533	0.999	1	13	glutamate receptor signaling pathway	BP	1	NETO1	up_VSAP_VSMC
GO:0042398	0.0533	0.999	1	12	cellular modified amino acid biosynthetic process	BP	1	GATM	up_VSAP_VSMC
GO:0007352	0.053328539	0.99927	1	2	zygotic specification of dorsal/ventral axis	BP	1	SMAD6	up_ACOR_EC

GO:0010988	0.053391673	0.99927	1	2	regulation of low-density lipoprotein particle	BP	1	NR1H4	up_ACOR_EC
GO:0009888	0.0534	0.985	5	545	tissue development	BP	1	NR1H4,TBX5,HOXA3,ARHGEF26,NPNT	up_APUL_EC
GO:0035150	0.0534	0.99	3	36	regulation of tube size	BP	1	HTR1D,AVPR1A,TBXA2R	up_AFEM_EC
GO:0050880	0.0534	0.99	3	36	regulation of blood vessel size	BP	1	HTR1D,AVPR1A,TBXA2R	up_AFEM_EC
GO:0019953	0.0534	0.994	2	152	sexual reproduction	BP	1	HOXA9,HOXA11	up_VFEM_VSMC
GO:0097193	0.0534	0.994	2	109	intrinsic apoptotic signaling pathway	BP	1	PTGER2,ARHGEF2	up_VPUL_EC
GO:0002438	0.0534	1	1	1	acute inflammatory response to antigenic	BP	1	PARK7	down_VCAV_EC
GO:0002866	0.0534	1	1	1	positive regulation of acute inflammatory response to antigenic	BP	1	PARK7	down_VCAV_EC
GO:0006030	0.0534	1	1	1	chitin metabolic process	BP	1	CHID1	down_VCAV_EC
GO:0006032	0.0534	1	1	1	chitin catabolic process	BP	1	CHID1	down_VCAV_EC
GO:0006780	0.0534	1	1	1	uroporphyrinogen III biosynthetic process	BP	1	UROS	down_VCAV_EC
GO:0010273	0.0534	1	1	1	detoxification of copper ion	BP	1	PARK7	down_VCAV_EC
GO:0015680	0.0534	1	1	1	protein maturation by copper ion transfer	BP	1	ATOX1	down_VCAV_EC
GO:0017187	0.0534	1	1	1	peptidyl-glutamic acid carboxylation	BP	1	VKORC1	down_VCAV_EC
GO:0018323	0.0534	1	1	1	enzyme active site formation via L-cysteine	BP	1	PARK7	down_VCAV_EC
GO:0032905	0.0534	1	1	1	transforming growth factor beta1 production	BP	1	LUM	down_VCAV_EC
GO:0032908	0.0534	1	1	1	regulation of transforming growth factor beta1	BP	1	LUM	down_VCAV_EC
GO:0032914	0.0534	1	1	1	positive regulation of transforming growth factor beta1 production	BP	1	LUM	down_VCAV_EC
GO:0033860	0.0534	1	1	1	regulation of NAD(P)H oxidase activity	BP	1	PARK7	down_VCAV_EC
GO:0033864	0.0534	1	1	1	positive regulation of NAD(P)H oxidase activity	BP	1	PARK7	down_VCAV_EC
GO:0002158	0.0534	1	1	1	osteoclast proliferation	BP	1	GREM1	down_VCAV_EC
GO:0034164	0.0534	1	1	1	negative regulation of toll-like receptor 9 signaling	BP	1	RAB7B	down_VCAV_EC
GO:0036525	0.0534	1	1	1	protein deglycation	BP	1	PARK7	down_VCAV_EC
GO:0036526	0.0534	1	1	1	peptidyl-cysteine deglycation	BP	1	PARK7	down_VCAV_EC
GO:0036527	0.0534	1	1	1	peptidyl-arginine deglycation	BP	1	PARK7	down_VCAV_EC
GO:0036528	0.0534	1	1	1	peptidyl-lysine deglycation	BP	1	PARK7	down_VCAV_EC
GO:0036529	0.0534	1	1	1	protein deglycation, glyoxal removal	BP	1	PARK7	down_VCAV_EC
GO:0036530	0.0534	1	1	1	protein deglycation, methylglyoxal removal	BP	1	PARK7	down_VCAV_EC
GO:0036531	0.0534	1	1	1	glutathione deglycation	BP	1	PARK7	down_VCAV_EC
GO:0042373	0.0534	1	1	1	vitamin K metabolic process	BP	1	VKORC1	down_VCAV_EC
GO:0046826	0.0534	1	1	1	negative regulation of protein export from	BP	1	PARK7	down_VCAV_EC
GO:0046851	0.0534	1	1	1	negative regulation of bone remodeling	BP	1	GREM1	down_VCAV_EC
GO:0002863	0.0534	1	1	1	positive regulation of inflammatory response to antigenic stimulus	BP	1	PARK7	down_VCAV_EC
GO:0002864	0.0534	1	1	1	regulation of acute inflammatory response to	BP	1	PARK7	down_VCAV_EC
GO:0050787	0.0534	1	1	1	detoxification of mercury ion	BP	1	PARK7	down_VCAV_EC
GO:0060081	0.0534	1	1	1	membrane hyperpolarization	BP	1	PARK7	down_VCAV_EC
GO:0061687	0.0534	1	1	1	detoxification of inorganic compound	BP	1	PARK7	down_VCAV_EC
GO:0090086	0.0534	1	1	1	negative regulation of protein deubiquitination	BP	1	PARK7	down_VCAV_EC

GO:0090289	0.0534	1	1	1	regulation of osteoclast proliferation	BP	1	GREM1	down_VCAV_EC
GO:0090291	0.0534	1	1	1	negative regulation of osteoclast proliferation	BP	1	GREM1	down_VCAV_EC
GO:0097238	0.0534	1	1	1	cellular response to methylglyoxal	BP	1	PARK7	down_VCAV_EC
GO:0097501	0.0534	1	1	1	stress response to metal ion	BP	1	PARK7	down_VCAV_EC
GO:0106044	0.0534	1	1	1	guanine deglycation	BP	1	PARK7	down_VCAV_EC
GO:0106045	0.0534	1	1	1	guanine deglycation, methylglyoxal removal	BP	1	PARK7	down_VCAV_EC
GO:0106046	0.0534	1	1	1	guanine deglycation, glyoxal removal	BP	1	PARK7	down_VCAV_EC
GO:0140041	0.0534	1	1	1	cellular detoxification of methylglyoxal	BP	1	PARK7	down_VCAV_EC
GO:1901671	0.0534	1	1	1	positive regulation of superoxide dismutase	BP	1	PARK7	down_VCAV_EC
GO:1902938	0.0534	1	1	1	regulation of intracellular calcium activated chloride	BP	1	PRNP	down_VCAV_EC
GO:1902958	0.0534	1	1	1	positive regulation of mitochondrial electron transport, NADH to	BP	1	PARK7	down_VCAV_EC
GO:1903031	0.0534	1	1	1	regulation of microtubule plus-end binding	BP	1	MAPRE3	down_VCAV_EC
GO:1903033	0.0534	1	1	1	positive regulation of microtubule plus-end	BP	1	MAPRE3	down_VCAV_EC
GO:1903094	0.0534	1	1	1	negative regulation of protein K48-linked	BP	1	PARK7	down_VCAV_EC
GO:1903122	0.0534	1	1	1	negative regulation of TRAIL-activated apoptotic signaling pathway	BP	1	PARK7	down_VCAV_EC
GO:1903167	0.0534	1	1	1	regulation of pyrroline-5-carboxylate reductase	BP	1	PARK7	down_VCAV_EC
GO:1903168	0.0534	1	1	1	positive regulation of pyrroline-5-carboxylate	BP	1	PARK7	down_VCAV_EC
GO:1903184	0.0534	1	1	1	L-dopa metabolic process	BP	1	PARK7	down_VCAV_EC
GO:1903185	0.0534	1	1	1	L-dopa biosynthetic process	BP	1	PARK7	down_VCAV_EC
GO:1903189	0.0534	1	1	1	glyoxal metabolic process	BP	1	PARK7	down_VCAV_EC
GO:1903195	0.0534	1	1	1	regulation of L-dopa biosynthetic process	BP	1	PARK7	down_VCAV_EC
GO:1903197	0.0534	1	1	1	positive regulation of L-dopa biosynthetic process	BP	1	PARK7	down_VCAV_EC
GO:1903383	0.0534	1	1	1	regulation of hydrogen peroxide-induced neuron intrinsic apoptotic	BP	1	PARK7	down_VCAV_EC
GO:1903384	0.0534	1	1	1	negative regulation of hydrogen peroxide-induced neuron intrinsic	BP	1	PARK7	down_VCAV_EC
GO:1903750	0.0534	1	1	1	regulation of intrinsic apoptotic signaling pathway in response to	BP	1	PARK7	down_VCAV_EC
GO:1903751	0.0534	1	1	1	negative regulation of intrinsic apoptotic signaling pathway in	BP	1	PARK7	down_VCAV_EC
GO:1903795	0.0534	1	1	1	regulation of inorganic anion transmembrane	BP	1	PRNP	down_VCAV_EC
GO:1904833	0.0534	1	1	1	positive regulation of removal of superoxide	BP	1	PARK7	down_VCAV_EC
GO:1905258	0.0534	1	1	1	regulation of nitrosative stress-induced intrinsic apoptotic signaling	BP	1	PARK7	down_VCAV_EC
GO:1905259	0.0534	1	1	1	negative regulation of nitrosative stress-induced intrinsic apoptotic	BP	1	PARK7	down_VCAV_EC
GO:2000284	0.0534	1	1	1	positive regulation of cellular amino acid	BP	1	PARK7	down_VCAV_EC
GO:0009441	0.0534	1	1	1	glycolate metabolic process	BP	1	PARK7	down_VCAV_EC
GO:0018214	0.0534	1	1	1	protein carboxylation	BP	1	VKORC1	down_VCAV_EC
GO:0031204	0.0534	1	1	1	posttranslational protein targeting to membrane, translocation	BP	1	SEC61B	down_VCAV_EC
GO:0036376	0.0534	1	1	1	sodium ion export across plasma membrane	BP	1	FXYD1	down_VCAV_EC

GO:0036471	0.0534	1	1	1	cellular response to glyoxal	BP	1	PARK7	down_VCAV_EC
GO:0036482	0.0534	1	1	1	neuron intrinsic apoptotic signaling pathway in response to hydrogen	BP	1	PARK7	down_VCAV_EC
GO:0046295	0.0534	1	1	1	glycolate biosynthetic process	BP	1	PARK7	down_VCAV_EC
GO:0046360	0.0534	1	1	1	2-oxobutyrate biosynthetic process	BP	1	THNSL2	down_VCAV_EC
GO:0046361	0.0534	1	1	1	2-oxobutyrate metabolic process	BP	1	THNSL2	down_VCAV_EC
GO:0046502	0.0534	1	1	1	uroporphyrinogen III metabolic process	BP	1	UROS	down_VCAV_EC
GO:0048874	0.0534	1	1	1	host-mediated regulation of intestinal microbiota	BP	1	NAPEPLD	down_VCAV_EC
GO:0070291	0.0534	1	1	1	N-acylethanolamine metabolic process	BP	1	NAPEPLD	down_VCAV_EC
GO:0070292	0.0534	1	1	1	N-acylphosphatidylethanola	BP	1	NAPEPLD	down_VCAV_EC
GO:0097403	0.0534	1	1	1	cellular response to raffinose	BP	1	ZFP36L1	down_VCAV_EC
GO:1901545	0.0534	1	1	1	response to raffinose	BP	1	ZFP36L1	down_VCAV_EC
GO:1903259	0.0534	1	1	1	exon-exon junction complex disassembly	BP	1	PYM1	down_VCAV_EC
GO:1903276	0.0534	1	1	1	regulation of sodium ion export across plasma	BP	1	FXYD1	down_VCAV_EC
GO:1903278	0.0534	1	1	1	positive regulation of sodium ion export across	BP	1	FXYD1	down_VCAV_EC
GO:1904580	0.0534	1	1	1	regulation of intracellular mRNA localization	BP	1	ZFP36L1	down_VCAV_EC
GO:1904582	0.0534	1	1	1	positive regulation of intracellular mRNA	BP	1	ZFP36L1	down_VCAV_EC
GO:1990169	0.0534	1	1	1	stress response to copper ion	BP	1	PARK7	down_VCAV_EC
GO:2000152	0.0534	1	1	1	regulation of ubiquitin-specific protease activity	BP	1	PARK7	down_VCAV_EC
GO:2000157	0.0534	1	1	1	negative regulation of ubiquitin-specific	BP	1	PARK7	down_VCAV_EC
GO:2000277	0.0534	1	1	1	positive regulation of oxidative phosphorylation uncoupler activity	BP	1	PARK7	down_VCAV_EC
GO:0007498	0.0534	0.999	1	38	mesoderm development	BP	1	EYA1	up_AFEM_VSMC
GO:0048639	0.0534	0.999	1	39	positive regulation of developmental growth	BP	1	LIMK1	up_VCAV_VSMC
GO:0003197	0.0534	0.999	1	16	endocardial cushion development	BP	1	TBX5	up_VPUL_EC
GO:0060079	0.0534	0.999	1	15	excitatory postsynaptic potential	BP	1	NETO1	up_VSAP_VSMC
GO:0003285	0.053406829	0.99927	1	2	septum secundum development	BP	1	GATA4	up_ACOR_EC
GO:0032502	0.053417361	0.96248	54	1661	developmental process	BP	1	SH3TC2,MYOZ2,CPAMD8,CDH3,NR1H4,TMOD1,GATA4,BRSK1,TRPS1,TBX18,SOX9,RHOU,PTH1R,NDRG2,AQP1,ENSCAFG00000004331,FZD5,CCDC3,SEMA6D,DIXDC1,PDLIM3,RGMA,LHX9,SMAD6,PLS1,ACTN2,ESM1,PAK3,IL11RA,DLX3,TRIM8,TNFRSF19,PTGER4,ARHGDIB,PIAS1,DCLK2,ADAMTSL2,MAPK8IP2,FOXC2,FIGNL1,TBX5,LRP4,CLSTN1,PRELP,RBM38,C23H3orf58,TP53INP2,RARRES2,NRP1,ARHGEF26,PRKCD,PYGO2,DBNDD2,EFEMP1	up_ACOR_EC
GO:0003209	0.053420275	0.99433	2	14	cardiac atrium morphogenesis	BP	1	GATA4,TBX5	up_ACOR_EC
GO:0003230	0.053420275	0.99433	2	14	cardiac atrium development	BP	1	GATA4,TBX5	up_ACOR_EC
GO:0048865	0.053435013	0.99927	1	2	stem cell fate commitment	BP	1	SOX9	up_ACOR_EC
GO:0048866	0.053435013	0.99927	1	2	stem cell fate specification	BP	1	SOX9	up_ACOR_EC
GO:0046813	0.053449184	0.99927	1	2	receptor-mediated virion attachment to host cell	BP	1	LRRC15	up_ACOR_EC
GO:0048870	0.053450813	0.96992	18	451	cell motility	BP	1	SOX9,AQP1,ENSCAFG00000004331,SEMA6D,DIXDC1,ZMYND8,PAK3,MMP28,PTGER4,ARHGDIB,ELMO2,FOXC2,LRRC15,TBX5,RARRES2,NRP1,PRKCD,CCNYL1	up_ACOR_EC

GO:0051674	0.053450813	0.96992	18	451	localization of cell	BP	1	SOX9,AQP1,ENSCAFG00000004331,SEMA6D,DIXDC1,ZMYND8,PAK3,MMP28,PTGER4,ARHGDIB,ELMO2,FOXC2,LRRC15,TBX5,RARRES2,NRP1,PRKCD,CCNYL1	up_ACOR_EC
GO:0003164	0.053475349	0.99927	1	2	His-Purkinje system development	BP	1	TBX5	up_ACOR_EC
GO:0003166	0.053475349	0.99927	1	2	bundle of His development	BP	1	TBX5	up_ACOR_EC
GO:0040008	0.0535	0.967	24	179	regulation of growth	BP	1	ENSCAFG00000009113,WNT11,HNF4A,FAM107A,ACACB,SLIT2,SLC6A4,ABL1,DLL1,SAV1,PLXNA4,MT2A,FXN,MYD88,DPYSL2,WISP2,KDM2B,IGFBP6,TTL,SMARCA2,ACVRL1,HTRA2,TGFBR2,LATS2	up_VSAP_EC
GO:0022603	0.0535	0.974	12	301	regulation of anatomical structure morphogenesis	BP	1	LEP,ANGPT4,DLL1,F3,PLXNA4,THRB,TMEM100,GREM1,TBXA2R,BVES,CDC42EP5,KANK1	up_AFEM_EC
GO:0043542	0.0535	0.984	5	86	endothelial cell migration	BP	1	ANGPT4,GREM1,TBXA2R,FAP,ADAM17	up_AFEM_EC
GO:1990001	0.0535	0.996	2	5	inhibition of cysteine-type endopeptidase activity involved in apoptotic	BP	1	BIRC5,ENSCAFG00000005299	down_ACOR_VSMC
GO:0071363	0.0535	0.994	2	188	cellular response to growth factor stimulus	BP	1	GATA5,SLIT2	up_VPUL_VSMC
GO:0044706	0.0535	0.999	1	27	multi-multicellular organism process	BP	1	CITED2	down_VPOR_EC
GO:2000117	0.0535	0.999	1	27	negative regulation of cysteine-type	BP	1	CST3	down_VPOR_EC
GO:0030010	0.0535	0.999	1	41	establishment of cell polarity	BP	1	WNT7A	up_AORT_VSMC
GO:0010922	0.0535	0.999	1	14	positive regulation of phosphatase activity	BP	1	NPNT	up_APUL_EC
GO:0060330	0.0536	0.995	2	6	regulation of response to interferon-gamma	BP	0.874	PTPN2,CDC37	down_VSAP_EC
GO:0060334	0.0536	0.995	2	6	regulation of interferon-gamma-mediated	BP	0.874	PTPN2,CDC37	down_VSAP_EC
GO:0089718	0.0536	0.996	2	4	amino acid import across plasma membrane	BP	1	PSEN1,CLN8	up_ACOR_VSMC
GO:1902475	0.0536	0.996	2	4	L-alpha-amino acid transmembrane transport	BP	1	PSEN1,CLN8	up_ACOR_VSMC
GO:0043090	0.0536	0.996	2	4	amino acid import	BP	1	PSEN1,CLN8	up_ACOR_VSMC
GO:0050866	0.0536	0.999	1	48	negative regulation of cell activation	BP	1	ARG2	up_AORT_VSMC
GO:0060043	0.0536	0.999	1	14	regulation of cardiac muscle cell proliferation	BP	1	TBX5	up_APUL_EC
GO:0038034	0.0536	0.999	1	21	signal transduction in absence of ligand	BP	1	EYA1	up_VFEM_VSMC
GO:0097192	0.0536	0.999	1	21	extrinsic apoptotic signaling pathway in	BP	1	EYA1	up_VFEM_VSMC
GO:0010828	0.0536	0.999	1	16	positive regulation of glucose transmembrane	BP	1	NR1H4	up_VPUL_EC
GO:1902714	0.053630067	0.99927	1	2	negative regulation of interferon-gamma	BP	1	NR1H4	up_ACOR_EC
GO:1904425	0.053652523	0.99926	1	2	negative regulation of GTP binding	BP	1	ARHGAP28	up_ACOR_EC
GO:0055021	0.0537	0.988	4	15	regulation of cardiac muscle tissue growth	BP	1	FGFR2,TBX20,TBX5,EDN1	up_ACOR_VSMC
GO:0045600	0.0537	0.987	4	21	positive regulation of fat cell differentiation	BP	0.884	PPARG,BMP2,PTGS2,CREB1	up_VCAV_EC
GO:0071363	0.0537	0.999	1	188	cellular response to growth factor stimulus	BP	1	GATA5	down_VFEM_VSMC
GO:0045055	0.0537	0.999	1	52	regulated exocytosis	BP	1	WNT7A	up_AORT_VSMC
GO:0097178	0.0537	0.999	1	14	ruffle assembly	BP	1	ARHGEF26	up_APUL_EC
GO:0048813	0.0537	0.999	1	39	dendrite morphogenesis	BP	1	SLITRK5	up_VCAV_VSMC
GO:0007350	0.0537	0.999	1	7	blastoderm segmentation	BP	1	SEMA3A	up_VPOR_EC
GO:0001569	0.053700717	0.99428	2	14	branching involved in blood vessel	BP	1	FOXC2,NRP1	up_ACOR_EC
GO:1902308	0.053725797	0.99926	1	2	regulation of peptidyl-serine dephosphorylation	BP	1	DUSP26	up_ACOR_EC
GO:0071398	0.053783166	0.99427	2	14	cellular response to fatty acid	BP	1	NR1H4,PTGER4	up_ACOR_EC
GO:0051648	0.0538	0.994	2	69	vesicle localization	BP	1	SPG11,STX4	down_ACOR_EC
GO:0038083	0.0538	0.994	2	16	peptidyl-tyrosine autophosphorylation	BP	1	HCK,GREM1	up_AFEM_EC

GO:0002704	0.0538	0.996	2	5	negative regulation of leukocyte mediated	BP	0.884	IL7R,THOC1	up_VCAV_EC
GO:0006635	0.0538	0.999	1	25	fatty acid beta-oxidation	BP	1	ETFA	down_AFEM_EC
GO:0097581	0.0538	0.999	1	26	lamellipodium organization	BP	1	FSCN1	down_AFEM_EC
GO:0070201	0.0538	0.999	1	187	regulation of establishment of protein	BP	1	JUP	down_VFEM_VSMC
GO:0014916	0.0538	1	1	1	regulation of lung blood pressure	BP	0.884	GCH1	up_VCAV_EC
GO:0046654	0.0538	1	1	1	tetrahydrofolate biosynthetic process	BP	0.884	GCH1	up_VCAV_EC
GO:0006720	0.0538	0.999	1	25	isoprenoid metabolic process	BP	1	CYP26B1	up_VPOR_VSMC
GO:2000736	0.0538	0.999	1	21	regulation of stem cell differentiation	BP	1	NKX2-5	up_VPOR_VSMC
GO:0001523	0.0538	0.999	1	12	retinoid metabolic process	BP	1	CYP26B1	up_VFEM_EC
GO:0035136	0.0538	0.999	1	16	forelimb morphogenesis	BP	1	TBX5	up_VPUL_EC
GO:0014904	0.0538	0.999	1	12	myotube cell development	BP	1	SHOX2	up_VSAP_VSMC
GO:0048741	0.0538	0.999	1	12	skeletal muscle fiber development	BP	1	SHOX2	up_VSAP_VSMC
GO:1901628	0.053805325	0.99926	1	2	positive regulation of postsynaptic membrane organization	BP	1	LRP4	up_ACOR_EC
GO:1901629	0.053805325	0.99926	1	2	regulation of presynaptic membrane organization	BP	1	LRP4	up_ACOR_EC
GO:0030837	0.053863716	0.99425	2	14	negative regulation of actin filament	BP	1	TMOD1,PRKCD	up_ACOR_EC
GO:0045666	0.0539	0.978	8	68	positive regulation of neuron differentiation	BP	0.874	ARHGEF2,DPYSL3,APOE,LYN,MMD,VLDLR,REST,NRP1	down_VSAP_EC
GO:0002687	0.0539	0.99	3	36	positive regulation of leukocyte migration	BP	1	CMKLR1,CCL5,ADAM17	up_AFEM_EC
GO:0032682	0.0539	0.995	2	7	negative regulation of chemokine production	BP	0.874	NR1H4,SOCS5	down_VSAP_EC
GO:0106049	0.0539	0.996	2	4	regulation of cellular response to osmotic	BP	0.884	PTGS2,ARHGEF2	up_VCAV_EC
GO:1902218	0.0539	0.996	2	4	regulation of intrinsic apoptotic signaling pathway in response to	BP	0.884	PTGS2,ARHGEF2	up_VCAV_EC
GO:1902219	0.0539	0.996	2	4	negative regulation of intrinsic apoptotic signaling pathway in	BP	0.884	PTGS2,ARHGEF2	up_VCAV_EC
GO:0034439	0.0539	1	1	1	lipoprotein lipid oxidation	BP	0.884	APOD	up_VCAV_EC
GO:0034442	0.0539	1	1	1	regulation of lipoprotein oxidation	BP	0.884	APOD	up_VCAV_EC
GO:0034443	0.0539	1	1	1	negative regulation of lipoprotein oxidation	BP	0.884	APOD	up_VCAV_EC
GO:0042160	0.0539	1	1	1	lipoprotein modification	BP	0.884	APOD	up_VCAV_EC
GO:0042161	0.0539	1	1	1	lipoprotein oxidation	BP	0.884	APOD	up_VCAV_EC
GO:0050748	0.0539	1	1	1	negative regulation of lipoprotein metabolic	BP	0.884	APOD	up_VCAV_EC
GO:0060587	0.0539	1	1	1	regulation of lipoprotein lipid oxidation	BP	0.884	APOD	up_VCAV_EC
GO:0060588	0.0539	1	1	1	negative regulation of lipoprotein lipid oxidation	BP	0.884	APOD	up_VCAV_EC
GO:2000405	0.0539	1	1	1	negative regulation of T cell migration	BP	0.884	APOD	up_VCAV_EC
GO:2001258	0.0539	0.999	1	11	negative regulation of cation channel activity	BP	1	CAV1	up_VSAP_VSMC
GO:0070858	0.053948127	0.99926	1	2	negative regulation of bile acid biosynthetic process	BP	1	NR1H4	up_ACOR_EC
GO:1904252	0.053948127	0.99926	1	2	negative regulation of bile acid metabolic process	BP	1	NR1H4	up_ACOR_EC
GO:2000145	0.054	0.967	24	279	regulation of cell motility	BP	0.874	ARHGEF2,VEGFA,SULF1,HSPA5,SERPINE1,SPARC,JCAD,DPYSL3,PRCP,MYO1C,PIK3C2A,MSN,HIF1A,ADAM17,STARD13,APOE,PTP4A1,MMP28,LYN,STRAP,NEXN,SDC4,ZMYND8,NRP1	down_VSAP_EC
GO:0050849	0.054	0.99	3	15	negative regulation of calcium-mediated	BP	0.874	FHL2,GSK3B,CALM1	down_VSAP_EC
GO:0014823	0.054	0.996	2	5	response to activity	BP	0.884	SLC25A25,HIF1A	up_VCAV_EC
GO:0032504	0.054	0.994	2	152	multicellular organism reproduction	BP	1	HOXA9,HOXA11	up_VFEM_VSMC

GO:0048609	0.054	0.994	2	152	multicellular organismal reproductive process	BP	1	HOXA9,HOXA11	up_VFEM_VSMC
GO:0035493	0.054	0.999	1	11	SNARE complex assembly	BP	1	STX4	down_ACOR_EC
GO:0060041	0.054	0.999	1	41	retina development in camera-type eye	BP	1	IRX5	up_AFEM_VSMC
GO:0043393	0.054	0.999	1	77	regulation of protein binding	BP	1	HOXA3	up_APUL_VSMC
GO:0016998	0.054	1	1	1	cell wall macromolecule catabolic process	BP	0.884	LYZ	up_VCAV_EC
GO:0044036	0.054	1	1	1	cell wall macromolecule metabolic process	BP	0.884	LYZ	up_VCAV_EC
GO:0071554	0.054	1	1	1	cell wall organization or biogenesis	BP	0.884	LYZ	up_VCAV_EC
GO:0006680	0.054090452	0.99925	1	2	glucosylceramide catabolic process	BP	1	PRKCD	up_ACOR_EC
GO:0031324	0.0541	0.961	55	735	negative regulation of cellular metabolic process	BP	0.874	PRICKLE1,NR1H4,PLK3,SERPINE1,SOCS5,ETV6,ENPP1,ERRFI1,SNX25,CBX5,TRIB3,FHL2,SMARCA4,SESN2,PTGS2,HIF1A,DYNLL1,DNAJC10,NXN,PTPN2,UCHL5,MORF4L2,SPOPL,PEG3,MTDH,EIF4EBP1,CNOT7,MAGED1,CTNNB1,CRYAB,ENSCAFG00000002015,APOE,WWC3,KLF5,LYN,BNIP3,STRAP,DDIT3,CBLC,ATF3,VLDLR,REST,ZMYND8,EIF2S1,ARFGEF3,CDYL,PAK2,LRRTM2,A2M,HIF1AN,SIX4,CDKN1A,SPINK2,USP14,KDM3A	down_VSAP_EC
GO:0007167	0.0541	0.968	20	274	enzyme linked receptor protein signaling pathway	BP	1	PDGFRB,SMAD9,DCN,NDN,ANGPT1,VASN,FBN1,BMPR1A,IGFBP6,ENSCAFG00000014978,AKT1S1,CHRDL1,AKT2,CSPG4,ASPN,FUT8,ENSCAFG00000017708,SHC1,GREM1,RGMA	down_VCAV_EC
GO:0034641	0.0541	0.987	6	2171	cellular nitrogen compound metabolic	BP	1	TLR2,TBX15,EYA1,HOXA9,HOXA10,IRX5	up_AFEM_VSMC
GO:1903959	0.0541	0.995	2	8	regulation of anion transmembrane transport	BP	1	PRNP,AKT2	down_VCAV_EC
GO:0043586	0.0541	0.995	2	6	tongue development	BP	0.874	CTNNB1,SIX4	down_VSAP_EC
GO:0007632	0.0541	0.999	1	14	visual behavior	BP	1	HOXA1	up_APUL_EC
GO:0048635	0.0541	0.999	1	14	negative regulation of muscle organ	BP	1	TBX5	up_APUL_EC
GO:2000826	0.0541	0.999	1	14	regulation of heart morphogenesis	BP	1	TBX5	up_APUL_EC
GO:0010524	0.0541	0.999	1	12	positive regulation of calcium ion transport into	BP	1	CAV1	up_VSAP_VSMC
GO:1901021	0.0541	0.999	1	13	positive regulation of calcium ion transmembrane	BP	1	STIM1	up_VSAP_VSMC
GO:0035356	0.054176071	0.99925	1	2	cellular triglyceride homeostasis	BP	1	NR1H4	up_ACOR_EC
GO:1904177	0.054176071	0.99925	1	2	regulation of adipose tissue development	BP	1	NR1H4	up_ACOR_EC
GO:1904179	0.054176071	0.99925	1	2	positive regulation of adipose tissue	BP	1	NR1H4	up_ACOR_EC
GO:0019222	0.0542	0.976	13	1934	regulation of metabolic process	BP	1	HOXA11,HOXA10,HOXA9,PITX1,EMX2,GATM,SALL1,HOXC10,IRX5,PRR16,TLR2,CAV1,SHOX2	up_VSAP_VSMC
GO:0048008	0.0542	0.985	5	24	platelet-derived growth factor receptor signaling	BP	0.884	PDGFB,TIPARP,RAPGEF1,APOD,PDGFD	up_VCAV_EC
GO:0042987	0.0542	0.991	3	10	amyloid precursor protein catabolic process	BP	1	PSEN2,APP,PSEN1	up_ACOR_VSMC
GO:0031579	0.0542	0.995	2	6	membrane raft organization	BP	1	CAV1,EMP2	down_ACOR_VSMC
GO:0032490	0.0542	0.996	2	4	detection of molecule of bacterial origin	BP	1	TLR1,NOD2	up_ACOR_VSMC
GO:0035136	0.0542	0.994	2	16	forelimb morphogenesis	BP	1	HOXA9,RECK	up_AFEM_EC
GO:0034142	0.0542	0.999	1	14	toll-like receptor 4 signaling pathway	BP	1	NR1H4	up_APUL_EC
GO:1901862	0.0542	0.999	1	14	negative regulation of muscle tissue	BP	1	TBX5	up_APUL_EC
GO:0090331	0.054214401	0.99925	1	2	negative regulation of platelet aggregation	BP	1	PRKCD	up_ACOR_EC
GO:1903532	0.05421703	0.98351	5	78	positive regulation of secretion by cell	BP	1	NR1H4,FZD5,NOD2,PTGER4,MAVS	up_ACOR_EC
GO:0001951	0.054225243	0.99925	1	2	intestinal D-glucose absorption	BP	1	PLS1	up_ACOR_EC
GO:0032532	0.054225243	0.99925	1	2	regulation of microvillus length	BP	1	PLS1	up_ACOR_EC

GO:0106001	0.054225243	0.99925	1	2	intestinal hexose absorption	BP	1	PLS1	up_ACOR_EC
GO:1902896	0.054225243	0.99925	1	2	terminal web assembly	BP	1	PLS1	up_ACOR_EC
GO:2000826	0.054269018	0.99419	2	14	regulation of heart morphogenesis	BP	1	FOXC2,TBX5	up_ACOR_EC
GO:0008219	0.0543	0.962	46	611	cell death	BP	0.874	ARHGEF2,VEGFA,P4HB,ASNS,NR1H4,PLK3,HSPA5,SERPINE1,ETV6,ERO1A,CASP3,HYOU1,EXOC5,AARS,FHL2,PTGS2,UACA,HIF1A,DNAJC10,TNIK,ZPR1,PTPN2,MTDH,MAGED1,CTNNB1,CRYAB,APOE,LYN,BNIP3,DDIT3,FAM162A,ATF3,GSK3B,SH3GLB1,HTT,REST,EIF2S1,GLO1,PAK2,INHBA,SIX4,CDKN1A,NRP1,SPINK2,ARF4,MADD	down_VSAP_EC
GO:0006873	0.0543	0.968	22	160	cellular ion homeostasis	BP	1	ADRA1B,PLCG2,SLC26A7,HTR2B,MICU2,ITPR1,F2RL1,EGLN1,DMPK,ATP7A,PRKCE,APP,PSEN1,CHD7,DMXL1,EDN1,DISC1,HERPUD1,WFS1,ATP2C1,SLC8A1,STC2	up_ACOR_VSMC
GO:0051341	0.0543	0.985	5	22	regulation of oxidoreductase activity	BP	1	LEP,HTR2B,ATP7A,EDN1,PARK7	up_ACOR_VSMC
GO:0032653	0.0543	0.991	3	11	regulation of interleukin-10 production	BP	0.884	CD34,TLR4,HMGB1	up_VCAV_EC
GO:0023052	0.0543	1	2	1602	signaling	BP	1	JUP,GATA5	down_VFEM_VSMC
GO:0030900	0.0543	0.994	2	101	forebrain development	BP	1	SLITRK5,NTRK2	up_APUL_EC
GO:0043277	0.0543	0.999	1	9	apoptotic cell clearance	BP	1	SPG11	down_ACOR_EC
GO:1903707	0.0543	0.999	1	48	negative regulation of hemopoiesis	BP	1	HOXA9	up_AORT_VSMC
GO:0019229	0.0543	0.999	1	12	regulation of vasoconstriction	BP	1	CAV1	up_VSAP_VSMC
GO:0060717	0.054366379	0.99924	1	2	chorion development	BP	1	FZD5	up_ACOR_EC
GO:0060718	0.054366379	0.99924	1	2	chorionic trophoblast cell differentiation	BP	1	FZD5	up_ACOR_EC
GO:0008360	0.0544	0.982	6	46	regulation of cell shape	BP	0.874	VEGFA,MYH9,FMNL3,AKAP2,WDR1,MSN	down_VSAP_EC
GO:0046888	0.0544	0.99	3	14	negative regulation of hormone secretion	BP	0.874	PTPN11,REST,RAB11FIP1	down_VSAP_EC
GO:0060339	0.0544	0.995	2	5	negative regulation of type I interferon-mediated signaling	BP	0.884	NLRC5,CNOT7	up_VCAV_EC
GO:1903312	0.0544	0.999	1	25	negative regulation of mRNA metabolic process	BP	1	SRSF6	down_AFEM_EC
GO:0051098	0.0544	0.999	1	129	regulation of binding	BP	1	PLCL1	down_VFEM_EC
GO:0006749	0.0544	0.999	1	14	glutathione metabolic process	BP	1	ENSCAFG00000028814	up_APUL_EC
GO:0051926	0.0544	0.999	1	14	negative regulation of calcium ion transport	BP	1	HOXA3	up_APUL_EC
GO:0048638	0.0544	0.999	1	82	regulation of developmental growth	BP	1	TBX5	up_APUL_VSMC
GO:0003104	0.0544	1	1	1	positive regulation of glomerular filtration	BP	0.884	PDGFB	up_VCAV_EC
GO:0010046	0.0544	1	1	1	response to mycotoxin	BP	0.884	PDGFB	up_VCAV_EC
GO:0035655	0.0544	1	1	1	interleukin-18-mediated signaling pathway	BP	0.884	PDGFB	up_VCAV_EC
GO:0035787	0.0544	1	1	1	cell migration involved in kidney development	BP	0.884	PDGFB	up_VCAV_EC
GO:0035789	0.0544	1	1	1	metanephric mesenchymal cell	BP	0.884	PDGFB	up_VCAV_EC
GO:0035793	0.0544	1	1	1	positive regulation of metanephric mesenchymal cell migration by platelet-derived growth factor	BP	0.884	PDGFB	up_VCAV_EC
GO:0036146	0.0544	1	1	1	cellular response to mycotoxin	BP	0.884	PDGFB	up_VCAV_EC
GO:0038001	0.0544	1	1	1	paracrine signaling	BP	0.884	PDGFB	up_VCAV_EC
GO:0043696	0.0544	1	1	1	dedifferentiation	BP	0.884	PDGFB	up_VCAV_EC
GO:0043697	0.0544	1	1	1	cell dedifferentiation	BP	0.884	PDGFB	up_VCAV_EC
GO:0071351	0.0544	1	1	1	cellular response to interleukin-18	BP	0.884	PDGFB	up_VCAV_EC
GO:0071505	0.0544	1	1	1	response to mycophenolic acid	BP	0.884	PDGFB	up_VCAV_EC

GO:0071506	0.0544	1	1	1	cellular response to mycophenolic acid	BP	0.884	PDGFB	up_VCAV_EC
GO:0072126	0.0544	1	1	1	positive regulation of glomerular mesangial cell	BP	0.884	PDGFB	up_VCAV_EC
GO:0072209	0.0544	1	1	1	metanephric mesangial cell differentiation	BP	0.884	PDGFB	up_VCAV_EC
GO:0072223	0.0544	1	1	1	metanephric glomerular mesangium development	BP	0.884	PDGFB	up_VCAV_EC
GO:0072254	0.0544	1	1	1	metanephric glomerular mesangial cell	BP	0.884	PDGFB	up_VCAV_EC
GO:0072255	0.0544	1	1	1	metanephric glomerular mesangial cell	BP	0.884	PDGFB	up_VCAV_EC
GO:0090193	0.0544	1	1	1	positive regulation of glomerulus development	BP	0.884	PDGFB	up_VCAV_EC
GO:0090677	0.0544	1	1	1	reversible differentiation	BP	0.884	PDGFB	up_VCAV_EC
GO:0090678	0.0544	1	1	1	cell dedifferentiation involved in phenotypic	BP	0.884	PDGFB	up_VCAV_EC
GO:1900127	0.0544	1	1	1	positive regulation of hyaluronan biosynthetic	BP	0.884	PDGFB	up_VCAV_EC
GO:1900238	0.0544	1	1	1	regulation of metanephric mesenchymal cell migration by platelet-derived growth factor	BP	0.884	PDGFB	up_VCAV_EC
GO:1900241	0.0544	1	1	1	positive regulation of phenotypic switching	BP	0.884	PDGFB	up_VCAV_EC
GO:1901724	0.0544	1	1	1	positive regulation of cell proliferation involved in kidney development	BP	0.884	PDGFB	up_VCAV_EC
GO:1905174	0.0544	1	1	1	regulation of vascular smooth muscle cell	BP	0.884	PDGFB	up_VCAV_EC
GO:1905176	0.0544	1	1	1	positive regulation of vascular smooth muscle cell dedifferentiation	BP	0.884	PDGFB	up_VCAV_EC
GO:1990936	0.0544	1	1	1	vascular smooth muscle cell dedifferentiation	BP	0.884	PDGFB	up_VCAV_EC
GO:2000589	0.0544	1	1	1	regulation of metanephric mesenchymal cell	BP	0.884	PDGFB	up_VCAV_EC
GO:2000591	0.0544	1	1	1	positive regulation of metanephric mesenchymal cell	BP	0.884	PDGFB	up_VCAV_EC
GO:0010512	0.0544	1	1	1	negative regulation of phosphatidylinositol biosynthetic process	BP	0.884	PDGFB	up_VCAV_EC
GO:0035788	0.0544	1	1	1	cell migration involved in metanephros	BP	0.884	PDGFB	up_VCAV_EC
GO:0043271	0.0544	0.999	1	28	negative regulation of ion transport	BP	1	HOXA3	up_VPUL_VSMC
GO:0002692	0.054417727	0.99924	1	2	negative regulation of cellular extravasation	BP	1	PTGER4	up_ACOR_EC
GO:1900063	0.054464932	0.99924	1	2	regulation of peroxisome organization	BP	1	MAVS	up_ACOR_EC
GO:0015846	0.054491371	0.99924	1	2	polyamine transport	BP	1	AZIN2	up_ACOR_EC
GO:1902047	0.054491371	0.99924	1	2	polyamine transmembrane transport	BP	1	AZIN2	up_ACOR_EC
GO:1902267	0.054491371	0.99924	1	2	regulation of polyamine transmembrane transport	BP	1	AZIN2	up_ACOR_EC
GO:1902269	0.054491371	0.99924	1	2	positive regulation of polyamine	BP	1	AZIN2	up_ACOR_EC
GO:0071732	0.054494275	0.99924	1	2	cellular response to nitric oxide	BP	1	GUCY1B1	up_ACOR_EC
GO:0051338	0.0545	0.986	4	288	regulation of transferase activity	BP	1	LYN,IGF1R,PRKAR2A,ASPN	down_ACOR_EC
GO:0048589	0.0545	0.994	2	188	developmental growth	BP	1	TBX5,SLIT2	up_VPUL_VSMC
GO:0006885	0.0545	0.999	1	39	regulation of pH	BP	1	CA2	up_VCAV_VSMC
GO:0043408	0.054518488	0.97518	10	211	regulation of MAPK cascade	BP	1	NDRG2,FZD5,DUSP26,NOD2,PAK3,TNFRSF19,MAPK8IP2,SH3RF3,NRP1,PRKCD	up_ACOR_EC
GO:0019953	0.0546	0.969	19	152	sexual reproduction	BP	1	NDC1,TRIP13,CTDNEP1,FBXO5,AAAS,NCAPH,POC1A,B4GALT1,HIST1H1A,FOSL1,HMGB2,SGPL1,TTK,ASPM,HOXA9,GLI1,H2AFX,CCT4,TTLL1	down_ACOR_VSMC
GO:0007166	0.0546	0.98	7	686	cell surface receptor signaling pathway	BP	1	ACVRL1,LYN,NRP2,SULF1,WDR12,STX4,ASPN	down_ACOR_EC
GO:0002088	0.0546	0.984	5	26	lens development in camera-type eye	BP	0.884	GATA3,TMOD1,PROX1,SKIL,CDON	up_VCAV_EC
GO:0014904	0.0546	0.991	3	12	myotube cell development	BP	0.884	PPP3CA,CAV2,P2RX2	up_VCAV_EC

GO:0048741	0.0546	0.991	3	12	skeletal muscle fiber development	BP	0.884	PPP3CA,CAV2,P2RX2	up_VCAV_EC
GO:0032530	0.0546	0.995	2	6	regulation of microvillus organization	BP	0.874	FSCN1,KLF5	down_VSAP_EC
GO:0042775	0.0546	0.999	1	28	mitochondrial ATP synthesis coupled	BP	1	NDUFA10	down_AFEM_EC
GO:1902750	0.0546	0.999	1	24	negative regulation of cell cycle G2/M phase	BP	1	TAOK1	up_AORT_EC
GO:2000378	0.0546	0.999	1	12	negative regulation of reactive oxygen species	BP	1	CAV1	up_VSAP_VSMC
GO:2000739	0.05460252	0.99924	1	2	regulation of mesenchymal stem cell	BP	1	SOX9	up_ACOR_EC
GO:0090176	0.054609228	0.99924	1	2	microtubule cytoskeleton organization involved in establishment of planar	BP	1	BRSK1	up_ACOR_EC
GO:0050680	0.0547	0.98	7	44	negative regulation of epithelial cell proliferation	BP	0.884	MEF2C,DLL4,GATA3,MTSS1,CAV2,MCC,VASH1	up_VCAV_EC
GO:0001932	0.0547	0.984	5	416	regulation of protein phosphorylation	BP	1	ACVRL1,LYN,IGF1R,PRKAR2A,ASPN	down_ACOR_EC
GO:0043933	0.0547	0.988	4	698	protein-containing complex subunit	BP	1	EIF3M,SRSF6,NDUFA10,FSCN1	down_AFEM_EC
GO:0035050	0.0547	0.999	1	28	embryonic heart tube development	BP	1	CITED2	down_VPOR_EC
GO:0006551	0.0547	1	1	1	leucine metabolic process	BP	0.874	BCAT1	down_VSAP_EC
GO:0009098	0.0547	1	1	1	leucine biosynthetic process	BP	0.874	BCAT1	down_VSAP_EC
GO:0009099	0.0547	1	1	1	valine biosynthetic process	BP	0.874	BCAT1	down_VSAP_EC
GO:0009082	0.0547	1	1	1	branched-chain amino acid biosynthetic process	BP	0.874	BCAT1	down_VSAP_EC
GO:0070663	0.0547	0.999	1	50	regulation of leukocyte proliferation	BP	1	ARG2	up_AORT_VSMC
GO:0021602	0.0547	0.999	1	16	cranial nerve morphogenesis	BP	1	HOXA3	up_VPUL_EC
GO:0061476	0.054762266	0.99923	1	2	response to anticoagulant	BP	1	SOX9	up_ACOR_EC
GO:0071503	0.054762266	0.99923	1	2	response to heparin	BP	1	SOX9	up_ACOR_EC
GO:0071504	0.054762266	0.99923	1	2	cellular response to heparin	BP	1	SOX9	up_ACOR_EC
GO:0030595	0.0548	0.978	8	57	leukocyte chemotaxis	BP	0.884	EDNRB,KIT,CXCL8,PDGFB,PDE4D,MMP28,SWAP70,HMGB1	up_VCAV_EC
GO:0035023	0.0548	0.978	8	46	regulation of Rho protein signal transduction	BP	1	ARHGEF5,ARHGEF6,ABL1,F2RL1,TIAM1,DLC1,FGD4,FLOT1	up_VSAP_EC
GO:0051239	0.0548	0.985	5	859	regulation of multicellular organismal process	BP	1	NKX2-5,BAMBI,ANKRD6,CYP26B1,C3AR1	up_VPOR_VSMC
GO:0060113	0.0548	0.99	3	18	inner ear receptor cell differentiation	BP	1	SDC4,NAGLU,GSDME	down_VCAV_EC
GO:0071310	0.0548	0.991	3	634	cellular response to organic substance	BP	1	CYP1A1,GATA5,CA2	up_VCAV_VSMC
GO:2000479	0.0548	0.999	1	8	regulation of cAMP-dependent protein kinase	BP	1	PRKAR2A	down_ACOR_EC
GO:0048817	0.054816115	0.99923	1	2	negative regulation of hair follicle maturation	BP	1	CDH3	up_ACOR_EC
GO:0035977	0.054880787	0.99923	1	2	protein deglycosylation involved in glycoprotein	BP	1	EDEM2	up_ACOR_EC
GO:0036507	0.054880787	0.99923	1	2	protein demannosylation	BP	1	EDEM2	up_ACOR_EC
GO:0036508	0.054880787	0.99923	1	2	protein alpha-1,2-demannosylation	BP	1	EDEM2	up_ACOR_EC
GO:0097466	0.054880787	0.99923	1	2	ubiquitin-dependent glycoprotein ERAD	BP	1	EDEM2	up_ACOR_EC
GO:1904382	0.054880787	0.99923	1	2	mannose trimming involved in glycoprotein	BP	1	EDEM2	up_ACOR_EC
GO:1904587	0.054880787	0.99923	1	2	response to glycoprotein	BP	1	EDEM2	up_ACOR_EC
GO:0071635	0.054881257	0.99923	1	2	negative regulation of transforming growth factor beta production	BP	1	CDH3	up_ACOR_EC
GO:0042221	0.054892142	0.96379	35	1012	response to chemical	BP	1	NR1H4,GATA4,SOX9,FNDC4,AQP1,ENSCAFG00000004331,KCNB1,EDEM2,FZD5,CCDC3,AGRN,PTGIR,NOD2,RGMA,PDE4A,SMAD6,ACTN2,PAK3,MMP28,GUCY1B1,PTGER4,ELMO2,ADAMTSL2,MAVS,FOXC2,LRRC15,IFIT2,LRP4,LRRC4,CLU,RARRES2,PRKACA,NRP1,HM13,PRKCD	up_ACOR_EC

GO:0042325	0.0549	0.964	31	463	regulation of phosphorylation	BP	1	NOTCH2,PDGFRB,KIRREL1,DCN,PRNP,GLIPR2,BGN,BMPR1A,LMO4,CLIP3,ENSCAFG00000014978,AKT1S1,AKT2,EEF2K,PLCL1,CSPG4,ASPN,THY1,PARK7,MYDGF,SASH1,ARL2BP,MAVS,CDH2,GSDME,SPRED1,MAPRE3,DDR2,SHC1,FBH1,GREM1	down_VCAV_EC
GO:0002526	0.0549	0.987	4	17	acute inflammatory response	BP	0.884	PRCP,F8,IL6,PTGS2	up_VCAV_EC
GO:0043066	0.0549	0.99	3	253	negative regulation of apoptotic process	BP	1	NR1H4,PTGER2,ARHGEF2	up_VPUL_EC
GO:0071803	0.0549	0.995	2	6	positive regulation of podosome assembly	BP	0.874	FSCN1,MSN	down_VSAP_EC
GO:1904645	0.0549	0.999	1	7	response to amyloid-beta	BP	1	IGF1R	down_ACOR_EC
GO:0007268	0.0549	0.999	1	130	chemical synaptic transmission	BP	1	PLCL1	down_VFEM_EC
GO:0098916	0.0549	0.999	1	130	anterograde trans-synaptic signaling	BP	1	PLCL1	down_VFEM_EC
GO:0038110	0.0549	1	1	1	interleukin-2-mediated signaling pathway	BP	0.874	PTPN2	down_VSAP_EC
GO:0060331	0.0549	1	1	1	negative regulation of response to interferon-	BP	0.874	PTPN2	down_VSAP_EC
GO:0060336	0.0549	1	1	1	negative regulation of interferon-gamma-mediated signaling	BP	0.874	PTPN2	down_VSAP_EC
GO:0070103	0.0549	1	1	1	regulation of interleukin-6-mediated signaling	BP	0.874	PTPN2	down_VSAP_EC
GO:0070104	0.0549	1	1	1	negative regulation of interleukin-6-mediated	BP	0.874	PTPN2	down_VSAP_EC
GO:0070669	0.0549	1	1	1	response to interleukin-2	BP	0.874	PTPN2	down_VSAP_EC
GO:0071352	0.0549	1	1	1	cellular response to interleukin-2	BP	0.874	PTPN2	down_VSAP_EC
GO:1902205	0.0549	1	1	1	regulation of interleukin-2-mediated signaling	BP	0.874	PTPN2	down_VSAP_EC
GO:1902206	0.0549	1	1	1	negative regulation of interleukin-2-mediated	BP	0.874	PTPN2	down_VSAP_EC
GO:1902214	0.0549	1	1	1	regulation of interleukin-4-mediated signaling	BP	0.874	PTPN2	down_VSAP_EC
GO:1902215	0.0549	1	1	1	negative regulation of interleukin-4-mediated	BP	0.874	PTPN2	down_VSAP_EC
GO:1902232	0.0549	1	1	1	regulation of positive thymic T cell selection	BP	0.874	PTPN2	down_VSAP_EC
GO:1902233	0.0549	1	1	1	negative regulation of positive thymic T cell	BP	0.874	PTPN2	down_VSAP_EC
GO:0010633	0.0549	0.999	1	28	negative regulation of epithelial cell migration	BP	1	SLIT2	up_VPUL_VSMC
GO:0060669	0.0549	0.999	1	12	embryonic placenta morphogenesis	BP	1	GJB5	up_VSAP_VSMC
GO:0031348	0.055	0.981	6	46	negative regulation of defense response	BP	0.874	NR1H4,SOCS5,PTPN2,CNOT7,APOE,A2M	down_VSAP_EC
GO:0090175	0.055	0.987	4	15	regulation of establishment of planar	BP	1	MLLT3,SEC24B,ZNRF3,FZD3	up_ACOR_VSMC
GO:0051457	0.055	0.995	2	8	maintenance of protein location in nucleus	BP	1	PARK7,ARL2BP	down_VCAV_EC
GO:0042219	0.055	0.996	2	4	cellular modified amino acid catabolic process	BP	1	SARDH,PCYOX1	up_ACOR_VSMC
GO:0015991	0.055	0.994	2	16	ATP hydrolysis coupled proton transport	BP	1	ATP6V1B2,ATP6V0B	up_AFEM_EC
GO:0090662	0.055	0.994	2	16	ATP hydrolysis coupled transmembrane transport	BP	1	ATP6V1B2,ATP6V0B	up_AFEM_EC
GO:0099131	0.055	0.994	2	16	ATP hydrolysis coupled ion transmembrane	BP	1	ATP6V1B2,ATP6V0B	up_AFEM_EC
GO:0060546	0.055	0.999	1	7	negative regulation of necroptotic process	BP	1	ARHGEF2	up_VPOR_EC
GO:0060547	0.055	0.999	1	7	negative regulation of necrotic cell death	BP	1	ARHGEF2	up_VPOR_EC
GO:1903727	0.055	0.999	1	16	positive regulation of phospholipid metabolic	BP	1	NR1H4	up_VPUL_EC
GO:0060481	0.055005143	0.99923	1	2	lobar bronchus epithelium development	BP	1	ADAMTSL2	up_ACOR_EC
GO:0060482	0.055005143	0.99923	1	2	lobar bronchus development	BP	1	ADAMTSL2	up_ACOR_EC
GO:0045333	0.0551	0.974	12	74	cellular respiration	BP	1	DLST,COQ9,NDUFS1,ENSCAFG00000004260,SLC25A12,FXN,NDUFS2,MTFR1L,IDH1,IDH3A,ENSCAFG00000006639,NDUFV1	up_VSAP_EC
GO:0010557	0.0551	0.984	5	492	positive regulation of macromolecule biosynthetic process	BP	1	HOXA11,HOXA9,SALL1,PRR16,SHOX2	up_VSAP_VSMC

GO:0070848	0.0551	0.999	1	193	response to growth factor	BP	1	GATA5	down_VFEM_VSMC
GO:0006517	0.0551	0.999	1	7	protein deglycosylation	BP	1	OGA	up_VPOR_EC
GO:0003143	0.0551	0.999	1	22	embryonic heart tube morphogenesis	BP	1	NKX2-5	up_VPOR_VSMC
GO:0071621	0.0551	0.999	1	27	granulocyte chemotaxis	BP	1	C3AR1	up_VPOR_VSMC
GO:0030104	0.0551	0.999	1	13	water homeostasis	BP	1	CYP26B1	up_VFEM_EC
GO:0070266	0.0551	0.999	1	14	necroptotic process	BP	1	CAV1	up_VSAP_VSMC
GO:1901890	0.0551	0.999	1	13	positive regulation of cell junction assembly	BP	1	CAV1	up_VSAP_VSMC
GO:0009117	0.0552	0.966	24	208	nucleotide metabolic process	BP	1	PRPSAP1,ACLY,HMGCR,NNT,RRM1,RAN,CDK1,PFAS,RNASEH2B,MVD,ELOVL1,CYCS,ELOVL6,CYC1,ATP5F1A,DCK,PRPS1,ENSCAFG00000013366,PDHA1,NUDT1,PGD,CTPS1,ATP5F1D,NUDT3	down_ACOR_VSMC
GO:0071219	0.0552	0.978	8	50	cellular response to molecule of bacterial	BP	0.884	CXCL8,TNFAIP3,TNIP1,PDE4D,MEF2C,TLR4,IRF8,ABCA1	up_VCAV_EC
GO:0050793	0.0552	0.982	6	731	regulation of developmental process	BP	1	NR1H4,TBX5,SLITRK5,WIF1,NPNT,NTRK2	up_APUL_EC
GO:0002709	0.0552	0.987	4	19	regulation of T cell mediated immunity	BP	0.884	IL7R,GATA3,NECTIN2,HMGB1	up_VCAV_EC
GO:0034109	0.0552	0.99	3	14	homotypic cell-cell adhesion	BP	0.874	LYN,SLC7A11,ITGB3	down_VSAP_EC
GO:0003094	0.0552	0.999	1	11	glomerular filtration	BP	1	SULF1	down_ACOR_EC
GO:0002042	0.0552	0.999	1	29	cell migration involved in sprouting angiogenesis	BP	1	HDAC7	down_VPOR_EC
GO:0002062	0.0552	0.999	1	48	chondrocyte differentiation	BP	1	WNT7A	up_AORT_VSMC
GO:0070542	0.0552	0.999	1	15	response to fatty acid	BP	1	NR1H4	up_APUL_EC
GO:1900120	0.0552	0.999	1	7	regulation of receptor binding	BP	1	HOXA3	up_VPOR_EC
GO:0046660	0.0552	0.999	1	28	female sex differentiation	BP	1	SLIT2	up_VPUL_VSMC
GO:1900452	0.055222993	0.99922	1	2	regulation of long-term synaptic depression	BP	1	KCNB1	up_ACOR_EC
GO:0007166	0.0553	0.967	23	686	cell surface receptor signaling pathway	BP	1	LEP,GAS1,ANGPT4,DLL1,F3,PLXNA4,MLLT11,HCK,TMEM100,GREM1,FAM53B,GREM2,CYLD,POSTN,LRRC15,RECK,LGR6,RAB7A,AXIN2,CCL5,DMPK,ADAM17,KANK1	up_AFEM_EC
GO:1901699	0.0553	0.967	21	164	cellular response to nitrogen compound	BP	0.884	PPARG,BAIAP2L1,TMEM173,PDE4D,MEF2C,RAPGEF1,RALB,MTOR,MAPK13,RIOK3,NCK1,TIMELESS,GUCY1B1,RAPGEF2,CTNNA1,BLM,PDGFD,ATRX,NR4A1,ZEB1,ARHGEF2	up_VCAV_EC
GO:0045216	0.0553	0.981	6	44	cell-cell junction organization	BP	0.874	NR1H4,FSCN1,WDR1,MYO1C,CTNNB1,ENSCAFG00000002015	down_VSAP_EC
GO:0098773	0.0553	0.982	6	29	skin epidermis development	BP	1	FGF7,DLL1,SAV1,HPSE,LRP4,TRADD	up_VSAP_EC
GO:0046889	0.0553	0.987	4	24	positive regulation of lipid biosynthetic process	BP	0.874	NR1H4,PTGS2,SLC45A3,APOE	down_VSAP_EC
GO:0070542	0.0553	0.987	4	15	response to fatty acid	BP	1	PRKAA2,ADCY6,PTGDR,PRKCE	up_ACOR_VSMC
GO:0010720	0.0553	0.989	3	110	positive regulation of cell development	BP	1	ARHGEF2,ENSCAFG00000002015,SEMA3A	up_VPOR_EC
GO:0002347	0.0553	0.995	2	6	response to tumor cell	BP	0.884	NECTIN2,HMGB1	up_VCAV_EC
GO:0099537	0.0553	0.999	1	131	trans-synaptic signaling	BP	1	PLCL1	down_VFEM_EC
GO:0042742	0.0553	0.999	1	39	defense response to bacterium	BP	1	TLR2	up_AFEM_VSMC
GO:0055123	0.0553	0.999	1	40	digestive system development	BP	1	GATA5	up_VCAV_VSMC
GO:0045880	0.0553	0.999	1	14	positive regulation of smoothened signaling	BP	1	SHOX2	up_VSAP_VSMC
GO:1902305	0.0553	0.999	1	12	regulation of sodium ion transmembrane transport	BP	1	NETO1	up_VSAP_VSMC
GO:0150018	0.055377795	0.99922	1	2	basal dendrite development	BP	1	NRP1	up_ACOR_EC
GO:0150019	0.055377795	0.99922	1	2	basal dendrite morphogenesis	BP	1	NRP1	up_ACOR_EC
GO:0150020	0.055377795	0.99922	1	2	basal dendrite arborization	BP	1	NRP1	up_ACOR_EC
GO:1901606	0.0554	0.984	5	22	alpha-amino acid catabolic process	BP	1	GOT1,ADHFE1,ALDH6A1,ENSCAFG00000002977,IDO2	up_VSAP_EC

GO:0001709	0.0554	0.991	3	11	cell fate determination	BP	0.884	MEF2C,GATA3,PROX1	up_VCAV_EC
GO:1904666	0.0554	0.995	2	5	regulation of ubiquitin protein ligase activity	BP	1	UBE2S,CDC20	down_ACOR_VSMC
GO:0048247	0.0554	0.994	2	16	lymphocyte chemotaxis	BP	1	CCL5,ADAM17	up_AFEM_EC
GO:0051051	0.0554	0.994	2	112	negative regulation of transport	BP	1	HOXA3,NR1H4	up_VPUL_EC
GO:0048485	0.0554	0.999	1	9	sympathetic nervous system development	BP	1	NRP2	down_ACOR_EC
GO:1901983	0.0554	0.999	1	24	regulation of protein acetylation	BP	1	TAOK1	up_AORT_EC
GO:0046622	0.0554	0.999	1	15	positive regulation of organ growth	BP	1	TBX5	up_APUL_EC
GO:0061010	0.0554	1	1	1	gall bladder development	BP	0.884	HHEX	up_VCAV_EC
GO:0061011	0.0554	1	1	1	hepatic duct development	BP	0.884	HHEX	up_VCAV_EC
GO:0061017	0.0554	1	1	1	hepatoblast differentiation	BP	0.884	HHEX	up_VCAV_EC
GO:0021545	0.0554	0.999	1	21	cranial nerve development	BP	1	HOXA3	up_VPOR_VSMC
GO:0021510	0.0554	0.999	1	23	spinal cord development	BP	1	HOXC10	up_VFEM_VSMC
GO:0045834	0.0555	0.98	7	41	positive regulation of lipid metabolic process	BP	0.884	TEK,ADGRF5,PDGFB,MTOR,SH3GLB1,PTGS2,CREB1	up_VCAV_EC
GO:0050804	0.0555	0.983	5	87	modulation of chemical synaptic transmission	BP	1	KCNC4,CHRM2,SLC6A6,CAMK2A,DMPK	up_AFEM_EC
GO:0099177	0.0555	0.983	5	87	regulation of trans-synaptic signaling	BP	1	KCNC4,CHRM2,SLC6A6,CAMK2A,DMPK	up_AFEM_EC
GO:0070613	0.0555	0.986	4	25	regulation of protein processing	BP	0.874	MYH9,SERPINE1,PRKACB,A2M	down_VSAP_EC
GO:1903317	0.0555	0.986	4	25	regulation of protein maturation	BP	0.874	MYH9,SERPINE1,PRKACB,A2M	down_VSAP_EC
GO:0050767	0.0555	0.986	4	184	regulation of neurogenesis	BP	1	HOOK3,ARHGEF2,SEMA3A,RNF6	up_VPOR_EC
GO:0061213	0.0555	0.991	3	11	positive regulation of mesonephros	BP	1	GREM1,HOXB7,SIX4	down_ACOR_VSMC
GO:0032507	0.0555	0.99	3	18	maintenance of protein location in cell	BP	1	TLN2,PARK7,ARL2BP	down_VCAV_EC
GO:0018196	0.0555	0.99	3	15	peptidyl-asparagine modification	BP	0.874	STT3A,HIF1AN,RPN1	down_VSAP_EC
GO:0016048	0.0555	0.995	2	5	detection of temperature stimulus	BP	0.884	EPHB1,MMP24	up_VCAV_EC
GO:0050951	0.0555	0.995	2	5	sensory perception of temperature stimulus	BP	0.884	EPHB1,MMP24	up_VCAV_EC
GO:0050961	0.0555	0.995	2	5	detection of temperature stimulus involved in sensory perception	BP	0.884	EPHB1,MMP24	up_VCAV_EC
GO:0050965	0.0555	0.995	2	5	detection of temperature stimulus involved in sensory perception of	BP	0.884	EPHB1,MMP24	up_VCAV_EC
GO:0060840	0.0555	0.999	1	42	artery development	BP	1	EYA1	up_AFEM_VSMC
GO:1901164	0.0555	1	1	1	negative regulation of trophoblast cell migration	BP	0.884	ARHGDIB	up_VCAV_EC
GO:0032535	0.055515491	0.97882	7	128	regulation of cellular component size	BP	1	TMOD1,AQP1,PLS1,ACTN2,PAK3,NRP1,PRKCD	up_ACOR_EC
GO:1903909	0.05559869	0.99921	1	2	regulation of receptor clustering	BP	1	LRP4	up_ACOR_EC
GO:1904393	0.05559869	0.99921	1	2	regulation of skeletal muscle acetylcholine-gated channel clustering	BP	1	LRP4	up_ACOR_EC
GO:0048545	0.0556	0.974	11	68	response to steroid hormone	BP	1	FAM107A,PPARGC1B,SLIT2,GOT1,ZFP36L1,CREBRF,PRMT2,ZFP36L2,NCOA2,LBH,DDIT4	up_VSAP_EC
GO:0051046	0.0556	0.977	8	174	regulation of secretion	BP	1	LEP,KCNC4,CRY2,GPAM,CHRM2,CAMK2A,RAB7A,CCL5	up_AFEM_EC
GO:0034622	0.0556	0.99	3	421	cellular protein-containing complex assembly	BP	1	EIF3M,SRSF6,NDUFA10	down_AFEM_EC
GO:0007154	0.0556	1	2	1622	cell communication	BP	1	JUP,GATA5	down_VFEM_VSMC
GO:0001759	0.0556	0.999	1	13	organ induction	BP	1	SIX1	up_VFEM_EC
GO:0050830	0.0556	0.999	1	12	defense response to Gram-positive bacterium	BP	1	TLR2	up_VSAP_VSMC
GO:0072202	0.0556	0.999	1	13	cell differentiation involved in metanephros	BP	1	SALL1	up_VSAP_VSMC
GO:0021649	0.05561755	0.99921	1	2	vestibulocochlear nerve structural organization	BP	1	NRP1	up_ACOR_EC

GO:0061552	0.05561755	0.99921	1	2	ganglion morphogenesis	BP	1	NRP1	up_ACOR_EC
GO:0097374	0.05561755	0.99921	1	2	sensory neuron axon guidance	BP	1	NRP1	up_ACOR_EC
GO:1904835	0.05561755	0.99921	1	2	dorsal root ganglion morphogenesis	BP	1	NRP1	up_ACOR_EC
GO:0042144	0.055624625	0.99921	1	2	vacuole fusion, non-autophagic	BP	1	VPS16	up_ACOR_EC
GO:0097576	0.055624625	0.99921	1	2	vacuole fusion	BP	1	VPS16	up_ACOR_EC
GO:0007409	0.0557	0.994	2	109	axonogenesis	BP	1	FLRT3,NEO1	up_VPUL_EC
GO:0006929	0.0557	0.999	1	10	substrate-dependent cell migration	BP	1	NRP2	down_ACOR_EC
GO:0055011	0.0557	1	1	1	atrial cardiac muscle cell differentiation	BP	0.874	FHL2	down_VSAP_EC
GO:0055014	0.0557	1	1	1	atrial cardiac muscle cell development	BP	0.874	FHL2	down_VSAP_EC
GO:0010959	0.0557	0.999	1	79	regulation of metal ion transport	BP	1	HOXA3	up_APUL_VSMC
GO:0002548	0.0557	0.999	1	17	monocyte chemotaxis	BP	1	CCL24	up_VPUL_EC
GO:0050772	0.0557	0.999	1	13	positive regulation of axonogenesis	BP	1	SHOX2	up_VSAP_VSMC
GO:1901018	0.055713201	0.99921	1	2	positive regulation of potassium ion transmembrane	BP	1	ACTN2	up_ACOR_EC
GO:0060978	0.055728307	0.99921	1	2	angiogenesis involved in coronary vascular	BP	1	NRP1	up_ACOR_EC
GO:0048699	0.0558	0.971	14	371	generation of neurons	BP	1	IRX3,IRX5,HOXC10,LEP,DLL1,VXN,PLXNA4,NEFH,THRB,LGI4,CAMK2A,LRRN2,KANK1,TNC	up_AFEM_EC
GO:0051017	0.0558	0.978	8	49	actin filament bundle assembly	BP	0.886	ARHGEF15,BAIAP2L1,ARHGAP28,MTOR,STMN1,SWAP70,TNFAIP1,FSCN1	up_VCAV_EC
GO:0006811	0.0558	0.986	4	330	ion transport	BP	1	NETO1,SLC16A12,STIM1,CAV1	up_VSAP_VSMC
GO:0035295	0.0558	0.99	3	356	tube development	BP	1	EYA1,HOXA11,SHOX2	up_VFEM_VSMC
GO:0003157	0.0558	0.995	2	5	endocardium development	BP	0.886	SOX18,PROX1	up_VCAV_EC
GO:0030003	0.0558	0.994	2	157	cellular cation homeostasis	BP	1	TRPC4,C3AR1	up_VPOR_VSMC
GO:0001678	0.0558	0.999	1	29	cellular glucose homeostasis	BP	1	RAB11B	down_VPOR_EC
GO:0006879	0.0558	0.999	1	23	cellular iron ion homeostasis	BP	1	ENSCAFG00000005494	up_AORT_EC
GO:0007097	0.0558	0.999	1	7	nuclear migration	BP	1	HOOK3	up_VPOR_EC
GO:0051647	0.0558	0.999	1	7	nucleus localization	BP	1	HOOK3	up_VPOR_EC
GO:0090259	0.055848505	0.9992	1	2	regulation of retinal ganglion cell axon	BP	1	NRP1	up_ACOR_EC
GO:0009653	0.0559	0.96	48	773	anatomical structure morphogenesis	BP	1	NOTCH2,HSPB6,PDGFRB,DCN,NAB2,NDN,ANGPT1,SDC4,FBN1,THBS3,BMPR1A,LOX,LMO4,RECK,TRPV2,ANPEP,COL6A1,NUMBL,NAGLU,TBX15,HOXC4,LAMA3,CLSTN1,SEMA3C,ASPN,POLDIP2,CRISPLD2,THY1,TLX2,B4GALT1,GLI3,ZFP36L1,MYDGF,SASH1,IFT57,CDH2,ENSCAFG00000017708,SPRED1,IRX5,UST,SHC1,RDH10,GREM1,CLIC4,FOXO3,THRB,ZBTB16,BRSK1	down_VCAV_EC
GO:2000113	0.0559	0.961	42	387	negative regulation of cellular macromolecule biosynthetic process	BP	1	ELK3,HMGB1,SREBF1,ILF3,CBX5,ATAD2,HMGA1,EIF4A3,SREBF2,SMC1A,NEDD4,MTDH,NAB2,BIRC5,CAV1,TIMELESS,FAM129B,AURKB,EZH2,GREM1,PLK3,HOXA7,UHRF1,GMNN,NOC2L,PPARD,NCL,SUV39H1,HMGB2,DMAP1,SMC3,CDK2,ENSCAFG00000005299,EZR,RBM14,ERF,TCERG1,HAT1,HIST2H2AC,TINF2,H2AFX,SIX4	down_ACOR_VSMC
GO:0090257	0.0559	0.978	8	52	regulation of muscle system process	BP	0.886	DOCK4,PDE4D,STC1,EDN1,PPP3CA,GUCY1A1,KCNJ2,ATP2A1	up_VCAV_EC
GO:0060048	0.0559	0.982	6	30	cardiac muscle contraction	BP	1	TNNT2,ADRA1B,KCNE3,PDE5A,JUP,GAA	up_ACOR_VSMC
GO:0060402	0.0559	0.983	5	35	calcium ion transport into cytosol	BP	0.874	ERO1A,LYN,DDIT3,HTT,CALM1	down_VSAP_EC
GO:0003171	0.0559	0.991	3	10	atrioventricular valve development	BP	1	SMAD6,TBX20,TBX5	up_ACOR_VSMC
GO:0071559	0.0559	0.994	2	75	response to transforming growth factor beta	BP	1	ACVRL1,ASPN	down_ACOR_EC

GO:0042531	0.0559	0.999	1	13	positive regulation of tyrosine phosphorylation	BP	1	LYN	down_ACOR_EC
GO:0007507	0.0559	0.999	1	194	heart development	BP	1	GATA5	down_VFEM_VSMC
GO:0043985	0.0559	1	1	1	histone H4-R3 methylation	BP	0.874	PRMT1	down_VSAP_EC
GO:0048593	0.0559	0.999	1	41	camera-type eye morphogenesis	BP	1	IRX5	up_AFEM_VSMC
GO:0031110	0.0559	0.999	1	24	regulation of microtubule polymerization or depolymerization	BP	1	TAOK1	up_AORT_EC
GO:0050795	0.0559	0.999	1	15	regulation of behavior	BP	1	HOXA1	up_APUL_EC
GO:0010977	0.055942237	0.98927	3	33	negative regulation of neuron projection	BP	1	RGMA,LRP4,NRP1	up_ACOR_EC
GO:0040017	0.056	0.986	4	180	positive regulation of locomotion	BP	1	C3AR1,ARHGEF2,SEMA3A,CCAR1	up_VPOR_EC
GO:0060713	0.056	0.991	3	10	labyrinthine layer morphogenesis	BP	1	FGFR2,SPINT1,SPINT2	up_ACOR_VSMC
GO:0048646	0.056	0.99	3	349	anatomical structure formation involved in	BP	1	HOXA3,NKX2-5,C3AR1	up_VPOR_VSMC
GO:0051094	0.056	1	1	388	positive regulation of developmental process	BP	1	GATA5	down_AFEM_VSMC
GO:0010896	0.056	1	1	1	regulation of triglyceride catabolic process	BP	0.874	ABHD5	down_VSAP_EC
GO:0010898	0.056	1	1	1	positive regulation of triglyceride catabolic	BP	0.874	ABHD5	down_VSAP_EC
GO:0001704	0.056	0.999	1	43	formation of primary germ layer	BP	1	EYA1	up_AFEM_VSMC
GO:0061326	0.056	0.999	1	41	renal tubule development	BP	1	EYA1	up_AFEM_VSMC
GO:0072080	0.056	0.999	1	41	nephron tubule development	BP	1	EYA1	up_AFEM_VSMC
GO:0007196	0.056	1	1	1	adenylate cyclase-inhibiting G protein-coupled glutamate	BP	0.886	GRM8	up_VCAV_EC
GO:0007216	0.056	1	1	1	G protein-coupled glutamate receptor	BP	0.886	GRM8	up_VCAV_EC
GO:0048731	0.0561	0.955	137	1247	system development	BP	1	EXPH5,TNNT2,SH3TC2,PDLIM3,FGF13,VEGFD,TMEM176B,TMEM176A,SMAD6,ACTN2,PLCG2,LEP,LBH,GATA5,GATA4,CD83,ANGPT1,EPYC,HTR2B,RASSF2,MBP,PRDX4,RECK,SHROOM3,FEZ1,MLLT3,ITM2C,FERMT1,BMP4,LNPK,TBX18,FGFR2,TP53BP1,GJA1,ATRX,TLR3,SEC24B,XIRP1,WASF3,PPARGC1A,LATS2,CITED2,SPINT1,GLRB,ISG15,ACSL6,ZNRF3,CD274,ZNF365,SASH1,LMO4,TMEM108,FGF18,FAM210B,NDRG2,ATRN,P4HTM,NEBL,CDKN2B,LIMS2,ANKRD1,F2RL1,CAMK2D,CTSB,EGLN1,ENSCAFG00000006238,CLSTN1,NRP1,PTPN22,GPM6A,CHD2,ULK2,NPNT,UNC5B,SMARCD3,SPINT2,SLITRK5,TBX20,BBX,TBX5,FZD3,FRS2,CPAMD8,RND1,DAG1,POMK,APP,PSEN1,SEMA6D,TGFBR1,SYNJ2BP,CHD7,JUP,SPINK2,SIPA1L1,DDR2,CLN8,FOSL2,PERP,PSKH1,RTKN2,SSH3,EDN1,NID1,HPGD,ARID5B,ASAP1,DMC1,SERPINB5,JAM3,B2M,DISC1,AP2B1,TNFRSF11B,TACC1,GAA,ATXN10,FEZ2,SUN1,CNOT4,LHX9,WFS1,WDR48,WASL,ACP2,ERAP1,ID3,SLC8A1,TEAD1,ENSCAFG00000003530,IL18R1,BVES,RREB1,ZDHHC21,CRKL,PIK3C2A,PTPN14	up_ACOR_VSMC
GO:0042221	0.0561	0.977	9	1012	response to chemical	BP	1	ACVRL1,LYN,IGF1R,PDPN,NRP2,SULF1,STX4,ASPN,NEDD4	down_ACOR_EC
GO:0046483	0.0561	0.992	4	1948	heterocycle metabolic process	BP	1	AK5,GATA5,TGFBR1,MEIS2	down_VSAP_VSMC
GO:0001960	0.0561	0.99	3	15	negative regulation of cytokine-mediated	BP	0.874	NR1H4,PTPN2,CNOT7	down_VSAP_EC
GO:0060761	0.0561	0.99	3	15	negative regulation of response to cytokine	BP	0.874	NR1H4,PTPN2,CNOT7	down_VSAP_EC
GO:0050919	0.0561	0.991	3	10	negative chemotaxis	BP	1	SLIT2,PLXNA4,SEMA5A	up_VSAP_EC
GO:0070848	0.0561	0.994	2	193	response to growth factor	BP	1	GATA5,SLIT2	up_VPUL_VSMC
GO:0099536	0.0561	0.999	1	133	synaptic signaling	BP	1	PLCL1	down_VFEM_EC
GO:0050902	0.0561	1	1	1	leukocyte adhesive activation	BP	0.874	CX3CL1	down_VSAP_EC
GO:0002690	0.0561	0.999	1	26	positive regulation of leukocyte chemotaxis	BP	1	C3AR1	up_VPOR_VSMC

GO:0002520	0.0562	0.965	24	279	immune system development	BP	0.874	VEGFA,MYH9,ACTN1,SOCS5,ETV6,CASP3,PTPN11,WDR1,HOXB7,HIF1A,PTPN2,EVI2B,CTNNB1,ANLN,TFRC,LYN,MYO1E,REST,PRMT1,SH2B3,GLO1,INHBA,BTK,SIX4	down_VSAP_EC
GO:0032269	0.0562	0.986	4	306	negative regulation of cellular protein metabolic	BP	1	LYN,IGF1R,PRKAR2A,ASPN	down_ACOR_EC
GO:0002682	0.0562	0.986	4	343	regulation of immune system process	BP	1	HOXA9,TOX,HOXA7,CYP26B1	up_VFEM_EC
GO:0030225	0.0562	0.99	3	12	macrophage differentiation	BP	0.889	HCLS1,CASP8,RIPK1	up_VCAV_EC
GO:0031641	0.0562	0.995	2	6	regulation of myelination	BP	0.874	ZPR1,CTNNB1	down_VSAP_EC
GO:2000008	0.0562	0.999	1	12	regulation of protein localization to cell surface	BP	1	STX4	down_ACOR_EC
GO:0048844	0.0562	0.999	1	30	artery morphogenesis	BP	1	CITED2	down_VPOR_EC
GO:0032722	0.0562	0.999	1	14	positive regulation of chemokine production	BP	1	TLR2	up_VSAP_VSMC
GO:0050794	0.0563	0.954	202	3075	regulation of cellular process	BP	0.874	ARHGEF2,PRICKLE1,VEGFA,P4HB,SIPA1L1,SULF1,ARHGAP31,ASNS,MYH9,NR1H4,FMNL3,PLK3,ENSCAFG00000013214,HSPA5,SERPINE1,ACTN1,SOCS5,ETV6,SPARC,MEIS2,SGK1,ERO1A,PRKACB,PTPN14,ENPP1,ERRFI1,ITGA2,JCAD,SNX25,CBX5,CASP3,FAM49B,ENSCAFG00000003477,HYOU1,AKAP2,WSB1,BCAR3,NUAK1,PTPN11,FSCN1,AARS,DPYSL3,LRRC8A,HOOK3,TRIB3,PGAM1,IFRD1,FARP1,PUM3,PM20D2,FBLIM1,ILK,FHL2,BHLHE40,PPP1R12A,PRCP,SMARCA4,ANKRA2,SESN2,TGFBR1,PTGS2,REM1,WDR1,MYO1C,PIK3C2A,CHSY1,MSN,UACA,KIF5B,LMAN1,HIF1A,CX3CL1,ENSCAFG00000000562,DYNLL1,SLC45A3,TUBB,IWS1,DNAJC10,YIPF5,TNIK,NAA10,NXN,RGS2,ZPR1,PTPN2,UCHL5,MORF4L2,SPOPL,EIF2B1,EVI2B,YES1,NEO1,PLCL1,PEG3,SMG7,MTDH,ESM1,DDX39B,ADAM17,AGRN,BRD2,EIF4EBP1,SKAP2,UST,PDIA4,CNOT7,CDH11,ACTR3,ADGRE5,MAGED1,GNAQ,CTNNB1,KPNB1,ANLN,CRYAB,STARD13,SND1,ENSCAFG00000002015,TFRC,CDK8,APOE,PTP4A1,WWC3,PDE1A,ZC3H15,IGFBP7,KLF5,UBA5,MMP28,LYN,BNIP3,STRAP,DDIT3,TWF1,PDS5B,FAM162A,BACH1,CCNYL1,PLEKHG2,TLN1,EIF5,MMD,CBLC,ATF3,GSK3B,VLDLR,NEXN,CDC37,GTF2H1,ARMCX3,IT	down_VSAP_EC
GO:0040017	0.0563	0.966	24	180	positive regulation of locomotion	BP	1	TWIST2,WNT11,FAM107A,ENPP2,SLIT2,FGF7,ABL1,HDAC7,PDGFD,CCBE1,STAT5A,F2RL1,DDR2,PIK3R1,TIAM1,CFAP69,SASH1,PC,ZNF580,STX4,HMOX1,MAP2K2,TGFBR2,BAG4	up_VSAP_EC
GO:0001655	0.0563	0.98	6	116	urogenital system development	BP	1	IRX3,DLL1,GREM1,OVOL1,GCNT4,TNC	up_AFEM_EC
GO:0042092	0.0563	0.99	3	11	type 2 immune response	BP	0.889	BCL6B,GATA3,ARG2	up_VCAV_EC
GO:0051092	0.0563	0.993	2	47	positive regulation of NF-kappaB transcription	BP	1	ARHGEF2,MTDH	up_VPOR_EC
GO:0007568	0.0563	0.999	1	50	aging	BP	1	ARG2	up_AORT_VSMC
GO:0010646	0.0564	0.964	30	945	regulation of cell communication	BP	1	CMKLR1,LEP,KCNC4,LBH,CRY2,GAS1,DLL1,F3,MLLT11,LMCD1,CHRM2,TMEM100,SLC6A6,PLAGL2,GREM1,FAM53B,CAMK2A,GREM2,CLEC16A,CYLD,POSTN,LRRC15,RECK,LGR6,RAB7A,AXIN2,CCL5,DMPK,ADAM17,KANK1	up_AFEM_EC
GO:0042493	0.0564	0.966	24	178	response to drug	BP	1	GATA4,HTR2B,PRKAA2,ATRX,CASTOR1,TP53INP1,ADCY6,ANKRD1,EGLN1,P2RX7,PTPN22,TCN2,PRKCE,AGRN,PSEN1,JUP,CLN8,NR3C1,EDN1,NOD2,DDIT4,GNAQ,CAT,PARK7	up_ACOR_VSMC
GO:0098542	0.0564	0.97	15	100	defense response to other organism	BP	1	MAP3K14,IL17RC,F2RL1,BATF2,PLA2G6,MYD88,NLRX1,TKFC,RNF216,MAPKBP1,CYBA,PQBP1,MAVS,JCHAIN,DDIT4	up_VSAP_EC
GO:0031589	0.0564	0.972	13	93	cell-substrate adhesion	BP	0.889	EPHB1,TEK,ADAMTS9,VWF,CD34,FERMT1,NEDD9,SLK,ANGPT2,APOD,JAG1,ITGA6,ENSCAFG00000002015	up_VCAV_EC
GO:0050663	0.0564	0.976	9	52	cytokine secretion	BP	1	CD274,C5AR2,ABL1,IL17RC,ANXA4,F2RL1,PNP,MAPKBP1,MAVS	up_VSAP_EC
GO:0050707	0.0564	0.978	8	44	regulation of cytokine secretion	BP	1	CD274,C5AR2,ABL1,IL17RC,ANXA4,F2RL1,MAPKBP1,MAVS	up_VSAP_EC
GO:0007530	0.0564	0.995	2	5	sex determination	BP	1	SF1,SIX4	down_ACOR_VSMC

GO:0033578	0.0564	0.996	2	4	protein glycosylation in Golgi	BP	1	FUT8,LMF1	up_ACOR_VSMC
GO:0048864	0.0564	0.999	1	29	stem cell development	BP	1	CITED2	down_VPOR_EC
GO:0021885	0.0564	0.999	1	24	forebrain cell migration	BP	1	EMX2	up_VFEM_VSMC
GO:0045844	0.0564	0.999	1	24	positive regulation of striated muscle tissue	BP	1	SHOX2	up_VFEM_VSMC
GO:0048636	0.0564	0.999	1	24	positive regulation of muscle organ	BP	1	SHOX2	up_VFEM_VSMC
GO:0044057	0.0565	0.971	14	110	regulation of system process	BP	0.889	DOCK4,PDGFB,RELN,PDE4D,STC1,EDN1,HOMER3,PPP3CA,GUCY1A1,ACPP,KCNJ2,GCH1,SPTBN4,ATP2A1	up_VCAV_EC
GO:0045944	0.0565	0.994	2	287	positive regulation of transcription by RNA polymerase II	BP	1	EYA1,HOXA9	up_AFEM_VSMC
GO:0072109	0.0565	0.995	2	6	glomerular mesangium development	BP	0.889	PDGFB,GPR4	up_VCAV_EC
GO:0035914	0.0565	0.999	1	22	skeletal muscle cell differentiation	BP	1	CYP26B1	up_VPOR_VSMC
GO:0035904	0.0565	0.999	1	25	aorta development	BP	1	EYA1	up_VFEM_VSMC
GO:0009142	0.0566	0.993	2	46	nucleoside triphosphate biosynthetic process	BP	1	AK5,FBP1	up_VPOR_EC
GO:0071346	0.0566	0.999	1	41	cellular response to interferon-gamma	BP	1	TLR2	up_AFEM_VSMC
GO:0044106	0.0566	0.999	1	17	cellular amine metabolic process	BP	1	NR1H4	up_VPUL_EC
GO:0002763	0.0566	0.999	1	17	positive regulation of myeloid leukocyte	BP	1	EVI2B	up_VPUL_EC
GO:0009893	0.0567	0.978	8	995	positive regulation of metabolic process	BP	1	HOXA11,HOXA9,GATM,SALL1,PRR16,TLR2,CAV1,SHOX2	up_VSAP_VSMC
GO:0030835	0.0567	0.99	3	11	negative regulation of actin filament	BP	0.891	TMOD1,SWAP70,TMOD3	up_VCAV_EC
GO:0010603	0.0567	0.996	2	4	regulation of cytoplasmic mRNA processing body	BP	0.891	CNOT6,PAN3	up_VCAV_EC
GO:0007283	0.0567	0.993	2	91	spermatogenesis	BP	1	HOXA9,CYP26B1	up_VFEM_EC
GO:0006679	0.0567	1	1	1	glucosylceramide biosynthetic process	BP	0.874	UGCG	down_VSAP_EC
GO:0099003	0.0567	0.999	1	50	vesicle-mediated transport in synapse	BP	1	WNT7A	up_AORT_VSMC
GO:0001759	0.0567	0.999	1	13	organ induction	BP	1	HOXA11	up_VSAP_VSMC
GO:0070371	0.0568	0.972	12	91	ERK1 and ERK2 cascade	BP	0.891	EPHB1,TEK,TNIP1,PDGFB,ATF3,RAPGEF1,TLR4,BMP2,PRKD2,RAPGEF2,HMGB1,PDGFD	up_VCAV_EC
GO:0048522	0.0568	0.982	7	1484	positive regulation of cellular process	BP	1	FGF5,HOXA3,NKX2-5,BAMBI,ANKRD6,CYP26B1,C3AR1	up_VPOR_VSMC
GO:0043244	0.0568	0.983	5	34	regulation of protein complex disassembly	BP	0.874	ARHGEF2,WDR1,BNIP3,CAPG,MID1	down_VSAP_EC
GO:0016477	0.0568	0.986	4	426	cell migration	BP	1	TBX5,CCL24,FLRT3,ARHGEF2	up_VPUL_EC
GO:0046637	0.0568	0.99	3	18	regulation of alpha-beta T cell differentiation	BP	1	GLI3,LOXL3,ZBTB16	down_VCAV_EC
GO:0071322	0.0568	0.999	1	29	cellular response to carbohydrate stimulus	BP	1	RAB11B	down_VPOR_EC
GO:0032057	0.0568	1	1	1	negative regulation of translational initiation in response to stress	BP	0.874	EIF2S1	down_VSAP_EC
GO:0010243	0.056898105	0.97282	11	238	response to organonitrogen compound	BP	1	NR1H4,SOX9,AQP1,ENSCAFG00000004331,EDEM2,NOD2,ACTN2,FOXC2,RARRES2,HM13,PRKCD	up_ACOR_EC
GO:0071900	0.0569	0.971	13	131	regulation of protein serine/threonine kinase activity	BP	0.874	VEGFA,PTPN11,TRIB3,SESN2,TNIK,MAGED1,APOE,LYN,CCNYL1,CBLC,HTT,CDKN1A,MADD	down_VSAP_EC
GO:0002040	0.0569	0.981	6	46	sprouting angiogenesis	BP	0.874	VEGFA,JCAD,PIK3C2A,ESM1,STARD13,NRP1	down_VSAP_EC
GO:0032481	0.0569	0.984	5	24	positive regulation of type I interferon production	BP	0.892	TMEM173,TLR4,RIOK3,TLR3,PLCG2	up_VCAV_EC
GO:0006725	0.0569	0.992	4	1956	cellular aromatic compound metabolic	BP	1	AK5,GATA5,TGFBR1,MEIS2	down_VSAP_VSMC
GO:0047496	0.0569	0.99	3	15	vesicle transport along microtubule	BP	0.874	KIF5B,HTT,AP3S1	down_VSAP_EC
GO:0007507	0.0569	0.994	2	194	heart development	BP	1	TBX5,GATA5	up_VPUL_VSMC
GO:0001960	0.0569	0.999	1	15	negative regulation of cytokine-mediated	BP	1	NR1H4	up_APUL_EC
GO:0060761	0.0569	0.999	1	15	negative regulation of response to cytokine	BP	1	NR1H4	up_APUL_EC

GO:0030878	0.0569	0.999	1	13	thyroid gland development	BP	1	SIX1	up_VFEM_EC
GO:1903578	0.0569	0.999	1	22	regulation of ATP metabolic process	BP	1	FBP1	up_VFEM_VSMC
GO:0010225	0.057	0.995	2	5	response to UV-C	BP	1	POLH,HMGN1	down_ACOR_VSMC
GO:0050877	0.057	0.994	2	164	nervous system process	BP	1	HOXC10,GPR155	up_AORT_EC
GO:0022617	0.057	0.999	1	11	extracellular matrix disassembly	BP	1	PDPN	down_ACOR_EC
GO:0021884	0.057	0.999	1	10	forebrain neuron development	BP	1	NRP2	down_ACOR_EC
GO:0060384	0.057	0.999	1	9	innervation	BP	1	SULF1	down_ACOR_EC
GO:0120034	0.057	0.999	1	28	positive regulation of plasma membrane bounded cell projection	BP	1	FSCN1	down_AFEM_EC
GO:0001300	0.057	1	1	1	chronological cell aging	BP	0.874	SERPINE1	down_VSAP_EC
GO:0002538	0.057	1	1	1	arachidonic acid metabolite production involved in inflammatory	BP	0.874	SERPINE1	down_VSAP_EC
GO:0002540	0.057	1	1	1	leukotriene production involved in inflammatory	BP	0.874	SERPINE1	down_VSAP_EC
GO:0035490	0.057	1	1	1	regulation of leukotriene production involved in inflammatory response	BP	0.874	SERPINE1	down_VSAP_EC
GO:0035491	0.057	1	1	1	positive regulation of leukotriene production involved in inflammatory	BP	0.874	SERPINE1	down_VSAP_EC
GO:0061044	0.057	1	1	1	negative regulation of vascular wound healing	BP	0.874	SERPINE1	down_VSAP_EC
GO:0097187	0.057	1	1	1	dentinogenesis	BP	0.874	SERPINE1	down_VSAP_EC
GO:1901329	0.057	1	1	1	regulation of odontoblast differentiation	BP	0.874	SERPINE1	down_VSAP_EC
GO:1901331	0.057	1	1	1	positive regulation of odontoblast	BP	0.874	SERPINE1	down_VSAP_EC
GO:1902913	0.057	1	1	1	positive regulation of neuroepithelial cell differentiation	BP	0.874	SERPINE1	down_VSAP_EC
GO:0007492	0.057	0.998	1	27	endoderm development	BP	1	DUSP2	up_AORT_EC
GO:0055024	0.057	0.999	1	22	regulation of cardiac muscle tissue	BP	1	NKX2-5	up_VPOR_VSMC
GO:0002683	0.0571	0.968	18	126	negative regulation of immune system process	BP	1	HOXA7,HOXA9,CD274,C5AR2,TBC1D10C,SLIT2,DLL1,TRAFD1,ZBTB16,STAT6,F2RL1,ZFP36L1,PIK3R1,MYC,FBXO7,NLRX1,TKFC,PPP3CB	up_VSAP_EC
GO:0019221	0.0571	0.971	13	102	cytokine-mediated signaling pathway	BP	0.893	ACKR1,KIT,CXCL8,CXCR4,PDGFB,NLRC5,IFNGR1,OTUD4,CNOT7,ADIPOR2,LSM14A,ENSCAFG00000002015,TJP2	up_VCAV_EC
GO:0099601	0.0571	0.99	3	10	regulation of neurotransmitter	BP	0.893	RELN,MEF2C,HOMER3	up_VCAV_EC
GO:0048729	0.0571	0.989	3	209	tissue morphogenesis	BP	1	HOXA11,SALL1,SHOX2	up_VSAP_VSMC
GO:0045595	0.0571	0.995	2	469	regulation of cell differentiation	BP	1	GATA5,TGFBR1	down_VSAP_VSMC
GO:0030162	0.0571	0.999	1	202	regulation of proteolysis	BP	1	CST3	down_AORT_EC
GO:0002042	0.0571	0.999	1	29	cell migration involved in sprouting angiogenesis	BP	1	SLIT2	up_VPUL_VSMC
GO:0008542	0.0571	0.999	1	13	visual learning	BP	1	NETO1	up_VSAP_VSMC
GO:0010459	0.057113736	0.99916	1	2	negative regulation of heart rate	BP	1	RNLS	up_ACOR_EC
GO:0044332	0.057121713	0.99916	1	2	Wnt signaling pathway involved in dorsal/ventral	BP	1	LRP4	up_ACOR_EC
GO:0009966	0.0572	0.984	5	868	regulation of signal transduction	BP	1	BARX1,NKX2-5,BAMBI,ANKRD6,CYP26B1	up_VPOR_VSMC
GO:0072521	0.0572	0.994	2	185	purine-containing compound metabolic	BP	1	SUCLG2,NDUFA10	down_AFEM_EC
GO:0098876	0.0572	0.999	1	29	vesicle-mediated transport to the plasma	BP	1	RAB11B	down_VPOR_EC
GO:0019563	0.0572	1	1	1	glycerol catabolic process	BP	0.874	TPI1	down_VSAP_EC
GO:1905207	0.0572	0.998	1	17	regulation of cardiocyte differentiation	BP	1	TBX5	up_VPUL_EC
GO:0048663	0.0572	0.999	1	13	neuron fate commitment	BP	1	HOXC10	up_VSAP_VSMC

GO:0032872	0.057265821	0.98232	5	77	regulation of stress-activated MAPK cascade	BP	1	FZD5,NOD2,TNFRSF19,MAPK8IP2,SH3RF3	up_ACOR_EC
GO:0002262	0.0573	0.977	8	54	myeloid cell homeostasis	BP	0.895	HCLS1,ADGRF5,KIT,GATA3,ITPKB,HMGB1,HIF1A,TMOD3	up_VCAV_EC
GO:0065009	0.0573	0.981	6	735	regulation of molecular function	BP	1	NR1H4,HOXA3,PLCL1,PCSK1N,NPNT,NTRK2	up_APUL_EC
GO:0051155	0.0573	0.986	4	18	positive regulation of striated muscle cell	BP	0.895	MEF2C,EDN1,MTOR,PROX1	up_VCAV_EC
GO:0048762	0.0573	0.993	2	77	mesenchymal cell differentiation	BP	1	PDPN,NRP2	down_ACOR_EC
GO:0043268	0.0573	0.996	2	4	positive regulation of potassium ion transport	BP	1	ACTN2,AKAP6	up_ACOR_VSMC
GO:0003007	0.0573	0.993	2	94	heart morphogenesis	BP	1	EYA1,SIX1	up_VFEM_EC
GO:0021783	0.0573	0.999	1	9	preganglionic parasympathetic fiber	BP	1	NRP2	down_ACOR_EC
GO:0071675	0.0573	0.998	1	13	regulation of mononuclear cell	BP	1	LYN	down_ACOR_EC
GO:0055021	0.0573	0.998	1	15	regulation of cardiac muscle tissue growth	BP	1	TBX5	up_APUL_EC
GO:0050810	0.0573	0.998	1	17	regulation of steroid biosynthetic process	BP	1	NR1H4	up_VPUL_EC
GO:0051965	0.0573	0.998	1	17	positive regulation of synapse assembly	BP	1	FLRT3	up_VPUL_EC
GO:0060042	0.0573	0.998	1	14	retina morphogenesis in camera-type eye	BP	1	IRX5	up_VSAP_VSMC
GO:0044267	0.057302888	0.95982	51	1583	cellular protein metabolic process	BP	1	NR1H4,BRSK1,PLCL1,PKIB,SOX9,GALNT5,PCSK1N,PHF20,NDRG2,PORCN,ENSCAFG00000004331,EDEM2,PI3,FZD5,IFI6,DUSP26,NOD2,SMAD6,B3GNT7,KLHL25,ZMYND8,PAK3,GRK7,TNFRSF19,CST3,PIAS1,DCLK2,USP53,MAPK8IP2,MAVS,LRRC15,KLHDC8B,PIGS,LRP4,SH3RF3,LRRC4,CLU,CHMP4B,TP53INP2,RARRES2,AGBL5,RIOX2,RNF114,UBE2H,STK32C,NRP1,HM13,PRKCD,PYGO2,DBNDD2,CCNYL1	up_ACOR_EC
GO:0009790	0.0574	0.961	37	335	embryo development	BP	1	ACSL4,EIF4A3,HINFP,HOXA3,GINS4,INSIG1,PRMT1,XYLT1,NF2,MMP14,SLC30A1,GREM1,MAP3K20,NEK2,RNASEH2B,HOXA7,HOXC10,HOXB7,BRCA1,E2F8,UGDH,FOSL1,PLK4,HOXC6,SMARCB1,HOXB5,RBBP8,HOXC4,NOLC1,PHACTR4,TBX15,ITGA2,HOXA9,GLI1,EMG1,RTCB,SIX4	down_ACOR_VSMC
GO:0001654	0.0574	0.969	16	110	eye development	BP	1	THRB,CDON,OSR2,MAB21L1,IRX5,DLL1,RARG,MFN2,ZEB1,CTNS,CLCN2,SMG9,KDM2B,ACVRL1,TGFBR2,CITED2	up_VSAP_EC
GO:0048880	0.0574	0.969	16	110	sensory system development	BP	1	THRB,CDON,OSR2,MAB21L1,IRX5,DLL1,RARG,MFN2,ZEB1,CTNS,CLCN2,SMG9,KDM2B,ACVRL1,TGFBR2,CITED2	up_VSAP_EC
GO:0150063	0.0574	0.969	16	110	visual system development	BP	1	THRB,CDON,OSR2,MAB21L1,IRX5,DLL1,RARG,MFN2,ZEB1,CTNS,CLCN2,SMG9,KDM2B,ACVRL1,TGFBR2,CITED2	up_VSAP_EC
GO:0030282	0.0574	0.989	3	37	bone mineralization	BP	1	GREM1,PTHLH,AXIN2	up_AFEM_EC
GO:1904706	0.0574	0.994	2	7	negative regulation of vascular smooth muscle	BP	0.874	ENSCAFG00000017326,CDKN1A	down_VSAP_EC
GO:0031349	0.0574	0.993	2	90	positive regulation of defense response	BP	1	TLR2,CAV1	up_VSAP_VSMC
GO:0045682	0.0574	0.998	1	27	regulation of epidermis development	BP	1	SRSF6	down_AFEM_EC
GO:0009719	0.0574	1	1	395	response to endogenous stimulus	BP	1	GATA5	down_AFEM_VSMC
GO:0009566	0.0574	0.998	1	24	fertilization	BP	1	HOXA9	up_AORT_EC
GO:0046326	0.0574	0.998	1	15	positive regulation of glucose import	BP	1	NR1H4	up_APUL_EC
GO:0006940	0.0574	0.998	1	14	regulation of smooth muscle contraction	BP	1	CAV1	up_VSAP_VSMC
GO:0043009	0.0575	0.964	26	227	chordate embryonic development	BP	0.896	HOXB7,PCDH12,ADGRF5,TIE1,AMOT,PDGFB,MEF2C,CHD7,GATA3,EDN1,HOXB8,SOX18,BMP2,HHEX,MDFI,HOXB5,BCL10,NUP133,SKIL,CUL4A,HIF1A,LUZP1,RBBP6,VASH1,ZEB1,GINS4	up_VCAV_EC
GO:0051270	0.0575	0.964	25	296	regulation of cellular component movement	BP	0.874	ARHGEF2,VEGFA,SULF1,HSPA5,SERPINE1,ACTN1,SPARC,JCAD,DPYSL3,PRCP,MYO1C,PIK3C2A,MSN,HIF1A,ADAM17,STARD13,APOE,PTP4A1,MMP28,LYN,STRAP,NEXN,SDC4,ZMYND8,NRP1	down_VSAP_EC
GO:0008284	0.0575	0.974	10	238	positive regulation of cell proliferation	BP	1	LEP,LBH,DLL1,F3,GPAM,MAB21L1,GREM1,ADAM17,FOSL2,TNC	up_AFEM_EC

GO:2000112	0.0575	0.992	3	1119	regulation of cellular macromolecule biosynthetic process	BP	1	GATA5,TGFBR1,MEIS2	down_VSAP_VSMC
GO:0001503	0.0575	0.989	3	109	ossification	BP	1	HOXA3,ASPN,ACVR2A	up_VPOR_EC
GO:0051962	0.0575	0.993	2	112	positive regulation of nervous system	BP	1	FLRT3,ARHGEF2	up_VPUL_EC
GO:0044346	0.0575	0.999	1	7	fibroblast apoptotic process	BP	1	STK17B	up_VPOR_EC
GO:2000269	0.0575	0.999	1	7	regulation of fibroblast apoptotic process	BP	1	STK17B	up_VPOR_EC
GO:0005976	0.0576	0.983	5	34	polysaccharide metabolic process	BP	0.874	ENPP1,ENSCAFG00000032470,PPP1R3C,GSK3B,EXT1	down_VSAP_EC
GO:0035295	0.0576	0.989	3	356	tube development	BP	1	HOXA3,NKX2-5,C3AR1	up_VPOR_VSMC
GO:0021522	0.0576	0.994	2	8	spinal cord motor neuron differentiation	BP	1	LMO4,GLI3	down_VCAV_EC
GO:0055025	0.0576	0.998	1	17	positive regulation of cardiac muscle tissue	BP	1	TBX5	up_VPUL_EC
GO:0019076	0.057664246	0.99356	2	15	viral release from host cell	BP	1	TRIM8,CHMP4B	up_ACOR_EC
GO:0035890	0.057664246	0.99356	2	15	exit from host	BP	1	TRIM8,CHMP4B	up_ACOR_EC
GO:0035891	0.057664246	0.99356	2	15	exit from host cell	BP	1	TRIM8,CHMP4B	up_ACOR_EC
GO:0052126	0.057664246	0.99356	2	15	movement in host environment	BP	1	TRIM8,CHMP4B	up_ACOR_EC
GO:0052192	0.057664246	0.99356	2	15	movement in environment of other organism involved in	BP	1	TRIM8,CHMP4B	up_ACOR_EC
GO:0001763	0.057671931	0.98215	5	78	morphogenesis of a branching structure	BP	1	SOX9,FZD5,FOXC2,CLSTN1,NRP1	up_ACOR_EC
GO:0006941	0.0577	0.981	6	36	striated muscle contraction	BP	0.896	PDE4D,STC1,ARG2,MTOR,KCNJ2,ATP2A1	up_VCAV_EC
GO:0016045	0.0577	0.996	2	4	detection of bacterium	BP	1	TLR1,NOD2	up_ACOR_VSMC
GO:0098543	0.0577	0.996	2	4	detection of other organism	BP	1	TLR1,NOD2	up_ACOR_VSMC
GO:0021773	0.0577	1	1	1	striatal medium spiny neuron differentiation	BP	0.874	INHBA	down_VSAP_EC
GO:0032276	0.0577	1	1	1	regulation of gonadotropin secretion	BP	0.874	INHBA	down_VSAP_EC
GO:0046884	0.0577	1	1	1	follicle-stimulating hormone secretion	BP	0.874	INHBA	down_VSAP_EC
GO:0060278	0.0577	1	1	1	regulation of ovulation	BP	0.874	INHBA	down_VSAP_EC
GO:0060279	0.0577	1	1	1	positive regulation of ovulation	BP	0.874	INHBA	down_VSAP_EC
GO:0046880	0.0577	1	1	1	regulation of follicle-stimulating hormone	BP	0.874	INHBA	down_VSAP_EC
GO:0060993	0.0577	0.999	1	42	kidney morphogenesis	BP	1	EYA1	up_AFEM_VSMC
GO:0035773	0.0577	0.998	1	15	insulin secretion involved in cellular response to glucose stimulus	BP	1	NR1H4	up_APUL_EC
GO:0046718	0.0577	0.998	1	13	viral entry into host cell	BP	1	CAV1	up_VSAP_VSMC
GO:0030154	0.0578	0.98	7	1040	cell differentiation	BP	1	TBX5,EVI2B,MYBPC2,WIF1,FLRT3,ARHGEF2,NEO1	up_VPUL_EC
GO:0030512	0.0578	0.986	4	24	negative regulation of transforming growth factor beta receptor	BP	0.874	HSPA5,SNX25,ADAM17,STRAP	down_VSAP_EC
GO:1903845	0.0578	0.986	4	24	negative regulation of cellular response to transforming growth	BP	0.874	HSPA5,SNX25,ADAM17,STRAP	down_VSAP_EC
GO:0006509	0.0578	0.99	3	15	membrane protein ectodomain proteolysis	BP	0.874	MYH9,ADAM17,APOE	down_VSAP_EC
GO:0002263	0.0578	0.993	2	68	cell activation involved in immune response	BP	1	LYN,STX4	down_ACOR_EC
GO:0050878	0.0578	0.993	2	75	regulation of body fluid levels	BP	1	LYN,PDPN	down_ACOR_EC
GO:0098542	0.0578	0.993	2	100	defense response to other organism	BP	1	NR1H4,BPI	up_APUL_EC
GO:0046470	0.0578	0.998	1	13	phosphatidylcholine metabolic process	BP	1	ACSL3	down_ACOR_EC
GO:0002218	0.0578	0.999	1	53	activation of innate immune response	BP	1	ESR1	up_AORT_VSMC
GO:0090050	0.057804376	0.99354	2	15	positive regulation of cell migration involved in sprouting angiogenesis	BP	1	FOXC2,NRP1	up_ACOR_EC

GO:0001959	0.05782273	0.98876	3	33	regulation of cytokine-mediated signaling	BP	1	NR1H4,CCDC3,MAVS	up_ACOR_EC
GO:0002682	0.0579	0.96	42	343	regulation of immune system process	BP	1	HOXA7,HOXA9,CD274,C5AR2,SLC8B1,PPARGC1B,TBC1D10C,SLIT2,ABL1,DLL1,TRAFD1,ANKRD54,ZBTB16,TOX,RARG,STAT6,F2RL1,ZFP36L1,PIK3R1,RBCK1,MYC,ZEB1,MYD88,TFE3,CFD,FBXO7,NLRX1,TKFC,CD83,RNF216,ZFP36L2,PNP,ZNF580,CYBA,STX4,PQBP1,UNC93B1,FLOT1,PPP3CB,NCK1,MAVS,TGFBR2	up_VSAP_EC
GO:0060341	0.0579	0.965	22	255	regulation of cellular localization	BP	0.874	NR1H4,PLK3,PTPN14,PTPN11,PPP1R12A,SESN2,MYO1C,MSN,KIF5B,HIF1A,IWS1,YIPF5,ZPR1,CTNNB1,CRYAB,LYN,GSK3B,SH3GLB1,TAX1BP3,HTT,CALM1,CDKN1A	down_VSAP_EC
GO:0061515	0.0579	0.983	5	24	myeloid cell development	BP	0.896	ADGRF5,KIT,FLI1,TMOD3,TJP2	up_VCAV_EC
GO:0002287	0.0579	0.986	4	17	alpha-beta T cell activation involved in	BP	0.896	IL6,GATA3,MTOR,HMGB1	up_VCAV_EC
GO:0002292	0.0579	0.986	4	17	T cell differentiation involved in immune	BP	0.896	IL6,GATA3,MTOR,HMGB1	up_VCAV_EC
GO:0002293	0.0579	0.986	4	17	alpha-beta T cell differentiation involved in	BP	0.896	IL6,GATA3,MTOR,HMGB1	up_VCAV_EC
GO:0002294	0.0579	0.986	4	17	CD4-positive, alpha-beta T cell differentiation involved in immune	BP	0.896	IL6,GATA3,MTOR,HMGB1	up_VCAV_EC
GO:0042093	0.0579	0.986	4	17	T-helper cell differentiation	BP	0.896	IL6,GATA3,MTOR,HMGB1	up_VCAV_EC
GO:0061351	0.0579	0.993	2	50	neural precursor cell proliferation	BP	1	HOOK3,ARHGEF2	up_VPOR_EC
GO:0097242	0.0579	0.999	1	7	amyloid-beta clearance	BP	1	IGF1R	down_ACOR_EC
GO:0120093	0.0579	1	1	1	regulation of peptidyl-lysine crotonylation	BP	0.874	CDYL	down_VSAP_EC
GO:0120094	0.0579	1	1	1	negative regulation of peptidyl-lysine	BP	0.874	CDYL	down_VSAP_EC
GO:0140066	0.0579	1	1	1	peptidyl-lysine crotonylation	BP	0.874	CDYL	down_VSAP_EC
GO:0106106	0.0579	0.999	1	48	cold-induced thermogenesis	BP	1	HOXC10	up_AORT_VSMC
GO:0120161	0.0579	0.999	1	48	regulation of cold-induced thermogenesis	BP	1	HOXC10	up_AORT_VSMC
GO:0043124	0.0579	0.998	1	17	negative regulation of I-kappaB kinase/NF-kappaB	BP	1	NR1H4	up_VPUL_EC
GO:0021675	0.0579	0.998	1	29	nerve development	BP	1	HOXA3	up_VPUL_VSMC
GO:0032613	0.0579	0.998	1	14	interleukin-10 production	BP	1	TLR2	up_VSAP_VSMC
GO:0032757	0.0579	0.998	1	14	positive regulation of interleukin-8 production	BP	1	TLR2	up_VSAP_VSMC
GO:0072087	0.0579	0.998	1	14	renal vesicle development	BP	1	SALL1	up_VSAP_VSMC
GO:1903115	0.0579	0.998	1	12	regulation of actin filament-based movement	BP	1	CAV1	up_VSAP_VSMC
GO:0098815	0.058	0.991	3	9	modulation of excitatory postsynaptic potential	BP	1	TMEM108,DMPK,APP	up_ACOR_VSMC
GO:0032075	0.058	0.995	2	5	positive regulation of nuclease activity	BP	1	PCNA,HMGB2	down_ACOR_VSMC
GO:0006873	0.058	0.993	2	160	cellular ion homeostasis	BP	1	TRPC4,C3AR1	up_VPOR_VSMC
GO:0014033	0.058	0.998	1	30	neural crest cell differentiation	BP	1	CITED2	down_VPOR_EC
GO:1903670	0.058	0.998	1	30	regulation of sprouting angiogenesis	BP	1	HDAC7	down_VPOR_EC
GO:0010595	0.058	0.999	1	52	positive regulation of endothelial cell migration	BP	1	WNT7A	up_AORT_VSMC
GO:0006816	0.058	0.999	1	84	calcium ion transport	BP	1	HOXA3	up_APUL_VSMC
GO:0034754	0.058	0.998	1	24	cellular hormone metabolic process	BP	1	CYP26B1	up_VPOR_VSMC
GO:0002791	0.058037524	0.97961	6	104	regulation of peptide secretion	BP	1	NR1H4,KCNB1,FZD5,NOD2,PTGER4,MAVS	up_ACOR_EC
GO:0090087	0.058056955	0.97452	9	185	regulation of peptide transport	BP	1	SH3TC2,NR1H4,KCNB1,EDEM2,FZD5,NOD2,PTGER4,MAVS,PRKCD	up_ACOR_EC
GO:0045605	0.0581	0.995	2	5	negative regulation of epidermal cell	BP	1	EZH2,HOXA7	down_ACOR_VSMC
GO:0042982	0.0581	0.998	1	15	amyloid precursor protein metabolic process	BP	1	LYN	down_ACOR_EC
GO:0016101	0.0581	0.998	1	13	diterpenoid metabolic process	BP	1	CYP26B1	up_VFEM_EC

GO:0097300	0.0581	0.998	1	17	programmed necrotic cell death	BP	1	ARHGEF2	up_VPUL_EC
GO:0006950	0.0582	0.954	107	1102	response to stress	BP	1	ELK3,HMGB1,SREBF1,FLNB,USP1,IRAK1,MCM7,RPA1,DDX39A,CBX5,HMGA1,TFPI,SREBF2,SMC1A,TFPI2,HINFP,NEDD4,WDR76,INTS7,MNDA,POLA1,SLC29A1,TRIP13,PPP1R15B,PARPBP,GINS4,SDC1,RAD54L,POLE,HM13,UBE2T,RFC2,INSIG1,CAV1,CDK1,RFC5,RFC3,RAD51,CASP2,FANCI,FANCD2,TIMELESS,TOPBP1,EZH2,MAP3K20,RFC4,ADIPOR2,FEN1,TRIM35,MND1,RNASEH2B,PLK3,PCNA,TONSL,ARG2,MCM10,RNF152,DHCR24,BRCA1,B4GALT1,PRCP,PPARD,POLH,ITGB3,NEIL3,UBE2J2,AHSA1,DTL,SUV39H1,RSL1D1,CACTIN,HMGB2,DMAP1,CDC42EP4,SMARCB1,DDX39B,ENSCAFG00000030087,RBBP8,EZR,CLSPN,SMC6,RBM14,MIOS,RBM17,RAD23A,CORO1A,POLR3F,SIGMAR1,BAP1,MELK,SMARCAL1,ELF4,TXNDC12,PSMC1,AIFM1,ARHGEF2,GLI1,H2AFX,CDC5L,ATRIP,NUDT1,EXOSC5,HMGN1,APEX1,GEN1,ANXA7,PIK3CD	down_ACOR_VSMC
GO:0009888	0.0582	0.957	63	545	tissue development	BP	1	HOXA11,FZD1,THRB,HOXA7,CDON,WNT11,HNF4A,AXIN2,OSR2,GJB5,SLIT2,HOXD3,FGF7,ABL1,EBF2,BTG1,DLL1,OSR1,FUZ,ADAMTSL4,SAV1,KLF15,ANXA4,HPSE,RILPL2,ZBTB16,TOX,RARG,STAT6,HOXC4,GTF3C5,AMBRA1,F2RL1,DLX3,DDR2,ZFP36L1,TIAM1,OMD,IRX3,ZEB1,FBXW4,SULF2,SHOX2,MYD88,LRP4,FRMD6,E2F4,NTN4,KDM2B,NFIB,ZFAND5,ARHGAP12,ACVRL1,FLOT1,BGLAP,MAP2K2,TGFBR2,NPRL3,CITED2,TRADD,LBH,KEAP1,LATS2	up_VSAP_EC
GO:0002682	0.0582	0.964	24	343	regulation of immune system process	BP	1	GPAM,NOTCH2,CFD,PRNP,SDC4,FBN1,TFE3,CLSTN1,THY1,RNF216,GLI3,PARK7,ZFP36L1,BPI,CYBA,MAVS,LOXL3,RAB7B,A2M,TSC22D3,RAC2,FOXO3,ZBTB16,ANKRD54	down_VCAV_EC
GO:0008361	0.0582	0.993	2	50	regulation of cell size	BP	1	SEMA3A,RNF6	up_VPOR_EC
GO:0009719	0.0582	1	1	395	response to endogenous stimulus	BP	1	MXRA5	down_AORT_VSMC
GO:0097699	0.0582	1	1	1	vascular endothelial cell response to fluid shear	BP	0.874	SOCS5	down_VSAP_EC
GO:0001657	0.0582	0.998	1	42	ureteric bud development	BP	1	EYA1	up_AFEM_VSMC
GO:0072163	0.0582	0.998	1	42	mesonephric epithelium development	BP	1	EYA1	up_AFEM_VSMC
GO:0072164	0.0582	0.998	1	42	mesonephric tubule development	BP	1	EYA1	up_AFEM_VSMC
GO:0007268	0.058230247	0.97754	7	130	chemical synaptic transmission	BP	1	CNTNAP4,BRSK1,PLCL1,KCNB1,INSYN1,MAPK8IP2,LRRC4	up_ACOR_EC
GO:0098916	0.058230247	0.97754	7	130	anterograde trans-synaptic signaling	BP	1	CNTNAP4,BRSK1,PLCL1,KCNB1,INSYN1,MAPK8IP2,LRRC4	up_ACOR_EC
GO:0001960	0.058265545	0.99345	2	15	negative regulation of cytokine-mediated	BP	1	NR1H4,CCDC3	up_ACOR_EC
GO:0060761	0.058265545	0.99345	2	15	negative regulation of response to cytokine	BP	1	NR1H4,CCDC3	up_ACOR_EC

GO:0051716	0.0583	0.951	212	2001	cellular response to stimulus	BP	1	SH3TC2,FGF13,RHOT1,ADRA1B,VEGFD,TLR1,SMAD6,ATRNL1,ARHGAP28,ACTN2,LGR6,PLCG2,IFNGR1,LEP,PLCL1,LBH,MAP3K8,MID1,GATA5,ADGRD1,CD83,EFNB2,CDC7,ANGPT1,ENSCAFG00000016285,HTR2B,RASSF2,MBP,ZRANB1,C28H10orf71,RECK,SKP2,NACC2,PRKAA2,MLLT3,ITM2C,FERMT1,BMP4,TM7SF3,FGFR2,TP53BP1,MFAP4,ABCA1,EIF4A2,GJA1,DOCK3,ELF1,ATRX,MED14,HRH1,CASTOR1,TLR3,SH3RF2,PPARGC1A,LATS2,PDE5A,CITED2,TP53INP1,RERG,GLRB,ARHGAP29,ZBTB40,ZNRF3,CD274,ZNF365,PLPP2,AR,PTGIR,SASH1,SLC2A5,GPR155,TMEM108,FGF18,FAM210B,ARHGEF26,NDRG2,TMX3,CHN2,CDKN2B,ADCY6,ZBTB4,POLK,ITPR1,GRK7,LIMS2,BLNK,ANKRD1,F2RL1,ADGRL1,FAM129A,CAMK2D,PSEN2,PPP2R5E,GABARAPL1,DAPK1,CTSB,ARHGAP5,BNIP3,NCEH1,AKAP6,CDKN1A,JMY,GNA14,CRLF3,CLSTN1,LRRC8C,CREBRF,NRP1,UBR1,PDE1A,PTPN22,CHD2,DMPK,NPNT,OS9,UNC5B,STT3B,EVL,SMPD1,RCAN2,APLP1,PTGDR,TBX20,SESN3,SH3RF1,CCDC3,PRKCE,MARF1,FZD3,FRS2,AGRN,RND1,SCG5,RGS2,DAG1,FUT8,PARP9,CHD6,APP,PSEN1,TGFBR1,SYNJ2BP,TESK2,SHPRH,PIK3C3,LAMP2,JUP,SIPA1L1,SENP7,CCL5,NR3C1,WWC2,PERP,PSKH1,RTKN2,D	up_ACOR_VSMC
GO:0051172	0.0583	0.958	50	663	negative regulation of nitrogen compound metabolic process	BP	0.874	PRICKLE1,NR1H4,PLK3,SERPINE1,SOCS5,ETV6,ENPP1,ERRFI1,SNX25,CBX5,TRIB3,FHL2,SMARCA4,SESN2,PTGS2,DNAJC10,NXN,PTPN2,UCHL5,MORF4L2,SPOPL,PEG3,MTDH,EIF4EBP1,CNOT7,MAGED1,CTNNB1,CRYAB,ENSCAFG00000002015,APOE,WWC3,KLF5,LYN,STRAP,DDIT3,CBLC,ATF3,VLDLR,REST,ZMYND8,EIF2S1,CDYL,PAK2,A2M,HIF1AN,SIX4,CDKN1A,SPINK2,USP14,KDM3A	down_VSAP_EC
GO:0033157	0.0583	0.971	13	82	regulation of intracellular protein transport	BP	1	SH3TC2,LEP,TCAF1,FERMT1,BMP4,CDKN1A,PTPN22,OS9,PSEN1,JUP,INPP5K,PARK7,PTPN14	up_ACOR_VSMC
GO:0051654	0.0583	0.995	2	6	establishment of mitochondrion	BP	0.874	HIF1A,TRAK1	down_VSAP_EC
GO:0044087	0.0583	0.994	2	339	regulation of cellular component biogenesis	BP	1	WNT7A,ESR1	up_AORT_VSMC
GO:0036138	0.0583	1	1	1	peptidyl-histidine hydroxylation	BP	0.874	HIF1AN	down_VSAP_EC
GO:0042265	0.0583	1	1	1	peptidyl-asparagine hydroxylation	BP	0.874	HIF1AN	down_VSAP_EC
GO:0061428	0.0583	1	1	1	negative regulation of transcription from RNA polymerase II promoter in response to hypoxia	BP	0.874	HIF1AN	down_VSAP_EC
GO:1903727	0.0583	0.998	1	16	positive regulation of phospholipid metabolic	BP	1	NR1H4	up_APUL_EC
GO:0014855	0.0583	0.998	1	22	striated muscle cell proliferation	BP	1	NKX2-5	up_VPOR_VSMC
GO:0030204	0.0583	0.998	1	14	chondroitin sulfate metabolic process	BP	1	CHST7	up_VFEM_EC
GO:0009135	0.0583	0.998	1	22	purine nucleoside diphosphate metabolic	BP	1	FBP1	up_VFEM_VSMC
GO:0009179	0.0583	0.998	1	22	purine ribonucleoside diphosphate metabolic	BP	1	FBP1	up_VFEM_VSMC
GO:0046031	0.0583	0.998	1	22	ADP metabolic process	BP	1	FBP1	up_VFEM_VSMC
GO:0006809	0.0583	0.998	1	14	nitric oxide biosynthetic process	BP	1	CAV1	up_VSAP_VSMC
GO:0046209	0.0583	0.998	1	14	nitric oxide metabolic process	BP	1	CAV1	up_VSAP_VSMC
GO:0001570	0.0584	0.979	7	37	vasculogenesis	BP	1	ACKR3,HDAC7,EMP2,WARS2,ZFP36L1,TGFBR2,CITED2	up_VSAP_EC
GO:0032502	0.0584	1	2	1661	developmental process	BP	1	JUP,GATA5	down_VFEM_VSMC
GO:0030182	0.0584	0.994	2	327	neuron differentiation	BP	1	HOXC10,WNT7A	up_AORT_VSMC
GO:0021554	0.0584	0.996	2	4	optic nerve development	BP	0.899	EPHB1,NAV2	up_VCAV_EC
GO:0006865	0.0584	0.998	1	23	amino acid transport	BP	1	TRPC4	up_VPOR_VSMC
GO:0060419	0.0584	0.998	1	22	heart growth	BP	1	NKX2-5	up_VPOR_VSMC
GO:0003229	0.0584	0.998	1	17	ventricular cardiac muscle tissue development	BP	1	TBX5	up_VPUL_EC

GO:0007015	0.0585	0.967	18	144	actin filament organization	BP	0.899	HCLS1,ARHGEF15,BAIAP2L1,ARHGAP28,TMOD1,MTOR,NCKAP1,PROX1,IQGAP2,STMN1,SWAP70,NCK1,CTNNA1,ARPC5L,TNFAIP1,TMOD3,ARHGEF2,FSCN1	up_VCAV_EC
GO:0031638	0.0585	0.989	3	15	zymogen activation	BP	0.874	SERPINE1,ENSCAFG00000005575,PGK1	down_VSAP_EC
GO:0048562	0.0585	0.993	2	102	embryonic organ morphogenesis	BP	1	HOXA3,HOXA1	up_APUL_EC
GO:0035709	0.0585	1	1	1	memory T cell activation	BP	0.874	FAM49B	down_VSAP_EC
GO:2000567	0.0585	1	1	1	regulation of memory T cell activation	BP	0.874	FAM49B	down_VSAP_EC
GO:2000568	0.0585	1	1	1	positive regulation of memory T cell activation	BP	0.874	FAM49B	down_VSAP_EC
GO:1901863	0.0585	0.998	1	25	positive regulation of muscle tissue	BP	1	SHOX2	up_VFEM_VSMC
GO:0016070	0.0586	0.981	7	1479	RNA metabolic process	BP	1	EYA1,HOXA10,HOXA9,TBX15,EMX2,HOXA11,SHOX2	up_VFEM_VSMC
GO:0032781	0.0586	0.99	3	10	positive regulation of ATPase activity	BP	1	TNNT2,DYNC1I2,DYNC1I1	up_ACOR_VSMC
GO:0044764	0.0586	0.99	3	11	multi-organism cellular process	BP	0.899	NECTIN2,RIOK3,LSM14A	up_VCAV_EC
GO:0051047	0.0586	0.993	2	84	positive regulation of secretion	BP	1	ACSL3,STX4	down_ACOR_EC
GO:0050798	0.0586	0.994	2	8	activated T cell proliferation	BP	1	GPAM,PRNP	down_VCAV_EC
GO:0032964	0.0586	0.994	2	7	collagen biosynthetic process	BP	0.874	SERPINH1,P3H4	down_VSAP_EC
GO:0003012	0.0586	0.993	2	90	muscle system process	BP	1	GATM,CAV1	up_VSAP_VSMC
GO:0002765	0.0586	1	1	1	immune response-inhibiting signal	BP	0.874	LYN	down_VSAP_EC
GO:0002767	0.0586	1	1	1	immune response-inhibiting cell surface receptor signaling	BP	0.874	LYN	down_VSAP_EC
GO:0002774	0.0586	1	1	1	Fc receptor mediated inhibitory signaling	BP	0.874	LYN	down_VSAP_EC
GO:0034136	0.0586	1	1	1	negative regulation of toll-like receptor 2 signaling	BP	0.874	LYN	down_VSAP_EC
GO:0070667	0.0586	1	1	1	negative regulation of mast cell proliferation	BP	0.874	LYN	down_VSAP_EC
GO:2001022	0.0586	0.998	1	42	positive regulation of response to DNA damage	BP	1	EYA1	up_AFEM_VSMC
GO:0030336	0.0586	0.999	1	86	negative regulation of cell migration	BP	1	TBX5	up_APUL_VSMC
GO:0055067	0.0586	0.998	1	42	monovalent inorganic cation homeostasis	BP	1	CA2	up_VCAV_VSMC
GO:0055008	0.0586	0.998	1	24	cardiac muscle tissue morphogenesis	BP	1	NKX2-5	up_VPOR_VSMC
GO:0048660	0.0587	0.98	6	35	regulation of smooth muscle cell proliferation	BP	0.899	CALCRL,TNFAIP3,PDGFB,MEF2C,EDN1,APOD	up_VCAV_EC
GO:0051261	0.0587	0.98	6	33	protein depolymerization	BP	0.899	TMOD1,STMN1,SWAP70,TAOK1,TMOD3,ARHGEF2	up_VCAV_EC
GO:0003215	0.0587	0.99	3	9	cardiac right ventricle morphogenesis	BP	1	SMARCD3,TBX20,CHD7	up_ACOR_VSMC
GO:0015711	0.0587	0.993	2	81	organic anion transport	BP	1	PDPN,ACSL3	down_ACOR_EC
GO:0050926	0.0587	0.994	2	6	regulation of positive chemotaxis	BP	0.899	CXCL8,ANGPT2	up_VCAV_EC
GO:0003281	0.0587	0.998	1	30	ventricular septum development	BP	1	TBX5	up_VPUL_VSMC
GO:0044087	0.0588	0.962	28	339	regulation of cellular component biogenesis	BP	0.874	VEGFA,PTPN11,FSCN1,DPYSL3,FBLIM1,ANKRA2,MYO1C,MSN,DYNLL1,ACTR3,CTNNB1,ANLN,CRYAB,ENSCAFG00000002015,APOE,KLF5,TWF1,PLEKHG2,GSK3B,GBP5,SH3GLB1,SDC4,HTT,REST,LRRTM2,CAPG,SIX4,NRP1	down_VSAP_EC
GO:0048016	0.0588	0.986	4	19	inositol phosphate-mediated signaling	BP	0.899	EDN1,HOMER3,PPP3CA,MTOR	up_VCAV_EC
GO:0070664	0.0588	0.989	3	18	negative regulation of leukocyte proliferation	BP	1	PRNP,SDC4,GREM1	down_VCAV_EC
GO:0048518	0.0588	1	2	1676	positive regulation of biological process	BP	1	JUP,GATA5	down_VFEM_VSMC
GO:0002686	0.0588	0.998	1	12	negative regulation of leukocyte migration	BP	1	HOXA7	down_ACOR_EC
GO:0061099	0.0588	0.998	1	13	negative regulation of protein tyrosine kinase	BP	1	CAV1	up_VSAP_VSMC
GO:0009880	0.0589	0.983	5	22	embryonic pattern specification	BP	1	SMAD6,FGFR2,NRP1,FRS2,PCSK6	up_ACOR_VSMC
GO:0050856	0.0589	0.99	3	12	regulation of T cell receptor signaling	BP	0.899	NECTIN2,PRKD2,BCL10	up_VCAV_EC

GO:0003094	0.0589	0.99	3	11	glomerular filtration	BP	0.899	ADGRF5,PDGFB,CD34	up_VCAV_EC
GO:0051130	0.0589	0.994	2	342	positive regulation of cellular component	BP	1	WNT7A,ESR1	up_AORT_VSMC
GO:0070163	0.0589	1	1	1	regulation of adiponectin secretion	BP	0.874	RAB11FIP1	down_VSAP_EC
GO:0070164	0.0589	1	1	1	negative regulation of adiponectin secretion	BP	0.874	RAB11FIP1	down_VSAP_EC
GO:0070162	0.0589	1	1	1	adiponectin secretion	BP	0.874	RAB11FIP1	down_VSAP_EC
GO:0032637	0.0589	0.998	1	26	interleukin-8 production	BP	1	BPI	up_AORT_EC
GO:2001239	0.0589	0.998	1	14	regulation of extrinsic apoptotic signaling pathway in absence of	BP	1	EYA1	up_VFEM_EC
GO:0051640	0.059	0.988	3	174	organelle localization	BP	1	LYN,SPG11,STX4	down_ACOR_EC
GO:0046599	0.059	0.99	3	11	regulation of centriole replication	BP	1	NUP62,PLK4,RBM14	down_ACOR_VSMC
GO:0090279	0.059	0.994	2	8	regulation of calcium ion import	BP	1	PRNP,TRPV2	down_VCAV_EC
GO:0003421	0.059	1	1	1	growth plate cartilage axis specification	BP	0.899	STC1	up_VCAV_EC
GO:0003422	0.059	1	1	1	growth plate cartilage morphogenesis	BP	0.899	STC1	up_VCAV_EC
GO:0044722	0.059	1	1	1	renal phosphate excretion	BP	0.899	STC1	up_VCAV_EC
GO:1903402	0.059	1	1	1	regulation of renal phosphate excretion	BP	0.899	STC1	up_VCAV_EC
GO:1903403	0.059	1	1	1	negative regulation of renal phosphate excretion	BP	0.899	STC1	up_VCAV_EC
GO:0098742	0.059	0.998	1	44	cell-cell adhesion via plasma-membrane	BP	1	GATA5	up_VCAV_VSMC
GO:0042493	0.0591	0.965	21	178	response to drug	BP	0.899	TNFAIP3,PDGFB,P2RX7,SELL,MEF2C,EDN1,PPP3CA,RALBP1,MAPK13,APOD,TIMELESS,JAG1,GUCY1B1,TOP1,CREB1,CTNNA1,BLM,RIPK1,ATRX,P2RX2,ARHGEF2	up_VCAV_EC
GO:0006875	0.0591	0.968	16	129	cellular metal ion homeostasis	BP	0.899	CP,ATP2B1,PDE4D,CHD7,GPR4,STC1,EDN1,APLNR,SLC39A8,PDZD8,SWAP70,PLCG2,HMGB1,MCU,HIF1A,ATP2A1	up_VCAV_EC
GO:0070848	0.0591	0.968	15	193	response to growth factor	BP	1	PDGFRB,SMAD9,DCN,NDN,VASN,FBN1,AKT1S1,CHRDL1,NR3C1,ASPN,ZFP36L1,FUT8,SHC1,GREM1,RGMA	down_VCAV_EC
GO:0048639	0.0591	0.978	7	39	positive regulation of developmental growth	BP	0.899	MEF2C,CHD7,EDN1,RAB11A,PROX1,CREB1,SPTBN4	up_VCAV_EC
GO:0090090	0.0591	0.982	5	44	negative regulation of canonical Wnt signaling	BP	1	GLI3,CDH2,GREM1,NPHP4,FOXO3	down_VCAV_EC
GO:0043628	0.0591	0.986	4	19	ncRNA 3'-end processing	BP	1	INTS7,FBL,ENSCAFG00000019620,EXOSC5	down_ACOR_VSMC
GO:0003281	0.0591	0.998	1	30	ventricular septum development	BP	1	CITED2	down_VPOR_EC
GO:2000042	0.0591	0.998	1	8	negative regulation of double-strand break repair via homologous	BP	1	CXHXorf57	up_VPOR_EC
GO:0055085	0.0592	0.96	35	284	transmembrane transport	BP	1	ACTN2,PLCG2,LEP,SLC26A7,KCNE3,HTR2B,TCAF1,GJA1,SLC2A5,GPR155,PTPN3,CLCN3,MICU2,ITPR1,DAPK1,CACNB3,AKAP6,LRRC8C,KCNJ4,PRKCE,PSEN1,CHD7,MFSD9,CLN8,ATP6V0E1,EDN1,KCNC4,FXYD1,INPP5K,AZIN2,SLC25A12,NDUFA4L2,PARK7,SLC8A1,SLC2A12	up_ACOR_VSMC
GO:0032273	0.0592	0.978	7	44	positive regulation of protein polymerization	BP	0.899	BAIAP2L1,MTOR,NCKAP1,IQGAP2,NCK1,FES,ARPC5L	up_VCAV_EC
GO:0060485	0.0592	0.993	2	93	mesenchyme development	BP	1	SIX1,TIAM1	up_VFEM_EC
GO:0099156	0.0592	1	1	1	cell-cell signaling via exosome	BP	0.874	KLF5	down_VSAP_EC
GO:0007399	0.0593	0.982	5	564	nervous system development	BP	1	MAPK10,HOXA3,SLITRK5,NTRK2,HOXA1	up_APUL_EC
GO:0031111	0.0593	0.99	3	11	negative regulation of microtubule polymerization or	BP	0.9	STMN1,TAOK1,ARHGEF2	up_VCAV_EC
GO:0051238	0.0593	0.998	1	7	sequestering of metal ion	BP	1	ENSCAFG00000005494	up_VPOR_EC
GO:0001953	0.0593	0.998	1	14	negative regulation of cell-matrix adhesion	BP	1	HOXA7	up_VFEM_EC
GO:0022408	0.0594	0.976	8	44	negative regulation of cell-cell adhesion	BP	1	MBP,BMP4,PDE5A,CD274,PTPN22,SPINT2,DUSP22,PAG1	up_ACOR_VSMC
GO:0097300	0.0594	0.986	4	17	programmed necrotic cell death	BP	0.9	PELI1,TLR3,RIPK1,ARHGEF2	up_VCAV_EC

GO:1904355	0.0594	0.99	3	11	positive regulation of telomere capping	BP	1	HNRNPD,AURKB,NEK2	down_ACOR_VSMC
GO:0006085	0.0594	0.994	2	8	acetyl-CoA biosynthetic process	BP	1	ACSS2,ACAT1	down_VCAV_EC
GO:0002253	0.0594	0.993	2	92	activation of immune response	BP	1	TLR2,CAV1	up_VSAP_VSMC
GO:0007632	0.0594	0.998	1	14	visual behavior	BP	1	HOXA1	down_ACOR_EC
GO:0021879	0.059472616	0.99324	2	15	forebrain neuron differentiation	BP	1	DCLK2,NRP1	up_ACOR_EC
GO:0048514	0.0595	0.965	19	214	blood vessel morphogenesis	BP	0.874	VEGFA,SULF1,MYH9,SERPINE1,SPARC,JCAD,PGK1,PRCP,PIK3C2A,HIF1A,THBS2,MTDH,ESM1,CTNNB1,STARD13,APOE,KLF5,MYO1E,NRP1	down_VSAP_EC
GO:0021561	0.0595	0.998	1	8	facial nerve development	BP	1	SEMA3A	up_VPOR_EC
GO:0021610	0.0595	0.998	1	8	facial nerve morphogenesis	BP	1	SEMA3A	up_VPOR_EC
GO:0006942	0.0595	0.998	1	26	regulation of striated muscle contraction	BP	1	NKX2-5	up_VPOR_VSMC
GO:0046887	0.0595	0.998	1	18	positive regulation of hormone secretion	BP	1	NR1H4	up_VPUL_EC
GO:0003338	0.0595	0.998	1	15	metanephros morphogenesis	BP	1	SALL1	up_VSAP_VSMC
GO:0061572	0.0596	0.976	8	50	actin filament bundle organization	BP	0.902	ARHGEF15,BAIAP2L1,ARHGAP28,MTOR,STMN1,SWAP70,TNFAIP1,FSCN1	up_VCAV_EC
GO:0032675	0.0596	0.98	6	34	regulation of interleukin-6 production	BP	0.902	TNFAIP3,TLR4,MAPK13,TLR3,HMGB1,ARHGEF2	up_VCAV_EC
GO:1903313	0.0596	0.986	4	16	positive regulation of mRNA metabolic process	BP	1	ZFP36L1,NUP98,ZFP36L2,TNRC6C	up_VSAP_EC
GO:0009798	0.0596	0.998	1	31	axis specification	BP	1	CITED2	down_VPOR_EC
GO:1902010	0.0596	1	1	1	negative regulation of translation in response to endoplasmic reticulum	BP	0.874	SESN2	down_VSAP_EC
GO:0034341	0.0596	0.998	1	43	response to interferon-gamma	BP	1	TLR2	up_AFEM_VSMC
GO:0035136	0.0597	0.986	4	16	forelimb morphogenesis	BP	1	RECK,LNPK,ATRX,TBX5	up_ACOR_VSMC
GO:0070231	0.0597	0.989	3	18	T cell apoptotic process	BP	1	GPAM,GLI3,TSC22D3	down_VCAV_EC
GO:0006290	0.0597	0.995	2	5	pyrimidine dimer repair	BP	1	POLH,HMGN1	down_ACOR_VSMC
GO:0043029	0.0597	0.994	2	8	T cell homeostasis	BP	1	GPAM,TSC22D3	down_VCAV_EC
GO:0010817	0.0597	0.993	2	102	regulation of hormone levels	BP	1	NR1H4,PCSK1N	up_APUL_EC
GO:0098876	0.0597	0.998	1	29	vesicle-mediated transport to the plasma	BP	1	ANKRD50	down_AFEM_EC
GO:0006808	0.0597	1	1	1	regulation of nitrogen utilization	BP	0.874	NR1H4	down_VSAP_EC
GO:0019676	0.0597	1	1	1	ammonia assimilation cycle	BP	0.874	NR1H4	down_VSAP_EC
GO:0019740	0.0597	1	1	1	nitrogen utilization	BP	0.874	NR1H4	down_VSAP_EC
GO:0034255	0.0597	1	1	1	regulation of urea metabolic process	BP	0.874	NR1H4	down_VSAP_EC
GO:0034971	0.0597	1	1	1	histone H3-R17 methylation	BP	0.874	NR1H4	down_VSAP_EC
GO:0038183	0.0597	1	1	1	bile acid signaling pathway	BP	0.874	NR1H4	down_VSAP_EC
GO:0038185	0.0597	1	1	1	intracellular bile acid receptor signaling	BP	0.874	NR1H4	down_VSAP_EC
GO:0045848	0.0597	1	1	1	positive regulation of nitrogen utilization	BP	0.874	NR1H4	down_VSAP_EC
GO:1903314	0.0597	1	1	1	regulation of nitrogen cycle metabolic process	BP	0.874	NR1H4	down_VSAP_EC
GO:1905693	0.0597	1	1	1	regulation of phosphatidic acid biosynthetic process	BP	0.874	NR1H4	down_VSAP_EC
GO:1905695	0.0597	1	1	1	positive regulation of phosphatidic acid biosynthetic process	BP	0.874	NR1H4	down_VSAP_EC
GO:2000211	0.0597	1	1	1	regulation of glutamate metabolic process	BP	0.874	NR1H4	down_VSAP_EC
GO:2000213	0.0597	1	1	1	positive regulation of glutamate metabolic	BP	0.874	NR1H4	down_VSAP_EC
GO:2001248	0.0597	1	1	1	regulation of ammonia assimilation cycle	BP	0.874	NR1H4	down_VSAP_EC
GO:2001250	0.0597	1	1	1	positive regulation of ammonia assimilation	BP	0.874	NR1H4	down_VSAP_EC

GO:0071695	0.0597	0.998	1	45	anatomical structure maturation	BP	1	IRX5	up_AFEM_VSMC
GO:0046620	0.0597	0.998	1	30	regulation of organ growth	BP	1	TBX5	up_VPUL_VSMC
GO:0001934	0.059741546	0.97021	12	274	positive regulation of protein phosphorylation	BP	1	SOX9,ENSCAFG00000004331,FZD5,NOD2,PAK3,TNFRSF19,MAVS,LRP4,SH3RF3,RARRES2,NRP1,PRKCD	up_ACOR_EC
GO:0008610	0.0598	0.964	21	177	lipid biosynthetic process	BP	1	SCD,ACSL3,SQLE,LSS,SREBF1,HMGCR,SF1,CTDNEP1,INSIG1,ENSCAFG00000018572,CDK4,DHCR24,BRCA1,MVD,ELOVL1,VAC14,ELOVL6,FDFT1,LIPC,NUS1,PDSS1	down_ACOR_VSMC
GO:0034762	0.0598	0.97	12	122	regulation of transmembrane transport	BP	0.874	NR1H4,ENPP1,PTPN11,TRIB3,REM1,WDR1,YES1,LYN,HTT,CALM1,YWHAH,HOMER1	down_VSAP_EC
GO:0051216	0.0598	0.977	7	70	cartilage development	BP	1	SMAD9,THBS3,BMPR1A,HOXC4,GLI3,GREM1,ZBTB16	down_VCAV_EC
GO:1903038	0.0598	0.985	4	30	negative regulation of leukocyte cell-cell	BP	1	PRNP,SDC4,GLI3,LOXL3	down_VCAV_EC
GO:0010556	0.0598	0.992	3	1137	regulation of macromolecule	BP	1	GATA5,TGFBR1,MEIS2	down_VSAP_VSMC
GO:0006012	0.0598	0.994	2	6	galactose metabolic process	BP	0.902	GALE,CHST1	up_VCAV_EC
GO:0006874	0.0598	0.993	2	91	cellular calcium ion homeostasis	BP	1	STIM1,CAV1	up_VSAP_VSMC
GO:0001834	0.0598	1	1	1	trophectodermal cell proliferation	BP	0.874	ZPR1	down_VSAP_EC
GO:0006713	0.0598	1	1	1	glucocorticoid catabolic process	BP	0.874	YWHAH	down_VSAP_EC
GO:0033566	0.0598	1	1	1	gamma-tubulin complex localization	BP	0.874	MZT1	down_VSAP_EC
GO:0042447	0.0598	1	1	1	hormone catabolic process	BP	0.874	YWHAH	down_VSAP_EC
GO:0044324	0.0598	1	1	1	regulation of transcription involved in anterior/posterior axis	BP	0.874	DDIT3	down_VSAP_EC
GO:0071931	0.0598	1	1	1	positive regulation of transcription involved in G1/S transition of mitotic	BP	0.874	ZPR1	down_VSAP_EC
GO:1903026	0.0598	1	1	1	negative regulation of RNA polymerase II regulatory region	BP	0.874	DDIT3	down_VSAP_EC
GO:1990261	0.0598	1	1	1	pre-mRNA catabolic process	BP	0.874	ZPR1	down_VSAP_EC
GO:2000016	0.0598	1	1	1	negative regulation of determination of dorsal	BP	0.874	DDIT3	down_VSAP_EC
GO:0009311	0.0598	0.998	1	21	oligosaccharide metabolic process	BP	1	FBP1	up_VFEM_VSMC
GO:0070302	0.05988216	0.98127	5	78	regulation of stress-activated protein kinase	BP	1	FZD5,NOD2,TNFRSF19,MAPK8IP2,SH3RF3	up_ACOR_EC
GO:0035265	0.05988877	0.98432	4	54	organ growth	BP	1	SOX9,FOXC2,TBX5,C23H3orf58	up_ACOR_EC
GO:0048699	0.0599	0.958	41	371	generation of neurons	BP	0.902	EPHB1,EDNRB,PRKCH,IFRD1,RELN,MEF2C,CHD7,DLL4,RAPGEF1,GATA3,RAB11A,TCF4,NTN4,PPP3CA,MTOR,HOOK3,BMP2,HMG20A,PREX2,GDPD5,PROX1,ENSCAFG00000010224,STMN1,PAK2,APOD,NCK1,FES,JAG1,MMD,RAPGEF2,CREB1,ZC4H2,CTNNA1,SPTBN4,SKIL,HIF1A,ALS2,ZEB1,ARHGEF2,CDON,ZNF609	up_VCAV_EC
GO:2000145	0.0599	0.965	20	279	regulation of cell motility	BP	1	PDGFRB,DCN,SDC4,GLIPR2,BMPR1A,LMO4,RECK,RHOD,AKT2,DOCK10,SYDE1,LAMA3,SEMA3C,THY1,SASH1,SPRED1,TWIST2,DDR2,RAC2,FOXO3	down_VCAV_EC
GO:0045664	0.0599	0.969	14	144	regulation of neuron differentiation	BP	0.874	ARHGEF2,SIPA1L1,DPYSL3,IFRD1,TNIK,UST,APOE,LYN,MMD,VLDLR,REST,DISC1,NRP1,YWHAH	down_VSAP_EC
GO:0048592	0.0599	0.974	9	53	eye morphogenesis	BP	1	THRB,CDON,IRX5,DLL1,RARG,MFN2,ZEB1,KDM2B,CITED2	up_VSAP_EC
GO:0006997	0.0599	0.978	7	42	nucleus organization	BP	1	NDC1,SF1,CTDNEP1,REEP4,HMGB2,NOLC1,EMG1	down_ACOR_VSMC
GO:0051261	0.0599	0.98	6	33	protein depolymerization	BP	1	CAPG,LIMA1,STMN1,PDXP,ARHGEF2,KIF24	down_ACOR_VSMC
GO:0051028	0.0599	0.98	6	33	mRNA transport	BP	0.902	IWS1,QKI,HHEX,THOC1,SMG7,NUP133	up_VCAV_EC
GO:0043069	0.0599	0.988	3	262	negative regulation of programmed cell death	BP	1	NR1H4,PTGER2,ARHGEF2	up_VPUL_EC
GO:0045596	0.0599	0.993	2	186	negative regulation of cell differentiation	BP	1	SRSF6,GABPA	down_AFEM_EC
GO:1901879	0.0599	0.998	1	26	regulation of protein depolymerization	BP	1	TAOK1	up_AORT_EC

GO:0035136	0.0599	0.998	1	16	forelimb morphogenesis	BP	1	TBX5	up_APUL_EC
GO:0006054	0.0599	1	1	1	N-acetylneuraminate metabolic process	BP	0.902	ST6GAL1	up_VCAV_EC
GO:0046622	0.06	0.986	4	15	positive regulation of organ growth	BP	1	FGFR2,TBX20,TBX5,EDN1	up_ACOR_VSMC
GO:0046602	0.06	0.995	2	5	regulation of mitotic centrosome separation	BP	1	NEK2,KIF11	down_ACOR_VSMC
GO:0045992	0.06	0.998	1	12	negative regulation of embryonic development	BP	1	SULF1	down_ACOR_EC
GO:0033628	0.06	0.998	1	14	regulation of cell adhesion mediated by integrin	BP	1	LYN	down_ACOR_EC
GO:0050707	0.06	0.998	1	44	regulation of cytokine secretion	BP	1	TLR2	up_AFEM_VSMC
GO:0050663	0.06	0.998	1	52	cytokine secretion	BP	1	ARG2	up_AORT_VSMC
GO:0048588	0.06	0.998	1	44	developmental cell growth	BP	1	LIMK1	up_VCAV_VSMC
GO:0032652	0.06	0.998	1	18	regulation of interleukin-1 production	BP	1	NR1H4	up_VPUL_EC
GO:0098771	0.0601	0.963	24	180	inorganic ion homeostasis	BP	1	ADRA1B,PLCG2,SLC26A7,HTR2B,CNNM2,MICU2,ITPR1,F2RL1,EGLN1,DMPK,ATP7A,PRKCE,APP,PSEN1,CHD7,DMXL1,EDN1,B2M,DISC1,HERPUD1,WFS1,ATP2C1,SLC8A1,STC2	up_ACOR_VSMC
GO:0072089	0.0601	0.976	8	43	stem cell proliferation	BP	1	FERMT1,FGFR2,ACSL6,ZNRF3,SMARCD3,FZD3,FRS2,DISC1	up_ACOR_VSMC
GO:0061448	0.0601	0.981	5	89	connective tissue development	BP	1	LEP,GREM1,PTHLH,AXIN2,PPARGC1A	up_AFEM_EC
GO:1900024	0.0601	0.986	4	16	regulation of substrate adhesion-dependent cell	BP	1	NRP1,DOCK5,RREB1,CRKL	up_ACOR_VSMC
GO:1902895	0.0601	0.989	3	15	positive regulation of pri-miRNA transcription by RNA polymerase II	BP	0.874	SMARCA4,HIF1A,KLF5	down_VSAP_EC
GO:0046461	0.0601	0.995	2	5	neutral lipid catabolic process	BP	1	ABHD5,LIPC	down_ACOR_VSMC
GO:0046464	0.0601	0.995	2	5	acylglycerol catabolic process	BP	1	ABHD5,LIPC	down_ACOR_VSMC
GO:0050806	0.0601	0.998	1	25	positive regulation of synaptic transmission	BP	1	NETO1	up_VFEM_VSMC
GO:1900182	0.060109853	0.98813	3	33	positive regulation of protein localization to	BP	1	TRIM8,MAVS,PRKCD	up_ACOR_EC
GO:0048870	0.0602	0.961	30	451	cell motility	BP	1	PDGFRB,DCN,NDN,SDC4,GLIPR2,KANK2,BMPR1A,LMO4,RECK,RHOD,AKT2,DOCK10,SYDE1,HSD3B7,LAMA3,CSPG4,SEMA3C,THY1,PEX5,B4GALT1,GLI3,SASH1,CDH2,FUT8,SPRED1,TWIST2,DDR2,GREM1,RAC2,FOXO3	down_VCAV_EC
GO:0051674	0.0602	0.961	30	451	localization of cell	BP	1	PDGFRB,DCN,NDN,SDC4,GLIPR2,KANK2,BMPR1A,LMO4,RECK,RHOD,AKT2,DOCK10,SYDE1,HSD3B7,LAMA3,CSPG4,SEMA3C,THY1,PEX5,B4GALT1,GLI3,SASH1,CDH2,FUT8,SPRED1,TWIST2,DDR2,GREM1,RAC2,FOXO3	down_VCAV_EC
GO:0006357	0.0602	0.98	6	547	regulation of transcription by RNA polymerase II	BP	1	ACVRL1,HOXA7,SULF1,ELK3,HOXA1,NEDD4	down_ACOR_EC
GO:0032615	0.0602	0.985	4	18	interleukin-12 production	BP	0.904	TLR4,IRF8,TLR3,HMGB1	up_VCAV_EC
GO:0048869	0.0602	0.987	4	1119	cellular developmental process	BP	1	SLITRK5,LIMK1,GATA5,LRRC17	up_VCAV_VSMC
GO:0002762	0.0602	0.989	3	15	negative regulation of myeloid leukocyte	BP	0.874	PTPN2,CTNNB1,LYN	down_VSAP_EC
GO:0014015	0.0602	0.99	3	11	positive regulation of gliogenesis	BP	0.904	PRKCH,MTOR,BMP2	up_VCAV_EC
GO:0045893	0.0602	1	1	413	positive regulation of transcription, DNA-	BP	1	GATA5	down_AFEM_VSMC
GO:1901222	0.0602	0.998	1	32	regulation of NIK/NF-kappaB signaling	BP	1	HDAC7	down_VPOR_EC
GO:0016477	0.060211142	0.96617	17	426	cell migration	BP	1	SOX9,AQP1,ENSCAFG00000004331,SEMA6D,DIXDC1,ZMYND8,PAK3,MMP28,PTGER4,ARHGDIB,ELMO2,FOXC2,LRRC15,TBX5,RARRES2,NRP1,PRKCD	up_ACOR_EC
GO:0002065	0.0603	0.978	7	37	columnar/cuboidal epithelial cell	BP	1	WNT11,HNF4A,ABL1,DLL1,SAV1,RARG,DLX3	up_VSAP_EC
GO:0048771	0.0603	0.981	5	43	tissue remodeling	BP	1	NOTCH2,SEMA3C,B4GALT1,NCDN,GREM1	down_VCAV_EC
GO:0048523	0.0603	0.989	4	1399	negative regulation of cellular process	BP	1	HOXA9,WNT7A,ARG2,ESR1	up_AORT_VSMC
GO:0034340	0.0603	0.989	3	15	response to type I interferon	BP	0.874	PTPN2,CNOT7,CDC37	down_VSAP_EC
GO:0048232	0.0603	0.993	2	95	male gamete generation	BP	1	HOXA9,CYP26B1	up_VFEM_EC

GO:0007283	0.0603	0.993	2	91	spermatogenesis	BP	1	HOXA11,HOXA9	up_VSAP_VSMC
GO:0032413	0.0603	0.998	1	15	negative regulation of ion transmembrane transporter activity	BP	1	NEDD4	down_ACOR_EC
GO:0007043	0.0603	0.998	1	28	cell-cell junction assembly	BP	1	FSCN1	down_AFEM_EC
GO:0061548	0.0603	0.998	1	8	ganglion development	BP	1	SEMA3A	up_VPOR_EC
GO:0009749	0.06034159	0.98804	3	34	response to glucose	BP	1	NR1H4,KCNB1,PRKACA	up_ACOR_EC
GO:0099537	0.060347598	0.97654	7	131	trans-synaptic signaling	BP	1	CNTNAP4,BRSK1,PLCL1,KCNB1,INSYN1,MAPK8IP2,LRRC4	up_ACOR_EC
GO:0031128	0.060363937	0.99308	2	15	developmental induction	BP	1	SOX9,FZD5	up_ACOR_EC
GO:2001235	0.0604	0.974	9	69	positive regulation of apoptotic signaling	BP	0.904	ATF3,PAK2,SH3GLB1,NCK1,BCL10,CTNNA1,PMAIP1,RIPK1,SKIL	up_VCAV_EC
GO:0045667	0.0604	0.988	3	38	regulation of osteoblast differentiation	BP	1	TWIST2,GREM1,AXIN2	up_AFEM_EC
GO:0007608	0.0604	0.995	2	5	sensory perception of smell	BP	1	B3GNT2,NAV2	down_ACOR_VSMC
GO:0045898	0.0604	0.995	2	5	regulation of RNA polymerase II transcriptional	BP	1	HMGB1,PSMC1	down_ACOR_VSMC
GO:0050905	0.0604	0.998	1	26	neuromuscular process	BP	1	HOXC10	up_AORT_EC
GO:0002030	0.0604	1	1	1	inhibitory G protein-coupled receptor	BP	0.904	APLNR	up_VCAV_EC
GO:1904323	0.0604	1	1	1	regulation of inhibitory G protein-coupled receptor	BP	0.904	APLNR	up_VCAV_EC
GO:1904325	0.0604	1	1	1	positive regulation of inhibitory G protein-coupled receptor	BP	0.904	APLNR	up_VCAV_EC
GO:0030857	0.0604	0.998	1	15	negative regulation of epithelial cell	BP	1	CAV1	up_VSAP_VSMC
GO:0051270	0.0605	0.964	21	296	regulation of cellular component movement	BP	1	PDGFRB,DCN,SDC4,GLIPR2,BMPR1A,LMO4,RECK,RHOD,AKT2,DOCK10,SYDE1,LAMA3,SEMA3C,THY1,B4GALT1,SASH1,SPRED1,TWIST2,DDR2,RAC2,FOXO3	down_VCAV_EC
GO:0034220	0.0605	0.988	3	188	ion transmembrane transport	BP	1	LYN,PDPN,NEDD4	down_ACOR_EC
GO:0051171	0.0605	1	2	1697	regulation of nitrogen compound metabolic	BP	1	JUP,GATA5	down_VFEM_VSMC
GO:0001756	0.0605	0.993	2	17	somitogenesis	BP	1	DLL1,AXIN2	up_AFEM_EC
GO:0042058	0.0605	0.993	2	17	regulation of epidermal growth factor receptor	BP	1	RAB7A,ADAM17	up_AFEM_EC
GO:0002158	0.0605	1	1	1	osteoclast proliferation	BP	1	GREM1	down_ACOR_VSMC
GO:0046851	0.0605	1	1	1	negative regulation of bone remodeling	BP	1	GREM1	down_ACOR_VSMC
GO:0090289	0.0605	1	1	1	regulation of osteoclast proliferation	BP	1	GREM1	down_ACOR_VSMC
GO:0090291	0.0605	1	1	1	negative regulation of osteoclast proliferation	BP	1	GREM1	down_ACOR_VSMC
GO:1902680	0.0605	1	1	415	positive regulation of RNA biosynthetic process	BP	1	GATA5	down_AFEM_VSMC
GO:1903508	0.0605	1	1	415	positive regulation of nucleic acid-templated	BP	1	GATA5	down_AFEM_VSMC
GO:0048729	0.0605	0.999	1	209	tissue morphogenesis	BP	1	GATA5	down_VFEM_VSMC
GO:1902624	0.0605	0.998	1	7	positive regulation of neutrophil migration	BP	1	C3AR1	up_VPOR_EC
GO:0019359	0.0605	0.998	1	23	nicotinamide nucleotide biosynthetic process	BP	1	FBP1	up_VFEM_VSMC
GO:0019363	0.0605	0.998	1	23	pyridine nucleotide biosynthetic process	BP	1	FBP1	up_VFEM_VSMC
GO:0003209	0.0605	0.998	1	14	cardiac atrium morphogenesis	BP	1	SHOX2	up_VSAP_VSMC
GO:0003230	0.0605	0.998	1	14	cardiac atrium development	BP	1	SHOX2	up_VSAP_VSMC
GO:0050795	0.060583875	0.99304	2	15	regulation of behavior	BP	1	CNTNAP4,PTGDS	up_ACOR_EC
GO:0032874	0.0606	0.974	9	57	positive regulation of stress-activated MAPK	BP	0.905	MAP3K11,TLR4,EDN1,MAP3K3,BMP2,TLR3,TAOK1,HMGB1,RIPK1	up_VCAV_EC
GO:0046320	0.0606	0.99	3	10	regulation of fatty acid oxidation	BP	1	ACACB,C1QTNF2,LONP2	up_VSAP_EC
GO:0098927	0.0606	0.993	2	17	vesicle-mediated transport between endosomal compartments	BP	1	MYO1D,RAB7A	up_AFEM_EC

GO:1905207	0.0606	0.993	2	17	regulation of cardiocyte differentiation	BP	1	DLL1,GREM1	up_AFEM_EC
GO:0071560	0.0606	0.998	1	72	cellular response to transforming growth	BP	1	TGFBR1	down_VSAP_VSMC
GO:0030512	0.0606	0.998	1	24	negative regulation of transforming growth factor beta receptor	BP	1	BAMBI	up_VPOR_VSMC
GO:1903845	0.0606	0.998	1	24	negative regulation of cellular response to transforming growth	BP	1	BAMBI	up_VPOR_VSMC
GO:0006094	0.0606	0.998	1	24	gluconeogenesis	BP	1	FBP1	up_VFEM_VSMC
GO:0009185	0.0606	0.998	1	23	ribonucleoside diphosphate metabolic	BP	1	FBP1	up_VFEM_VSMC
GO:1902903	0.0607	0.966	18	125	regulation of supramolecular fiber organization	BP	1	SYNPO2L,FGF13,ARHGAP28,ACTN2,MID1,SYNPO2,TUBB4A,F2RL1,JMY,NRP1,EVL,PRKCE,APP,PSEN1,SSH3,EDN1,INPP5K,WASL	up_ACOR_VSMC
GO:0072395	0.0607	0.989	3	11	signal transduction involved in cell cycle	BP	1	CASP2,DTL,CDC5L	down_ACOR_VSMC
GO:0072401	0.0607	0.989	3	11	signal transduction involved in DNA integrity	BP	1	CASP2,DTL,CDC5L	down_ACOR_VSMC
GO:0072422	0.0607	0.989	3	11	signal transduction involved in DNA damage	BP	1	CASP2,DTL,CDC5L	down_ACOR_VSMC
GO:0008217	0.0607	0.988	3	38	regulation of blood pressure	BP	1	DLL1,AVPR1A,TBXA2R	up_AFEM_EC
GO:0044091	0.0607	0.99	3	10	membrane biogenesis	BP	1	EMP2,LRP4,FLOT1	up_VSAP_EC
GO:0042692	0.0607	0.993	2	105	muscle cell differentiation	BP	1	TBX5,NPNT	up_APUL_EC
GO:0006482	0.0607	0.995	2	5	protein demethylation	BP	1	KDM2B,KDM3B	up_VSAP_EC
GO:0008214	0.0607	0.995	2	5	protein dealkylation	BP	1	KDM2B,KDM3B	up_VSAP_EC
GO:0016577	0.0607	0.995	2	5	histone demethylation	BP	1	KDM2B,KDM3B	up_VSAP_EC
GO:0070076	0.0607	0.995	2	5	histone lysine demethylation	BP	1	KDM2B,KDM3B	up_VSAP_EC
GO:0006835	0.0607	0.998	1	13	dicarboxylic acid transport	BP	1	PDPN	down_ACOR_EC
GO:2001271	0.0607	1	1	1	negative regulation of cysteine-type endopeptidase activity involved in execution	BP	0.874	PAK2	down_VSAP_EC
GO:2000027	0.0607	0.998	1	72	regulation of animal organ morphogenesis	BP	1	GATA5	down_VSAP_VSMC
GO:0070570	0.0608	0.994	2	8	regulation of neuron projection regeneration	BP	1	THY1,RGMA	down_VCAV_EC
GO:0050886	0.0608	0.993	2	17	endocrine process	BP	1	CRY2,AVPR1A	up_AFEM_EC
GO:0110111	0.0608	0.993	2	17	negative regulation of animal organ	BP	1	THRB,GREM1	up_AFEM_EC
GO:2000108	0.0608	0.998	1	12	positive regulation of leukocyte apoptotic	BP	1	LYN	down_ACOR_EC
GO:0007528	0.0608	0.998	1	17	neuromuscular junction development	BP	1	NTRK2	up_APUL_EC
GO:0046626	0.0608	0.998	1	18	regulation of insulin receptor signaling	BP	1	NR1H4	up_VPUL_EC
GO:0051253	0.0609	0.958	39	359	negative regulation of RNA metabolic process	BP	1	ELK3,HMGB1,RNPS1,SREBF1,ILF3,CBX5,ATAD2,HMGA1,SREBF2,NEDD4,U2AF2,MTDH,NAB2,BIRC5,CAV1,TIMELESS,THRAP3,FAM129B,AURKB,EZH2,GREM1,PLK3,HOXA7,UHRF1,GMNN,NOC2L,PPARD,SUV39H1,HMGB2,DMAP1,CDK2,ENSCAFG00000005299,EZR,ERF,TCERG1,HAT1,HIST2H2AC,H2AFX,SIX4	down_ACOR_VSMC
GO:0048641	0.0609	0.986	4	16	regulation of skeletal muscle tissue	BP	1	CDON,DLL1,SHOX2,FLOT1	up_VSAP_EC
GO:0090304	0.0609	1	2	1694	nucleic acid metabolic process	BP	1	JUP,GATA5	down_VFEM_VSMC
GO:0045604	0.0609	0.993	2	17	regulation of epidermal cell differentiation	BP	1	HOXA7,DLL1	up_AFEM_EC
GO:1901654	0.0609	0.998	1	31	response to ketone	BP	1	SLIT2	up_VPUL_VSMC
GO:0070201	0.060904286	0.97302	9	187	regulation of establishment of protein	BP	1	SH3TC2,NR1H4,KCNB1,EDEM2,FZD5,NOD2,PTGER4,MAVS,PRKCD	up_ACOR_EC
GO:0055082	0.061	0.962	25	189	cellular chemical homeostasis	BP	1	ADRA1B,PLCG2,SLC26A7,HTR2B,PRKAA2,MICU2,ITPR1,F2RL1,EGLN1,DMPK,ATP7A,ORMDL3,PRKCE,APP,PSEN1,CHD7,DMXL1,RAB11B,EDN1,DISC1,HERPUD1,WFS1,ATP2C1,SLC8A1,STC2	up_ACOR_VSMC

GO:0044106	0.061	0.993	2	17	cellular amine metabolic process	BP	1	MOXD1,NAPEPLD	up_AFEM_EC
GO:0090100	0.061	0.998	1	31	positive regulation of transmembrane receptor protein serine/threonine kinase signaling pathway	BP	1	CITED2	down_VPOR_EC
GO:1904888	0.061	0.998	1	31	cranial skeletal system development	BP	1	TBX15	down_VPOR_EC
GO:0006352	0.061	0.998	1	56	DNA-templated transcription, initiation	BP	1	ESR1	up_AORT_VSMC
GO:0060078	0.061	0.998	1	17	regulation of postsynaptic membrane potential	BP	1	NETO1	up_VSAP_VSMC
GO:0099565	0.061	0.998	1	17	chemical synaptic transmission, postsynaptic	BP	1	NETO1	up_VSAP_VSMC
GO:1904029	0.0611	0.982	5	28	regulation of cyclin-dependent protein kinase	BP	0.905	TNFAIP3,PDGFB,HHEX,PROX1,BLM	up_VCAV_EC
GO:0120039	0.0611	0.988	3	158	plasma membrane bounded cell projection	BP	1	SPG11,PDPN,NRP2	down_ACOR_EC
GO:0051149	0.0611	0.998	1	25	positive regulation of muscle cell differentiation	BP	1	SHOX2	up_VFEM_VSMC
GO:0044093	0.0612	0.956	52	437	positive regulation of molecular function	BP	1	TNNT2,ACTN2,PLCG2,LEP,PLCL1,MAP3K8,HTR2B,RASSF2,TCAF1,USP6NL,MBP,BMP4,EVI5L,FGFR2,TLR3,PPARGC1A,PDE5A,PKP4,AR,SASH1,LMO4,FGF18,F2RL1,DAPK1,AKAP6,JMY,NPNT,ATP7A,CCNT1,FRS2,PARP9,APP,PSEN1,SGSM2,JUP,CCL5,DDR2,PERP,EDN1,DYNC1I2,AKTIP,NOD2,ARID5B,B2M,DCUN1D4,HERPUD1,MAP3K7,DYNC1I1,PARK7,IL18R1,HMBOX1,LMF1	up_ACOR_VSMC
GO:0034655	0.0612	0.966	17	135	nucleobase-containing compound catabolic process	BP	1	RNPS1,EIF4A3,EDC3,HNRNPD,THRAP3,EDC4,FEN1,RNASEH2B,SMG9,DIS3,FASTKD3,AIFM1,ENSCAFG00000015189,RNASEH2C,NUDT1,EXOSC5,NUDT3	down_ACOR_VSMC
GO:0044237	0.0612	0.982	11	3491	cellular metabolic process	BP	1	ETFA,IVNS1ABP,EXTL2,DARS,SUCLG2,EIF3M,SRSF6,USP33,GABPA,NDUFA10,TSEN15	down_AFEM_EC
GO:0033135	0.0612	0.977	7	40	regulation of peptidyl-serine phosphorylation	BP	0.905	HCLS1,PDE4D,PRKD2,NCK1,NSD1,PTGS2,SPTBN4	up_VCAV_EC
GO:0072359	0.0612	0.988	3	368	circulatory system development	BP	1	HOXA3,NKX2-5,C3AR1	up_VPOR_VSMC
GO:0006098	0.0612	0.994	2	8	pentose-phosphate shunt	BP	1	H6PD,PGLS	down_VCAV_EC
GO:0051156	0.0612	0.994	2	8	glucose 6-phosphate metabolic process	BP	1	H6PD,PGLS	down_VCAV_EC
GO:0048598	0.0612	0.993	2	207	embryonic morphogenesis	BP	1	CITED2,TBX15	down_VPOR_EC
GO:0090200	0.0612	0.993	2	10	positive regulation of release of cytochrome c	BP	0.874	BNIP3,FAM162A	down_VSAP_EC
GO:0032989	0.0612	0.993	2	292	cellular component morphogenesis	BP	1	SLITRK5,LIMK1	up_VCAV_VSMC
GO:0018105	0.0612	0.993	2	116	peptidyl-serine phosphorylation	BP	1	STK32B,PTGER2	up_VPUL_EC
GO:0048486	0.0612	0.998	1	10	parasympathetic nervous system development	BP	1	NRP2	down_ACOR_EC
GO:0000041	0.0612	0.998	1	31	transition metal ion transport	BP	1	RAB11B	down_VPOR_EC
GO:0048514	0.0612	0.999	1	214	blood vessel morphogenesis	BP	1	JUP	down_VFEM_VSMC
GO:0010828	0.0612	0.998	1	16	positive regulation of glucose transmembrane	BP	1	NR1H4	up_APUL_EC
GO:0060038	0.0612	0.998	1	18	cardiac muscle cell proliferation	BP	1	TBX5	up_VPUL_EC
GO:0055007	0.0612	0.998	1	31	cardiac muscle cell differentiation	BP	1	TBX5	up_VPUL_VSMC
GO:0070542	0.061240418	0.99293	2	15	response to fatty acid	BP	1	NR1H4,PTGER4	up_ACOR_EC
GO:0032675	0.061281293	0.98778	3	34	regulation of interleukin-6 production	BP	1	NR1H4,NOD2,MAVS	up_ACOR_EC
GO:0043604	0.0613	0.963	21	288	amide biosynthetic process	BP	1	UBA52,LARP6,COA1,SAMD4B,RPL23,RPL13,EIF3K,MRPL37,RPL27A,EEF2,ZFP36L1,RPL27,MRPS11,ORMDL3,RPS27,ACSS2,ENSCAFG00000015122,PYM1,FOXO3,EIF4EBP1,ACAT1	down_VCAV_EC
GO:0034113	0.0613	0.989	3	10	heterotypic cell-cell adhesion	BP	1	MBP,JUP,PERP	up_ACOR_VSMC
GO:0006692	0.0613	0.989	3	11	prostanoid metabolic process	BP	0.905	EDN1,PTGS1,PTGS2	up_VCAV_EC
GO:0006693	0.0613	0.989	3	11	prostaglandin metabolic process	BP	0.905	EDN1,PTGS1,PTGS2	up_VCAV_EC
GO:1903428	0.0613	0.994	2	8	positive regulation of reactive oxygen species biosynthetic process	BP	1	PARK7,CYBA	down_VCAV_EC

GO:0015988	0.0613	0.993	2	17	energy coupled proton transmembrane transport, against	BP	1	ATP6V1B2,ATP6V0B	up_AFEM_EC
GO:0046661	0.0613	0.998	1	45	male sex differentiation	BP	1	HOXA9	up_AFEM_VSMC
GO:0030857	0.0613	0.998	1	15	negative regulation of epithelial cell	BP	1	HOXA7	up_VFEM_EC
GO:0002762	0.0613	0.998	1	15	negative regulation of myeloid leukocyte	BP	1	HOXA7	up_VFEM_EC
GO:0008652	0.0613	0.998	1	24	cellular amino acid biosynthetic process	BP	1	GATM	up_VFEM_VSMC
GO:1901607	0.0613	0.998	1	24	alpha-amino acid biosynthetic process	BP	1	GATM	up_VFEM_VSMC
GO:0007165	0.0614	0.98	7	1496	signal transduction	BP	1	FGF5,BARX1,NKX2-5,BAMBI,ANKRD6,CYP26B1,C3AR1	up_VPOR_VSMC
GO:0015672	0.0614	0.981	5	89	monovalent inorganic cation transport	BP	1	KCNC4,ATP6V1B2,FXYD1,ATP6V0B,DMPK	up_AFEM_EC
GO:0006336	0.0614	0.989	3	11	DNA replication-independent nucleosome	BP	0.905	NASP,ASF1A,ATRX	up_VCAV_EC
GO:0048514	0.0614	0.988	3	214	blood vessel morphogenesis	BP	1	EYA1,HOXA7,SIX1	up_VFEM_EC
GO:0099132	0.0614	0.993	2	17	ATP hydrolysis coupled cation transmembrane	BP	1	ATP6V1B2,ATP6V0B	up_AFEM_EC
GO:0003197	0.0614	0.998	1	16	endocardial cushion development	BP	1	TBX5	up_APUL_EC
GO:0051049	0.0615	0.958	37	479	regulation of transport	BP	0.874	VEGFA,NR1H4,PLK3,SERPINE1,PTPN14,ENPP1,PTPN11,TRIB3,PPP1R12A,REM1,WDR1,MYO1C,MSN,KIF5B,HIF1A,IWS1,YIPF5,ZPR1,YES1,CTNNB1,CRYAB,APOE,LYN,GSK3B,NKAIN1,SH3GLB1,SDC4,HTT,REST,CALM1,RAB11FIP1,LRRTM2,INHBA,CDKN1A,RHBDF2,YWHAH,HOMER1	down_VSAP_EC
GO:0045892	0.0615	0.958	36	328	negative regulation of transcription, DNA-templated	BP	1	ELK3,HMGB1,SREBF1,ILF3,CBX5,ATAD2,HMGA1,SREBF2,NEDD4,MTDH,NAB2,BIRC5,CAV1,TIMELESS,FAM129B,AURKB,EZH2,GREM1,PLK3,HOXA7,UHRF1,GMNN,NOC2L,PPARD,SUV39H1,HMGB2,DMAP1,CDK2,ENSCAFG00000005299,EZR,ERF,TCERG1,HAT1,HIST2H2AC,H2AFX,SIX4	down_ACOR_VSMC
GO:0060326	0.0615	0.971	11	84	cell chemotaxis	BP	0.905	EPHB1,EDNRB,KIT,CXCL8,DOCK4,PDGFB,PDE4D,MMP28,PRKD2,SWAP70,HMGB1	up_VCAV_EC
GO:0043536	0.0615	0.998	1	32	positive regulation of blood vessel endothelial	BP	1	HDAC7	down_VPOR_EC
GO:0043316	0.0615	1	1	1	cytotoxic T cell degranulation	BP	0.905	RAB27A	up_VCAV_EC
GO:1903433	0.0615	1	1	1	regulation of constitutive secretory pathway	BP	0.905	RAB27A	up_VCAV_EC
GO:0097278	0.0615	1	1	1	complement-dependent cytotoxicity	BP	0.905	RAB27A	up_VCAV_EC
GO:1903435	0.0615	1	1	1	positive regulation of constitutive secretory	BP	0.905	RAB27A	up_VCAV_EC
GO:0060420	0.0615	0.998	1	18	regulation of heart growth	BP	1	TBX5	up_VPUL_EC
GO:0006605	0.0616	0.973	9	99	protein targeting	BP	1	PRNP,RHOD,AKT2,SSR2,SSR3,PEX5,SEC61B,LONP2,SEC61A1	down_VCAV_EC
GO:0071705	0.0616	0.979	6	582	nitrogen compound transport	BP	1	LYN,PDPN,IPO7,ACSL3,STX4,NEDD4	down_ACOR_EC
GO:0051493	0.0616	0.993	2	188	regulation of cytoskeleton organization	BP	1	GPSM2,FSCN1	down_AFEM_EC
GO:0022414	0.0616	0.993	2	294	reproductive process	BP	1	HOXA9,IRX5	up_AFEM_VSMC
GO:0042773	0.0616	0.998	1	32	ATP synthesis coupled electron transport	BP	1	NDUFA10	down_AFEM_EC
GO:0017157	0.0616	0.998	1	58	regulation of exocytosis	BP	1	WNT7A	up_AORT_VSMC
GO:0061448	0.0616	0.998	1	89	connective tissue development	BP	1	HOXA3	up_APUL_VSMC
GO:0030593	0.0616	0.998	1	19	neutrophil chemotaxis	BP	1	CCL24	up_VPUL_EC
GO:0097305	0.0616	0.998	1	31	response to alcohol	BP	1	SLIT2	up_VPUL_VSMC
GO:0098662	0.0617	0.965	18	131	inorganic cation transmembrane transport	BP	1	ACTN2,PLCG2,KCNE3,HTR2B,PTPN3,MICU2,ITPR1,CACNB3,AKAP6,KCNJ4,PRKCE,CHD7,ATP6V0E1,KCNC4,FXYD1,NDUFA4L2,PARK7,SLC8A1	up_ACOR_VSMC
GO:0048858	0.0617	0.988	3	159	cell projection morphogenesis	BP	1	SPG11,PDPN,NRP2	down_ACOR_EC
GO:0071825	0.0617	0.998	1	12	protein-lipid complex subunit organization	BP	1	ACSL3	down_ACOR_EC

GO:0071827	0.0617	0.998	1	12	plasma lipoprotein particle organization	BP	1	ACSL3	down_ACOR_EC
GO:0006434	0.0617	1	1	1	seryl-tRNA aminoacylation	BP	0.874	SARS	down_VSAP_EC
GO:0061351	0.0617	0.998	1	50	neural precursor cell proliferation	BP	1	WNT7A	up_AORT_VSMC
GO:0007026	0.0617	0.998	1	8	negative regulation of microtubule	BP	1	ARHGEF2	up_VPOR_EC
GO:0031326	0.0618	0.974	10	1176	regulation of cellular biosynthetic process	BP	1	ACVRL1,YEATS2,YBX3,HIVEP2,HOXA7,SULF1,ELK3,HOXA1,ACSL3,NEDD4	down_ACOR_EC
GO:0051248	0.0618	0.984	4	316	negative regulation of protein metabolic process	BP	1	LYN,IGF1R,PRKAR2A,ASPN	down_ACOR_EC
GO:0000132	0.0618	0.989	3	11	establishment of mitotic spindle orientation	BP	1	ZW10,CENPA,NDC80	down_ACOR_VSMC
GO:0051294	0.0618	0.989	3	11	establishment of spindle orientation	BP	1	ZW10,CENPA,NDC80	down_ACOR_VSMC
GO:0019219	0.0618	0.991	3	1151	regulation of nucleobase-containing compound metabolic process	BP	1	GATA5,TGFBR1,MEIS2	down_VSAP_VSMC
GO:0055002	0.0618	0.998	1	45	striated muscle cell development	BP	1	BEND2	up_AFEM_VSMC
GO:0048522	0.0619	0.95	140	1484	positive regulation of cellular process	BP	1	ACSL3,FSCN1,HMGB1,RNPS1,SREBF1,IRAK1,ANLN,ILF3,PPRC1,ATAD2,HMGA1,EIF4A3,HNRNPD,SREBF2,HINFP,UBE2S,NEDD4,U2AF2,MNDA,SLC29A1,MTDH,HOXA3,COLGALT1,SLC25A5,SRRT,SDC1,CTDNEP1,FBXO5,SLC35C2,PRELID1,CDCA7L,RFC2,BIRC5,INSIG1,PRMT1,CAV1,CDK1,RFC5,RFC3,CASP2,FANCI,TIMELESS,THRAP3,TRUB2,FAM129B,NF2,AURKB,MMP14,ABHD5,EZH2,STMN1,PPP2R5D,GREM1,MAP3K20,RFC4,NEK2,FEN1,CDK4,TRIM35,SMARCC1,RNASEH2B,PLK3,PCNA,DHX33,BID,ARG2,SF3B4,CKS2,PDCD2,RNF152,POC1A,BRCA1,MVD,FASTKD3,EIF5A,E2F8,B4GALT1,TFRC,NRAS,PPARD,CDC6,NDC80,RAP2C,HIST1H1A,CYCS,FAM49B,NCL,RAC1,NUP62,EMP2,DTL,PDXP,FOSL1,PLK4,IQGAP2,HMGB2,GSTO1,CDC42EP4,CDK2,FGF5,DDX39B,CCNA2,ENSCAFG00000005299,EZR,CLSPN,SMC6,TOX,RBM14,MIOS,RAD23A,CORO1A,BEND6,CDC20,ASPM,MELK,ARPC1B,PRMT5,PSMC1,CTH,AIFM1,ARHGEF2,CDC25A,SUPT16H,TWIST2,TADA2A,HOXA9,GLI1,JMJD6,UBE2C,NUS1,CCT4,AMOTL1,MTBP,DEPDC1B,TPBG,ENSCAFG00000017103,GEN1,AMER1,DCUN1D5,PIK3CD	down_ACOR_VSMC
GO:0006641	0.0619	0.984	4	25	triglyceride metabolic process	BP	0.874	PTPN11,SEL1L,APOE,ABHD5	down_VSAP_EC
GO:0050900	0.0619	0.992	2	89	leukocyte migration	BP	1	LYN,HOXA7	down_ACOR_EC
GO:0001822	0.0619	0.992	2	98	kidney development	BP	1	HOXA11,SALL1	up_VSAP_VSMC
GO:0003229	0.0619	0.998	1	17	ventricular cardiac muscle tissue development	BP	1	TBX5	up_APUL_EC
GO:0071225	0.0619	0.998	1	8	cellular response to muramyl dipeptide	BP	1	ARHGEF2	up_VPOR_EC
GO:0003170	0.0619	0.998	1	25	heart valve development	BP	1	SHOX2	up_VFEM_VSMC
GO:0048660	0.061949341	0.98757	3	35	regulation of smooth muscle cell proliferation	BP	1	NDRG2,PTGIR,CLSTN1	up_ACOR_EC
GO:0031327	0.062	0.956	44	412	negative regulation of cellular biosynthetic process	BP	1	ELK3,HMGB1,SREBF1,ILF3,CBX5,ATAD2,HMGA1,EIF4A3,SREBF2,SMC1A,NEDD4,MTDH,NAB2,BIRC5,INSIG1,CAV1,TIMELESS,FAM129B,AURKB,EZH2,GREM1,PLK3,HOXA7,UHRF1,GMNN,BRCA1,NOC2L,PPARD,NCL,SUV39H1,HMGB2,DMAP1,SMC3,CDK2,ENSCAFG00000005299,EZR,RBM14,ERF,TCERG1,HAT1,HIST2H2AC,TINF2,H2AFX,SIX4	down_ACOR_VSMC
GO:0032147	0.062	0.971	11	77	activation of protein kinase activity	BP	0.905	KIT,MAP3K11,PDGFB,STK10,TLR4,MAP3K3,BMP2,SLK,MADD,PAK2,RIPK1	up_VCAV_EC
GO:0000003	0.062	0.993	2	295	reproduction	BP	1	HOXA9,IRX5	up_AFEM_VSMC
GO:0010575	0.062	0.998	1	8	positive regulation of vascular endothelial growth factor production	BP	1	C3AR1	up_VPOR_EC
GO:0034637	0.062	0.998	1	25	cellular carbohydrate biosynthetic process	BP	1	FBP1	up_VFEM_VSMC
GO:0002053	0.062	0.998	1	15	positive regulation of mesenchymal cell	BP	1	SHOX2	up_VSAP_VSMC

GO:0050879	0.0621	0.985	4	16	multicellular organismal movement	BP	1	ITPR1,DMPK,CLN8,GAA	up_ACOR_VSMC
GO:0050881	0.0621	0.985	4	16	musculoskeletal movement	BP	1	ITPR1,DMPK,CLN8,GAA	up_ACOR_VSMC
GO:0042737	0.0621	0.988	3	18	drug catabolic process	BP	1	MAOA,CHID1,ACAT1	down_VCAV_EC
GO:0010571	0.0621	0.995	2	4	positive regulation of nuclear cell cycle DNA	BP	1	CDC7,ATRX	up_ACOR_VSMC
GO:0048709	0.0621	0.998	1	17	oligodendrocyte differentiation	BP	1	NTRK2	up_APUL_EC
GO:0007632	0.0621	0.998	1	14	visual behavior	BP	1	NETO1	up_VSAP_VSMC
GO:0035116	0.0621	0.998	1	15	embryonic hindlimb morphogenesis	BP	1	PITX1	up_VSAP_VSMC
GO:0048646	0.0622	0.961	24	349	anatomical structure formation involved in morphogenesis	BP	1	HSPB6,PDGFRB,DCN,ANGPT1,SDC4,THBS3,BMPR1A,LMO4,ANPEP,COL6A1,LAMA3,SEMA3C,THY1,TLX2,B4GALT1,GLI3,MYDGF,SASH1,IFT57,SPRED1,SHC1,RDH10,GREM1,CLIC4	down_VCAV_EC
GO:0043410	0.0622	0.964	18	144	positive regulation of MAPK cascade	BP	0.905	TEK,KIT,MAP3K11,PDGFB,TLR4,EDN1,MAP3K3,BMP2,MADD,PRKD2,TLR3,RAPGEF2,TAOK1,HMGB1,RIPK1,PDGFD,JCAD,CDON	up_VCAV_EC
GO:0043931	0.0622	0.993	2	9	ossification involved in bone maturation	BP	1	THBS3,GREM1	down_VCAV_EC
GO:0070977	0.0622	0.993	2	9	bone maturation	BP	1	THBS3,GREM1	down_VCAV_EC
GO:0050862	0.0622	0.994	2	5	positive regulation of T cell receptor signaling	BP	0.905	NECTIN2,PRKD2	up_VCAV_EC
GO:0006721	0.0622	0.998	1	14	terpenoid metabolic process	BP	1	CYP26B1	up_VFEM_EC
GO:0003401	0.0622	0.998	1	15	axis elongation	BP	1	SIX1	up_VFEM_EC
GO:0060996	0.0623	0.981	5	26	dendritic spine development	BP	0.905	EPHB1,RELN,MEF2C,ENSCAFG00000010224,PAK2	up_VCAV_EC
GO:0045601	0.0623	0.989	3	10	regulation of endothelial cell differentiation	BP	1	BTG1,ZEB1,ACVRL1	up_VSAP_EC
GO:0045844	0.0623	0.998	1	24	positive regulation of striated muscle tissue	BP	1	CYP26B1	up_VPOR_VSMC
GO:0048636	0.0623	0.998	1	24	positive regulation of muscle organ	BP	1	CYP26B1	up_VPOR_VSMC
GO:0071396	0.0624	0.964	19	134	cellular response to lipid	BP	1	LBH,SKP2,PRKAA2,BMP4,ABCA1,MED14,AR,SASH1,FAM210B,ADCY6,ANKRD1,CREBRF,PTGDR,PRKCE,NR3C1,NOD2,FAM107A,DDIT4,PARK7	up_ACOR_VSMC
GO:0030282	0.0624	0.977	7	37	bone mineralization	BP	1	WNT11,AXIN2,OSR2,OSR1,DDR2,OMD,BGLAP	up_VSAP_EC
GO:2000648	0.0624	0.988	3	14	positive regulation of stem cell proliferation	BP	0.874	HIF1A,CTNNB1,DISC1	down_VSAP_EC
GO:0034142	0.0624	0.998	1	14	toll-like receptor 4 signaling pathway	BP	1	LYN	down_ACOR_EC
GO:0030216	0.0624	0.998	1	29	keratinocyte differentiation	BP	1	SRSF6	down_AFEM_EC
GO:0031397	0.0624	0.998	1	30	negative regulation of protein ubiquitination	BP	1	IVNS1ABP	down_AFEM_EC
GO:1990845	0.0624	0.998	1	51	adaptive thermogenesis	BP	1	HOXC10	up_AORT_VSMC
GO:0060544	0.0624	0.998	1	8	regulation of necroptotic process	BP	1	ARHGEF2	up_VPOR_EC
GO:0040007	0.0625	0.97	11	276	growth	BP	1	MT2A,LEP,ENSCAFG00000009113,GAS1,DLL1,PLXNA4,GPAM,DCUN1D3,LGR6,ACACB,ADAM17	up_AFEM_EC
GO:1903670	0.0625	0.979	6	30	regulation of sprouting angiogenesis	BP	1	ABL1,HDAC7,DLL1,EPN2,TBXA2R,HMOX1	up_VSAP_EC
GO:0030855	0.0625	0.987	3	183	epithelial cell differentiation	BP	1	ACVRL1,PDPN,HOXA7	down_ACOR_EC
GO:0015672	0.0625	0.992	2	89	monovalent inorganic cation transport	BP	1	NETO1,CAV1	up_VSAP_VSMC
GO:0097205	0.0625	0.998	1	12	renal filtration	BP	1	SULF1	down_ACOR_EC
GO:1904464	0.0625	1	1	1	regulation of matrix metallopeptidase	BP	0.905	CD200	up_VCAV_EC
GO:1904465	0.0625	1	1	1	negative regulation of matrix metallopeptidase	BP	0.905	CD200	up_VCAV_EC
GO:1990773	0.0625	1	1	1	matrix metallopeptidase secretion	BP	0.905	CD200	up_VCAV_EC
GO:0008045	0.0625	0.998	1	9	motor neuron axon guidance	BP	1	SEMA3A	up_VPOR_EC
GO:0050654	0.0625	0.998	1	15	chondroitin sulfate proteoglycan metabolic	BP	1	CHST7	up_VFEM_EC

GO:0034330	0.062578839	0.98017	5	80	cell junction organization	BP	1	NR1H4,ARHGAP6,FZD5,ACTN2,NRP1	up_ACOR_EC
GO:0035966	0.0626	0.975	8	47	response to topologically incorrect protein	BP	1	TM7SF3,CREBRF,STT3B,JKAMP,HERPUD1,WFS1,DZIP3,STC2	up_ACOR_VSMC
GO:0003012	0.0626	0.98	5	90	muscle system process	BP	1	LMCD1,CHRM2,HTR1D,TBXA2R,DMPK	up_AFEM_EC
GO:0007229	0.0626	0.984	4	32	integrin-mediated signaling pathway	BP	1	ITGB5,THY1,LOXL3,FUT8	down_VCAV_EC
GO:0045766	0.0626	0.983	4	63	positive regulation of angiogenesis	BP	1	ANGPT4,DLL1,F3,GREM1	up_AFEM_EC
GO:0006312	0.0626	0.989	3	11	mitotic recombination	BP	1	RAD51,SMC6,GEN1	down_ACOR_VSMC
GO:0001775	0.0626	0.987	3	219	cell activation	BP	1	HOXA9,TOX,CYP26B1	up_VFEM_EC
GO:0090232	0.0626	0.994	2	5	positive regulation of spindle checkpoint	BP	1	NDC80,GEN1	down_ACOR_VSMC
GO:0090267	0.0626	0.994	2	5	positive regulation of mitotic cell cycle spindle	BP	1	NDC80,GEN1	down_ACOR_VSMC
GO:0045935	0.0626	0.994	2	491	positive regulation of nucleobase-containing compound metabolic	BP	1	GATA5,TGFBR1	down_VSAP_VSMC
GO:0009967	0.0626	1	1	435	positive regulation of signal transduction	BP	1	GATA5	down_AFEM_VSMC
GO:0060411	0.0626	0.998	1	33	cardiac septum morphogenesis	BP	1	CITED2	down_VPOR_EC
GO:2000146	0.0626	0.998	1	92	negative regulation of cell motility	BP	1	TBX5	up_APUL_VSMC
GO:0010469	0.0626	0.998	1	26	regulation of signaling receptor activity	BP	1	NETO1	up_VFEM_VSMC
GO:0045446	0.0626	0.998	1	32	endothelial cell differentiation	BP	1	HOXB5	up_VPUL_VSMC
GO:0006753	0.0627	0.961	24	211	nucleoside phosphate metabolic process	BP	1	PRPSAP1,ACLY,HMGCR,NNT,RRM1,RAN,CDK1,PFAS,RNASEH2B,MVD,ELOVL1,CYCS,ELOVL6,CYC1,ATP5F1A,DCK,PRPS1,ENSCAFG00000013366,PDHA1,NUDT1,PGD,CTPS1,ATP5F1D,NUDT3	down_ACOR_VSMC
GO:0002822	0.0627	0.976	7	42	regulation of adaptive immune response based on somatic recombination of immune receptors built from immunoglobulin superfamily domains	BP	0.905	IL7R,TNFAIP3,MEF2C,GATA3,NECTIN2,THOC1,HMGB1	up_VCAV_EC
GO:0071482	0.0627	0.979	6	34	cellular response to light stimulus	BP	1	NEDD4,AURKB,PCNA,NOC2L,POLH,CDC25A	down_ACOR_VSMC
GO:1901879	0.0627	0.981	5	26	regulation of protein depolymerization	BP	0.905	TMOD1,SWAP70,TAOK1,TMOD3,ARHGEF2	up_VCAV_EC
GO:0015698	0.0627	0.984	4	24	inorganic anion transport	BP	0.874	ENPP1,SLC20A1,CLCN3,SLC20A2	down_VSAP_EC
GO:0050918	0.0627	0.989	3	10	positive chemotaxis	BP	1	VEGFD,F2RL1,NRP1	up_ACOR_VSMC
GO:0045824	0.0627	0.989	3	11	negative regulation of innate immune response	BP	0.905	TNFAIP3,NLRC5,CNOT7	up_VCAV_EC
GO:0010557	0.0627	0.994	2	492	positive regulation of macromolecule biosynthetic process	BP	1	GATA5,TGFBR1	down_VSAP_VSMC
GO:1904754	0.0627	0.995	2	4	positive regulation of vascular associated smooth muscle cell	BP	0.905	DOCK4,PDGFB	up_VCAV_EC
GO:0046061	0.0627	1	1	1	dATP catabolic process	BP	1	NUDT1	down_ACOR_VSMC
GO:0001823	0.0627	0.998	1	45	mesonephros development	BP	1	EYA1	up_AFEM_VSMC
GO:0007164	0.0627	0.998	1	23	establishment of tissue polarity	BP	1	ANKRD6	up_VPOR_VSMC
GO:0001736	0.0627	0.998	1	23	establishment of planar polarity	BP	1	ANKRD6	up_VPOR_VSMC
GO:0002053	0.0627	0.998	1	15	positive regulation of mesenchymal cell	BP	1	SIX1	up_VFEM_EC
GO:0042472	0.0627	0.998	1	27	inner ear morphogenesis	BP	1	EYA1	up_VFEM_VSMC
GO:0050905	0.0627	0.998	1	26	neuromuscular process	BP	1	HOXC10	up_VFEM_VSMC
GO:0032635	0.0628	0.977	7	37	interleukin-6 production	BP	1	TLR1,LEP,MBP,TLR3,F2RL1,PTPN22,NOD2	up_ACOR_VSMC
GO:0060438	0.0628	0.989	3	10	trachea development	BP	1	RARG,MAP2K2,TGFBR2	up_VSAP_EC
GO:0060571	0.0628	0.998	1	13	morphogenesis of an epithelial fold	BP	1	SULF1	down_ACOR_EC
GO:0051254	0.0628	1	1	431	positive regulation of RNA metabolic process	BP	1	GATA5	down_AFEM_VSMC

GO:0032621	0.0628	1	1	1	interleukin-18 production	BP	0.874	GBP5	down_VSAP_EC
GO:0072616	0.0628	1	1	1	interleukin-18 secretion	BP	0.874	GBP5	down_VSAP_EC
GO:0018108	0.0629	0.992	2	79	peptidyl-tyrosine phosphorylation	BP	1	LYN,IGF1R	down_ACOR_EC
GO:0008203	0.063	0.976	7	37	cholesterol metabolic process	BP	1	LEP,LCAT,ABCA1,LIPE,APP,CLN8,LMF1	up_ACOR_VSMC
GO:0009064	0.063	0.981	5	23	glutamine family amino acid metabolic process	BP	1	GOT1,GLUL,ADHFE1,ENSCAFG00000016140,ASS1	up_VSAP_EC
GO:0034637	0.063	0.984	4	25	cellular carbohydrate biosynthetic process	BP	0.874	ENPP1,ENSCAFG00000032470,PPP1R3C,EXT1	down_VSAP_EC
GO:1901360	0.063	0.991	4	2016	organic cyclic compound metabolic process	BP	1	AK5,GATA5,TGFBR1,MEIS2	down_VSAP_VSMC
GO:0006101	0.063	0.985	4	16	citrate metabolic process	BP	1	DLST,IDH1,IDH3A,ENSCAFG00000006639	up_VSAP_EC
GO:0002369	0.063	0.989	3	10	T cell cytokine production	BP	1	B2M,MAP3K7,IL18R1	up_ACOR_VSMC
GO:0040036	0.063	0.998	1	11	regulation of fibroblast growth factor receptor	BP	1	SULF1	down_ACOR_EC
GO:0060539	0.063	1	1	1	diaphragm development	BP	1	MSC	down_ACOR_VSMC
GO:0033277	0.063	1	1	1	abortive mitotic cell cycle	BP	0.874	PTPN11	down_VSAP_EC
GO:0043400	0.063	1	1	1	cortisol secretion	BP	0.874	PTPN11	down_VSAP_EC
GO:0051462	0.063	1	1	1	regulation of cortisol secretion	BP	0.874	PTPN11	down_VSAP_EC
GO:0051463	0.063	1	1	1	negative regulation of cortisol secretion	BP	0.874	PTPN11	down_VSAP_EC
GO:0060125	0.063	1	1	1	negative regulation of growth hormone	BP	0.874	PTPN11	down_VSAP_EC
GO:0009653	0.0631	0.951	88	773	anatomical structure morphogenesis	BP	1	TNNT2,VEGFD,SMAD6,ACTN2,LEP,AFF3,GATA5,GATA4,CDC7,ANGPT1,EPYC,HTR2B,MBP,ZRANB1,RECK,SHROOM3,FEZ1,MLLT3,BMP4,LNPK,FGFR2,GJA1,ATRX,TLR3,SEC24B,WASF3,LATS2,CITED2,SPINT1,ZNRF3,ZNF365,SASH1,LMO4,TMEM108,FGF18,ARHGEF26,NEBL,ZMYM2,LIMS2,ANKRD1,MARCH5,BNIP3,EGLN1,ENSCAFG00000006238,CLSTN1,NRP1,GPM6A,ULK2,NPNT,UNC5B,SMARCD3,SPINT2,SLITRK5,TBX20,TBX5,FZD3,FRS2,DAG1,APP,LAMA3,PSEN1,TGFBR1,SYNJ2BP,CHD7,JUP,SIPA1L1,PERP,SSH3,C10H2orf49,EDN1,HPGD,ARID5B,SERPINB5,JAM3,TNFRSF11B,GAA,FEZ2,LHX9,WDR48,WASL,DOCK5,ERAP1,BVES,RREB1,CRKL,MYOZ2,PIK3C2A,PTPN14	up_ACOR_VSMC
GO:0097435	0.0631	0.959	30	230	supramolecular fiber organization	BP	1	SYNPO2L,PDLIM3,FGF13,FMOD,ARHGAP28,ACTN2,MID1,SYNPO2,TUBB4A,MFAP4,NEBL,F2RL1,JMY,NRP1,EVL,P4HA1,DES,PRKCE,MYO1B,TTC17,RND1,APP,PSEN1,DDR2,SSH3,EDN1,FAM107A,INPP5K,WASL,MYOZ2	up_ACOR_VSMC
GO:0045859	0.0631	0.96	25	219	regulation of protein kinase activity	BP	0.905	KIT,TNFAIP3,MAP3K11,PDGFB,STK10,RELN,TLR4,EDN1,RALB,NEDD9,MTOR,MAP3K3,BMP2,HHEX,PREX2,SLK,SH3BP5,PROX1,MADD,PAK2,MMD,RAPGEF2,BLM,RIPK1,PDGFD	up_VCAV_EC
GO:0036503	0.0631	0.978	6	47	ERAD pathway	BP	0.874	HM13,HSP90B1,SEL1L,DNAJC10,DNAJB9,USP14	down_VSAP_EC
GO:0080090	0.0631	1	2	1734	regulation of primary metabolic process	BP	1	JUP,GATA5	down_VFEM_VSMC
GO:0009060	0.0631	0.998	1	31	aerobic respiration	BP	1	SUCLG2	down_AFEM_EC
GO:0006551	0.0631	1	1	1	leucine metabolic process	BP	0.905	BCAT1	up_VCAV_EC
GO:0009082	0.0631	1	1	1	branched-chain amino acid biosynthetic process	BP	0.905	BCAT1	up_VCAV_EC
GO:0009098	0.0631	1	1	1	leucine biosynthetic process	BP	0.905	BCAT1	up_VCAV_EC
GO:0009099	0.0631	1	1	1	valine biosynthetic process	BP	0.905	BCAT1	up_VCAV_EC
GO:0045739	0.0631	0.998	1	26	positive regulation of DNA repair	BP	1	EYA1	up_VFEM_VSMC
GO:0070670	0.0632	0.989	3	11	response to interleukin-4	BP	1	SLC29A1,CDK4,CORO1A	down_ACOR_VSMC
GO:0071353	0.0632	0.989	3	11	cellular response to interleukin-4	BP	1	SLC29A1,CDK4,CORO1A	down_ACOR_VSMC
GO:0045835	0.0632	0.994	2	5	negative regulation of meiotic nuclear division	BP	1	FBXO5,TTK	down_ACOR_VSMC

GO:0071559	0.0632	0.998	1	75	response to transforming growth factor beta	BP	1	TGFBR1	down_VSAP_VSMC
GO:0048706	0.0632	0.998	1	59	embryonic skeletal system development	BP	1	HOXA9	up_AORT_VSMC
GO:0051149	0.0632	0.998	1	25	positive regulation of muscle cell differentiation	BP	1	CYP26B1	up_VPOR_VSMC
GO:0021761	0.0632	0.998	1	27	limbic system development	BP	1	EMX2	up_VFEM_VSMC
GO:0003151	0.063262581	0.98722	3	34	outflow tract morphogenesis	BP	1	SMAD6,FOXC2,NRP1	up_ACOR_EC
GO:0009719	0.063291092	0.96486	16	395	response to endogenous stimulus	BP	1	NR1H4,SOX9,FNDC4,AQP1,ENSCAFG00000004331,AGRN,NOD2,RGMA,SMAD6,ACTN2,PTGER4,ADAMTSL2,FOXC2,LRP4,RARRES2,PRKCD	up_ACOR_EC
GO:1902531	0.0633	0.953	63	536	regulation of intracellular signal transduction	BP	1	ADRA1B,LEP,MID1,HTR2B,RASSF2,C28H10orf71,NACC2,PRKAA2,BMP4,FGFR2,ABCA1,CASTOR1,TLR3,SH3RF2,PDE5A,SASH1,FGF18,ARHGEF26,NDRG2,CHN2,ITPR1,ANKRD1,F2RL1,CRLF3,NRP1,UBR1,PTPN22,NPNT,UNC5B,SMPD1,APLP1,SESN3,SH3RF1,PRKCE,FRS2,DAG1,PARP9,APP,PSEN1,SYNJ2BP,SIPA1L1,CCL5,WWC2,DUSP22,SIAH1,EDN1,SESN1,DUSP26,NOD2,LRRC4,DDIT4,SH3RF3,SERINC3,HERPUD1,MAP3K7,INPP5K,BMT2,WFS1,SH3BP4,ENSCAFG00000029553,PARK7,IL18R1,CRKL	up_ACOR_VSMC
GO:0051249	0.0633	0.967	14	107	regulation of lymphocyte activation	BP	0.905	IL7R,TNFAIP3,MEF2C,TLR4,GATA3,PELI1,ARG2,ITPKB,THOC1,NCK1,BCL10,HMGB1,ATP11C,ZEB1	up_VCAV_EC
GO:0031328	0.0633	0.981	5	512	positive regulation of cellular biosynthetic	BP	1	HOXA11,HOXA9,SALL1,PRR16,SHOX2	up_VSAP_VSMC
GO:0003229	0.0633	0.985	4	17	ventricular cardiac muscle tissue development	BP	0.905	ADAMTS9,CHD7,DLL4,PROX1	up_VCAV_EC
GO:0090207	0.0633	0.989	3	11	regulation of triglyceride metabolic process	BP	1	SREBF1,CTDNEP1,ABHD5	down_ACOR_VSMC
GO:0006654	0.0633	0.993	2	7	phosphatidic acid biosynthetic process	BP	0.874	NR1H4,SH3GLB1	down_VSAP_EC
GO:0046473	0.0633	0.993	2	7	phosphatidic acid metabolic process	BP	0.874	NR1H4,SH3GLB1	down_VSAP_EC
GO:0038061	0.0633	0.998	1	34	NIK/NF-kappaB signaling	BP	1	HDAC7	down_VPOR_EC
GO:0006529	0.0633	1	1	1	asparagine biosynthetic process	BP	0.874	ASNS	down_VSAP_EC
GO:0032944	0.0634	0.978	6	48	regulation of mononuclear cell	BP	0.874	CTNNB1,TFRC,LYN,SDC4,BTK,CDKN1A	down_VSAP_EC
GO:0050670	0.0634	0.978	6	48	regulation of lymphocyte proliferation	BP	0.874	CTNNB1,TFRC,LYN,SDC4,BTK,CDKN1A	down_VSAP_EC
GO:0035282	0.0634	0.979	6	30	segmentation	BP	1	MLLT3,BMP4,NRP1,FRS2,PSEN1,PCSK6	up_ACOR_VSMC
GO:0036294	0.0634	0.978	6	37	cellular response to decreased oxygen levels	BP	0.905	CD34,MTOR,PTGS2,PMAIP1,HIF1A,HP1BP3	up_VCAV_EC
GO:0071456	0.0634	0.978	6	37	cellular response to hypoxia	BP	0.905	CD34,MTOR,PTGS2,PMAIP1,HIF1A,HP1BP3	up_VCAV_EC
GO:0016999	0.0634	0.979	6	30	antibiotic metabolic process	BP	1	DLST,ACSS2,ZNF205,IDH1,IDH3A,ENSCAFG00000006639	up_VSAP_EC
GO:0030334	0.0634	0.987	3	267	regulation of cell migration	BP	1	TBX5,CCL24,ARHGEF2	up_VPUL_EC
GO:0072659	0.0634	0.992	2	82	protein localization to plasma membrane	BP	1	ACSL3,STX4	down_ACOR_EC
GO:0032957	0.0634	0.994	2	5	inositol trisphosphate metabolic process	BP	0.905	ITPKB,PLCG2	up_VCAV_EC
GO:0003333	0.0634	0.998	1	12	amino acid transmembrane transport	BP	1	PDPN	down_ACOR_EC
GO:0006622	0.0634	0.998	1	11	protein targeting to lysosome	BP	1	NEDD4	down_ACOR_EC
GO:0010644	0.0634	1	1	1	cell communication by electrical coupling	BP	1	CAV1	down_ACOR_VSMC
GO:0048550	0.0634	1	1	1	negative regulation of pinocytosis	BP	1	CAV1	down_ACOR_VSMC
GO:0072584	0.0634	1	1	1	caveolin-mediated endocytosis	BP	1	CAV1	down_ACOR_VSMC
GO:0075509	0.0634	1	1	1	endocytosis involved in viral entry into host cell	BP	1	CAV1	down_ACOR_VSMC
GO:0086064	0.0634	1	1	1	cell communication by electrical coupling involved in cardiac	BP	1	CAV1	down_ACOR_VSMC
GO:0086098	0.0634	1	1	1	angiotensin-activated signaling pathway	BP	1	CAV1	down_ACOR_VSMC

GO:1901844	0.0634	1	1	1	regulation of cell communication by electrical coupling	BP	1	CAV1	down_ACOR_VSMC
GO:1901979	0.0634	1	1	1	regulation of inward rectifier potassium	BP	1	CAV1	down_ACOR_VSMC
GO:1903596	0.0634	1	1	1	regulation of gap junction assembly	BP	1	CAV1	down_ACOR_VSMC
GO:1903598	0.0634	1	1	1	positive regulation of gap junction assembly	BP	1	CAV1	down_ACOR_VSMC
GO:1903609	0.0634	1	1	1	negative regulation of inward rectifier potassium channel activity	BP	1	CAV1	down_ACOR_VSMC
GO:0010649	0.0634	1	1	1	regulation of cell communication by	BP	1	CAV1	down_ACOR_VSMC
GO:0019065	0.0634	1	1	1	receptor-mediated endocytosis of virus by	BP	1	CAV1	down_ACOR_VSMC
GO:0033484	0.0634	1	1	1	nitric oxide homeostasis	BP	1	CAV1	down_ACOR_VSMC
GO:0044860	0.0634	1	1	1	protein localization to plasma membrane raft	BP	1	CAV1	down_ACOR_VSMC
GO:1900085	0.0634	1	1	1	negative regulation of peptidyl-tyrosine	BP	1	CAV1	down_ACOR_VSMC
GO:0097530	0.0634	0.998	1	33	granulocyte migration	BP	1	SLIT2	up_VPUL_VSMC
GO:0007033	0.063494456	0.97978	5	82	vacuole organization	BP	1	ATG16L2,CHMP4B,TP53INP2,VPS16,ENSCAFG00000010493	up_ACOR_EC
GO:0042490	0.0635	0.988	3	19	mechanoreceptor differentiation	BP	1	SDC4,NAGLU,GSDME	down_VCAV_EC
GO:1900746	0.0635	0.993	2	7	regulation of vascular endothelial growth factor	BP	0.874	JCAD,MYO1C	down_VSAP_EC
GO:1902547	0.0635	0.993	2	7	regulation of cellular response to vascular endothelial growth factor	BP	0.874	JCAD,MYO1C	down_VSAP_EC
GO:0000460	0.0635	0.998	1	15	maturation of 5.8S rRNA	BP	1	WDR12	down_ACOR_EC
GO:0061951	0.0635	0.998	1	16	establishment of protein localization to plasma	BP	1	ACSL3	down_ACOR_EC
GO:0010466	0.0635	0.998	1	32	negative regulation of peptidase activity	BP	1	CST3	down_VPOR_EC
GO:0010951	0.0635	0.998	1	32	negative regulation of endopeptidase activity	BP	1	CST3	down_VPOR_EC
GO:0072525	0.0635	0.998	1	24	pyridine-containing compound biosynthetic	BP	1	FBP1	up_VFEM_VSMC
GO:0045595	0.0636	0.958	31	469	regulation of cell differentiation	BP	1	NOTCH2,VASN,FBN1,GLIPR2,BMPR1A,LMO4,TRPV2,NUMBL,TFE3,CLSTN1,SEMA3C,THY1,TLX2,GLI3,ZFP36L1,RBM38,CDH2,LOXL3,RAB7B,UST,TWIST2,PRKCSH,DDR2,GREM1,NAPEPLD,FOXO3,THRB,ZBTB16,ANKRD54,RGMA,BRSK1	down_VCAV_EC
GO:0060976	0.0636	0.981	5	22	coronary vasculature development	BP	1	SMAD6,BMP4,SEC24B,NRP1,AP2B1	up_ACOR_VSMC
GO:0031086	0.0636	0.994	2	5	nuclear-transcribed mRNA catabolic process, deadenylation-	BP	1	EDC3,EDC4	down_ACOR_VSMC
GO:0031087	0.0636	0.994	2	5	deadenylation-independent decapping of nuclear-transcribed mRNA	BP	1	EDC3,EDC4	down_ACOR_VSMC
GO:0090025	0.0636	0.993	2	7	regulation of monocyte chemotaxis	BP	0.874	SERPINE1,LYN	down_VSAP_EC
GO:0006244	0.0636	1	1	1	pyrimidine nucleotide catabolic process	BP	0.874	ENTPD4	down_VSAP_EC
GO:0006256	0.0636	1	1	1	UDP catabolic process	BP	0.874	ENTPD4	down_VSAP_EC
GO:0009138	0.0636	1	1	1	pyrimidine nucleoside diphosphate metabolic	BP	0.874	ENTPD4	down_VSAP_EC
GO:0009140	0.0636	1	1	1	pyrimidine nucleoside diphosphate catabolic	BP	0.874	ENTPD4	down_VSAP_EC
GO:0009193	0.0636	1	1	1	pyrimidine ribonucleoside diphosphate metabolic	BP	0.874	ENTPD4	down_VSAP_EC
GO:0009195	0.0636	1	1	1	pyrimidine ribonucleoside diphosphate catabolic	BP	0.874	ENTPD4	down_VSAP_EC
GO:0009222	0.0636	1	1	1	pyrimidine ribonucleotide catabolic process	BP	0.874	ENTPD4	down_VSAP_EC
GO:0046048	0.0636	1	1	1	UDP metabolic process	BP	0.874	ENTPD4	down_VSAP_EC
GO:0010944	0.0636	1	1	1	negative regulation of transcription by competitive promoter	BP	0.905	CREB1	up_VCAV_EC
GO:0051289	0.0636	0.998	1	25	protein homotetramerization	BP	1	FBP1	up_VFEM_VSMC

GO:0016042	0.063680064	0.97972	5	80	lipid catabolic process	BP	1	PLCL1,PLCE1,CFI,RARRES2,PRKCD	up_ACOR_EC
GO:0007166	0.0637	0.955	43	686	cell surface receptor signaling pathway	BP	1	NOTCH2,PDGFRB,SMAD9,ITGB5,DCN,NDN,ANGPT1,PRNP,VASN,FBN1,BGN,BMPR1A,EVC,RECK,IGFBP6,NUMBL,ENSCAFG00000014978,GABARAP,AKT1S1,CHRDL1,AKT2,CLSTN1,CSPG4,SEMA3C,ASPN,THY1,ATP6V0C,GLI3,PARK7,SASH1,IFT57,MAVS,CDH2,LOXL3,FUT8,ENSCAFG00000017708,GLIS2,SHC1,GREM1,NPHP4,FOXO3,RGMA,CDK14	down_VCAV_EC
GO:0035264	0.0637	0.972	9	56	multicellular organism growth	BP	0.905	PDE4D,CHD7,MTOR,HHEX,SLC25A25,CREB1,SPTBN4,ATRX,RBBP6	up_VCAV_EC
GO:0003334	0.0637	0.995	2	4	keratinocyte development	BP	1	EXPH5,FOSL2	up_ACOR_VSMC
GO:0002155	0.0637	1	1	1	regulation of thyroid hormone mediated	BP	0.874	PRCP	down_VSAP_EC
GO:0002353	0.0637	1	1	1	plasma kallikrein-kinin cascade	BP	0.874	PRCP	down_VSAP_EC
GO:0002254	0.0637	1	1	1	kinin cascade	BP	0.874	PRCP	down_VSAP_EC
GO:0021953	0.0637	0.998	1	49	central nervous system neuron differentiation	BP	1	HOXC10	up_AFEM_VSMC
GO:0003007	0.0637	0.998	1	94	heart morphogenesis	BP	1	TBX5	up_APUL_VSMC
GO:0021602	0.0637	0.998	1	16	cranial nerve morphogenesis	BP	1	SIX1	up_VFEM_EC
GO:0032663	0.0637	0.998	1	19	regulation of interleukin-2 production	BP	1	NR1H4	up_VPUL_EC
GO:1902679	0.0638	0.957	36	329	negative regulation of RNA biosynthetic process	BP	1	ELK3,HMGB1,SREBF1,ILF3,CBX5,ATAD2,HMGA1,SREBF2,NEDD4,MTDH,NAB2,BIRC5,CAV1,TIMELESS,FAM129B,AURKB,EZH2,GREM1,PLK3,HOXA7,UHRF1,GMNN,NOC2L,PPARD,SUV39H1,HMGB2,DMAP1,CDK2,ENSCAFG00000005299,EZR,ERF,TCERG1,HAT1,HIST2H2AC,H2AFX,SIX4	down_ACOR_VSMC
GO:1903507	0.0638	0.957	36	329	negative regulation of nucleic acid-templated transcription	BP	1	ELK3,HMGB1,SREBF1,ILF3,CBX5,ATAD2,HMGA1,SREBF2,NEDD4,MTDH,NAB2,BIRC5,CAV1,TIMELESS,FAM129B,AURKB,EZH2,GREM1,PLK3,HOXA7,UHRF1,GMNN,NOC2L,PPARD,SUV39H1,HMGB2,DMAP1,CDK2,ENSCAFG00000005299,EZR,ERF,TCERG1,HAT1,HIST2H2AC,H2AFX,SIX4	down_ACOR_VSMC
GO:0001819	0.0638	0.964	17	120	positive regulation of cytokine production	BP	1	TLR1,PLCG2,LEP,CD83,HTR2B,MBP,FERMT1,TLR3,CD274,F2RL1,PTPN22,NOD2,B2M,SCAMP5,MAP3K7,PARK7,IL18R1	up_ACOR_VSMC
GO:0051251	0.0638	0.971	10	69	positive regulation of lymphocyte activation	BP	0.905	IL7R,MEF2C,TLR4,GATA3,PELI1,ITPKB,NCK1,BCL10,HMGB1,ATP11C	up_VCAV_EC
GO:1903038	0.0638	0.978	6	30	negative regulation of leukocyte cell-cell	BP	1	BMP4,PDE5A,CD274,PTPN22,DUSP22,PAG1	up_ACOR_VSMC
GO:0042035	0.0638	0.984	4	20	regulation of cytokine biosynthetic process	BP	0.905	TLR4,GATA3,TLR3,BCL10	up_VCAV_EC
GO:0044092	0.0638	0.987	3	270	negative regulation of molecular function	BP	1	NR1H4,PTGER2,FLRT3	up_VPUL_EC
GO:0046503	0.0638	0.989	3	10	glycerolipid catabolic process	BP	1	LIPC,ENPP2,ABHD16A	up_VSAP_EC
GO:0006282	0.0638	0.998	1	48	regulation of DNA repair	BP	1	EYA1	up_AFEM_VSMC
GO:1901491	0.0638	1	1	1	negative regulation of lymphangiogenesis	BP	0.905	VASH1	up_VCAV_EC
GO:0008344	0.0638	0.998	1	25	adult locomotory behavior	BP	1	CHL1	up_VPOR_VSMC
GO:0003208	0.0638	0.998	1	24	cardiac ventricle morphogenesis	BP	1	NKX2-5	up_VPOR_VSMC
GO:2000026	0.0639	0.978	6	560	regulation of multicellular organismal development	BP	1	ACVRL1,LYN,PDPN,HOXA7,SULF1,ASPN	down_ACOR_EC
GO:0043010	0.0639	0.992	2	96	camera-type eye development	BP	1	IRX5,MAB21L1	up_VSAP_VSMC
GO:0048232	0.0639	0.992	2	95	male gamete generation	BP	1	HOXA11,HOXA9	up_VSAP_VSMC
GO:0046530	0.0639	0.998	1	17	photoreceptor cell differentiation	BP	1	NTRK2	up_APUL_EC
GO:0031128	0.0639	0.998	1	15	developmental induction	BP	1	SIX1	up_VFEM_EC
GO:0038083	0.0639	0.998	1	16	peptidyl-tyrosine autophosphorylation	BP	1	CAV1	up_VSAP_VSMC
GO:0051100	0.064	0.972	9	53	negative regulation of binding	BP	1	ARHGAP28,XIRP1,CLSTN1,LRPAP1,PSEN1,MITD1,B2M,DISC1,PARK7	up_ACOR_VSMC
GO:0035556	0.064	0.979	6	845	intracellular signal transduction	BP	1	NR1H4,STK32B,CCL24,PTGER2,FLRT3,ARHGEF2	up_VPUL_EC

GO:2000311	0.064	0.995	2	4	regulation of AMPA receptor activity	BP	0.905	RELN,MEF2C	up_VCAV_EC
GO:0046717	0.064	0.998	1	17	acid secretion	BP	1	NTRK2	up_APUL_EC
GO:0002699	0.064	0.998	1	46	positive regulation of immune effector process	BP	1	C6	up_VCAV_VSMC
GO:0097006	0.064	0.998	1	19	regulation of plasma lipoprotein particle levels	BP	1	NR1H4	up_VPUL_EC
GO:0009991	0.0641	0.965	16	110	response to extracellular stimulus	BP	1	LEP,PRKAA2,CASTOR1,PPARGC1A,CDKN2B,GABARAPL1,CDKN1A,P2RX7,ULK2,SESN3,PIK3C3,LAMP2,SESN1,FAM107A,BMT2,ZFYVE1	up_ACOR_VSMC
GO:0040017	0.0641	0.973	8	180	positive regulation of locomotion	BP	1	CMKLR1,ANGPT4,TWIST2,LRRC15,BVES,LGR6,CCL5,ADAM17	up_AFEM_EC
GO:0003281	0.0641	0.978	6	30	ventricular septum development	BP	1	FZD1,WNT11,SAV1,TGFBR2,NPRL3,CITED2	up_VSAP_EC
GO:0045730	0.0641	0.993	2	8	respiratory burst	BP	1	CYBA,RAC2	down_VCAV_EC
GO:0051651	0.0641	0.998	1	31	maintenance of location in cell	BP	1	GPSM2	down_AFEM_EC
GO:0000103	0.0641	1	1	1	sulfate assimilation	BP	0.874	PAPSS1	down_VSAP_EC
GO:0034036	0.0641	1	1	1	purine ribonucleoside bisphosphate biosynthetic process	BP	0.874	PAPSS1	down_VSAP_EC
GO:0035508	0.0641	1	1	1	positive regulation of myosin-light-chain-	BP	0.874	PPP1R12A	down_VSAP_EC
GO:0050428	0.0641	1	1	1	3'-phosphoadenosine 5'-phosphosulfate biosynthetic process	BP	0.874	PAPSS1	down_VSAP_EC
GO:0002221	0.0641	0.998	1	46	pattern recognition receptor signaling	BP	1	TLR2	up_AFEM_VSMC
GO:0002758	0.0641	0.998	1	46	innate immune response-activating signal	BP	1	TLR2	up_AFEM_VSMC
GO:0044773	0.0641	0.998	1	28	mitotic DNA damage checkpoint	BP	1	TAOK1	up_AORT_EC
GO:0055001	0.0642	0.998	1	47	muscle cell development	BP	1	BEND2	up_AFEM_VSMC
GO:0038159	0.0642	1	1	1	C-X-C chemokine receptor CXCR4 signaling pathway	BP	0.905	CXCR4	up_VCAV_EC
GO:0038160	0.0642	1	1	1	CXCL12-activated CXCR4 signaling pathway	BP	0.905	CXCR4	up_VCAV_EC
GO:0043271	0.0642	0.998	1	28	negative regulation of ion transport	BP	1	HOXA3	up_VPOR_VSMC
GO:1901880	0.0642	0.998	1	19	negative regulation of protein depolymerization	BP	1	ARHGEF2	up_VPUL_EC
GO:0008593	0.0642	0.998	1	33	regulation of Notch signaling pathway	BP	1	GATA5	up_VPUL_VSMC
GO:0009725	0.064234236	0.97125	9	188	response to hormone	BP	1	NR1H4,AQP1,ENSCAFG00000004331,AGRN,ACTN2,PTGER4,FOXC2,RARRES2,PRKCD	up_ACOR_EC
GO:0021915	0.0643	0.977	6	58	neural tube development	BP	1	SDC4,LMO4,SEMA3C,GLI3,ZFP36L1,IFT57	down_VCAV_EC
GO:0016999	0.0643	0.998	1	30	antibiotic metabolic process	BP	1	SUCLG2	down_AFEM_EC
GO:0021783	0.0643	0.998	1	9	preganglionic parasympathetic fiber	BP	1	SEMA3A	up_VPOR_EC
GO:0070265	0.0643	0.998	1	19	necrotic cell death	BP	1	ARHGEF2	up_VPUL_EC
GO:0048641	0.0643	0.998	1	16	regulation of skeletal muscle tissue	BP	1	SHOX2	up_VSAP_VSMC
GO:0045785	0.0644	0.966	14	107	positive regulation of cell adhesion	BP	0.905	TEK,IL7R,PODXL,FERMT1,GATA3,NEDD9,PPP3CA,ITPKB,PRKD2,NCK1,BCL10,HMGB1,ITGA6,ENSCAFG00000002015	up_VCAV_EC
GO:0055074	0.0644	0.992	2	95	calcium ion homeostasis	BP	1	STIM1,CAV1	up_VSAP_VSMC
GO:0048024	0.0644	0.998	1	30	regulation of mRNA splicing, via spliceosome	BP	1	SRSF6	down_AFEM_EC
GO:0072009	0.0644	0.998	1	48	nephron epithelium development	BP	1	EYA1	up_AFEM_VSMC
GO:0043124	0.0644	0.998	1	17	negative regulation of I-kappaB kinase/NF-kappaB	BP	1	NR1H4	up_APUL_EC
GO:0071539	0.0644	0.998	1	8	protein localization to centrosome	BP	1	HOOK3	up_VPOR_EC
GO:1905508	0.0644	0.998	1	8	protein localization to microtubule organizing	BP	1	HOOK3	up_VPOR_EC
GO:0003179	0.0644	0.998	1	19	heart valve morphogenesis	BP	1	TBX5	up_VPUL_EC

GO:0009967	0.0645	0.956	34	435	positive regulation of signal transduction	BP	0.874	VEGFA,SULF1,NR1H4,JCAD,PTPN11,ILK,SMARCA4,MYO1C,CHSY1,HIF1A,TNIK,PTPN2,NEO1,MTDH,ESM1,ADAM17,MAGED1,CTNNB1,ENSCAFG00000002015,LYN,BNIP3,FAM162A,ATF3,SH3GLB1,HTT,CALM1,DISC1,PAK2,INHBA,NRP1,SH3RF3,MADD,SEC13,MID1	down_VSAP_EC
GO:0051223	0.0645	0.987	3	181	regulation of protein transport	BP	1	LYN,ACSL3,STX4	down_ACOR_EC
GO:0003151	0.0645	0.998	1	34	outflow tract morphogenesis	BP	1	CITED2	down_VPOR_EC
GO:0048701	0.0645	0.998	1	25	embryonic cranial skeleton morphogenesis	BP	1	TBX15	up_VFEM_VSMC
GO:0055017	0.0645	0.998	1	19	cardiac muscle tissue growth	BP	1	TBX5	up_VPUL_EC
GO:2001293	0.0645	1	1	1	malonyl-CoA metabolic process	BP	1	ACACB	up_VSAP_EC
GO:2001295	0.0645	1	1	1	malonyl-CoA biosynthetic process	BP	1	ACACB	up_VSAP_EC
GO:0099536	0.064513878	0.97453	7	133	synaptic signaling	BP	1	CNTNAP4,BRSK1,PLCL1,KCNB1,INSYN1,MAPK8IP2,LRRC4	up_ACOR_EC
GO:0015748	0.06458545	0.99226	2	16	organophosphate ester transport	BP	1	SLC25A42,PRKCD	up_ACOR_EC
GO:0070555	0.0646	0.98	5	29	response to interleukin-1	BP	0.905	SELE,CXCL8,MAPK13,OTUD4,HIF1A	up_VCAV_EC
GO:0016310	0.0646	0.981	5	641	phosphorylation	BP	1	STK32B,CCL24,PTGER2,FLRT3,ARHGEF2	up_VPUL_EC
GO:0030048	0.0646	0.981	5	23	actin filament-based movement	BP	1	EMP2,KCNE3,FRMD6,WIPF1,MYO1B	up_VSAP_EC
GO:0035295	0.0646	0.983	4	356	tube development	BP	1	HOXA11,SALL1,CAV1,SHOX2	up_VSAP_VSMC
GO:0006885	0.0646	0.987	3	39	regulation of pH	BP	1	RAB20,RAB7A,ATP6V0B	up_AFEM_EC
GO:0070302	0.0646	0.992	2	78	regulation of stress-activated protein kinase	BP	1	LYN,IGF1R	down_ACOR_EC
GO:0071300	0.0646	0.998	1	12	cellular response to retinoic acid	BP	1	LYN	down_ACOR_EC
GO:0051092	0.0646	0.998	1	47	positive regulation of NF-kappaB transcription	BP	1	TLR2	up_AFEM_VSMC
GO:0050810	0.0646	0.998	1	17	regulation of steroid biosynthetic process	BP	1	NR1H4	up_APUL_EC
GO:0060485	0.0646	0.998	1	93	mesenchyme development	BP	1	TBX5	up_APUL_VSMC
GO:0007528	0.0646	0.998	1	17	neuromuscular junction development	BP	1	SIX1	up_VFEM_EC
GO:0001959	0.0646	0.998	1	33	regulation of cytokine-mediated signaling	BP	1	SLIT2	up_VPUL_VSMC
GO:0007612	0.0646	0.998	1	33	learning	BP	1	LRRN4	up_VPUL_VSMC
GO:0070838	0.0647	0.97	10	96	divalent metal ion transport	BP	0.874	SLC39A7,ERO1A,ATP2B1,REM1,CTNNB1,LYN,DDIT3,HTT,CALM1,HOMER1	down_VSAP_EC
GO:0001919	0.0647	0.994	2	5	regulation of receptor recycling	BP	1	PSEN1,BVES	up_ACOR_VSMC
GO:0007631	0.0647	0.998	1	17	feeding behavior	BP	1	NTRK2	up_APUL_EC
GO:0010976	0.0647	0.998	1	47	positive regulation of neuron projection	BP	1	LIMK1	up_VCAV_VSMC
GO:0009410	0.0647	0.998	1	27	response to xenobiotic stimulus	BP	1	CYP26B1	up_VPOR_VSMC
GO:0010977	0.0647	0.998	1	33	negative regulation of neuron projection	BP	1	SLIT2	up_VPUL_VSMC
GO:0008654	0.064782766	0.98255	4	58	phospholipid biosynthetic process	BP	1	NR1H4,CHPT1,PIGS,AGPAT2	up_ACOR_EC
GO:0048259	0.0648	0.981	5	24	regulation of receptor-mediated endocytosis	BP	1	DNAJC6,PLCG2,ATXN2,LRPAP1,B2M	up_ACOR_VSMC
GO:0003014	0.0648	0.98	5	27	renal system process	BP	0.905	ADGRF5,PDGFB,CD34,STC1,EDN1	up_VCAV_EC
GO:0048026	0.0648	0.994	2	5	positive regulation of mRNA splicing, via	BP	1	THRAP3,SF3B4	down_ACOR_VSMC
GO:0042311	0.0648	0.993	2	7	vasodilation	BP	0.874	APOE,PLOD3	down_VSAP_EC
GO:0055025	0.0648	0.998	1	17	positive regulation of cardiac muscle tissue	BP	1	TBX5	up_APUL_EC
GO:0031128	0.0648	0.998	1	15	developmental induction	BP	1	HOXA11	up_VSAP_VSMC
GO:0048754	0.064817253	0.98255	4	57	branching morphogenesis of an epithelial tube	BP	1	SOX9,FOXC2,CLSTN1,NRP1	up_ACOR_EC
GO:0009746	0.064850636	0.98675	3	35	response to hexose	BP	1	NR1H4,KCNB1,PRKACA	up_ACOR_EC

GO:0034284	0.064850636	0.98675	3	35	response to monosaccharide	BP	1	NR1H4,KCNB1,PRKACA	up_ACOR_EC
GO:0015672	0.0649	0.967	13	89	monovalent inorganic cation transport	BP	1	FGF13,ACTN2,KCNE3,PTPN3,AKAP6,DMPK,KCNJ4,ATP6V0E1,KCNC4,FXYD1,NDUFA4L2,PARK7,SLC8A1	up_ACOR_VSMC
GO:0009889	0.0649	0.972	10	1190	regulation of biosynthetic process	BP	1	ACVRL1,YEATS2,YBX3,HIVEP2,HOXA7,SULF1,ELK3,HOXA1,ACSL3,NEDD4	down_ACOR_EC
GO:0002573	0.0649	0.973	8	71	myeloid leukocyte differentiation	BP	0.874	VEGFA,MYH9,PTPN2,EVI2B,CTNNB1,TFRC,LYN,GLO1	down_VSAP_EC
GO:0044242	0.0649	0.977	6	57	cellular lipid catabolic process	BP	1	AKT2,ACAA1,PEX5,ENSCAFG00000017708,LONP2,ACAT1	down_VCAV_EC
GO:0071363	0.0649	0.987	3	188	cellular response to growth factor stimulus	BP	1	ACVRL1,SULF1,ASPN	down_ACOR_EC
GO:2000725	0.0649	0.989	3	10	regulation of cardiac muscle cell differentiation	BP	1	BMP4,FRS2,EDN1	up_ACOR_VSMC
GO:0071495	0.0649	0.987	3	365	cellular response to endogenous stimulus	BP	1	FGF5,NKX2-5,BAMBI	up_VPOR_VSMC
GO:0061564	0.0649	0.992	2	119	axon development	BP	1	FLRT3,NEO1	up_VPUL_EC
GO:0048645	0.0649	0.998	1	33	animal organ formation	BP	1	CITED2	down_VPOR_EC
GO:1904716	0.0649	1	1	1	positive regulation of chaperone-mediated	BP	0.874	PLK3	down_VSAP_EC
GO:2000777	0.0649	1	1	1	positive regulation of proteasomal ubiquitin-dependent protein catabolic process involved	BP	0.874	PLK3	down_VSAP_EC
GO:0001659	0.0649	0.998	1	54	temperature homeostasis	BP	1	HOXC10	up_AORT_VSMC
GO:0044106	0.0649	0.998	1	17	cellular amine metabolic process	BP	1	NR1H4	up_APUL_EC
GO:0120163	0.0649	0.998	1	17	negative regulation of cold-induced	BP	1	PLCL1	up_APUL_EC
GO:0014861	0.0649	1	1	1	regulation of skeletal muscle contraction via regulation of action	BP	0.905	KCNJ2	up_VCAV_EC
GO:0100001	0.0649	1	1	1	regulation of skeletal muscle contraction by	BP	0.905	KCNJ2	up_VCAV_EC
GO:0060411	0.0649	0.998	1	33	cardiac septum morphogenesis	BP	1	TBX5	up_VPUL_VSMC
GO:0001937	0.0649	0.998	1	15	negative regulation of endothelial cell	BP	1	CAV1	up_VSAP_VSMC
GO:0071495	0.064959492	0.96453	15	365	cellular response to endogenous stimulus	BP	1	NR1H4,SOX9,AQP1,ENSCAFG00000004331,AGRN,NOD2,RGMA,SMAD6,ACTN2,PTGER4,ADAMTSL2,FOXC2,LRP4,RARRES2,PRKCD	up_ACOR_EC
GO:0031348	0.065	0.975	7	46	negative regulation of defense response	BP	0.905	TNFAIP3,NLRC5,GATA3,ARG2,RIOK3,APOD,CNOT7	up_VCAV_EC
GO:0048515	0.065	0.977	6	36	spermatid differentiation	BP	0.905	KIT,PSME4,NECTIN2,CDYL,AFF4,KDM3A	up_VCAV_EC
GO:0035239	0.065	0.993	2	299	tube morphogenesis	BP	1	C6,TGFBI	up_VCAV_VSMC
GO:0071345	0.065	0.992	2	210	cellular response to cytokine stimulus	BP	1	IL22RA1,SLIT2	up_VPUL_VSMC
GO:0032418	0.065	0.998	1	17	lysosome localization	BP	1	LYN	down_ACOR_EC
GO:0035773	0.065	0.998	1	15	insulin secretion involved in cellular response to glucose stimulus	BP	1	STX4	down_ACOR_EC
GO:0009749	0.065	0.998	1	34	response to glucose	BP	1	RAB11B	down_VPOR_EC
GO:0035265	0.065	0.998	1	54	organ growth	BP	1	ESR1	up_AORT_VSMC
GO:0050803	0.065	0.998	1	55	regulation of synapse structure or activity	BP	1	WNT7A	up_AORT_VSMC
GO:0050807	0.065	0.998	1	55	regulation of synapse organization	BP	1	WNT7A	up_AORT_VSMC
GO:0060384	0.065	0.998	1	9	innervation	BP	1	SEMA3A	up_VPOR_EC
GO:0003407	0.065	0.998	1	15	neural retina development	BP	1	IRX5	up_VSAP_VSMC
GO:0018212	0.0651	0.992	2	81	peptidyl-tyrosine modification	BP	1	LYN,IGF1R	down_ACOR_EC
GO:0048514	0.0651	0.992	2	214	blood vessel morphogenesis	BP	1	HDAC7,CITED2	down_VPOR_EC
GO:0010563	0.0652	0.959	26	203	negative regulation of phosphorus metabolic process	BP	1	SMAD6,RASSF2,BMP4,SH3RF2,LATS2,NDRG2,CDKN2B,F2RL1,FAM129A,CDKN1A,PTPN22,SMPD1,MARF1,DAG1,PPP1R26,PSEN1,SYNJ2BP,DUSP22,DUSP26,LRRC4,DDIT4,INPP5K,PARK7,GPATCH2,ZFYVE1,CRKL	up_ACOR_VSMC

GO:0045936	0.0652	0.959	26	203	negative regulation of phosphate metabolic process	BP	1	SMAD6,RASSF2,BMP4,SH3RF2,LATS2,NDRG2,CDKN2B,F2RL1,FAM129A,CDKN1A,PTPN22,SMPD1,MARF1,DAG1,PPP1R26,PSEN1,SYNJ2BP,DUSP22,DUSP26,LRRC4,DDIT4,INPP5K,PARK7,GPATCH2,ZFYVE1,CRKL	up_ACOR_VSMC
GO:1903530	0.0652	0.96	22	165	regulation of secretion by cell	BP	1	EXPH5,TLR1,LEP,HTR2B,MBP,FBXL20,FERMT1,TM7SF3,CD274,ANKRD1,F2RL1,PTPN22,PRKCE,SCG5,CHD7,CCL5,EDN1,KCNC4,NOD2,SCAMP5,PREPL,PFKL	up_ACOR_VSMC
GO:0007265	0.0652	0.967	12	121	Ras protein signal transduction	BP	0.874	ARHGEF2,FARP1,KPNB1,APOE,PLEKHG2,TAX1BP3,ARFGEF3,PAK2,CDKN1A,NRP1,TNFAIP1,MADD	down_VSAP_EC
GO:0070304	0.0652	0.972	9	58	positive regulation of stress-activated protein kinase signaling cascade	BP	0.905	MAP3K11,TLR4,EDN1,MAP3K3,BMP2,TLR3,TAOK1,HMGB1,RIPK1	up_VCAV_EC
GO:0008344	0.0652	0.98	5	25	adult locomotory behavior	BP	0.905	CHD7,HOXB8,TMOD1,PREX2,SPTBN4	up_VCAV_EC
GO:0048144	0.0652	0.981	5	23	fibroblast proliferation	BP	1	PEX2,MORC3,DDR2,MYC,FBXO4	up_VSAP_EC
GO:0048145	0.0652	0.981	5	23	regulation of fibroblast proliferation	BP	1	PEX2,MORC3,DDR2,MYC,FBXO4	up_VSAP_EC
GO:0031326	0.0652	0.991	3	1176	regulation of cellular biosynthetic process	BP	1	GATA5,TGFBR1,MEIS2	down_VSAP_VSMC
GO:0034123	0.0652	0.989	3	10	positive regulation of toll-like receptor signaling	BP	1	F2RL1,CYBA,FLOT1	up_VSAP_EC
GO:0010867	0.0652	0.994	2	5	positive regulation of triglyceride biosynthetic	BP	1	SREBF1,CTDNEP1	down_ACOR_VSMC
GO:0043552	0.0652	0.998	1	11	positive regulation of phosphatidylinositol 3-kinase activity	BP	1	LYN	down_ACOR_EC
GO:0090218	0.0652	0.998	1	11	positive regulation of lipid kinase activity	BP	1	LYN	down_ACOR_EC
GO:1901863	0.0652	0.998	1	25	positive regulation of muscle tissue	BP	1	CYP26B1	up_VPOR_VSMC
GO:0097178	0.0652	0.998	1	14	ruffle assembly	BP	1	CAV1	up_VSAP_VSMC
GO:0035107	0.0653	0.97	10	60	appendage morphogenesis	BP	1	AFF3,RECK,BMP4,LNPK,FGFR2,GJA1,ATRX,TBX5,PSEN1,CHD7	up_ACOR_VSMC
GO:0035108	0.0653	0.97	10	60	limb morphogenesis	BP	1	AFF3,RECK,BMP4,LNPK,FGFR2,GJA1,ATRX,TBX5,PSEN1,CHD7	up_ACOR_VSMC
GO:0046883	0.0653	0.977	6	49	regulation of hormone secretion	BP	0.874	NR1H4,PTPN11,HIF1A,REST,RAB11FIP1,INHBA	down_VSAP_EC
GO:0048659	0.0653	0.977	6	36	smooth muscle cell proliferation	BP	0.905	CALCRL,TNFAIP3,PDGFB,MEF2C,EDN1,APOD	up_VCAV_EC
GO:0070167	0.0653	0.98	5	29	regulation of biomineral tissue development	BP	0.905	MEF2C,BMP2,BMP2K,HIF1A,FBXO5	up_VCAV_EC
GO:0050867	0.0653	0.992	2	83	positive regulation of cell activation	BP	1	PDPN,STX4	down_ACOR_EC
GO:0070838	0.0653	0.992	2	96	divalent metal ion transport	BP	1	STIM1,CAV1	up_VSAP_VSMC
GO:0070489	0.0653	1	1	1	T cell aggregation	BP	0.874	MSN	down_VSAP_EC
GO:0106106	0.0653	0.998	1	48	cold-induced thermogenesis	BP	1	HOXC10	up_AFEM_VSMC
GO:0120161	0.0653	0.998	1	48	regulation of cold-induced thermogenesis	BP	1	HOXC10	up_AFEM_VSMC
GO:1905207	0.0653	0.998	1	17	regulation of cardiocyte differentiation	BP	1	TBX5	up_APUL_EC
GO:0009066	0.065354241	0.99212	2	16	aspartate family amino acid metabolic process	BP	1	NMNAT2,NIT2	up_ACOR_EC
GO:0050921	0.0654	0.987	3	39	positive regulation of chemotaxis	BP	1	CMKLR1,CCL5,ADAM17	up_AFEM_EC
GO:1902105	0.0654	0.992	2	80	regulation of leukocyte differentiation	BP	1	LYN,HOXA7	down_ACOR_EC
GO:0017144	0.0654	0.992	2	199	drug metabolic process	BP	1	SUCLG2,NDUFA10	down_AFEM_EC
GO:0048485	0.0654	0.998	1	9	sympathetic nervous system development	BP	1	SEMA3A	up_VPOR_EC
GO:0042326	0.06549206	0.97217	8	163	negative regulation of phosphorylation	BP	1	PKIB,NDRG2,DUSP26,SMAD6,LRRC15,LRRC4,PRKCD,DBNDD2	up_ACOR_EC
GO:0010558	0.0655	0.954	42	392	negative regulation of macromolecule biosynthetic process	BP	1	ELK3,HMGB1,SREBF1,ILF3,CBX5,ATAD2,HMGA1,EIF4A3,SREBF2,SMC1A,NEDD4,MTDH,NAB2,BIRC5,CAV1,TIMELESS,FAM129B,AURKB,EZH2,GREM1,PLK3,HOXA7,UHRF1,GMNN,NOC2L,PPARD,NCL,SUV39H1,HMGB2,DMAP1,SMC3,CDK2,ENSCAFG00000005299,EZR,RBM14,ERF,TCERG1,HAT1,HIST2H2AC,TINF2,H2AFX,SIX4	down_ACOR_VSMC

GO:0031098	0.0655	0.964	16	109	stress-activated protein kinase signaling cascade	BP	1	LEP,MAP3K8,MID1,RASSF2,TLR3,SH3RF2,SASH1,F2RL1,PTPN22,SH3RF1,APP,DUSP22,EDN1,NOD2,SH3RF3,MAP3K7	up_ACOR_VSMC
GO:0062013	0.0655	0.983	4	27	positive regulation of small molecule metabolic	BP	0.874	NR1H4,PTGS2,SLC45A3,PTPN2	down_VSAP_EC
GO:0001775	0.0655	0.987	3	219	cell activation	BP	1	HOXA9,TLR2,CAV1	up_VSAP_VSMC
GO:0007000	0.0655	0.994	2	5	nucleolus organization	BP	1	NOLC1,EMG1	down_ACOR_VSMC
GO:0002688	0.0655	0.998	1	34	regulation of leukocyte chemotaxis	BP	1	SLIT2	up_VPUL_VSMC
GO:0048771	0.0656	0.975	7	43	tissue remodeling	BP	0.905	MEF2C,CHD7,DLL4,PPP3CA,JAG1,HMGB1,HIF1A	up_VCAV_EC
GO:2000104	0.0656	0.988	3	11	negative regulation of DNA-dependent DNA	BP	1	SMC1A,GMNN,SMC3	down_ACOR_VSMC
GO:0001893	0.0656	0.993	2	8	maternal placenta development	BP	0.874	TMED2,PTGS2	down_VSAP_EC
GO:0086005	0.0656	0.994	2	5	ventricular cardiac muscle cell action potential	BP	1	KCNE3,JUP	up_ACOR_VSMC
GO:0035759	0.0656	1	1	1	mesangial cell-matrix adhesion	BP	0.905	CD34	up_VCAV_EC
GO:1900035	0.0656	1	1	1	negative regulation of cellular response to heat	BP	0.905	CD34	up_VCAV_EC
GO:0030260	0.0656	0.998	1	15	entry into host cell	BP	1	CAV1	up_VSAP_VSMC
GO:0044409	0.0656	0.998	1	15	entry into host	BP	1	CAV1	up_VSAP_VSMC
GO:0051806	0.0656	0.998	1	15	entry into cell of other organism involved in	BP	1	CAV1	up_VSAP_VSMC
GO:0051828	0.0656	0.998	1	15	entry into other organism involved in symbiotic	BP	1	CAV1	up_VSAP_VSMC
GO:0018193	0.0657	0.954	39	368	peptidyl-amino acid modification	BP	0.905	PLK2,HCLS1,KIT,PRKCH,PDGFB,RELN,ST6GAL1,PDE4D,IWS1,GATA3,NEDD9,YES1,MTOR,HMG20A,SH3BP5,ST3GAL6,MAPK13,ARID4B,PAK2,PRKD2,NCK1,ESCO1,DPH5,FES,NSD1,PTGS2,TOP1,SENP6,CNOT7,NOS2,SPTBN4,RIPK1,PDGFD,BMX,KDM3A,LMTK2,ATRX,TET2,ARHGEF2	up_VCAV_EC
GO:0043409	0.0657	0.971	9	54	negative regulation of MAPK cascade	BP	1	BMP4,NDRG2,F2RL1,PTPN22,SMPD1,DAG1,SYNJ2BP,DUSP26,INPP5K	up_ACOR_VSMC
GO:0006633	0.0657	0.975	7	38	fatty acid biosynthetic process	BP	1	ACSM3,LIPC,INSIG2,ACACB,HSD17B8,PTGES,HACD4	up_VSAP_EC
GO:0034446	0.0657	0.98	5	23	substrate adhesion-dependent cell spreading	BP	1	NRP1,DOCK5,BVES,RREB1,CRKL	up_ACOR_VSMC
GO:0090114	0.0657	0.987	3	15	COPII-coated vesicle budding	BP	0.874	SEC24D,SEC23A,SEC13	down_VSAP_EC
GO:1902175	0.0657	0.993	2	7	regulation of oxidative stress-induced intrinsic apoptotic signaling	BP	0.874	P4HB,HIF1A	down_VSAP_EC
GO:1902667	0.0657	0.994	2	5	regulation of axon guidance	BP	1	SLIT2,PLXNA4	up_VSAP_EC
GO:0055074	0.0657	0.998	1	95	calcium ion homeostasis	BP	1	HOXA3	up_APUL_VSMC
GO:0010830	0.0657	0.998	1	16	regulation of myotube differentiation	BP	1	CYP26B1	up_VFEM_EC
GO:0001960	0.0657	0.998	1	15	negative regulation of cytokine-mediated	BP	1	CAV1	up_VSAP_VSMC
GO:0060761	0.0657	0.998	1	15	negative regulation of response to cytokine	BP	1	CAV1	up_VSAP_VSMC
GO:0060326	0.065773283	0.97883	5	84	cell chemotaxis	BP	1	MMP28,ELMO2,RARRES2,NRP1,PRKCD	up_ACOR_EC
GO:0001841	0.0658	0.973	8	44	neural tube formation	BP	1	SHROOM3,BMP4,SEC24B,CITED2,SPINT1,LMO4,SPINT2,FZD3	up_ACOR_VSMC
GO:0010972	0.0658	0.984	4	18	negative regulation of G2/M transition of mitotic	BP	1	CDK1,FANCI,TOPBP1,CLSPN	down_ACOR_VSMC
GO:0070228	0.0658	0.983	4	20	regulation of lymphocyte apoptotic process	BP	0.905	IL7R,ARG2,BCL10,HIF1A	up_VCAV_EC
GO:0050927	0.0658	0.994	2	5	positive regulation of positive chemotaxis	BP	1	VEGFD,F2RL1	up_ACOR_VSMC
GO:0001503	0.0658	0.992	2	109	ossification	BP	1	HOXA3,NPNT	up_APUL_EC
GO:0030204	0.0658	0.998	1	14	chondroitin sulfate metabolic process	BP	1	CSGALNACT1	down_ACOR_EC
GO:0071109	0.0658	1	1	1	superior temporal gyrus development	BP	0.874	GSK3B	down_VSAP_EC
GO:1905319	0.0658	1	1	1	mesenchymal stem cell migration	BP	0.905	FBXO5	up_VCAV_EC
GO:1905320	0.0658	1	1	1	regulation of mesenchymal stem cell	BP	0.905	FBXO5	up_VCAV_EC

GO:1905322	0.0658	1	1	1	positive regulation of mesenchymal stem cell	BP	0.905	FBXO5	up_VCAV_EC
GO:0007056	0.0658	1	1	1	spindle assembly involved in female meiosis	BP	0.905	FBXO5	up_VCAV_EC
GO:0007057	0.0658	1	1	1	spindle assembly involved in female meiosis I	BP	0.905	FBXO5	up_VCAV_EC
GO:0007399	0.0659	0.955	36	564	nervous system development	BP	1	SMAD9,LRP1,NAB2,NDN,SDC4,BMPR1A,LMO4,TRPV2,NUMBL,NAGLU,AKT2,ENSCAFG00000018277,CSPG4,SEMA3C,THY1,HAPLN3,PEX5,TLX2,VCAN,GLI3,ZFP36L1,NCDN,IFT57,CDH2,LOXL3,GSDME,CAMK2D,ENSCAFG00000017708,IRX5,UST,PRKCSH,FOXO3,THRB,ZBTB16,RGMA,BRSK1	down_VCAV_EC
GO:0006607	0.0659	0.994	2	5	NLS-bearing protein import into nucleus	BP	1	ENSCAFG00000011598,IPO4	down_ACOR_VSMC
GO:0060740	0.0659	0.998	1	13	prostate gland epithelium morphogenesis	BP	1	SULF1	down_ACOR_EC
GO:1903674	0.0659	1	1	1	regulation of cap-dependent translational	BP	0.905	NCK1	up_VCAV_EC
GO:1903676	0.0659	1	1	1	positive regulation of cap-dependent translational	BP	0.905	NCK1	up_VCAV_EC
GO:0019319	0.0659	0.998	1	26	hexose biosynthetic process	BP	1	FBP1	up_VFEM_VSMC
GO:0048546	0.0659	0.998	1	16	digestive tract morphogenesis	BP	1	SHOX2	up_VSAP_VSMC
GO:0098900	0.0659	0.998	1	15	regulation of action potential	BP	1	CAV1	up_VSAP_VSMC
GO:0003215	0.066	0.993	2	9	cardiac right ventricle morphogenesis	BP	1	BMPR1A,SEMA3C	down_VCAV_EC
GO:0061912	0.066	0.992	2	18	selective autophagy	BP	1	CLEC16A,RAB7A	up_AFEM_EC
GO:0019369	0.066	0.994	2	5	arachidonic acid metabolic process	BP	0.905	PTGS1,PTGS2	up_VCAV_EC
GO:0070252	0.066	0.998	1	17	actin-mediated cell contraction	BP	1	PDPN	down_ACOR_EC
GO:2000242	0.066	0.998	1	15	negative regulation of reproductive process	BP	1	SULF1	down_ACOR_EC
GO:1903707	0.066	0.998	1	48	negative regulation of hemopoiesis	BP	1	HOXA9	up_AFEM_VSMC
GO:0060395	0.066	0.998	1	27	SMAD protein signal transduction	BP	1	LNPEP	up_AORT_EC
GO:0048754	0.066	0.998	1	57	branching morphogenesis of an epithelial tube	BP	1	ESR1	up_AORT_VSMC
GO:0007422	0.066	0.998	1	18	peripheral nervous system development	BP	1	NTRK2	up_APUL_EC
GO:0060415	0.066	0.998	1	27	muscle tissue morphogenesis	BP	1	NKX2-5	up_VPOR_VSMC
GO:0023014	0.066031952	0.96763	11	248	signal transduction by protein phosphorylation	BP	1	SOX9,NDRG2,FZD5,DUSP26,NOD2,PAK3,TNFRSF19,MAPK8IP2,SH3RF3,NRP1,PRKCD	up_ACOR_EC
GO:0140056	0.0661	0.98	5	28	organelle localization by membrane tethering	BP	0.905	EXOC6,CAV2,PDZD8,EXOC5,STX11	up_VCAV_EC
GO:0065002	0.0661	0.983	4	30	intracellular protein transmembrane transport	BP	1	AKT2,PEX5,SEC61B,LONP2	down_VCAV_EC
GO:0071806	0.0661	0.983	4	30	protein transmembrane transport	BP	1	AKT2,PEX5,SEC61B,LONP2	down_VCAV_EC
GO:0010647	0.0661	1	1	460	positive regulation of cell communication	BP	1	GATA5	down_AFEM_VSMC
GO:0032272	0.0661	0.998	1	18	negative regulation of protein polymerization	BP	1	TUBB4A	up_APUL_EC
GO:0010758	0.0661	0.998	1	8	regulation of macrophage chemotaxis	BP	1	C3AR1	up_VPOR_EC
GO:0060415	0.0661	0.998	1	27	muscle tissue morphogenesis	BP	1	SHOX2	up_VFEM_VSMC
GO:0046426	0.0661	0.998	1	20	negative regulation of JAK-STAT cascade	BP	1	FLRT3	up_VPUL_EC
GO:1904893	0.0661	0.998	1	20	negative regulation of STAT cascade	BP	1	FLRT3	up_VPUL_EC
GO:0032270	0.0662	0.954	37	479	positive regulation of cellular protein metabolic process	BP	0.874	ARHGEF2,PRICKLE1,VEGFA,MYH9,PLK3,HSPA5,SOCS5,JCAD,BCAR3,PTPN11,TRIB3,ILK,PPP1R12A,PTGS2,MSN,TNIK,YES1,ADAM17,MAGED1,CTNNB1,APOE,LYN,FAM162A,MMD,GSK3B,VLDLR,REST,CALM1,DISC1,RAD23A,PAK2,INHBA,TMTC3,NRP1,SH3RF3,MADD,MID1	down_VSAP_EC
GO:0007283	0.0662	0.967	12	91	spermatogenesis	BP	0.905	KIT,TSPAN8,PSME4,NECTIN2,CDYL,CREM,ARID4B,HIST1H1A,AFF4,KDM3A,ATRX,SPATA6	up_VCAV_EC
GO:0002695	0.0662	0.979	5	44	negative regulation of leukocyte activation	BP	1	PRNP,SDC4,GLI3,BPI,LOXL3	down_VCAV_EC

GO:0009132	0.0662	0.998	1	25	nucleoside diphosphate metabolic process	BP	1	FBP1	up_VFEM_VSMC
GO:0110110	0.0662	0.998	1	34	positive regulation of animal organ	BP	1	GATA5	up_VPUL_VSMC
GO:0031401	0.0663	0.954	38	358	positive regulation of protein modification process	BP	0.905	TEK,HCLS1,KIT,PPP2R5A,MAP3K11,TNIP1,PDGFB,STK10,RELN,TLR4,GATA3,EDN1,RALB,APLNR,NEDD9,PELI1,YES1,MTOR,MAP3K3,BMP2,SLK,PROX1,MADD,PAK2,PRKD2,TLR3,PTGS2,MMD,RAPGEF2,BCL10,TAOK1,HMGB1,RIPK1,PDGFD,UBE2D1,JCAD,ARHGEF2,CDON	up_VCAV_EC
GO:0060337	0.0663	0.988	3	12	type I interferon signaling pathway	BP	0.905	NLRC5,CNOT7,LSM14A	up_VCAV_EC
GO:0071357	0.0663	0.988	3	12	cellular response to type I interferon	BP	0.905	NLRC5,CNOT7,LSM14A	up_VCAV_EC
GO:0002697	0.0663	0.992	2	85	regulation of immune effector process	BP	1	LYN,STX4	down_ACOR_EC
GO:0001654	0.0663	0.992	2	110	eye development	BP	1	ZDHHC16,NTRK2	up_APUL_EC
GO:0048880	0.0663	0.992	2	110	sensory system development	BP	1	ZDHHC16,NTRK2	up_APUL_EC
GO:0150063	0.0663	0.992	2	110	visual system development	BP	1	ZDHHC16,NTRK2	up_APUL_EC
GO:0090085	0.0663	0.994	2	5	regulation of protein deubiquitination	BP	0.905	TNIP1,OTUD4	up_VCAV_EC
GO:0072511	0.0663	0.992	2	97	divalent inorganic cation transport	BP	1	STIM1,CAV1	up_VSAP_VSMC
GO:0035136	0.0663	0.998	1	16	forelimb morphogenesis	BP	1	HOXA9	up_VFEM_EC
GO:0048538	0.0663	0.998	1	20	thymus development	BP	1	HOXA3	up_VPUL_EC
GO:0048518	0.0664	0.984	5	1676	positive regulation of biological process	BP	1	SLITRK5,LIMK1,GATA5,CA2,C6	up_VCAV_VSMC
GO:0006576	0.0664	0.988	3	10	cellular biogenic amine metabolic process	BP	1	NAPEPLD,AZIN2,IDO2	up_VSAP_EC
GO:0001914	0.0664	0.993	2	8	regulation of T cell mediated cytotoxicity	BP	0.874	FAM49B,PVR	down_VSAP_EC
GO:0023056	0.0664	1	1	462	positive regulation of signaling	BP	1	GATA5	down_AFEM_VSMC
GO:0050679	0.0664	0.998	1	62	positive regulation of epithelial cell proliferation	BP	1	WNT7A	up_AORT_VSMC
GO:0051271	0.0664	0.998	1	98	negative regulation of cellular component	BP	1	TBX5	up_APUL_VSMC
GO:0009308	0.0664	0.998	1	20	amine metabolic process	BP	1	NR1H4	up_VPUL_EC
GO:0003156	0.0664	0.998	1	20	regulation of animal organ formation	BP	1	TBX5	up_VPUL_EC
GO:0060760	0.0664	0.998	1	17	positive regulation of response to cytokine	BP	1	TLR2	up_VSAP_VSMC
GO:1901020	0.0665	0.993	2	8	negative regulation of calcium ion transmembrane	BP	0.874	REM1,CALM1	down_VSAP_EC
GO:1903170	0.0665	0.993	2	8	negative regulation of calcium ion	BP	0.874	REM1,CALM1	down_VSAP_EC
GO:0007422	0.0665	0.992	2	18	peripheral nervous system development	BP	1	PLXNA4,LGI4	up_AFEM_EC
GO:0009062	0.0665	0.998	1	31	fatty acid catabolic process	BP	1	ETFA	down_AFEM_EC
GO:0048532	0.0665	0.998	1	9	anatomical structure arrangement	BP	1	SEMA3A	up_VPOR_EC
GO:0006090	0.0665	0.998	1	26	pyruvate metabolic process	BP	1	FBP1	up_VFEM_VSMC
GO:0048659	0.066506337	0.98625	3	36	smooth muscle cell proliferation	BP	1	NDRG2,PTGIR,CLSTN1	up_ACOR_EC
GO:0009792	0.0666	0.958	26	231	embryo development ending in birth or egg hatching	BP	0.905	HOXB7,PCDH12,ADGRF5,TIE1,AMOT,PDGFB,MEF2C,CHD7,GATA3,EDN1,HOXB8,SOX18,BMP2,HHEX,MDFI,HOXB5,BCL10,NUP133,SKIL,CUL4A,HIF1A,LUZP1,RBBP6,VASH1,ZEB1,GINS4	up_VCAV_EC
GO:0006066	0.0666	0.967	12	89	alcohol metabolic process	BP	1	SQLE,LSS,SREBF1,HMGCR,INSIG1,DHCR24,MVD,FDFT1,LIPC,NUS1,GK5,NUDT3	down_ACOR_VSMC
GO:0045861	0.0666	0.974	7	71	negative regulation of proteolysis	BP	1	CST3,RECK,TIMP2,PSMF1,PARK7,CLEC16A,A2M	down_VCAV_EC
GO:0010467	0.0666	1	2	1787	gene expression	BP	1	JUP,GATA5	down_VFEM_VSMC
GO:0050854	0.0666	0.998	1	16	regulation of antigen receptor-mediated	BP	1	LYN	down_ACOR_EC
GO:1903707	0.0666	0.998	1	48	negative regulation of hemopoiesis	BP	1	LRRC17	up_VCAV_VSMC

GO:0042733	0.0666	0.998	1	27	embryonic digit morphogenesis	BP	1	HOXA11	up_VFEM_VSMC
GO:1900542	0.0666	0.998	1	26	regulation of purine nucleotide metabolic	BP	1	FBP1	up_VFEM_VSMC
GO:0018209	0.0667	0.963	16	125	peptidyl-serine modification	BP	0.905	PLK2,HCLS1,PRKCH,PDGFB,PDE4D,MTOR,MAPK13,PAK2,PRKD2,NCK1,NSD1,PTGS2,TOP1,SPTBN4,RIPK1,LMTK2	up_VCAV_EC
GO:0006006	0.0667	0.974	7	61	glucose metabolic process	BP	0.874	ENPP1,TPI1,PGAM1,SESN2,SLC45A3,PTPN2,ATF3	down_VSAP_EC
GO:0034763	0.0667	0.98	5	27	negative regulation of transmembrane transport	BP	1	NEDD4,CAV1,SLC30A1,GSTO1,REM1	down_ACOR_VSMC
GO:0033619	0.0667	0.987	3	19	membrane protein proteolysis	BP	1	TIMP2,HM13,PACSIN3	down_VCAV_EC
GO:0032615	0.0667	0.992	2	18	interleukin-12 production	BP	1	CMKLR1,LEP	up_AFEM_EC
GO:0072503	0.0667	0.992	2	97	cellular divalent inorganic cation homeostasis	BP	1	STIM1,CAV1	up_VSAP_VSMC
GO:0071544	0.0667	1	1	1	diphosphoinositol polyphosphate catabolic	BP	1	NUDT3	down_ACOR_VSMC
GO:0051050	0.0667	0.999	1	235	positive regulation of transport	BP	1	JUP	down_VFEM_VSMC
GO:0002339	0.0667	1	1	1	B cell selection	BP	0.874	BTK	down_VSAP_EC
GO:0002343	0.0667	1	1	1	peripheral B cell selection	BP	0.874	BTK	down_VSAP_EC
GO:0002344	0.0667	1	1	1	B cell affinity maturation	BP	0.874	BTK	down_VSAP_EC
GO:0098760	0.0667	1	1	1	response to interleukin-7	BP	0.874	BTK	down_VSAP_EC
GO:0098761	0.0667	1	1	1	cellular response to interleukin-7	BP	0.874	BTK	down_VSAP_EC
GO:0060420	0.0667	0.998	1	18	regulation of heart growth	BP	1	TBX5	up_APUL_EC
GO:0070838	0.0667	0.998	1	96	divalent metal ion transport	BP	1	HOXA3	up_APUL_VSMC
GO:2000145	0.066785918	0.96609	12	279	regulation of cell motility	BP	1	SOX9,AQP1,SEMA6D,ZMYND8,MMP28,PTGER4,ARHGDIB,FOXC2,LRRC15,TBX5,RARRES2,NRP1	up_ACOR_EC
GO:0032943	0.0668	0.974	7	60	mononuclear cell proliferation	BP	0.874	MSN,CTNNB1,TFRC,LYN,SDC4,BTK,CDKN1A	down_VSAP_EC
GO:0046651	0.0668	0.974	7	60	lymphocyte proliferation	BP	0.874	MSN,CTNNB1,TFRC,LYN,SDC4,BTK,CDKN1A	down_VSAP_EC
GO:0032990	0.0668	0.986	3	167	cell part morphogenesis	BP	1	SPG11,PDPN,NRP2	down_ACOR_EC
GO:0061383	0.0668	0.987	3	20	trabecula morphogenesis	BP	1	THBS3,BMPR1A,GREM1	down_VCAV_EC
GO:0016601	0.0668	0.988	3	11	Rac protein signal transduction	BP	0.905	ARAP3,NCKAP1,ELMO1	up_VCAV_EC
GO:0002009	0.0668	0.992	2	177	morphogenesis of an epithelium	BP	1	EYA1,HOXA11	up_VFEM_VSMC
GO:0007186	0.0668	0.992	2	124	G protein-coupled receptor signaling	BP	1	RGS1,CCL24	up_VPUL_EC
GO:0006637	0.0668	0.998	1	32	acyl-CoA metabolic process	BP	1	SUCLG2	down_AFEM_EC
GO:0035383	0.0668	0.998	1	32	thioester metabolic process	BP	1	SUCLG2	down_AFEM_EC
GO:0008589	0.0668	0.998	1	28	regulation of smoothened signaling pathway	BP	1	SHOX2	up_VFEM_VSMC
GO:0050701	0.066823181	0.99184	2	16	interleukin-1 secretion	BP	1	FZD5,NOD2	up_ACOR_EC
GO:0060255	0.0669	1	2	1782	regulation of macromolecule metabolic	BP	1	JUP,GATA5	down_VFEM_VSMC
GO:0043266	0.0669	0.998	1	14	regulation of potassium ion transport	BP	1	NEDD4	down_ACOR_EC
GO:0002244	0.0669	0.998	1	35	hematopoietic progenitor cell differentiation	BP	1	CITED2	down_VPOR_EC
GO:0009746	0.0669	0.998	1	35	response to hexose	BP	1	RAB11B	down_VPOR_EC
GO:0034284	0.0669	0.998	1	35	response to monosaccharide	BP	1	RAB11B	down_VPOR_EC
GO:0016333	0.0669	1	1	1	morphogenesis of follicular epithelium	BP	0.874	WDR1	down_VSAP_EC
GO:0016334	0.0669	1	1	1	establishment or maintenance of polarity	BP	0.874	WDR1	down_VSAP_EC
GO:0042247	0.0669	1	1	1	establishment of planar polarity of follicular	BP	0.874	WDR1	down_VSAP_EC
GO:0048839	0.0669	0.998	1	50	inner ear development	BP	1	EYA1	up_AFEM_VSMC
GO:0032943	0.0669	0.998	1	60	mononuclear cell proliferation	BP	1	ARG2	up_AORT_VSMC

GO:0046651	0.0669	0.998	1	60	lymphocyte proliferation	BP	1	ARG2	up_AORT_VSMC
GO:0002218	0.067	0.971	9	53	activation of innate immune response	BP	1	F2RL1,MYD88,NLRX1,TKFC,CYBA,PQBP1,UNC93B1,FLOT1,MAVS	up_VSAP_EC
GO:0051961	0.067	0.974	7	73	negative regulation of nervous system	BP	1	BMPR1A,THY1,TLX2,GLI3,PRKCSH,THRB,RGMA	down_VCAV_EC
GO:0051785	0.067	0.987	3	14	positive regulation of nuclear division	BP	0.905	PDGFB,EDN1,SMC5	up_VCAV_EC
GO:0051482	0.067	0.994	2	5	positive regulation of cytosolic calcium ion concentration involved in phospholipase C-activating G protein-	BP	1	F2RL1,EDN1	up_ACOR_VSMC
GO:0002548	0.067	0.998	1	17	monocyte chemotaxis	BP	1	LYN	down_ACOR_EC
GO:0048597	0.067	1	1	1	post-embryonic camera-type eye morphogenesis	BP	1	HMGN1	down_ACOR_VSMC
GO:0030041	0.0671	0.97	9	62	actin filament polymerization	BP	0.905	HCLS1,BAIAP2L1,TMOD1,MTOR,NCKAP1,IQGAP2,NCK1,ARPC5L,TMOD3	up_VCAV_EC
GO:0046661	0.0671	0.972	8	45	male sex differentiation	BP	1	GATA4,PRDX4,ATRX,CITED2,SPINK2,ARID5B,LHX9,WDR48	up_ACOR_VSMC
GO:0048870	0.0671	0.983	4	451	cell motility	BP	1	TBX5,CCL24,FLRT3,ARHGEF2	up_VPUL_EC
GO:0051674	0.0671	0.983	4	451	localization of cell	BP	1	TBX5,CCL24,FLRT3,ARHGEF2	up_VPUL_EC
GO:0007167	0.0671	0.986	3	274	enzyme linked receptor protein signaling pathway	BP	1	NR1H4,FLRT3,NEO1	up_VPUL_EC
GO:0031323	0.0671	1	2	1788	regulation of cellular metabolic process	BP	1	JUP,GATA5	down_VFEM_VSMC
GO:0001525	0.0671	0.992	2	173	angiogenesis	BP	1	HOXA3,C3AR1	up_VPOR_VSMC
GO:0021577	0.0671	1	1	1	hindbrain structural organization	BP	0.874	HSPA5	down_VSAP_EC
GO:0021589	0.0671	1	1	1	cerebellum structural organization	BP	0.874	HSPA5	down_VSAP_EC
GO:0006814	0.0671	0.998	1	28	sodium ion transport	BP	1	NKX2-5	up_VPOR_VSMC
GO:0046324	0.0671	0.998	1	20	regulation of glucose import	BP	1	NR1H4	up_VPUL_EC
GO:0001656	0.067115262	0.98609	3	35	metanephros development	BP	1	SOX9,AQP1,FOXC2	up_ACOR_EC
GO:0071229	0.0672	0.971	9	51	cellular response to acid chemical	BP	1	PRKAA2,ABCA1,CASTOR1,ADCY6,UBR1,PTGDR,SESN3,PRKCE,SH3BP4	up_ACOR_VSMC
GO:2000146	0.0672	0.978	5	92	negative regulation of cell motility	BP	1	ANGPT4,HOXA7,RECK,TBXA2R,KANK1	up_AFEM_EC
GO:0045596	0.0672	0.986	3	186	negative regulation of cell differentiation	BP	1	ACVRL1,LYN,HOXA7	down_ACOR_EC
GO:0009889	0.0672	0.99	3	1190	regulation of biosynthetic process	BP	1	GATA5,TGFBR1,MEIS2	down_VSAP_VSMC
GO:0001934	0.0672	0.986	3	274	positive regulation of protein phosphorylation	BP	1	CCL24,PTGER2,ARHGEF2	up_VPUL_EC
GO:0032501	0.0672	1	2	1782	multicellular organismal process	BP	1	JUP,GATA5	down_VFEM_VSMC
GO:0008016	0.0672	0.998	1	48	regulation of heart contraction	BP	1	IRX5	up_AFEM_VSMC
GO:0042339	0.0672	1	1	1	keratan sulfate metabolic process	BP	0.905	CHST1	up_VCAV_EC
GO:0072709	0.0672	1	1	1	cellular response to sorbitol	BP	0.905	MAPK13	up_VCAV_EC
GO:0072740	0.0672	1	1	1	cellular response to anisomycin	BP	0.905	MAPK13	up_VCAV_EC
GO:0010632	0.0673	0.964	14	95	regulation of epithelial cell migration	BP	1	ENPP2,SLIT2,FGF7,ABL1,HDAC7,EMP2,CCBE1,STAT5A,TBXA2R,SASH1,ZNF580,ACVRL1,HMOX1,TGFBR2	up_VSAP_EC
GO:2001257	0.0673	0.977	6	30	regulation of cation channel activity	BP	1	ACTN2,PLCG2,KCNE3,DAPK1,CACNB3,AKAP6	up_ACOR_VSMC
GO:0032963	0.0673	0.992	2	18	collagen metabolic process	BP	1	MFAP4,FAP	up_AFEM_EC
GO:0044703	0.0673	0.992	2	172	multi-organism reproductive process	BP	1	HOXA9,HOXA11	up_VFEM_VSMC
GO:0042908	0.0673	0.998	1	3	xenobiotic transport	BP	1	SLC6A6	up_AFEM_EC
GO:0060038	0.0673	0.998	1	18	cardiac muscle cell proliferation	BP	1	TBX5	up_APUL_EC
GO:0002424	0.0673	1	1	1	T cell mediated immune response to tumor cell	BP	0.905	HMGB1	up_VCAV_EC
GO:0002840	0.0673	1	1	1	regulation of T cell mediated immune	BP	0.905	HMGB1	up_VCAV_EC

GO:0017055	0.0673	1	1	1	negative regulation of RNA polymerase II transcriptional	BP	0.905	HMGB1	up_VCAV_EC
GO:0032072	0.0673	1	1	1	regulation of restriction endodeoxyribonuclease	BP	0.905	HMGB1	up_VCAV_EC
GO:0032425	0.0673	1	1	1	positive regulation of mismatch repair	BP	0.905	HMGB1	up_VCAV_EC
GO:2000425	0.0673	1	1	1	regulation of apoptotic cell clearance	BP	0.905	HMGB1	up_VCAV_EC
GO:2000426	0.0673	1	1	1	negative regulation of apoptotic cell clearance	BP	0.905	HMGB1	up_VCAV_EC
GO:0002009	0.0674	0.958	24	177	morphogenesis of an epithelium	BP	1	SHROOM3,MLLT3,BMP4,FGFR2,GJA1,SEC24B,CITED2,SPINT1,ZNRF3,LMO4,ENSCAFG00000006238,CLSTN1,NRP1,NPNT,SPINT2,TBX20,TBX5,FZD3,FRS2,DAG1,PSEN1,EDN1,SERPINB5,RREB1	up_ACOR_VSMC
GO:0022008	0.0674	0.979	5	406	neurogenesis	BP	1	LYN,ACSL4,SPG11,NRP2,NEDD4	down_ACOR_EC
GO:0031328	0.0674	0.993	2	512	positive regulation of cellular biosynthetic	BP	1	GATA5,TGFBR1	down_VSAP_VSMC
GO:1901017	0.0674	0.994	2	5	negative regulation of potassium ion transmembrane	BP	1	ACTN2,KCNE3	up_ACOR_VSMC
GO:1901380	0.0674	0.994	2	5	negative regulation of potassium ion	BP	1	ACTN2,KCNE3	up_ACOR_VSMC
GO:0050919	0.0674	0.998	1	10	negative chemotaxis	BP	1	NRP2	down_ACOR_EC
GO:0072511	0.0674	0.998	1	97	divalent inorganic cation transport	BP	1	HOXA3	up_APUL_VSMC
GO:0090066	0.067473032	0.97116	8	162	regulation of anatomical structure size	BP	1	TMOD1,AQP1,PLS1,ACTN2,PAK3,FOXC2,NRP1,PRKCD	up_ACOR_EC
GO:0071560	0.0675	0.973	7	72	cellular response to transforming growth	BP	1	SMAD9,VASN,FBN1,NR3C1,ASPN,ZFP36L1,FUT8	down_VCAV_EC
GO:0008217	0.0675	0.974	7	38	regulation of blood pressure	BP	1	ADRA1B,SLC2A5,F2RL1,RNLS,EDN1,NCALD,ERAP1	up_ACOR_VSMC
GO:0030838	0.0675	0.976	6	37	positive regulation of actin filament	BP	0.905	BAIAP2L1,MTOR,NCKAP1,IQGAP2,NCK1,ARPC5L	up_VCAV_EC
GO:0043367	0.0675	0.983	4	18	CD4-positive, alpha-beta T cell differentiation	BP	0.905	IL6,GATA3,MTOR,HMGB1	up_VCAV_EC
GO:0051496	0.0675	0.983	4	17	positive regulation of stress fiber assembly	BP	1	WNT11,ARHGEF5,ABL1,BAG4	up_VSAP_EC
GO:0048514	0.0675	0.992	2	214	blood vessel morphogenesis	BP	1	HOXA3,SLIT2	up_VPUL_VSMC
GO:0006418	0.0675	0.998	1	31	tRNA aminoacylation for protein translation	BP	1	DARS	down_AFEM_EC
GO:0021559	0.0675	0.998	1	3	trigeminal nerve development	BP	1	PLXNA4	up_AFEM_EC
GO:0021636	0.0675	0.998	1	3	trigeminal nerve morphogenesis	BP	1	PLXNA4	up_AFEM_EC
GO:0021637	0.0675	0.998	1	3	trigeminal nerve structural organization	BP	1	PLXNA4	up_AFEM_EC
GO:0021785	0.0675	0.998	1	3	branchiomotor neuron axon guidance	BP	1	PLXNA4	up_AFEM_EC
GO:1902287	0.0675	0.998	1	3	semaphorin-plexin signaling pathway involved in axon guidance	BP	1	PLXNA4	up_AFEM_EC
GO:0051017	0.0675	0.998	1	49	actin filament bundle assembly	BP	1	LIMK1	up_VCAV_VSMC
GO:0008543	0.0675	0.998	1	27	fibroblast growth factor receptor signaling	BP	1	FGF5	up_VPOR_VSMC

GO:0009987	0.067547022	0.95262	143	5086	cellular process	BP	1	CNTNAP4,SH3TC2,MYOZ2,FMOD,CDH3,NR1H4,TMOD1,GATA4,BRSK1,TRPS1,KCTD12,TBX18,PLCL1,PKIB,CALY,THNSL2,SOX9,NMNAT2,RHOU,PKNOX2,ARHGAP6,PTH1R,GALNT5,RGS1,PCSK1N,PHF20,ATG16L2,NDRG2,AQP1,PORCN,ENSCAFG00000004331,SYNPO2,KCNB1,ADAMTS13,SARDH,EDEM2,PI3,FZD5,CCDC3,AGRN,PDE1A,IFI6,SEMA6D,GNAO1,HSF4,DUSP26,DIXDC1,RASL11A,PTGIR,PDLIM3,NOD2,IL33,RGMA,CASP10,LHX9,ENSCAFG00000028814,PDE4A,INSYN1,SMAD6,B3GNT7,DYNC1I1,KLHL25,PLCE1,PLS1,ACTN2,ZMYND8,ARHGAP28,ESM1,NIT2,PTGDS,PAK3,CFI,MMP28,DLX3,GUCY1B1,GRK7,BCL2,TNFRSF19,PTGER4,ARHGDIB,ELMO2,PREB,CST3,PIAS1,DCLK2,ADAMTSL2,USP53,CHPT1,MAPK8IP2,PDRG1,RAPGEF4,MAVS,FOXC2,FIGNL1,LRRC15,IFIT2,KLHDC8B,TBX5,PIGS,LRP4,CLSTN1,SH3RF3,AK7,LRRC4,PRELP,RBM38,CLU,SLC9A9,SFMBT2,ALDOC,C23H3orf58,ZNF229,CHMP4B,TP53INP2,RARRES2,VPS16,GMEB2,AGBL5,RIOX2,PRKACA,RNF114,UBE2H,STK32C,NRP1,AZIN2,AGPAT2,HM13,FKBP11,ARHGEF26,PRKCD,GNAZ,RIN2,PPP1R26,PM20D1,PYGO2,DBNDD2,EFEMP1,ENSCAFG00000010493,CCNYL1,MFAP4,ADGRL1,TUBB4A,TLE2	up_ACOR_EC
GO:0048584	0.0676	0.976	6	592	positive regulation of response to stimulus	BP	1	ACVRL1,LYN,PDPN,SULF1,STX4,NEDD4	down_ACOR_EC
GO:0071695	0.0676	0.978	5	45	anatomical structure maturation	BP	1	THBS3,RECK,IRX5,GREM1,FOXO3	down_VCAV_EC
GO:0031060	0.0676	0.98	5	23	regulation of histone methylation	BP	0.905	IWS1,GATA3,NSD1,KDM3A,ATRX	up_VCAV_EC
GO:0002052	0.0676	0.988	3	9	positive regulation of neuroblast proliferation	BP	1	SMARCD3,FZD3,DISC1	up_ACOR_VSMC
GO:0070262	0.0676	0.993	2	6	peptidyl-serine dephosphorylation	BP	0.905	SWAP70,NCK1	up_VCAV_EC
GO:0030201	0.0676	0.998	1	12	heparan sulfate proteoglycan metabolic	BP	1	SULF1	down_ACOR_EC
GO:0002440	0.0676	0.998	1	51	production of molecular mediator of immune	BP	1	TLR2	up_AFEM_VSMC
GO:0006024	0.0676	0.998	1	16	glycosaminoglycan biosynthetic process	BP	1	CHST7	up_VFEM_EC
GO:1901655	0.0676	0.998	1	20	cellular response to ketone	BP	1	PTGER2	up_VPUL_EC
GO:2001057	0.0676	0.998	1	16	reactive nitrogen species metabolic process	BP	1	CAV1	up_VSAP_VSMC
GO:0055085	0.067602285	0.9656	12	284	transmembrane transport	BP	1	NR1H4,CLIC3,AQP1,ENSCAFG00000004331,KCNB1,ACTN2,SLC25A42,MAPK8IP2,SLC9A9,RARRES2,AZIN2,PM20D1	up_ACOR_EC
GO:0060284	0.0677	0.96	19	212	regulation of cell development	BP	0.874	ARHGEF2,SIPA1L1,DPYSL3,HOOK3,IFRD1,HIF1A,SLC45A3,TNIK,UST,CTNNB1,ENSCAFG00000002015,APOE,LYN,MMD,VLDLR,REST,DISC1,NRP1,YWHAH	down_VSAP_EC
GO:0043297	0.0677	0.983	4	17	apical junction assembly	BP	0.905	PRKCH,OCLN,TJP1,CTNNA1	up_VCAV_EC
GO:0006265	0.0677	0.994	2	5	DNA topological change	BP	1	TOP3A,HMGB2	down_ACOR_VSMC
GO:0048730	0.0677	0.993	2	7	epidermis morphogenesis	BP	0.874	CTNNB1,PLOD3	down_VSAP_EC
GO:0051962	0.0677	0.991	2	112	positive regulation of nervous system	BP	1	SLITRK5,NTRK2	up_APUL_EC
GO:0007517	0.0677	0.991	2	100	muscle organ development	BP	1	SIX1,CYP26B1	up_VFEM_EC
GO:0031998	0.0677	0.994	2	5	regulation of fatty acid beta-oxidation	BP	1	ACACB,LONP2	up_VSAP_EC
GO:0045595	0.0677	1	1	469	regulation of cell differentiation	BP	1	GATA5	down_AFEM_VSMC
GO:0010830	0.0677	0.998	1	16	regulation of myotube differentiation	BP	1	SHOX2	up_VSAP_VSMC
GO:0097300	0.0677	0.998	1	17	programmed necrotic cell death	BP	1	CAV1	up_VSAP_VSMC
GO:0032874	0.067784641	0.98148	4	57	positive regulation of stress-activated MAPK	BP	1	FZD5,NOD2,TNFRSF19,SH3RF3	up_ACOR_EC
GO:0031503	0.0678	0.967	11	79	protein-containing complex localization	BP	1	DDX39A,STXBP6,NDC1,RAN,BIRC5,NUP62,HASPIN,DDX39B,ENSCAFG00000005299,EZR,MIOS	down_ACOR_VSMC
GO:0065009	0.0678	0.974	7	735	regulation of molecular function	BP	1	LYN,HOXA3,IGF1R,PRKAR2A,MYO1D,ASPN,NEDD4	down_ACOR_EC
GO:0000226	0.0678	0.981	4	197	microtubule cytoskeleton organization	BP	1	MZT1,HOOK3,ARHGEF2,GCC2	up_VPOR_EC

GO:0033036	0.0678	0.989	3	852	macromolecule localization	BP	1	WNT7A,ARG2,ESR1	up_AORT_VSMC
GO:0043405	0.0678	0.991	2	85	regulation of MAP kinase activity	BP	1	LYN,IGF1R	down_ACOR_EC
GO:0042116	0.0678	0.998	1	18	macrophage activation	BP	1	BPI	up_APUL_EC
GO:0006554	0.0678	1	1	1	lysine catabolic process	BP	0.905	PIPOX	up_VCAV_EC
GO:0019474	0.0678	1	1	1	L-lysine catabolic process to acetyl-CoA	BP	0.905	PIPOX	up_VCAV_EC
GO:0019477	0.0678	1	1	1	L-lysine catabolic process	BP	0.905	PIPOX	up_VCAV_EC
GO:0033514	0.0678	1	1	1	L-lysine catabolic process to acetyl-CoA via L-	BP	0.905	PIPOX	up_VCAV_EC
GO:0046440	0.0678	1	1	1	L-lysine metabolic process	BP	0.905	PIPOX	up_VCAV_EC
GO:0007389	0.067889239	0.97287	7	137	pattern specification process	BP	1	FZD5,SMAD6,FOXC2,TBX5,LRP4,CLSTN1,NRP1	up_ACOR_EC
GO:0051239	0.0679	0.977	6	859	regulation of multicellular organismal process	BP	1	TBX5,NR1H4,EVI2B,CCL24,FLRT3,ARHGEF2	up_VPUL_EC
GO:0009891	0.0679	0.979	5	521	positive regulation of biosynthetic process	BP	1	HOXA11,HOXA9,SALL1,PRR16,SHOX2	up_VSAP_VSMC
GO:0045165	0.0679	0.981	4	65	cell fate commitment	BP	1	HOXC10,GAS1,DLL1,CYLD	up_AFEM_EC
GO:0000272	0.0679	0.994	2	4	polysaccharide catabolic process	BP	1	PYGM,GAA	up_ACOR_VSMC
GO:0009251	0.0679	0.994	2	4	glucan catabolic process	BP	1	PYGM,GAA	up_ACOR_VSMC
GO:0005980	0.0679	0.994	2	4	glycogen catabolic process	BP	1	PYGM,GAA	up_ACOR_VSMC
GO:0044247	0.0679	0.994	2	4	cellular polysaccharide catabolic process	BP	1	PYGM,GAA	up_ACOR_VSMC
GO:0071704	0.068	0.979	11	3535	organic substance metabolic process	BP	1	ETFA,IVNS1ABP,EXTL2,DARS,SUCLG2,EIF3M,SRSF6,USP33,GABPA,NDUFA10,TSEN15	down_AFEM_EC
GO:0044085	0.068	0.981	5	1076	cellular component biogenesis	BP	1	GPSM2,EIF3M,SRSF6,NDUFA10,FSCN1	down_AFEM_EC
GO:0048227	0.068	0.994	2	5	plasma membrane to endosome transport	BP	1	USP6NL,RAB5C	up_ACOR_VSMC
GO:1904063	0.068	0.998	1	17	negative regulation of cation transmembrane	BP	1	NEDD4	down_ACOR_EC
GO:0060167	0.068	1	1	1	regulation of adenosine receptor signaling	BP	0.905	ACPP	up_VCAV_EC
GO:0060168	0.068	1	1	1	positive regulation of adenosine receptor	BP	0.905	ACPP	up_VCAV_EC
GO:0060048	0.068	0.998	1	30	cardiac muscle contraction	BP	1	NKX2-5	up_VPOR_VSMC
GO:0035270	0.068	0.998	1	35	endocrine system development	BP	1	HOXA3	up_VPUL_VSMC
GO:0046879	0.0681	0.969	10	63	hormone secretion	BP	1	LEP,TM7SF3,PRKCE,SCG5,CHD7,CCL5,RAB11B,EDN1,PFKL,PARK7	up_ACOR_VSMC
GO:0070482	0.0681	0.968	10	75	response to oxygen levels	BP	0.905	CXCR4,CD34,EDN1,MTOR,BMP2,PTGS2,PMAIP1,HIF1A,HP1BP3,P2RX2	up_VCAV_EC
GO:0051171	0.0681	0.984	5	1697	regulation of nitrogen compound metabolic	BP	1	TLR2,EYA1,HOXA9,HOXA10,IRX5	up_AFEM_VSMC
GO:0051893	0.0681	0.983	4	20	regulation of focal adhesion assembly	BP	0.905	TEK,SLK,APOD,ENSCAFG00000002015	up_VCAV_EC
GO:0090109	0.0681	0.983	4	20	regulation of cell-substrate junction	BP	0.905	TEK,SLK,APOD,ENSCAFG00000002015	up_VCAV_EC
GO:0007519	0.0681	0.998	1	49	skeletal muscle tissue development	BP	1	BEND2	up_AFEM_VSMC
GO:1903727	0.06816801	0.99158	2	16	positive regulation of phospholipid metabolic	BP	1	NR1H4,PRKCD	up_ACOR_EC
GO:0023052	0.0682	0.971	10	1602	signaling	BP	1	NR1H4,TBX5,MAPK10,HTR7,PLCL1,ARHGEF26,SLITRK5,NPNT,NTRK2,PDE1A	up_APUL_EC
GO:0030198	0.0682	0.97	9	81	extracellular matrix organization	BP	0.874	SULF1,SERPINH1,ERO1A,P4HA1,FSCN1,PLOD3,MYO1E,P3H4,COL12A1	down_VSAP_EC
GO:0090092	0.0682	0.971	8	72	regulation of transmembrane receptor protein serine/threonine	BP	0.874	SULF1,HSPA5,SNX25,ILK,NEO1,ADAM17,STRAP,INHBA	down_VSAP_EC
GO:0006816	0.0682	0.991	2	84	calcium ion transport	BP	1	LYN,HOXA3	down_ACOR_EC
GO:0046040	0.0682	0.992	2	9	IMP metabolic process	BP	1	NT5C2,AMPD2	down_VCAV_EC
GO:0019511	0.0682	0.994	2	5	peptidyl-proline hydroxylation	BP	1	EGLN1,P4HA1	up_ACOR_VSMC
GO:0030888	0.0682	0.998	1	13	regulation of B cell proliferation	BP	1	LYN	down_ACOR_EC
GO:0003197	0.0682	0.998	1	16	endocardial cushion development	BP	1	ACVRL1	down_ACOR_EC

GO:0021960	0.0682	0.998	1	3	anterior commissure morphogenesis	BP	1	PLXNA4	up_AFEM_EC
GO:0071295	0.0682	0.998	1	3	cellular response to vitamin	BP	1	POSTN	up_AFEM_EC
GO:0071219	0.0682	0.998	1	50	cellular response to molecule of bacterial	BP	1	TLR2	up_AFEM_VSMC
GO:0040013	0.0682	0.998	1	100	negative regulation of locomotion	BP	1	TBX5	up_APUL_VSMC
GO:0048870	0.0683	0.962	16	451	cell motility	BP	1	CMKLR1,ANGPT4,HOXA7,TWIST2,HCK,GREM1,CAMK2A,LRRC15,RECK,TBXA2R,BVES,LGR6,CCL5,FAP,ADAM17,KANK1	up_AFEM_EC
GO:0051674	0.0683	0.962	16	451	localization of cell	BP	1	CMKLR1,ANGPT4,HOXA7,TWIST2,HCK,GREM1,CAMK2A,LRRC15,RECK,TBXA2R,BVES,LGR6,CCL5,FAP,ADAM17,KANK1	up_AFEM_EC
GO:0022603	0.0683	0.982	4	301	regulation of anatomical structure morphogenesis	BP	1	ACVRL1,PDPN,SULF1,ASPN	down_ACOR_EC
GO:0097006	0.0683	0.986	3	19	regulation of plasma lipoprotein particle levels	BP	1	LCAT,LRPAP1,SOAT1	down_VCAV_EC
GO:0035725	0.0683	0.998	1	16	sodium ion transmembrane transport	BP	1	NEDD4	down_ACOR_EC
GO:0000117	0.0683	1	1	1	regulation of transcription involved in G2/M transition of mitotic cell	BP	0.874	BACH1	down_VSAP_EC
GO:0010172	0.0683	0.998	1	3	embryonic body morphogenesis	BP	1	GREM2	up_AFEM_EC
GO:0032310	0.0683	0.998	1	3	prostaglandin secretion	BP	1	LEP	up_AFEM_EC
GO:0061502	0.0683	0.998	1	3	early endosome to recycling endosome	BP	1	MYO1D	up_AFEM_EC
GO:0010389	0.0683	0.998	1	30	regulation of G2/M transition of mitotic cell	BP	1	TAOK1	up_AORT_EC
GO:0046626	0.0683	0.998	1	18	regulation of insulin receptor signaling	BP	1	NR1H4	up_APUL_EC
GO:0016925	0.0683	0.998	1	16	protein sumoylation	BP	1	EYA1	up_VFEM_EC
GO:0090066	0.0684	0.958	22	162	regulation of anatomical structure size	BP	1	ADRA1B,ACTN2,HTR2B,NPPB,HRH1,CLCN3,ADCY6,F2RL1,HRH2,SLC12A7,JMY,LRRC8C,NRP1,ULK2,EVL,PTGDR,PRKCE,CLN8,SSH3,EDN1,WASL,DOCK5	up_ACOR_VSMC
GO:0046085	0.0684	0.994	2	5	adenosine metabolic process	BP	1	PTGDR,NT5C2	up_ACOR_VSMC
GO:0032410	0.0684	0.998	1	17	negative regulation of transporter activity	BP	1	NEDD4	down_ACOR_EC
GO:0002181	0.0684	0.998	1	35	cytoplasmic translation	BP	1	EIF3M	down_AFEM_EC
GO:0098856	0.0684	0.998	1	3	intestinal lipid absorption	BP	1	LEP	up_AFEM_EC
GO:0030299	0.0684	0.998	1	3	intestinal cholesterol absorption	BP	1	LEP	up_AFEM_EC
GO:0048251	0.0684	0.998	1	3	elastic fiber assembly	BP	1	MFAP4	up_AFEM_EC
GO:0030177	0.0684	0.998	1	51	positive regulation of Wnt signaling pathway	BP	1	TLR2	up_AFEM_VSMC
GO:0043122	0.0684	0.998	1	62	regulation of I-kappaB kinase/NF-kappaB	BP	1	ESR1	up_AORT_VSMC
GO:0008361	0.0684	0.998	1	50	regulation of cell size	BP	1	LIMK1	up_VCAV_VSMC
GO:0046364	0.0684	0.998	1	27	monosaccharide biosynthetic process	BP	1	FBP1	up_VFEM_VSMC
GO:0045600	0.0684	0.998	1	21	positive regulation of fat cell differentiation	BP	1	WIF1	up_VPUL_EC
GO:0031952	0.0684	0.998	1	15	regulation of protein autophosphorylation	BP	1	CAV1	up_VSAP_VSMC
GO:0044270	0.0685	0.96	18	147	cellular nitrogen compound catabolic process	BP	1	RNPS1,EIF4A3,EDC3,HNRNPD,THRAP3,EDC4,FEN1,RNASEH2B,SMG9,DIS3,FASTKD3,PDXP,AIFM1,ENSCAFG00000015189,RNASEH2C,NUDT1,EXOSC5,NUDT3	down_ACOR_VSMC
GO:0006836	0.0685	0.972	8	47	neurotransmitter transport	BP	1	FBXL20,STX11,PSEN1,CLN8,EDN1,KCNC4,PREPL,PARK7	up_ACOR_VSMC
GO:2000179	0.0685	0.987	3	14	positive regulation of neural precursor cell	BP	0.874	HIF1A,CTNNB1,DISC1	down_VSAP_EC
GO:0003148	0.0685	0.998	1	13	outflow tract septum morphogenesis	BP	1	NRP2	down_ACOR_EC
GO:0030029	0.0685	0.999	1	239	actin filament-based process	BP	1	JUP	down_VFEM_VSMC
GO:0044774	0.0685	0.998	1	30	mitotic DNA integrity checkpoint	BP	1	TAOK1	up_AORT_EC
GO:0001818	0.0685	0.998	1	65	negative regulation of cytokine production	BP	1	ARG2	up_AORT_VSMC

GO:1990784	0.0685	1	1	1	response to dsDNA	BP	0.905	RIOK3	up_VCAV_EC
GO:1990786	0.0685	1	1	1	cellular response to dsDNA	BP	0.905	RIOK3	up_VCAV_EC
GO:0051153	0.0685	0.998	1	28	regulation of striated muscle cell differentiation	BP	1	SHOX2	up_VFEM_VSMC
GO:0060491	0.0686	0.973	7	62	regulation of cell projection assembly	BP	0.874	FSCN1,DPYSL3,DYNLL1,ANLN,KLF5,HTT,NRP1	down_VSAP_EC
GO:0120032	0.0686	0.973	7	62	regulation of plasma membrane bounded cell	BP	0.874	FSCN1,DPYSL3,DYNLL1,ANLN,KLF5,HTT,NRP1	down_VSAP_EC
GO:0002088	0.0686	0.982	4	26	lens development in camera-type eye	BP	0.874	BCAR3,CTNNB1,CRYAB,PDS5B	down_VSAP_EC
GO:0015800	0.0686	0.988	3	10	acidic amino acid transport	BP	1	PSEN1,CLN8,SLC25A12	up_ACOR_VSMC
GO:0071495	0.0686	0.992	2	365	cellular response to endogenous stimulus	BP	1	WNT7A,ESR1	up_AORT_VSMC
GO:0021615	0.0686	0.998	1	3	glossopharyngeal nerve morphogenesis	BP	1	PLXNA4	up_AFEM_EC
GO:1901096	0.0686	0.998	1	3	regulation of autophagosome	BP	1	CLEC16A	up_AFEM_EC
GO:0072677	0.068655123	0.99839	1	3	eosinophil migration	BP	1	PTGER4	up_ACOR_EC
GO:0033157	0.068664659	0.97765	5	82	regulation of intracellular protein transport	BP	1	SH3TC2,KCNB1,EDEM2,MAVS,PRKCD	up_ACOR_EC
GO:0071310	0.0687	0.983	4	634	cellular response to organic substance	BP	1	FGF5,NKX2-5,BAMBI,CYP26B1	up_VPOR_VSMC
GO:0090189	0.0687	0.987	3	12	regulation of branching involved in ureteric bud	BP	1	GREM1,HOXB7,SIX4	down_ACOR_VSMC
GO:0045646	0.0687	0.986	3	19	regulation of erythrocyte differentiation	BP	1	ZFP36L1,FOXO3,ANKRD54	down_VCAV_EC
GO:0046885	0.0687	0.993	2	7	regulation of hormone biosynthetic process	BP	0.874	HIF1A,REST	down_VSAP_EC
GO:1903321	0.0687	0.998	1	33	negative regulation of protein modification by small protein conjugation	BP	1	IVNS1ABP	down_AFEM_EC
GO:2000322	0.0687	0.998	1	3	regulation of glucocorticoid receptor	BP	1	CRY2	up_AFEM_EC
GO:2000323	0.0687	0.998	1	3	negative regulation of glucocorticoid receptor signaling pathway	BP	1	CRY2	up_AFEM_EC
GO:0032105	0.0687	0.998	1	3	negative regulation of response to extracellular	BP	1	LEP	up_AFEM_EC
GO:0032108	0.0687	0.998	1	3	negative regulation of response to nutrient	BP	1	LEP	up_AFEM_EC
GO:0046322	0.0687	0.998	1	3	negative regulation of fatty acid oxidation	BP	1	ACACB	up_AFEM_EC
GO:0097343	0.0687	0.998	1	3	ripoptosome assembly	BP	1	CYLD	up_AFEM_EC
GO:1901026	0.0687	0.998	1	3	ripoptosome assembly involved in necroptotic	BP	1	CYLD	up_AFEM_EC
GO:1990845	0.0687	0.998	1	51	adaptive thermogenesis	BP	1	HOXC10	up_AFEM_VSMC
GO:0003179	0.0687	0.998	1	19	heart valve morphogenesis	BP	1	TBX5	up_APUL_EC
GO:0046700	0.0688	0.96	18	147	heterocycle catabolic process	BP	1	RNPS1,EIF4A3,EDC3,HNRNPD,THRAP3,EDC4,FEN1,RNASEH2B,SMG9,DIS3,FASTKD3,PDXP,AIFM1,ENSCAFG00000015189,RNASEH2C,NUDT1,EXOSC5,NUDT3	down_ACOR_VSMC
GO:0010467	0.0688	0.969	12	1787	gene expression	BP	1	HOXA11,TBX15,HOXA10,HOXA9,PITX1,EMX2,SALL1,IRX5,PRR16,TLR2,CAV1,SHOX2	up_VSAP_VSMC
GO:0072511	0.0688	0.968	10	97	divalent inorganic cation transport	BP	0.874	SLC39A7,ERO1A,ATP2B1,REM1,CTNNB1,LYN,DDIT3,HTT,CALM1,HOMER1	down_VSAP_EC
GO:0006491	0.0688	0.992	2	9	N-glycan processing	BP	1	FUT8,PRKCSH	down_VCAV_EC
GO:0055089	0.0688	0.993	2	7	fatty acid homeostasis	BP	0.874	NR1H4,APOE	down_VSAP_EC
GO:0030198	0.0688	0.998	1	81	extracellular matrix organization	BP	1	TGFBI	down_VSAP_VSMC
GO:0016358	0.0688	0.998	1	58	dendrite development	BP	1	WNT7A	up_AORT_VSMC
GO:0120093	0.0688	1	1	1	regulation of peptidyl-lysine crotonylation	BP	0.905	CDYL	up_VCAV_EC
GO:0120094	0.0688	1	1	1	negative regulation of peptidyl-lysine	BP	0.905	CDYL	up_VCAV_EC
GO:0140066	0.0688	1	1	1	peptidyl-lysine crotonylation	BP	0.905	CDYL	up_VCAV_EC
GO:0019233	0.0688	0.998	1	16	sensory perception of pain	BP	1	MME	up_VFEM_EC
GO:0010463	0.0688	0.998	1	28	mesenchymal cell proliferation	BP	1	SHOX2	up_VFEM_VSMC

GO:0006816	0.0689	0.969	9	84	calcium ion transport	BP	0.874	ERO1A,ATP2B1,REM1,CTNNB1,LYN,DDIT3,HTT,CALM1,HOMER1	down_VSAP_EC
GO:0002695	0.0689	0.973	7	44	negative regulation of leukocyte activation	BP	0.905	ADGRF5,TNFAIP3,PELI1,ARG2,THOC1,CD200,HMGB1	up_VCAV_EC
GO:0048699	0.0689	0.982	4	371	generation of neurons	BP	1	EMX2,HOXC10,IRX5,SHOX2	up_VSAP_VSMC
GO:0070201	0.0689	0.986	3	187	regulation of establishment of protein	BP	1	LYN,ACSL3,STX4	down_ACOR_EC
GO:0060348	0.0689	0.991	2	78	bone development	BP	1	SULF1,CSGALNACT1	down_ACOR_EC
GO:0001842	0.0689	0.998	1	3	neural fold formation	BP	1	LUZP1	up_AFEM_EC
GO:0001955	0.0689	0.998	1	3	blood vessel maturation	BP	1	RECK	up_AFEM_EC
GO:0042490	0.0689	0.998	1	19	mechanoreceptor differentiation	BP	1	NTRK2	up_APUL_EC
GO:0061572	0.0689	0.998	1	50	actin filament bundle organization	BP	1	LIMK1	up_VCAV_VSMC
GO:0032413	0.0689	0.998	1	15	negative regulation of ion transmembrane transporter activity	BP	1	CAV1	up_VSAP_VSMC
GO:0098662	0.069	0.966	11	131	inorganic cation transmembrane transport	BP	1	PRNP,FXYD1,SLC39A6,CACNB3,ATP5MC1,THY1,ATP6V0C,PARK7,SCARA5,CYBA,ARHGAP1	down_VCAV_EC
GO:0006937	0.069	0.985	3	40	regulation of muscle contraction	BP	1	CHRM2,TBXA2R,DMPK	up_AFEM_EC
GO:0030810	0.069	0.998	1	3	positive regulation of nucleotide biosynthetic	BP	1	PPARGC1A	up_AFEM_EC
GO:0050995	0.069	0.998	1	3	negative regulation of lipid catabolic process	BP	1	ACACB	up_AFEM_EC
GO:1900373	0.069	0.998	1	3	positive regulation of purine nucleotide	BP	1	PPARGC1A	up_AFEM_EC
GO:0034764	0.069	0.998	1	50	positive regulation of transmembrane transport	BP	1	CA2	up_VCAV_VSMC
GO:0006814	0.069	0.998	1	28	sodium ion transport	BP	1	NETO1	up_VFEM_VSMC
GO:0010770	0.069	0.998	1	29	positive regulation of cell morphogenesis involved in differentiation	BP	1	SHOX2	up_VFEM_VSMC
GO:0006464	0.0691	0.945	108	1162	cellular protein modification process	BP	0.905	EPHB1,PLK2,TEK,HCLS1,ST8SIA6,KIT,PPP2R5A,PRKCH,TNFAIP3,MAP3K11,TNIP1,PDGFB,STK10,SH2D3C,RELN,ST6GAL1,PDE4D,IWS1,MEF2C,ATF3,TIPARP,RAPGEF1,TLR4,GATA3,USP33,EDN1,RALB,APLNR,CTTNBP2NL,ZDHHC22,NEDD9,PELI1,YES1,ENSCAFG00000010893,EPHB4,B3GNT5,MTOR,ASB9,HHAT,MAP3K3,BMP2,CDYL,HMG20A,HHEX,PREX2,SLK,BMP2K,SH3BP5,ST3GAL6,ENC1,ABCA1,PPP1R2,ERC1,MAPK13,ITPKB,PROX1,UHRF2,ENSCAFG00000010596,MADD,ARID4B,PAK2,GALNT18,PRKD2,SWAP70,TLR3,MFNG,NCK1,ESCO1,DPH5,VHL,OTUD4,WSB1,PARP4,FES,NSD1,PTGS2,MMD,TOP1,RAPGEF2,CREB1,BCL10,SENP6,USP28,TAOK1,CNOT7,HMGB1,NOS2,BLM,SPTBN4,RIPK1,USP13,USP38,PDGFD,USP37,C7H18orf25,CUL4A,BMX,UBE2D1,KDM5A,KDM3A,LMTK2,ATRX,ZDHHC6,IVNS1ABP,JCAD,TET2,ARHGEF2,CDON	up_VCAV_EC
GO:0036211	0.0691	0.945	108	1162	protein modification process	BP	0.905	EPHB1,PLK2,TEK,HCLS1,ST8SIA6,KIT,PPP2R5A,PRKCH,TNFAIP3,MAP3K11,TNIP1,PDGFB,STK10,SH2D3C,RELN,ST6GAL1,PDE4D,IWS1,MEF2C,ATF3,TIPARP,RAPGEF1,TLR4,GATA3,USP33,EDN1,RALB,APLNR,CTTNBP2NL,ZDHHC22,NEDD9,PELI1,YES1,ENSCAFG00000010893,EPHB4,B3GNT5,MTOR,ASB9,HHAT,MAP3K3,BMP2,CDYL,HMG20A,HHEX,PREX2,SLK,BMP2K,SH3BP5,ST3GAL6,ENC1,ABCA1,PPP1R2,ERC1,MAPK13,ITPKB,PROX1,UHRF2,ENSCAFG00000010596,MADD,ARID4B,PAK2,GALNT18,PRKD2,SWAP70,TLR3,MFNG,NCK1,ESCO1,DPH5,VHL,OTUD4,WSB1,PARP4,FES,NSD1,PTGS2,MMD,TOP1,RAPGEF2,CREB1,BCL10,SENP6,USP28,TAOK1,CNOT7,HMGB1,NOS2,BLM,SPTBN4,RIPK1,USP13,USP38,PDGFD,USP37,C7H18orf25,CUL4A,BMX,UBE2D1,KDM5A,KDM3A,LMTK2,ATRX,ZDHHC6,IVNS1ABP,JCAD,TET2,ARHGEF2,CDON	up_VCAV_EC
GO:0043648	0.0691	0.982	4	26	dicarboxylic acid metabolic process	BP	0.874	NR1H4,ALDH1L2,NMNAT2,ALDH18A1	down_VSAP_EC

GO:0045665	0.0691	0.991	2	55	negative regulation of neuron differentiation	BP	1	SEMA3A,RNF6	up_VPOR_EC
GO:0046887	0.0691	0.998	1	18	positive regulation of hormone secretion	BP	1	NR1H4	up_APUL_EC
GO:0035050	0.0691	0.998	1	28	embryonic heart tube development	BP	1	NKX2-5	up_VPOR_VSMC
GO:1902202	0.069183983	0.99836	1	3	regulation of hepatocyte growth factor receptor	BP	1	ESM1	up_ACOR_EC
GO:0007204	0.0692	0.971	8	46	positive regulation of cytosolic calcium ion	BP	1	ADRA1B,PLCG2,HTR2B,ITPR1,F2RL1,PRKCE,CHD7,EDN1	up_ACOR_VSMC
GO:0042552	0.0692	0.976	6	30	myelination	BP	1	SH3TC2,MBP,WASF3,ATRN,DAG1,JAM3	up_ACOR_VSMC
GO:1903580	0.0692	0.993	2	5	positive regulation of ATP metabolic process	BP	1	PPARGC1A,PARK7	up_ACOR_VSMC
GO:0006452	0.0692	1	1	1	translational frameshifting	BP	1	EIF5A	down_ACOR_VSMC
GO:0045905	0.0692	1	1	1	positive regulation of translational termination	BP	1	EIF5A	down_ACOR_VSMC
GO:0001892	0.0692	0.998	1	35	embryonic placenta development	BP	1	CITED2	down_VPOR_EC
GO:0010243	0.0692	0.999	1	238	response to organonitrogen	BP	1	JUP	down_VFEM_VSMC
GO:0034350	0.0692	0.998	1	3	regulation of glial cell apoptotic process	BP	1	DLL1	up_AFEM_EC
GO:0034351	0.0692	0.998	1	3	negative regulation of glial cell apoptotic process	BP	1	DLL1	up_AFEM_EC
GO:0032652	0.0692	0.998	1	18	regulation of interleukin-1 production	BP	1	NR1H4	up_APUL_EC
GO:0031114	0.0692	0.998	1	9	regulation of microtubule depolymerization	BP	1	ARHGEF2	up_VPOR_EC
GO:0061337	0.0692	0.998	1	16	cardiac conduction	BP	1	CAV1	up_VSAP_VSMC
GO:0042592	0.0693	0.96	18	522	homeostatic process	BP	1	CMKLR1,HOXC10,LEP,CRY2,RAB20,DLL1,GPAM,MLLT11,NFE2L1,CYLD,CNNM4,RAB7A,ATP6V0B,ACACB,NAPEPLD,PPARGC1A,DMPK,GCNT4	up_AFEM_EC
GO:0055006	0.0693	0.982	4	18	cardiac cell development	BP	0.905	EDN1,MTOR,PROX1,JAG1	up_VCAV_EC
GO:0035270	0.0693	0.998	1	35	endocrine system development	BP	1	CITED2	down_VPOR_EC
GO:0086013	0.0693	1	1	1	membrane repolarization during cardiac muscle cell action potential	BP	1	KCNE3	up_ACOR_VSMC
GO:0098915	0.0693	1	1	1	membrane repolarization during ventricular cardiac muscle cell action	BP	1	KCNE3	up_ACOR_VSMC
GO:0106135	0.0693	1	1	1	negative regulation of cardiac muscle cell	BP	1	KCNE3	up_ACOR_VSMC
GO:1902302	0.0693	1	1	1	regulation of potassium ion export	BP	1	KCNE3	up_ACOR_VSMC
GO:1902303	0.0693	1	1	1	negative regulation of potassium ion export	BP	1	KCNE3	up_ACOR_VSMC
GO:1903764	0.0693	1	1	1	regulation of potassium ion export across plasma	BP	1	KCNE3	up_ACOR_VSMC
GO:1903765	0.0693	1	1	1	negative regulation of potassium ion export across plasma membrane	BP	1	KCNE3	up_ACOR_VSMC
GO:1903946	0.0693	1	1	1	negative regulation of ventricular cardiac muscle cell action potential	BP	1	KCNE3	up_ACOR_VSMC
GO:1905024	0.0693	1	1	1	regulation of membrane repolarization during ventricular cardiac muscle cell action potential	BP	1	KCNE3	up_ACOR_VSMC
GO:1905025	0.0693	1	1	1	negative regulation of membrane repolarization during ventricular cardiac muscle cell action	BP	1	KCNE3	up_ACOR_VSMC
GO:1905031	0.0693	1	1	1	regulation of membrane repolarization during cardiac muscle cell action	BP	1	KCNE3	up_ACOR_VSMC
GO:1905032	0.0693	1	1	1	negative regulation of membrane repolarization during cardiac muscle cell	BP	1	KCNE3	up_ACOR_VSMC
GO:0002315	0.0693	0.998	1	3	marginal zone B cell differentiation	BP	1	DLL1	up_AFEM_EC
GO:1990523	0.0693	0.998	1	3	bone regeneration	BP	1	LGR6	up_AFEM_EC
GO:0021520	0.0693	0.998	1	3	spinal cord motor neuron cell fate specification	BP	1	HOXC10	up_AFEM_EC

GO:0070602	0.0693	0.998	1	3	regulation of centromeric sister chromatid cohesion	BP	1	AXIN2	up_AFEM_EC
GO:0033030	0.0693	1	1	1	negative regulation of neutrophil apoptotic	BP	0.905	ITPKB	up_VCAV_EC
GO:0045806	0.0693	0.998	1	15	negative regulation of endocytosis	BP	1	CAV1	up_VSAP_VSMC
GO:0051603	0.0694	0.956	25	305	proteolysis involved in cellular protein catabolic process	BP	0.874	PRICKLE1,PLK3,HSPA5,SOCS5,HM13,HSP90B1,TRIB3,SEL1L,USP12,DNAJC10,UBA6,UCHL5,SPOPL,DNAJB9,USP48,DDIT3,CBLC,GSK3B,CUL2,SPOP,DISC1,RAD23A,TMTC3,USP14,TNFAIP1	down_VSAP_EC
GO:0007166	0.0694	0.976	6	686	cell surface receptor signaling pathway	BP	1	NETO1,SALL1,TLR2,CAV1,SHOX2,FGF5	up_VSAP_VSMC
GO:0070098	0.0694	0.987	3	14	chemokine-mediated signaling pathway	BP	0.905	ACKR1,CXCL8,CXCR4	up_VCAV_EC
GO:0072711	0.0694	0.993	2	5	cellular response to hydroxyurea	BP	1	RAD51,TIMELESS	down_ACOR_VSMC
GO:0009891	0.0694	0.993	2	521	positive regulation of biosynthetic process	BP	1	GATA5,TGFBR1	down_VSAP_VSMC
GO:0033145	0.0694	0.993	2	5	positive regulation of intracellular steroid hormone receptor	BP	1	SKP2,PARK7	up_ACOR_VSMC
GO:0042481	0.0694	0.993	2	5	regulation of odontogenesis	BP	1	BMP4,TNFRSF11B	up_ACOR_VSMC
GO:0036316	0.0694	1	1	1	SREBP-SCAP complex retention in endoplasmic	BP	1	INSIG1	down_ACOR_VSMC
GO:1901303	0.0694	1	1	1	negative regulation of cargo loading into COPII-	BP	1	INSIG1	down_ACOR_VSMC
GO:2000638	0.0694	1	1	1	regulation of SREBP signaling pathway	BP	1	INSIG1	down_ACOR_VSMC
GO:2000639	0.0694	1	1	1	negative regulation of SREBP signaling pathway	BP	1	INSIG1	down_ACOR_VSMC
GO:0070166	0.0694	0.998	1	3	enamel mineralization	BP	1	CNNM4	up_AFEM_EC
GO:0070661	0.0694	0.998	1	63	leukocyte proliferation	BP	1	ARG2	up_AORT_VSMC
GO:1990138	0.0694	0.998	1	35	neuron projection extension	BP	1	SLIT2	up_VPUL_VSMC
GO:0007589	0.0694	0.998	1	16	body fluid secretion	BP	1	CAV1	up_VSAP_VSMC
GO:0090087	0.0695	0.985	3	185	regulation of peptide transport	BP	1	LYN,ACSL3,STX4	down_ACOR_EC
GO:0006767	0.0695	0.987	3	12	water-soluble vitamin metabolic process	BP	1	PDXP,GSTO1,ENSCAFG00000005637	down_ACOR_VSMC
GO:0051336	0.0695	0.986	3	251	regulation of hydrolase activity	BP	1	PCSK1N,NPNT,NTRK2	up_APUL_EC
GO:1902903	0.0695	0.991	2	125	regulation of supramolecular fiber	BP	1	CCL24,ARHGEF2	up_VPUL_EC
GO:0043537	0.0695	0.998	1	15	negative regulation of blood vessel endothelial	BP	1	ACVRL1	down_ACOR_EC
GO:0045638	0.0695	0.998	1	33	negative regulation of myeloid cell	BP	1	GABPA	down_AFEM_EC
GO:0007517	0.0695	0.998	1	100	muscle organ development	BP	1	TBX5	up_APUL_VSMC
GO:1904888	0.0696	0.981	4	31	cranial skeletal system development	BP	1	TBX15,GLI3,IRX5,RDH10	down_VCAV_EC
GO:0042509	0.0696	0.986	3	17	regulation of tyrosine phosphorylation of STAT	BP	0.874	PTPN2,CNOT7,LYN	down_VSAP_EC
GO:0032876	0.0696	0.993	2	5	negative regulation of DNA endoreduplication	BP	1	SMC1A,SMC3	down_ACOR_VSMC
GO:0018209	0.0696	0.991	2	125	peptidyl-serine modification	BP	1	STK32B,PTGER2	up_VPUL_EC
GO:0050654	0.0696	0.998	1	15	chondroitin sulfate proteoglycan metabolic	BP	1	CSGALNACT1	down_ACOR_EC
GO:0009650	0.0696	0.998	1	3	UV protection	BP	1	MFAP4	up_AFEM_EC
GO:0035930	0.0696	0.998	1	3	corticosteroid hormone secretion	BP	1	CRY2	up_AFEM_EC
GO:0035933	0.0696	0.998	1	3	glucocorticoid secretion	BP	1	CRY2	up_AFEM_EC
GO:1901525	0.0696	0.998	1	3	negative regulation of mitophagy	BP	1	CLEC16A	up_AFEM_EC
GO:1902459	0.0696	0.998	1	3	positive regulation of stem cell population	BP	1	LBH	up_AFEM_EC
GO:2000832	0.0696	0.998	1	3	negative regulation of steroid hormone	BP	1	CRY2	up_AFEM_EC
GO:2000846	0.0696	0.998	1	3	regulation of corticosteroid hormone	BP	1	CRY2	up_AFEM_EC
GO:2000847	0.0696	0.998	1	3	negative regulation of corticosteroid hormone	BP	1	CRY2	up_AFEM_EC

GO:2000849	0.0696	0.998	1	3	regulation of glucocorticoid secretion	BP	1	CRY2	up_AFEM_EC
GO:2000850	0.0696	0.998	1	3	negative regulation of glucocorticoid secretion	BP	1	CRY2	up_AFEM_EC
GO:0051055	0.0696	0.998	1	21	negative regulation of lipid biosynthetic process	BP	1	NR1H4	up_VPUL_EC
GO:0044088	0.069664821	0.99124	2	17	regulation of vacuole organization	BP	1	CHMP4B,VPS16	up_ACOR_EC
GO:0033365	0.0697	0.957	23	276	protein localization to organelle	BP	0.874	PRICKLE1,HSPA5,TRAM1,HOOK3,SESN2,SRP54,MSN,ZPR1,SRPRA,KPNB1,SRP72,KPNA4,DDIT3,GSK3B,GBP5,SH3GLB1,KPNA1,KDELR2,SIX4,CDKN1A,NRP1,ARF4,MID1	down_VSAP_EC
GO:1903078	0.0697	0.986	3	19	positive regulation of protein localization to	BP	1	LRP1,PRNP,CLIP3	down_VCAV_EC
GO:0032060	0.0697	0.993	2	6	bleb assembly	BP	1	ANLN,EMP2	down_ACOR_VSMC
GO:0043584	0.0697	0.993	2	5	nose development	BP	0.905	CHD7,ALDH1A3	up_VCAV_EC
GO:0045061	0.0697	0.993	2	6	thymic T cell selection	BP	0.905	GATA3,ITPKB	up_VCAV_EC
GO:0042509	0.0697	0.998	1	17	regulation of tyrosine phosphorylation of STAT	BP	1	LYN	down_ACOR_EC
GO:0060512	0.0697	0.998	1	14	prostate gland morphogenesis	BP	1	SULF1	down_ACOR_EC
GO:0006487	0.0697	0.998	1	34	protein N-linked glycosylation	BP	1	MGAT4B	down_VPOR_EC
GO:0060509	0.0697	0.998	1	3	type I pneumocyte differentiation	BP	1	THRB	up_AFEM_EC
GO:0071493	0.0697	0.998	1	3	cellular response to UV-B	BP	1	MFAP4	up_AFEM_EC
GO:2000726	0.0697	0.998	1	3	negative regulation of cardiac muscle cell	BP	1	DLL1	up_AFEM_EC
GO:0071772	0.0697	0.998	1	50	response to BMP	BP	1	GATA5	up_VCAV_VSMC
GO:0071773	0.0697	0.998	1	50	cellular response to BMP stimulus	BP	1	GATA5	up_VCAV_VSMC
GO:0007338	0.0697	0.998	1	16	single fertilization	BP	1	HOXA9	up_VFEM_EC
GO:0048844	0.0697	0.998	1	30	artery morphogenesis	BP	1	EYA1	up_VFEM_VSMC
GO:0006626	0.0698	0.978	5	29	protein targeting to mitochondrion	BP	1	SREBF1,TIMM44,TOMM34,SAMM50,PMPCA	down_ACOR_VSMC
GO:2000310	0.0698	0.994	2	4	regulation of NMDA receptor activity	BP	0.905	RELN,MEF2C	up_VCAV_EC
GO:0016024	0.0698	0.998	1	3	CDP-diacylglycerol biosynthetic process	BP	1	GPAM	up_AFEM_EC
GO:0046341	0.0698	0.998	1	3	CDP-diacylglycerol metabolic process	BP	1	GPAM	up_AFEM_EC
GO:0006766	0.0698	0.998	1	16	vitamin metabolic process	BP	1	CYP26B1	up_VFEM_EC
GO:0042310	0.0698	0.998	1	21	vasoconstriction	BP	1	HTR7	up_VPUL_EC
GO:0097756	0.0698	0.998	1	21	negative regulation of blood vessel diameter	BP	1	HTR7	up_VPUL_EC
GO:0007530	0.0699	0.993	2	5	sex determination	BP	1	CITED2,PTGDR	up_ACOR_VSMC
GO:0097529	0.0699	0.991	2	52	myeloid leukocyte migration	BP	1	C3AR1,ENSCAFG00000032731	up_VPOR_EC
GO:1900028	0.0699	0.998	1	3	negative regulation of ruffle assembly	BP	1	KANK1	up_AFEM_EC
GO:0006924	0.0699	0.998	1	3	activation-induced cell death of T cells	BP	1	GPAM	up_AFEM_EC
GO:0019336	0.0699	0.998	1	3	phenol-containing compound catabolic	BP	1	MOXD1	up_AFEM_EC
GO:0019614	0.0699	0.998	1	3	catechol-containing compound catabolic	BP	1	MOXD1	up_AFEM_EC
GO:0042420	0.0699	0.998	1	3	dopamine catabolic process	BP	1	MOXD1	up_AFEM_EC
GO:0042424	0.0699	0.998	1	3	catecholamine catabolic process	BP	1	MOXD1	up_AFEM_EC
GO:2001214	0.0699	0.998	1	3	positive regulation of vasculogenesis	BP	1	TMEM100	up_AFEM_EC
GO:0021859	0.069952341	0.99833	1	3	pyramidal neuron differentiation	BP	1	DCLK2	up_ACOR_EC
GO:0021860	0.069952341	0.99833	1	3	pyramidal neuron development	BP	1	DCLK2	up_ACOR_EC
GO:0044272	0.07	0.975	6	35	sulfur compound biosynthetic process	BP	1	ACLY,XYLT1,ELOVL1,ELOVL6,CTH,PDHA1	down_ACOR_VSMC
GO:0050663	0.07	0.998	1	52	cytokine secretion	BP	1	TLR2	up_AFEM_VSMC

GO:0051651	0.07	0.998	1	31	maintenance of location in cell	BP	1	ENSCAFG00000005494	up_AORT_EC
GO:0097530	0.07	0.998	1	33	granulocyte migration	BP	1	C3AR1	up_VPOR_VSMC
GO:0019883	0.0701	0.993	2	5	antigen processing and presentation of endogenous antigen	BP	1	B2M,ERAP1	up_ACOR_VSMC
GO:0006024	0.0701	0.998	1	16	glycosaminoglycan biosynthetic process	BP	1	CSGALNACT1	down_ACOR_EC
GO:0019395	0.0701	0.998	1	33	fatty acid oxidation	BP	1	ETFA	down_AFEM_EC
GO:0003257	0.0701	0.998	1	3	positive regulation of transcription from RNA polymerase II promoter involved in myocardial precursor cell	BP	1	GREM1	up_AFEM_EC
GO:0042104	0.0701	0.998	1	3	positive regulation of activated T cell	BP	1	GPAM	up_AFEM_EC
GO:0051446	0.0701	0.998	1	3	positive regulation of meiotic cell cycle	BP	1	OVOL1	up_AFEM_EC
GO:0051346	0.0702	0.965	12	78	negative regulation of hydrolase activity	BP	1	TNNT2,RECK,SH3RF2,CST3,SH3RF1,MARF1,PPP1R26,SPINK2,SH3BP4,PARK7,GPATCH2,ZFYVE1	up_ACOR_VSMC
GO:0046104	0.0702	1	1	1	thymidine metabolic process	BP	1	TK1	down_ACOR_VSMC
GO:0046125	0.0702	1	1	1	pyrimidine deoxyribonucleoside	BP	1	TK1	down_ACOR_VSMC
GO:0007271	0.0702	0.998	1	3	synaptic transmission, cholinergic	BP	1	CHRM2	up_AFEM_EC
GO:0033600	0.0702	1	1	1	negative regulation of mammary gland epithelial	BP	0.905	GATA3	up_VCAV_EC
GO:0035799	0.0702	1	1	1	ureter maturation	BP	0.905	GATA3	up_VCAV_EC
GO:0060995	0.0702	1	1	1	cell-cell signaling involved in kidney development	BP	0.905	GATA3	up_VCAV_EC
GO:0061289	0.0702	1	1	1	Wnt signaling pathway involved in kidney	BP	0.905	GATA3	up_VCAV_EC
GO:0061290	0.0702	1	1	1	canonical Wnt signaling pathway involved in metanephric kidney	BP	0.905	GATA3	up_VCAV_EC
GO:0071442	0.0702	1	1	1	positive regulation of histone H3-K14	BP	0.905	GATA3	up_VCAV_EC
GO:0071481	0.0702	1	1	1	cellular response to X-ray	BP	0.905	GATA3	up_VCAV_EC
GO:0072204	0.0702	1	1	1	cell-cell signaling involved in metanephros	BP	0.905	GATA3	up_VCAV_EC
GO:1901536	0.0702	1	1	1	negative regulation of DNA demethylation	BP	0.905	GATA3	up_VCAV_EC
GO:1905277	0.0702	1	1	1	negative regulation of epithelial tube formation	BP	0.905	GATA3	up_VCAV_EC
GO:2000606	0.0702	1	1	1	regulation of cell proliferation involved in mesonephros	BP	0.905	GATA3	up_VCAV_EC
GO:2000607	0.0702	1	1	1	negative regulation of cell proliferation involved in mesonephros	BP	0.905	GATA3	up_VCAV_EC
GO:2000683	0.0702	1	1	1	regulation of cellular response to X-ray	BP	0.905	GATA3	up_VCAV_EC
GO:2000699	0.0702	1	1	1	fibroblast growth factor receptor signaling pathway involved in	BP	0.905	GATA3	up_VCAV_EC
GO:2000701	0.0702	1	1	1	glial cell-derived neurotrophic factor receptor signaling pathway involved in	BP	0.905	GATA3	up_VCAV_EC
GO:2000702	0.0702	1	1	1	regulation of fibroblast growth factor receptor signaling pathway involved in ureteric bud	BP	0.905	GATA3	up_VCAV_EC
GO:2000703	0.0702	1	1	1	negative regulation of fibroblast growth factor receptor signaling pathway involved in	BP	0.905	GATA3	up_VCAV_EC
GO:2000733	0.0702	1	1	1	regulation of glial cell-derived neurotrophic factor receptor signaling pathway involved in	BP	0.905	GATA3	up_VCAV_EC

GO:2000734	0.0702	1	1	1	negative regulation of glial cell-derived neurotrophic factor receptor signaling pathway involved in	BP	0.905	GATA3	up_VCAV_EC
GO:0010573	0.0702	0.998	1	9	vascular endothelial growth factor production	BP	1	C3AR1	up_VPOR_EC
GO:0010574	0.0702	0.998	1	9	regulation of vascular endothelial growth factor	BP	1	C3AR1	up_VPOR_EC
GO:0032623	0.0702	0.998	1	21	interleukin-2 production	BP	1	NR1H4	up_VPUL_EC
GO:0050663	0.0703	0.971	8	52	cytokine secretion	BP	0.905	GBP5,FERMT1,TLR4,GATA3,ARG2,ABCA1,HMGB1,NOS2	up_VCAV_EC
GO:0006278	0.0703	0.978	5	26	RNA-dependent DNA biosynthetic process	BP	1	SLC29A1,AURKB,NEK2,TINF2,CCT4	down_ACOR_VSMC
GO:0007004	0.0703	0.978	5	26	telomere maintenance via telomerase	BP	1	SLC29A1,AURKB,NEK2,TINF2,CCT4	down_ACOR_VSMC
GO:0031297	0.0703	0.982	4	18	replication fork processing	BP	1	RAD51,TONSL,SMARCAL1,GEN1	down_ACOR_VSMC
GO:0050768	0.0703	0.98	4	66	negative regulation of neurogenesis	BP	1	IRX3,DLL1,THRB,KANK1	up_AFEM_EC
GO:0044275	0.0703	0.987	3	10	cellular carbohydrate catabolic process	BP	1	ENOSF1,PYGM,GAA	up_ACOR_VSMC
GO:0022604	0.0703	0.985	3	122	regulation of cell morphogenesis	BP	1	ENSCAFG00000002015,SEMA3A,RNF6	up_VPOR_EC
GO:0072498	0.0703	0.992	2	8	embryonic skeletal joint development	BP	0.874	CTNNB1,EXT1	down_VSAP_EC
GO:0150065	0.0703	0.993	2	5	regulation of deacetylase activity	BP	0.905	PRKD2,KDM5A	up_VCAV_EC
GO:0030309	0.0703	1	1	1	poly-N-acetyllactosamine metabolic process	BP	1	B3GNT2	down_ACOR_VSMC
GO:0030311	0.0703	1	1	1	poly-N-acetyllactosamine biosynthetic process	BP	1	B3GNT2	down_ACOR_VSMC
GO:0001843	0.0703	0.998	1	38	neural tube closure	BP	1	CITED2	down_VPOR_EC
GO:0001921	0.0703	0.998	1	3	positive regulation of receptor recycling	BP	1	BVES	up_AFEM_EC
GO:0032693	0.0703	0.998	1	3	negative regulation of interleukin-10 production	BP	1	DLL1	up_AFEM_EC
GO:0097006	0.0703	0.998	1	19	regulation of plasma lipoprotein particle levels	BP	1	NR1H4	up_APUL_EC
GO:0051239	0.070325301	0.95385	30	859	regulation of multicellular organismal process	BP	1	CDH3,NR1H4,BRSK1,PLCL1,SOX9,NDRG2,AQP1,FZD5,GNAO1,DIXDC1,NOD2,IL33,RGMA,SMAD6,PLS1,RNLS,PTGDS,PAK3,PTGER4,ARHGDIB,MAVS,FOXC2,TBX5,LRP4,CLSTN1,NRP1,AGPAT2,PRKCD,PYGO2,EFEMP1	up_ACOR_EC
GO:2000145	0.0704	0.985	3	279	regulation of cell motility	BP	1	TBX5,CCL24,ARHGEF2	up_VPUL_EC
GO:0022613	0.0704	0.991	2	208	ribonucleoprotein complex biogenesis	BP	1	EIF3M,SRSF6	down_AFEM_EC
GO:0009713	0.0704	0.993	2	5	catechol-containing compound biosynthetic	BP	1	MOXD1,PARK7	up_ACOR_VSMC
GO:0042423	0.0704	0.993	2	5	catecholamine biosynthetic process	BP	1	MOXD1,PARK7	up_ACOR_VSMC
GO:0050651	0.0704	0.998	1	3	dermatan sulfate proteoglycan biosynthetic	BP	1	CSGALNACT2	up_AFEM_EC
GO:0045617	0.0704	0.998	1	3	negative regulation of keratinocyte	BP	1	HOXA7	up_AFEM_EC
GO:0051930	0.0704	0.998	1	3	regulation of sensory perception of pain	BP	1	TMEM100	up_AFEM_EC
GO:0051931	0.0704	0.998	1	3	regulation of sensory perception	BP	1	TMEM100	up_AFEM_EC
GO:0043330	0.0704	0.998	1	16	response to exogenous dsRNA	BP	1	CAV1	up_VSAP_VSMC
GO:0035904	0.0705	0.978	5	25	aorta development	BP	0.905	ADAMTS9,CHD7,DLL4,PROX1,JAG1	up_VCAV_EC
GO:0048736	0.0705	0.98	4	66	appendage development	BP	1	HOXA9,HOXC10,GREM1,RECK	up_AFEM_EC
GO:0060173	0.0705	0.98	4	66	limb development	BP	1	HOXA9,HOXC10,GREM1,RECK	up_AFEM_EC
GO:0000186	0.0705	0.987	3	10	activation of MAPKK activity	BP	1	BMP4,PSEN1,MAP3K7	up_ACOR_VSMC
GO:2000737	0.0705	0.992	2	7	negative regulation of stem cell differentiation	BP	0.874	PRICKLE1,REST	down_VSAP_EC
GO:0001501	0.0705	0.991	2	178	skeletal system development	BP	1	HOXA3,CYP26B1	up_VPOR_VSMC
GO:0050767	0.0705	0.991	2	184	regulation of neurogenesis	BP	1	EYA1,SHOX2	up_VFEM_VSMC
GO:0045656	0.0705	0.998	1	3	negative regulation of monocyte differentiation	BP	1	HOXA7	up_AFEM_EC

GO:0072047	0.0705	0.998	1	3	proximal/distal pattern formation involved in	BP	1	IRX3	up_AFEM_EC
GO:0072081	0.0705	0.998	1	3	specification of nephron tubule identity	BP	1	IRX3	up_AFEM_EC
GO:1901605	0.0705	0.998	1	67	alpha-amino acid metabolic process	BP	1	ARG2	up_AORT_VSMC
GO:0006367	0.0705	0.998	1	32	transcription initiation from RNA polymerase II	BP	1	NKX2-5	up_VPOR_VSMC
GO:0045667	0.0706	0.973	7	38	regulation of osteoblast differentiation	BP	1	SMAD6,RASSF2,BMP4,GJA1,CLSTN1,NPNT,DDR2	up_ACOR_VSMC
GO:0048844	0.0706	0.975	6	30	artery morphogenesis	BP	1	BMP4,SEC24B,CITED2,NRP1,CHD7,HPGD	up_ACOR_VSMC
GO:0030325	0.0706	0.993	2	5	adrenal gland development	BP	1	WNT11,CITED2	up_VSAP_EC
GO:0090191	0.0706	0.998	1	3	negative regulation of branching involved in ureteric bud	BP	1	GREM1	up_AFEM_EC
GO:0048592	0.0706	0.998	1	53	eye morphogenesis	BP	1	IRX5	up_AFEM_VSMC
GO:0046167	0.0706	1	1	1	glycerol-3-phosphate biosynthetic process	BP	0.905	GK5	up_VCAV_EC
GO:0035725	0.0706	0.998	1	16	sodium ion transmembrane transport	BP	1	NETO1	up_VSAP_VSMC
GO:0001780	0.0707	0.993	2	6	neutrophil homeostasis	BP	0.905	ITPKB,HMGB1	up_VCAV_EC
GO:2001243	0.0707	0.998	1	33	negative regulation of intrinsic apoptotic	BP	1	IVNS1ABP	down_AFEM_EC
GO:0051240	0.0707	1	1	489	positive regulation of multicellular organismal	BP	1	GATA5	down_AFEM_VSMC
GO:0051336	0.0707	0.999	1	251	regulation of hydrolase activity	BP	1	CST3	down_AORT_EC
GO:0043435	0.0707	1	1	1	response to corticotropin-releasing hormone	BP	0.874	NR4A1	down_VSAP_EC
GO:0071376	0.0707	1	1	1	cellular response to corticotropin-releasing	BP	0.874	NR4A1	down_VSAP_EC
GO:0060216	0.0707	0.998	1	3	definitive hemopoiesis	BP	1	HOXA9	up_AFEM_EC
GO:0043255	0.0707	0.998	1	29	regulation of carbohydrate biosynthetic	BP	1	FBP1	up_VFEM_VSMC
GO:0030239	0.0707	0.998	1	21	myofibril assembly	BP	1	MYBPC2	up_VPUL_EC
GO:2000736	0.0707	0.998	1	21	regulation of stem cell differentiation	BP	1	TBX5	up_VPUL_EC
GO:0032637	0.0708	0.978	5	26	interleukin-8 production	BP	0.905	PRKD2,TLR3,BCL10,NOS2,RIPK1	up_VCAV_EC
GO:0051051	0.0708	0.991	2	112	negative regulation of transport	BP	1	NR1H4,HOXA3	up_APUL_EC
GO:0051561	0.0708	0.993	2	6	positive regulation of mitochondrial calcium ion concentration	BP	0.905	MCU,ATP2A1	up_VCAV_EC
GO:0051136	0.0708	0.993	2	5	regulation of NK T cell differentiation	BP	1	ZBTB16,TGFBR2	up_VSAP_EC
GO:1904672	0.0708	0.998	1	3	regulation of somatic stem cell population	BP	1	LBH	up_AFEM_EC
GO:0001824	0.0709	0.978	5	27	blastocyst development	BP	1	GINS4,NEK2,SMARCB1,RBBP8,EMG1	down_ACOR_VSMC
GO:0051560	0.0709	0.987	3	12	mitochondrial calcium ion homeostasis	BP	0.905	PDZD8,MCU,ATP2A1	up_VCAV_EC
GO:0009117	0.0709	0.991	2	208	nucleotide metabolic process	BP	1	SUCLG2,NDUFA10	down_AFEM_EC
GO:0070233	0.0709	0.992	2	9	negative regulation of T cell apoptotic process	BP	1	GPAM,TSC22D3	down_VCAV_EC
GO:0010628	0.0709	0.993	2	527	positive regulation of gene expression	BP	1	GATA5,TGFBR1	down_VSAP_VSMC
GO:0042692	0.0709	0.991	2	105	muscle cell differentiation	BP	1	SIX1,CYP26B1	up_VFEM_EC
GO:1903044	0.0709	0.998	1	3	protein localization to membrane raft	BP	1	REEP2	up_AFEM_EC
GO:0001885	0.0709	0.998	1	19	endothelial cell development	BP	1	ARHGEF26	up_APUL_EC
GO:0043242	0.0709	0.998	1	21	negative regulation of protein complex	BP	1	ARHGEF2	up_VPUL_EC
GO:0048519	0.071	0.979	6	1554	negative regulation of biological process	BP	1	HOXA3,TBX5,GATA5,TMEM176A,TMEM176B,SLIT2	up_VPUL_VSMC
GO:0009206	0.071	0.977	5	39	purine ribonucleoside triphosphate biosynthetic	BP	0.874	PGK1,TPI1,PGAM1,HIF1A,ALDOC	down_VSAP_EC
GO:0006592	0.071	1	1	1	ornithine biosynthetic process	BP	0.874	ALDH18A1	down_VSAP_EC
GO:0140115	0.071	0.998	1	3	export across plasma membrane	BP	1	FXYD1	up_AFEM_EC

GO:0072507	0.071	0.998	1	103	divalent inorganic cation homeostasis	BP	1	HOXA3	up_APUL_VSMC
GO:0071606	0.071	1	1	1	chemokine (C-C motif) ligand 4 production	BP	0.905	ARG2	up_VCAV_EC
GO:0071608	0.071	1	1	1	macrophage inflammatory protein-1	BP	0.905	ARG2	up_VCAV_EC
GO:0071640	0.071	1	1	1	regulation of macrophage inflammatory protein 1 alpha production	BP	0.905	ARG2	up_VCAV_EC
GO:0071641	0.071	1	1	1	negative regulation of macrophage inflammatory protein 1	BP	0.905	ARG2	up_VCAV_EC
GO:0071643	0.071	1	1	1	regulation of chemokine (C-C motif) ligand 4	BP	0.905	ARG2	up_VCAV_EC
GO:0071644	0.071	1	1	1	negative regulation of chemokine (C-C motif)	BP	0.905	ARG2	up_VCAV_EC
GO:1905076	0.071	1	1	1	regulation of interleukin-17 secretion	BP	0.905	ARG2	up_VCAV_EC
GO:1905077	0.071	1	1	1	negative regulation of interleukin-17 secretion	BP	0.905	ARG2	up_VCAV_EC
GO:1905403	0.071	1	1	1	negative regulation of activated CD8-positive, alpha-beta T cell	BP	0.905	ARG2	up_VCAV_EC
GO:2000666	0.071	1	1	1	negative regulation of interleukin-13 secretion	BP	0.905	ARG2	up_VCAV_EC
GO:0006110	0.071	0.998	1	9	regulation of glycolytic process	BP	1	FBP1	up_VPOR_EC
GO:0030811	0.071	0.998	1	9	regulation of nucleotide catabolic process	BP	1	FBP1	up_VPOR_EC
GO:0043470	0.071	0.998	1	9	regulation of carbohydrate catabolic	BP	1	FBP1	up_VPOR_EC
GO:0032984	0.0711	0.967	10	70	protein-containing complex disassembly	BP	1	CAPG,LIMA1,STMN1,SMARCC1,EIF5A,PDXP,SMARCB1,GRWD1,ARHGEF2,KIF24	down_ACOR_VSMC
GO:0021515	0.0711	0.992	2	9	cell differentiation in spinal cord	BP	1	LMO4,GLI3	down_VCAV_EC
GO:0044249	0.0711	1	2	1851	cellular biosynthetic process	BP	1	JUP,GATA5	down_VFEM_VSMC
GO:0007213	0.0711	0.993	2	5	G protein-coupled acetylcholine receptor	BP	1	AGRN,GNAQ	up_ACOR_VSMC
GO:0034141	0.0711	0.993	2	5	positive regulation of toll-like receptor 3 signaling	BP	1	F2RL1,PTPN22	up_ACOR_VSMC
GO:0007034	0.0711	0.991	2	55	vacuolar transport	BP	1	HOOK3,GCC2	up_VPOR_EC
GO:2001253	0.0711	1	1	1	regulation of histone H3-K36 trimethylation	BP	0.874	IWS1	down_VSAP_EC
GO:0051901	0.0711	0.998	1	3	positive regulation of mitochondrial	BP	1	MLLT11	up_AFEM_EC
GO:1904181	0.0711	0.998	1	3	positive regulation of membrane depolarization	BP	1	MLLT11	up_AFEM_EC
GO:0060538	0.0711	0.998	1	51	skeletal muscle organ development	BP	1	BEND2	up_AFEM_VSMC
GO:0055017	0.0711	0.998	1	19	cardiac muscle tissue growth	BP	1	TBX5	up_APUL_EC
GO:0042692	0.0711	0.998	1	105	muscle cell differentiation	BP	1	TBX5	up_APUL_VSMC
GO:0010939	0.0711	0.998	1	9	regulation of necrotic cell death	BP	1	ARHGEF2	up_VPOR_EC
GO:0070861	0.0711	0.998	1	9	regulation of protein exit from endoplasmic	BP	1	GCC2	up_VPOR_EC

GO:0050789	0.0712	0.942	214	3295	regulation of biological process	BP	0.874	ARHGEF2,PRICKLE1,VEGFA,P4HB,SIPA1L1,SULF1,ARHGAP31,CELF1,ASNS,MYH9,NR1H4,FMNL3,PLK3,ENSCAFG00000013214,HSPA5,SERPINE1,ACTN1,SOCS5,ETV6,SPARC,MEIS2,SGK1,ERO1A,PRKACB,PTPN14,ENPP1,ERRFI1,ITGA2,JCAD,SNX25,CBX5,CASP3,FAM49B,ENSCAFG00000003477,HYOU1,PGK1,AKAP2,WSB1,BCAR3,NUAK1,PTPN11,FSCN1,AARS,DPYSL3,LRRC8A,HOOK3,TRIB3,PGAM1,IFRD1,FARP1,TMED2,PUM3,PM20D2,FBLIM1,ILK,FHL2,BHLHE40,PPP1R12A,PRCP,SMARCA4,ANKRA2,SESN2,TGFBR1,PTGS2,REM1,WDR1,MYO1C,PIK3C2A,CHSY1,MSN,UACA,HOXB7,KIF5B,LMAN1,HIF1A,CX3CL1,ENSCAFG00000000562,DYNLL1,SLC45A3,TUBB,IWS1,THBS2,DNAJC10,YIPF5,ACTG2,TNIK,NAA10,NXN,RGS2,ZPR1,PTPN2,UCHL5,MORF4L2,SPOPL,EIF2B1,EVI2B,PVR,YES1,NEO1,PLCL1,PEG3,SMG7,MTDH,ESM1,DDX39B,ADAM17,AGRN,ACTA2,BRD2,EIF4EBP1,SKAP2,UST,PDIA4,CNOT7,CDH11,ACTR3,ADGRE5,MAGED1,GNAQ,CTNNB1,KPNB1,ANLN,CRYAB,STARD13,SND1,ENSCAFG00000002015,TFRC,CDK8,APOE,PTP4A1,WWC3,PDE1A,ZC3H15,IGFBP7,KLF5,UBA5,MMP28,LYN,BNIP3,STRAP,DDIT3,TWF1,PDS5B,FAM162A,BACH1,CCNYL1,PLEKHG2,TLN1,EIF5,MMD	down_VSAP_EC
GO:0071214	0.0712	0.964	12	93	cellular response to abiotic stimulus	BP	0.906	LRRC8D,GATA3,FIGNL1,TMEM150C,CASP8,MAPK13,TLR3,PTGS2,BCL10,USP28,BLM,ARHGEF2	up_VCAV_EC
GO:0104004	0.0712	0.964	12	93	cellular response to environmental stimulus	BP	0.906	LRRC8D,GATA3,FIGNL1,TMEM150C,CASP8,MAPK13,TLR3,PTGS2,BCL10,USP28,BLM,ARHGEF2	up_VCAV_EC
GO:2000345	0.0712	0.993	2	5	regulation of hepatocyte proliferation	BP	1	LIMS2,SULF2	up_VSAP_EC
GO:0043039	0.0712	0.998	1	33	tRNA aminoacylation	BP	1	DARS	down_AFEM_EC
GO:0061041	0.0712	0.998	1	34	regulation of wound healing	BP	1	SRSF6	down_AFEM_EC
GO:0002248	0.0712	1	1	1	connective tissue replacement involved in inflammatory response	BP	0.874	HIF1A	down_VSAP_EC
GO:0002069	0.0712	1	1	1	columnar/cuboidal epithelial cell maturation	BP	0.874	HIF1A	down_VSAP_EC
GO:0051541	0.0712	1	1	1	elastin metabolic process	BP	0.874	HIF1A	down_VSAP_EC
GO:0060574	0.0712	1	1	1	intestinal epithelial cell maturation	BP	0.874	HIF1A	down_VSAP_EC
GO:0061030	0.0712	1	1	1	epithelial cell differentiation involved in mammary gland alveolus	BP	0.874	HIF1A	down_VSAP_EC
GO:0007021	0.0712	0.998	1	3	tubulin complex assembly	BP	1	CRYAB	up_AFEM_EC
GO:0001738	0.0712	0.998	1	27	morphogenesis of a polarized epithelium	BP	1	ANKRD6	up_VPOR_VSMC
GO:0030216	0.0712	0.998	1	29	keratinocyte differentiation	BP	1	CYP26B1	up_VPOR_VSMC
GO:0048640	0.0712	0.998	1	21	negative regulation of developmental growth	BP	1	TBX5	up_VPUL_EC
GO:1904659	0.071290267	0.98483	3	36	glucose transmembrane transport	BP	1	NR1H4,ENSCAFG00000004331,RARRES2	up_ACOR_EC
GO:0043254	0.0713	0.961	15	164	regulation of protein complex assembly	BP	0.874	VEGFA,PTPN11,FSCN1,FBLIM1,ANKRA2,MSN,ACTR3,CTNNB1,CRYAB,APOE,TWF1,PLEKHG2,GSK3B,GBP5,CAPG	down_VSAP_EC
GO:0022029	0.0713	0.978	5	23	telencephalon cell migration	BP	1	FGF13,NRP1,PSEN1,DISC1,SUN1	up_ACOR_VSMC
GO:2001256	0.0713	0.993	2	5	regulation of store-operated calcium entry	BP	1	SARAF,STC2	up_ACOR_VSMC
GO:0050920	0.0713	0.991	2	53	regulation of chemotaxis	BP	1	C3AR1,SEMA3A	up_VPOR_EC
GO:0035624	0.0713	1	1	1	receptor transactivation	BP	0.874	ADAM17	down_VSAP_EC
GO:0051088	0.0713	1	1	1	PMA-inducible membrane protein ectodomain	BP	0.874	ADAM17	down_VSAP_EC
GO:0071403	0.0713	1	1	1	cellular response to high density lipoprotein	BP	0.874	ADAM17	down_VSAP_EC
GO:0050999	0.0713	0.998	1	9	regulation of nitric-oxide synthase activity	BP	1	ACVR2A	up_VPOR_EC
GO:2001257	0.0713	0.998	1	30	regulation of cation channel activity	BP	1	NETO1	up_VFEM_VSMC

GO:0070304	0.071310322	0.98017	4	58	positive regulation of stress-activated protein kinase signaling cascade	BP	1	FZD5,NOD2,TNFRSF19,SH3RF3	up_ACOR_EC
GO:0043549	0.0714	0.956	21	244	regulation of kinase activity	BP	0.874	VEGFA,SOCS5,ERRFI1,PTPN11,TRIB3,SESN2,TNIK,PTPN2,ADAM17,MAGED1,APOE,LYN,CCNYL1,MMD,CBLC,VLDLR,SH3GLB1,HTT,PAK2,CDKN1A,MADD	down_VSAP_EC
GO:0070663	0.0714	0.974	6	50	regulation of leukocyte proliferation	BP	0.874	CTNNB1,TFRC,LYN,SDC4,BTK,CDKN1A	down_VSAP_EC
GO:0021782	0.0714	0.978	5	23	glial cell development	BP	1	SH3TC2,WASF3,DAG1,APP,PSEN1	up_ACOR_VSMC
GO:0002009	0.0714	0.985	3	177	morphogenesis of an epithelium	BP	1	ACVRL1,PDPN,SULF1	down_ACOR_EC
GO:0009948	0.0714	0.986	3	16	anterior/posterior axis specification	BP	0.874	TMED2,CTNNB1,DDIT3	down_VSAP_EC
GO:0045935	0.0714	1	1	491	positive regulation of nucleobase-containing compound metabolic	BP	1	GATA5	down_AFEM_VSMC
GO:0051892	0.0714	1	1	1	negative regulation of cardioblast differentiation	BP	0.874	PRICKLE1	down_VSAP_EC
GO:2000690	0.0714	1	1	1	regulation of cardiac muscle cell myoblast	BP	0.874	PRICKLE1	down_VSAP_EC
GO:2000691	0.0714	1	1	1	negative regulation of cardiac muscle cell myoblast differentiation	BP	0.874	PRICKLE1	down_VSAP_EC
GO:0006813	0.071414465	0.98478	3	37	potassium ion transport	BP	1	KCNB1,ACTN2,SLC9A9	up_ACOR_EC
GO:0019755	0.071463191	0.99825	1	3	one-carbon compound transport	BP	1	AQP1	up_ACOR_EC
GO:0009790	0.0715	0.955	23	335	embryo development	BP	1	SDC4,FBN1,BMPR1A,LMO4,RECK,COL6A1,NAGLU,TBX15,HOXC4,LAMA3,SEMA3C,TEAD1,TLX2,GLI3,ZFP36L1,IFT57,LOXL3,IRX5,PRKCSH,RDH10,PCSK6,GREM1,ZBTB16	down_VCAV_EC
GO:0050877	0.0715	0.956	22	164	nervous system process	BP	1	FGF13,MBP,COCH,HRH1,LRRN4,WASF3,GLRB,GPR155,TMEM108,USP53,ABCA4,DMPK,POMK,APP,PSEN1,CHD7,CLN8,FAM107A,INSYN1,JAM3,GAA,WFS1	up_ACOR_VSMC
GO:0072657	0.0715	0.962	13	144	protein localization to membrane	BP	0.874	TRAM1,EXOC5,SRP54,MYO1C,KIF5B,SSR1,TNIK,SRPRA,AGRN,SRP72,APOE,SH3GLB1,BLZF1	down_VSAP_EC
GO:0030852	0.0715	0.992	2	6	regulation of granulocyte differentiation	BP	0.909	HCLS1,CUL4A	up_VCAV_EC
GO:0010557	0.0715	1	1	492	positive regulation of macromolecule biosynthetic process	BP	1	GATA5	down_AFEM_VSMC
GO:0045667	0.0715	0.998	1	38	regulation of osteoblast differentiation	BP	1	HDAC7	down_VPOR_EC
GO:1900084	0.0715	0.998	1	3	regulation of peptidyl-tyrosine	BP	1	GREM1	up_AFEM_EC
GO:0010927	0.0715	0.998	1	28	cellular component assembly involved in	BP	1	NKX2-5	up_VPOR_VSMC
GO:0048167	0.0715	0.998	1	28	regulation of synaptic plasticity	BP	1	NETO1	up_VFEM_VSMC
GO:0098657	0.0716	0.961	14	181	import into cell	BP	1	SNX18,LRP1,PRNP,FOLR2,CLIP3,SLC39A6,ANXA11,LRPAP1,CLTB,PARK7,CYBA,RAB31,GREM1,PACSIN3	down_VCAV_EC
GO:0010647	0.0716	0.977	5	460	positive regulation of cell communication	BP	1	ACVRL1,LYN,SULF1,STX4,NEDD4	down_ACOR_EC
GO:0060996	0.0716	0.981	4	26	dendritic spine development	BP	0.874	UBA6,APOE,PAK2,ARF4	down_VSAP_EC
GO:0048662	0.0716	0.982	4	18	negative regulation of smooth muscle cell	BP	1	PTGIR,NDRG2,CDKN1A,CLSTN1	up_ACOR_VSMC
GO:0060041	0.0716	0.985	3	41	retina development in camera-type eye	BP	1	IRX5,DLL1,THRB	up_AFEM_EC
GO:0048878	0.0716	0.985	3	283	chemical homeostasis	BP	1	HOXA3,NR1H4,NEO1	up_VPUL_EC
GO:0007266	0.0717	0.967	10	64	Rho protein signal transduction	BP	1	ARHGEF5,ARHGEF6,ABL1,CDC42EP5,CDC42EP2,F2RL1,TIAM1,DLC1,FGD4,FLOT1	up_VSAP_EC
GO:0032006	0.0717	0.968	9	52	regulation of TOR signaling	BP	1	LEP,PRKAA2,CASTOR1,UBR1,SESN3,SESN1,DDIT4,BMT2,SH3BP4	up_ACOR_VSMC
GO:1902652	0.0717	0.972	7	38	secondary alcohol metabolic process	BP	1	LEP,LCAT,ABCA1,LIPE,APP,CLN8,LMF1	up_ACOR_VSMC
GO:0010638	0.0717	0.991	2	207	positive regulation of organelle organization	BP	1	GPSM2,FSCN1	down_AFEM_EC
GO:0001568	0.0717	0.999	1	252	blood vessel development	BP	1	JUP	down_VFEM_VSMC
GO:0110111	0.0717	0.998	1	17	negative regulation of animal organ	BP	1	SIX1	up_VFEM_EC
GO:0006140	0.0717	0.998	1	28	regulation of nucleotide metabolic process	BP	1	FBP1	up_VFEM_VSMC

GO:0050866	0.0718	0.974	6	48	negative regulation of cell activation	BP	0.874	SOCS5,PTPN2,APOE,LYN,SDC4,BTK	down_VSAP_EC
GO:0051094	0.0718	0.992	2	388	positive regulation of developmental process	BP	1	WNT7A,ARG2	up_AORT_VSMC
GO:0002260	0.0718	0.998	1	15	lymphocyte homeostasis	BP	1	LYN	down_ACOR_EC
GO:0055072	0.0718	0.998	1	30	iron ion homeostasis	BP	1	ENSCAFG00000005494	up_AORT_EC
GO:0015691	0.0718	1	1	1	cadmium ion transport	BP	0.91	SLC39A8	up_VCAV_EC
GO:0070574	0.0718	1	1	1	cadmium ion transmembrane transport	BP	0.91	SLC39A8	up_VCAV_EC
GO:0045604	0.0718	0.998	1	17	regulation of epidermal cell differentiation	BP	1	HOXA7	up_VFEM_EC
GO:0050886	0.0718	0.998	1	17	endocrine process	BP	1	AVPR1A	up_VFEM_EC
GO:0045655	0.0719	0.992	2	9	regulation of monocyte differentiation	BP	1	CLSTN1,ZFP36L1	down_VCAV_EC
GO:0072073	0.0719	0.998	1	60	kidney epithelium development	BP	1	ARG2	up_AORT_VSMC
GO:0003281	0.0719	0.998	1	30	ventricular septum development	BP	1	NKX2-5	up_VPOR_VSMC
GO:0060541	0.072	0.971	7	73	respiratory system development	BP	1	ADAMTS2,BMPR1A,LOX,GLI3,LOXL3,RDH10,THRB	down_VCAV_EC
GO:0048519	0.072	0.976	7	1554	negative regulation of biological process	BP	1	CHL1,HOXA3,BARX1,NKX2-5,BAMBI,ANKRD6,CYP26B1	up_VPOR_VSMC
GO:0048583	0.072	0.974	7	1095	regulation of response to stimulus	BP	1	NR1H4,RGS1,CCL24,PTGER2,FLRT3,ARHGEF2,NEO1	up_VPUL_EC
GO:0046323	0.072	0.981	4	26	glucose import	BP	0.874	NR1H4,ENPP1,PTPN11,SESN2	down_VSAP_EC
GO:0042417	0.072	0.992	2	9	dopamine metabolic process	BP	1	MAOA,PARK7	down_VCAV_EC
GO:0110110	0.072	0.998	1	34	positive regulation of animal organ	BP	1	SRSF6	down_AFEM_EC
GO:0007368	0.072	0.998	1	37	determination of left/right symmetry	BP	1	CITED2	down_VPOR_EC
GO:0045597	0.072	0.999	1	253	positive regulation of cell differentiation	BP	1	GATA5	down_VFEM_VSMC
GO:0000041	0.072	0.998	1	31	transition metal ion transport	BP	1	TRPC4	up_VPOR_VSMC
GO:0050865	0.0721	0.964	11	135	regulation of cell activation	BP	1	GPAM,PRNP,SDC4,LMO4,THY1,GLI3,ZFP36L1,BPI,LOXL3,RAC2,ZBTB16	down_VCAV_EC
GO:0045445	0.0721	0.981	4	27	myoblast differentiation	BP	0.874	PRICKLE1,DDIT3,REST,HIF1AN	down_VSAP_EC
GO:0032526	0.0721	0.981	4	19	response to retinoic acid	BP	0.91	TIE1,PPARG,YES1,ABCA1	up_VCAV_EC
GO:1902893	0.0721	0.981	4	19	regulation of pri-miRNA transcription by RNA polymerase II	BP	0.91	PDGFB,BMP2,SMARCA4,HIF1A	up_VCAV_EC
GO:0007188	0.0721	0.984	3	41	adenylate cyclase-modulating G protein-coupled receptor signaling	BP	1	CHRM2,HTR1D,PTHLH	up_AFEM_EC
GO:0035051	0.0721	0.985	3	41	cardiocyte differentiation	BP	1	DLL1,GREM1,BVES	up_AFEM_EC
GO:0001568	0.0721	0.985	3	252	blood vessel development	BP	1	HOXA3,NTRK2,HOXA1	up_APUL_EC
GO:0032102	0.0721	0.998	1	67	negative regulation of response to external	BP	1	ARG2	up_AORT_VSMC
GO:0090257	0.0721	0.998	1	52	regulation of muscle system process	BP	1	GATA5	up_VCAV_VSMC
GO:0060688	0.0721	0.998	1	29	regulation of morphogenesis of a	BP	1	SHOX2	up_VFEM_VSMC
GO:0044106	0.072131067	0.99075	2	17	cellular amine metabolic process	BP	1	NR1H4,AZIN2	up_ACOR_EC
GO:0072073	0.0722	0.968	9	60	kidney epithelium development	BP	0.91	HOXB7,PODXL,MEF2C,GATA3,MTSS1,ARG2,BMP2,JAG1,BASP1	up_VCAV_EC
GO:0043123	0.0722	0.97	8	46	positive regulation of I-kappaB kinase/NF-kappaB	BP	1	MAP3K14,ABL1,F2RL1,RBCK1,MYD88,S100A4,ENSCAFG00000011993,TRADD	up_VSAP_EC
GO:0035023	0.0722	0.974	6	46	regulation of Rho protein signal transduction	BP	0.874	ARHGEF2,FARP1,APOE,PLEKHG2,NRP1,TNFAIP1	down_VSAP_EC
GO:0042325	0.0722	0.981	4	463	regulation of phosphorylation	BP	1	CCL24,PTGER2,FLRT3,ARHGEF2	up_VPUL_EC
GO:0070848	0.0722	0.985	3	193	response to growth factor	BP	1	ACVRL1,SULF1,ASPN	down_ACOR_EC
GO:0043009	0.0722	0.991	2	227	chordate embryonic development	BP	1	CITED2,TBX15	down_VPOR_EC
GO:0018105	0.0722	0.99	2	116	peptidyl-serine phosphorylation	BP	1	PLCL1,NTRK2	up_APUL_EC
GO:0016458	0.0722	0.998	1	59	gene silencing	BP	1	ESR1	up_AORT_VSMC

GO:1903037	0.0722	0.998	1	65	regulation of leukocyte cell-cell adhesion	BP	1	ARG2	up_AORT_VSMC
GO:0048486	0.0722	0.998	1	10	parasympathetic nervous system development	BP	1	SEMA3A	up_VPOR_EC
GO:0006023	0.0722	0.998	1	17	aminoglycan biosynthetic process	BP	1	CHST7	up_VFEM_EC
GO:0008654	0.0723	0.969	8	58	phospholipid biosynthetic process	BP	0.91	ADGRF5,PDGFB,SPTLC2,SAMD8,SH3GLB1,PLCG2,IMPA1,PIP5K1B	up_VCAV_EC
GO:0090128	0.0723	0.993	2	5	regulation of synapse maturation	BP	0.91	ARHGEF15,RELN	up_VCAV_EC
GO:0060606	0.0723	0.998	1	39	tube closure	BP	1	CITED2	down_VPOR_EC
GO:0046034	0.0723	0.998	1	88	ATP metabolic process	BP	1	AK5	down_VSAP_VSMC
GO:0060759	0.0723	0.998	1	37	regulation of response to cytokine stimulus	BP	1	SLIT2	up_VPUL_VSMC
GO:0046847	0.072338342	0.99071	2	17	filopodium assembly	BP	1	NRP1,PRKCD	up_ACOR_EC
GO:0032989	0.0724	0.951	33	292	cellular component morphogenesis	BP	0.91	EPHB1,TEK,IL7R,KIT,IFRD1,RELN,MEF2C,STC1,GATA3,EDN1,RAB11A,ARAP3,NEDD9,TMOD1,PPP3CA,NECTIN2,PREX2,PDZD8,PROX1,ENSCAFG00000010224,STMN1,FLNB,SNX2,FES,RAPGEF2,CREB1,FMNL3,SPTBN4,ALS2,ENSCAFG00000002015,TMOD3,ARHGEF2,ZMYM2	up_VCAV_EC
GO:0002573	0.0724	0.965	11	71	myeloid leukocyte differentiation	BP	1	HOXA7,PPARGC1B,F2RL1,ZFP36L1,PIK3R1,BATF2,MYC,TFE3,TGFBR2,CITED2,PIR	up_VSAP_EC
GO:0061326	0.0724	0.984	3	41	renal tubule development	BP	1	IRX3,DLL1,GREM1	up_AFEM_EC
GO:0072080	0.0724	0.984	3	41	nephron tubule development	BP	1	IRX3,DLL1,GREM1	up_AFEM_EC
GO:1905906	0.0724	0.993	2	5	regulation of amyloid fibril formation	BP	1	APP,PSEN1	up_ACOR_VSMC
GO:0032922	0.0724	0.991	2	19	circadian regulation of gene expression	BP	1	CRY2,PPARGC1A	up_AFEM_EC
GO:0098957	0.0724	1	1	1	anterograde axonal transport of	BP	0.874	TRAK1	down_VSAP_EC
GO:0032663	0.0724	0.998	1	19	regulation of interleukin-2 production	BP	1	NR1H4	up_APUL_EC
GO:0002065	0.0724	0.998	1	37	columnar/cuboidal epithelial cell	BP	1	GATA5	up_VPUL_VSMC
GO:0023056	0.0725	0.977	5	462	positive regulation of signaling	BP	1	ACVRL1,LYN,SULF1,STX4,NEDD4	down_ACOR_EC
GO:0048666	0.0725	0.985	3	258	neuron development	BP	1	MAPK10,SLITRK5,NTRK2	up_APUL_EC
GO:0042060	0.0725	0.99	2	103	wound healing	BP	1	CAV1,F5	up_VSAP_VSMC
GO:0019827	0.0725	0.998	1	59	stem cell population maintenance	BP	1	WNT7A	up_AORT_VSMC
GO:0021545	0.0725	0.998	1	21	cranial nerve development	BP	1	HOXA3	up_VPUL_EC
GO:0120163	0.0725	0.998	1	17	negative regulation of cold-induced	BP	1	HOXC10	up_VSAP_VSMC
GO:0019318	0.0726	0.969	8	75	hexose metabolic process	BP	0.874	ENPP1,TPI1,PGAM1,SESN2,SLC45A3,PTPN2,ATF3,GALE	down_VSAP_EC
GO:0070509	0.0726	0.981	4	21	calcium ion import	BP	0.91	PDGFB,STC1,MCU,ATP2A1	up_VCAV_EC
GO:1901655	0.0726	0.985	3	20	cellular response to ketone	BP	1	ADCY6,NR3C1,PARK7	down_VCAV_EC
GO:0007628	0.0726	0.986	3	11	adult walking behavior	BP	1	FXN,CTNS,HTRA2	up_VSAP_EC
GO:0006753	0.0726	0.991	2	211	nucleoside phosphate metabolic process	BP	1	SUCLG2,NDUFA10	down_AFEM_EC
GO:0006423	0.0726	1	1	1	cysteinyl-tRNA aminoacylation	BP	0.874	CARS	down_VSAP_EC
GO:0043122	0.0727	0.966	10	62	regulation of I-kappaB kinase/NF-kappaB signaling	BP	1	MAP3K14,ABL1,F2RL1,RBCK1,MYD88,S100A4,NLRX1,ENSCAFG00000011993,MAPKBP1,TRADD	up_VSAP_EC
GO:0044271	0.0727	0.985	4	1501	cellular nitrogen compound biosynthetic	BP	1	HOXA9,WNT7A,ARG2,ESR1	up_AORT_VSMC
GO:0007340	0.0727	0.993	2	5	acrosome reaction	BP	1	GLRB,SPINK2	up_ACOR_VSMC
GO:0080134	0.0727	0.992	2	386	regulation of response to stress	BP	1	ARG2,ESR1	up_AORT_VSMC
GO:0060341	0.0727	0.999	1	255	regulation of cellular localization	BP	1	JUP	down_VFEM_VSMC
GO:0061817	0.0727	1	1	1	endoplasmic reticulum-plasma membrane	BP	0.874	ESYT3	down_VSAP_EC
GO:0001659	0.0727	0.998	1	54	temperature homeostasis	BP	1	HOXC10	up_AFEM_VSMC

GO:0002793	0.0727	0.998	1	54	positive regulation of peptide secretion	BP	1	TLR2	up_AFEM_VSMC
GO:0050714	0.0727	0.998	1	54	positive regulation of protein secretion	BP	1	TLR2	up_AFEM_VSMC
GO:0071466	0.0727	0.998	1	17	cellular response to xenobiotic stimulus	BP	1	CYP26B1	up_VFEM_EC
GO:0007405	0.0727	0.998	1	21	neuroblast proliferation	BP	1	ARHGEF2	up_VPUL_EC
GO:0035137	0.0727	0.998	1	18	hindlimb morphogenesis	BP	1	PITX1	up_VSAP_VSMC
GO:0098655	0.0728	0.957	19	142	cation transmembrane transport	BP	1	ACTN2,PLCG2,KCNE3,HTR2B,PTPN3,MICU2,ITPR1,DAPK1,CACNB3,AKAP6,KCNJ4,PRKCE,CHD7,ATP6V0E1,KCNC4,FXYD1,NDUFA4L2,PARK7,SLC8A1	up_ACOR_VSMC
GO:0051271	0.0728	0.965	10	98	negative regulation of cellular component	BP	0.874	SULF1,SERPINE1,ACTN1,DPYSL3,STARD13,APOE,MMP28,STRAP,ZMYND8,NRP1	down_VSAP_EC
GO:0010762	0.0728	0.991	2	9	regulation of fibroblast migration	BP	1	SDC4,DDR2	down_VCAV_EC
GO:0001755	0.0728	0.998	1	16	neural crest cell migration	BP	1	NRP2	down_ACOR_EC
GO:0010629	0.0728	1	1	502	negative regulation of gene expression	BP	1	GATA5	down_AFEM_VSMC
GO:0002063	0.0728	0.998	1	18	chondrocyte development	BP	1	SHOX2	up_VSAP_VSMC
GO:0071895	0.072858025	0.99818	1	3	odontoblast differentiation	BP	1	DLX3	up_ACOR_EC
GO:0050896	0.0729	0.947	65	2346	response to stimulus	BP	1	CMKLR1,MT2A,LEP,LBH,CRY2,ENSCAFG00000009113,RAB20,GAS1,F5,ANGPT4,CD40,DLL1,CRYAB,CPNE7,CTSS,F3,PLXNA4,BPI,GPAM,FIBIN,MLLT11,ENSCAFG00000018194,GPR1,LMCD1,CHRM2,SLC17A5,MFAP4,HCK,TMEM100,PLAGL2,GREM1,HTR1D,NFE2L1,FAM53B,AVPR1A,CAMK2A,GREM2,CLEC16A,CYLD,RASGEF1B,POSTN,LRRC15,FAM13A,RECK,NR3C1,PTHLH,TBXA2R,DCUN1D3,TFPI2,BVES,LGR6,ACKR4,RAB7A,AXIN2,ACACB,NAPEPLD,PPARGC1A,CCL5,CDC42EP5,DMPK,ADAM17,KANK1,TNC,ANXA11,SOD1	up_AFEM_EC
GO:0009895	0.0729	0.976	5	94	negative regulation of catabolic process	BP	1	LEP,TENT5C,CLEC16A,AXIN2,ACACB	up_AFEM_EC
GO:0048522	0.0729	0.985	4	1484	positive regulation of cellular process	BP	1	HOXA9,WNT7A,ARG2,ESR1	up_AORT_VSMC
GO:1901861	0.0729	0.984	3	41	regulation of muscle tissue development	BP	1	DLL1,GREM1,PPARGC1A	up_AFEM_EC
GO:0042493	0.0729	0.991	2	178	response to drug	BP	1	FBP1,EMX2	up_VFEM_VSMC
GO:0072329	0.0729	0.998	1	34	monocarboxylic acid catabolic process	BP	1	ETFA	down_AFEM_EC
GO:0032642	0.0729	0.998	1	22	regulation of chemokine production	BP	1	NR1H4	up_VPUL_EC
GO:0031325	0.073	0.943	88	914	positive regulation of cellular metabolic process	BP	0.91	PLK2,TEK,HCLS1,ADGRF5,KIT,SELE,PPP2R5A,TNFAIP3,PPARG,MAP3K11,TNIP1,PDGFB,STK10,TMEM173,RELN,MEF2C,ATF3,CHD7,TLR4,NLRC5,GATA3,EDN1,RALB,APLNR,RAB27A,TCF4,FOXN2,ENSCAFG00000017103,NEDD9,IRF8,PELI1,PPP3CA,YES1,SPTLC2,CASP8,MTOR,MAP3K3,BMP2,HHEX,CREM,SLK,CNOT6,PROX1,MADD,ARID4B,HMBOX1,PAK2,PRKD2,THOC1,SH3GLB1,SMARCA4,TLR3,NCK1,TIMELESS,OTUD4,LDB2,JAG1,NSD1,PTGS2,MMD,RAPGEF2,PLCG2,BCL10,TAOK1,CNOT7,HMGB1,AHR,NOS2,PMAIP1,BLM,RIPK1,ITGA6,GPBP1,PDGFD,MTA2,NR1D2,UBE2D1,KDM5A,HIF1A,KDM3A,ATRX,NR4A1,JCAD,ZEB1,TET2,ARHGEF2,CDON,ZNF609	up_VCAV_EC
GO:0032386	0.073	0.962	13	137	regulation of intracellular transport	BP	0.874	PLK3,PTPN14,PTPN11,PPP1R12A,MYO1C,MSN,IWS1,YIPF5,ZPR1,CRYAB,LYN,GSK3B,CDKN1A	down_VSAP_EC
GO:0044843	0.073	0.971	7	62	cell cycle G1/S phase transition	BP	0.874	PLK3,ZPR1,EIF4EBP1,BACH1,RBBP8,INHBA,CDKN1A	down_VSAP_EC
GO:0006887	0.073	0.99	2	99	exocytosis	BP	1	LYN,STX4	down_ACOR_EC
GO:1901576	0.073	1	2	1877	organic substance biosynthetic process	BP	1	JUP,GATA5	down_VFEM_VSMC
GO:0030166	0.073	0.99	2	19	proteoglycan biosynthetic process	BP	1	XYLT1,CSGALNACT2	up_AFEM_EC
GO:0006023	0.073	0.997	1	17	aminoglycan biosynthetic process	BP	1	CSGALNACT1	down_ACOR_EC
GO:1904375	0.073	0.998	1	35	regulation of protein localization to cell	BP	1	GPSM2	down_AFEM_EC

GO:0120162	0.073	0.997	1	31	positive regulation of cold-induced thermogenesis	BP	1	GATM	up_VFEM_VSMC
GO:0015718	0.073	0.998	1	30	monocarboxylic acid transport	BP	1	SLC16A12	up_VFEM_VSMC
GO:0007154	0.0731	0.969	10	1622	cell communication	BP	1	NR1H4,TBX5,MAPK10,HTR7,PLCL1,ARHGEF26,SLITRK5,NPNT,NTRK2,PDE1A	up_APUL_EC
GO:0006289	0.0731	0.974	6	35	nucleotide-excision repair	BP	1	RPA1,POLE,NEIL3,RBBP8,RAD23A,HMGN1	down_ACOR_VSMC
GO:1905477	0.0731	0.98	4	32	positive regulation of protein localization to	BP	1	LRP1,PRNP,CLIP3,AKT2	down_VCAV_EC
GO:0030856	0.0731	0.984	3	41	regulation of epithelial cell differentiation	BP	1	HOXA7,DLL1,TMEM100	up_AFEM_EC
GO:1903531	0.0731	0.984	3	41	negative regulation of secretion by cell	BP	1	LEP,CRY2,RAB7A	up_AFEM_EC
GO:0048261	0.0731	0.993	2	5	negative regulation of receptor-mediated	BP	1	ATXN2,LRPAP1	up_ACOR_VSMC
GO:0007155	0.0731	0.991	2	324	cell adhesion	BP	1	GATA5,TGFBI	up_VCAV_VSMC
GO:0061061	0.0731	0.991	2	179	muscle structure development	BP	1	NKX2-5,CYP26B1	up_VPOR_VSMC
GO:0042692	0.0731	0.99	2	105	muscle cell differentiation	BP	1	STIM1,SHOX2	up_VSAP_VSMC
GO:0038190	0.0731	1	1	1	VEGF-activated neuropilin signaling pathway	BP	0.874	NRP1	down_VSAP_EC
GO:0048842	0.0731	1	1	1	positive regulation of axon extension involved in axon guidance	BP	0.874	NRP1	down_VSAP_EC
GO:0061441	0.0731	1	1	1	renal artery morphogenesis	BP	0.874	NRP1	down_VSAP_EC
GO:1902336	0.0731	1	1	1	positive regulation of retinal ganglion cell axon	BP	0.874	NRP1	down_VSAP_EC
GO:1902378	0.0731	1	1	1	VEGF-activated neuropilin signaling pathway involved in axon guidance	BP	0.874	NRP1	down_VSAP_EC
GO:1902669	0.0731	1	1	1	positive regulation of axon guidance	BP	0.874	NRP1	down_VSAP_EC
GO:1905040	0.0731	1	1	1	otic placode development	BP	0.874	NRP1	down_VSAP_EC
GO:0060560	0.0731	0.998	1	58	developmental growth involved in	BP	1	ESR1	up_AORT_VSMC
GO:0032612	0.0731	0.997	1	22	interleukin-1 production	BP	1	NR1H4	up_VPUL_EC
GO:0032008	0.0732	0.99	2	19	positive regulation of TOR signaling	BP	1	LEP,CLEC16A	up_AFEM_EC
GO:0042759	0.0732	1	1	1	long-chain fatty acid biosynthetic process	BP	1	ELOVL6	down_ACOR_VSMC
GO:0098930	0.0733	0.985	3	17	axonal transport	BP	0.874	HIF1A,TRAK1,AP3S1	down_VSAP_EC
GO:0038083	0.0733	0.985	3	16	peptidyl-tyrosine autophosphorylation	BP	0.874	VEGFA,LYN,BTK	down_VSAP_EC
GO:0051173	0.0733	0.987	3	874	positive regulation of nitrogen compound	BP	1	HOXA9,WNT7A,ESR1	up_AORT_VSMC
GO:0045070	0.0733	0.992	2	8	positive regulation of viral genome replication	BP	0.874	CNOT7,RAD23A	down_VSAP_EC
GO:0046697	0.0733	0.993	2	5	decidualization	BP	1	CITED2,CTSB	up_ACOR_VSMC
GO:0060396	0.0733	0.993	2	5	growth hormone receptor signaling pathway	BP	1	STAT5A,PIK3R1	up_VSAP_EC
GO:0060416	0.0733	0.993	2	5	response to growth hormone	BP	1	STAT5A,PIK3R1	up_VSAP_EC
GO:0071378	0.0733	0.993	2	5	cellular response to growth hormone stimulus	BP	1	STAT5A,PIK3R1	up_VSAP_EC
GO:0043038	0.0733	0.997	1	34	amino acid activation	BP	1	DARS	down_AFEM_EC
GO:0002218	0.0733	0.998	1	53	activation of innate immune response	BP	1	TLR2	up_AFEM_VSMC
GO:0015718	0.0733	0.997	1	30	monocarboxylic acid transport	BP	1	TRPC4	up_VPOR_VSMC
GO:0072006	0.073330503	0.97941	4	59	nephron development	BP	1	SOX9,AQP1,FOXC2,CLSTN1	up_ACOR_EC

GO:0009893	0.0734	0.942	95	995	positive regulation of metabolic process	BP	0.91	PLK2,TEK,HCLS1,IL7R,ADGRF5,KIT,SELE,PPP2R5A,TNFAIP3,PPARG,MAP3K11,TNIP1,PDGFB,STK10,TMEM173,RELN,MEF2C,ATF3,CHD7,TIPARP,DLL4,TLR4,NLRC5,GATA3,EDN1,RALB,APLNR,RAB27A,TCF4,FOXN2,ENSCAFG00000017103,NEDD9,IRF8,PELI1,PPP3CA,YES1,SPTLC2,CASP8,MTOR,MAP3K3,BMP2,HHEX,CREM,SLK,CNOT6,PROX1,MADD,ARID4B,HMBOX1,PAK2,PRKD2,THOC1,SH3GLB1,SMARCA4,TLR3,NCK1,TIMELESS,OTUD4,LDB2,JAG1,NSD1,PTGS2,MMD,RAPGEF2,PLCG2,CREB1,BCL10,TAOK1,CNOT7,GCH1,ADIPOR2,HMGB1,AHR,NOS2,PMAIP1,BLM,RIPK1,ITGA6,GPBP1,PDGFD,MTA2,NR1D2,UBE2D1,KDM5A,HIF1A,KDM3A,ATRX,NR4A1,ENSCAFG00000002015,JCAD,ZEB1,TET2,ARHGEF2,CDON,ZNF609	up_VCAV_EC
GO:0006412	0.0734	0.957	18	245	translation	BP	1	UBA52,LARP6,COA1,SAMD4B,RPL23,RPL13,EIF3K,MRPL37,RPL27A,EEF2,ZFP36L1,RPL27,MRPS11,RPS27,ENSCAFG00000015122,PYM1,FOXO3,EIF4EBP1	down_VCAV_EC
GO:0051252	0.0734	0.969	9	1051	regulation of RNA metabolic process	BP	1	ACVRL1,YEATS2,YBX3,HIVEP2,HOXA7,SULF1,ELK3,HOXA1,NEDD4	down_ACOR_EC
GO:0071214	0.0734	0.99	2	93	cellular response to abiotic stimulus	BP	1	USP28,NEDD4	down_ACOR_EC
GO:0104004	0.0734	0.99	2	93	cellular response to environmental stimulus	BP	1	USP28,NEDD4	down_ACOR_EC
GO:0031644	0.0734	0.99	2	19	regulation of neurological system process	BP	1	TMEM100,DMPK	up_AFEM_EC
GO:0060560	0.0734	0.99	2	58	developmental growth involved in	BP	1	SEMA3A,RNF6	up_VPOR_EC
GO:0008584	0.0734	0.997	1	39	male gonad development	BP	1	CITED2	down_VPOR_EC
GO:0046546	0.0734	0.997	1	39	development of primary male sexual	BP	1	CITED2	down_VPOR_EC
GO:1900543	0.0734	0.998	1	9	negative regulation of purine nucleotide	BP	1	FBP1	up_VPOR_EC
GO:1903579	0.0734	0.998	1	9	negative regulation of ATP metabolic process	BP	1	FBP1	up_VPOR_EC
GO:0051155	0.0734	0.997	1	18	positive regulation of striated muscle cell	BP	1	CYP26B1	up_VFEM_EC
GO:0021987	0.0734	0.998	1	37	cerebral cortex development	BP	1	SLIT2	up_VPUL_VSMC
GO:2000514	0.0735	0.986	3	13	regulation of CD4-positive, alpha-beta T cell	BP	0.91	GATA3,ARG2,HMGB1	up_VCAV_EC
GO:0060449	0.0735	0.993	2	5	bud elongation involved in lung branching	BP	1	BMP4,FGFR2	up_ACOR_VSMC
GO:0038066	0.0735	0.99	2	19	p38MAPK cascade	BP	1	LEP,CYLD	up_AFEM_EC
GO:0071163	0.0735	1	1	1	DNA replication preinitiation complex	BP	1	GMNN	down_ACOR_VSMC
GO:0003183	0.073508341	0.99815	1	3	mitral valve morphogenesis	BP	1	SMAD6	up_ACOR_EC
GO:0070295	0.073564911	0.99814	1	3	renal water absorption	BP	1	AQP1	up_ACOR_EC
GO:0002064	0.0736	0.968	8	71	epithelial cell development	BP	0.874	FLNB,MSN,HIF1A,ACTA2,CTNNB1,PLOD3,KLF5,MYO1E	down_VSAP_EC
GO:0048699	0.0736	0.991	2	371	generation of neurons	BP	1	HOXC10,WNT7A	up_AORT_VSMC
GO:0046427	0.0736	0.997	1	16	positive regulation of JAK-STAT cascade	BP	1	LYN	down_ACOR_EC
GO:0030505	0.0736	1	1	1	inorganic diphosphate transport	BP	0.874	ENPP1	down_VSAP_EC
GO:0035426	0.0736	1	1	1	extracellular matrix-cell signaling	BP	0.91	NTN4	up_VCAV_EC
GO:0060668	0.0736	1	1	1	regulation of branching involved in salivary gland morphogenesis by extracellular matrix-	BP	0.91	NTN4	up_VCAV_EC
GO:1901879	0.0737	0.98	4	26	regulation of protein depolymerization	BP	0.874	ARHGEF2,WDR1,CAPG,MID1	down_VSAP_EC
GO:1905207	0.0737	0.981	4	17	regulation of cardiocyte differentiation	BP	1	BMP4,TBX5,FRS2,EDN1	up_ACOR_VSMC
GO:0001708	0.0737	0.985	3	20	cell fate specification	BP	1	BMPR1A,LMO4,GLI3	down_VCAV_EC
GO:0002028	0.0737	0.99	2	19	regulation of sodium ion transport	BP	1	FXYD1,DMPK	up_AFEM_EC
GO:0034375	0.0737	0.993	2	5	high-density lipoprotein particle remodeling	BP	1	LIPC,LCAT	up_VSAP_EC

GO:0007498	0.0737	0.997	1	38	mesoderm development	BP	1	CITED2	down_VPOR_EC
GO:0007403	0.0737	1	1	1	glial cell fate determination	BP	0.874	CTNNB1	down_VSAP_EC
GO:0010908	0.0737	1	1	1	regulation of heparan sulfate proteoglycan	BP	0.874	CTNNB1	down_VSAP_EC
GO:0010909	0.0737	1	1	1	positive regulation of heparan sulfate proteoglycan biosynthetic	BP	0.874	CTNNB1	down_VSAP_EC
GO:0021781	0.0737	1	1	1	glial cell fate commitment	BP	0.874	CTNNB1	down_VSAP_EC
GO:0044334	0.0737	1	1	1	canonical Wnt signaling pathway involved in positive regulation of epithelial to mesenchymal	BP	0.874	CTNNB1	down_VSAP_EC
GO:0055059	0.0737	1	1	1	asymmetric neuroblast division	BP	0.874	ARHGEF2	down_VSAP_EC
GO:0060769	0.0737	1	1	1	positive regulation of epithelial cell proliferation involved in prostate gland	BP	0.874	CTNNB1	down_VSAP_EC
GO:0060789	0.0737	1	1	1	hair follicle placode formation	BP	0.874	CTNNB1	down_VSAP_EC
GO:0061198	0.0737	1	1	1	fungiform papilla formation	BP	0.874	CTNNB1	down_VSAP_EC
GO:0061324	0.0737	1	1	1	canonical Wnt signaling pathway involved in positive regulation of cardiac outflow tract cell	BP	0.874	CTNNB1	down_VSAP_EC
GO:0072053	0.0737	1	1	1	renal inner medulla development	BP	0.874	CTNNB1	down_VSAP_EC
GO:0072054	0.0737	1	1	1	renal outer medulla development	BP	0.874	CTNNB1	down_VSAP_EC
GO:0072383	0.0737	1	1	1	plus-end-directed vesicle transport along	BP	0.874	KIF5B	down_VSAP_EC
GO:0072386	0.0737	1	1	1	plus-end-directed organelle transport along	BP	0.874	KIF5B	down_VSAP_EC
GO:1902730	0.0737	1	1	1	positive regulation of proteoglycan biosynthetic	BP	0.874	CTNNB1	down_VSAP_EC
GO:1904499	0.0737	1	1	1	regulation of chromatin-mediated maintenance of	BP	0.874	CTNNB1	down_VSAP_EC
GO:1904501	0.0737	1	1	1	positive regulation of chromatin-mediated maintenance of	BP	0.874	CTNNB1	down_VSAP_EC
GO:1904793	0.0737	1	1	1	regulation of euchromatin binding	BP	0.874	CTNNB1	down_VSAP_EC
GO:2000017	0.0737	1	1	1	positive regulation of determination of dorsal	BP	0.874	CTNNB1	down_VSAP_EC
GO:0098727	0.0737	0.998	1	60	maintenance of cell number	BP	1	WNT7A	up_AORT_VSMC
GO:1905521	0.0737	0.998	1	9	regulation of macrophage migration	BP	1	C3AR1	up_VPOR_EC
GO:0055024	0.0737	0.997	1	22	regulation of cardiac muscle tissue	BP	1	TBX5	up_VPUL_EC
GO:0032092	0.0737	0.997	1	38	positive regulation of protein binding	BP	1	HOXA3	up_VPUL_VSMC
GO:0080090	0.0738	0.982	5	1734	regulation of primary metabolic process	BP	1	TLR2,EYA1,HOXA9,HOXA10,IRX5	up_AFEM_VSMC
GO:0034765	0.0738	0.99	2	91	regulation of ion transmembrane transport	BP	1	LYN,NEDD4	down_ACOR_EC
GO:0014821	0.0738	0.993	2	5	phasic smooth muscle contraction	BP	1	HTR2B,EDN1	up_ACOR_VSMC
GO:0015909	0.0738	0.997	1	16	long-chain fatty acid transport	BP	1	ACSL3	down_ACOR_EC
GO:0051602	0.0738	1	1	1	response to electrical stimulus	BP	0.874	REST	down_VSAP_EC
GO:0061110	0.0738	1	1	1	dense core granule biogenesis	BP	0.874	REST	down_VSAP_EC
GO:0071257	0.0738	1	1	1	cellular response to electrical stimulus	BP	0.874	REST	down_VSAP_EC
GO:1903381	0.0738	1	1	1	regulation of endoplasmic reticulum stress-induced neuron intrinsic apoptotic signaling pathway	BP	0.874	HYOU1	down_VSAP_EC
GO:1903382	0.0738	1	1	1	negative regulation of endoplasmic reticulum stress-induced neuron intrinsic apoptotic	BP	0.874	HYOU1	down_VSAP_EC
GO:2000705	0.0738	1	1	1	regulation of dense core granule biogenesis	BP	0.874	REST	down_VSAP_EC

GO:2000706	0.0738	1	1	1	negative regulation of dense core granule	BP	0.874	REST	down_VSAP_EC
GO:2000740	0.0738	1	1	1	negative regulation of mesenchymal stem cell differentiation	BP	0.874	REST	down_VSAP_EC
GO:2000797	0.0738	1	1	1	regulation of amniotic stem cell differentiation	BP	0.874	REST	down_VSAP_EC
GO:2000798	0.0738	1	1	1	negative regulation of amniotic stem cell	BP	0.874	REST	down_VSAP_EC
GO:0036483	0.0738	1	1	1	neuron intrinsic apoptotic signaling pathway in response to endoplasmic	BP	0.874	HYOU1	down_VSAP_EC
GO:0097086	0.0738	1	1	1	amniotic stem cell differentiation	BP	0.874	REST	down_VSAP_EC
GO:0120163	0.073847257	0.99041	2	17	negative regulation of cold-induced	BP	1	PLCL1,FOXC2	up_ACOR_EC
GO:0051241	0.0739	0.954	23	336	negative regulation of multicellular organismal process	BP	1	DCN,PRNP,VASN,FBN1,BMPR1A,CHID1,PLCL1,ASPN,LRPAP1,THY1,TLX2,GLI3,ZFP36L1,BPI,LOXL3,SPRED1,TSC22D3,TWIST2,PRKCSH,GREM1,THRB,ZBTB16,RGMA	down_VCAV_EC
GO:0040012	0.0739	0.955	21	303	regulation of locomotion	BP	1	PDGFRB,DCN,SDC4,GLIPR2,BMPR1A,LMO4,RECK,RHOD,AKT2,DOCK10,SYDE1,LAMA3,TRIM8,SEMA3C,THY1,SASH1,SPRED1,TWIST2,DDR2,RAC2,FOXO3	down_VCAV_EC
GO:0031214	0.0739	0.976	5	45	biomineral tissue development	BP	1	BMPR1A,ASPN,DDR2,OMD,GREM1	down_VCAV_EC
GO:0014074	0.0739	0.977	5	26	response to purine-containing compound	BP	0.91	P2RX7,SELL,RAPGEF1,RAPGEF2,P2RX2	up_VCAV_EC
GO:0090279	0.0739	0.991	2	8	regulation of calcium ion import	BP	0.874	CTNNB1,HOMER1	down_VSAP_EC
GO:0055026	0.0739	0.993	2	5	negative regulation of cardiac muscle tissue	BP	1	TBX5,FRS2	up_ACOR_VSMC
GO:0022610	0.0739	0.991	2	326	biological adhesion	BP	1	GATA5,TGFBI	up_VCAV_VSMC
GO:0034440	0.0739	0.997	1	35	lipid oxidation	BP	1	ETFA	down_AFEM_EC
GO:0006664	0.0739	0.997	1	37	glycolipid metabolic process	BP	1	GBGT1	down_VPOR_EC
GO:1903509	0.0739	0.997	1	37	liposaccharide metabolic process	BP	1	GBGT1	down_VPOR_EC
GO:0031529	0.0739	0.997	1	20	ruffle organization	BP	1	ARHGEF26	up_APUL_EC
GO:0048246	0.0739	0.998	1	9	macrophage chemotaxis	BP	1	C3AR1	up_VPOR_EC
GO:1901631	0.0739	1	1	1	positive regulation of presynaptic membrane	BP	1	LRP4	up_VSAP_EC
GO:1903911	0.0739	1	1	1	positive regulation of receptor clustering	BP	1	LRP4	up_VSAP_EC
GO:1904395	0.0739	1	1	1	positive regulation of skeletal muscle acetylcholine-gated	BP	1	LRP4	up_VSAP_EC
GO:1904398	0.0739	1	1	1	positive regulation of neuromuscular junction development	BP	1	LRP4	up_VSAP_EC
GO:0009914	0.074	0.965	10	64	hormone transport	BP	1	LEP,TM7SF3,PRKCE,SCG5,CHD7,CCL5,RAB11B,EDN1,PFKL,PARK7	up_ACOR_VSMC
GO:0022408	0.074	0.971	7	44	negative regulation of cell-cell adhesion	BP	0.91	PODXL,PELI1,ARG2,BMP2,SWAP70,JAG1,HMGB1	up_VCAV_EC
GO:0010812	0.074	0.99	2	19	negative regulation of cell-substrate adhesion	BP	1	HOXA7,KANK1	up_AFEM_EC
GO:0007079	0.074	1	1	1	mitotic chromosome movement towards	BP	0.874	KPNB1	down_VSAP_EC
GO:0009256	0.074	1	1	1	10-formyltetrahydrofolate metabolic process	BP	0.874	ALDH1L2	down_VSAP_EC
GO:0009258	0.074	1	1	1	10-formyltetrahydrofolate catabolic process	BP	0.874	ALDH1L2	down_VSAP_EC
GO:0009397	0.074	1	1	1	folic acid-containing compound catabolic	BP	0.874	ALDH1L2	down_VSAP_EC
GO:0021764	0.074	1	1	1	amygdala development	BP	0.874	UBA6	down_VSAP_EC
GO:0042560	0.074	1	1	1	pteridine-containing compound catabolic	BP	0.874	ALDH1L2	down_VSAP_EC
GO:0051014	0.074	1	1	1	actin filament severing	BP	0.874	MYH9	down_VSAP_EC
GO:0051305	0.074	1	1	1	chromosome movement towards spindle pole	BP	0.874	KPNB1	down_VSAP_EC
GO:0097498	0.074	1	1	1	endothelial tube lumen extension	BP	0.874	STARD13	down_VSAP_EC
GO:1900756	0.074	1	1	1	protein processing in phagocytic vesicle	BP	0.874	MYH9	down_VSAP_EC

GO:1903918	0.074	1	1	1	regulation of actin filament severing	BP	0.874	MYH9	down_VSAP_EC
GO:1903919	0.074	1	1	1	negative regulation of actin filament severing	BP	0.874	MYH9	down_VSAP_EC
GO:1903921	0.074	1	1	1	regulation of protein processing in phagocytic	BP	0.874	MYH9	down_VSAP_EC
GO:1903923	0.074	1	1	1	positive regulation of protein processing in	BP	0.874	MYH9	down_VSAP_EC
GO:0001979	0.074	1	1	1	regulation of systemic arterial blood pressure by chemoreceptor signaling	BP	0.91	P2RX2	up_VCAV_EC
GO:0003027	0.074	1	1	1	regulation of systemic arterial blood pressure by carotid body	BP	0.91	P2RX2	up_VCAV_EC
GO:0003029	0.074	1	1	1	detection of hypoxic conditions in blood by carotid body	BP	0.91	P2RX2	up_VCAV_EC
GO:0002007	0.074	1	1	1	detection of hypoxic conditions in blood by	BP	0.91	P2RX2	up_VCAV_EC
GO:0048644	0.074	0.997	1	31	muscle organ morphogenesis	BP	1	NKX2-5	up_VPOR_VSMC
GO:0048483	0.074	0.997	1	19	autonomic nervous system development	BP	1	SIX1	up_VFEM_EC
GO:0003158	0.074	0.997	1	38	endothelium development	BP	1	HOXB5	up_VPUL_VSMC
GO:0016571	0.0741	0.971	7	39	histone methylation	BP	0.91	IWS1,GATA3,ARID4B,NSD1,KDM3A,ATRX,TET2	up_VCAV_EC
GO:0061098	0.0741	0.986	3	12	positive regulation of protein tyrosine kinase	BP	0.91	PDGFB,RELN,NEDD9	up_VCAV_EC
GO:0006464	0.0741	0.995	2	1162	cellular protein modification process	BP	1	PLCL1,USP12	down_VFEM_EC
GO:0036211	0.0741	0.995	2	1162	protein modification process	BP	1	PLCL1,USP12	down_VFEM_EC
GO:0072507	0.0741	0.99	2	103	divalent inorganic cation homeostasis	BP	1	STIM1,CAV1	up_VSAP_VSMC
GO:0060393	0.0741	0.997	1	19	regulation of pathway-restricted SMAD protein	BP	1	ACVRL1	down_ACOR_EC
GO:0060349	0.0741	0.997	1	38	bone morphogenesis	BP	1	CITED2	down_VPOR_EC
GO:0001868	0.0741	1	1	1	regulation of complement activation, lectin pathway	BP	0.874	A2M	down_VSAP_EC
GO:0001869	0.0741	1	1	1	negative regulation of complement activation, lectin pathway	BP	0.874	A2M	down_VSAP_EC
GO:0002921	0.0741	1	1	1	negative regulation of humoral immune	BP	0.874	A2M	down_VSAP_EC
GO:0007290	0.0741	1	1	1	spermatid nucleus elongation	BP	0.874	KDM3A	down_VSAP_EC
GO:0010335	0.0741	1	1	1	response to non-ionic osmotic stress	BP	0.874	PTGS2	down_VSAP_EC
GO:0031394	0.0741	1	1	1	positive regulation of prostaglandin biosynthetic process	BP	0.874	PTGS2	down_VSAP_EC
GO:0031585	0.0741	1	1	1	regulation of inositol 1,4,5-trisphosphate-sensitive calcium-release channel	BP	0.874	HTT	down_VSAP_EC
GO:0031587	0.0741	1	1	1	positive regulation of inositol 1,4,5-trisphosphate-sensitive	BP	0.874	HTT	down_VSAP_EC
GO:0042297	0.0741	1	1	1	vocal learning	BP	0.874	HTT	down_VSAP_EC
GO:0045916	0.0741	1	1	1	negative regulation of complement activation	BP	0.874	A2M	down_VSAP_EC
GO:0046293	0.0741	1	1	1	formaldehyde biosynthetic process	BP	0.874	KDM3A	down_VSAP_EC
GO:0071471	0.0741	1	1	1	cellular response to non-ionic osmotic stress	BP	0.874	PTGS2	down_VSAP_EC
GO:0097402	0.0741	1	1	1	neuroblast migration	BP	0.874	PDS5B	down_VSAP_EC
GO:0098596	0.0741	1	1	1	imitative learning	BP	0.874	HTT	down_VSAP_EC
GO:0098597	0.0741	1	1	1	observational learning	BP	0.874	HTT	down_VSAP_EC
GO:0098598	0.0741	1	1	1	learned vocalization behavior or vocal learning	BP	0.874	HTT	down_VSAP_EC
GO:0140018	0.0741	1	1	1	regulation of cytoplasmic translational fidelity	BP	0.874	AARS	down_VSAP_EC
GO:1904170	0.0741	1	1	1	regulation of bleb assembly	BP	0.874	ANLN	down_VSAP_EC

GO:1904172	0.0741	1	1	1	positive regulation of bleb assembly	BP	0.874	ANLN	down_VSAP_EC
GO:1905335	0.0741	1	1	1	regulation of aggrephagy	BP	0.874	HTT	down_VSAP_EC
GO:1905337	0.0741	1	1	1	positive regulation of aggrephagy	BP	0.874	HTT	down_VSAP_EC
GO:1990034	0.0741	1	1	1	calcium ion export across plasma membrane	BP	0.874	ATP2B1	down_VSAP_EC
GO:2000258	0.0741	1	1	1	negative regulation of protein activation cascade	BP	0.874	A2M	down_VSAP_EC
GO:1903756	0.0741	1	1	1	regulation of transcription from RNA polymerase II promoter by histone	BP	0.874	ZMYND8	down_VSAP_EC
GO:1903758	0.0741	1	1	1	negative regulation of transcription from RNA polymerase II promoter by histone modification	BP	0.874	ZMYND8	down_VSAP_EC
GO:0002174	0.0741	1	1	1	mammary stem cell proliferation	BP	1	LBH	up_ACOR_VSMC
GO:1904674	0.0741	1	1	1	positive regulation of somatic stem cell	BP	1	LBH	up_ACOR_VSMC
GO:1904675	0.0741	1	1	1	regulation of somatic stem cell division	BP	1	LBH	up_ACOR_VSMC
GO:1904677	0.0741	1	1	1	positive regulation of somatic stem cell division	BP	1	LBH	up_ACOR_VSMC
GO:2000035	0.0741	1	1	1	regulation of stem cell division	BP	1	LBH	up_ACOR_VSMC
GO:2000101	0.0741	1	1	1	regulation of mammary stem cell proliferation	BP	1	LBH	up_ACOR_VSMC
GO:2000103	0.0741	1	1	1	positive regulation of mammary stem cell	BP	1	LBH	up_ACOR_VSMC
GO:0030431	0.07410108	0.99812	1	3	sleep	BP	1	PTGDS	up_ACOR_EC
GO:0048468	0.0742	0.974	6	553	cell development	BP	1	LYN,SPG11,PDPN,NRP2,SULF1,NEDD4	down_ACOR_EC
GO:0032609	0.0742	0.977	5	25	interferon-gamma production	BP	1	TLR3,ISG15,CD274,F2RL1,IL18R1	up_ACOR_VSMC
GO:0046683	0.0742	0.977	5	26	response to organophosphorus	BP	0.91	P2RX7,SELL,RAPGEF1,RAPGEF2,P2RX2	up_VCAV_EC
GO:0034660	0.0742	0.99	2	211	ncRNA metabolic process	BP	1	DARS,TSEN15	down_AFEM_EC
GO:0032462	0.0742	0.991	2	8	regulation of protein homooligomerization	BP	0.874	CRYAB,APOE	down_VSAP_EC
GO:0008283	0.0742	0.992	2	545	cell proliferation	BP	1	TGFBI,TGFBR1	down_VSAP_VSMC
GO:0000041	0.0742	0.997	1	31	transition metal ion transport	BP	1	ENSCAFG00000005494	up_AORT_EC
GO:2001237	0.0742	0.997	1	33	negative regulation of extrinsic apoptotic	BP	1	EYA1	up_VFEM_VSMC
GO:0021644	0.0742	1	1	1	vagus nerve morphogenesis	BP	1	PLXNA4	up_VSAP_EC
GO:0021793	0.0742	1	1	1	chemorepulsion of branchiomotor axon	BP	1	PLXNA4	up_VSAP_EC
GO:0009435	0.074241256	0.99811	1	3	NAD biosynthetic process	BP	1	NMNAT2	up_ACOR_EC
GO:0002237	0.07428579	0.97525	5	85	response to molecule of bacterial origin	BP	1	NR1H4,FZD5,PTGIR,NOD2,PTGER4	up_ACOR_EC
GO:0001501	0.0743	0.955	21	178	skeletal system development	BP	1	USP1,EIF4A3,HOXA3,NAB2,INSIG1,XYLT1,MMP14,GREM1,HOXA7,HOXC10,HOXB7,POC1A,HOXC6,HOXB5,HOXC4,SGPL1,TBX15,HOXA9,SERPINH1,SIX4,AMER1	down_ACOR_VSMC
GO:0001823	0.0743	0.976	5	45	mesonephros development	BP	1	SMAD9,CLSTN1,GLI3,GREM1,ZBTB16	down_VCAV_EC
GO:0046634	0.0743	0.977	5	24	regulation of alpha-beta T cell activation	BP	1	CD274,ZBTB16,CD83,PNP,TGFBR2	up_VSAP_EC
GO:0051893	0.0743	0.985	3	20	regulation of focal adhesion assembly	BP	1	RHOD,THY1,GREM1	down_VCAV_EC
GO:0090109	0.0743	0.985	3	20	regulation of cell-substrate junction	BP	1	RHOD,THY1,GREM1	down_VCAV_EC
GO:0090263	0.0743	0.997	1	33	positive regulation of canonical Wnt signaling	BP	1	DKK2	up_AORT_EC
GO:0001503	0.0743	0.998	1	109	ossification	BP	1	HOXA3	up_APUL_VSMC
GO:0002688	0.0743	0.997	1	34	regulation of leukocyte chemotaxis	BP	1	C3AR1	up_VPOR_VSMC
GO:0014855	0.0743	0.997	1	22	striated muscle cell proliferation	BP	1	TBX5	up_VPUL_EC
GO:1904427	0.0743	0.997	1	18	positive regulation of calcium ion	BP	1	STIM1	up_VSAP_VSMC

GO:0031098	0.074337919	0.97219	6	109	stress-activated protein kinase signaling cascade	BP	1	FZD5,NOD2,PAK3,TNFRSF19,MAPK8IP2,SH3RF3	up_ACOR_EC
GO:0007169	0.0744	0.955	21	178	transmembrane receptor protein tyrosine kinase signaling pathway	BP	0.91	TEK,KIT,PDGFB,BAIAP2L1,KDR,TIPARP,RAPGEF1,GATA3,BMP2,HHEX,ANGPT2,STMN1,PRKD2,APOD,NCK1,FES,RAPGEF2,PDGFD,BMX,HIF1A,JCAD	up_VCAV_EC
GO:0032755	0.0744	0.977	5	24	positive regulation of interleukin-6 production	BP	1	TLR1,MBP,TLR3,F2RL1,NOD2	up_ACOR_VSMC
GO:0032682	0.0744	0.992	2	7	negative regulation of chemokine production	BP	0.91	ARG2,APOD	up_VCAV_EC
GO:0051249	0.0744	0.99	2	107	regulation of lymphocyte activation	BP	1	TOX,CYP26B1	up_VFEM_EC
GO:0010763	0.0744	0.993	2	5	positive regulation of fibroblast migration	BP	1	DDR2,BAG4	up_VSAP_EC
GO:0031328	0.0744	1	1	512	positive regulation of cellular biosynthetic	BP	1	GATA5	down_AFEM_VSMC
GO:0006575	0.0744	0.997	1	53	cellular modified amino acid metabolic process	BP	1	ENSCAFG00000028814	up_VCAV_VSMC
GO:0021884	0.0744	0.997	1	10	forebrain neuron development	BP	1	SEMA3A	up_VPOR_EC
GO:1902904	0.0744	0.997	1	38	negative regulation of supramolecular fiber	BP	1	SLIT2	up_VPUL_VSMC
GO:0001505	0.0745	0.998	1	69	regulation of neurotransmitter levels	BP	1	WNT7A	up_AORT_VSMC
GO:0048103	0.0745	0.997	1	10	somatic stem cell division	BP	1	ARHGEF2	up_VPOR_EC
GO:0048565	0.0745	0.997	1	38	digestive tract development	BP	1	GATA5	up_VPUL_VSMC
GO:0071605	0.07458498	0.99809	1	3	monocyte chemotactic protein-1 production	BP	1	NR1H4	up_ACOR_EC
GO:0071637	0.07458498	0.99809	1	3	regulation of monocyte chemotactic protein-1	BP	1	NR1H4	up_ACOR_EC
GO:0071638	0.07458498	0.99809	1	3	negative regulation of monocyte chemotactic	BP	1	NR1H4	up_ACOR_EC
GO:1903506	0.0746	0.941	94	977	regulation of nucleic acid-templated transcription	BP	0.91	EDNRB,HCLS1,KIT,PRKCH,FLI1,TNFAIP3,PPARG,TNIP1,PDGFB,TMEM173,RELN,IWS1,MEF2C,ATF3,CHD7,DLL4,WWP1,TLR4,NLRC5,GATA3,EDN1,HOXB8,TCF4,SMAD1,FOXN2,MEOX1,ENSCAFG00000017103,IRF8,SOX18,PELI1,PPP3CA,YES1,MTOR,ELF4,BMP2,HMG20A,HHEX,CREM,CNOT6,RSC1A1,CCAR1,PROX1,UHRF2,MDFI,ENSCAFG00000010596,ARID4B,HMBOX1,PRKD2,THOC1,SMARCA4,TLR3,NCK1,TIMELESS,LDB2,CD200,JAG1,NSD1,TCERG1,CTDSPL2,CREB1,BCL10,BASP1,CNOT7,ENSCAFG00000002897,HMGB1,AHR,ZNF789,BLM,NUP133,RIPK1,ZNF317,USP13,ITGA6,SKIL,GPBP1,MTA2,NR1D2,KDM5A,HIF1A,KDM3A,ZNF146,ATRX,NR4A1,HIST2H2AC,HP1BP3,ARID5A,ENSCAFG00000002015,ZNF354A,ZEB1,TET2,ARHGEF2,CDON,ELK3,ZNF609	up_VCAV_EC
GO:0009792	0.0746	0.99	2	231	embryo development ending in birth or egg	BP	1	CITED2,TBX15	down_VPOR_EC
GO:0003006	0.0746	0.99	2	180	developmental process involved in reproduction	BP	1	HOXA9,HOXA11	up_VFEM_VSMC
GO:0042102	0.0747	0.99	2	19	positive regulation of T cell proliferation	BP	1	LEP,GPAM	up_AFEM_EC
GO:0030855	0.0747	0.99	2	183	epithelial cell differentiation	BP	1	BARX1,CYP26B1	up_VPOR_VSMC
GO:0051261	0.0747	0.997	1	33	protein depolymerization	BP	1	TAOK1	up_AORT_EC
GO:0003308	0.0747	1	1	1	negative regulation of Wnt signaling pathway involved in heart	BP	0.91	BMP2	up_VCAV_EC
GO:0021984	0.0747	1	1	1	adenohypophysis development	BP	0.91	BMP2	up_VCAV_EC
GO:0035054	0.0747	1	1	1	embryonic heart tube anterior/posterior pattern specification	BP	0.91	BMP2	up_VCAV_EC
GO:0043569	0.0747	1	1	1	negative regulation of insulin-like growth factor receptor signaling	BP	0.91	BMP2	up_VCAV_EC
GO:0051040	0.0747	1	1	1	regulation of calcium-independent cell-cell	BP	0.91	BMP2	up_VCAV_EC
GO:0051042	0.0747	1	1	1	negative regulation of calcium-independent cell-	BP	0.91	BMP2	up_VCAV_EC
GO:0060129	0.0747	1	1	1	thyroid-stimulating hormone-secreting cell	BP	0.91	BMP2	up_VCAV_EC

GO:0060804	0.0747	1	1	1	positive regulation of Wnt signaling pathway by BMP signaling pathway	BP	0.91	BMP2	up_VCAV_EC
GO:0060956	0.0747	1	1	1	endocardial cell differentiation	BP	0.91	SOX18	up_VCAV_EC
GO:0003348	0.0747	1	1	1	cardiac endothelial cell differentiation	BP	0.91	SOX18	up_VCAV_EC
GO:0006021	0.0747	1	1	1	inositol biosynthetic process	BP	0.91	IMPA1	up_VCAV_EC
GO:0033566	0.0747	1	1	1	gamma-tubulin complex localization	BP	0.91	MZT1	up_VCAV_EC
GO:0060128	0.0747	1	1	1	corticotropin hormone secreting cell	BP	0.91	BMP2	up_VCAV_EC
GO:0090022	0.0747	0.997	1	9	regulation of neutrophil chemotaxis	BP	1	C3AR1	up_VPOR_EC
GO:0060419	0.0747	0.997	1	22	heart growth	BP	1	TBX5	up_VPUL_EC
GO:0090611	0.074757806	0.99808	1	3	ubiquitin-independent protein catabolic process via the multivesicular body sorting pathway	BP	1	CHMP4B	up_ACOR_EC
GO:0039689	0.074765307	0.99808	1	3	negative stranded viral RNA replication	BP	1	ATG16L2	up_ACOR_EC
GO:0048050	0.074796834	0.99808	1	3	post-embryonic eye morphogenesis	BP	1	EFEMP1	up_ACOR_EC
GO:0002312	0.0748	0.977	5	24	B cell activation involved in immune response	BP	0.91	TLR4,THOC1,SWAP70,MFNG,PLCG2	up_VCAV_EC
GO:0007162	0.0748	0.998	1	66	negative regulation of cell adhesion	BP	1	ARG2	up_AORT_VSMC
GO:0061450	0.074855495	0.99808	1	3	trophoblast cell migration	BP	1	ARHGDIB	up_ACOR_EC
GO:1901163	0.074855495	0.99808	1	3	regulation of trophoblast cell migration	BP	1	ARHGDIB	up_ACOR_EC
GO:0045934	0.0749	0.947	43	407	negative regulation of nucleobase-containing compound metabolic process	BP	1	ELK3,HMGB1,RNPS1,SREBF1,ILF3,CBX5,ATAD2,HMGA1,SREBF2,SMC1A,NEDD4,U2AF2,MTDH,PARPBP,NAB2,BIRC5,CAV1,TIMELESS,THRAP3,FAM129B,AURKB,EZH2,GREM1,PLK3,HOXA7,UHRF1,GMNN,NOC2L,PPARD,SUV39H1,HMGB2,DMAP1,SMC3,CDK2,ENSCAFG00000005299,EZR,ERF,TCERG1,HAT1,HIST2H2AC,TINF2,H2AFX,SIX4	down_ACOR_VSMC
GO:0060840	0.0749	0.971	7	42	artery development	BP	0.91	ADGRF5,ADAMTS9,CHD7,DLL4,PROX1,JAG1,LUZP1	up_VCAV_EC
GO:1903391	0.0749	0.98	4	21	regulation of adherens junction organization	BP	0.91	TEK,SLK,APOD,ENSCAFG00000002015	up_VCAV_EC
GO:0051881	0.0749	0.984	3	19	regulation of mitochondrial membrane	BP	1	DCN,AKT2,PARK7	down_VCAV_EC
GO:0001944	0.0749	0.999	1	263	vasculature development	BP	1	JUP	down_VFEM_VSMC
GO:0071674	0.0749	0.997	1	23	mononuclear cell migration	BP	1	CCL24	up_VPUL_EC
GO:0051155	0.0749	0.997	1	18	positive regulation of striated muscle cell	BP	1	SHOX2	up_VSAP_VSMC
GO:1900122	0.074950439	0.99807	1	3	positive regulation of receptor binding	BP	1	PLCL1	up_ACOR_EC
GO:0045595	0.075	0.98	4	469	regulation of cell differentiation	BP	1	TBX5,EVI2B,WIF1,ARHGEF2	up_VPUL_EC
GO:0045604	0.075	0.981	4	17	regulation of epidermal cell differentiation	BP	1	HOXA7,DLL1,ZFP36L1,KEAP1	up_VSAP_EC
GO:0009888	0.075	0.992	2	545	tissue development	BP	1	GATA5,TGFBR1	down_VSAP_VSMC
GO:0008627	0.075	0.993	2	5	intrinsic apoptotic signaling pathway in response to osmotic	BP	0.91	PTGS2,ARHGEF2	up_VCAV_EC
GO:0030182	0.075	0.991	2	327	neuron differentiation	BP	1	SLITRK5,LIMK1	up_VCAV_VSMC
GO:0043009	0.075	0.99	2	227	chordate embryonic development	BP	1	HOXA3,HOXB5	up_VPUL_VSMC
GO:0002689	0.075	0.993	2	5	negative regulation of leukocyte chemotaxis	BP	1	C5AR2,SLIT2	up_VSAP_EC
GO:0021768	0.075	1	1	1	nucleus accumbens development	BP	0.91	ALDH1A3	up_VCAV_EC
GO:0060166	0.075	1	1	1	olfactory pit development	BP	0.91	ALDH1A3	up_VCAV_EC
GO:0032924	0.075	0.997	1	9	activin receptor signaling pathway	BP	1	ACVR2A	up_VPOR_EC
GO:0030224	0.075	0.997	1	18	monocyte differentiation	BP	1	HOXA7	up_VFEM_EC
GO:1903131	0.075	0.997	1	18	mononuclear cell differentiation	BP	1	HOXA7	up_VFEM_EC

GO:0032760	0.075	0.997	1	22	positive regulation of tumor necrosis factor	BP	1	ARHGEF2	up_VPUL_EC
GO:1903557	0.075	0.997	1	22	positive regulation of tumor necrosis factor superfamily cytokine	BP	1	ARHGEF2	up_VPUL_EC
GO:0051338	0.0751	0.95	31	288	regulation of transferase activity	BP	0.91	TEK,KIT,PPP2R5A,TNFAIP3,MAP3K11,PDGFB,STK10,RELN,TLR4,NLRC5,EDN1,RALB,NEDD9,MTOR,MAP3K3,BMP2,HHEX,PREX2,SLK,SH3BP5,PROX1,MADD,HMBOX1,PAK2,SH3GLB1,LDB2,MMD,RAPGEF2,BLM,RIPK1,PDGFD	up_VCAV_EC
GO:0046620	0.0751	0.973	6	30	regulation of organ growth	BP	1	FGFR2,LATS2,TBX20,TBX5,WWC2,EDN1	up_ACOR_VSMC
GO:0033108	0.0751	0.997	1	39	mitochondrial respiratory chain complex assembly	BP	1	NDUFA10	down_AFEM_EC
GO:0050921	0.0751	0.997	1	39	positive regulation of chemotaxis	BP	1	SLIT2	up_VPUL_VSMC
GO:0051055	0.0752	0.98	4	21	negative regulation of lipid biosynthetic process	BP	0.91	PDGFB,BMP2,PROX1,ENSCAFG00000010596	up_VCAV_EC
GO:1902808	0.0752	0.985	3	12	positive regulation of cell cycle G1/S phase	BP	1	EZH2,GLI1,MTBP	down_ACOR_VSMC
GO:0009404	0.0752	0.992	2	6	toxin metabolic process	BP	1	GSTO1,CTH	down_ACOR_VSMC
GO:0071801	0.0752	0.991	2	7	regulation of podosome assembly	BP	0.884	FSCN1,MSN	down_VSAP_EC
GO:0050774	0.0752	0.992	2	6	negative regulation of dendrite morphogenesis	BP	0.91	PPP3CA,RAPGEF2	up_VCAV_EC
GO:0003012	0.0752	0.998	1	90	muscle system process	BP	1	GATA5	down_VSAP_VSMC
GO:0072593	0.0752	0.997	1	68	reactive oxygen species metabolic process	BP	1	ARG2	up_AORT_VSMC
GO:0006929	0.0752	0.997	1	10	substrate-dependent cell migration	BP	1	SEMA3A	up_VPOR_EC
GO:0006812	0.0753	0.984	3	222	cation transport	BP	1	NETO1,STIM1,CAV1	up_VSAP_VSMC
GO:1990778	0.0753	0.99	2	91	protein localization to cell periphery	BP	1	ACSL3,STX4	down_ACOR_EC
GO:0017015	0.0753	0.997	1	40	regulation of transforming growth factor beta receptor signaling	BP	1	CITED2	down_VPOR_EC
GO:1903844	0.0753	0.997	1	40	regulation of cellular response to transforming growth factor beta	BP	1	CITED2	down_VPOR_EC
GO:0007507	0.07538168	0.96518	9	194	heart development	BP	1	GATA4,SOX9,PDLIM3,SMAD6,ACTN2,FOXC2,TBX5,C23H3orf58,NRP1	up_ACOR_EC
GO:0050793	0.0754	0.946	45	731	regulation of developmental process	BP	1	GPAM,NOTCH2,HSPB6,DCN,NAB2,VASN,FBN1,GLIPR2,BMPR1A,LMO4,TRPV2,NUMBL,TFE3,SYDE1,LAMA3,CLSTN1,SEMA3C,ASPN,THY1,PEX5,TLX2,B4GALT1,GLI3,ZFP36L1,MYDGF,SASH1,RBM38,CDH2,LOXL3,SPRED1,RAB7B,TSC22D3,UST,TWIST2,PRKCSH,DDR2,OMD,GREM1,NAPEPLD,FOXO3,THRB,ZBTB16,ANKRD54,RGMA,BRSK1	down_VCAV_EC
GO:0014020	0.0754	0.997	1	41	primary neural tube formation	BP	1	CITED2	down_VPOR_EC
GO:0032649	0.0754	0.997	1	23	regulation of interferon-gamma production	BP	1	NR1H4	up_VPUL_EC
GO:0042742	0.0754	0.997	1	39	defense response to bacterium	BP	1	IL22RA1	up_VPUL_VSMC
GO:1903426	0.0754	0.997	1	17	regulation of reactive oxygen species	BP	1	CAV1	up_VSAP_VSMC
GO:0051604	0.0755	0.965	9	102	protein maturation	BP	1	ADAMTS2,RHBDL1,PCSK7,GLI3,SEC11C,A2M,PCSK6,ATOX1,LONP2	down_VCAV_EC
GO:0030101	0.0755	0.985	3	12	natural killer cell activation	BP	1	TOX,CORO1A,ELF4	down_ACOR_VSMC
GO:1903523	0.0755	0.986	3	10	negative regulation of blood circulation	BP	0.912	DOCK4,PDE4D,SPTBN4	up_VCAV_EC
GO:0009058	0.0755	1	2	1910	biosynthetic process	BP	1	JUP,GATA5	down_VFEM_VSMC
GO:0019369	0.0755	0.992	2	5	arachidonic acid metabolic process	BP	1	DAGLB,ENSCAFG00000023820	up_VSAP_EC
GO:0072070	0.0755	0.992	2	5	loop of Henle development	BP	1	DLL1,IRX3	up_VSAP_EC
GO:0050803	0.0755	0.997	1	55	regulation of synapse structure or activity	BP	1	SLITRK5	up_VCAV_VSMC
GO:0050807	0.0755	0.997	1	55	regulation of synapse organization	BP	1	SLITRK5	up_VCAV_VSMC
GO:0010594	0.0756	0.963	11	71	regulation of endothelial cell migration	BP	1	SLIT2,ABL1,HDAC7,EMP2,CCBE1,STAT5A,TBXA2R,SASH1,ZNF580,ACVRL1,HMOX1	up_VSAP_EC

GO:0001558	0.0756	0.964	10	101	regulation of cell growth	BP	0.887	ENPP1,IFRD1,SMARCA4,SESN2,ESM1,ADAM17,IGFBP7,INHBA,CDKN1A,NRP1	down_VSAP_EC
GO:0046847	0.0756	0.984	3	17	filopodium assembly	BP	0.887	FSCN1,DPYSL3,NRP1	down_VSAP_EC
GO:0046887	0.0756	0.997	1	18	positive regulation of hormone secretion	BP	1	STX4	down_ACOR_EC
GO:0009891	0.0756	1	1	521	positive regulation of biosynthetic process	BP	1	GATA5	down_AFEM_VSMC
GO:0046324	0.0756	0.997	1	20	regulation of glucose import	BP	1	NR1H4	up_APUL_EC
GO:0000470	0.0756	0.997	1	9	maturation of LSU-rRNA	BP	1	RRP15	up_VPOR_EC
GO:0030166	0.0756	0.997	1	19	proteoglycan biosynthetic process	BP	1	CHST7	up_VFEM_EC
GO:0042158	0.0757	0.97	7	40	lipoprotein biosynthetic process	BP	1	LCAT,ABCA1,PIGN,PIGS,PIGG,PIK3C3,ZDHHC21	up_ACOR_VSMC
GO:0006955	0.0757	0.979	4	327	immune response	BP	1	LYN,IGF1R,IPO7,STX4	down_ACOR_EC
GO:0021675	0.0757	0.997	1	29	nerve development	BP	1	HOXA3	up_VPOR_VSMC
GO:0032635	0.075772516	0.98345	3	37	interleukin-6 production	BP	1	NR1H4,NOD2,MAVS	up_ACOR_EC
GO:0030042	0.075777571	0.99001	2	17	actin filament depolymerization	BP	1	TMOD1,ACTN2	up_ACOR_EC
GO:0030834	0.075777571	0.99001	2	17	regulation of actin filament depolymerization	BP	1	TMOD1,ACTN2	up_ACOR_EC
GO:0007275	0.0758	0.941	79	1370	multicellular organism development	BP	1	ADAMTS2,NOTCH2,HSPB6,PDGFRB,SMAD9,LRP1,DCN,NAB2,NDN,ANGPT1,SDC4,VASN,FBN1,GLIPR2,KANK2,THBS3,BMPR1A,LOX,LMO4,RECK,TRPV2,ANPEP,COL6A1,NUMBL,NAGLU,CHRDL1,TBX15,AKT2,TFE3,ENSCAFG00000018277,SYDE1,HOXC4,LAMA3,CLSTN1,CSPG4,PKD1,ENSCAFG00000013404,SEMA3C,ASPN,THY1,TEAD1,HAPLN3,PEX5,TLX2,VCAN,B4GALT1,GLI3,EEF2,ZFP36L1,MYDGF,SASH1,NCDN,IFT57,CDH2,LOXL3,GSDME,CAMK2D,ENSCAFG00000017708,SPRED1,RAB7B,IRX5,TSC22D3,UST,GLIS2,PRKCSH,DDR2,SHC1,RDH10,PCSK6,OMD,GREM1,CLIC4,FOXO3,THRB,ZBTB16,ANKRD54,RGMA,SEC61A1,BRSK1	down_VCAV_EC
GO:0051493	0.0758	0.953	22	188	regulation of cytoskeleton organization	BP	1	FSCN1,SMC1A,CAPG,LIMA1,NF2,STMN1,NEK2,POC1A,RAC1,NUP62,PDXP,PLK4,IQGAP2,CDC42EP4,SMC3,STAG1,RBM14,CORO1A,ARPC1B,ARHGEF2,KIF11,GEN1	down_ACOR_VSMC
GO:0090195	0.0758	0.992	2	5	chemokine secretion	BP	1	MBP,F2RL1	up_ACOR_VSMC
GO:0001702	0.0758	0.997	1	10	gastrulation with mouth forming second	BP	1	ACVR2A	up_VPOR_EC
GO:0042509	0.0758	0.997	1	17	regulation of tyrosine phosphorylation of STAT	BP	1	CAV1	up_VSAP_VSMC
GO:0071472	0.075894645	0.99802	1	3	cellular response to salt stress	BP	1	AQP1	up_ACOR_EC
GO:0007268	0.0759	0.957	16	130	chemical synaptic transmission	BP	0.912	EPHB1,PLK2,ENSCAFG00000007112,RELN,MEF2C,EDN1,SNCAIP,HOMER3,PPP3CA,GUCY1A1,SNAP23,SLC12A7,GRM8,STX11,ALS2,P2RX2	up_VCAV_EC
GO:0098916	0.0759	0.957	16	130	anterograde trans-synaptic signaling	BP	0.912	EPHB1,PLK2,ENSCAFG00000007112,RELN,MEF2C,EDN1,SNCAIP,HOMER3,PPP3CA,GUCY1A1,SNAP23,SLC12A7,GRM8,STX11,ALS2,P2RX2	up_VCAV_EC
GO:0001764	0.0759	0.968	8	45	neuron migration	BP	1	GJA1,NRP1,GPM6A,TBX20,FZD3,POMK,PSEN1,DISC1	up_ACOR_VSMC
GO:0060759	0.0759	0.975	5	37	regulation of response to cytokine stimulus	BP	0.889	NR1H4,PTPN2,ADAM17,CNOT7,CDC37	down_VSAP_EC
GO:0042100	0.0759	0.98	4	19	B cell proliferation	BP	0.912	IL7R,MEF2C,TLR4,PELI1	up_VCAV_EC
GO:0032350	0.0759	0.985	3	12	regulation of hormone metabolic process	BP	0.912	GATA3,BMP2,HIF1A	up_VCAV_EC
GO:0045662	0.0759	0.991	2	8	negative regulation of myoblast differentiation	BP	0.889	PRICKLE1,DDIT3	down_VSAP_EC
GO:0008406	0.0759	0.99	2	57	gonad development	BP	1	SEMA3A,ACVR2A	up_VPOR_EC
GO:0045137	0.0759	0.99	2	57	development of primary sexual characteristics	BP	1	SEMA3A,ACVR2A	up_VPOR_EC
GO:0044092	0.0759	0.999	1	270	negative regulation of molecular function	BP	1	CST3	down_AORT_EC
GO:1900086	0.0759	0.999	1	2	positive regulation of peptidyl-tyrosine	BP	0.889	VEGFA	down_VSAP_EC

GO:0009889	0.076	0.939	112	1190	regulation of biosynthetic process	BP	0.912	EDNRB,HCLS1,ADGRF5,KIT,PRKCH,FLI1,TNFAIP3,PPARG,TNIP1,PDGFB,TMEM173,RELN,CD34,IWS1,MEF2C,ATF3,CHD7,DLL4,WWP1,TLR4,NLRC5,QKI,GATA3,EDN1,HOXB8,RAB27A,TCF4,SMAD1,FOXN2,MEOX1,ENSCAFG00000017103,IRF8,SOX18,PELI1,PPP3CA,YES1,FAM3C,ARG2,MTOR,SAMD8,ELF4,BMP2,HMG20A,HHEX,CREM,CNOT6,RSC1A1,ENC1,CCAR1,PROX1,UHRF2,SECISBP2L,MDFI,ENSCAFG00000010596,ARID4B,HMBOX1,PRKD2,THOC1,SMARCA4,TLR3,NCK1,ESCO1,DPH5,TIMELESS,LDB2,CD200,JAG1,NSD1,PTGS2,RAPGEF2,TCERG1,CTDSPL2,CREB1,BCL10,BASP1,CNOT7,ENSCAFG00000002897,HMGB1,AHR,NOS2,ZNF789,BLM,NUP133,RIPK1,ZNF317,USP13,ITGA6,RAD17,SKIL,GPBP1,USP37,MTA2,NR1D2,KDM5A,HIF1A,KDM3A,ZNF146,ATRX,NR4A1,HIST2H2AC,HP1BP3,ARID5A,ENSCAFG00000002015,RBBP6,ZNF354A,DCP2,ZEB1,TET2,ARHGEF2,CDON,ELK3,ZNF609	up_VCAV_EC
GO:0016202	0.076	0.97	7	39	regulation of striated muscle tissue	BP	1	BMP4,FGFR2,GJA1,TBX20,TBX5,FRS2,EDN1	up_ACOR_VSMC
GO:0061035	0.076	0.976	5	24	regulation of cartilage development	BP	1	HOXA11,AXIN2,ZBTB16,RARG,SHOX2	up_VSAP_EC
GO:0070228	0.076	0.98	4	20	regulation of lymphocyte apoptotic process	BP	1	PRELID1,AURKB,ARG2,NOC2L	down_ACOR_VSMC
GO:0048731	0.076	0.988	3	1247	system development	BP	1	TGFBI,GATA5,TGFBR1	down_VSAP_VSMC
GO:0051567	0.076	0.986	3	10	histone H3-K9 methylation	BP	0.912	ARID4B,KDM3A,ATRX	up_VCAV_EC
GO:0007512	0.076	0.992	2	6	adult heart development	BP	0.912	CHD7,APLNR	up_VCAV_EC
GO:0045604	0.076	0.997	1	17	regulation of epidermal cell differentiation	BP	1	HOXA7	down_ACOR_EC
GO:0006470	0.076	0.997	1	54	protein dephosphorylation	BP	1	EYA1	up_AFEM_VSMC
GO:0045089	0.076	0.997	1	70	positive regulation of innate immune response	BP	1	ESR1	up_AORT_VSMC
GO:0009308	0.076	0.997	1	20	amine metabolic process	BP	1	NR1H4	up_APUL_EC
GO:0050732	0.076	0.997	1	18	negative regulation of peptidyl-tyrosine	BP	1	CAV1	up_VSAP_VSMC
GO:0061299	0.076008634	0.99802	1	3	retina vasculature morphogenesis in camera-	BP	1	NRP1	up_ACOR_EC
GO:0006911	0.0761	0.985	3	11	phagocytosis, engulfment	BP	1	F2RL1,XKR8,ARHGAP12	up_VSAP_EC
GO:0050779	0.0761	0.992	2	5	RNA destabilization	BP	1	ZFP36L1,ZFP36L2	up_VSAP_EC
GO:0061157	0.0761	0.992	2	5	mRNA destabilization	BP	1	ZFP36L1,ZFP36L2	up_VSAP_EC
GO:1903792	0.0761	0.992	2	5	negative regulation of anion transport	BP	1	GABRE,OSR1	up_VSAP_EC
GO:0032880	0.0761	0.998	1	266	regulation of protein localization	BP	1	JUP	down_VFEM_VSMC
GO:0060765	0.0761	0.997	1	10	regulation of androgen receptor signaling	BP	1	RNF6	up_VPOR_EC
GO:0043154	0.0761	0.997	1	23	negative regulation of cysteine-type endopeptidase activity	BP	1	PTGER2	up_VPUL_EC
GO:0016202	0.0761	0.997	1	39	regulation of striated muscle tissue	BP	1	TBX5	up_VPUL_VSMC
GO:0008645	0.07615329	0.98333	3	37	hexose transmembrane transport	BP	1	NR1H4,ENSCAFG00000004331,RARRES2	up_ACOR_EC
GO:0015749	0.07615329	0.98333	3	37	monosaccharide transmembrane transport	BP	1	NR1H4,ENSCAFG00000004331,RARRES2	up_ACOR_EC
GO:0034219	0.07615329	0.98333	3	37	carbohydrate transmembrane transport	BP	1	NR1H4,ENSCAFG00000004331,RARRES2	up_ACOR_EC
GO:0110053	0.0762	0.96	13	98	regulation of actin filament organization	BP	0.912	HCLS1,ARHGEF15,BAIAP2L1,ARHGAP28,TMOD1,MTOR,NCKAP1,IQGAP2,STMN1,SWAP70,NCK1,ARPC5L,TMOD3	up_VCAV_EC
GO:0046717	0.0762	0.98	4	17	acid secretion	BP	1	LEP,SLC26A7,HRH2,CFI	up_ACOR_VSMC
GO:0031098	0.0762	0.99	2	109	stress-activated protein kinase signaling cascade	BP	1	MAP2K6,CDC42EP5	up_VFEM_EC
GO:0007163	0.0762	0.997	1	60	establishment or maintenance of cell	BP	1	WNT7A	up_AORT_VSMC
GO:0003156	0.0762	0.997	1	20	regulation of animal organ formation	BP	1	TBX5	up_APUL_EC

GO:0048644	0.0762	0.997	1	31	muscle organ morphogenesis	BP	1	SHOX2	up_VFEM_VSMC
GO:0032602	0.0762	0.997	1	23	chemokine production	BP	1	NR1H4	up_VPUL_EC
GO:0097306	0.0762	0.997	1	22	cellular response to alcohol	BP	1	PTGER2	up_VPUL_EC
GO:0032611	0.076232966	0.98992	2	17	interleukin-1 beta production	BP	1	FZD5,NOD2	up_ACOR_EC
GO:0060759	0.07625201	0.9833	3	37	regulation of response to cytokine stimulus	BP	1	NR1H4,CCDC3,MAVS	up_ACOR_EC
GO:0032946	0.0763	0.976	5	29	positive regulation of mononuclear cell	BP	0.912	MEF2C,TLR4,PELI1,NCK1,HMGB1	up_VCAV_EC
GO:0050671	0.0763	0.976	5	29	positive regulation of lymphocyte proliferation	BP	0.912	MEF2C,TLR4,PELI1,NCK1,HMGB1	up_VCAV_EC
GO:0036010	0.0763	0.992	2	6	protein localization to endosome	BP	1	RAB35,EZR	down_ACOR_VSMC
GO:0046949	0.0763	0.992	2	6	fatty-acyl-CoA biosynthetic process	BP	1	ELOVL1,ELOVL6	down_ACOR_VSMC
GO:0035051	0.0763	0.997	1	41	cardiocyte differentiation	BP	1	CITED2	down_VPOR_EC
GO:0030334	0.0763	0.998	1	267	regulation of cell migration	BP	1	JUP	down_VFEM_VSMC
GO:0072358	0.0763	0.998	1	268	cardiovascular system development	BP	1	JUP	down_VFEM_VSMC
GO:0070265	0.0764	0.98	4	19	necrotic cell death	BP	0.912	PELI1,TLR3,RIPK1,ARHGEF2	up_VCAV_EC
GO:0046887	0.0764	0.984	3	18	positive regulation of hormone secretion	BP	0.892	NR1H4,PTPN11,HIF1A	down_VSAP_EC
GO:0009651	0.0764	0.992	2	5	response to salt stress	BP	1	ZFP36L1,SLC12A6	up_VSAP_EC
GO:0048538	0.0764	0.997	1	20	thymus development	BP	1	HOXA3	up_APUL_EC
GO:2000626	0.0764	1	1	1	negative regulation of miRNA catabolic process	BP	0.912	TENT2	up_VCAV_EC
GO:2000629	0.0764	1	1	1	negative regulation of miRNA metabolic process	BP	0.912	TENT2	up_VCAV_EC
GO:0003229	0.076488134	0.98986	2	17	ventricular cardiac muscle tissue development	BP	1	FOXC2,TBX5	up_ACOR_EC
GO:0060993	0.0765	0.983	3	42	kidney morphogenesis	BP	1	IRX3,GREM1,GCNT4	up_AFEM_EC
GO:0042327	0.0765	0.984	3	289	positive regulation of phosphorylation	BP	1	CCL24,PTGER2,ARHGEF2	up_VPUL_EC
GO:0010613	0.0765	0.992	2	5	positive regulation of cardiac muscle	BP	0.912	EDN1,PPP3CA	up_VCAV_EC
GO:0014742	0.0765	0.992	2	5	positive regulation of muscle hypertrophy	BP	0.912	EDN1,PPP3CA	up_VCAV_EC
GO:0019348	0.0765	1	1	1	dolichol metabolic process	BP	1	NUS1	down_ACOR_VSMC
GO:0019408	0.0765	1	1	1	dolichol biosynthetic process	BP	1	NUS1	down_ACOR_VSMC
GO:0010628	0.0765	1	1	527	positive regulation of gene expression	BP	1	GATA5	down_AFEM_VSMC
GO:0051894	0.0765	0.997	1	10	positive regulation of focal adhesion assembly	BP	1	ENSCAFG00000002015	up_VPOR_EC
GO:0032526	0.0765	0.997	1	19	response to retinoic acid	BP	1	CYP26B1	up_VFEM_EC
GO:0009887	0.076576027	0.95911	13	314	animal organ morphogenesis	BP	1	GATA4,SOX9,AQP1,FZD5,LHX9,SMAD6,DLX3, FOXC2,TBX5,LRP4,CLSTN1,NRP1,EFEMP1	up_ACOR_EC

GO:0044238	0.0766	0.935	300	3383	primary metabolic process	BP	1	FADS2,ELK3,SCD,ACSL3,PRPSAP1,SQLE,FADS1,HMGB1,MCM6,RNPS1,LSS,SREBF1,ACLY,MCM4,POLE3,USP1,IRAK1,MCM7,RPA1,PTBP1,ILF3,DDX39A,CBX5,ENSCAFG00000016095,PPRC1,USP4,SF3B2,BCAT1,ATAD2,HMGA1,EIF4A3,HMGCR,HNRNPUL1,EDC3,NNT,HNRNPD,SREBF2,MCM3,SMC1A,HINFP,VARS,UBE2S,NEDD4,SRSF2,RRM1,MED17,U2AF2,INTS7,PSMD3,POLA1,PSMD7,SLC29A1,MTDH,HEY1,METAP2,PPM1G,RRBP1,SNRPB2,TOP3A,TRIP13,PPP1R15B,SF1,PARPBP,POLA2,MPDU1,SRRT,GINS4,NOP56,CTDNEP1,RAD54L,POLE,DDX23,NAB2,SLC35C2,MRPS18B,RAN,B3GNT2,PRELID1,HM13,UBE2T,PSMD13,CDCA7L,FUS,RFC2,BIRC5,MYBBP1A,INSIG1,PRMT1,CAV1,CDK1,RFC5,RFC3,RAD51,CASP2,FANCI,XYLT1,FANCD2,U2AF1,TIMELESS,THRAP3,TRUB2,ENSCAFG00000018572,FAM129B,TCEA1,NF2,PFAS,EDC4,AURKB,NONO,MMP14,HNRNPL,TK1,CDKN2C,TOPBP1,ABHD5,EZH2,PPP2R5D,GREM1,MAP3K20,RFC4,NEK2,FBL,FEN1,CDK4,SMARCC1,MND1,RNASEH2B,PLK3,SFSWAP,PCNA,DHX33,HOXA7,SMG9,TONSL,ARG2,MCM10,SF3B4,PHF5A,UHRF1,CKS2,PDCD2,RNF152,DIS3,GMNN,DHCR24,BRCA1,NOC4L,MVD,WDR62,FASTKD3,NOC2L,EIF5A,E2F8,B4GALT1,P	down_ACOR_VSMC
GO:0045766	0.0766	0.969	7	63	positive regulation of angiogenesis	BP	0.894	VEGFA,SERPINE1,JCAD,PIK3C2A,HIF1A,MTDH,NRP1	down_VSAP_EC
GO:0010646	0.0766	0.977	5	945	regulation of cell communication	BP	1	BARX1,NKX2-5,BAMBI,ANKRD6,CYP26B1	up_VPOR_VSMC
GO:0006584	0.0766	0.985	3	11	catecholamine metabolic process	BP	1	MAOA,MOXD1,PARK7	up_ACOR_VSMC
GO:0009712	0.0766	0.985	3	11	catechol-containing compound metabolic	BP	1	MAOA,MOXD1,PARK7	up_ACOR_VSMC
GO:0070232	0.0766	0.985	3	13	regulation of T cell apoptotic process	BP	0.912	IL7R,ARG2,HIF1A	up_VCAV_EC
GO:0006591	0.0766	0.992	2	5	ornithine metabolic process	BP	1	HNF4A,AZIN2	up_VSAP_EC
GO:0050863	0.0766	0.997	1	69	regulation of T cell activation	BP	1	ARG2	up_AORT_VSMC
GO:0009312	0.0766	0.997	1	9	oligosaccharide biosynthetic process	BP	1	FBP1	up_VPOR_EC
GO:0010717	0.0766	0.997	1	32	regulation of epithelial to mesenchymal transition	BP	1	BAMBI	up_VPOR_VSMC
GO:0031331	0.076640956	0.9711	6	110	positive regulation of cellular catabolic process	BP	1	EDEM2,PIAS1,CLU,TP53INP2,RNF114,PRKCD	up_ACOR_EC
GO:0030010	0.0767	0.97	7	41	establishment of cell polarity	BP	0.912	EPHB1,FRMD4B,AMOT,FERMT1,GATA3,ZW10,FSCN1	up_VCAV_EC
GO:0090184	0.0767	0.985	3	14	positive regulation of kidney development	BP	0.912	HOXB7,PDGFB,GATA3	up_VCAV_EC
GO:0006139	0.0767	1	2	1909	nucleobase-containing compound metabolic	BP	1	JUP,GATA5	down_VFEM_VSMC
GO:0002009	0.0767	0.99	2	177	morphogenesis of an epithelium	BP	1	NKX2-5,ANKRD6	up_VPOR_VSMC
GO:0031663	0.0767	0.997	1	17	lipopolysaccharide-mediated signaling	BP	1	LYN	down_ACOR_EC
GO:0009743	0.0767	0.997	1	40	response to carbohydrate	BP	1	RAB11B	down_VPOR_EC
GO:0060562	0.0768	0.956	17	120	epithelial tube morphogenesis	BP	1	SHROOM3,BMP4,FGFR2,GJA1,SEC24B,CITED2,SPINT1,LMO4,CLSTN1,NRP1,NPNT,SPINT2,TBX20,FZD3,DAG1,PSEN1,EDN1	up_ACOR_VSMC
GO:0032502	0.0768	0.974	7	1661	developmental process	BP	1	HOXC10,DKK2,HOXA9,HOXC6,SGCD,TAOK1,DUSP2	up_AORT_EC
GO:0045669	0.0768	0.976	5	24	positive regulation of osteoblast differentiation	BP	1	BMP4,GJA1,CLSTN1,NPNT,DDR2	up_ACOR_VSMC
GO:0002465	0.0768	1	1	1	peripheral tolerance induction	BP	1	CD274	up_ACOR_VSMC
GO:0002652	0.0768	1	1	1	regulation of tolerance induction dependent upon immune response	BP	1	CD274	up_ACOR_VSMC
GO:0002654	0.0768	1	1	1	positive regulation of tolerance induction dependent upon immune	BP	1	CD274	up_ACOR_VSMC
GO:0002658	0.0768	1	1	1	regulation of peripheral tolerance induction	BP	1	CD274	up_ACOR_VSMC
GO:0002660	0.0768	1	1	1	positive regulation of peripheral tolerance	BP	1	CD274	up_ACOR_VSMC
GO:0002845	0.0768	1	1	1	positive regulation of tolerance induction to	BP	1	CD274	up_ACOR_VSMC

GO:0002413	0.0768	1	1	1	tolerance induction to tumor cell	BP	1	CD274	up_ACOR_VSMC
GO:0002461	0.0768	1	1	1	tolerance induction dependent upon immune	BP	1	CD274	up_ACOR_VSMC
GO:0002843	0.0768	1	1	1	regulation of tolerance induction to tumor cell	BP	1	CD274	up_ACOR_VSMC
GO:1905404	0.0768	1	1	1	positive regulation of activated CD8-positive, alpha-beta T cell	BP	1	CD274	up_ACOR_VSMC
GO:2001186	0.0768	1	1	1	negative regulation of CD8-positive, alpha-beta T cell activation	BP	1	CD274	up_ACOR_VSMC
GO:0021816	0.0768	1	1	1	extension of a leading process involved in cell motility in cerebral cortex radial glia guided	BP	0.912	ENSCAFG00000010224	up_VCAV_EC
GO:1902041	0.0768	0.997	1	23	regulation of extrinsic apoptotic signaling pathway via death	BP	1	ARHGEF2	up_VPUL_EC
GO:0050770	0.0768	0.997	1	39	regulation of axonogenesis	BP	1	SLIT2	up_VPUL_VSMC
GO:0034645	0.0769	0.973	7	1546	cellular macromolecule biosynthetic process	BP	1	EYA1,HOXA10,HOXA9,TBX15,EMX2,HOXA11, SHOX2	up_VFEM_VSMC
GO:0032945	0.0769	0.984	3	17	negative regulation of mononuclear cell	BP	0.897	LYN,SDC4,BTK	down_VSAP_EC
GO:0050672	0.0769	0.984	3	17	negative regulation of lymphocyte proliferation	BP	0.897	LYN,SDC4,BTK	down_VSAP_EC
GO:0032094	0.0769	0.992	2	6	response to food	BP	0.912	SLC25A25,BCL10	up_VCAV_EC
GO:0035405	0.0769	1	1	1	histone-threonine phosphorylation	BP	1	HASPIN	down_ACOR_VSMC
GO:0072355	0.0769	1	1	1	histone H3-T3 phosphorylation	BP	1	HASPIN	down_ACOR_VSMC
GO:0072356	0.0769	1	1	1	chromosome passenger complex localization to	BP	1	HASPIN	down_ACOR_VSMC
GO:2000751	0.0769	1	1	1	histone H3-T3 phosphorylation involved in chromosome passenger complex localization to	BP	1	HASPIN	down_ACOR_VSMC
GO:0048639	0.0769	0.997	1	39	positive regulation of developmental growth	BP	1	TBX5	up_VPUL_VSMC
GO:0006107	0.076917604	0.99797	1	3	oxaloacetate metabolic process	BP	1	NIT2	up_ACOR_EC
GO:0051651	0.077	0.972	6	31	maintenance of location in cell	BP	1	OS9,POLR2M,DAG1,GAA,SUN1,PARK7	up_ACOR_VSMC
GO:0043552	0.077	0.985	3	11	positive regulation of phosphatidylinositol 3-kinase activity	BP	0.912	TEK,PDGFB,SH3GLB1	up_VCAV_EC
GO:0090218	0.077	0.985	3	11	positive regulation of lipid kinase activity	BP	0.912	TEK,PDGFB,SH3GLB1	up_VCAV_EC
GO:0006730	0.077	0.991	2	8	one-carbon metabolic process	BP	0.897	ALDH1L2,ENSCAFG00000008716	down_VSAP_EC
GO:0045986	0.077	0.993	2	4	negative regulation of smooth muscle	BP	0.912	DOCK4,GUCY1A1	up_VCAV_EC
GO:0043407	0.077	0.997	1	19	negative regulation of MAP kinase activity	BP	1	LYN	down_ACOR_EC
GO:1901698	0.077	0.998	1	266	response to nitrogen compound	BP	1	JUP	down_VFEM_VSMC
GO:0036376	0.077	1	1	1	sodium ion export across plasma membrane	BP	1	FXYD1	up_ACOR_VSMC
GO:1903276	0.077	1	1	1	regulation of sodium ion export across plasma	BP	1	FXYD1	up_ACOR_VSMC
GO:1903278	0.077	1	1	1	positive regulation of sodium ion export across	BP	1	FXYD1	up_ACOR_VSMC
GO:0014706	0.077	0.998	1	112	striated muscle tissue development	BP	1	TBX5	up_APUL_VSMC

GO:2001141	0.0771	0.939	94	979	regulation of RNA biosynthetic process	BP	0.912	EDNRB,HCLS1,KIT,PRKCH,FLI1,TNFAIP3,PPARG,TNIP1,PDGFB,TMEM173,RELN,IWS1,MEF2C,ATF3,CHD7,DLL4,WWP1,TLR4,NLRC5,GATA3,EDN1,HOXB8,TCF4,SMAD1,FOXN2,MEOX1,ENSCAFG00000017103,IRF8,SOX18,PELI1,PPP3CA,YES1,MTOR,ELF4,BMP2,HMG20A,HHEX,CREM,CNOT6,RSC1A1,CCAR1,PROX1,UHRF2,MDFI,ENSCAFG00000010596,ARID4B,HMBOX1,PRKD2,THOC1,SMARCA4,TLR3,NCK1,TIMELESS,LDB2,CD200,JAG1,NSD1,TCERG1,CTDSPL2,CREB1,BCL10,BASP1,CNOT7,ENSCAFG00000002897,HMGB1,AHR,ZNF789,BLM,NUP133,RIPK1,ZNF317,USP13,ITGA6,SKIL,GPBP1,MTA2,NR1D2,KDM5A,HIF1A,KDM3A,ZNF146,ATRX,NR4A1,HIST2H2AC,HP1BP3,ARID5A,ENSCAFG00000002015,ZNF354A,ZEB1,TET2,ARHGEF2,CDON,ELK3,ZNF609	up_VCAV_EC
GO:2000026	0.0771	0.967	8	560	regulation of multicellular organismal development	BP	1	C3AR1,HOOK3,ARHGEF2,SEMA3A,ASPN,ACVR2A,MTDH,RNF6	up_VPOR_EC
GO:0002819	0.0771	0.969	7	44	regulation of adaptive immune response	BP	0.912	IL7R,TNFAIP3,MEF2C,GATA3,NECTIN2,THOC1,HMGB1	up_VCAV_EC
GO:1903076	0.0771	0.979	4	32	regulation of protein localization to plasma	BP	1	LRP1,PRNP,CLIP3,ENSCAFG00000014978	down_VCAV_EC
GO:0032330	0.0771	0.984	3	20	regulation of chondrocyte differentiation	BP	1	GLI3,GREM1,ZBTB16	down_VCAV_EC
GO:0055067	0.0771	0.983	3	42	monovalent inorganic cation homeostasis	BP	1	RAB20,RAB7A,ATP6V0B	up_AFEM_EC
GO:0016049	0.0771	0.983	3	124	cell growth	BP	1	SEMA3A,FBP1,RNF6	up_VPOR_EC
GO:1900003	0.0771	1	1	1	regulation of serine-type endopeptidase activity	BP	1	SPINK2	up_ACOR_VSMC
GO:1900004	0.0771	1	1	1	negative regulation of serine-type	BP	1	SPINK2	up_ACOR_VSMC
GO:1902571	0.0771	1	1	1	regulation of serine-type peptidase activity	BP	1	SPINK2	up_ACOR_VSMC
GO:1902572	0.0771	1	1	1	negative regulation of serine-type peptidase	BP	1	SPINK2	up_ACOR_VSMC
GO:0009064	0.0771	0.997	1	23	glutamine family amino acid metabolic process	BP	1	NR1H4	up_VPUL_EC
GO:0032079	0.077145777	0.99796	1	3	positive regulation of endodeoxyribonuclease	BP	1	PRKCD	up_ACOR_EC
GO:0051235	0.0772	0.966	8	90	maintenance of location	BP	1	FBN1,TLN2,THY1,PARK7,CYBA,ARL2BP,ENSCAFG00000017708,SOAT1	down_VCAV_EC
GO:0065008	0.0772	0.969	8	1056	regulation of biological quality	BP	1	NETO1,GATM,HOXC10,IRX5,PRR16,STIM1,CAV1,F5	up_VSAP_VSMC
GO:0009719	0.0772	0.984	3	395	response to endogenous stimulus	BP	1	FGF5,NKX2-5,BAMBI	up_VPOR_VSMC
GO:0009792	0.0772	0.99	2	231	embryo development ending in birth or egg	BP	1	HOXA3,HOXB5	up_VPUL_VSMC
GO:0030858	0.0772	0.997	1	17	positive regulation of epithelial cell	BP	1	ACVRL1	down_ACOR_EC
GO:0060840	0.0772	0.997	1	42	artery development	BP	1	CITED2	down_VPOR_EC
GO:0015849	0.0772	0.997	1	56	organic acid transport	BP	1	SLC16A12	up_AFEM_VSMC
GO:0046942	0.0772	0.997	1	56	carboxylic acid transport	BP	1	SLC16A12	up_AFEM_VSMC
GO:0072006	0.0772	0.997	1	59	nephron development	BP	1	EYA1	up_AFEM_VSMC
GO:0010092	0.0772	0.997	1	19	specification of animal organ identity	BP	1	HOXA11	up_VSAP_VSMC
GO:0032410	0.0772	0.997	1	17	negative regulation of transporter activity	BP	1	CAV1	up_VSAP_VSMC
GO:0070265	0.0772	0.997	1	19	necrotic cell death	BP	1	CAV1	up_VSAP_VSMC
GO:0071731	0.077249275	0.99795	1	3	response to nitric oxide	BP	1	GUCY1B1	up_ACOR_EC
GO:0071241	0.0773	0.983	3	42	cellular response to inorganic substance	BP	1	MT2A,ENSCAFG00000009113,CPNE7	up_AFEM_EC
GO:0045603	0.0773	0.992	2	5	positive regulation of endothelial cell	BP	1	BTG1,ACVRL1	up_VSAP_EC
GO:1904894	0.0773	0.997	1	17	positive regulation of STAT cascade	BP	1	LYN	down_ACOR_EC
GO:0071804	0.0773	0.997	1	31	cellular potassium ion transport	BP	1	NETO1	up_VFEM_VSMC
GO:0071805	0.0773	0.997	1	31	potassium ion transmembrane transport	BP	1	NETO1	up_VFEM_VSMC
GO:0038189	0.077329312	0.99795	1	3	neuropilin signaling pathway	BP	1	NRP1	up_ACOR_EC

GO:0072525	0.0774	0.976	5	24	pyridine-containing compound biosynthetic	BP	1	NADK,GALK1,PNPO,PNP,DDIT4	up_VSAP_EC
GO:0048545	0.0774	0.978	4	68	response to steroid hormone	BP	1	LBH,CRY2,FIBIN,NR3C1	up_AFEM_EC
GO:0060337	0.0774	0.985	3	12	type I interferon signaling pathway	BP	1	IRAK1,SLC29A1,CACTIN	down_ACOR_VSMC
GO:0071357	0.0774	0.985	3	12	cellular response to type I interferon	BP	1	IRAK1,SLC29A1,CACTIN	down_ACOR_VSMC
GO:0003018	0.0774	0.983	3	42	vascular process in circulatory system	BP	1	HTR1D,AVPR1A,TBXA2R	up_AFEM_EC
GO:0050870	0.0774	0.983	3	42	positive regulation of T cell activation	BP	1	LEP,GPAM,CYLD	up_AFEM_EC
GO:0051048	0.0774	0.983	3	42	negative regulation of secretion	BP	1	LEP,CRY2,RAB7A	up_AFEM_EC
GO:0048799	0.0774	0.99	2	10	animal organ maturation	BP	1	THBS3,GREM1	down_VCAV_EC
GO:2000780	0.0774	0.997	1	11	negative regulation of double-strand break	BP	1	CXHXorf57	up_VPOR_EC
GO:1904063	0.0774	0.997	1	17	negative regulation of cation transmembrane	BP	1	CAV1	up_VSAP_VSMC
GO:0033240	0.077449616	0.99794	1	3	positive regulation of cellular amine metabolic	BP	1	NR1H4	up_ACOR_EC
GO:1902531	0.077499773	0.95298	20	536	regulation of intracellular signal transduction	BP	1	NR1H4,SOX9,NDRG2,FZD5,DUSP26,NOD2,PDE4A,PAK3,TNFRSF19,ARHGDIB,MAPK8IP2,FIGNL1,LRRC15,SH3RF3,LRRC4,CLU,C23H3orf58,NRP1,ARHGEF26,PRKCD	up_ACOR_EC
GO:0032970	0.0775	0.955	18	130	regulation of actin filament-based process	BP	1	SYNPO2L,FGF13,ARHGAP28,ACTN2,KCNE3,SYNPO2,F2RL1,JMY,NRP1,EVL,PRKCE,JUP,SSH3,EDN1,FAM107A,JAM3,INPP5K,WASL	up_ACOR_VSMC
GO:0006952	0.0775	0.96	12	319	defense response	BP	1	LEP,RAB20,CD40,F3,BPI,GPAM,HCK,CYLD,TBXA2R,NAPEPLD,CCL5,ADAM17	up_AFEM_EC
GO:0006839	0.0775	0.962	11	86	mitochondrial transport	BP	1	SREBF1,SLC25A5,TIMM44,SLC25A15,TOMM34,SAMM50,CYC1,SLC25A16,PMPCA,MRPL18,SLC25A19	down_ACOR_VSMC
GO:0009145	0.0775	0.974	5	40	purine nucleoside triphosphate biosynthetic	BP	0.902	PGK1,TPI1,PGAM1,HIF1A,ALDOC	down_VSAP_EC
GO:0034103	0.0775	0.985	3	12	regulation of tissue remodeling	BP	1	GREM1,B4GALT1,TFRC	down_ACOR_VSMC
GO:0032516	0.0775	0.991	2	8	positive regulation of phosphoprotein phosphatase activity	BP	0.902	PPP1R12A,CALM1	down_VSAP_EC
GO:0045605	0.0775	0.992	2	5	negative regulation of epidermal cell	BP	1	HOXA7,DLL1	up_VSAP_EC
GO:0072643	0.0775	0.992	2	5	interferon-gamma secretion	BP	1	ABL1,F2RL1	up_VSAP_EC
GO:0043244	0.0775	0.997	1	34	regulation of protein complex disassembly	BP	1	TAOK1	up_AORT_EC
GO:0042310	0.0775	0.997	1	21	vasoconstriction	BP	1	HTR7	up_APUL_EC
GO:0048640	0.0775	0.997	1	21	negative regulation of developmental growth	BP	1	TBX5	up_APUL_EC
GO:0097756	0.0775	0.997	1	21	negative regulation of blood vessel diameter	BP	1	HTR7	up_APUL_EC
GO:2001271	0.0775	1	1	1	negative regulation of cysteine-type endopeptidase activity involved in execution	BP	0.913	PAK2	up_VCAV_EC
GO:0051051	0.0776	0.958	14	112	negative regulation of transport	BP	1	HMGB1,SREBF1,SREBF2,NEDD4,HOXA3,SLC25A5,INSIG1,CAV1,SLC30A1,ARG2,GSTO1,EZR,CORO1A,REM1	down_ACOR_VSMC
GO:0031109	0.0776	0.972	6	36	microtubule polymerization or	BP	0.913	MZT1,STMN1,FES,TAOK1,ARHGEF2,FBXO5	up_VCAV_EC
GO:0009855	0.0776	0.983	3	42	determination of bilateral symmetry	BP	1	DLL1,GREM1,GREM2	up_AFEM_EC
GO:0048752	0.0776	0.992	2	5	semicircular canal morphogenesis	BP	0.913	CHD7,ZEB1	up_VCAV_EC
GO:0070307	0.0776	0.991	2	6	lens fiber cell development	BP	0.913	TMOD1,PROX1	up_VCAV_EC
GO:1901524	0.0776	0.992	2	5	regulation of mitophagy	BP	1	MFN2,HTRA2	up_VSAP_EC
GO:0010720	0.0776	0.989	2	110	positive regulation of cell development	BP	1	HOXA11,SHOX2	up_VSAP_VSMC
GO:0048593	0.0776	0.997	1	41	camera-type eye morphogenesis	BP	1	CITED2	down_VPOR_EC
GO:0070586	0.0776	1	1	1	cell-cell adhesion involved in gastrulation	BP	1	MBP	up_ACOR_VSMC
GO:1904207	0.0776	1	1	1	regulation of chemokine (C-C motif) ligand 2	BP	1	MBP	up_ACOR_VSMC
GO:1904209	0.0776	1	1	1	positive regulation of chemokine (C-C motif)	BP	1	MBP	up_ACOR_VSMC

GO:0034115	0.0776	1	1	1	negative regulation of heterotypic cell-cell	BP	1	MBP	up_ACOR_VSMC
GO:0035633	0.0776	1	1	1	maintenance of permeability of blood-	BP	1	MBP	up_ACOR_VSMC
GO:0070587	0.0776	1	1	1	regulation of cell-cell adhesion involved in	BP	1	MBP	up_ACOR_VSMC
GO:2000343	0.0776	1	1	1	positive regulation of chemokine (C-X-C motif)	BP	1	MBP	up_ACOR_VSMC
GO:1990266	0.0776	0.997	1	24	neutrophil migration	BP	1	CCL24	up_VPUL_EC
GO:0031644	0.0776	0.997	1	19	regulation of neurological system process	BP	1	NETO1	up_VSAP_VSMC
GO:0071609	0.077650952	0.99793	1	3	chemokine (C-C motif) ligand 5 production	BP	1	MAVS	up_ACOR_EC
GO:0071649	0.077650952	0.99793	1	3	regulation of chemokine (C-C motif) ligand 5	BP	1	MAVS	up_ACOR_EC
GO:0043043	0.0777	0.954	18	247	peptide biosynthetic process	BP	1	UBA52,LARP6,COA1,SAMD4B,RPL23,RPL13,EIF3K,MRPL37,RPL27A,EEF2,ZFP36L1,RPL27,MRPS11,RPS27,ENSCAFG00000015122,PYM1,FOXO3,EIF4EBP1	down_VCAV_EC
GO:0007249	0.0777	0.962	11	71	I-kappaB kinase/NF-kappaB signaling	BP	1	MAP3K14,ABL1,F2RL1,RBCK1,MYD88,S100A4,NLRX1,ENSCAFG00000011993,MAPKBP1,IRAK1BP1,TRADD	up_VSAP_EC
GO:0033002	0.0777	0.965	9	55	muscle cell proliferation	BP	1	BMP4,FGFR2,PTGIR,NDRG2,CDKN1A,CLSTN1,TBX20,TBX5,EDN1	up_ACOR_VSMC
GO:0046822	0.0777	0.965	9	53	regulation of nucleocytoplasmic	BP	1	LEP,FERMT1,BMP4,CDKN1A,PTPN22,PSEN1,JUP,PARK7,PTPN14	up_ACOR_VSMC
GO:0050896	0.0777	0.98	6	2346	response to stimulus	BP	1	SLITRK5,CYP1A1,LIMK1,GATA5,CA2,C6	up_VCAV_VSMC
GO:0006807	0.0777	0.99	5	3221	nitrogen compound metabolic process	BP	1	AK5,GATA5,TGFBR1,MEIS2,ENSCAFG00000028814	down_VSAP_VSMC
GO:0043457	0.0777	0.985	3	11	regulation of cellular respiration	BP	1	PPARGC1A,BNIP3,PARK7	up_ACOR_VSMC
GO:0055082	0.0777	0.99	2	189	cellular chemical homeostasis	BP	1	TRPC4,C3AR1	up_VPOR_VSMC
GO:0000413	0.0777	1	1	1	protein peptidyl-prolyl isomerization	BP	1	PPIB	down_ACOR_VSMC
GO:1901077	0.0777	1	1	1	regulation of relaxation of muscle	BP	0.913	PDE4D	up_VCAV_EC
GO:1901078	0.0777	1	1	1	negative regulation of relaxation of muscle	BP	0.913	PDE4D	up_VCAV_EC
GO:1901897	0.0777	1	1	1	regulation of relaxation of cardiac muscle	BP	0.913	PDE4D	up_VCAV_EC
GO:1901898	0.0777	1	1	1	negative regulation of relaxation of cardiac	BP	0.913	PDE4D	up_VCAV_EC
GO:0048584	0.0778	0.944	44	592	positive regulation of response to stimulus	BP	0.903	VEGFA,SULF1,NR1H4,SERPINE1,SOCS5,JCAD,FAM49B,PTPN11,ILK,SMARCA4,PTGS2,MYO1C,CHSY1,HIF1A,TNIK,PTPN2,EIF2B1,PVR,NEO1,MTDH,ESM1,ADAM17,SKAP2,MAGED1,CTNNB1,ENSCAFG00000002015,TFRC,LYN,BNIP3,FAM162A,ATF3,GBP5,SH3GLB1,HTT,CALM1,DISC1,PAK2,A2M,INHBA,NRP1,SH3RF3,MADD,SEC13,MID1	down_VSAP_EC
GO:0072073	0.0778	0.968	7	60	kidney epithelium development	BP	0.903	ENSCAFG00000032470,ILK,HOXB7,MAGED1,CTNNB1,MYO1E,SIX4	down_VSAP_EC
GO:0045638	0.0778	0.997	1	33	negative regulation of myeloid cell	BP	1	HOXA9	up_AORT_EC
GO:0010594	0.0778	0.997	1	71	regulation of endothelial cell migration	BP	1	WNT7A	up_AORT_VSMC
GO:0055007	0.0778	0.997	1	31	cardiac muscle cell differentiation	BP	1	NKX2-5	up_VPOR_VSMC
GO:0010092	0.0778	0.997	1	19	specification of animal organ identity	BP	1	SIX1	up_VFEM_EC
GO:0010977	0.0779	0.978	4	33	negative regulation of neuron projection	BP	1	THY1,TLX2,PRKCSH,RGMA	down_VCAV_EC
GO:0048585	0.0779	0.979	4	475	negative regulation of response to stimulus	BP	1	NR1H4,PTGER2,FLRT3,ARHGEF2	up_VPUL_EC
GO:0071345	0.0779	0.983	3	210	cellular response to cytokine stimulus	BP	1	NRP2,STX4,ASPN	down_ACOR_EC
GO:0043170	0.0779	1	3	2980	macromolecule metabolic process	BP	1	PLCL1,MOSPD1,USP12	down_VFEM_EC
GO:0051090	0.0779	0.989	2	134	regulation of DNA-binding transcription factor	BP	1	NR1H4,ARHGEF2	up_VPUL_EC
GO:0009407	0.0779	1	1	1	toxin catabolic process	BP	1	GSTO1	down_ACOR_VSMC
GO:0006952	0.077926707	0.95826	13	319	defense response	BP	1	NR1H4,FNDC4,NOD2,IL33,TRIM8,PTGER4,MAPK8IP2,MAVS,IFIT2,CLSTN1,RARRES2,AGBL5,PRKCD	up_ACOR_EC
GO:0007212	0.078	0.985	3	11	dopamine receptor signaling pathway	BP	1	ADCY6,GNA14,GNAQ	up_ACOR_VSMC

GO:2000241	0.078	0.997	1	40	regulation of reproductive process	BP	1	CITED2	down_VPOR_EC
GO:1904888	0.078	0.997	1	31	cranial skeletal system development	BP	1	TBX15	up_VFEM_VSMC
GO:0048634	0.078	0.997	1	40	regulation of muscle organ development	BP	1	TBX5	up_VPUL_VSMC
GO:0032740	0.07806648	0.99791	1	3	positive regulation of interleukin-17 production	BP	1	NOD2	up_ACOR_EC
GO:0000820	0.078077209	0.99791	1	3	regulation of glutamine family amino acid	BP	1	NR1H4	up_ACOR_EC
GO:0009894	0.0781	0.951	23	277	regulation of catabolic process	BP	0.905	PRICKLE1,PLK3,SOCS5,TRIB3,PGAM1,SESN2,MSN,HIF1A,UCHL5,SMG7,MTDH,CNOT7,APOE,BNIP3,GSK3B,CDC37,SH3GLB1,HTT,ABHD5,DISC1,RAD23A,TMTC3,USP14	down_VSAP_EC
GO:0060055	0.0781	0.991	2	8	angiogenesis involved in wound healing	BP	0.905	SERPINE1,PRCP	down_VSAP_EC
GO:0007267	0.0781	0.99	2	332	cell-cell signaling	BP	1	SLITRK5,CA2	up_VCAV_VSMC
GO:0035801	0.0781	1	1	1	adrenal cortex development	BP	1	CITED2	up_ACOR_VSMC
GO:0035802	0.0781	1	1	1	adrenal cortex formation	BP	1	CITED2	up_ACOR_VSMC
GO:0048754	0.0781	0.997	1	57	branching morphogenesis of an epithelial tube	BP	1	EYA1	up_AFEM_VSMC
GO:0046503	0.0781	0.997	1	10	glycerolipid catabolic process	BP	1	ABHD5	up_VPOR_EC
GO:0070633	0.078106349	0.9979	1	3	transepithelial transport	BP	1	AQP1	up_ACOR_EC
GO:0070863	0.078183618	0.9979	1	3	positive regulation of protein exit from	BP	1	EDEM2	up_ACOR_EC
GO:1901701	0.0782	0.947	33	271	cellular response to oxygen-containing compound	BP	1	PTGFR,PPARGC1B,SLIT2,CDH1,ABL1,GOT1,CHRM1,OSR1,MAP3K5,PDGFD,KLF15,RARG,MGST1,STAT5A,RAB11B,PDK2,ZFP36L1,PIK3R1,ZEB1,SASH1,MYD88,CASTOR1,KCNB1,OSBPL7,RAF1,ZNF580,STX4,HTRA2,PPP3CB,NCK1,BGLAP,DDIT4,RAB11FIP2	up_VSAP_EC
GO:0042742	0.0782	0.969	7	39	defense response to bacterium	BP	1	F2RL1,PLA2G6,MYD88,MAPKBP1,CYBA,MAVS,JCHAIN	up_VSAP_EC
GO:0098739	0.0782	0.983	3	20	import across plasma membrane	BP	1	PRNP,FOLR2,SLC39A6	down_VCAV_EC
GO:0000266	0.0782	0.984	3	17	mitochondrial fission	BP	0.905	ENSCAFG00000007260,BNIP3,SLC25A46	down_VSAP_EC
GO:0043243	0.0782	0.985	3	11	positive regulation of protein complex	BP	1	ACTN2,F2RL1,BNIP3	up_ACOR_VSMC
GO:0051241	0.0782	0.99	2	336	negative regulation of multicellular organismal	BP	1	HOXC10,HOXA9	up_AFEM_VSMC
GO:0019730	0.0782	0.992	2	5	antimicrobial humoral response	BP	1	PLA2G6,JCHAIN	up_VSAP_EC
GO:0042771	0.0782	0.997	1	16	intrinsic apoptotic signaling pathway in response to DNA damage	BP	1	USP28	down_ACOR_EC
GO:0000494	0.0782	1	1	1	box C/D snoRNA 3'-end processing	BP	1	FBL	down_ACOR_VSMC
GO:1990258	0.0782	1	1	1	histone glutamine methylation	BP	1	FBL	down_ACOR_VSMC
GO:0033967	0.0782	1	1	1	box C/D snoRNA metabolic process	BP	1	FBL	down_ACOR_VSMC
GO:0034963	0.0782	1	1	1	box C/D snoRNA processing	BP	1	FBL	down_ACOR_VSMC
GO:0030879	0.0782	0.997	1	34	mammary gland development	BP	1	HOXA9	up_AORT_EC
GO:0007249	0.0782	0.997	1	71	I-kappaB kinase/NF-kappaB signaling	BP	1	ESR1	up_AORT_VSMC
GO:0051650	0.0782	0.997	1	65	establishment of vesicle localization	BP	1	WNT7A	up_AORT_VSMC
GO:0021545	0.0782	0.997	1	21	cranial nerve development	BP	1	SIX1	up_VFEM_EC
GO:0055123	0.0782	0.997	1	40	digestive system development	BP	1	GATA5	up_VPUL_VSMC
GO:0003278	0.078236973	0.9979	1	3	apoptotic process involved in heart	BP	1	FOXC2	up_ACOR_EC
GO:0030950	0.078245193	0.9979	1	3	establishment or maintenance of actin	BP	1	AQP1	up_ACOR_EC
GO:0019627	0.078258124	0.9979	1	3	urea metabolic process	BP	1	NR1H4	up_ACOR_EC
GO:0030500	0.0783	0.975	5	25	regulation of bone mineralization	BP	1	BMP4,GJA1,ISG15,DDR2,SLC8A1	up_ACOR_VSMC
GO:0048627	0.0783	1	1	1	myoblast development	BP	1	SDC1	down_ACOR_VSMC

GO:0019064	0.0783	1	1	1	fusion of virus membrane with host plasma	BP	0.913	NECTIN2	up_VCAV_EC
GO:0030382	0.0783	1	1	1	sperm mitochondrion organization	BP	0.913	NECTIN2	up_VCAV_EC
GO:0039663	0.0783	1	1	1	membrane fusion involved in viral entry into	BP	0.913	NECTIN2	up_VCAV_EC
GO:0046814	0.0783	1	1	1	coreceptor-mediated virion attachment to host	BP	0.913	NECTIN2	up_VCAV_EC
GO:0044800	0.0783	1	1	1	multi-organism membrane fusion	BP	0.913	NECTIN2	up_VCAV_EC
GO:0044803	0.0783	1	1	1	multi-organism membrane organization	BP	0.913	NECTIN2	up_VCAV_EC
GO:1902532	0.0784	0.954	18	204	negative regulation of intracellular signal transduction	BP	0.906	ARHGEF2,NR1H4,SOCS5,HYOU1,FHL2,SESN2,PTGS2,HIF1A,PTPN2,APOE,WWC3,LYN,DDIT3,CBLC,ATF3,GSK3B,CALM1,TNFAIP1	down_VSAP_EC
GO:0007600	0.0784	0.963	10	73	sensory perception	BP	0.913	EPHB1,KIT,CHD7,HOXB8,TMEM150C,NAV2,ACPP,MMP24,SPTBN4,P2RX2	up_VCAV_EC
GO:0007160	0.0784	0.965	9	56	cell-matrix adhesion	BP	1	HOXA7,FAM107A,HOXD3,ABL1,EMP2,HPSE,PIK3R1,TIAM1,ACVRL1	up_VSAP_EC
GO:0007272	0.0784	0.972	6	31	ensheathment of neurons	BP	1	SH3TC2,MBP,WASF3,ATRN,DAG1,JAM3	up_ACOR_VSMC
GO:0008366	0.0784	0.972	6	31	axon ensheathment	BP	1	SH3TC2,MBP,WASF3,ATRN,DAG1,JAM3	up_ACOR_VSMC
GO:0034141	0.0784	0.992	2	5	positive regulation of toll-like receptor 3 signaling	BP	1	F2RL1,FLOT1	up_VSAP_EC
GO:0006119	0.0784	0.997	1	40	oxidative phosphorylation	BP	1	NDUFA10	down_AFEM_EC
GO:0033365	0.0784	0.998	1	276	protein localization to organelle	BP	1	JUP	down_VFEM_VSMC
GO:1901701	0.0784	0.998	1	271	cellular response to oxygen-containing	BP	1	JUP	down_VFEM_VSMC
GO:0032675	0.0784	0.997	1	34	regulation of interleukin-6 production	BP	1	BPI	up_AORT_EC
GO:0010634	0.0784	0.997	1	68	positive regulation of epithelial cell migration	BP	1	WNT7A	up_AORT_VSMC
GO:0060560	0.0784	0.997	1	58	developmental growth involved in	BP	1	LIMK1	up_VCAV_VSMC
GO:0021987	0.0784	0.997	1	37	cerebral cortex development	BP	1	EMX2	up_VFEM_VSMC
GO:0032930	0.078498869	0.99788	1	3	positive regulation of superoxide anion	BP	1	PRKCD	up_ACOR_EC
GO:0040008	0.0785	0.955	16	179	regulation of growth	BP	0.907	CELF1,ENPP1,PTPN11,IFRD1,SMARCA4,SESN2,HIF1A,ZPR1,ESM1,ADAM17,WWC3,IGFBP7,INHBA,SIX4,CDKN1A,NRP1	down_VSAP_EC
GO:0071103	0.0785	0.961	11	80	DNA conformation change	BP	0.913	PSME4,NASP,HHEX,HIST1H1A,TOP1,ASF1A,HMGB1,BLM,ATRX,HP1BP3,GINS4	up_VCAV_EC
GO:0043405	0.0785	0.964	9	85	regulation of MAP kinase activity	BP	0.907	VEGFA,PTPN11,TRIB3,TNIK,MAGED1,APOE,LYN,CBLC,MADD	down_VSAP_EC
GO:0098868	0.0785	0.985	3	11	bone growth	BP	1	RARG,DDR2,TGFBR2	up_VSAP_EC
GO:0019222	0.0785	1	2	1934	regulation of metabolic process	BP	1	JUP,GATA5	down_VFEM_VSMC
GO:0051216	0.0785	0.997	1	70	cartilage development	BP	1	WNT7A	up_AORT_VSMC
GO:0048518	0.0786	0.965	11	1676	positive regulation of biological process	BP	1	EYA1,HOXA9,TOX,MAP2K6,AVPR1A,HOXB7,SIX1,MAB21L1,TIAM1,CYP26B1,CDC42EP5	up_VFEM_EC
GO:0051494	0.0786	0.969	7	44	negative regulation of cytoskeleton organization	BP	1	CAPG,LIMA1,STMN1,NEK2,RBM14,CORO1A,ARHGEF2	down_ACOR_VSMC
GO:0072376	0.0786	0.985	3	11	protein activation cascade	BP	1	KLKB1,CFD,FBLN1	up_VSAP_EC
GO:0097340	0.0786	0.991	2	6	inhibition of cysteine-type endopeptidase activity	BP	1	BIRC5,ENSCAFG00000005299	down_ACOR_VSMC
GO:0097341	0.0786	0.991	2	6	zymogen inhibition	BP	1	BIRC5,ENSCAFG00000005299	down_ACOR_VSMC
GO:0040034	0.0786	0.992	2	5	regulation of development,	BP	1	DLL1,GDPD5	up_VSAP_EC
GO:0002888	0.0786	0.992	2	5	positive regulation of myeloid leukocyte	BP	1	F2RL1,STX4	up_VSAP_EC
GO:2000736	0.0786	0.997	1	21	regulation of stem cell differentiation	BP	1	TBX5	up_APUL_EC
GO:0007497	0.0786	1	1	1	posterior midgut development	BP	0.913	EDNRB	up_VCAV_EC
GO:0014043	0.0786	1	1	1	negative regulation of neuron maturation	BP	0.913	EDNRB	up_VCAV_EC
GO:0035645	0.0786	1	1	1	enteric smooth muscle cell differentiation	BP	0.913	EDNRB	up_VCAV_EC
GO:0086100	0.0786	1	1	1	endothelin receptor signaling pathway	BP	0.913	EDNRB	up_VCAV_EC
GO:1903430	0.0786	1	1	1	negative regulation of cell maturation	BP	0.913	EDNRB	up_VCAV_EC

GO:1902338	0.07862149	0.99788	1	3	negative regulation of apoptotic process involved in	BP	1	FOXC2	up_ACOR_EC
GO:1904746	0.07862149	0.99788	1	3	negative regulation of apoptotic process	BP	1	FOXC2	up_ACOR_EC
GO:0045446	0.0787	0.972	6	32	endothelial cell differentiation	BP	1	BTG1,DLL1,F2RL1,ZEB1,MYD88,ACVRL1	up_VSAP_EC
GO:0042089	0.0787	0.979	4	21	cytokine biosynthetic process	BP	0.913	TLR4,GATA3,TLR3,BCL10	up_VCAV_EC
GO:0040001	0.0787	0.984	3	12	establishment of mitotic spindle localization	BP	1	ZW10,CENPA,NDC80	down_ACOR_VSMC
GO:0007193	0.0787	0.984	3	13	adenylate cyclase-inhibiting G protein-coupled receptor signaling	BP	0.913	EDN1,AKAP12,GRM8	up_VCAV_EC
GO:0060548	0.0787	0.983	3	294	negative regulation of cell death	BP	1	NR1H4,PTGER2,ARHGEF2	up_VPUL_EC
GO:2000026	0.0787	0.991	2	560	regulation of multicellular organismal development	BP	1	GATA5,TGFBR1	down_VSAP_VSMC
GO:0042635	0.0787	0.992	2	5	positive regulation of hair cycle	BP	1	HPSE,TRADD	up_VSAP_EC
GO:0051798	0.0787	0.992	2	5	positive regulation of hair follicle development	BP	1	HPSE,TRADD	up_VSAP_EC
GO:0070571	0.078763932	0.99787	1	3	negative regulation of neuron projection	BP	1	RGMA	up_ACOR_EC
GO:0051891	0.07877685	0.99787	1	3	positive regulation of cardioblast differentiation	BP	1	TBX5	up_ACOR_EC
GO:0009890	0.0788	0.943	44	420	negative regulation of biosynthetic process	BP	1	ELK3,HMGB1,SREBF1,ILF3,CBX5,ATAD2,HMGA1,EIF4A3,SREBF2,SMC1A,NEDD4,MTDH,NAB2,BIRC5,INSIG1,CAV1,TIMELESS,FAM129B,AURKB,EZH2,GREM1,PLK3,HOXA7,UHRF1,GMNN,BRCA1,NOC2L,PPARD,NCL,SUV39H1,HMGB2,DMAP1,SMC3,CDK2,ENSCAFG00000005299,EZR,RBM14,ERF,TCERG1,HAT1,HIST2H2AC,TINF2,H2AFX,SIX4	down_ACOR_VSMC
GO:0031668	0.0788	0.96	12	79	cellular response to extracellular stimulus	BP	1	PRKAA2,CASTOR1,CDKN2B,GABARAPL1,CDKN1A,SESN3,PIK3C3,LAMP2,SESN1,FAM107A,BMT2,ZFYVE1	up_ACOR_VSMC
GO:1903727	0.0788	0.984	3	16	positive regulation of phospholipid metabolic	BP	0.907	NR1H4,LYN,SH3GLB1	down_VSAP_EC
GO:0002428	0.0788	1	1	1	antigen processing and presentation of peptide antigen via MHC class Ib	BP	1	B2M	up_ACOR_VSMC
GO:0002477	0.0788	1	1	1	antigen processing and presentation of exogenous peptide	BP	1	B2M	up_ACOR_VSMC
GO:0002481	0.0788	1	1	1	antigen processing and presentation of exogenous protein antigen via MHC class Ib,	BP	1	B2M	up_ACOR_VSMC
GO:0010041	0.0788	1	1	1	response to iron(III) ion	BP	1	B2M	up_ACOR_VSMC
GO:0071283	0.0788	1	1	1	cellular response to iron(III) ion	BP	1	B2M	up_ACOR_VSMC
GO:1900390	0.0788	1	1	1	regulation of iron ion import	BP	1	B2M	up_ACOR_VSMC
GO:1904435	0.0788	1	1	1	regulation of transferrin receptor binding	BP	1	B2M	up_ACOR_VSMC
GO:1904437	0.0788	1	1	1	positive regulation of transferrin receptor	BP	1	B2M	up_ACOR_VSMC
GO:0034756	0.0788	1	1	1	regulation of iron ion transport	BP	1	B2M	up_ACOR_VSMC
GO:1904432	0.0788	1	1	1	regulation of ferrous iron binding	BP	1	B2M	up_ACOR_VSMC
GO:1904434	0.0788	1	1	1	positive regulation of ferrous iron binding	BP	1	B2M	up_ACOR_VSMC
GO:0018216	0.078823351	0.99786	1	3	peptidyl-arginine methylation	BP	1	NR1H4	up_ACOR_EC
GO:0045786	0.0789	0.951	22	195	negative regulation of cell cycle	BP	0.913	PLK2,CXCL8,TNFAIP3,GATA3,BMP2,HHEX,PROX1,UHRF2,THOC1,ETAA1,USP28,TAOK1,BLM,RAD21,RAD17,SKIL,CUL4A,ZW10,ATRX,NR4A1,TOPBP1,FBXO5	up_VCAV_EC
GO:0034654	0.0789	0.965	10	1236	nucleobase-containing compound biosynthetic	BP	1	ACVRL1,YEATS2,YBX3,HIVEP2,HOXA7,SULF1,ELK3,HOXA1,TNRC6B,NEDD4	down_ACOR_EC
GO:0019882	0.0789	0.975	5	25	antigen processing and presentation	BP	1	CTSS,DLA88,B2M,ERAP1,TAPBPL	up_ACOR_VSMC
GO:0033559	0.0789	0.979	4	21	unsaturated fatty acid metabolic process	BP	0.913	EDN1,PTGS1,ELOVL7,PTGS2	up_VCAV_EC
GO:0003071	0.0789	0.992	2	5	renal system process involved in regulation of systemic arterial blood	BP	1	EMP2,F2RL1	up_VSAP_EC

GO:0055026	0.0789	0.992	2	5	negative regulation of cardiac muscle tissue	BP	1	DLL1,SAV1	up_VSAP_EC
GO:0071526	0.0789	0.997	1	12	semaphorin-plexin signaling pathway	BP	1	NRP2	down_ACOR_EC
GO:0009888	0.0789	1	1	545	tissue development	BP	1	GATA5	down_AFEM_VSMC
GO:0071560	0.0789	0.997	1	72	cellular response to transforming growth	BP	1	WNT7A	up_AORT_VSMC
GO:0051055	0.0789	0.997	1	21	negative regulation of lipid biosynthetic process	BP	1	NR1H4	up_APUL_EC
GO:0070252	0.0789	0.997	1	17	actin-mediated cell contraction	BP	1	CAV1	up_VSAP_VSMC
GO:0031115	0.07894771	0.99786	1	3	negative regulation of microtubule	BP	1	TUBB4A	up_ACOR_EC
GO:0070661	0.079	0.968	7	63	leukocyte proliferation	BP	0.909	MSN,CTNNB1,TFRC,LYN,SDC4,BTK,CDKN1A	down_VSAP_EC
GO:0006109	0.079	0.971	6	51	regulation of carbohydrate metabolic	BP	0.909	ENPP1,PGAM1,SESN2,HIF1A,SLC45A3,PTPN2	down_VSAP_EC
GO:0021879	0.079	0.997	1	15	forebrain neuron differentiation	BP	1	NRP2	down_ACOR_EC
GO:0030974	0.079	1	1	1	thiamine pyrophosphate transmembrane transport	BP	1	SLC25A19	down_ACOR_VSMC
GO:0048732	0.079	0.997	1	116	gland development	BP	1	HOXA3	up_APUL_VSMC
GO:0021772	0.079	0.997	1	11	olfactory bulb development	BP	1	SEMA3A	up_VPOR_EC
GO:0021988	0.079	0.997	1	11	olfactory lobe development	BP	1	SEMA3A	up_VPOR_EC
GO:0070527	0.079	0.997	1	10	platelet aggregation	BP	1	SLC7A11	up_VPOR_EC
GO:0002408	0.079	1	1	1	myeloid dendritic cell chemotaxis	BP	1	ARHGEF5	up_VSAP_EC
GO:1904468	0.079086013	0.99785	1	3	negative regulation of tumor necrosis factor	BP	1	NR1H4	up_ACOR_EC
GO:0006678	0.079086168	0.99785	1	3	glucosylceramide metabolic process	BP	1	PRKCD	up_ACOR_EC
GO:0032496	0.0791	0.962	10	77	response to lipopolysaccharide	BP	0.913	CXCL8,TNFAIP3,TNIP1,PDE4D,MEF2C,IRF8,PELI1,ABCA1,PLCG2,GCH1	up_VCAV_EC
GO:0043414	0.0791	0.962	10	70	macromolecule methylation	BP	0.913	IWS1,GATA3,ARID4B,NSD1,WTAP,MTA2,KDM3A,ATRX,ZC3H13,TET2	up_VCAV_EC
GO:0006301	0.0791	0.984	3	12	postreplication repair	BP	1	PCNA,POLH,DTL	down_ACOR_VSMC
GO:0000075	0.0791	0.989	2	91	cell cycle checkpoint	BP	1	USP28,ATRIP	down_ACOR_EC
GO:0045923	0.0791	0.99	2	8	positive regulation of fatty acid metabolic process	BP	0.909	PTGS2,SLC45A3	down_VSAP_EC
GO:0046461	0.0791	0.992	2	5	neutral lipid catabolic process	BP	1	LIPC,ABHD16A	up_VSAP_EC
GO:0046464	0.0791	0.992	2	5	acylglycerol catabolic process	BP	1	LIPC,ABHD16A	up_VSAP_EC
GO:0050727	0.0791	0.997	1	74	regulation of inflammatory response	BP	1	ESR1	up_AORT_VSMC
GO:0051051	0.0791	0.997	1	112	negative regulation of transport	BP	1	HOXA3	up_APUL_VSMC
GO:0035457	0.079182062	0.99784	1	3	cellular response to interferon-alpha	BP	1	IFIT2	up_ACOR_EC
GO:0007276	0.0792	0.955	16	130	gamete generation	BP	1	NDC1,TRIP13,CTDNEP1,FBXO5,NCAPH,POC1A,HIST1H1A,FOSL1,HMGB2,SGPL1,TTK,ASPM,HOXA9,GLI1,H2AFX,TTLL1	down_ACOR_VSMC
GO:0051403	0.0792	0.959	12	89	stress-activated MAPK cascade	BP	0.913	EPHB1,MAP3K11,SH2D3C,TLR4,EDN1,MAP3K3,BMP2,MAPK13,TLR3,TAOK1,HMGB1,RIPK1	up_VCAV_EC
GO:0071559	0.0792	0.968	7	75	response to transforming growth factor beta	BP	1	SMAD9,VASN,FBN1,NR3C1,ASPN,ZFP36L1,FUT8	down_VCAV_EC
GO:0003008	0.0792	0.99	2	336	system process	BP	1	HOXC10,IRX5	up_AFEM_VSMC
GO:0009790	0.0792	0.99	2	335	embryo development	BP	1	HOXC6,C6	up_VCAV_VSMC
GO:0035909	0.0792	0.997	1	14	aorta morphogenesis	BP	1	ACVRL1	down_ACOR_EC
GO:0010812	0.0792	0.997	1	19	negative regulation of cell-substrate adhesion	BP	1	HOXA7	up_VFEM_EC
GO:0023051	0.0793	0.976	5	954	regulation of signaling	BP	1	BARX1,NKX2-5,BAMBI,ANKRD6,CYP26B1	up_VPOR_VSMC
GO:0060441	0.0793	0.984	3	11	epithelial tube branching involved in lung	BP	1	BMP4,FGFR2,DAG1	up_ACOR_VSMC
GO:0031629	0.0793	0.99	2	8	synaptic vesicle fusion to presynaptic active zone membrane	BP	0.913	SNAP23,STX11	up_VCAV_EC

GO:0099500	0.0793	0.99	2	8	vesicle fusion to plasma membrane	BP	0.913	SNAP23,STX11	up_VCAV_EC
GO:0006071	0.0793	0.992	2	5	glycerol metabolic process	BP	1	GOT1,TKFC	up_VSAP_EC
GO:2001256	0.0793	0.992	2	5	regulation of store-operated calcium entry	BP	1	SLC8B1,CRACR2B	up_VSAP_EC
GO:0006531	0.0793	0.992	2	5	aspartate metabolic process	BP	1	GOT1,ASS1	up_VSAP_EC
GO:0034766	0.0793	0.997	1	19	negative regulation of ion transmembrane transport	BP	1	NEDD4	down_ACOR_EC
GO:0016358	0.0793	0.997	1	58	dendrite development	BP	1	SLITRK5	up_VCAV_VSMC
GO:0016255	0.079342525	0.99784	1	3	attachment of GPI anchor to protein	BP	1	PIGS	up_ACOR_EC
GO:0008610	0.0794	0.952	19	177	lipid biosynthetic process	BP	0.913	ADGRF5,PDGFB,EDN1,PTGS1,SPTLC2,MTOR,SAMD8,BMP2,PROX1,ENSCAFG00000010596,SH3GLB1,ELOVL7,PTGS2,PLCG2,CREB1,LBR,IMPA1,PIP5K1B,ALDH1A3	up_VCAV_EC
GO:0050794	0.0794	0.964	16	3075	regulation of cellular process	BP	1	NR1H4,TBX5,MAPK10,BPI,HOXA3,PLCL1,TUBB4A,PCSK1N,ARHGEF26,LZTS1,SLITRK5,WIF1,NPNT,NTRK2,HOXA1,PDE1A	up_APUL_EC
GO:0002683	0.0794	0.97	6	126	negative regulation of immune system process	BP	1	HOXA9,HOXA7,DLL1,BPI,GPAM,CYLD	up_AFEM_EC
GO:0050886	0.0794	0.983	3	17	endocrine process	BP	0.911	PTPN11,RAB11FIP1,INHBA	down_VSAP_EC
GO:0010468	0.0794	0.988	3	1270	regulation of gene expression	BP	1	GATA5,TGFBR1,MEIS2	down_VSAP_VSMC
GO:0002437	0.0794	0.991	2	6	inflammatory response to antigenic stimulus	BP	1	HMGB1,HMGB2	down_ACOR_VSMC
GO:0006002	0.0794	0.992	2	5	fructose 6-phosphate metabolic process	BP	1	PFKM,PFKL	up_ACOR_VSMC
GO:0007389	0.0794	0.989	2	137	pattern specification process	BP	1	TBX5,HOXA3	up_VPUL_EC
GO:0006357	0.0794	1	1	547	regulation of transcription by RNA polymerase II	BP	1	GATA5	down_AFEM_VSMC
GO:0051455	0.0794	1	1	1	attachment of spindle microtubules to kinetochore involved in homologous chromosome	BP	0.914	CENPC	up_VCAV_EC
GO:0072210	0.0794	0.997	1	19	metanephric nephron development	BP	1	SALL1	up_VSAP_VSMC
GO:0045596	0.0795	0.956	14	186	negative regulation of cell differentiation	BP	1	VASN,FBN1,BMPR1A,THY1,TLX2,GLI3,ZFP36L1,LOXL3,TWIST2,PRKCSH,GREM1,THRB,ZBTB16,RGMA	down_VCAV_EC
GO:0002526	0.0795	0.983	3	17	acute inflammatory response	BP	0.912	PRCP,PTGS2,A2M	down_VSAP_EC
GO:0097205	0.0795	0.984	3	12	renal filtration	BP	0.914	ADGRF5,PDGFB,CD34	up_VCAV_EC
GO:0002643	0.0795	0.992	2	5	regulation of tolerance induction	BP	1	CD274,TGFBR2	up_VSAP_EC
GO:0061004	0.0795	0.992	2	5	pattern specification involved in kidney	BP	1	OSR1,IRX3	up_VSAP_EC
GO:0072048	0.0795	0.992	2	5	renal system pattern specification	BP	1	OSR1,IRX3	up_VSAP_EC
GO:0043583	0.0795	0.997	1	59	ear development	BP	1	EYA1	up_AFEM_VSMC
GO:2001236	0.0795	0.997	1	59	regulation of extrinsic apoptotic signaling	BP	1	EYA1	up_AFEM_VSMC
GO:0051145	0.0795	0.997	1	21	smooth muscle cell differentiation	BP	1	NPNT	up_APUL_EC
GO:0003415	0.07951581	0.99783	1	3	chondrocyte hypertrophy	BP	1	SOX9	up_ACOR_EC
GO:0072513	0.079519756	0.99783	1	3	positive regulation of secondary heart field cardioblast proliferation	BP	1	TBX5	up_ACOR_EC
GO:0003214	0.079564451	0.99782	1	3	cardiac left ventricle morphogenesis	BP	1	TBX5	up_ACOR_EC
GO:0060737	0.079578835	0.99782	1	3	prostate gland morphogenetic growth	BP	1	SOX9	up_ACOR_EC
GO:0001932	0.0796	0.942	49	416	regulation of protein phosphorylation	BP	1	ADRA1B,SMAD6,LEP,PLCL1,MAP3K8,MID1,HTR2B,RASSF2,BMP4,FGFR2,TLR3,SH3RF2,LATS2,PDE5A,SASH1,FGF18,NDRG2,CDKN2B,F2RL1,FAM129A,CDKN1A,NRP1,PTPN22,NPNT,SMPD1,SH3RF1,PRKCE,CCNT1,FRS2,DAG1,PARP9,APP,PSEN1,SYNJ2BP,CCL5,DDR2,DUSP22,EDN1,AKTIP,DUSP26,NOD2,LRRC4,PRKAG3,SH3RF3,MAP3K7,INPP5K,ENSCAFG00000029553,PARK7,CRKL	up_ACOR_VSMC

GO:0016310	0.0796	0.944	40	641	phosphorylation	BP	1	NOTCH2,PDGFRB,KIRREL1,SMAD9,DCN,PRNP,GLIPR2,BGN,BMPR1A,LMO4,CLIP3,ENSCAFG00000014978,AKT1S1,AKT2,EEF2K,PLCL1,CSPG4,UQCR10,ASPN,THY1,ADK,PARK7,MAST2,ZFP36L1,MYDGF,SASH1,ARL2BP,MAVS,CDH2,GSDME,CAMK2D,SPRED1,MAPRE3,CDKL2,DDR2,SHC1,FBH1,GREM1,BRSK1,CDK14	down_VCAV_EC
GO:0044092	0.0796	0.946	33	270	negative regulation of molecular function	BP	1	TNNT2,ARHGAP28,ACTN2,KCNE3,RECK,BMP4,EIF4A2,SH3RF2,XIRP1,LATS2,CST3,EGLN1,CDKN1A,CLSTN1,LRPAP1,SMPD1,SH3RF1,MARF1,PPP1R26,PSEN1,SPINK2,MITD1,DUSP22,DUSP26,LRRC4,B2M,DISC1,INPP5K,WFS1,SH3BP4,PARK7,GPATCH2,ZFYVE1	up_ACOR_VSMC
GO:2000145	0.0796	0.998	1	279	regulation of cell motility	BP	1	JUP	down_VFEM_VSMC
GO:0003151	0.0796	0.997	1	34	outflow tract morphogenesis	BP	1	EYA1	up_VFEM_VSMC
GO:0044387	0.079624361	0.99782	1	3	negative regulation of protein kinase activity by regulation of protein	BP	1	DUSP26	up_ACOR_EC
GO:1903867	0.079665132	0.99782	1	3	extraembryonic membrane development	BP	1	FZD5	up_ACOR_EC
GO:0019439	0.0797	0.953	18	150	aromatic compound catabolic process	BP	1	RNPS1,EIF4A3,EDC3,HNRNPD,THRAP3,EDC4,FEN1,RNASEH2B,SMG9,DIS3,FASTKD3,PDXP,AIFM1,ENSCAFG00000015189,RNASEH2C,NUDT1,EXOSC5,NUDT3	down_ACOR_VSMC
GO:0045926	0.0797	0.961	11	72	negative regulation of growth	BP	1	ENSCAFG00000009113,WNT11,HNF4A,SLIT2,SLC6A4,SAV1,MT2A,FXN,MYD88,SMARCA2,ACVRL1	up_VSAP_EC
GO:0043547	0.0797	0.989	2	61	positive regulation of GTPase activity	BP	1	ENSCAFG00000002015,ENSCAFG00000032731	up_VPOR_EC
GO:0050768	0.0797	0.997	1	66	negative regulation of neurogenesis	BP	1	WNT7A	up_AORT_VSMC
GO:0032623	0.0797	0.997	1	21	interleukin-2 production	BP	1	NR1H4	up_APUL_EC
GO:0071230	0.0797	0.997	1	23	cellular response to amino acid stimulus	BP	1	NTRK2	up_APUL_EC
GO:1901224	0.0797	0.997	1	20	positive regulation of NIK/NF-kappaB signaling	BP	1	TLR2	up_VSAP_VSMC
GO:0007200	0.079703476	0.98913	2	18	phospholipase C-activating G protein-coupled receptor signaling	BP	1	PTH1R,ACTN2	up_ACOR_EC
GO:0006930	0.079728098	0.99781	1	3	substrate-dependent cell migration, cell extension	BP	1	NRP1	up_ACOR_EC
GO:0060078	0.079777864	0.98917	2	17	regulation of postsynaptic membrane potential	BP	1	INSYN1,MAPK8IP2	up_ACOR_EC
GO:0099565	0.079777864	0.98917	2	17	chemical synaptic transmission, postsynaptic	BP	1	INSYN1,MAPK8IP2	up_ACOR_EC
GO:0043068	0.0798	0.954	16	190	positive regulation of programmed cell death	BP	0.914	CASP3,PTPN2,MAGED1,CTNNB1,LYN,BNIP3,DDIT3,FAM162A,ATF3,SH3GLB1,HTT,REST,PAK2,INHBA,CDKN1A,SPINK2	down_VSAP_EC
GO:0001944	0.0798	0.982	3	263	vasculature development	BP	1	HOXA3,NTRK2,HOXA1	up_APUL_EC
GO:0046483	0.0798	1	2	1948	heterocycle metabolic process	BP	1	JUP,GATA5	down_VFEM_VSMC
GO:0002089	0.0798	0.99	2	8	lens morphogenesis in camera-type eye	BP	0.914	BCAR3,CTNNB1	down_VSAP_EC
GO:0060047	0.0798	0.997	1	57	heart contraction	BP	1	IRX5	up_AFEM_VSMC
GO:0071493	0.079819296	0.99781	1	3	cellular response to UV-B	BP	1	MFAP4	up_ACOR_EC
GO:0023021	0.079829232	0.99781	1	3	termination of signal transduction	BP	1	PRKCD	up_ACOR_EC
GO:0009650	0.079877785	0.99781	1	3	UV protection	BP	1	MFAP4	up_ACOR_EC
GO:0098609	0.0799	0.949	24	181	cell-cell adhesion	BP	1	LEP,GATA5,CD83,MBP,BMP4,PDE5A,CITED2,PKP4,CD274,LIMS2,PTPN22,NPNT,SPINT2,VCAM1,PSEN1,JUP,CDH18,PERP,DUSP22,JAM3,CELSR2,ATP2C1,BVES,PAG1	up_ACOR_VSMC
GO:0035295	0.0799	0.957	13	356	tube development	BP	1	IRX3,LEP,ANGPT4,HOXA7,DLL1,F3,THRB,TMEM100,GREM1,LUZP1,TBXA2R,PTPRB,CLIC4	up_AFEM_EC
GO:0006139	0.0799	0.971	8	1909	nucleobase-containing compound metabolic	BP	1	EYA1,HOXA10,HOXA9,TBX15,FBP1,EMX2,HOXA11,SHOX2	up_VFEM_VSMC
GO:0051094	0.0799	0.978	4	388	positive regulation of developmental process	BP	1	TOX,HOXB7,SIX1,CYP26B1	up_VFEM_EC
GO:0030947	0.0799	0.984	3	12	regulation of vascular endothelial growth factor receptor signaling	BP	0.917	HHEX,PRKD2,HIF1A	up_VCAV_EC

GO:0009719	0.0799	0.99	2	395	response to endogenous stimulus	BP	1	WNT7A,ESR1	up_AORT_VSMC
GO:0036446	0.0799	1	1	1	myofibroblast differentiation	BP	1	TP53INP1	up_ACOR_VSMC
GO:0072702	0.0799	1	1	1	response to methyl methanesulfonate	BP	1	TP53INP1	up_ACOR_VSMC
GO:0072703	0.0799	1	1	1	cellular response to methyl methanesulfonate	BP	1	TP53INP1	up_ACOR_VSMC
GO:1904760	0.0799	1	1	1	regulation of myofibroblast	BP	1	TP53INP1	up_ACOR_VSMC
GO:1904761	0.0799	1	1	1	negative regulation of myofibroblast	BP	1	TP53INP1	up_ACOR_VSMC
GO:1905937	0.0799	1	1	1	negative regulation of germ cell proliferation	BP	1	PRDX4	up_ACOR_VSMC
GO:2000255	0.0799	1	1	1	negative regulation of male germ cell	BP	1	PRDX4	up_ACOR_VSMC
GO:0072073	0.0799	0.997	1	60	kidney epithelium development	BP	1	EYA1	up_AFEM_VSMC
GO:1901861	0.0799	0.997	1	41	regulation of muscle tissue development	BP	1	TBX5	up_VPUL_VSMC
GO:0009166	0.08	0.997	1	31	nucleotide catabolic process	BP	1	FBP1	up_VFEM_VSMC
GO:0031110	0.08	0.997	1	24	regulation of microtubule polymerization or depolymerization	BP	1	ARHGEF2	up_VPUL_EC
GO:0032755	0.08	0.997	1	24	positive regulation of interleukin-6 production	BP	1	ARHGEF2	up_VPUL_EC
GO:0035051	0.08	0.997	1	41	cardiocyte differentiation	BP	1	TBX5	up_VPUL_VSMC
GO:0034111	0.080092378	0.99779	1	3	negative regulation of homotypic cell-cell	BP	1	PRKCD	up_ACOR_EC
GO:1901700	0.0801	0.944	39	364	response to oxygen-containing compound	BP	0.917	CXCL8,CALCRL,TIE1,TNFAIP3,PPARG,TNIP1,PDGFB,P2RX7,BAIAP2L1,SELL,PDE4D,MEF2C,RAPGEF1,TLR4,IRF8,PELI1,YES1,MTOR,ABCA1,MAPK13,SMARCA4,APOD,NCK1,TIMELESS,JAG1,GUCY1B1,RAPGEF2,PLCG2,CREB1,CTNNA1,GCH1,BLM,RIPK1,PDGFD,HIF1A,NR4A1,P2RX2,ZEB1,ARHGEF2	up_VCAV_EC
GO:0006796	0.0801	0.967	8	938	phosphate-containing compound metabolic	BP	1	ACVRL1,LYN,IGF1R,PRKAR2A,ACSL3,MYO1D,TNRC6B,ASPN	down_ACOR_EC
GO:0032092	0.0801	0.971	6	38	positive regulation of protein binding	BP	0.917	TMEM173,MEF2C,RALB,BMP2,MFNG,RAPGEF2	up_VCAV_EC
GO:0032411	0.0801	0.975	5	24	positive regulation of transporter activity	BP	1	ACTN2,PLCG2,TCAF1,AKAP6,PARK7	up_ACOR_VSMC
GO:0042149	0.0801	0.979	4	17	cellular response to glucose starvation	BP	1	PRKAA2,SESN3,PIK3C3,SESN1	up_ACOR_VSMC
GO:0061614	0.0801	0.978	4	20	pri-miRNA transcription by RNA polymerase II	BP	0.917	PDGFB,BMP2,SMARCA4,HIF1A	up_VCAV_EC
GO:0003094	0.0801	0.984	3	11	glomerular filtration	BP	1	EMP2,SULF2,JCHAIN	up_VSAP_EC
GO:0051241	0.0801	0.99	2	336	negative regulation of multicellular organismal	BP	1	GATA5,LRRC17	up_VCAV_VSMC
GO:0032945	0.0801	0.997	1	17	negative regulation of mononuclear cell	BP	1	LYN	down_ACOR_EC
GO:0050672	0.0801	0.997	1	17	negative regulation of lymphocyte proliferation	BP	1	LYN	down_ACOR_EC
GO:0032680	0.0801	0.997	1	36	regulation of tumor necrosis factor production	BP	1	BPI	up_AORT_EC
GO:0045600	0.0801	0.997	1	21	positive regulation of fat cell differentiation	BP	1	WIF1	up_APUL_EC
GO:0060537	0.0801	0.997	1	117	muscle tissue development	BP	1	TBX5	up_APUL_VSMC
GO:0046889	0.0801	0.997	1	24	positive regulation of lipid biosynthetic process	BP	1	NR1H4	up_VPUL_EC
GO:1903115	0.0802	0.984	3	12	regulation of actin filament-based movement	BP	1	FGF13,KCNE3,JUP	up_ACOR_VSMC
GO:0018958	0.0802	0.989	2	20	phenol-containing compound metabolic	BP	1	MOXD1,GCNT4	up_AFEM_EC
GO:0001654	0.0802	0.989	2	110	eye development	BP	1	IRX5,MAB21L1	up_VSAP_VSMC
GO:0048880	0.0802	0.989	2	110	sensory system development	BP	1	IRX5,MAB21L1	up_VSAP_VSMC
GO:0150063	0.0802	0.989	2	110	visual system development	BP	1	IRX5,MAB21L1	up_VSAP_VSMC
GO:0050795	0.0802	0.997	1	15	regulation of behavior	BP	1	HOXA1	down_ACOR_EC
GO:0033209	0.0802	0.997	1	24	tumor necrosis factor-mediated signaling	BP	1	NR1H4	up_VPUL_EC
GO:0042637	0.080220067	0.99779	1	3	catagen	BP	1	CDH3	up_ACOR_EC

GO:0051794	0.080220067	0.99779	1	3	regulation of timing of catagen	BP	1	CDH3	up_ACOR_EC
GO:0061013	0.0803	0.971	6	32	regulation of mRNA catabolic process	BP	1	AXIN2,TENT5C,ZFP36L1,SAMD4B,ZFP36L2,TNRC6C	up_VSAP_EC
GO:0070979	0.0803	0.984	3	13	protein K11-linked ubiquitination	BP	1	UBE2S,UBE2T,UBE2C	down_ACOR_VSMC
GO:0060676	0.0803	0.991	2	6	ureteric bud formation	BP	1	GREM1,SIX4	down_ACOR_VSMC
GO:1901137	0.0803	0.989	2	232	carbohydrate derivative biosynthetic process	BP	1	MGAT4B,GBGT1	down_VPOR_EC
GO:0001708	0.0803	0.989	2	20	cell fate specification	BP	1	HOXC10,DLL1	up_AFEM_EC
GO:0009205	0.0803	0.997	1	98	purine ribonucleoside triphosphate metabolic	BP	1	AK5	down_VSAP_VSMC
GO:0060419	0.0803	0.997	1	22	heart growth	BP	1	TBX5	up_APUL_EC
GO:0044344	0.0803	0.997	1	33	cellular response to fibroblast growth factor	BP	1	FGF5	up_VPOR_VSMC
GO:0071774	0.0803	0.997	1	33	response to fibroblast growth factor	BP	1	FGF5	up_VPOR_VSMC
GO:0046620	0.0803	0.997	1	30	regulation of organ growth	BP	1	NKX2-5	up_VPOR_VSMC
GO:0014902	0.0803	0.997	1	34	myotube differentiation	BP	1	SHOX2	up_VFEM_VSMC
GO:0006596	0.080316726	0.99778	1	3	polyamine biosynthetic process	BP	1	AZIN2	up_ACOR_EC
GO:0009446	0.080316726	0.99778	1	3	putrescine biosynthetic process	BP	1	AZIN2	up_ACOR_EC
GO:0033387	0.080316726	0.99778	1	3	putrescine biosynthetic process from ornithine	BP	1	AZIN2	up_ACOR_EC
GO:1902713	0.080355927	0.99778	1	3	regulation of interferon-gamma secretion	BP	1	NR1H4	up_ACOR_EC
GO:0006725	0.0804	1	2	1956	cellular aromatic compound metabolic	BP	1	JUP,GATA5	down_VFEM_VSMC
GO:0002639	0.0804	0.997	1	14	positive regulation of immunoglobulin	BP	1	STX4	down_ACOR_EC
GO:0042471	0.0804	0.997	1	34	ear morphogenesis	BP	1	EYA1	up_VFEM_VSMC
GO:0140059	0.080445053	0.99777	1	3	dendrite arborization	BP	1	NRP1	up_ACOR_EC
GO:0008283	0.0805	0.974	5	545	cell proliferation	BP	1	EYA1,SIX1,MAB21L1,TIAM1,MTSS1	up_VFEM_EC
GO:0046426	0.0805	0.983	3	20	negative regulation of JAK-STAT cascade	BP	1	DCN,BGN,ASPN	down_VCAV_EC
GO:1904893	0.0805	0.983	3	20	negative regulation of STAT cascade	BP	1	DCN,BGN,ASPN	down_VCAV_EC
GO:0045844	0.0805	0.997	1	24	positive regulation of striated muscle tissue	BP	1	TBX5	up_VPUL_EC
GO:0048636	0.0805	0.997	1	24	positive regulation of muscle organ	BP	1	TBX5	up_VPUL_EC
GO:0051017	0.0806	0.97	6	49	actin filament bundle assembly	BP	0.922	ACTN1,FSCN1,DPYSL3,PLS3,NRP1,TNFAIP1	down_VSAP_EC
GO:0007095	0.0806	0.984	3	11	mitotic G2 DNA damage checkpoint	BP	0.917	TAOK1,BLM,TOPBP1	up_VCAV_EC
GO:0008284	0.0806	0.989	2	238	positive regulation of cell proliferation	BP	1	HOXA3,TBX5	up_VPUL_VSMC
GO:0002687	0.0806	0.997	1	36	positive regulation of leukocyte migration	BP	1	C3AR1	up_VPOR_VSMC
GO:0007612	0.0806	0.997	1	33	learning	BP	1	NETO1	up_VFEM_VSMC
GO:0060385	0.080644783	0.99776	1	3	axonogenesis involved in innervation	BP	1	NRP1	up_ACOR_EC
GO:0072497	0.080681767	0.99776	1	3	mesenchymal stem cell differentiation	BP	1	SOX9	up_ACOR_EC
GO:0051668	0.080689854	0.98186	3	39	localization within membrane	BP	1	CALY,AGRN,LRP4	up_ACOR_EC
GO:0030203	0.0807	0.977	4	27	glycosaminoglycan metabolic process	BP	0.923	ENSCAFG00000032470,CEMIP2,CHSY1,EXT1	down_VSAP_EC
GO:0032535	0.0807	0.982	3	128	regulation of cellular component size	BP	1	SEMA3A,TWF1,RNF6	up_VPOR_EC
GO:0006622	0.0807	0.997	1	11	protein targeting to lysosome	BP	1	GCC2	up_VPOR_EC
GO:0021869	0.080700661	0.99776	1	3	forebrain ventricular zone progenitor cell division	BP	1	DIXDC1	up_ACOR_EC
GO:0034350	0.080734633	0.99776	1	3	regulation of glial cell apoptotic process	BP	1	PRKCD	up_ACOR_EC
GO:0034351	0.080734633	0.99776	1	3	negative regulation of glial cell apoptotic process	BP	1	PRKCD	up_ACOR_EC
GO:0006685	0.080759903	0.99776	1	3	sphingomyelin catabolic process	BP	1	PRKCD	up_ACOR_EC

GO:0019221	0.0808	0.961	10	102	cytokine-mediated signaling pathway	BP	0.923	NR1H4,SOCS5,ILK,CX3CL1,PTPN2,ADAM17,CNOT7,ENSCAFG00000002015,ZC3H15,CDC37	down_VSAP_EC
GO:0033500	0.0808	0.961	10	64	carbohydrate homeostasis	BP	1	ADRA1B,LEP,PRKAA2,ERO1B,SESN3,PRKCE,RAB11B,INPP5K,WFS1,PARK7	up_ACOR_VSMC
GO:0042593	0.0808	0.961	10	64	glucose homeostasis	BP	1	ADRA1B,LEP,PRKAA2,ERO1B,SESN3,PRKCE,RAB11B,INPP5K,WFS1,PARK7	up_ACOR_VSMC
GO:0051494	0.0808	0.968	7	44	negative regulation of cytoskeleton organization	BP	0.917	ARHGAP28,TMOD1,STMN1,SWAP70,TAOK1,TMOD3,ARHGEF2	up_VCAV_EC
GO:0042325	0.0808	0.973	5	463	regulation of phosphorylation	BP	1	ACVRL1,LYN,IGF1R,PRKAR2A,ASPN	down_ACOR_EC
GO:0051897	0.0808	0.989	2	20	positive regulation of protein kinase B signaling	BP	1	LEP,F3	up_AFEM_EC
GO:1901184	0.0808	0.989	2	20	regulation of ERBB signaling pathway	BP	1	RAB7A,ADAM17	up_AFEM_EC
GO:0034097	0.0808	0.989	2	238	response to cytokine	BP	1	IL22RA1,SLIT2	up_VPUL_VSMC
GO:0098927	0.0808	0.997	1	17	vesicle-mediated transport between endosomal compartments	BP	1	MYO1D	down_ACOR_EC
GO:1903522	0.0808	0.997	1	58	regulation of blood circulation	BP	1	IRX5	up_AFEM_VSMC
GO:0031424	0.080839774	0.99775	1	3	keratinization	BP	1	CDH3	up_ACOR_EC
GO:0060080	0.080856613	0.99775	1	3	inhibitory postsynaptic potential	BP	1	INSYN1	up_ACOR_EC
GO:0046628	0.080863426	0.99775	1	3	positive regulation of insulin receptor signaling	BP	1	NR1H4	up_ACOR_EC
GO:0046890	0.0809	0.97	6	51	regulation of lipid biosynthetic process	BP	0.923	NR1H4,TRIB3,PTGS2,SLC45A3,APOE,REST	down_VSAP_EC
GO:0021885	0.0809	0.974	5	24	forebrain cell migration	BP	1	FGF13,NRP1,PSEN1,DISC1,SUN1	up_ACOR_VSMC
GO:0042149	0.0809	0.983	3	17	cellular response to glucose starvation	BP	0.923	ASNS,HSPA5,SESN2	down_VSAP_EC
GO:0051270	0.0809	0.982	3	296	regulation of cellular component movement	BP	1	TBX5,CCL24,ARHGEF2	up_VPUL_EC
GO:0048645	0.0809	0.997	1	33	animal organ formation	BP	1	HOXA3	up_VPOR_VSMC
GO:0048843	0.080954484	0.99775	1	3	negative regulation of axon extension involved in axon guidance	BP	1	NRP1	up_ACOR_EC
GO:0032502	0.081	0.936	94	1661	developmental process	BP	1	ADAMTS2,GPAM,NOTCH2,HSPB6,PDGFRB,SMAD9,LRP1,DCN,NAB2,NDN,ANGPT1,PRNP,SDC4,VASN,FBN1,GLIPR2,KANK2,THBS3,BMPR1A,LOX,LMO4,EVC,RECK,TRPV2,ANPEP,COL6A1,CLIP3,NUMBL,NAGLU,CHRDL1,TBX15,AKT2,TFE3,ENSCAFG00000018277,SYDE1,HOXC4,LAMA3,CLSTN1,CSPG4,TRIM8,PKD1,PRMT6,ENSCAFG00000013404,PRELP,SEMA3C,ASPN,POLDIP2,CRISPLD2,THY1,TEAD1,HAPLN3,PEX5,TLX2,VCAN,B4GALT1,GLI3,ADAMTSL4,EEF2,ZFP36L1,MYDGF,SASH1,NCDN,RBM38,IFT57,CDH2,LOXL3,GSDME,CAMK2D,ENSCAFG00000017708,SPRED1,RAB7B,A2M,IRX5,TSC22D3,UST,GLIS2,TWIST2,PRKCSH,DDR2,SHC1,RDH10,PCSK6,OMD,GREM1,NAPEPLD,CLIC4,FOXO3,SOAT1,THRB,ZBTB16,ANKRD54,RGMA,SEC61A1,BRSK1	down_VCAV_EC
GO:0042592	0.081	0.94	52	522	homeostatic process	BP	0.917	EDNRB,HCLS1,IL7R,ADGRF5,KIT,PRCP,CP,TNFAIP3,LRRC8D,ATP2B1,CD34,PDE4D,MEF2C,CHD7,TLR4,GPR4,STC1,GATA3,EDN1,APLNR,SLC39A8,LRRC8C,SLC12A7,PDZD8,ABCA1,ITPKB,STMN1,HMBOX1,SWAP70,SMARCA4,EXOC5,LDB2,PDIA5,PTGS2,PLCG2,BCL10,TAOK1,SMG7,VPS54,ADIPOR2,HMGB1,BLM,SMC5,SKIL,MCU,NR1D2,HIF1A,KDM3A,ATRX,DCP2,ATP2A1,TMOD3	up_VCAV_EC
GO:0060341	0.081	0.96	10	255	regulation of cellular localization	BP	1	LEP,KCNC4,GAS1,CRYAB,CHRM2,CAMK2A,CYLD,LRRC15,REEP2,BVES	up_AFEM_EC
GO:0009059	0.081	0.971	7	1563	macromolecule biosynthetic process	BP	1	EYA1,HOXA10,HOXA9,TBX15,EMX2,HOXA11,SHOX2	up_VFEM_VSMC
GO:0060255	0.081	0.979	5	1782	regulation of macromolecule metabolic	BP	1	TLR2,EYA1,HOXA9,HOXA10,IRX5	up_AFEM_VSMC
GO:0060291	0.081	0.984	3	11	long-term synaptic potentiation	BP	1	FAM107A,ABL1,STX4	up_VSAP_EC
GO:0002027	0.081	0.989	2	20	regulation of heart rate	BP	1	IRX5,BVES	up_AFEM_EC
GO:0044087	0.081	0.99	2	339	regulation of cellular component biogenesis	BP	1	SLITRK5,LIMK1	up_VCAV_VSMC

GO:0007023	0.081	1	1	1	post-chaperonin tubulin folding pathway	BP	1	TBCC	down_ACOR_VSMC
GO:2000026	0.081	1	1	560	regulation of multicellular organismal development	BP	1	GATA5	down_AFEM_VSMC
GO:0070966	0.081	1	1	1	nuclear-transcribed mRNA catabolic process, no-go	BP	0.917	CNOT6	up_VCAV_EC
GO:0048021	0.081003363	0.99774	1	3	regulation of melanin biosynthetic process	BP	1	CDH3	up_ACOR_EC
GO:1900376	0.081003363	0.99774	1	3	regulation of secondary metabolite biosynthetic	BP	1	CDH3	up_ACOR_EC
GO:0006667	0.0811	1	1	1	sphinganine metabolic process	BP	0.917	SPTLC2	up_VCAV_EC
GO:0046511	0.0811	1	1	1	sphinganine biosynthetic process	BP	0.917	SPTLC2	up_VCAV_EC
GO:0003018	0.0811	0.997	1	42	vascular process in circulatory system	BP	1	SLIT2	up_VPUL_VSMC
GO:0061438	0.081110476	0.99774	1	3	renal system vasculature morphogenesis	BP	1	NRP1	up_ACOR_EC
GO:0061439	0.081110476	0.99774	1	3	kidney vasculature morphogenesis	BP	1	NRP1	up_ACOR_EC
GO:1901381	0.081145653	0.99773	1	3	positive regulation of potassium ion	BP	1	ACTN2	up_ACOR_EC
GO:1901361	0.0812	0.955	14	154	organic cyclic compound catabolic process	BP	0.926	ENPP1,PGK1,TPI1,PGAM1,ALDH1L2,HIF1A,ZPR1,ENTPD4,SMG7,CNOT7,ALDOC,SND1,APOE,YWHAH	down_VSAP_EC
GO:0002703	0.0812	0.965	8	47	regulation of leukocyte mediated immunity	BP	1	LEP,TP53BP1,TLR3,F2RL1,DUSP22,B2M,MAP3K7,IL18R1	up_ACOR_VSMC
GO:0042559	0.0812	0.991	2	6	pteridine-containing compound biosynthetic	BP	1	PCBD1,PTS	up_ACOR_VSMC
GO:0009308	0.0812	0.989	2	20	amine metabolic process	BP	1	MOXD1,NAPEPLD	up_AFEM_EC
GO:0003015	0.0812	0.997	1	58	heart process	BP	1	IRX5	up_AFEM_VSMC
GO:0035701	0.0812	1	1	1	hematopoietic stem cell migration	BP	0.917	KIT	up_VCAV_EC
GO:0036017	0.0812	1	1	1	response to erythropoietin	BP	0.917	KIT	up_VCAV_EC
GO:0036018	0.0812	1	1	1	cellular response to erythropoietin	BP	0.917	KIT	up_VCAV_EC
GO:0036215	0.0812	1	1	1	response to stem cell factor	BP	0.917	KIT	up_VCAV_EC
GO:0036216	0.0812	1	1	1	cellular response to stem cell factor stimulus	BP	0.917	KIT	up_VCAV_EC
GO:0038162	0.0812	1	1	1	erythropoietin-mediated signaling pathway	BP	0.917	KIT	up_VCAV_EC
GO:0097324	0.0812	1	1	1	melanocyte migration	BP	0.917	KIT	up_VCAV_EC
GO:0097326	0.0812	1	1	1	melanocyte adhesion	BP	0.917	KIT	up_VCAV_EC
GO:0032762	0.0812	1	1	1	mast cell cytokine production	BP	0.917	KIT	up_VCAV_EC
GO:0038109	0.0812	1	1	1	Kit signaling pathway	BP	0.917	KIT	up_VCAV_EC
GO:0034616	0.081212043	0.99773	1	3	response to laminar fluid shear stress	BP	1	SMAD6	up_ACOR_EC
GO:1902170	0.081291036	0.99773	1	3	cellular response to reactive nitrogen species	BP	1	GUCY1B1	up_ACOR_EC
GO:2000147	0.0813	0.949	22	169	positive regulation of cell motility	BP	1	TWIST2,WNT11,FAM107A,ENPP2,FGF7,ABL1,HDAC7,PDGFD,CCBE1,STAT5A,F2RL1,DDR2,PIK3R1,TIAM1,CFAP69,SASH1,ZNF580,STX4,HMOX1,MAP2K2,TGFBR2,BAG4	up_VSAP_EC
GO:1903902	0.0813	0.983	3	18	positive regulation of viral life cycle	BP	0.926	P4HB,CNOT7,RAD23A	down_VSAP_EC
GO:0010332	0.0813	0.989	2	20	response to gamma radiation	BP	1	CRYAB,DCUN1D3	up_AFEM_EC
GO:1902803	0.0813	0.989	2	20	regulation of synaptic vesicle transport	BP	1	CHRM2,CAMK2A	up_AFEM_EC
GO:2000300	0.0813	0.989	2	20	regulation of synaptic vesicle exocytosis	BP	1	CHRM2,CAMK2A	up_AFEM_EC
GO:0001841	0.0813	0.997	1	44	neural tube formation	BP	1	CITED2	down_VPOR_EC
GO:0002028	0.0813	0.997	1	19	regulation of sodium ion transport	BP	1	NETO1	up_VSAP_VSMC
GO:0048251	0.081380936	0.99772	1	3	elastic fiber assembly	BP	1	MFAP4	up_ACOR_EC
GO:0000165	0.0814	0.95	20	236	MAPK cascade	BP	0.926	VEGFA,JCAD,PTPN11,TRIB3,ILK,TNIK,PTPN2,MAGED1,CTNNB1,APOE,LYN,CBLC,ATF3,MAPK10,PAK2,INHBA,NRP1,SH3RF3,MADD,MID1	down_VSAP_EC

GO:0007507	0.0814	0.989	2	194	heart development	BP	1	EYA1,SHOX2	up_VFEM_VSMC
GO:1902523	0.081464534	0.99772	1	3	positive regulation of protein K63-linked	BP	1	NOD2	up_ACOR_EC
GO:0007163	0.0815	0.967	7	60	establishment or maintenance of cell	BP	0.928	FSCN1,ILK,WDR1,MSN,UST,HTT,ARF4	down_VSAP_EC
GO:0001822	0.0815	0.972	5	98	kidney development	BP	1	IRX3,DLL1,GREM1,OVOL1,GCNT4	up_AFEM_EC
GO:0061912	0.0815	0.978	4	18	selective autophagy	BP	1	RETREG1,AMBRA1,MFN2,HTRA2	up_VSAP_EC
GO:0045471	0.0815	0.991	2	5	response to ethanol	BP	1	TP53INP1,PRKCE	up_ACOR_VSMC
GO:0060429	0.0815	0.99	2	341	epithelium development	BP	1	GATA5,CA2	up_VCAV_VSMC
GO:0007219	0.0815	0.997	1	59	Notch signaling pathway	BP	1	GATA5	up_VCAV_VSMC
GO:1905517	0.0815	0.997	1	10	macrophage migration	BP	1	C3AR1	up_VPOR_EC
GO:0090263	0.0815	0.997	1	33	positive regulation of canonical Wnt signaling	BP	1	BAMBI	up_VPOR_VSMC
GO:0030534	0.0815	0.997	1	33	adult behavior	BP	1	CHL1	up_VPOR_VSMC
GO:0003208	0.0815	0.997	1	24	cardiac ventricle morphogenesis	BP	1	TBX5	up_VPUL_EC
GO:0001657	0.0815	0.997	1	42	ureteric bud development	BP	1	SLIT2	up_VPUL_VSMC
GO:0072163	0.0815	0.997	1	42	mesonephric epithelium development	BP	1	SLIT2	up_VPUL_VSMC
GO:0072164	0.0815	0.997	1	42	mesonephric tubule development	BP	1	SLIT2	up_VPUL_VSMC
GO:0007264	0.0816	0.954	15	164	small GTPase mediated signal transduction	BP	0.928	ARHGEF2,SIPA1L1,ARHGAP31,BCAR3,FARP1,KPNB1,APOE,PLEKHG2,TAX1BP3,ARFGEF3,PAK2,CDKN1A,NRP1,TNFAIP1,MADD	down_VSAP_EC
GO:0006351	0.0816	0.964	9	1069	transcription, DNA-templated	BP	1	ACVRL1,YEATS2,YBX3,HIVEP2,HOXA7,SULF1,ELK3,HOXA1,NEDD4	down_ACOR_EC
GO:0001505	0.0816	0.976	4	69	regulation of neurotransmitter levels	BP	1	KCNC4,CHRM2,CAMK2A,MOXD1	up_AFEM_EC
GO:0060119	0.0816	0.989	2	10	inner ear receptor cell development	BP	1	SDC4,NAGLU	down_VCAV_EC
GO:0010763	0.0816	0.991	2	5	positive regulation of fibroblast migration	BP	1	PRKCE,DDR2	up_ACOR_VSMC
GO:0030073	0.0816	0.997	1	42	insulin secretion	BP	1	RAB11B	down_VPOR_EC
GO:0006790	0.0816	0.997	1	99	sulfur compound metabolic process	BP	1	ENSCAFG00000028814	down_VSAP_VSMC
GO:0032609	0.0816	0.997	1	25	interferon-gamma production	BP	1	NR1H4	up_VPUL_EC
GO:0016236	0.081602566	0.97204	5	88	macroautophagy	BP	1	ATG16L2,CHMP4B,TP53INP2,VPS16,ENSCAFG00000010493	up_ACOR_EC
GO:0043123	0.0817	0.967	7	46	positive regulation of I-kappaB kinase/NF-kappaB	BP	0.922	TLR4,PELI1,CASP8,MAP3K3,TLR3,BCL10,RIPK1	up_VCAV_EC
GO:0045646	0.0817	0.997	1	19	regulation of erythrocyte differentiation	BP	1	LYN	down_ACOR_EC
GO:0071216	0.0817	0.997	1	61	cellular response to biotic stimulus	BP	1	TLR2	up_AFEM_VSMC
GO:0006614	0.0817	0.997	1	10	SRP-dependent cotranslational protein	BP	1	SRP54	up_VPOR_EC
GO:0060600	0.081732816	0.9977	1	3	dichotomous subdivision of an epithelial terminal	BP	1	NRP1	up_ACOR_EC
GO:0060666	0.081732816	0.9977	1	3	dichotomous subdivision of terminal units involved in salivary gland	BP	1	NRP1	up_ACOR_EC
GO:0034470	0.0818	0.952	18	154	ncRNA processing	BP	1	INTS7,SRRT,NOP56,FBL,DIS3,NOC4L,RSL1D1,THUMPD2,RRP1,OSGEP,ENSCAFG00000019620,NOP14,NOP58,ENSCAFG00000015189,EMG1,RTCB,EXOSC5,NOB1	down_ACOR_VSMC
GO:0006520	0.0818	0.953	16	114	cellular amino acid metabolic process	BP	1	HNF4A,GOT1,GLUL,ADHFE1,WARS2,ALDH6A1,ABAT,BCKDHB,ENSCAFG00000016140,AZIN2,FARS2,ENSCAFG00000002977,QARS,ASS1,LARS2,IDO2	up_VSAP_EC
GO:0032103	0.0818	0.976	4	69	positive regulation of response to external	BP	1	CMKLR1,NAPEPLD,CCL5,ADAM17	up_AFEM_EC
GO:1903039	0.0818	0.981	3	43	positive regulation of leukocyte cell-cell	BP	1	LEP,GPAM,CYLD	up_AFEM_EC
GO:0043412	0.0818	0.995	2	1225	macromolecule modification	BP	1	PLCL1,USP12	down_VFEM_EC
GO:0009855	0.0818	0.997	1	42	determination of bilateral symmetry	BP	1	CITED2	down_VPOR_EC
GO:0010883	0.0818	0.997	1	11	regulation of lipid storage	BP	1	ABHD5	up_VPOR_EC

GO:0006941	0.0818	0.997	1	36	striated muscle contraction	BP	1	NKX2-5	up_VPOR_VSMC
GO:0007260	0.0818	0.997	1	19	tyrosine phosphorylation of STAT protein	BP	1	CAV1	up_VSAP_VSMC
GO:0030111	0.0819	0.962	9	108	regulation of Wnt signaling pathway	BP	1	RECK,CLSTN1,ATP6V0C,GLI3,CDH2,GREM1,NPHP4,FOXO3,CDK14	down_VCAV_EC
GO:0031325	0.0819	0.985	3	914	positive regulation of cellular metabolic process	BP	1	HOXA9,WNT7A,ESR1	up_AORT_VSMC
GO:0030325	0.0819	0.991	2	5	adrenal gland development	BP	1	CITED2,ARID5B	up_ACOR_VSMC
GO:0044546	0.0819	0.991	2	6	NLRP3 inflammasome complex assembly	BP	0.922	GBP5,TLR4	up_VCAV_EC
GO:1900225	0.0819	0.991	2	6	regulation of NLRP3 inflammasome complex	BP	0.922	GBP5,TLR4	up_VCAV_EC
GO:0090659	0.0819	0.997	1	12	walking behavior	BP	1	SPG11	down_ACOR_EC
GO:0051456	0.0819	1	1	1	attachment of spindle microtubules to kinetochore involved in meiotic sister chromatid	BP	1	DSN1	down_ACOR_VSMC
GO:0009199	0.0819	0.997	1	100	ribonucleoside triphosphate metabolic	BP	1	AK5	down_VSAP_VSMC
GO:0060819	0.0819	1	1	1	inactivation of X chromosome by genetic	BP	1	PCGF5	up_ACOR_VSMC
GO:0014855	0.0819	0.997	1	22	striated muscle cell proliferation	BP	1	TBX5	up_APUL_EC
GO:0033627	0.0819	0.997	1	22	cell adhesion mediated by integrin	BP	1	NPNT	up_APUL_EC
GO:0009225	0.0819	0.997	1	10	nucleotide-sugar metabolic process	BP	1	GNPNAT1	up_VPOR_EC
GO:0001655	0.082	0.954	15	116	urogenital system development	BP	0.922	HOXB7,PDGFB,PODXL,MEF2C,TIPARP,GPR4,GATA3,MTSS1,ODC1,ARG2,BMP2,PROX1,JAG1,BASP1,ITGA6	up_VCAV_EC
GO:0032388	0.082	0.969	6	62	positive regulation of intracellular transport	BP	1	PRNP,ENSCAFG00000014978,AKT2,SCP2,GLI3,MAVS	down_VCAV_EC
GO:0031086	0.082	0.991	2	5	nuclear-transcribed mRNA catabolic process, deadenylation-	BP	1	PNRC1,DCP1B	up_ACOR_VSMC
GO:0031087	0.082	0.991	2	5	deadenylation-independent decapping of nuclear-transcribed mRNA	BP	1	PNRC1,DCP1B	up_ACOR_VSMC
GO:0002682	0.082	0.989	2	343	regulation of immune system process	BP	1	TLR2,HOXA9	up_AFEM_VSMC
GO:0015786	0.082	1	1	1	UDP-glucose transmembrane transport	BP	1	SLC35C2	down_ACOR_VSMC
GO:0046101	0.082	1	1	1	hypoxanthine biosynthetic process	BP	1	PRPS1	down_ACOR_VSMC
GO:0006551	0.082	1	1	1	leucine metabolic process	BP	1	BCAT1	down_ACOR_VSMC
GO:0009082	0.082	1	1	1	branched-chain amino acid biosynthetic process	BP	1	BCAT1	down_ACOR_VSMC
GO:0009098	0.082	1	1	1	leucine biosynthetic process	BP	1	BCAT1	down_ACOR_VSMC
GO:0009099	0.082	1	1	1	valine biosynthetic process	BP	1	BCAT1	down_ACOR_VSMC
GO:0046100	0.082	1	1	1	hypoxanthine metabolic process	BP	1	PRPS1	down_ACOR_VSMC
GO:0009126	0.082	0.997	1	100	purine nucleoside monophosphate metabolic process	BP	1	AK5	down_VSAP_VSMC
GO:0009167	0.082	0.997	1	100	purine ribonucleoside monophosphate metabolic process	BP	1	AK5	down_VSAP_VSMC
GO:0010212	0.082	0.997	1	60	response to ionizing radiation	BP	1	EYA1	up_AFEM_VSMC
GO:0048706	0.082	0.997	1	59	embryonic skeletal system development	BP	1	HOXC6	up_VCAV_VSMC
GO:0045980	0.082	0.997	1	10	negative regulation of nucleotide metabolic	BP	1	FBP1	up_VPOR_EC
GO:0071347	0.082	0.997	1	25	cellular response to interleukin-1	BP	1	CCL24	up_VPUL_EC
GO:0021766	0.082	0.997	1	20	hippocampus development	BP	1	EMX2	up_VSAP_VSMC
GO:0021824	0.082018822	0.99768	1	3	cerebral cortex tangential migration using cell-axon interactions	BP	1	NRP1	up_ACOR_EC
GO:0021828	0.082018822	0.99768	1	3	gonadotrophin-releasing hormone neuronal migration to the	BP	1	NRP1	up_ACOR_EC

GO:0021856	0.082018822	0.99768	1	3	hypothalamic tangential migration using cell-axon interactions	BP	1	NRP1	up_ACOR_EC
GO:0021886	0.082018822	0.99768	1	3	hypothalamus gonadotrophin-releasing hormone neuron	BP	1	NRP1	up_ACOR_EC
GO:0021888	0.082018822	0.99768	1	3	hypothalamus gonadotrophin-releasing hormone neuron	BP	1	NRP1	up_ACOR_EC
GO:0021979	0.082018822	0.99768	1	3	hypothalamus cell differentiation	BP	1	NRP1	up_ACOR_EC
GO:0035290	0.082018822	0.99768	1	3	trunk segmentation	BP	1	NRP1	up_ACOR_EC
GO:0036484	0.082018822	0.99768	1	3	trunk neural crest cell migration	BP	1	NRP1	up_ACOR_EC
GO:0036486	0.082018822	0.99768	1	3	ventral trunk neural crest cell migration	BP	1	NRP1	up_ACOR_EC
GO:0097490	0.082018822	0.99768	1	3	sympathetic neuron projection extension	BP	1	NRP1	up_ACOR_EC
GO:0097491	0.082018822	0.99768	1	3	sympathetic neuron projection guidance	BP	1	NRP1	up_ACOR_EC
GO:1901166	0.082018822	0.99768	1	3	neural crest cell migration involved in autonomic nervous system	BP	1	NRP1	up_ACOR_EC
GO:1903375	0.082018822	0.99768	1	3	facioacoustic ganglion development	BP	1	NRP1	up_ACOR_EC
GO:0010721	0.0821	0.964	8	73	negative regulation of cell development	BP	0.933	DPYSL3,HOOK3,IFRD1,CTNNB1,APOE,REST,NRP1,YWHAH	down_VSAP_EC
GO:0007166	0.0821	0.974	5	686	cell surface receptor signaling pathway	BP	1	NR1H4,CCL24,FLRT3,ARHGEF2,NEO1	up_VPUL_EC
GO:0006959	0.0821	0.982	3	21	humoral immune response	BP	1	CFD,TFE3,A2M	down_VCAV_EC
GO:0009799	0.0821	0.981	3	43	specification of symmetry	BP	1	DLL1,GREM1,GREM2	up_AFEM_EC
GO:0006692	0.0821	0.984	3	11	prostanoid metabolic process	BP	1	PTGES,ABHD16A,HPGD	up_VSAP_EC
GO:0006693	0.0821	0.984	3	11	prostaglandin metabolic process	BP	1	PTGES,ABHD16A,HPGD	up_VSAP_EC
GO:1900024	0.0821	0.997	1	16	regulation of substrate adhesion-dependent cell	BP	1	PDPN	down_ACOR_EC
GO:0030218	0.0821	0.997	1	42	erythrocyte differentiation	BP	1	CITED2	down_VPOR_EC
GO:0031109	0.0821	0.997	1	36	microtubule polymerization or	BP	1	TAOK1	up_AORT_EC
GO:0006936	0.0821	0.997	1	74	muscle contraction	BP	1	ARG2	up_AORT_VSMC
GO:0050928	0.0821	1	1	1	negative regulation of positive chemotaxis	BP	0.922	ANGPT2	up_VCAV_EC
GO:0014812	0.0821	0.997	1	21	muscle cell migration	BP	1	SIX1	up_VFEM_EC
GO:0051781	0.0821	0.997	1	18	positive regulation of cell division	BP	1	FGF5	up_VSAP_VSMC
GO:0010468	0.0822	0.972	6	1270	regulation of gene expression	BP	1	EYA1,HOXA10,HOXA9,EMX2,HOXA11,SHOX2	up_VFEM_VSMC
GO:0051250	0.0822	0.973	5	38	negative regulation of lymphocyte activation	BP	0.933	SOCS5,PTPN2,LYN,SDC4,BTK	down_VSAP_EC
GO:0042737	0.0822	0.978	4	18	drug catabolic process	BP	1	MAOA,FMO4,MOXD1,CAT	up_ACOR_VSMC
GO:0033059	0.0822	0.983	3	12	cellular pigmentation	BP	0.922	RAB11A,RAB27A,SHROOM2	up_VCAV_EC
GO:0034724	0.0822	0.983	3	12	DNA replication-independent nucleosome	BP	0.922	NASP,ASF1A,ATRX	up_VCAV_EC
GO:0045638	0.0822	0.997	1	33	negative regulation of myeloid cell	BP	1	HOXA9	up_VFEM_VSMC
GO:0007613	0.0822	0.997	1	20	memory	BP	1	NETO1	up_VSAP_VSMC
GO:0043161	0.0823	0.954	15	171	proteasome-mediated ubiquitin-dependent protein catabolic process	BP	0.934	PRICKLE1,PLK3,SOCS5,HSP90B1,TRIB3,DNAJC10,UCHL5,SPOPL,DNAJB9,DDIT3,GSK3B,SPOP,RAD23A,USP14,TNFAIP1	down_VSAP_EC
GO:0035137	0.0823	0.978	4	18	hindlimb morphogenesis	BP	1	OSR2,OSR1,ZBTB16,RARG	up_VSAP_EC
GO:0070838	0.0823	0.988	2	96	divalent metal ion transport	BP	1	LYN,HOXA3	down_ACOR_EC
GO:0051130	0.0823	0.989	2	342	positive regulation of cellular component	BP	1	SLITRK5,LIMK1	up_VCAV_VSMC
GO:0072676	0.0823	0.997	1	25	lymphocyte migration	BP	1	CCL24	up_VPUL_EC
GO:1904016	0.0823	1	1	1	response to Thyroglobulin triiodothyronine	BP	1	NCOA2	up_VSAP_EC

GO:1904017	0.0823	1	1	1	cellular response to Thyroglobulin	BP	1	NCOA2	up_VSAP_EC
GO:1990791	0.082313577	0.99767	1	3	dorsal root ganglion development	BP	1	NRP1	up_ACOR_EC
GO:0021648	0.08238223	0.99766	1	3	vestibulocochlear nerve morphogenesis	BP	1	NRP1	up_ACOR_EC
GO:0097105	0.082382502	0.99766	1	3	presynaptic membrane assembly	BP	1	LRP4	up_ACOR_EC
GO:0032880	0.0824	0.944	33	266	regulation of protein localization	BP	1	EXPH5,SH3TC2,TLR1,ACTN2,LEP,HTR2B,TCAF1,MBP,PRKAA2,FERMT1,BMP4,TM7SF3,CD274,AR,ANKRD1,F2RL1,CDKN1A,PTPN22,OS9,PRKCE,DAG1,PARP9,PSEN1,JUP,CCL5,RAB11B,NOD2,SCAMP5,INPP5K,PFKL,WASL,PARK7,PTPN14	up_ACOR_VSMC
GO:0048592	0.0824	0.964	8	53	eye morphogenesis	BP	0.924	EPHB1,PROX1,TSPAN12,JAG1,HIF1A,ALDH1A3,ZEB1,CDON	up_VCAV_EC
GO:0071359	0.0824	0.984	3	11	cellular response to dsRNA	BP	1	PQBP1,FLOT1,MAVS	up_VSAP_EC
GO:0051050	0.0824	0.981	3	235	positive regulation of transport	BP	1	STIM1,TLR2,CAV1	up_VSAP_VSMC
GO:1903055	0.0824	0.991	2	6	positive regulation of extracellular matrix	BP	1	FSCN1,COLGALT1	down_ACOR_VSMC
GO:0003139	0.0824	0.991	2	5	secondary heart field specification	BP	1	BMP4,SMARCD3	up_ACOR_VSMC
GO:0010872	0.0824	0.998	1	2	regulation of cholesterol esterification	BP	0.934	APOE	down_VSAP_EC
GO:0032612	0.0824	0.997	1	22	interleukin-1 production	BP	1	NR1H4	up_APUL_EC
GO:0060411	0.0824	0.997	1	33	cardiac septum morphogenesis	BP	1	NKX2-5	up_VPOR_VSMC
GO:0032835	0.0824	0.997	1	21	glomerulus development	BP	1	MTSS1	up_VFEM_EC
GO:0030879	0.0824	0.997	1	34	mammary gland development	BP	1	HOXA9	up_VFEM_VSMC
GO:0099537	0.0825	0.953	16	131	trans-synaptic signaling	BP	0.924	EPHB1,PLK2,ENSCAFG00000007112,RELN,MEF2C,EDN1,SNCAIP,HOMER3,PPP3CA,GUCY1A1,SNAP23,SLC12A7,GRM8,STX11,ALS2,P2RX2	up_VCAV_EC
GO:0097659	0.0825	0.964	9	1072	nucleic acid-templated transcription	BP	1	ACVRL1,YEATS2,YBX3,HIVEP2,HOXA7,SULF1,ELK3,HOXA1,NEDD4	down_ACOR_EC
GO:0048025	0.0825	0.991	2	6	negative regulation of mRNA splicing, via	BP	1	RNPS1,U2AF2	down_ACOR_VSMC
GO:0018209	0.0825	0.988	2	125	peptidyl-serine modification	BP	1	PLCL1,NTRK2	up_APUL_EC
GO:0051546	0.0825	0.991	2	6	keratinocyte migration	BP	0.924	FERMT1,MTOR	up_VCAV_EC
GO:0045216	0.0825	0.997	1	44	cell-cell junction organization	BP	1	HDAC7	down_VPOR_EC
GO:0006897	0.0826	0.965	7	158	endocytosis	BP	1	LEP,RAB20,DLL1,GREM1,ICAM5,RAB7A,ANXA11	up_AFEM_EC
GO:0007369	0.0826	0.997	1	63	gastrulation	BP	1	EYA1	up_AFEM_VSMC
GO:1900026	0.0826	0.997	1	11	positive regulation of substrate adhesion-dependent cell spreading	BP	1	ENSCAFG00000002015	up_VPOR_EC
GO:0050729	0.0826	0.997	1	25	positive regulation of inflammatory response	BP	1	CCL24	up_VPUL_EC
GO:0009653	0.0827	0.961	10	773	anatomical structure morphogenesis	BP	1	C3AR1,PTK7,HOXA3,ARHGEF2,ENSCAFG00000002015,SEMA3A,ASPN,ACVR2A,MTDH,RNF6	up_VPOR_EC
GO:0046661	0.0827	0.967	7	45	male sex differentiation	BP	1	SF1,DHCR24,HMGB2,SGPL1,ASPM,HOXA9,SIX4	down_ACOR_VSMC
GO:0031323	0.0827	0.979	5	1788	regulation of cellular metabolic process	BP	1	TLR2,EYA1,HOXA9,HOXA10,IRX5	up_AFEM_VSMC
GO:0042743	0.0827	0.991	2	5	hydrogen peroxide metabolic process	BP	1	CAT,PARK7	up_ACOR_VSMC
GO:0070664	0.0827	0.997	1	18	negative regulation of leukocyte proliferation	BP	1	LYN	down_ACOR_EC
GO:0032677	0.0827	0.997	1	22	regulation of interleukin-8 production	BP	1	BPI	up_APUL_EC
GO:0045927	0.0827	0.997	1	61	positive regulation of growth	BP	1	LIMK1	up_VCAV_VSMC
GO:0001554	0.0827	1	1	1	luteolysis	BP	1	SLIT2	up_VSAP_EC
GO:0010593	0.0827	1	1	1	negative regulation of lamellipodium assembly	BP	1	SLIT2	up_VSAP_EC
GO:0021823	0.0827	1	1	1	cerebral cortex tangential migration using cell-cell interactions	BP	1	SLIT2	up_VSAP_EC

GO:0021827	0.0827	1	1	1	postnatal olfactory bulb interneuron migration	BP	1	SLIT2	up_VSAP_EC
GO:0021836	0.0827	1	1	1	chemorepulsion involved in postnatal olfactory bulb interneuron migration	BP	1	SLIT2	up_VSAP_EC
GO:0021966	0.0827	1	1	1	corticospinal neuron axon guidance	BP	1	SLIT2	up_VSAP_EC
GO:0021972	0.0827	1	1	1	corticospinal neuron axon guidance through spinal	BP	1	SLIT2	up_VSAP_EC
GO:0035385	0.0827	1	1	1	Roundabout signaling pathway	BP	1	SLIT2	up_VSAP_EC
GO:0050924	0.0827	1	1	1	positive regulation of negative chemotaxis	BP	1	SLIT2	up_VSAP_EC
GO:0050929	0.0827	1	1	1	induction of negative chemotaxis	BP	1	SLIT2	up_VSAP_EC
GO:0051414	0.0827	1	1	1	response to cortisol	BP	1	SLIT2	up_VSAP_EC
GO:0061364	0.0827	1	1	1	apoptotic process involved in luteolysis	BP	1	SLIT2	up_VSAP_EC
GO:0070099	0.0827	1	1	1	regulation of chemokine-mediated signaling	BP	1	SLIT2	up_VSAP_EC
GO:0070100	0.0827	1	1	1	negative regulation of chemokine-mediated	BP	1	SLIT2	up_VSAP_EC
GO:0071670	0.0827	1	1	1	smooth muscle cell chemotaxis	BP	1	SLIT2	up_VSAP_EC
GO:0071671	0.0827	1	1	1	regulation of smooth muscle cell chemotaxis	BP	1	SLIT2	up_VSAP_EC
GO:0071672	0.0827	1	1	1	negative regulation of smooth muscle cell	BP	1	SLIT2	up_VSAP_EC
GO:0090260	0.0827	1	1	1	negative regulation of retinal ganglion cell axon	BP	1	SLIT2	up_VSAP_EC
GO:0006366	0.0828	0.938	59	583	transcription by RNA polymerase II	BP	0.926	EDNRB,HCLS1,FLI1,PPARG,TNIP1,PDGFB,TMEM173,MEF2C,ATF3,CHD7,DLL4,TLR4,NLRC5,GATA3,EDN1,HOXB8,TCF4,SMAD1,ENSCAFG00000017103,IRF8,SOX18,PPP3CA,YES1,ELF4,BMP2,HMG20A,HHEX,CREM,PROX1,ARID4B,HMBOX1,PRKD2,SMARCA4,TLR3,NCK1,TIMELESS,LDB2,JAG1,TCERG1,CTDSPL2,CNOT7,HMGB1,AHR,RIPK1,ITGA6,SKIL,MTA2,KDM5A,HIF1A,KDM3A,ATRX,NR4A1,ARID5A,ZEB1,TET2,ARHGEF2,CDON,ELK3,ZNF609	up_VCAV_EC
GO:0043062	0.0828	0.955	14	99	extracellular structure organization	BP	1	LIPC,ABL1,CTSS,ADAMTSL4,RECK,DDR2,SULF2,LOX,ECM2,LCAT,FBLN1,FLOT1,DPT,SOAT1	up_VSAP_EC
GO:0032774	0.0828	0.964	9	1073	RNA biosynthetic process	BP	1	ACVRL1,YEATS2,YBX3,HIVEP2,HOXA7,SULF1,ELK3,HOXA1,NEDD4	down_ACOR_EC
GO:0031032	0.0828	0.966	7	61	actomyosin structure organization	BP	0.936	ACTN1,WDR1,ENSCAFG00000017326,SIX4,NRP1,TNFAIP1,MYBPC2	down_VSAP_EC
GO:0018195	0.0828	0.991	2	6	peptidyl-arginine modification	BP	1	PRMT1,PRMT5	down_ACOR_VSMC
GO:0071539	0.0828	0.99	2	8	protein localization to centrosome	BP	0.936	HOOK3,GSK3B	down_VSAP_EC
GO:1905508	0.0828	0.99	2	8	protein localization to microtubule organizing	BP	0.936	HOOK3,GSK3B	down_VSAP_EC
GO:0007595	0.0828	0.991	2	6	lactation	BP	0.926	CREB1,HIF1A	up_VCAV_EC
GO:0021798	0.0828	0.998	1	2	forebrain dorsal/ventral pattern formation	BP	1	GLI3	down_VCAV_EC
GO:0035441	0.0828	0.998	1	2	cell migration involved in vasculogenesis	BP	1	PDGFRB	down_VCAV_EC
GO:1903555	0.0828	0.997	1	37	regulation of tumor necrosis factor superfamily cytokine	BP	1	BPI	up_AORT_EC
GO:0071333	0.0828	0.997	1	25	cellular response to glucose stimulus	BP	1	NR1H4	up_VPUL_EC
GO:0042327	0.082830342	0.95641	12	289	positive regulation of phosphorylation	BP	1	SOX9,ENSCAFG00000004331,FZD5,NOD2,PAK3,TNFRSF19,MAVS,LRP4,SH3RF3,RARRES2,NRP1,PRKCD	up_ACOR_EC
GO:0021559	0.082874666	0.99763	1	3	trigeminal nerve development	BP	1	NRP1	up_ACOR_EC
GO:0021636	0.082874666	0.99763	1	3	trigeminal nerve morphogenesis	BP	1	NRP1	up_ACOR_EC
GO:0021637	0.082874666	0.99763	1	3	trigeminal nerve structural organization	BP	1	NRP1	up_ACOR_EC
GO:0021785	0.082874666	0.99763	1	3	branchiomotor neuron axon guidance	BP	1	NRP1	up_ACOR_EC
GO:1902287	0.082874666	0.99763	1	3	semaphorin-plexin signaling pathway involved in axon guidance	BP	1	NRP1	up_ACOR_EC

GO:0071222	0.0829	0.966	7	46	cellular response to lipopolysaccharide	BP	0.926	CXCL8,TNFAIP3,TNIP1,PDE4D,MEF2C,IRF8,ABCA1	up_VCAV_EC
GO:0045833	0.0829	0.977	4	29	negative regulation of lipid metabolic process	BP	0.937	NR1H4,TRIB3,APOE,REST	down_VSAP_EC
GO:0022008	0.0829	0.982	3	406	neurogenesis	BP	1	CHL1,NKX2-5,TRPC4	up_VPOR_VSMC
GO:0046426	0.0829	0.997	1	20	negative regulation of JAK-STAT cascade	BP	1	ASPN	down_ACOR_EC
GO:1904893	0.0829	0.997	1	20	negative regulation of STAT cascade	BP	1	ASPN	down_ACOR_EC
GO:1904628	0.0829	1	1	1	cellular response to phorbol 13-acetate 12-	BP	1	CDK4	down_ACOR_VSMC
GO:1904627	0.0829	1	1	1	response to phorbol 13-acetate 12-myristate	BP	1	CDK4	down_ACOR_VSMC
GO:0061478	0.0829	0.998	1	2	response to platelet aggregation inhibitor	BP	1	ADCY6	down_VCAV_EC
GO:1904321	0.0829	0.998	1	2	response to forskolin	BP	1	ADCY6	down_VCAV_EC
GO:1904322	0.0829	0.998	1	2	cellular response to forskolin	BP	1	ADCY6	down_VCAV_EC
GO:0002757	0.0829	0.997	1	78	immune response-activating signal	BP	1	ESR1	up_AORT_VSMC
GO:0048511	0.0829	0.997	1	72	rhythmic process	BP	1	ESR1	up_AORT_VSMC
GO:0090101	0.0829	0.997	1	34	negative regulation of transmembrane receptor protein serine/threonine kinase signaling pathway	BP	1	BAMBI	up_VPOR_VSMC
GO:0051249	0.083	0.961	9	107	regulation of lymphocyte activation	BP	1	GPAM,PRNP,SDC4,THY1,GLI3,ZFP36L1,LOXL3,RAC2,ZBTB16	down_VCAV_EC
GO:0010604	0.083	0.985	3	919	positive regulation of macromolecule metabolic	BP	1	HOXA9,WNT7A,ESR1	up_AORT_VSMC
GO:0050691	0.083	0.983	3	11	regulation of defense response to virus by host	BP	1	RNF216,PQBP1,MAVS	up_VSAP_EC
GO:0038179	0.083	0.989	2	10	neurotrophin signaling pathway	BP	1	NDN,AKT1S1	down_VCAV_EC
GO:0030595	0.083	0.988	2	57	leukocyte chemotaxis	BP	1	C3AR1,ENSCAFG00000032731	up_VPOR_EC
GO:0043985	0.083	1	1	1	histone H4-R3 methylation	BP	1	PRMT1	down_ACOR_VSMC
GO:0022904	0.083	0.997	1	43	respiratory electron transport chain	BP	1	NDUFA10	down_AFEM_EC
GO:0045944	0.083	0.998	1	287	positive regulation of transcription by RNA polymerase II	BP	1	GATA5	down_VFEM_VSMC
GO:0045956	0.083	1	1	1	positive regulation of calcium ion-dependent	BP	1	SCAMP5	up_ACOR_VSMC
GO:0045637	0.083	0.997	1	73	regulation of myeloid cell differentiation	BP	1	HOXA9	up_AORT_VSMC
GO:0051648	0.083	0.997	1	69	vesicle localization	BP	1	WNT7A	up_AORT_VSMC
GO:0072329	0.083	0.997	1	34	monocarboxylic acid catabolic process	BP	1	CYP26B1	up_VPOR_VSMC
GO:0035307	0.083044283	0.98841	2	18	positive regulation of protein	BP	1	DUSP26,PRKCD	up_ACOR_EC
GO:0000904	0.0831	0.981	3	181	cell morphogenesis involved in differentiation	BP	1	SPG11,PDPN,NRP2	down_ACOR_EC
GO:0031111	0.0831	0.983	3	11	negative regulation of microtubule polymerization or	BP	1	FGF13,MID1,TUBB4A	up_ACOR_VSMC
GO:0010896	0.0831	1	1	1	regulation of triglyceride catabolic process	BP	1	ABHD5	down_ACOR_VSMC
GO:0010898	0.0831	1	1	1	positive regulation of triglyceride catabolic	BP	1	ABHD5	down_ACOR_VSMC
GO:0009953	0.0831	0.997	1	34	dorsal/ventral pattern formation	BP	1	HOXA11	up_VFEM_VSMC
GO:0019218	0.0831	0.997	1	25	regulation of steroid metabolic process	BP	1	NR1H4	up_VPUL_EC
GO:0060982	0.083125298	0.99762	1	3	coronary artery morphogenesis	BP	1	NRP1	up_ACOR_EC
GO:0051271	0.0832	0.971	5	98	negative regulation of cellular component	BP	1	ANGPT4,HOXA7,RECK,TBXA2R,KANK1	up_AFEM_EC
GO:0051150	0.0832	0.983	3	13	regulation of smooth muscle cell differentiation	BP	0.928	KIT,PDGFB,ZEB1	up_VCAV_EC
GO:0055074	0.0832	0.988	2	95	calcium ion homeostasis	BP	1	LYN,HOXA3	down_ACOR_EC
GO:1903034	0.0832	0.997	1	40	regulation of response to wounding	BP	1	SRSF6	down_AFEM_EC
GO:0006694	0.0832	0.997	1	42	steroid biosynthetic process	BP	1	HSD3B7	down_VPOR_EC

GO:2000224	0.0832	1	1	1	regulation of testosterone biosynthetic process	BP	1	DKKL1	up_ACOR_VSMC
GO:2000225	0.0832	1	1	1	negative regulation of testosterone biosynthetic	BP	1	DKKL1	up_ACOR_VSMC
GO:0061370	0.0832	1	1	1	testosterone biosynthetic process	BP	1	DKKL1	up_ACOR_VSMC
GO:0051249	0.0833	0.953	15	107	regulation of lymphocyte activation	BP	1	CD274,TBC1D10C,ABL1,ZBTB16,TOX,STAT6,ZFP36L1,ZEB1,MYD88,FBXO7,CD83,ZFP36L2,PNP,NCK1,TGFBR2	up_VSAP_EC
GO:0032488	0.0833	0.991	2	5	Cdc42 protein signal transduction	BP	1	ABCA1,NRP1	up_ACOR_VSMC
GO:0032489	0.0833	0.991	2	5	regulation of Cdc42 protein signal	BP	1	ABCA1,NRP1	up_ACOR_VSMC
GO:0002507	0.0833	0.99	2	7	tolerance induction	BP	0.929	TNFAIP3,HMGB1	up_VCAV_EC
GO:0000022	0.0833	1	1	1	mitotic spindle elongation	BP	1	AURKB	down_ACOR_VSMC
GO:0051256	0.0833	1	1	1	mitotic spindle midzone assembly	BP	1	AURKB	down_ACOR_VSMC
GO:0043062	0.0833	0.997	1	99	extracellular structure organization	BP	1	TGFBI	down_VSAP_VSMC
GO:0055024	0.0833	0.997	1	22	regulation of cardiac muscle tissue	BP	1	TBX5	up_APUL_EC
GO:0032259	0.0834	0.96	10	71	methylation	BP	0.929	IWS1,GATA3,ARID4B,NSD1,WTAP,MTA2,KDM3A,ATRX,ZC3H13,TET2	up_VCAV_EC
GO:0050810	0.0834	0.982	3	17	regulation of steroid biosynthetic process	BP	0.941	NR1H4,APOE,REST	down_VSAP_EC
GO:0007026	0.0834	0.99	2	8	negative regulation of microtubule	BP	0.941	ARHGEF2,MID1	down_VSAP_EC
GO:0060333	0.0834	0.99	2	8	interferon-gamma-mediated signaling	BP	0.941	PTPN2,CDC37	down_VSAP_EC
GO:0007056	0.0834	1	1	1	spindle assembly involved in female meiosis	BP	1	FBXO5	down_ACOR_VSMC
GO:0007057	0.0834	1	1	1	spindle assembly involved in female meiosis I	BP	1	FBXO5	down_ACOR_VSMC
GO:1905319	0.0834	1	1	1	mesenchymal stem cell migration	BP	1	FBXO5	down_ACOR_VSMC
GO:1905320	0.0834	1	1	1	regulation of mesenchymal stem cell	BP	1	FBXO5	down_ACOR_VSMC
GO:1905322	0.0834	1	1	1	positive regulation of mesenchymal stem cell	BP	1	FBXO5	down_ACOR_VSMC
GO:0031032	0.0834	0.997	1	61	actomyosin structure organization	BP	1	LIMK1	up_VCAV_VSMC
GO:0003170	0.0834	0.997	1	25	heart valve development	BP	1	TBX5	up_VPUL_EC
GO:1903364	0.083459916	0.9755	4	61	positive regulation of cellular protein catabolic	BP	1	EDEM2,PIAS1,CLU,RNF114	up_ACOR_EC
GO:0040013	0.0835	0.959	10	100	negative regulation of locomotion	BP	0.941	SULF1,SERPINE1,DPYSL3,PTPN2,STARD13,APOE,MMP28,STRAP,ZMYND8,NRP1	down_VSAP_EC
GO:0033572	0.0835	0.991	2	5	transferrin transport	BP	1	RAB11B,LMTK2	up_ACOR_VSMC
GO:0002682	0.0835	0.989	2	343	regulation of immune system process	BP	1	LRRC17,C6	up_VCAV_VSMC
GO:0060978	0.0835	0.998	1	2	angiogenesis involved in coronary vascular	BP	1	PDGFRB	down_VCAV_EC
GO:0003073	0.0835	0.997	1	20	regulation of systemic arterial blood pressure	BP	1	AVPR1A	up_VFEM_EC
GO:0048771	0.0835	0.997	1	43	tissue remodeling	BP	1	HOXA3	up_VPUL_VSMC
GO:0015722	0.0835	1	1	1	canalicular bile acid transport	BP	1	ABCB11	up_VSAP_EC
GO:0045727	0.0836	0.966	7	40	positive regulation of translation	BP	1	PRR16,PYM1,EEF2,LARP6,RPUSD4,EIF3D,NCK1	up_VSAP_EC
GO:0001894	0.0836	0.968	6	49	tissue homeostasis	BP	0.941	PTPN11,EXOC5,CTNNB1,TFRC,STRAP,HOMER1	down_VSAP_EC
GO:0046916	0.0836	0.997	1	35	cellular transition metal ion homeostasis	BP	1	ENSCAFG00000005494	up_AORT_EC
GO:0032642	0.0836	0.997	1	22	regulation of chemokine production	BP	1	NR1H4	up_APUL_EC
GO:0032621	0.0836	1	1	1	interleukin-18 production	BP	0.929	GBP5	up_VCAV_EC
GO:0072616	0.0836	1	1	1	interleukin-18 secretion	BP	0.929	GBP5	up_VCAV_EC
GO:0071526	0.0836	0.997	1	12	semaphorin-plexin signaling pathway	BP	1	SEMA3A	up_VPOR_EC
GO:0043407	0.0836	0.997	1	19	negative regulation of MAP kinase activity	BP	1	CAV1	up_VSAP_VSMC
GO:0046626	0.083678674	0.98827	2	18	regulation of insulin receptor signaling	BP	1	NR1H4,PRKCD	up_ACOR_EC

GO:0070664	0.0837	0.982	3	18	negative regulation of leukocyte proliferation	BP	0.941	LYN,SDC4,BTK	down_VSAP_EC
GO:0032653	0.0837	0.983	3	11	regulation of interleukin-10 production	BP	1	CD274,DLL1,CD83	up_VSAP_EC
GO:1902622	0.0837	0.983	3	11	regulation of neutrophil migration	BP	1	C5AR2,SLIT2,MYD88	up_VSAP_EC
GO:0008626	0.0837	1	1	1	granzyme-mediated apoptotic signaling	BP	1	BNIP3	up_ACOR_VSMC
GO:0061138	0.0837	0.997	1	73	morphogenesis of a branching epithelium	BP	1	ESR1	up_AORT_VSMC
GO:0046785	0.0837	0.997	1	23	microtubule polymerization	BP	1	TUBB4A	up_APUL_EC
GO:0045332	0.0837	1	1	1	phospholipid translocation	BP	0.929	ATP8A1	up_VCAV_EC
GO:0061091	0.0837	1	1	1	regulation of phospholipid translocation	BP	0.929	ATP8A1	up_VCAV_EC
GO:0061092	0.0837	1	1	1	positive regulation of phospholipid	BP	0.929	ATP8A1	up_VCAV_EC
GO:1901019	0.0837	0.997	1	20	regulation of calcium ion transmembrane transporter activity	BP	1	STIM1	up_VSAP_VSMC
GO:1903707	0.0838	0.964	8	48	negative regulation of hemopoiesis	BP	1	HOXA7,HOXA9,DLL1,ZBTB16,ZFP36L1,PIK3R1,MYC,FBXO7	up_VSAP_EC
GO:0001912	0.0838	0.989	2	9	positive regulation of leukocyte mediated	BP	0.941	FAM49B,PVR	down_VSAP_EC
GO:0046184	0.0838	0.99	2	6	aldehyde biosynthetic process	BP	0.929	BMP2,KDM3A	up_VCAV_EC
GO:0030224	0.0838	0.997	1	18	monocyte differentiation	BP	1	HOXA7	down_ACOR_EC
GO:1903131	0.0838	0.997	1	18	mononuclear cell differentiation	BP	1	HOXA7	down_ACOR_EC
GO:0018395	0.0838	1	1	1	peptidyl-lysine hydroxylation to 5-	BP	1	JMJD6	down_ACOR_VSMC
GO:0009799	0.0838	0.997	1	43	specification of symmetry	BP	1	CITED2	down_VPOR_EC
GO:0002438	0.0838	1	1	1	acute inflammatory response to antigenic	BP	1	PARK7	up_ACOR_VSMC
GO:0002866	0.0838	1	1	1	positive regulation of acute inflammatory response to antigenic	BP	1	PARK7	up_ACOR_VSMC
GO:0009441	0.0838	1	1	1	glycolate metabolic process	BP	1	PARK7	up_ACOR_VSMC
GO:0010273	0.0838	1	1	1	detoxification of copper ion	BP	1	PARK7	up_ACOR_VSMC
GO:0018323	0.0838	1	1	1	enzyme active site formation via L-cysteine	BP	1	PARK7	up_ACOR_VSMC
GO:0033860	0.0838	1	1	1	regulation of NAD(P)H oxidase activity	BP	1	PARK7	up_ACOR_VSMC
GO:0033864	0.0838	1	1	1	positive regulation of NAD(P)H oxidase activity	BP	1	PARK7	up_ACOR_VSMC
GO:0036471	0.0838	1	1	1	cellular response to glyoxal	BP	1	PARK7	up_ACOR_VSMC
GO:0036482	0.0838	1	1	1	neuron intrinsic apoptotic signaling pathway in response to hydrogen	BP	1	PARK7	up_ACOR_VSMC
GO:0036525	0.0838	1	1	1	protein deglycation	BP	1	PARK7	up_ACOR_VSMC
GO:0036526	0.0838	1	1	1	peptidyl-cysteine deglycation	BP	1	PARK7	up_ACOR_VSMC
GO:0036527	0.0838	1	1	1	peptidyl-arginine deglycation	BP	1	PARK7	up_ACOR_VSMC
GO:0036528	0.0838	1	1	1	peptidyl-lysine deglycation	BP	1	PARK7	up_ACOR_VSMC
GO:0036529	0.0838	1	1	1	protein deglycation, glyoxal removal	BP	1	PARK7	up_ACOR_VSMC
GO:0036530	0.0838	1	1	1	protein deglycation, methylglyoxal removal	BP	1	PARK7	up_ACOR_VSMC
GO:0036531	0.0838	1	1	1	glutathione deglycation	BP	1	PARK7	up_ACOR_VSMC
GO:0046295	0.0838	1	1	1	glycolate biosynthetic process	BP	1	PARK7	up_ACOR_VSMC
GO:0046826	0.0838	1	1	1	negative regulation of protein export from	BP	1	PARK7	up_ACOR_VSMC
GO:0002863	0.0838	1	1	1	positive regulation of inflammatory response to antigenic stimulus	BP	1	PARK7	up_ACOR_VSMC
GO:0002864	0.0838	1	1	1	regulation of acute inflammatory response to	BP	1	PARK7	up_ACOR_VSMC
GO:0050787	0.0838	1	1	1	detoxification of mercury ion	BP	1	PARK7	up_ACOR_VSMC

GO:0060081	0.0838	1	1	1	membrane hyperpolarization	BP	1	PARK7	up_ACOR_VSMC
GO:0061687	0.0838	1	1	1	detoxification of inorganic compound	BP	1	PARK7	up_ACOR_VSMC
GO:0090086	0.0838	1	1	1	negative regulation of protein deubiquitination	BP	1	PARK7	up_ACOR_VSMC
GO:0097238	0.0838	1	1	1	cellular response to methylglyoxal	BP	1	PARK7	up_ACOR_VSMC
GO:0097501	0.0838	1	1	1	stress response to metal ion	BP	1	PARK7	up_ACOR_VSMC
GO:0106044	0.0838	1	1	1	guanine deglycation	BP	1	PARK7	up_ACOR_VSMC
GO:0106045	0.0838	1	1	1	guanine deglycation, methylglyoxal removal	BP	1	PARK7	up_ACOR_VSMC
GO:0106046	0.0838	1	1	1	guanine deglycation, glyoxal removal	BP	1	PARK7	up_ACOR_VSMC
GO:0140041	0.0838	1	1	1	cellular detoxification of methylglyoxal	BP	1	PARK7	up_ACOR_VSMC
GO:1901671	0.0838	1	1	1	positive regulation of superoxide dismutase	BP	1	PARK7	up_ACOR_VSMC
GO:1902958	0.0838	1	1	1	positive regulation of mitochondrial electron transport, NADH to	BP	1	PARK7	up_ACOR_VSMC
GO:1903094	0.0838	1	1	1	negative regulation of protein K48-linked	BP	1	PARK7	up_ACOR_VSMC
GO:1903122	0.0838	1	1	1	negative regulation of TRAIL-activated apoptotic signaling pathway	BP	1	PARK7	up_ACOR_VSMC
GO:1903167	0.0838	1	1	1	regulation of pyrroline-5-carboxylate reductase	BP	1	PARK7	up_ACOR_VSMC
GO:1903168	0.0838	1	1	1	positive regulation of pyrroline-5-carboxylate	BP	1	PARK7	up_ACOR_VSMC
GO:1903184	0.0838	1	1	1	L-dopa metabolic process	BP	1	PARK7	up_ACOR_VSMC
GO:1903185	0.0838	1	1	1	L-dopa biosynthetic process	BP	1	PARK7	up_ACOR_VSMC
GO:1903189	0.0838	1	1	1	glyoxal metabolic process	BP	1	PARK7	up_ACOR_VSMC
GO:1903195	0.0838	1	1	1	regulation of L-dopa biosynthetic process	BP	1	PARK7	up_ACOR_VSMC
GO:1903197	0.0838	1	1	1	positive regulation of L-dopa biosynthetic process	BP	1	PARK7	up_ACOR_VSMC
GO:1903383	0.0838	1	1	1	regulation of hydrogen peroxide-induced neuron intrinsic apoptotic	BP	1	PARK7	up_ACOR_VSMC
GO:1903384	0.0838	1	1	1	negative regulation of hydrogen peroxide-induced neuron intrinsic	BP	1	PARK7	up_ACOR_VSMC
GO:1903750	0.0838	1	1	1	regulation of intrinsic apoptotic signaling pathway in response to	BP	1	PARK7	up_ACOR_VSMC
GO:1903751	0.0838	1	1	1	negative regulation of intrinsic apoptotic signaling pathway in	BP	1	PARK7	up_ACOR_VSMC
GO:1904833	0.0838	1	1	1	positive regulation of removal of superoxide	BP	1	PARK7	up_ACOR_VSMC
GO:1905258	0.0838	1	1	1	regulation of nitrosative stress-induced intrinsic apoptotic signaling	BP	1	PARK7	up_ACOR_VSMC
GO:1905259	0.0838	1	1	1	negative regulation of nitrosative stress-induced intrinsic apoptotic	BP	1	PARK7	up_ACOR_VSMC
GO:1990169	0.0838	1	1	1	stress response to copper ion	BP	1	PARK7	up_ACOR_VSMC
GO:2000152	0.0838	1	1	1	regulation of ubiquitin-specific protease activity	BP	1	PARK7	up_ACOR_VSMC
GO:2000157	0.0838	1	1	1	negative regulation of ubiquitin-specific	BP	1	PARK7	up_ACOR_VSMC
GO:2000277	0.0838	1	1	1	positive regulation of oxidative phosphorylation uncoupler activity	BP	1	PARK7	up_ACOR_VSMC
GO:2000284	0.0838	1	1	1	positive regulation of cellular amino acid	BP	1	PARK7	up_ACOR_VSMC
GO:1901863	0.0838	0.997	1	25	positive regulation of muscle tissue	BP	1	TBX5	up_VPUL_EC

GO:0010604	0.0839	0.934	88	919	positive regulation of macromolecule metabolic process	BP	0.929	PLK2,TEK,HCLS1,IL7R,KIT,SELE,PPP2R5A,TNFAIP3,PPARG,MAP3K11,TNIP1,PDGFB,STK10,TMEM173,RELN,MEF2C,ATF3,CHD7,TIPARP,DLL4,TLR4,NLRC5,GATA3,EDN1,RALB,APLNR,RAB27A,TCF4,FOXN2,ENSCAFG00000017103,NEDD9,IRF8,PELI1,PPP3CA,YES1,CASP8,MTOR,MAP3K3,BMP2,HHEX,CREM,SLK,CNOT6,PROX1,MADD,ARID4B,HMBOX1,PAK2,PRKD2,THOC1,SMARCA4,TLR3,NCK1,TIMELESS,OTUD4,LDB2,JAG1,NSD1,PTGS2,MMD,RAPGEF2,PLCG2,BCL10,TAOK1,CNOT7,HMGB1,AHR,PMAIP1,BLM,RIPK1,ITGA6,GPBP1,PDGFD,MTA2,NR1D2,UBE2D1,KDM5A,HIF1A,KDM3A,ATRX,NR4A1,ENSCAFG00000002015,JCAD,ZEB1,TET2,ARHGEF2,CDON,ZNF609	up_VCAV_EC
GO:0043086	0.0839	0.947	22	170	negative regulation of catalytic activity	BP	1	TNNT2,RECK,BMP4,EIF4A2,SH3RF2,LATS2,CST3,CDKN1A,SMPD1,SH3RF1,MARF1,PPP1R26,PSEN1,SPINK2,DUSP22,DUSP26,LRRC4,INPP5K,SH3BP4,PARK7,GPATCH2,ZFYVE1	up_ACOR_VSMC
GO:1902532	0.0839	0.981	3	204	negative regulation of intracellular signal	BP	1	LYN,IGF1R,ASPN	down_ACOR_EC
GO:0060193	0.0839	0.983	3	11	positive regulation of lipase activity	BP	1	HTR2B,FGFR2,LMF1	up_ACOR_VSMC
GO:0072511	0.0839	0.988	2	97	divalent inorganic cation transport	BP	1	LYN,HOXA3	down_ACOR_EC
GO:0007260	0.0839	0.997	1	19	tyrosine phosphorylation of STAT protein	BP	1	LYN	down_ACOR_EC
GO:0002840	0.0839	1	1	1	regulation of T cell mediated immune	BP	1	HMGB1	down_ACOR_VSMC
GO:0017055	0.0839	1	1	1	negative regulation of RNA polymerase II transcriptional	BP	1	HMGB1	down_ACOR_VSMC
GO:0032072	0.0839	1	1	1	regulation of restriction endodeoxyribonuclease	BP	1	HMGB1	down_ACOR_VSMC
GO:0032425	0.0839	1	1	1	positive regulation of mismatch repair	BP	1	HMGB1	down_ACOR_VSMC
GO:0002424	0.0839	1	1	1	T cell mediated immune response to tumor cell	BP	1	HMGB1	down_ACOR_VSMC
GO:2000425	0.0839	1	1	1	regulation of apoptotic cell clearance	BP	1	HMGB1	down_ACOR_VSMC
GO:2000426	0.0839	1	1	1	negative regulation of apoptotic cell clearance	BP	1	HMGB1	down_ACOR_VSMC
GO:0009791	0.083971817	0.98083	3	38	post-embryonic development	BP	1	FZD5,PYGO2,EFEMP1	up_ACOR_EC
GO:0061387	0.084	0.977	4	18	regulation of extent of cell growth	BP	1	ABL1,PLXNA4,DPYSL2,TTL	up_VSAP_EC
GO:0007566	0.084	0.989	2	10	embryo implantation	BP	1	RECK,SYDE1	down_VCAV_EC
GO:1903126	0.084	1	1	1	negative regulation of centriole-centriole	BP	1	NEK2	down_ACOR_VSMC
GO:0030856	0.084	0.997	1	41	regulation of epithelial cell differentiation	BP	1	SRSF6	down_AFEM_EC
GO:0034067	0.084	0.997	1	11	protein localization to Golgi apparatus	BP	1	GCC2	up_VPOR_EC
GO:0021555	0.084	1	1	1	midbrain-hindbrain boundary morphogenesis	BP	1	KDM2B	up_VSAP_EC
GO:0021592	0.084	1	1	1	fourth ventricle development	BP	1	KDM2B	up_VSAP_EC
GO:0021993	0.084	1	1	1	initiation of neural tube closure	BP	1	KDM2B	up_VSAP_EC
GO:0070544	0.084	1	1	1	histone H3-K36 demethylation	BP	1	KDM2B	up_VSAP_EC
GO:0007266	0.0840082	0.97524	4	64	Rho protein signal transduction	BP	1	PAK3,ARHGDIB,NRP1,ARHGEF26	up_ACOR_EC
GO:0015980	0.0841	0.953	15	106	energy derivation by oxidation of organic compounds	BP	1	DLST,C1QTNF2,COQ9,NDUFS1,ENSCAFG00000004260,SLC25A12,PHKA2,FXN,NDUFS2,PASK,MTFR1L,IDH1,IDH3A,ENSCAFG00000006639,NDUFV1	up_VSAP_EC
GO:0023052	0.0841	0.97	7	1602	signaling	BP	1	FGF5,BARX1,NKX2-5,BAMBI,ANKRD6,CYP26B1,C3AR1	up_VPOR_VSMC
GO:1902904	0.0841	0.972	5	38	negative regulation of supramolecular fiber	BP	0.941	ARHGEF2,CRYAB,TWF1,CAPG,MID1	down_VSAP_EC
GO:0050873	0.0841	0.982	3	18	brown fat cell differentiation	BP	0.941	ERO1A,PTGS2,BNIP3	down_VSAP_EC
GO:0003400	0.0841	1	1	1	regulation of COPII vesicle coating	BP	1	PREB	down_ACOR_VSMC
GO:1905661	0.0841	1	1	1	regulation of telomerase RNA reverse transcriptase	BP	1	HNRNPD	down_ACOR_VSMC

GO:1905663	0.0841	1	1	1	positive regulation of telomerase RNA reverse	BP	1	HNRNPD	down_ACOR_VSMC
GO:0090113	0.0841	1	1	1	regulation of ER to Golgi vesicle-mediated transport by GTP	BP	1	PREB	down_ACOR_VSMC
GO:0017198	0.0841	0.998	1	2	N-terminal peptidyl-serine acetylation	BP	0.941	NAA10	down_VSAP_EC
GO:0018002	0.0841	0.998	1	2	N-terminal peptidyl-glutamic acid acetylation	BP	0.941	NAA10	down_VSAP_EC
GO:0030920	0.0841	0.998	1	2	peptidyl-serine acetylation	BP	0.941	NAA10	down_VSAP_EC
GO:0050922	0.0841	0.997	1	11	negative regulation of chemotaxis	BP	1	SEMA3A	up_VPOR_EC
GO:0043281	0.0842	0.963	8	57	regulation of cysteine-type endopeptidase activity involved in	BP	1	HMGB1,BIRC5,PDCD2,DHCR24,CYCS,ENSCAFG00000005299,AIFM1,DELE1	down_ACOR_VSMC
GO:0060389	0.0842	0.997	1	21	pathway-restricted SMAD protein phosphorylation	BP	1	ACVRL1	down_ACOR_EC
GO:0097724	0.0842	1	1	1	sperm flagellum movement	BP	1	TTLL1	down_ACOR_VSMC
GO:0046900	0.0842	1	1	1	tetrahydrofolylpolyglutamate metabolic process	BP	1	GGH	down_ACOR_VSMC
GO:1905419	0.0842	1	1	1	sperm flagellum movement involved in flagellated sperm motility	BP	1	TTLL1	down_ACOR_VSMC
GO:0051270	0.0842	0.998	1	296	regulation of cellular component movement	BP	1	JUP	down_VFEM_VSMC
GO:0009144	0.0842	0.997	1	103	purine nucleoside triphosphate metabolic	BP	1	AK5	down_VSAP_VSMC
GO:0032635	0.0842	0.997	1	37	interleukin-6 production	BP	1	BPI	up_AORT_EC
GO:1903391	0.0843	0.981	3	21	regulation of adherens junction organization	BP	1	RHOD,THY1,GREM1	down_VCAV_EC
GO:0039019	0.0843	0.998	1	2	pronephric nephron development	BP	1	SEC61A1	down_VCAV_EC
GO:1900223	0.0843	0.998	1	2	positive regulation of amyloid-beta clearance	BP	1	LRP1	down_VCAV_EC
GO:1904352	0.0843	0.998	1	2	positive regulation of protein catabolic process	BP	1	LRP1	down_VCAV_EC
GO:1905167	0.0843	0.998	1	2	positive regulation of lysosomal protein	BP	1	LRP1	down_VCAV_EC
GO:0065004	0.0843	0.997	1	81	protein-DNA complex assembly	BP	1	ESR1	up_AORT_VSMC
GO:0048666	0.0844	0.958	10	258	neuron development	BP	1	IRX5,LEP,PLXNA4,NEFH,THRB,LGI4,CAMK2A,LRRN2,KANK1,TNC	up_AFEM_EC
GO:0090090	0.0844	0.981	3	44	negative regulation of canonical Wnt signaling	BP	1	GREM1,CYLD,AXIN2	up_AFEM_EC
GO:0022407	0.0844	0.988	2	96	regulation of cell-cell adhesion	BP	1	LYN,PDPN	down_ACOR_EC
GO:1903825	0.0844	0.997	1	17	organic acid transmembrane transport	BP	1	PDPN	down_ACOR_EC
GO:1905039	0.0844	0.997	1	17	carboxylic acid transmembrane transport	BP	1	PDPN	down_ACOR_EC
GO:0007130	0.0844	1	1	1	synaptonemal complex assembly	BP	1	TRIP13	down_ACOR_VSMC
GO:0070561	0.0844	0.998	1	2	vitamin D receptor signaling pathway	BP	1	KANK2	down_VCAV_EC
GO:0070562	0.0844	0.998	1	2	regulation of vitamin D receptor signaling	BP	1	KANK2	down_VCAV_EC
GO:0071305	0.0844	0.998	1	2	cellular response to vitamin D	BP	1	KANK2	down_VCAV_EC
GO:0009161	0.0844	0.997	1	103	ribonucleoside monophosphate	BP	1	AK5	down_VSAP_VSMC
GO:1903977	0.0844	1	1	1	positive regulation of glial cell migration	BP	1	CRKL	up_ACOR_VSMC
GO:2000649	0.0845	0.983	3	11	regulation of sodium ion transmembrane transporter activity	BP	1	OSR1,KLHL24,FXYD1	up_VSAP_EC
GO:0006012	0.0845	0.99	2	6	galactose metabolic process	BP	1	B4GALT1,GALE	down_ACOR_VSMC
GO:0072592	0.0845	1	1	1	oxygen metabolic process	BP	1	LIPT2	up_ACOR_VSMC
GO:2000374	0.0845	1	1	1	regulation of oxygen metabolic process	BP	1	LIPT2	up_ACOR_VSMC
GO:2000376	0.0845	1	1	1	positive regulation of oxygen metabolic process	BP	1	LIPT2	up_ACOR_VSMC
GO:0043500	0.0845	0.997	1	21	muscle adaptation	BP	1	GATM	up_VSAP_VSMC

GO:0042326	0.0846	0.948	21	163	negative regulation of phosphorylation	BP	1	SMAD6,RASSF2,BMP4,LATS2,NDRG2,CDKN2B,F2RL1,FAM129A,CDKN1A,PTPN22,SMPD1,DAG1,PSEN1,SYNJ2BP,DUSP22,DUSP26,LRRC4,DDIT4,INPP5K,PARK7,CRKL	up_ACOR_VSMC
GO:0006935	0.0846	0.95	17	143	chemotaxis	BP	0.933	EPHB1,EDNRB,KIT,CXCL8,CXCR4,DOCK4,AMOT,PDGFB,RELN,PDE4D,GATA3,MMP28,ANGPT2,PRKD2,SWAP70,FES,HMGB1	up_VCAV_EC
GO:0042330	0.0846	0.95	17	143	taxis	BP	0.933	EPHB1,EDNRB,KIT,CXCL8,CXCR4,DOCK4,AMOT,PDGFB,RELN,PDE4D,GATA3,MMP28,ANGPT2,PRKD2,SWAP70,FES,HMGB1	up_VCAV_EC
GO:0045637	0.0846	0.958	11	73	regulation of myeloid cell differentiation	BP	1	HOXA7,HOXA9,PPARGC1B,DLL1,ANKRD54,ZBTB16,RARG,ZFP36L1,PIK3R1,MYC,TFE3	up_VSAP_EC
GO:0061217	0.0846	0.982	3	13	regulation of mesonephros	BP	1	GREM1,HOXB7,SIX4	down_ACOR_VSMC
GO:0035709	0.0846	1	1	1	memory T cell activation	BP	1	FAM49B	down_ACOR_VSMC
GO:0046167	0.0846	1	1	1	glycerol-3-phosphate biosynthetic process	BP	1	GK5	down_ACOR_VSMC
GO:2000567	0.0846	1	1	1	regulation of memory T cell activation	BP	1	FAM49B	down_ACOR_VSMC
GO:2000568	0.0846	1	1	1	positive regulation of memory T cell activation	BP	1	FAM49B	down_ACOR_VSMC
GO:0006366	0.0846	1	1	583	transcription by RNA polymerase II	BP	1	GATA5	down_AFEM_VSMC
GO:0046661	0.0846	0.997	1	45	male sex differentiation	BP	1	CITED2	down_VPOR_EC
GO:0021700	0.0846	0.997	1	65	developmental maturation	BP	1	IRX5	up_AFEM_VSMC
GO:0048538	0.0846	0.997	1	20	thymus development	BP	1	SIX1	up_VFEM_EC
GO:0048747	0.0846	0.997	1	20	muscle fiber development	BP	1	SHOX2	up_VSAP_VSMC
GO:0034198	0.0847	0.976	4	27	cellular response to amino acid starvation	BP	0.945	SESN2,ATF3,EIF2S1,CDKN1A	down_VSAP_EC
GO:1990928	0.0847	0.976	4	27	response to amino acid starvation	BP	0.945	SESN2,ATF3,EIF2S1,CDKN1A	down_VSAP_EC
GO:0051716	0.0847	1	2	2001	cellular response to stimulus	BP	1	JUP,GATA5	down_VFEM_VSMC
GO:0071606	0.0847	1	1	1	chemokine (C-C motif) ligand 4 production	BP	1	ARG2	down_ACOR_VSMC
GO:0071608	0.0847	1	1	1	macrophage inflammatory protein-1	BP	1	ARG2	down_ACOR_VSMC
GO:0071640	0.0847	1	1	1	regulation of macrophage inflammatory protein 1 alpha production	BP	1	ARG2	down_ACOR_VSMC
GO:0071641	0.0847	1	1	1	negative regulation of macrophage inflammatory protein 1	BP	1	ARG2	down_ACOR_VSMC
GO:0071643	0.0847	1	1	1	regulation of chemokine (C-C motif) ligand 4	BP	1	ARG2	down_ACOR_VSMC
GO:0071644	0.0847	1	1	1	negative regulation of chemokine (C-C motif)	BP	1	ARG2	down_ACOR_VSMC
GO:1905076	0.0847	1	1	1	regulation of interleukin-17 secretion	BP	1	ARG2	down_ACOR_VSMC
GO:1905077	0.0847	1	1	1	negative regulation of interleukin-17 secretion	BP	1	ARG2	down_ACOR_VSMC
GO:1905403	0.0847	1	1	1	negative regulation of activated CD8-positive, alpha-beta T cell	BP	1	ARG2	down_ACOR_VSMC
GO:2000666	0.0847	1	1	1	negative regulation of interleukin-13 secretion	BP	1	ARG2	down_ACOR_VSMC
GO:0033633	0.0847	1	1	1	negative regulation of cell-cell adhesion mediated by	BP	0.933	SWAP70	up_VCAV_EC
GO:1902309	0.0847	1	1	1	negative regulation of peptidyl-serine	BP	0.933	SWAP70	up_VCAV_EC
GO:0034766	0.0847	0.997	1	19	negative regulation of ion transmembrane transport	BP	1	CAV1	up_VSAP_VSMC
GO:0034614	0.084728206	0.98056	3	39	cellular response to reactive oxygen species	BP	1	AQP1,GUCY1B1,PRKCD	up_ACOR_EC
GO:0018958	0.0848	0.977	4	20	phenol-containing compound metabolic	BP	0.933	GATA3,SNCAIP,RAPGEF2,GCH1	up_VCAV_EC
GO:0070601	0.0848	0.99	2	6	centromeric sister chromatid cohesion	BP	1	SGO1,SGO2	down_ACOR_VSMC
GO:0002327	0.0848	0.991	2	5	immature B cell differentiation	BP	0.933	KIT,ATP11C	up_VCAV_EC
GO:2000807	0.0848	0.998	1	2	regulation of synaptic vesicle clustering	BP	1	CDH2	down_VCAV_EC
GO:2000809	0.0848	0.998	1	2	positive regulation of synaptic vesicle clustering	BP	1	CDH2	down_VCAV_EC

GO:0000578	0.0849	0.983	3	11	embryonic axis specification	BP	1	SMAD6,FRS2,PCSK6	up_ACOR_VSMC
GO:0043412	0.085	0.931	112	1225	macromolecule modification	BP	0.933	EPHB1,PLK2,TEK,HCLS1,ST8SIA6,KIT,PPP2R5A,PRKCH,TNFAIP3,MAP3K11,TNIP1,PDGFB,STK10,SH2D3C,RELN,ST6GAL1,PDE4D,IWS1,MEF2C,ATF3,TIPARP,RAPGEF1,TLR4,GATA3,USP33,EDN1,RALB,APLNR,CTTNBP2NL,ZDHHC22,NEDD9,PELI1,YES1,ENSCAFG00000010893,EPHB4,B3GNT5,MTOR,ASB9,HHAT,MAP3K3,BMP2,CDYL,HMG20A,HHEX,PREX2,SLK,BMP2K,SH3BP5,ST3GAL6,ENC1,ABCA1,PPP1R2,ERC1,MAPK13,ITPKB,PROX1,UHRF2,ENSCAFG00000010596,MADD,ARID4B,PAK2,GALNT18,PRKD2,SWAP70,TLR3,MFNG,NCK1,ESCO1,TRMT9B,DPH5,VHL,OTUD4,WSB1,PARP4,FES,NSD1,PTGS2,MMD,TOP1,RAPGEF2,CREB1,BCL10,SENP6,USP28,TAOK1,CNOT7,HMGB1,NOS2,BLM,SPTBN4,RIPK1,USP13,USP38,PDGFD,USP37,WTAP,MTA2,C7H18orf25,CUL4A,BMX,UBE2D1,KDM5A,KDM3A,LMTK2,ATRX,ZC3H13,ZDHHC6,IVNS1ABP,JCAD,TET2,ARHGEF2,CDON	up_VCAV_EC
GO:0070059	0.085	0.972	5	25	intrinsic apoptotic signaling pathway in response to endoplasmic	BP	1	ITPR1,SERINC3,HERPUD1,WFS1,PARK7	up_ACOR_VSMC
GO:0048538	0.085	0.997	1	20	thymus development	BP	1	HOXA3	down_ACOR_EC
GO:0009992	0.085	1	1	1	cellular water homeostasis	BP	1	ANXA7	down_ACOR_VSMC
GO:0032361	0.085	1	1	1	pyridoxal phosphate catabolic process	BP	1	PDXP	down_ACOR_VSMC
GO:0042365	0.085	1	1	1	water-soluble vitamin catabolic process	BP	1	PDXP	down_ACOR_VSMC
GO:0042820	0.085	1	1	1	vitamin B6 catabolic process	BP	1	PDXP	down_ACOR_VSMC
GO:0071579	0.085	1	1	1	regulation of zinc ion transport	BP	1	SLC30A1	down_ACOR_VSMC
GO:0071580	0.085	1	1	1	regulation of zinc ion transmembrane transport	BP	1	SLC30A1	down_ACOR_VSMC
GO:0071581	0.085	1	1	1	regulation of zinc ion transmembrane import	BP	1	SLC30A1	down_ACOR_VSMC
GO:0071582	0.085	1	1	1	negative regulation of zinc ion transport	BP	1	SLC30A1	down_ACOR_VSMC
GO:0071583	0.085	1	1	1	negative regulation of zinc ion transmembrane	BP	1	SLC30A1	down_ACOR_VSMC
GO:0071584	0.085	1	1	1	negative regulation of zinc ion transmembrane	BP	1	SLC30A1	down_ACOR_VSMC
GO:0090281	0.085	1	1	1	negative regulation of calcium ion import	BP	1	SLC30A1	down_ACOR_VSMC
GO:1902298	0.085	1	1	1	cell cycle DNA replication maintenance of fidelity	BP	1	RAD51	down_ACOR_VSMC
GO:1904026	0.085	1	1	1	regulation of collagen fibril organization	BP	1	COLGALT1	down_ACOR_VSMC
GO:1904028	0.085	1	1	1	positive regulation of collagen fibril	BP	1	COLGALT1	down_ACOR_VSMC
GO:1990426	0.085	1	1	1	mitotic recombination-dependent replication	BP	1	RAD51	down_ACOR_VSMC
GO:1990505	0.085	1	1	1	mitotic DNA replication maintenance of fidelity	BP	1	RAD51	down_ACOR_VSMC
GO:0005988	0.085	1	1	1	lactose metabolic process	BP	1	B4GALT1	down_ACOR_VSMC
GO:0005989	0.085	1	1	1	lactose biosynthetic process	BP	1	B4GALT1	down_ACOR_VSMC
GO:0016480	0.085	1	1	1	negative regulation of transcription by RNA polymerase III	BP	1	NAB2	down_ACOR_VSMC
GO:0031247	0.085	1	1	1	actin rod assembly	BP	1	PDXP	down_ACOR_VSMC
GO:0045578	0.085	1	1	1	negative regulation of B cell differentiation	BP	1	HMGB3	down_ACOR_VSMC
GO:0060057	0.085	1	1	1	apoptotic process involved in mammary	BP	1	B4GALT1	down_ACOR_VSMC
GO:0060058	0.085	1	1	1	positive regulation of apoptotic process involved in mammary	BP	1	B4GALT1	down_ACOR_VSMC
GO:0061386	0.085	1	1	1	closure of optic fissure	BP	1	PHACTR4	down_ACOR_VSMC
GO:0070084	0.085	1	1	1	protein initiator methionine removal	BP	1	METAP2	down_ACOR_VSMC

GO:1903519	0.085	1	1	1	regulation of mammary gland involution	BP	1	B4GALT1	down_ACOR_VSMC
GO:1903521	0.085	1	1	1	positive regulation of mammary gland	BP	1	B4GALT1	down_ACOR_VSMC
GO:2001247	0.085	1	1	1	positive regulation of phosphatidylcholine biosynthetic process	BP	1	ACSL3	down_ACOR_VSMC
GO:1902904	0.085	0.997	1	38	negative regulation of supramolecular fiber	BP	1	TAOK1	up_AORT_EC
GO:1903393	0.085	0.997	1	11	positive regulation of adherens junction	BP	1	ENSCAFG00000002015	up_VPOR_EC
GO:0031929	0.0851	0.959	10	62	TOR signaling	BP	1	LEP,PRKAA2,CASTOR1,UBR1,SESN3,SESN1,DDIT4,DISC1,BMT2,SH3BP4	up_ACOR_VSMC
GO:0006633	0.0851	0.968	6	38	fatty acid biosynthetic process	BP	1	SCD,INSIG1,BRCA1,ELOVL1,ELOVL6,LIPC	down_ACOR_VSMC
GO:0090316	0.0851	0.971	5	48	positive regulation of intracellular protein	BP	1	PRNP,ENSCAFG00000014978,AKT2,GLI3,MAVS	down_VCAV_EC
GO:0032675	0.0851	0.975	4	34	regulation of interleukin-6 production	BP	1	BPI,CYBA,MAVS,RAB7B	down_VCAV_EC
GO:0003229	0.0851	0.977	4	17	ventricular cardiac muscle tissue development	BP	1	TNNT2,FGFR2,TBX5,CHD7	up_ACOR_VSMC
GO:0044091	0.0851	0.989	2	10	membrane biogenesis	BP	1	CLIP3,CDH2	down_VCAV_EC
GO:0046823	0.0851	0.99	2	6	negative regulation of nucleocytoplasmic	BP	1	FERMT1,PARK7	up_ACOR_VSMC
GO:0032796	0.0851	1	1	1	uropod organization	BP	1	CORO1A	down_ACOR_VSMC
GO:0009123	0.0851	0.997	1	104	nucleoside monophosphate	BP	1	AK5	down_VSAP_VSMC
GO:0060193	0.0851	0.997	1	11	positive regulation of lipase activity	BP	1	ABHD5	up_VPOR_EC
GO:0042136	0.0851	0.997	1	21	neurotransmitter biosynthetic process	BP	1	CAV1	up_VSAP_VSMC
GO:0044403	0.085139747	0.96698	6	116	symbiont process	BP	1	ATG16L2,AQP1,TRIM8,MAVS,LRRC15,CHMP4B	up_ACOR_EC
GO:2001233	0.0852	0.95	17	149	regulation of apoptotic signaling pathway	BP	0.933	TNFAIP3,ATF3,FIGNL1,MADD,PAK2,SH3GLB1,NCK1,PTGS2,BCL10,CTNNA1,PMAIP1,RIPK1,ITGA6,SKIL,HIF1A,IVNS1ABP,ARHGEF2	up_VCAV_EC
GO:0042107	0.0852	0.976	4	22	cytokine metabolic process	BP	0.933	TLR4,GATA3,TLR3,BCL10	up_VCAV_EC
GO:1903829	0.0852	0.988	2	91	positive regulation of cellular protein	BP	1	ACSL3,STX4	down_ACOR_EC
GO:0032954	0.0852	1	1	1	regulation of cytokinetic process	BP	1	NUP62	down_ACOR_VSMC
GO:1902412	0.0852	1	1	1	regulation of mitotic cytokinesis	BP	1	NUP62	down_ACOR_VSMC
GO:1903438	0.0852	1	1	1	positive regulation of mitotic cytokinetic	BP	1	NUP62	down_ACOR_VSMC
GO:0021816	0.0852	1	1	1	extension of a leading process involved in cell motility in cerebral cortex radial glia guided	BP	1	ENSCAFG00000010224	down_ACOR_VSMC
GO:1903436	0.0852	1	1	1	regulation of mitotic cytokinetic process	BP	1	NUP62	down_ACOR_VSMC
GO:1903490	0.0852	1	1	1	positive regulation of mitotic cytokinesis	BP	1	NUP62	down_ACOR_VSMC
GO:0048584	0.0852	1	1	592	positive regulation of response to stimulus	BP	1	GATA5	down_AFEM_VSMC
GO:0032414	0.0852	0.997	1	21	positive regulation of ion transmembrane transporter activity	BP	1	STIM1	up_VSAP_VSMC
GO:0034762	0.0853	0.958	10	122	regulation of transmembrane transport	BP	1	PRNP,FXYD1,CLIP3,ENSCAFG00000014978,AKT2,CACNB3,THY1,PARK7,CYBA,CLIC4	down_VCAV_EC
GO:1902600	0.0853	0.972	5	31	proton transmembrane transport	BP	1	NNT,CYC1,ATP5F1A,ENSCAFG00000013366,ATP5F1D	down_ACOR_VSMC
GO:0002902	0.0853	0.99	2	6	regulation of B cell apoptotic process	BP	1	AURKB,NOC2L	down_ACOR_VSMC
GO:0030097	0.0853	0.988	2	244	hemopoiesis	BP	1	TMEM176A,TMEM176B	up_VPUL_VSMC
GO:0006696	0.0853	1	1	1	ergosterol biosynthetic process	BP	1	FDFT1	down_ACOR_VSMC
GO:0044107	0.0853	1	1	1	cellular alcohol metabolic process	BP	1	FDFT1	down_ACOR_VSMC
GO:0061519	0.0853	1	1	1	macrophage homeostasis	BP	1	BAP1	down_ACOR_VSMC
GO:0008204	0.0853	1	1	1	ergosterol metabolic process	BP	1	FDFT1	down_ACOR_VSMC
GO:0044108	0.0853	1	1	1	cellular alcohol biosynthetic process	BP	1	FDFT1	down_ACOR_VSMC

GO:0045338	0.0853	1	1	1	farnesyl diphosphate metabolic process	BP	1	FDFT1	down_ACOR_VSMC
GO:0002764	0.0853	0.997	1	80	immune response-regulating signaling	BP	1	ESR1	up_AORT_VSMC
GO:0034765	0.0854	0.962	8	91	regulation of ion transmembrane transport	BP	1	PRNP,FXYD1,AKT2,CACNB3,THY1,PARK7,CYBA,CLIC4	down_VCAV_EC
GO:0061572	0.0854	0.968	6	50	actin filament bundle organization	BP	0.951	ACTN1,FSCN1,DPYSL3,PLS3,NRP1,TNFAIP1	down_VSAP_EC
GO:0060563	0.0854	0.977	4	18	neuroepithelial cell differentiation	BP	1	WNT11,ABL1,DLL1,DLX3	up_VSAP_EC
GO:0042474	0.0854	0.983	3	11	middle ear morphogenesis	BP	1	OSR2,INSIG2,OSR1	up_VSAP_EC
GO:0090659	0.0854	0.982	3	12	walking behavior	BP	1	FXN,CTNS,HTRA2	up_VSAP_EC
GO:0032070	0.0854	0.99	2	6	regulation of deoxyribonuclease	BP	1	HMGB1,PCNA	down_ACOR_VSMC
GO:1901360	0.0854	1	2	2016	organic cyclic compound metabolic process	BP	1	JUP,GATA5	down_VFEM_VSMC
GO:0034312	0.0854	0.989	2	8	diol biosynthetic process	BP	0.933	SPTLC2,GCH1	up_VCAV_EC
GO:0021691	0.0854	1	1	1	cerebellar Purkinje cell layer maturation	BP	1	ARCN1	down_ACOR_VSMC
GO:0035239	0.0854	0.998	1	299	tube morphogenesis	BP	1	JUP	down_VFEM_VSMC
GO:1990569	0.0854	1	1	1	UDP-N-acetylglucosamine transmembrane transport	BP	0.933	SLC35A3	up_VCAV_EC
GO:0031111	0.0854	0.997	1	11	negative regulation of microtubule polymerization or	BP	1	ARHGEF2	up_VPOR_EC
GO:0050770	0.0854	0.997	1	39	regulation of axonogenesis	BP	1	SHOX2	up_VFEM_VSMC
GO:0008064	0.0855	0.959	10	72	regulation of actin polymerization or	BP	0.933	HCLS1,BAIAP2L1,TMOD1,MTOR,NCKAP1,IQGAP2,SWAP70,NCK1,ARPC5L,TMOD3	up_VCAV_EC
GO:0030832	0.0855	0.959	10	72	regulation of actin filament length	BP	0.933	HCLS1,BAIAP2L1,TMOD1,MTOR,NCKAP1,IQGAP2,SWAP70,NCK1,ARPC5L,TMOD3	up_VCAV_EC
GO:0016239	0.0855	0.976	4	20	positive regulation of macroautophagy	BP	0.933	RALB,SPTLC2,SH3GLB1,HIF1A	up_VCAV_EC
GO:0040012	0.0855	0.981	3	303	regulation of locomotion	BP	1	TBX5,CCL24,ARHGEF2	up_VPUL_EC
GO:0051447	0.0855	0.99	2	6	negative regulation of meiotic cell cycle	BP	1	FBXO5,TTK	down_ACOR_VSMC
GO:0016558	0.0855	0.989	2	10	protein import into peroxisome matrix	BP	1	PEX5,LONP2	down_VCAV_EC
GO:0035986	0.0855	1	1	1	senescence-associated heterochromatin focus	BP	1	HMGA1	down_ACOR_VSMC
GO:0090402	0.0855	1	1	1	oncogene-induced cell senescence	BP	1	HMGA1	down_ACOR_VSMC
GO:0009064	0.0855	0.997	1	23	glutamine family amino acid metabolic process	BP	1	NR1H4	up_APUL_EC
GO:1902042	0.0855	0.997	1	11	negative regulation of extrinsic apoptotic signaling pathway via	BP	1	ARHGEF2	up_VPOR_EC
GO:2001169	0.0855	0.997	1	11	regulation of ATP biosynthetic process	BP	1	FBP1	up_VPOR_EC
GO:0003156	0.0855	0.997	1	20	regulation of animal organ formation	BP	1	HOXA11	up_VSAP_VSMC
GO:0051272	0.0856	0.946	22	170	positive regulation of cellular component movement	BP	1	TWIST2,WNT11,FAM107A,ENPP2,FGF7,ABL1,HDAC7,PDGFD,CCBE1,STAT5A,F2RL1,DDR2,PIK3R1,TIAM1,CFAP69,SASH1,ZNF580,STX4,HMOX1,MAP2K2,TGFBR2,BAG4	up_VSAP_EC
GO:0033500	0.0856	0.959	10	64	carbohydrate homeostasis	BP	1	HNF4A,SLC8B1,RAB11B,PDK2,PIK3R1,KCNB1,RAF1,STX4,PPP3CB,RAB11FIP2	up_VSAP_EC
GO:0042593	0.0856	0.959	10	64	glucose homeostasis	BP	1	HNF4A,SLC8B1,RAB11B,PDK2,PIK3R1,KCNB1,RAF1,STX4,PPP3CB,RAB11FIP2	up_VSAP_EC
GO:0043406	0.0856	0.96	9	62	positive regulation of MAP kinase activity	BP	0.933	KIT,MAP3K11,PDGFB,TLR4,EDN1,BMP2,MADD,RIPK1,PDGFD	up_VCAV_EC
GO:0019322	0.0856	1	1	1	pentose biosynthetic process	BP	1	PGD	down_ACOR_VSMC
GO:0009628	0.0856	0.998	1	302	response to abiotic stimulus	BP	1	JUP	down_VFEM_VSMC
GO:0071559	0.0856	0.997	1	75	response to transforming growth factor beta	BP	1	WNT7A	up_AORT_VSMC
GO:0021846	0.0856	0.997	1	11	cell proliferation in forebrain	BP	1	HOOK3	up_VPOR_EC
GO:0051494	0.0856	0.997	1	44	negative regulation of cytoskeleton organization	BP	1	SLIT2	up_VPUL_VSMC
GO:0090288	0.0856	0.997	1	44	negative regulation of cellular response to growth factor stimulus	BP	1	SLIT2	up_VPUL_VSMC

GO:0008584	0.085699529	0.98025	3	39	male gonad development	BP	1	GATA4,SOX9,LHX9	up_ACOR_EC
GO:0046546	0.085699529	0.98025	3	39	development of primary male sexual	BP	1	GATA4,SOX9,LHX9	up_ACOR_EC
GO:0090288	0.0857	0.98	3	44	negative regulation of cellular response to growth factor stimulus	BP	1	GREM1,GREM2,ADAM17	up_AFEM_EC
GO:0046636	0.0857	0.989	2	10	negative regulation of alpha-beta T cell	BP	1	GLI3,LOXL3	down_VCAV_EC
GO:0010613	0.0857	0.991	2	5	positive regulation of cardiac muscle	BP	1	PDE5A,EDN1	up_ACOR_VSMC
GO:0014742	0.0857	0.991	2	5	positive regulation of muscle hypertrophy	BP	1	PDE5A,EDN1	up_ACOR_VSMC
GO:0002155	0.0857	1	1	1	regulation of thyroid hormone mediated	BP	0.933	PRCP	up_VCAV_EC
GO:0002254	0.0857	1	1	1	kinin cascade	BP	0.933	PRCP	up_VCAV_EC
GO:0002353	0.0857	1	1	1	plasma kallikrein-kinin cascade	BP	0.933	PRCP	up_VCAV_EC
GO:0090207	0.0857	0.997	1	11	regulation of triglyceride metabolic process	BP	1	ABHD5	up_VPOR_EC
GO:0016052	0.0857	0.997	1	34	carbohydrate catabolic process	BP	1	FBP1	up_VFEM_VSMC
GO:0010464	0.0857	0.997	1	21	regulation of mesenchymal cell	BP	1	SHOX2	up_VSAP_VSMC
GO:2000576	0.085730656	0.99716	1	4	positive regulation of microtubule motor	BP	1	DYNC1I1	up_ACOR_EC
GO:2000580	0.085730656	0.99716	1	4	regulation of ATP-dependent microtubule motor activity, plus-end-	BP	1	DYNC1I1	up_ACOR_EC
GO:2000582	0.085730656	0.99716	1	4	positive regulation of ATP-dependent microtubule motor activity, plus-end-	BP	1	DYNC1I1	up_ACOR_EC
GO:0019438	0.0858	0.961	10	1258	aromatic compound biosynthetic process	BP	1	ACVRL1,YEATS2,YBX3,HIVEP2,HOXA7,SULF1,ELK3,HOXA1,TNRC6B,NEDD4	down_ACOR_EC
GO:0048706	0.0858	0.987	2	59	embryonic skeletal system development	BP	1	HOXA3,ACVR2A	up_VPOR_EC
GO:0048732	0.0858	0.987	2	116	gland development	BP	1	HOXA9,SIX1	up_VFEM_EC
GO:0032269	0.0858	0.998	1	306	negative regulation of cellular protein metabolic	BP	1	CST3	down_AORT_EC
GO:0038167	0.0858	1	1	1	epidermal growth factor receptor signaling pathway via positive regulation of NF-kappaB	BP	1	RASSF2	up_ACOR_VSMC
GO:0038168	0.0858	1	1	1	epidermal growth factor receptor signaling pathway via I-kappaB	BP	1	RASSF2	up_ACOR_VSMC
GO:0045738	0.0858	0.997	1	12	negative regulation of DNA repair	BP	1	CXHXorf57	up_VPOR_EC
GO:0010648	0.0859	0.937	48	407	negative regulation of cell communication	BP	1	SMAD6,LEP,LBH,HTR2B,RASSF2,PRKAA2,MLLT3,FERMT1,BMP4,ELF1,CASTOR1,LATS2,ZNRF3,AR,PTGIR,NDRG2,ITPR1,F2RL1,DAPK1,CLSTN1,CREBRF,NRP1,UBR1,PTPN22,UNC5B,SMPD1,APLP1,TBX20,SESN3,SH3RF1,CCDC3,RGS2,DAG1,PSEN1,SYNJ2BP,WWC2,DUSP22,SESN1,DUSP26,LRRC4,DDIT4,HERPUD1,INPP5K,BMT2,PFKL,WFS1,SH3BP4,PARK7	up_ACOR_VSMC
GO:0048634	0.0859	0.965	7	40	regulation of muscle organ development	BP	1	BMP4,FGFR2,GJA1,TBX20,TBX5,FRS2,EDN1	up_ACOR_VSMC
GO:0009201	0.0859	0.971	5	41	ribonucleoside triphosphate biosynthetic	BP	0.956	PGK1,TPI1,PGAM1,HIF1A,ALDOC	down_VSAP_EC
GO:0051147	0.0859	0.98	3	44	regulation of muscle cell differentiation	BP	1	DLL1,GREM1,DMPK	up_AFEM_EC
GO:0003156	0.0859	0.996	1	20	regulation of animal organ formation	BP	1	SULF1	down_ACOR_EC
GO:0040018	0.086	0.983	3	11	positive regulation of multicellular organism	BP	0.933	CHD7,CREB1,SPTBN4	up_VCAV_EC
GO:0046320	0.086	0.989	2	10	regulation of fatty acid oxidation	BP	1	AKT2,LONP2	down_VCAV_EC
GO:0010575	0.086	0.989	2	8	positive regulation of vascular endothelial growth factor production	BP	0.957	SULF1,HIF1A	down_VSAP_EC
GO:0014010	0.086	1	1	1	Schwann cell proliferation	BP	1	NF2	down_ACOR_VSMC
GO:0043535	0.086	0.997	1	45	regulation of blood vessel endothelial cell migration	BP	1	HDAC7	down_VPOR_EC
GO:0043648	0.086	0.996	1	26	dicarboxylic acid metabolic process	BP	1	NR1H4	up_VPUL_EC

GO:0071326	0.086	0.996	1	26	cellular response to monosaccharide stimulus	BP	1	NR1H4	up_VPUL_EC
GO:0071331	0.086	0.996	1	26	cellular response to hexose stimulus	BP	1	NR1H4	up_VPUL_EC
GO:0010563	0.086081228	0.95915	9	203	negative regulation of phosphorus metabolic	BP	1	PKIB,NDRG2,DUSP26,SMAD6,LRRC15,LRRC4,PRKCD,PPP1R26,DBNDD2	up_ACOR_EC
GO:0045936	0.086081228	0.95915	9	203	negative regulation of phosphate metabolic	BP	1	PKIB,NDRG2,DUSP26,SMAD6,LRRC15,LRRC4,PRKCD,PPP1R26,DBNDD2	up_ACOR_EC
GO:0051271	0.0861	0.953	14	98	negative regulation of cellular component movement	BP	1	KCNE3,TCAF1,RECK,TP53INP1,NRP1,EVL,SPINT2,TBX5,DAG1,SEMA6D,SYNJ2BP,JUP,DUSP22,WASL	up_ACOR_VSMC
GO:0018272	0.0861	1	1	1	protein-pyridoxal-5-phosphate linkage via peptidyl-N6-pyridoxal	BP	1	CTH	down_ACOR_VSMC
GO:0018352	0.0861	1	1	1	protein-pyridoxal-5-phosphate linkage	BP	1	CTH	down_ACOR_VSMC
GO:0019343	0.0861	1	1	1	cysteine biosynthetic process via cystathionine	BP	1	CTH	down_ACOR_VSMC
GO:0019344	0.0861	1	1	1	cysteine biosynthetic process	BP	1	CTH	down_ACOR_VSMC
GO:0019346	0.0861	1	1	1	transsulfuration	BP	1	CTH	down_ACOR_VSMC
GO:0035887	0.0861	1	1	1	aortic smooth muscle cell differentiation	BP	1	CTH	down_ACOR_VSMC
GO:0071266	0.0861	1	1	1	'de novo' L-methionine biosynthetic process	BP	1	CTH	down_ACOR_VSMC
GO:1904829	0.0861	1	1	1	regulation of aortic smooth muscle cell	BP	1	CTH	down_ACOR_VSMC
GO:1904831	0.0861	1	1	1	positive regulation of aortic smooth muscle cell	BP	1	CTH	down_ACOR_VSMC
GO:0009092	0.0861	1	1	1	homoserine metabolic process	BP	1	CTH	down_ACOR_VSMC
GO:0044524	0.0861	1	1	1	protein sulfhydration	BP	1	CTH	down_ACOR_VSMC
GO:0006537	0.0861	0.998	1	2	glutamate biosynthetic process	BP	1	GLS2	down_VCAV_EC
GO:0010769	0.0861	0.997	1	69	regulation of cell morphogenesis involved	BP	1	WNT7A	up_AORT_VSMC
GO:0008063	0.0861	1	1	1	Toll signaling pathway	BP	0.933	PELI1	up_VCAV_EC
GO:0097298	0.0861	1	1	1	regulation of nucleus size	BP	0.933	HP1BP3	up_VCAV_EC
GO:0050921	0.0861	0.996	1	39	positive regulation of chemotaxis	BP	1	C3AR1	up_VPOR_VSMC
GO:0032088	0.0861	0.996	1	26	negative regulation of NF-kappaB transcription	BP	1	NR1H4	up_VPUL_EC
GO:0018130	0.0862	0.961	10	1261	heterocycle biosynthetic process	BP	1	ACVRL1,YEATS2,YBX3,HIVEP2,HOXA7,SULF1,ELK3,HOXA1,TNRC6B,NEDD4	down_ACOR_EC
GO:0033628	0.0862	0.982	3	14	regulation of cell adhesion mediated by integrin	BP	0.933	PODXL,FERMT1,SWAP70	up_VCAV_EC
GO:0098581	0.0862	0.991	2	5	detection of external biotic stimulus	BP	1	TLR1,NOD2	up_ACOR_VSMC
GO:0048667	0.0862	0.987	2	140	cell morphogenesis involved in neuron	BP	1	FLRT3,NEO1	up_VPUL_EC
GO:0055081	0.0862	0.996	1	17	anion homeostasis	BP	1	HOXA3	down_ACOR_EC
GO:0050679	0.0862	0.997	1	62	positive regulation of epithelial cell proliferation	BP	1	EYA1	up_AFEM_VSMC
GO:0003151	0.0862	0.996	1	34	outflow tract morphogenesis	BP	1	NKX2-5	up_VPOR_VSMC
GO:0046323	0.0862	0.996	1	26	glucose import	BP	1	NR1H4	up_VPUL_EC
GO:0010648	0.0863	0.938	42	407	negative regulation of cell communication	BP	0.933	CXCL8,TNFAIP3,TNIP1,PDE4D,FERMT1,ATF3,DLL4,RAPGEF1,TLR4,NLRC5,GATA3,APLNR,ARAP3,HOMER3,EGFL7,FIGNL1,CASP8,MTOR,BMP2,HHEX,PREX2,GDPD5,RIOK3,STMN1,MCC,SMARCA4,APOD,NCK1,OTUD4,PTGS2,CTDSPL2,CTNNA1,CNOT7,RIPK1,ITGA6,RGS2,SKIL,HIF1A,TNFAIP1,IVNS1ABP,ARHGEF2,PEAR1	up_VCAV_EC
GO:0048709	0.0863	0.981	3	17	oligodendrocyte differentiation	BP	0.958	SLC45A3,EIF2B1,CTNNB1	down_VSAP_EC
GO:0097028	0.0863	0.982	3	11	dendritic cell differentiation	BP	1	F2RL1,BATF2,TGFBR2	up_VSAP_EC
GO:0048661	0.0863	0.996	1	17	positive regulation of smooth muscle cell	BP	1	SULF1	down_ACOR_EC
GO:0040012	0.0863	0.998	1	303	regulation of locomotion	BP	1	JUP	down_VFEM_VSMC
GO:2000346	0.0863	1	1	1	negative regulation of hepatocyte proliferation	BP	1	LIMS2	up_ACOR_VSMC

GO:0006865	0.0863	0.996	1	23	amino acid transport	BP	1	NTRK2	up_APUL_EC
GO:0010464	0.0863	0.996	1	21	regulation of mesenchymal cell	BP	1	SIX1	up_VFEM_EC
GO:0007033	0.0864	0.955	12	82	vacuole organization	BP	1	FEZ1,ABCA1,TP53INP1,GABARAPL1,PSEN1,PIK3C3,CLN8,AKTIP,ATG16L2,GAA,FEZ2,ACP2	up_ACOR_VSMC
GO:0046637	0.0864	0.976	4	18	regulation of alpha-beta T cell differentiation	BP	1	ZBTB16,CD83,PNP,TGFBR2	up_VSAP_EC
GO:0045776	0.0864	0.99	2	6	negative regulation of blood pressure	BP	0.933	PRCP,NOS2	up_VCAV_EC
GO:0030282	0.0864	0.996	1	37	bone mineralization	BP	1	HOXA3	up_VPOR_VSMC
GO:0048588	0.0864	0.996	1	44	developmental cell growth	BP	1	SLIT2	up_VPUL_VSMC
GO:1903900	0.086450791	0.97998	3	40	regulation of viral life cycle	BP	1	TRIM8,MAVS,CHMP4B	up_ACOR_EC
GO:0009187	0.0865	0.983	3	10	cyclic nucleotide metabolic process	BP	0.933	NPR1,GUCY1A1,GUCY1B1	up_VCAV_EC
GO:0014037	0.0865	0.988	2	10	Schwann cell differentiation	BP	1	NAB2,AKT2	down_VCAV_EC
GO:0032924	0.0865	0.989	2	9	activin receptor signaling pathway	BP	0.96	TGFBR1,INHBA	down_VSAP_EC
GO:1901097	0.0865	0.998	1	2	negative regulation of autophagosome	BP	1	CLEC16A	down_VCAV_EC
GO:0045580	0.0865	0.996	1	35	regulation of T cell differentiation	BP	1	CYP26B1	up_VPOR_VSMC
GO:0042310	0.0865	0.996	1	21	vasoconstriction	BP	1	AVPR1A	up_VFEM_EC
GO:0097756	0.0865	0.996	1	21	negative regulation of blood vessel diameter	BP	1	AVPR1A	up_VFEM_EC
GO:0010996	0.086569656	0.99741	1	3	response to auditory stimulus	BP	1	USP53	up_ACOR_EC
GO:0030334	0.0866	0.94	33	267	regulation of cell migration	BP	1	VEGFD,LGR6,TCAF1,RECK,BMP4,SH3RF2,TP53INP1,SASH1,LMO4,FGF18,F2RL1,NRP1,PTPN22,EVL,TBX5,PRKCE,DAG1,APP,LAMA3,SEMA6D,SYNJ2BP,JUP,CCL5,DDR2,DUSP22,EDN1,FAM107A,JAM3,WASL,DOCK5,RREB1,CRKL,PIK3C2A	up_ACOR_VSMC
GO:0045597	0.0866	0.946	21	253	positive regulation of cell differentiation	BP	0.96	ARHGEF2,SERPINE1,SOCS5,DPYSL3,ILK,PTGS2,HIF1A,EVI2B,CTNNB1,ENSCAFG00000002015,APOE,KLF5,LYN,MMD,VLDLR,REST,PRMT1,DISC1,INHBA,HIF1AN,NRP1	down_VSAP_EC
GO:0060255	0.0866	0.958	13	1782	regulation of macromolecule metabolic process	BP	1	ACVRL1,YEATS2,LYN,IGF1R,YBX3,HIVEP2,HOXA7,PRKAR2A,SULF1,ELK3,HOXA1,ASPN,NEDD4	down_ACOR_EC
GO:0034330	0.0866	0.956	11	80	cell junction organization	BP	0.933	TEK,PRKCH,OCLN,RAPGEF1,TJP1,SLK,APOD,RAPGEF2,CTNNA1,ENSCAFG00000002015,FSCN1	up_VCAV_EC
GO:0014015	0.0866	0.982	3	11	positive regulation of gliogenesis	BP	1	ZNF365,APP,CRKL	up_ACOR_VSMC
GO:0007265	0.0866	0.987	2	121	Ras protein signal transduction	BP	1	TIAM1,CDC42EP5	up_VFEM_EC
GO:0002155	0.0866	1	1	1	regulation of thyroid hormone mediated	BP	1	PRCP	down_ACOR_VSMC
GO:0002353	0.0866	1	1	1	plasma kallikrein-kinin cascade	BP	1	PRCP	down_ACOR_VSMC
GO:0002254	0.0866	1	1	1	kinin cascade	BP	1	PRCP	down_ACOR_VSMC
GO:0051145	0.0866	0.996	1	21	smooth muscle cell differentiation	BP	1	SIX1	up_VFEM_EC
GO:0110110	0.0866	0.996	1	34	positive regulation of animal organ	BP	1	HOXA11	up_VFEM_VSMC
GO:1901879	0.0866	0.996	1	26	regulation of protein depolymerization	BP	1	ARHGEF2	up_VPUL_EC
GO:0098742	0.0866	0.996	1	44	cell-cell adhesion via plasma-membrane	BP	1	GATA5	up_VPUL_VSMC
GO:0060041	0.0867	0.965	7	41	retina development in camera-type eye	BP	1	THRB,CDON,IRX5,DLL1,RARG,CLCN2,ACVRL1	up_VSAP_EC
GO:0010611	0.0867	0.982	3	11	regulation of cardiac muscle hypertrophy	BP	1	GATA5,PDE5A,EDN1	up_ACOR_VSMC
GO:0014743	0.0867	0.982	3	11	regulation of muscle hypertrophy	BP	1	GATA5,PDE5A,EDN1	up_ACOR_VSMC
GO:0050708	0.0867	0.987	2	101	regulation of protein secretion	BP	1	LYN,STX4	down_ACOR_EC
GO:0009744	0.0867	1	1	1	response to sucrose	BP	0.933	CALCRL	up_VCAV_EC
GO:0034285	0.0867	1	1	1	response to disaccharide	BP	0.933	CALCRL	up_VCAV_EC

GO:0071324	0.0867	1	1	1	cellular response to disaccharide stimulus	BP	0.933	CALCRL	up_VCAV_EC
GO:0071329	0.0867	1	1	1	cellular response to sucrose stimulus	BP	0.933	CALCRL	up_VCAV_EC
GO:0097646	0.0867	1	1	1	calcitonin family receptor signaling pathway	BP	0.933	CALCRL	up_VCAV_EC
GO:1990408	0.0867	1	1	1	calcitonin gene-related peptide receptor signaling	BP	0.933	CALCRL	up_VCAV_EC
GO:1990410	0.0867	1	1	1	adrenomedullin receptor signaling pathway	BP	0.933	CALCRL	up_VCAV_EC
GO:0051963	0.0867	0.996	1	26	regulation of synapse assembly	BP	1	FLRT3	up_VPUL_EC
GO:0032210	0.0868	0.976	4	20	regulation of telomere maintenance via	BP	1	AURKB,NEK2,TINF2,CCT4	down_ACOR_VSMC
GO:1904356	0.0868	0.976	4	20	regulation of telomere maintenance via telomere lengthening	BP	1	AURKB,NEK2,TINF2,CCT4	down_ACOR_VSMC
GO:0055081	0.0868	0.981	3	17	anion homeostasis	BP	0.961	NR1H4,ENPP1,APOE	down_VSAP_EC
GO:0007339	0.0868	0.99	2	6	binding of sperm to zona pellucida	BP	1	B4GALT1,CCT4	down_ACOR_VSMC
GO:0035036	0.0868	0.99	2	6	sperm-egg recognition	BP	1	B4GALT1,CCT4	down_ACOR_VSMC
GO:0035455	0.0868	0.99	2	6	response to interferon-alpha	BP	0.933	MX2,GATA3	up_VCAV_EC
GO:0071621	0.0868	0.996	1	27	granulocyte chemotaxis	BP	1	CCL24	up_VPUL_EC
GO:0007155	0.0869	0.938	40	324	cell adhesion	BP	1	SMAD6,ACTN2,LEP,GATA5,CD83,MBP,FERMT1,BMP4,PDE5A,CITED2,PKP4,CD274,TINAGL1,LIMS2,NRP1,PTPN22,NPNT,SPINT2,VCAM1,PRKCE,RND1,ECM2,LAMA3,PSEN1,JUP,CDH18,PERP,DUSP22,NID1,DUSP26,FAM107A,JAM3,IGFBP7,CELSR2,ATP2C1,DOCK5,BVES,RREB1,PAG1,CRKL	up_ACOR_VSMC
GO:0009605	0.0869	0.948	18	538	response to external stimulus	BP	1	CMKLR1,LEP,CRY2,CD40,PLXNA4,BPI,GPAM,CHRM2,SLC17A5,CLEC16A,POSTN,TBXA2R,ACKR4,ACACB,NAPEPLD,PPARGC1A,CCL5,ADAM17	up_AFEM_EC
GO:0030224	0.0869	0.976	4	18	monocyte differentiation	BP	1	HOXA7,ZFP36L1,MYC,PIR	up_VSAP_EC
GO:1903131	0.0869	0.976	4	18	mononuclear cell differentiation	BP	1	HOXA7,ZFP36L1,MYC,PIR	up_VSAP_EC
GO:0006623	0.0869	0.996	1	16	protein targeting to vacuole	BP	1	NEDD4	down_ACOR_EC
GO:1900041	0.0869	1	1	1	negative regulation of interleukin-2 secretion	BP	1	EZR	down_ACOR_VSMC
GO:0032640	0.0869	0.996	1	39	tumor necrosis factor production	BP	1	BPI	up_AORT_EC
GO:0001823	0.0869	0.996	1	45	mesonephros development	BP	1	SLIT2	up_VPUL_VSMC
GO:0032474	0.0869	1	1	1	otolith morphogenesis	BP	1	LRIG3	up_VSAP_EC
GO:0046426	0.0869	0.996	1	20	negative regulation of JAK-STAT cascade	BP	1	CAV1	up_VSAP_VSMC
GO:1904893	0.0869	0.996	1	20	negative regulation of STAT cascade	BP	1	CAV1	up_VSAP_VSMC
GO:0000288	0.087	0.971	5	25	nuclear-transcribed mRNA catabolic process, deadenylation-dependent	BP	1	EXOSC7,ZFP36L1,SAMD4B,ZFP36L2,TNRC6C	up_VSAP_EC
GO:0001568	0.087	0.988	2	252	blood vessel development	BP	1	HDAC7,CITED2	down_VPOR_EC
GO:0022008	0.087	0.989	2	406	neurogenesis	BP	1	HOXC10,WNT7A	up_AORT_VSMC
GO:0006413	0.087	0.996	1	42	translational initiation	BP	1	EIF3M	down_AFEM_EC
GO:0051961	0.087	0.997	1	73	negative regulation of nervous system	BP	1	WNT7A	up_AORT_VSMC
GO:0043467	0.087	0.996	1	35	regulation of generation of precursor metabolites	BP	1	FBP1	up_VFEM_VSMC
GO:1901292	0.087	0.996	1	34	nucleoside phosphate catabolic process	BP	1	FBP1	up_VFEM_VSMC
GO:0048813	0.08709393	0.97979	3	39	dendrite morphogenesis	BP	1	PAK3,MAPK8IP2,NRP1	up_ACOR_EC
GO:0046883	0.0871	0.962	8	49	regulation of hormone secretion	BP	1	LEP,TM7SF3,PRKCE,SCG5,CHD7,CCL5,EDN1,PFKL	up_ACOR_VSMC
GO:0071216	0.0871	0.964	7	61	cellular response to biotic stimulus	BP	0.963	NR1H4,HSPA5,SERPINE1,MTDH,LYN,DDIT3,GSK3B	down_VSAP_EC
GO:0051049	0.0871	0.971	5	479	regulation of transport	BP	1	LYN,HOXA3,ACSL3,STX4,NEDD4	down_ACOR_EC

GO:0031347	0.0871	0.987	2	144	regulation of defense response	BP	1	NR1H4,CCL24	up_VPUL_EC
GO:0060364	0.0871	0.998	1	2	frontal suture morphogenesis	BP	1	GLI3	down_VCAV_EC
GO:0002040	0.0871	0.996	1	46	sprouting angiogenesis	BP	1	HDAC7	down_VPOR_EC
GO:0003208	0.0871	0.996	1	24	cardiac ventricle morphogenesis	BP	1	TBX5	up_APUL_EC
GO:0045766	0.0871	0.997	1	63	positive regulation of angiogenesis	BP	1	C6	up_VCAV_VSMC
GO:0022900	0.0872	0.996	1	46	electron transport chain	BP	1	NDUFA10	down_AFEM_EC
GO:0032602	0.0872	0.996	1	23	chemokine production	BP	1	NR1H4	up_APUL_EC
GO:0062014	0.0872	0.996	1	35	negative regulation of small molecule metabolic	BP	1	FBP1	up_VFEM_VSMC
GO:0006955	0.0873	0.938	39	327	immune response	BP	1	TLR1,SMAD6,PLCG2,LEP,CD83,COCH,TP53BP1,MX1,ELF1,TLR3,ISG15,CD274,CTSS,TINAGL1,BLNK,F2RL1,HRH2,DAPK1,C1S,EDA,TNFSF10,PTPN22,EVL,C7,PRKCE,PARP9,APP,PSEN1,CCL5,DUSP22,NOD2,DLA88,JAM3,B2M,SERINC3,TNFRSF11B,MAP3K7,PARK7,IL18R1	up_ACOR_VSMC
GO:0032504	0.0873	0.948	18	152	multicellular organism reproduction	BP	1	ACSL4,NDC1,TRIP13,CTDNEP1,FBXO5,NCAPH,POC1A,B4GALT1,HIST1H1A,FOSL1,HMGB2,SGPL1,TTK,ASPM,HOXA9,GLI1,H2AFX,TTLL1	down_ACOR_VSMC
GO:0048609	0.0873	0.948	18	152	multicellular organismal reproductive process	BP	1	ACSL4,NDC1,TRIP13,CTDNEP1,FBXO5,NCAPH,POC1A,B4GALT1,HIST1H1A,FOSL1,HMGB2,SGPL1,TTK,ASPM,HOXA9,GLI1,H2AFX,TTLL1	down_ACOR_VSMC
GO:0001780	0.0873	0.99	2	6	neutrophil homeostasis	BP	1	HMGB1,PIK3CD	down_ACOR_VSMC
GO:1904716	0.0873	1	1	1	positive regulation of chaperone-mediated	BP	1	PLK3	down_ACOR_VSMC
GO:2000777	0.0873	1	1	1	positive regulation of proteasomal ubiquitin-dependent protein catabolic process involved	BP	1	PLK3	down_ACOR_VSMC
GO:0007159	0.0873	0.997	1	79	leukocyte cell-cell adhesion	BP	1	ARG2	up_AORT_VSMC
GO:0042391	0.0873	0.997	1	81	regulation of membrane potential	BP	1	WNT7A	up_AORT_VSMC
GO:0006406	0.0874	0.971	5	27	mRNA export from nucleus	BP	0.933	IWS1,HHEX,THOC1,SMG7,NUP133	up_VCAV_EC
GO:0071427	0.0874	0.971	5	27	mRNA-containing ribonucleoprotein complex export from	BP	0.933	IWS1,HHEX,THOC1,SMG7,NUP133	up_VCAV_EC
GO:0002755	0.0874	0.99	2	6	MyD88-dependent toll-like receptor signaling	BP	0.933	TNIP1,TLR4	up_VCAV_EC
GO:0034101	0.0874	0.996	1	45	erythrocyte homeostasis	BP	1	CITED2	down_VPOR_EC
GO:0032649	0.0874	0.996	1	23	regulation of interferon-gamma production	BP	1	NR1H4	up_APUL_EC
GO:0071108	0.0875	0.982	3	11	protein K48-linked deubiquitination	BP	0.933	TNFAIP3,OTUD4,USP37	up_VCAV_EC
GO:0010591	0.0875	0.982	3	12	regulation of lamellipodium assembly	BP	0.933	MTOR,NCKAP1,FSCN1	up_VCAV_EC
GO:0016477	0.0875	0.981	3	426	cell migration	BP	1	CHL1,BAMBI,C3AR1	up_VPOR_VSMC
GO:0010742	0.0875	0.99	2	6	macrophage derived foam cell differentiation	BP	0.933	PRKCH,PPARG	up_VCAV_EC
GO:0090077	0.0875	0.99	2	6	foam cell differentiation	BP	0.933	PRKCH,PPARG	up_VCAV_EC
GO:0044419	0.08750821	0.96582	6	117	interspecies interaction between organisms	BP	1	ATG16L2,AQP1,TRIM8,MAVS,LRRC15,CHMP4B	up_ACOR_EC
GO:0009987	0.0876	0.964	29	5086	cellular process	BP	1	ACVRL1,YEATS2,LYN,HOXA3,IGF1R,ACSL4,SPG11,PDPN,NRP2,YBX3,HIVEP2,HOXA7,PRKAR2A,USP28,IPO7,RPSA,ATRIP,ANPEP,SULF1,ELK3,HOXA1,CSGALNACT1,ACSL3,WDR12,STX4,MYO1D,TNRC6B,ASPN,NEDD4	down_ACOR_EC
GO:0001822	0.0876	0.957	10	98	kidney development	BP	0.967	SULF1,ENSCAFG00000032470,ILK,HOXB7,ACTA2,MAGED1,CTNNB1,MYO1E,SIX4,NRP1	down_VSAP_EC
GO:0044773	0.0876	0.971	5	28	mitotic DNA damage checkpoint	BP	1	CDK1,CASP2,FANCI,TOPBP1,CLSPN	down_ACOR_VSMC
GO:0050869	0.0876	0.989	2	8	negative regulation of B cell activation	BP	0.967	LYN,BTK	down_VSAP_EC
GO:0003163	0.0876	1	1	1	sinoatrial node development	BP	1	BVES	up_ACOR_VSMC

GO:0060921	0.0876	1	1	1	sinoatrial node cell differentiation	BP	1	BVES	up_ACOR_VSMC
GO:0060931	0.0876	1	1	1	sinoatrial node cell development	BP	1	BVES	up_ACOR_VSMC
GO:0042539	0.0876	1	1	1	hypotonic salinity response	BP	1	SLC12A6	up_VSAP_EC
GO:0071477	0.0876	1	1	1	cellular hypotonic salinity response	BP	1	SLC12A6	up_VSAP_EC
GO:0003002	0.087657417	0.96574	6	118	regionalization	BP	1	FZD5,SMAD6,FOXC2,LRP4,CLSTN1,NRP1	up_ACOR_EC
GO:0001866	0.0877	1	1	1	NK T cell proliferation	BP	1	ELF4	down_ACOR_VSMC
GO:0007052	0.0877	0.996	1	42	mitotic spindle organization	BP	1	GPSM2	down_AFEM_EC
GO:0035262	0.0877	1	1	1	gonad morphogenesis	BP	1	LHX9	up_ACOR_VSMC
GO:0008584	0.0877	0.996	1	39	male gonad development	BP	1	HOXA9	up_AORT_EC
GO:0046546	0.0877	0.996	1	39	development of primary male sexual	BP	1	HOXA9	up_AORT_EC
GO:0001570	0.0877	0.996	1	37	vasculogenesis	BP	1	NKX2-5	up_VPOR_VSMC
GO:0014855	0.0877	0.996	1	22	striated muscle cell proliferation	BP	1	SIX1	up_VFEM_EC
GO:0046580	0.0878	0.976	4	18	negative regulation of Ras protein signal	BP	0.933	RAPGEF1,ARAP3,STMN1,TNFAIP1	up_VCAV_EC
GO:0010835	0.0878	0.99	2	6	regulation of protein ADP-ribosylation	BP	1	PUM3,TINF2	down_ACOR_VSMC
GO:0048537	0.0878	1	1	1	mucosal-associated lymphoid tissue	BP	1	TOX	down_ACOR_VSMC
GO:0048541	0.0878	1	1	1	Peyer's patch development	BP	1	TOX	down_ACOR_VSMC
GO:1901293	0.0878	0.997	1	106	nucleoside phosphate biosynthetic process	BP	1	AK5	down_VSAP_VSMC
GO:0090360	0.0878	1	1	1	platelet-derived growth factor production	BP	1	NDRG2	up_ACOR_VSMC
GO:0090361	0.0878	1	1	1	regulation of platelet-derived growth factor	BP	1	NDRG2	up_ACOR_VSMC
GO:0008217	0.0878	0.996	1	38	regulation of blood pressure	BP	1	LNPEP	up_AORT_EC
GO:0045088	0.0878	0.997	1	81	regulation of innate immune response	BP	1	ESR1	up_AORT_VSMC
GO:0031529	0.0878	0.996	1	20	ruffle organization	BP	1	CAV1	up_VSAP_VSMC
GO:0008654	0.0879	0.959	9	58	phospholipid biosynthetic process	BP	1	PIP5K1B,PLCG2,HTR2B,LCAT,CHPT1,PIGN,PIGS,PIGG,CRLS1	up_ACOR_VSMC
GO:1904377	0.0879	0.98	3	21	positive regulation of protein localization to cell	BP	1	LRP1,PRNP,CLIP3	down_VCAV_EC
GO:0045661	0.0879	0.981	3	18	regulation of myoblast differentiation	BP	0.969	PRICKLE1,DDIT3,HIF1AN	down_VSAP_EC
GO:0003413	0.0879	0.99	2	6	chondrocyte differentiation involved in endochondral bone	BP	1	POC1A,SERPINH1	down_ACOR_VSMC
GO:0051098	0.0879	0.987	2	129	regulation of binding	BP	1	HOXA3,PLCL1	up_APUL_EC
GO:0060009	0.0879	0.99	2	5	Sertoli cell development	BP	0.933	ARID4B,ATRX	up_VCAV_EC
GO:0034394	0.0879	0.996	1	19	protein localization to cell surface	BP	1	STX4	down_ACOR_EC
GO:1902749	0.0879	0.996	1	39	regulation of cell cycle G2/M phase transition	BP	1	TAOK1	up_AORT_EC
GO:0046889	0.0879	0.996	1	24	positive regulation of lipid biosynthetic process	BP	1	NR1H4	up_APUL_EC
GO:0048565	0.0879	0.996	1	38	digestive tract development	BP	1	SHOX2	up_VFEM_VSMC
GO:0043331	0.0879	0.996	1	20	response to dsRNA	BP	1	CAV1	up_VSAP_VSMC
GO:0010596	0.088	0.987	2	21	negative regulation of endothelial cell migration	BP	1	ANGPT4,TBXA2R	up_AFEM_EC
GO:0071417	0.088	0.987	2	142	cellular response to organonitrogen	BP	1	NR1H4,ARHGEF2	up_VPUL_EC
GO:1902510	0.088	1	1	1	regulation of apoptotic DNA fragmentation	BP	1	AIFM1	down_ACOR_VSMC
GO:1903624	0.088	1	1	1	regulation of DNA catabolic process	BP	1	AIFM1	down_ACOR_VSMC
GO:0038034	0.088	0.996	1	21	signal transduction in absence of ligand	BP	1	EYA1	up_VFEM_EC
GO:0097192	0.088	0.996	1	21	extrinsic apoptotic signaling pathway in	BP	1	EYA1	up_VFEM_EC

GO:0006469	0.0881	0.961	8	78	negative regulation of protein kinase activity	BP	0.97	SOCS5,ERRFI1,TRIB3,PTPN2,APOE,LYN,CBLC,CDKN1A	down_VSAP_EC
GO:0006893	0.0881	0.996	1	20	Golgi to plasma membrane transport	BP	1	ACSL3	down_ACOR_EC
GO:1903034	0.088195945	0.9794	3	40	regulation of response to wounding	BP	1	RGMA,FOXC2,PRKCD	up_ACOR_EC
GO:0030509	0.0882	0.97	5	47	BMP signaling pathway	BP	1	SMAD9,FBN1,CHRDL1,GREM1,RGMA	down_VCAV_EC
GO:1905340	0.0882	1	1	1	regulation of protein localization to	BP	1	NDC80	down_ACOR_VSMC
GO:1905342	0.0882	1	1	1	positive regulation of protein localization to	BP	1	NDC80	down_ACOR_VSMC
GO:0031110	0.0882	0.996	1	24	regulation of microtubule polymerization or depolymerization	BP	1	TUBB4A	up_APUL_EC
GO:0007411	0.0882	0.996	1	45	axon guidance	BP	1	SLIT2	up_VPUL_VSMC
GO:0097485	0.0882	0.996	1	45	neuron projection guidance	BP	1	SLIT2	up_VPUL_VSMC
GO:0072210	0.088247192	0.98719	2	19	metanephric nephron development	BP	1	SOX9,AQP1	up_ACOR_EC
GO:0071363	0.0883	0.945	21	188	cellular response to growth factor stimulus	BP	0.933	CXCL8,PDGFB,MEF2C,FERMT1,DLL4,RAPGEF1,GATA3,SMAD1,YES1,BMP2,HHEX,PRKD2,RAPGEF2,CTDSPL2,CREB1,RIPK1,SKIL,HIF1A,ENSCAFG00000002015,JCAD,ZEB1	up_VCAV_EC
GO:0002064	0.0883	0.955	11	71	epithelial cell development	BP	1	EXPH5,SHROOM3,BMP4,GJA1,ATRX,SEC24B,ARHGEF26,F2RL1,SPINT2,FRS2,FOSL2	up_ACOR_VSMC
GO:0032507	0.0883	0.98	3	18	maintenance of protein location in cell	BP	0.97	HSPA5,TLN1,KDELR2	down_VSAP_EC
GO:0035924	0.0883	0.987	2	21	cellular response to vascular endothelial growth factor stimulus	BP	1	GAS1,DLL1	up_AFEM_EC
GO:0055065	0.0883	0.987	2	145	metal ion homeostasis	BP	1	HOXA3,NEO1	up_VPUL_EC
GO:0001658	0.0883	0.996	1	24	branching involved in ureteric bud	BP	1	NPNT	up_APUL_EC
GO:0062013	0.0883	0.996	1	27	positive regulation of small molecule metabolic	BP	1	NR1H4	up_VPUL_EC
GO:0008306	0.0883	0.996	1	21	associative learning	BP	1	NETO1	up_VSAP_VSMC
GO:0032880	0.0884	0.944	22	266	regulation of protein localization	BP	0.97	NR1H4,PLK3,PTPN14,PTPN11,SESN2,MYO1C,MSN,KIF5B,HIF1A,IWS1,ZPR1,CTNNB1,LYN,MMD,GSK3B,SH3GLB1,TAX1BP3,REST,RAB11FIP1,SIX4,CDKN1A,RHBDF2	down_VSAP_EC
GO:0006511	0.0884	0.945	21	253	ubiquitin-dependent protein catabolic process	BP	0.97	PRICKLE1,PLK3,SOCS5,HSP90B1,TRIB3,USP12,DNAJC10,UBA6,UCHL5,SPOPL,DNAJB9,USP48,DDIT3,CBLC,GSK3B,CUL2,SPOP,DISC1,RAD23A,USP14,TNFAIP1	down_VSAP_EC
GO:0007154	0.0884	0.968	7	1622	cell communication	BP	1	FGF5,BARX1,NKX2-5,BAMBI,ANKRD6,CYP26B1,C3AR1	up_VPOR_VSMC
GO:0097581	0.0884	0.971	5	26	lamellipodium organization	BP	1	WASF3,ENPP2,SLIT2,FGD4,NCK1	up_VSAP_EC
GO:0060571	0.0884	0.981	3	13	morphogenesis of an epithelial fold	BP	0.933	HHEX,HIF1A,LUZP1	up_VCAV_EC
GO:0001782	0.0884	0.989	2	8	B cell homeostasis	BP	0.97	HIF1A,LYN	down_VSAP_EC
GO:2000574	0.0884	0.989	2	6	regulation of microtubule motor activity	BP	1	DYNC1I2,DYNC1I1	up_ACOR_VSMC
GO:0010721	0.0884	0.997	1	73	negative regulation of cell development	BP	1	WNT7A	up_AORT_VSMC
GO:0042632	0.0884	0.996	1	27	cholesterol homeostasis	BP	1	NR1H4	up_VPUL_EC
GO:0051270	0.0885	0.938	36	296	regulation of cellular component movement	BP	1	FGF13,VEGFD,LGR6,KCNE3,TCAF1,RECK,BMP4,SH3RF2,TP53INP1,SASH1,LMO4,FGF18,F2RL1,NRP1,PTPN22,EVL,SPINT2,TBX5,PRKCE,DAG1,APP,LAMA3,SEMA6D,SYNJ2BP,JUP,CCL5,DDR2,DUSP22,EDN1,FAM107A,JAM3,WASL,DOCK5,RREB1,CRKL,PIK3C2A	up_ACOR_VSMC
GO:0051336	0.0885	0.94	31	251	regulation of hydrolase activity	BP	1	TNNT2,HTR2B,USP6NL,MBP,RECK,EVI5L,FGFR2,SCARB2,SH3RF2,PKP4,CHN2,CST3,F2RL1,DAPK1,NPNT,SH3RF1,MARF1,PPP1R26,SGSM2,SPINK2,CCL5,PERP,DYNC1I2,SBF2,SH3BP4,DYNC1I1,PARK7,GPATCH2,ZFYVE1,BVES,LMF1	up_ACOR_VSMC
GO:0010565	0.0885	0.97	5	40	regulation of cellular ketone metabolic process	BP	0.971	NR1H4,TRIB3,PTGS2,SLC45A3,REST	down_VSAP_EC
GO:0060671	0.0885	1	1	1	epithelial cell differentiation involved in embryonic placenta	BP	1	SPINT2	up_ACOR_VSMC

GO:0060672	0.0885	1	1	1	epithelial cell morphogenesis involved	BP	1	SPINT2	up_ACOR_VSMC
GO:1902083	0.0885	1	1	1	negative regulation of peptidyl-cysteine S-	BP	1	OXR1	up_ACOR_VSMC
GO:2000169	0.0885	1	1	1	regulation of peptidyl-cysteine S-nitrosylation	BP	1	OXR1	up_ACOR_VSMC
GO:0042891	0.0885	1	1	1	antibiotic transport	BP	0.933	RALBP1	up_VCAV_EC
GO:1900753	0.0885	1	1	1	doxorubicin transport	BP	0.933	RALBP1	up_VCAV_EC
GO:1901656	0.0885	1	1	1	glycoside transport	BP	0.933	RALBP1	up_VCAV_EC
GO:0007275	0.0886	0.926	147	1370	multicellular organism development	BP	1	EXPH5,TNNT2,SH3TC2,PDLIM3,FGF13,VEGFD,TMEM176B,TMEM176A,SMAD6,ACTN2,PLCG2,LEP,LBH,AFF3,GATA5,GATA4,CD83,ANGPT1,EPYC,HTR2B,RASSF2,MBP,PRDX4,RECK,SHROOM3,FEZ1,MLLT3,ITM2C,FERMT1,BMP4,LNPK,TBX18,FGFR2,TP53BP1,GJA1,ATRX,TLR3,SEC24B,XIRP1,WASF3,PPARGC1A,LATS2,CITED2,SPINT1,GLRB,ISG15,ACSL6,ZNRF3,CD274,ZNF365,SASH1,LMO4,TMEM108,FGF18,FAM210B,NDRG2,ATRN,P4HTM,NEBL,CDKN2B,EBF4,ZMYM2,ITPR1,LIMS2,ANKRD1,F2RL1,CAMK2D,CTSB,EGLN1,ENSCAFG00000006238,CLSTN1,NRP1,PTPN22,GPM6A,CHD2,ULK2,NPNT,UNC5B,SMARCD3,SPINT2,SLITRK5,PTGDR,TBX20,BBX,TBX5,FZD3,FRS2,CPAMD8,RND1,DAG1,POMK,APP,LAMA3,PSEN1,SEMA6D,TGFBR1,PCSK6,SYNJ2BP,CHD7,JUP,SPINK2,SIPA1L1,DDR2,CLN8,FOSL2,PERP,PSKH1,RTKN2,SIAH1,SSH3,C10H2orf49,EDN1,NID1,HPGD,ARID5B,ASAP1,DMC1,SERPINB5,JAM3,B2M,DISC1,AP2B1,TNFRSF11B,TACC1,GAA,ATXN10,FEZ2,SUN1,INPP5K,CNOT4,LHX9,WFS1,WDR48,WASL,ACP2,ERAP1,ID3,SLC8A1,TEAD1,ENSCAFG00000003530,IL18R1,BVES,RREB1,ZDHHC21,CRKL,PIK3C2A,PTPN14	up_ACOR_VSMC
GO:0007163	0.0886	0.959	9	60	establishment or maintenance of cell	BP	0.933	EPHB1,TEK,FRMD4B,AMOT,FERMT1,GATA3,CTNNA1,ZW10,FSCN1	up_VCAV_EC
GO:0002295	0.0886	0.99	2	5	T-helper cell lineage commitment	BP	0.933	IL6,MTOR	up_VCAV_EC
GO:0002363	0.0886	0.99	2	5	alpha-beta T cell lineage commitment	BP	0.933	IL6,MTOR	up_VCAV_EC
GO:0043373	0.0886	0.99	2	5	CD4-positive, alpha-beta T cell lineage commitment	BP	0.933	IL6,MTOR	up_VCAV_EC
GO:0007195	0.0886	1	1	1	adenylate cyclase-inhibiting dopamine receptor signaling	BP	1	PRMT5	down_ACOR_VSMC
GO:0019918	0.0886	1	1	1	peptidyl-arginine methylation, to	BP	1	PRMT5	down_ACOR_VSMC
GO:0035246	0.0886	1	1	1	peptidyl-arginine N-methylation	BP	1	PRMT5	down_ACOR_VSMC
GO:0035247	0.0886	1	1	1	peptidyl-arginine omega-N-methylation	BP	1	PRMT5	down_ACOR_VSMC
GO:1904990	0.0886	1	1	1	regulation of adenylate cyclase-inhibiting dopamine receptor	BP	1	PRMT5	down_ACOR_VSMC
GO:1904992	0.0886	1	1	1	positive regulation of adenylate cyclase-inhibiting dopamine	BP	1	PRMT5	down_ACOR_VSMC
GO:0051248	0.0886	0.998	1	316	negative regulation of protein metabolic process	BP	1	CST3	down_AORT_EC
GO:2000117	0.0886	0.996	1	27	negative regulation of cysteine-type	BP	1	PTGER2	up_VPUL_EC

GO:0006355	0.0887	0.929	93	974	regulation of transcription, DNA-templated	BP	0.933	EDNRB,HCLS1,KIT,PRKCH,FLI1,TNFAIP3,PPARG,TNIP1,PDGFB,TMEM173,RELN,IWS1,MEF2C,ATF3,CHD7,DLL4,WWP1,TLR4,NLRC5,GATA3,EDN1,HOXB8,TCF4,SMAD1,FOXN2,MEOX1,ENSCAFG00000017103,IRF8,SOX18,PELI1,PPP3CA,YES1,MTOR,ELF4,BMP2,HMG20A,HHEX,CREM,RSC1A1,CCAR1,PROX1,UHRF2,MDFI,ENSCAFG00000010596,ARID4B,HMBOX1,PRKD2,THOC1,SMARCA4,TLR3,NCK1,TIMELESS,LDB2,CD200,JAG1,NSD1,TCERG1,CTDSPL2,CREB1,BCL10,BASP1,CNOT7,ENSCAFG00000002897,HMGB1,AHR,ZNF789,BLM,NUP133,RIPK1,ZNF317,USP13,ITGA6,SKIL,GPBP1,MTA2,NR1D2,KDM5A,HIF1A,KDM3A,ZNF146,ATRX,NR4A1,HIST2H2AC,HP1BP3,ARID5A,ENSCAFG00000002015,ZNF354A,ZEB1,TET2,ARHGEF2,CDON,ELK3,ZNF609	up_VCAV_EC
GO:0052027	0.0887	1	1	1	modulation by symbiont of host signal transduction	BP	0.933	TNIP1	up_VCAV_EC
GO:0085032	0.0887	1	1	1	modulation by symbiont of host I-kappaB kinase/NF-kappaB	BP	0.933	TNIP1	up_VCAV_EC
GO:0022409	0.0888	0.961	8	56	positive regulation of cell-cell adhesion	BP	0.933	IL7R,PODXL,GATA3,ITPKB,NCK1,BCL10,HMGB1,ITGA6	up_VCAV_EC
GO:0006812	0.0888	0.979	3	222	cation transport	BP	1	LYN,HOXA3,NEDD4	down_ACOR_EC
GO:0031333	0.0888	0.996	1	46	negative regulation of protein complex assembly	BP	1	SLIT2	up_VPUL_VSMC
GO:0032677	0.0888	0.996	1	22	regulation of interleukin-8 production	BP	1	TLR2	up_VSAP_VSMC
GO:0010155	0.088845327	0.99694	1	4	regulation of proton transport	BP	1	PM20D1	up_ACOR_EC
GO:0060348	0.0889	0.963	7	78	bone development	BP	1	NOTCH2,SMAD9,NAB2,FBN1,THBS3,GLI3,GREM1	down_VCAV_EC
GO:0040013	0.0889	0.969	5	100	negative regulation of locomotion	BP	1	ANGPT4,HOXA7,RECK,TBXA2R,KANK1	up_AFEM_EC
GO:0032228	0.0889	0.988	2	8	regulation of synaptic transmission, GABAergic	BP	0.974	KIF5B,PLCL1	down_VSAP_EC
GO:0036363	0.0889	1	1	1	transforming growth factor beta activation	BP	1	SLC29A1	down_ACOR_VSMC
GO:1901388	0.0889	1	1	1	regulation of transforming growth factor beta	BP	1	SLC29A1	down_ACOR_VSMC
GO:1901389	0.0889	1	1	1	negative regulation of transforming growth factor beta activation	BP	1	SLC29A1	down_ACOR_VSMC
GO:1905323	0.0889	1	1	1	telomerase holoenzyme complex assembly	BP	1	SLC29A1	down_ACOR_VSMC
GO:0007178	0.0889	0.997	1	107	transmembrane receptor protein serine/threonine kinase signaling pathway	BP	1	TGFBR1	down_VSAP_VSMC
GO:0003334	0.0889	0.997	1	4	keratinocyte development	BP	1	FOSL2	up_AFEM_EC
GO:0035637	0.0889	0.996	1	21	multicellular organismal signaling	BP	1	CAV1	up_VSAP_VSMC
GO:0045861	0.089	0.973	4	71	negative regulation of proteolysis	BP	1	GAS1,CRYAB,CLEC16A,RECK	up_AFEM_EC
GO:0010033	0.089	0.982	3	772	response to organic substance	BP	1	CYP1A1,GATA5,CA2	up_VCAV_VSMC
GO:0035162	0.089	0.988	2	8	embryonic hemopoiesis	BP	0.974	HIF1A,SH2B3	down_VSAP_EC
GO:0031345	0.089	0.996	1	46	negative regulation of cell projection organization	BP	1	SLIT2	up_VPUL_VSMC
GO:0002040	0.089	0.996	1	46	sprouting angiogenesis	BP	1	SLIT2	up_VPUL_VSMC
GO:1903901	0.08903468	0.98701	2	19	negative regulation of viral life cycle	BP	1	TRIM8,MAVS	up_ACOR_EC
GO:2000377	0.0891	0.969	5	41	regulation of reactive oxygen species metabolic	BP	0.975	PRCP,HIF1A,BNIP3,CDKN1A,ARF4	down_VSAP_EC
GO:0030500	0.0891	0.971	5	25	regulation of bone mineralization	BP	1	OSR2,OSR1,DDR2,OMD,BGLAP	up_VSAP_EC
GO:0001738	0.0891	0.974	4	27	morphogenesis of a polarized epithelium	BP	0.975	PTK7,EXOC5,WDR1,MSN	down_VSAP_EC
GO:0031297	0.0891	0.975	4	18	replication fork processing	BP	0.933	THOC1,ETAA1,BLM,ATRX	up_VCAV_EC
GO:0050873	0.0891	0.975	4	18	brown fat cell differentiation	BP	1	NAPEPLD,EBF2,ARL4A,ALDH6A1	up_VSAP_EC
GO:0033314	0.0891	0.989	2	6	mitotic DNA replication checkpoint	BP	1	TOPBP1,CLSPN	down_ACOR_VSMC

GO:0070493	0.0891	0.989	2	6	thrombin-activated receptor signaling	BP	1	STMN1,IQGAP2	down_ACOR_VSMC
GO:0032877	0.0891	1	1	1	positive regulation of DNA endoreduplication	BP	1	E2F8	down_ACOR_VSMC
GO:0033301	0.0891	1	1	1	cell cycle comprising mitosis without	BP	1	E2F8	down_ACOR_VSMC
GO:0033865	0.0891	0.996	1	43	nucleoside bisphosphate metabolic process	BP	1	SUCLG2	down_AFEM_EC
GO:0033875	0.0891	0.996	1	43	ribonucleoside bisphosphate metabolic	BP	1	SUCLG2	down_AFEM_EC
GO:0034032	0.0891	0.996	1	43	purine nucleoside bisphosphate metabolic	BP	1	SUCLG2	down_AFEM_EC
GO:0043624	0.0891	0.996	1	39	cellular protein complex disassembly	BP	1	TAOK1	up_AORT_EC
GO:0048762	0.0892	0.962	7	77	mesenchymal cell differentiation	BP	1	VASN,GLIPR2,BMPR1A,SEMA3C,LOXL3,RDH10,GREM1	down_VCAV_EC
GO:0097305	0.0892	0.967	6	31	response to alcohol	BP	1	PRKAA2,TP53INP1,ADCY6,PTGDR,PRKCE,JUP	up_ACOR_VSMC
GO:0022008	0.0892	0.974	4	406	neurogenesis	BP	1	EMX2,HOXC10,IRX5,SHOX2	up_VSAP_VSMC
GO:0009887	0.0892	0.998	1	314	animal organ morphogenesis	BP	1	GATA5	down_VFEM_VSMC
GO:1902285	0.0892	0.997	1	4	semaphorin-plexin signaling pathway involved in neuron	BP	1	PLXNA4	up_AFEM_EC
GO:0051098	0.0892	0.997	1	129	regulation of binding	BP	1	HOXA3	up_APUL_VSMC
GO:0009743	0.089273988	0.97905	3	40	response to carbohydrate	BP	1	NR1H4,KCNB1,PRKACA	up_ACOR_EC
GO:0019219	0.0893	0.928	110	1151	regulation of nucleobase-containing compound metabolic process	BP	1	ELK3,HMGB1,RNPS1,SREBF1,USP1,IRAK1,ILF3,CBX5,PPRC1,ATAD2,HMGA1,HNRNPD,SREBF2,SMC1A,HINFP,NEDD4,SRSF2,MED17,U2AF2,SLC29A1,MTDH,HEY1,PARPBP,SRRT,NAB2,CDCA7L,FUS,RFC2,BIRC5,MYBBP1A,INSIG1,PRMT1,CAV1,CDK1,RFC5,RFC3,RAD51,TIMELESS,THRAP3,FAM129B,TCEA1,AURKB,NONO,HNRNPL,EZH2,GREM1,RFC4,NEK2,SMARCC1,PLK3,PCNA,DHX33,HOXA7,SF3B4,UHRF1,CKS2,GMNN,BRCA1,FASTKD3,NOC2L,E2F8,TFRC,PPARD,YBX3,HNRNPF,NCL,NUP62,FOSL1,SUV39H1,ZNF367,CACTIN,HMGB2,DMAP1,SMC3,CDK2,SMARCB1,DDX39B,CCNA2,ENSCAFG00000005299,EZR,RBM14,RBM17,HOXA10,ERF,TCERG1,SMARCAL1,ELF4,PRMT5,PSMC1,CTH,AIFM1,ARHGEF2,SUPT16H,TADA2A,WTAP,HAT1,HIST2H2AC,HOXA9,GLI1,TINF2,JMJD6,H2AFX,CDC5L,MIS18A,HMGN1,CCT4,SIX4,SF3A1,MED16,ENSCAFG00000017103	down_ACOR_VSMC
GO:0046483	0.0893	0.966	8	1948	heterocycle metabolic process	BP	1	EYA1,HOXA10,HOXA9,TBX15,FBP1,EMX2,HOXA11,SHOX2	up_VFEM_VSMC
GO:1903146	0.0893	0.98	3	17	regulation of autophagy of mitochondrion	BP	0.976	HIF1A,CDC37,HTT	down_VSAP_EC
GO:0006614	0.0893	0.988	2	10	SRP-dependent cotranslational protein	BP	1	SSR3,SEC61B	down_VCAV_EC
GO:0022607	0.0894	0.929	90	969	cellular component assembly	BP	0.933	EPHB1,PLK2,TEK,HCLS1,GBP5,SELP,KIT,PRKCH,ARHGEF15,CDA,MX2,PODXL,BAIAP2L1,VWF,LAMC2,OCLN,MEF2C,RAPGEF1,TLR4,EDN1,RAB11A,RALB,SMAD1,SNCAIP,IRF8,SNAP91,ARHGAP28,TMOD1,NASP,NECTIN2,MTOR,ACPP,SNAP23,LRRC8C,TJP1,LNX2,CAV2,SLK,SCAF11,CNOT6,ABCA1,NCKAP1,MZT1,RIOK3,PROX1,IQGAP2,ENSCAFG00000010224,STMN1,SNX2,SH3GLB1,SWAP70,APOD,CENPC,NCK1,KCNJ2,EXOC5,FES,HIST1H1A,EHD4,RAPGEF2,BCL10,SENP6,PAN3,PRPF39,CTNNA1,CNOT7,GCH1,ASF1A,HMGB1,PMAIP1,ARPC5L,BLM,RIPK1,LSM14A,ITGA6,MCU,ZW10,IQUB,ATRX,HAUS3,ALDH1A3,HP1BP3,ENSCAFG00000002015,P2RX2,TNFAIP1,SPATA6,TMOD3,FSCN1,FBXO5,SASS6	up_VCAV_EC
GO:0060560	0.0894	0.958	9	58	developmental growth involved in	BP	1	WNT11,SLIT2,ABL1,PLXNA4,RARG,DPYSL2,TTL,ULK1,TGFBR2	up_VSAP_EC
GO:0048260	0.0894	0.981	3	15	positive regulation of receptor-mediated	BP	0.933	SELE,HIST1H1A,PLCG2	up_VCAV_EC
GO:0006954	0.0894	0.987	2	147	inflammatory response	BP	1	NR1H4,CCL24	up_VPUL_EC
GO:0051852	0.0894	1	1	1	disruption by host of symbiont cells	BP	1	F2RL1	up_ACOR_VSMC

GO:0051873	0.0894	1	1	1	killing by host of symbiont cells	BP	1	F2RL1	up_ACOR_VSMC
GO:0070942	0.0894	1	1	1	neutrophil mediated cytotoxicity	BP	1	F2RL1	up_ACOR_VSMC
GO:0070943	0.0894	1	1	1	neutrophil mediated killing of symbiont cell	BP	1	F2RL1	up_ACOR_VSMC
GO:0070944	0.0894	1	1	1	neutrophil mediated killing of bacterium	BP	1	F2RL1	up_ACOR_VSMC
GO:0070945	0.0894	1	1	1	neutrophil mediated killing of gram-negative	BP	1	F2RL1	up_ACOR_VSMC
GO:0070948	0.0894	1	1	1	regulation of neutrophil mediated cytotoxicity	BP	1	F2RL1	up_ACOR_VSMC
GO:0070949	0.0894	1	1	1	regulation of neutrophil mediated killing of	BP	1	F2RL1	up_ACOR_VSMC
GO:0070950	0.0894	1	1	1	regulation of neutrophil mediated killing of	BP	1	F2RL1	up_ACOR_VSMC
GO:0070951	0.0894	1	1	1	regulation of neutrophil mediated killing of gram-	BP	1	F2RL1	up_ACOR_VSMC
GO:0070960	0.0894	1	1	1	positive regulation of neutrophil mediated	BP	1	F2RL1	up_ACOR_VSMC
GO:0070962	0.0894	1	1	1	positive regulation of neutrophil mediated	BP	1	F2RL1	up_ACOR_VSMC
GO:0070963	0.0894	1	1	1	positive regulation of neutrophil mediated killing of gram-negative	BP	1	F2RL1	up_ACOR_VSMC
GO:0090198	0.0894	1	1	1	negative regulation of chemokine secretion	BP	1	F2RL1	up_ACOR_VSMC
GO:0097029	0.0894	1	1	1	mature conventional dendritic cell	BP	1	F2RL1	up_ACOR_VSMC
GO:1900135	0.0894	1	1	1	positive regulation of renin secretion into blood	BP	1	F2RL1	up_ACOR_VSMC
GO:0070961	0.0894	1	1	1	positive regulation of neutrophil mediated	BP	1	F2RL1	up_ACOR_VSMC
GO:0003100	0.0894	1	1	1	regulation of systemic arterial blood pressure by	BP	0.933	EDN1	up_VCAV_EC
GO:0003298	0.0894	1	1	1	physiological muscle hypertrophy	BP	0.933	EDN1	up_VCAV_EC
GO:0003301	0.0894	1	1	1	physiological cardiac muscle hypertrophy	BP	0.933	EDN1	up_VCAV_EC
GO:0031583	0.0894	1	1	1	phospholipase D-activating G protein-coupled receptor signaling	BP	0.933	EDN1	up_VCAV_EC
GO:0035812	0.0894	1	1	1	renal sodium excretion	BP	0.933	EDN1	up_VCAV_EC
GO:0035813	0.0894	1	1	1	regulation of renal sodium excretion	BP	0.933	EDN1	up_VCAV_EC
GO:0035815	0.0894	1	1	1	positive regulation of renal sodium excretion	BP	0.933	EDN1	up_VCAV_EC
GO:0042313	0.0894	1	1	1	protein kinase C deactivation	BP	0.933	EDN1	up_VCAV_EC
GO:0043179	0.0894	1	1	1	rhythmic excitation	BP	0.933	EDN1	up_VCAV_EC
GO:0051771	0.0894	1	1	1	negative regulation of nitric-oxide synthase	BP	0.933	EDN1	up_VCAV_EC
GO:0060024	0.0894	1	1	1	rhythmic synaptic transmission	BP	0.933	EDN1	up_VCAV_EC
GO:0060584	0.0894	1	1	1	regulation of prostaglandin-	BP	0.933	EDN1	up_VCAV_EC
GO:0060585	0.0894	1	1	1	positive regulation of prostaglandin-endoperoxide synthase	BP	0.933	EDN1	up_VCAV_EC
GO:0061049	0.0894	1	1	1	cell growth involved in cardiac muscle cell	BP	0.933	EDN1	up_VCAV_EC
GO:0061050	0.0894	1	1	1	regulation of cell growth involved in cardiac muscle cell development	BP	0.933	EDN1	up_VCAV_EC
GO:0061051	0.0894	1	1	1	positive regulation of cell growth involved in cardiac muscle cell development	BP	0.933	EDN1	up_VCAV_EC
GO:0060349	0.0894	0.996	1	38	bone morphogenesis	BP	1	CYP26B1	up_VPOR_VSMC
GO:0016241	0.0895	0.963	7	41	regulation of macroautophagy	BP	1	MFN2,POLDIP2,PIP4K2B,UBQLN2,HTRA2,HMOX1,NPRL3	up_VSAP_EC
GO:1904062	0.0895	0.965	6	62	regulation of cation transmembrane transport	BP	1	PRNP,FXYD1,CACNB3,THY1,PARK7,CYBA	down_VCAV_EC
GO:0006412	0.0895	0.987	2	245	translation	BP	1	DARS,EIF3M	down_AFEM_EC
GO:0045600	0.0895	0.987	2	21	positive regulation of fat cell differentiation	BP	1	CMKLR1,NAPEPLD	up_AFEM_EC

GO:0003223	0.0895	0.998	1	2	ventricular compact myocardium	BP	1	BMPR1A	down_VCAV_EC
GO:0001697	0.0895	1	1	1	histamine-induced gastric acid secretion	BP	1	HRH2	up_ACOR_VSMC
GO:0006679	0.0895	1	1	1	glucosylceramide biosynthetic process	BP	1	UGCG	up_ACOR_VSMC
GO:0021563	0.0895	0.997	1	4	glossopharyngeal nerve development	BP	1	PLXNA4	up_AFEM_EC
GO:0045669	0.0895	0.996	1	24	positive regulation of osteoblast differentiation	BP	1	NPNT	up_APUL_EC
GO:0016477	0.0896	0.934	50	426	cell migration	BP	1	FGF13,VEGFD,LGR6,HTR2B,TCAF1,ZRANB1,RECK,FERMT1,BMP4,GJA1,HRH1,SH3RF2,TP53INP1,SASH1,LMO4,FGF18,F2RL1,NRP1,PTPN22,GPM6A,EVL,TBX20,TBX5,PRKCE,FZD3,DAG1,FUT8,POMK,APP,LAMA3,PSEN1,SEMA6D,SYNJ2BP,JUP,CCL5,DDR2,DUSP22,EDN1,ARID5B,FAM107A,JAM3,DISC1,SUN1,WASL,DOCK5,ENSCAFG00000029553,BVES,RREB1,CRKL,PIK3C2A	up_ACOR_VSMC
GO:0060693	0.0896	0.989	2	6	regulation of branching involved in salivary gland	BP	0.934	NTN4,BTBD7	up_VCAV_EC
GO:0001568	0.0896	0.987	2	252	blood vessel development	BP	1	HOXA3,SLIT2	up_VPUL_VSMC
GO:0003137	0.0896	1	1	1	Notch signaling pathway involved in heart	BP	1	GATA5	up_ACOR_VSMC
GO:0061999	0.0896	1	1	1	regulation of cardiac endothelial to	BP	1	GATA5	up_ACOR_VSMC
GO:0062000	0.0896	1	1	1	positive regulation of cardiac endothelial to mesenchymal transition	BP	1	GATA5	up_ACOR_VSMC
GO:0090381	0.0896	1	1	1	regulation of heart induction	BP	1	GATA5	up_ACOR_VSMC
GO:1901321	0.0896	1	1	1	positive regulation of heart induction	BP	1	GATA5	up_ACOR_VSMC
GO:0035480	0.0896	1	1	1	regulation of Notch signaling pathway involved in heart	BP	1	GATA5	up_ACOR_VSMC
GO:0035481	0.0896	1	1	1	positive regulation of Notch signaling pathway involved in heart	BP	1	GATA5	up_ACOR_VSMC
GO:0071706	0.0896	0.996	1	40	tumor necrosis factor superfamily cytokine	BP	1	BPI	up_AORT_EC
GO:0008202	0.0896	0.996	1	80	steroid metabolic process	BP	1	ESR1	up_AORT_VSMC
GO:0033209	0.0896	0.996	1	24	tumor necrosis factor-mediated signaling	BP	1	NR1H4	up_APUL_EC
GO:0030510	0.0896	0.996	1	27	regulation of BMP signaling pathway	BP	1	NEO1	up_VPUL_EC
GO:0040011	0.0897	0.938	32	505	locomotion	BP	1	PDGFRB,DCN,NDN,SDC4,GLIPR2,KANK2,BMPR1A,LMO4,RECK,RHOD,AKT2,DOCK10,SYDE1,HSD3B7,LAMA3,CSPG4,TRIM8,SEMA3C,THY1,PEX5,B4GALT1,GLI3,SASH1,CDH2,FUT8,SPRED1,SPNS1,TWIST2,DDR2,GREM1,RAC2,FOXO3	down_VCAV_EC
GO:0090066	0.0897	0.962	7	162	regulation of anatomical structure size	BP	1	PRR16,PLXNA4,HTR1D,AVPR1A,TBXA2R,CDC42EP5,KANK1	up_AFEM_EC
GO:0042254	0.0897	0.979	3	128	ribosome biogenesis	BP	1	RSL24D1,RPL10L,RRP15	up_VPOR_EC
GO:0032660	0.0897	0.989	2	7	regulation of interleukin-17 production	BP	0.935	TLR4,ARG2	up_VCAV_EC
GO:1901533	0.0897	1	1	1	negative regulation of hematopoietic progenitor cell differentiation	BP	1	HSPA9	down_ACOR_VSMC
GO:1902037	0.0897	1	1	1	negative regulation of hematopoietic stem cell differentiation	BP	1	HSPA9	down_ACOR_VSMC
GO:0042489	0.0897	1	1	1	negative regulation of odontogenesis of dentin-containing tooth	BP	1	TNFRSF11B	up_ACOR_VSMC
GO:1900046	0.0897	0.996	1	21	regulation of hemostasis	BP	1	CAV1	up_VSAP_VSMC
GO:0030193	0.0897	0.996	1	21	regulation of blood coagulation	BP	1	CAV1	up_VSAP_VSMC
GO:0032436	0.08975112	0.97889	3	40	positive regulation of proteasomal ubiquitin-dependent protein	BP	1	PIAS1,CLU,RNF114	up_ACOR_EC
GO:0001525	0.0898	0.951	13	173	angiogenesis	BP	1	HSPB6,PDGFRB,DCN,ANGPT1,ANPEP,THY1,B4GALT1,MYDGF,SASH1,SPRED1,SHC1,GREM1,CLIC4	down_VCAV_EC

GO:0051960	0.0898	0.973	4	216	regulation of nervous system development	BP	1	HOOK3,ARHGEF2,SEMA3A,RNF6	up_VPOR_EC
GO:0070887	0.0898	0.982	3	775	cellular response to chemical stimulus	BP	1	CYP1A1,GATA5,CA2	up_VCAV_VSMC
GO:0009141	0.0898	0.997	1	110	nucleoside triphosphate metabolic process	BP	1	AK5	down_VSAP_VSMC
GO:0048841	0.0898	0.997	1	4	regulation of axon extension involved in	BP	1	PLXNA4	up_AFEM_EC
GO:0045844	0.0898	0.996	1	24	positive regulation of striated muscle tissue	BP	1	TBX5	up_APUL_EC
GO:0048636	0.0898	0.996	1	24	positive regulation of muscle organ	BP	1	TBX5	up_APUL_EC
GO:2000009	0.089811381	0.99687	1	4	negative regulation of protein localization to cell	BP	1	ACTN2	up_ACOR_EC
GO:0061448	0.089817182	0.96835	5	89	connective tissue development	BP	1	NR1H4,SOX9,PTH1R,FOXC2,EFEMP1	up_ACOR_EC
GO:0048639	0.089888664	0.97886	3	39	positive regulation of developmental growth	BP	1	PLS1,TBX5,NRP1	up_ACOR_EC
GO:1903707	0.0899	0.969	5	48	negative regulation of hemopoiesis	BP	1	FBN1,GLI3,ZFP36L1,LOXL3,ZBTB16	down_VCAV_EC
GO:0060045	0.0899	0.981	3	11	positive regulation of cardiac muscle cell	BP	1	FGFR2,TBX20,TBX5	up_ACOR_VSMC
GO:0045876	0.0899	0.989	2	6	positive regulation of sister chromatid cohesion	BP	0.936	SMC5,RAD21	up_VCAV_EC
GO:1904772	0.0899	1	1	1	response to tetrachloromethane	BP	1	EZH2	down_ACOR_VSMC
GO:0036333	0.0899	1	1	1	hepatocyte homeostasis	BP	1	EZH2	down_ACOR_VSMC
GO:0001763	0.0899	0.996	1	78	morphogenesis of a branching structure	BP	1	ESR1	up_AORT_VSMC
GO:0035306	0.0899	0.996	1	24	positive regulation of dephosphorylation	BP	1	NPNT	up_APUL_EC
GO:0051668	0.0899	0.996	1	39	localization within membrane	BP	1	NETO1	up_VFEM_VSMC
GO:0045664	0.089995275	0.96159	7	144	regulation of neuron differentiation	BP	1	BRSK1,SOX9,DIXDC1,RGMA,PAK3,LRP4,NRP1	up_ACOR_EC
GO:0050866	0.09	0.969	5	48	negative regulation of cell activation	BP	1	PRNP,SDC4,GLI3,BPI,LOXL3	down_VCAV_EC
GO:1903169	0.09	0.973	4	34	regulation of calcium ion transmembrane transport	BP	1	PRNP,CACNB3,THY1,CYBA	down_VCAV_EC
GO:0006122	0.09	0.989	2	7	mitochondrial electron transport, ubiquinol to	BP	1	CYCS,CYC1	down_ACOR_VSMC
GO:0003231	0.09	0.996	1	48	cardiac ventricle development	BP	1	CITED2	down_VPOR_EC
GO:0007210	0.09	1	1	1	serotonin receptor signaling pathway	BP	1	HTR2B	up_ACOR_VSMC
GO:0014827	0.09	1	1	1	intestine smooth muscle contraction	BP	1	HTR2B	up_ACOR_VSMC
GO:0071502	0.09	1	1	1	cellular response to temperature stimulus	BP	1	HTR2B	up_ACOR_VSMC
GO:0110026	0.09	1	1	1	regulation of DNA strand resection involved in replication fork processing	BP	1	ZNF365	up_ACOR_VSMC
GO:0010513	0.09	1	1	1	positive regulation of phosphatidylinositol biosynthetic process	BP	1	HTR2B	up_ACOR_VSMC
GO:0006954	0.09000216	0.96157	7	147	inflammatory response	BP	1	NR1H4,FNDC4,NOD2,IL33,PTGER4,CLSTN1,RARRES2	up_ACOR_EC
GO:0042594	0.0901	0.956	10	64	response to starvation	BP	1	PRKAA2,GABARAPL1,CDKN1A,ULK2,SESN3,PIK3C3,LAMP2,SESN1,BMT2,ZFYVE1	up_ACOR_VSMC
GO:0070229	0.0901	0.981	3	13	negative regulation of lymphocyte apoptotic	BP	1	AURKB,ARG2,NOC2L	down_ACOR_VSMC
GO:0046133	0.0901	0.998	1	2	pyrimidine ribonucleoside catabolic process	BP	0.98	ENTPD4	down_VSAP_EC
GO:0046135	0.0901	0.998	1	2	pyrimidine nucleoside catabolic process	BP	0.98	ENTPD4	down_VSAP_EC
GO:0001866	0.0901	1	1	1	NK T cell proliferation	BP	0.937	ELF4	up_VCAV_EC
GO:0071248	0.0901	0.996	1	35	cellular response to metal ion	BP	1	FBP1	up_VFEM_VSMC
GO:0045597	0.0902	0.987	2	253	positive regulation of cell differentiation	BP	1	TBX5,GATA5	up_VPUL_VSMC
GO:0003279	0.0902	0.996	1	48	cardiac septum development	BP	1	CITED2	down_VPOR_EC
GO:0032104	0.0902	0.997	1	4	regulation of response to extracellular stimulus	BP	1	LEP	up_AFEM_EC
GO:0032107	0.0902	0.997	1	4	regulation of response to nutrient levels	BP	1	LEP	up_AFEM_EC
GO:0061298	0.0903	0.988	2	8	retina vasculature development in camera-	BP	0.98	HIF1A,NRP1	down_VSAP_EC

GO:0047484	0.0903	0.99	2	5	regulation of response to osmotic stress	BP	0.938	PTGS2,ARHGEF2	up_VCAV_EC
GO:0061724	0.0903	0.997	1	4	lipophagy	BP	1	RAB7A	up_AFEM_EC
GO:2000392	0.0903	0.997	1	4	regulation of lamellipodium	BP	1	KANK1	up_AFEM_EC
GO:0008543	0.0903	0.996	1	27	fibroblast growth factor receptor signaling	BP	1	FLRT3	up_VPUL_EC
GO:0034220	0.0904	0.949	14	188	ion transmembrane transport	BP	1	PRNP,FOLR2,FXYD1,AKT2,SLC39A6,CACNB3,ATP5MC1,THY1,ATP6V0C,PARK7,SCARA5,CYBA,ARHGAP1,CLIC4	down_VCAV_EC
GO:0007605	0.0904	0.97	5	27	sensory perception of sound	BP	0.938	KIT,CHD7,NAV2,SPTBN4,P2RX2	up_VCAV_EC
GO:0042108	0.0904	0.98	3	14	positive regulation of cytokine biosynthetic	BP	0.938	TLR4,TLR3,BCL10	up_VCAV_EC
GO:0002523	0.0904	1	1	1	leukocyte migration involved in inflammatory	BP	1	JAM3	up_ACOR_VSMC
GO:0061740	0.0904	1	1	1	protein targeting to lysosome involved in chaperone-mediated	BP	1	LAMP2	up_ACOR_VSMC
GO:0071211	0.0904	1	1	1	protein targeting to vacuole involved in	BP	1	LAMP2	up_ACOR_VSMC
GO:0090138	0.0904	1	1	1	regulation of actin cytoskeleton organization	BP	1	JAM3	up_ACOR_VSMC
GO:0007368	0.0904	0.996	1	37	determination of left/right symmetry	BP	1	NKX2-5	up_VPOR_VSMC
GO:0033500	0.0905	0.962	7	64	carbohydrate homeostasis	BP	0.98	NR1H4,PTPN11,PRCP,SMARCA4,SESN2,HIF1A,PTPN2	down_VSAP_EC
GO:0042593	0.0905	0.962	7	64	glucose homeostasis	BP	0.98	NR1H4,PTPN11,PRCP,SMARCA4,SESN2,HIF1A,PTPN2	down_VSAP_EC
GO:2000106	0.0905	0.97	5	29	regulation of leukocyte apoptotic process	BP	1	PRELID1,AURKB,ARG2,NOC2L,PIK3CD	down_ACOR_VSMC
GO:0019216	0.0905	0.986	2	101	regulation of lipid metabolic process	BP	1	LYN,ACSL3	down_ACOR_EC
GO:0033210	0.0905	0.997	1	4	leptin-mediated signaling pathway	BP	1	LEP	up_AFEM_EC
GO:0044320	0.0905	0.997	1	4	cellular response to leptin stimulus	BP	1	LEP	up_AFEM_EC
GO:0021954	0.0905	0.996	1	26	central nervous system neuron development	BP	1	NTRK2	up_APUL_EC
GO:0009636	0.0905	0.996	1	66	response to toxic substance	BP	1	CYP1A1	up_VCAV_VSMC
GO:1902622	0.0905	0.996	1	11	regulation of neutrophil migration	BP	1	C3AR1	up_VPOR_EC
GO:0007018	0.0906	0.954	11	82	microtubule-based movement	BP	1	KIF2A,CFAP298,CENPE,KIF22,TRAK1,KIF4A,KIF15,KIF11,TTLL1,KIF21B,KIF24	down_ACOR_VSMC
GO:0021543	0.0906	0.965	6	49	pallium development	BP	0.98	HIF1A,UBA6,CTNNB1,GSK3B,DISC1,NRP1	down_VSAP_EC
GO:0035304	0.0906	0.974	4	29	regulation of protein dephosphorylation	BP	0.98	NUAK1,PPP1R12A,HTT,CALM1	down_VSAP_EC
GO:0061549	0.0906	0.99	2	5	sympathetic ganglion development	BP	1	NRP1,FZD3	up_ACOR_VSMC
GO:0070228	0.0906	0.996	1	20	regulation of lymphocyte apoptotic process	BP	1	LYN	down_ACOR_EC
GO:0002313	0.0906	0.997	1	4	mature B cell differentiation involved in	BP	1	DLL1	up_AFEM_EC
GO:0002335	0.0906	0.997	1	4	mature B cell differentiation	BP	1	DLL1	up_AFEM_EC
GO:1903510	0.0906	0.996	1	22	mucopolysaccharide metabolic process	BP	1	CHST7	up_VFEM_EC
GO:0031349	0.090605566	0.96798	5	90	positive regulation of defense response	BP	1	NR1H4,NOD2,IL33,PTGER4,MAVS	up_ACOR_EC
GO:0051893	0.090650158	0.98658	2	20	regulation of focal adhesion assembly	BP	1	ARHGAP6,NRP1	up_ACOR_EC
GO:0090109	0.090650158	0.98658	2	20	regulation of cell-substrate junction	BP	1	ARHGAP6,NRP1	up_ACOR_EC
GO:0000904	0.0907	0.942	24	181	cell morphogenesis involved in differentiation	BP	1	EPYC,FEZ1,FGFR2,SEC24B,LATS2,ZNF365,ARHGEF26,NRP1,ULK2,UNC5B,SPINT2,SLITRK5,FZD3,DAG1,APP,PSEN1,SIPA1L1,SSH3,FEZ2,WASL,DOCK5,BVES,RREB1,CRKL	up_ACOR_VSMC
GO:0007492	0.0907	0.974	4	27	endoderm development	BP	0.98	CTNNB1,EXT1,INHBA,COL12A1	down_VSAP_EC
GO:0050777	0.0907	0.974	4	28	negative regulation of immune response	BP	0.98	SOCS5,PTPN2,CNOT7,A2M	down_VSAP_EC
GO:0042761	0.0907	0.989	2	7	very long-chain fatty acid biosynthetic process	BP	1	ELOVL1,ELOVL6	down_ACOR_VSMC
GO:0048704	0.0907	0.996	1	46	embryonic skeletal system morphogenesis	BP	1	TBX15	down_VPOR_EC
GO:0015732	0.0907	0.997	1	4	prostaglandin transport	BP	1	LEP	up_AFEM_EC

GO:0003170	0.0907	0.996	1	25	heart valve development	BP	1	TBX5	up_APUL_EC
GO:0043200	0.0907	0.996	1	26	response to amino acid	BP	1	NTRK2	up_APUL_EC
GO:0048738	0.090714758	0.97256	4	64	cardiac muscle tissue development	BP	1	ACTN2,FOXC2,TBX5,C23H3orf58	up_ACOR_EC
GO:0090169	0.0908	0.981	3	13	regulation of spindle assembly	BP	1	SMC1A,SMC3,STAG1	down_ACOR_VSMC
GO:0001568	0.0908	0.979	3	252	blood vessel development	BP	1	EYA1,HOXA7,SIX1	up_VFEM_EC
GO:0043043	0.0908	0.987	2	247	peptide biosynthetic process	BP	1	DARS,EIF3M	down_AFEM_EC
GO:1900017	0.0908	0.989	2	6	positive regulation of cytokine production involved in inflammatory	BP	0.938	GBP5,TLR4	up_VCAV_EC
GO:0043484	0.0908	0.996	1	43	regulation of RNA splicing	BP	1	SRSF6	down_AFEM_EC
GO:0070836	0.0908	0.998	1	2	caveola assembly	BP	0.938	CAV2	up_VCAV_EC
GO:0019362	0.0908	0.996	1	35	pyridine nucleotide metabolic process	BP	1	FBP1	up_VFEM_VSMC
GO:0046496	0.0908	0.996	1	35	nicotinamide nucleotide metabolic process	BP	1	FBP1	up_VFEM_VSMC
GO:0043535	0.0909	0.978	3	45	regulation of blood vessel endothelial cell migration	BP	1	ANGPT4,TBXA2R,ADAM17	up_AFEM_EC
GO:0046325	0.0909	0.997	1	4	negative regulation of glucose import	BP	1	LEP	up_AFEM_EC
GO:0043112	0.091	0.96	8	57	receptor metabolic process	BP	0.938	SELE,CXCL8,CALCRL,EDN1,PLCG2,HIF1A,ALS2,LMTK2	up_VCAV_EC
GO:0021987	0.091	0.969	5	37	cerebral cortex development	BP	0.982	HIF1A,CTNNB1,GSK3B,DISC1,NRP1	down_VSAP_EC
GO:0051051	0.091	0.986	2	112	negative regulation of transport	BP	1	HOXA3,NEDD4	down_ACOR_EC
GO:0046467	0.091	0.996	1	45	membrane lipid biosynthetic process	BP	1	GBGT1	down_VPOR_EC
GO:0000122	0.091	0.997	1	218	negative regulation of transcription by RNA polymerase II	BP	1	MOSPD1	down_VFEM_EC
GO:0002583	0.091	1	1	1	regulation of antigen processing and presentation of peptide	BP	1	TAPBPL	up_ACOR_VSMC
GO:0002584	0.091	1	1	1	negative regulation of antigen processing and presentation of peptide	BP	1	TAPBPL	up_ACOR_VSMC
GO:0002589	0.091	1	1	1	regulation of antigen processing and presentation of peptide	BP	1	TAPBPL	up_ACOR_VSMC
GO:0002590	0.091	1	1	1	negative regulation of antigen processing and presentation of peptide	BP	1	TAPBPL	up_ACOR_VSMC
GO:0021527	0.091	1	1	1	spinal cord association neuron differentiation	BP	1	LMO4	up_ACOR_VSMC
GO:0044241	0.091	0.997	1	4	lipid digestion	BP	1	LEP	up_AFEM_EC
GO:0045793	0.091	0.997	1	4	positive regulation of cell size	BP	1	PRR16	up_AFEM_EC
GO:0043031	0.091	0.997	1	4	negative regulation of macrophage activation	BP	1	BPI	up_AFEM_EC
GO:0090304	0.0911	0.957	11	1694	nucleic acid metabolic process	BP	1	HOXA11,TBX15,HOXA10,HOXA9,PITX1,EMX2,SALL1,IRX5,TLR2,CAV1,SHOX2	up_VSAP_VSMC
GO:0070887	0.0911	0.956	10	775	cellular response to chemical stimulus	BP	1	C3AR1,ARHGEF2,ENSCAFG00000002015,ASPN,ACVR2A,FBP1,MTDH,ENSCAFG00000032731,GNPNAT1,RNF6	up_VPOR_EC
GO:0006725	0.0911	0.965	8	1956	cellular aromatic compound metabolic	BP	1	EYA1,HOXA10,HOXA9,TBX15,FBP1,EMX2,HOXA11,SHOX2	up_VFEM_VSMC
GO:0042594	0.0911	0.962	7	64	response to starvation	BP	0.983	ASNS,HSPA5,SESN2,DDIT3,ATF3,EIF2S1,CDKN1A	down_VSAP_EC
GO:0001910	0.0911	0.981	3	12	regulation of leukocyte mediated cytotoxicity	BP	1	LEP,F2RL1,B2M	up_ACOR_VSMC
GO:0006740	0.0911	1	1	1	NADPH regeneration	BP	1	NNT	down_ACOR_VSMC
GO:0044057	0.0911	0.996	1	110	regulation of system process	BP	1	GATA5	down_VSAP_VSMC
GO:0010643	0.0911	1	1	1	cell communication by chemical coupling	BP	1	GJA1	up_ACOR_VSMC
GO:0060156	0.0911	1	1	1	milk ejection reflex	BP	1	GJA1	up_ACOR_VSMC
GO:1903753	0.0911	0.997	1	4	negative regulation of p38MAPK cascade	BP	1	CYLD	up_AFEM_EC

GO:2000647	0.0911	0.997	1	4	negative regulation of stem cell proliferation	BP	1	OVOL1	up_AFEM_EC
GO:0043271	0.0911	0.996	1	28	negative regulation of ion transport	BP	1	HOXA3	up_VPUL_EC
GO:0023057	0.0912	0.934	42	409	negative regulation of signaling	BP	0.938	CXCL8,TNFAIP3,TNIP1,PDE4D,FERMT1,ATF3,DLL4,RAPGEF1,TLR4,NLRC5,GATA3,APLNR,ARAP3,HOMER3,EGFL7,FIGNL1,CASP8,MTOR,BMP2,HHEX,PREX2,GDPD5,RIOK3,STMN1,MCC,SMARCA4,APOD,NCK1,OTUD4,PTGS2,CTDSPL2,CTNNA1,CNOT7,RIPK1,ITGA6,RGS2,SKIL,HIF1A,TNFAIP1,IVNS1ABP,ARHGEF2,PEAR1	up_VCAV_EC
GO:0007160	0.0912	0.957	9	56	cell-matrix adhesion	BP	1	ACTN2,FERMT1,NRP1,NPNT,JUP,DUSP22,NID1,FAM107A,JAM3	up_ACOR_VSMC
GO:0045879	0.0912	0.987	2	10	negative regulation of smoothened signaling	BP	1	GLI3,GLIS2	down_VCAV_EC
GO:2000765	0.0912	0.989	2	6	regulation of cytoplasmic translation	BP	0.938	NCK1,DPH5	up_VCAV_EC
GO:0008015	0.0912	0.986	2	120	blood circulation	BP	1	MAP2K6,AVPR1A	up_VFEM_EC
GO:0072132	0.0912	0.996	1	23	mesenchyme morphogenesis	BP	1	ACVRL1	down_ACOR_EC
GO:0070970	0.0912	0.997	1	4	interleukin-2 secretion	BP	1	GPAM	up_AFEM_EC
GO:0002194	0.0912	1	1	1	hepatocyte cell migration	BP	0.938	PROX1	up_VCAV_EC
GO:0061114	0.0912	1	1	1	branching involved in pancreas morphogenesis	BP	0.938	PROX1	up_VCAV_EC
GO:0090425	0.0912	1	1	1	acinar cell differentiation	BP	0.938	PROX1	up_VCAV_EC
GO:2000977	0.0912	1	1	1	regulation of forebrain neuron differentiation	BP	0.938	PROX1	up_VCAV_EC
GO:2000979	0.0912	1	1	1	positive regulation of forebrain neuron	BP	0.938	PROX1	up_VCAV_EC
GO:0060414	0.0912	1	1	1	aorta smooth muscle tissue morphogenesis	BP	0.938	PROX1	up_VCAV_EC
GO:0060849	0.0912	1	1	1	regulation of transcription involved in lymphatic endothelial cell fate	BP	0.938	PROX1	up_VCAV_EC
GO:0002694	0.0913	0.946	17	126	regulation of leukocyte activation	BP	1	CD274,TBC1D10C,ABL1,ZBTB16,TOX,STAT6,F2RL1,ZFP36L1,ZEB1,MYD88,FBXO7,CD83,ZFP36L2,PNP,STX4,NCK1,TGFBR2	up_VSAP_EC
GO:1901888	0.0913	0.973	4	29	regulation of cell junction assembly	BP	0.983	VEGFA,MYO1C,ENSCAFG00000002015,NRP1	down_VSAP_EC
GO:0007063	0.0913	0.981	3	12	regulation of sister chromatid cohesion	BP	0.938	SMC5,RAD21,ATRX	up_VCAV_EC
GO:0090110	0.0913	0.988	2	8	cargo loading into COPII-coated vesicle	BP	0.983	SEC24D,SEC13	down_VSAP_EC
GO:0035459	0.0913	0.988	2	8	cargo loading into vesicle	BP	0.983	SEC24D,SEC13	down_VSAP_EC
GO:0042754	0.0913	0.997	1	4	negative regulation of circadian rhythm	BP	1	CRY2	up_AFEM_EC
GO:1900119	0.0913	0.997	1	4	positive regulation of execution phase of	BP	1	FAP	up_AFEM_EC
GO:0023057	0.0914	0.933	48	409	negative regulation of signaling	BP	1	SMAD6,LEP,LBH,HTR2B,RASSF2,PRKAA2,MLLT3,FERMT1,BMP4,ELF1,CASTOR1,LATS2,ZNRF3,AR,PTGIR,NDRG2,ITPR1,F2RL1,DAPK1,CLSTN1,CREBRF,NRP1,UBR1,PTPN22,UNC5B,SMPD1,APLP1,TBX20,SESN3,SH3RF1,CCDC3,RGS2,DAG1,PSEN1,SYNJ2BP,WWC2,DUSP22,SESN1,DUSP26,LRRC4,DDIT4,HERPUD1,INPP5K,BMT2,PFKL,WFS1,SH3BP4,PARK7	up_ACOR_VSMC
GO:0099536	0.0914	0.947	16	133	synaptic signaling	BP	0.938	EPHB1,PLK2,ENSCAFG00000007112,RELN,MEF2C,EDN1,SNCAIP,HOMER3,PPP3CA,GUCY1A1,SNAP23,SLC12A7,GRM8,STX11,ALS2,P2RX2	up_VCAV_EC
GO:0002700	0.0914	0.962	7	40	regulation of production of molecular mediator of immune response	BP	1	TP53BP1,TLR3,F2RL1,PTPN22,B2M,MAP3K7,IL18R1	up_ACOR_VSMC
GO:0043030	0.0914	0.981	3	12	regulation of macrophage activation	BP	0.938	ADGRF5,TLR4,CD200	up_VCAV_EC
GO:0043117	0.0914	1	1	1	positive regulation of vascular permeability	BP	1	TRPV4	down_ACOR_VSMC
GO:0001867	0.0914	0.998	1	2	complement activation, lectin pathway	BP	1	A2M	down_VCAV_EC
GO:0051775	0.0914	0.998	1	2	response to redox state	BP	1	VASN	down_VCAV_EC
GO:0007617	0.0914	0.997	1	4	mating behavior	BP	1	THRB	up_AFEM_EC

GO:0009309	0.0914	0.997	1	4	amine biosynthetic process	BP	1	MOXD1	up_AFEM_EC
GO:0042401	0.0914	0.997	1	4	cellular biogenic amine biosynthetic process	BP	1	MOXD1	up_AFEM_EC
GO:0045742	0.0914	0.997	1	4	positive regulation of epidermal growth factor receptor signaling	BP	1	ADAM17	up_AFEM_EC
GO:1904683	0.0914	0.997	1	4	regulation of metalloendopeptidase	BP	1	RECK	up_AFEM_EC
GO:0001704	0.0914	0.996	1	43	formation of primary germ layer	BP	1	DUSP2	up_AORT_EC
GO:0090630	0.0914	0.996	1	23	activation of GTPase activity	BP	1	TIAM1	up_VFEM_EC
GO:0019915	0.0914	0.996	1	22	lipid storage	BP	1	CAV1	up_VSAP_VSMC
GO:0042310	0.0914	0.996	1	21	vasoconstriction	BP	1	CAV1	up_VSAP_VSMC
GO:0097756	0.0914	0.996	1	21	negative regulation of blood vessel diameter	BP	1	CAV1	up_VSAP_VSMC
GO:0032231	0.0915	0.966	6	32	regulation of actin filament bundle assembly	BP	1	SYNPO2L,ARHGAP28,SYNPO2,NRP1,EVL,INPP5K	up_ACOR_VSMC
GO:0045056	0.0915	0.99	2	5	transcytosis	BP	1	LRPAP1,RAB11B	up_ACOR_VSMC
GO:0030949	0.0915	0.989	2	6	positive regulation of vascular endothelial growth factor receptor	BP	0.938	PRKD2,HIF1A	up_VCAV_EC
GO:0071310	0.0915	1	1	634	cellular response to organic substance	BP	1	GATA5	down_AFEM_VSMC
GO:0099171	0.0915	1	1	1	presynaptic modulation of chemical synaptic	BP	1	FBXL20	up_ACOR_VSMC
GO:0099575	0.0915	1	1	1	regulation of protein catabolic process at presynapse, modulating	BP	1	FBXL20	up_ACOR_VSMC
GO:1905208	0.0915	0.997	1	4	negative regulation of cardiocyte differentiation	BP	1	DLL1	up_AFEM_EC
GO:0034446	0.0915	0.996	1	23	substrate adhesion-dependent cell spreading	BP	1	RADIL	up_VFEM_EC
GO:0006813	0.0915	0.996	1	37	potassium ion transport	BP	1	NETO1	up_VFEM_VSMC
GO:0032677	0.0916	0.979	3	22	regulation of interleukin-8 production	BP	1	PARK7,BPI,MAVS	down_VCAV_EC
GO:0046777	0.0916	0.986	2	86	protein autophosphorylation	BP	1	LYN,IGF1R	down_ACOR_EC
GO:0071495	0.0916	0.987	2	365	cellular response to endogenous stimulus	BP	1	GATA5,CA2	up_VCAV_VSMC
GO:0001993	0.0916	1	1	1	regulation of systemic arterial blood pressure by norepinephrine-	BP	1	ADRA1B	up_ACOR_VSMC
GO:0001994	0.0916	1	1	1	norepinephrine-epinephrine vasoconstriction involved in regulation of systemic	BP	1	ADRA1B	up_ACOR_VSMC
GO:0003321	0.0916	1	1	1	positive regulation of blood pressure by epinephrine-	BP	1	ADRA1B	up_ACOR_VSMC
GO:0009952	0.0916	0.996	1	66	anterior/posterior pattern specification	BP	1	HOXC6	up_VCAV_VSMC
GO:0055092	0.0916	0.996	1	28	sterol homeostasis	BP	1	NR1H4	up_VPUL_EC
GO:0016561	0.0916	1	1	1	protein import into peroxisome matrix,	BP	1	PEX6	up_VSAP_EC
GO:0032103	0.0917	0.957	9	69	positive regulation of response to external	BP	0.939	GBP5,CXCL8,PDGFB,TLR4,PRKD2,SWAP70,TLR3,PTGS2,HMGB1	up_VCAV_EC
GO:0051924	0.0917	0.964	6	53	regulation of calcium ion transport	BP	0.985	REM1,CTNNB1,LYN,HTT,CALM1,HOMER1	down_VSAP_EC
GO:0043551	0.0917	0.996	1	17	regulation of phosphatidylinositol 3-	BP	1	LYN	down_ACOR_EC
GO:0042661	0.0917	0.998	1	2	regulation of mesodermal cell fate specification	BP	1	BMPR1A	down_VCAV_EC
GO:0048369	0.0917	0.998	1	2	lateral mesoderm morphogenesis	BP	1	BMPR1A	down_VCAV_EC
GO:0048370	0.0917	0.998	1	2	lateral mesoderm formation	BP	1	BMPR1A	down_VCAV_EC
GO:0048371	0.0917	0.998	1	2	lateral mesodermal cell differentiation	BP	1	BMPR1A	down_VCAV_EC
GO:0048372	0.0917	0.998	1	2	lateral mesodermal cell fate commitment	BP	1	BMPR1A	down_VCAV_EC
GO:0048377	0.0917	0.998	1	2	lateral mesodermal cell fate specification	BP	1	BMPR1A	down_VCAV_EC

GO:0048378	0.0917	0.998	1	2	regulation of lateral mesodermal cell fate	BP	1	BMPR1A	down_VCAV_EC
GO:1905770	0.0917	0.998	1	2	regulation of mesodermal cell differentiation	BP	1	BMPR1A	down_VCAV_EC
GO:1905902	0.0917	0.998	1	2	regulation of mesoderm formation	BP	1	BMPR1A	down_VCAV_EC
GO:0034349	0.0917	0.997	1	4	glial cell apoptotic process	BP	1	DLL1	up_AFEM_EC
GO:0046321	0.0917	0.997	1	4	positive regulation of fatty acid oxidation	BP	1	PPARGC1A	up_AFEM_EC
GO:0048505	0.0917	0.997	1	4	regulation of timing of cell differentiation	BP	1	DLL1	up_AFEM_EC
GO:1900025	0.0917	0.997	1	4	negative regulation of substrate adhesion-dependent cell spreading	BP	1	KANK1	up_AFEM_EC
GO:0030154	0.0918	0.964	7	1040	cell differentiation	BP	1	TBX5,MAPK10,ARHGEF26,SLITRK5,WIF1,NPNT,NTRK2	up_APUL_EC
GO:0043651	0.0918	1	1	1	linoleic acid metabolic process	BP	1	CYP2J2	up_ACOR_VSMC
GO:0090107	0.0918	1	1	1	regulation of high-density lipoprotein particle	BP	1	LCAT	up_ACOR_VSMC
GO:0034505	0.0918	0.997	1	4	tooth mineralization	BP	1	CNNM4	up_AFEM_EC
GO:0099173	0.0919	0.962	7	40	postsynapse organization	BP	1	GLRB,ZNF365,TMEM108,EGLN1,NRP1,LRRC4,WASL	up_ACOR_VSMC
GO:0060393	0.0919	0.979	3	19	regulation of pathway-restricted SMAD protein	BP	0.986	SNX25,STRAP,INHBA	down_VSAP_EC
GO:0033316	0.0919	1	1	1	meiotic spindle assembly checkpoint	BP	1	TTK	down_ACOR_VSMC
GO:0044779	0.0919	1	1	1	meiotic spindle checkpoint	BP	1	TTK	down_ACOR_VSMC
GO:0044785	0.0919	1	1	1	metaphase/anaphase transition of meiotic cell	BP	1	TTK	down_ACOR_VSMC
GO:1902102	0.0919	1	1	1	regulation of metaphase/anaphase transition of meiotic cell	BP	1	TTK	down_ACOR_VSMC
GO:1902103	0.0919	1	1	1	negative regulation of metaphase/anaphase transition of meiotic cell	BP	1	TTK	down_ACOR_VSMC
GO:1903096	0.0919	1	1	1	protein localization to meiotic spindle midzone	BP	1	TTK	down_ACOR_VSMC
GO:1905132	0.0919	1	1	1	regulation of meiotic chromosome separation	BP	1	TTK	down_ACOR_VSMC
GO:1905133	0.0919	1	1	1	negative regulation of meiotic chromosome	BP	1	TTK	down_ACOR_VSMC
GO:1905359	0.0919	1	1	1	protein localization to meiotic spindle	BP	1	TTK	down_ACOR_VSMC
GO:0030072	0.0919	0.996	1	48	peptide hormone secretion	BP	1	RAB11B	down_VPOR_EC
GO:0010715	0.0919	0.997	1	4	regulation of extracellular matrix disassembly	BP	1	FAP	up_AFEM_EC
GO:0044058	0.0919	0.997	1	4	regulation of digestive system process	BP	1	LEP	up_AFEM_EC
GO:0046337	0.0919	0.997	1	4	phosphatidylethanolamine metabolic process	BP	1	NAPEPLD	up_AFEM_EC
GO:2000831	0.0919	0.997	1	4	regulation of steroid hormone secretion	BP	1	CRY2	up_AFEM_EC
GO:0072507	0.092	0.954	10	103	divalent inorganic cation homeostasis	BP	0.986	SLC39A7,ERO1A,ATP2B1,TMTC2,APOE,LYN,DDIT3,HTT,CALM1,DISC1	down_VSAP_EC
GO:0000956	0.092	0.959	8	55	nuclear-transcribed mRNA catabolic process	BP	1	RNPS1,EIF4A3,EDC3,THRAP3,EDC4,SMG9,ENSCAFG00000015189,EXOSC5	down_ACOR_VSMC
GO:0051094	0.092	0.964	6	388	positive regulation of developmental process	BP	1	C3AR1,ARHGEF2,ENSCAFG00000002015,SEMA3A,ACVR2A,MTDH	up_VPOR_EC
GO:0033314	0.092	0.989	2	6	mitotic DNA replication checkpoint	BP	0.942	RAD17,TOPBP1	up_VCAV_EC
GO:2001233	0.092	0.986	2	149	regulation of apoptotic signaling pathway	BP	1	PTGER2,ARHGEF2	up_VPUL_EC
GO:0014850	0.092	0.997	1	4	response to muscle activity	BP	1	PPARGC1A	up_AFEM_EC
GO:0030502	0.092	0.997	1	4	negative regulation of bone mineralization	BP	1	GREM1	up_AFEM_EC
GO:0051923	0.092	0.997	1	4	sulfation	BP	1	TPST2	up_AFEM_EC
GO:2001135	0.092	0.997	1	4	regulation of endocytic recycling	BP	1	BVES	up_AFEM_EC
GO:0050806	0.092	0.996	1	25	positive regulation of synaptic transmission	BP	1	NTRK2	up_APUL_EC
GO:0021510	0.092	0.996	1	23	spinal cord development	BP	1	HOXC10	up_VFEM_EC

GO:0006754	0.092	0.996	1	36	ATP biosynthetic process	BP	1	FBP1	up_VFEM_VSMC
GO:0035115	0.0921	0.98	3	12	embryonic forelimb morphogenesis	BP	1	RECK,LNPK,TBX5	up_ACOR_VSMC
GO:0007220	0.0921	0.997	1	4	Notch receptor processing	BP	1	ADAM17	up_AFEM_EC
GO:0014819	0.0921	0.997	1	4	regulation of skeletal muscle contraction	BP	1	DMPK	up_AFEM_EC
GO:0042118	0.0921	0.997	1	4	endothelial cell activation	BP	1	HOXA9	up_AFEM_EC
GO:0060839	0.0921	0.997	1	4	endothelial cell fate commitment	BP	1	DLL1	up_AFEM_EC
GO:0072148	0.0921	0.997	1	4	epithelial cell fate commitment	BP	1	DLL1	up_AFEM_EC
GO:0040029	0.0921	0.996	1	74	regulation of gene expression, epigenetic	BP	1	ESR1	up_AORT_VSMC
GO:0010906	0.0921	0.996	1	38	regulation of glucose metabolic process	BP	1	FBP1	up_VFEM_VSMC
GO:0055065	0.0922	0.949	13	145	metal ion homeostasis	BP	0.987	SLC39A7,ERO1A,ATP2B1,TMTC2,HIF1A,NEO1,TFRC,APOE,LYN,DDIT3,HTT,CALM1,DISC1	down_VSAP_EC
GO:0071706	0.0922	0.962	7	40	tumor necrosis factor superfamily cytokine	BP	1	TLR1,LEP,TLR3,CD274,PTPN22,APP,NOD2	up_ACOR_VSMC
GO:1903432	0.0922	0.974	4	18	regulation of TORC1 signaling	BP	1	CASTOR1,SESN3,SESN1,BMT2	up_ACOR_VSMC
GO:0021517	0.0922	0.98	3	12	ventral spinal cord development	BP	0.942	RELN,DLL4,GDPD5	up_VCAV_EC
GO:1901532	0.0922	0.989	2	6	regulation of hematopoietic progenitor	BP	1	HSPA9,PDCD2	down_ACOR_VSMC
GO:0032530	0.0922	0.989	2	6	regulation of microvillus organization	BP	1	FSCN1,EZR	down_ACOR_VSMC
GO:0034105	0.0922	0.989	2	6	positive regulation of tissue remodeling	BP	1	B4GALT1,TFRC	down_ACOR_VSMC
GO:0018198	0.0922	0.987	2	10	peptidyl-cysteine modification	BP	1	CLIP3,PARK7	down_VCAV_EC
GO:0001913	0.0922	0.987	2	10	T cell mediated cytotoxicity	BP	0.987	FAM49B,PVR	down_VSAP_EC
GO:0010768	0.0922	1	1	1	negative regulation of transcription from RNA polymerase II promoter in response to UV-induced DNA damage	BP	1	NEDD4	down_ACOR_VSMC
GO:0019089	0.0922	1	1	1	transmission of virus	BP	1	NEDD4	down_ACOR_VSMC
GO:0044007	0.0922	1	1	1	dissemination or transmission of symbiont	BP	1	NEDD4	down_ACOR_VSMC
GO:0044111	0.0922	1	1	1	development involved in symbiotic interaction	BP	1	NEDD4	down_ACOR_VSMC
GO:0051821	0.0922	1	1	1	dissemination or transmission of organism from other organism	BP	1	NEDD4	down_ACOR_VSMC
GO:0098957	0.0922	1	1	1	anterograde axonal transport of	BP	1	TRAK1	down_ACOR_VSMC
GO:0051562	0.0922	1	1	1	negative regulation of mitochondrial calcium ion concentration	BP	1	MICU2	up_ACOR_VSMC
GO:0003256	0.0922	0.997	1	4	regulation of transcription from RNA polymerase II promoter involved in myocardial precursor cell	BP	1	GREM1	up_AFEM_EC
GO:0002332	0.0922	1	1	1	transitional stage B cell differentiation	BP	1	ABL1	up_VSAP_EC
GO:0002333	0.0922	1	1	1	transitional one stage B cell differentiation	BP	1	ABL1	up_VSAP_EC
GO:0010519	0.0922	1	1	1	negative regulation of phospholipase activity	BP	1	ABL1	up_VSAP_EC
GO:0090135	0.0922	1	1	1	actin filament branching	BP	1	ABL1	up_VSAP_EC
GO:1900042	0.0922	1	1	1	positive regulation of interleukin-2 secretion	BP	1	ABL1	up_VSAP_EC
GO:1900275	0.0922	1	1	1	negative regulation of phospholipase C activity	BP	1	ABL1	up_VSAP_EC
GO:1902715	0.0922	1	1	1	positive regulation of interferon-gamma	BP	1	ABL1	up_VSAP_EC
GO:1904529	0.0922	1	1	1	regulation of actin filament binding	BP	1	ABL1	up_VSAP_EC
GO:1904531	0.0922	1	1	1	positive regulation of actin filament binding	BP	1	ABL1	up_VSAP_EC
GO:1904616	0.0922	1	1	1	regulation of actin binding	BP	1	ABL1	up_VSAP_EC

GO:1904618	0.0922	1	1	1	positive regulation of actin binding	BP	1	ABL1	up_VSAP_EC
GO:1905244	0.0922	1	1	1	regulation of modification of synaptic structure	BP	1	ABL1	up_VSAP_EC
GO:0048639	0.0923	0.962	7	39	positive regulation of developmental growth	BP	1	LEP,FGFR2,NRP1,TBX20,TBX5,CHD7,EDN1	up_ACOR_VSMC
GO:0003198	0.0923	0.997	1	4	epithelial to mesenchymal transition involved in endocardial cushion	BP	1	TMEM100	up_AFEM_EC
GO:1903147	0.0923	0.997	1	4	negative regulation of autophagy of	BP	1	CLEC16A	up_AFEM_EC
GO:0090673	0.0923	1	1	1	endothelial cell-matrix adhesion	BP	0.943	ADAMTS9	up_VCAV_EC
GO:0009165	0.0924	0.95	13	104	nucleotide biosynthetic process	BP	1	PRPSAP1,ACLY,RRM1,PFAS,ELOVL1,ELOVL6,CYC1,ATP5F1A,PRPS1,ENSCAFG00000013366,PDHA1,CTPS1,ATP5F1D	down_ACOR_VSMC
GO:0070374	0.0924	0.959	8	57	positive regulation of ERK1 and ERK2 cascade	BP	0.943	TEK,PDGFB,TLR4,BMP2,PRKD2,RAPGEF2,HMGB1,PDGFD	up_VCAV_EC
GO:0070665	0.0924	0.969	5	31	positive regulation of leukocyte proliferation	BP	0.943	MEF2C,TLR4,PELI1,NCK1,HMGB1	up_VCAV_EC
GO:0050848	0.0924	0.973	4	30	regulation of calcium-mediated signaling	BP	0.987	FHL2,GSK3B,HTT,CALM1	down_VSAP_EC
GO:1901379	0.0924	0.98	3	12	regulation of potassium ion transmembrane	BP	1	ACTN2,KCNE3,AKAP6	up_ACOR_VSMC
GO:0003007	0.0924	0.986	2	94	heart morphogenesis	BP	1	ACVRL1,NRP2	down_ACOR_EC
GO:2000479	0.0924	0.988	2	8	regulation of cAMP-dependent protein kinase	BP	0.987	SESN2,HTT	down_VSAP_EC
GO:0007089	0.0924	1	1	1	traversing start control point of mitotic cell cycle	BP	1	MTBP	down_ACOR_VSMC
GO:0051309	0.0924	1	1	1	female meiosis chromosome separation	BP	1	NCAPH	down_ACOR_VSMC
GO:0038166	0.0924	0.997	1	4	angiotensin-activated signaling pathway	BP	1	CAMK2A	up_AFEM_EC
GO:0010827	0.0924	0.996	1	28	regulation of glucose transmembrane transport	BP	1	NR1H4	up_VPUL_EC
GO:0050709	0.0924	0.996	1	28	negative regulation of protein secretion	BP	1	NR1H4	up_VPUL_EC
GO:0010604	0.0925	0.963	7	919	positive regulation of macromolecule metabolic	BP	1	HOXA11,HOXA9,SALL1,PRR16,TLR2,CAV1,SHOX2	up_VSAP_VSMC
GO:0001775	0.0925	0.986	2	219	cell activation	BP	1	HOXA9,BPI	up_AORT_EC
GO:0051893	0.0925	0.996	1	20	regulation of focal adhesion assembly	BP	1	ACVRL1	down_ACOR_EC
GO:0090109	0.0925	0.996	1	20	regulation of cell-substrate junction	BP	1	ACVRL1	down_ACOR_EC
GO:0090336	0.0925	0.997	1	4	positive regulation of brown fat cell	BP	1	NAPEPLD	up_AFEM_EC
GO:1901228	0.0925	0.997	1	4	positive regulation of transcription from RNA polymerase II promoter involved in heart	BP	1	GREM1	up_AFEM_EC
GO:0010769	0.0925	0.996	1	69	regulation of cell morphogenesis involved	BP	1	LIMK1	up_VCAV_VSMC
GO:0021730	0.0925	1	1	1	trigeminal sensory nucleus development	BP	1	NFIB	up_VSAP_EC
GO:0021740	0.0925	1	1	1	principal sensory nucleus of trigeminal nerve	BP	1	NFIB	up_VSAP_EC
GO:0061141	0.0925	1	1	1	lung ciliated cell differentiation	BP	1	NFIB	up_VSAP_EC
GO:2000790	0.0925	1	1	1	regulation of mesenchymal cell proliferation involved in	BP	1	NFIB	up_VSAP_EC
GO:2000791	0.0925	1	1	1	negative regulation of mesenchymal cell proliferation involved in	BP	1	NFIB	up_VSAP_EC
GO:2000795	0.0925	1	1	1	negative regulation of epithelial cell proliferation involved in lung	BP	1	NFIB	up_VSAP_EC
GO:0051341	0.0925	0.996	1	22	regulation of oxidoreductase activity	BP	1	CAV1	up_VSAP_VSMC
GO:0051403	0.092543726	0.9671	5	89	stress-activated MAPK cascade	BP	1	FZD5,NOD2,TNFRSF19,MAPK8IP2,SH3RF3	up_ACOR_EC
GO:0044249	0.0926	0.954	13	1851	cellular biosynthetic process	BP	1	ACVRL1,YEATS2,YBX3,HIVEP2,HOXA7,RPSA,SULF1,ELK3,HOXA1,CSGALNACT1,ACSL3,TNRC6B,NEDD4	down_ACOR_EC
GO:0009893	0.0926	0.97	5	995	positive regulation of metabolic process	BP	1	EYA1,GATM,HOXA9,HOXA11,SHOX2	up_VFEM_VSMC
GO:0060078	0.0926	0.974	4	17	regulation of postsynaptic membrane potential	BP	1	TMEM108,DMPK,APP,INSYN1	up_ACOR_VSMC

GO:0099565	0.0926	0.974	4	17	chemical synaptic transmission, postsynaptic	BP	1	TMEM108,DMPK,APP,INSYN1	up_ACOR_VSMC
GO:0009225	0.0926	0.987	2	10	nucleotide-sugar metabolic process	BP	1	GFPT2,FUT8	down_VCAV_EC
GO:0003013	0.0926	0.986	2	121	circulatory system process	BP	1	MAP2K6,AVPR1A	up_VFEM_EC
GO:0034769	0.0926	1	1	1	basement membrane disassembly	BP	1	CTSS	up_ACOR_VSMC
GO:0030854	0.0926	0.997	1	4	positive regulation of granulocyte	BP	1	EVI2B	up_AFEM_EC
GO:0035630	0.0926	0.997	1	4	bone mineralization involved in bone	BP	1	GREM1	up_AFEM_EC
GO:0060219	0.0926	0.997	1	4	camera-type eye photoreceptor cell	BP	1	THRB	up_AFEM_EC
GO:0071333	0.0926	0.996	1	25	cellular response to glucose stimulus	BP	1	NR1H4	up_APUL_EC
GO:0010866	0.0927	0.989	2	6	regulation of triglyceride biosynthetic process	BP	1	SREBF1,CTDNEP1	down_ACOR_VSMC
GO:0072376	0.0927	0.987	2	11	protein activation cascade	BP	1	CFD,A2M	down_VCAV_EC
GO:0071498	0.0927	0.988	2	8	cellular response to fluid shear stress	BP	0.989	SOCS5,PTGS2	down_VSAP_EC
GO:0033147	0.0927	0.997	1	4	negative regulation of intracellular estrogen receptor signaling	BP	1	LBH	up_AFEM_EC
GO:2001212	0.0927	0.997	1	4	regulation of vasculogenesis	BP	1	TMEM100	up_AFEM_EC
GO:0038044	0.0927	1	1	1	transforming growth factor-beta secretion	BP	0.944	FERMT1	up_VCAV_EC
GO:2001201	0.0927	1	1	1	regulation of transforming growth factor-beta	BP	0.944	FERMT1	up_VCAV_EC
GO:2001203	0.0927	1	1	1	positive regulation of transforming growth factor-beta secretion	BP	0.944	FERMT1	up_VCAV_EC
GO:0051886	0.0927	1	1	1	negative regulation of timing of anagen	BP	0.944	FERMT1	up_VCAV_EC
GO:0045666	0.0927	0.996	1	68	positive regulation of neuron differentiation	BP	1	LIMK1	up_VCAV_VSMC
GO:0006937	0.0927	0.996	1	40	regulation of muscle contraction	BP	1	NKX2-5	up_VPOR_VSMC
GO:0019682	0.0928	0.987	2	10	glyceraldehyde-3-phosphate metabolic	BP	1	H6PD,PGLS	down_VCAV_EC
GO:0071674	0.0928	0.996	1	23	mononuclear cell migration	BP	1	LYN	down_ACOR_EC
GO:0050655	0.0928	0.997	1	4	dermatan sulfate proteoglycan metabolic	BP	1	CSGALNACT2	up_AFEM_EC
GO:0015693	0.0928	0.997	1	4	magnesium ion transport	BP	1	NIPAL1	up_AFEM_EC
GO:0042742	0.0928	0.996	1	39	defense response to bacterium	BP	1	BPI	up_AORT_EC
GO:0051270	0.092834212	0.95018	12	296	regulation of cellular component movement	BP	1	SOX9,AQP1,SEMA6D,ZMYND8,MMP28,PTGER4,ARHGDIB,FOXC2,LRRC15,TBX5,RARRES2,NRP1	up_ACOR_EC
GO:0008610	0.092878276	0.95745	8	177	lipid biosynthetic process	BP	1	NR1H4,THNSL2,CCDC3,PTGDS,CHPT1,PIGS,AGPAT2,PRKCD	up_ACOR_EC
GO:0001933	0.0929	0.943	19	146	negative regulation of protein phosphorylation	BP	1	SMAD6,RASSF2,BMP4,LATS2,NDRG2,F2RL1,FAM129A,CDKN1A,PTPN22,SMPD1,DAG1,PSEN1,SYNJ2BP,DUSP22,DUSP26,LRRC4,INPP5K,PARK7,CRKL	up_ACOR_VSMC
GO:0006643	0.0929	0.954	10	68	membrane lipid metabolic process	BP	1	PIGN,PIGS,UGCG,PIGG,ORMDL3,SMPD1,CLN8,SERINC3,NEU3,SERINC1	up_ACOR_VSMC
GO:0097755	0.0929	0.98	3	12	positive regulation of blood vessel diameter	BP	0.945	DOCK4,GUCY1A1,NOS2	up_VCAV_EC
GO:0007442	0.0929	0.998	1	2	hindgut morphogenesis	BP	1	GLI3	down_VCAV_EC
GO:0031666	0.0929	0.998	1	2	positive regulation of lipopolysaccharide-mediated signaling	BP	1	SASH1	down_VCAV_EC
GO:0042478	0.0929	0.998	1	2	regulation of eye photoreceptor cell	BP	1	THRB	down_VCAV_EC
GO:0061218	0.0929	0.997	1	4	negative regulation of mesonephros	BP	1	GREM1	up_AFEM_EC
GO:1901857	0.0929	0.997	1	4	positive regulation of cellular respiration	BP	1	PPARGC1A	up_AFEM_EC
GO:0097150	0.0929	0.996	1	13	neuronal stem cell population maintenance	BP	1	HOOK3	up_VPOR_EC
GO:0070293	0.092925084	0.99665	1	4	renal absorption	BP	1	AQP1	up_ACOR_EC

GO:0031401	0.092947167	0.94758	14	358	positive regulation of protein modification process	BP	1	SOX9,ENSCAFG00000004331,FZD5,DUSP26,NOD2,PAK3,TNFRSF19,PIAS1,MAVS,LRP4,SH3RF3,RARRES2,NRP1,PRKCD	up_ACOR_EC
GO:0006732	0.093	0.947	15	107	coenzyme metabolic process	BP	1	ACSM3,ACACB,NADK,ENSCAFG00000013986,COQ9,PDK2,GALK1,MOCOS,PCBD1,ACSS2,MVK,PNPO,DCAKD,PNP,DDIT4	up_VSAP_EC
GO:0006403	0.093	0.956	9	64	RNA localization	BP	1	DDX39A,RAN,FBL,NUP62,NUP188,DDX39B,NOP58,MRPL18,CCT4	down_ACOR_VSMC
GO:0002824	0.093	0.973	4	29	positive regulation of adaptive immune response based on somatic recombination of immune receptors built from immunoglobulin	BP	0.992	SOCS5,FAM49B,PVR,TFRC	down_VSAP_EC
GO:0045765	0.093	0.986	2	105	regulation of angiogenesis	BP	1	ACVRL1,SULF1	down_ACOR_EC
GO:0097006	0.093	0.996	1	19	regulation of plasma lipoprotein particle levels	BP	1	ACSL3	down_ACOR_EC
GO:0055059	0.093	1	1	1	asymmetric neuroblast division	BP	1	ARHGEF2	down_ACOR_VSMC
GO:0045216	0.093	0.996	1	44	cell-cell junction organization	BP	1	FSCN1	down_AFEM_EC
GO:0021775	0.093	0.998	1	2	smoothened signaling pathway involved in ventral spinal cord	BP	1	GLI3	down_VCAV_EC
GO:0021776	0.093	0.998	1	2	smoothened signaling pathway involved in spinal cord motor neuron cell	BP	1	GLI3	down_VCAV_EC
GO:0060573	0.093	0.998	1	2	cell fate specification involved in pattern	BP	1	GLI3	down_VCAV_EC
GO:0021521	0.093	0.998	1	2	ventral spinal cord interneuron specification	BP	1	GLI3	down_VCAV_EC
GO:1904684	0.093	0.998	1	2	negative regulation of metalloendopeptidase	BP	1	RECK	down_VCAV_EC
GO:1905049	0.093	0.998	1	2	negative regulation of metallopeptidase activity	BP	1	RECK	down_VCAV_EC
GO:0007500	0.093	1	1	1	mesodermal cell fate determination	BP	1	BMP4	up_ACOR_VSMC
GO:0035990	0.093	1	1	1	tendon cell differentiation	BP	1	BMP4	up_ACOR_VSMC
GO:0035992	0.093	1	1	1	tendon formation	BP	1	BMP4	up_ACOR_VSMC
GO:0060503	0.093	1	1	1	bud dilation involved in lung branching	BP	1	BMP4	up_ACOR_VSMC
GO:0061149	0.093	1	1	1	BMP signaling pathway involved in ureter	BP	1	BMP4	up_ACOR_VSMC
GO:0061150	0.093	1	1	1	renal system segmentation	BP	1	BMP4	up_ACOR_VSMC
GO:0061151	0.093	1	1	1	BMP signaling pathway involved in renal system	BP	1	BMP4	up_ACOR_VSMC
GO:0061155	0.093	1	1	1	pulmonary artery endothelial tube	BP	1	BMP4	up_ACOR_VSMC
GO:0061216	0.093	1	1	1	regulation of transcription from RNA polymerase II promoter involved in mesonephros	BP	1	BMP4	up_ACOR_VSMC
GO:0071893	0.093	1	1	1	BMP signaling pathway involved in nephric duct	BP	1	BMP4	up_ACOR_VSMC
GO:0072096	0.093	1	1	1	negative regulation of branch elongation involved in ureteric bud	BP	1	BMP4	up_ACOR_VSMC
GO:0072097	0.093	1	1	1	negative regulation of branch elongation involved in ureteric bud branching by BMP	BP	1	BMP4	up_ACOR_VSMC
GO:0072099	0.093	1	1	1	anterior/posterior pattern specification involved in ureteric bud development	BP	1	BMP4	up_ACOR_VSMC
GO:0072100	0.093	1	1	1	specification of ureteric bud anterior/posterior	BP	1	BMP4	up_ACOR_VSMC
GO:0072101	0.093	1	1	1	specification of ureteric bud anterior/posterior symmetry by BMP	BP	1	BMP4	up_ACOR_VSMC
GO:0072192	0.093	1	1	1	ureter epithelial cell differentiation	BP	1	BMP4	up_ACOR_VSMC
GO:0072197	0.093	1	1	1	ureter morphogenesis	BP	1	BMP4	up_ACOR_VSMC
GO:0072200	0.093	1	1	1	negative regulation of mesenchymal cell proliferation involved in	BP	1	BMP4	up_ACOR_VSMC

GO:1901964	0.093	1	1	1	positive regulation of cell proliferation involved in outflow tract	BP	1	BMP4	up_ACOR_VSMC
GO:2000005	0.093	1	1	1	negative regulation of metanephric S-shaped body morphogenesis	BP	1	BMP4	up_ACOR_VSMC
GO:2000006	0.093	1	1	1	regulation of metanephric comma-shaped body	BP	1	BMP4	up_ACOR_VSMC
GO:2000007	0.093	1	1	1	negative regulation of metanephric comma-shaped body	BP	1	BMP4	up_ACOR_VSMC
GO:2000137	0.093	1	1	1	negative regulation of cell proliferation involved in heart morphogenesis	BP	1	BMP4	up_ACOR_VSMC
GO:0048390	0.093	1	1	1	intermediate mesoderm morphogenesis	BP	1	BMP4	up_ACOR_VSMC
GO:0048391	0.093	1	1	1	intermediate mesoderm formation	BP	1	BMP4	up_ACOR_VSMC
GO:0048392	0.093	1	1	1	intermediate mesodermal cell differentiation	BP	1	BMP4	up_ACOR_VSMC
GO:0055018	0.093	1	1	1	regulation of cardiac muscle fiber development	BP	1	BMP4	up_ACOR_VSMC
GO:0055020	0.093	1	1	1	positive regulation of cardiac muscle fiber	BP	1	BMP4	up_ACOR_VSMC
GO:0060235	0.093	1	1	1	lens induction in camera-type eye	BP	1	BMP4	up_ACOR_VSMC
GO:2000004	0.093	1	1	1	regulation of metanephric S-shaped body	BP	1	BMP4	up_ACOR_VSMC
GO:0051668	0.093	0.996	1	39	localization within membrane	BP	1	LHFPL4	up_VPOR_VSMC
GO:0003231	0.093	0.996	1	48	cardiac ventricle development	BP	1	TBX5	up_VPUL_VSMC
GO:0003279	0.093	0.996	1	48	cardiac septum development	BP	1	TBX5	up_VPUL_VSMC
GO:0030070	0.0931	1	1	1	insulin processing	BP	1	ERO1B	up_ACOR_VSMC
GO:1901142	0.0931	1	1	1	insulin metabolic process	BP	1	ERO1B	up_ACOR_VSMC
GO:0022615	0.0931	0.997	1	4	protein to membrane docking	BP	1	RAB7A	up_AFEM_EC
GO:0046533	0.0931	0.997	1	4	negative regulation of photoreceptor cell	BP	1	THRB	up_AFEM_EC
GO:0002274	0.0931	0.996	1	40	myeloid leukocyte activation	BP	1	BPI	up_AORT_EC
GO:0021510	0.0931	0.996	1	23	spinal cord development	BP	1	HOXC10	up_VSAP_VSMC
GO:0032642	0.0931	0.996	1	22	regulation of chemokine production	BP	1	TLR2	up_VSAP_VSMC
GO:0001818	0.093158591	0.97155	4	65	negative regulation of cytokine production	BP	1	CDH3,NR1H4,NDRG2,PTGER4	up_ACOR_EC
GO:0050852	0.0932	0.969	5	26	T cell receptor signaling pathway	BP	1	PLCG2,ELF1,PTPN22,PSEN1,DUSP22	up_ACOR_VSMC
GO:1904353	0.0932	0.98	3	13	regulation of telomere capping	BP	1	HNRNPD,AURKB,NEK2	down_ACOR_VSMC
GO:0071788	0.0932	1	1	1	endoplasmic reticulum tubular network	BP	1	LNPK	up_ACOR_VSMC
GO:1902307	0.0932	0.997	1	4	positive regulation of sodium ion	BP	1	FXYD1	up_AFEM_EC
GO:0055123	0.0932	0.996	1	40	digestive system development	BP	1	SHOX2	up_VFEM_VSMC
GO:0022610	0.0933	0.933	40	326	biological adhesion	BP	1	SMAD6,ACTN2,LEP,GATA5,CD83,MBP,FERMT1,BMP4,PDE5A,CITED2,PKP4,CD274,TINAGL1,LIMS2,NRP1,PTPN22,NPNT,SPINT2,VCAM1,PRKCE,RND1,ECM2,LAMA3,PSEN1,JUP,CDH18,PERP,DUSP22,NID1,DUSP26,FAM107A,JAM3,IGFBP7,CELSR2,ATP2C1,DOCK5,BVES,RREB1,PAG1,CRKL	up_ACOR_VSMC
GO:0006807	0.0933	0.968	10	3221	nitrogen compound metabolic process	BP	1	IVNS1ABP,EXTL2,DARS,SUCLG2,EIF3M,SRSF6,USP33,GABPA,NDUFA10,TSEN15	down_AFEM_EC
GO:0048010	0.0933	0.973	4	20	vascular endothelial growth factor receptor	BP	0.948	KDR,HHEX,PRKD2,HIF1A	up_VCAV_EC
GO:0045893	0.0933	0.972	4	413	positive regulation of transcription, DNA-	BP	1	HOXA11,HOXA9,SALL1,SHOX2	up_VSAP_VSMC
GO:0032879	0.0933	0.989	2	767	regulation of localization	BP	1	HOXA3,TBX5	up_APUL_VSMC
GO:0048699	0.0933	0.987	2	371	generation of neurons	BP	1	SLITRK5,LIMK1	up_VCAV_VSMC
GO:0003062	0.0933	1	1	1	regulation of heart rate by chemical signal	BP	1	SREBF1	down_ACOR_VSMC

GO:0009786	0.0933	1	1	1	regulation of asymmetric cell division	BP	1	ASPM	down_ACOR_VSMC
GO:0060032	0.0933	1	1	1	notochord regression	BP	1	GLI1	down_ACOR_VSMC
GO:0045004	0.0933	1	1	1	DNA replication proofreading	BP	1	POLE	down_ACOR_VSMC
GO:0045769	0.0933	1	1	1	negative regulation of asymmetric cell division	BP	1	ASPM	down_ACOR_VSMC
GO:1904170	0.0933	1	1	1	regulation of bleb assembly	BP	1	ANLN	down_ACOR_VSMC
GO:1904172	0.0933	1	1	1	positive regulation of bleb assembly	BP	1	ANLN	down_ACOR_VSMC
GO:0010746	0.0933	0.998	1	2	regulation of plasma membrane long-chain	BP	1	AKT2	down_VCAV_EC
GO:0010748	0.0933	0.998	1	2	negative regulation of plasma membrane long-chain fatty acid transport	BP	1	AKT2	down_VCAV_EC
GO:0015911	0.0933	0.998	1	2	plasma membrane long-chain fatty acid transport	BP	1	AKT2	down_VCAV_EC
GO:1902001	0.0933	0.998	1	2	fatty acid transmembrane transport	BP	1	AKT2	down_VCAV_EC
GO:2000192	0.0933	0.998	1	2	negative regulation of fatty acid transport	BP	1	AKT2	down_VCAV_EC
GO:0071375	0.0933	0.996	1	68	cellular response to peptide hormone stimulus	BP	1	CA2	up_VCAV_VSMC
GO:0000722	0.0934	0.989	2	6	telomere maintenance via recombination	BP	1	RAD51,SMC6	down_ACOR_VSMC
GO:0021861	0.0934	0.998	1	2	forebrain radial glial cell differentiation	BP	1	GLI3	down_VCAV_EC
GO:0061709	0.0934	0.998	1	2	reticulophagy	BP	1	RETREG1	down_VCAV_EC
GO:0048701	0.0934	0.996	1	25	embryonic cranial skeleton morphogenesis	BP	1	HOXA1	up_APUL_EC
GO:1901863	0.0934	0.996	1	25	positive regulation of muscle tissue	BP	1	TBX5	up_APUL_EC
GO:0043269	0.0934	0.996	1	135	regulation of ion transport	BP	1	HOXA3	up_APUL_VSMC
GO:0072593	0.0934	0.996	1	68	reactive oxygen species metabolic process	BP	1	CYP1A1	up_VCAV_VSMC
GO:0060633	0.093470578	0.99661	1	4	negative regulation of transcription initiation from RNA polymerase II	BP	1	CLSTN1	up_ACOR_EC
GO:2000143	0.093470578	0.99661	1	4	negative regulation of DNA-templated	BP	1	CLSTN1	up_ACOR_EC
GO:0051093	0.0935	0.935	33	273	negative regulation of developmental process	BP	1	THRB,HOXA7,TWIST2,HOXA9,AXIN2,LIMS2,SLIT2,SLC6A4,ABL1,HDAC7,DLL1,EPN2,OSR1,SAV1,ZBTB16,RARG,ZFP36L1,FXN,PIK3R1,MYC,IRX3,ZEB1,TBXA2R,RTN4RL1,LRP4,ZADH2,FBXO7,LIMD1,FBLN1,ZFP36L2,NFIB,ACVRL1,LBH	up_VSAP_EC
GO:0043408	0.0935	0.943	18	211	regulation of MAPK cascade	BP	0.996	VEGFA,JCAD,PTPN11,TRIB3,TNIK,PTPN2,MAGED1,CTNNB1,APOE,LYN,CBLC,ATF3,PAK2,INHBA,NRP1,SH3RF3,MADD,MID1	down_VSAP_EC
GO:0010257	0.0935	0.969	5	25	NADH dehydrogenase complex assembly	BP	1	NDUFS7,DMAC2,NDUFAF3,NDUFB10,COA1	up_VSAP_EC
GO:0032981	0.0935	0.969	5	25	mitochondrial respiratory chain complex I assembly	BP	1	NDUFS7,DMAC2,NDUFAF3,NDUFB10,COA1	up_VSAP_EC
GO:0001944	0.0935	0.986	2	263	vasculature development	BP	1	HDAC7,CITED2	down_VPOR_EC
GO:0002028	0.0935	0.996	1	19	regulation of sodium ion transport	BP	1	NEDD4	down_ACOR_EC
GO:0035581	0.0935	0.998	1	2	sequestering of extracellular ligand from	BP	1	FBN1	down_VCAV_EC
GO:0043383	0.0935	0.998	1	2	negative T cell selection	BP	1	GLI3	down_VCAV_EC
GO:0051595	0.0935	0.998	1	2	response to methylglyoxal	BP	1	PARK7	down_VCAV_EC
GO:1900115	0.0935	0.998	1	2	extracellular regulation of signal transduction	BP	1	FBN1	down_VCAV_EC
GO:1900116	0.0935	0.998	1	2	extracellular negative regulation of signal	BP	1	FBN1	down_VCAV_EC
GO:1901668	0.0935	0.998	1	2	regulation of superoxide dismutase activity	BP	1	PARK7	down_VCAV_EC
GO:1902177	0.0935	0.998	1	2	positive regulation of oxidative stress-induced intrinsic apoptotic	BP	1	PARK7	down_VCAV_EC
GO:1905874	0.0935	0.998	1	2	regulation of postsynaptic density organization	BP	1	CDH2	down_VCAV_EC

GO:0038086	0.0935	0.998	1	2	VEGF-activated platelet-derived growth factor receptor signaling	BP	1	PDGFRB	down_VCAV_EC
GO:0038091	0.0935	0.998	1	2	positive regulation of cell proliferation by VEGF-activated platelet derived growth factor receptor	BP	1	PDGFRB	down_VCAV_EC
GO:0045060	0.0935	0.998	1	2	negative thymic T cell selection	BP	1	GLI3	down_VCAV_EC
GO:1902897	0.0935	0.998	1	2	regulation of postsynaptic density protein 95	BP	1	CDH2	down_VCAV_EC
GO:2000282	0.0935	0.998	1	2	regulation of cellular amino acid biosynthetic	BP	1	PARK7	down_VCAV_EC
GO:0010927	0.0935	0.996	1	28	cellular component assembly involved in	BP	1	MYBPC2	up_VPUL_EC
GO:0051649	0.0936	0.964	6	630	establishment of localization in cell	BP	1	LYN,SPG11,IPO7,STX4,MYO1D,NEDD4	down_ACOR_EC
GO:0045646	0.0936	0.973	4	19	regulation of erythrocyte differentiation	BP	1	ISG15,FAM210B,P4HTM,B2M	up_ACOR_VSMC
GO:0046633	0.0936	0.988	2	7	alpha-beta T cell proliferation	BP	0.948	ARG2,ELF4	up_VCAV_EC
GO:0031334	0.0936	0.996	1	84	positive regulation of protein complex assembly	BP	1	ESR1	up_AORT_VSMC
GO:0032768	0.0936	0.996	1	12	regulation of monooxygenase activity	BP	1	ACVR2A	up_VPOR_EC
GO:0055117	0.0936	0.996	1	21	regulation of cardiac muscle contraction	BP	1	CAV1	up_VSAP_VSMC
GO:0097191	0.0937	0.957	8	79	extrinsic apoptotic signaling pathway	BP	0.997	ARHGEF2,SERPINE1,ATF3,HTT,PAK2,INHBA,NRP1,MADD	down_VSAP_EC
GO:0010721	0.0937	0.971	4	73	negative regulation of cell development	BP	1	IRX3,DLL1,THRB,KANK1	up_AFEM_EC
GO:0036445	0.0937	0.988	2	6	neuronal stem cell division	BP	1	ASPM,ARHGEF2	down_ACOR_VSMC
GO:0055057	0.0937	0.988	2	6	neuroblast division	BP	1	ASPM,ARHGEF2	down_ACOR_VSMC
GO:0009617	0.0937	0.986	2	133	response to bacterium	BP	1	NR1H4,BPI	up_APUL_EC
GO:0098722	0.0937	0.998	1	2	asymmetric stem cell division	BP	1	ZBTB16	down_VCAV_EC
GO:0006886	0.0937	0.998	1	332	intracellular protein transport	BP	1	JUP	down_VFEM_VSMC
GO:1903576	0.0937	1	1	1	response to L-arginine	BP	1	CASTOR1	up_ACOR_VSMC
GO:1903577	0.0937	1	1	1	cellular response to L-arginine	BP	1	CASTOR1	up_ACOR_VSMC
GO:0019218	0.0937	0.996	1	25	regulation of steroid metabolic process	BP	1	NR1H4	up_APUL_EC
GO:0021872	0.093732133	0.98591	2	19	forebrain generation of neurons	BP	1	DCLK2,NRP1	up_ACOR_EC
GO:0007389	0.0938	0.944	18	137	pattern specification process	BP	1	SMAD6,GATA5,MLLT3,BMP4,FGFR2,GJA1,CITED2,CLSTN1,NRP1,SMARCD3,TBX20,TBX5,FRS2,PSEN1,PCSK6,PSKH1,EDN1,CRKL	up_ACOR_VSMC
GO:2000177	0.0938	0.973	4	26	regulation of neural precursor cell	BP	0.997	ILK,HIF1A,CTNNB1,DISC1	down_VSAP_EC
GO:0031076	0.0938	0.979	3	14	embryonic camera-type eye development	BP	0.948	PROX1,ALDH1A3,ZEB1	up_VCAV_EC
GO:0090176	0.0938	0.998	1	2	microtubule cytoskeleton organization involved in establishment of planar	BP	1	BRSK1	down_VCAV_EC
GO:0007519	0.0938	0.996	1	49	skeletal muscle tissue development	BP	1	CITED2	down_VPOR_EC
GO:0002713	0.0938	1	1	1	negative regulation of B cell mediated immunity	BP	0.948	THOC1	up_VCAV_EC
GO:0045829	0.0938	1	1	1	negative regulation of isotype switching	BP	0.948	THOC1	up_VCAV_EC
GO:0048297	0.0938	1	1	1	negative regulation of isotype switching to IgA	BP	0.948	THOC1	up_VCAV_EC
GO:0002890	0.0938	1	1	1	negative regulation of immunoglobulin mediated immune	BP	0.948	THOC1	up_VCAV_EC
GO:0090067	0.0938	1	1	1	regulation of thalamus size	BP	1	SLC6A4	up_VSAP_EC
GO:0032456	0.0939	0.973	4	19	endocytic recycling	BP	1	ACTN2,RAB11B,LMTK2,BVES	up_ACOR_VSMC
GO:0051961	0.0939	0.971	4	73	negative regulation of nervous system	BP	1	IRX3,DLL1,THRB,KANK1	up_AFEM_EC
GO:0030032	0.0939	0.973	4	19	lamellipodium assembly	BP	1	WASF3,SLIT2,FGD4,NCK1	up_VSAP_EC
GO:0051960	0.0939	0.986	2	216	regulation of nervous system development	BP	1	EYA1,SHOX2	up_VFEM_VSMC

GO:0002036	0.0939	1	1	1	regulation of L-glutamate import across plasma	BP	1	PSEN1	up_ACOR_VSMC
GO:0002038	0.0939	1	1	1	positive regulation of L-glutamate import across	BP	1	PSEN1	up_ACOR_VSMC
GO:0015871	0.0939	1	1	1	choline transport	BP	1	PSEN1	up_ACOR_VSMC
GO:0051582	0.0939	1	1	1	positive regulation of neurotransmitter uptake	BP	1	PSEN1	up_ACOR_VSMC
GO:1904797	0.0939	1	1	1	negative regulation of core promoter binding	BP	1	PSEN1	up_ACOR_VSMC
GO:0010958	0.0939	1	1	1	regulation of amino acid import across plasma	BP	1	PSEN1	up_ACOR_VSMC
GO:0016080	0.0939	1	1	1	synaptic vesicle targeting	BP	1	PSEN1	up_ACOR_VSMC
GO:0021870	0.0939	1	1	1	Cajal-Retzius cell differentiation	BP	1	PSEN1	up_ACOR_VSMC
GO:0098629	0.0939	1	1	1	trans-Golgi network membrane organization	BP	1	AZIN2	up_ACOR_VSMC
GO:0006796	0.094	0.966	6	938	phosphate-containing compound metabolic	BP	1	NR1H4,STK32B,CCL24,PTGER2,FLRT3,ARHGEF2	up_VPUL_EC
GO:0043949	0.094	0.98	3	13	regulation of cAMP-mediated signaling	BP	0.948	PDE4D,APLNR,RAPGEF2	up_VCAV_EC
GO:0002528	0.094	0.998	1	2	regulation of vascular permeability involved in acute inflammatory	BP	1	CLSTN1	down_VCAV_EC
GO:0036306	0.094	1	1	1	embryonic heart tube elongation	BP	1	TBX20	up_ACOR_VSMC
GO:1900477	0.094	1	1	1	negative regulation of G1/S transition of mitotic cell cycle by negative regulation of transcription from RNA polymerase II	BP	1	NACC2	up_ACOR_VSMC
GO:1903996	0.094	1	1	1	negative regulation of non-membrane spanning protein tyrosine kinase	BP	1	DUSP22	up_ACOR_VSMC
GO:0021524	0.094	1	1	1	visceral motor neuron differentiation	BP	1	TBX20	up_ACOR_VSMC
GO:0042985	0.094	1	1	1	negative regulation of amyloid precursor protein biosynthetic process	BP	1	ITM2C	up_ACOR_VSMC
GO:0051685	0.094	1	1	1	maintenance of ER location	BP	1	POLR2M	up_ACOR_VSMC
GO:0003290	0.094	1	1	1	atrial septum secundum morphogenesis	BP	1	GATA4	up_ACOR_VSMC
GO:1901373	0.094	1	1	1	lipid hydroperoxide transport	BP	1	SCP2	up_ACOR_VSMC
GO:1902310	0.094	1	1	1	positive regulation of peptidyl-serine	BP	1	DUSP26	up_ACOR_VSMC
GO:0043542	0.094	0.996	1	86	endothelial cell migration	BP	1	WNT7A	up_AORT_VSMC
GO:0036289	0.094	1	1	1	peptidyl-serine autophosphorylation	BP	0.948	RIPK1	up_VCAV_EC
GO:0060545	0.094	1	1	1	positive regulation of necroptotic process	BP	0.948	RIPK1	up_VCAV_EC
GO:0070926	0.094	1	1	1	regulation of ATP:ADP antiporter activity	BP	0.948	RIPK1	up_VCAV_EC
GO:1905206	0.094	1	1	1	positive regulation of hydrogen peroxide-	BP	0.948	RIPK1	up_VCAV_EC
GO:2001040	0.094	1	1	1	positive regulation of cellular response to drug	BP	0.948	RIPK1	up_VCAV_EC
GO:0033138	0.094	0.996	1	28	positive regulation of peptidyl-serine	BP	1	PTGER2	up_VPUL_EC
GO:0065007	0.0941	0.93	92	3499	biological regulation	BP	1	HOXA9,CMKLR1,MT2A,IRX3,IRX5,HOXC10,HOXA10,LEP,KCNC4,LBH,PRR16,CRY2,ENSCAFG00000009113,RAB20,GAS1,F5,ANGPT4,HOXA7,CD40,DLL1,CRYAB,KCND1,F3,PLXNA4,BPI,GPAM,FIBIN,MLLT11,ENSCAFG00000018194,GPR1,LMCD1,CHRM2,TWIST2,MAB21L1,THRB,LGI4,MYO1D,MFAP4,HCK,TMEM100,SLC6A6,PLAGL2,GREM1,HTR1D,NFE2L1,OVOL1,FAM53B,AVPR1A,TENT5C,CAMK2A,GREM2,CLEC16A,CYLD,RASGEF1B,POSTN,FXYD1,LRRC15,FAM13A,MYBL1,RECK,NR3C1,PTHLH,TBXA2R,EVI2B,MOXD1,DCUN1D3,CNNM4,TFPI2,REEP2,EAF1,BVES,LGR6,RAB7A,AXIN2,YEATS2,ATP6V0B,ACACB,NAPEPLD,PPARGC1A,CCL5,CDC42EP5,CTIF,FAP,DMPK,ADAM17,SAMD4B,CLIC4,AGPAT2,GCNT4,FOSL2,KANK1,TNC	up_AFEM_EC

GO:0051050	0.0941	0.936	29	235	positive regulation of transport	BP	1	EXPH5,TLR1,ACTN2,PLCG2,LEP,HTR2B,TCAF1,MBP,FERMT1,TM7SF3,ABCA1,CD274,ANKRD1,F2RL1,AKAP6,PTPN22,SCP2,PRKCE,PSEN1,JUP,EDN1,NOD2,B2M,FXYD1,SCAMP5,INPP5K,WFS1,AZIN2,PARK7	up_ACOR_VSMC
GO:0006811	0.0941	0.949	12	330	ion transport	BP	1	LEP,KCNC4,KCND1,SLC17A5,ATP6V1B2,SLC6A6,CAMK2A,FXYD1,ATP6V0B,NIPAL1,DMPK,CLIC4	up_AFEM_EC
GO:0007623	0.0941	0.958	8	55	circadian rhythm	BP	0.948	PPARG,PROX1,ENSCAFG00000010596,TIMELESS,TOP1,CREB1,NR1D2,KDM5A	up_VCAV_EC
GO:0030073	0.0941	0.961	7	42	insulin secretion	BP	1	LEP,TM7SF3,PRKCE,CCL5,RAB11B,PFKL,PARK7	up_ACOR_VSMC
GO:0006937	0.0941	0.964	6	40	regulation of muscle contraction	BP	0.948	DOCK4,PDE4D,STC1,GUCY1A1,KCNJ2,ATP2A1	up_VCAV_EC
GO:0048846	0.0941	0.988	2	6	axon extension involved in axon guidance	BP	1	SLIT2,PLXNA4	up_VSAP_EC
GO:1902284	0.0941	0.988	2	6	neuron projection extension involved in neuron projection	BP	1	SLIT2,PLXNA4	up_VSAP_EC
GO:1904528	0.0941	0.998	1	2	positive regulation of microtubule binding	BP	1	MAPRE3	down_VCAV_EC
GO:0032103	0.0941	0.996	1	69	positive regulation of response to external	BP	1	TLR2	up_AFEM_VSMC
GO:0030308	0.0941	0.996	1	49	negative regulation of cell growth	BP	1	SLIT2	up_VPUL_VSMC
GO:1900454	0.0941	1	1	1	positive regulation of long-term synaptic depression	BP	1	KCNB1	up_VSAP_EC
GO:0040007	0.0942	0.935	33	276	growth	BP	1	HOXA11,ENSCAFG00000009113,WNT11,HNF4A,FAM107A,ACACB,SLIT2,SLC6A4,FGF7,ABL1,DLL1,SAV1,PLXNA4,RARG,DDR2,ZFP36L1,MT2A,FXN,GAS1,MYD88,LRP4,DPYSL2,WISP2,KDM2B,IGFBP6,TAF8,TTL,SMARCA2,ACVRL1,HTRA2,ULK1,TGFBR2,LATS2	up_VSAP_EC
GO:0048522	0.0942	0.957	10	1484	positive regulation of cellular process	BP	1	HOXA11,HOXA9,NETO1,SALL1,MAB21L1,PRR16,TLR2,CAV1,SHOX2,FGF5	up_VSAP_VSMC
GO:0045766	0.0942	0.985	2	63	positive regulation of angiogenesis	BP	1	C3AR1,MTDH	up_VPOR_EC
GO:1905953	0.0942	0.996	1	12	negative regulation of lipid localization	BP	1	ABHD5	up_VPOR_EC
GO:0002011	0.0942	0.996	1	28	morphogenesis of an epithelial sheet	BP	1	FLRT3	up_VPUL_EC
GO:0007043	0.0942	0.996	1	28	cell-cell junction assembly	BP	1	NR1H4	up_VPUL_EC
GO:0048289	0.0942	1	1	1	isotype switching to IgE isotypes	BP	1	STAT6	up_VSAP_EC
GO:0048293	0.0942	1	1	1	regulation of isotype switching to IgE isotypes	BP	1	STAT6	up_VSAP_EC
GO:0048295	0.0942	1	1	1	positive regulation of isotype switching to IgE	BP	1	STAT6	up_VSAP_EC
GO:0060711	0.0942	0.996	1	21	labyrinthine layer development	BP	1	GJB5	up_VSAP_VSMC
GO:0035690	0.0943	0.95	12	91	cellular response to drug	BP	0.949	TNFAIP3,PDGFB,MEF2C,PPP3CA,MAPK13,TIMELESS,GUCY1B1,CTNNA1,BLM,RIPK1,ATRX,ARHGEF2	up_VCAV_EC
GO:0006888	0.0943	0.996	1	49	ER to Golgi vesicle-mediated transport	BP	1	ERGIC1	down_VPOR_EC
GO:1902959	0.0943	0.998	1	2	regulation of aspartic-type endopeptidase activity involved in amyloid precursor protein	BP	0.998	LYN	down_VSAP_EC
GO:1902961	0.0943	0.998	1	2	positive regulation of aspartic-type endopeptidase activity involved in amyloid	BP	0.998	LYN	down_VSAP_EC
GO:1905245	0.0943	0.998	1	2	regulation of aspartic-type peptidase activity	BP	0.998	LYN	down_VSAP_EC
GO:1905247	0.0943	0.998	1	2	positive regulation of aspartic-type peptidase	BP	0.998	LYN	down_VSAP_EC
GO:0009750	0.0943	1	1	1	response to fructose	BP	1	SLC2A5	up_ACOR_VSMC
GO:0015755	0.0943	1	1	1	fructose transmembrane transport	BP	1	SLC2A5	up_ACOR_VSMC
GO:0032445	0.0943	1	1	1	fructose import	BP	1	SLC2A5	up_ACOR_VSMC
GO:0071332	0.0943	1	1	1	cellular response to fructose stimulus	BP	1	SLC2A5	up_ACOR_VSMC
GO:0098704	0.0943	1	1	1	carbohydrate import across plasma membrane	BP	1	SLC2A5	up_ACOR_VSMC
GO:1900259	0.0943	1	1	1	regulation of RNA-directed 5'-3' RNA	BP	1	EIF4A2	up_ACOR_VSMC

GO:1900260	0.0943	1	1	1	negative regulation of RNA-directed 5'-3' RNA polymerase activity	BP	1	EIF4A2	up_ACOR_VSMC
GO:1990539	0.0943	1	1	1	fructose import across plasma membrane	BP	1	SLC2A5	up_ACOR_VSMC
GO:0090407	0.094371837	0.95662	8	177	organophosphate biosynthetic process	BP	1	NR1H4,NMNAT2,GUCY1B1,CHPT1,PIGS,AK7,ALDOC,AGPAT2	up_ACOR_EC
GO:0031326	0.0944	0.923	110	1176	regulation of cellular biosynthetic process	BP	0.949	EDNRB,HCLS1,ADGRF5,KIT,PRKCH,FLI1,TNFAIP3,PPARG,TNIP1,PDGFB,TMEM173,RELN,CD34,IWS1,MEF2C,ATF3,CHD7,DLL4,WWP1,TLR4,NLRC5,QKI,GATA3,EDN1,HOXB8,TCF4,SMAD1,FOXN2,MEOX1,ENSCAFG00000017103,IRF8,SOX18,PELI1,PPP3CA,YES1,FAM3C,MTOR,SAMD8,ELF4,BMP2,HMG20A,HHEX,CREM,CNOT6,RSC1A1,ENC1,CCAR1,PROX1,UHRF2,SECISBP2L,MDFI,ENSCAFG00000010596,ARID4B,HMBOX1,PRKD2,THOC1,SMARCA4,TLR3,NCK1,ESCO1,DPH5,TIMELESS,LDB2,CD200,JAG1,NSD1,PTGS2,RAPGEF2,TCERG1,CTDSPL2,CREB1,BCL10,BASP1,CNOT7,ENSCAFG00000002897,HMGB1,AHR,NOS2,ZNF789,BLM,NUP133,RIPK1,ZNF317,USP13,ITGA6,RAD17,SKIL,GPBP1,USP37,MTA2,NR1D2,KDM5A,HIF1A,KDM3A,ZNF146,ATRX,NR4A1,HIST2H2AC,HP1BP3,ARID5A,ENSCAFG00000002015,RBBP6,ZNF354A,DCP2,ZEB1,TET2,ARHGEF2,CDON,ELK3,ZNF609	up_VCAV_EC
GO:0031325	0.0944	0.927	65	914	positive regulation of cellular metabolic process	BP	0.998	ARHGEF2,PRICKLE1,VEGFA,MYH9,NR1H4,PLK3,HSPA5,SOCS5,JCAD,BCAR3,PTPN11,TRIB3,ILK,PPP1R12A,SMARCA4,SESN2,TGFBR1,PTGS2,MSN,HIF1A,SLC45A3,TNIK,ZPR1,PTPN2,YES1,PEG3,MTDH,DDX39B,ADAM17,CNOT7,MAGED1,CTNNB1,TFRC,APOE,KLF5,LYN,BNIP3,DDIT3,FAM162A,MMD,ATF3,GSK3B,VLDLR,CDC37,GTF2H1,ARMCX3,SH3GLB1,HTT,REST,ABHD5,CALM1,DISC1,RAD23A,PAK2,INHBA,NR4A1,CDKN1A,TMTC3,NRP1,SH3RF3,ARF4,YWHAH,MADD,KDM3A,MID1	down_VSAP_EC
GO:0001933	0.0944	0.948	13	146	negative regulation of protein phosphorylation	BP	0.998	SOCS5,ENPP1,ERRFI1,SNX25,TRIB3,DNAJC10,PTPN2,APOE,LYN,STRAP,CBLC,ATF3,CDKN1A	down_VSAP_EC
GO:0060485	0.0944	0.957	8	93	mesenchyme development	BP	1	VASN,GLIPR2,BMPR1A,SEMA3C,ZFP36L1,LOXL3,RDH10,GREM1	down_VCAV_EC
GO:0031667	0.0944	0.966	5	102	response to nutrient levels	BP	1	LEP,CLEC16A,POSTN,ACACB,PPARGC1A	up_AFEM_EC
GO:0032507	0.0944	0.973	4	18	maintenance of protein location in cell	BP	1	OS9,DAG1,SUN1,PARK7	up_ACOR_VSMC
GO:0120036	0.0944	0.987	2	373	plasma membrane bounded cell projection	BP	1	SLITRK5,LIMK1	up_VCAV_VSMC
GO:0030850	0.0944	0.996	1	20	prostate gland development	BP	1	SULF1	down_ACOR_EC
GO:0060046	0.0944	0.998	1	2	regulation of acrosome reaction	BP	0.998	SPINK2	down_VSAP_EC
GO:0030329	0.0944	1	1	1	prenylcysteine metabolic process	BP	1	PCYOX1	up_ACOR_VSMC
GO:0015739	0.0944	1	1	1	sialic acid transport	BP	1	SLC17A5	up_ACOR_VSMC
GO:0030328	0.0944	1	1	1	prenylcysteine catabolic process	BP	1	PCYOX1	up_ACOR_VSMC
GO:0070555	0.0944	0.996	1	29	response to interleukin-1	BP	1	CCL24	up_VPUL_EC
GO:0010562	0.0945	0.94	21	312	positive regulation of phosphorus metabolic process	BP	1	NOTCH2,PDGFRB,PRNP,GLIPR2,BMPR1A,LMO4,CLIP3,AKT2,CSPG4,PARK7,MYDGF,SASH1,ARL2BP,MAVS,CDH2,GSDME,MAPRE3,DDR2,SHC1,FBH1,GREM1	down_VCAV_EC
GO:0045937	0.0945	0.94	21	312	positive regulation of phosphate metabolic process	BP	1	NOTCH2,PDGFRB,PRNP,GLIPR2,BMPR1A,LMO4,CLIP3,AKT2,CSPG4,PARK7,MYDGF,SASH1,ARL2BP,MAVS,CDH2,GSDME,MAPRE3,DDR2,SHC1,FBH1,GREM1	down_VCAV_EC
GO:0007019	0.0945	0.979	3	13	microtubule depolymerization	BP	1	STMN1,ARHGEF2,KIF24	down_ACOR_VSMC
GO:0031643	0.0945	1	1	1	positive regulation of myelination	BP	1	WASF3	up_ACOR_VSMC

GO:1901575	0.0946	0.93	47	643	organic substance catabolic process	BP	1	PRICKLE1,PLK3,HSPA5,SOCS5,HM13,HSP90B1,ENPP1,SNX25,PGK1,TPI1,TRIB3,PGAM1,ALDH1L2,SESN2,CEMIP2,MSN,SEL1L,HIF1A,USP12,DNAJC10,UBA6,ZPR1,ENTPD4,UCHL5,SPOPL,PLCL1,SMG7,CNOT7,DNAJB9,ALDOC,SND1,APOE,USP48,BNIP3,DDIT3,CBLC,GSK3B,CUL2,GALE,ABHD5,SPOP,DISC1,RAD23A,TMTC3,USP14,YWHAH,TNFAIP1	down_VSAP_EC
GO:0042100	0.0946	0.996	1	19	B cell proliferation	BP	1	LYN	down_ACOR_EC
GO:1900063	0.0946	0.998	1	2	regulation of peroxisome organization	BP	1	MAVS	down_VCAV_EC
GO:0090090	0.0946	0.996	1	44	negative regulation of canonical Wnt signaling	BP	1	DKK2	up_AORT_EC
GO:0019751	0.0947	0.964	6	33	polyol metabolic process	BP	1	GOT1,INPP5E,PPIP5K1,GALK1,PCBD1,TKFC	up_VSAP_EC
GO:1902680	0.0947	0.972	4	415	positive regulation of RNA biosynthetic process	BP	1	HOXA11,HOXA9,SALL1,SHOX2	up_VSAP_VSMC
GO:1903508	0.0947	0.972	4	415	positive regulation of nucleic acid-templated	BP	1	HOXA11,HOXA9,SALL1,SHOX2	up_VSAP_VSMC
GO:0003206	0.0947	0.996	1	51	cardiac chamber morphogenesis	BP	1	CITED2	down_VPOR_EC
GO:0032609	0.0947	0.996	1	25	interferon-gamma production	BP	1	NR1H4	up_APUL_EC
GO:0014859	0.0947	1	1	1	negative regulation of skeletal muscle cell	BP	0.949	EPHB1	up_VCAV_EC
GO:1902723	0.0947	1	1	1	negative regulation of skeletal muscle satellite	BP	0.949	EPHB1	up_VCAV_EC
GO:0007597	0.0947	1	1	1	blood coagulation, intrinsic pathway	BP	0.949	KLKB1	up_VCAV_EC
GO:0051919	0.0947	1	1	1	positive regulation of fibrinolysis	BP	0.949	KLKB1	up_VCAV_EC
GO:0060696	0.094717513	0.99651	1	4	regulation of phospholipid catabolic process	BP	1	PRKCD	up_ACOR_EC
GO:0061326	0.094739956	0.97718	3	41	renal tubule development	BP	1	SOX9,AQP1,CLSTN1	up_ACOR_EC
GO:0072080	0.094739956	0.97718	3	41	nephron tubule development	BP	1	SOX9,AQP1,CLSTN1	up_ACOR_EC
GO:0072224	0.094762481	0.99651	1	4	metanephric glomerulus development	BP	1	AQP1	up_ACOR_EC
GO:0072239	0.094762481	0.99651	1	4	metanephric glomerulus vasculature development	BP	1	AQP1	up_ACOR_EC
GO:0060249	0.0948	0.947	14	110	anatomical structure homeostasis	BP	0.949	ADGRF5,CD34,TLR4,HMBOX1,EXOC5,LDB2,TAOK1,SMG7,VPS54,BLM,SMC5,HIF1A,ATRX,DCP2	up_VCAV_EC
GO:0003300	0.0948	0.973	4	18	cardiac muscle hypertrophy	BP	1	GATA5,HTR2B,PDE5A,EDN1	up_ACOR_VSMC
GO:0014896	0.0948	0.973	4	18	muscle hypertrophy	BP	1	GATA5,HTR2B,PDE5A,EDN1	up_ACOR_VSMC
GO:0014897	0.0948	0.973	4	18	striated muscle hypertrophy	BP	1	GATA5,HTR2B,PDE5A,EDN1	up_ACOR_VSMC
GO:0002791	0.0948	0.985	2	104	regulation of peptide secretion	BP	1	LYN,STX4	down_ACOR_EC
GO:0045964	0.0948	0.998	1	2	positive regulation of dopamine metabolic	BP	1	PARK7	down_VCAV_EC
GO:1903179	0.0948	0.998	1	2	regulation of dopamine biosynthetic process	BP	1	PARK7	down_VCAV_EC
GO:1903181	0.0948	0.998	1	2	positive regulation of dopamine biosynthetic	BP	1	PARK7	down_VCAV_EC
GO:0045915	0.0948	0.998	1	2	positive regulation of catecholamine metabolic	BP	1	PARK7	down_VCAV_EC
GO:0032330	0.094812102	0.9856	2	20	regulation of chondrocyte differentiation	BP	1	SOX9,EFEMP1	up_ACOR_EC
GO:0048732	0.0949	0.945	16	116	gland development	BP	1	LBH,BMP4,FGFR2,GJA1,CITED2,LMO4,LIMS2,NRP1,FRS2,DAG1,PSEN1,ARID5B,SERPINB5,RREB1,ZDHHC21,CRKL	up_ACOR_VSMC
GO:0002694	0.0949	0.952	10	126	regulation of leukocyte activation	BP	1	GPAM,PRNP,SDC4,THY1,GLI3,ZFP36L1,BPI,LOXL3,RAC2,ZBTB16	down_VCAV_EC
GO:0045600	0.0949	0.978	3	21	positive regulation of fat cell differentiation	BP	1	ZFP36L1,NAPEPLD,ZBTB16	down_VCAV_EC
GO:0007605	0.0949	0.996	1	27	sensory perception of sound	BP	1	HOXA1	up_APUL_EC
GO:0016202	0.0949	0.996	1	39	regulation of striated muscle tissue	BP	1	SHOX2	up_VFEM_VSMC
GO:0060840	0.0949	0.996	1	42	artery development	BP	1	EYA1	up_VFEM_VSMC
GO:0045833	0.0949	0.996	1	29	negative regulation of lipid metabolic process	BP	1	NR1H4	up_VPUL_EC
GO:0045844	0.0949	0.996	1	24	positive regulation of striated muscle tissue	BP	1	SHOX2	up_VSAP_VSMC

GO:0048636	0.0949	0.996	1	24	positive regulation of muscle organ	BP	1	SHOX2	up_VSAP_VSMC
GO:0006644	0.095	0.945	16	118	phospholipid metabolic process	BP	1	PIP5K1B,PLCG2,HTR2B,LCAT,CHPT1,PIGN,PIGS,PLPP2,PIGG,SMPD1,CRLS1,CLN8,SERINC3,SERINC1,CFI,INPP5K	up_ACOR_VSMC
GO:0051640	0.095	0.945	15	174	organelle localization	BP	1	MYH9,SHROOM2,EXOC5,HOOK3,MYO1C,KIF5B,HIF1A,TRAK1,CTNNB1,KPNB1,LYN,GSK3B,HTT,AP3S1,ESYT3	down_VSAP_EC
GO:0061138	0.095	0.951	11	73	morphogenesis of a branching epithelium	BP	1	BMP4,FGFR2,SPINT1,CLSTN1,NRP1,NPNT,SPINT2,TBX20,FRS2,DAG1,EDN1	up_ACOR_VSMC
GO:0042110	0.095	0.954	9	109	T cell activation	BP	1	GPAM,PRNP,SDC4,THY1,GLI3,ZFP36L1,LOXL3,RAC2,ZBTB16	down_VCAV_EC
GO:0045637	0.095	0.971	4	73	regulation of myeloid cell differentiation	BP	1	HOXA9,HOXA7,DLL1,EVI2B	up_AFEM_EC
GO:0042127	0.095	0.978	3	452	regulation of cell proliferation	BP	1	HOXA3,NKX2-5,BAMBI	up_VPOR_VSMC
GO:0019915	0.095	0.985	2	22	lipid storage	BP	1	CRY2,ACACB	up_AFEM_EC
GO:1903391	0.095	0.996	1	21	regulation of adherens junction organization	BP	1	ACVRL1	down_ACOR_EC
GO:1903093	0.095	0.998	1	2	regulation of protein K48-linked deubiquitination	BP	1	PARK7	down_VCAV_EC
GO:0003218	0.095	1	1	1	cardiac left ventricle formation	BP	1	TBX5	up_ACOR_VSMC
GO:0048593	0.095008718	0.97708	3	41	camera-type eye morphogenesis	BP	1	SOX9,AQP1,FZD5	up_ACOR_EC
GO:0000209	0.0951	0.95	12	83	protein polyubiquitination	BP	1	SKP2,SASH1,KLHL20,MARCH5,PTPN22,UBE2H,RNF24,RNF19A,SHPRH,NOD2,RNF181,DZIP3	up_ACOR_VSMC
GO:0055024	0.0951	0.985	2	22	regulation of cardiac muscle tissue	BP	1	DLL1,GREM1	up_AFEM_EC
GO:0010507	0.0951	0.996	1	26	negative regulation of autophagy	BP	1	LZTS1	up_APUL_EC
GO:1904018	0.0951	0.996	1	69	positive regulation of vasculature development	BP	1	C6	up_VCAV_VSMC
GO:0048562	0.0952	0.966	5	102	embryonic organ morphogenesis	BP	1	TBX15,IRX5,HOXA7,DLL1,HOXA6	up_AFEM_EC
GO:0032526	0.0952	0.996	1	19	response to retinoic acid	BP	1	LYN	down_ACOR_EC
GO:0071322	0.0952	0.996	1	29	cellular response to carbohydrate stimulus	BP	1	NR1H4	up_VPUL_EC
GO:0051246	0.0953	0.929	50	832	regulation of protein metabolic process	BP	1	CST3,NOTCH2,PDGFRB,KIRREL1,LRP1,DCN,PRNP,GLIPR2,BGN,LARP6,BMPR1A,RECK,CLIP3,AKT1S1,TIMP2,AKT2,PSMF1,SAMD4B,EEF2K,PLCL1,CSPG4,PRMT6,ASPN,EIF3K,THY1,NECAB3,PARK7,EEF2,MAST2,ZFP36L1,MYDGF,SASH1,CLEC16A,ARL2BP,IFT57,MAVS,CDH2,GSDME,SPRED1,A2M,MAPRE3,DDR2,SHC1,PYM1,FBH1,GREM1,FOXO3,SOAT1,PACSIN3,EIF4EBP1	down_VCAV_EC
GO:0015893	0.0953	0.968	5	26	drug transport	BP	1	TCN2,PSEN1,CLN8,EDN1,PARK7	up_ACOR_VSMC
GO:0002040	0.0953	0.977	3	46	sprouting angiogenesis	BP	1	DLL1,GREM1,TBXA2R	up_AFEM_EC
GO:0010907	0.0953	0.987	2	10	positive regulation of glucose metabolic process	BP	1	SLC45A3,PTPN2	down_VSAP_EC
GO:0071156	0.0954	0.972	4	21	regulation of cell cycle arrest	BP	1	SLC29A1,CASP2,CDK4,BRCA1	down_ACOR_VSMC
GO:0006925	0.0954	0.988	2	6	inflammatory cell apoptotic process	BP	0.953	MEF2C,ITPKB	up_VCAV_EC
GO:0016556	0.0954	0.988	2	7	mRNA modification	BP	0.954	WTAP,ZC3H13	up_VCAV_EC
GO:0061053	0.0954	0.996	1	24	somite development	BP	1	SIX1	up_VFEM_EC
GO:1903531	0.095421936	0.97694	3	41	negative regulation of secretion by cell	BP	1	NR1H4,KCNB1,PTGER4	up_ACOR_EC
GO:0009071	0.095475794	0.99646	1	4	serine family amino acid catabolic process	BP	1	THNSL2	up_ACOR_EC
GO:0007179	0.0955	0.955	9	57	transforming growth factor beta receptor	BP	1	SMAD6,FERMT1,CITED2,CDKN2B,NPNT,FUT8,TGFBR1,DUSP22,HPGD	up_ACOR_VSMC
GO:0040018	0.0955	0.986	2	11	positive regulation of multicellular organism	BP	1	GPAM,PEX5	down_VCAV_EC
GO:0050926	0.0955	0.988	2	6	regulation of positive chemotaxis	BP	1	VEGFD,F2RL1	up_ACOR_VSMC
GO:0021953	0.0955	0.996	1	49	central nervous system neuron differentiation	BP	1	SLIT2	up_VPUL_VSMC
GO:0021885	0.0955	0.996	1	24	forebrain cell migration	BP	1	EMX2	up_VSAP_VSMC
GO:0033692	0.0956	0.996	1	20	cellular polysaccharide biosynthetic process	BP	1	CSGALNACT1	down_ACOR_EC

GO:1903377	0.0956	0.998	1	2	negative regulation of oxidative stress-induced neuron intrinsic apoptotic	BP	1	PARK7	down_VCAV_EC
GO:0062028	0.0956	1	1	1	regulation of stress granule assembly	BP	1	PRKAA2	up_ACOR_VSMC
GO:1904428	0.0956	1	1	1	negative regulation of tubulin deacetylation	BP	1	PRKAA2	up_ACOR_VSMC
GO:0042592	0.0957	0.931	39	522	homeostatic process	BP	1	P4HB,SLC39A7,NR1H4,ENSCAFG00000013214,ERO1A,ENPP1,CASP3,ENSCAFG00000003477,PTPN11,EXOC5,ATP2B1,LRRC8A,PRCP,SMARCA4,SESN2,PTGS2,TMTC2,HIF1A,DNAJC10,NXN,PTPN2,NEO1,PLCL1,SMG7,PDIA4,CTNNB1,TFRC,APOE,LYN,STRAP,DDIT3,CLCN3,HTT,PRMT1,CALM1,DISC1,INHBA,KDM3A,HOMER1	down_VSAP_EC
GO:2000145	0.0957	0.933	34	279	regulation of cell motility	BP	1	VEGFD,LGR6,TCAF1,RECK,BMP4,SH3RF2,TP53INP1,SASH1,LMO4,FGF18,F2RL1,NRP1,PTPN22,EVL,SPINT2,TBX5,PRKCE,DAG1,APP,LAMA3,SEMA6D,SYNJ2BP,JUP,CCL5,DDR2,DUSP22,EDN1,FAM107A,JAM3,WASL,DOCK5,RREB1,CRKL,PIK3C2A	up_ACOR_VSMC
GO:0006355	0.0957	0.981	3	974	regulation of transcription, DNA-	BP	1	HOXA9,WNT7A,ESR1	up_AORT_VSMC
GO:0072015	0.0957	0.989	2	5	glomerular visceral epithelial cell	BP	0.954	PODXL,JAG1	up_VCAV_EC
GO:0072310	0.0957	0.989	2	5	glomerular epithelial cell development	BP	0.954	PODXL,JAG1	up_VCAV_EC
GO:1904424	0.0957	0.988	2	6	regulation of GTP binding	BP	0.954	ARHGAP28,STMN1	up_VCAV_EC
GO:1902430	0.0957	0.998	1	2	negative regulation of amyloid-beta formation	BP	1	APOE	down_VSAP_EC
GO:1902992	0.0957	0.998	1	2	negative regulation of amyloid precursor protein catabolic process	BP	1	APOE	down_VSAP_EC
GO:0002792	0.0957	0.996	1	29	negative regulation of peptide secretion	BP	1	NR1H4	up_VPUL_EC
GO:0021543	0.0957	0.996	1	49	pallium development	BP	1	SLIT2	up_VPUL_VSMC
GO:0045089	0.0958	0.996	1	70	positive regulation of innate immune response	BP	1	TLR2	up_AFEM_VSMC
GO:0001678	0.0958	0.996	1	29	cellular glucose homeostasis	BP	1	NR1H4	up_VPUL_EC
GO:0061734	0.0958	1	1	1	parkin-mediated stimulation of mitophagy in response to	BP	1	MFN2	up_VSAP_EC
GO:1901605	0.0959	0.953	10	67	alpha-amino acid metabolic process	BP	1	PRDX4,ENSCAFG00000002977,NIT2,ERO1B,EGLN1,P4HA1,SARDH,AZIN2,NMNAT1,PARK7	up_ACOR_VSMC
GO:0001818	0.0959	0.953	10	65	negative regulation of cytokine production	BP	1	WNT11,CD274,C5AR2,HDAC7,DLL1,ANXA4,F2RL1,NLRX1,CD83,MAPKBP1	up_VSAP_EC
GO:0006022	0.0959	0.971	4	29	aminoglycan metabolic process	BP	1	ENSCAFG00000032470,CEMIP2,CHSY1,EXT1	down_VSAP_EC
GO:0071772	0.0959	0.996	1	50	response to BMP	BP	1	GATA5	up_VPUL_VSMC
GO:0071773	0.0959	0.996	1	50	cellular response to BMP stimulus	BP	1	GATA5	up_VPUL_VSMC
GO:0072507	0.096	0.985	2	103	divalent inorganic cation homeostasis	BP	1	LYN,HOXA3	down_ACOR_EC
GO:0050892	0.096	0.988	2	6	intestinal absorption	BP	1	LIMA1,EZR	down_ACOR_VSMC
GO:0002063	0.096	0.996	1	18	chondrocyte development	BP	1	SULF1	down_ACOR_EC
GO:0050804	0.096	0.996	1	87	modulation of chemical synaptic transmission	BP	1	WNT7A	up_AORT_VSMC
GO:0099177	0.096	0.996	1	87	regulation of trans-synaptic signaling	BP	1	WNT7A	up_AORT_VSMC
GO:0042060	0.0961	0.952	10	103	wound healing	BP	1	SERPINE1,IFRD1,PRCP,HIF1A,ADAM17,APOE,LYN,SLC7A11,ITGB3,SDC4	down_VSAP_EC
GO:0001654	0.0961	0.954	9	110	eye development	BP	1	PDGFRB,FBN1,NAGLU,CHRDL1,THY1,GLI3,IRX5,RDH10,THRB	down_VCAV_EC
GO:0048880	0.0961	0.954	9	110	sensory system development	BP	1	PDGFRB,FBN1,NAGLU,CHRDL1,THY1,GLI3,IRX5,RDH10,THRB	down_VCAV_EC
GO:0150063	0.0961	0.954	9	110	visual system development	BP	1	PDGFRB,FBN1,NAGLU,CHRDL1,THY1,GLI3,IRX5,RDH10,THRB	down_VCAV_EC
GO:2001234	0.0961	0.956	8	75	negative regulation of apoptotic signaling	BP	1	ARHGEF2,SERPINE1,HYOU1,PTGS2,HIF1A,CTNNB1,HTT,NRP1	down_VSAP_EC
GO:0030030	0.0961	0.986	2	377	cell projection organization	BP	1	SLITRK5,LIMK1	up_VCAV_VSMC
GO:0006478	0.0961	1	1	1	peptidyl-tyrosine sulfation	BP	1	TPST2	up_ACOR_VSMC

GO:0007249	0.0961	0.996	1	71	I-kappaB kinase/NF-kappaB signaling	BP	1	TLR2	up_AFEM_VSMC
GO:0046533	0.096175766	0.9964	1	4	negative regulation of photoreceptor cell	BP	1	SOX9	up_ACOR_EC
GO:0051336	0.0962	0.936	27	251	regulation of hydrolase activity	BP	0.959	SELE,AMOT,FGD6,MEF2C,RAPGEF1,CASP8,MTOR,BMP2,CHN2,PPP1R2,IQGAP2,PAK2,PRKD2,PTGS2,RAPGEF2,BCL10,HMGB1,TBC1D1,PMAIP1,ITGA6,KDM5A,ALS2,ENSCAFG00000002015,FKBP15,PSMB9,RABGAP1,USP6NL	up_VCAV_EC
GO:0061462	0.0962	0.996	1	15	protein localization to lysosome	BP	1	NEDD4	down_ACOR_EC
GO:0071326	0.0962	0.996	1	26	cellular response to monosaccharide stimulus	BP	1	NR1H4	up_APUL_EC
GO:0071331	0.0962	0.996	1	26	cellular response to hexose stimulus	BP	1	NR1H4	up_APUL_EC
GO:0043524	0.0962	0.996	1	39	negative regulation of neuron apoptotic process	BP	1	CHL1	up_VPOR_VSMC
GO:0007190	0.0962	1	1	1	activation of adenylate cyclase activity	BP	1	RAF1	up_VSAP_EC
GO:0071550	0.0962	1	1	1	death-inducing signaling complex assembly	BP	1	RAF1	up_VSAP_EC
GO:0007162	0.0963	0.954	9	66	negative regulation of cell adhesion	BP	0.96	PODXL,PELI1,ARG2,BMP2,ANGPT2,SWAP70,APOD,JAG1,HMGB1	up_VCAV_EC
GO:0045599	0.0963	0.978	3	19	negative regulation of fat cell differentiation	BP	1	ENPP1,TRIB3,DDIT3	down_VSAP_EC
GO:0007275	0.0963	0.984	3	1370	multicellular organism development	BP	1	TGFBI,GATA5,TGFBR1	down_VSAP_VSMC
GO:0072710	0.0963	0.988	2	6	response to hydroxyurea	BP	1	RAD51,TIMELESS	down_ACOR_VSMC
GO:0097329	0.0963	0.988	2	6	response to antimetabolite	BP	1	RAD51,TIMELESS	down_ACOR_VSMC
GO:0050678	0.0963	0.996	1	117	regulation of epithelial cell proliferation	BP	1	TGFBR1	down_VSAP_VSMC
GO:1905123	0.0963	1	1	1	regulation of glucosylceramidase	BP	1	SCARB2	up_ACOR_VSMC
GO:0060445	0.0963	0.996	1	13	branching involved in salivary gland	BP	1	SEMA3A	up_VPOR_EC
GO:0072524	0.0963	0.996	1	37	pyridine-containing compound metabolic	BP	1	FBP1	up_VFEM_VSMC
GO:0006083	0.0963	1	1	1	acetate metabolic process	BP	1	ACSS2	up_VSAP_EC
GO:0006208	0.0963	1	1	1	pyrimidine nucleobase catabolic process	BP	1	ALDH6A1	up_VSAP_EC
GO:0006210	0.0963	1	1	1	thymine catabolic process	BP	1	ALDH6A1	up_VSAP_EC
GO:0019427	0.0963	1	1	1	acetyl-CoA biosynthetic process from acetate	BP	1	ACSS2	up_VSAP_EC
GO:0019859	0.0963	1	1	1	thymine metabolic process	BP	1	ALDH6A1	up_VSAP_EC
GO:0038161	0.0963	1	1	1	prolactin signaling pathway	BP	1	CREBRF	up_VSAP_EC
GO:0043402	0.0963	1	1	1	glucocorticoid mediated signaling pathway	BP	1	CREBRF	up_VSAP_EC
GO:1900169	0.0963	1	1	1	regulation of glucocorticoid mediated	BP	1	CREBRF	up_VSAP_EC
GO:1900170	0.0963	1	1	1	negative regulation of glucocorticoid mediated signaling pathway	BP	1	CREBRF	up_VSAP_EC
GO:1902211	0.0963	1	1	1	regulation of prolactin signaling pathway	BP	1	CREBRF	up_VSAP_EC
GO:1902213	0.0963	1	1	1	positive regulation of prolactin signaling	BP	1	CREBRF	up_VSAP_EC
GO:0032268	0.0964	0.929	47	778	regulation of cellular protein metabolic process	BP	1	CST3,NOTCH2,PDGFRB,KIRREL1,LRP1,DCN,PRNP,GLIPR2,BGN,LARP6,BMPR1A,RECK,CLIP3,AKT1S1,TIMP2,AKT2,PSMF1,SAMD4B,EEF2K,PLCL1,CSPG4,PRMT6,ASPN,EIF3K,THY1,PARK7,EEF2,ZFP36L1,MYDGF,SASH1,CLEC16A,ARL2BP,IFT57,MAVS,CDH2,GSDME,SPRED1,A2M,MAPRE3,DDR2,SHC1,PYM1,FBH1,GREM1,FOXO3,PACSIN3,EIF4EBP1	down_VCAV_EC
GO:0043297	0.0964	0.978	3	17	apical junction assembly	BP	1	WDR1,MYO1C,CTNNB1	down_VSAP_EC
GO:1903506	0.0964	0.981	3	977	regulation of nucleic acid-templated transcription	BP	1	HOXA9,WNT7A,ESR1	up_AORT_VSMC
GO:0072358	0.0964	0.986	2	268	cardiovascular system development	BP	1	HDAC7,CITED2	down_VPOR_EC
GO:0060575	0.0964	0.987	2	9	intestinal epithelial cell differentiation	BP	1	HIF1A,KLF5	down_VSAP_EC

GO:0001944	0.0964	0.986	2	263	vasculature development	BP	1	HOXA3,SLIT2	up_VPUL_VSMC
GO:1903899	0.0964	0.998	1	2	positive regulation of PERK-mediated unfolded protein response	BP	1	PTPN2	down_VSAP_EC
GO:0032637	0.0964	0.996	1	26	interleukin-8 production	BP	1	BPI	up_APUL_EC
GO:1900076	0.0964	0.996	1	29	regulation of cellular response to insulin	BP	1	NR1H4	up_VPUL_EC
GO:0007597	0.0964	1	1	1	blood coagulation, intrinsic pathway	BP	1	KLKB1	up_VSAP_EC
GO:0051919	0.0964	1	1	1	positive regulation of fibrinolysis	BP	1	KLKB1	up_VSAP_EC
GO:0019538	0.0965	0.923	97	1713	protein metabolic process	BP	1	PCOLCE,CST3,ADAMTS2,NOTCH2,PDGFRB,KIRREL1,SMAD9,LRP1,DCN,CFD,PRNP,UBA52,GLIPR2,BGN,LARP6,COA1,BMPR1A,KRTCAP2,VKORC1,RECK,CLIP3,NUMBL,AKT1S1,TIMP2,AKT2,PSMF1,LCAT,RHBDL1,SAMD4B,EEF2K,HR,RPL23,RPL13,PCSK7,PLCL1,DDOST,CSPG4,PRMT6,GFPT2,ASPN,PIGS,HS3ST3B1,EIF3K,THY1,NECAB3,RNF216,WDR45,MRPL37,B4GALT1,GLI3,PARK7,RPL27A,EEF2,MAST2,ZFP36L1,MYDGF,SASH1,SEC11C,CLEC16A,RNPEP,RPL27,COPS7A,ARL2BP,IFT57,MAVS,MRPS11,CDH2,LOXL3,GSDME,CAMK2D,RPS27,FUT8,SPRED1,A2M,SEC61B,MAPRE3,CDKL2,HM13,PRKCSH,DDR2,SHC1,CRTAP,PCSK6,ENSCAFG00000015122,PYM1,FBH1,GREM1,RPN2,KLHL29,ATOX1,LONP2,FOXO3,SOAT1,PACSIN3,EIF4EBP1,BRSK1,CDK14	down_VCAV_EC
GO:0009968	0.0965	0.929	46	392	negative regulation of signal transduction	BP	1	SMAD6,LBH,HTR2B,RASSF2,PRKAA2,MLLT3,FERMT1,BMP4,ELF1,CASTOR1,LATS2,ZNRF3,AR,PTGIR,NDRG2,ITPR1,F2RL1,DAPK1,CLSTN1,CREBRF,NRP1,UBR1,PTPN22,UNC5B,SMPD1,APLP1,TBX20,SESN3,SH3RF1,CCDC3,RGS2,DAG1,PSEN1,SYNJ2BP,WWC2,DUSP22,SESN1,DUSP26,LRRC4,DDIT4,HERPUD1,INPP5K,BMT2,WFS1,SH3BP4,PARK7	up_ACOR_VSMC
GO:0090314	0.0965	0.986	2	11	positive regulation of protein targeting to	BP	1	PRNP,AKT2	down_VCAV_EC
GO:0045844	0.0965	0.996	1	24	positive regulation of striated muscle tissue	BP	1	CYP26B1	up_VFEM_EC
GO:0048636	0.0965	0.996	1	24	positive regulation of muscle organ	BP	1	CYP26B1	up_VFEM_EC
GO:0048634	0.0965	0.996	1	40	regulation of muscle organ development	BP	1	SHOX2	up_VFEM_VSMC
GO:0042044	0.0966	0.988	2	6	fluid transport	BP	1	ITPR1,INPP5K	up_ACOR_VSMC
GO:0030512	0.0966	0.996	1	24	negative regulation of transforming growth factor beta receptor	BP	1	CAV1	up_VSAP_VSMC
GO:1903845	0.0966	0.996	1	24	negative regulation of cellular response to transforming growth	BP	1	CAV1	up_VSAP_VSMC
GO:1903391	0.09664098	0.98511	2	21	regulation of adherens junction organization	BP	1	ARHGAP6,NRP1	up_ACOR_EC
GO:0050864	0.0967	0.963	6	36	regulation of B cell activation	BP	0.961	TNFAIP3,MEF2C,TLR4,PELI1,THOC1,ATP11C	up_VCAV_EC
GO:0043407	0.0967	0.978	3	19	negative regulation of MAP kinase activity	BP	1	APOE,LYN,CBLC	down_VSAP_EC
GO:2001141	0.0967	0.981	3	979	regulation of RNA biosynthetic process	BP	1	HOXA9,WNT7A,ESR1	up_AORT_VSMC
GO:0071300	0.0967	0.979	3	12	cellular response to retinoic acid	BP	1	OSR1,RARG,HTRA2	up_VSAP_EC
GO:0001914	0.0967	0.987	2	8	regulation of T cell mediated cytotoxicity	BP	0.961	IL7R,NECTIN2	up_VCAV_EC
GO:0031349	0.0967	0.996	1	90	positive regulation of defense response	BP	1	ESR1	up_AORT_VSMC
GO:0032602	0.0967	0.996	1	23	chemokine production	BP	1	TLR2	up_VSAP_VSMC
GO:0009314	0.0968	0.942	17	143	response to radiation	BP	1	USP1,HMGCR,SMC1A,NEDD4,INTS7,RAD54L,RAD51,AURKB,TOPBP1,MAP3K20,PLK3,PCNA,NOC2L,POLH,DTL,CDC25A,HMGN1	down_ACOR_VSMC
GO:1901362	0.0968	0.955	10	1293	organic cyclic compound biosynthetic process	BP	1	ACVRL1,YEATS2,YBX3,HIVEP2,HOXA7,SULF1,ELK3,HOXA1,TNRC6B,NEDD4	down_ACOR_EC
GO:0014033	0.0968	0.967	5	30	neural crest cell differentiation	BP	0.961	EDNRB,MEF2C,EDN1,JAG1,HIF1A	up_VCAV_EC
GO:0033046	0.0968	0.972	4	21	negative regulation of sister chromatid	BP	0.961	RAD21,ZW10,ATRX,FBXO5	up_VCAV_EC

GO:0033048	0.0968	0.972	4	21	negative regulation of mitotic sister chromatid	BP	0.961	RAD21,ZW10,ATRX,FBXO5	up_VCAV_EC
GO:0051985	0.0968	0.972	4	21	negative regulation of chromosome segregation	BP	0.961	RAD21,ZW10,ATRX,FBXO5	up_VCAV_EC
GO:0071825	0.0968	0.979	3	12	protein-lipid complex subunit organization	BP	1	LIPC,LCAT,SOAT1	up_VSAP_EC
GO:0071827	0.0968	0.979	3	12	plasma lipoprotein particle organization	BP	1	LIPC,LCAT,SOAT1	up_VSAP_EC
GO:1903778	0.0968	0.998	1	2	protein localization to vacuolar membrane	BP	1	SH3GLB1	down_VSAP_EC
GO:0050905	0.0968	0.996	1	26	neuromuscular process	BP	1	HOXA1	up_APUL_EC
GO:0002573	0.0968	0.996	1	71	myeloid leukocyte differentiation	BP	1	LRRC17	up_VCAV_VSMC
GO:0001974	0.0968	0.996	1	13	blood vessel remodeling	BP	1	HOXA3	up_VPOR_EC
GO:0001757	0.0968	1	1	1	somite specification	BP	1	DLL1	up_VSAP_EC
GO:0002085	0.0968	1	1	1	inhibition of neuroepithelial cell	BP	1	DLL1	up_VSAP_EC
GO:0007386	0.0968	1	1	1	compartment pattern specification	BP	1	DLL1	up_VSAP_EC
GO:0014807	0.0968	1	1	1	regulation of somitogenesis	BP	1	DLL1	up_VSAP_EC
GO:0021688	0.0968	1	1	1	cerebellar molecular layer formation	BP	1	DLL1	up_VSAP_EC
GO:0021693	0.0968	1	1	1	cerebellar Purkinje cell layer structural	BP	1	DLL1	up_VSAP_EC
GO:0021698	0.0968	1	1	1	cerebellar cortex structural organization	BP	1	DLL1	up_VSAP_EC
GO:0045168	0.0968	1	1	1	cell-cell signaling involved in cell fate commitment	BP	1	DLL1	up_VSAP_EC
GO:0045608	0.0968	1	1	1	negative regulation of inner ear auditory receptor cell	BP	1	DLL1	up_VSAP_EC
GO:0045632	0.0968	1	1	1	negative regulation of mechanoreceptor	BP	1	DLL1	up_VSAP_EC
GO:0046331	0.0968	1	1	1	lateral inhibition	BP	1	DLL1	up_VSAP_EC
GO:0048630	0.0968	1	1	1	skeletal muscle tissue growth	BP	1	DLL1	up_VSAP_EC
GO:0048631	0.0968	1	1	1	regulation of skeletal muscle tissue growth	BP	1	DLL1	up_VSAP_EC
GO:0048633	0.0968	1	1	1	positive regulation of skeletal muscle tissue	BP	1	DLL1	up_VSAP_EC
GO:0060853	0.0968	1	1	1	Notch signaling pathway involved in arterial endothelial cell fate	BP	1	DLL1	up_VSAP_EC
GO:0097102	0.0968	1	1	1	endothelial tip cell fate specification	BP	1	DLL1	up_VSAP_EC
GO:2000981	0.0968	1	1	1	negative regulation of inner ear receptor cell	BP	1	DLL1	up_VSAP_EC
GO:0009308	0.096821377	0.98512	2	20	amine metabolic process	BP	1	NR1H4,AZIN2	up_ACOR_EC
GO:0046426	0.096870147	0.98511	2	20	negative regulation of JAK-STAT cascade	BP	1	LRRC15,LRRC4	up_ACOR_EC
GO:1904893	0.096870147	0.98511	2	20	negative regulation of STAT cascade	BP	1	LRRC15,LRRC4	up_ACOR_EC
GO:0003161	0.0969	0.988	2	6	cardiac conduction system development	BP	1	TBX5,BVES	up_ACOR_VSMC
GO:0007571	0.0969	1	1	1	age-dependent general metabolic decline	BP	1	CLN8	up_ACOR_VSMC
GO:0051935	0.0969	1	1	1	glutamate reuptake	BP	1	CLN8	up_ACOR_VSMC
GO:0001306	0.0969	1	1	1	age-dependent response to oxidative stress	BP	1	CLN8	up_ACOR_VSMC
GO:0021523	0.0969	1	1	1	somatic motor neuron differentiation	BP	1	CLN8	up_ACOR_VSMC
GO:0051933	0.0969	1	1	1	amino acid neurotransmitter	BP	1	CLN8	up_ACOR_VSMC
GO:1903672	0.096938297	0.98509	2	20	positive regulation of sprouting angiogenesis	BP	1	FOXC2,NRP1	up_ACOR_EC
GO:0038166	0.096972227	0.99634	1	4	angiotensin-activated signaling pathway	BP	1	ACTN2	up_ACOR_EC

GO:0030154	0.097	0.921	111	1040	cell differentiation	BP	1	HOXA11,FZD1,THRB,EMX2,HOXA7,PEX7,NAPEPLD,TWIST2,HOXA9,CDON,WNT11,HNF4A,ACKR3,AXIN2,WASF3,OSR2,AMIGO1,RADIL,PPARGC1B,GJB5,IRX5,SLIT2,ZHX3,HOXD3,ABL1,EBF2,BTG1,HDAC7,EMP2,DLL1,OSR1,MAP3K5,ADAMTSL4,WARS2,SAV1,KLF15,ANXA4,PLXNA4,ARL4A,RILPL2,ANKRD54,ZBTB16,TOX,ALDH6A1,RARG,STAT6,PATZ1,PLPP3,GTF3C5,F2RL1,DLX3,GLIS2,DDR2,ZFP36L1,PIK3R1,TIAM1,GAS1,BATF2,MYC,IRX3,ZEB1,SULF2,SHOX2,EEF2,RTN4RL1,HOXC10,MYD88,TFE3,LRP4,DPYSL2,GDPD5,ZADH2,DAGLB,HPS4,FRMD6,CLCN2,E2F4,FBXO7,NTN4,LIMD1,CDK9,CD83,NR2F6,FBLN1,ZFP36L2,PLEKHB2,RAF1,PNP,TAF8,NFIB,CYB5D2,CDNF,ANPEP,PQBP1,TTL,ACVRL1,HTRA2,FLOT1,PPP3CB,NCK1,BGLAP,ULK1,MAP2K2,TGFBR2,CITED2,OGN,PIR,LBH,KEAP1,SOAT1,LATS2	up_VSAP_EC
GO:0050878	0.097	0.95	11	75	regulation of body fluid levels	BP	1	HNF4A,KLKB1,EMP2,CHRM1,PDGFD,HPSE,CLDN1,F2RL1,HPS4,FBLN1,CYBA	up_VSAP_EC
GO:0019229	0.097	0.979	3	12	regulation of vasoconstriction	BP	1	CHRM1,TBXA2R,AGTR1	up_VSAP_EC
GO:0060429	0.097	0.998	1	341	epithelium development	BP	1	GATA5	down_VFEM_VSMC
GO:1901135	0.0971	0.935	26	390	carbohydrate derivative metabolic process	BP	1	GPAM,DCN,ADCY6,KRTCAP2,CHID1,H6PD,DDOST,NT5C2,PGLS,ATP5MC1,GFPT2,UQCR10,PIGS,NECAB3,ADK,B4GALT1,PARK7,AMPD2,FUT8,ENSCAFG00000017708,ACSS2,PRKCSH,PCSK6,RPN2,SOAT1,ACAT1	down_VCAV_EC
GO:1901361	0.0971	0.941	18	154	organic cyclic compound catabolic process	BP	1	RNPS1,EIF4A3,EDC3,HNRNPD,THRAP3,EDC4,FEN1,RNASEH2B,SMG9,DIS3,FASTKD3,PDXP,AIFM1,ENSCAFG00000015189,RNASEH2C,NUDT1,EXOSC5,NUDT3	down_ACOR_VSMC
GO:0060284	0.0971	0.944	15	212	regulation of cell development	BP	1	NOTCH2,FBN1,BMPR1A,TRPV2,NUMBL,SEMA3C,THY1,TLX2,GLI3,CDH2,UST,PRKCSH,THRB,RGMA,BRSK1	down_VCAV_EC
GO:0034248	0.0971	0.951	10	125	regulation of cellular amide metabolic process	BP	1	PRNP,LARP6,SAMD4B,EIF3K,EEF2,ZFP36L1,ORMDL3,PYM1,FOXO3,EIF4EBP1	down_VCAV_EC
GO:0030335	0.0971	0.958	7	165	positive regulation of cell migration	BP	1	CMKLR1,ANGPT4,TWIST2,LRRC15,LGR6,CCL5,ADAM17	up_AFEM_EC
GO:0035556	0.0971	0.964	6	845	intracellular signal transduction	BP	1	NR1H4,MAPK10,PLCL1,ARHGEF26,NPNT,NTRK2	up_APUL_EC
GO:0046173	0.0971	0.972	4	19	polyol biosynthetic process	BP	1	PLCG2,PCBD1,PPIP5K1,PTS	up_ACOR_VSMC
GO:0010596	0.0971	0.995	1	21	negative regulation of endothelial cell migration	BP	1	ACVRL1	down_ACOR_EC
GO:1902743	0.0971	0.996	1	16	regulation of lamellipodium	BP	1	PDPN	down_ACOR_EC
GO:0003002	0.0971	0.996	1	118	regionalization	BP	1	GATA5	down_VSAP_VSMC
GO:0021537	0.0971	0.996	1	73	telencephalon development	BP	1	SLITRK5	up_VCAV_VSMC
GO:0048537	0.0971	1	1	1	mucosal-associated lymphoid tissue	BP	1	TOX	up_VSAP_EC
GO:0048541	0.0971	1	1	1	Peyer's patch development	BP	1	TOX	up_VSAP_EC
GO:0006468	0.0972	0.925	62	544	protein phosphorylation	BP	1	FGF13,ADRA1B,SMAD6,LEP,PLCL1,MAP3K8,MID1,CDC7,HTR2B,RASSF2,MBP,PRKAA2,BMP4,FGFR2,TLR3,SH3RF2,LATS2,PDE5A,SASH1,FGF18,NDRG2,CDKN2B,GRK7,F2RL1,FAM129A,CAMK2D,DAPK1,CDKN1A,NRP1,PTPN22,ULK2,DMPK,NPNT,SMPD1,SH3RF1,PRKCE,CCNT1,FRS2,DAG1,PARP9,APP,PSEN1,SYNJ2BP,PIK3C3,CCL5,DDR2,PSKH1,DUSP22,EDN1,AKTIP,DUSP26,NOD2,LRRC4,LMTK2,PRKAG3,SH3RF3,MAP3K7,INPP5K,ENSCAFG00000029553,PARK7,CRKL,CDKL2	up_ACOR_VSMC
GO:0050906	0.0972	0.979	3	13	detection of stimulus involved in sensory	BP	0.964	EPHB1,KIT,MMP24	up_VCAV_EC
GO:0009611	0.0972	0.985	2	122	response to wounding	BP	1	CAV1,F5	up_VSAP_VSMC
GO:0050684	0.0972	0.996	1	46	regulation of mRNA processing	BP	1	SRSF6	down_AFEM_EC
GO:0140074	0.0972	0.998	1	2	cardiac endothelial to mesenchymal transition	BP	1	SEMA3C	down_VCAV_EC
GO:0003150	0.0972	1	1	1	muscular septum morphogenesis	BP	1	FZD1	up_VSAP_EC
GO:0035425	0.0972	1	1	1	autocrine signaling	BP	1	FZD1	up_VSAP_EC

GO:0044338	0.0972	1	1	1	canonical Wnt signaling pathway involved in mesenchymal stem cell	BP	1	FZD1	up_VSAP_EC
GO:0060022	0.0972	1	1	1	hard palate development	BP	1	FZD1	up_VSAP_EC
GO:0045834	0.09728099	0.9763	3	41	positive regulation of lipid metabolic process	BP	1	NR1H4,CCDC3,PRKCD	up_ACOR_EC
GO:2000317	0.0973	0.998	1	2	negative regulation of T-helper 17 type immune	BP	1	LOXL3	down_VCAV_EC
GO:2000319	0.0973	0.998	1	2	regulation of T-helper 17 cell differentiation	BP	1	LOXL3	down_VCAV_EC
GO:2000320	0.0973	0.998	1	2	negative regulation of T-helper 17 cell	BP	1	LOXL3	down_VCAV_EC
GO:0034334	0.0973	0.998	1	2	adherens junction maintenance	BP	1	RASSF8	down_VCAV_EC
GO:2000316	0.0973	0.998	1	2	regulation of T-helper 17 type immune response	BP	1	LOXL3	down_VCAV_EC
GO:0043648	0.0973	0.995	1	26	dicarboxylic acid metabolic process	BP	1	NR1H4	up_APUL_EC
GO:0046462	0.0973	1	1	1	monoacylglycerol metabolic process	BP	1	ABHD16A	up_VSAP_EC
GO:0052651	0.0973	1	1	1	monoacylglycerol catabolic process	BP	1	ABHD16A	up_VSAP_EC
GO:1901523	0.0973	1	1	1	icosanoid catabolic process	BP	1	ABHD16A	up_VSAP_EC
GO:1905344	0.0973	1	1	1	prostaglandin catabolic process	BP	1	ABHD16A	up_VSAP_EC
GO:0019538	0.097353617	0.9285	53	1713	protein metabolic process	BP	1	C7,NR1H4,BRSK1,PLCL1,PKIB,SOX9,GALNT5,PCSK1N,PHF20,NDRG2,PORCN,ENSCAFG00000004331,EDEM2,PI3,FZD5,IFI6,DUSP26,NOD2,SMAD6,B3GNT7,KLHL25,ZMYND8,PAK3,GRK7,TNFRSF19,CST3,PIAS1,DCLK2,USP53,MAPK8IP2,MAVS,LRRC15,KLHDC8B,PIGS,LRP4,SH3RF3,LRRC4,CLU,CHMP4B,TP53INP2,RARRES2,AGBL5,RIOX2,RNF114,UBE2H,STK32C,NRP1,AZIN2,HM13,PRKCD,PYGO2,DBNDD2,CCNYL1	up_ACOR_EC
GO:0051293	0.0974	0.978	3	13	establishment of spindle localization	BP	1	ZW10,CENPA,NDC80	down_ACOR_VSMC
GO:0045869	0.0974	1	1	1	negative regulation of single stranded viral RNA replication via double stranded DNA	BP	1	INPP5K	up_ACOR_VSMC
GO:2000466	0.0974	1	1	1	negative regulation of glycogen (starch) synthase	BP	1	INPP5K	up_ACOR_VSMC
GO:2001151	0.0974	1	1	1	regulation of renal water transport	BP	1	INPP5K	up_ACOR_VSMC
GO:2001153	0.0974	1	1	1	positive regulation of renal water transport	BP	1	INPP5K	up_ACOR_VSMC
GO:0007019	0.0974	0.996	1	13	microtubule depolymerization	BP	1	ARHGEF2	up_VPOR_EC
GO:0048536	0.0974	0.996	1	12	spleen development	BP	1	BARX1	up_VPOR_EC
GO:0035850	0.0974	0.995	1	25	epithelial cell differentiation involved in	BP	1	MTSS1	up_VFEM_EC
GO:0051262	0.0974	0.995	1	40	protein tetramerization	BP	1	FBP1	up_VFEM_VSMC
GO:0007624	0.0974	1	1	1	ultradian rhythm	BP	1	ENOX1	up_VSAP_EC
GO:0010638	0.0975	0.937	23	207	positive regulation of organelle organization	BP	1	FSCN1,SREBF1,HNRNPD,SDC1,BIRC5,NF2,AURKB,NEK2,FEN1,POC1A,BRCA1,RAC1,NUP62,PDXP,PLK4,IQGAP2,CDC42EP4,ENSCAFG00000005299,CORO1A,ARPC1B,TADA2A,UBE2C,CCT4	down_ACOR_VSMC
GO:0060537	0.0975	0.943	16	117	muscle tissue development	BP	1	TNNT2,ACTN2,BMP4,FGFR2,GJA1,PPARGC1A,CITED2,NEBL,ANKRD1,EGLN1,TBX20,TBX5,FRS2,CHD7,EDN1,BVES	up_ACOR_VSMC
GO:0002685	0.0975	0.961	6	55	regulation of leukocyte migration	BP	1	VEGFA,SERPINE1,MSN,ADAM17,MMP28,LYN	down_VSAP_EC
GO:0010811	0.0975	0.97	4	36	positive regulation of cell-substrate adhesion	BP	1	FBLN2,THY1,EDIL3,ECM2	down_VCAV_EC
GO:0051642	0.0975	0.978	3	13	centrosome localization	BP	1	EZR,ASPM,CEP83	down_ACOR_VSMC
GO:0061842	0.0975	0.978	3	13	microtubule organizing center localization	BP	1	EZR,ASPM,CEP83	down_ACOR_VSMC
GO:0060343	0.0975	0.986	2	11	trabecula formation	BP	1	THBS3,GREM1	down_VCAV_EC
GO:0051899	0.0975	0.995	1	22	membrane depolarization	BP	1	CAV1	up_VSAP_VSMC

GO:0003174	0.097575049	0.9963	1	4	mitral valve development	BP	1	SMAD6	up_ACOR_EC
GO:0051279	0.0976	0.977	3	19	regulation of release of sequestered calcium ion into cytosol	BP	1	LYN,HTT,CALM1	down_VSAP_EC
GO:0051494	0.0976	0.995	1	44	negative regulation of cytoskeleton organization	BP	1	TAOK1	up_AORT_EC
GO:0032088	0.0976	0.995	1	26	negative regulation of NF-kappaB transcription	BP	1	NR1H4	up_APUL_EC
GO:0046323	0.0976	0.995	1	26	glucose import	BP	1	NR1H4	up_APUL_EC
GO:0030258	0.0976	0.996	1	71	lipid modification	BP	1	CYP1A1	up_VCAV_VSMC
GO:0010862	0.0976	0.996	1	12	positive regulation of pathway-restricted SMAD protein phosphorylation	BP	1	ACVR2A	up_VPOR_EC
GO:1901317	0.0976	1	1	1	regulation of flagellated sperm motility	BP	1	CFAP69	up_VSAP_EC
GO:1902093	0.0976	1	1	1	positive regulation of flagellated sperm motility	BP	1	CFAP69	up_VSAP_EC
GO:1905516	0.0976	1	1	1	positive regulation of fertilization	BP	1	CFAP69	up_VSAP_EC
GO:0010605	0.0977	0.926	54	745	negative regulation of macromolecule metabolic process	BP	1	PRICKLE1,NR1H4,PLK3,SERPINE1,SOCS5,ETV6,ENPP1,ERRFI1,SNX25,CBX5,TRIB3,FHL2,SMARCA4,SESN2,PTGS2,HIF1A,DNAJC10,NXN,PTPN2,UCHL5,MORF4L2,SPOPL,PEG3,SMG7,MTDH,EIF4EBP1,CNOT7,MAGED1,CTNNB1,CRYAB,SND1,ENSCAFG00000002015,APOE,WWC3,KLF5,LYN,STRAP,DDIT3,CBLC,ATF3,VLDLR,REST,ZMYND8,EIF2S1,CDYL,PAK2,LRRTM2,A2M,HIF1AN,SIX4,CDKN1A,SPINK2,USP14,KDM3A	down_VSAP_EC
GO:0043588	0.0977	0.97	4	74	skin development	BP	1	HOXA7,DLL1,OVOL1,FOSL2	up_AFEM_EC
GO:0002833	0.0977	0.979	3	12	positive regulation of response to biotic	BP	1	CD274,SASH1,F2RL1	up_ACOR_VSMC
GO:0071310	0.0977	0.988	2	634	cellular response to organic substance	BP	1	GATA5,TGFBR1	down_VSAP_VSMC
GO:0032000	0.0977	0.997	1	2	positive regulation of fatty acid beta-oxidation	BP	1	AKT2	down_VCAV_EC
GO:0017145	0.0977	0.996	1	13	stem cell division	BP	1	ARHGEF2	up_VPOR_EC
GO:0032411	0.0977	0.995	1	24	positive regulation of transporter activity	BP	1	STIM1	up_VSAP_VSMC
GO:0008152	0.0978	0.967	11	3714	metabolic process	BP	1	ETFA,IVNS1ABP,EXTL2,DARS,SUCLG2,EIF3M,SRSF6,USP33,GABPA,NDUFA10,TSEN15	down_AFEM_EC
GO:0002262	0.0978	0.961	6	54	myeloid cell homeostasis	BP	1	CASP3,HIF1A,PTPN2,LYN,PRMT1,INHBA	down_VSAP_EC
GO:0006793	0.0978	0.964	6	948	phosphorus metabolic process	BP	1	NR1H4,STK32B,CCL24,PTGER2,FLRT3,ARHGEF2	up_VPUL_EC
GO:0002709	0.0978	0.972	4	19	regulation of T cell mediated immunity	BP	1	DUSP22,B2M,MAP3K7,IL18R1	up_ACOR_VSMC
GO:0060538	0.0978	0.995	1	51	skeletal muscle organ development	BP	1	CITED2	down_VPOR_EC
GO:2000027	0.0978	0.996	1	72	regulation of animal organ morphogenesis	BP	1	EYA1	up_AFEM_VSMC
GO:0043568	0.097868676	0.99627	1	4	positive regulation of insulin-like growth factor receptor signaling	BP	1	CDH3	up_ACOR_EC
GO:0071310	0.0979	0.924	71	634	cellular response to organic substance	BP	1	SMAD6,ACTN2,IFNGR1,LEP,LBH,GATA5,SKP2,PRKAA2,FERMT1,BMP4,TM7SF3,FGFR2,ABCA1,EIF4A2,MED14,CASTOR1,LATS2,CITED2,TP53INP1,AR,SASH1,SLC2A5,TMEM108,FGF18,FAM210B,CDKN2B,ADCY6,ANKRD1,F2RL1,DAPK1,CTSB,AKAP6,CREBRF,NRP1,UBR1,PTPN22,NPNT,EVL,APLP1,PTGDR,SESN3,CCDC3,PRKCE,FRS2,AGRN,FUT8,PARP9,APP,PSEN1,TGFBR1,JUP,CCL5,NR3C1,DUSP22,RAB11B,HPGD,NOD2,LRRC4,ARID5B,FAM107A,DDIT4,ENSCAFG00000018360,HERPUD1,GNAQ,INPP5K,WFS1,DZIP3,SH3BP4,PARK7,STC2,CRKL	up_ACOR_VSMC
GO:1904375	0.0979	0.97	4	35	regulation of protein localization to cell	BP	1	LRP1,PRNP,CLIP3,ENSCAFG00000014978	down_VCAV_EC
GO:0007260	0.0979	0.977	3	19	tyrosine phosphorylation of STAT protein	BP	1	PTPN2,CNOT7,LYN	down_VSAP_EC
GO:0048592	0.0979	0.995	1	53	eye morphogenesis	BP	1	CITED2	down_VPOR_EC
GO:0006613	0.0979	0.996	1	12	cotranslational protein targeting to membrane	BP	1	SRP54	up_VPOR_EC

GO:1900186	0.0979	1	1	1	negative regulation of clathrin-dependent	BP	1	UBQLN2	up_VSAP_EC
GO:1904020	0.0979	1	1	1	regulation of G protein-coupled receptor	BP	1	UBQLN2	up_VSAP_EC
GO:1904021	0.0979	1	1	1	negative regulation of G protein-coupled receptor	BP	1	UBQLN2	up_VSAP_EC
GO:0016125	0.098	0.959	7	41	sterol metabolic process	BP	1	LEP,LCAT,ABCA1,LIPE,APP,CLN8,LMF1	up_ACOR_VSMC
GO:0048812	0.098	0.985	2	151	neuron projection morphogenesis	BP	1	FLRT3,NEO1	up_VPUL_EC
GO:0042472	0.098	0.995	1	27	inner ear morphogenesis	BP	1	HOXA1	up_APUL_EC
GO:0010868	0.098	1	1	1	negative regulation of triglyceride biosynthetic	BP	0.969	ENSCAFG00000010596	up_VCAV_EC
GO:1902905	0.098	0.996	1	72	positive regulation of supramolecular fiber	BP	1	LIMK1	up_VCAV_VSMC
GO:0055072	0.098	0.995	1	30	iron ion homeostasis	BP	1	NEO1	up_VPUL_EC
GO:0030326	0.098	0.995	1	51	embryonic limb morphogenesis	BP	1	TBX5	up_VPUL_VSMC
GO:0035113	0.098	0.995	1	51	embryonic appendage morphogenesis	BP	1	TBX5	up_VPUL_VSMC
GO:0032755	0.098	0.995	1	24	positive regulation of interleukin-6 production	BP	1	TLR2	up_VSAP_VSMC
GO:0007423	0.0981	0.94	18	155	sensory organ development	BP	0.969	EPHB1,ARHGEF15,CHD7,DLL4,GATA3,EDN1,TMOD1,BMP2,PROX1,TSPAN12,SMARCA4,SLC25A25,JAG1,SKIL,HIF1A,ALDH1A3,ZEB1,CDON	up_VCAV_EC
GO:0010970	0.0981	0.956	8	48	transport along microtubule	BP	1	RHOT1,TMEM108,IFT43,APP,DYNC1I2,SUN1,DYNC1I1,ENSCAFG00000013536	up_ACOR_VSMC
GO:0001890	0.0981	0.956	8	51	placenta development	BP	1	LEP,FGFR2,CITED2,SPINT1,CTSB,EGLN1,ENSCAFG00000006238,SPINT2	up_ACOR_VSMC
GO:0099111	0.0981	0.956	8	48	microtubule-based transport	BP	1	RHOT1,TMEM108,IFT43,APP,DYNC1I2,SUN1,DYNC1I1,ENSCAFG00000013536	up_ACOR_VSMC
GO:0007215	0.0981	0.978	3	13	glutamate receptor signaling pathway	BP	0.969	RELN,MEF2C,GRM8	up_VCAV_EC
GO:0097205	0.0981	0.979	3	12	renal filtration	BP	1	EMP2,SULF2,JCHAIN	up_VSAP_EC
GO:0034641	0.0981	1	2	2171	cellular nitrogen compound metabolic	BP	1	JUP,GATA5	down_VFEM_VSMC
GO:0071800	0.0981	0.987	2	8	podosome assembly	BP	1	FSCN1,MSN	down_VSAP_EC
GO:1901165	0.0981	0.997	1	2	positive regulation of trophoblast cell migration	BP	1	SYDE1	down_VCAV_EC
GO:1903984	0.0981	0.997	1	2	positive regulation of TRAIL-activated apoptotic signaling pathway	BP	1	ATF3	down_VSAP_EC
GO:0009966	0.0982	0.921	95	868	regulation of signal transduction	BP	1	SH3TC2,ADRA1B,TLR1,SMAD6,LGR6,LEP,LBH,MID1,GATA5,HTR2B,RASSF2,ZRANB1,C28H10orf71,RECK,SKP2,NACC2,PRKAA2,MLLT3,ITM2C,FERMT1,BMP4,FGFR2,ABCA1,ELF1,CASTOR1,TLR3,SH3RF2,LATS2,PDE5A,CITED2,TP53INP1,ZNRF3,AR,PTGIR,SASH1,TMEM108,FGF18,ARHGEF26,NDRG2,CHN2,CDKN2B,ITPR1,LIMS2,ANKRD1,F2RL1,DAPK1,BNIP3,CRLF3,CLSTN1,CREBRF,NRP1,UBR1,PTPN22,DMPK,NPNT,UNC5B,SMPD1,APLP1,TBX20,SESN3,SH3RF1,CCDC3,PRKCE,FRS2,RGS2,DAG1,PARP9,APP,PSEN1,SYNJ2BP,SIPA1L1,CCL5,WWC2,DUSP22,SIAH1,EDN1,SESN1,DUSP26,NOD2,LRRC4,DDIT4,DISC1,SH3RF3,SERINC3,USP20,HERPUD1,MAP3K7,INPP5K,BMT2,WFS1,SH3BP4,ENSCAFG00000029553,PARK7,IL18R1,CRKL	up_ACOR_VSMC
GO:0051049	0.0982	0.927	47	479	regulation of transport	BP	0.969	HCLS1,SELE,ATP8A1,MX2,PDGFB,TMEM173,RELN,PDE4D,IWS1,MEF2C,FERMT1,CHD7,TLR4,STC1,GATA3,EDN1,RAB11A,APLNR,RAB27A,CTTNBP2NL,SNCAIP,PPP3CA,YES1,ARG2,BMP2K,RSC1A1,ABCA1,KCNJ3,SH3GLB1,APOD,KCNJ2,CD200,FES,HIST1H1A,PLCG2,CTDSPL2,CREB1,GCC2,HMGB1,TBC1D1,SPTBN4,RIPK1,MCU,DNAJC13,HIF1A,RABGAP1,USP6NL	up_VCAV_EC
GO:0034121	0.0982	0.995	1	22	regulation of toll-like receptor signaling	BP	1	LYN	down_ACOR_EC
GO:0006063	0.0982	1	1	1	uronic acid metabolic process	BP	1	PRKCE	up_ACOR_VSMC
GO:0019585	0.0982	1	1	1	glucuronate metabolic process	BP	1	PRKCE	up_ACOR_VSMC

GO:0030473	0.0982	1	1	1	nuclear migration along microtubule	BP	1	SUN1	up_ACOR_VSMC
GO:0043578	0.0982	1	1	1	nuclear matrix organization	BP	1	SUN1	up_ACOR_VSMC
GO:0070197	0.0982	1	1	1	meiotic attachment of telomere to nuclear	BP	1	SUN1	up_ACOR_VSMC
GO:0090286	0.0982	1	1	1	cytoskeletal anchoring at nuclear membrane	BP	1	SUN1	up_ACOR_VSMC
GO:0090292	0.0982	1	1	1	nuclear matrix anchoring at nuclear membrane	BP	1	SUN1	up_ACOR_VSMC
GO:0097240	0.0982	1	1	1	chromosome attachment to the nuclear envelope	BP	1	SUN1	up_ACOR_VSMC
GO:2001029	0.0982	1	1	1	regulation of cellular glucuronidation	BP	1	PRKCE	up_ACOR_VSMC
GO:2001031	0.0982	1	1	1	positive regulation of cellular glucuronidation	BP	1	PRKCE	up_ACOR_VSMC
GO:0021817	0.0982	1	1	1	nucleokinesis involved in cell motility in cerebral cortex radial glia guided	BP	1	SUN1	up_ACOR_VSMC
GO:0035668	0.0982	1	1	1	TRAM-dependent toll-like receptor signaling	BP	1	PRKCE	up_ACOR_VSMC
GO:0035669	0.0982	1	1	1	TRAM-dependent toll-like receptor 4 signaling	BP	1	PRKCE	up_ACOR_VSMC
GO:0044821	0.0982	1	1	1	meiotic telomere tethering at nuclear	BP	1	SUN1	up_ACOR_VSMC
GO:0052695	0.0982	1	1	1	cellular glucuronidation	BP	1	PRKCE	up_ACOR_VSMC
GO:0045637	0.0982	0.996	1	73	regulation of myeloid cell differentiation	BP	1	HOXA9	up_AFEM_VSMC
GO:0006114	0.0982	1	1	1	glycerol biosynthetic process	BP	1	GOT1	up_VSAP_EC
GO:0006532	0.0982	1	1	1	aspartate biosynthetic process	BP	1	GOT1	up_VSAP_EC
GO:0019401	0.0982	1	1	1	alditol biosynthetic process	BP	1	GOT1	up_VSAP_EC
GO:0019550	0.0982	1	1	1	glutamate catabolic process to aspartate	BP	1	GOT1	up_VSAP_EC
GO:0019551	0.0982	1	1	1	glutamate catabolic process to 2-oxoglutarate	BP	1	GOT1	up_VSAP_EC
GO:0048812	0.0983	0.944	14	151	neuron projection morphogenesis	BP	1	SIPA1L1,SGK1,PTPN11,IFRD1,KIF5B,TNIK,NEO1,UST,CDH11,CTNNB1,VLDLR,EXT1,NRP1,YWHAH	down_VSAP_EC
GO:0046165	0.0983	0.962	6	42	alcohol biosynthetic process	BP	0.969	SPTLC2,BMP2,ITPKB,PLCG2,GCH1,IMPA1	up_VCAV_EC
GO:0048813	0.0983	0.965	5	39	dendrite morphogenesis	BP	1	SIPA1L1,TNIK,VLDLR,NRP1,YWHAH	down_VSAP_EC
GO:0070885	0.0983	0.986	2	9	negative regulation of calcineurin-NFAT signaling	BP	1	FHL2,GSK3B	down_VSAP_EC
GO:0106057	0.0983	0.986	2	9	negative regulation of calcineurin-mediated	BP	1	FHL2,GSK3B	down_VSAP_EC
GO:0097195	0.0983	1	1	1	pilomotor reflex	BP	1	NPNT	up_ACOR_VSMC
GO:0061138	0.0983	0.996	1	73	morphogenesis of a branching epithelium	BP	1	EYA1	up_AFEM_VSMC
GO:0031346	0.0983	0.996	1	73	positive regulation of cell projection organization	BP	1	LIMK1	up_VCAV_VSMC
GO:0002514	0.0983	1	1	1	B cell tolerance induction	BP	1	TGFBR2	up_VSAP_EC
GO:0002649	0.0983	1	1	1	regulation of tolerance induction to self antigen	BP	1	TGFBR2	up_VSAP_EC
GO:0002651	0.0983	1	1	1	positive regulation of tolerance induction to self	BP	1	TGFBR2	up_VSAP_EC
GO:0002661	0.0983	1	1	1	regulation of B cell tolerance induction	BP	1	TGFBR2	up_VSAP_EC
GO:0002663	0.0983	1	1	1	positive regulation of B cell tolerance induction	BP	1	TGFBR2	up_VSAP_EC
GO:0010310	0.0983	1	1	1	regulation of hydrogen peroxide metabolic	BP	1	ZNF205	up_VSAP_EC
GO:0010726	0.0983	1	1	1	positive regulation of hydrogen peroxide	BP	1	ZNF205	up_VSAP_EC
GO:0010728	0.0983	1	1	1	regulation of hydrogen peroxide biosynthetic	BP	1	ZNF205	up_VSAP_EC
GO:0010729	0.0983	1	1	1	positive regulation of hydrogen peroxide	BP	1	ZNF205	up_VSAP_EC
GO:0030167	0.0983	1	1	1	proteoglycan catabolic process	BP	1	HPSE	up_VSAP_EC
GO:0030200	0.0983	1	1	1	heparan sulfate proteoglycan catabolic	BP	1	HPSE	up_VSAP_EC
GO:0042128	0.0983	1	1	1	nitrate assimilation	BP	1	SUOX	up_VSAP_EC

GO:0042986	0.0983	1	1	1	positive regulation of amyloid precursor protein biosynthetic process	BP	1	SOAT1	up_VSAP_EC
GO:0062042	0.0983	1	1	1	regulation of cardiac epithelial to mesenchymal	BP	1	TGFBR2	up_VSAP_EC
GO:0062043	0.0983	1	1	1	positive regulation of cardiac epithelial to mesenchymal transition	BP	1	TGFBR2	up_VSAP_EC
GO:1905005	0.0983	1	1	1	regulation of epithelial to mesenchymal transition involved in endocardial	BP	1	TGFBR2	up_VSAP_EC
GO:1905007	0.0983	1	1	1	positive regulation of epithelial to mesenchymal transition involved in	BP	1	TGFBR2	up_VSAP_EC
GO:1905317	0.0983	1	1	1	inferior endocardial cushion morphogenesis	BP	1	TGFBR2	up_VSAP_EC
GO:1990086	0.0983	1	1	1	lens fiber cell apoptotic process	BP	1	TGFBR2	up_VSAP_EC
GO:1990428	0.0983	1	1	1	miRNA transport	BP	1	TGFBR2	up_VSAP_EC
GO:2000563	0.0983	1	1	1	positive regulation of CD4-positive, alpha-beta T cell proliferation	BP	1	TGFBR2	up_VSAP_EC
GO:1901863	0.0983	0.995	1	25	positive regulation of muscle tissue	BP	1	SHOX2	up_VSAP_VSMC
GO:0051171	0.0984	0.953	11	1697	regulation of nitrogen compound metabolic	BP	1	HOXA11,HOXA10,HOXA9,PITX1,EMX2,SALL1,IRX5,PRR16,TLR2,CAV1,SHOX2	up_VSAP_VSMC
GO:0044342	0.0984	0.987	2	7	type B pancreatic cell proliferation	BP	1	BIRC5,ENSCAFG00000005299	down_ACOR_VSMC
GO:0034091	0.0984	0.988	2	6	regulation of maintenance of sister chromatid	BP	0.969	SMC5,ATRX	up_VCAV_EC
GO:0034182	0.0984	0.988	2	6	regulation of maintenance of mitotic sister chromatid	BP	0.969	SMC5,ATRX	up_VCAV_EC
GO:0055123	0.0984	0.995	1	40	digestive system development	BP	1	BARX1	up_VPOR_VSMC
GO:0003281	0.0984	0.995	1	30	ventricular septum development	BP	1	TBX5	up_VPUL_EC
GO:0001819	0.098439234	0.96033	6	120	positive regulation of cytokine production	BP	1	FZD5,NOD2,IL33,PTGER4,MAVS,AGPAT2	up_ACOR_EC
GO:1901033	0.0985	0.997	1	2	positive regulation of response to reactive	BP	1	PARK7	down_VCAV_EC
GO:0008625	0.0985	0.995	1	30	extrinsic apoptotic signaling pathway via death domain receptors	BP	1	ARHGEF2	up_VPUL_EC
GO:0021675	0.0985	0.995	1	29	nerve development	BP	1	HOXA3	up_VPUL_EC
GO:0033227	0.0985	1	1	1	dsRNA transport	BP	1	FLOT1	up_VSAP_EC
GO:1904924	0.0985	1	1	1	negative regulation of mitophagy in response to mitochondrial	BP	1	HTRA2	up_VSAP_EC
GO:0071612	0.0985	1	1	1	IP-10 production	BP	1	MAVS	up_VSAP_EC
GO:0071651	0.0985	1	1	1	positive regulation of chemokine (C-C motif)	BP	1	MAVS	up_VSAP_EC
GO:0071658	0.0985	1	1	1	regulation of IP-10 production	BP	1	MAVS	up_VSAP_EC
GO:0071660	0.0985	1	1	1	positive regulation of IP-10 production	BP	1	MAVS	up_VSAP_EC
GO:1902902	0.098578665	0.99622	1	4	negative regulation of autophagosome assembly	BP	1	CHMP4B	up_ACOR_EC
GO:0008406	0.0986	0.953	9	57	gonad development	BP	1	GATA4,PRDX4,ATRX,CITED2,SPINK2,ARID5B,DMC1,LHX9,WDR48	up_ACOR_VSMC
GO:0045137	0.0986	0.953	9	57	development of primary sexual characteristics	BP	1	GATA4,PRDX4,ATRX,CITED2,SPINK2,ARID5B,DMC1,LHX9,WDR48	up_ACOR_VSMC
GO:0006807	0.0986	1	3	3221	nitrogen compound metabolic process	BP	1	PLCL1,MOSPD1,USP12	down_VFEM_EC
GO:2000036	0.0986	0.978	3	12	regulation of stem cell population maintenance	BP	1	SAV1,KDM2B,LBH	up_VSAP_EC
GO:0046640	0.0986	0.987	2	6	regulation of alpha-beta T cell proliferation	BP	1	CD274,PTPN22	up_ACOR_VSMC
GO:0010595	0.0986	0.995	1	52	positive regulation of endothelial cell migration	BP	1	HDAC7	down_VPOR_EC
GO:0061302	0.0986	0.997	1	2	smooth muscle cell-matrix adhesion	BP	1	SERPINE1	down_VSAP_EC
GO:2000097	0.0986	0.997	1	2	regulation of smooth muscle cell-matrix	BP	1	SERPINE1	down_VSAP_EC
GO:2000098	0.0986	0.997	1	2	negative regulation of smooth muscle cell-matrix	BP	1	SERPINE1	down_VSAP_EC

GO:0006589	0.0986	1	1	1	octopamine biosynthetic process	BP	1	MOXD1	up_ACOR_VSMC
GO:0046333	0.0986	1	1	1	octopamine metabolic process	BP	1	MOXD1	up_ACOR_VSMC
GO:0051091	0.0986	0.996	1	87	positive regulation of DNA-binding transcription factor activity	BP	1	ESR1	up_AORT_VSMC
GO:0006957	0.0986	1	1	1	complement activation, alternative pathway	BP	1	CFD	up_VSAP_EC
GO:0007619	0.0986	1	1	1	courtship behavior	BP	1	THRB	up_VSAP_EC
GO:0007621	0.0986	1	1	1	negative regulation of female receptivity	BP	1	THRB	up_VSAP_EC
GO:0008050	0.0986	1	1	1	female courtship behavior	BP	1	THRB	up_VSAP_EC
GO:0031643	0.0986	1	1	1	positive regulation of myelination	BP	1	WASF3	up_VSAP_EC
GO:0033299	0.0986	1	1	1	secretion of lysosomal enzymes	BP	1	NAGPA	up_VSAP_EC
GO:0042480	0.0986	1	1	1	negative regulation of eye photoreceptor cell	BP	1	THRB	up_VSAP_EC
GO:0045924	0.0986	1	1	1	regulation of female receptivity	BP	1	THRB	up_VSAP_EC
GO:0060180	0.0986	1	1	1	female mating behavior	BP	1	THRB	up_VSAP_EC
GO:0070782	0.0986	1	1	1	phosphatidylserine exposure on apoptotic cell	BP	1	XKR8	up_VSAP_EC
GO:0097474	0.0986	1	1	1	retinal cone cell apoptotic process	BP	1	THRB	up_VSAP_EC
GO:0106064	0.0986	1	1	1	regulation of cobalamin metabolic process	BP	1	CLYBL	up_VSAP_EC
GO:1901401	0.0986	1	1	1	regulation of tetrapyrrole metabolic process	BP	1	CLYBL	up_VSAP_EC
GO:1902068	0.0986	1	1	1	regulation of sphingolipid mediated signaling	BP	1	PLPP3	up_VSAP_EC
GO:1903576	0.0986	1	1	1	response to L-arginine	BP	1	CASTOR1	up_VSAP_EC
GO:1903577	0.0986	1	1	1	cellular response to L-arginine	BP	1	CASTOR1	up_VSAP_EC
GO:1903837	0.0986	1	1	1	regulation of mRNA 3'-UTR binding	BP	1	CDK9	up_VSAP_EC
GO:1903839	0.0986	1	1	1	positive regulation of mRNA 3'-UTR binding	BP	1	CDK9	up_VSAP_EC
GO:2001226	0.0986	1	1	1	negative regulation of chloride transport	BP	1	GABRE	up_VSAP_EC
GO:0098629	0.0986	1	1	1	trans-Golgi network membrane organization	BP	1	AZIN2	up_VSAP_EC
GO:0071280	0.098663517	0.99621	1	4	cellular response to copper ion	BP	1	AQP1	up_ACOR_EC
GO:1901701	0.0987	0.932	30	271	cellular response to oxygen-containing compound	BP	0.969	CXCL8,CALCRL,TNFAIP3,PPARG,TNIP1,PDGFB,BAIAP2L1,PDE4D,MEF2C,RAPGEF1,TLR4,IRF8,YES1,MTOR,ABCA1,MAPK13,SMARCA4,NCK1,TIMELESS,GUCY1B1,RAPGEF2,CTNNA1,GCH1,BLM,RIPK1,PDGFD,HIF1A,NR4A1,ZEB1,ARHGEF2	up_VCAV_EC
GO:0051607	0.0987	0.953	9	58	defense response to virus	BP	1	MX1,TLR3,ISG15,F2RL1,BNIP3,PTPN22,PARP9,DDIT4,SERINC3	up_ACOR_VSMC
GO:0010804	0.0987	0.987	2	6	negative regulation of tumor necrosis factor-mediated signaling	BP	1	F2RL1,CCDC3	up_ACOR_VSMC
GO:0030166	0.0987	0.995	1	19	proteoglycan biosynthetic process	BP	1	CSGALNACT1	down_ACOR_EC
GO:0071470	0.0987	0.995	1	13	cellular response to osmotic stress	BP	1	ARHGEF2	up_VPOR_EC
GO:0003206	0.0987	0.995	1	51	cardiac chamber morphogenesis	BP	1	TBX5	up_VPUL_VSMC
GO:0006145	0.0987	1	1	1	purine nucleobase catabolic process	BP	1	GDA	up_VSAP_EC
GO:0006147	0.0987	1	1	1	guanine catabolic process	BP	1	GDA	up_VSAP_EC
GO:0006539	0.0987	1	1	1	glutamate catabolic process via 2-oxoglutarate	BP	1	ADHFE1	up_VSAP_EC
GO:0035426	0.0987	1	1	1	extracellular matrix-cell signaling	BP	1	NTN4	up_VSAP_EC
GO:0046098	0.0987	1	1	1	guanine metabolic process	BP	1	GDA	up_VSAP_EC
GO:0060040	0.0987	1	1	1	retinal bipolar neuron differentiation	BP	1	IRX5	up_VSAP_EC

GO:0060668	0.0987	1	1	1	regulation of branching involved in salivary gland morphogenesis by extracellular matrix-	BP	1	NTN4	up_VSAP_EC
GO:2001144	0.0987	1	1	1	regulation of phosphatidylinositol-3,4,5-trisphosphate 5-	BP	1	BAG4	up_VSAP_EC
GO:2001145	0.0987	1	1	1	negative regulation of phosphatidylinositol-3,4,5-trisphosphate 5-phosphatase activity	BP	1	BAG4	up_VSAP_EC
GO:0034769	0.0987	1	1	1	basement membrane disassembly	BP	1	CTSS	up_VSAP_EC
GO:0072008	0.098719431	0.99621	1	4	glomerular mesangial cell differentiation	BP	1	FOXC2	up_ACOR_EC
GO:0072144	0.098719431	0.99621	1	4	glomerular mesangial cell development	BP	1	FOXC2	up_ACOR_EC
GO:0060767	0.098797999	0.9962	1	4	epithelial cell proliferation involved in prostate gland development	BP	1	SOX9	up_ACOR_EC
GO:0048518	0.0988	0.964	7	1676	positive regulation of biological process	BP	1	FGF5,HOXA3,NKX2-5,BAMBI,ANKRD6,CYP26B1,C3AR1	up_VPOR_VSMC
GO:0002694	0.0988	0.984	2	126	regulation of leukocyte activation	BP	1	TOX,CYP26B1	up_VFEM_EC
GO:0003186	0.0988	0.997	1	2	tricuspid valve morphogenesis	BP	1	BMPR1A	down_VCAV_EC
GO:0002253	0.0988	0.996	1	92	activation of immune response	BP	1	ESR1	up_AORT_VSMC
GO:0061448	0.0988	0.996	1	89	connective tissue development	BP	1	WNT7A	up_AORT_VSMC
GO:1901861	0.0988	0.995	1	41	regulation of muscle tissue development	BP	1	SHOX2	up_VFEM_VSMC
GO:0097529	0.0988	0.995	1	52	myeloid leukocyte migration	BP	1	SLIT2	up_VPUL_VSMC
GO:0000053	0.0988	1	1	1	argininosuccinate metabolic process	BP	1	ASS1	up_VSAP_EC
GO:0000101	0.0988	1	1	1	sulfur amino acid transport	BP	1	CTNS	up_VSAP_EC
GO:0002174	0.0988	1	1	1	mammary stem cell proliferation	BP	1	LBH	up_VSAP_EC
GO:0002238	0.0988	1	1	1	response to molecule of fungal origin	BP	1	MYD88	up_VSAP_EC
GO:0002413	0.0988	1	1	1	tolerance induction to tumor cell	BP	1	CD274	up_VSAP_EC
GO:0002461	0.0988	1	1	1	tolerance induction dependent upon immune	BP	1	CD274	up_VSAP_EC
GO:0002465	0.0988	1	1	1	peripheral tolerance induction	BP	1	CD274	up_VSAP_EC
GO:0002652	0.0988	1	1	1	regulation of tolerance induction dependent upon immune response	BP	1	CD274	up_VSAP_EC
GO:0002654	0.0988	1	1	1	positive regulation of tolerance induction dependent upon immune	BP	1	CD274	up_VSAP_EC
GO:0002658	0.0988	1	1	1	regulation of peripheral tolerance induction	BP	1	CD274	up_VSAP_EC
GO:0002660	0.0988	1	1	1	positive regulation of peripheral tolerance	BP	1	CD274	up_VSAP_EC
GO:0002843	0.0988	1	1	1	regulation of tolerance induction to tumor cell	BP	1	CD274	up_VSAP_EC
GO:0002845	0.0988	1	1	1	positive regulation of tolerance induction to	BP	1	CD274	up_VSAP_EC
GO:0006041	0.0988	1	1	1	glucosamine metabolic process	BP	1	GNPDA1	up_VSAP_EC
GO:0006043	0.0988	1	1	1	glucosamine catabolic process	BP	1	GNPDA1	up_VSAP_EC
GO:0006102	0.0988	1	1	1	isocitrate metabolic process	BP	1	IDH1	up_VSAP_EC
GO:0006148	0.0988	1	1	1	inosine catabolic process	BP	1	PNP	up_VSAP_EC
GO:0006526	0.0988	1	1	1	arginine biosynthetic process	BP	1	ASS1	up_VSAP_EC
GO:0006542	0.0988	1	1	1	glutamine biosynthetic process	BP	1	GLUL	up_VSAP_EC
GO:0006738	0.0988	1	1	1	nicotinamide riboside catabolic process	BP	1	PNP	up_VSAP_EC
GO:0006982	0.0988	1	1	1	response to lipid hydroperoxide	BP	1	MGST1	up_VSAP_EC
GO:0007231	0.0988	1	1	1	osmosensory signaling pathway	BP	1	OSR1	up_VSAP_EC

GO:0007284	0.0988	1	1	1	spermatogonial cell division	BP	1	ZNF449	up_VSAP_EC
GO:0008614	0.0988	1	1	1	pyridoxine metabolic process	BP	1	PNPO	up_VSAP_EC
GO:0008615	0.0988	1	1	1	pyridoxine biosynthetic process	BP	1	PNPO	up_VSAP_EC
GO:0009110	0.0988	1	1	1	vitamin biosynthetic process	BP	1	PNPO	up_VSAP_EC
GO:0009682	0.0988	1	1	1	induced systemic resistance	BP	1	MYD88	up_VSAP_EC
GO:0015677	0.0988	1	1	1	copper ion import	BP	1	STEAP3	up_VSAP_EC
GO:0015811	0.0988	1	1	1	L-cystine transport	BP	1	CTNS	up_VSAP_EC
GO:0018166	0.0988	1	1	1	C-terminal protein-tyrosinylation	BP	1	TTL	up_VSAP_EC
GO:0018322	0.0988	1	1	1	protein tyrosinylation	BP	1	TTL	up_VSAP_EC
GO:0019402	0.0988	1	1	1	galactitol metabolic process	BP	1	GALK1	up_VSAP_EC
GO:0021796	0.0988	1	1	1	cerebral cortex regionalization	BP	1	EMX2	up_VSAP_EC
GO:0023016	0.0988	1	1	1	signal transduction by trans-phosphorylation	BP	1	OSR1	up_VSAP_EC
GO:0032747	0.0988	1	1	1	positive regulation of interleukin-23 production	BP	1	MYD88	up_VSAP_EC
GO:0034356	0.0988	1	1	1	NAD biosynthesis via nicotinamide riboside salvage pathway	BP	1	PNP	up_VSAP_EC
GO:0034395	0.0988	1	1	1	regulation of transcription from RNA polymerase II promoter in response to	BP	1	HMOX1	up_VSAP_EC
GO:0035801	0.0988	1	1	1	adrenal cortex development	BP	1	CITED2	up_VSAP_EC
GO:0035802	0.0988	1	1	1	adrenal cortex formation	BP	1	CITED2	up_VSAP_EC
GO:0036376	0.0988	1	1	1	sodium ion export across plasma membrane	BP	1	FXYD1	up_VSAP_EC
GO:0042078	0.0988	1	1	1	germ-line stem cell division	BP	1	ZBTB16	up_VSAP_EC
GO:0042364	0.0988	1	1	1	water-soluble vitamin biosynthetic process	BP	1	PNPO	up_VSAP_EC
GO:0042732	0.0988	1	1	1	D-xylose metabolic process	BP	1	DHDH	up_VSAP_EC
GO:0042819	0.0988	1	1	1	vitamin B6 biosynthetic process	BP	1	PNPO	up_VSAP_EC
GO:0042823	0.0988	1	1	1	pyridoxal phosphate biosynthetic process	BP	1	PNPO	up_VSAP_EC
GO:0042843	0.0988	1	1	1	D-xylose catabolic process	BP	1	DHDH	up_VSAP_EC
GO:0042904	0.0988	1	1	1	9-cis-retinoic acid biosynthetic process	BP	1	DHRS9	up_VSAP_EC
GO:0042905	0.0988	1	1	1	9-cis-retinoic acid metabolic process	BP	1	DHRS9	up_VSAP_EC
GO:0043251	0.0988	1	1	1	sodium-dependent organic anion transport	BP	1	SLC10A6	up_VSAP_EC
GO:0046495	0.0988	1	1	1	nicotinamide riboside metabolic process	BP	1	PNP	up_VSAP_EC
GO:0048133	0.0988	1	1	1	male germ-line stem cell asymmetric division	BP	1	ZBTB16	up_VSAP_EC
GO:0048250	0.0988	1	1	1	iron import into the mitochondrion	BP	1	SLC25A28	up_VSAP_EC
GO:0048743	0.0988	1	1	1	positive regulation of skeletal muscle fiber	BP	1	SHOX2	up_VSAP_EC
GO:0051852	0.0988	1	1	1	disruption by host of symbiont cells	BP	1	F2RL1	up_VSAP_EC
GO:0051873	0.0988	1	1	1	killing by host of symbiont cells	BP	1	F2RL1	up_VSAP_EC
GO:0051956	0.0988	1	1	1	negative regulation of amino acid transport	BP	1	OSR1	up_VSAP_EC
GO:0061615	0.0988	1	1	1	glycolytic process through fructose-6-phosphate	BP	1	GALK1	up_VSAP_EC
GO:0061620	0.0988	1	1	1	glycolytic process through glucose-6-phosphate	BP	1	GALK1	up_VSAP_EC
GO:0061622	0.0988	1	1	1	glycolytic process through glucose-1-phosphate	BP	1	GALK1	up_VSAP_EC
GO:0061623	0.0988	1	1	1	glycolytic process from galactose	BP	1	GALK1	up_VSAP_EC
GO:0070291	0.0988	1	1	1	N-acylethanolamine metabolic process	BP	1	NAPEPLD	up_VSAP_EC

GO:0070292	0.0988	1	1	1	N-acylphosphatidylethanola	BP	1	NAPEPLD	up_VSAP_EC
GO:0070637	0.0988	1	1	1	pyridine nucleoside metabolic process	BP	1	PNP	up_VSAP_EC
GO:0070638	0.0988	1	1	1	pyridine nucleoside catabolic process	BP	1	PNP	up_VSAP_EC
GO:0070682	0.0988	1	1	1	proteasome regulatory particle assembly	BP	1	PSMD9	up_VSAP_EC
GO:0070942	0.0988	1	1	1	neutrophil mediated cytotoxicity	BP	1	F2RL1	up_VSAP_EC
GO:0070943	0.0988	1	1	1	neutrophil mediated killing of symbiont cell	BP	1	F2RL1	up_VSAP_EC
GO:0070944	0.0988	1	1	1	neutrophil mediated killing of bacterium	BP	1	F2RL1	up_VSAP_EC
GO:0070945	0.0988	1	1	1	neutrophil mediated killing of gram-negative	BP	1	F2RL1	up_VSAP_EC
GO:0070948	0.0988	1	1	1	regulation of neutrophil mediated cytotoxicity	BP	1	F2RL1	up_VSAP_EC
GO:0070949	0.0988	1	1	1	regulation of neutrophil mediated killing of	BP	1	F2RL1	up_VSAP_EC
GO:0070950	0.0988	1	1	1	regulation of neutrophil mediated killing of	BP	1	F2RL1	up_VSAP_EC
GO:0070951	0.0988	1	1	1	regulation of neutrophil mediated killing of gram-	BP	1	F2RL1	up_VSAP_EC
GO:0070960	0.0988	1	1	1	positive regulation of neutrophil mediated	BP	1	F2RL1	up_VSAP_EC
GO:0070962	0.0988	1	1	1	positive regulation of neutrophil mediated	BP	1	F2RL1	up_VSAP_EC
GO:0070963	0.0988	1	1	1	positive regulation of neutrophil mediated killing of gram-negative	BP	1	F2RL1	up_VSAP_EC
GO:0071449	0.0988	1	1	1	cellular response to lipid hydroperoxide	BP	1	MGST1	up_VSAP_EC
GO:0071499	0.0988	1	1	1	cellular response to laminar fluid shear stress	BP	1	ASS1	up_VSAP_EC
GO:0072071	0.0988	1	1	1	kidney interstitial fibroblast differentiation	BP	1	OSR1	up_VSAP_EC
GO:0072141	0.0988	1	1	1	renal interstitial fibroblast development	BP	1	OSR1	up_VSAP_EC
GO:0072165	0.0988	1	1	1	anterior mesonephric tubule development	BP	1	OSR1	up_VSAP_EC
GO:0072166	0.0988	1	1	1	posterior mesonephric tubule development	BP	1	OSR1	up_VSAP_EC
GO:0072167	0.0988	1	1	1	specification of mesonephric tubule	BP	1	OSR1	up_VSAP_EC
GO:0072168	0.0988	1	1	1	specification of anterior mesonephric tubule	BP	1	OSR1	up_VSAP_EC
GO:0072169	0.0988	1	1	1	specification of posterior mesonephric tubule	BP	1	OSR1	up_VSAP_EC
GO:0072184	0.0988	1	1	1	renal vesicle progenitor cell differentiation	BP	1	OSR1	up_VSAP_EC
GO:0072185	0.0988	1	1	1	metanephric cap development	BP	1	OSR1	up_VSAP_EC
GO:0072186	0.0988	1	1	1	metanephric cap morphogenesis	BP	1	OSR1	up_VSAP_EC
GO:0072194	0.0988	1	1	1	kidney smooth muscle tissue development	BP	1	OSR1	up_VSAP_EC
GO:0072208	0.0988	1	1	1	metanephric smooth muscle tissue	BP	1	OSR1	up_VSAP_EC
GO:0072258	0.0988	1	1	1	metanephric interstitial fibroblast differentiation	BP	1	OSR1	up_VSAP_EC
GO:0072259	0.0988	1	1	1	metanephric interstitial fibroblast development	BP	1	OSR1	up_VSAP_EC
GO:0072566	0.0988	1	1	1	chemokine (C-X-C motif) ligand 1 production	BP	1	MYD88	up_VSAP_EC
GO:0086013	0.0988	1	1	1	membrane repolarization during cardiac muscle cell action potential	BP	1	KCNE3	up_VSAP_EC
GO:0090094	0.0988	1	1	1	metanephric cap mesenchymal cell proliferation involved in	BP	1	OSR1	up_VSAP_EC
GO:0090107	0.0988	1	1	1	regulation of high-density lipoprotein particle	BP	1	LCAT	up_VSAP_EC
GO:0090198	0.0988	1	1	1	negative regulation of chemokine secretion	BP	1	F2RL1	up_VSAP_EC
GO:0097029	0.0988	1	1	1	mature conventional dendritic cell	BP	1	F2RL1	up_VSAP_EC
GO:0097039	0.0988	1	1	1	protein linear polyubiquitination	BP	1	RBCK1	up_VSAP_EC

GO:0097403	0.0988	1	1	1	cellular response to raffinose	BP	1	ZFP36L1	up_VSAP_EC
GO:0097712	0.0988	1	1	1	vesicle targeting, trans-Golgi to periciliary membrane compartment	BP	1	CEP19	up_VSAP_EC
GO:0098728	0.0988	1	1	1	germline stem cell asymmetric division	BP	1	ZBTB16	up_VSAP_EC
GO:0098915	0.0988	1	1	1	membrane repolarization during ventricular cardiac muscle cell action	BP	1	KCNE3	up_VSAP_EC
GO:0106135	0.0988	1	1	1	negative regulation of cardiac muscle cell	BP	1	KCNE3	up_VSAP_EC
GO:1900135	0.0988	1	1	1	positive regulation of renin secretion into blood	BP	1	F2RL1	up_VSAP_EC
GO:1901492	0.0988	1	1	1	positive regulation of lymphangiogenesis	BP	1	CCBE1	up_VSAP_EC
GO:1901545	0.0988	1	1	1	response to raffinose	BP	1	ZFP36L1	up_VSAP_EC
GO:1902302	0.0988	1	1	1	regulation of potassium ion export	BP	1	KCNE3	up_VSAP_EC
GO:1902303	0.0988	1	1	1	negative regulation of potassium ion export	BP	1	KCNE3	up_VSAP_EC
GO:1903259	0.0988	1	1	1	exon-exon junction complex disassembly	BP	1	PYM1	up_VSAP_EC
GO:1903276	0.0988	1	1	1	regulation of sodium ion export across plasma	BP	1	FXYD1	up_VSAP_EC
GO:1903278	0.0988	1	1	1	positive regulation of sodium ion export across	BP	1	FXYD1	up_VSAP_EC
GO:1903674	0.0988	1	1	1	regulation of cap-dependent translational	BP	1	NCK1	up_VSAP_EC
GO:1903676	0.0988	1	1	1	positive regulation of cap-dependent translational	BP	1	NCK1	up_VSAP_EC
GO:1903764	0.0988	1	1	1	regulation of potassium ion export across plasma	BP	1	KCNE3	up_VSAP_EC
GO:1903765	0.0988	1	1	1	negative regulation of potassium ion export across plasma membrane	BP	1	KCNE3	up_VSAP_EC
GO:1903946	0.0988	1	1	1	negative regulation of ventricular cardiac muscle cell action potential	BP	1	KCNE3	up_VSAP_EC
GO:1904580	0.0988	1	1	1	regulation of intracellular mRNA localization	BP	1	ZFP36L1	up_VSAP_EC
GO:1904582	0.0988	1	1	1	positive regulation of intracellular mRNA	BP	1	ZFP36L1	up_VSAP_EC
GO:1904674	0.0988	1	1	1	positive regulation of somatic stem cell	BP	1	LBH	up_VSAP_EC
GO:1904675	0.0988	1	1	1	regulation of somatic stem cell division	BP	1	LBH	up_VSAP_EC
GO:1904677	0.0988	1	1	1	positive regulation of somatic stem cell division	BP	1	LBH	up_VSAP_EC
GO:1905024	0.0988	1	1	1	regulation of membrane repolarization during ventricular cardiac muscle cell action potential	BP	1	KCNE3	up_VSAP_EC
GO:1905025	0.0988	1	1	1	negative regulation of membrane repolarization during ventricular cardiac muscle cell action	BP	1	KCNE3	up_VSAP_EC
GO:1905031	0.0988	1	1	1	regulation of membrane repolarization during cardiac muscle cell action	BP	1	KCNE3	up_VSAP_EC
GO:1905032	0.0988	1	1	1	negative regulation of membrane repolarization during cardiac muscle cell	BP	1	KCNE3	up_VSAP_EC
GO:1905404	0.0988	1	1	1	positive regulation of activated CD8-positive, alpha-beta T cell	BP	1	CD274	up_VSAP_EC
GO:1905407	0.0988	1	1	1	regulation of creatine transmembrane transporter activity	BP	1	OSR1	up_VSAP_EC
GO:1905408	0.0988	1	1	1	negative regulation of creatine transmembrane transporter activity	BP	1	OSR1	up_VSAP_EC
GO:2000035	0.0988	1	1	1	regulation of stem cell division	BP	1	LBH	up_VSAP_EC
GO:2000101	0.0988	1	1	1	regulation of mammary stem cell proliferation	BP	1	LBH	up_VSAP_EC
GO:2000103	0.0988	1	1	1	positive regulation of mammary stem cell	BP	1	LBH	up_VSAP_EC

GO:2000338	0.0988	1	1	1	regulation of chemokine (C-X-C motif) ligand 1	BP	1	MYD88	up_VSAP_EC
GO:2000346	0.0988	1	1	1	negative regulation of hepatocyte proliferation	BP	1	LIMS2	up_VSAP_EC
GO:2000543	0.0988	1	1	1	positive regulation of gastrulation	BP	1	OSR1	up_VSAP_EC
GO:2001186	0.0988	1	1	1	negative regulation of CD8-positive, alpha-beta T cell activation	BP	1	CD274	up_VSAP_EC
GO:0048874	0.0988	1	1	1	host-mediated regulation of intestinal microbiota	BP	1	NAPEPLD	up_VSAP_EC
GO:0051024	0.0988	1	1	1	positive regulation of immunoglobulin secretion	BP	1	STX4	up_VSAP_EC
GO:0070961	0.0988	1	1	1	positive regulation of neutrophil mediated	BP	1	F2RL1	up_VSAP_EC
GO:0003097	0.098885674	0.99619	1	4	renal water transport	BP	1	AQP1	up_ACOR_EC
GO:0010562	0.0989	0.935	25	312	positive regulation of phosphorus metabolic process	BP	1	ARHGEF2,VEGFA,NR1H4,JCAD,BCAR3,PTPN11,ILK,PPP1R12A,PTGS2,TNIK,YES1,ADAM17,MAGED1,CTNNB1,LYN,MMD,VLDLR,SH3GLB1,CALM1,PAK2,INHBA,NRP1,SH3RF3,MADD,MID1	down_VSAP_EC
GO:0045937	0.0989	0.935	25	312	positive regulation of phosphate metabolic process	BP	1	ARHGEF2,VEGFA,NR1H4,JCAD,BCAR3,PTPN11,ILK,PPP1R12A,PTGS2,TNIK,YES1,ADAM17,MAGED1,CTNNB1,LYN,MMD,VLDLR,SH3GLB1,CALM1,PAK2,INHBA,NRP1,SH3RF3,MADD,MID1	down_VSAP_EC
GO:0010927	0.0989	0.97	4	28	cellular component assembly involved in	BP	1	ACTN1,WDR1,SIX4,MYBPC2	down_VSAP_EC
GO:0048870	0.0989	0.977	3	451	cell motility	BP	1	CHL1,BAMBI,C3AR1	up_VPOR_VSMC
GO:0051674	0.0989	0.977	3	451	localization of cell	BP	1	CHL1,BAMBI,C3AR1	up_VPOR_VSMC
GO:0045668	0.0989	0.986	2	11	negative regulation of osteoblast differentiation	BP	1	TWIST2,GREM1	down_VCAV_EC
GO:0080134	0.0989	0.986	2	386	regulation of response to stress	BP	1	TLR2,EYA1	up_AFEM_VSMC
GO:2000027	0.0989	0.995	1	72	regulation of animal organ morphogenesis	BP	1	GATA5	up_VCAV_VSMC
GO:0072001	0.099	0.964	5	104	renal system development	BP	1	IRX3,DLL1,GREM1,OVOL1,GCNT4	up_AFEM_EC
GO:0007596	0.099	0.976	3	47	blood coagulation	BP	1	F5,F3,TFPI2	up_AFEM_EC
GO:0007389	0.099	0.984	2	137	pattern specification process	BP	1	TBX5,HOXA3	up_APUL_EC
GO:0070493	0.099	0.987	2	6	thrombin-activated receptor signaling	BP	0.969	IQGAP2,STMN1	up_VCAV_EC
GO:2001234	0.099	0.995	1	75	negative regulation of apoptotic signaling	BP	1	EYA1	up_AFEM_VSMC
GO:0016125	0.099	0.995	1	41	sterol metabolic process	BP	1	CYP26B1	up_VPOR_VSMC
GO:0017015	0.099	0.995	1	40	regulation of transforming growth factor beta receptor signaling	BP	1	BAMBI	up_VPOR_VSMC
GO:1903844	0.099	0.995	1	40	regulation of cellular response to transforming growth factor beta	BP	1	BAMBI	up_VPOR_VSMC
GO:0009206	0.099	0.995	1	39	purine ribonucleoside triphosphate biosynthetic	BP	1	FBP1	up_VFEM_VSMC
GO:0010817	0.0991	0.944	14	102	regulation of hormone levels	BP	1	LEP,TM7SF3,ERO1B,PRKCE,DKKL1,SCG5,PCSK6,CHD7,CCL5,RAB11B,EDN1,PFKL,PARK7,STC2	up_ACOR_VSMC
GO:1901576	0.0991	0.95	13	1877	organic substance biosynthetic process	BP	1	ACVRL1,YEATS2,YBX3,HIVEP2,HOXA7,RPSA,SULF1,ELK3,HOXA1,CSGALNACT1,ACSL3,TNRC6B,NEDD4	down_ACOR_EC
GO:0007626	0.0991	0.955	8	53	locomotory behavior	BP	0.969	CHD7,HOXB8,TMOD1,NAV2,PREX2,SPTBN4,ALS2,ALDH1A3	up_VCAV_EC
GO:0033619	0.0991	0.971	4	19	membrane protein proteolysis	BP	1	PTPN3,DAG1,PSEN1,ERAP1	up_ACOR_VSMC
GO:0006936	0.0991	0.969	4	74	muscle contraction	BP	1	CHRM2,HTR1D,TBXA2R,DMPK	up_AFEM_EC
GO:0009913	0.0991	0.976	3	47	epidermal cell differentiation	BP	1	HOXA7,DLL1,FOSL2	up_AFEM_EC
GO:0002686	0.0991	0.978	3	12	negative regulation of leukocyte migration	BP	1	HOXA7,C5AR2,SLIT2	up_VSAP_EC
GO:0090230	0.0991	1	1	1	regulation of centromere complex assembly	BP	0.969	SENP6	up_VCAV_EC
GO:0090234	0.0991	1	1	1	regulation of kinetochore assembly	BP	0.969	SENP6	up_VCAV_EC

GO:0009566	0.0991	0.995	1	24	fertilization	BP	1	HOXA9	up_VFEM_EC
GO:0030048	0.0991	0.995	1	23	actin filament-based movement	BP	1	CAV1	up_VSAP_VSMC
GO:0048010	0.099100922	0.98456	2	20	vascular endothelial growth factor receptor	BP	1	FOXC2,NRP1	up_ACOR_EC
GO:0050832	0.09918758	0.99617	1	4	defense response to fungus	BP	1	RARRES2	up_ACOR_EC
GO:0016052	0.0992	0.962	6	34	carbohydrate catabolic process	BP	1	ENOSF1,PYGM,ALDOC,DDIT4,NEU3,GAA	up_ACOR_VSMC
GO:0042129	0.0992	0.97	4	35	regulation of T cell proliferation	BP	1	GPAM,PRNP,SDC4,RAC2	down_VCAV_EC
GO:0008625	0.0992	0.97	4	30	extrinsic apoptotic signaling pathway via death domain receptors	BP	1	ARHGEF2,SERPINE1,ATF3,MADD	down_VSAP_EC
GO:1990089	0.0992	0.987	2	6	response to nerve growth factor	BP	0.969	RAPGEF1,RAPGEF2	up_VCAV_EC
GO:1990090	0.0992	0.987	2	6	cellular response to nerve growth factor stimulus	BP	0.969	RAPGEF1,RAPGEF2	up_VCAV_EC
GO:0030334	0.0992	0.985	2	267	regulation of cell migration	BP	1	TBX5,SLIT2	up_VPUL_VSMC
GO:0009913	0.0992	0.995	1	47	epidermal cell differentiation	BP	1	SRSF6	down_AFEM_EC
GO:0007166	0.0992	1	1	686	cell surface receptor signaling pathway	BP	1	GATA5	down_AFEM_VSMC
GO:0055065	0.0992	0.996	1	145	metal ion homeostasis	BP	1	HOXA3	up_APUL_VSMC
GO:0022008	0.0993	0.944	14	406	neurogenesis	BP	1	IRX3,IRX5,HOXC10,LEP,DLL1,VXN,PLXNA4,NEFH,THRB,LGI4,CAMK2A,LRRN2,KANK1,TNC	up_AFEM_EC
GO:0051099	0.0993	0.952	9	70	positive regulation of binding	BP	0.969	TMEM173,MEF2C,GATA3,RALB,BMP2,STMN1,MFNG,RAPGEF2,HMGB1	up_VCAV_EC
GO:1900182	0.0993	0.962	6	33	positive regulation of protein localization to	BP	1	LEP,PTPN22,PARP9,PSEN1,JUP,PARK7	up_ACOR_VSMC
GO:0032387	0.0993	0.971	4	20	negative regulation of intracellular transport	BP	1	FERMT1,OS9,INPP5K,PARK7	up_ACOR_VSMC
GO:0003219	0.0993	1	1	1	cardiac right ventricle formation	BP	1	SMARCD3	up_ACOR_VSMC
GO:0014722	0.0993	1	1	1	regulation of skeletal muscle contraction by calcium ion signaling	BP	1	DMPK	up_ACOR_VSMC
GO:0014852	0.0993	1	1	1	regulation of skeletal muscle contraction by neural stimulation via	BP	1	DMPK	up_ACOR_VSMC
GO:0014853	0.0993	1	1	1	regulation of excitatory postsynaptic membrane potential involved in skeletal muscle	BP	1	DMPK	up_ACOR_VSMC
GO:0051823	0.0993	1	1	1	regulation of synapse structural plasticity	BP	1	DMPK	up_ACOR_VSMC
GO:0010544	0.099362248	0.99616	1	4	negative regulation of platelet activation	BP	1	PRKCD	up_ACOR_EC
GO:0060429	0.0994	0.97	4	341	epithelium development	BP	1	ACVRL1,PDPN,HOXA7,SULF1	down_ACOR_EC
GO:0001890	0.0994	0.995	1	51	placenta development	BP	1	CITED2	down_VPOR_EC
GO:0034454	0.0994	0.997	1	2	microtubule anchoring at centrosome	BP	1	HOOK3	down_VSAP_EC
GO:0045637	0.0994	0.995	1	73	regulation of myeloid cell differentiation	BP	1	LRRC17	up_VCAV_VSMC
GO:0022411	0.0995	0.946	12	130	cellular component disassembly	BP	1	ARHGEF2,PLK3,FSCN1,SMARCA4,WDR1,KIF5B,HIF1A,BNIP3,CDC37,HTT,CAPG,MID1	down_VSAP_EC
GO:0034504	0.0995	0.952	9	111	protein localization to nucleus	BP	1	ENSCAFG00000014978,SNUPN,TRIM8,GLI3,PARK7,ARL2BP,MAVS,SEC61B,GLIS2	down_VCAV_EC
GO:0033077	0.0995	0.971	4	22	T cell differentiation in thymus	BP	0.969	IL7R,GATA3,ITPKB,ZEB1	up_VCAV_EC
GO:0050858	0.0995	0.987	2	6	negative regulation of antigen receptor-mediated signaling	BP	1	ELF1,DUSP22	up_ACOR_VSMC
GO:0050860	0.0995	0.987	2	6	negative regulation of T cell receptor signaling	BP	1	ELF1,DUSP22	up_ACOR_VSMC
GO:0034754	0.0995	0.995	1	24	cellular hormone metabolic process	BP	1	CYP26B1	up_VFEM_EC
GO:0060420	0.0996	0.971	4	18	regulation of heart growth	BP	1	FGFR2,TBX20,TBX5,EDN1	up_ACOR_VSMC
GO:2000001	0.0996	0.978	3	13	regulation of DNA damage checkpoint	BP	1	WDR76,RNASEH2B,DDX39B	down_ACOR_VSMC
GO:0072358	0.0996	0.985	2	268	cardiovascular system development	BP	1	HOXA3,SLIT2	up_VPUL_VSMC

GO:1903510	0.0996	0.995	1	22	mucopolysaccharide metabolic process	BP	1	CSGALNACT1	down_ACOR_EC
GO:0042632	0.0996	0.995	1	27	cholesterol homeostasis	BP	1	NR1H4	up_APUL_EC
GO:0009855	0.0996	0.995	1	42	determination of bilateral symmetry	BP	1	NKX2-5	up_VPOR_VSMC
GO:1901655	0.099695033	0.98441	2	20	cellular response to ketone	BP	1	AQP1,PTGER4	up_ACOR_EC
GO:0030334	0.0997	0.931	32	267	regulation of cell migration	BP	1	HOXA7,TWIST2,WNT11,FAM107A,C5AR2,ENPP2,SLC8B1,SLIT2,CDH1,FGF7,ABL1,HDAC7,EMP2,FUZ,PDGFD,CCBE1,STAT5A,RECK,F2RL1,DDR2,PIK3R1,TIAM1,TBXA2R,SASH1,MYD88,ZNF580,STX4,ACVRL1,NCK1,HMOX1,TGFBR2,BAG4	up_VSAP_EC
GO:0050673	0.0997	0.946	12	135	epithelial cell proliferation	BP	1	VEGFA,SULF1,SPARC,JCAD,TGFBR1,ADAM17,MAGED1,CTNNB1,APOE,STRAP,NR4A1,SIX4	down_VSAP_EC
GO:0061351	0.0997	0.961	6	50	neural precursor cell proliferation	BP	1	ARHGEF2,HOOK3,ILK,HIF1A,CTNNB1,DISC1	down_VSAP_EC
GO:0010592	0.0997	0.987	2	7	positive regulation of lamellipodium assembly	BP	1	FSCN1,RAC1	down_ACOR_VSMC
GO:1903204	0.0997	0.986	2	9	negative regulation of oxidative stress-induced	BP	1	HIF1A,CTNNB1	down_VSAP_EC
GO:0003012	0.0997	0.996	1	90	muscle system process	BP	1	ARG2	up_AORT_VSMC
GO:1903725	0.0997	0.995	1	30	regulation of phospholipid metabolic process	BP	1	NR1H4	up_VPUL_EC
GO:0090257	0.0997	0.995	1	52	regulation of muscle system process	BP	1	GATA5	up_VPUL_VSMC
GO:1903409	0.0997	0.995	1	24	reactive oxygen species biosynthetic process	BP	1	CAV1	up_VSAP_VSMC
GO:0050905	0.0998	0.966	5	26	neuromuscular process	BP	1	GLRB,POMK,APP,CLN8,GAA	up_ACOR_VSMC
GO:0010880	0.0998	0.987	2	7	regulation of release of sequestered calcium ion into cytosol by	BP	0.969	PDE4D,CHD7	up_VCAV_EC
GO:0014808	0.0998	0.987	2	7	release of sequestered calcium ion into cytosol by sarcoplasmic reticulum	BP	0.969	PDE4D,CHD7	up_VCAV_EC
GO:1903514	0.0998	0.987	2	7	release of sequestered calcium ion into cytosol by endoplasmic reticulum	BP	0.969	PDE4D,CHD7	up_VCAV_EC
GO:0010615	0.0998	1	1	1	positive regulation of cardiac muscle adaptation	BP	0.969	PPP3CA	up_VCAV_EC
GO:0014744	0.0998	1	1	1	positive regulation of muscle adaptation	BP	0.969	PPP3CA	up_VCAV_EC
GO:1903244	0.0998	1	1	1	positive regulation of cardiac muscle hypertrophy in response	BP	0.969	PPP3CA	up_VCAV_EC
GO:1905203	0.0998	1	1	1	regulation of connective tissue replacement	BP	0.969	PPP3CA	up_VCAV_EC
GO:1905205	0.0998	1	1	1	positive regulation of connective tissue	BP	0.969	PPP3CA	up_VCAV_EC
GO:0048563	0.099850546	0.99612	1	4	post-embryonic animal organ morphogenesis	BP	1	EFEMP1	up_ACOR_EC
GO:0009725	0.0999	0.935	24	188	response to hormone	BP	1	ACTN2,LEP,LBH,SKP2,PRKAA2,BMP4,MED14,LATS2,RERG,AR,FAM210B,ADCY6,CTSB,CREBRF,PTGDR,SESN3,PRKCE,AGRN,NR3C1,FAM107A,DDIT4,GNAQ,INPP5K,PARK7	up_ACOR_VSMC
GO:0046626	0.0999	0.977	3	18	regulation of insulin receptor signaling	BP	1	NR1H4,ENPP1,PTPN2	down_VSAP_EC
GO:0071470	0.0999	0.978	3	13	cellular response to osmotic stress	BP	0.969	LRRC8D,PTGS2,ARHGEF2	up_VCAV_EC
GO:0006211	0.0999	1	1	1	5-methylcytosine catabolic process	BP	0.969	TET2	up_VCAV_EC
GO:0019857	0.0999	1	1	1	5-methylcytosine metabolic process	BP	0.969	TET2	up_VCAV_EC
GO:0043588	0.0999	0.995	1	74	skin development	BP	1	SLITRK5	up_VCAV_VSMC
GO:0002483	0.099918162	0.99611	1	4	antigen processing and presentation of endogenous peptide	BP	1	TAPBP	up_ACOR_EC
GO:0019885	0.099918162	0.99611	1	4	antigen processing and presentation of endogenous peptide	BP	1	TAPBP	up_ACOR_EC
GO:0051046	0.1	0.936	22	174	regulation of secretion	BP	1	EXPH5,TLR1,LEP,HTR2B,MBP,FBXL20,FERMT1,TM7SF3,CD274,ANKRD1,F2RL1,PTPN22,PRKCE,SCG5,CHD7,CCL5,EDN1,KCNC4,NOD2,SCAMP5,PREPL,PFKL	up_ACOR_VSMC

GO:0044271	0.1	0.952	11	1501	cellular nitrogen compound biosynthetic	BP	1	ACVRL1,YEATS2,YBX3,HIVEP2,HOXA7,RPSA,SULF1,ELK3,HOXA1,TNRC6B,NEDD4	down_ACOR_EC
GO:0065008	0.1	0.959	7	1056	regulation of biological quality	BP	1	NR1H4,HTR7,HOXA3,PLCL1,PCSK1N,SLITRK5,NTRK2	up_APUL_EC
GO:0007431	0.1	0.971	4	19	salivary gland development	BP	1	FGF7,CLCN2,NTN4,NFIB	up_VSAP_EC
GO:0001944	0.1	0.976	3	263	vasculature development	BP	1	EYA1,HOXA7,SIX1	up_VFEM_EC
GO:0006820	0.1	0.984	2	109	anion transport	BP	1	PDPN,ACSL3	down_ACOR_EC
GO:0015695	0.1	0.987	2	6	organic cation transport	BP	1	PSEN1,SLC25A20	up_ACOR_VSMC
GO:0034143	0.1	0.987	2	6	regulation of toll-like receptor 4 signaling	BP	1	F2RL1,PTPN22	up_ACOR_VSMC
GO:0095500	0.1	0.987	2	6	acetylcholine receptor signaling pathway	BP	1	AGRN,GNAQ	up_ACOR_VSMC
GO:1903831	0.1	0.987	2	6	signal transduction involved in cellular response to ammonium	BP	1	AGRN,GNAQ	up_ACOR_VSMC
GO:1905144	0.1	0.987	2	6	response to acetylcholine	BP	1	AGRN,GNAQ	up_ACOR_VSMC
GO:1905145	0.1	0.987	2	6	cellular response to acetylcholine	BP	1	AGRN,GNAQ	up_ACOR_VSMC
GO:0071417	0.1	0.984	2	142	cellular response to organonitrogen	BP	1	NR1H4,NTRK2	up_APUL_EC
GO:0048729	0.1	0.984	2	209	tissue morphogenesis	BP	1	NKX2-5,ANKRD6	up_VPOR_VSMC
GO:0010559	0.1	0.987	2	6	regulation of glycoprotein biosynthetic process	BP	1	NECAB3,SOAT1	up_VSAP_EC
GO:1903018	0.1	0.987	2	6	regulation of glycoprotein metabolic process	BP	1	NECAB3,SOAT1	up_VSAP_EC
GO:0051279	0.1	0.995	1	19	regulation of release of sequestered calcium ion into cytosol	BP	1	LYN	down_ACOR_EC
GO:1903707	0.1	0.995	1	48	negative regulation of hemopoiesis	BP	1	GABPA	down_AFEM_EC
GO:0031584	0.1	0.997	1	2	activation of phospholipase D activity	BP	1	ARF4	down_VSAP_EC
GO:0035602	0.1	1	1	1	fibroblast growth factor receptor signaling pathway involved in negative regulation of	BP	1	FGFR2	up_ACOR_VSMC
GO:0035603	0.1	1	1	1	fibroblast growth factor receptor signaling pathway involved in	BP	1	FGFR2	up_ACOR_VSMC
GO:0035604	0.1	1	1	1	fibroblast growth factor receptor signaling pathway involved in positive regulation of cell	BP	1	FGFR2	up_ACOR_VSMC
GO:0003100	0.1	1	1	1	regulation of systemic arterial blood pressure by	BP	1	EDN1	up_ACOR_VSMC
GO:0060529	0.1	1	1	1	squamous basal epithelial stem cell differentiation involved in prostate gland acinus development	BP	1	FGFR2	up_ACOR_VSMC
GO:0003298	0.1	1	1	1	physiological muscle hypertrophy	BP	1	EDN1	up_ACOR_VSMC
GO:0003301	0.1	1	1	1	physiological cardiac muscle hypertrophy	BP	1	EDN1	up_ACOR_VSMC
GO:0031583	0.1	1	1	1	phospholipase D-activating G protein-coupled receptor signaling	BP	1	EDN1	up_ACOR_VSMC
GO:0035812	0.1	1	1	1	renal sodium excretion	BP	1	EDN1	up_ACOR_VSMC
GO:0035813	0.1	1	1	1	regulation of renal sodium excretion	BP	1	EDN1	up_ACOR_VSMC
GO:0035815	0.1	1	1	1	positive regulation of renal sodium excretion	BP	1	EDN1	up_ACOR_VSMC
GO:0042313	0.1	1	1	1	protein kinase C deactivation	BP	1	EDN1	up_ACOR_VSMC
GO:0043179	0.1	1	1	1	rhythmic excitation	BP	1	EDN1	up_ACOR_VSMC
GO:0051771	0.1	1	1	1	negative regulation of nitric-oxide synthase	BP	1	EDN1	up_ACOR_VSMC
GO:0060024	0.1	1	1	1	rhythmic synaptic transmission	BP	1	EDN1	up_ACOR_VSMC
GO:0060584	0.1	1	1	1	regulation of prostaglandin-	BP	1	EDN1	up_ACOR_VSMC

GO:0060585	0.1	1	1	1	positive regulation of prostaglandin-endoperoxide synthase	BP	1	EDN1	up_ACOR_VSMC
GO:0061049	0.1	1	1	1	cell growth involved in cardiac muscle cell	BP	1	EDN1	up_ACOR_VSMC
GO:0061050	0.1	1	1	1	regulation of cell growth involved in cardiac muscle cell development	BP	1	EDN1	up_ACOR_VSMC
GO:0061051	0.1	1	1	1	positive regulation of cell growth involved in cardiac muscle cell development	BP	1	EDN1	up_ACOR_VSMC
GO:0002695	0.1	0.995	1	44	negative regulation of leukocyte activation	BP	1	BPI	up_AORT_EC
GO:0023061	0.1	0.995	1	95	signal release	BP	1	WNT7A	up_AORT_VSMC
GO:0002677	0.1	1	1	1	negative regulation of chronic inflammatory	BP	0.969	TNFAIP3	up_VCAV_EC
GO:0034146	0.1	1	1	1	toll-like receptor 5 signaling pathway	BP	0.969	TNFAIP3	up_VCAV_EC
GO:0034147	0.1	1	1	1	regulation of toll-like receptor 5 signaling	BP	0.969	TNFAIP3	up_VCAV_EC
GO:0034148	0.1	1	1	1	negative regulation of toll-like receptor 5 signaling	BP	0.969	TNFAIP3	up_VCAV_EC
GO:0070429	0.1	1	1	1	negative regulation of nucleotide-binding oligomerization domain	BP	0.969	TNFAIP3	up_VCAV_EC
GO:0072573	0.1	1	1	1	tolerance induction to lipopolysaccharide	BP	0.969	TNFAIP3	up_VCAV_EC
GO:2000348	0.1	1	1	1	regulation of CD40 signaling pathway	BP	0.969	TNFAIP3	up_VCAV_EC
GO:2000349	0.1	1	1	1	negative regulation of CD40 signaling pathway	BP	0.969	TNFAIP3	up_VCAV_EC
GO:0016485	0.1	0.995	1	73	protein processing	BP	1	C6	up_VCAV_VSMC
GO:0045910	0.1	0.995	1	14	negative regulation of DNA recombination	BP	1	CXHXorf57	up_VPOR_EC
GO:0072698	0.1	0.995	1	13	protein localization to microtubule cytoskeleton	BP	1	HOOK3	up_VPOR_EC
GO:1901890	0.1	0.995	1	13	positive regulation of cell junction assembly	BP	1	ENSCAFG00000002015	up_VPOR_EC
GO:0035051	0.1	0.995	1	41	cardiocyte differentiation	BP	1	NKX2-5	up_VPOR_VSMC
GO:1901863	0.1	0.995	1	25	positive regulation of muscle tissue	BP	1	CYP26B1	up_VFEM_EC
GO:0035850	0.1	0.995	1	25	epithelial cell differentiation involved in	BP	1	SALL1	up_VSAP_VSMC
GO:0051694	0.100146294	0.99609	1	4	pointed-end actin filament capping	BP	1	TMOD1	up_ACOR_EC
GO:0030916	0.100303419	0.99608	1	4	otic vesicle formation	BP	1	SOX9	up_ACOR_EC
GO:1905941	0.100401623	0.99607	1	4	positive regulation of gonad development	BP	1	SOX9	up_ACOR_EC
GO:2000018	0.100401623	0.99607	1	4	regulation of male gonad development	BP	1	SOX9	up_ACOR_EC
GO:2000020	0.100401623	0.99607	1	4	positive regulation of male gonad development	BP	1	SOX9	up_ACOR_EC
GO:0032077	0.100407462	0.99607	1	4	positive regulation of deoxyribonuclease	BP	1	PRKCD	up_ACOR_EC
GO:0006521	0.100507091	0.99606	1	4	regulation of cellular amino acid metabolic	BP	1	NR1H4	up_ACOR_EC
GO:0009445	0.100861993	0.99604	1	4	putrescine metabolic process	BP	1	AZIN2	up_ACOR_EC
GO:0010628	0.101	0.923	59	527	positive regulation of gene expression	BP	1	HOXA11,FZD1,PRR16,THRB,HOXA9,CDON,WNT11,AXIN2,OSR2,INSIG2,PTGFR,PPARGC1B,HOXD3,CDH1,SLC6A4,FGF7,DLL1,OSR1,MAP3K5,TENT5C,KLF15,ZBTB16,RARG,CCBE1,PYM1,F2RL1,ICE1,PIK3R1,MYC,SSBP2,ZEB1,SHOX2,ZC3H10,EEF2,LARP6,MYD88,IKZF2,TFE3,NUP98,NPAS2,PRKAB1,RPUSD4,CDK9,PRMT2,RAF1,TAF8,NFIB,ZNF580,ABHD14B,NCOA2,SMARCA2,EIF3D,ACVRL1,PPP3CB,NCK1,MAVS,CITED2,CXXC1,LBH	up_VSAP_EC
GO:0022008	0.101	0.932	26	406	neurogenesis	BP	1	LRP1,NAB2,NDN,SDC4,BMPR1A,LMO4,TRPV2,NUMBL,NAGLU,AKT2,CSPG4,SEMA3C,THY1,PEX5,TLX2,GLI3,NCDN,CDH2,GSDME,ENSCAFG00000017708,IRX5,UST,PRKCSH,THRB,RGMA,BRSK1	down_VCAV_EC

GO:0072359	0.101	0.933	24	368	circulatory system development	BP	1	NOTCH2,HSPB6,PDGFRB,DCN,ANGPT1,FBN1,BMPR1A,LOX,LMO4,RECK,ANPEP,SEMA3C,THY1,B4GALT1,GLI3,ZFP36L1,MYDGF,SASH1,IFT57,CDH2,SPRED1,SHC1,GREM1,CLIC4	down_VCAV_EC
GO:0019941	0.101	0.936	21	258	modification-dependent protein catabolic process	BP	1	PRICKLE1,PLK3,SOCS5,HSP90B1,TRIB3,USP12,DNAJC10,UBA6,UCHL5,SPOPL,DNAJB9,USP48,DDIT3,CBLC,GSK3B,CUL2,SPOP,DISC1,RAD23A,USP14,TNFAIP1	down_VSAP_EC
GO:0050896	0.101	0.948	16	2346	response to stimulus	BP	1	ACVRL1,LYN,IGF1R,PDPN,NRP2,HIVEP2,USP28,IPO7,ATRIP,SULF1,ELK3,HOXA1,WDR12,STX4,ASPN,NEDD4	down_ACOR_EC
GO:0006820	0.101	0.942	15	109	anion transport	BP	1	LEP,SLC26A7,TCAF1,ABCA1,CLCN3,SLC17A5,LRRC8C,SCP2,SLCO1A2,PSEN1,CLN8,RAB11B,PLIN2,CFI,SLC25A12	up_ACOR_VSMC
GO:0007162	0.101	0.949	10	66	negative regulation of cell adhesion	BP	1	MBP,BMP4,PDE5A,CD274,PTPN22,SPINT2,RND1,DUSP22,FAM107A,PAG1	up_ACOR_VSMC
GO:0001952	0.101	0.961	6	34	regulation of cell-matrix adhesion	BP	1	HOXA7,FAM107A,ABL1,EMP2,PIK3R1,ACVRL1	up_VSAP_EC
GO:1990138	0.101	0.961	6	35	neuron projection extension	BP	1	SLIT2,ABL1,PLXNA4,DPYSL2,TTL,ULK1	up_VSAP_EC
GO:0034249	0.101	0.964	5	41	negative regulation of cellular amide metabolic	BP	1	SESN2,EIF4EBP1,CNOT7,APOE,EIF2S1	down_VSAP_EC
GO:0060401	0.101	0.964	5	42	cytosolic calcium ion transport	BP	1	ERO1A,LYN,DDIT3,HTT,CALM1	down_VSAP_EC
GO:1903531	0.101	0.964	5	41	negative regulation of secretion by cell	BP	1	NR1H4,PTPN11,REST,RAB11FIP1,RHBDF2	down_VSAP_EC
GO:0045807	0.101	0.969	4	35	positive regulation of endocytosis	BP	1	LRP1,CLIP3,CYBA,GREM1	down_VCAV_EC
GO:0097006	0.101	0.971	4	19	regulation of plasma lipoprotein particle levels	BP	1	LIPC,LMF1,LCAT,SOAT1	up_VSAP_EC
GO:0006298	0.101	0.977	3	13	mismatch repair	BP	1	HMGB1,RPA1,PCNA	down_ACOR_VSMC
GO:0090184	0.101	0.977	3	14	positive regulation of kidney development	BP	1	GREM1,HOXB7,SIX4	down_ACOR_VSMC
GO:0007045	0.101	0.976	3	23	cell-substrate adherens junction assembly	BP	1	RHOD,THY1,GREM1	down_VCAV_EC
GO:0048041	0.101	0.976	3	23	focal adhesion assembly	BP	1	RHOD,THY1,GREM1	down_VCAV_EC
GO:0051899	0.101	0.976	3	22	membrane depolarization	BP	1	DCN,PARK7,TPC3	down_VCAV_EC
GO:0002063	0.101	0.976	3	18	chondrocyte development	BP	1	SULF1,SERPINH1,CHSY1	down_VSAP_EC
GO:0006836	0.101	0.975	3	47	neurotransmitter transport	BP	1	KCNC4,CHRM2,CAMK2A	up_AFEM_EC
GO:0030509	0.101	0.975	3	47	BMP signaling pathway	BP	1	TMEM100,GREM1,GREM2	up_AFEM_EC
GO:0009893	0.101	0.98	3	995	positive regulation of metabolic process	BP	1	HOXA9,WNT7A,ESR1	up_AORT_VSMC
GO:0030888	0.101	0.977	3	13	regulation of B cell proliferation	BP	0.974	MEF2C,TLR4,PELI1	up_VCAV_EC
GO:0048048	0.101	0.977	3	13	embryonic eye morphogenesis	BP	0.971	PROX1,ALDH1A3,ZEB1	up_VCAV_EC
GO:0046839	0.101	0.977	3	12	phospholipid dephosphorylation	BP	1	INPP5E,PLPP3,PLPP5	up_VSAP_EC
GO:0021846	0.101	0.985	2	11	cell proliferation in forebrain	BP	1	NUMBL,GLI3	down_VCAV_EC
GO:2001275	0.101	0.985	2	11	positive regulation of glucose import in response to insulin	BP	1	ENSCAFG00000014978,AKT2	down_VCAV_EC
GO:0048873	0.101	0.985	2	9	homeostasis of number of cells within a tissue	BP	1	PTPN11,EXOC5	down_VSAP_EC
GO:1905331	0.101	0.986	2	9	negative regulation of morphogenesis of an	BP	1	SULF1,CTNNB1	down_VSAP_EC
GO:0043267	0.101	0.987	2	6	negative regulation of potassium ion transport	BP	1	ACTN2,KCNE3	up_ACOR_VSMC
GO:0072201	0.101	0.987	2	6	negative regulation of mesenchymal cell	BP	1	BMP4,CLSTN1	up_ACOR_VSMC
GO:0043009	0.101	0.984	2	227	chordate embryonic development	BP	1	HOXA9,HOXC6	up_AORT_EC
GO:0007548	0.101	0.983	2	68	sex differentiation	BP	1	SEMA3A,ACVR2A	up_VPOR_EC
GO:0002683	0.101	0.984	2	126	negative regulation of immune system process	BP	1	HOXA9,HOXA7	up_VFEM_EC
GO:0017144	0.101	0.984	2	199	drug metabolic process	BP	1	GATM,FBP1	up_VFEM_VSMC
GO:0044283	0.101	0.984	2	201	small molecule biosynthetic process	BP	1	GATM,FBP1	up_VFEM_VSMC
GO:0001865	0.101	0.987	2	6	NK T cell differentiation	BP	1	ZBTB16,TGFBR2	up_VSAP_EC

GO:0030048	0.101	0.995	1	23	actin filament-based movement	BP	1	PDPN	down_ACOR_EC
GO:0043618	0.101	0.995	1	22	regulation of transcription from RNA polymerase II promoter in response to	BP	1	NEDD4	down_ACOR_EC
GO:0045921	0.101	0.995	1	24	positive regulation of exocytosis	BP	1	STX4	down_ACOR_EC
GO:0051225	0.101	0.995	1	49	spindle assembly	BP	1	GPSM2	down_AFEM_EC
GO:0036089	0.101	0.997	1	2	cleavage furrow formation	BP	1	SNX18	down_VCAV_EC
GO:0097176	0.101	0.997	1	2	epoxide metabolic process	BP	1	EPHX1	down_VCAV_EC
GO:1902548	0.101	0.997	1	2	negative regulation of cellular response to vascular endothelial	BP	1	DCN	down_VCAV_EC
GO:0016560	0.101	0.997	1	2	protein import into peroxisome matrix,	BP	1	PEX5	down_VCAV_EC
GO:0042439	0.101	0.997	1	2	ethanolamine-containing compound metabolic	BP	1	NAPEPLD	down_VCAV_EC
GO:1900747	0.101	0.997	1	2	negative regulation of vascular endothelial growth factor signaling	BP	1	DCN	down_VCAV_EC
GO:2000275	0.101	0.997	1	2	regulation of oxidative phosphorylation uncoupler activity	BP	1	PARK7	down_VCAV_EC
GO:0071962	0.101	0.997	1	2	mitotic sister chromatid cohesion, centromeric	BP	1	NAA10	down_VSAP_EC
GO:0060012	0.101	1	1	1	synaptic transmission, glycinergic	BP	1	GLRB	up_ACOR_VSMC
GO:0071873	0.101	1	1	1	response to norepinephrine	BP	1	APLP1	up_ACOR_VSMC
GO:0071874	0.101	1	1	1	cellular response to norepinephrine stimulus	BP	1	APLP1	up_ACOR_VSMC
GO:0097112	0.101	1	1	1	gamma-aminobutyric acid receptor clustering	BP	1	GLRB	up_ACOR_VSMC
GO:0038044	0.101	1	1	1	transforming growth factor-beta secretion	BP	1	FERMT1	up_ACOR_VSMC
GO:0051886	0.101	1	1	1	negative regulation of timing of anagen	BP	1	FERMT1	up_ACOR_VSMC
GO:1901860	0.101	1	1	1	positive regulation of mitochondrial DNA metabolic process	BP	1	PPARGC1A	up_ACOR_VSMC
GO:2001201	0.101	1	1	1	regulation of transforming growth factor-beta	BP	1	FERMT1	up_ACOR_VSMC
GO:2001203	0.101	1	1	1	positive regulation of transforming growth factor-beta secretion	BP	1	FERMT1	up_ACOR_VSMC
GO:0050727	0.101	0.995	1	74	regulation of inflammatory response	BP	1	TLR2	up_AFEM_VSMC
GO:0051146	0.101	0.995	1	76	striated muscle cell differentiation	BP	1	BEND2	up_AFEM_VSMC
GO:0046661	0.101	0.995	1	45	male sex differentiation	BP	1	HOXA9	up_AORT_EC
GO:0062013	0.101	0.995	1	27	positive regulation of small molecule metabolic	BP	1	NR1H4	up_APUL_EC
GO:0001707	0.101	0.995	1	25	mesoderm formation	BP	1	EYA1	up_VFEM_EC
GO:0051149	0.101	0.995	1	25	positive regulation of muscle cell differentiation	BP	1	CYP26B1	up_VFEM_EC
GO:0046620	0.101	0.995	1	30	regulation of organ growth	BP	1	TBX5	up_VPUL_EC
GO:0050728	0.101	0.995	1	31	negative regulation of inflammatory response	BP	1	NR1H4	up_VPUL_EC
GO:0090100	0.101	0.995	1	31	positive regulation of transmembrane receptor protein serine/threonine kinase signaling pathway	BP	1	NEO1	up_VPUL_EC
GO:0050920	0.101	0.995	1	53	regulation of chemotaxis	BP	1	SLIT2	up_VPUL_VSMC
GO:0051924	0.101	0.995	1	53	regulation of calcium ion transport	BP	1	HOXA3	up_VPUL_VSMC
GO:0001658	0.101	0.995	1	24	branching involved in ureteric bud	BP	1	HOXA11	up_VSAP_VSMC
GO:2001238	0.101	0.995	1	23	positive regulation of extrinsic apoptotic	BP	1	CAV1	up_VSAP_VSMC
GO:0051048	0.101082623	0.97494	3	42	negative regulation of secretion	BP	1	NR1H4,KCNB1,PTGER4	up_ACOR_EC
GO:0021795	0.101087924	0.98407	2	20	cerebral cortex cell migration	BP	1	DIXDC1,NRP1	up_ACOR_EC

GO:0016032	0.101457178	0.96289	5	94	viral process	BP	1	ATG16L2,TRIM8,MAVS,LRRC15,CHMP4B	up_ACOR_EC
GO:0071214	0.101509072	0.96287	5	93	cellular response to abiotic stimulus	BP	1	SOX9,AQP1,PTGER4,FIGNL1,MFAP4	up_ACOR_EC
GO:0104004	0.101509072	0.96287	5	93	cellular response to environmental stimulus	BP	1	SOX9,AQP1,PTGER4,FIGNL1,MFAP4	up_ACOR_EC
GO:0060485	0.101556423	0.96285	5	93	mesenchyme development	BP	1	GATA4,SOX9,FOXC2,TBX5,NRP1	up_ACOR_EC
GO:0060219	0.1016224	0.99598	1	4	camera-type eye photoreceptor cell	BP	1	SOX9	up_ACOR_EC
GO:0032502	0.102	0.913	175	1661	developmental process	BP	1	EXPH5,TNNT2,SH3TC2,PDLIM3,FGF13,VEGFD,TMEM176B,TMEM176A,SMAD6,ACTN2,LGR6,PLCG2,LEP,LBH,AFF3,GATA5,GATA4,CD83,CDC7,ANGPT1,EPYC,HTR2B,RASSF2,FNDC5,MBP,ZRANB1,PRDX4,RECK,SHROOM3,FEZ1,MLLT3,ITM2C,FERMT1,BMP4,LNPK,TBX18,FGFR2,TP53BP1,GJA1,ELF1,ATRX,MED14,TRPS1,TLR3,SEC24B,XIRP1,WASF3,PPARGC1A,LATS2,PDE5A,CITED2,TP53INP1,SPINT1,GLRB,ISG15,ACSL6,ZNRF3,CD274,ZNF365,SASH1,LMO4,TMEM108,FGF18,FAM210B,ARHGEF26,NDRG2,ATRN,P4HTM,NEBL,CDKN2B,EBF4,ZMYM2,ITPR1,PLAC8B,LIMS2,ANKRD1,PLEKHB2,F2RL1,CAMK2D,MARCH5,CTSB,BNIP3,EGLN1,CDKN1A,ENSCAFG00000006238,CLSTN1,LRRC8C,NRP1,PTPN22,GPM6A,CHD2,ULK2,DMPK,NPNT,UNC5B,SMARCD3,SPINT2,SLITRK5,PTGDR,TBX20,BBX,TPT1,TBX5,CCDC3,MARF1,FZD3,AFF4,DKKL1,FRS2,CPAMD8,RND1,DAG1,POMK,APP,LAMA3,PSEN1,SEMA6D,TGFBR1,PCSK6,SYNJ2BP,CHD7,JUP,SPINK2,SIPA1L1,DDR2,CLN8,WWC2,FOSL2,PERP,PSKH1,RTKN2,SIAH1,SSH3,C10H2orf49,EDN1,NID1,HPGD,ARID5B,ASAP1,DMC1,PRELP,SERPINB5,JAM3,B2M,DISC1,AP2B1,TNFRSF11B,TACC1,GAA,ATXN10,FEZ2,SUN1,INPP5K,CNOT4,LHX9,WFS1,	up_ACOR_VSMC
GO:0042326	0.102	0.942	14	163	negative regulation of phosphorylation	BP	1	SOCS5,ENPP1,ERRFI1,SNX25,TRIB3,DYNLL1,DNAJC10,PTPN2,APOE,LYN,STRAP,CBLC,ATF3,CDKN1A	down_VSAP_EC
GO:0051017	0.102	0.954	8	49	actin filament bundle assembly	BP	1	SYNPO2L,ARHGAP28,SYNPO2,NRP1,EVL,MYO1B,FAM107A,INPP5K	up_ACOR_VSMC
GO:0051480	0.102	0.954	8	50	regulation of cytosolic calcium ion concentration	BP	1	ADRA1B,PLCG2,HTR2B,ITPR1,F2RL1,PRKCE,CHD7,EDN1	up_ACOR_VSMC
GO:0006457	0.102	0.956	7	77	protein folding	BP	1	HSPB6,FKBP10,PDIA4,FKBP5,FKBP11,EMC6,CRTAP	down_VCAV_EC
GO:0006611	0.102	0.96	6	51	protein export from nucleus	BP	1	PTPN14,PTPN11,IWS1,SMG7,DDX39B,GSK3B	down_VSAP_EC
GO:0044706	0.102	0.965	5	27	multi-multicellular organism process	BP	1	RECK,CITED2,CTSB,DKKL1,HPGD	up_ACOR_VSMC
GO:0008544	0.102	0.968	4	75	epidermis development	BP	1	HOXA7,DLL1,OVOL1,FOSL2	up_AFEM_EC
GO:0045595	0.102	0.969	4	469	regulation of cell differentiation	BP	1	TBX5,WIF1,NPNT,NTRK2	up_APUL_EC
GO:0042136	0.102	0.97	4	21	neurotransmitter biosynthetic process	BP	0.976	CD34,TLR4,NOS3,NOS2	up_VCAV_EC
GO:0051129	0.102	0.968	4	221	negative regulation of cellular component	BP	1	ARHGEF2,SEMA3A,TWF1,RNF6	up_VPOR_EC
GO:0050793	0.102	0.97	4	731	regulation of developmental process	BP	1	EYA1,HOXA9,HOXA11,SHOX2	up_VFEM_VSMC
GO:0042307	0.102	0.976	3	23	positive regulation of protein import into	BP	1	ENSCAFG00000014978,GLI3,MAVS	down_VCAV_EC
GO:0046849	0.102	0.975	3	23	bone remodeling	BP	1	NOTCH2,NCDN,GREM1	down_VCAV_EC
GO:0061912	0.102	0.976	3	18	selective autophagy	BP	1	SESN2,CDC37,HTT	down_VSAP_EC
GO:0045071	0.102	0.977	3	12	negative regulation of viral genome replication	BP	1	MX1,ISG15,INPP5K	up_ACOR_VSMC
GO:0045598	0.102	0.975	3	47	regulation of fat cell differentiation	BP	1	CMKLR1,LEP,NAPEPLD	up_AFEM_EC
GO:0031146	0.102	0.977	3	12	SCF-dependent proteasomal ubiquitin-dependent protein	BP	1	FBXW4,FBXO4,FBXW5	up_VSAP_EC
GO:0048012	0.102	0.986	2	7	hepatocyte growth factor receptor signaling	BP	1	STMN1,RAC1	down_ACOR_VSMC
GO:0006110	0.102	0.985	2	9	regulation of glycolytic process	BP	1	PGAM1,HIF1A	down_VSAP_EC
GO:0010002	0.102	0.985	2	9	cardioblast differentiation	BP	1	PRICKLE1,REST	down_VSAP_EC
GO:0030811	0.102	0.985	2	9	regulation of nucleotide catabolic process	BP	1	PGAM1,HIF1A	down_VSAP_EC

GO:0042987	0.102	0.985	2	10	amyloid precursor protein catabolic process	BP	1	APOE,LYN	down_VSAP_EC
GO:0043470	0.102	0.985	2	9	regulation of carbohydrate catabolic	BP	1	PGAM1,HIF1A	down_VSAP_EC
GO:0003091	0.102	0.987	2	6	renal water homeostasis	BP	1	INPP5K,WFS1	up_ACOR_VSMC
GO:0006658	0.102	0.987	2	6	phosphatidylserine metabolic process	BP	1	SERINC3,SERINC1	up_ACOR_VSMC
GO:0006878	0.102	0.987	2	6	cellular copper ion homeostasis	BP	1	ATP7A,APP	up_ACOR_VSMC
GO:0071549	0.102	0.987	2	6	cellular response to dexamethasone stimulus	BP	1	NR3C1,DDIT4	up_ACOR_VSMC
GO:0098869	0.102	0.987	2	6	cellular oxidant detoxification	BP	1	SESN1,PARK7	up_ACOR_VSMC
GO:0021510	0.102	0.984	2	23	spinal cord development	BP	1	HOXC10,DLL1	up_AFEM_EC
GO:0021782	0.102	0.984	2	23	glial cell development	BP	1	DLL1,LGI4	up_AFEM_EC
GO:0034446	0.102	0.984	2	23	substrate adhesion-dependent cell spreading	BP	1	BVES,KANK1	up_AFEM_EC
GO:0046849	0.102	0.984	2	23	bone remodeling	BP	1	LEP,GREM1	up_AFEM_EC
GO:0045595	0.102	0.986	2	469	regulation of cell differentiation	BP	1	HOXA9,WNT7A	up_AORT_VSMC
GO:0035871	0.102	0.987	2	6	protein K11-linked deubiquitination	BP	0.978	TNFAIP3,USP37	up_VCAV_EC
GO:0031954	0.102	0.986	2	8	positive regulation of protein	BP	0.978	PDGFB,PDGFD	up_VCAV_EC
GO:0032530	0.102	0.987	2	6	regulation of microvillus organization	BP	0.976	PODXL,FSCN1	up_VCAV_EC
GO:0072574	0.102	0.987	2	6	hepatocyte proliferation	BP	1	LIMS2,SULF2	up_VSAP_EC
GO:0072575	0.102	0.987	2	6	epithelial cell proliferation involved in liver	BP	1	LIMS2,SULF2	up_VSAP_EC
GO:0072576	0.102	0.987	2	6	liver morphogenesis	BP	1	LIMS2,SULF2	up_VSAP_EC
GO:1900271	0.102	0.987	2	6	regulation of long-term synaptic potentiation	BP	1	FAM107A,ABL1	up_VSAP_EC
GO:0000271	0.102	0.995	1	22	polysaccharide biosynthetic process	BP	1	CSGALNACT1	down_ACOR_EC
GO:0007565	0.102	0.995	1	23	female pregnancy	BP	1	ACSL4	down_ACOR_EC
GO:0051017	0.102	0.995	1	49	actin filament bundle assembly	BP	1	FSCN1	down_AFEM_EC
GO:0006477	0.102	0.997	1	2	protein sulfation	BP	1	HS3ST3B1	down_VCAV_EC
GO:0009051	0.102	0.997	1	2	pentose-phosphate shunt, oxidative branch	BP	1	PGLS	down_VCAV_EC
GO:0046368	0.102	0.997	1	2	GDP-L-fucose metabolic process	BP	1	FUT8	down_VCAV_EC
GO:0001649	0.102	0.995	1	54	osteoblast differentiation	BP	1	HDAC7	down_VPOR_EC
GO:0045055	0.102	0.995	1	52	regulated exocytosis	BP	1	RAB11B	down_VPOR_EC
GO:0019835	0.102	0.997	1	2	cytolysis	BP	1	MMD	down_VSAP_EC
GO:0060158	0.102	0.997	1	2	phospholipase C-activating dopamine receptor signaling	BP	1	GNAQ	down_VSAP_EC
GO:0014004	0.102	1	1	1	microglia differentiation	BP	1	APP	up_ACOR_VSMC
GO:0014005	0.102	1	1	1	microglia development	BP	1	APP	up_ACOR_VSMC
GO:0048669	0.102	1	1	1	collateral sprouting in absence of injury	BP	1	APP	up_ACOR_VSMC
GO:0061890	0.102	1	1	1	positive regulation of astrocyte activation	BP	1	APP	up_ACOR_VSMC
GO:0150003	0.102	1	1	1	regulation of spontaneous synaptic transmission	BP	1	APP	up_ACOR_VSMC
GO:1904475	0.102	1	1	1	regulation of Ras GTPase binding	BP	1	CCPG1	up_ACOR_VSMC
GO:1990390	0.102	1	1	1	protein K33-linked ubiquitination	BP	1	KLHL20	up_ACOR_VSMC
GO:2001106	0.102	1	1	1	regulation of Rho guanyl-nucleotide exchange factor activity	BP	1	CCPG1	up_ACOR_VSMC
GO:0016199	0.102	1	1	1	axon midline choice point recognition	BP	1	APP	up_ACOR_VSMC
GO:0038161	0.102	1	1	1	prolactin signaling pathway	BP	1	CREBRF	up_ACOR_VSMC

GO:0043402	0.102	1	1	1	glucocorticoid mediated signaling pathway	BP	1	CREBRF	up_ACOR_VSMC
GO:0098814	0.102	1	1	1	spontaneous synaptic transmission	BP	1	APP	up_ACOR_VSMC
GO:1900169	0.102	1	1	1	regulation of glucocorticoid mediated	BP	1	CREBRF	up_ACOR_VSMC
GO:1900170	0.102	1	1	1	negative regulation of glucocorticoid mediated signaling pathway	BP	1	CREBRF	up_ACOR_VSMC
GO:1902211	0.102	1	1	1	regulation of prolactin signaling pathway	BP	1	CREBRF	up_ACOR_VSMC
GO:1902213	0.102	1	1	1	positive regulation of prolactin signaling	BP	1	CREBRF	up_ACOR_VSMC
GO:0006887	0.102	0.995	1	99	exocytosis	BP	1	WNT7A	up_AORT_VSMC
GO:0071824	0.102	0.995	1	92	protein-DNA complex subunit organization	BP	1	ESR1	up_AORT_VSMC
GO:0009410	0.102	0.995	1	27	response to xenobiotic stimulus	BP	1	SLITRK5	up_APUL_EC
GO:0033138	0.102	0.995	1	28	positive regulation of peptidyl-serine	BP	1	NTRK2	up_APUL_EC
GO:0043435	0.102	1	1	1	response to corticotropin-releasing hormone	BP	0.976	NR4A1	up_VCAV_EC
GO:0071376	0.102	1	1	1	cellular response to corticotropin-releasing	BP	0.976	NR4A1	up_VCAV_EC
GO:0050727	0.102	0.995	1	74	regulation of inflammatory response	BP	1	C6	up_VCAV_VSMC
GO:0051196	0.102	0.995	1	13	regulation of coenzyme metabolic process	BP	1	FBP1	up_VPOR_EC
GO:0009799	0.102	0.995	1	43	specification of symmetry	BP	1	NKX2-5	up_VPOR_VSMC
GO:0048704	0.102	0.995	1	46	embryonic skeletal system morphogenesis	BP	1	HOXA3	up_VPOR_VSMC
GO:0009145	0.102	0.995	1	40	purine nucleoside triphosphate biosynthetic	BP	1	FBP1	up_VFEM_VSMC
GO:0008277	0.102	0.995	1	31	regulation of G protein-coupled receptor signaling	BP	1	RGS1	up_VPUL_EC
GO:0006641	0.102	0.995	1	25	triglyceride metabolic process	BP	1	CAV1	up_VSAP_VSMC
GO:0008652	0.102	0.995	1	24	cellular amino acid biosynthetic process	BP	1	GATM	up_VSAP_VSMC
GO:0050806	0.102	0.995	1	25	positive regulation of synaptic transmission	BP	1	NETO1	up_VSAP_VSMC
GO:0051149	0.102	0.995	1	25	positive regulation of muscle cell differentiation	BP	1	SHOX2	up_VSAP_VSMC
GO:1901607	0.102	0.995	1	24	alpha-amino acid biosynthetic process	BP	1	GATM	up_VSAP_VSMC
GO:0032637	0.102	0.995	1	26	interleukin-8 production	BP	1	TLR2	up_VSAP_VSMC
GO:0032928	0.102027415	0.99594	1	4	regulation of superoxide anion generation	BP	1	PRKCD	up_ACOR_EC
GO:0010838	0.102152051	0.99593	1	4	positive regulation of keratinocyte proliferation	BP	1	CDH3	up_ACOR_EC
GO:0042493	0.102396081	0.95208	8	178	response to drug	BP	1	GATA4,SOX9,AQP1,AGRN,NOD2,PDE4A,GUCY1B1,PRKCD	up_ACOR_EC
GO:0046541	0.102398704	0.99591	1	4	saliva secretion	BP	1	AQP1	up_ACOR_EC
GO:0048207	0.102465629	0.99591	1	4	vesicle targeting, rough ER to cis-Golgi	BP	1	PREB	up_ACOR_EC
GO:0048208	0.102465629	0.99591	1	4	COPII vesicle coating	BP	1	PREB	up_ACOR_EC
GO:0044058	0.102580713	0.9959	1	4	regulation of digestive system process	BP	1	AQP1	up_ACOR_EC
GO:0072330	0.102584812	0.96757	4	68	monocarboxylic acid biosynthetic process	BP	1	NR1H4,THNSL2,PTGDS,ALDOC	up_ACOR_EC
GO:0016310	0.103	0.919	71	641	phosphorylation	BP	1	FZD1,CDON,WNT11,ACKR3,MAP3K14,ARHGEF5,AXIN2,C5AR2,ENPP2,PPARGC1B,TBC1D10C,CEP85,EEF2K,IL6R,SLIT2,FGF7,ABL1,EMP2,OSR1,MAP3K5,CDC42EP5,PDGFD,C1QTNF2,CNPPD1,COQ9,NDUFS1,ENSCAFG00000004260,PLPP3,CAMKK2,TNKS1BP1,AMBRA1,F2RL1,RBL2,PHKA2,MORC3,DDR2,ZFP36L1,TRAF7,FXN,GALK1,PIK3R1,MYC,PIK3IP1,RTN4RL1,SASH1,MYD88,NDUFS2,LRP4,PASK,STK16,FBXO7,LIMD1,TKFC,CDK9,FBLN1,ZFP36L2,RAF1,ZFYVE21,QARS,ACVRL1,FLOT1,PPP3CB,NCK1,NDUFV1,MAVS,ULK1,MAP2K2,TGFBR2,BAG4,DDIT4,LATS2	up_VSAP_EC

GO:0048584	0.103	0.92	66	592	positive regulation of response to stimulus	BP	1	ADRA1B,VEGFD,TLR1,LGR6,PLCG2,LEP,MID1,GATA5,HTR2B,RASSF2,ZRANB1,C28H10orf71,SKP2,NACC2,COCH,MLLT3,ITM2C,FERMT1,BMP4,FGFR2,TP53BP1,ELF1,TLR3,SH3RF2,PDE5A,CITED2,TP53INP1,CD274,SASH1,TMEM108,FGF18,CDKN2B,LIMS2,ANKRD1,F2RL1,ENPP4,BNIP3,C1S,CRLF3,CREBRF,NRP1,PTPN22,NPNT,UNC5B,C7,PRKCE,FRS2,PARP9,APP,PSEN1,CCL5,DUSP22,SIAH1,EDN1,NOD2,B2M,DISC1,SH3RF3,SERINC3,HERPUD1,MAP3K7,ENSCAFG00000029553,PARK7,IL18R1,RREB1,CRKL	up_ACOR_VSMC
GO:0050790	0.103	0.921	60	532	regulation of catalytic activity	BP	1	TNNT2,LEP,MAP3K8,HTR2B,RASSF2,USP6NL,MBP,RECK,BMP4,EVI5L,FGFR2,EIF4A2,SCARB2,SH3RF2,LATS2,PDE5A,PKP4,SASH1,LMO4,FGF18,CHN2,CDKN2B,CST3,F2RL1,DAPK1,CTSB,CDKN1A,NPNT,ATP7A,SMPD1,SH3RF1,CCNT1,MARF1,FRS2,PPP1R26,APP,PSEN1,SGSM2,SPINK2,CCL5,DDR2,PERP,DUSP22,EDN1,DYNC1I2,DUSP26,LRRC4,PRKAG3,DCUN1D4,SBF2,MAP3K7,INPP5K,SH3BP4,DYNC1I1,PARK7,GPATCH2,ZFYVE1,HMBOX1,BVES,LMF1	up_ACOR_VSMC
GO:0006793	0.103	0.922	56	948	phosphorus metabolic process	BP	1	GPAM,NOTCH2,PDGFRB,KIRREL1,SMAD9,DCN,PRNP,ADCY6,GLIPR2,BGN,BMPR1A,LMO4,CLIP3,ENSCAFG00000014978,AKT1S1,AKT2,LCAT,EEF2K,H6PD,PLCL1,CSPG4,NT5C2,ENTPD3,THNSL2,PGLS,ATP5MC1,GFPT2,UQCR10,ASPN,PIGS,THY1,ADK,PARK7,MAST2,ZFP36L1,MYDGF,SASH1,ARL2BP,AMPD2,MAVS,CDH2,GSDME,CAMK2D,FUT8,SPRED1,ACSS2,MAPRE3,CDKL2,DDR2,SHC1,FBH1,GREM1,NAPEPLD,ACAT1,BRSK1,CDK14	down_VCAV_EC
GO:0048598	0.103	0.932	26	207	embryonic morphogenesis	BP	1	AFF3,HTR2B,MBP,RECK,SHROOM3,BMP4,LNPK,FGFR2,GJA1,SEC24B,LATS2,CITED2,SPINT1,LMO4,ENSCAFG00000006238,SPINT2,TBX20,TBX5,FZD3,FRS2,DAG1,LAMA3,PSEN1,CHD7,C10H2orf49,EDN1	up_ACOR_VSMC
GO:0090407	0.103	0.936	19	177	organophosphate biosynthetic process	BP	0.978	NPR1,ADGRF5,PANK3,PDGFB,GK5,ADSS,GUCY1A1,SPTLC2,SAMD8,ITPKB,SH3GLB1,ELOVL7,ENSCAFG00000024323,GUCY1B1,PLCG2,NOS2,IMPA1,PIP5K1B,HIF1A	up_VCAV_EC
GO:0090596	0.103	0.946	11	84	sensory organ morphogenesis	BP	0.978	EPHB1,CHD7,GATA3,EDN1,PROX1,TSPAN12,JAG1,HIF1A,ALDH1A3,ZEB1,CDON	up_VCAV_EC
GO:0030100	0.103	0.948	10	70	regulation of endocytosis	BP	1	DNAJC6,PLCG2,ATXN2,F2RL1,LRPAP1,SYNJ2BP,GTF2A1L,B2M,SCAMP5,RAB5C	up_ACOR_VSMC
GO:0010959	0.103	0.955	7	79	regulation of metal ion transport	BP	1	PRNP,FXYD1,TRPV2,CACNB3,THY1,CYBA,PACSIN3	down_VCAV_EC
GO:1990823	0.103	0.959	6	39	response to leukemia inhibitory factor	BP	1	SLC25A5,B3GNT2,TFRC,NCL,CTH,MSC	down_ACOR_VSMC
GO:1990830	0.103	0.959	6	39	cellular response to leukemia inhibitory factor	BP	1	SLC25A5,B3GNT2,TFRC,NCL,CTH,MSC	down_ACOR_VSMC
GO:0050792	0.103	0.959	6	55	regulation of viral process	BP	1	P4HB,SMARCA4,CNOT7,APOE,REST,RAD23A	down_VSAP_EC
GO:0043269	0.103	0.958	6	135	regulation of ion transport	BP	1	KCNC4,KCND1,CAMK2A,FXYD1,DMPK,CLIC4	up_AFEM_EC
GO:0043624	0.103	0.96	6	39	cellular protein complex disassembly	BP	0.978	TMOD1,STMN1,SWAP70,TAOK1,TMOD3,ARHGEF2	up_VCAV_EC
GO:0015031	0.103	0.964	5	510	protein transport	BP	1	LYN,IPO7,ACSL3,STX4,NEDD4	down_ACOR_EC
GO:1901652	0.103	0.962	5	105	response to peptide	BP	1	LEP,CRY2,AVPR1A,CAMK2A,KANK1	up_AFEM_EC
GO:1901568	0.103	0.965	5	26	fatty acid derivative metabolic process	BP	1	PTGES,DAGLB,ABHD16A,HPGD,ENSCAFG00000023820	up_VSAP_EC
GO:0034097	0.103	0.975	3	238	response to cytokine	BP	1	NRP2,STX4,ASPN	down_ACOR_EC
GO:0035987	0.103	0.976	3	17	endodermal cell differentiation	BP	1	CTNNB1,INHBA,COL12A1	down_VSAP_EC
GO:0051893	0.103	0.975	3	20	regulation of focal adhesion assembly	BP	1	VEGFA,ENSCAFG00000002015,NRP1	down_VSAP_EC
GO:0090109	0.103	0.975	3	20	regulation of cell-substrate junction	BP	1	VEGFA,ENSCAFG00000002015,NRP1	down_VSAP_EC
GO:0050771	0.103	0.977	3	11	negative regulation of axonogenesis	BP	1	NRP1,ULK2,PSEN1	up_ACOR_VSMC
GO:0032418	0.103	0.976	3	17	lysosome localization	BP	0.978	KIT,SNAP23,FES	up_VCAV_EC
GO:0032613	0.103	0.977	3	14	interleukin-10 production	BP	0.978	CD34,TLR4,HMGB1	up_VCAV_EC
GO:0051646	0.103	0.977	3	12	mitochondrion localization	BP	0.978	NECTIN2,HIF1A,ATP2A1	up_VCAV_EC

GO:2000001	0.103	0.977	3	13	regulation of DNA damage checkpoint	BP	0.978	THOC1,ETAA1,CUL4A	up_VCAV_EC
GO:0030182	0.103	0.975	3	327	neuron differentiation	BP	1	FLRT3,ARHGEF2,NEO1	up_VPUL_EC
GO:0021952	0.103	0.977	3	13	central nervous system projection neuron	BP	1	SLIT2,PLXNA4,NFIB	up_VSAP_EC
GO:1901379	0.103	0.977	3	12	regulation of potassium ion transmembrane	BP	1	AMIGO1,KCNE3,KLHL24	up_VSAP_EC
GO:0050678	0.103	0.983	2	117	regulation of epithelial cell proliferation	BP	1	ACVRL1,SULF1	down_ACOR_EC
GO:0051222	0.103	0.983	2	103	positive regulation of protein transport	BP	1	ACSL3,STX4	down_ACOR_EC
GO:0072172	0.103	0.986	2	7	mesonephric tubule formation	BP	1	GREM1,SIX4	down_ACOR_VSMC
GO:0050691	0.103	0.985	2	11	regulation of defense response to virus by host	BP	1	RNF216,MAVS	down_VCAV_EC
GO:0031343	0.103	0.985	2	10	positive regulation of cell killing	BP	1	FAM49B,PVR	down_VSAP_EC
GO:2001258	0.103	0.985	2	11	negative regulation of cation channel activity	BP	1	REM1,CALM1	down_VSAP_EC
GO:0048585	0.103	0.986	2	475	negative regulation of response to stimulus	BP	1	ARG2,ESR1	up_AORT_VSMC
GO:0051049	0.103	0.986	2	479	regulation of transport	BP	1	WNT7A,ARG2	up_AORT_VSMC
GO:0050829	0.103	0.985	2	8	defense response to Gram-negative bacterium	BP	0.979	LYZ,TLR4	up_VCAV_EC
GO:0007276	0.103	0.983	2	130	gamete generation	BP	1	HOXA9,CYP26B1	up_VFEM_EC
GO:0009065	0.103	0.987	2	6	glutamine family amino acid catabolic process	BP	1	GOT1,ADHFE1	up_VSAP_EC
GO:0001776	0.103	0.995	1	23	leukocyte homeostasis	BP	1	LYN	down_ACOR_EC
GO:0008033	0.103	0.995	1	52	tRNA processing	BP	1	TSEN15	down_AFEM_EC
GO:0016579	0.103	0.995	1	49	protein deubiquitination	BP	1	USP33	down_AFEM_EC
GO:0009265	0.103	0.997	1	2	2'-deoxyribonucleotide biosynthetic process	BP	1	ADK	down_VCAV_EC
GO:0018171	0.103	0.997	1	2	peptidyl-cysteine oxidation	BP	1	PARK7	down_VCAV_EC
GO:0019249	0.103	0.997	1	2	lactate biosynthetic process	BP	1	PARK7	down_VCAV_EC
GO:0032382	0.103	0.997	1	2	positive regulation of intracellular sterol	BP	1	SCP2	down_VCAV_EC
GO:0032385	0.103	0.997	1	2	positive regulation of intracellular cholesterol	BP	1	SCP2	down_VCAV_EC
GO:0046356	0.103	0.997	1	2	acetyl-CoA catabolic process	BP	1	ACAT1	down_VCAV_EC
GO:0060054	0.103	0.997	1	2	positive regulation of epithelial cell proliferation involved in wound healing	BP	1	B4GALT1	down_VCAV_EC
GO:0003343	0.103	0.997	1	2	septum transversum development	BP	1	ZFP36L1	down_VCAV_EC
GO:0070269	0.103	0.997	1	2	pyroptosis	BP	1	GSDME	down_VCAV_EC
GO:1901029	0.103	0.997	1	2	negative regulation of mitochondrial outer membrane permeabilization involved	BP	1	SLC35F6	down_VCAV_EC
GO:1902224	0.103	0.997	1	2	ketone body metabolic process	BP	1	ACAT1	down_VCAV_EC
GO:1902339	0.103	0.997	1	2	positive regulation of apoptotic process involved in	BP	1	B4GALT1	down_VCAV_EC
GO:1902951	0.103	0.997	1	2	negative regulation of dendritic spine	BP	1	PRNP	down_VCAV_EC
GO:1904747	0.103	0.997	1	2	positive regulation of apoptotic process	BP	1	B4GALT1	down_VCAV_EC
GO:1905664	0.103	0.997	1	2	regulation of calcium ion import across plasma	BP	1	PRNP	down_VCAV_EC
GO:2000298	0.103	0.997	1	2	regulation of Rho-dependent protein serine/threonine kinase	BP	1	THY1	down_VCAV_EC
GO:2000823	0.103	0.997	1	2	regulation of androgen receptor activity	BP	1	PARK7	down_VCAV_EC
GO:2000825	0.103	0.997	1	2	positive regulation of androgen receptor	BP	1	PARK7	down_VCAV_EC
GO:0032379	0.103	0.997	1	2	positive regulation of intracellular lipid	BP	1	SCP2	down_VCAV_EC

GO:0042126	0.103	0.997	1	2	nitrate metabolic process	BP	1	SUOX	down_VCAV_EC
GO:0045764	0.103	0.997	1	2	positive regulation of cellular amino acid	BP	1	PARK7	down_VCAV_EC
GO:0046385	0.103	0.997	1	2	deoxyribose phosphate biosynthetic process	BP	1	ADK	down_VCAV_EC
GO:0046459	0.103	0.997	1	2	short-chain fatty acid metabolic process	BP	1	THNSL2	down_VCAV_EC
GO:0046950	0.103	0.997	1	2	cellular ketone body metabolic process	BP	1	ACAT1	down_VCAV_EC
GO:0048681	0.103	0.997	1	2	negative regulation of axon regeneration	BP	1	RGMA	down_VCAV_EC
GO:0051790	0.103	0.997	1	2	short-chain fatty acid biosynthetic process	BP	1	THNSL2	down_VCAV_EC
GO:0060994	0.103	0.997	1	2	regulation of transcription from RNA polymerase II promoter involved in	BP	1	GLIS2	down_VCAV_EC
GO:0003342	0.103	0.997	1	2	proepicardium development	BP	1	ZFP36L1	down_VCAV_EC
GO:0086036	0.103	0.997	1	2	regulation of cardiac muscle cell membrane	BP	1	FXYD1	down_VCAV_EC
GO:0090155	0.103	0.997	1	2	negative regulation of sphingolipid biosynthetic	BP	1	ORMDL3	down_VCAV_EC
GO:0090156	0.103	0.997	1	2	cellular sphingolipid homeostasis	BP	1	ORMDL3	down_VCAV_EC
GO:1900060	0.103	0.997	1	2	negative regulation of ceramide biosynthetic	BP	1	ORMDL3	down_VCAV_EC
GO:1990442	0.103	0.997	1	2	intrinsic apoptotic signaling pathway in response to nitrosative	BP	1	PARK7	down_VCAV_EC
GO:0034157	0.103	1	1	1	positive regulation of toll-like receptor 7 signaling	BP	1	PTPN22	up_ACOR_VSMC
GO:0071613	0.103	1	1	1	granzyme B production	BP	1	PTPN22	up_ACOR_VSMC
GO:0071661	0.103	1	1	1	regulation of granzyme B production	BP	1	PTPN22	up_ACOR_VSMC
GO:0071663	0.103	1	1	1	positive regulation of granzyme B production	BP	1	PTPN22	up_ACOR_VSMC
GO:2000566	0.103	1	1	1	positive regulation of CD8-positive, alpha-beta T cell proliferation	BP	1	PTPN22	up_ACOR_VSMC
GO:2001187	0.103	1	1	1	positive regulation of CD8-positive, alpha-beta T cell activation	BP	1	PTPN22	up_ACOR_VSMC
GO:0034155	0.103	1	1	1	regulation of toll-like receptor 7 signaling	BP	1	PTPN22	up_ACOR_VSMC
GO:0035644	0.103	1	1	1	phosphoanandamide dephosphorylation	BP	1	PTPN22	up_ACOR_VSMC
GO:0046330	0.103	0.995	1	47	positive regulation of JNK cascade	BP	1	TAOK1	up_AORT_EC
GO:0007283	0.103	0.995	1	91	spermatogenesis	BP	1	HOXA9	up_AORT_VSMC
GO:0007043	0.103	0.995	1	28	cell-cell junction assembly	BP	1	NR1H4	up_APUL_EC
GO:0055092	0.103	0.995	1	28	sterol homeostasis	BP	1	NR1H4	up_APUL_EC
GO:0006968	0.103	0.997	1	2	cellular defense response	BP	0.978	BCL10	up_VCAV_EC
GO:0002069	0.103	1	1	1	columnar/cuboidal epithelial cell maturation	BP	0.978	HIF1A	up_VCAV_EC
GO:0002248	0.103	1	1	1	connective tissue replacement involved in inflammatory response	BP	0.978	HIF1A	up_VCAV_EC
GO:0051541	0.103	1	1	1	elastin metabolic process	BP	0.978	HIF1A	up_VCAV_EC
GO:0060574	0.103	1	1	1	intestinal epithelial cell maturation	BP	0.978	HIF1A	up_VCAV_EC
GO:0061030	0.103	1	1	1	epithelial cell differentiation involved in mammary gland alveolus	BP	0.978	HIF1A	up_VCAV_EC
GO:2001253	0.103	1	1	1	regulation of histone H3-K36 trimethylation	BP	0.978	IWS1	up_VCAV_EC
GO:0045648	0.103	0.995	1	13	positive regulation of erythrocyte	BP	1	ACVR2A	up_VPOR_EC
GO:0070266	0.103	0.995	1	14	necroptotic process	BP	1	ARHGEF2	up_VPOR_EC
GO:0071675	0.103	0.995	1	13	regulation of mononuclear cell	BP	1	C3AR1	up_VPOR_EC
GO:0007605	0.103	0.995	1	27	sensory perception of sound	BP	1	SIX1	up_VFEM_EC

GO:0048332	0.103	0.995	1	26	mesoderm morphogenesis	BP	1	EYA1	up_VFEM_EC
GO:0009201	0.103	0.995	1	41	ribonucleoside triphosphate biosynthetic	BP	1	FBP1	up_VFEM_VSMC
GO:0071241	0.103	0.995	1	42	cellular response to inorganic substance	BP	1	FBP1	up_VFEM_VSMC
GO:2001022	0.103	0.995	1	42	positive regulation of response to DNA damage	BP	1	EYA1	up_VFEM_VSMC
GO:0055007	0.103	0.995	1	31	cardiac muscle cell differentiation	BP	1	TBX5	up_VPUL_EC
GO:1901654	0.103	0.995	1	31	response to ketone	BP	1	PTGER2	up_VPUL_EC
GO:0001707	0.103	0.995	1	25	mesoderm formation	BP	1	HOXA11	up_VSAP_VSMC
GO:0050818	0.103	0.995	1	24	regulation of coagulation	BP	1	CAV1	up_VSAP_VSMC
GO:0040017	0.103089993	0.95167	8	180	positive regulation of locomotion	BP	1	SOX9,AQP1,SEMA6D,FOXC2,LRRC15,CHMP4B,RARRES2,NRP1	up_ACOR_EC
GO:0097475	0.103334329	0.99584	1	4	motor neuron migration	BP	1	NRP1	up_ACOR_EC
GO:0071941	0.103352317	0.99583	1	4	nitrogen cycle metabolic process	BP	1	NR1H4	up_ACOR_EC
GO:0032287	0.10341734	0.99583	1	4	peripheral nervous system myelin	BP	1	SH3TC2	up_ACOR_EC
GO:2001135	0.103540947	0.99582	1	4	regulation of endocytic recycling	BP	1	ACTN2	up_ACOR_EC
GO:0048703	0.103593415	0.99581	1	4	embryonic viscerocranium morphogenesis	BP	1	FOXC2	up_ACOR_EC
GO:1902187	0.103620387	0.99581	1	4	negative regulation of viral release from host cell	BP	1	TRIM8	up_ACOR_EC
GO:2000480	0.103714145	0.9958	1	4	negative regulation of cAMP-dependent protein	BP	1	PKIB	up_ACOR_EC
GO:0030238	0.103745277	0.9958	1	4	male sex determination	BP	1	SOX9	up_ACOR_EC
[truncated: 531,451 more chars]
